# A statistical approach to detection of copy number variations in PCR-enriched targeted sequencing data

**DOI:** 10.1186/s12859-016-1272-6

**Published:** 2016-10-22

**Authors:** German Demidov, Tamara Simakova, Julia Vnuchkova, Anton Bragin

**Affiliations:** 1 Parseq Lab, Birzhevaya, 16, Saint-Petersburg, 199053 Russia; 2 Department of Mathematics and Information Technology in SPbAU RAS, Khlopina, 8/3, Saint-Petersburg, 194021 Russia; 3 Genomic and Epigenomic Variation in Disease Group, Centre for Genomic Regulation (CRG), The Barcelona Institute of Science and Technology, Dr. Aiguader 88, Barcelona, 08003 Spain; 4 Universitat Pompeu Fabra (UPF), Barcelona, Spain

**Keywords:** Machine learning, MPS, Germline CNV, Multiplex PCR, Targeted amplification

## Abstract

**Background:**

Multiplex polymerase chain reaction (PCR) is a common enrichment technique for targeted massive parallel sequencing (MPS) protocols. MPS is widely used in biomedical research and clinical diagnostics as the fast and accurate tool for the detection of short genetic variations. However, identification of larger variations such as structure variants and copy number variations (CNV) is still being a challenge for targeted MPS. Some approaches and tools for structural variants detection were proposed, but they have limitations and often require datasets of certain type, size and expected number of amplicons affected by CNVs. In the paper, we describe novel algorithm for high-resolution germinal CNV detection in the PCR-enriched targeted sequencing data and present accompanying tool.

**Results:**

We have developed a machine learning algorithm for the detection of large duplications and deletions in the targeted sequencing data generated with PCR-based enrichment step. We have performed verification studies and established the algorithm’s sensitivity and specificity. We have compared developed tool with other available methods applicable for the described data and revealed its higher performance.

**Conclusion:**

We showed that our method has high specificity and sensitivity for high-resolution copy number detection in targeted sequencing data using large cohort of samples.

**Electronic supplementary material:**

The online version of this article (doi:10.1186/s12859-016-1272-6) contains supplementary material, which is available to authorized users.

## Background

Targeted sequencing (TS) is a routine method that enables sequencing of specific genome regions that are of interest to researchers. In comparison with whole genome (WGS) or whole exome sequencing (WES) the TS has lower analysis cost, provides deeper target regions coverage and reduces storage and analysis infrastructure requirements [1]. In contrast, variability in efficiency of amplification during library preparation leads to uneven amplicon coverage from one experiment to another. This limits the usage of existing coverage-based CNV detection tools for a TS data. Well-known paired-end algorithms that use insert size and reads’ orientation are unapplicable for analysis of data produced with amplification-based sample preparation techniques [2, 3].

CNV corresponds to the deletions and duplications of some large (one hundred base pairs or larger) portions of the genome [4]. Many known CNVs are associated with genetic disorders and thus are classified as pathogenic mutations [5–7]. Several approaches for CNV detection are used in clinical diagnostics, but most of them use WGS or WES data and cannot be applied for amplification-based TS [8, 9].

The CNV detection tools that work with TS data usually use one of three approaches or combination thereof, which are on-target read depth (RD), B-allele frequencies (BAF) or off-target read depth (OR). The examples are ADTEx [10] (uses RD + BAF), ExomeCNV [11] (uses RD + BAF), CNVKit [12] (uses RD + OR). However, BAF and OR approaches have several limitations. At first, BAF approach is typically used in case-control studies for the detection of long CNVs while the probability of having at least one point mutation for CNV covered with small amount of short targets is low. OR approach is suitable only under the assumption that significant portion of reads are off-target so they provide a low-coverage sequencing of whole genome which is not always the case – there can be only negligible amount of reads that did not map on targets per sample. Another issue of OR approach in respect of amplification-based sequencing is a non-uniform coverage of off-target reads across the genome that arises due comparatively frequent non-specific amplification. Thus we conclude that RD is the most appropriate method for detection of CNVs that can be intersected with small number of targets.

Usually RD CNV detection methods evaluate the ratio between reads aligned to the target DNA segment and the total number of aligned reads [13]. If read count for the selected DNA segment in a sample differs from the value estimated for the control set of samples lacking CNVs, the testing sample is considered to carry CNV corresponding to the DNA segment [14]. This works well with WGS that provides somewhat uniform read distribution along the target region (moreover, the uniformity can be improved by taking into account sequence characteristics such as GC-content), but cannot be directly applied to PCR or hybridization enriched data since they are characterized by a substantial coverage variability due to the differences in DNA fragments capture or amplification efficiencies. Exponential growth of PCR product quantity further reduces the coverage uniformity. Therefore, existing CNV detection algorithms in WGS data are poorly applicable for TS data.

We have found several tools for CNV detection in PCR-enriched TS data [15–17]. As was described in papers, these tools were designed for detection of long variants intersected with substantial number of targets thus the sensitivity and/or specificity are expected to be low in case of TS data analysis that contain CNVs of regions covered with small (up to one) number of amplicons. Below we provide analysis that supports this conclusion.

We present a novel approach to the problem of CNV detection in TS data. In contrast to the vast majority of coverage based CNV detection tools for WGS/WES data, we do not use library size normalizations. At the heart of the algorithm is the idea of biochemical similarity of some amplicons in the large pool of sequences in multiplex PCR and machine learning techniques. We also present the tool named CONVector that implements this approach. To evaluate tool efficiency we performed verification using large dataset of more than 1000 sequencing results and made a comparison with existing tools. The tool is open source and is deposited on GitHub.

## Methods

### Implementation

Algorithm presented is implemented in CONVector software package that uses Python for user input collection and data preprocessing and Java (≥ 1.7) for data analysis. Source code is licensed under GPLv2 and is deposited on GitHub: https://github.com/parseq/convector.

### Model description

Amplification-based enrichment strategy is widely used for targeted sequencing. Commercially available enrichment systems, such as AmpliSeq (LifeTechnologies), provide the way to selectively amplify genomic regions of interest. Number and location of targets are determined by the analysis aim and varies from hundreds to thousands in one sequencing library thus making analysis highly multiplex. Quantity of PCR-product from certain target region (*N*) generally depends on the initial amount of target DNA segment in the sample (*N*_0_), number of PCR cycles (*c*), amplification efficiency (*e*), and is typically modelled as

1 $$  N=N_{0} \times (1 + e)^{c}.  $$

The main principle of coverage-based CNV detection is to estimate *N*_0_s from *N*s and to compare estimated *N*_0_s of targets to find the underrepresented (deletions) or overrepresented (duplications) sequences. The task of homozygous deletion detection is trivial since in case of *N*_0_ equals to zero the *N* is also a zero. Such condition can be detected by the lack of target sequence in the sequencing results. Other cases such of heterozygous deletions and homo- and heterozygous duplications require deduction of *N*_0_’s from *N*’s that can be done by counting reads from corresponding amplicons. The number of reads (*N*), i.e. amplicon coverage, is highly dependent on amplification efficiency that is influenced by amplicon structure, primer characteristics and reaction conditions. In highly concurrent multiplex PCR environment the amplification balance can be shifted by even minor condition changes that are beyond the control of the researcher. Resulted imbalance leads to an inability to directly compare target region absolute or relative coverages between samples to perform CNV detection, except of cases when analyzed genome region is covered by large number of amplicons (thousands of bases). Nevertheless, detection of relatively small portions of the genome, up to a single exon that is covered by one or few amplicons, may have biological or clinical importance.

To get CNV detection with an amplicon scale resolution we imply the idea that in large amplicon population, which is the case of target PCR, it is possible to find amplicons with similar respond to reaction conditions. Therefore to detect changes in the initial amount of targets we can compare coverages of targets which have similar amplification behavior in series of samples catching the anomalies (under- or overrepresentation), i.e. CNVs. Since the described approach accounts for any factors affecting amplification efficiency (even hidden and uncontrolled), it is expected to be more efficient than the normalization just on amplicon or primer characteristics.

The analysis consists of two steps: grouping amplicons with highly correlated coverages in the series of samples and CNV detection by multiple coverage comparison between amplicons within a group. From this perspective, CNV detection can be formulated as anomaly detection using methods of robust statistics.

The analysis requires aligned reads from samples sequenced with the same set of primers and under the same reaction conditions. The goal of analysis is to determine zygosity state for each amplicon in each sample of the dataset. The model suggests that if two amplicons have similar amplification efficiencies and the influence of stochastic effects is small enough, the coverages of these amplicons in set of samples are correlated. The analysis of intersection of confidence intervals for *λ* obtained using Box-Cox transformation applied to the pairs of correlated amplicons and model 1 suggests that log transformation of amplicons coverage should be used to stabilize the variance and to make the variables homoscedastic. We propose two algorithms, unsupervised and supervised, that can be used separately or in combination. First (unsupervised) algorithm operates with amplicons while the second one (supervised) operates with CNV sites that may include one or more amplicons. Since CNV breakpoints are usually located in intronic regions, it is useful to describe CNVs sites in terms of affected exons [18]. We assume that the dataset may contain a number of samples carrying CNVs, but each particular target is affected by CNV just in some subset of samples for the first algorithm. The prerequisite for the second algorithm is the presence of at least 20 samples that are free of CNVs in test dataset which can be satisfied with a priori known control dataset or be assumed based on low previously estimated populational frequency of CNVs in the particular genes of interest. The frequency of CNVs’ presence in the dataset and the dataset size influence on the robustness and effectiveness of the statistical model. Presented model is based on the assumption that each particular CNV is present in no more than 20 *%* of samples in the dataset, which is in consistence with holds for majority of real-life cases. We used the robust linear regression model to estimate the linear relationships between amplicons.

For CNV zygosity determination, we assumed that the amplification starts from the equal numbers of maternal and paternal copies of targets. The result of amplification, i.e. coverage, is a sum of independent random variables so we can build a linear model for any zygosity state, except of homozygous deletion (coverage is zero, the detection is trivial), by multiplication of coverage with corresponding factor: 0.5 for heterozygous deletion, 1.0 for wild type, 1.5 for heterozygous duplication and 2.0 for homozygous duplication and so on. Using the linearity of the sum of independent random variables’ variances, we can conclude that the variance is decreased when multiplier is equal to 0.5 and increased when it is greater than 1.0. This limitation results in the difficulty of duplication detection relatively to a deletion’s detection due the lower power of corresponding statistical tests. However, it is possible to build a statistical model for CNV statuses detection inside the group of correlated amplicons and use the likelihood considerations of certain CNV status for each amplicon in each sample, but the power to detect the exact number of copies is decreasing with the higher ploidy states.

Our algorithms use several robust statistical techniques that require comparatively large test dataset size for accurate CNVs’ detection. Using the real sequencing data (described below) we determined the lower threshold for the number of samples in the test dataset as 25. However, the tool can be used for datasets of smaller size in case the control dataset of sufficient size is available.

### CNV detection algorithms

**Fig. 1:**
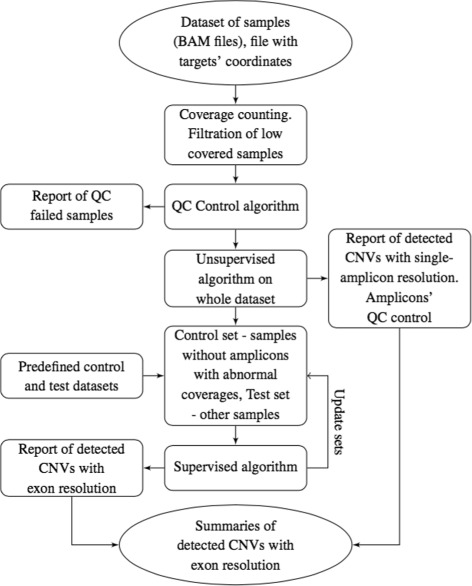
Pipeline scheme

The detection pipeline consists of two stages: unsupervised and supervised (Fig. 1). The unsupervised algorithm takes coverages of amplicons in the samples as an input and does not require a priori assumptions about the potential presence of CNVs except the maximum CNV frequency at any particular site (described above). The next algorithm (supervised) requires a control (free of CNVs) dataset. Control dataset can be made using the results of the unsupervised algorithm or alternative methods.

**Fig. 2:**
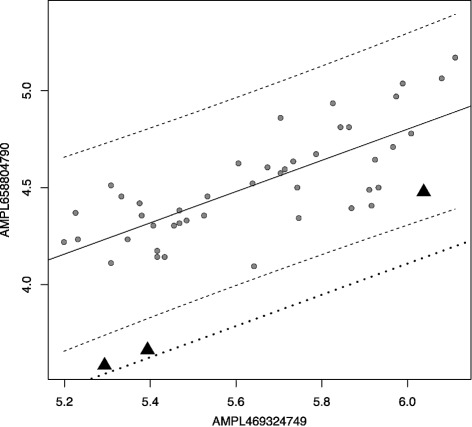
(Unsupervised algorithm idea). Linear model of amplicons coverage. *Axes*: logarithms of coverages of two different amplicons for one run (48 samples). *Dots*: wild type amplicons, *triangles*: heterozygous deletions. *Dash lines*: OLS regression for wild type amplicons and 0.99 prediction interval. *Dot line*: the regression line for heterozygous deletion

We use following notations: *a*_*j*_ means *j*-th amplicon in the dataset, *c* *o* *v*(*a*_*j*_) denotes the *a*_*j*_’s vector of coverages across the samples, *a*_*jk*_ denotes amplicon which vector of coverages is correlating, i.e., has correlation is higher than fixed threshold *T*, with *c* *o* *v*(*a*_*j*_) (Fig. 2). *T* can be choosen as biggest number from 0.0 to 1.0 such as all analysed exons have at least one amplicon that passes quality control procedure (described below). *c* *o* *v*(*a*_*j*_)[*i*] denotes coverage of amplicon *j* in the sample *i*. In order to exclude cases when both *a*_*j*_ and *a*_*jk*_ can be affected with CNV we restrict selection by setting minimum physical distance between *a*_*j*_ and *a*_*jk*_. In cases when target regions spread across genes it is possible to select *a*_*jk*_ from the set of target regions that are located on genes other than *a*_*j*_ is located on. Also we denote *M*_*Normal*_,*M*_*HetDel*_,*M*_*Dup*_ as statistical models for number of copies 2, 1 and greater or equal to 3, respectively.

We impy the idea that standard correction on features such as GC-content and read length removes a large part of variation, but there are many other sources for amplification-based sequencing (for instance, primers’ properties) and it is not usually possible to infer all of them. Thus we use different type of normalisation: we construct statistical models within clusters of correlated amplicons. They explain large proportion of variation in response variable which makes standard corrections unnecessary. Pearson’s correlation coefficient used as an effective non-robust estimator of explained variance for the situations where we are sure that there are no outliers. Robust estimators of correlation and regression’s parameters used as less effective robust alternative for the case where the possible presence of outliers is unknown. Finally, normalisation on correlated random variables makes covariance matrix more close to diagonal, consequently, robust to overfitting, which makes usage of regularized classification methods possible.

### Unsupervised algorithm

CNV states are determined by building linear models using a *c* *o* *v*(*a*_*j*_) as the response and *c* *o* *v*(*a*_*jk*_) as predictors (Fig. 2).

If *c* *o* *v*(*a*_*j*_)[*i*] can not be recognized as an outlier for linear model *M*_*Normal*_(*a*_*j*_), we will consider that this amplicon is in wild type state for sample *i*.

If *c* *o* *v*(*a*_*j*_)[*i*] is lower than detection threshold (i.e., close to 0), the amplicon is in homozygous deletion state for sample *i*.

If *c* *o* *v*(*a*_*j*_)[*i*] is lower than we can expect for wild type amplicon and it is closer to the linear model for heterozygous deletion *M*_*HetDel*_(*a*_*j*_) (i.e., the corresponding residual is smaller, which means that the simple Bayes factor $K = \frac {P(cov(a_{j})|M_{HetDel}(a_{j})) }{ P(cov(a_{j})|M_{Normal}(a_{j})) }$ for this models is greater then one), but were not recognized as the homozygous deletion, we can conclude that it is a heterozygous deletion (for sample *i*).

If *c* *o* *v*(*a*_*j*_)[*i*] is detected as an outlier for sample *i*, but it is higher that the regression line and it is closer to the linear model for heterozygous duplication, it can be considered that it is a duplication.

Our approach was developed for single-copy germline CNVs’ detection so this set of models covers vast majority of real-life cases, but number of models can be increased for detection of complex rearrangements involving quantitative change of higher order (4, 5, etc.). Such ploidy states are not considered as a separate case because the prediction intervals of the models for different types of duplications intersects largely. It is possible to determine the exact ploidy of the region only in case the region is large, but we considered the detection of CNVs with the highest possible resolution (up to one amplicon) as more important than detection of regions with higher possible ploidy with necessarily low resolution.

In order to avoid the influence of errors in predictors we determine the CNV state of an amplicon *a*_*j*_ by constructing *L* linear models that explain variance in *c* *o* *v*(*a*_*j*_) better than others. To do this, we use *L* other amplicons *a*_*jk*_, which vector of coverages in series of samples being analyzed shows the highest correlations with *c* *o* *v*(*a*_*j*_) in the same series of samples. Then we assume that if the coverage of *a*_*j*_ in a particular sample was detected as CNV in *N* of *L* such models, then it indicates the presence of CNV. *L* and *N* were empirically choosen as equal to 5 and 4 for our experimental data since larger or smaller numbers gave us worse performance on the train dataset.

*S*_*n*_-correlation coefficient is used as a measure of similarity [19]:

$$r_{S_{n}} = \frac{{S_{n}^{2}}(u) - {S_{n}^{2}}(v)}{{S_{n}^{2}}(u) + {S_{n}^{2}}(v),} $$

where *u* and *v* are the robust principal variables: $ u = \frac {x - med(x)}{\sqrt {2} MAD(x)} + \frac {y - med(y)}{\sqrt {2} MAD(y)}, v = \frac {x - med(x)}{\sqrt {2} MAD(x)} - \frac {y - med(y)}{\sqrt {2} MAD(y)}.$ Here and below *S*_*n*_ means Rousseeuw and Croux’s estimator of standard deviation [20].

We used Theil-Sen estimator [21] as a robust linear model because of its high breakdown point ($1 - \frac {1}{\sqrt 2} \approx 29.3~\%$), satisfactory effectiveness and simplicity.

We used the standard formula for internally studentized residuals with the standard deviation replaced with its robust analogue. We used empirical level of significance for the Student’s quantile as a threshold for outliers detection (*α*=0.02 for deletions and *α*=0.05 for duplications). The formula of calculating *i*-th studentized residual is:

$$\frac {\hat \varepsilon_{i}} {S_{n} \left(1 - \left(\frac{1}{n} + \frac{x_{i} - \bar{x}}{\sum_{j=1}^{n} (x_{j} - \bar{x})^{2} }\right)\right)}, $$

where $\hat \varepsilon _{i}$ means the *i*-th residual, $\bar {x}$ means mathematical expectation of the random variable. Algorithm’s pseudocode is available in Additional file 1.

### Supervised algoritm

Using the output from the unsupervised stage samples can be grouped using the following rule: if at least one of the amplicons is marked as outlier the sample is suspected of carrying CNV and added to test dataset, and if there is no outlier, the sample is added to control dataset. Also it can be performed using control dataset with a priori known absence of CNVs.

In general, we should search CNVs in any possible CNV site, which can be reformulated as a classification problem. We use ideas of linear discriminant analysis, however, the decision making is divided into 3 separate steps in order to make it configurable for the particular purposes.

Given that CNV site can be considered as a multivariate random variable and in practice we may have up to 100 samples per dataset (each sample consists of hundreds of amplicons), the direct usage of robust covariance matrix consisting of thousands of predictors is useless because of overfitting. To handle this we normalize the coverage values and use the block covariance matrix. In the presented work, we used the size of block one times one.

**Fig. 3:**
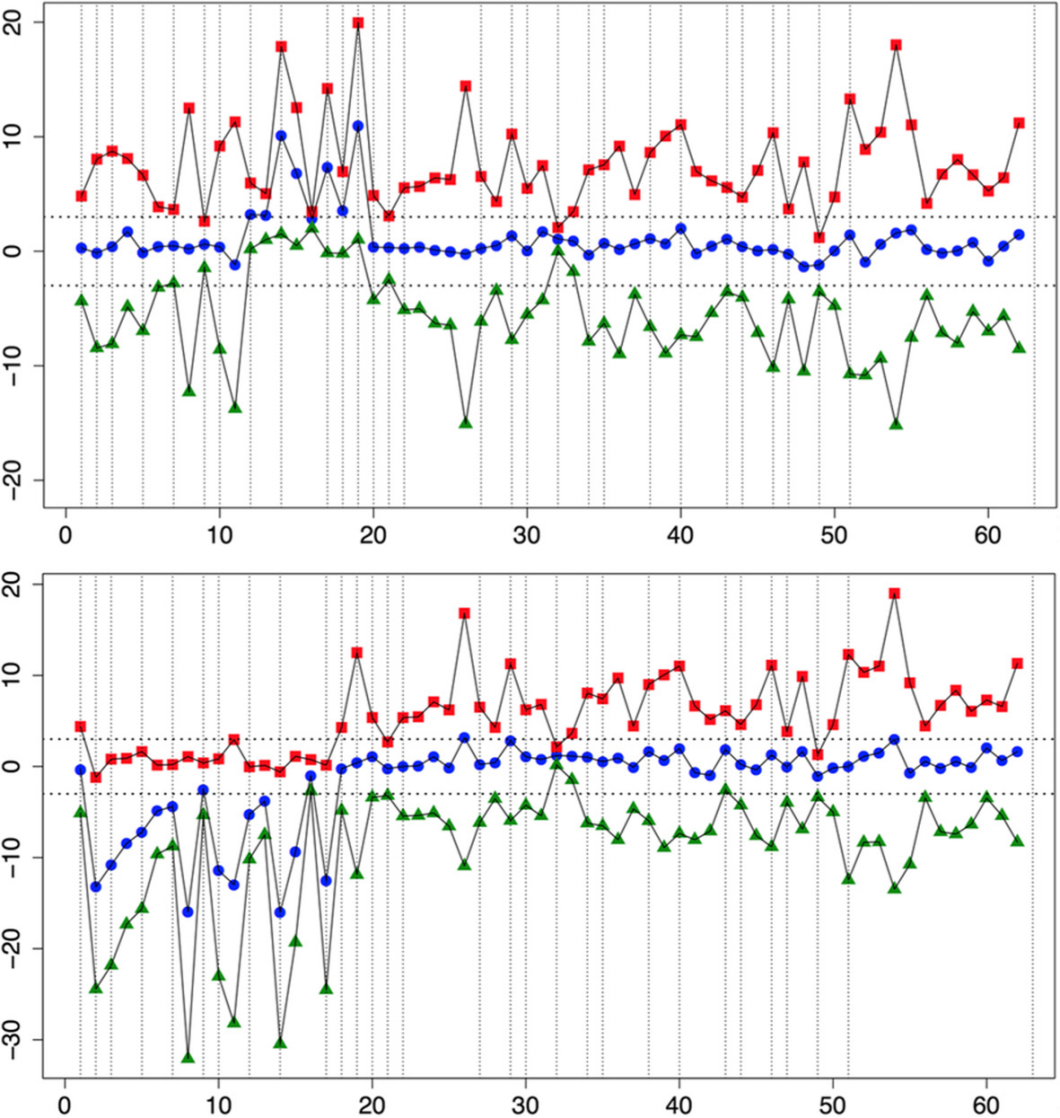
(Supervised algorithm idea). *Top*: duplication of exons 7–11 in CFTR gene (amplicons within [12;19]), *bottom*: deletion of exons 2–9, CFTR (amplicons within [2;17]). *y-axis:* distance from 0 in terms of standard deviations, *x-axis:* amplicons from CFTR gene. *Horizontal lines* ±3 standard deviations, *vertical lines* shows the exons’ structure. *Top line (points as squares):* distance to *M*_*HetDel*_, *middle line (points as circles):* distance to *M*_*Norm*_, *bottom line (points as triangles):* distance to *M*_*Dup*_. Distances inside each region are combined into one NED per model

The algorithm contains several steps:

1. For each amplicon *a*_*j*_ we determine cluster *C* of *k* other amplicons *a*_*jk*_. Algorithm uses Pearson’s correlation coefficient as an estimator of correlation in case the Control set is big enough (more than 20, according to bias and variance of permutation based simulations) and robust correlation estimator otherwise.
2. Normalisation of amplicons’ coverages: $x_{j} = \log (cov(a_{j})) - \Bigl (\frac {\sum _{k \in C} \log cov(a_{jk})} {|C|} \Bigr).$ In order to reduce variance we use a group of highly correlated amplicons located far enough from each other so they can not be affected by the same CNV (i.e., amplicons from different genes or chromosomes) and then normalise on its average instead of normalisation on total amount of reads. For datasets with large number of genes and possible CNVs more robust normalisation procedure can be applied. For example, instead of raw coverages a sampled distribution of coverages, predicted by linear models on the first step, can be created and considered as normalisation factor (correlated amplicons should be taken uniformly from different genes).
3. We construct several probabilistic models for each amplicon, respectively: *M*_*Normal*_(*a*_*j*_),*M*_*HetDel*_(*a*_*j*_),*M*_*Dup*_(*a*_*j*_). Assuming that all *x*_*j*_ are distributed normally, we calculate the robust mean estimations for models using vectors log(*c* *o* *v*(*a*_*j*_)), log(*c* *o* *v*(0.5*a*_*j*_)), log(*c* *o* *v*(1.5*x*_*j*_)) and robust scale estimation *S*_*n*_ for *x*_*j*_. For simplicity, we assume that number of reads produced from a fragment is distributed as *P* *o* *i* *s*(*N*), where *N* was described in 1. Since we applied log transformation that is much stronger than square root transformation (that keeps variance approximately constant), we have to use correction factors for variances. We can estimate the correction factors using simulation procedure as $\hat \sigma ^{2}(x_{jDel}) = 2 \cdot \hat \sigma ^{2}(\log (x_{j})), \hat \sigma ^{2}(x_{jDup}) = \frac {2}{3} \cdot \hat \sigma ^{2}(\log (x_{j}))$, where *x*_*jDel*_ and *x*_*jDup*_ denote expected transformed coverages for *M*_*HetDel*_(*a*_*j*_),*M*_*Dup*_(*a*_*j*_), respectively.
4. For each region we ask three questions consequently: a) can region’s vector of coverages be produced by probabilistic models *M*_*HetDel*_ or *M*_*HetDup*_? b) is it highly probable that the region was produced by *M*_*Normal*_? c) is *M*_*HetDel*_ or *M*_*HetDup*_ the most probable explanation for the observed coverage of the region?
  In order to answer the first question a), we test if each amplicon’ coverage can be produced by these models, using *χ* ^2^ statistics with low level of significance *α* (0.05 divided by expected number of amplicons that are altered in copy number, can be roughly estimated as “total number of amplicons in Test dataset divided by 2”, since we have an assumption on CNVs’ frequency). If all amplicons located inside CNV site can be produced by one of this models, we then mark this site as “suspicious”.
  Next, we are trying to reject the hypothesis that the normalised coverages within each “suspicious” CNV site can be produced by *M*_*Normal*_. We use normalised Euclidean distance as a test statistic (2) It follows *χ* ^2^-distribution because we can assume that the estimated covariance matrix is approximately diagonal after the normalisation step. False discovery rate is controled using Benjamini–Hochberg procedure. Obtained adjusted *p*-values are qualified as an answer for the second question.
  Finally, we check if region’s coverage Bayes factor (where *H*_0_ is the absence of CNV and *H*_1_ is one of the CNVs’ models) is bigger than some constant value. Using prior knowledge of CNVs frequency in our dataset we chose the value of 100 as a threshold and assumed equal prior probabilities of models. The graphical representation is depicted on (Fig. 3).
5. The larger size of Control set leads to more accurate statistical estimations and increase the quality of output so if it was found that some of samples from Test set are free of CNVs, we include them in the Control set and repeat the procedure from the first step. Otherwise, we provide the final report about CNVs on CNV-site resolution (currently, we use exons as potential CNV sites due to biological reasons).

Normalised Euclidean distance (NED) can be defined as:

2 $$ d(\vec{x}, \vec{y}) = \sqrt{\sum_{i=1}^{N} \frac{(x_{i} - y_{i})^{2}}{{s_{i}^{2}}}}  $$

where we used *S*_*n*_ estimator of standard deviation for *x*_*i*_−*y*_*i*_ values as *s*_*i*_ and *y* is a vector of the location estimations. We used the median of Walsh averages due its robustness and effectiveness as the estimation of location.

### Input data quality control

Low covered samples may lead to an unreliable results due the high influence of stochastic effects on amplification and sequencing therefore we filtered out samples that contain less than 15000 reads. We use the quality control procedure for amplicons as well: in case if amplicon has average coverage less than 50 or does not have the required number of correlating amplicons for the prediction on the unsupervised algorithm, we mark this amplicon as QC failed and do not consider. In case the whole exon is covered only with amplicons that did not pass QC control, we do not include this exon into analysis in all samples.

After initial filtering the algorithm defines all amplicons which coverage is close to zero as homozygous deletions. We use coverage 10 as a threshold for homozygous deletion since in practical cases the coverages for homozygous deletions do not exceeded this limit. Then it filters out samples that are not similar to other samples in the dataset. We call such samples “irregular” because they have no analogues in amplification behavior expressed in coverage. Statistical estimation on irregular samples and determination of their CNV states are inaccurate. The presence of large CNV leads to abnormal coverage ratios therefore only samples deviated by more than *n*/2 genes, where *n* denotes the overall amount of genes, *n*>2, are considered irregular. The assumption is that the presence of *n*/2 large scale CNVs in n genes in one sample is rare.

The algorithm can be divided into several steps. After read counting, we perform library size normalisation taking into account that the total number of reads can be influenced by the large CNV and lead to bias in normalised data. To solve this problem, normalisation is performed within the genes: we divide each amplicon’s coverage by the total number of reads aligned to corresponding gene. For simplicity, we assume that logarithms of obtained values are normally distributed.

Next we calculate NED (2) using this normalised log-transformed data for each gene separately. Working with real data, we found the significant proportion of amplicons can have extreme values which leads to unrealistically high NED for the whole gene. Due the fact that the presence of small proportion of outliers does not corrupt the further analysis due the used estimators’ robustness, we use only 80 *%* of coordinates (rounded up) with smallest values for NED calculation.

We compare obtained NED value with *χ* ^2^-distribution and degrees of freedom equal to 80 *%* of initial number of amplicons within the gene. The sample does not pass quality control if test statistics for more than $\frac {n}{2}$ genes exceed pre-defined level of significance.

## Results and discussion

### Basic data analysis

Tools from Life Technologies Torrent Suite™ version 4.2 with parameters recommended by manufacturer were used for reads’ alignment. We developed approach for mapping reads to target regions and describe it in Additional file 1.

### CONVector evaluation by experimental datasets

To test our model we used 26 experimental datasets generated on the Ion PGM™ platform using VariFind™ Neoscreen assay (Parseq Lab, Russia) with three different sequencing panels targeting CFTR, PAH and GALT genes. Panels IAD30284, IAD39777 and IAD75243 consisted of 126, 127 and 146 amplicons correspondingly (coordinates of targets are provided in Additional file 2). Each dataset comprised about 48 samples (see Additional file 3). Sequencing data from healthy individuals and patients diagnosed with cystic fibrosis, phenylketonuria and galactosemia were obtained from Parseq Lab biobank (read length was equal to 150, average coverage of samples was distributed from ~120 up to 1200 reads per amplicon). Total number of analyzed sequencing results was 1090. Total number of unique samples was 552. We used sequencing data generated by four independent labs, in order to evaluate robustness and reproducibility of the developed tool.

For panels IAD30284 and IAD39777 CNV detection was performed using unsupervised algorithm only, because 11 exons were covered with only one amplicon. For panel IAD75243 we used both algorithms in the single pipeline since the panel was designed in such a way that each exon was covered with more than 2 amplicons or has at least 2 flanking amplicons. CONVector evaluates coverage correlation between amplicons within a sample and within a run. PCR amplification biases result in deviations in amplicons behavior and decreased correlation. Datasets with highly correlated amplicons coverage are good quality and unbalanced datasets are poor quality. Poor quality may be the result of PCR bias or differences in sample preparation procedures. We revealed that the variance of amplicon relations varies greatly from experiment to experiment that makes CNV detection within set of samples sequenced in one run is more effective therefore, we analyzed each dataset separately.

Among 552 analyzed samples, 507 samples have successfully passed QC filter and 33 have carried CNVs detected by CONVector. Interestingly that only 23 of them have been previously found in corresponding samples using convinient methods. Yet four of previously known CNVs have not been detected by CONVector (one sample didn’t pass QC filter; three false-negative samples carrying CFTRdele2,3 had poor sequencing libraries). All newly detected CNVs have been confirmed by MLPA method (SALSA MLPA probe sets by MRC-Holland, Amsterdam, The Netherlands). One of the detected CNVs, PAHdele 4, is described for the first time in PKU patient carrying L48S on the other allele. Detected CNVs included deletions and duplications of the CFTR gene and deletions of the PAH gene. We did not detect CNVs in the GALT gene. All CNVs, except one homozygous CFTRdele2,3 detected in one analysis, have been heterozygous. CNVs size is been ranged from one exon to the whole gene. Thus, we can conclude that applied algorithm is suitable for targeted sequencing data and allows accurate detection of different types of CNVs (see Additional file 3).

In order to calculate sensitivity we have selected 64 analyses of 36 unique samples carrying different types of previously known CNVs (data from Parseq Lab biobank) and analyzed them with the CONVector tool (results are available in Additional file 4). Analysis was performed on data generated during 13 sequencing runs (6 runs with IAD30284 panel; 6 runs with IAD39777 panel; 1 run with IAD75243 panel). We defined sensitivity as the proportion of true positives among all positive results. Sensitivity of the developed tool comprised 84.62 % (95 % CI: 73.52-92.37). False negative results were mainly obtained for CNVs affecting one exon that is covered by one amplicon, such as PAHdele5 in IAD39777 panel. We achieved better results for this CNV using panel IAD75243, where this exon is covered by three amplicons. Thus, sensitivity of the detection may be increased by appropriate panel design with increased number of amplicons covering the exons. This is confirmed by the result, calculated only for exons that are covered by more than one amplicon. The sensitivity comprised 94.23 % (95 % CI: 84.05-98.79). In order to calculate specificity we have analyzed 244 unique clinical samples from compound heterozygous patients diagnosed with cystic fibrosis or phenylketonuria. All patients are carriers of two previously known pathogenic SNPs located in trans (data from Parseq Lab biobank). We assumed that the probability of appearing CNV in cis with pathogenic SNP is extremely small therefore we considered such samples as not carrying CNVs in corresponding genes, i.e. CFTR or PAH. We did not found false positive CNVs thus specificity calculated as the proportion of true negative among all negative results is comprised 100 % (95 % CI: 98.50-100.00 %).

We also provide the result of analysis of the whole dataset of 938 sequencing results that passed QC procedures, sequenced by panels described above and consisted of biological and technical replicates. Every reported result of one sample’s analysis is called *positive*. In case the reported result has intersection of at least 50 % with true CNV, we called it *true positive*, otherwise *false positive*. When CONVector does not detect CNVs detected previously, we consider it as a *false negative*. Duplications that affect only one exon were not considered as a call since, as was shown above, models for normal and duplicated data intersects largely, comparatively to deletion and normal models’ intersection.

**Fig. 4:**
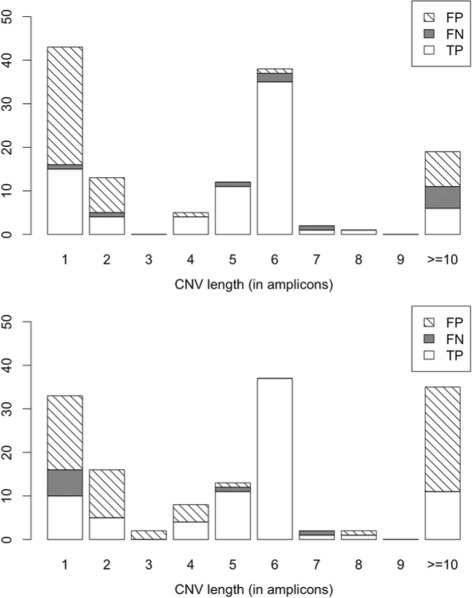
Barplots of CNVs’ lengths detected by two algorithms. *x-axis:* lengths of CNVs, in amplicons (after QC), *y-axis:* CNVs’ frequencies. *Top*: unsupervised algorithm (number of True Negatives: 805), *bottom*: supervised algorithm (number of True Negatives: 790)

Sensitivity and specificity of CONVector using 938 samples were equal to 87.5 % (95 % CI: 78.73–93.59 %) and 94.7 % (95 % CI: 92.98–96.11 %) for the unsupervised algorithm and 90.9 % (95 % CI: 82.87–95.99 %) and 92.94 % (95 % CI: 91.01–94.57 %) for the supervised one. As can be seen from the design of algorithms, they differ in detection sensitivity of short and long CNVs (Fig. 4). Large part of the false positive CNVs that covered only one or two amplicons arise recurrently in technical replicates, but was not confirmed by alternative methods. We suggested that they can be caused by technological artifacts such as inability of primers’ binding. All 8 false positively detected deletions covered with more than ≥10 amplicons also happen recurrently in technical replicates and were detected by other tools, but each of them has at least several heterozygous mutations inside so we consider these results as caused by wrong library preparation. The analysis of these CNVs with multiplex ligation-dependent probe amplification also showed the presence of large deletions (an example is provided in Additional file 1).

CONVector’s computational time for the dataset of 48 samples and panel of 128 amplicon is approximately 1 hour for coverage calculation and up to half a minute for quality control and two stages of algorithm on a laptop with 2 GHz Intel Core i7 and 8 GB RAM. Other tools’ computational time is less than one minute (except online version of IonReporter which can not be tested under the same conditions), however, the most time consuming step (coverage calculation with realignment, described in Additional file 1) can be replaced with simple, but less precise counting procedure, making tools’ computational time approximately equal.

### Comparison with other tools

In order to evaluate CONVector algorithm performance we have compared our tool with Ion Reporter (IR) CNV analysis tool (v.4.6) developed by Life Technologies, ONCOCNV (v. 6.4) [16], cn.mops (v. 1.16.2) [22] and Conifer [23]. Due the fact that first two tools has prerequisites such as presence of control samples with confirmed absence of CNVs in targeted regions and high quality of sequencing, we analysed them using comparatively small dataset sequenced with one panel, while the comparison with cn.mops was performed on the whole dataset and all 3 panels.

IR tool is optimized for Ion Torrent Sequencing technology. The algorithm finds the most likely copy number segmentation and ploidy state based on the comparison with control set of samples (baseline) and using Hidden Markov Model. IR can be run on the Ion AmpliSeq data however its sensitivity depends on the panel size. The smallest AmpliSeq panel that has been tested is comprised of 200 amplicons. We have run this tool on the panel IAD39777 consisted of 127 amplicons. We have tested IR on 13 positive and 56 negative samples, selected as described above and randomly. Only samples containing CNVs spanning of at least two exons or covered by at least six amplicons were selected as positive samples for sensitivity evaluation since none of the examined tools were able to detect single amplicon CNVs. The tool was run with the default settings and the medium sensitivity. CNV baseline was generated from 50 control samples, not carrying CNVs (results are available in Additional file 4). Sensitivity of the IR tool is comprised 64.29 % (95 % CI: 38.57–90.91 %); specificity – 100 % (95 % CI: 93.62–100.00 %). Analytical characteristics of the IR CNV detection established in this study is much lower than it was shown previously (see poster of Rhodes et al., (2013)). Therefore, we can assume that the IR CNV detection algorithm is much more useful for large CNVs covered by dozens of amplicon while CONVector algorithm allows detecting even small CNVs starting from hundreds bases and covered of at least few amplicons. Detection of such variations in targeted sequencing data is important since many of small CNVs are responsible for human diseases diagnosed with NGS panels [8].

We also made comparisons with ONCOCNV tool. We have choosen 5 control samples (sequenced in one run) that were free of CNVs. We used the same dataset as before for performance evaluation. Sensitivity of the ONCOCNV is comprised 57.14 % (95 % CI: 28.86-82.34 %), specificity – 89.29 % (95 % CI: 77.45-95.57 %). Six samples, sequenced in one experiment, were detected as having a duplication of whole gene, whereof we concluded that ONCOCNV’s performance may be improved with batch effect correction or quality control filtering as a preliminary step.

The comparison with cn.mops was of our special interest because cn.mops is also using matrices of coverages as an input so we evaluated this tool using exactly the same datasets as we used for CONVector testing. We tested cn.mops on 922 samples with the same preliminary QC steps as we used for our tool, but did not take samples that contained single-amplicon CNVs into account. We have choosen the threshold that allow us to have comparatively high sensitivity. After the filtration of samples that have single amplicon CNVs, we had 46 out of 74 CNVs as True Positives and 90 false positives. The sensitivity was equal to 62.16 % (95 % CI: 50.09-72.96 %), specificity: 89.38 % (95 % CI: 87.07-91.34 %).

The comparison with Conifer v0.2.2 was made using only the subset of available data due the technical issues of Conifer that we were not able to solve. Conifer also uses the whole cohort of available samples as an input, removes several singular values for batch effects removal and detects CNVs that are covered with at least 3 targets. QC control procedure was described in the paper, but were not implemented, however, all our samples would be removed using QC thresholds suggested by authors and there was no clear way to establish a new threshold suitable for all available experiments’ results. We decided to use basic QC procedure and removed all samples that were covered with less than 200 reads per target. We removed only 2 singular values from the data because after removal more the sensitivity dropped dramatically. We considered all variants covered with less than 3 targets as True Negatives. We had 721 samples from 17 experiments that passed quality control. We were able to detect 21 True Positive results among 43 possible while having 7 False Positive results. The sensitivity was equal to 48.84 % (95 % CI: 33.31-64.54 %), specificity: 98.97 % (95 % CI: 97.88-99.58 %). Raw output files (i.e., logs with the used parameters, plots and table reports) generated by CONVector and other tools used for the comparison (versions of the tools are specified above) are available in Additional files 5, 6, 7 and 8, described in Additional files section.

We also have tried to compare the performance of CONVector with other tools. This comparison is described in Additional file 9 and was not included into the main comparison section because of low quality of results, different requirements for the input data or technical problems we faced launching several tools.

## Conclusions

We described the new high resolution approach for CNVs detection that enables detection of CNVs with the size comparable to single exon (few hundred bases). The tool is based on novel approach that relies upon a fact of PCR efficiency correlations for subsets of amplicons in highly multiplex PCR. This approach can be also extended to hybridization based library preparation techniques. We also created CONVector – open-source tool designed for targeted sequencing CNVs detection. The tool has minimal requirements to input data, poses no limitations on CNV site size which can be as small as a single PCR amplicon, and does not requires control datasets or any a priory knowledge on potential CNVs sites location. We evaluated CONVector with the large dataset and made limited comparison with the analogs that showed superior specificity and sensitivity.

## Additional files

Additional file 1 Auxiliary procedures and descriptions. This pdf file contains details on counting of coverages procedure, illustration of non-uniformity of coverages across samples sequenced within one experiment, explanation of assumption of CNVs absence in the dataset where we did not make direct check with alternative methods, description of potential source of false positive results, description of comparison with cn.mops and ONCOCNV. Also CNV verification procedure is described here and pseudocode of the first algorithm provided in the end. (ZIP 968 kb)

Additional file 2 Description of Panels. xlsx file with bed files, containing: target’s chromosome, start coordinate, end coordinate, id of target, target’s region (exonic or intronic). (XLSX 27.6 kb)

Additional file 3 Description of Datasets. xlsx file with description of experiments: panel used for sequencing, internal id of experiment, number of samples within the experiment, laboratory where sequencing was performed, Average Robust Variance (absulte measure of noise, described in CONVector’s documentation), number of detected CNVs. (XLSX 9.74 kb)

Additional file 4 Supplementary table with comparison of CONVector, ONCOCNV, IR. xlsx file with data about samples: panel used for sequencing, id of experiment, internal sample’s code, laboratory, name of CNV, results of three methods. Status field denotes if particular variant was confirmed before analysis or was found by CONVector and validated. (XLSX 14.6 kb)

Additional file 5 Archive with cn.mops results. Zip archive with large pdf file (R markdown) with the results of cn.mops (plots) and short summary of calls. (ZIP 1771 kb)

Additional file 6 Archive with ONCOCNV results. Set of files (png plots and txt files) in zip archive that were provided by ONCOCNV after specificity and sensitivity testing. (ZIP 5007 kb)

Additional file 7 Final reports on CNVs analysis of whole dataset by CONVector. Tab delimited xls files with final reports of CONVector and short summary of calls. (ZIP 42.1 kb)

Additional file 8 Archive with Conifer results. txt files with CNV calls made by Conifer. (ZIP 1157 kb)

Additional file 9 Results of comparison with other tools. xls table with short summary of methods and performance (where possible) of 12 tools which we have tried to compare with CONVector. (XLSX 47.9 kb)

## Abbreviations

BAF: B-allele frequencies, proportion of total allele signal (A + B) explained by a single allele B
CNV: germline copy-number variant
MLPA: multiplex ligation-dependent probe amplification
NED: normalised Euclidean distance, explained in the main text
ORD: off-target read depth
PCR: polymerase chain reaction
QC: quality control
RD: read depth of enriched target regions
TS: targeted sequencing
WES: whole-exome sequencing
WGS: whole-genome sequencing
