# Supplementary material for: A statistical approach to detection of copy number variations in PCR-enriched targeted sequencing data
Source: BMC Bioinformatics. 2016 Oct 22;17:429. doi: 10.1186/s12859-016-1272-6 (PMC5075217; doi:10.1186/s12859-016-1272-6)
Supplement: Additional file 1 — Auxiliary procedures and descriptions. This pdf file contains details on counting of coverages procedure, illustration of non-uniformity of coverages across samples sequenced within one experiment, explanation of assumption of CNVs absence in the dataset where we did not make direct check with alternative methods, description of potential source of false positive results, description of comparison with cn.mops and ONCOCNV. Also CNV verification procedure is described here and pseudocode of the first algorithm provided in the end. (ZIP 968 kb) [file 12859_2016_1272_MOESM1_ESM.zip › Additonal File 1/template.pdf]

Noname manuscript No.
(will be inserted by the editor)

Supplementary 1: A Statistical Approach to Detection of Large-scale Structural Variations in PCR-enriched Target Sequencing Data

German Demidov [1,2,3,4], Tamara Simakova[1], Julia Vnuchkova[1], Anton Bragin[1]

Received: date / Accepted: date

1 Supplementary

1.1 Counting of coverages

The first step of coverage-based algorithm of CNV detection is the counting of coverages for each region. We developed our own tool that maps reads on targets. The standard approach for counting coverages is to align all reads on the reference genome and to map them using the rule: the read represents one of the targets if it has an intersection of maximum length with it than with any of other targets. This approach was not suitable for us beacuse we used 2 pools of amplicons that intersects with each other so it was more promising to calculate reads obtained from 2 pools separately, also due the fact that primers' are not always digested and because of the presence of chimeric parts of reads. We call a soft or hard clipped part of read "chimeric". Chimeric reads arise, probably, due to ligation artifacts during library construction step. Usually the overall amount of chimeric parts is 1-3% of total amount of reads. All these facts may lead to the wrong mapping and increase the overall noise. We suggest that all the chimeric parts of reads are represented in non-chimeric parts, also we suggest that the vast majority of reads actually came from the targets. So we have developed our own tool, called chimeric_solver.py, and it works in such way:

1. At first, all chimeric parts (parts with soft or hard clipping in CIGAR-strings) that are longer than 30 bp are trimmed off the reads and saved separately.

[1] Parseq Lab, St Petersburg, Russia.
[2] Department of Mathematics and Information Technology in SPbAU RAS, St Petersburg, Russia.
[3] Genomic and Epigenomic Variation in Disease Group, Centre for Genomic Regulation (CRG), The Barcelona Institute of Science and Technology, Dr. Aiguader 88, Barcelona, 08003, Spain. [4] Universitat Pompeu Fabra (UPF), Barcelona, Spain

2. The next stage is an accurate mapping of each read with trimmed chimeric parts to its own targets, defined by the coordinates in .bed file. We take into account that two targets from different pools can intersect and the potential presence of primers that were not digested. We form a list of targets that has an intersection of at least one nucletide with current read and then increase the coverage of targets only from one pool.
3. We form reference from the reads that were mapped on targets. We count the occurence of each nucleotide in each position and then create the sequences that represent our targets using the following principle:
   (a) If the current nucleotide occured in more then x percents of total coverage of this region, we use it as a “true” nucleotide of our reference, created based on sample's DNA. (We have choosen $x = 60$).
   (b) If there are two nucleotides that occures in more then y, but less then x percents of total coverage, we mark this nucleotide with N symbol. It represents the site of heterozygosity, and mapping of reads on such created “on-line” references is more accurate because it takes the individual variants into account. (We have choosen $y = 40$).
   (c) We reduce the size of homopolymer parts of reads to k symbols, we have choosen $k = 4$.
4. Finally we use local alignment algorithm to align our chimeric parts that are longer that l bp of reads on these targets generated from reference's reads, using specific scoring scheme (it is possible to make 10% of total length of chimeric read of mismatches and gaps, but the alignment of any nucleotide to “N” symbol is free of penalties). We have choosen $l = 30$.

1.2 Coverage of samples within one run of sequencing

Coverage shows high level of variability in both variance and mean value between amplicons within one run of sequencing, which makes the characterization of the panel using average coverage non-informative.

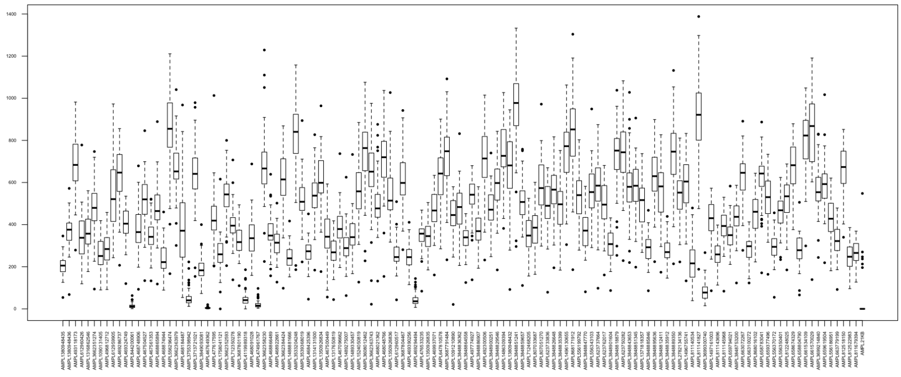

1.3 Results based on populational frequencies

We have 552 samples, only 507 of them passed our QC control. 244 of these 507 were compund heterozygous patients diagnosed with cystic fibrosis or phenylketonuria, 33 of samples have only one detected pathogenic SNP and 230 were obtained from people who were not diagnosed with these genetic diseases or have unknown status. Using the conservative estimation of each disease's carrier frequency less than one in 25 patients in Slavic population and the estimation of CNVs' frequencies of all of diseases approximately equal to the frequency of CNVs in cystic fibrosis that was estimated as 1.5% (Aurlie Vasson et al., "Custom oligonucleotide array-based CGH: a reliable diagnostic tool for detection of exonic copy-number changes in multiple targeted genes", *European Journal of Human Genetics* (2013) **21**, 977987), we can conclude that the probability of having 0 samples with CNV in the cohort of 230 relatively healthy or unknown samples can be estimated using Binomial distribution (with following R code):

```
> pbinom(0, 230, 3/25 * 0.015) ~ 0.661$.
```

So we assumed that there is no other samples that carry CNVs in our dataset.

1.4 On false positive results

In case we have detected a deletion and it was not confirmed by biochemical methods, then it is a false positive result (except the case that the only deletion we detect in sample is located in intronic region and was covered with only one amplicon).

Several false positive results (typically false positives that involves the whole gene) were produced by samples with low quality of DNA preparation and low DNA concentration. This samples were not filtered out by quality control due the fact that it controls the quality inside each gene, but such false positives are undistinguishable from true positive CNV.

Also several short false positive results (typically, exons covered with 1 amplicon) were arised in all technical replicates of particular samples so we assumed that they were produced by point mutations and indels in primer binding sites. It was proven for at least 2 of false positive cases (one point mutation and one large indel in primer binding site), but we were not able to make any conclusions about other cases because only small part of amplicons have overlaps in our panels which makes detection of such variation impossible (using our data).

1.5 On comparison with cn.mops and ONCOCNV

We have tested cn.mops in both exonic and whole-genome version. Surprisingly, whole-genome version worked better for our data, but the copy numbers

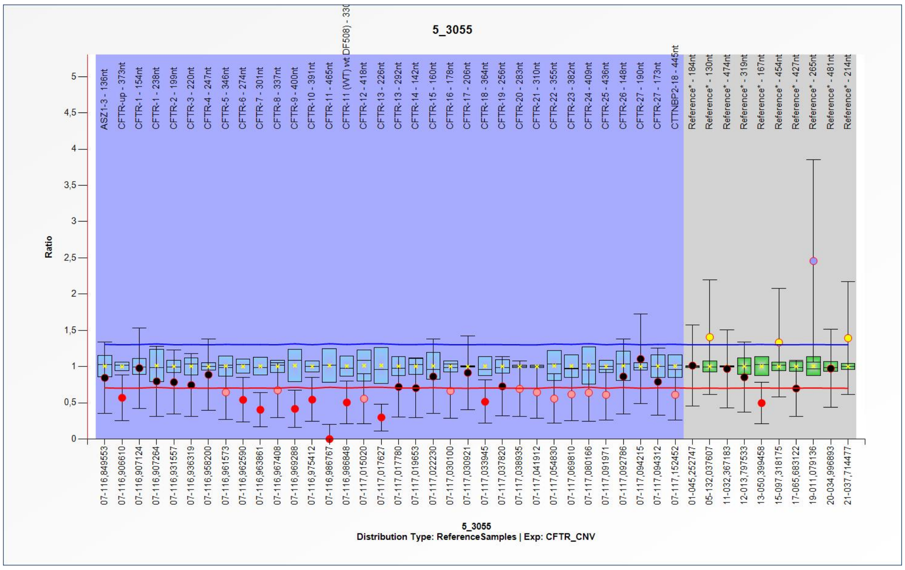

Fig. 1 Result of MLPA analysis of one of False Positive results, CFTR gene.

depicted on the plots and in the final report of the tool was different. We decided to detetrmine copy number states empirically (substantial segment with large deviation from 0 was considered as the indicator of CNV) since the sensitivity of the tool based on final report was extremely low (less than 20%).

The similar situation happen with ONCOCNV: the criteria of statistical significance was too strict so we had only 1 true positive result as significant in the final report. We contacted the author and decided to use intermediate report for CNV detection.

1.6 CNV verification

Multiplex ligation-dependent probe amplification (MLPA) was used for CNV verification in the CFTR gene. The MLPA was performed to screen exons of the CFTR gene using the SALSA MLPA probe set P091 (MRC-Holland, Amsterdam, The Netherlands) according to the manufacturers instructions.

The amplified products of MLPA reaction were separated using capillary electrophoresis on an ABI 3500 Genetic Analyzer. The area under the peak for each amplified fragment was measured and normalized in comparison with the peak areas of normal control individuals using Coffalyzer.Net software(MRC-Holland).

For CNV verification in regions with known breakpoints (CFTR exon 2-3 deletion, PAH exon 5 del4232ins268) Sanger sequencing was used. Two primer pairs flanking deletion boundaries were designed in such a way to get fusion amplicon in case of deletion. Resulting PCR products were analyzed using Sanger sequencing.

So in case of CFTR exon 2-3 deletion two amplicons (312 and 685 b.p). are expected in wild type samples, one amplicon (468 b.p) in case of homozygote deletion, and all three amplicons (312, 685 and 468 b.p) in case of heterozygote deletion. In case of PAH exon 5 del4232ins268 mutation wild type results in 4512 b.p. fragment, whereas the deletion carriers amplification results in 548 b.p. amplicon.

Sanger sequencing was performed in Evrogen company (Moscow, Russia) using 3500 AB capillary sequencer.

1.7 Unsupervised stage pseudocode

Data: coverages for pairs sample - amplicon;
Arguments *L - maximum number of models to compare with; M - minimum number of models for amplicon for QC control; N - minimum number of models required for current pair (Amplicon - Sample) for outlier detection.*
Result: information about CNVs
foreach *pair of amplicons* $A_1 \neq A_2$ **do**
    Calculate $cor(\log(cov(A_1)), \log(cov(A_2))$ and push it in priority queue Q;
end
foreach *amplicon Y in BED file* **do**
    $counter := 0$
    while *$counter < L$ and correlation $> C$* **do**
        Pop another amplicon X from priority queue Q that is located far from current (the distance between amplicons is bigger than expected size of CNV, usually, located on other gene); $counter++$;
        Constuct robust linear models $\log(cov(Y)) = \alpha \log(cov(X)) + \beta$, $\log(\frac{cov(Y)}{2}) = \alpha \log(cov(X)) + \beta$, $\log(\frac{3cov(Y)}{2}) = \alpha \log(cov(X)) + \beta$;
        Calcualte studentized residuals of the linear models;
        Detect all the residuals of the first model that 1) lie below (or higher) the choosen quantile of Student's t-distribution, 2) corresponding residuals for the second and third models are less than for the first.
    end
    if *$counter < M$* **then**
        Exclude this amplicon from the further analysis (QC control)
    end
    foreach *sample S* **do**
        if *Y was detected as more close to second or third model and an outlier for the first model in a sample S more than N times* **then**
            There is a CNV in sample S and amplicon Y.
        end
    end
end

Algorithm 1: Unsupervised stage of detection of CNVs
