# Supplementary material for: A statistical approach to detection of copy number variations in PCR-enriched targeted sequencing data
Source: BMC Bioinformatics. 2016 Oct 22;17:429. doi: 10.1186/s12859-016-1272-6 (PMC5075217; doi:10.1186/s12859-016-1272-6)
Supplement: Additional file 1 — Auxiliary procedures and descriptions. This pdf file contains details on counting of coverages procedure, illustration of non-uniformity of coverages across samples sequenced within one experiment, explanation of assumption of CNVs absence in the dataset where we did not make direct check with alternative methods, description of potential source of false positive results, description of comparison with cn.mops and ONCOCNV. Also CNV verification procedure is described here and pseudocode of the first algorithm provided in the end. (ZIP 968 kb) [file 12859_2016_1272_MOESM1_ESM.zip › Additonal File 1/usrguid3.pdf]

The SVJour3 document class users guide Version 3.2 – for Springer journals

© 2007, Springer Heidelberg
All rights reserved.

8 May 2007

Contents

1 Introduction **1**
1.1 Overview 2
1.2 Using PostScript fonts 2

2 Initializing the class **2**

3 The article header **4**
3.1 The title 4
3.2 Authors 4
3.3 Addresses 5
3.4 Footnotes to the title block 5
3.5 Changing the running heads 6
3.6 Typesetting the header 6

4 Abstract, keywords, MSC, PACS,and CR codes **6**

5 Theorem-like structures **7**
5.1 Predefined environments 7
5.2 Defining new structures 8

6 Additional commands **9**

1 Introduction

This documentation describes the SVJour3 LaTeX 2ε document class. It is not intended to be a general introduction to TeX or LaTeX. For this we refer to [2] and [3].

SVJour3 was derived from the LaTeX 2ε `article.cls`, based on TeX version 3.141 and LaTeX 2ε. Hence text, formulas, figures and tables are typed using the standard LaTeX 2ε commands. The standard sectioning commands are also used.

The main differences to the standard article class are the presence of additional high-level structuring commands for the article header, new environments for theorem-like structures, and some other useful commands.

Please always give a `\label` where possible and use `\ref` for cross-referencing. Such cross-references will be converted to hyper-links in the electronic version. The `\cite` and `\bibitem` mechanism for bibliographic references is also obligatory.

1.1 Overview

The documentation consists of this document—which describes the whole class (i.e. the differences to the `article.cls`)—and a ready-to-use template to allow you to start writing immediately.

1.2 Using PostScript fonts

Springer journals produced in TeX are typeset using the PostScript[1] Times fonts for the main text. As the use of PostScript fonts results in different line and page breaks than when using Computer Modern (CM) fonts, we encourage you to use our document class together with the `psnfss` package `mathptmx`. This package makes all the necessary font replacements to show you the page make-up nearly as it will be printed. Ask your local TeXpert for details. PostScript previewing is possible on most systems. On some installations, however, on-screen previewing may be possible only with CM fonts.

If, for technical reasons, you are not able to use the PS fonts, it is also possible to use our document class together with the ordinary Computer Modern fonts. Note, however, that in this case line and page breaks will change when we reTeX your file with PS fonts, making it necessary for you to check them again carefully once you receive the proofs from the printer.

2 Initializing the class

To use the document class, enter

[1] PostScript is a trademark of Adobe.

\documentclass [⟨*format,other options*⟩] {svjour3} [⟨*release-date*⟩]

at the beginning of your article. The first option [⟨*format*⟩] is required and should be set according to the journal for which you are planning to submit a contribution. Three formats are available. The format is pre-set in the template, but choose the one that suits your specific journal if there is no journal-specific template available for your journal.

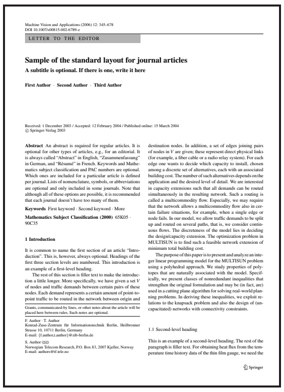

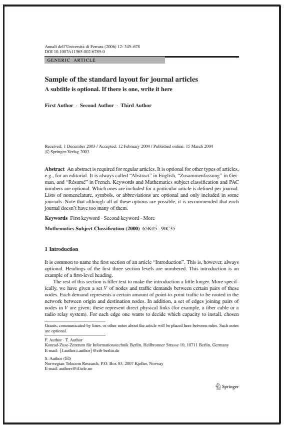

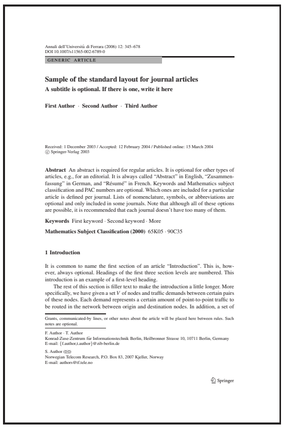

`[twocolumn]` `[smallcondensed]` (default) `[smallextended]`

available `format` options (samples not in full-scale)

There is one general option [⟨*glov3*⟩] that is auto-activated if no special option for the particular journal exists or is given. This option causes LaTeX to read in the class option file `svglov3.clo` (part of the package). Do not try to use those options of the old SVJour classes version 1 and 2 as these are not suitable for SVJour3—you will get a class error, tops.

Other options, valid for every journal, are

draft	to make overfull boxes visible,
final	the opposite, and
referee	required to produce a hardcopy for the referee with a special layout (bigger interline spacing).

The next four additional options control the automatic numbering of figures, tables, equations, and theorem-like environments. The fifth option described below disables the "Springer" theorems (see also Sect. 5). The last option describes the natbib package.

numbook	"numbering like the standard book class"—prefixes all the numbers mentioned above with the section number,
envcountsect	the same for theorem like environments only,
envcountsame	uses one counter for all theorem-like environments,
envcountreset	resets the theorem counter(s) every new section,

nospthms use it *only* if you want to suppress all Springer theorem-like environments (see Sect. 5) and use the theorem environments of original LaTeX package or other theorem packages instead. (Please check this with your editor.)

natbib handles reference entries in the author-year system (with or without BibTeX) by using the natbib package of Patrick W. Daly. It can be found at the *Comprehensive TeX Archive Network* (CTAN...tex-archive/macros/latex/contrib/supported/natbib/), see [4, 5, 6].

If a journal contains articles in languages other than English the class provides two options "`[deutsch]`" and "`[francais]`" that automatically translate supplied texts or phrases given from LaTeX.

There may be additional options for a specific journal—please refer to the extra documentation or to the template file.

As an example, we show how to begin a document for a two-column journal produced in draft mode:

```
\documentclass[twocolumn,draft]{svjour3}
```

3 The article header

In this section we describe the usage of the high-level structuring commands for the article header. Header in this context means everything that comes before the abstract.

3.1 The title

The commands for the title and subtitle of your article are

```
\title {⟨your title⟩}
\subtitle {⟨your subtitle⟩}
```

You can also dedicate your article to somebody by specifying

```
\dedication{⟨dedication⟩}
```

3.2 Authors

Information about the authors is provided with

\author {⟨*author name [\and author name]* ⟩}

If there is more than one author, the names should be separated by `\and`. To make this clear, we provide an example:

```
\author{John B. Doe \and Sally Q. Public \and Joe A. Smith}
```

3.3 Addresses

Address information is marked with

\institute {⟨*address information [\and address information]*⟩}

If there is more than one address, the entries are separated by `\and`.

As the address of the author appears as a footnote on the first page of your article, the author name is to be repeated in the address information with an `\at` depicting the affiliation. Addresses should be contained in one line, using commas to separate the parts of the address. In addition, you can use

\email {⟨*email address*⟩}

to provide an email address within `\institute`.

If there are authors appearing with different addresses the affiliations can be indicated with the same author listed "`\at`" (i.e. before) each particular address in the `\institute{...}` field—authors in such lists (read: at the same address) should again be separated by an `\and`.

To continue the example above, we could say

```
\institute{J.B. Doe
           \at Doe Institute, 281 Prime Street, Daisy Town, NA 02467,
               USA\\Tel.: +127-47-678901, Fax: +127-47-678907
        \and
        J.B. Doe \and S.Q. Public
           \at Public-Enterprises
        \and
        J.A. Smith
           \at Smith University,\\\email{smith@smith.edu}
        }
```

3.4 Footnotes to the title block

If footnotes to the title, subtitle, author's names or institute addresses are needed, please code them with

```
\thanks {⟨text of footnote⟩}
```

immediately after the word in the corresponding field. Please note that these footnotes are not marked—they will appear above the address information at the bottom of the first page, enclosed in rules.

3.5 Changing the running heads

Normally the running heads—if present in the specific journal—are produced automatically by the `\maketitle` command using the contents of `\title` and `\author`. If the result is too long for the page header (running head) the class will produce an error message and you will be asked to supply a shorter version. This is done using the syntax

```
\titlerunning{⟨text⟩}
\authorrunning{⟨first author et al.⟩}
```

These commands must be entered before `\maketitle`.

3.6 Typesetting the header

Having entered the commands described in this section, please format the heading with the standard `\maketitle` command. If you leave it out, the work done so far will produce *no* text.

4 Abstract, keywords, MSC, PACS, and CR codes

The environment for the abstract is the same as in the standard article class. To insert keywords, a "Mathematics Subject Classification" (MSC), "Physics and Astronomy Classification Scheme" (PACS), or "ACM Computing Classification" (CR) codes you should use

```
\keywords{⟨keywords⟩}
\subclass{⟨MSC codes⟩}
\PACS{⟨PACS codes⟩}
\CRclass{⟨CR codes⟩}
```

at the end—but still inside—of the abstract environment; the individual words or codes should be separated by `\and`.

Some journals published in other languages than English reapeat those elements in translation at the end of the header material before the actual article starts.

Please use the following environment for that and give the relevant codes (MSC, PACS, CR) only in the translated abstract (see also the particular template file)

```
\begin{translation}{english}
  \begin{abstract}
    ...
  \end{abstract}
\end{translation}
```

5 Theorem-like structures

5.1 Predefined environments

In the SVJour3 document class the functions of the standard `\newtheorem` command have been enhanced to allow a more flexible font selection. All standard functions though remain intact (e.g. adding an optional argument specifying additional text after the environment counter). To typeset environments such as definitions, theorems, lemmas or examples, we have predefined the environments in the list below. Note that the font selection of environment heading vs. its body font is depicted in this list with

environment name	=	**bold heading**	*italic text body*
**environment name**	=	**bold heading**	normal text body
environment name	=	*italic heading*	normal text body

Unnumbered environments will be produced by:
claim and *proof*.

Numbered environments will be produced by:
theorem, ***proposition***, ***lemma***, ***corollary***,
definition, **exercise**, **problem**, **solution**,
remark, *note*, *case*, *conjecture*, *example*, *property*, and *question*.

The syntax is exactly the same as described in [3, Sect. 3.4.3]:

```
\begin{⟨environment⟩}[⟨name⟩]
...
\end{⟨environment⟩}
```

where the optional *name* is often used for the common name of the theorem:

```
\begin{theorem}[Church, Rosser]
...
\end{theorem}
```

Sometimes the automatic braces around the optional argument are unwanted (e.g. when it consists only of a reference made with `\cite`). Then you can wrap the whole theorem-like structure in a `theopargself` environment. It suppresses the braces and gives you complete control over the optional argument, e.g.:

```
\begin{theopargself}
   \begin{theorem}[\cite{Church,Rosser}]
   ...
   \end{theorem}
\end{theopargself}
```

5.2 Defining new structures

For cases where you do not find an appropriate predefined theorem-like structure above, we provide two mechanisms to define your own environment. Use

```
\spdefaulttheorem{⟨env name⟩}{⟨caption⟩}{⟨cap font⟩}{⟨body font⟩}
```

to define an environment compliant with the selected class options (see Sect. 2) and designed as the predefined Springer theorem-like environments.

Continuative commands you can use here are

```
\spnewtheorem{⟨name⟩}{⟨label text⟩}[⟨numbered within⟩]{⟨label font⟩}{⟨body font⟩}
\spnewtheorem{⟨name⟩}[⟨numbered like⟩]{⟨label text⟩}{⟨label font⟩}{⟨body font⟩}
```

There is also a starred version, without optional arguments, which provides a theorem environment without numbers. Here *name* is the name of the environment, *label text* is the text to be typeset as heading, and the *label font* and *body font* are the font for the label text and the theorem body.

If you use the *numbered within* argument, the new structure will be numbered within the specified sectional unit—if you specify *numbered like*, it shares its numbering sequence with the referenced structure.

For instance, the predefined environments *theorem* and *proof* are defined as

```
\spnewtheorem{theorem}{Theorem}{\bf}{\it}
\spnewtheorem*{proof}{Theorem}{\it}{\rm}
```

whereas one could define a theorem-like structure *algorithm*, numbered within the current section as

```
\spnewtheorem{algorithm}{Algorithm}[section]{\bf}{\rm}
```

It is also possible to skip all theorem features of the SVJour3 document class (see Sect. 2) and/or to use the *theorem* package shipped with LaTeX 2_ε (see [1] for a complete description) or the *amsthm* package of AMSLaTeX to define new theorem environments. But note that once you use them you should not change the predefined structures.

6 Additional commands

We provide some additional useful commands which you can use in your manuscript. The first is the *acknowledgements* environment

```
\begin{acknowledgements}
...
\end{acknowledgements}
```

which is usually used as the last paragraph of the last section.

The next is an enhancement of the standard `\caption` command used inside of *figure* environments to produce the legend. The added command

```
\sidecaption
```

can be used to produce a figure legend beside the figure. To activate this feature you have to enter it as the very first command inside the *figure* environment

```
\begin{figure}\sidecaption
  \resizebox{0.3\hsize}{!}{\includegraphics*{figure.eps}}
  \caption{A figure}
\end{figure}
```

If there is not enough room for the legend the normal `\caption` command will be used. Also note that this works only for captions that come *after* the included images.

We also have enhanced the *description* environment by an optional parameter, which lets you specify the largest item label to appear within the list. The syntax now is

```
\begin{description}[⟨largelabel⟩]
...
\end{description}
```

The texts of all items are indented by the width of *largelabel* and the item labels are typeset flush left within this space. Note: The optional parameter will work only two levels deep.

The often missed command

```
\qed
```

yields the known □ symbol with appropriate spacing to close e.g. a proof, use the new declaration

```
\smartqed
```

to move the position of the predefined qed symbol to be flush right (in text mode). If you want to use this feature throughout your article the declaration

must be set in the preamble; otherwise it should be used individually in the relevant environment, i.e. proof. □

The last two commands working as markup in

```
\vec{⟨symbol⟩}
\tens{⟨symbol⟩}
```

mark vectors (e.g. $\boldsymbol{S}$, or $\mathbf{S}$) and tensors (e.g. S) respectively.

References

1. Mittelbach F., Goossens, M.: The LaTeX Companion, 2nd edn. Addison-Wesley, Boston, Massachusetts (2004)
2. Knuth D.E.: The TeXbook (revised to cover TeX3). Addison-Wesley, Reading, Massachusetts (1991)
3. Lamport L.: LaTeX: A Document Preparation System, 2nd edn. Addison-Wesley, Reading, Massachusetts (1994)
4. TeX Users Group (TUG), http://www.tug.org
5. Deutschsprachige Anwendervereinigung TeX e.V. (DANTE), Heidelberg, Germany, http://www.dante.de
6. UK TeX Users' Group (UK-TuG), http://uk.tug.org
