# Supplementary figures and images for: A statistical approach to detection of copy number variations in PCR-enriched targeted sequencing data
Source: BMC Bioinformatics. 2016 Oct 22;17:429. doi: 10.1186/s12859-016-1272-6 (PMC5075217; doi:10.1186/s12859-016-1272-6)

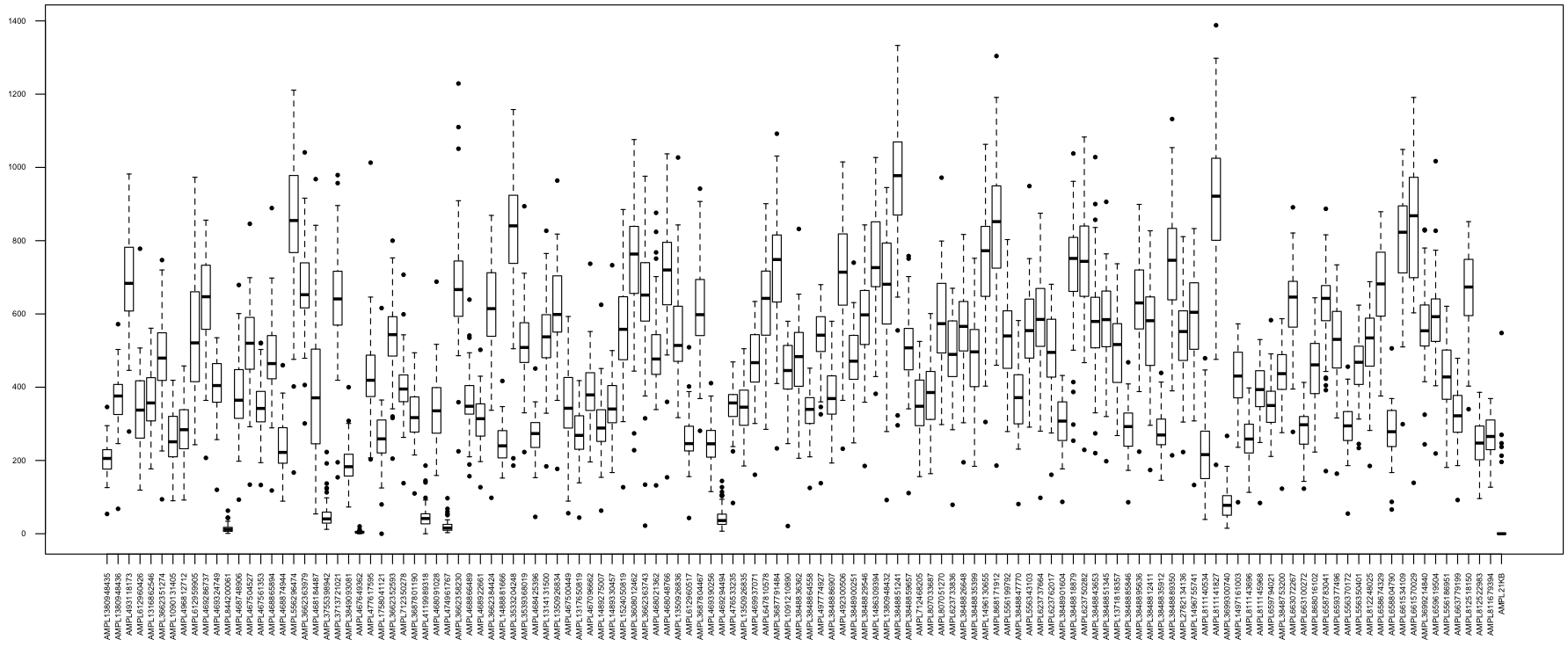

Supplement: Additional file 1 — Auxiliary procedures and descriptions. This pdf file contains details on counting of coverages procedure, illustration of non-uniformity of coverages across samples sequenced within one experiment, explanation of assumption of CNVs absence in the dataset where we did not make direct check with alternative methods, description of potential source of false positive results, description of comparison with cn.mops and ONCOCNV. Also CNV verification procedure is described here and pseudocode of the first algorithm provided in the end. (ZIP 968 kb) [file 12859_2016_1272_MOESM1_ESM.zip › Additonal File 1/coverage.png]

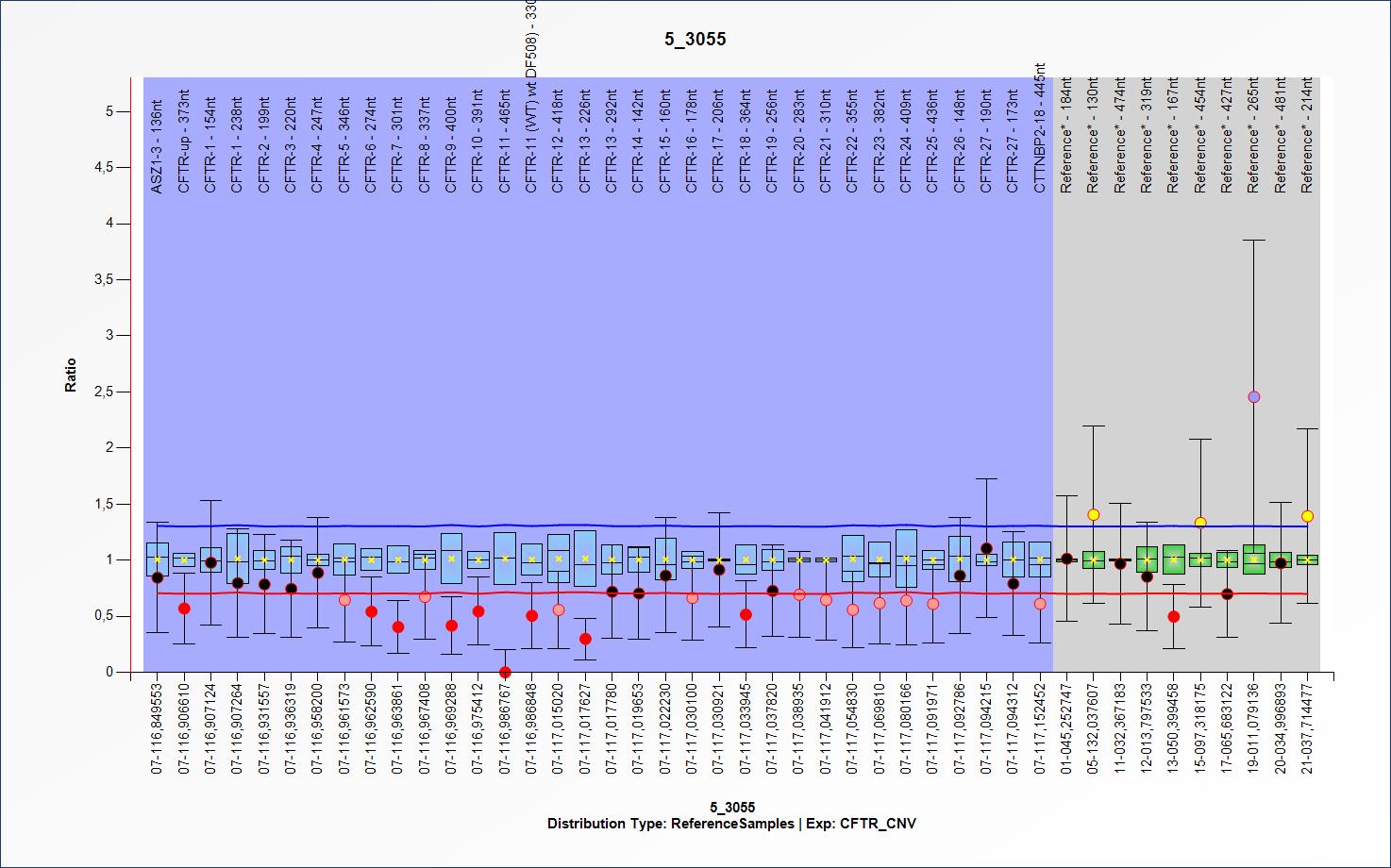

Supplement: Additional file 1 — Auxiliary procedures and descriptions. This pdf file contains details on counting of coverages procedure, illustration of non-uniformity of coverages across samples sequenced within one experiment, explanation of assumption of CNVs absence in the dataset where we did not make direct check with alternative methods, description of potential source of false positive results, description of comparison with cn.mops and ONCOCNV. Also CNV verification procedure is described here and pseudocode of the first algorithm provided in the end. (ZIP 968 kb) [file 12859_2016_1272_MOESM1_ESM.zip › Additonal File 1/mlpa.jpg]

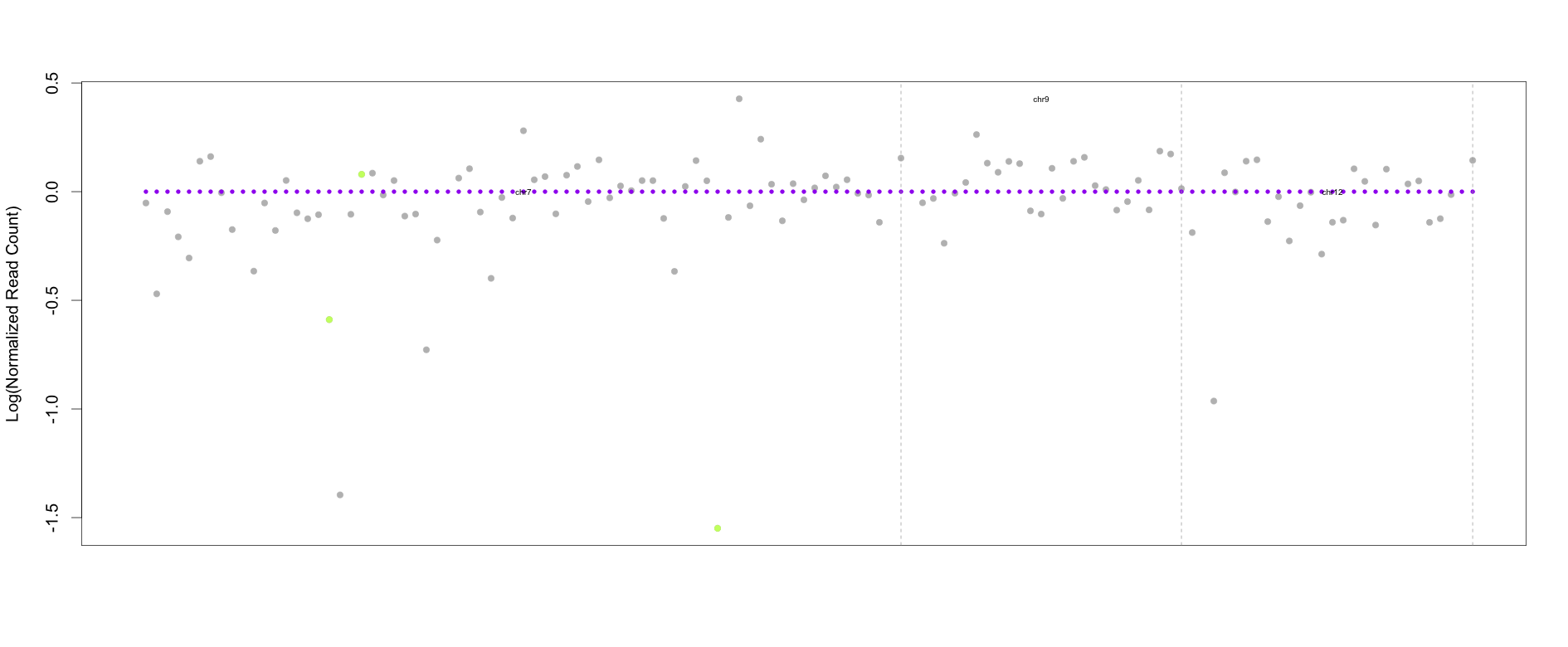

Supplement: Additional file 6 — Archive with ONCOCNV results. Set of files (png plots and txt files) in zip archive that were provided by ONCOCNV after specificity and sensitivity testing. (ZIP 5007 kb) [file 12859_2016_1272_MOESM6_ESM.zip › outputONCOCNV copy 2/B02.profile.png]

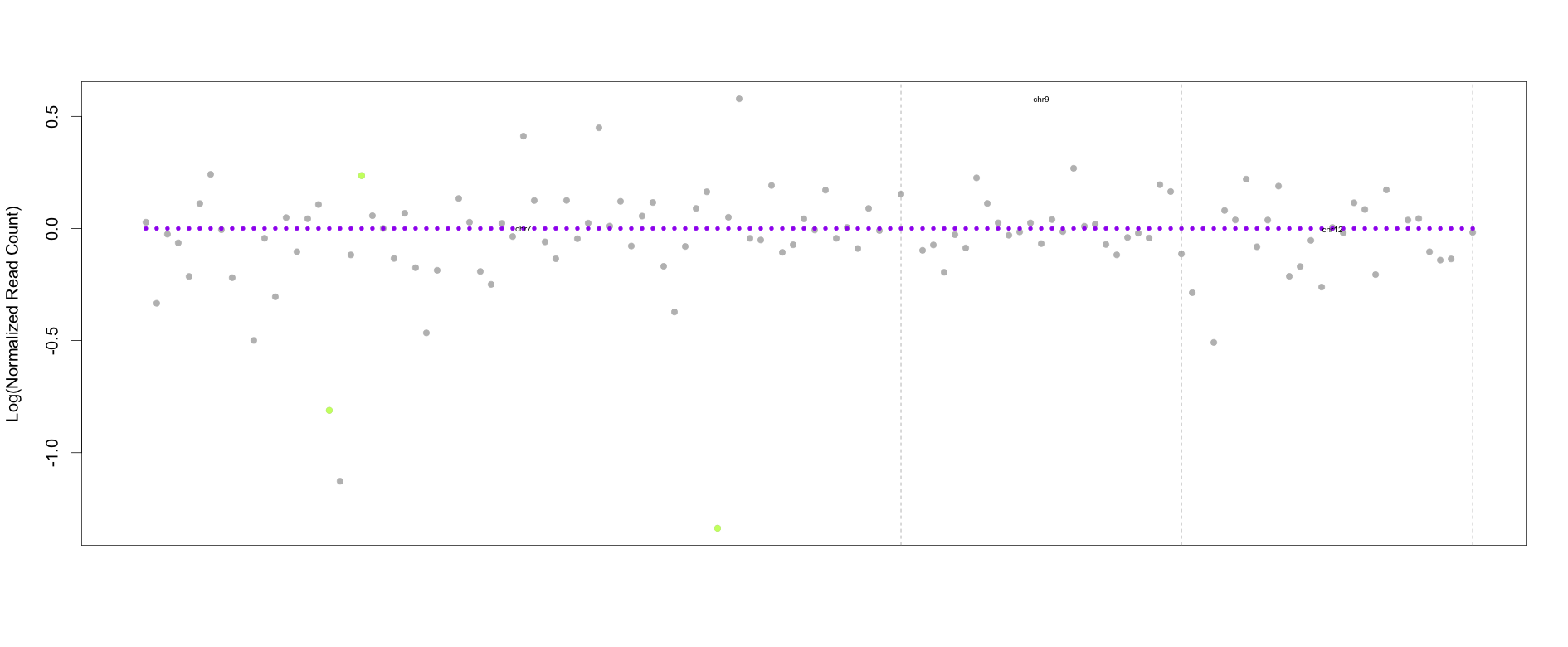

Supplement: Additional file 6 — Archive with ONCOCNV results. Set of files (png plots and txt files) in zip archive that were provided by ONCOCNV after specificity and sensitivity testing. (ZIP 5007 kb) [file 12859_2016_1272_MOESM6_ESM.zip › outputONCOCNV copy 2/B03.profile.png]

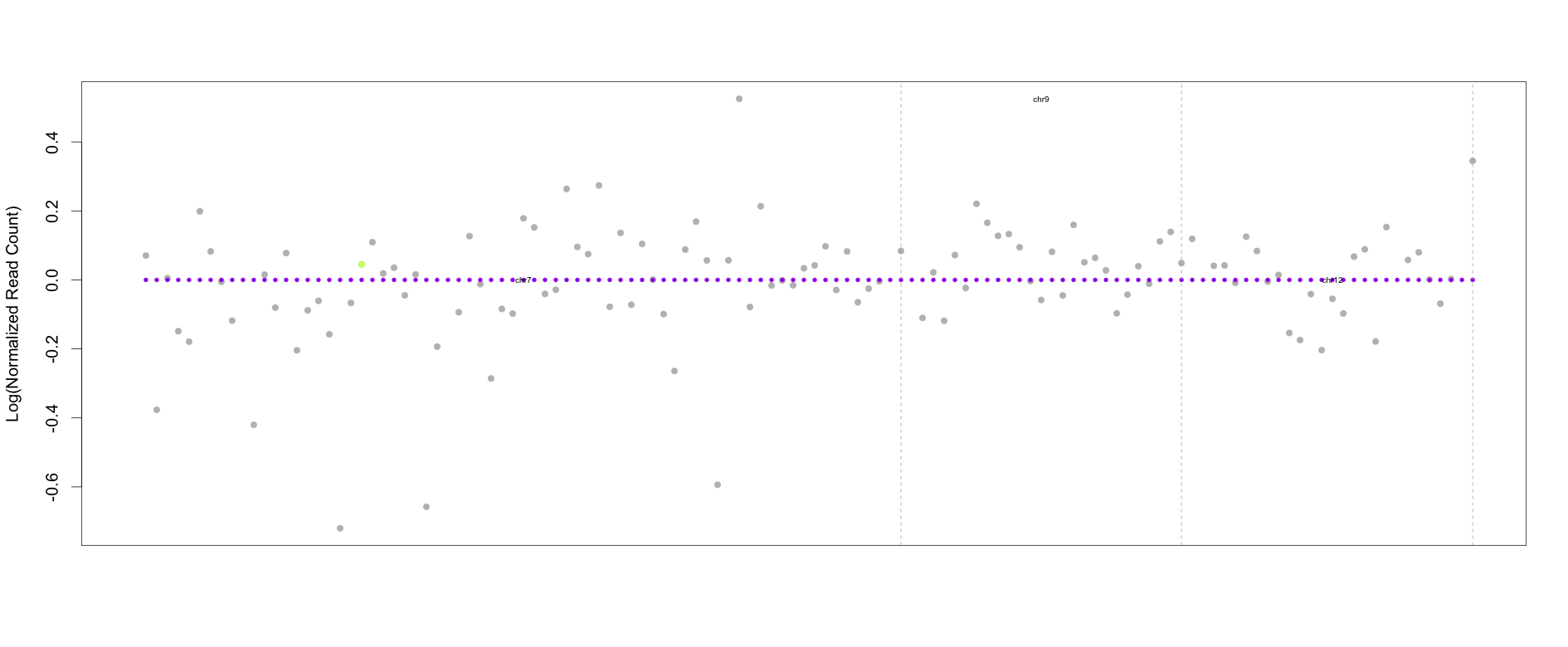

Supplement: Additional file 6 — Archive with ONCOCNV results. Set of files (png plots and txt files) in zip archive that were provided by ONCOCNV after specificity and sensitivity testing. (ZIP 5007 kb) [file 12859_2016_1272_MOESM6_ESM.zip › outputONCOCNV copy 2/B05.profile.png]

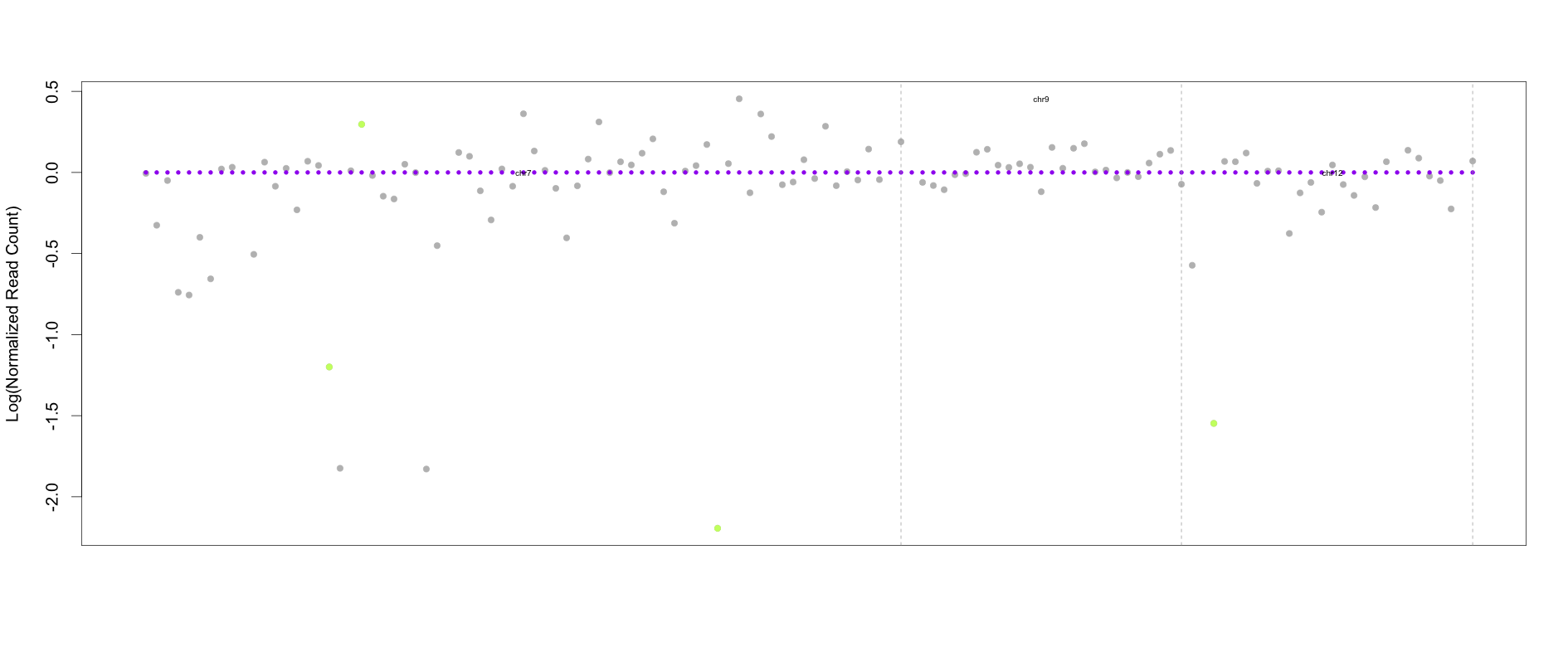

Supplement: Additional file 6 — Archive with ONCOCNV results. Set of files (png plots and txt files) in zip archive that were provided by ONCOCNV after specificity and sensitivity testing. (ZIP 5007 kb) [file 12859_2016_1272_MOESM6_ESM.zip › outputONCOCNV copy 2/B06.profile.png]

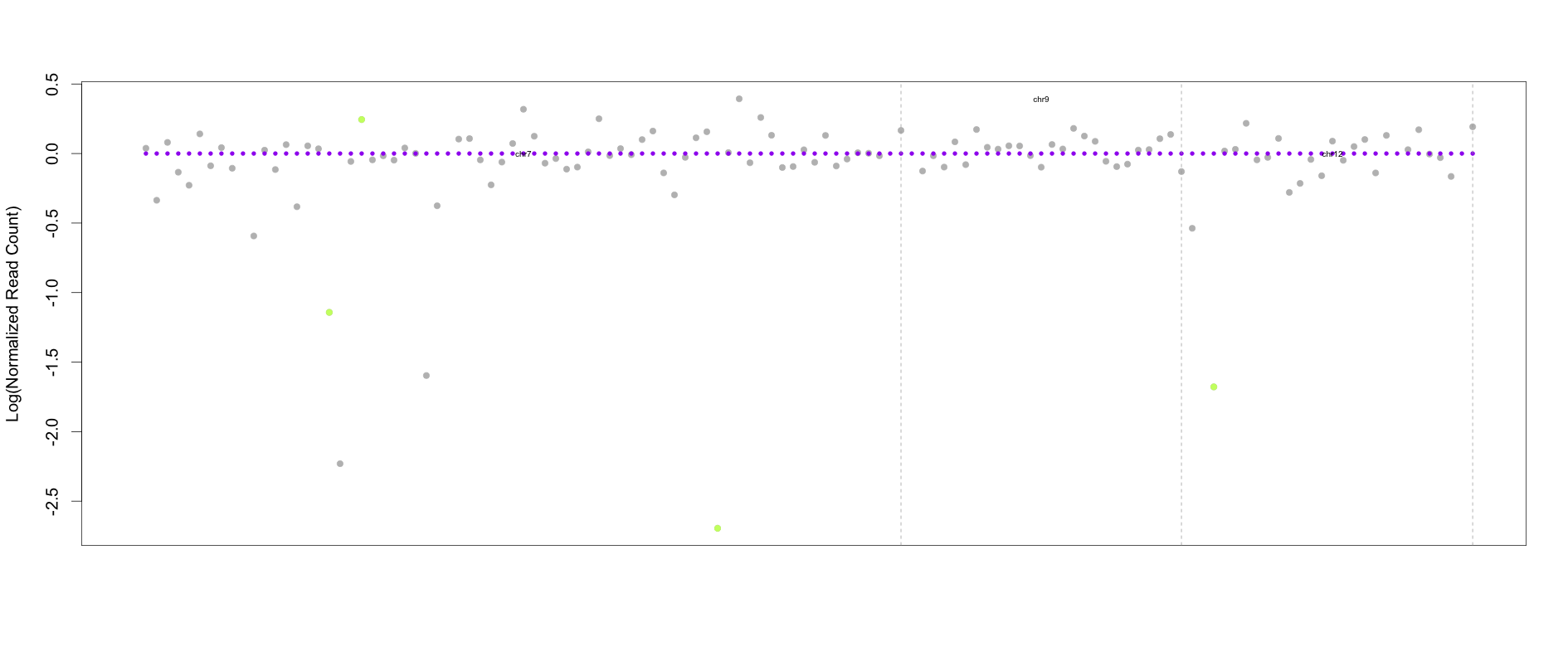

Supplement: Additional file 6 — Archive with ONCOCNV results. Set of files (png plots and txt files) in zip archive that were provided by ONCOCNV after specificity and sensitivity testing. (ZIP 5007 kb) [file 12859_2016_1272_MOESM6_ESM.zip › outputONCOCNV copy 2/B07.profile.png]

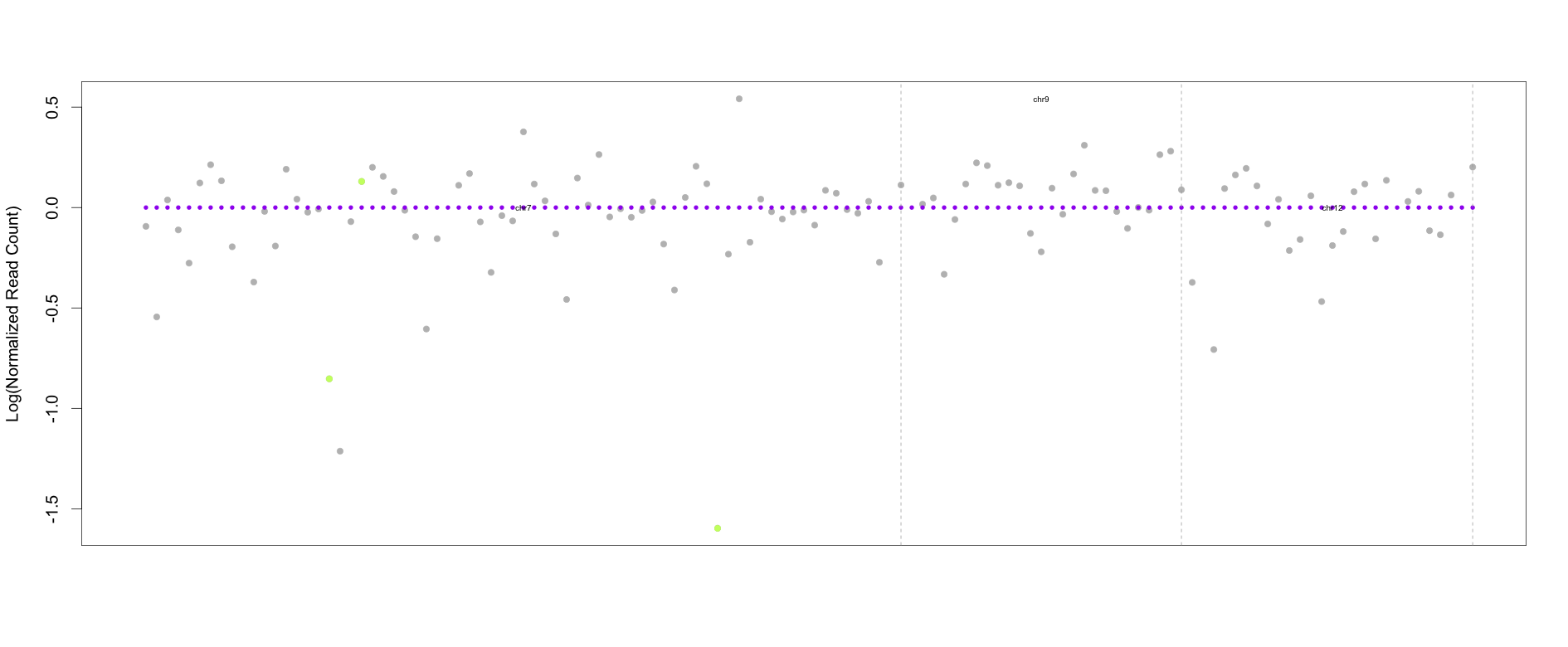

Supplement: Additional file 6 — Archive with ONCOCNV results. Set of files (png plots and txt files) in zip archive that were provided by ONCOCNV after specificity and sensitivity testing. (ZIP 5007 kb) [file 12859_2016_1272_MOESM6_ESM.zip › outputONCOCNV copy 2/B08.profile.png]

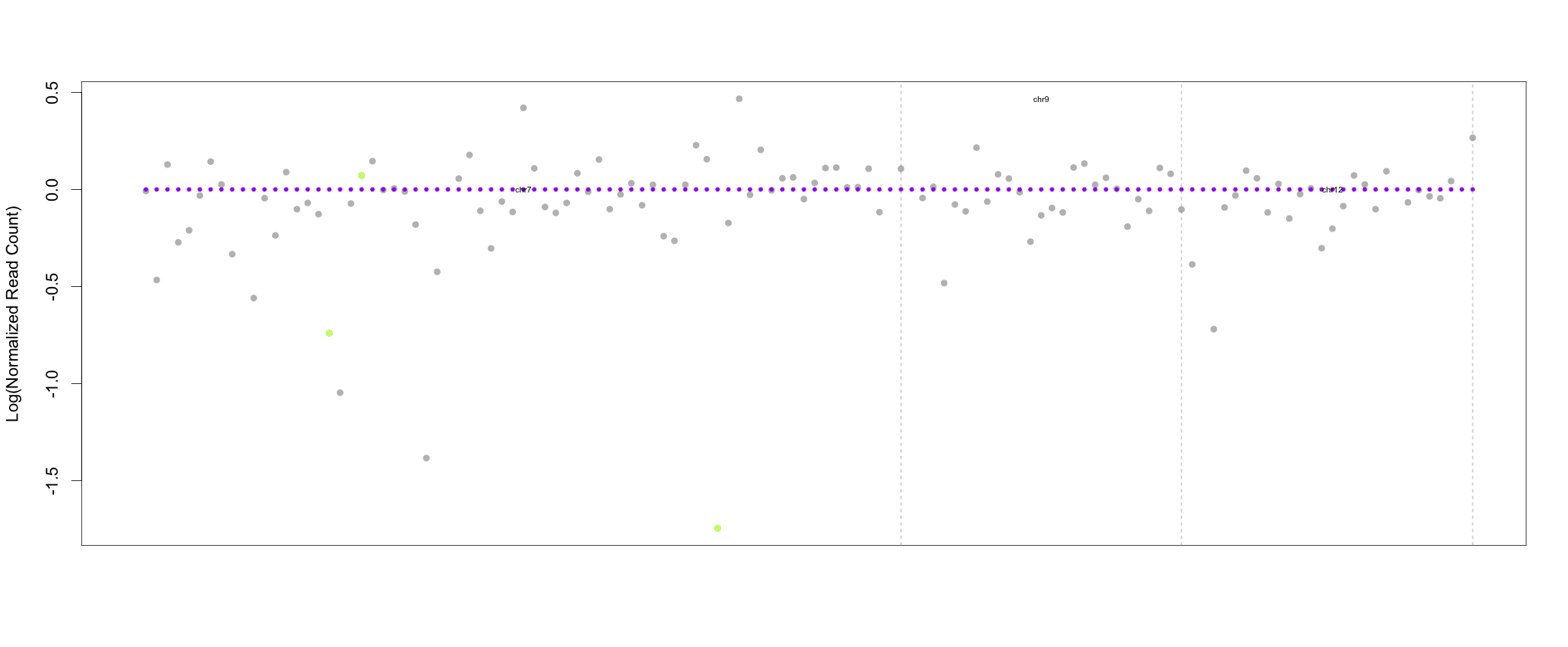

Supplement: Additional file 6 — Archive with ONCOCNV results. Set of files (png plots and txt files) in zip archive that were provided by ONCOCNV after specificity and sensitivity testing. (ZIP 5007 kb) [file 12859_2016_1272_MOESM6_ESM.zip › outputONCOCNV copy 2/B09.profile.png]

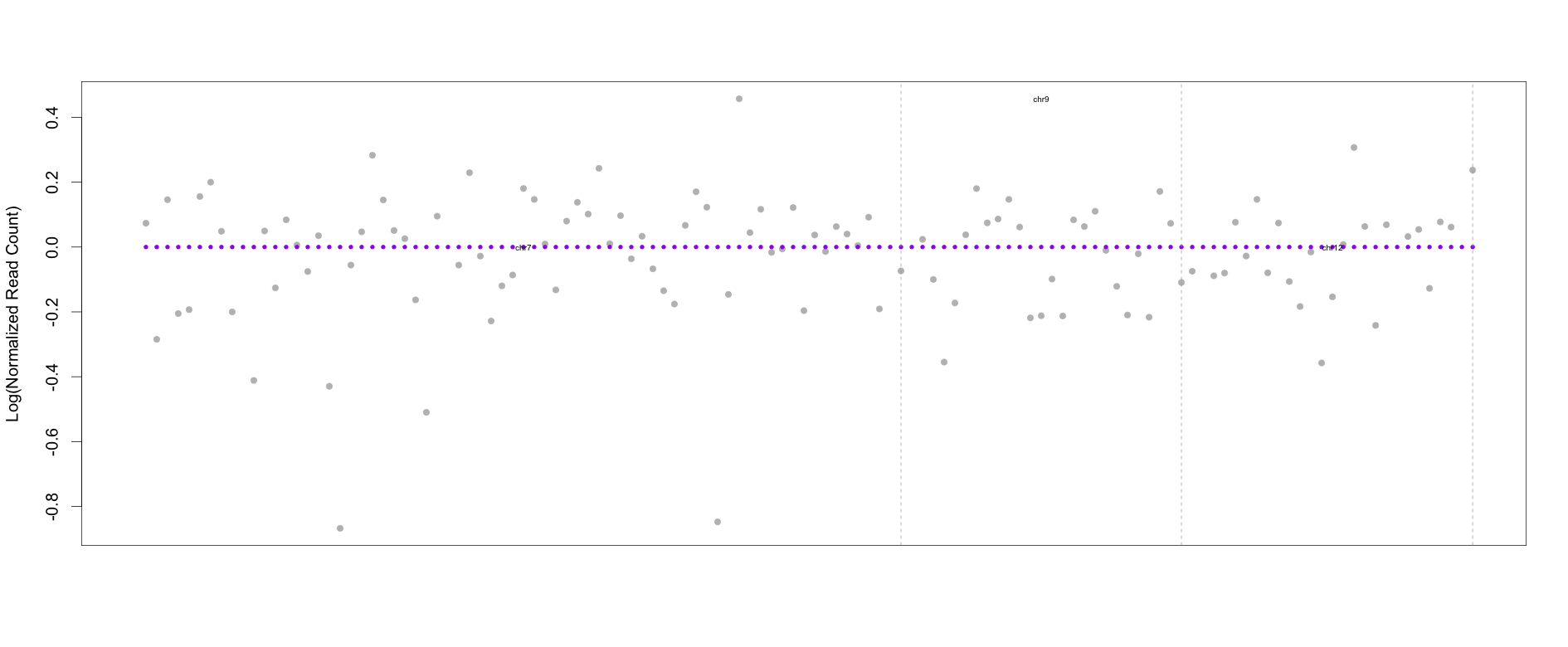

Supplement: Additional file 6 — Archive with ONCOCNV results. Set of files (png plots and txt files) in zip archive that were provided by ONCOCNV after specificity and sensitivity testing. (ZIP 5007 kb) [file 12859_2016_1272_MOESM6_ESM.zip › outputONCOCNV copy 2/B10.profile.png]

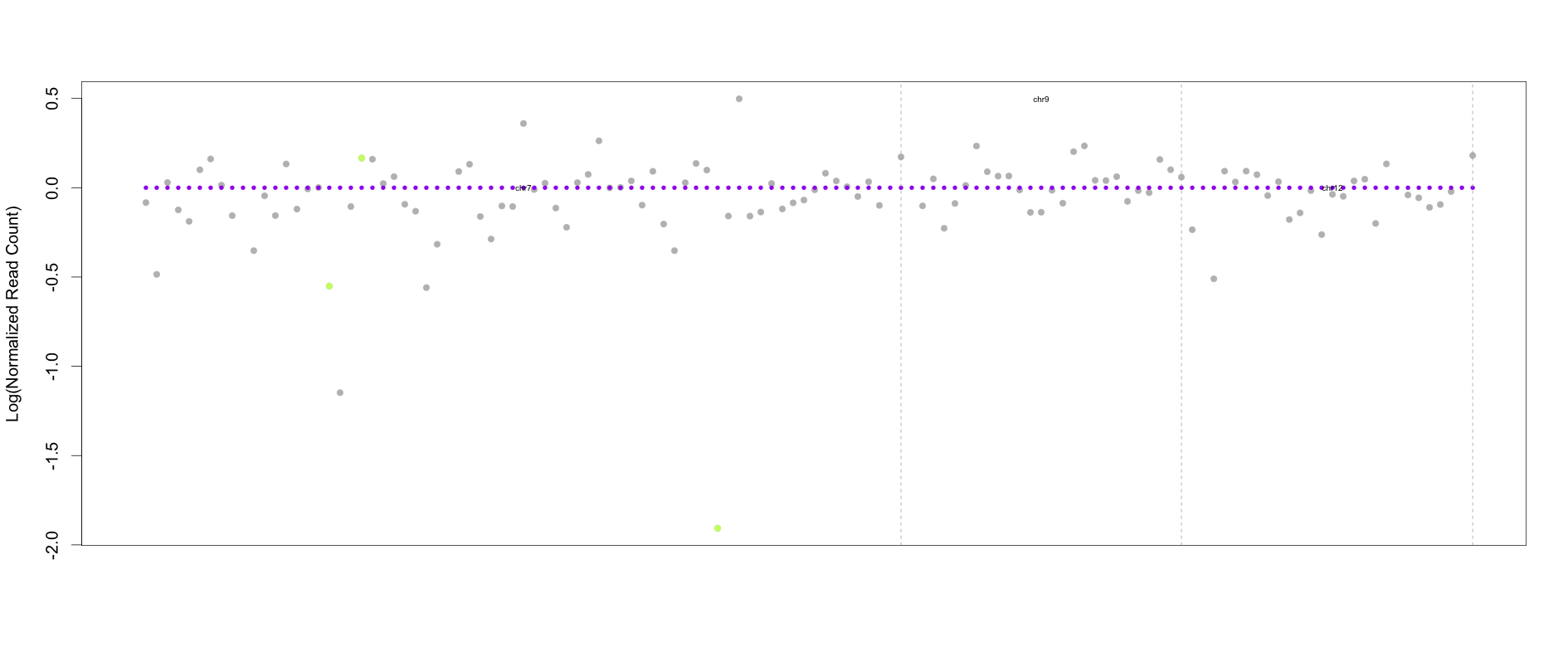

Supplement: Additional file 6 — Archive with ONCOCNV results. Set of files (png plots and txt files) in zip archive that were provided by ONCOCNV after specificity and sensitivity testing. (ZIP 5007 kb) [file 12859_2016_1272_MOESM6_ESM.zip › outputONCOCNV copy 2/B11.profile.png]

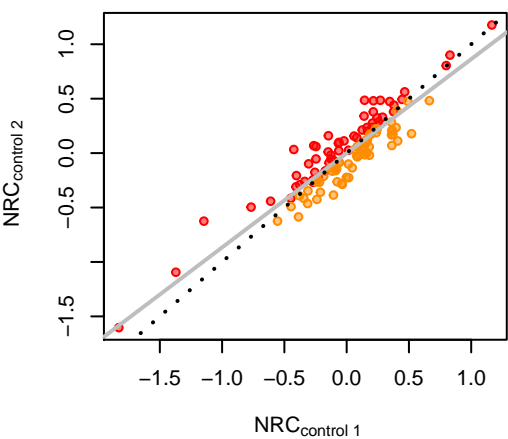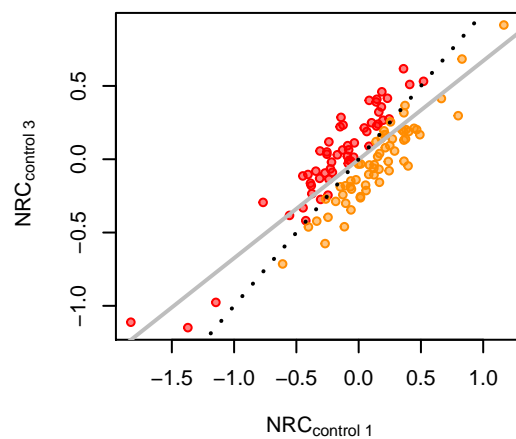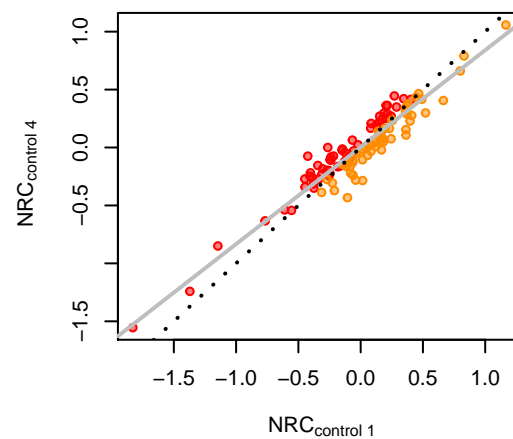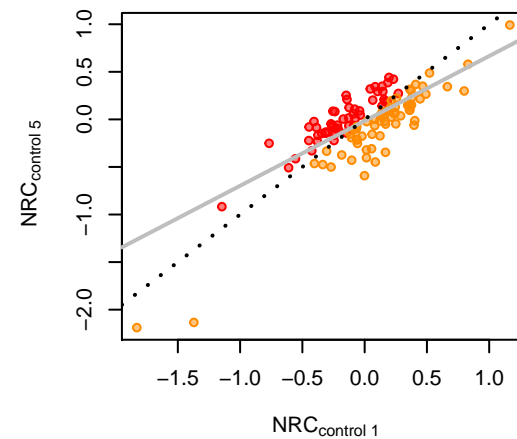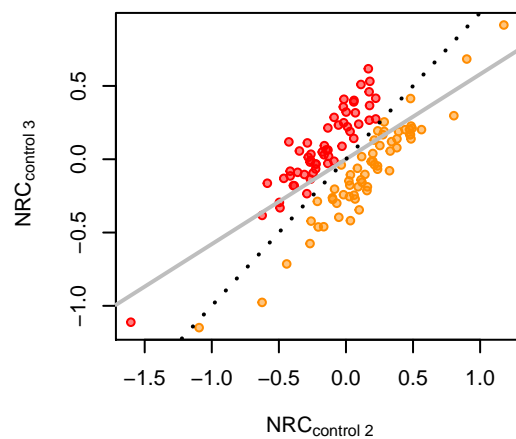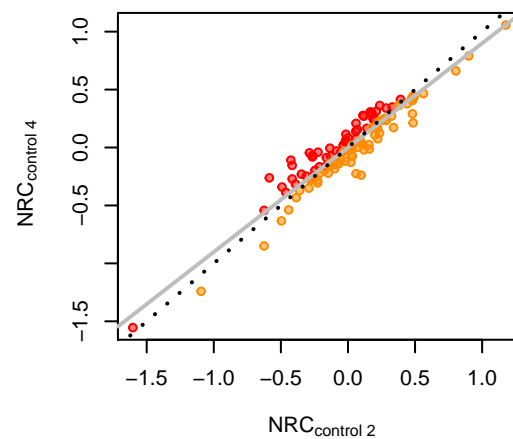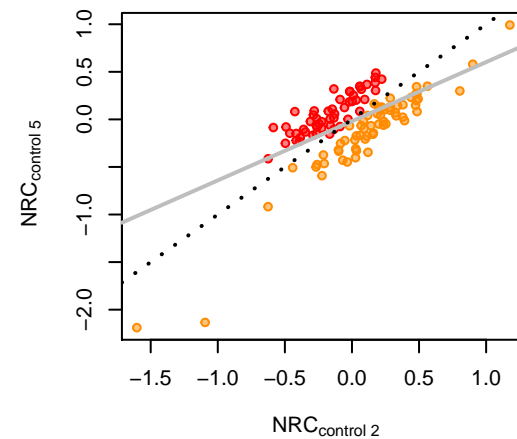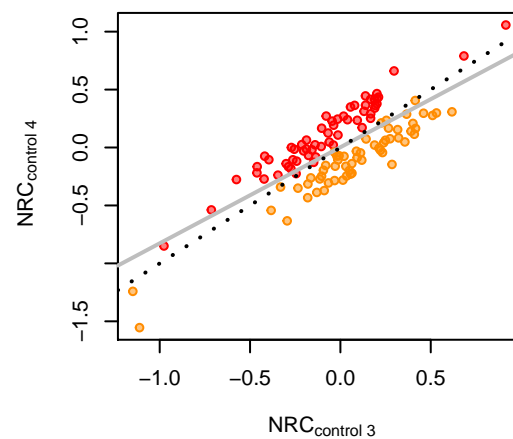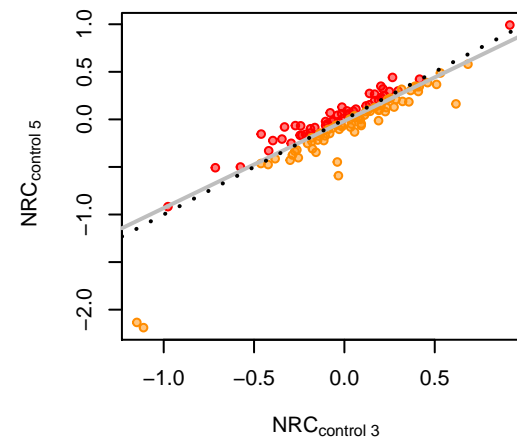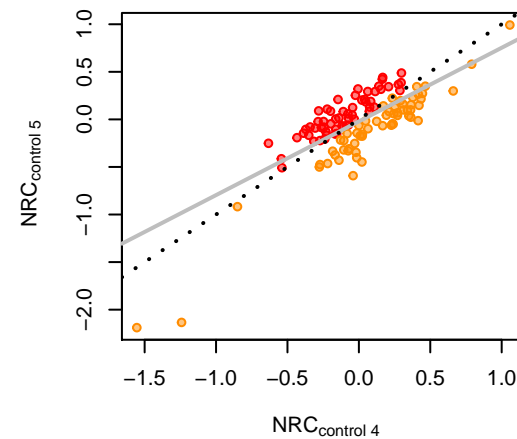

Supplement: Additional file 6 — Archive with ONCOCNV results. Set of files (png plots and txt files) in zip archive that were provided by ONCOCNV after specificity and sensitivity testing. (ZIP 5007 kb) [file 12859_2016_1272_MOESM6_ESM.zip › outputONCOCNV copy 2/Control.stats.Processed.txt.pdf]

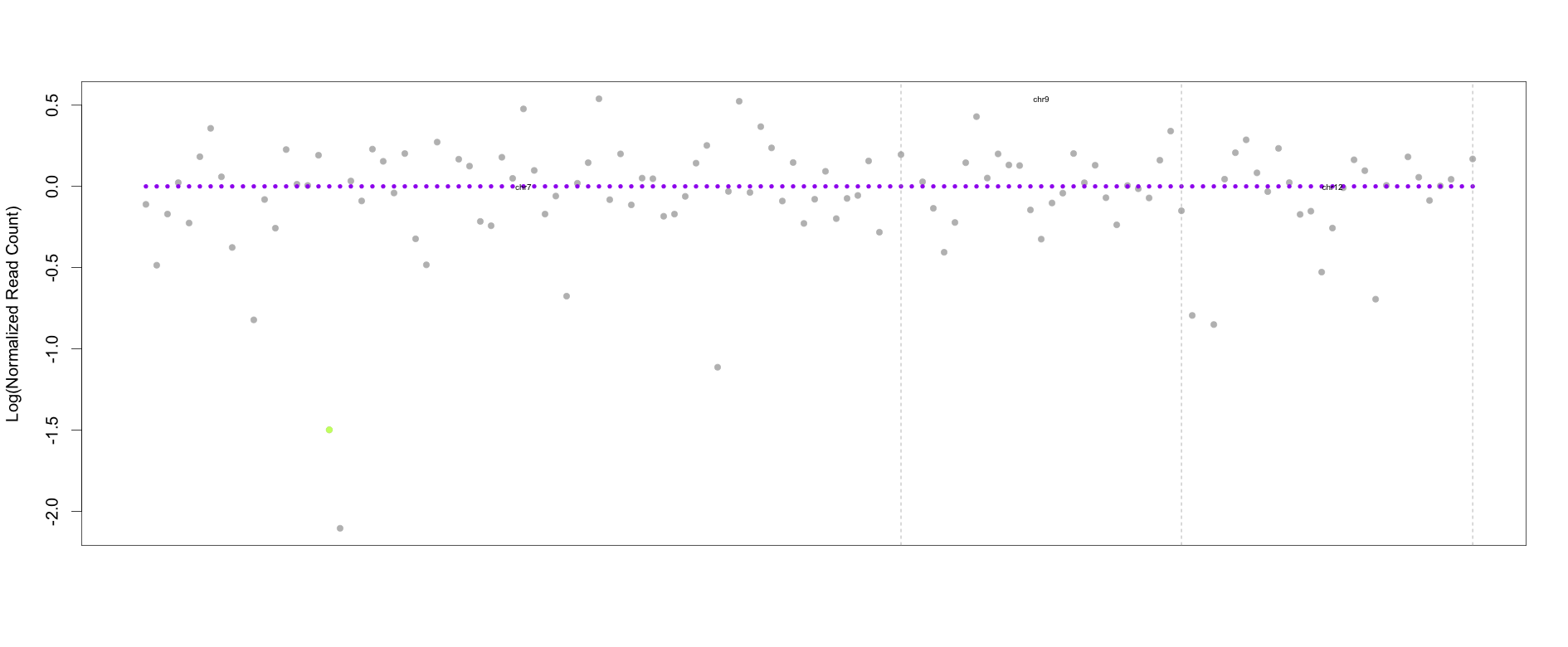

Supplement: Additional file 6 — Archive with ONCOCNV results. Set of files (png plots and txt files) in zip archive that were provided by ONCOCNV after specificity and sensitivity testing. (ZIP 5007 kb) [file 12859_2016_1272_MOESM6_ESM.zip › outputONCOCNV copy 2/FKU.1.profile.png]

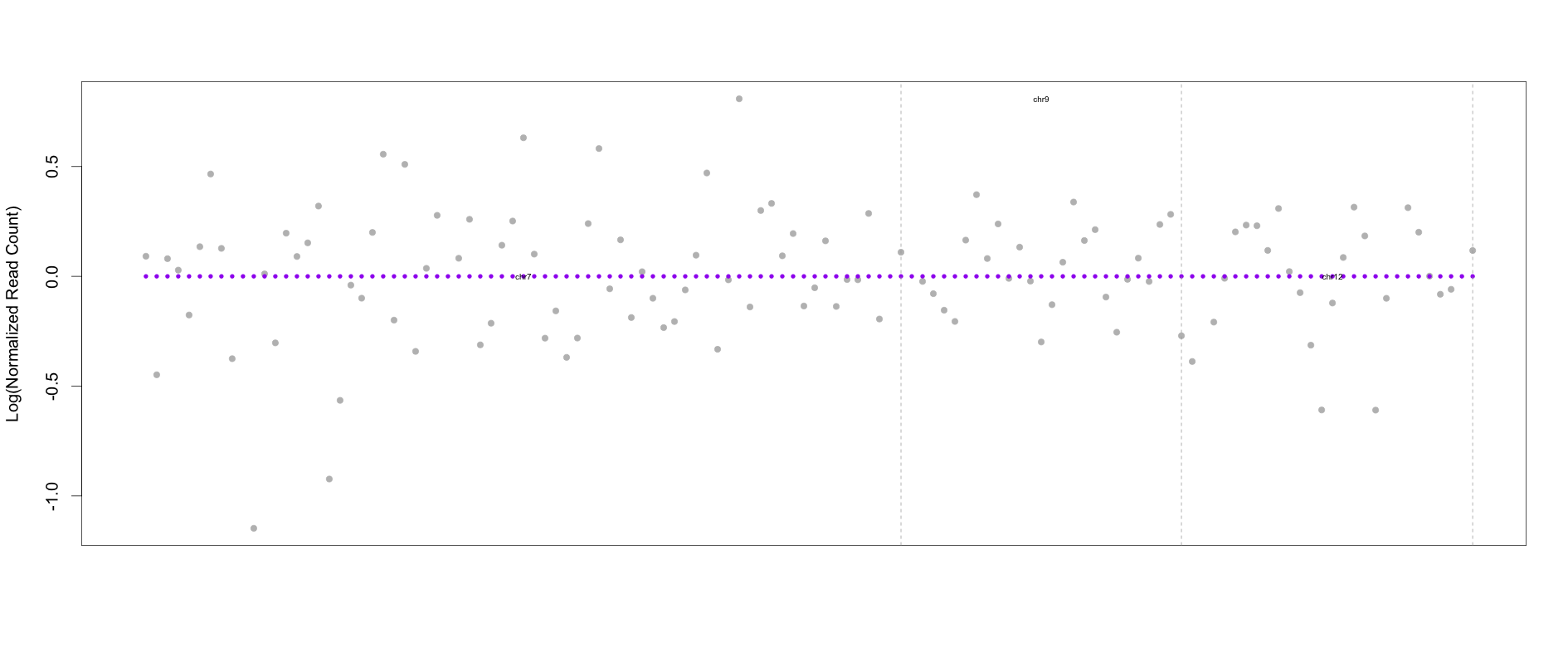

Supplement: Additional file 6 — Archive with ONCOCNV results. Set of files (png plots and txt files) in zip archive that were provided by ONCOCNV after specificity and sensitivity testing. (ZIP 5007 kb) [file 12859_2016_1272_MOESM6_ESM.zip › outputONCOCNV copy 2/FKU.2.profile.png]

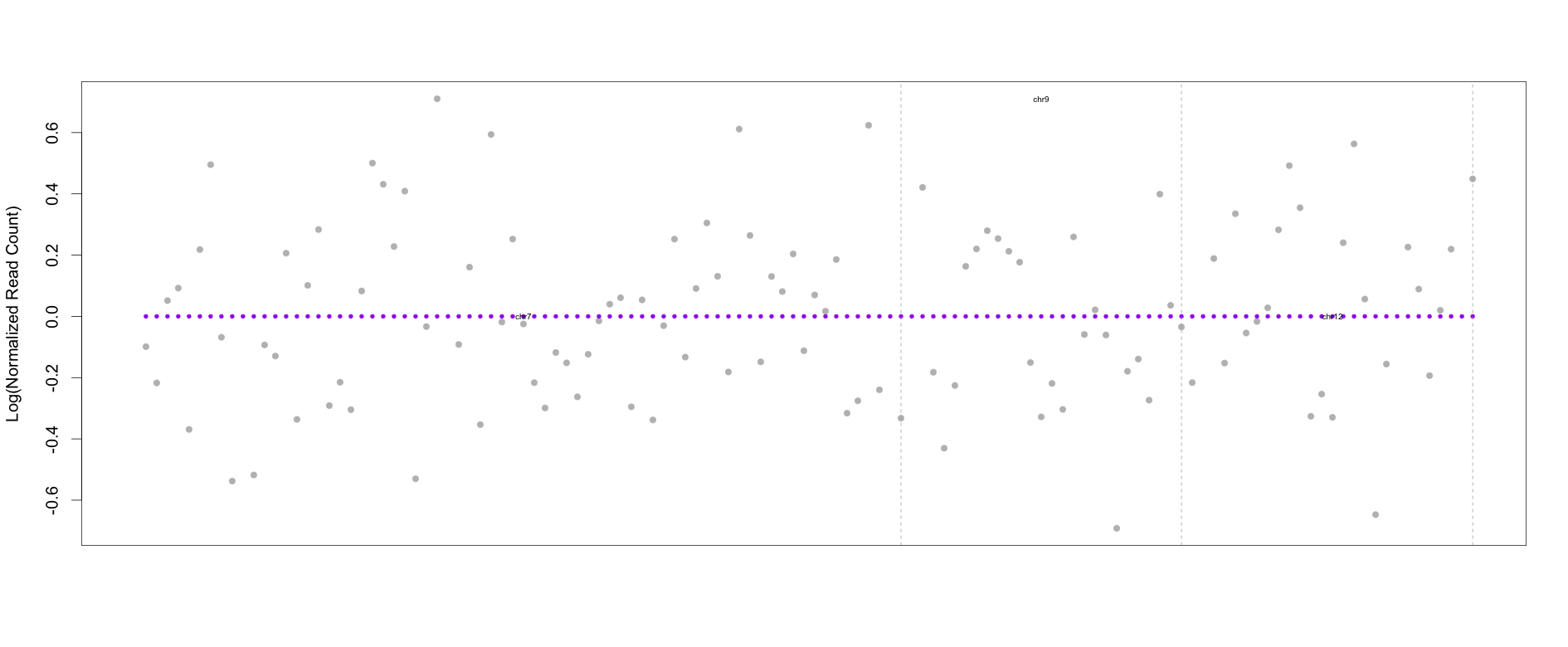

Supplement: Additional file 6 — Archive with ONCOCNV results. Set of files (png plots and txt files) in zip archive that were provided by ONCOCNV after specificity and sensitivity testing. (ZIP 5007 kb) [file 12859_2016_1272_MOESM6_ESM.zip › outputONCOCNV copy 2/IonXpress_003_R_2013_10_17_10_21_46_user_SN2.2.Neonatal.Assay.LEx.2run.18.11.13_Auto_user_SN2.2.Neonatal.Assay.LEx.2run.18.11.13_25.profile.png]

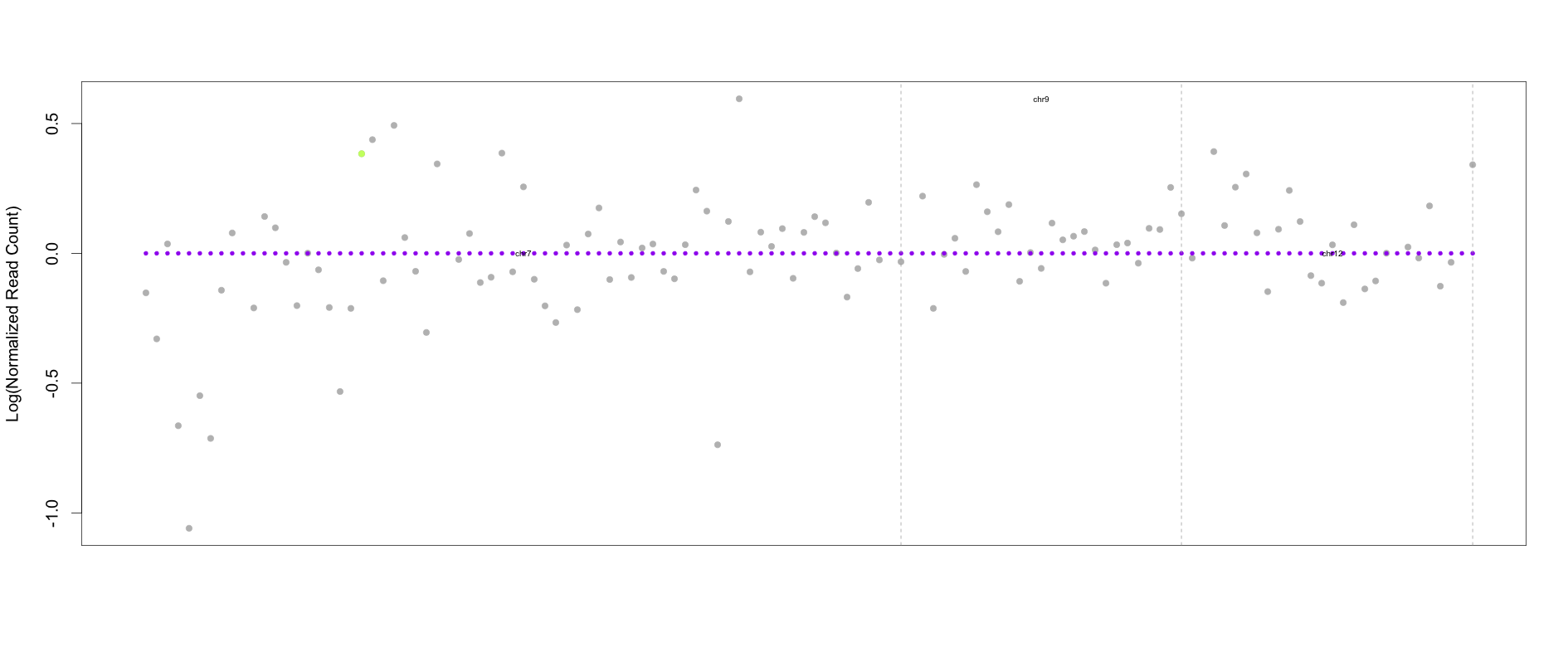

Supplement: Additional file 6 — Archive with ONCOCNV results. Set of files (png plots and txt files) in zip archive that were provided by ONCOCNV after specificity and sensitivity testing. (ZIP 5007 kb) [file 12859_2016_1272_MOESM6_ESM.zip › outputONCOCNV copy 2/IonXpress_003_R_2013_10_17_13_55_39_user_SN2.3.Neonatal.Assay_Lex.1re.run.18.11.13_Auto_user_SN2.3.Neonatal.Assay_Lex.1re.run.18.11.13_26.profile.png]

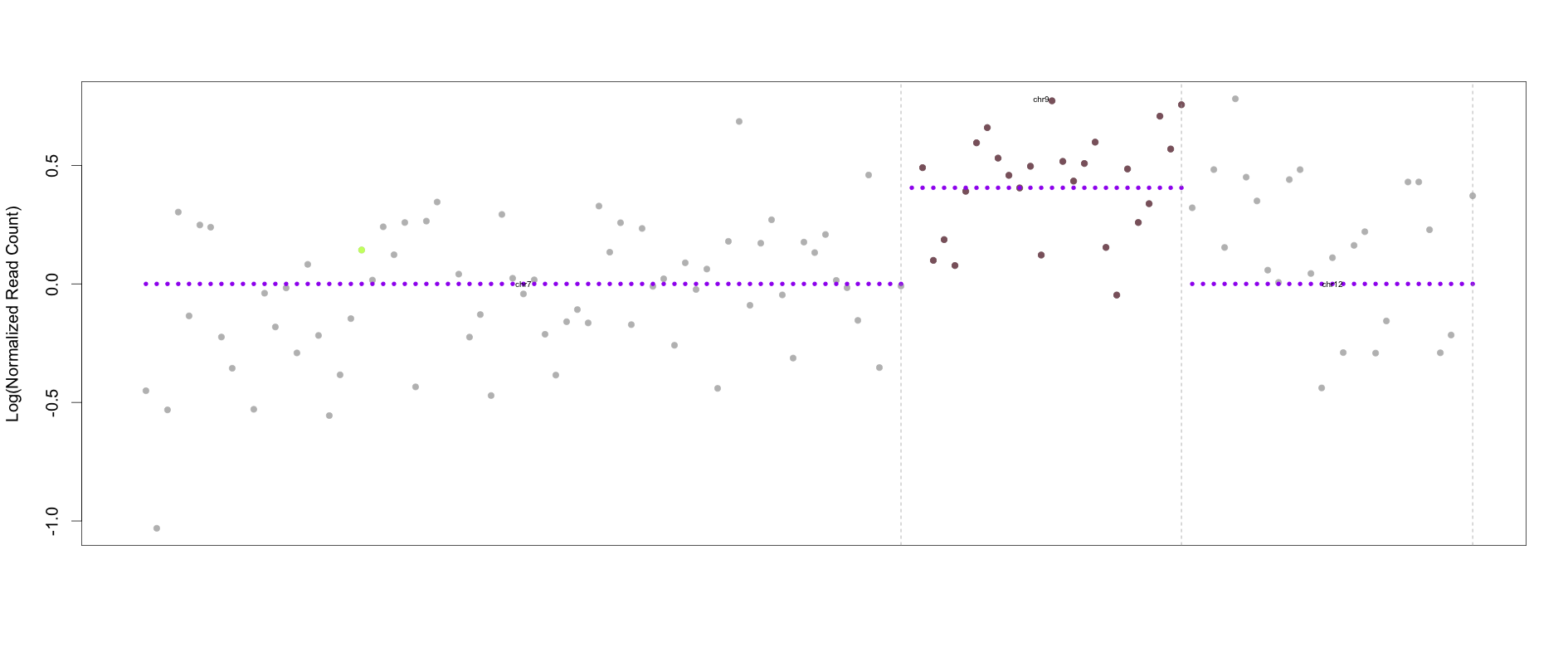

Supplement: Additional file 6 — Archive with ONCOCNV results. Set of files (png plots and txt files) in zip archive that were provided by ONCOCNV after specificity and sensitivity testing. (ZIP 5007 kb) [file 12859_2016_1272_MOESM6_ESM.zip › outputONCOCNV copy 2/IonXpress_004_R_2013_10_17_13_55_39_user_SN2.3.Neonatal.Assay_Lex.1re.run.18.11.13_Auto_user_SN2.3.Neonatal.Assay_Lex.1re.run.18.11.13_26.profile.png]

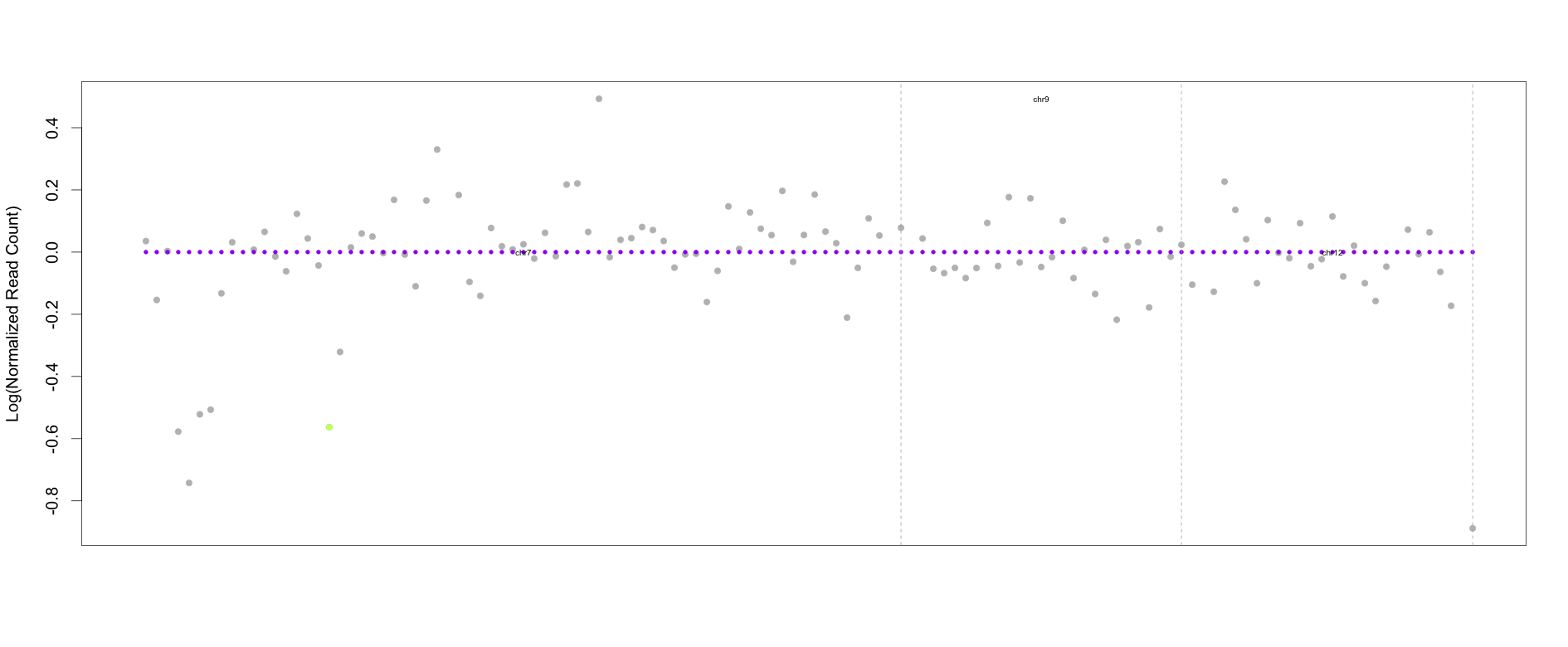

Supplement: Additional file 6 — Archive with ONCOCNV results. Set of files (png plots and txt files) in zip archive that were provided by ONCOCNV after specificity and sensitivity testing. (ZIP 5007 kb) [file 12859_2016_1272_MOESM6_ESM.zip › outputONCOCNV copy 2/IonXpress_006_R_2012_09_13_10_57_38_Sequoia_SN1.27.Run_21_Auto_Sequoia_SN1.27.Run_21_54.profile.png]

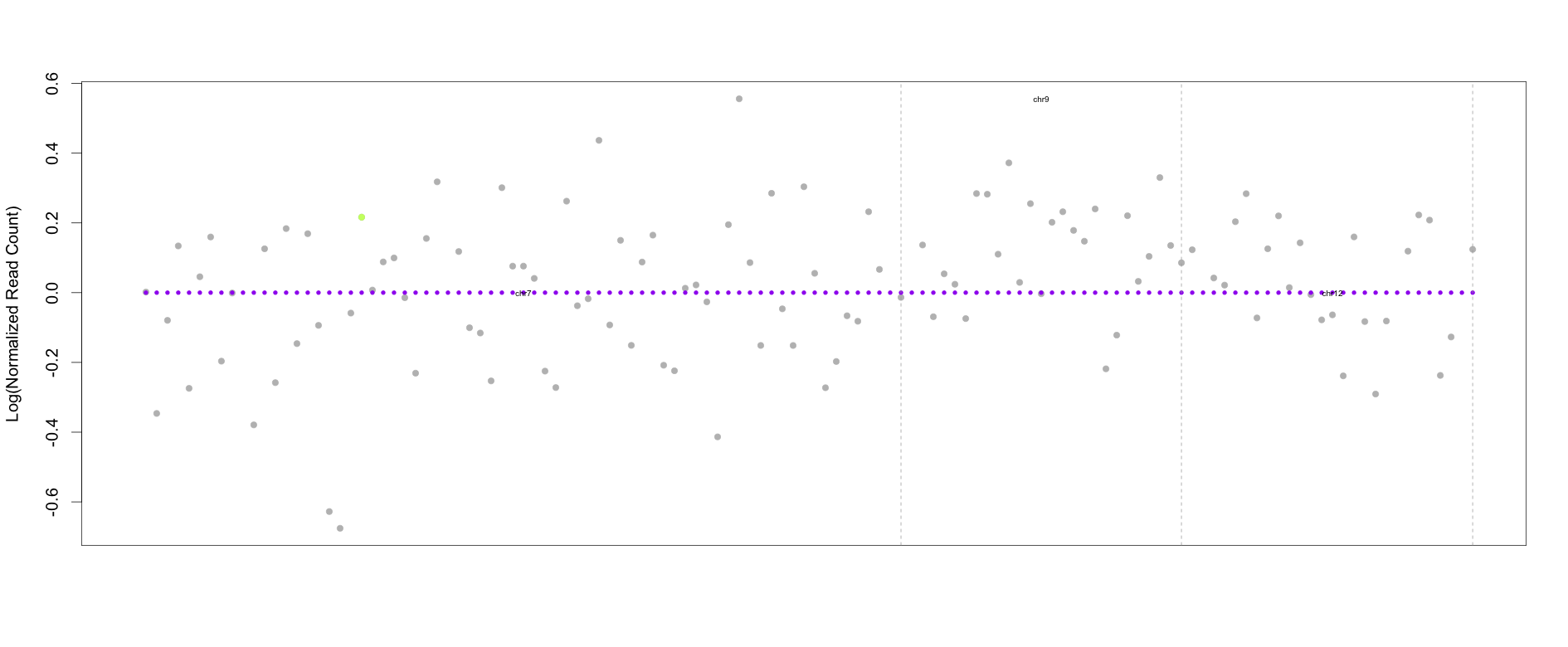

Supplement: Additional file 6 — Archive with ONCOCNV results. Set of files (png plots and txt files) in zip archive that were provided by ONCOCNV after specificity and sensitivity testing. (ZIP 5007 kb) [file 12859_2016_1272_MOESM6_ESM.zip › outputONCOCNV copy 2/IonXpress_006_R_2013_10_17_13_55_39_user_SN2.3.Neonatal.Assay_Lex.1re.run.18.11.13_Auto_user_SN2.3.Neonatal.Assay_Lex.1re.run.18.11.13_26.profile.png]

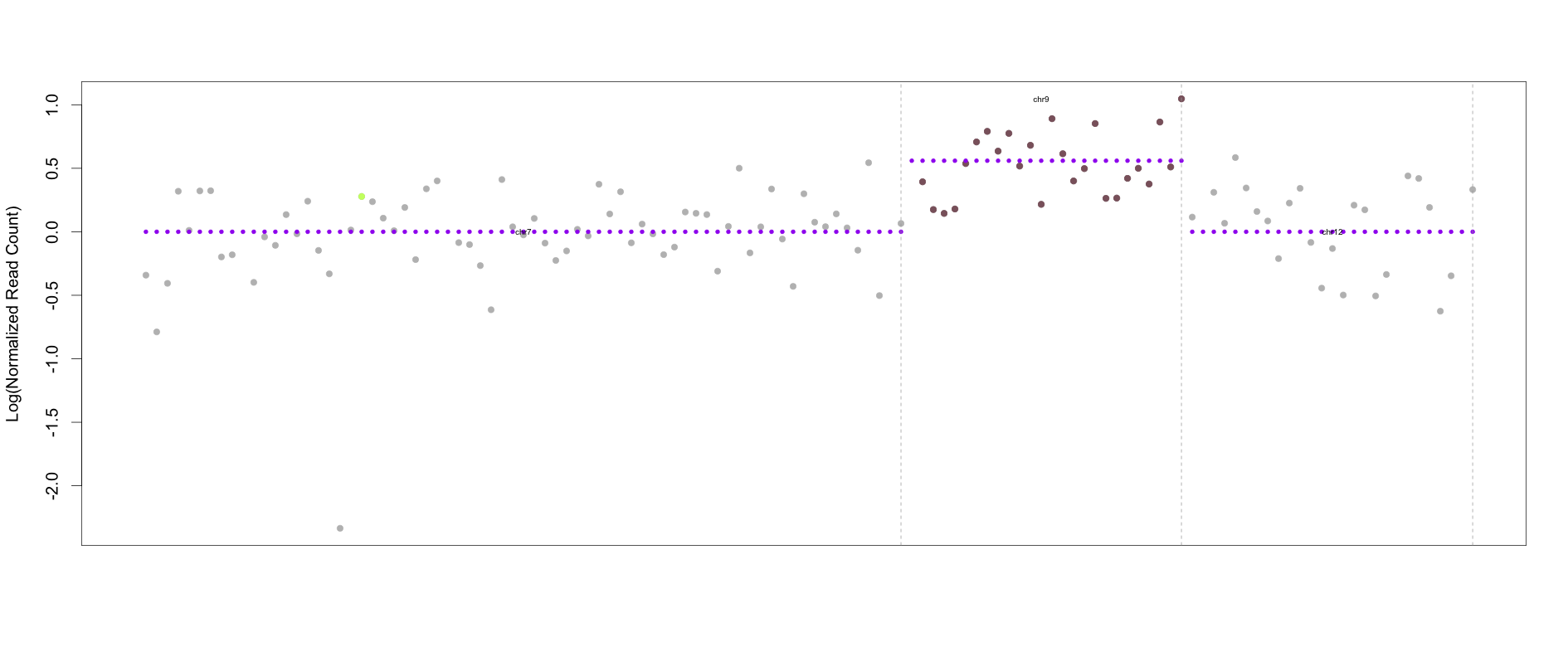

Supplement: Additional file 6 — Archive with ONCOCNV results. Set of files (png plots and txt files) in zip archive that were provided by ONCOCNV after specificity and sensitivity testing. (ZIP 5007 kb) [file 12859_2016_1272_MOESM6_ESM.zip › outputONCOCNV copy 2/IonXpress_007_R_2013_10_17_13_55_39_user_SN2.3.Neonatal.Assay_Lex.1re.run.18.11.13_Auto_user_SN2.3.Neonatal.Assay_Lex.1re.run.18.11.13_26.profile.png]

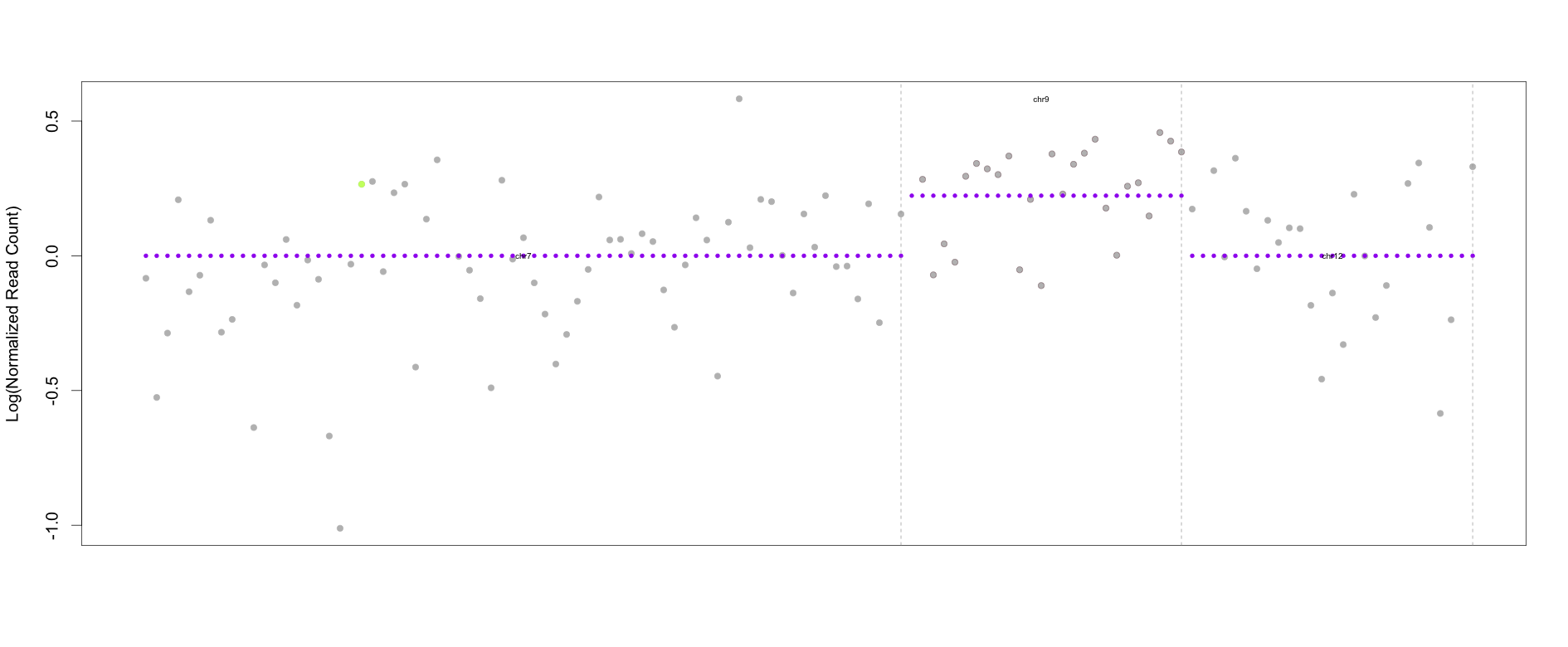

Supplement: Additional file 6 — Archive with ONCOCNV results. Set of files (png plots and txt files) in zip archive that were provided by ONCOCNV after specificity and sensitivity testing. (ZIP 5007 kb) [file 12859_2016_1272_MOESM6_ESM.zip › outputONCOCNV copy 2/IonXpress_010_R_2013_10_17_13_55_39_user_SN2.3.Neonatal.Assay_Lex.1re.run.18.11.13_Auto_user_SN2.3.Neonatal.Assay_Lex.1re.run.18.11.13_26.profile.png]

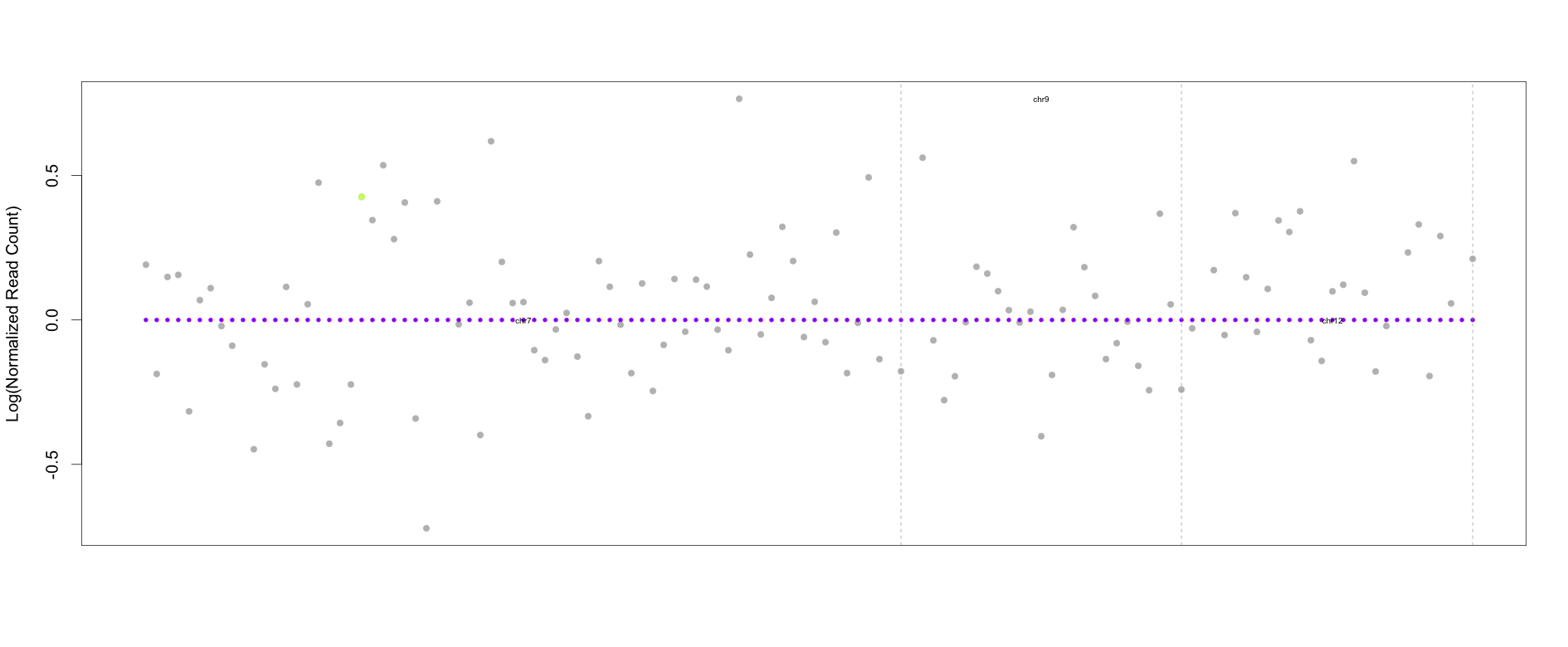

Supplement: Additional file 6 — Archive with ONCOCNV results. Set of files (png plots and txt files) in zip archive that were provided by ONCOCNV after specificity and sensitivity testing. (ZIP 5007 kb) [file 12859_2016_1272_MOESM6_ESM.zip › outputONCOCNV copy 2/IonXpress_011_R_2013_10_17_10_21_46_user_SN2.2.Neonatal.Assay.LEx.2run.18.11.13_Auto_user_SN2.2.Neonatal.Assay.LEx.2run.18.11.13_25.profile.png]

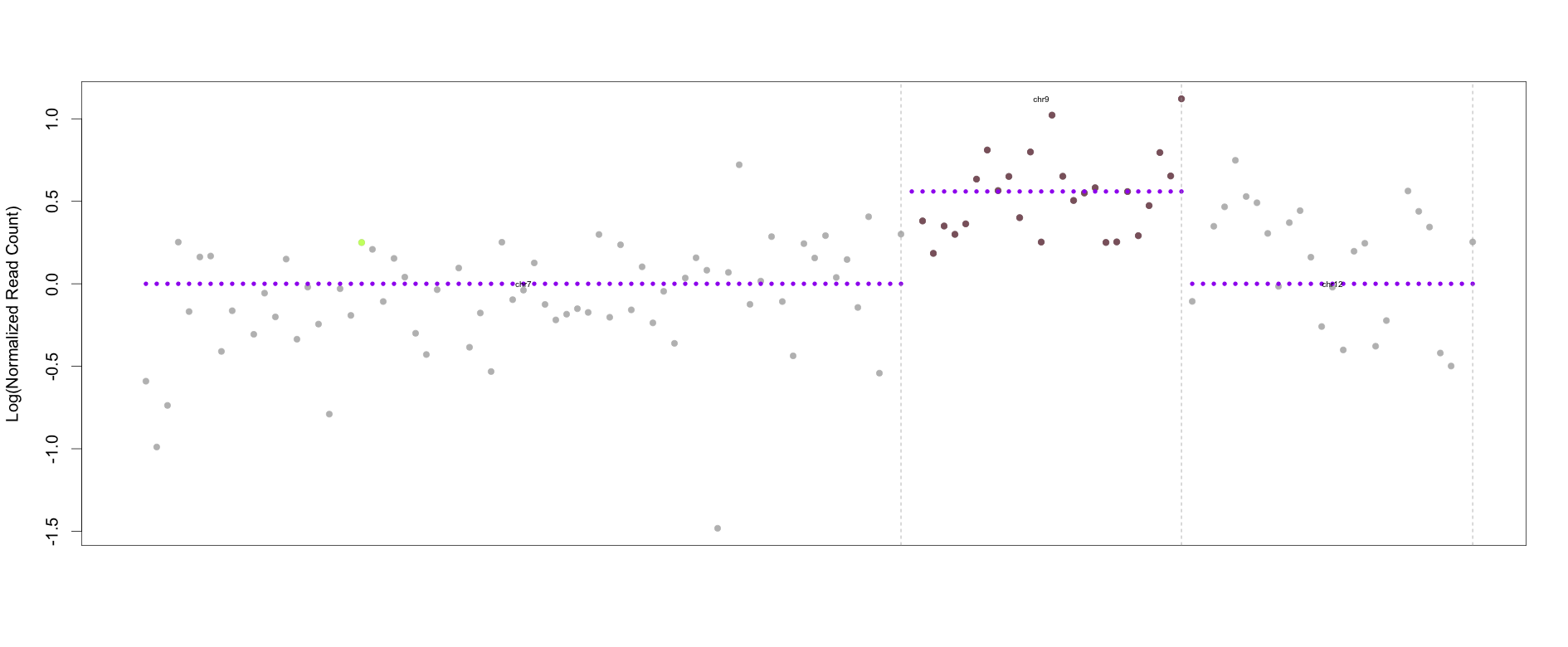

Supplement: Additional file 6 — Archive with ONCOCNV results. Set of files (png plots and txt files) in zip archive that were provided by ONCOCNV after specificity and sensitivity testing. (ZIP 5007 kb) [file 12859_2016_1272_MOESM6_ESM.zip › outputONCOCNV copy 2/IonXpress_011_R_2013_10_17_13_55_39_user_SN2.3.Neonatal.Assay_Lex.1re.run.18.11.13_Auto_user_SN2.3.Neonatal.Assay_Lex.1re.run.18.11.13_26.profile.png]

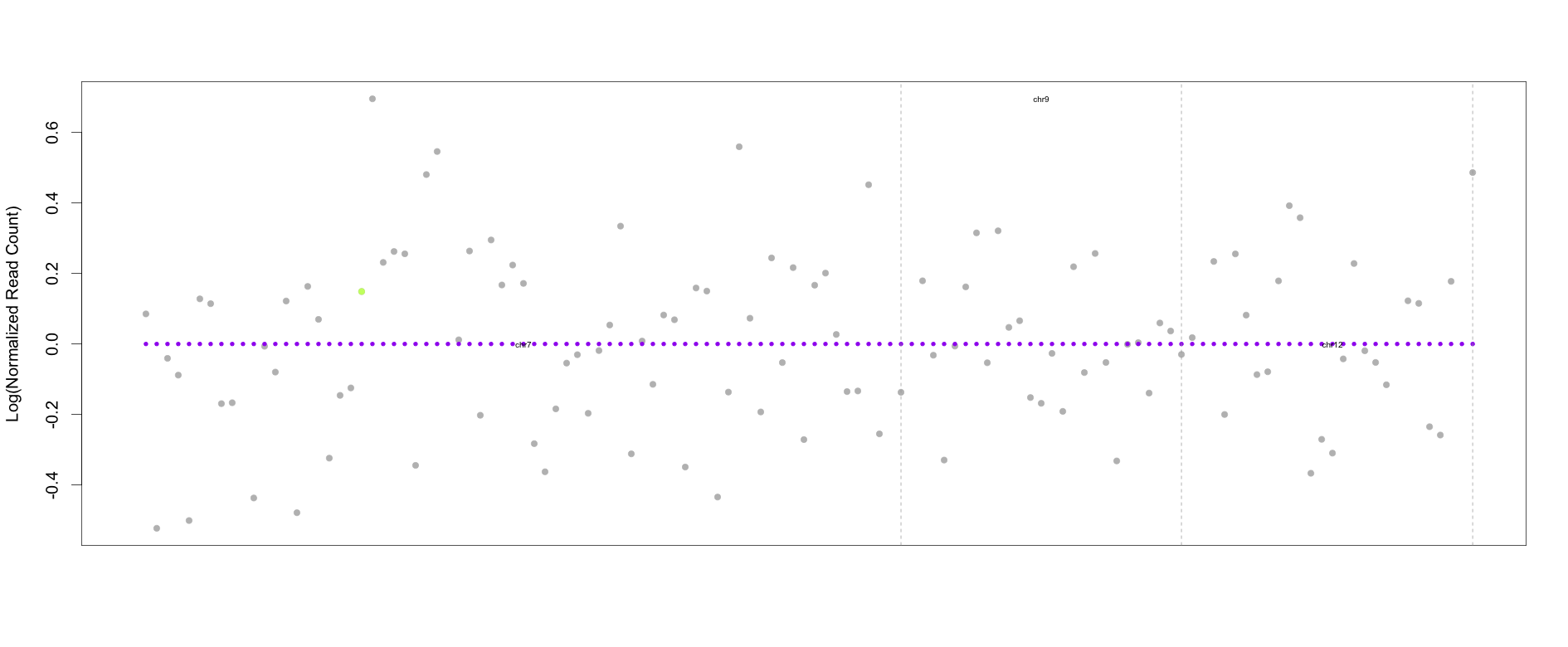

Supplement: Additional file 6 — Archive with ONCOCNV results. Set of files (png plots and txt files) in zip archive that were provided by ONCOCNV after specificity and sensitivity testing. (ZIP 5007 kb) [file 12859_2016_1272_MOESM6_ESM.zip › outputONCOCNV copy 2/IonXpress_012_R_2013_10_17_13_55_39_user_SN2.3.Neonatal.Assay_Lex.1re.run.18.11.13_Auto_user_SN2.3.Neonatal.Assay_Lex.1re.run.18.11.13_26.profile.png]

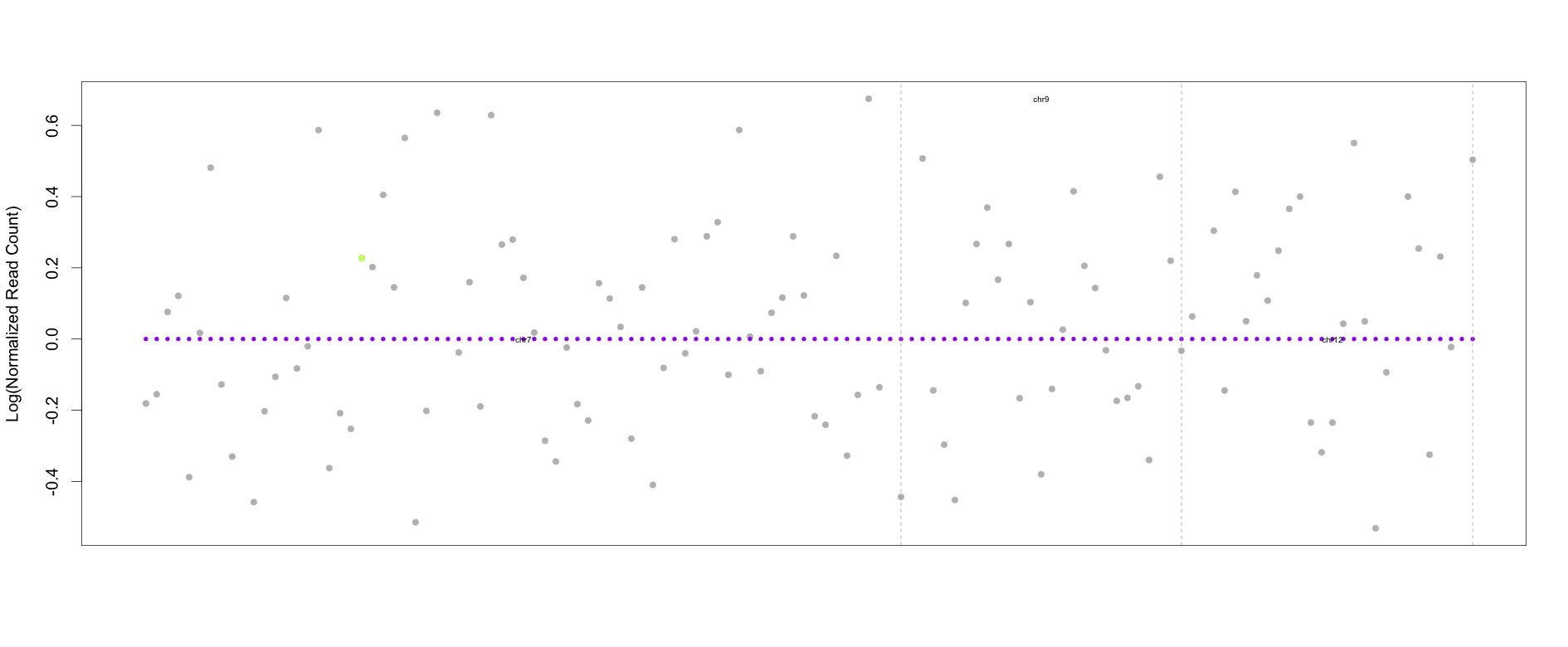

Supplement: Additional file 6 — Archive with ONCOCNV results. Set of files (png plots and txt files) in zip archive that were provided by ONCOCNV after specificity and sensitivity testing. (ZIP 5007 kb) [file 12859_2016_1272_MOESM6_ESM.zip › outputONCOCNV copy 2/IonXpress_013_R_2013_10_17_10_21_46_user_SN2.2.Neonatal.Assay.LEx.2run.18.11.13_Auto_user_SN2.2.Neonatal.Assay.LEx.2run.18.11.13_25.profile.png]

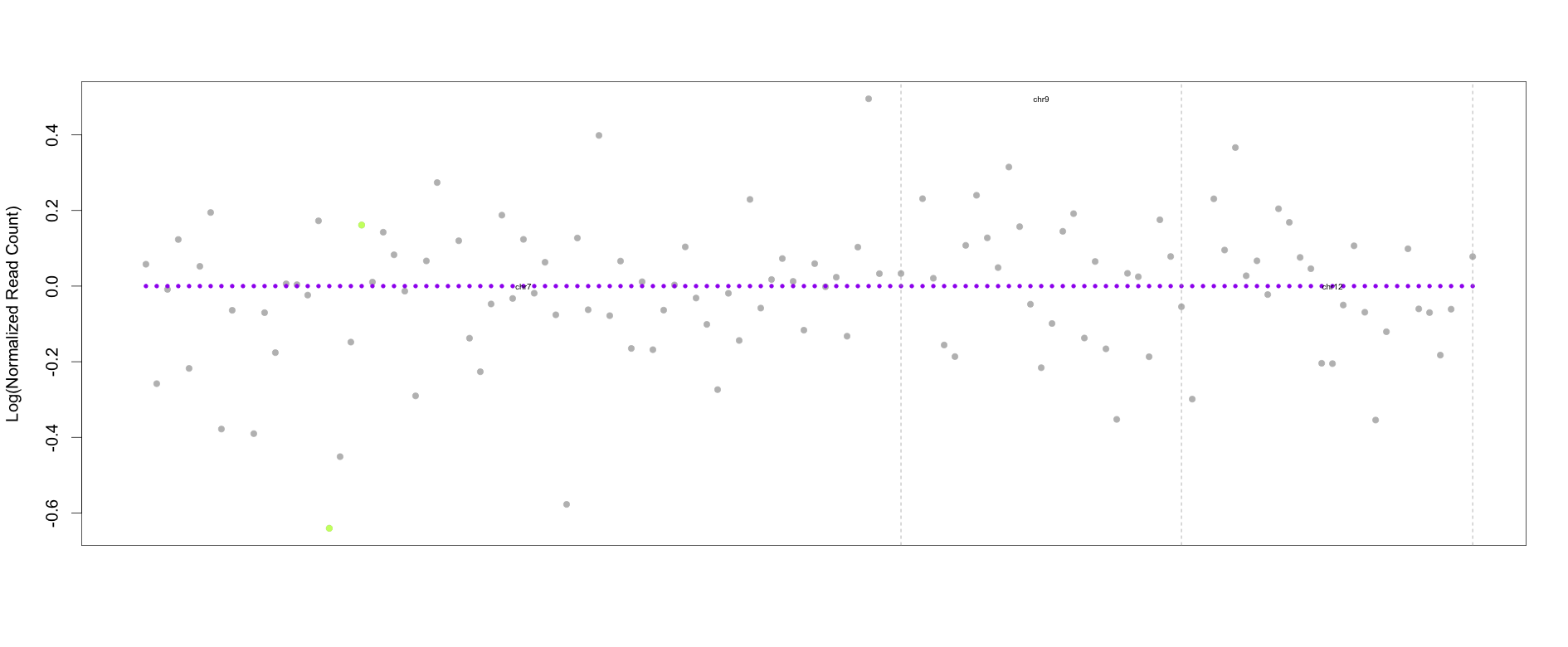

Supplement: Additional file 6 — Archive with ONCOCNV results. Set of files (png plots and txt files) in zip archive that were provided by ONCOCNV after specificity and sensitivity testing. (ZIP 5007 kb) [file 12859_2016_1272_MOESM6_ESM.zip › outputONCOCNV copy 2/IonXpress_014_R_2012_09_13_15_10_42_Sequoia_SN1.28.Run_22_Auto_Sequoia_SN1.28.Run_22_55.profile.png]

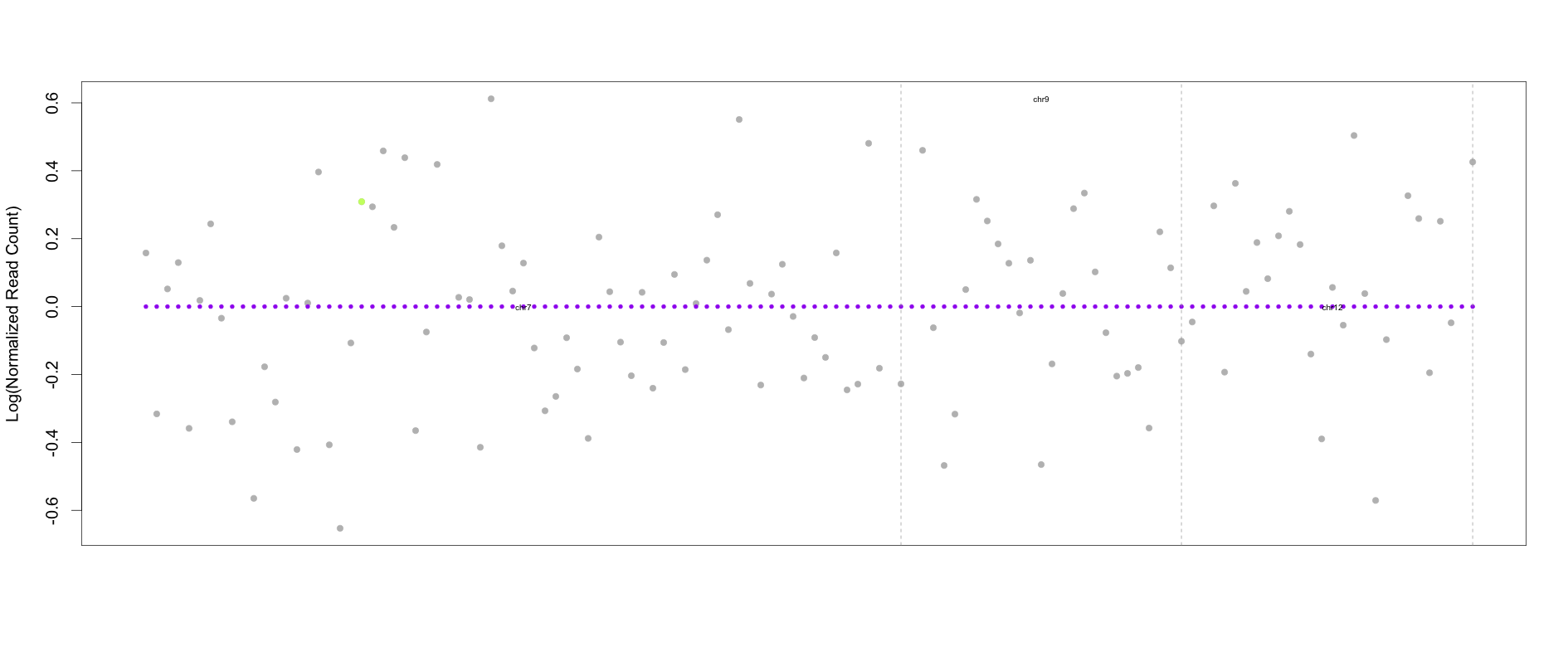

Supplement: Additional file 6 — Archive with ONCOCNV results. Set of files (png plots and txt files) in zip archive that were provided by ONCOCNV after specificity and sensitivity testing. (ZIP 5007 kb) [file 12859_2016_1272_MOESM6_ESM.zip › outputONCOCNV copy 2/IonXpress_014_R_2013_10_17_10_21_46_user_SN2.2.Neonatal.Assay.LEx.2run.18.11.13_Auto_user_SN2.2.Neonatal.Assay.LEx.2run.18.11.13_25.profile.png]

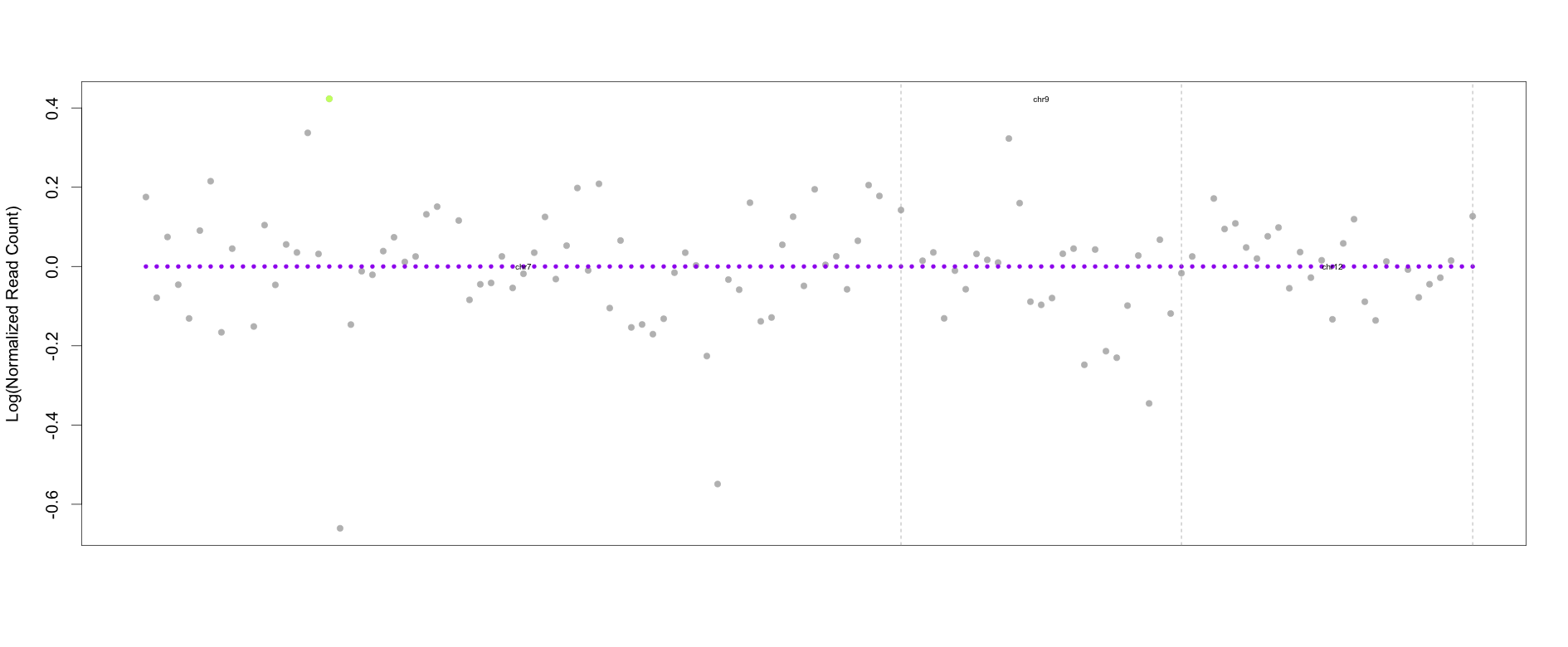

Supplement: Additional file 6 — Archive with ONCOCNV results. Set of files (png plots and txt files) in zip archive that were provided by ONCOCNV after specificity and sensitivity testing. (ZIP 5007 kb) [file 12859_2016_1272_MOESM6_ESM.zip › outputONCOCNV copy 2/IonXpress_015_R_2012_09_13_15_10_42_Sequoia_SN1.28.Run_22_Auto_Sequoia_SN1.28.Run_22_55.profile.png]

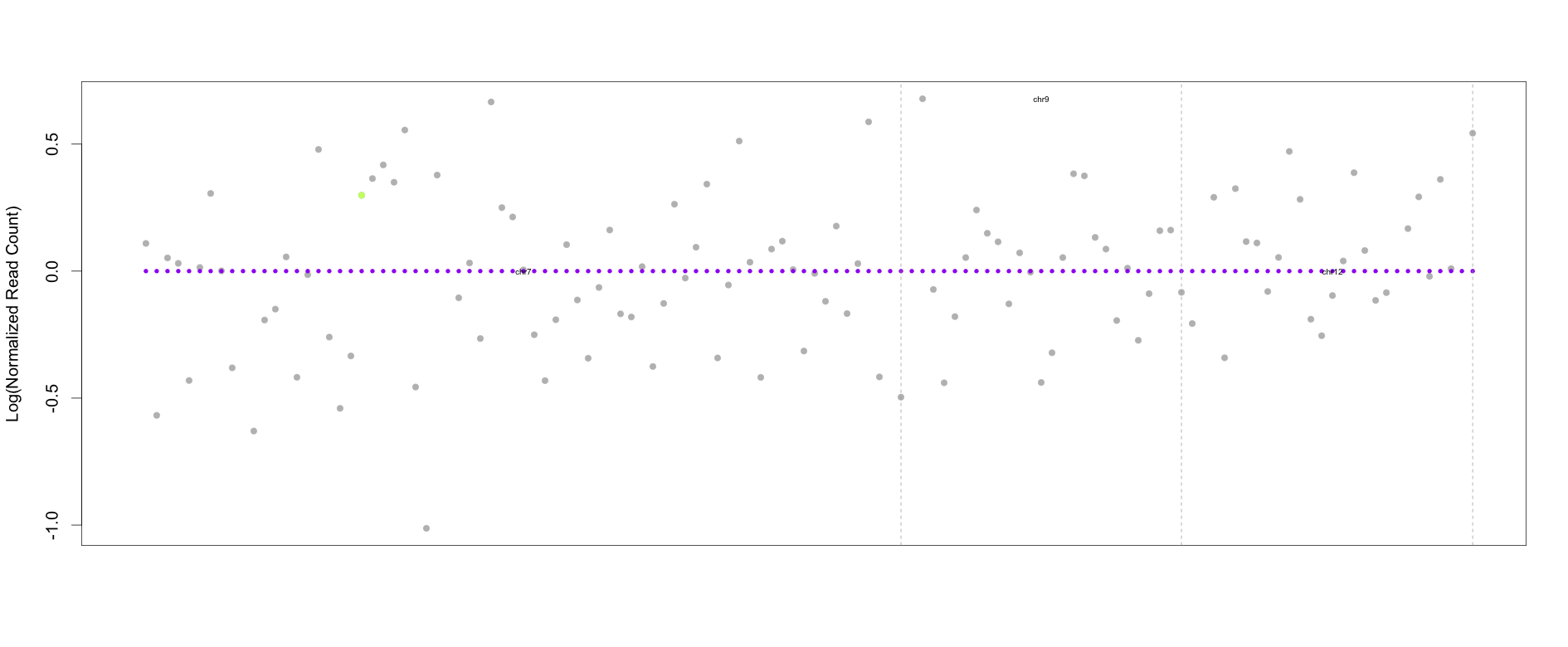

Supplement: Additional file 6 — Archive with ONCOCNV results. Set of files (png plots and txt files) in zip archive that were provided by ONCOCNV after specificity and sensitivity testing. (ZIP 5007 kb) [file 12859_2016_1272_MOESM6_ESM.zip › outputONCOCNV copy 2/IonXpress_015_R_2013_10_17_10_21_46_user_SN2.2.Neonatal.Assay.LEx.2run.18.11.13_Auto_user_SN2.2.Neonatal.Assay.LEx.2run.18.11.13_25.profile.png]

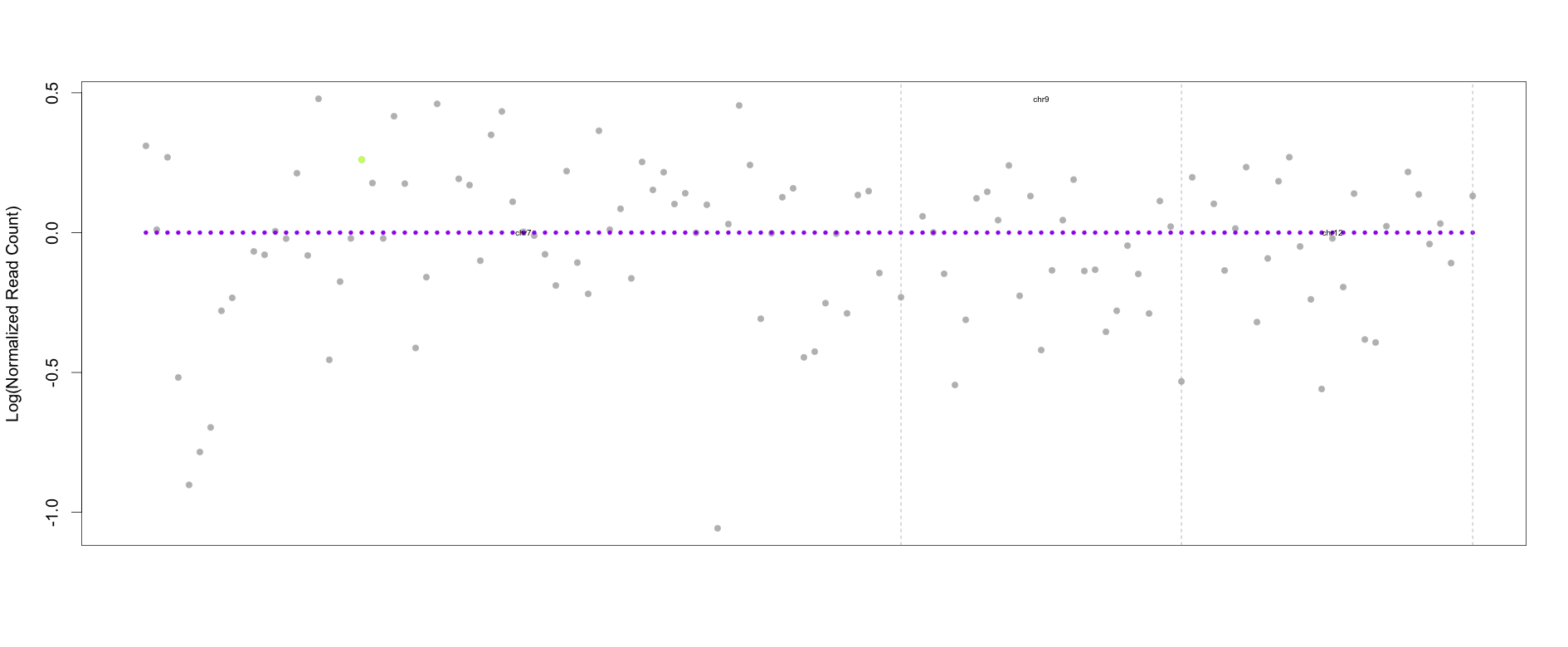

Supplement: Additional file 6 — Archive with ONCOCNV results. Set of files (png plots and txt files) in zip archive that were provided by ONCOCNV after specificity and sensitivity testing. (ZIP 5007 kb) [file 12859_2016_1272_MOESM6_ESM.zip › outputONCOCNV copy 2/IonXpress_015_R_2013_10_17_13_55_39_user_SN2.3.Neonatal.Assay_Lex.1re.run.18.11.13_Auto_user_SN2.3.Neonatal.Assay_Lex.1re.run.18.11.13_26.profile.png]

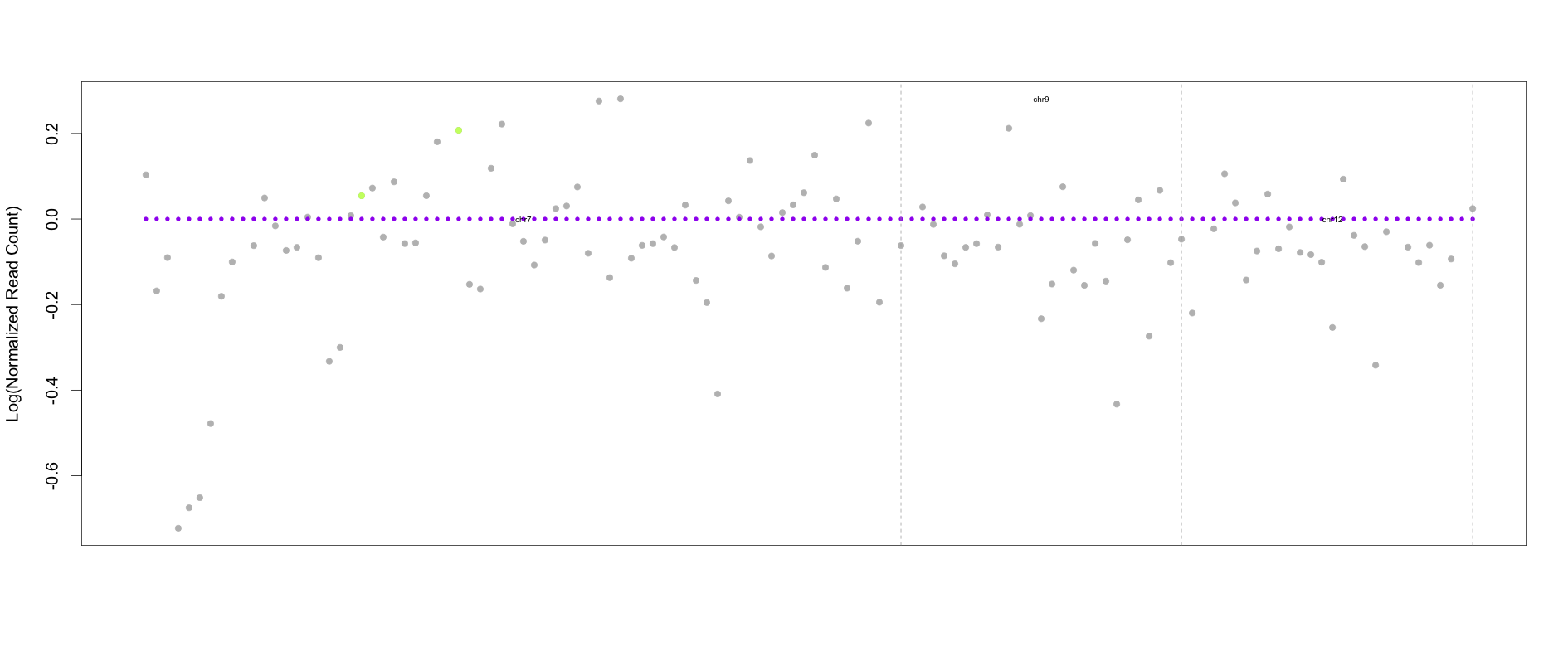

Supplement: Additional file 6 — Archive with ONCOCNV results. Set of files (png plots and txt files) in zip archive that were provided by ONCOCNV after specificity and sensitivity testing. (ZIP 5007 kb) [file 12859_2016_1272_MOESM6_ESM.zip › outputONCOCNV copy 2/IonXpress_016_R_2012_09_13_10_57_38_Sequoia_SN1.27.Run_21_Auto_Sequoia_SN1.27.Run_21_54.profile.png]

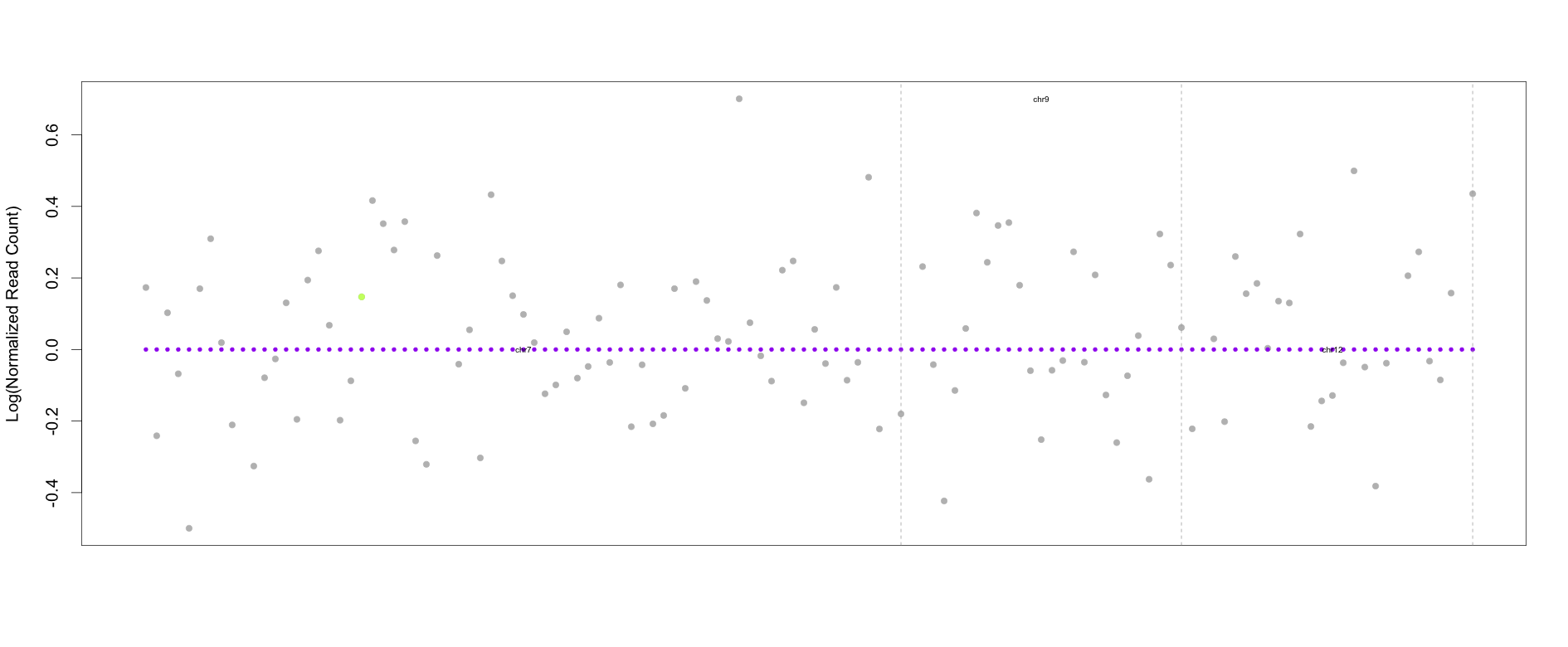

Supplement: Additional file 6 — Archive with ONCOCNV results. Set of files (png plots and txt files) in zip archive that were provided by ONCOCNV after specificity and sensitivity testing. (ZIP 5007 kb) [file 12859_2016_1272_MOESM6_ESM.zip › outputONCOCNV copy 2/IonXpress_016_R_2013_10_17_10_21_46_user_SN2.2.Neonatal.Assay.LEx.2run.18.11.13_Auto_user_SN2.2.Neonatal.Assay.LEx.2run.18.11.13_25.profile.png]

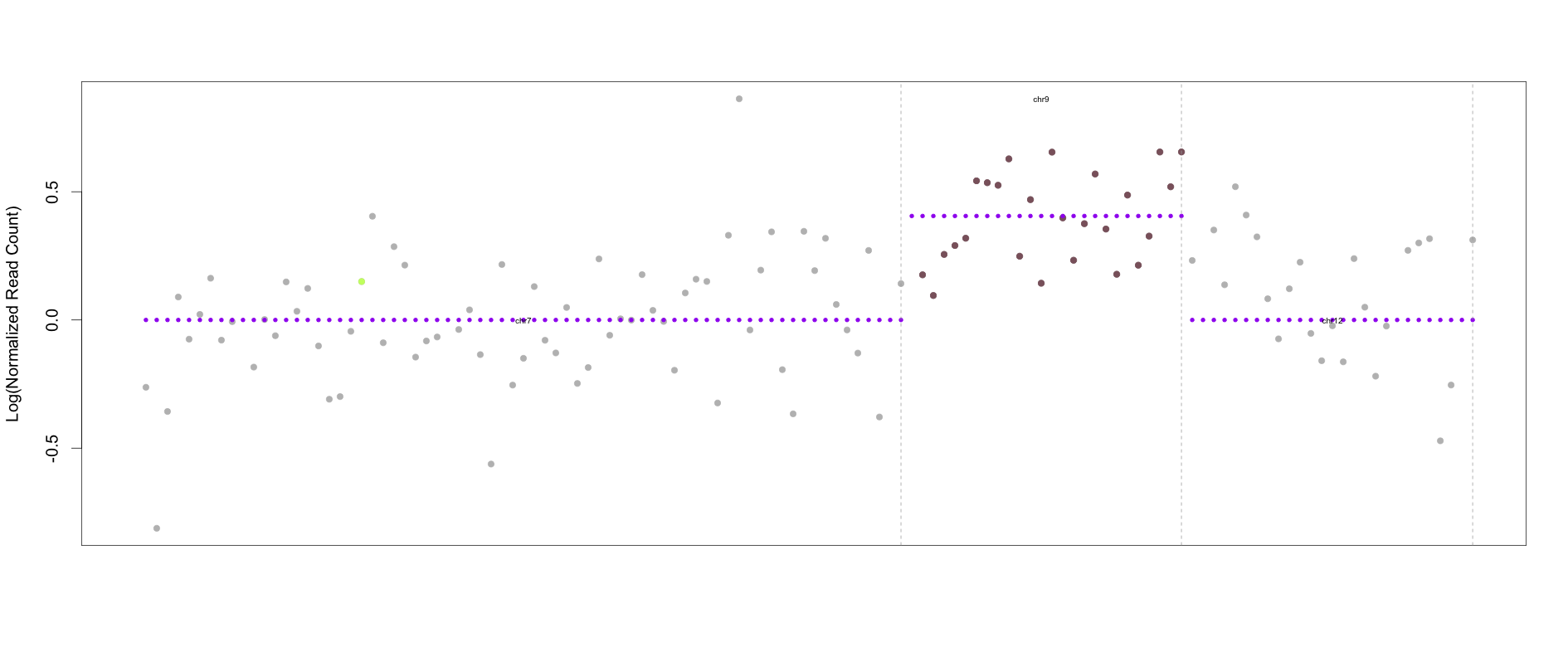

Supplement: Additional file 6 — Archive with ONCOCNV results. Set of files (png plots and txt files) in zip archive that were provided by ONCOCNV after specificity and sensitivity testing. (ZIP 5007 kb) [file 12859_2016_1272_MOESM6_ESM.zip › outputONCOCNV copy 2/IonXpress_016_R_2013_10_17_13_55_39_user_SN2.3.Neonatal.Assay_Lex.1re.run.18.11.13_Auto_user_SN2.3.Neonatal.Assay_Lex.1re.run.18.11.13_26.profile.png]

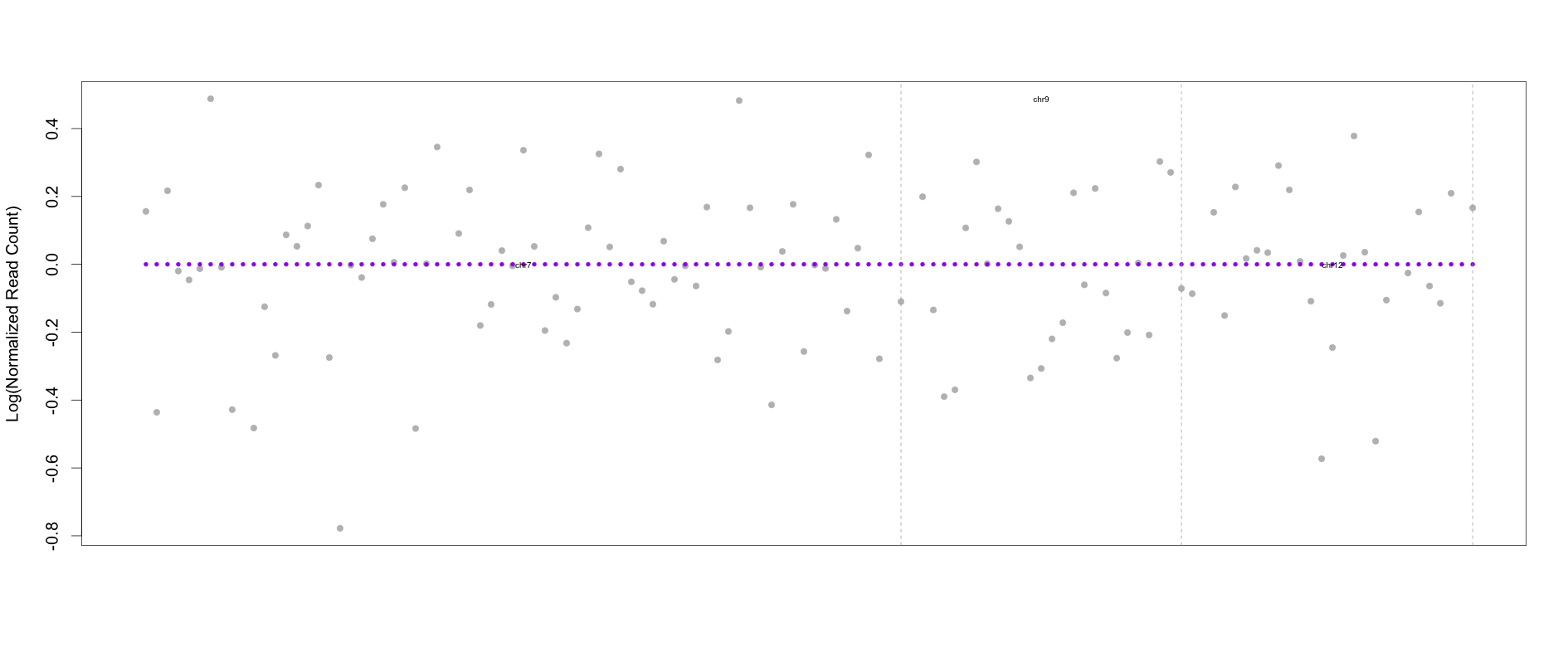

Supplement: Additional file 6 — Archive with ONCOCNV results. Set of files (png plots and txt files) in zip archive that were provided by ONCOCNV after specificity and sensitivity testing. (ZIP 5007 kb) [file 12859_2016_1272_MOESM6_ESM.zip › outputONCOCNV copy 2/IonXpress_017_R_2013_10_18_01_30_51_user_SN2.4.Neonatal.Assay2_new_bed_version_318_chip_v2_Auto_user_SN2.4.Neonatal.Assay2_new_bed_version_318_chip_v2_28.profile.png]

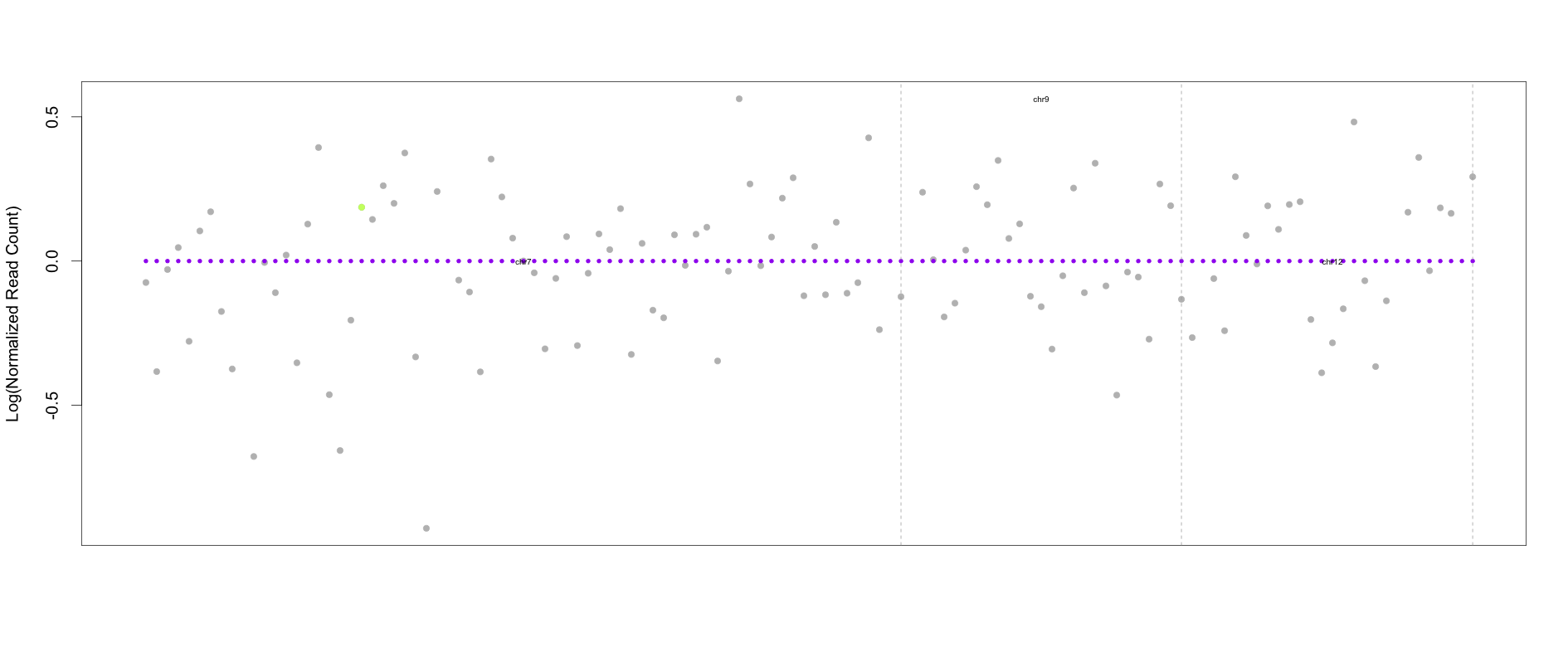

Supplement: Additional file 6 — Archive with ONCOCNV results. Set of files (png plots and txt files) in zip archive that were provided by ONCOCNV after specificity and sensitivity testing. (ZIP 5007 kb) [file 12859_2016_1272_MOESM6_ESM.zip › outputONCOCNV copy 2/IonXpress_018_R_2013_10_17_10_21_46_user_SN2.2.Neonatal.Assay.LEx.2run.18.11.13_Auto_user_SN2.2.Neonatal.Assay.LEx.2run.18.11.13_25.profile.png]

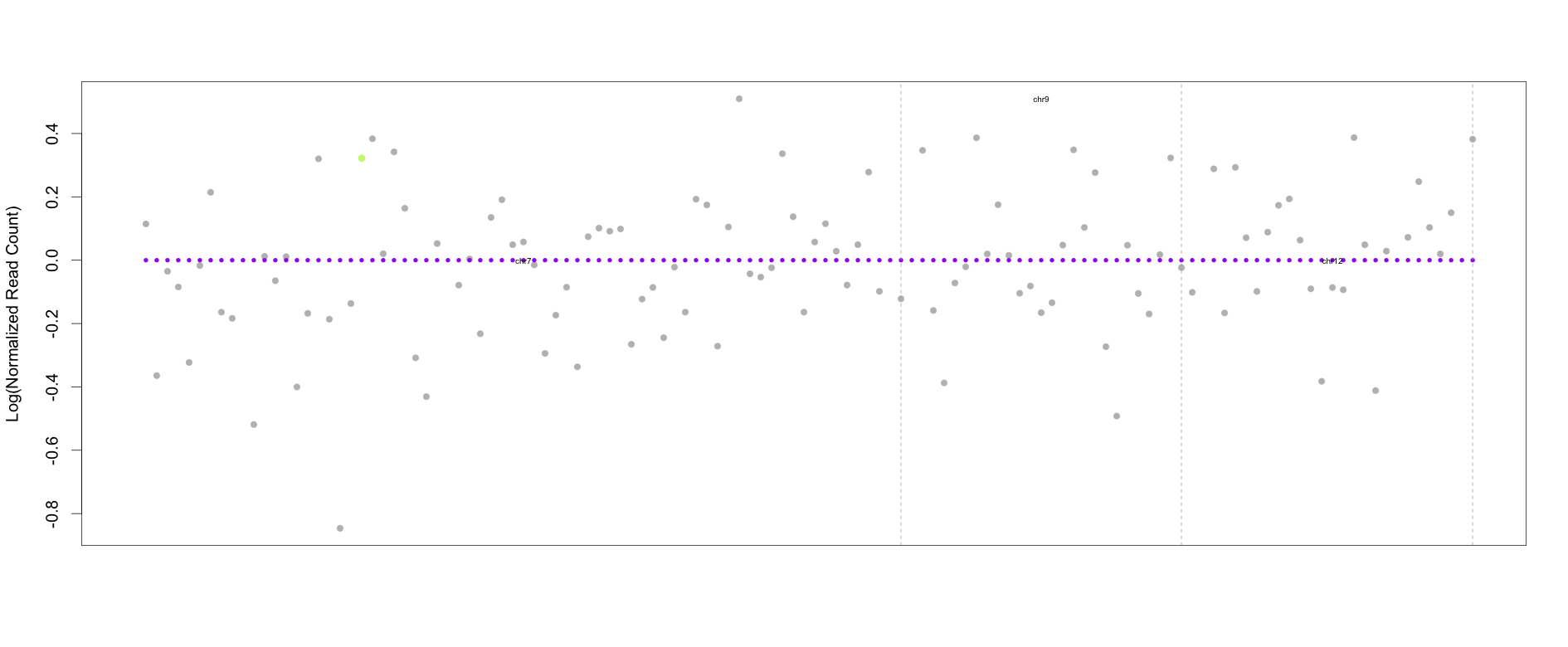

Supplement: Additional file 6 — Archive with ONCOCNV results. Set of files (png plots and txt files) in zip archive that were provided by ONCOCNV after specificity and sensitivity testing. (ZIP 5007 kb) [file 12859_2016_1272_MOESM6_ESM.zip › outputONCOCNV copy 2/IonXpress_018_R_2013_10_17_13_55_39_user_SN2.3.Neonatal.Assay_Lex.1re.run.18.11.13_Auto_user_SN2.3.Neonatal.Assay_Lex.1re.run.18.11.13_26.profile.png]

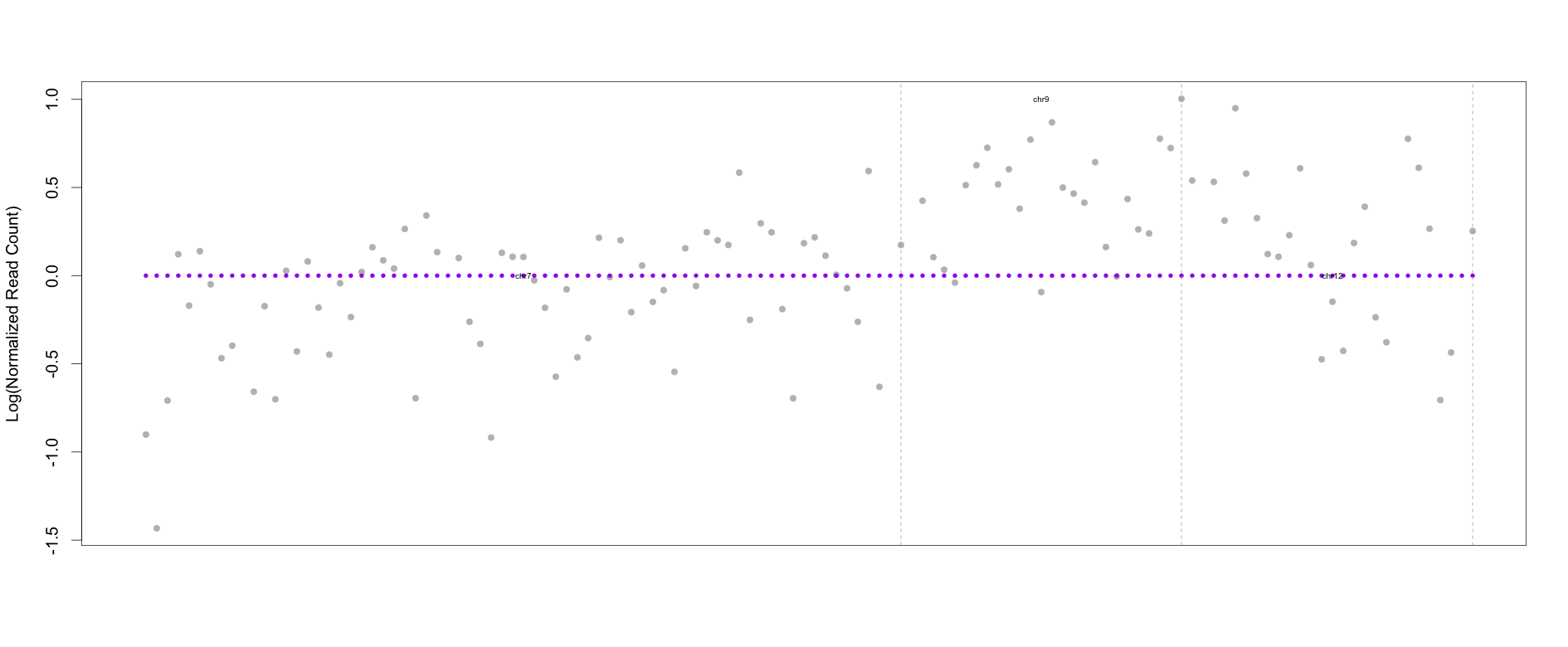

Supplement: Additional file 6 — Archive with ONCOCNV results. Set of files (png plots and txt files) in zip archive that were provided by ONCOCNV after specificity and sensitivity testing. (ZIP 5007 kb) [file 12859_2016_1272_MOESM6_ESM.zip › outputONCOCNV copy 2/IonXpress_019_R_2013_10_17_13_55_39_user_SN2.3.Neonatal.Assay_Lex.1re.run.18.11.13_Auto_user_SN2.3.Neonatal.Assay_Lex.1re.run.18.11.13_26.profile.png]

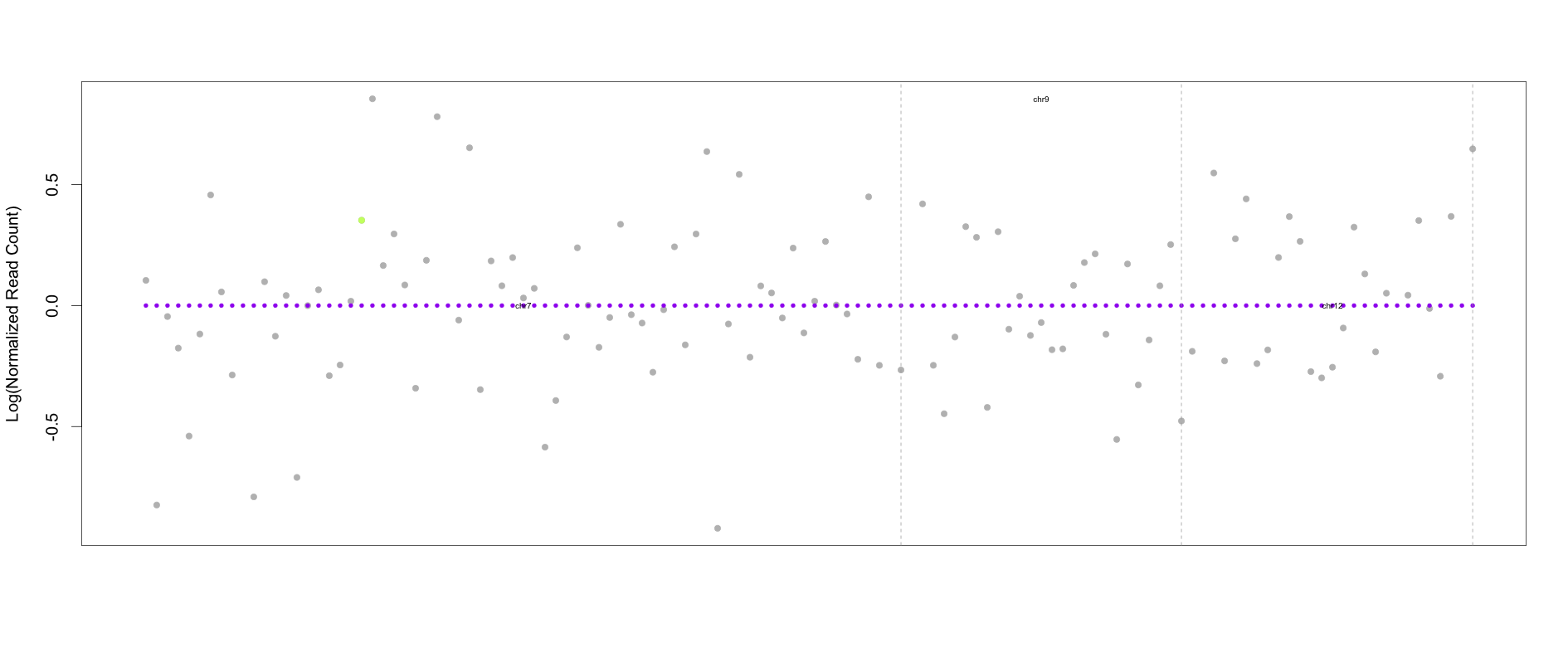

Supplement: Additional file 6 — Archive with ONCOCNV results. Set of files (png plots and txt files) in zip archive that were provided by ONCOCNV after specificity and sensitivity testing. (ZIP 5007 kb) [file 12859_2016_1272_MOESM6_ESM.zip › outputONCOCNV copy 2/IonXpress_020_R_2013_10_17_13_55_39_user_SN2.3.Neonatal.Assay_Lex.1re.run.18.11.13_Auto_user_SN2.3.Neonatal.Assay_Lex.1re.run.18.11.13_26.profile.png]

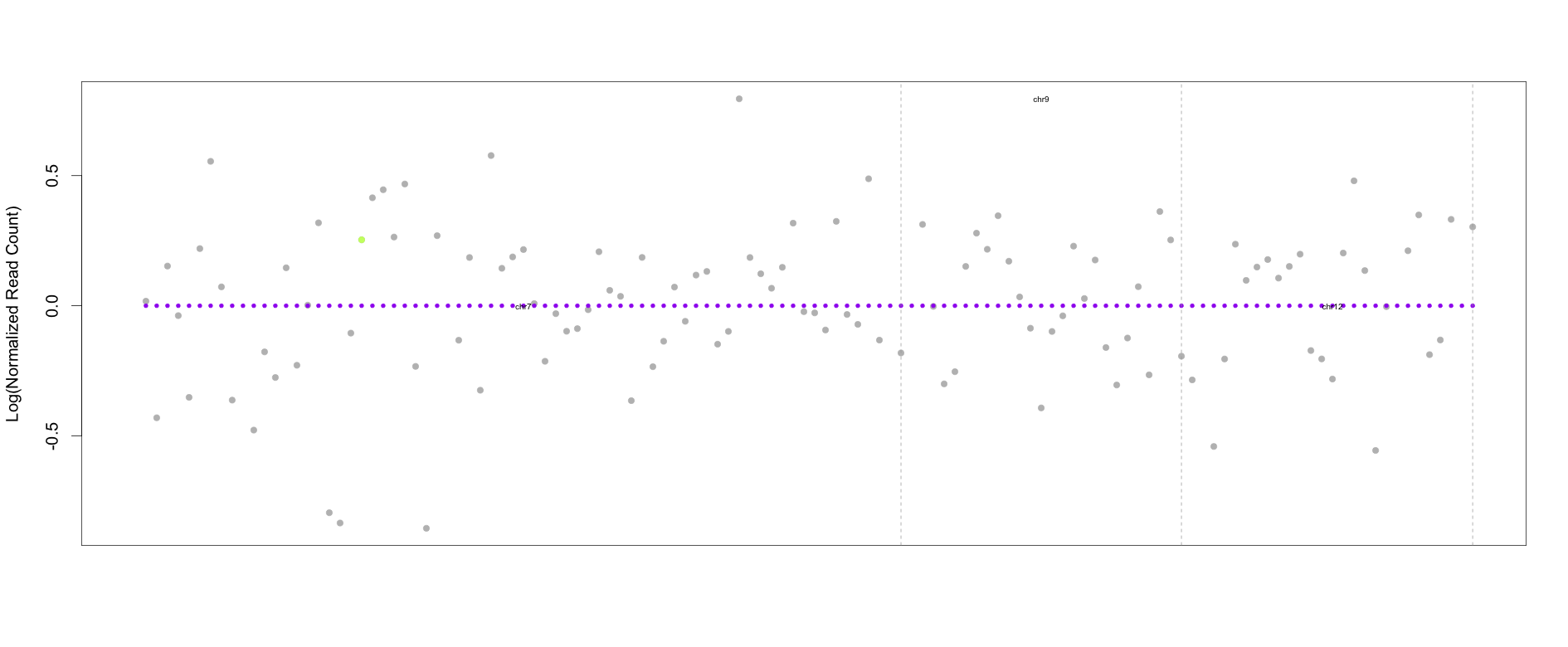

Supplement: Additional file 6 — Archive with ONCOCNV results. Set of files (png plots and txt files) in zip archive that were provided by ONCOCNV after specificity and sensitivity testing. (ZIP 5007 kb) [file 12859_2016_1272_MOESM6_ESM.zip › outputONCOCNV copy 2/IonXpress_021_R_2013_10_17_10_21_46_user_SN2.2.Neonatal.Assay.LEx.2run.18.11.13_Auto_user_SN2.2.Neonatal.Assay.LEx.2run.18.11.13_25.profile.png]

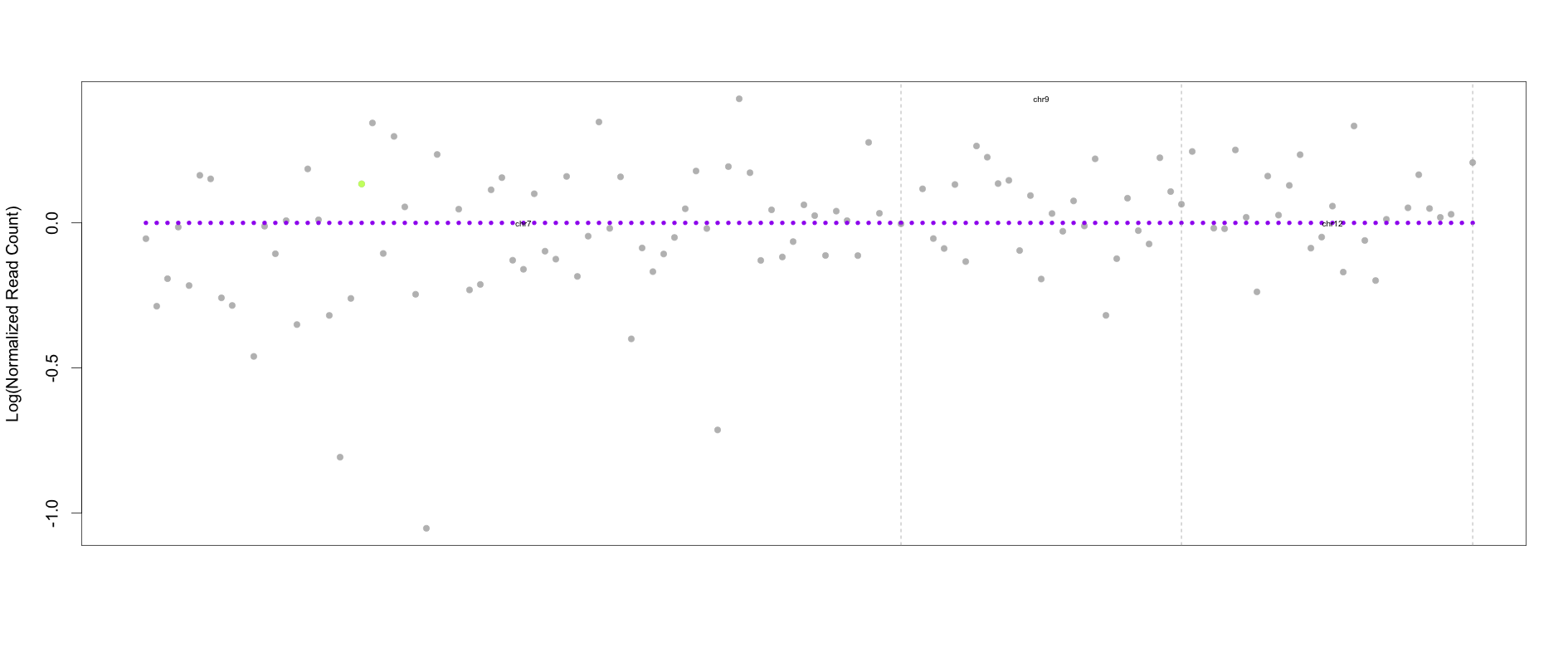

Supplement: Additional file 6 — Archive with ONCOCNV results. Set of files (png plots and txt files) in zip archive that were provided by ONCOCNV after specificity and sensitivity testing. (ZIP 5007 kb) [file 12859_2016_1272_MOESM6_ESM.zip › outputONCOCNV copy 2/IonXpress_024_R_2013_10_17_13_55_39_user_SN2.3.Neonatal.Assay_Lex.1re.run.18.11.13_Auto_user_SN2.3.Neonatal.Assay_Lex.1re.run.18.11.13_26.profile.png]

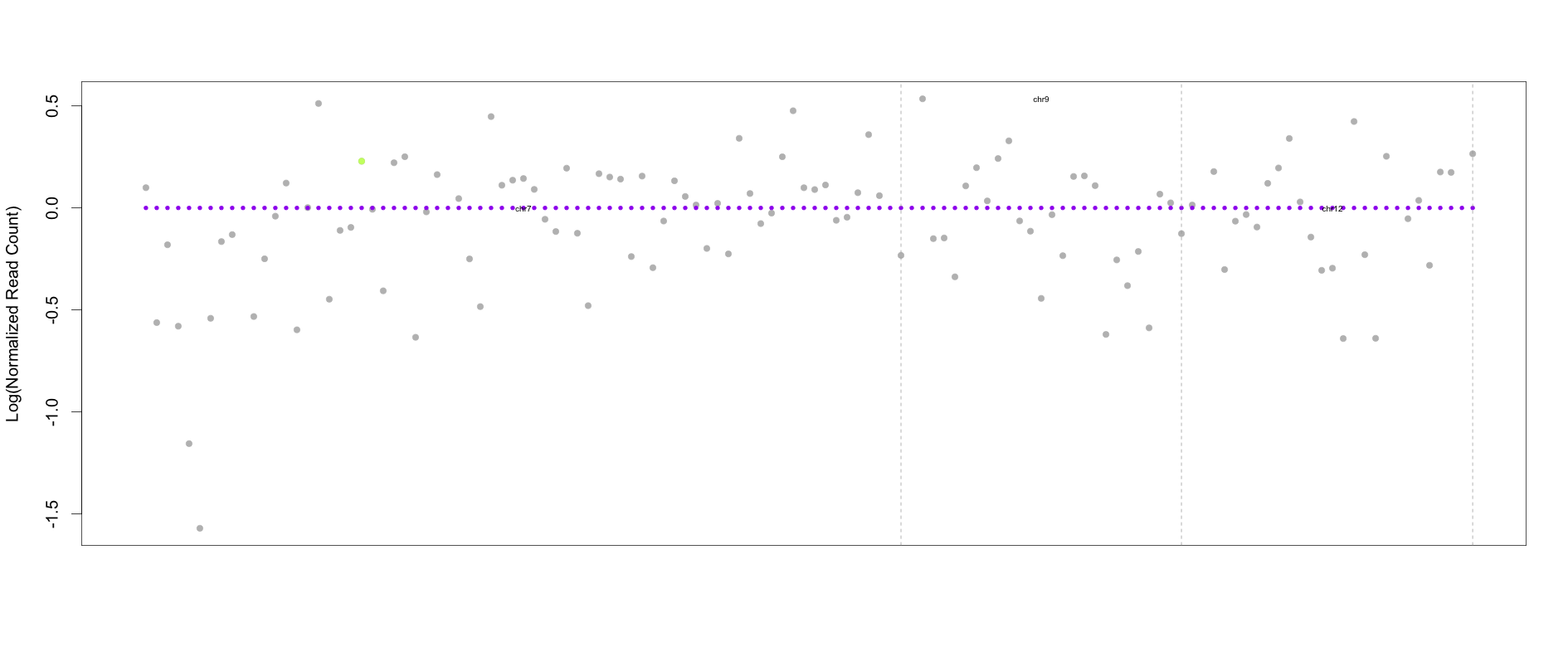

Supplement: Additional file 6 — Archive with ONCOCNV results. Set of files (png plots and txt files) in zip archive that were provided by ONCOCNV after specificity and sensitivity testing. (ZIP 5007 kb) [file 12859_2016_1272_MOESM6_ESM.zip › outputONCOCNV copy 2/IonXpress_025_R_2013_10_17_10_21_46_user_SN2.2.Neonatal.Assay.LEx.2run.18.11.13_Auto_user_SN2.2.Neonatal.Assay.LEx.2run.18.11.13_25.profile.png]

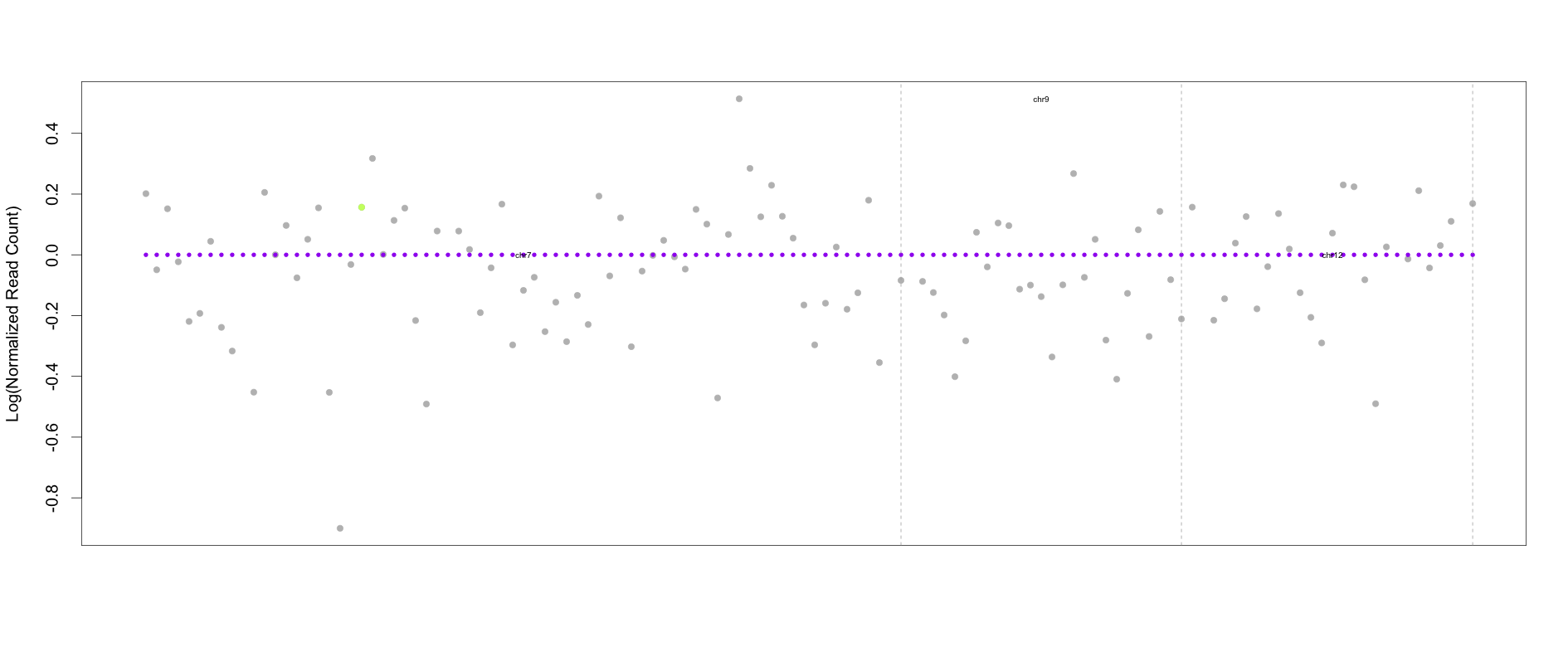

Supplement: Additional file 6 — Archive with ONCOCNV results. Set of files (png plots and txt files) in zip archive that were provided by ONCOCNV after specificity and sensitivity testing. (ZIP 5007 kb) [file 12859_2016_1272_MOESM6_ESM.zip › outputONCOCNV copy 2/IonXpress_025_R_2013_10_17_13_55_39_user_SN2.3.Neonatal.Assay_Lex.1re.run.18.11.13_Auto_user_SN2.3.Neonatal.Assay_Lex.1re.run.18.11.13_26.profile.png]

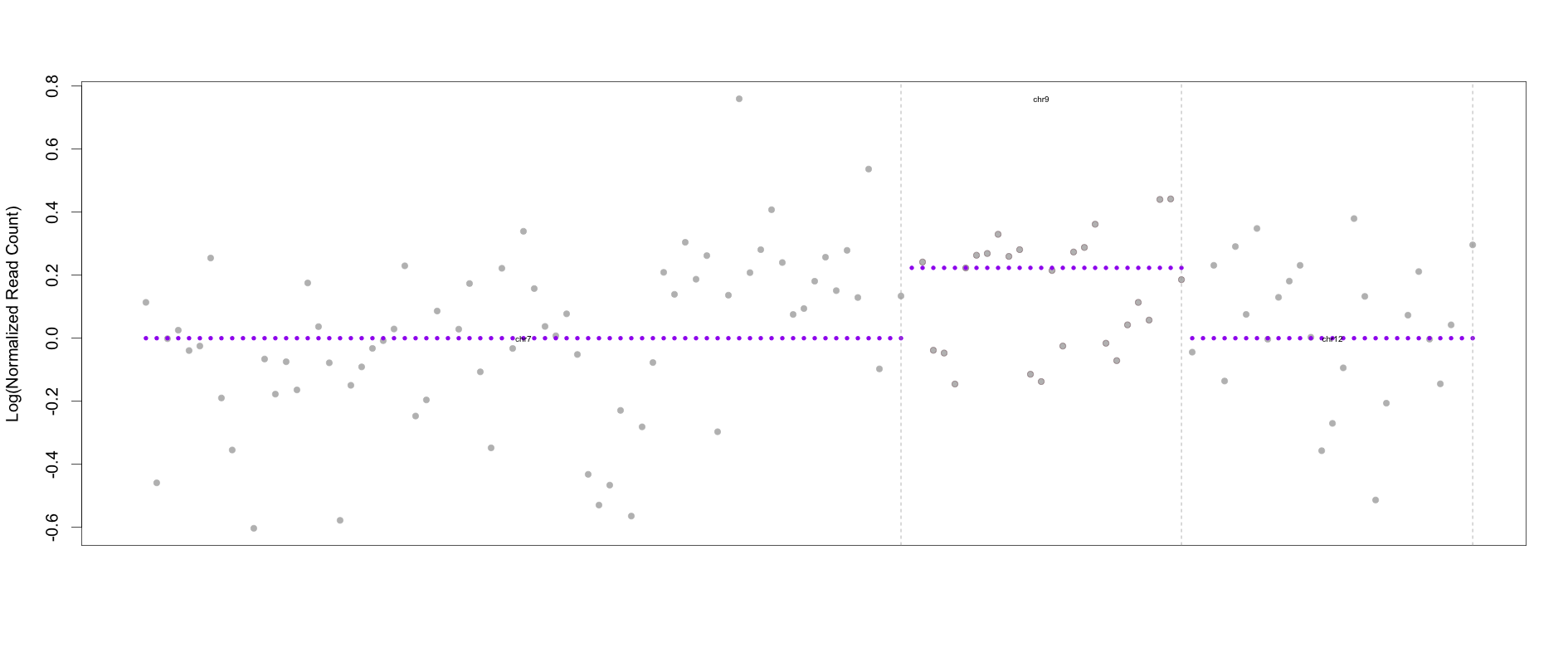

Supplement: Additional file 6 — Archive with ONCOCNV results. Set of files (png plots and txt files) in zip archive that were provided by ONCOCNV after specificity and sensitivity testing. (ZIP 5007 kb) [file 12859_2016_1272_MOESM6_ESM.zip › outputONCOCNV copy 2/IonXpress_025_R_2013_10_18_01_30_51_user_SN2.4.Neonatal.Assay2_new_bed_version_318_chip_v2_Auto_user_SN2.4.Neonatal.Assay2_new_bed_version_318_chip_v2_28.profile.png]

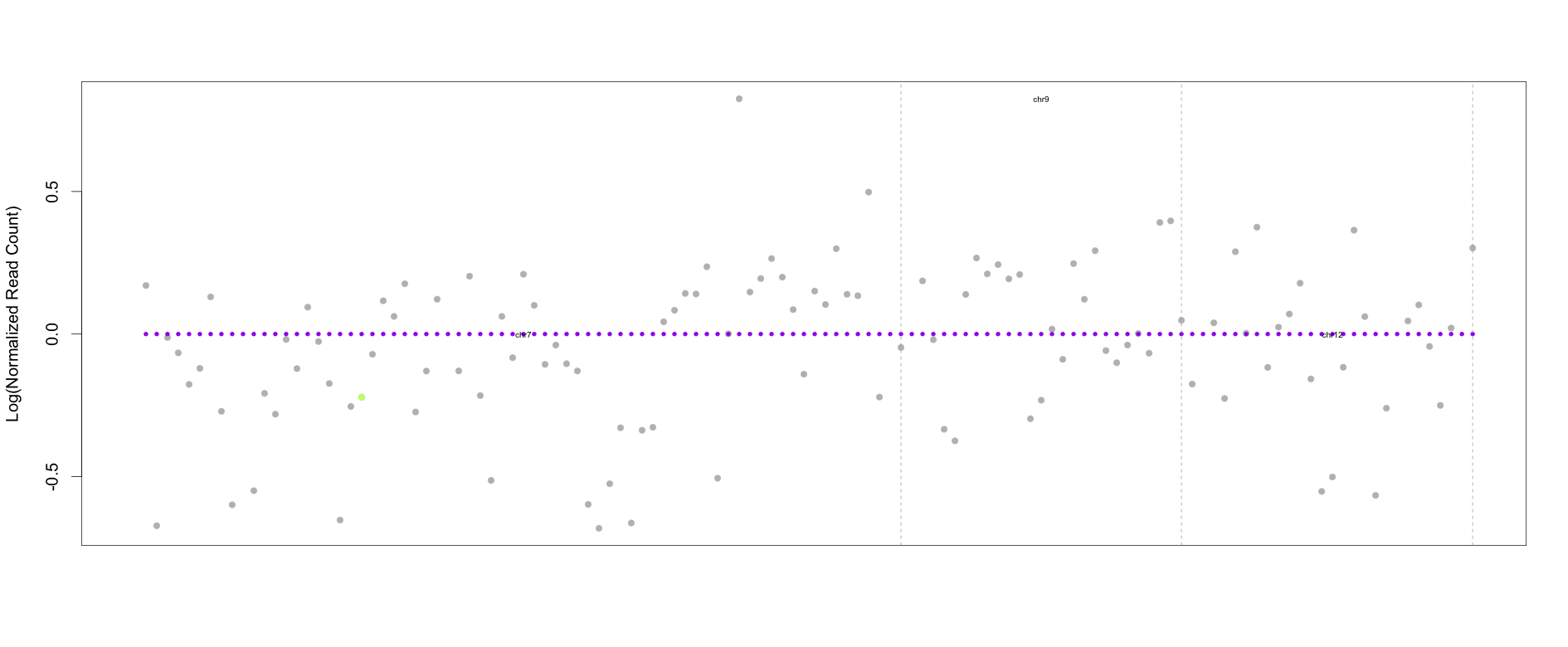

Supplement: Additional file 6 — Archive with ONCOCNV results. Set of files (png plots and txt files) in zip archive that were provided by ONCOCNV after specificity and sensitivity testing. (ZIP 5007 kb) [file 12859_2016_1272_MOESM6_ESM.zip › outputONCOCNV copy 2/IonXpress_026_R_2013_10_18_01_30_51_user_SN2.4.Neonatal.Assay2_new_bed_version_318_chip_v2_Auto_user_SN2.4.Neonatal.Assay2_new_bed_version_318_chip_v2_28.profile.png]

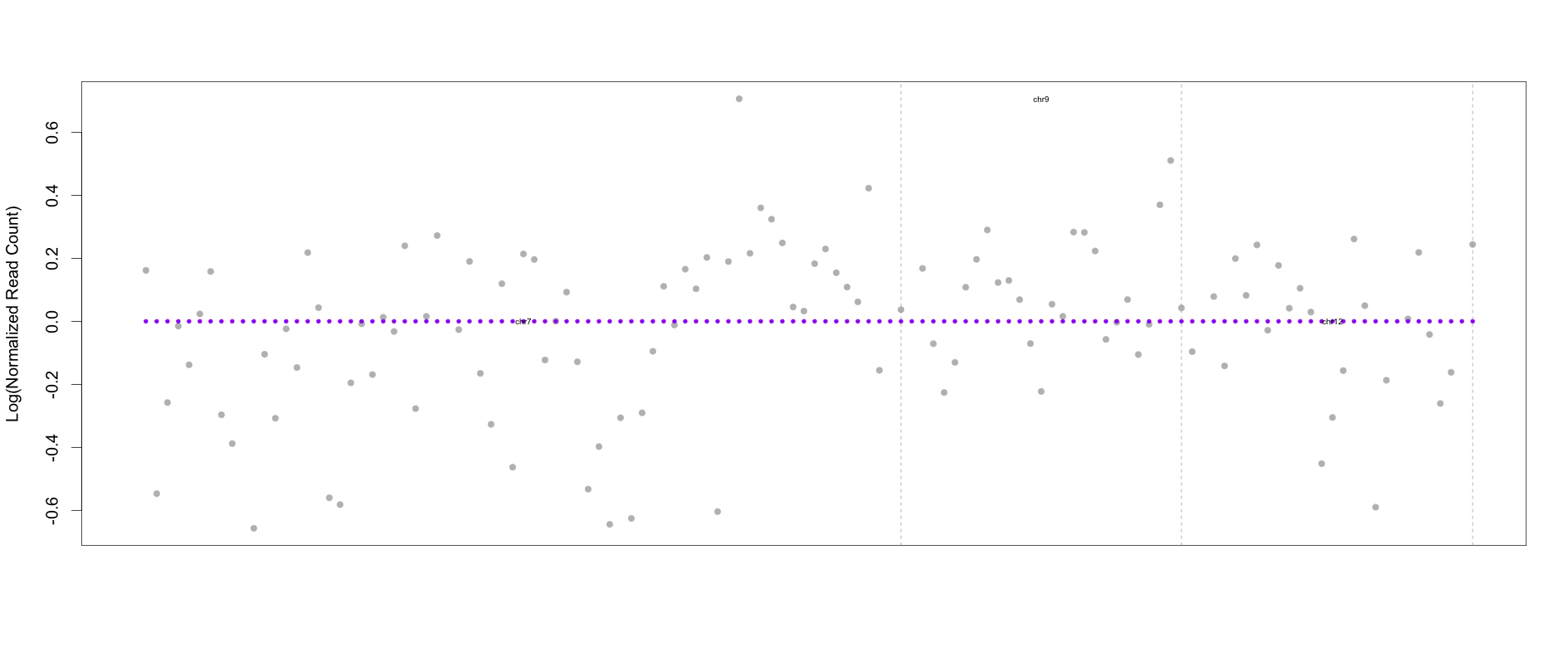

Supplement: Additional file 6 — Archive with ONCOCNV results. Set of files (png plots and txt files) in zip archive that were provided by ONCOCNV after specificity and sensitivity testing. (ZIP 5007 kb) [file 12859_2016_1272_MOESM6_ESM.zip › outputONCOCNV copy 2/IonXpress_027_R_2013_10_18_01_30_51_user_SN2.4.Neonatal.Assay2_new_bed_version_318_chip_v2_Auto_user_SN2.4.Neonatal.Assay2_new_bed_version_318_chip_v2_28.profile.png]

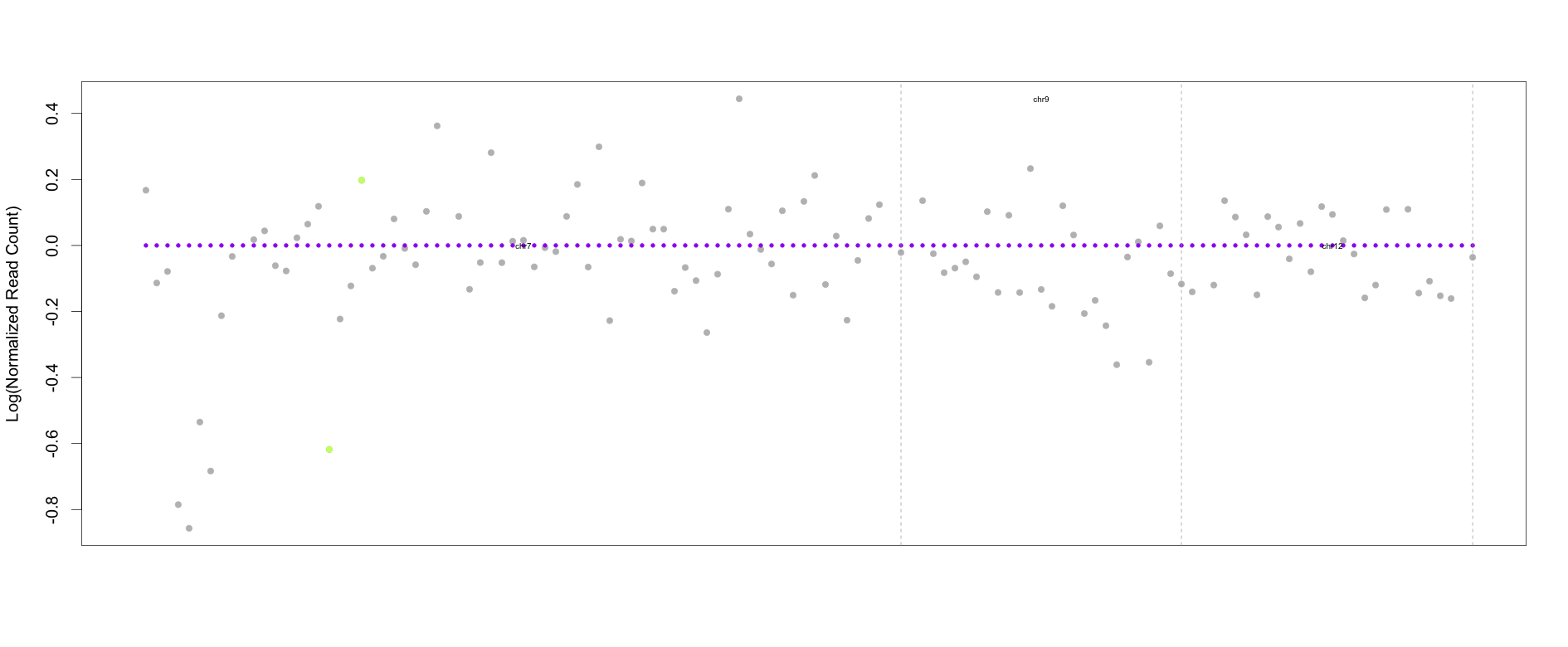

Supplement: Additional file 6 — Archive with ONCOCNV results. Set of files (png plots and txt files) in zip archive that were provided by ONCOCNV after specificity and sensitivity testing. (ZIP 5007 kb) [file 12859_2016_1272_MOESM6_ESM.zip › outputONCOCNV copy 2/IonXpress_028_R_2012_09_13_10_57_38_Sequoia_SN1.27.Run_21_Auto_Sequoia_SN1.27.Run_21_54.profile.png]

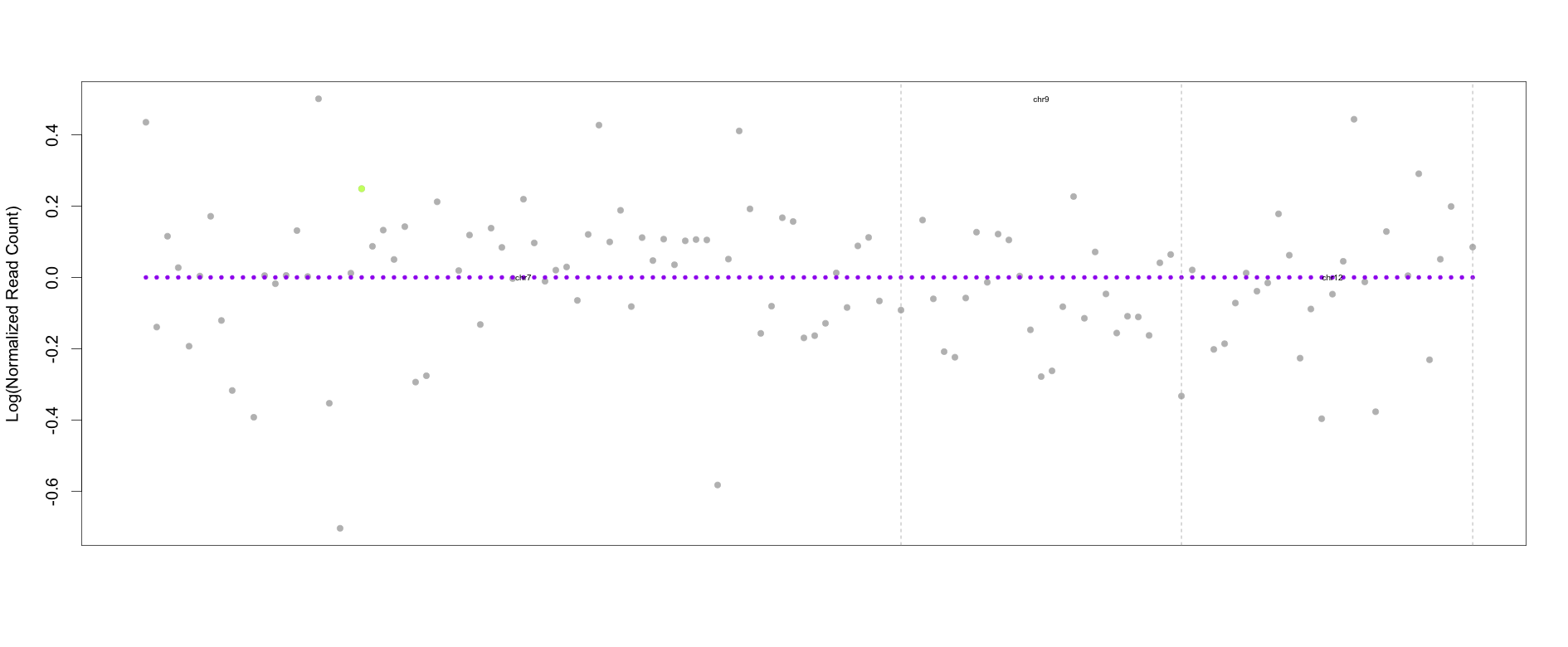

Supplement: Additional file 6 — Archive with ONCOCNV results. Set of files (png plots and txt files) in zip archive that were provided by ONCOCNV after specificity and sensitivity testing. (ZIP 5007 kb) [file 12859_2016_1272_MOESM6_ESM.zip › outputONCOCNV copy 2/IonXpress_028_R_2013_10_18_01_30_51_user_SN2.4.Neonatal.Assay2_new_bed_version_318_chip_v2_Auto_user_SN2.4.Neonatal.Assay2_new_bed_version_318_chip_v2_28.profile.png]

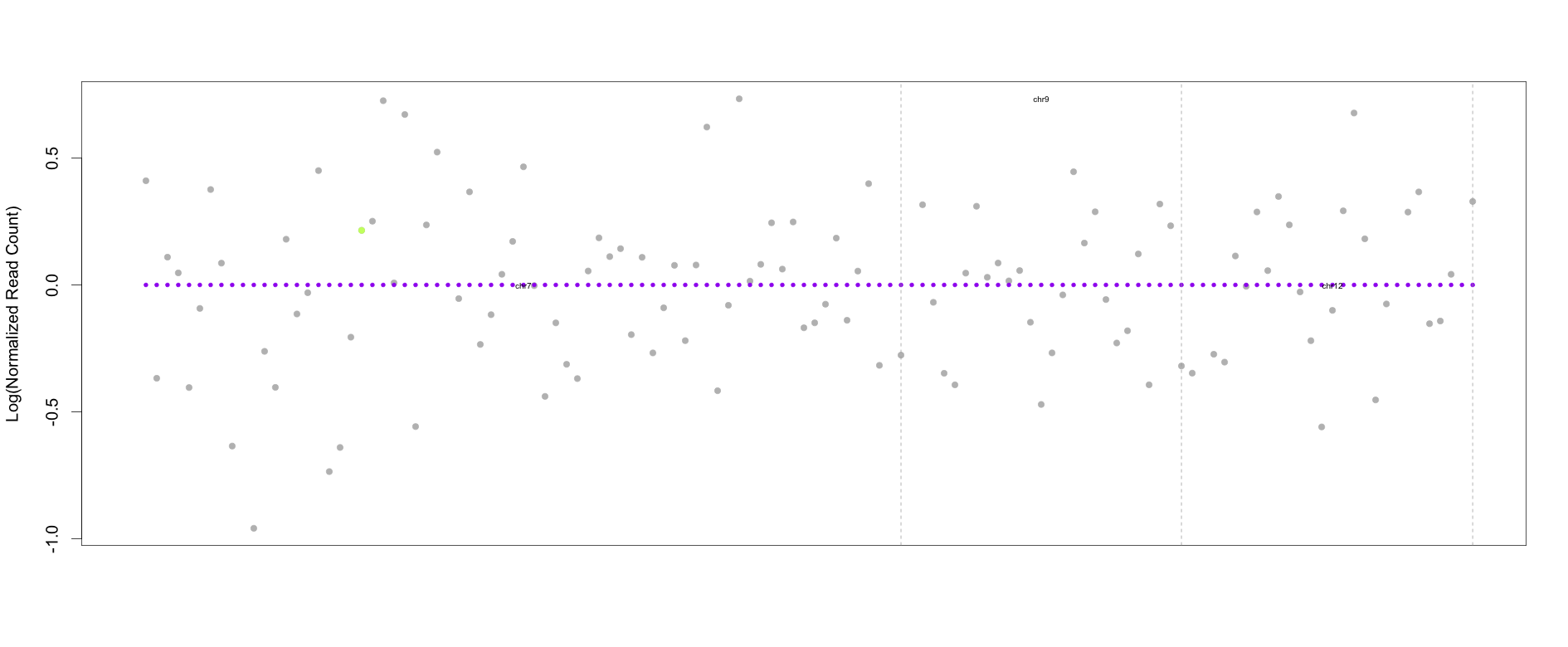

Supplement: Additional file 6 — Archive with ONCOCNV results. Set of files (png plots and txt files) in zip archive that were provided by ONCOCNV after specificity and sensitivity testing. (ZIP 5007 kb) [file 12859_2016_1272_MOESM6_ESM.zip › outputONCOCNV copy 2/IonXpress_030_R_2013_10_18_01_30_51_user_SN2.4.Neonatal.Assay2_new_bed_version_318_chip_v2_Auto_user_SN2.4.Neonatal.Assay2_new_bed_version_318_chip_v2_28.profile.png]

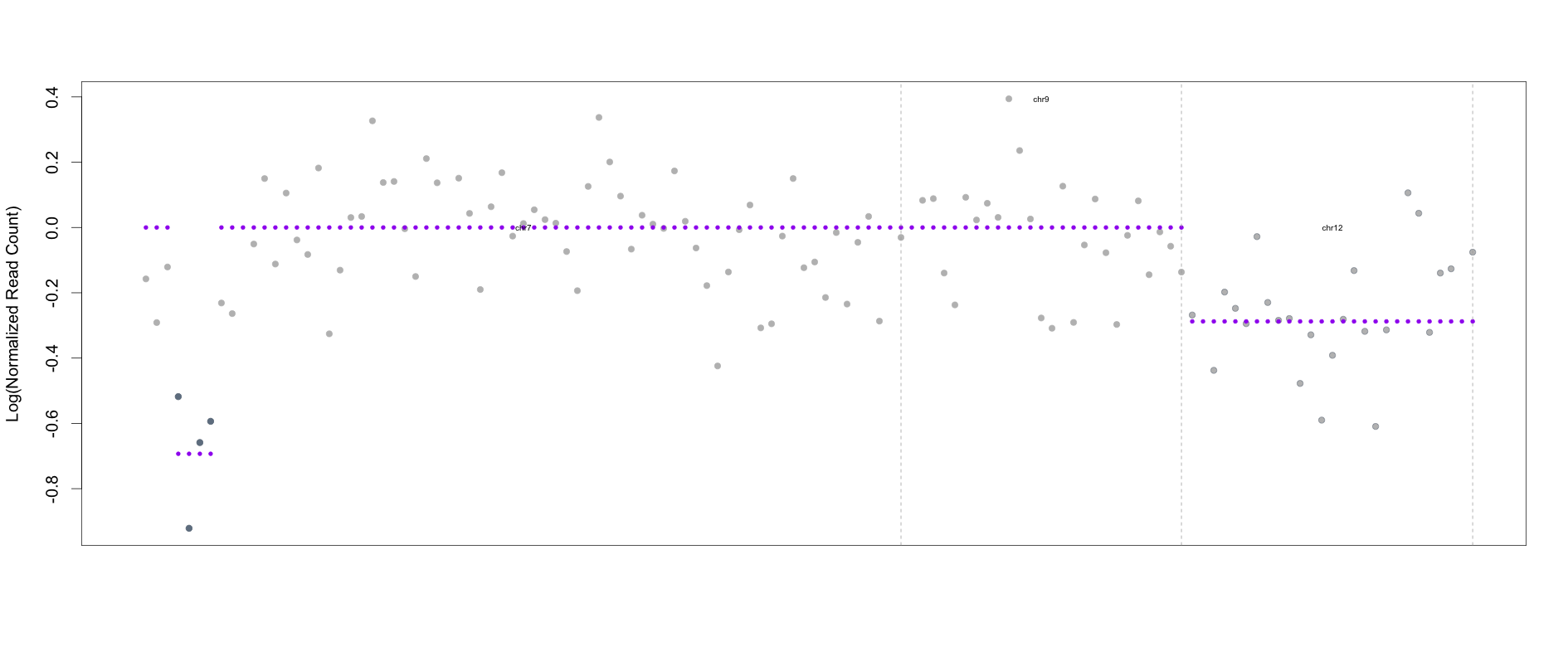

Supplement: Additional file 6 — Archive with ONCOCNV results. Set of files (png plots and txt files) in zip archive that were provided by ONCOCNV after specificity and sensitivity testing. (ZIP 5007 kb) [file 12859_2016_1272_MOESM6_ESM.zip › outputONCOCNV copy 2/IonXpress_032_R_2012_09_13_10_57_38_Sequoia_SN1.27.Run_21_Auto_Sequoia_SN1.27.Run_21_54.profile.png]

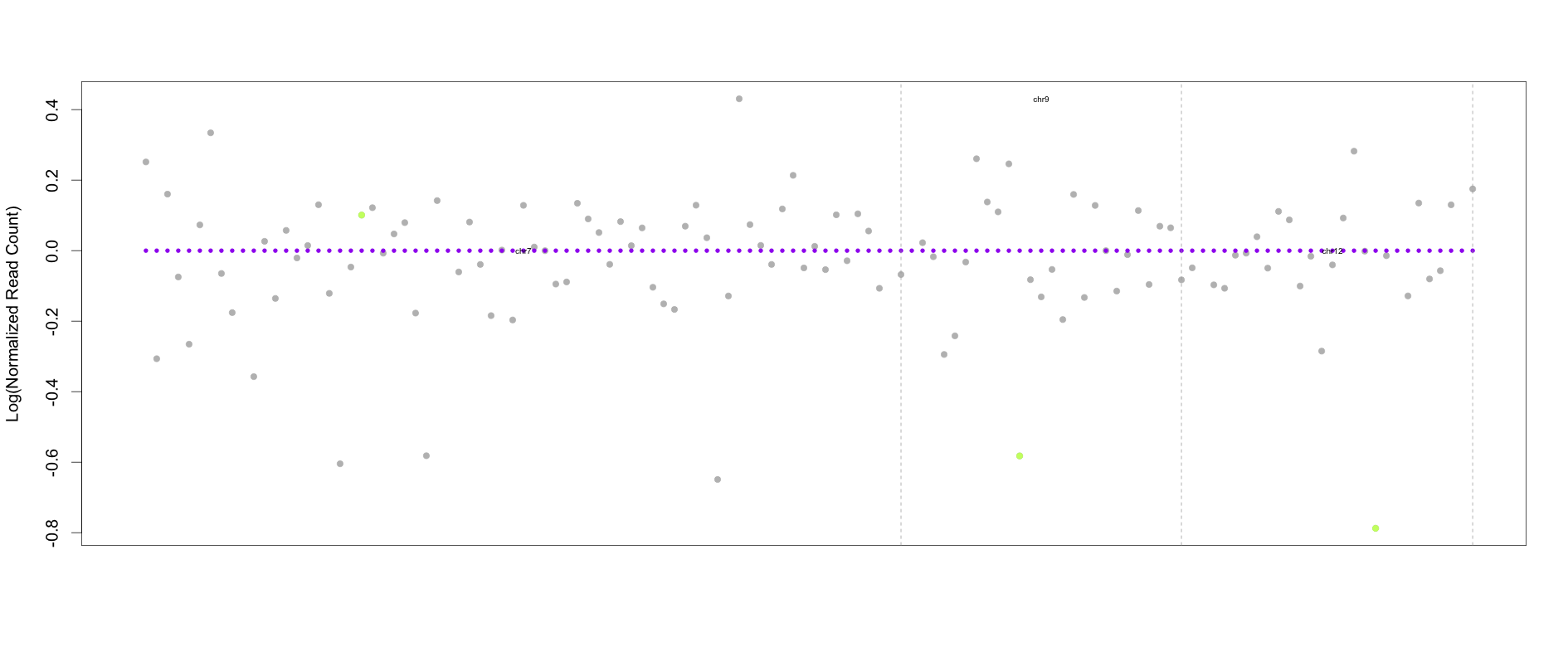

Supplement: Additional file 6 — Archive with ONCOCNV results. Set of files (png plots and txt files) in zip archive that were provided by ONCOCNV after specificity and sensitivity testing. (ZIP 5007 kb) [file 12859_2016_1272_MOESM6_ESM.zip › outputONCOCNV copy 2/IonXpress_039_R_2013_10_18_01_30_51_user_SN2.4.Neonatal.Assay2_new_bed_version_318_chip_v2_Auto_user_SN2.4.Neonatal.Assay2_new_bed_version_318_chip_v2_28.profile.png]

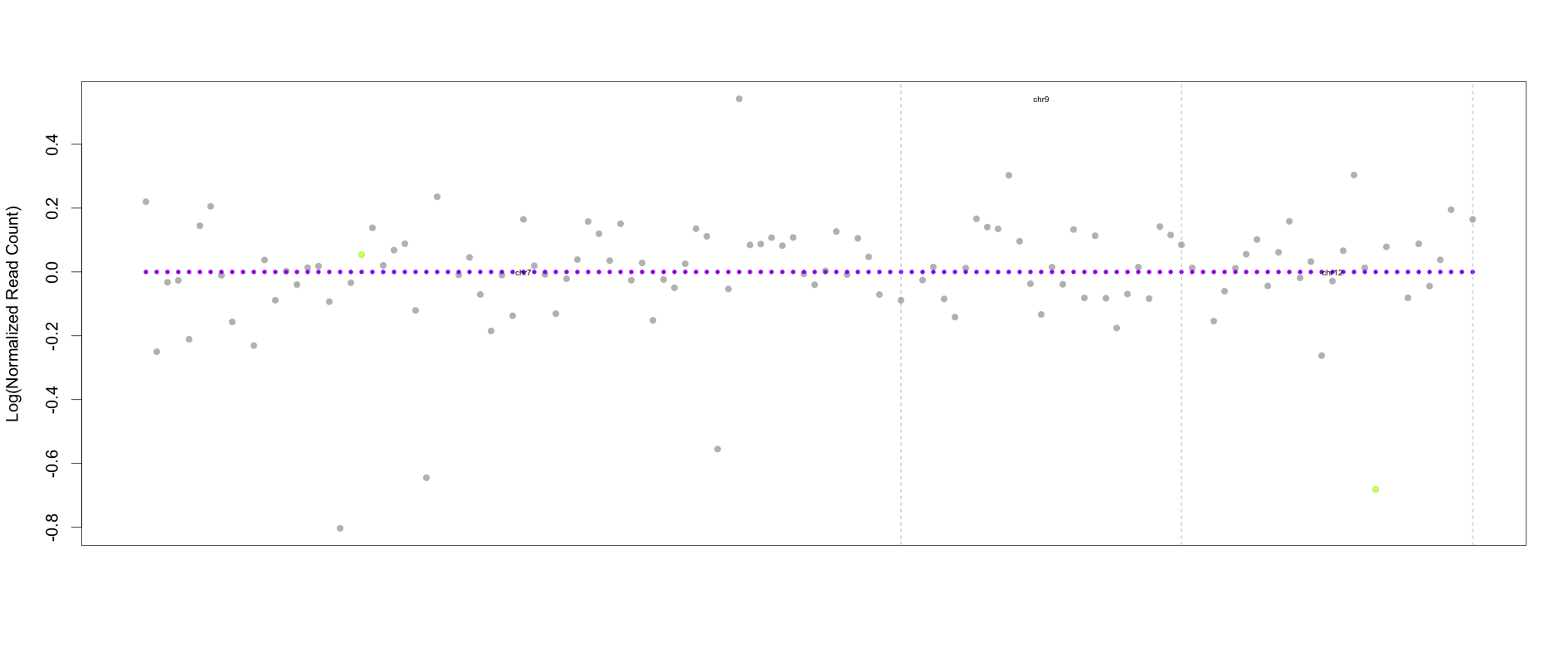

Supplement: Additional file 6 — Archive with ONCOCNV results. Set of files (png plots and txt files) in zip archive that were provided by ONCOCNV after specificity and sensitivity testing. (ZIP 5007 kb) [file 12859_2016_1272_MOESM6_ESM.zip › outputONCOCNV copy 2/IonXpress_040_R_2013_10_18_01_30_51_user_SN2.4.Neonatal.Assay2_new_bed_version_318_chip_v2_Auto_user_SN2.4.Neonatal.Assay2_new_bed_version_318_chip_v2_28.profile.png]

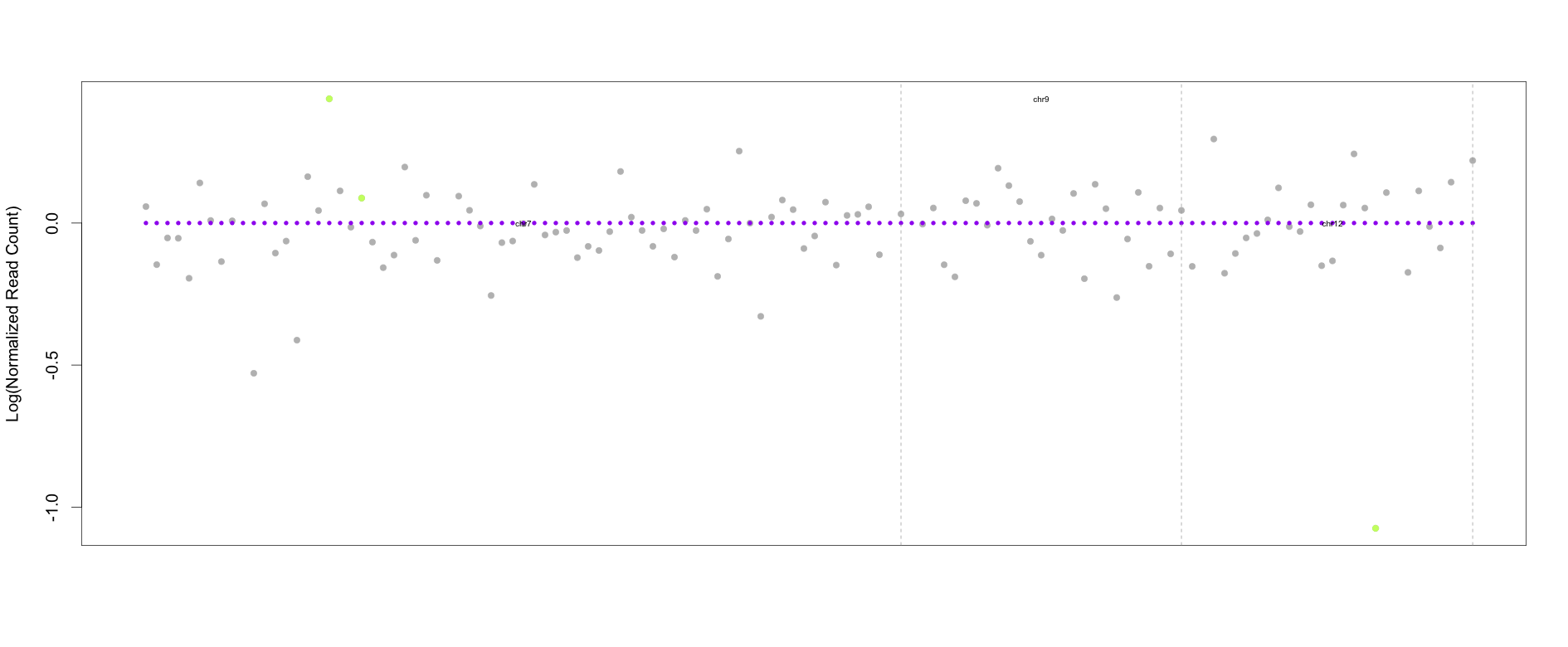

Supplement: Additional file 6 — Archive with ONCOCNV results. Set of files (png plots and txt files) in zip archive that were provided by ONCOCNV after specificity and sensitivity testing. (ZIP 5007 kb) [file 12859_2016_1272_MOESM6_ESM.zip › outputONCOCNV copy 2/IonXpress_041_R_2013_10_18_01_30_51_user_SN2.4.Neonatal.Assay2_new_bed_version_318_chip_v2_Auto_user_SN2.4.Neonatal.Assay2_new_bed_version_318_chip_v2_28.profile.png]

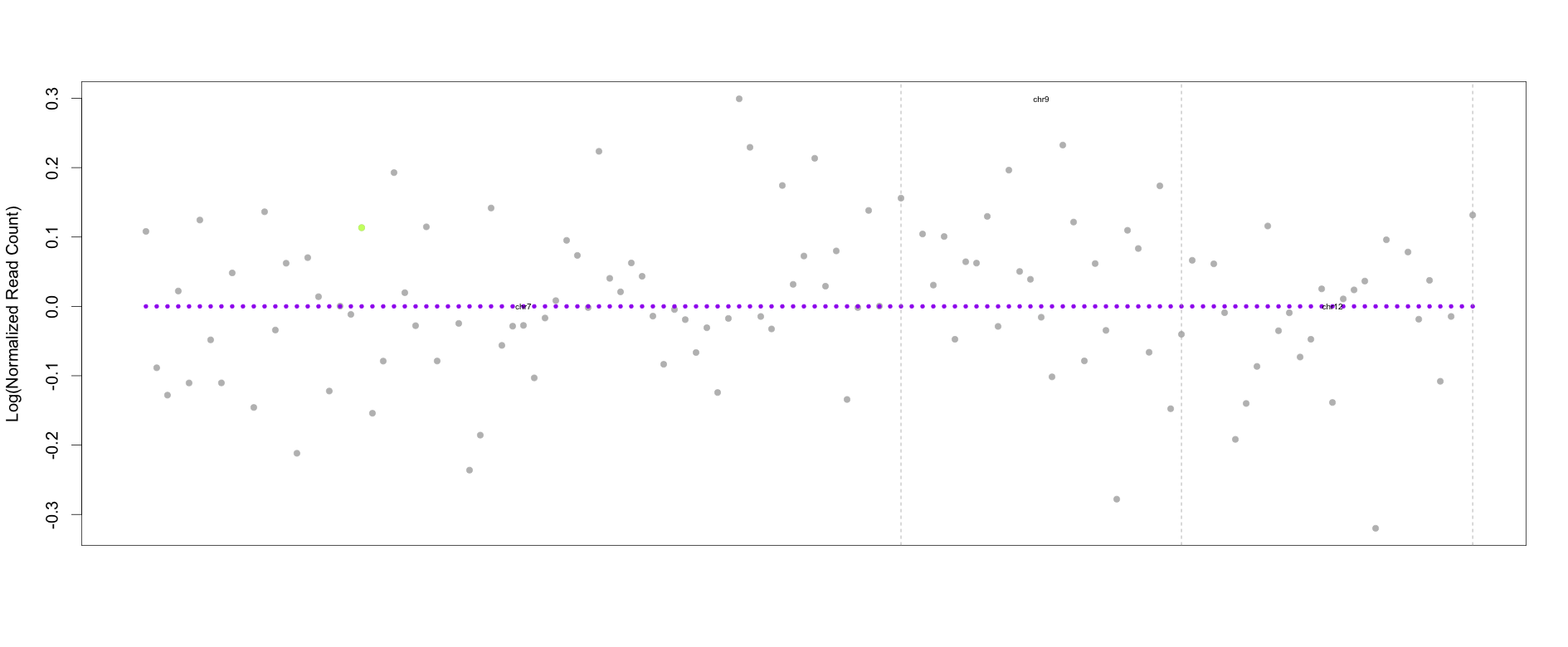

Supplement: Additional file 6 — Archive with ONCOCNV results. Set of files (png plots and txt files) in zip archive that were provided by ONCOCNV after specificity and sensitivity testing. (ZIP 5007 kb) [file 12859_2016_1272_MOESM6_ESM.zip › outputONCOCNV copy 2/IonXpress_042_R_2012_09_13_10_57_38_Sequoia_SN1.27.Run_21_Auto_Sequoia_SN1.27.Run_21_54.profile.png]

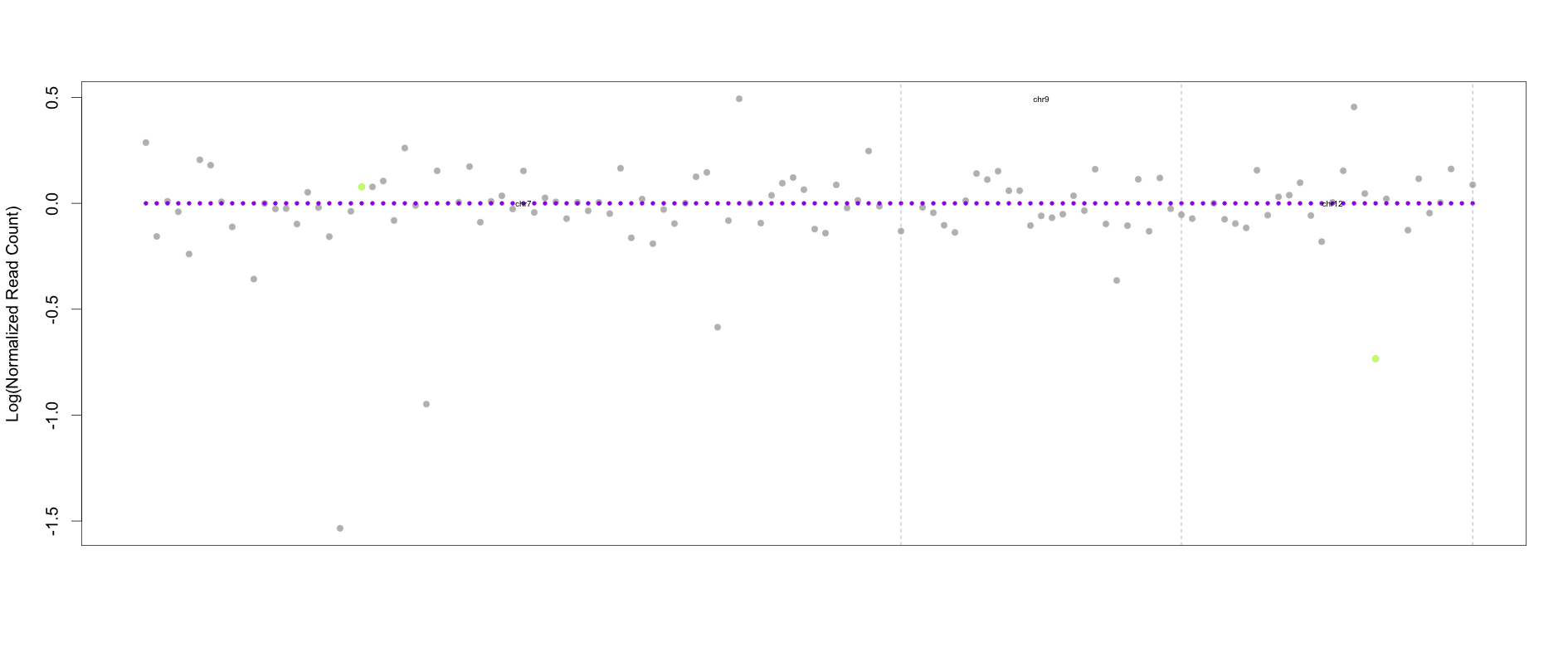

Supplement: Additional file 6 — Archive with ONCOCNV results. Set of files (png plots and txt files) in zip archive that were provided by ONCOCNV after specificity and sensitivity testing. (ZIP 5007 kb) [file 12859_2016_1272_MOESM6_ESM.zip › outputONCOCNV copy 2/IonXpress_042_R_2013_10_18_01_30_51_user_SN2.4.Neonatal.Assay2_new_bed_version_318_chip_v2_Auto_user_SN2.4.Neonatal.Assay2_new_bed_version_318_chip_v2_28.profile.png]

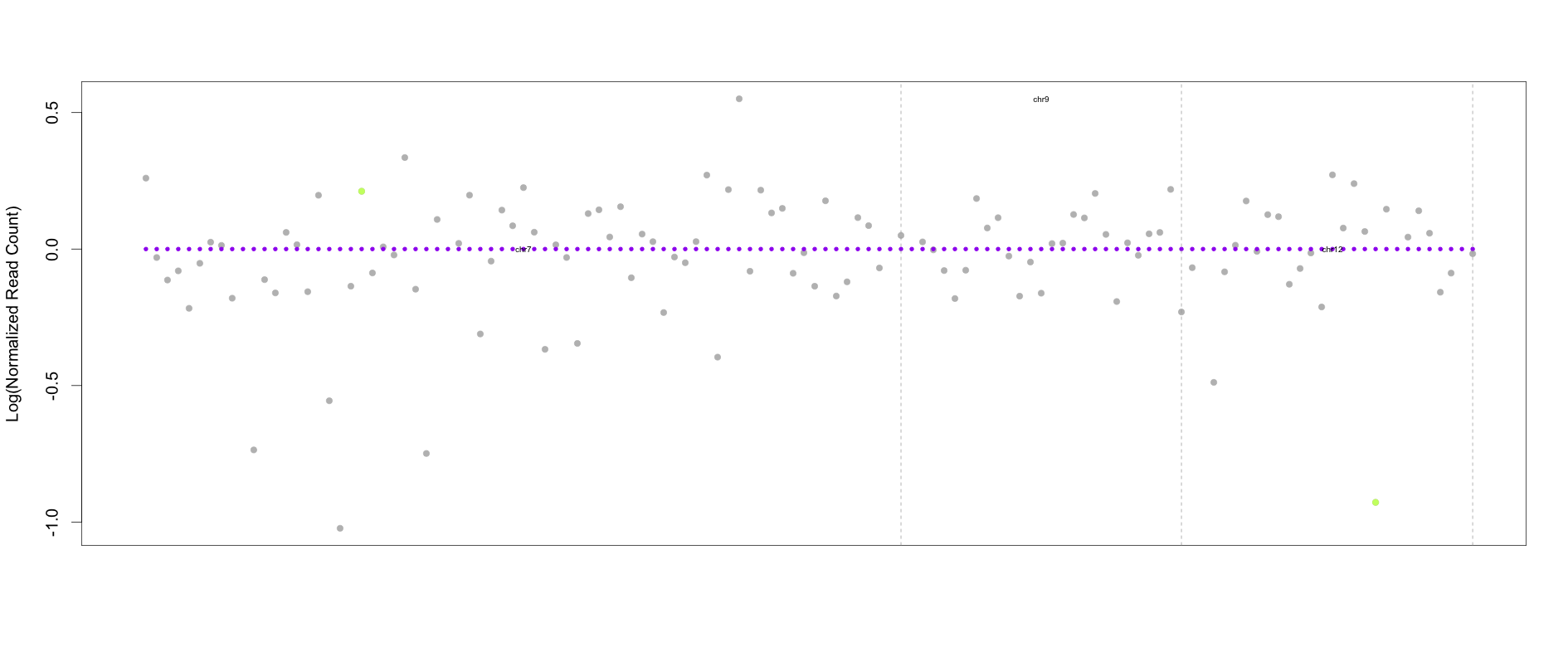

Supplement: Additional file 6 — Archive with ONCOCNV results. Set of files (png plots and txt files) in zip archive that were provided by ONCOCNV after specificity and sensitivity testing. (ZIP 5007 kb) [file 12859_2016_1272_MOESM6_ESM.zip › outputONCOCNV copy 2/IonXpress_043_R_2013_10_18_01_30_51_user_SN2.4.Neonatal.Assay2_new_bed_version_318_chip_v2_Auto_user_SN2.4.Neonatal.Assay2_new_bed_version_318_chip_v2_28.profile.png]

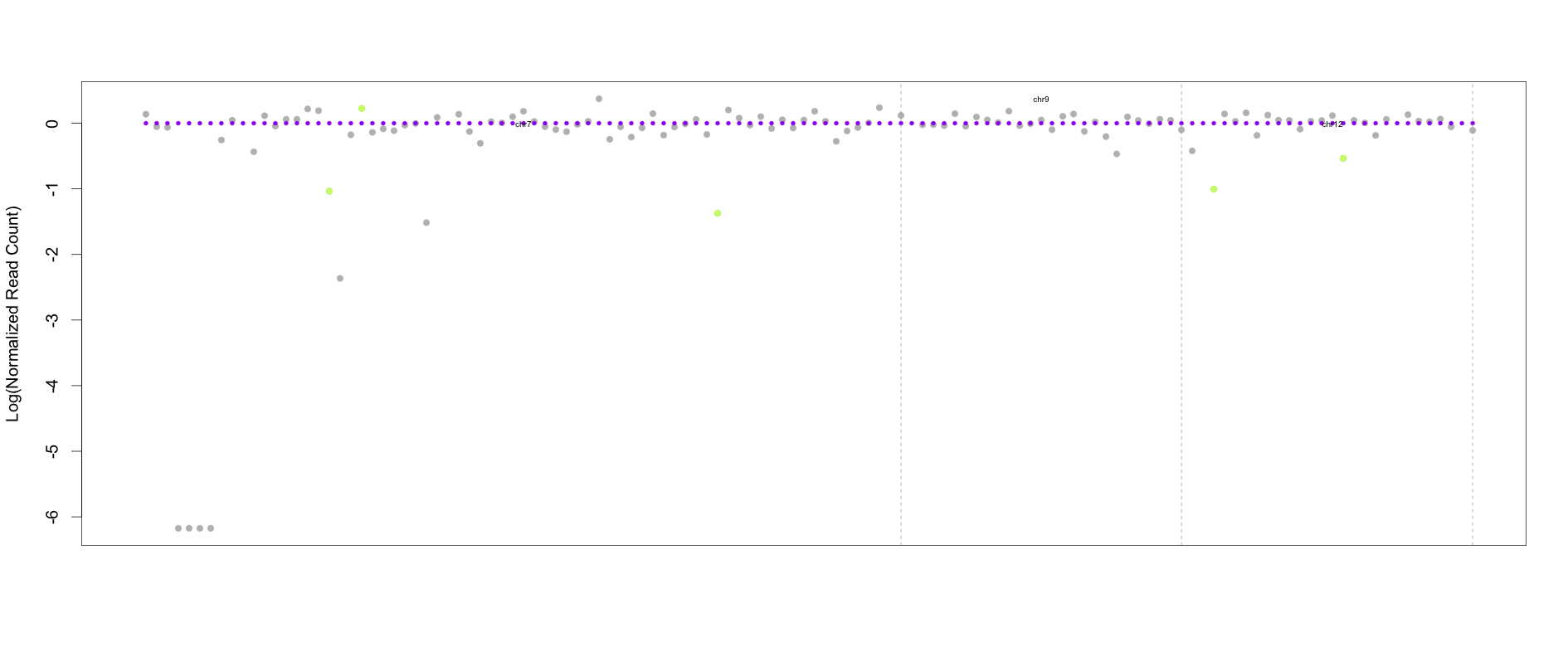

Supplement: Additional file 6 — Archive with ONCOCNV results. Set of files (png plots and txt files) in zip archive that were provided by ONCOCNV after specificity and sensitivity testing. (ZIP 5007 kb) [file 12859_2016_1272_MOESM6_ESM.zip › outputONCOCNV copy 2/IonXpress_044_R_2012_09_13_15_10_42_Sequoia_SN1.28.Run_22_Auto_Sequoia_SN1.28.Run_22_55.profile.png]

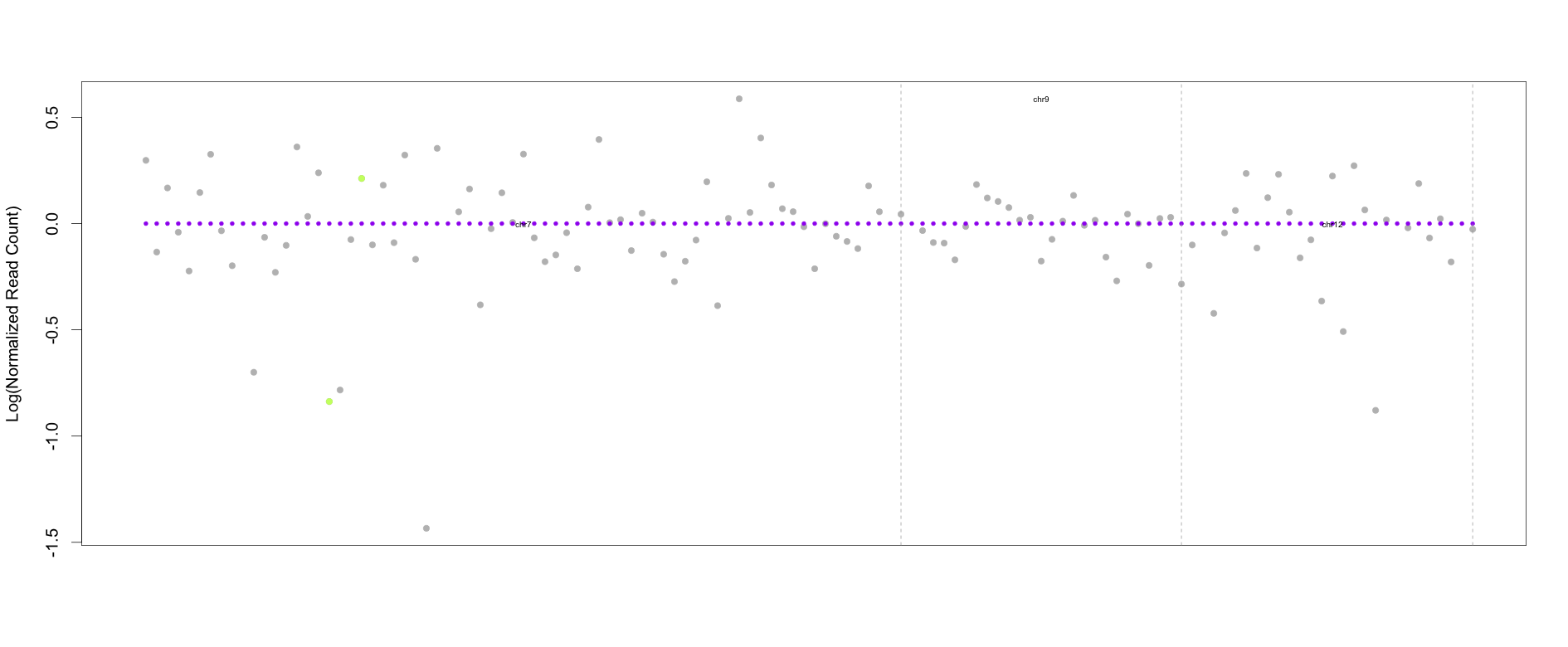

Supplement: Additional file 6 — Archive with ONCOCNV results. Set of files (png plots and txt files) in zip archive that were provided by ONCOCNV after specificity and sensitivity testing. (ZIP 5007 kb) [file 12859_2016_1272_MOESM6_ESM.zip › outputONCOCNV copy 2/IonXpress_044_R_2013_10_18_01_30_51_user_SN2.4.Neonatal.Assay2_new_bed_version_318_chip_v2_Auto_user_SN2.4.Neonatal.Assay2_new_bed_version_318_chip_v2_28.profile.png]

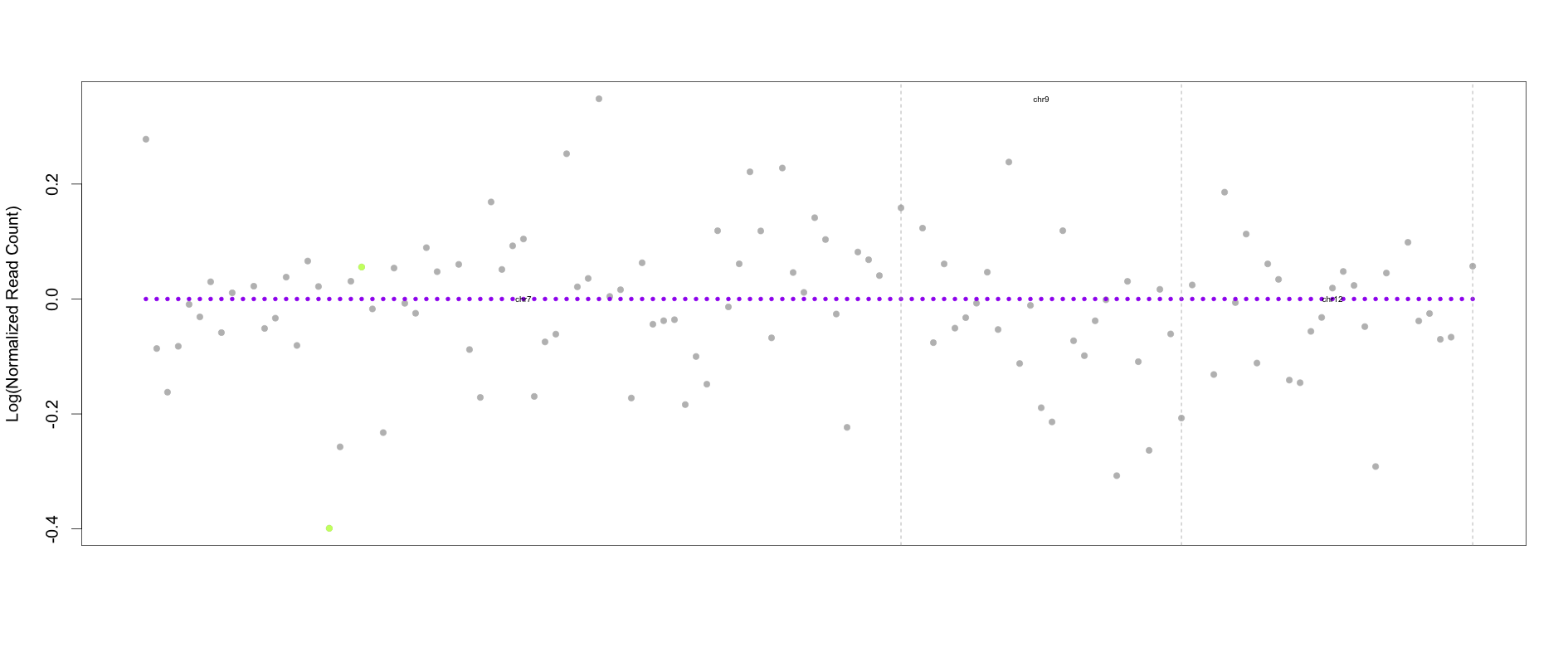

Supplement: Additional file 6 — Archive with ONCOCNV results. Set of files (png plots and txt files) in zip archive that were provided by ONCOCNV after specificity and sensitivity testing. (ZIP 5007 kb) [file 12859_2016_1272_MOESM6_ESM.zip › outputONCOCNV copy 2/IonXpress_045_R_2012_09_13_10_57_38_Sequoia_SN1.27.Run_21_Auto_Sequoia_SN1.27.Run_21_54.profile.png]

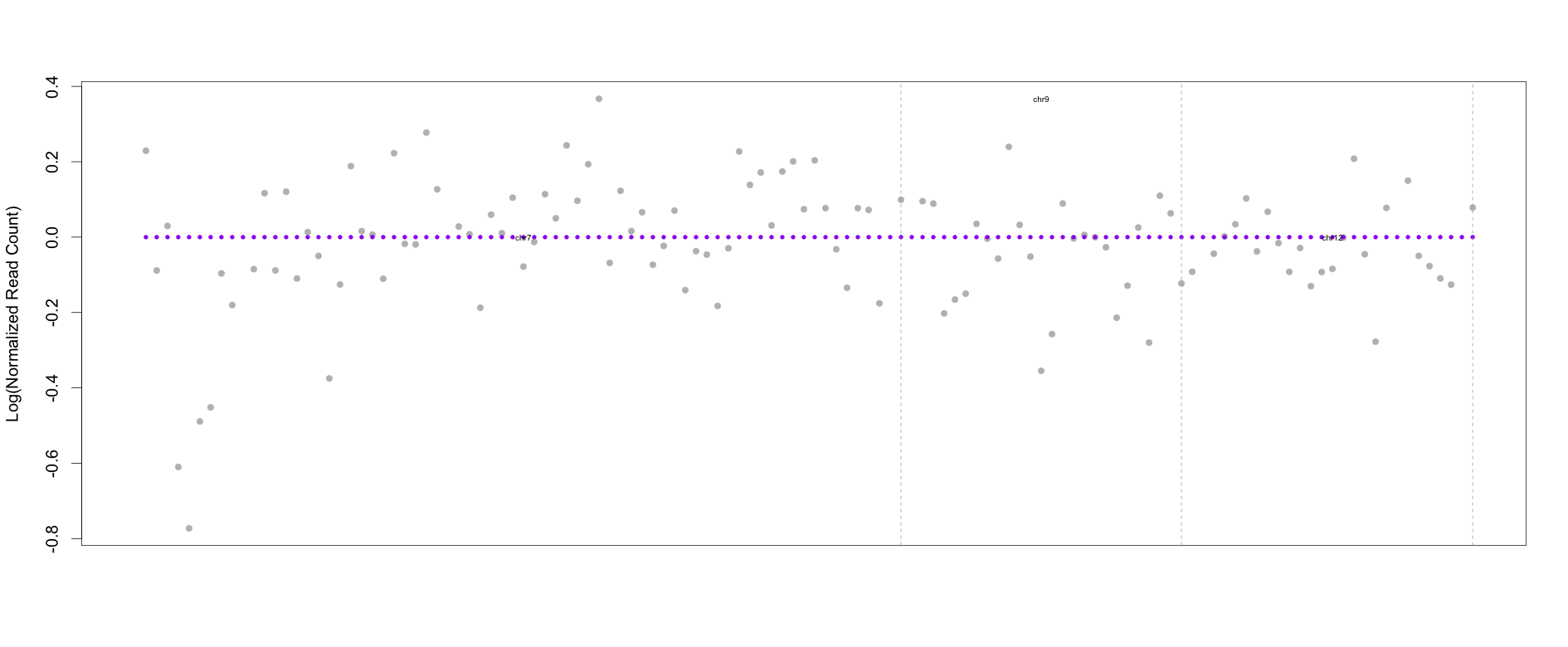

Supplement: Additional file 6 — Archive with ONCOCNV results. Set of files (png plots and txt files) in zip archive that were provided by ONCOCNV after specificity and sensitivity testing. (ZIP 5007 kb) [file 12859_2016_1272_MOESM6_ESM.zip › outputONCOCNV copy 2/IonXpress_046_R_2012_09_13_10_57_38_Sequoia_SN1.27.Run_21_Auto_Sequoia_SN1.27.Run_21_54.profile.png]

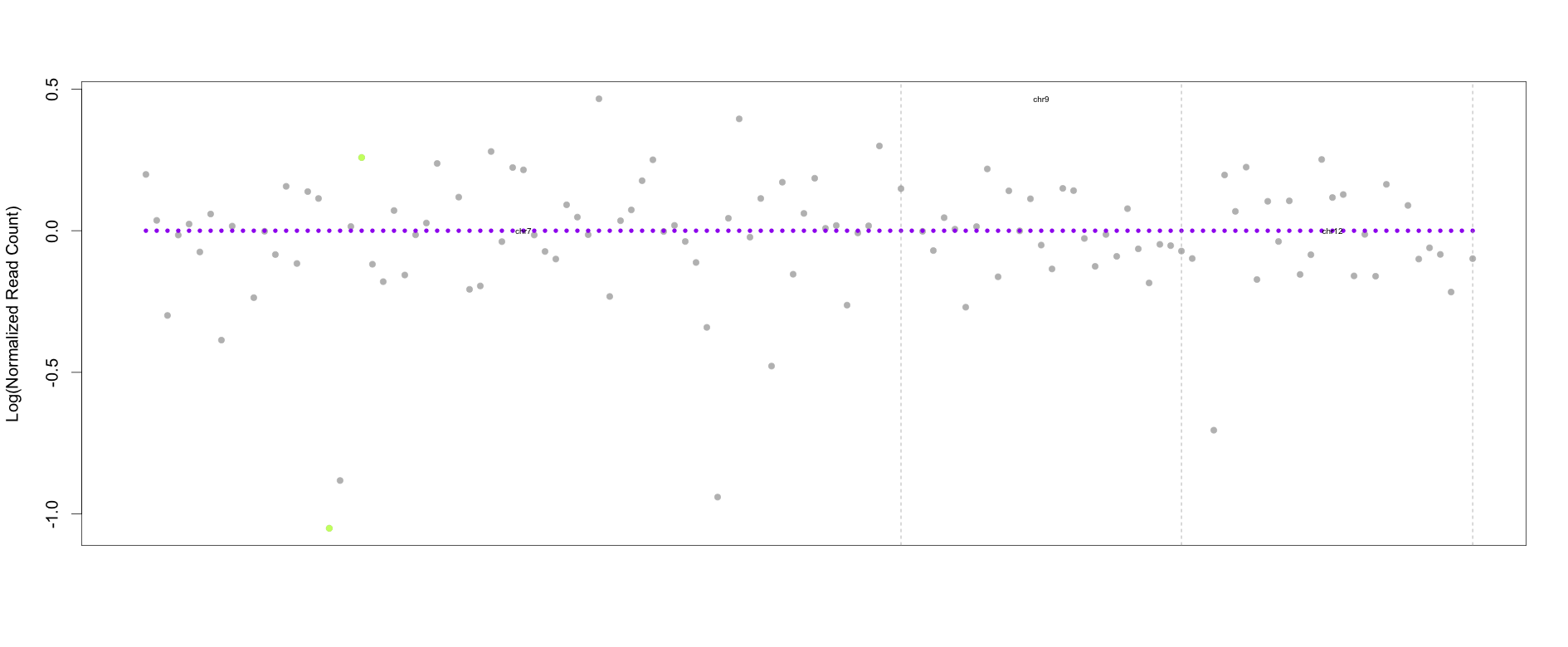

Supplement: Additional file 6 — Archive with ONCOCNV results. Set of files (png plots and txt files) in zip archive that were provided by ONCOCNV after specificity and sensitivity testing. (ZIP 5007 kb) [file 12859_2016_1272_MOESM6_ESM.zip › outputONCOCNV copy 2/IonXpress_047_R_2012_09_13_10_57_38_Sequoia_SN1.27.Run_21_Auto_Sequoia_SN1.27.Run_21_54.profile.png]

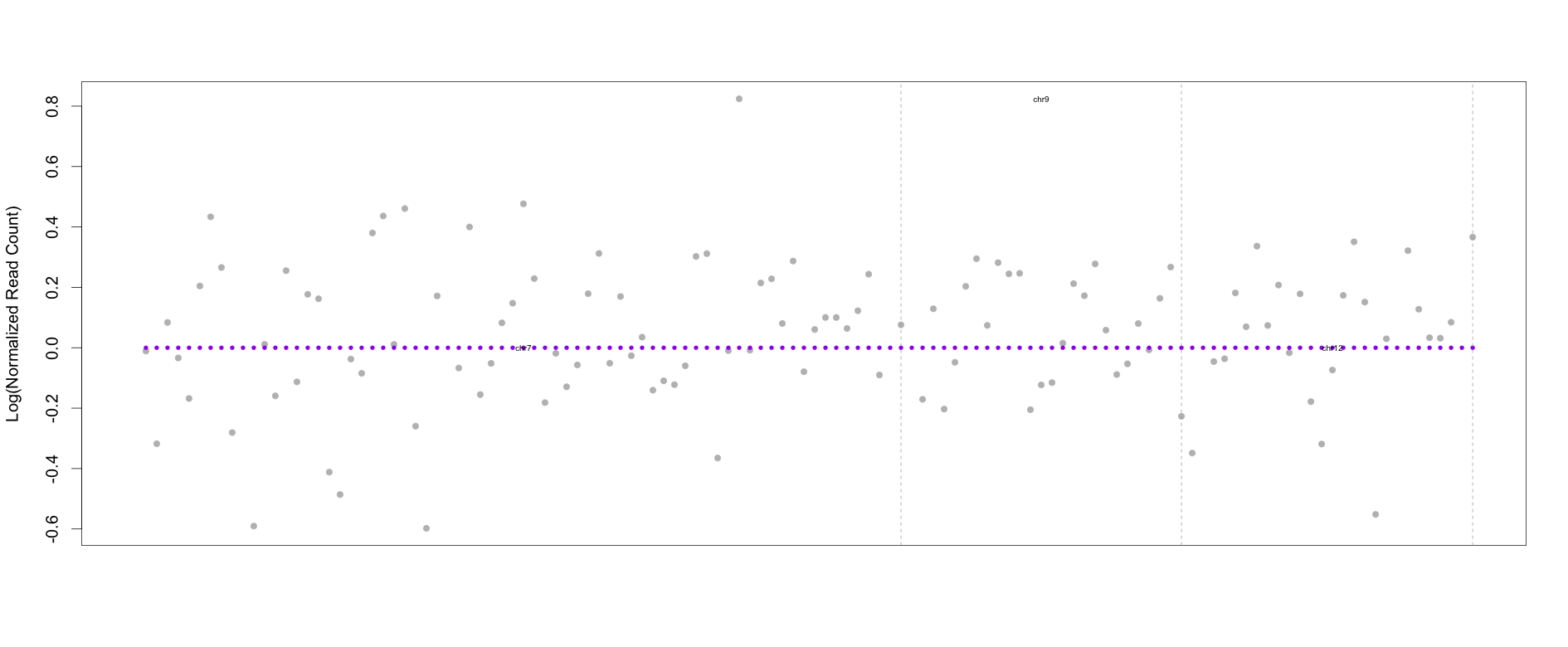

Supplement: Additional file 6 — Archive with ONCOCNV results. Set of files (png plots and txt files) in zip archive that were provided by ONCOCNV after specificity and sensitivity testing. (ZIP 5007 kb) [file 12859_2016_1272_MOESM6_ESM.zip › outputONCOCNV copy 2/MB.1.profile.png]

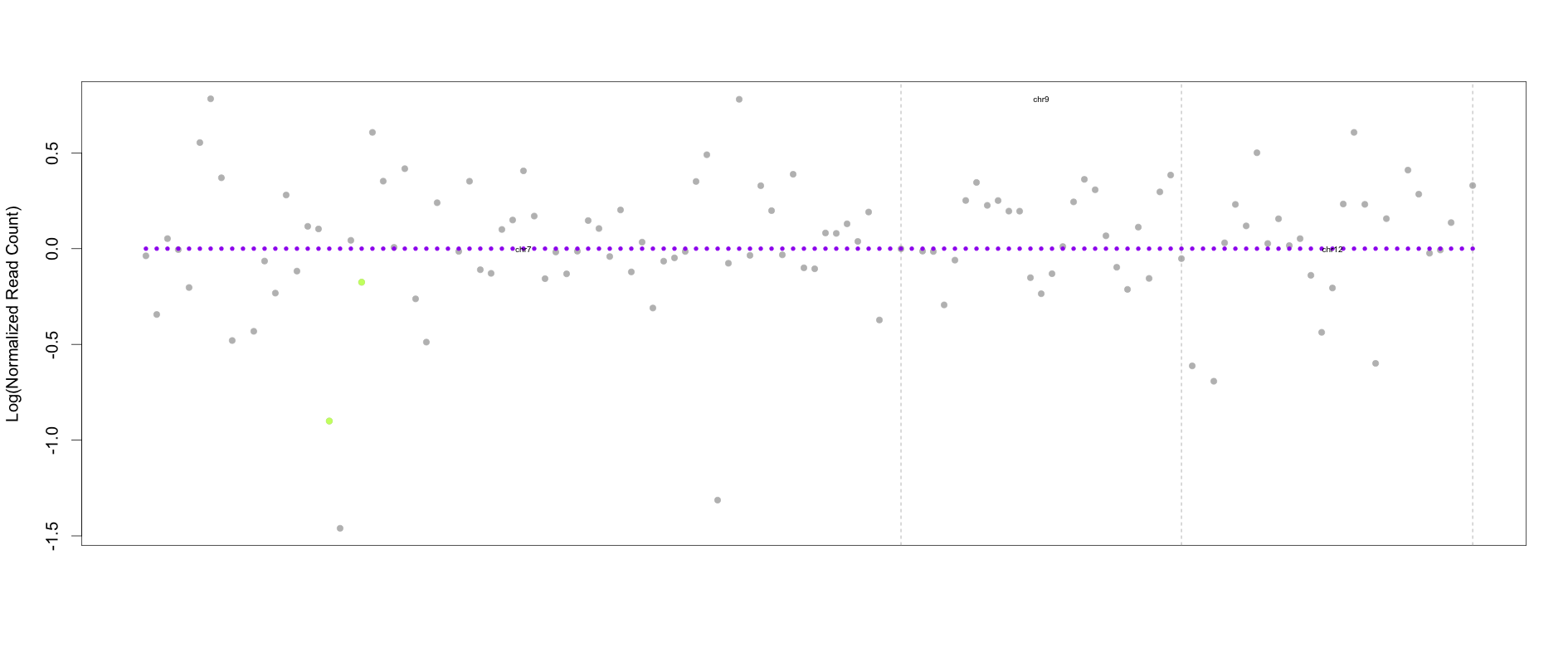

Supplement: Additional file 6 — Archive with ONCOCNV results. Set of files (png plots and txt files) in zip archive that were provided by ONCOCNV after specificity and sensitivity testing. (ZIP 5007 kb) [file 12859_2016_1272_MOESM6_ESM.zip › outputONCOCNV copy 2/MB.3.profile.png]

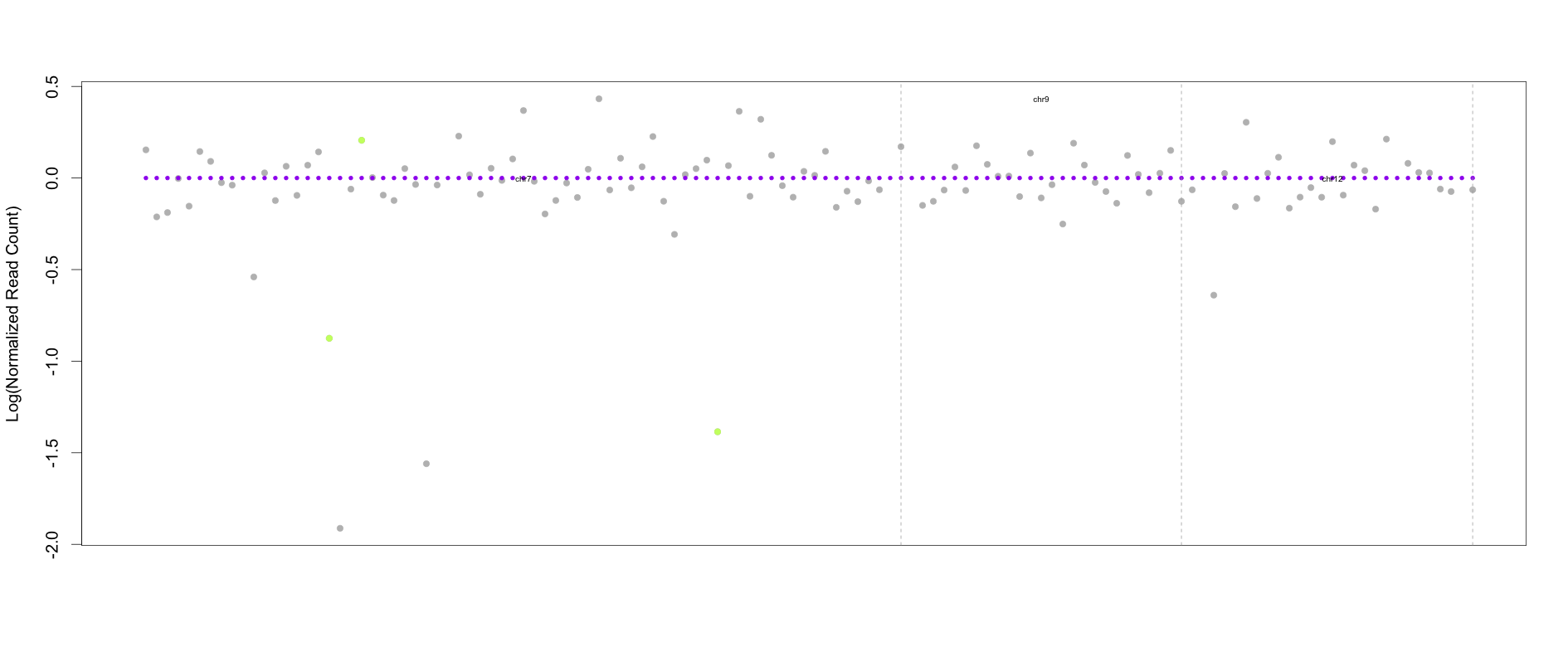

Supplement: Additional file 6 — Archive with ONCOCNV results. Set of files (png plots and txt files) in zip archive that were provided by ONCOCNV after specificity and sensitivity testing. (ZIP 5007 kb) [file 12859_2016_1272_MOESM6_ESM.zip › outputONCOCNV copy 2/X1599.1.1.profile.png]

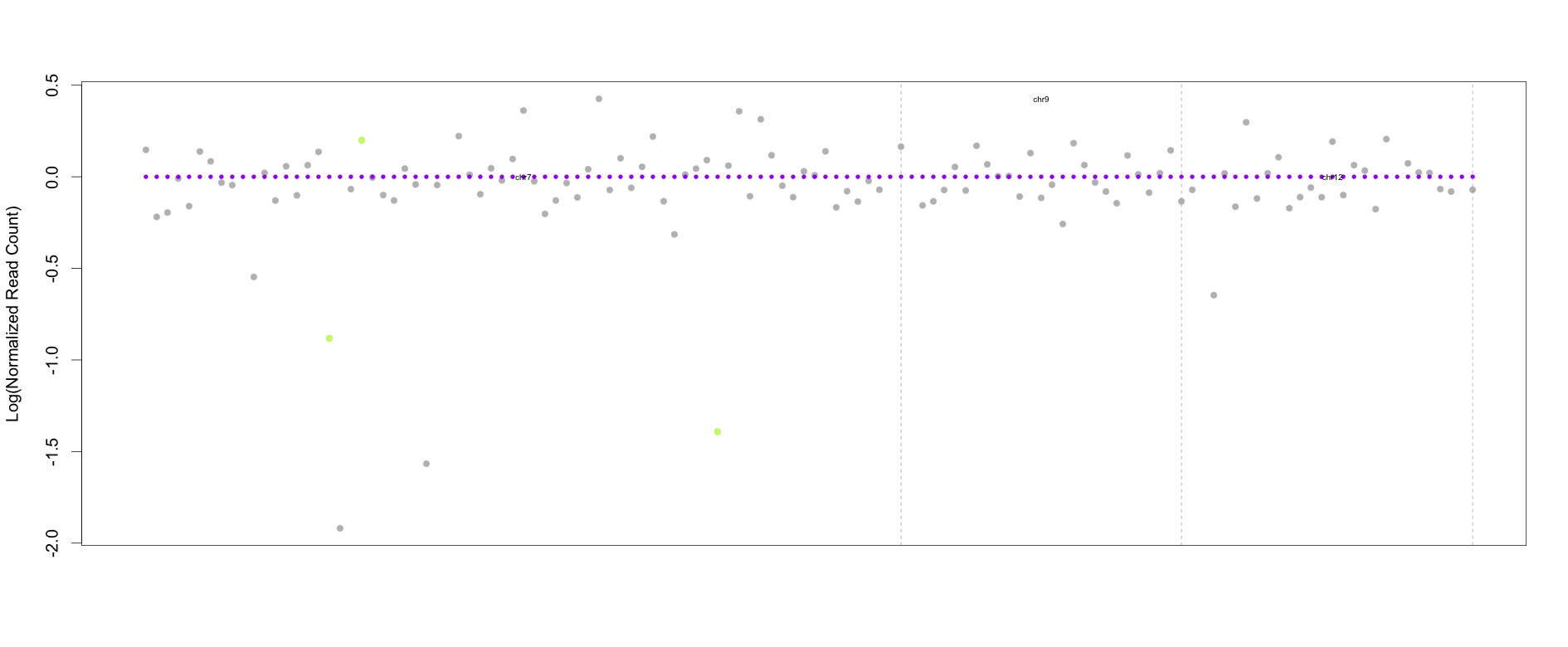

Supplement: Additional file 6 — Archive with ONCOCNV results. Set of files (png plots and txt files) in zip archive that were provided by ONCOCNV after specificity and sensitivity testing. (ZIP 5007 kb) [file 12859_2016_1272_MOESM6_ESM.zip › outputONCOCNV copy 2/X1599.1.profile.png]

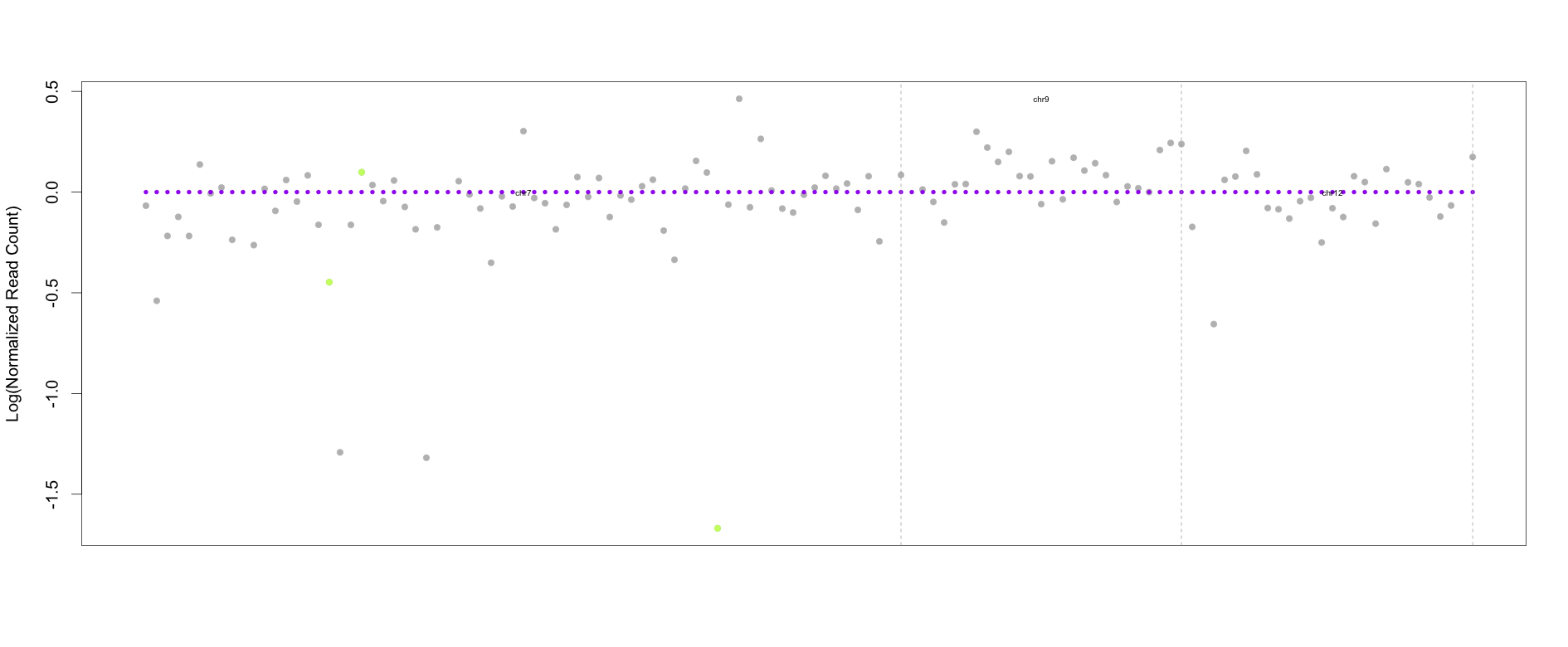

Supplement: Additional file 6 — Archive with ONCOCNV results. Set of files (png plots and txt files) in zip archive that were provided by ONCOCNV after specificity and sensitivity testing. (ZIP 5007 kb) [file 12859_2016_1272_MOESM6_ESM.zip › outputONCOCNV copy 2/X1602.1.1.profile.png]

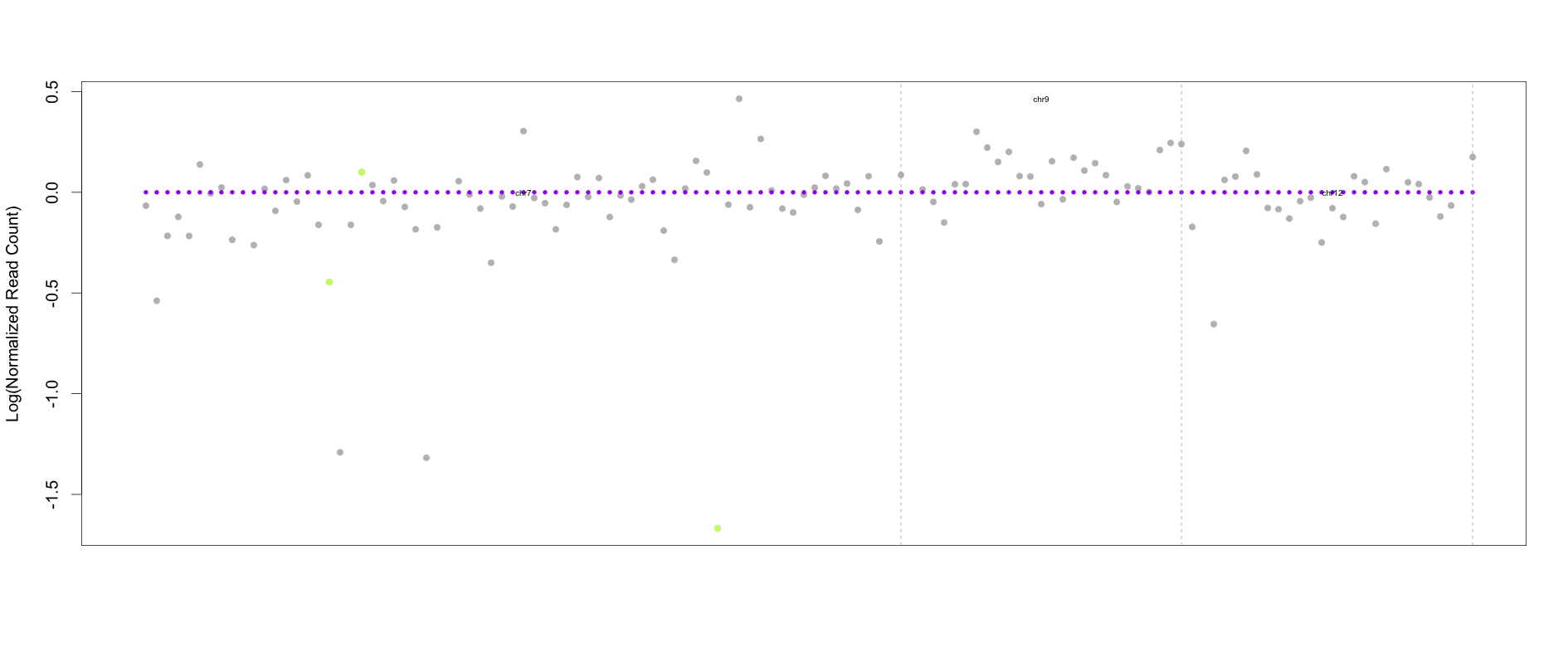

Supplement: Additional file 6 — Archive with ONCOCNV results. Set of files (png plots and txt files) in zip archive that were provided by ONCOCNV after specificity and sensitivity testing. (ZIP 5007 kb) [file 12859_2016_1272_MOESM6_ESM.zip › outputONCOCNV copy 2/X1602.1.profile.png]

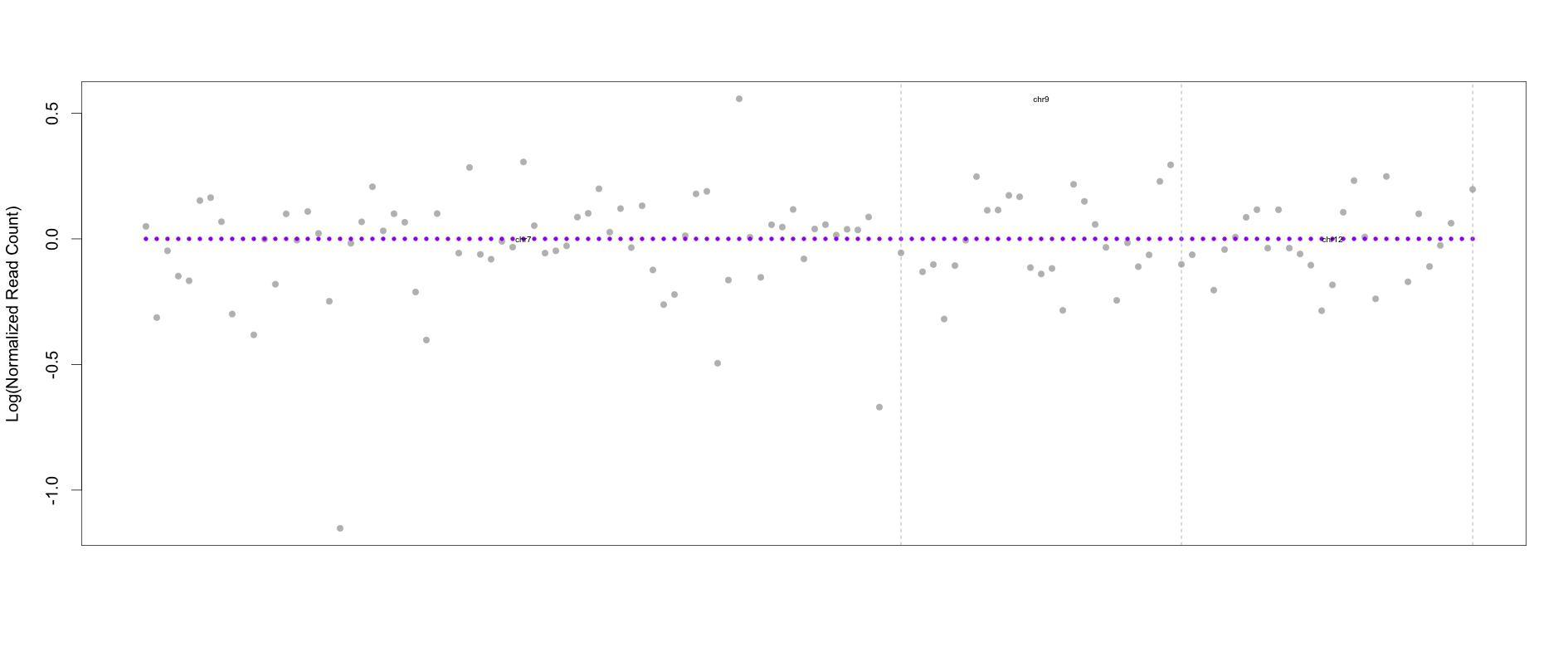

Supplement: Additional file 6 — Archive with ONCOCNV results. Set of files (png plots and txt files) in zip archive that were provided by ONCOCNV after specificity and sensitivity testing. (ZIP 5007 kb) [file 12859_2016_1272_MOESM6_ESM.zip › outputONCOCNV copy 2/X1642.1.1.profile.png]

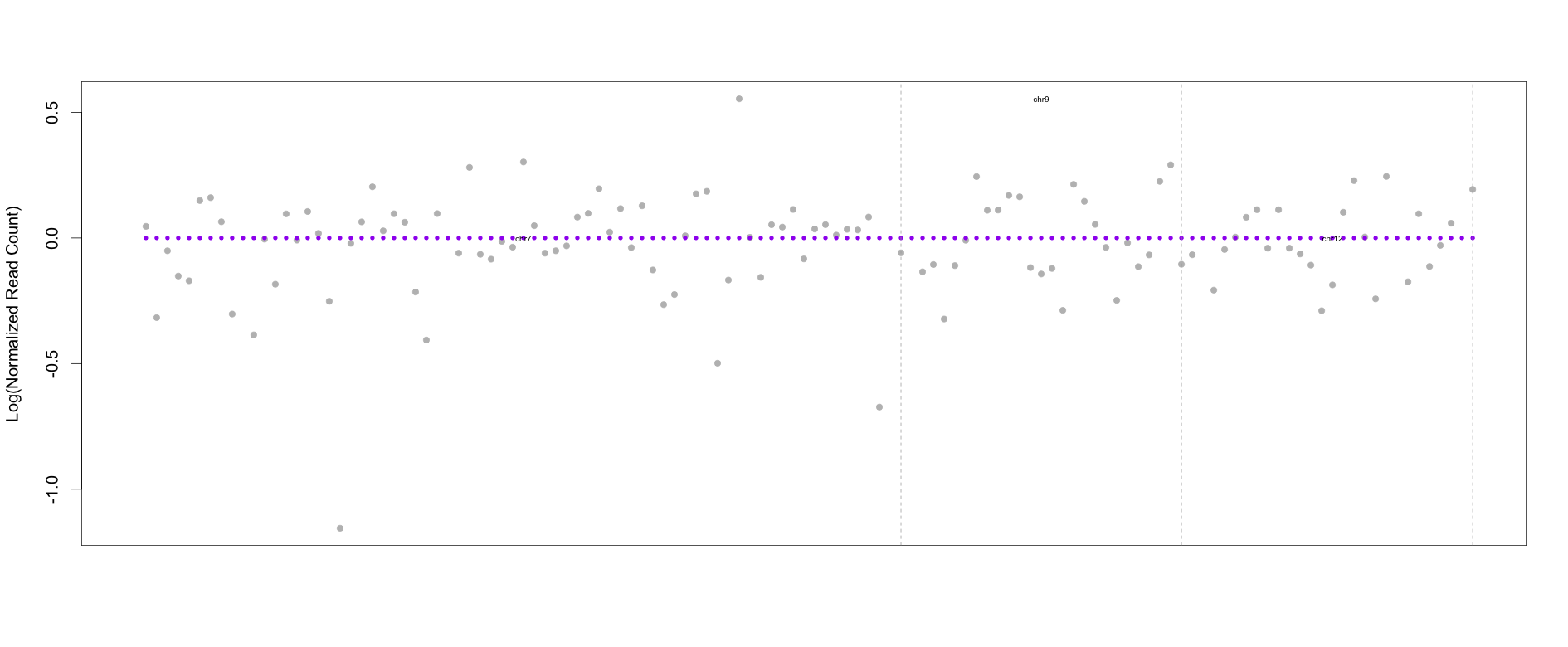

Supplement: Additional file 6 — Archive with ONCOCNV results. Set of files (png plots and txt files) in zip archive that were provided by ONCOCNV after specificity and sensitivity testing. (ZIP 5007 kb) [file 12859_2016_1272_MOESM6_ESM.zip › outputONCOCNV copy 2/X1642.1.profile.png]
