# Supplementary material for: A statistical approach to detection of copy number variations in PCR-enriched targeted sequencing data
Source: BMC Bioinformatics. 2016 Oct 22;17:429. doi: 10.1186/s12859-016-1272-6 (PMC5075217; doi:10.1186/s12859-016-1272-6)
Supplement: Additional file 5 — Archive with cn.mops results. Zip archive with large pdf file (R markdown) with the results of cn.mops (plots) and short summary of calls. (ZIP 1771 kb) [file 12859_2016_1272_MOESM5_ESM.zip › Additional file 5.pdf]

cn.mops

german

17 December 2015

```r
library(cn.mops)
```

```
## Warning: package 'cn.mops' was built under R version 3.2.3
```

```
## Loading required package: BiocGenerics
```

```
## Loading required package: parallel
```

```
##
## Attaching package: 'BiocGenerics'
```

```
## The following objects are masked from 'package:parallel':
##
##     clusterApply, clusterApplyLB, clusterCall, clusterEvalQ,
##     clusterExport, clusterMap, parApply, parCapply, parLapply,
##     parLapplyLB, parRapply, parSapply, parSapplyLB
```

```
## The following objects are masked from 'package:stats':
##
##     IQR, mad, xtabs
```

```
## The following objects are masked from 'package:base':
##
##     anyDuplicated, append, as.data.frame, as.vector, cbind,
##     colnames, do.call, duplicated, eval, evalq, Filter, Find, get,
##     grep, grepl, intersect, is.unsorted, lapply, lengths, Map,
##     mapply, match, mget, order, paste, pmax, pmax.int, pmin,
##     pmin.int, Position, rank, rbind, Reduce, rownames, sapply,
##     setdiff, sort, table, tapply, union, unique, unlist, unsplit
```

```
## Loading required package: Biobase
```

```
## Welcome to Bioconductor
##
##     Vignettes contain introductory material; view with
##     'browseVignettes()'. To cite Bioconductor, see
##     'citation("Biobase")', and for packages 'citation("pkgname")'.
```

```
## Loading required package: IRanges
```

```
## Warning: package 'IRanges' was built under R version 3.2.3
```

```
## Loading required package: S4Vectors
```

```
## Warning: package 'S4Vectors' was built under R version 3.2.3

## Loading required package: stats4

## Loading required package: GenomicRanges

## Warning: package 'GenomicRanges' was built under R version 3.2.3

## Loading required package: GenomeInfoDb

## Warning: package 'GenomeInfoDb' was built under R version 3.2.3

##
## Attaching package: 'cn.mops'

## The following object is masked from 'package:S4Vectors':
##
##     params
```

```
files <- list.files(path="/Users/gdemidov/Downloads/doc", pattern="*.xls", full.names=T, recursive=FALSE

for (i in seq(1:length(files))) {
  print(files[i])
readCountMatrix <- read.table(files[i],header = T,sep="\t")

bamDataRanges <- readCountMatrix[,c(-1,-2,-ncol(readCountMatrix))]

head(bamDataRanges)
resCNMOPS <- cn.mops(as.matrix(bamDataRanges) )
print("")
print(files[i])
print("")
for (j in 1:ncol(bamDataRanges)) {
  segplot(resCNMOPS,sampleIdx=j)
}
if (length(cnvs(resCNMOPS)) > 0) {print(calcIntegerCopyNumbers(resCNMOPS))}
}
```

```
## [1] "/Users/gdemidov/Downloads/doc/Merged_new_panel_05_qc.xls"

## Normalizing...

## Starting local modeling, please be patient...

## Reference sequence:  undef

## Starting segmentation algorithm...

## Using "fastseg" for segmentation.
```

```
## [1] ""
## [1] "/Users/gdemidov/Downloads/doc/Merged_new_panel_05_qc.xls"
## [1] ""
```

```
## Segplot might not work because of special characters in the sample names. Use only A-Z,a-z and 0-9!
##  There is a hidden function cn.mops:::.replaceNames that replaces the names in the "CNVDetectionResul
```

```
## Segplot might not work because of special characters in the sample names. Use only A-Z,a-z and 0-9!
##  There is a hidden function cn.mops:::.replaceNames that replaces the names in the "CNVDetectionResul
```

_11_07_23_43_Sequoia_SN1.59.11_04_15_Neoscreen_v1_Auto_Sequoia_SN1.59.1

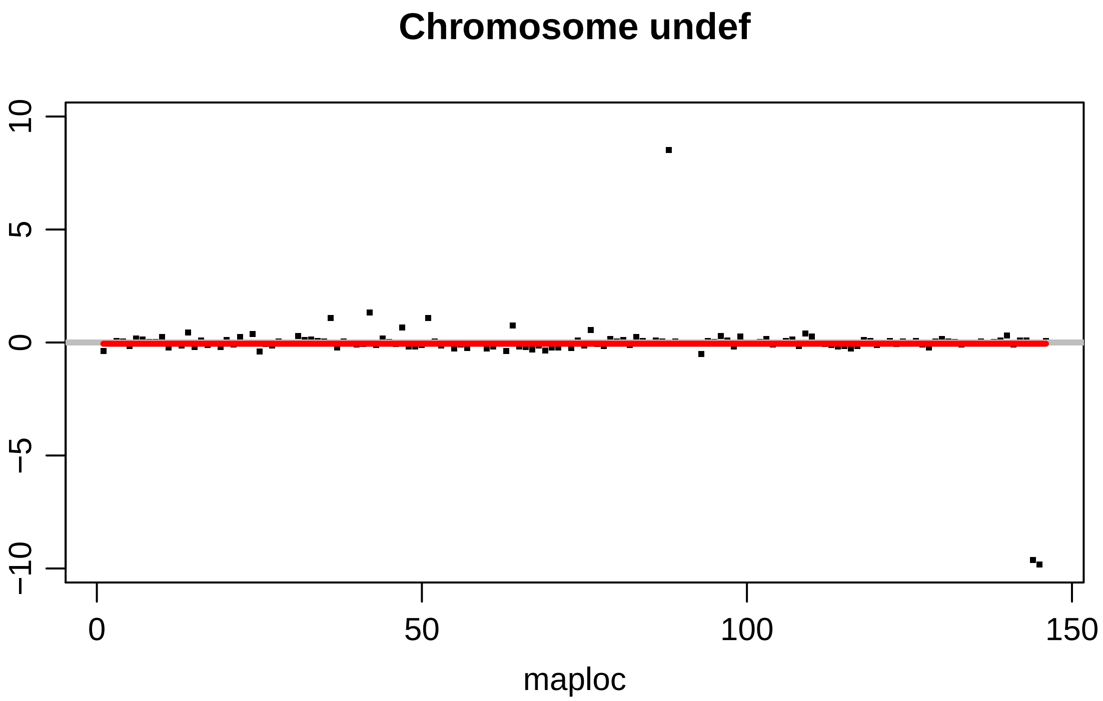


```
## Segplot might not work because of special characters in the sample names. Use only A-Z,a-z and 0-9!
##  There is a hidden function cn.mops:::.replaceNames that replaces the names in the "CNVDetectionResul
```

_11_07_23_43_Sequoia_SN1.59.11_04_15_Neoscreen_v1_Auto_Sequoia_SN1.59.1

Chromosome undef

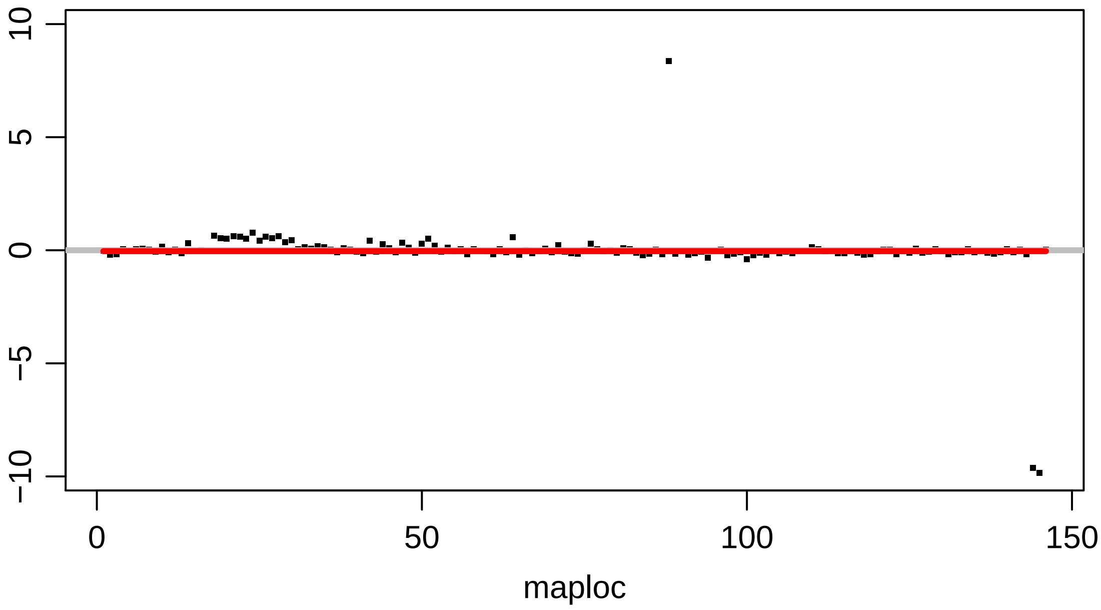


```
## Segplot might not work because of special characters in the sample names. Use only A-Z,a-z and 0-9!
##  There is a hidden function cn.mops:::.replaceNames that replaces the names in the "CNVDetectionResul
```

_11_07_23_43_Sequoia_SN1.59.11_04_15_Neoscreen_v1_Auto_Sequoia_SN1.59.1

Chromosome undef

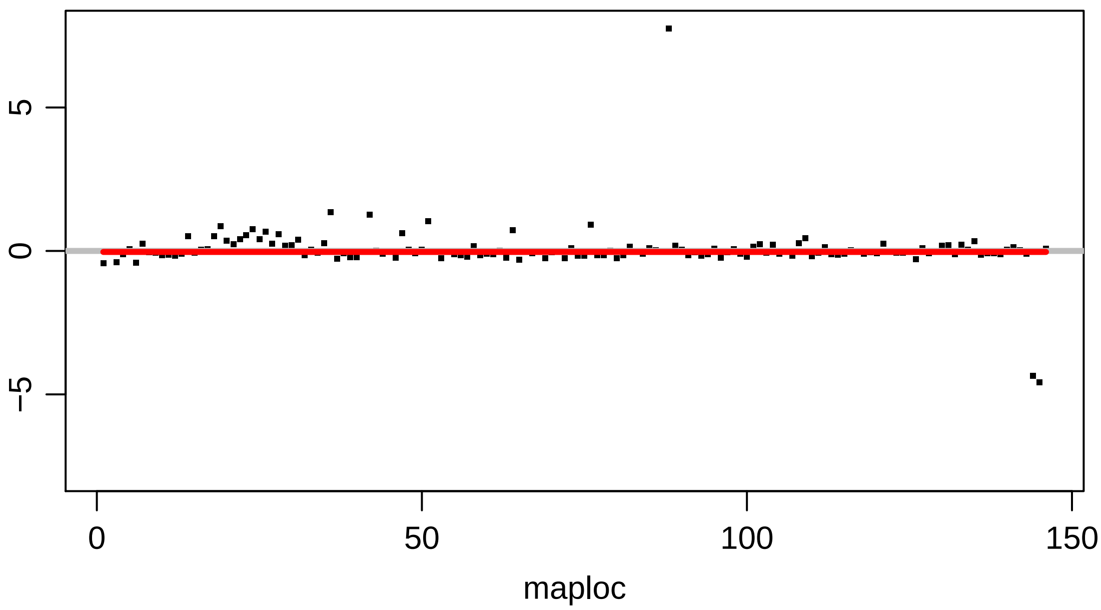

```
## Segplot might not work because of special characters in the sample names. Use only A-Z,a-z and 0-9!
##  There is a hidden function cn.mops:::.replaceNames that replaces the names in the "CNVDetectionResul
```

_11_07_23_43_Sequoia_SN1.59.11_04_15_Neoscreen_v1_Auto_Sequoia_SN1.59.1

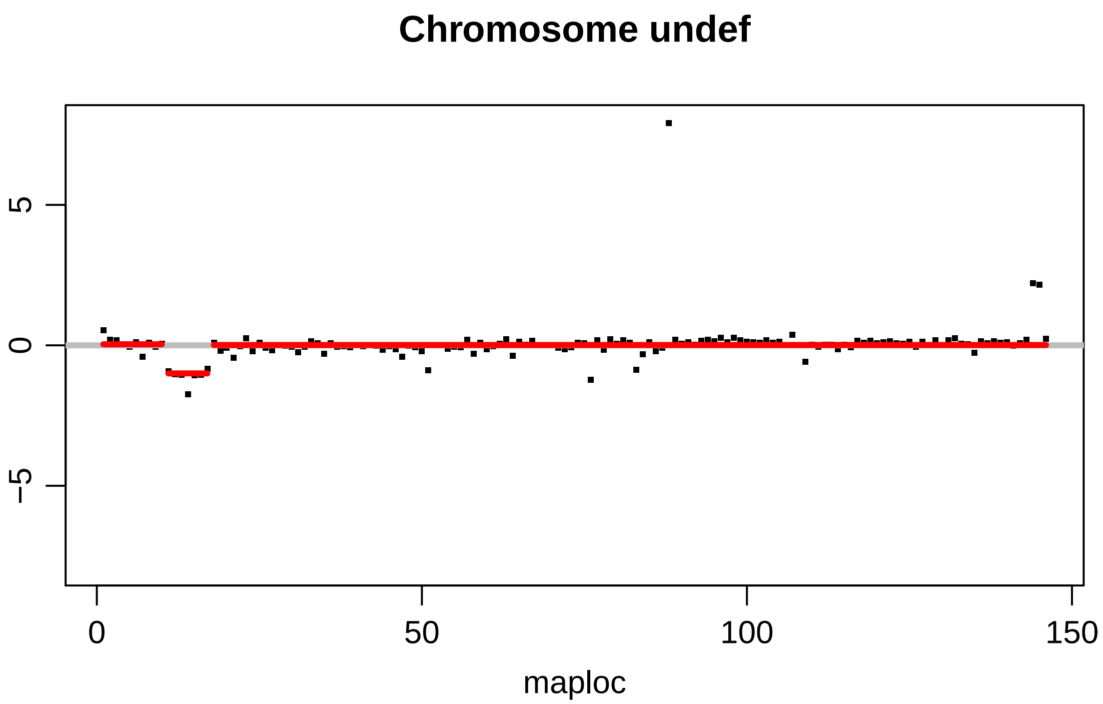


```
## Segplot might not work because of special characters in the sample names. Use only A-Z,a-z and 0-9!
##  There is a hidden function cn.mops:::.replaceNames that replaces the names in the "CNVDetectionResul
```

_11_07_23_43_Sequoia_SN1.59.11_04_15_Neoscreen_v1_Auto_Sequoia_SN1.59.1

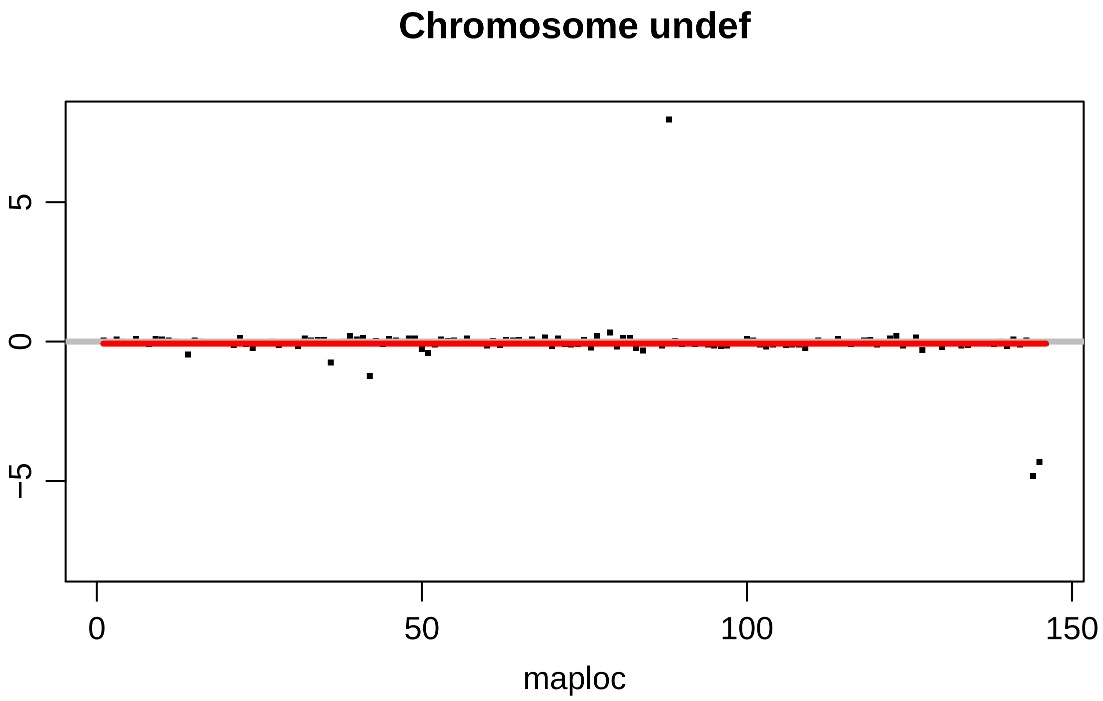


```
## Segplot might not work because of special characters in the sample names. Use only A-Z,a-z and 0-9!
##  There is a hidden function cn.mops:::.replaceNames that replaces the names in the "CNVDetectionResul
```

_11_07_23_43_Sequoia_SN1.59.11_04_15_Neoscreen_v1_Auto_Sequoia_SN1.59.1

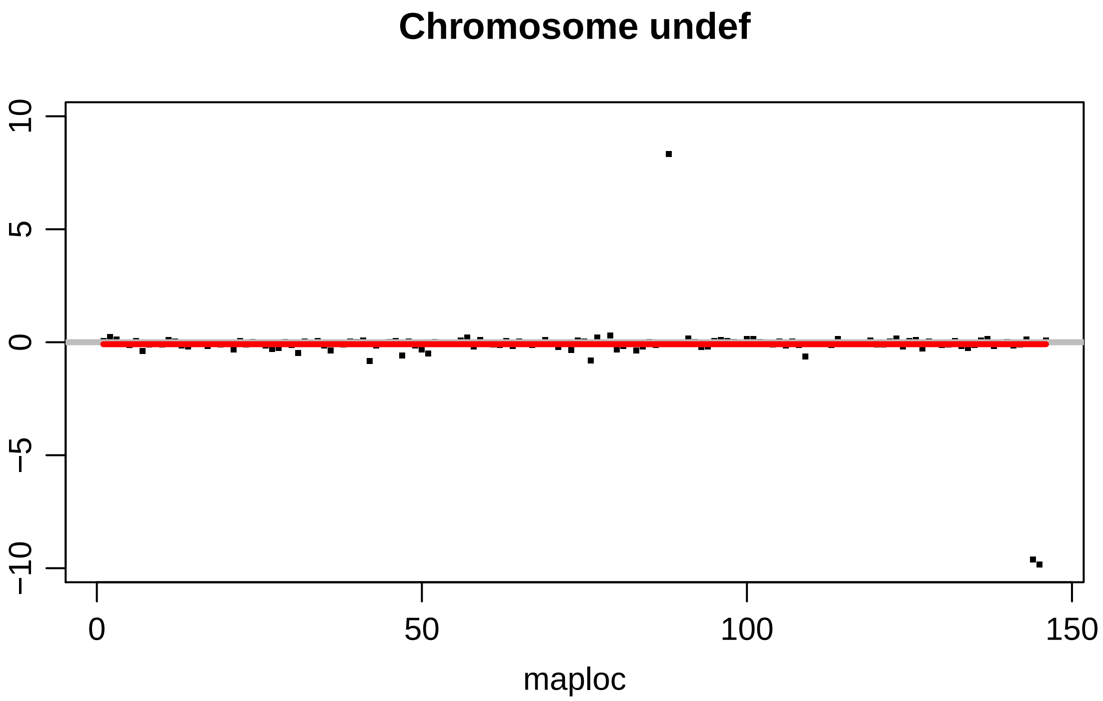

```
## Segplot might not work because of special characters in the sample names. Use only A-Z,a-z and 0-9!
##  There is a hidden function cn.mops:::.replaceNames that replaces the names in the "CNVDetectionResul
```

_11_07_23_43_Sequoia_SN1.59.11_04_15_Neoscreen_v1_Auto_Sequoia_SN1.59.1

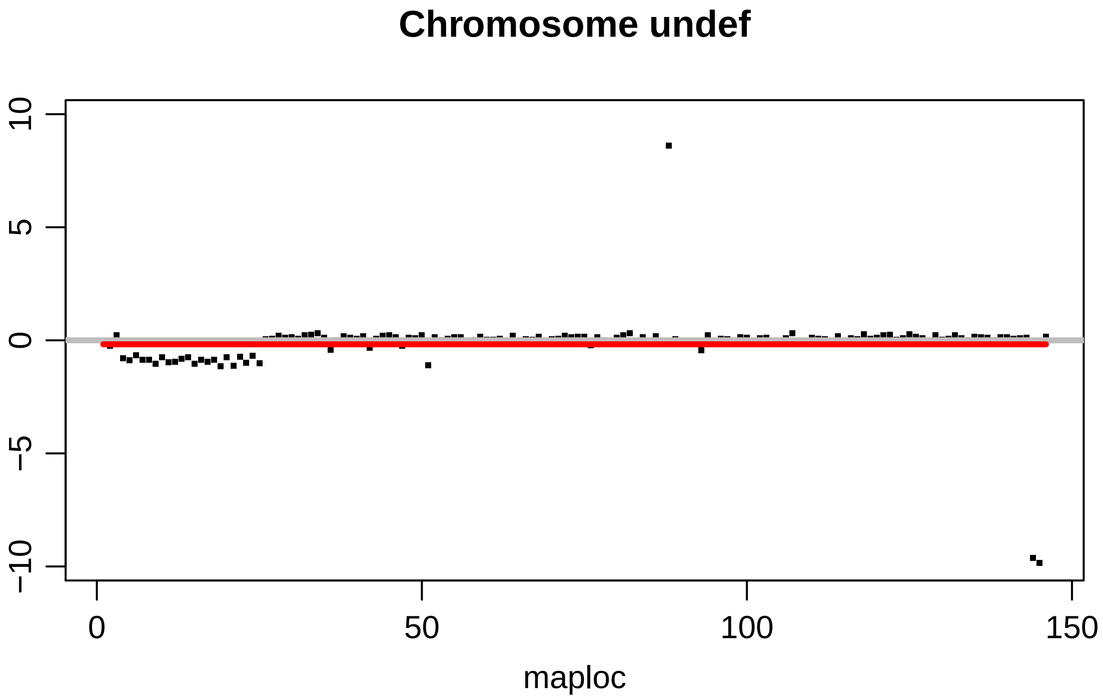


```
## Segplot might not work because of special characters in the sample names. Use only A-Z,a-z and 0-9!
##  There is a hidden function cn.mops:::.replaceNames that replaces the names in the "CNVDetectionResul
```

_11_07_23_43_Sequoia_SN1.59.11_04_15_Neoscreen_v1_Auto_Sequoia_SN1.59.1

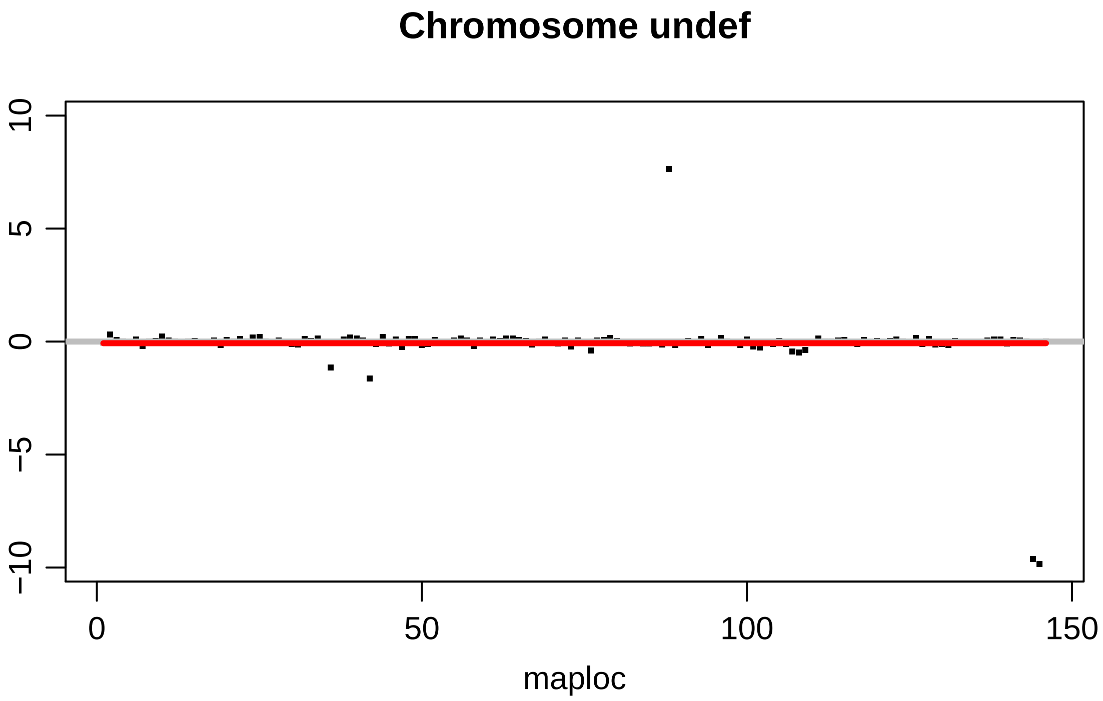


```
## Segplot might not work because of special characters in the sample names. Use only A-Z,a-z and 0-9!
##  There is a hidden function cn.mops:::.replaceNames that replaces the names in the "CNVDetectionResul
```

_11_07_23_43_Sequoia_SN1.59.11_04_15_Neoscreen_v1_Auto_Sequoia_SN1.59.1

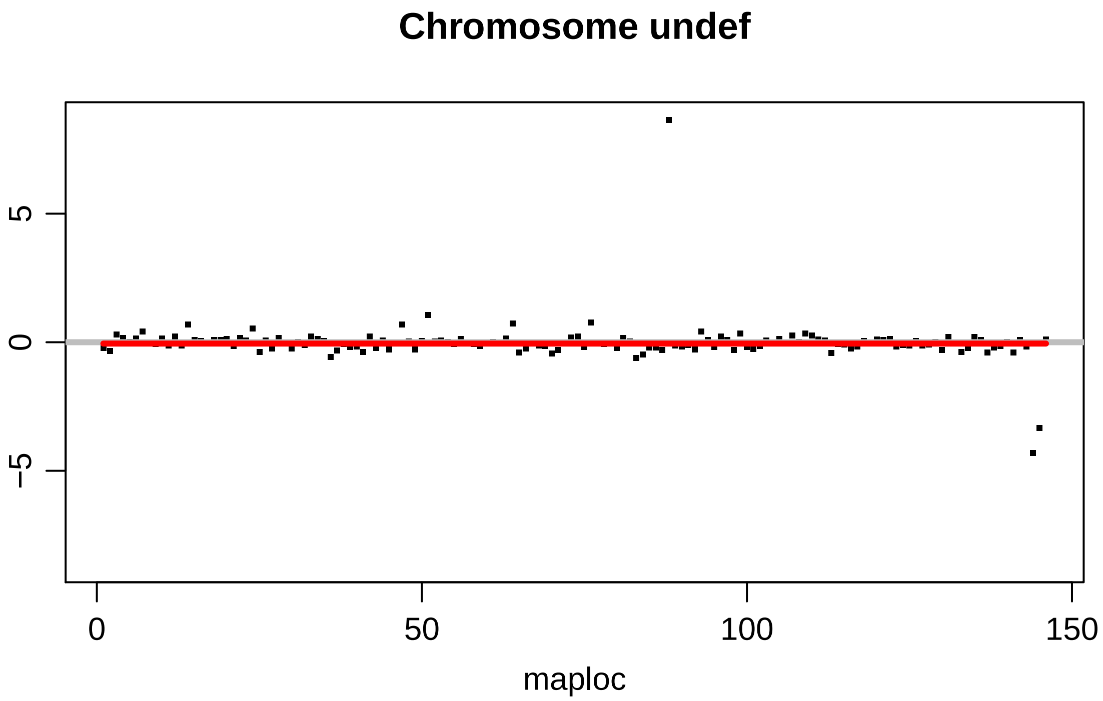

```
## Segplot might not work because of special characters in the sample names. Use only A-Z,a-z and 0-9!
##  There is a hidden function cn.mops:::.replaceNames that replaces the names in the "CNVDetectionResul
```

_11_07_23_43_Sequoia_SN1.59.11_04_15_Neoscreen_v1_Auto_Sequoia_SN1.59.1

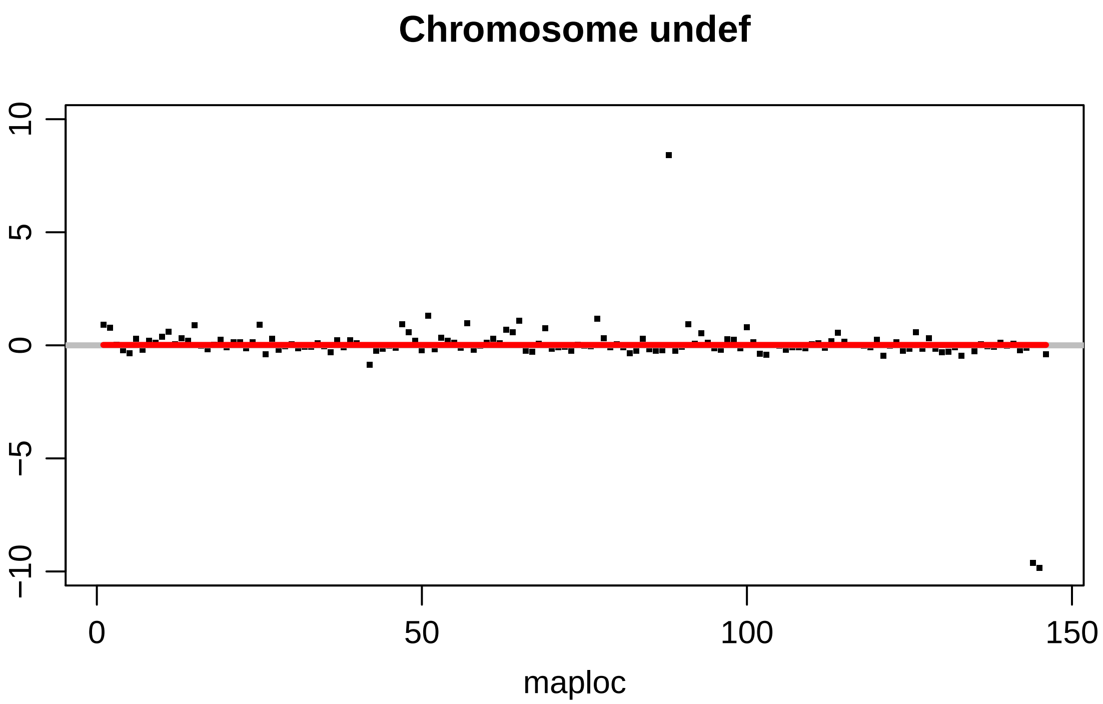


```
## Segplot might not work because of special characters in the sample names. Use only A-Z,a-z and 0-9!
##  There is a hidden function cn.mops:::.replaceNames that replaces the names in the "CNVDetectionResul
```

_11_07_23_43_Sequoia_SN1.59.11_04_15_Neoscreen_v1_Auto_Sequoia_SN1.59.1

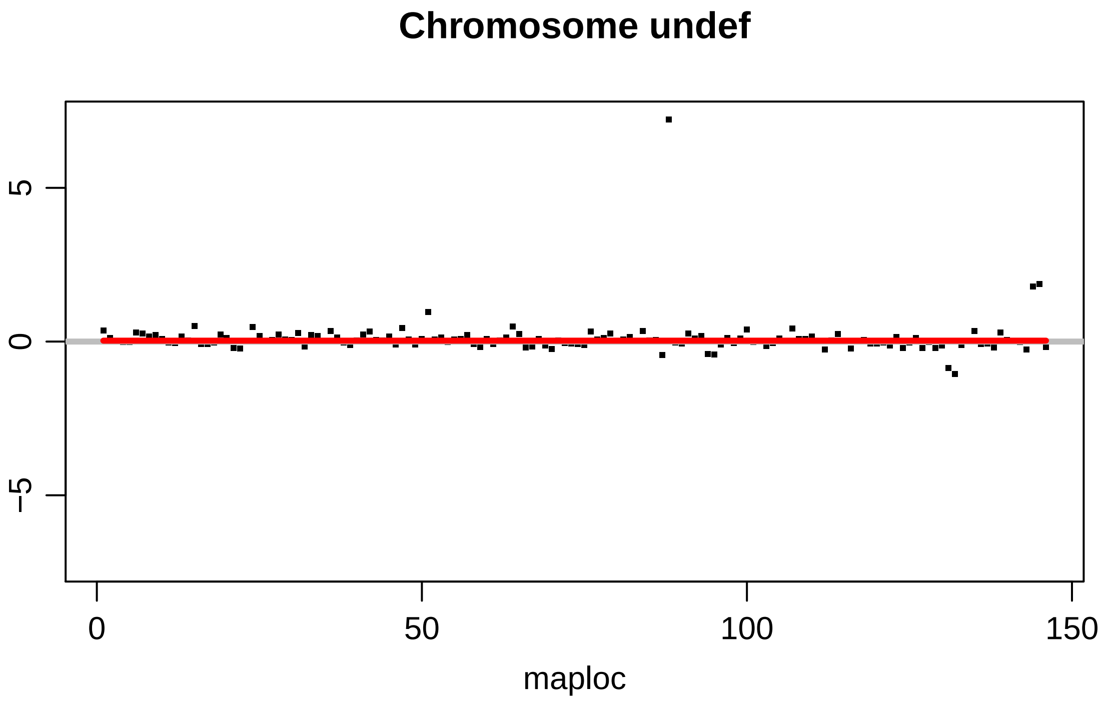


```
## Segplot might not work because of special characters in the sample names. Use only A-Z,a-z and 0-9!
##  There is a hidden function cn.mops:::.replaceNames that replaces the names in the "CNVDetectionResul
```

_11_07_23_43_Sequoia_SN1.59.11_04_15_Neoscreen_v1_Auto_Sequoia_SN1.59.1

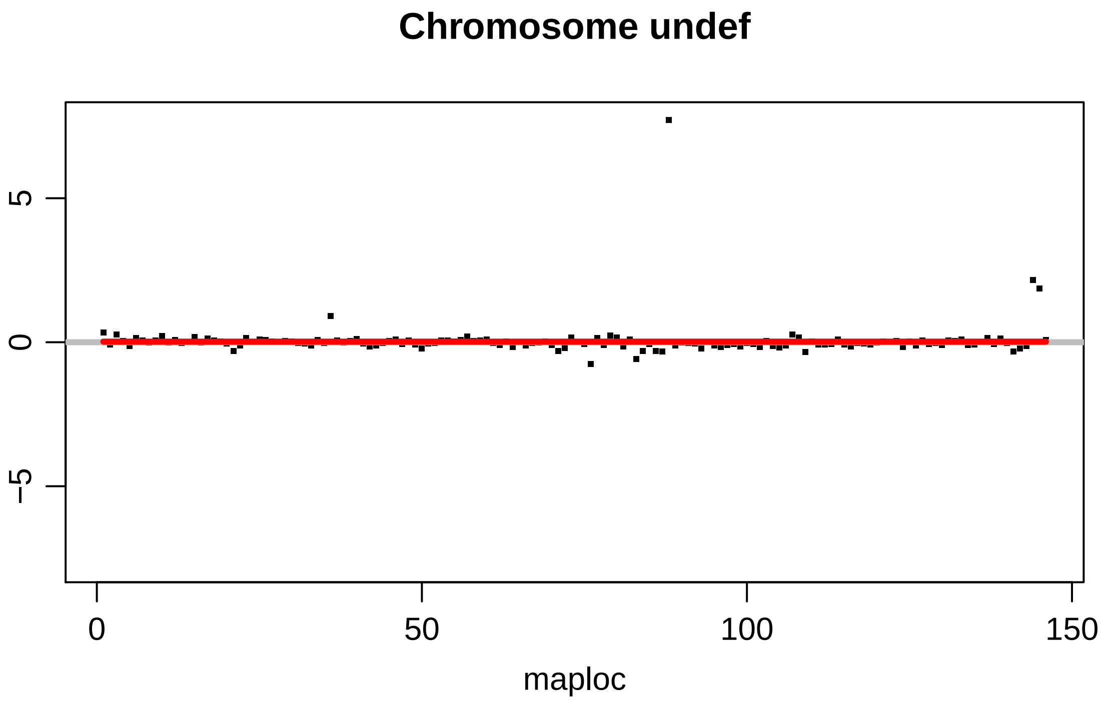

```
## Segplot might not work because of special characters in the sample names. Use only A-Z,a-z and 0-9!
##  There is a hidden function cn.mops:::.replaceNames that replaces the names in the "CNVDetectionResul
```

_11_07_23_43_Sequoia_SN1.59.11_04_15_Neoscreen_v1_Auto_Sequoia_SN1.59.1

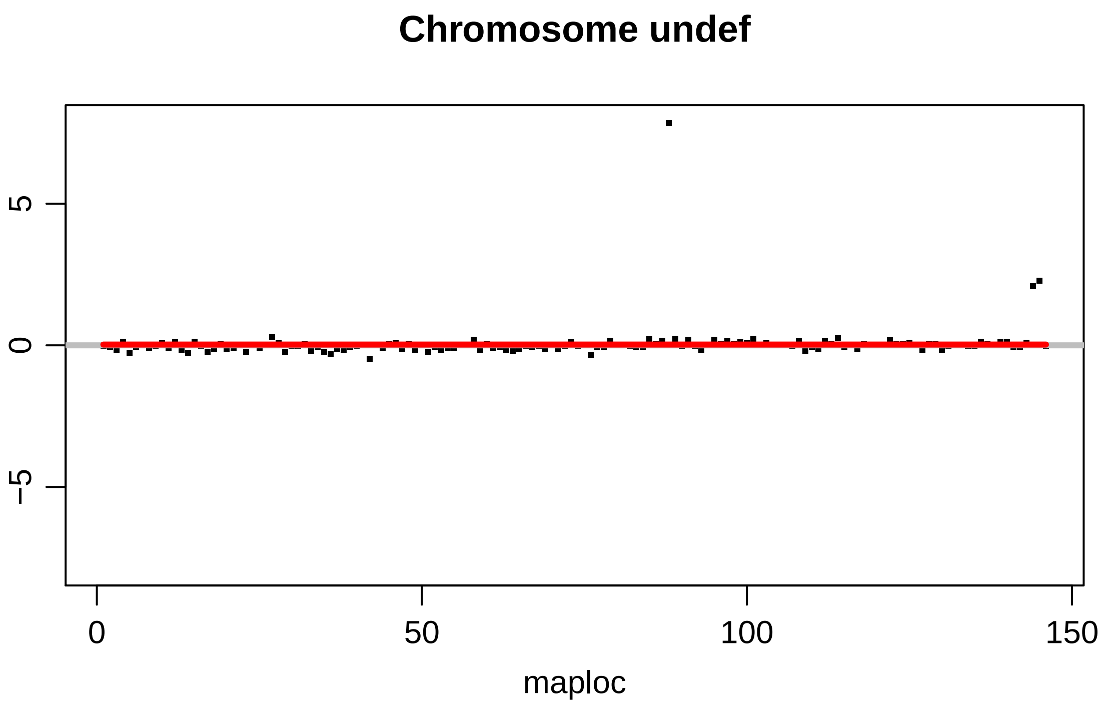


```
## Segplot might not work because of special characters in the sample names. Use only A-Z,a-z and 0-9!
##  There is a hidden function cn.mops:::.replaceNames that replaces the names in the "CNVDetectionResul
```

_11_07_23_43_Sequoia_SN1.59.11_04_15_Neoscreen_v1_Auto_Sequoia_SN1.59.1

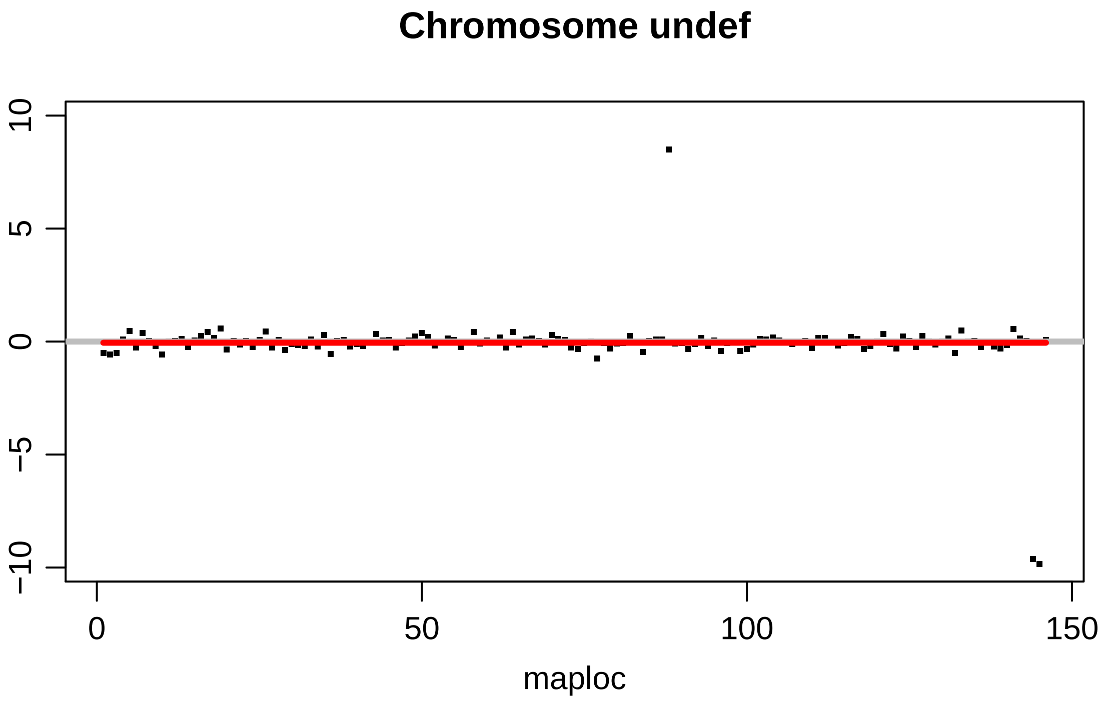


```
## Segplot might not work because of special characters in the sample names. Use only A-Z,a-z and 0-9!
##  There is a hidden function cn.mops:::.replaceNames that replaces the names in the "CNVDetectionResu
```

_11_07_23_43_Sequoia_SN1.59.11_04_15_Neoscreen_v1_Auto_Sequoia_SN1.59.1

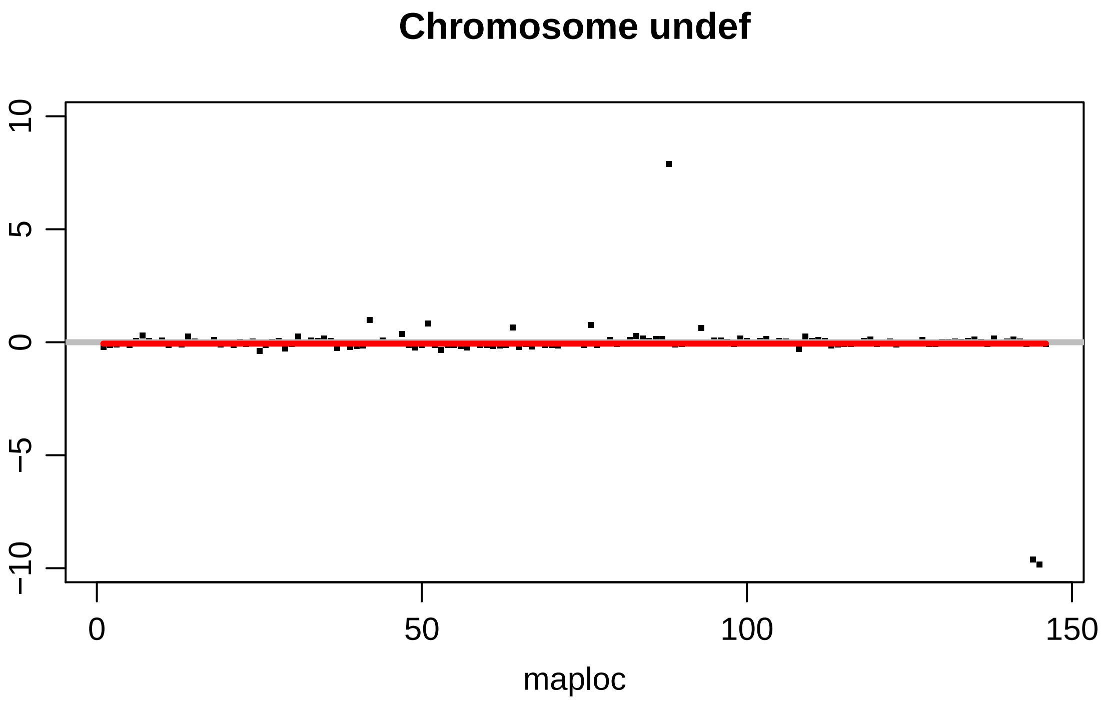

```
## Segplot might not work because of special characters in the sample names. Use only A-Z,a-z and 0-9!
##  There is a hidden function cn.mops:::.replaceNames that replaces the names in the "CNVDetectionResul
```

_11_07_23_43_Sequoia_SN1.59.11_04_15_Neoscreen_v1_Auto_Sequoia_SN1.59.1

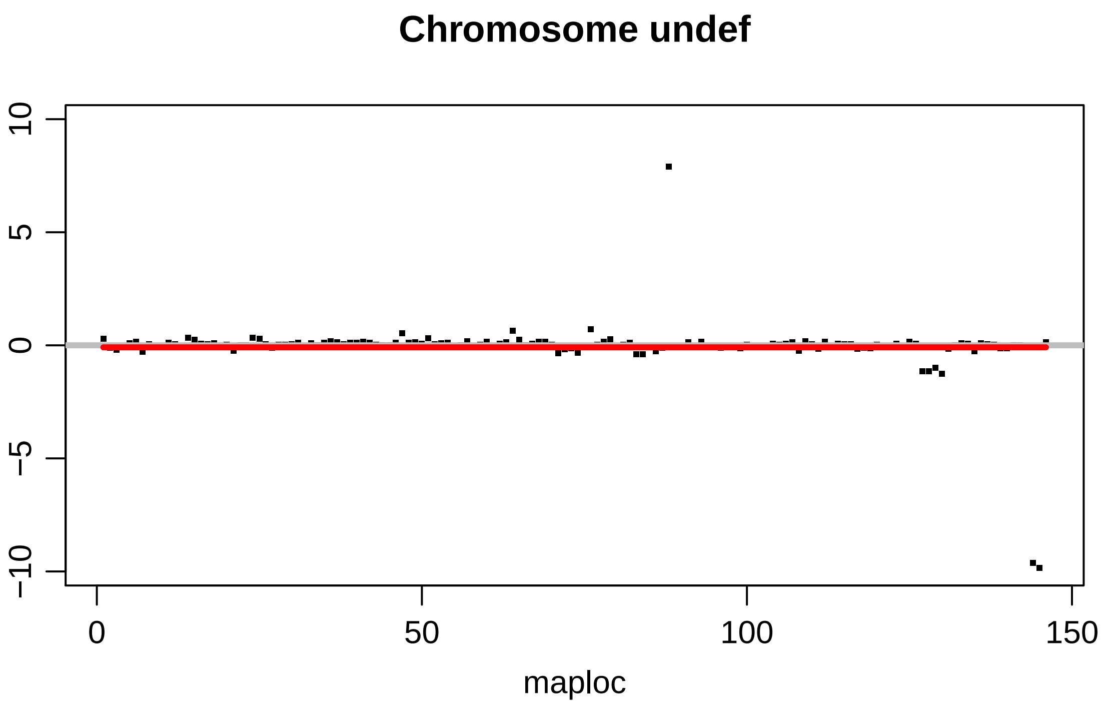


```
## Segplot might not work because of special characters in the sample names. Use only A-Z,a-z and 0-9!
##  There is a hidden function cn.mops:::.replaceNames that replaces the names in the "CNVDetectionResul
```

_11_07_23_43_Sequoia_SN1.59.11_04_15_Neoscreen_v1_Auto_Sequoia_SN1.59.1

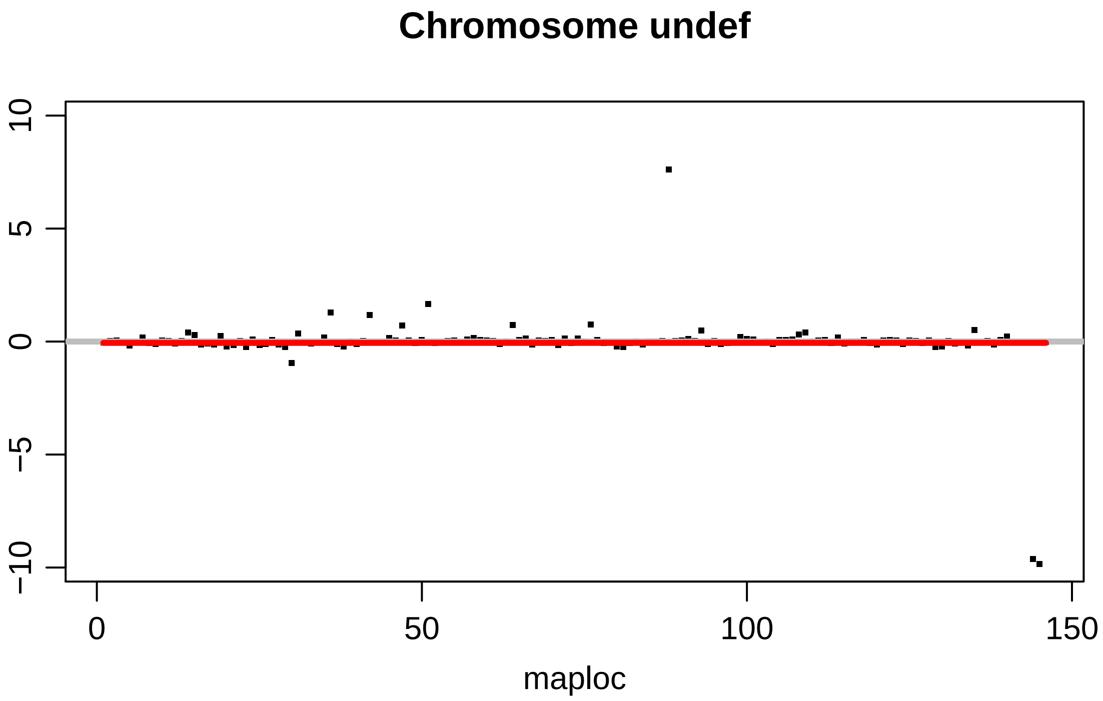


```
## Segplot might not work because of special characters in the sample names. Use only A-Z,a-z and 0-9!
##  There is a hidden function cn.mops:::.replaceNames that replaces the names in the "CNVDetectionResul
```

_11_07_23_43_Sequoia_SN1.59.11_04_15_Neoscreen_v1_Auto_Sequoia_SN1.59.1

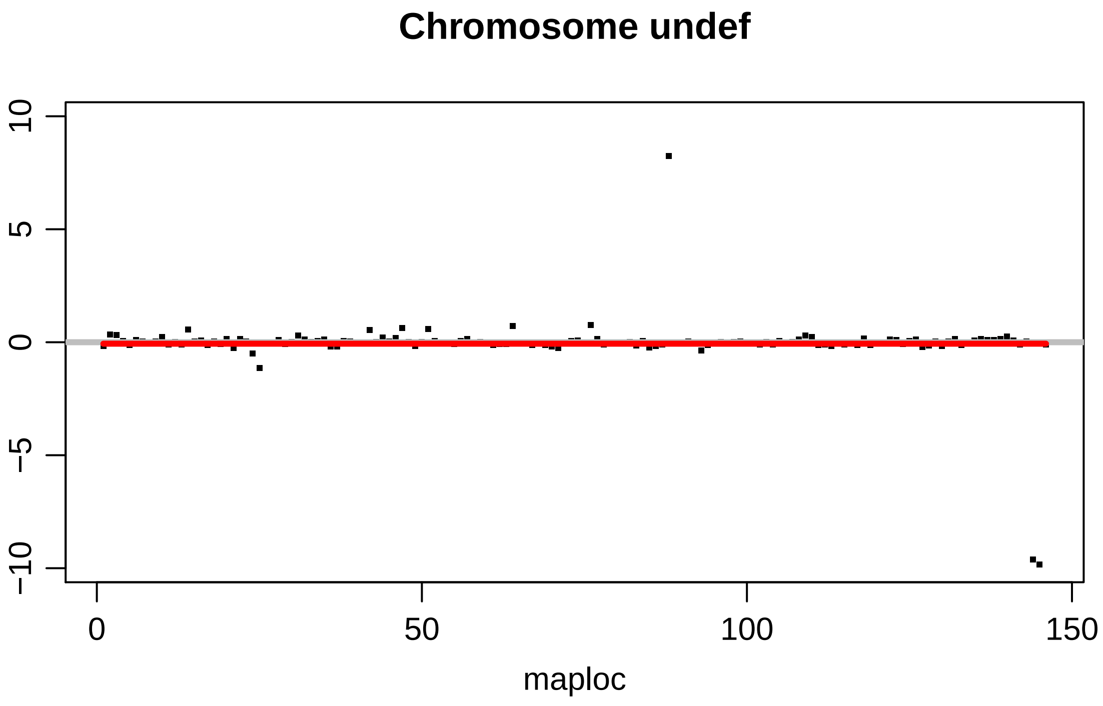

```
## Segplot might not work because of special characters in the sample names. Use only A-Z,a-z and 0-9!
##  There is a hidden function cn.mops:::.replaceNames that replaces the names in the "CNVDetectionResul
```

_11_07_23_43_Sequoia_SN1.59.11_04_15_Neoscreen_v1_Auto_Sequoia_SN1.59.1

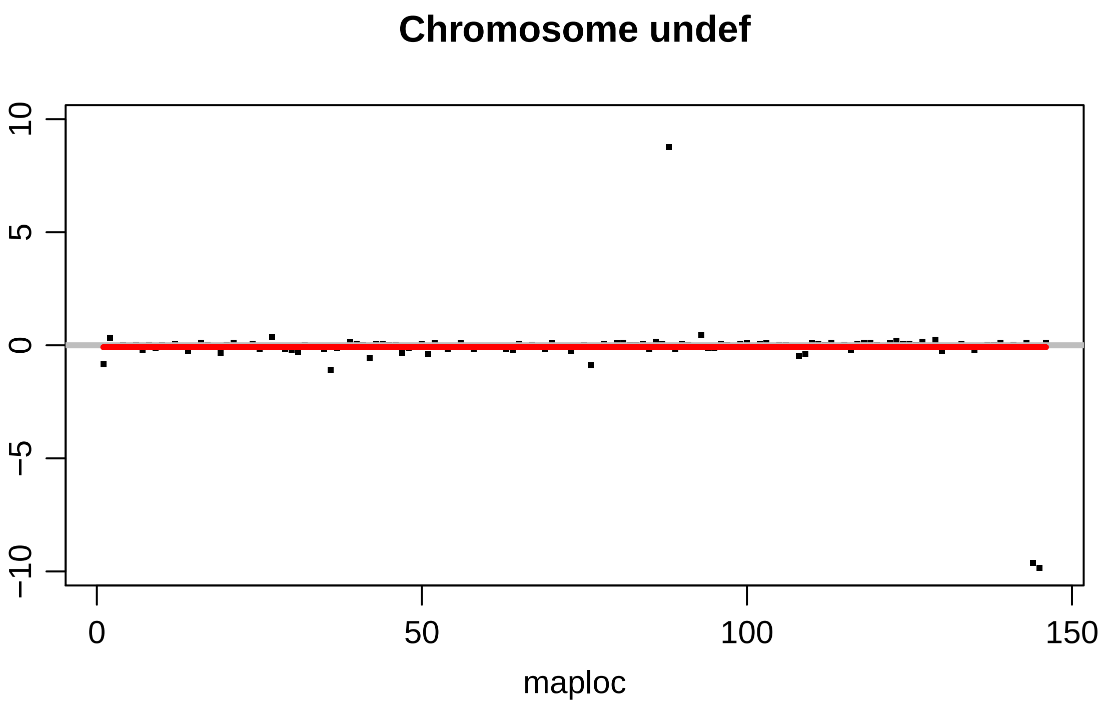


```
## Segplot might not work because of special characters in the sample names. Use only A-Z,a-z and 0-9!
##  There is a hidden function cn.mops:::.replaceNames that replaces the names in the "CNVDetectionResul
```

_11_07_23_43_Sequoia_SN1.59.11_04_15_Neoscreen_v1_Auto_Sequoia_SN1.59.1

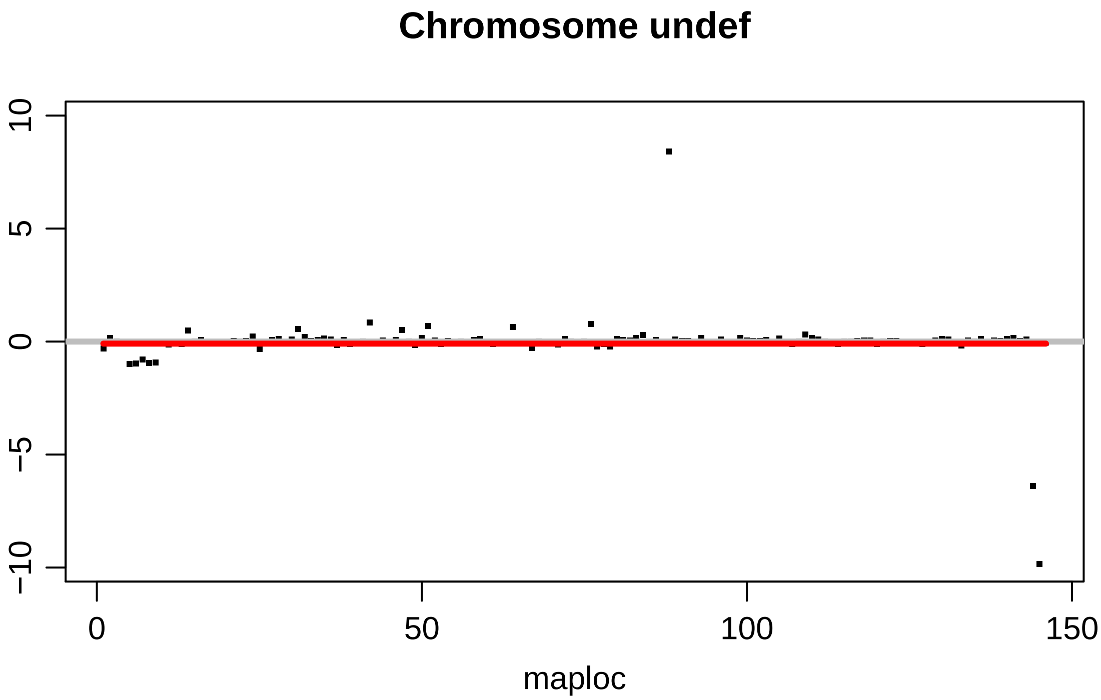


```
## Segplot might not work because of special characters in the sample names. Use only A-Z,a-z and 0-9!
##  There is a hidden function cn.mops:::.replaceNames that replaces the names in the "CNVDetectionResul
```

_11_07_23_43_Sequoia_SN1.59.11_04_15_Neoscreen_v1_Auto_Sequoia_SN1.59.1

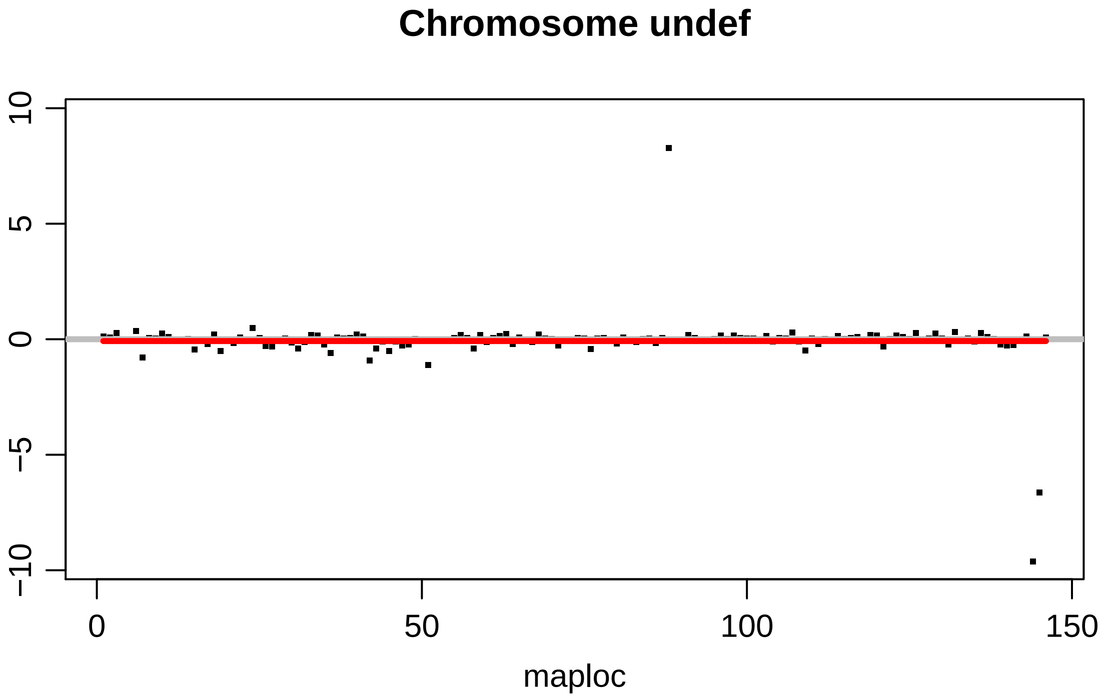

```
## Segplot might not work because of special characters in the sample names. Use only A-Z,a-z and 0-9!
##  There is a hidden function cn.mops:::.replaceNames that replaces the names in the "CNVDetectionResul
```

_11_07_23_43_Sequoia_SN1.59.11_04_15_Neoscreen_v1_Auto_Sequoia_SN1.59.1

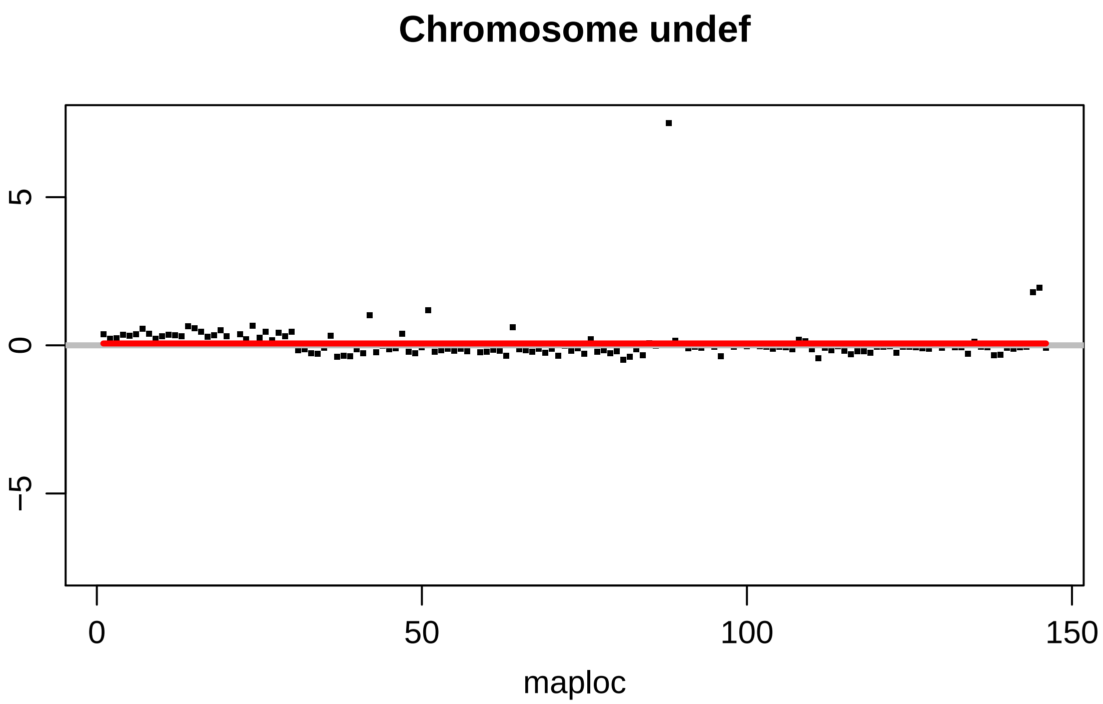


```
## Segplot might not work because of special characters in the sample names. Use only A-Z,a-z and 0-9!
##  There is a hidden function cn.mops:::.replaceNames that replaces the names in the "CNVDetectionResul
```

_11_07_23_43_Sequoia_SN1.59.11_04_15_Neoscreen_v1_Auto_Sequoia_SN1.59.1

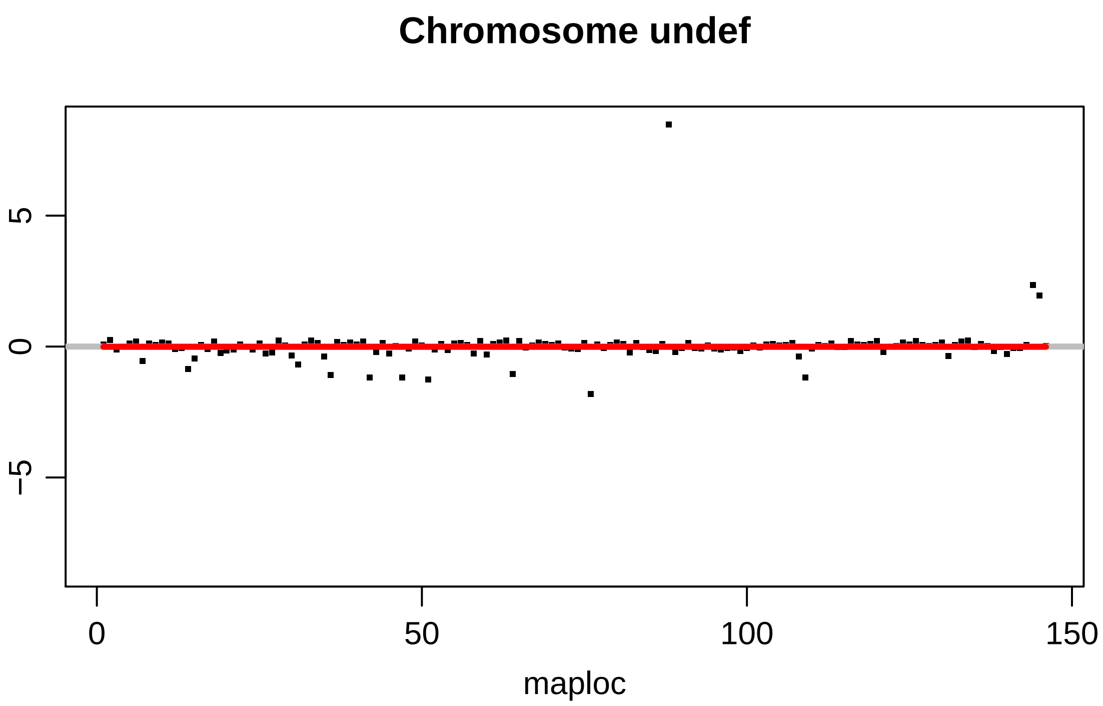


```
## Segplot might not work because of special characters in the sample names. Use only A-Z,a-z and 0-9!
##  There is a hidden function cn.mops:::.replaceNames that replaces the names in the "CNVDetectionResul
```

_11_07_23_43_Sequoia_SN1.59.11_04_15_Neoscreen_v1_Auto_Sequoia_SN1.59.1

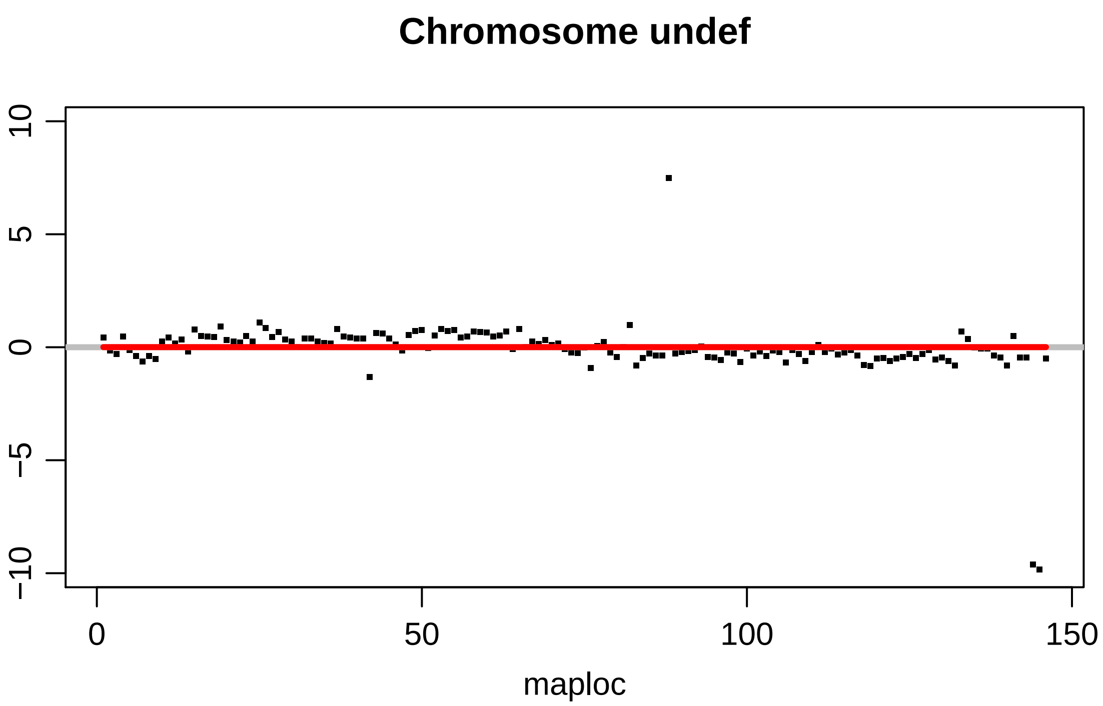

```
## Segplot might not work because of special characters in the sample names. Use only A-Z,a-z and 0-9!
##  There is a hidden function cn.mops:::.replaceNames that replaces the names in the "CNVDetectionResul
```

_11_07_23_43_Sequoia_SN1.59.11_04_15_Neoscreen_v1_Auto_Sequoia_SN1.59.1

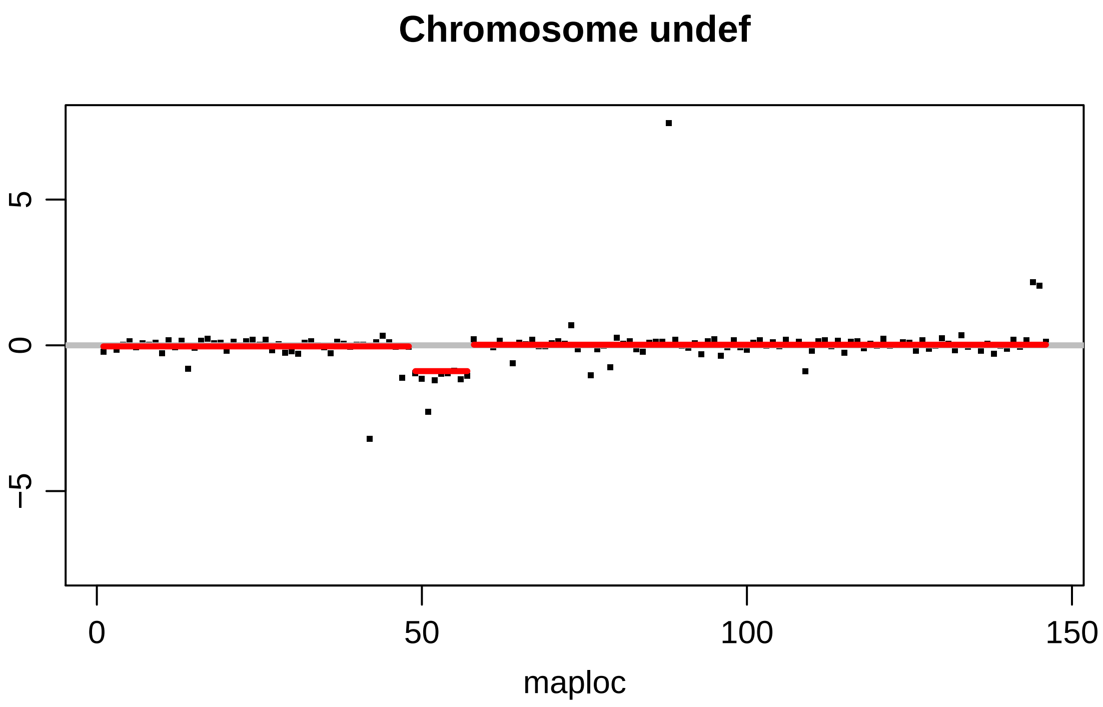


```
## Segplot might not work because of special characters in the sample names. Use only A-Z,a-z and 0-9!
##  There is a hidden function cn.mops:::.replaceNames that replaces the names in the "CNVDetectionResul
```

_11_07_23_43_Sequoia_SN1.59.11_04_15_Neoscreen_v1_Auto_Sequoia_SN1.59.1

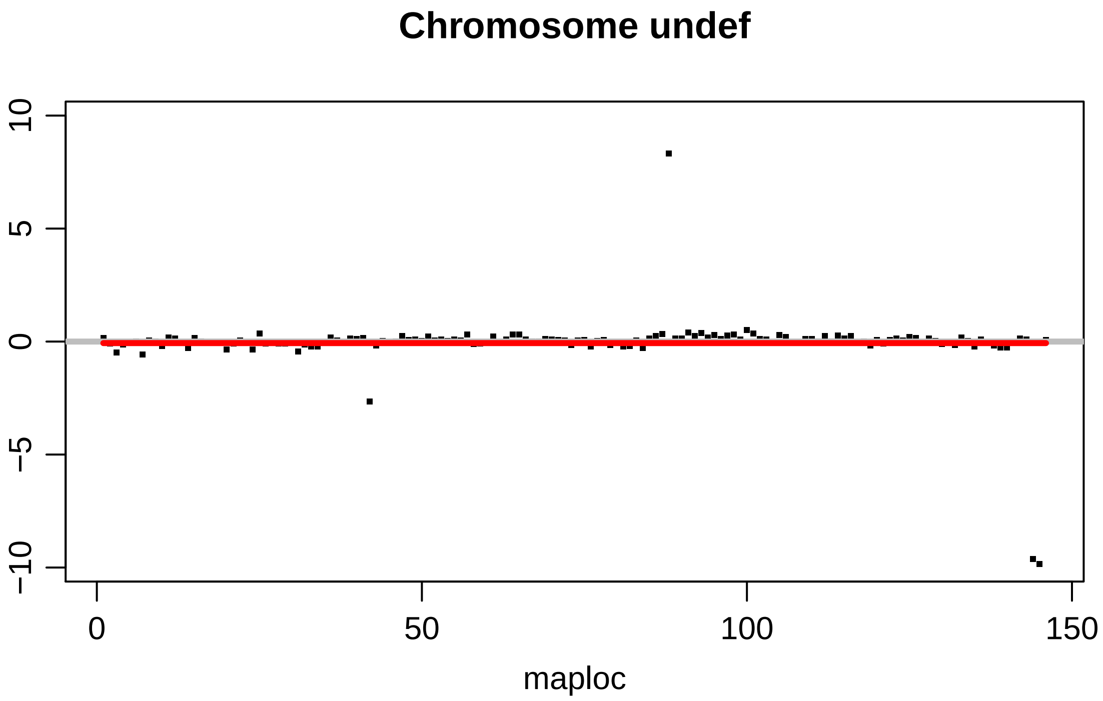


```
## Segplot might not work because of special characters in the sample names. Use only A-Z,a-z and 0-9!
##  There is a hidden function cn.mops:::.replaceNames that replaces the names in the "CNVDetectionResul
```

_11_07_23_43_Sequoia_SN1.59.11_04_15_Neoscreen_v1_Auto_Sequoia_SN1.59.1

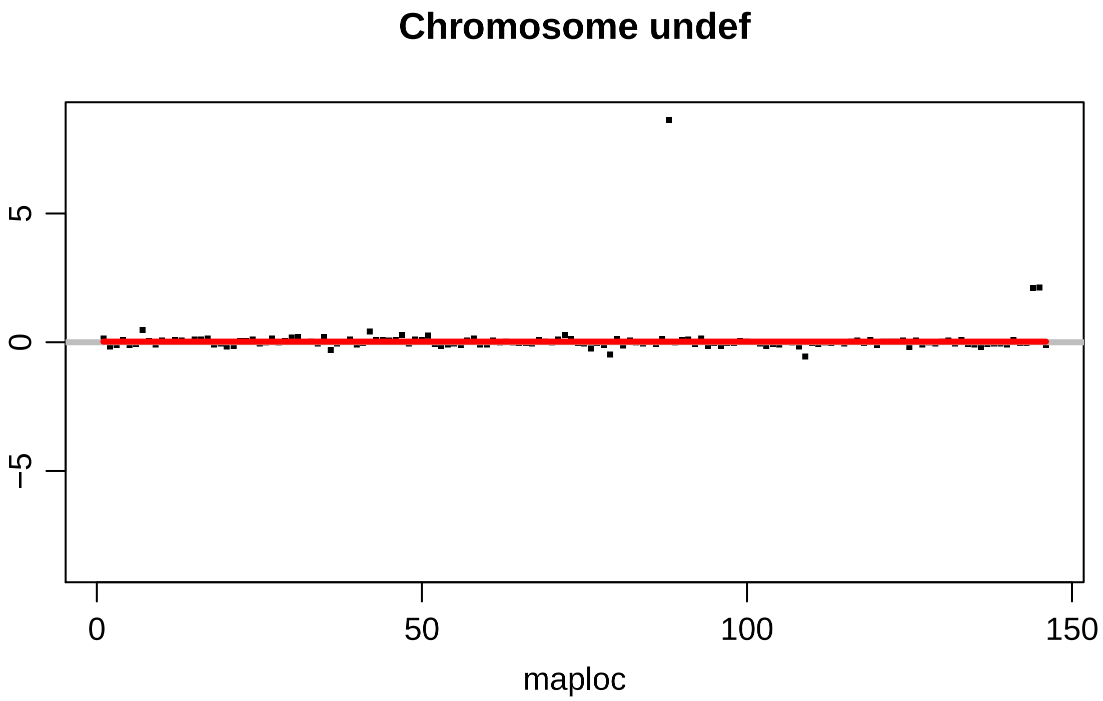

```
## Segplot might not work because of special characters in the sample names. Use only A-Z,a-z and 0-9!
##  There is a hidden function cn.mops:::.replaceNames that replaces the names in the "CNVDetectionResul
```

_11_07_23_43_Sequoia_SN1.59.11_04_15_Neoscreen_v1_Auto_Sequoia_SN1.59.1

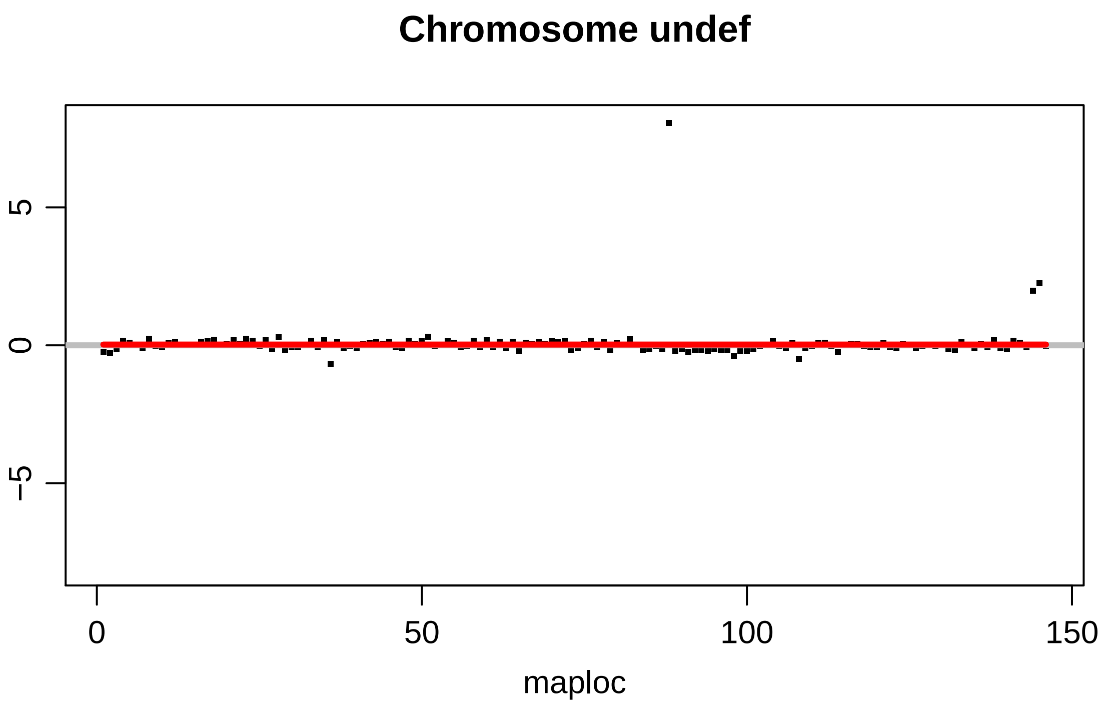


```
## Segplot might not work because of special characters in the sample names. Use only A-Z,a-z and 0-9!
##  There is a hidden function cn.mops:::.replaceNames that replaces the names in the "CNVDetectionResul
```

_11_07_23_43_Sequoia_SN1.59.11_04_15_Neoscreen_v1_Auto_Sequoia_SN1.59.1

Chromosome undef

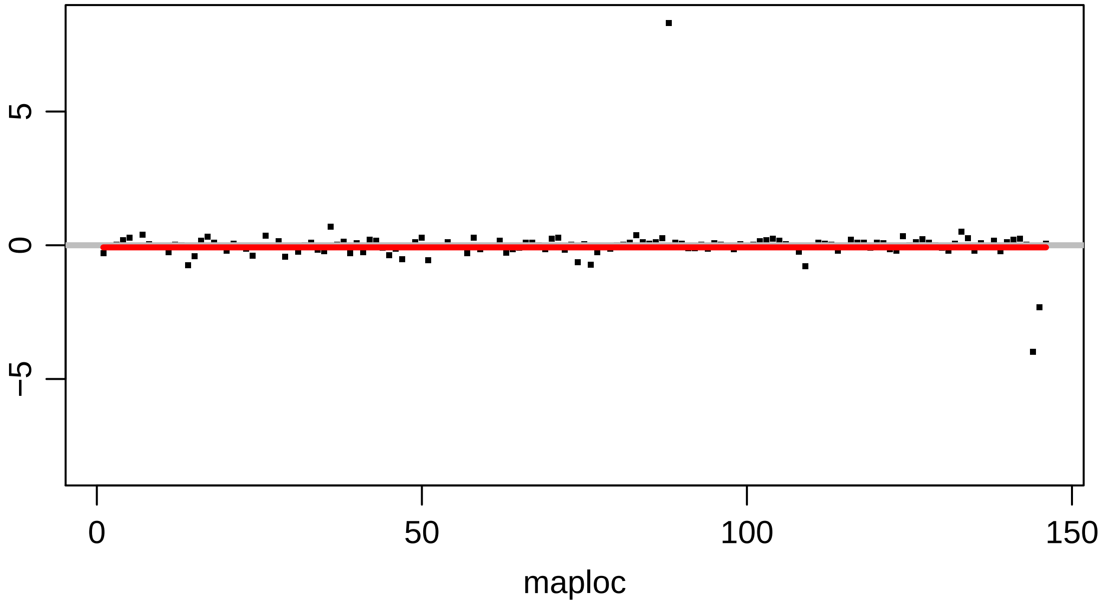


```
## Segplot might not work because of special characters in the sample names. Use only A-Z,a-z and 0-9!
##  There is a hidden function cn.mops:::.replaceNames that replaces the names in the "CNVDetectionResul
```

_11_07_23_43_Sequoia_SN1.59.11_04_15_Neoscreen_v1_Auto_Sequoia_SN1.59.1

Chromosome undef

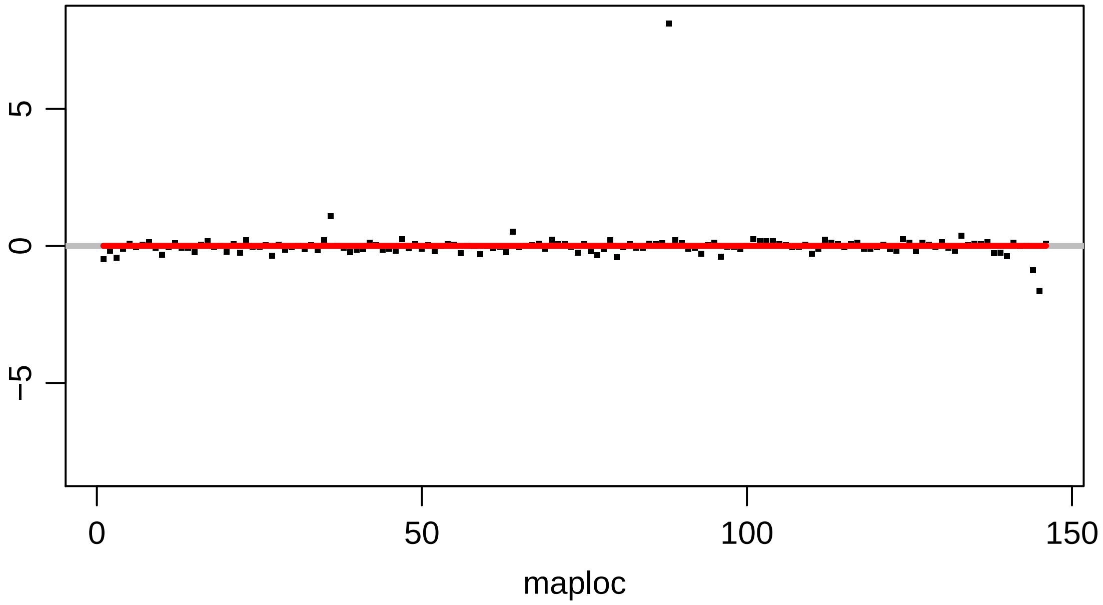

```
## Segplot might not work because of special characters in the sample names. Use only A-Z,a-z and 0-9!
##  There is a hidden function cn.mops:::.replaceNames that replaces the names in the "CNVDetectionResul[illegible]
```

_11_07_23_43_Sequoia_SN1.59.11_04_15_Neoscreen_v1_Auto_Sequoia_SN1.59.1

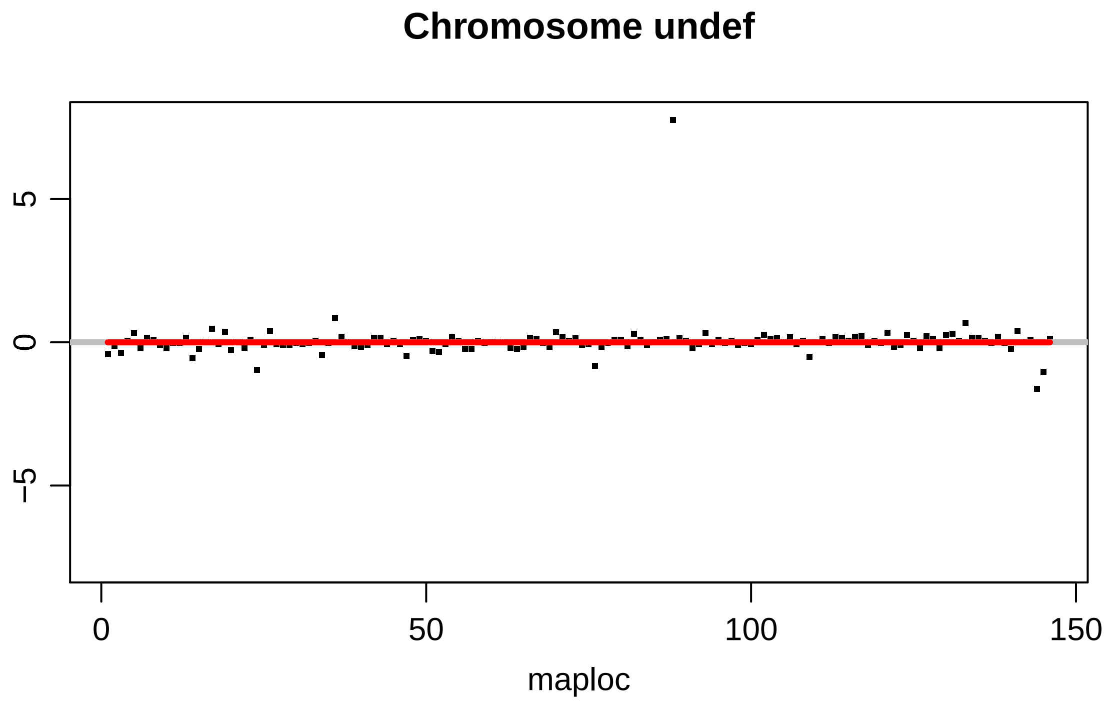


```
## Segplot might not work because of special characters in the sample names. Use only A-Z,a-z and 0-9!
##  There is a hidden function cn.mops:::.replaceNames that replaces the names in the "CNVDetectionResul[illegible]
```

_11_07_23_43_Sequoia_SN1.59.11_04_15_Neoscreen_v1_Auto_Sequoia_SN1.59.1

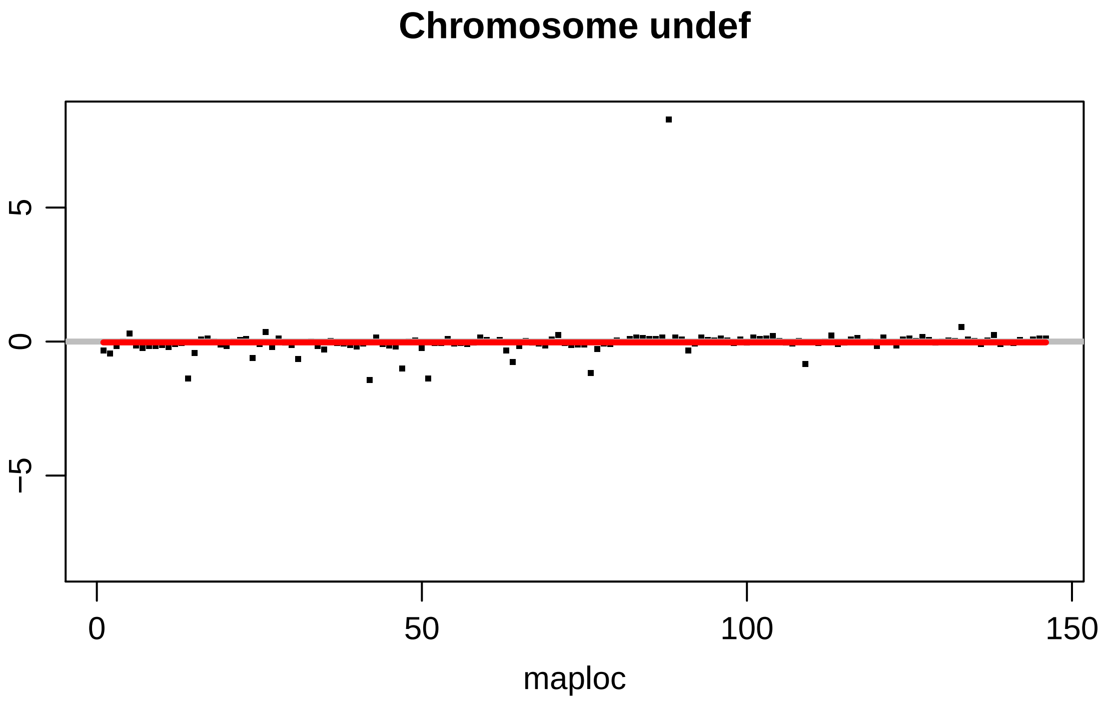


```
## Segplot might not work because of special characters in the sample names. Use only A-Z,a-z and 0-9!
##  There is a hidden function cn.mops:::.replaceNames that replaces the names in the "CNVDetectionResul
```

_11_07_23_43_Sequoia_SN1.59.11_04_15_Neoscreen_v1_Auto_Sequoia_SN1.59.1

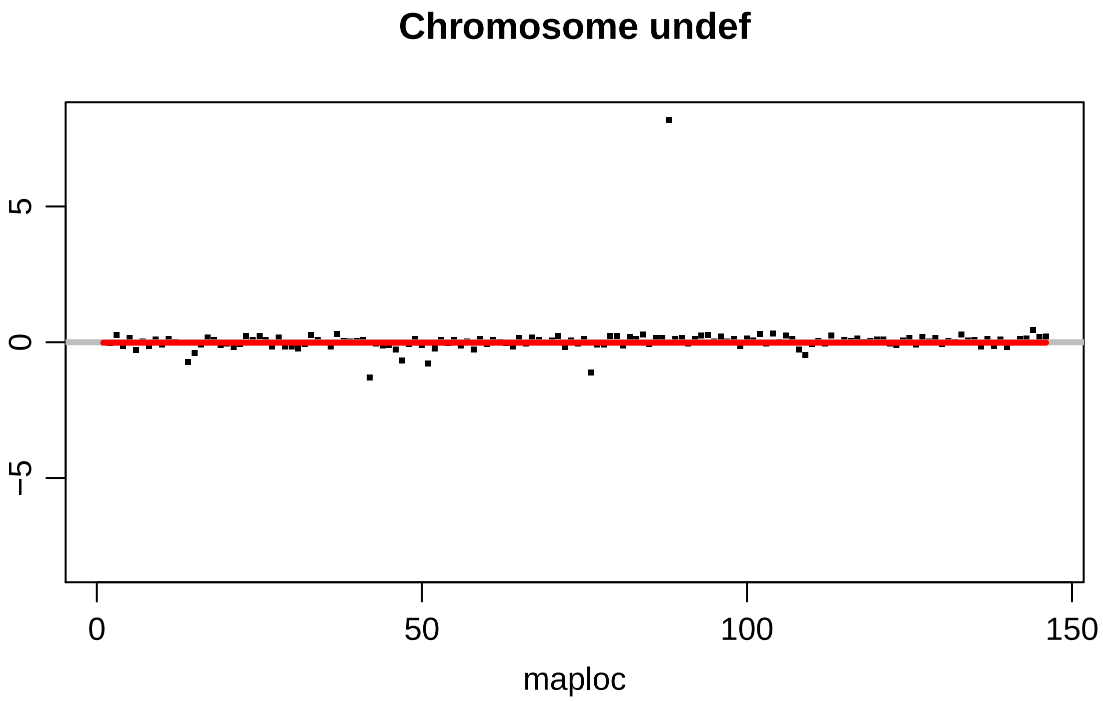

```
## Segplot might not work because of special characters in the sample names. Use only A-Z,a-z and 0-9!
##  There is a hidden function cn.mops:::.replaceNames that replaces the names in the "CNVDetectionResul
```

_11_07_23_43_Sequoia_SN1.59.11_04_15_Neoscreen_v1_Auto_Sequoia_SN1.59.1

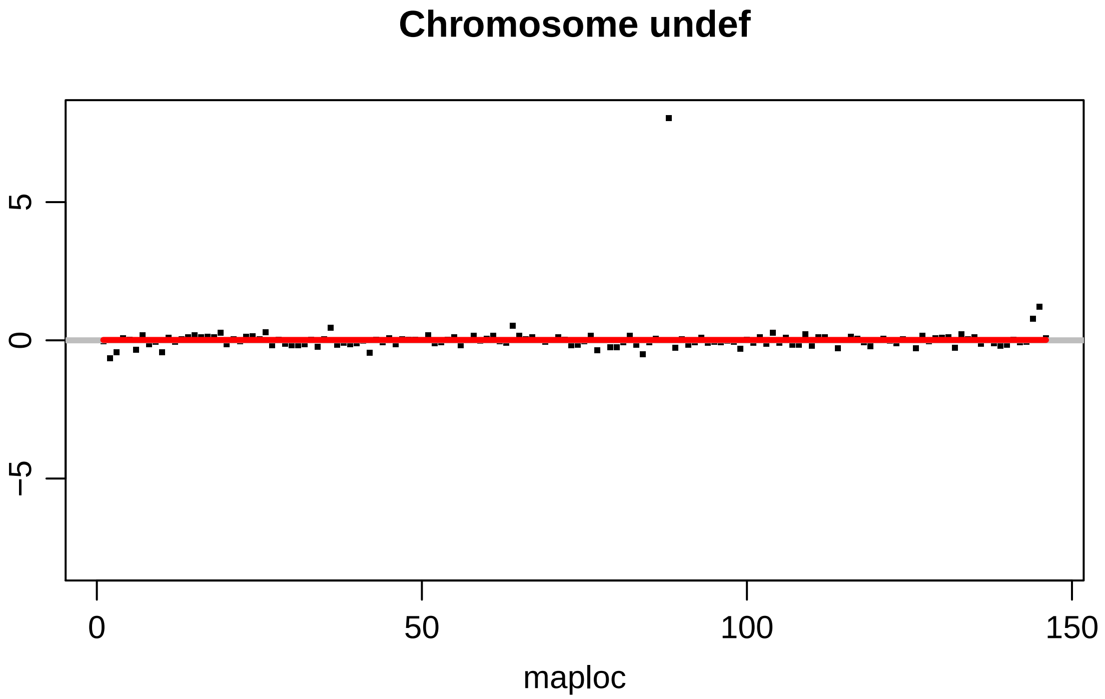


```
## Segplot might not work because of special characters in the sample names. Use only A-Z,a-z and 0-9!
##  There is a hidden function cn.mops:::.replaceNames that replaces the names in the "CNVDetectionResul
```

_11_07_23_43_Sequoia_SN1.59.11_04_15_Neoscreen_v1_Auto_Sequoia_SN1.59.1

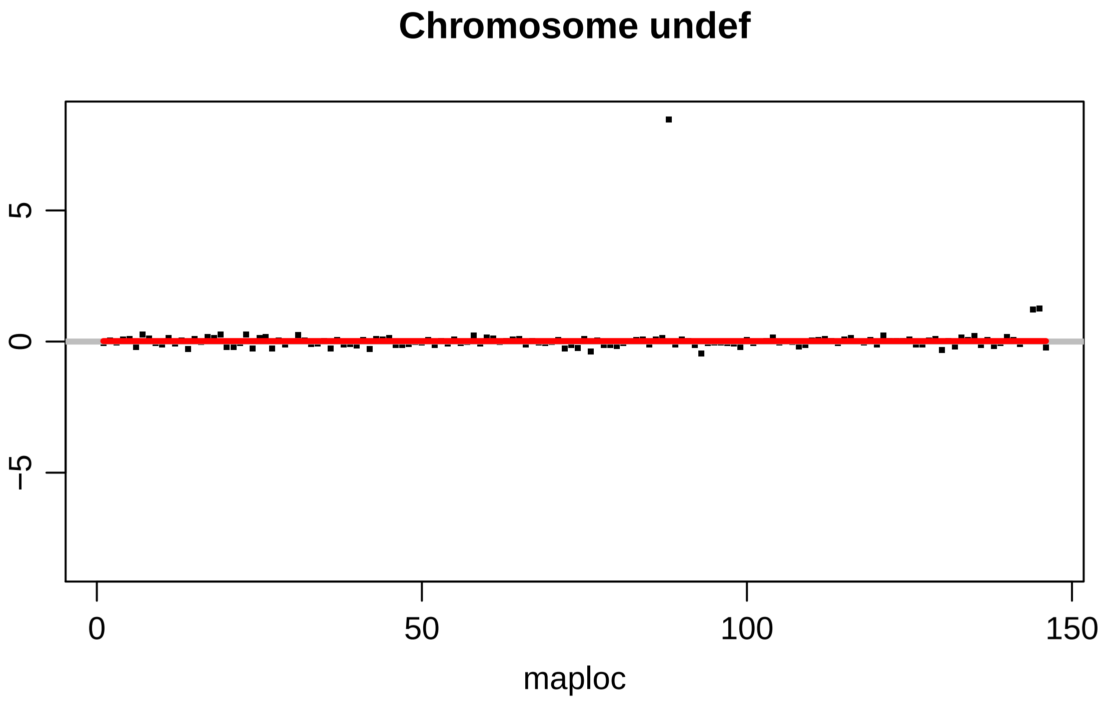


```
## Segplot might not work because of special characters in the sample names. Use only A-Z,a-z and 0-9!
##  There is a hidden function cn.mops:::.replaceNames that replaces the names in the "CNVDetectionResul
```

_11_07_23_43_Sequoia_SN1.59.11_04_15_Neoscreen_v1_Auto_Sequoia_SN1.59.1

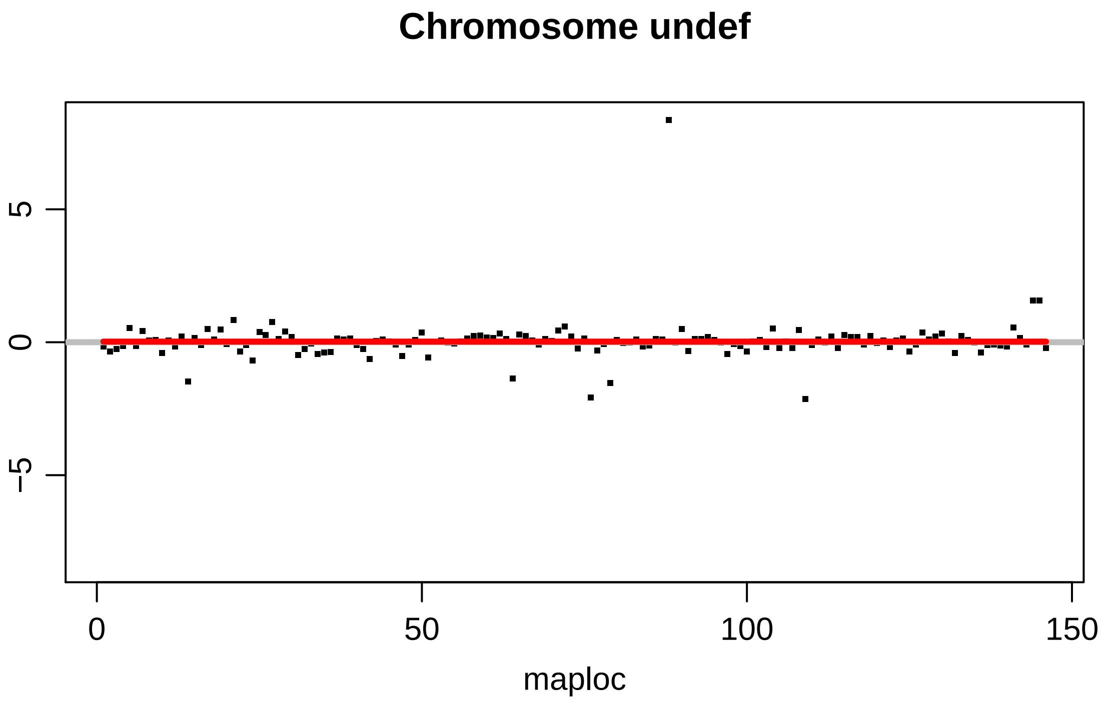

```
## Segplot might not work because of special characters in the sample names. Use only A-Z,a-z and 0-9!
##  There is a hidden function cn.mops:::.replaceNames that replaces the names in the "CNVDetectionResul
```

_11_07_23_43_Sequoia_SN1.59.11_04_15_Neoscreen_v1_Auto_Sequoia_SN1.59.1

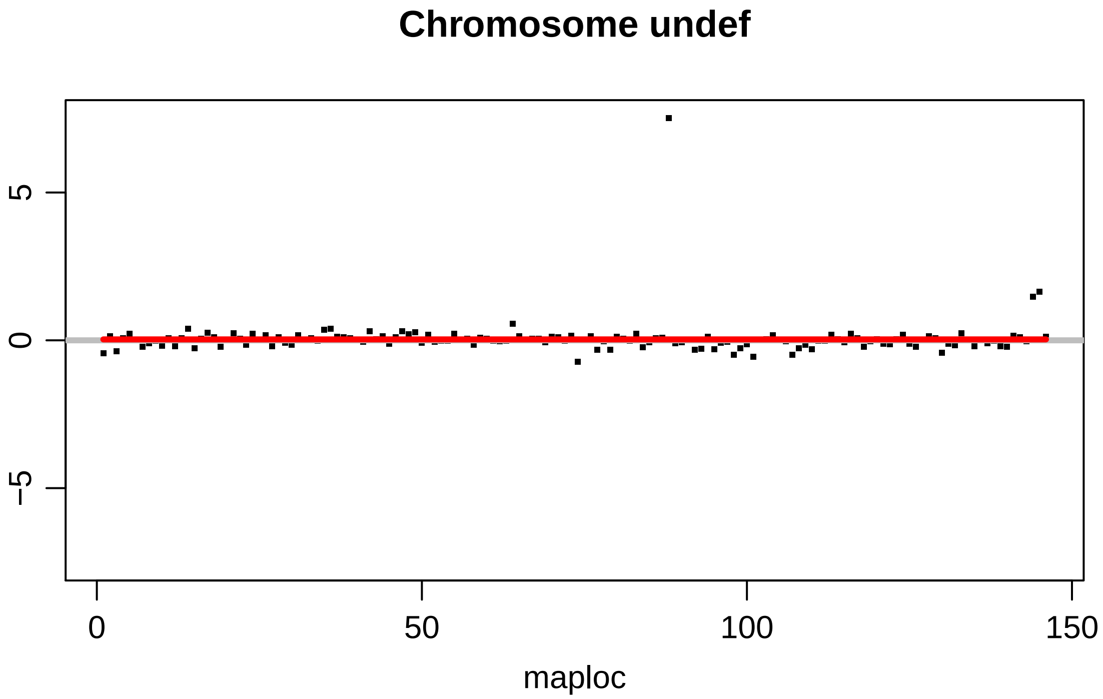


```
## Segplot might not work because of special characters in the sample names. Use only A-Z,a-z and 0-9!
##  There is a hidden function cn.mops:::.replaceNames that replaces the names in the "CNVDetectionResul
```

_11_07_23_43_Sequoia_SN1.59.11_04_15_Neoscreen_v1_Auto_Sequoia_SN1.59.1

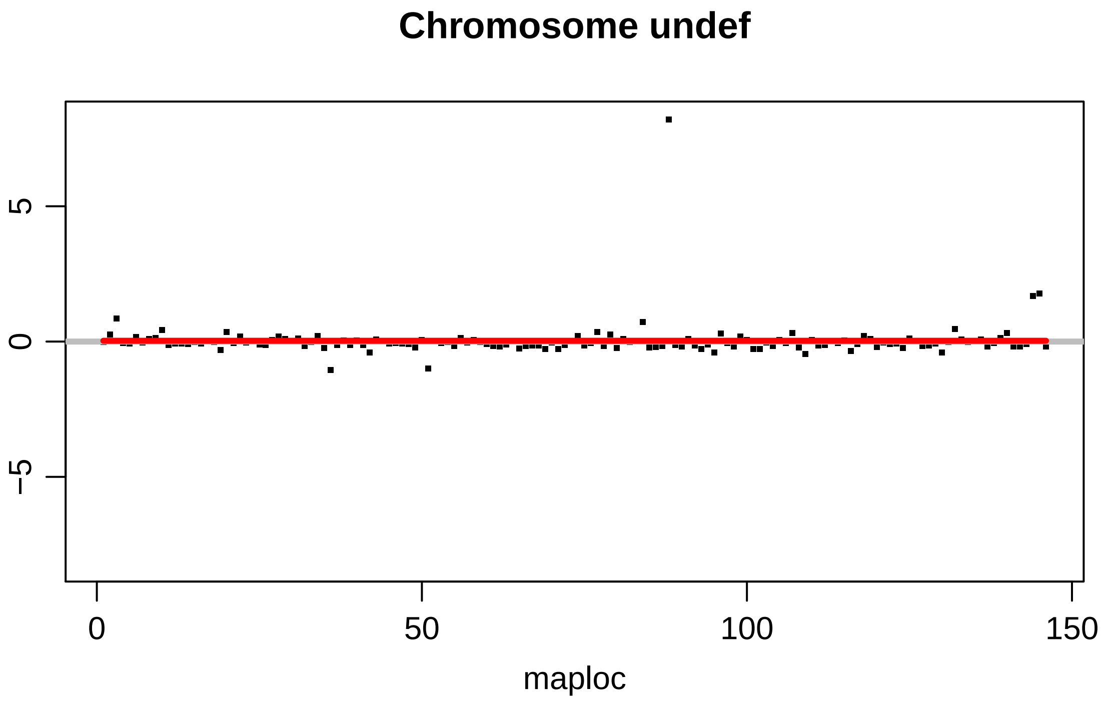


```
## Segplot might not work because of special characters in the sample names. Use only A-Z,a-z and 0-9!
##  There is a hidden function cn.mops:::.replaceNames that replaces the names in the "CNVDetectionResul
```

_11_07_23_43_Sequoia_SN1.59.11_04_15_Neoscreen_v1_Auto_Sequoia_SN1.59.1

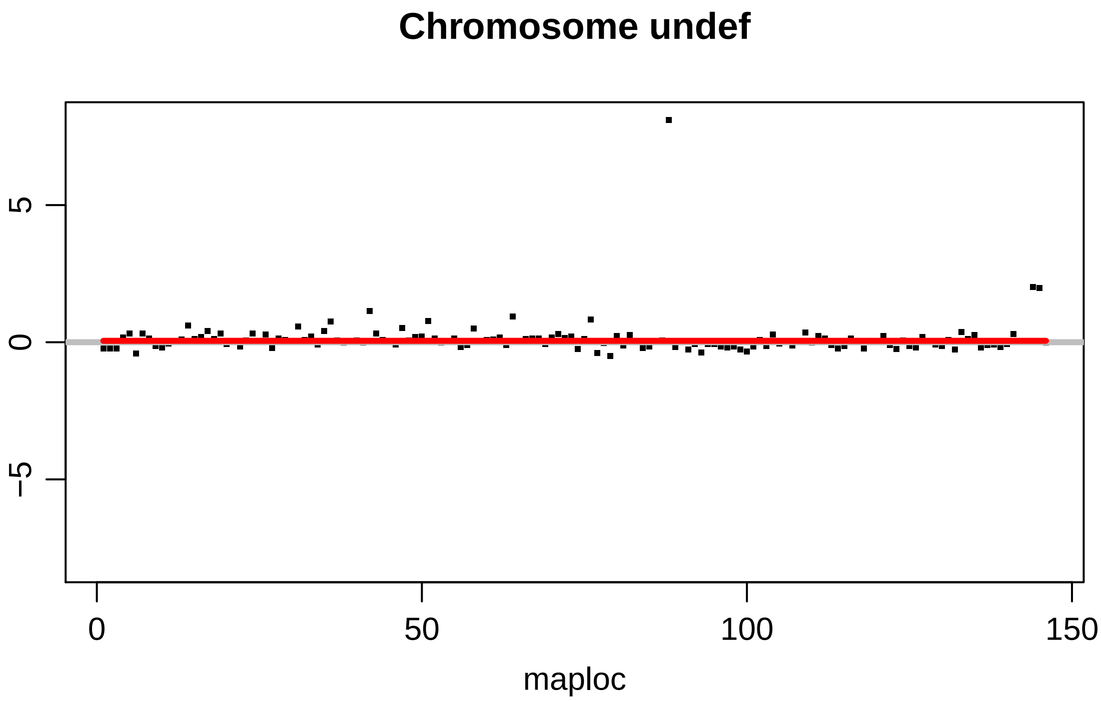

```
## Segplot might not work because of special characters in the sample names. Use only A-Z,a-z and 0-9!
##  There is a hidden function cn.mops:::.replaceNames that replaces the names in the "CNVDetectionResul
```

_11_07_23_43_Sequoia_SN1.59.11_04_15_Neoscreen_v1_Auto_Sequoia_SN1.59.1

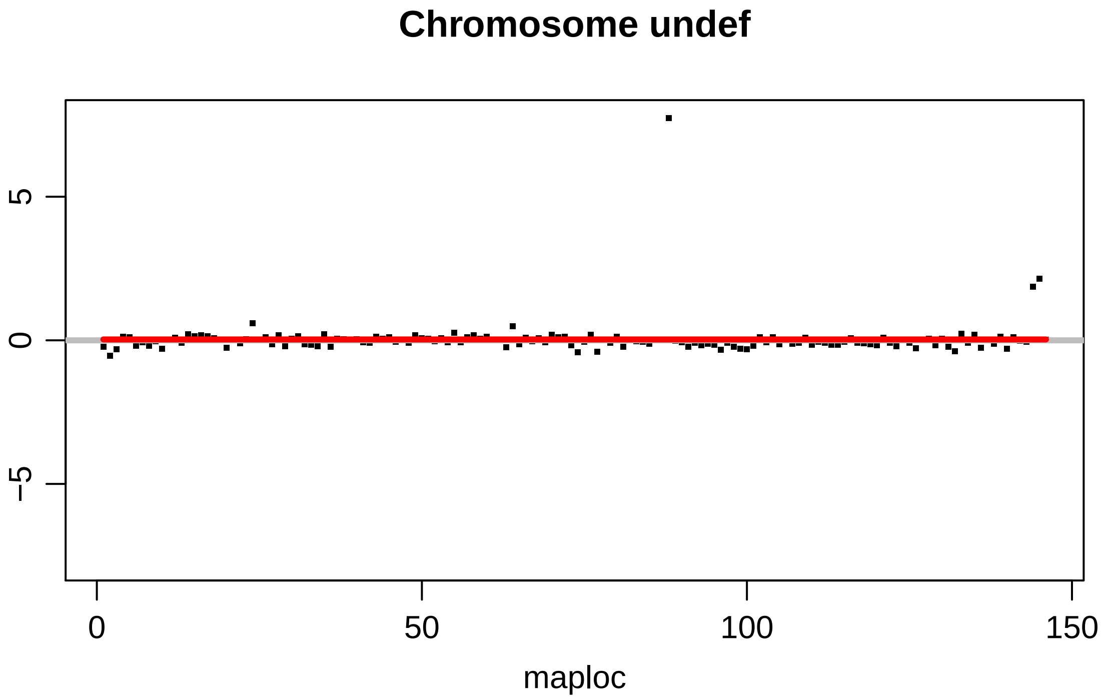


```
## Segplot might not work because of special characters in the sample names. Use only A-Z,a-z and 0-9!
##  There is a hidden function cn.mops:::.replaceNames that replaces the names in the "CNVDetectionResul
```

5_Sequoia_SN1.61.AmpliSeq_SNP.HID.L.316v2_190415_for_IC_Auto_Sequoia_SN1

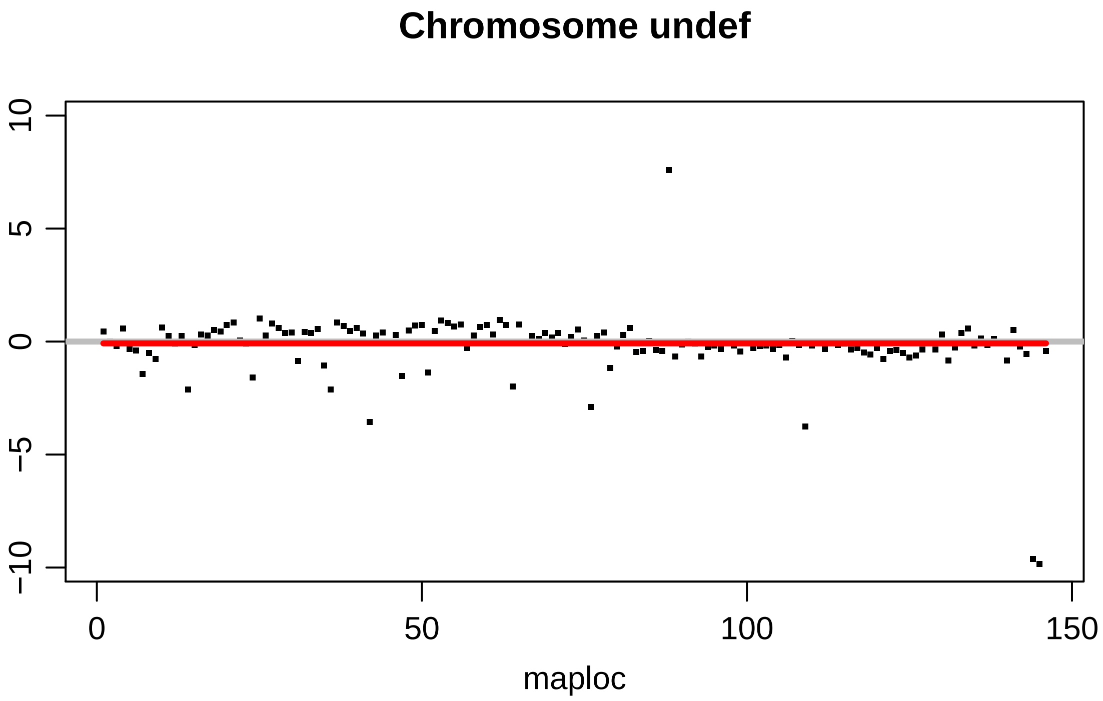


```
## Segplot might not work because of special characters in the sample names. Use only A-Z,a-z and 0-9!
##  There is a hidden function cn.mops:::.replaceNames that replaces the names in the "CNVDetectionResul
```

5_Sequoia_SN1.61.AmpliSeq_SNP.HID.L.316v2_190415_for_IC_Auto_Sequoia_SN1

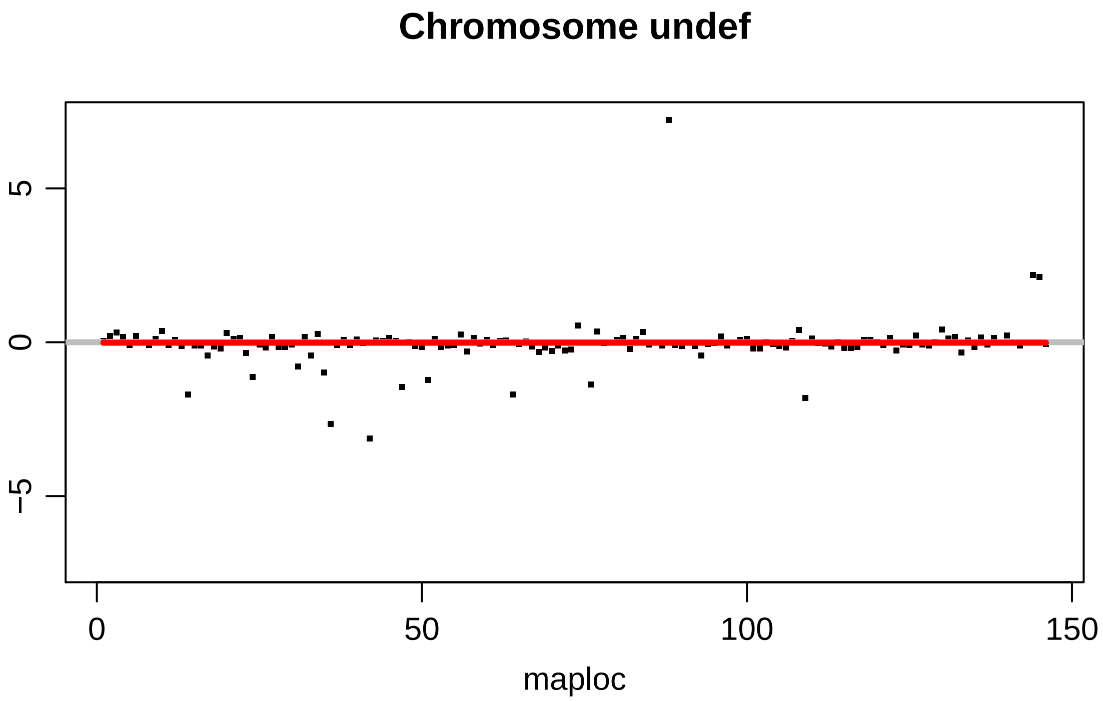

```
## Segplot might not work because of special characters in the sample names. Use only A-Z,a-z and 0-9!
##  There is a hidden function cn.mops:::.replaceNames that replaces the names in the "CNVDetectionResul
```

5_Sequoia_SN1.61.AmpliSeq_SNP.HID.L.316v2_190415_for_IC_Auto_Sequoia_SN1

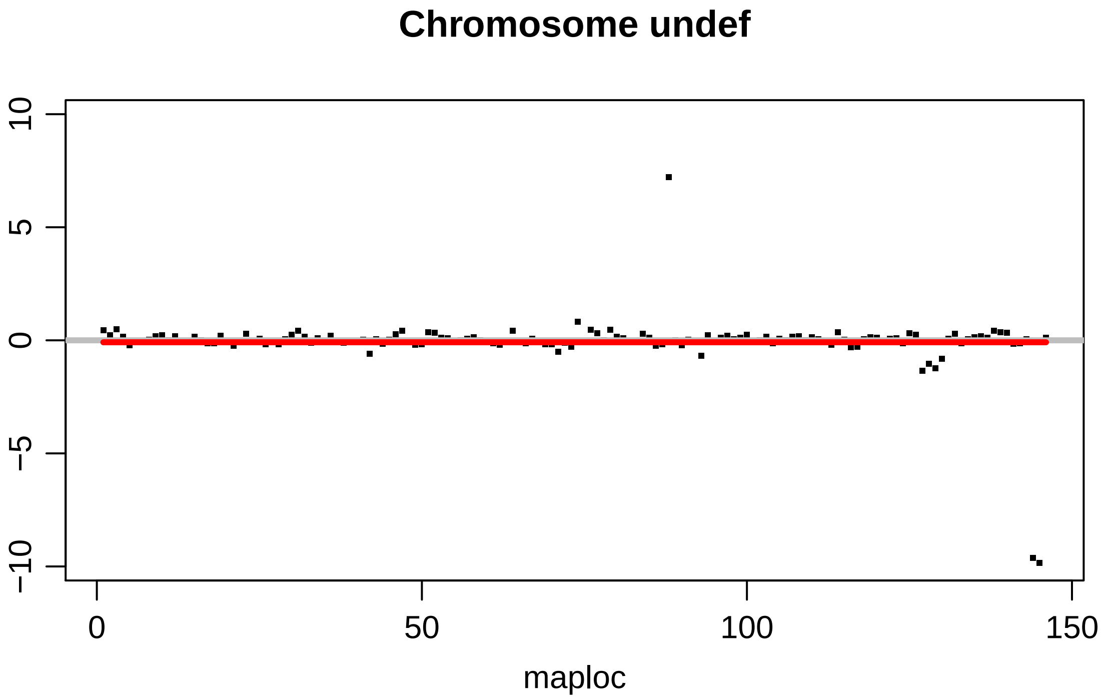


```
## Segplot might not work because of special characters in the sample names. Use only A-Z,a-z and 0-9!
##  There is a hidden function cn.mops:::.replaceNames that replaces the names in the "CNVDetectionResul
```

5_Sequoia_SN1.61.AmpliSeq_SNP.HID.L.316v2_190415_for_IC_Auto_Sequoia_SN1

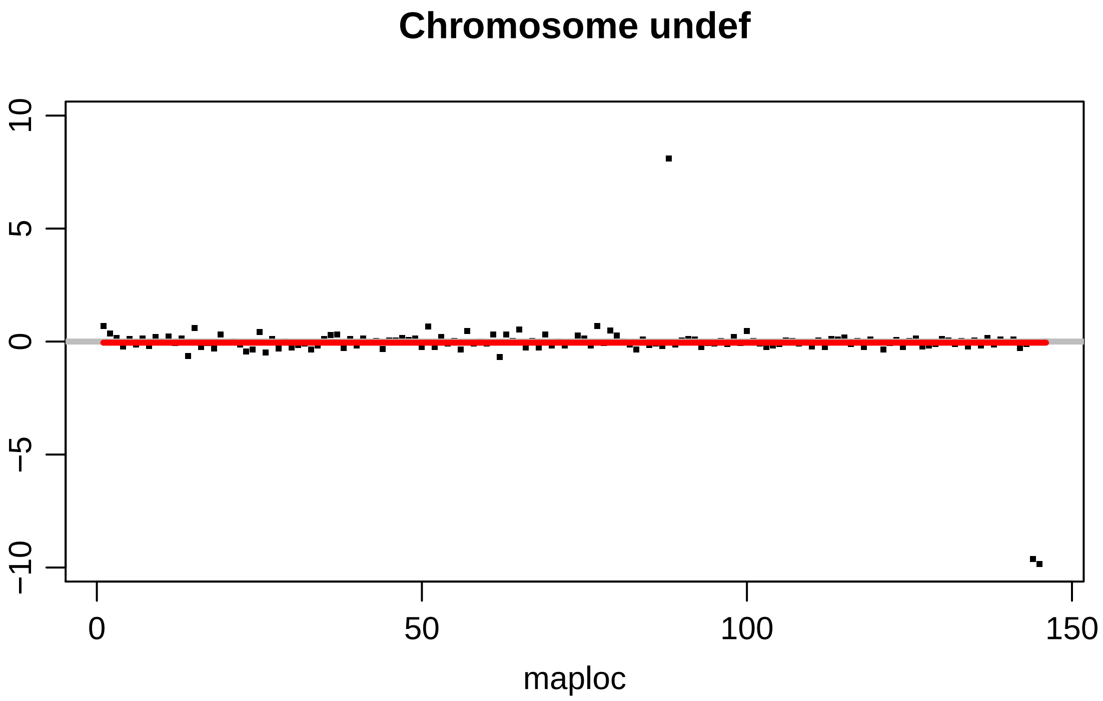


```
## Segplot might not work because of special characters in the sample names. Use only A-Z,a-z and 0-9!
##  There is a hidden function cn.mops:::.replaceNames that replaces the names in the "CNVDetectionResul
```

5_Sequoia_SN1.61.AmpliSeq_SNP.HID.L.316v2_190415_for_IC_Auto_Sequoia_SN1

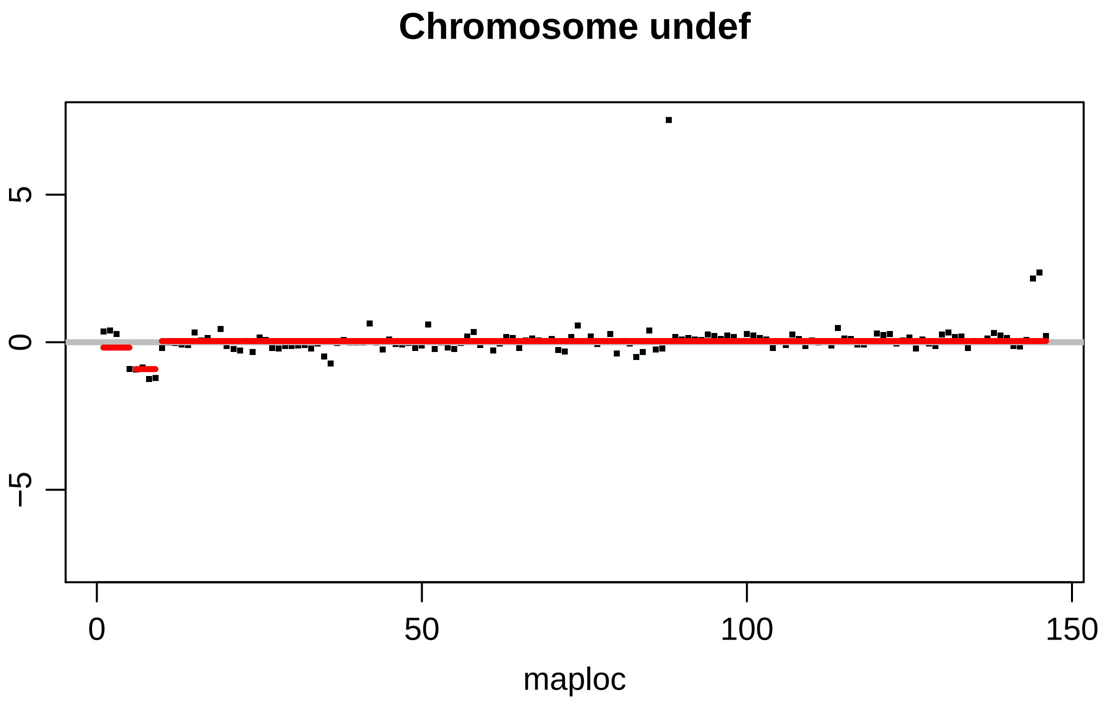

```
## Segplot might not work because of special characters in the sample names. Use only A-Z,a-z and 0-9!
##  There is a hidden function cn.mops:::.replaceNames that replaces the names in the "CNVDetectionResul
```

5_Sequoia_SN1.61.AmpliSeq_SNP.HID.L.316v2_190415_for_IC_Auto_Sequoia_SN1

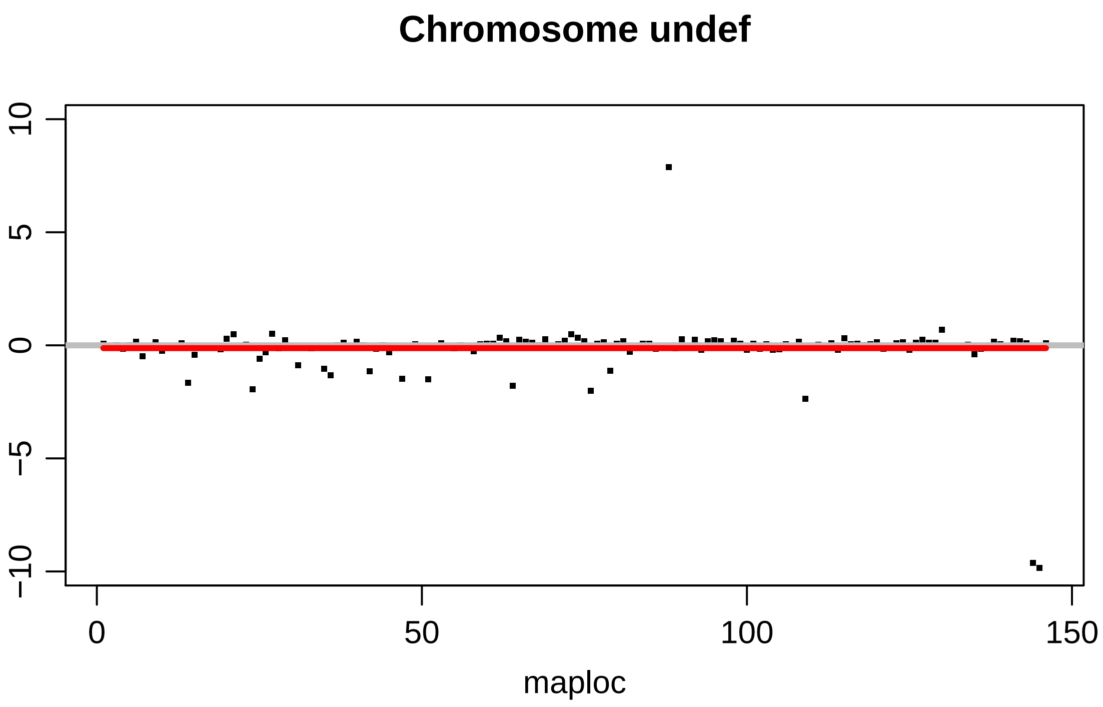


```
## Segplot might not work because of special characters in the sample names. Use only A-Z,a-z and 0-9!
##  There is a hidden function cn.mops:::.replaceNames that replaces the names in the "CNVDetectionResul
```

5_Sequoia_SN1.61.AmpliSeq_SNP.HID.L.316v2_190415_for_IC_Auto_Sequoia_SN1

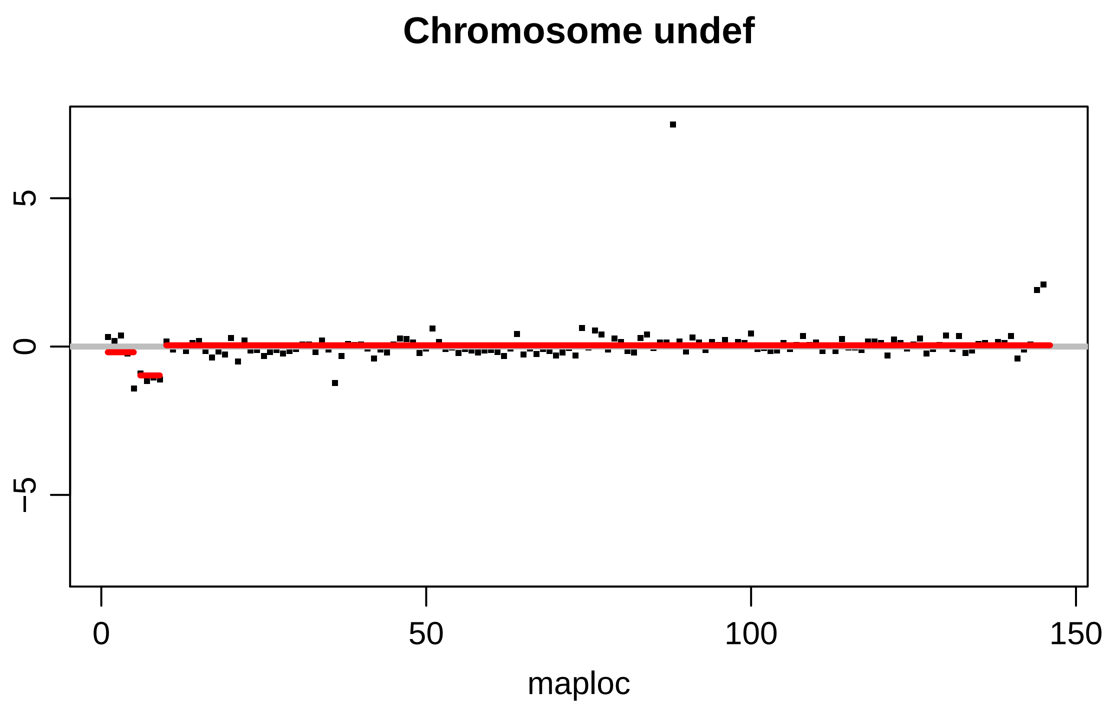


```
## Segplot might not work because of special characters in the sample names. Use only A-Z,a-z and 0-9!
##  There is a hidden function cn.mops:::.replaceNames that replaces the names in the "CNVDetectionResul
```

5_Sequoia_SN1.61.AmpliSeq_SNP.HID.L.316v2_190415_for_IC_Auto_Sequoia_SN1

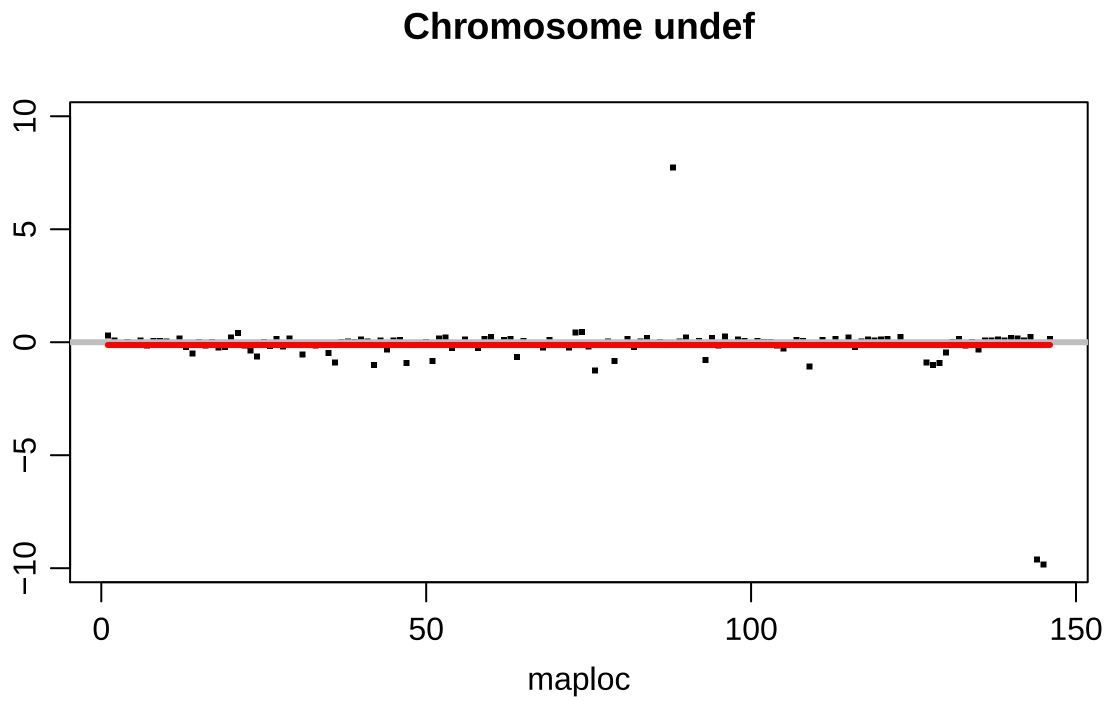

```
## Segplot might not work because of special characters in the sample names. Use only A-Z,a-z and 0-9!
##  There is a hidden function cn.mops:::.replaceNames that replaces the names in the "CNVDetectionResul
```

5_Sequoia_SN1.61.AmpliSeq_SNP.HID.L.316v2_190415_for_IC_Auto_Sequoia_SN1

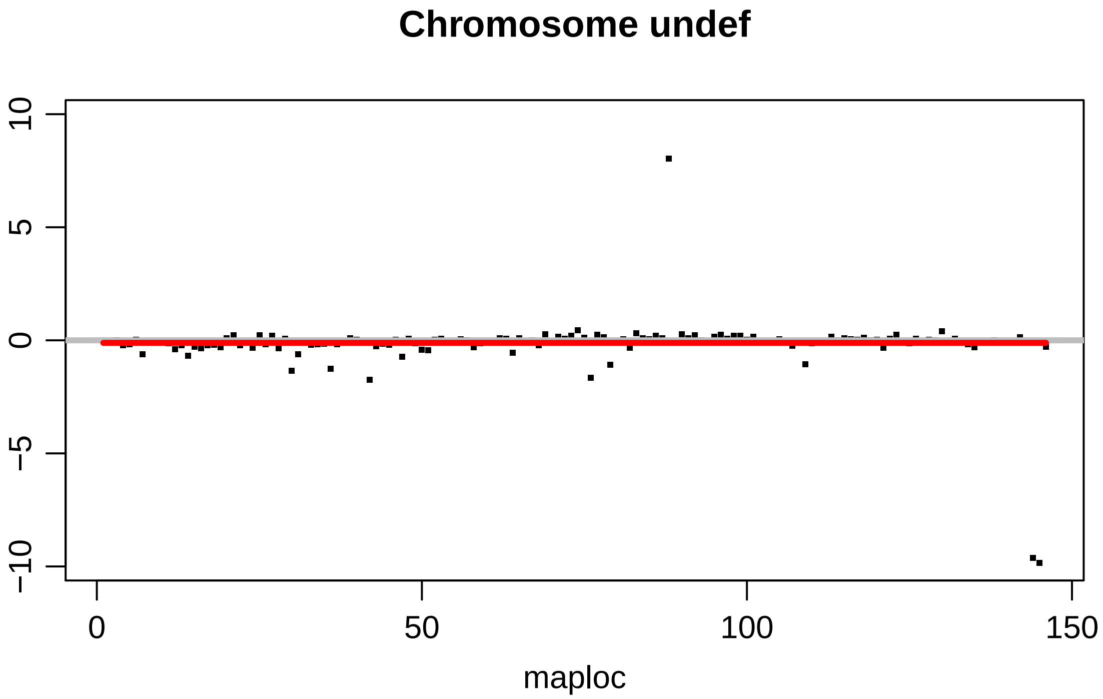


```
## Segplot might not work because of special characters in the sample names. Use only A-Z,a-z and 0-9!
##  There is a hidden function cn.mops:::.replaceNames that replaces the names in the "CNVDetectionResul
```

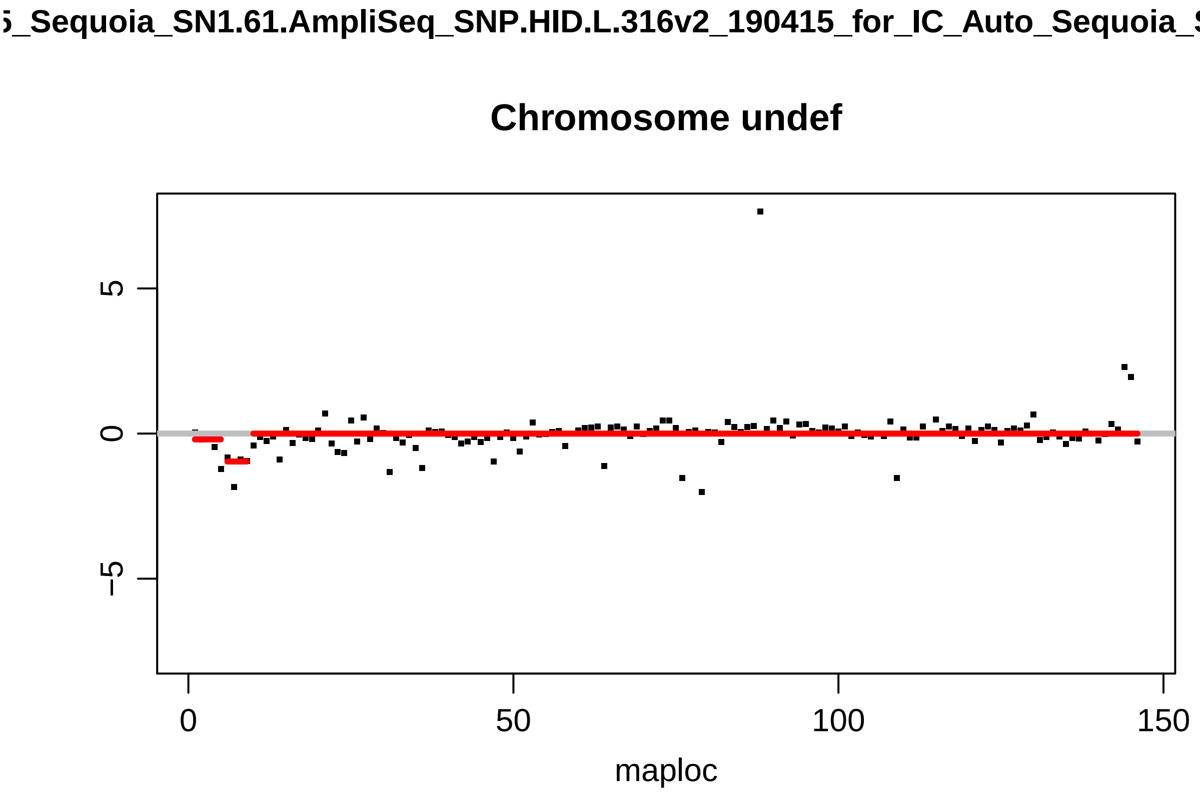


```
## Segplot might not work because of special characters in the sample names. Use only A-Z,a-z and 0-9!
##  There is a hidden function cn.mops:::.replaceNames that replaces the names in the "CNVDetectionResul
```

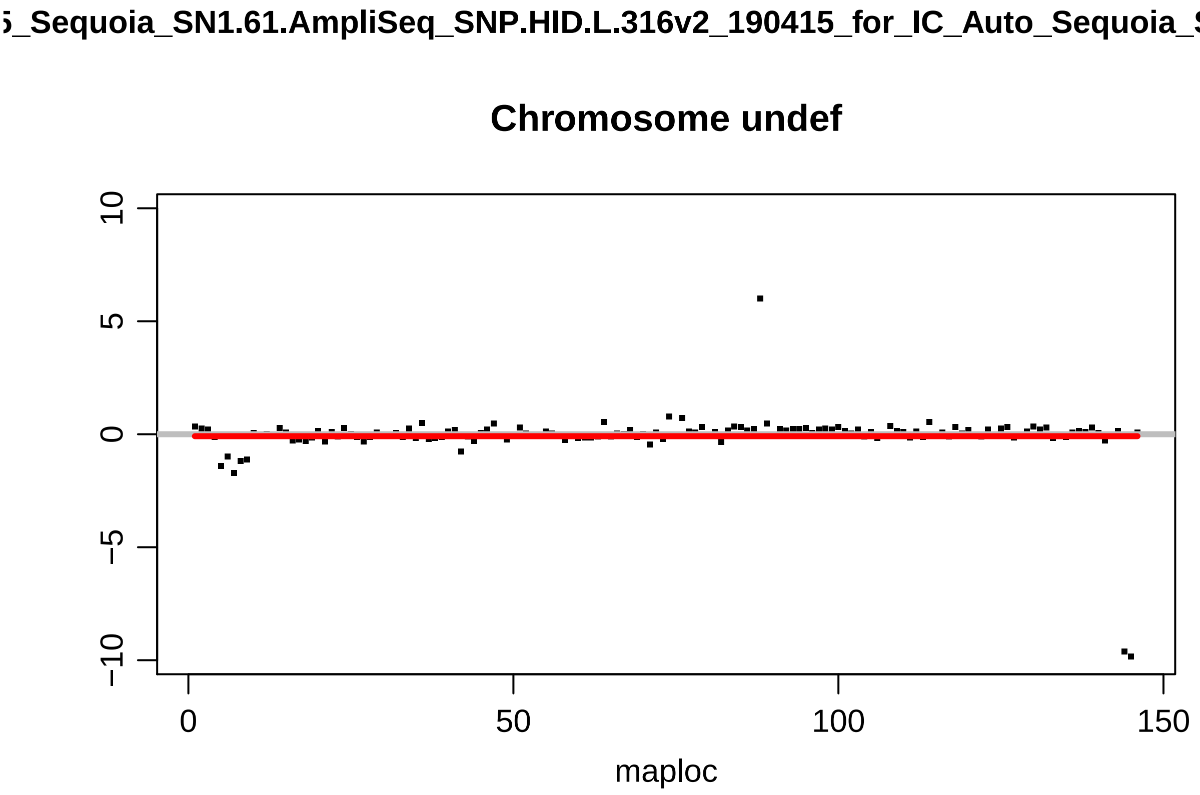

```
## Segplot might not work because of special characters in the sample names. Use only A-Z,a-z and 0-9!
##  There is a hidden function cn.mops:::.replaceNames that replaces the names in the "CNVDetectionResul
```

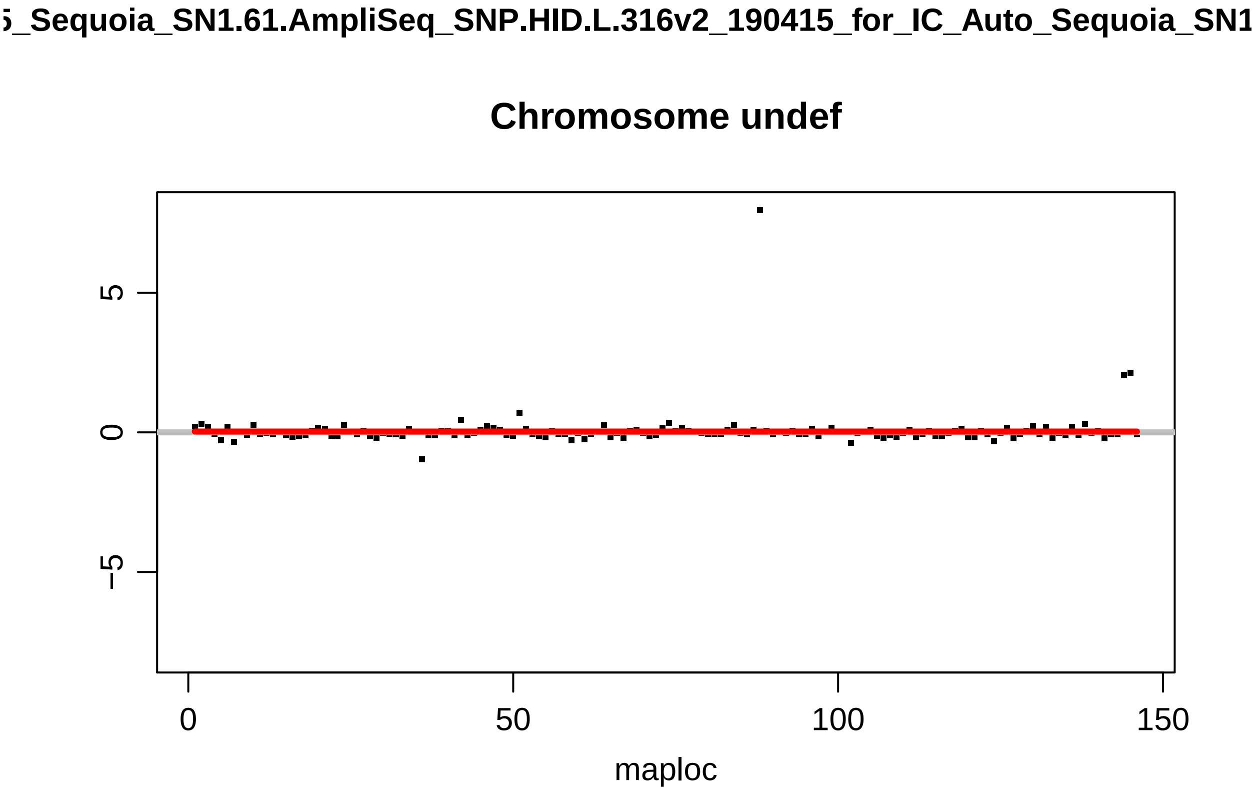


```
## Segplot might not work because of special characters in the sample names. Use only A-Z,a-z and 0-9!
##  There is a hidden function cn.mops:::.replaceNames that replaces the names in the "CNVDetectionResul
```

5_Sequoia_SN1.61.AmpliSeq_SNP.HID.L.316v2_190415_for_IC_Auto_Sequoia_SN1

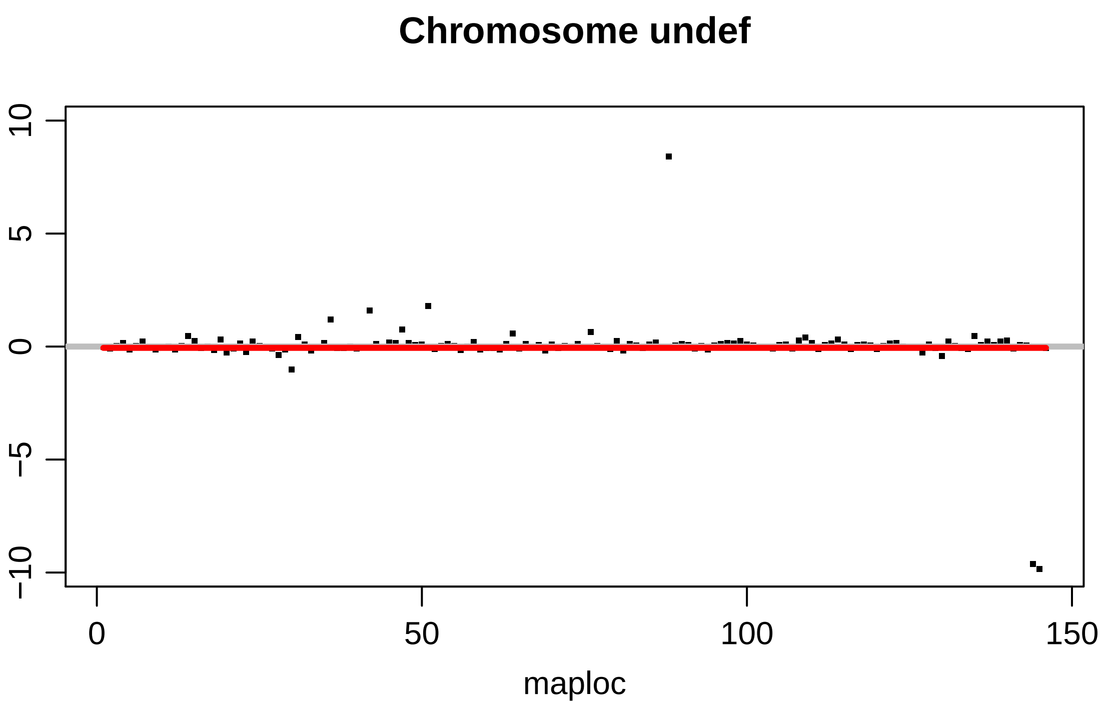


```
## Segplot might not work because of special characters in the sample names. Use only A-Z,a-z and 0-9!
##  There is a hidden function cn.mops:::.replaceNames that replaces the names in the "CNVDetectionResul
```

5_Sequoia_SN1.61.AmpliSeq_SNP.HID.L.316v2_190415_for_IC_Auto_Sequoia_SN1

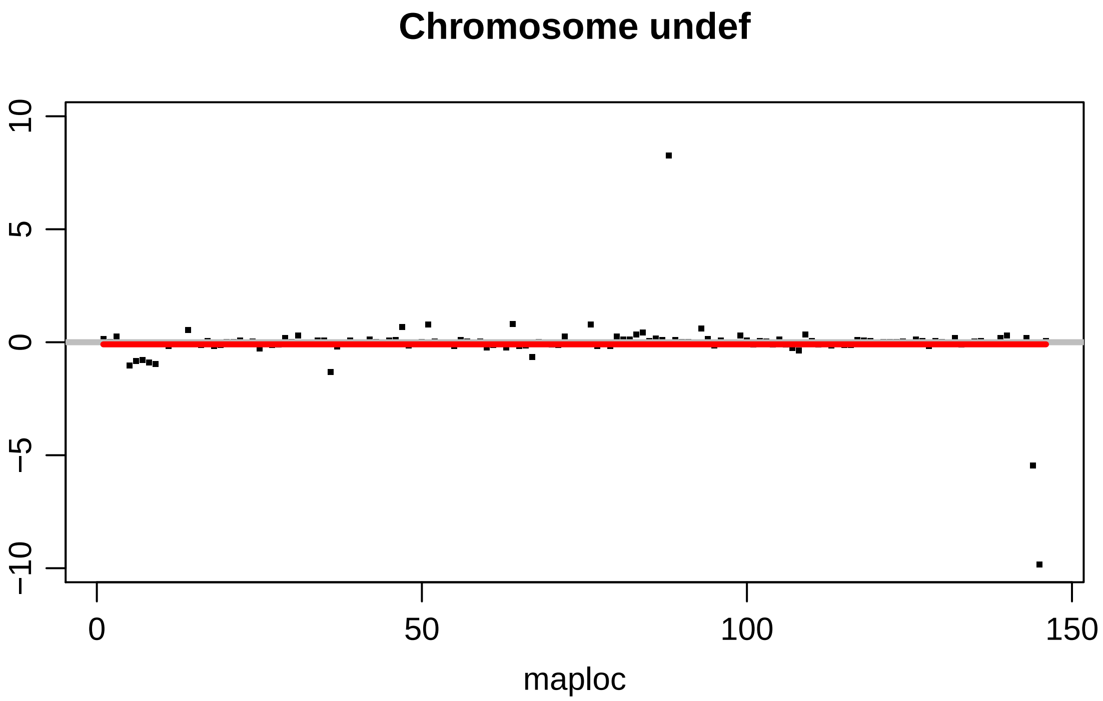

```
##
## CNV regions:
## GRanges object with 2 ranges and 55 metadata columns:
##       seqnames    ranges strand |
##          <Rle> <IRanges>  <Rle> |
##   [1]    undef  [ 6,  9]      * |
##   [2]    undef  [11, 17]      * |
##       Case_IonXpress_002_R_2015_04_11_07_23_43_Sequoia_SN1.59.11_04_15_Neoscreen_v1_Auto_Sequoia_SN1
##
##   [1]
##   [2]
##       Case_IonXpress_003_R_2015_04_11_07_23_43_Sequoia_SN1.59.11_04_15_Neoscreen_v1_Auto_Sequoia_SN1
##
##   [1]
##   [2]
##       Case_IonXpress_005_R_2015_04_11_07_23_43_Sequoia_SN1.59.11_04_15_Neoscreen_v1_Auto_Sequoia_SN1
##
##   [1]
##   [2]
##       Case_IonXpress_006_R_2015_04_11_07_23_43_Sequoia_SN1.59.11_04_15_Neoscreen_v1_Auto_Sequoia_SN1
##
##   [1]
##   [2]
##       Case_IonXpress_008_R_2015_04_11_07_23_43_Sequoia_SN1.59.11_04_15_Neoscreen_v1_Auto_Sequoia_SN1
##
##   [1]
##   [2]
##       Case_IonXpress_009_R_2015_04_11_07_23_43_Sequoia_SN1.59.11_04_15_Neoscreen_v1_Auto_Sequoia_SN1
##
##   [1]
##   [2]
##       Case_IonXpress_010_R_2015_04_11_07_23_43_Sequoia_SN1.59.11_04_15_Neoscreen_v1_Auto_Sequoia_SN1
##
##   [1]
##   [2]
##       Case_IonXpress_011_R_2015_04_11_07_23_43_Sequoia_SN1.59.11_04_15_Neoscreen_v1_Auto_Sequoia_SN1
##
##   [1]
##   [2]
##       Case_IonXpress_012_R_2015_04_11_07_23_43_Sequoia_SN1.59.11_04_15_Neoscreen_v1_Auto_Sequoia_SN1
##
##   [1]
##   [2]
##       Case_IonXpress_013_R_2015_04_11_07_23_43_Sequoia_SN1.59.11_04_15_Neoscreen_v1_Auto_Sequoia_SN1
##
##   [1]
##   [2]
##       Case_IonXpress_014_R_2015_04_11_07_23_43_Sequoia_SN1.59.11_04_15_Neoscreen_v1_Auto_Sequoia_SN1
##
##   [1]
##   [2]
##       Case_IonXpress_015_R_2015_04_11_07_23_43_Sequoia_SN1.59.11_04_15_Neoscreen_v1_Auto_Sequoia_SN1
##
##   [1]
```

```
## [2]
##     Case_IonXpress_018_R_2015_04_11_07_23_43_Sequoia_SN1.59.11_04_15_Neoscreen_v1_Auto_Sequoia_SN1
##
## [1]
## [2]
##     Case_IonXpress_021_R_2015_04_11_07_23_43_Sequoia_SN1.59.11_04_15_Neoscreen_v1_Auto_Sequoia_SN1
##
## [1]
## [2]
##     Case_IonXpress_022_R_2015_04_11_07_23_43_Sequoia_SN1.59.11_04_15_Neoscreen_v1_Auto_Sequoia_SN1
##
## [1]
## [2]
##     Case_IonXpress_023_R_2015_04_11_07_23_43_Sequoia_SN1.59.11_04_15_Neoscreen_v1_Auto_Sequoia_SN1
##
## [1]
## [2]
##     Case_IonXpress_026_R_2015_04_11_07_23_43_Sequoia_SN1.59.11_04_15_Neoscreen_v1_Auto_Sequoia_SN1
##
## [1]
## [2]
##     Case_IonXpress_028_R_2015_04_11_07_23_43_Sequoia_SN1.59.11_04_15_Neoscreen_v1_Auto_Sequoia_SN1
##
## [1]
## [2]
##     Case_IonXpress_029_R_2015_04_11_07_23_43_Sequoia_SN1.59.11_04_15_Neoscreen_v1_Auto_Sequoia_SN1
##
## [1]
## [2]
##     Case_IonXpress_030_R_2015_04_11_07_23_43_Sequoia_SN1.59.11_04_15_Neoscreen_v1_Auto_Sequoia_SN1
##
## [1]
## [2]
##     Case_IonXpress_031_R_2015_04_11_07_23_43_Sequoia_SN1.59.11_04_15_Neoscreen_v1_Auto_Sequoia_SN1
##
## [1]
## [2]
##     Case_IonXpress_032_R_2015_04_11_07_23_43_Sequoia_SN1.59.11_04_15_Neoscreen_v1_Auto_Sequoia_SN1
##
## [1]
## [2]
##     Case_IonXpress_034_R_2015_04_11_07_23_43_Sequoia_SN1.59.11_04_15_Neoscreen_v1_Auto_Sequoia_SN1
##
## [1]
## [2]
##     Case_IonXpress_035_R_2015_04_11_07_23_43_Sequoia_SN1.59.11_04_15_Neoscreen_v1_Auto_Sequoia_SN1
##
## [1]
## [2]
##     Case_IonXpress_036_R_2015_04_11_07_23_43_Sequoia_SN1.59.11_04_15_Neoscreen_v1_Auto_Sequoia_SN1
##
## [1]
## [2]
##     Case_IonXpress_049_R_2015_04_11_07_23_43_Sequoia_SN1.59.11_04_15_Neoscreen_v1_Auto_Sequoia_SN1
```

```
##
##   [1]
##   [2]
##        Case_IonXpress_051_R_2015_04_11_07_23_43_Sequoia_SN1.59.11_04_15_Neoscreen_v1_Auto_Sequoia_SN1
##
##   [1]
##   [2]
##        Case_IonXpress_052_R_2015_04_11_07_23_43_Sequoia_SN1.59.11_04_15_Neoscreen_v1_Auto_Sequoia_SN1
##
##   [1]
##   [2]
##        Case_IonXpress_053_R_2015_04_11_07_23_43_Sequoia_SN1.59.11_04_15_Neoscreen_v1_Auto_Sequoia_SN1
##
##   [1]
##   [2]
##        Case_IonXpress_054_R_2015_04_11_07_23_43_Sequoia_SN1.59.11_04_15_Neoscreen_v1_Auto_Sequoia_SN1
##
##   [1]
##   [2]
##        Case_IonXpress_055_R_2015_04_11_07_23_43_Sequoia_SN1.59.11_04_15_Neoscreen_v1_Auto_Sequoia_SN1
##
##   [1]
##   [2]
##        Case_IonXpress_056_R_2015_04_11_07_23_43_Sequoia_SN1.59.11_04_15_Neoscreen_v1_Auto_Sequoia_SN1
##
##   [1]
##   [2]
##        Case_IonXpress_057_R_2015_04_11_07_23_43_Sequoia_SN1.59.11_04_15_Neoscreen_v1_Auto_Sequoia_SN1
##
##   [1]
##   [2]
##        Case_IonXpress_058_R_2015_04_11_07_23_43_Sequoia_SN1.59.11_04_15_Neoscreen_v1_Auto_Sequoia_SN1
##
##   [1]
##   [2]
##        Case_IonXpress_059_R_2015_04_11_07_23_43_Sequoia_SN1.59.11_04_15_Neoscreen_v1_Auto_Sequoia_SN1
##
##   [1]
##   [2]
##        Case_IonXpress_060_R_2015_04_11_07_23_43_Sequoia_SN1.59.11_04_15_Neoscreen_v1_Auto_Sequoia_SN1
##
##   [1]
##   [2]
##        Case_IonXpress_061_R_2015_04_11_07_23_43_Sequoia_SN1.59.11_04_15_Neoscreen_v1_Auto_Sequoia_SN1
##
##   [1]
##   [2]
##        Case_IonXpress_062_R_2015_04_11_07_23_43_Sequoia_SN1.59.11_04_15_Neoscreen_v1_Auto_Sequoia_SN1
##
##   [1]
##   [2]
##        Case_IonXpress_063_R_2015_04_11_07_23_43_Sequoia_SN1.59.11_04_15_Neoscreen_v1_Auto_Sequoia_SN1
##
##   [1]
```

```
## [2]
##     Case_IonXpress_064_R_2015_04_11_07_23_43_Sequoia_SN1.59.11_04_15_Neoscreen_v1_Auto_Sequoia_SN1
##
## [1]
## [2]
##     Case_IonXpress_new_run_001_R_2015_04_19_13_34_45_Sequoia_SN1.61.AmpliSeq_SNP.HID.L.316v2_19041
##
## [1]
## [2]
##     Case_IonXpress_new_run_002_R_2015_04_19_13_34_45_Sequoia_SN1.61.AmpliSeq_SNP.HID.L.316v2_19041
##
## [1]
## [2]
##     Case_IonXpress_new_run_003_R_2015_04_19_13_34_45_Sequoia_SN1.61.AmpliSeq_SNP.HID.L.316v2_19041
##
## [1]
## [2]
##     Case_IonXpress_new_run_004_R_2015_04_19_13_34_45_Sequoia_SN1.61.AmpliSeq_SNP.HID.L.316v2_19041
##
## [1]
## [2]
##     Case_IonXpress_new_run_005_R_2015_04_19_13_34_45_Sequoia_SN1.61.AmpliSeq_SNP.HID.L.316v2_19041
##
## [1]
## [2]
##     Case_IonXpress_new_run_006_R_2015_04_19_13_34_45_Sequoia_SN1.61.AmpliSeq_SNP.HID.L.316v2_19041
##
## [1]
## [2]
##     Case_IonXpress_new_run_007_R_2015_04_19_13_34_45_Sequoia_SN1.61.AmpliSeq_SNP.HID.L.316v2_19041
##
## [1]
## [2]
##     Case_IonXpress_new_run_008_R_2015_04_19_13_34_45_Sequoia_SN1.61.AmpliSeq_SNP.HID.L.316v2_19041
##
## [1]
## [2]
##     Case_IonXpress_new_run_009_R_2015_04_19_13_34_45_Sequoia_SN1.61.AmpliSeq_SNP.HID.L.316v2_19041
##
## [1]
## [2]
##     Case_IonXpress_new_run_010_R_2015_04_19_13_34_45_Sequoia_SN1.61.AmpliSeq_SNP.HID.L.316v2_19041
##
## [1]
## [2]
##     Case_IonXpress_new_run_011_R_2015_04_19_13_34_45_Sequoia_SN1.61.AmpliSeq_SNP.HID.L.316v2_19041
##
## [1]
## [2]
##     Case_IonXpress_new_run_012_R_2015_04_19_13_34_45_Sequoia_SN1.61.AmpliSeq_SNP.HID.L.316v2_19041
##
## [1]
## [2]
##     Case_IonXpress_new_run_026_R_2015_04_19_13_34_45_Sequoia_SN1.61.AmpliSeq_SNP.HID.L.316v2_19041
```

```
##
##   [1]
##   [2]
##       Case_IonXpress_new_run_030_R_2015_04_19_13_34_45_Sequoia_SN1.61.AmpliSeq_SNP.HID.L.316v2_190415
##
##   [1]
##   [2]
##       Case_IonXpress_new_run_064_R_2015_04_19_13_34_45_Sequoia_SN1.61.AmpliSeq_SNP.HID.L.316v2_190415
##
##   [1]
##   [2]
##   -------
##   seqinfo: 1 sequence from an unspecified genome; no seqlengths
##
## Individual CNVs:
## GRanges object with 4 ranges and 4 metadata columns:
##       seqnames    ranges strand |
##          <Rle> <IRanges>  <Rle> |
##   [1]    undef  [11, 17]      * |
##   [2]    undef  [ 6,  9]      * |
##   [3]    undef  [ 6,  9]      * |
##   [4]    undef  [ 6,  9]      * |
##
##
##   [1]                                          Case_IonXpress_006_R_2015_04_11_07_23_43_Sequoia_SN1
##   [2] Case_IonXpress_new_run_005_R_2015_04_19_13_34_45_Sequoia_SN1.61.AmpliSeq_SNP.HID.L.316v2_190415
##   [3] Case_IonXpress_new_run_007_R_2015_04_19_13_34_45_Sequoia_SN1.61.AmpliSeq_SNP.HID.L.316v2_190415
##   [4] Case_IonXpress_new_run_010_R_2015_04_19_13_34_45_Sequoia_SN1.61.AmpliSeq_SNP.HID.L.316v2_190415
##           median       mean          CN
##        <numeric>  <numeric> <character>
##   [1] -0.9996570 -0.9995985         CN1
##   [2] -0.9757434 -0.9097077         CN1
##   [3] -0.9733714 -0.9727604         CN1
##   [4] -0.9956650 -0.9626556         CN1
##   -------
##   seqinfo: 1 sequence from an unspecified genome; no seqlengths
## [1] "/Users/gdemidov/Downloads/doc/Run_15_fin_05_qc.xls"

## Normalizing...

## Starting local modeling, please be patient...

## Reference sequence:  undef

## Starting segmentation algorithm...

## Using "fastseg" for segmentation.

## [1] ""
## [1] "/Users/gdemidov/Downloads/doc/Run_15_fin_05_qc.xls"
## [1] ""
```

```
## Segplot might not work because of special characters in the sample names. Use only A-Z,a-z and 0-9!
##  There is a hidden function cn.mops:::.replaceNames that replaces the names in the "CNVDetectionResul
```

5_Sequoia_SN1.61.AmpliSeq_SNP.HID.L.316v2_190415_for_IC_Auto_Sequoia_SN1

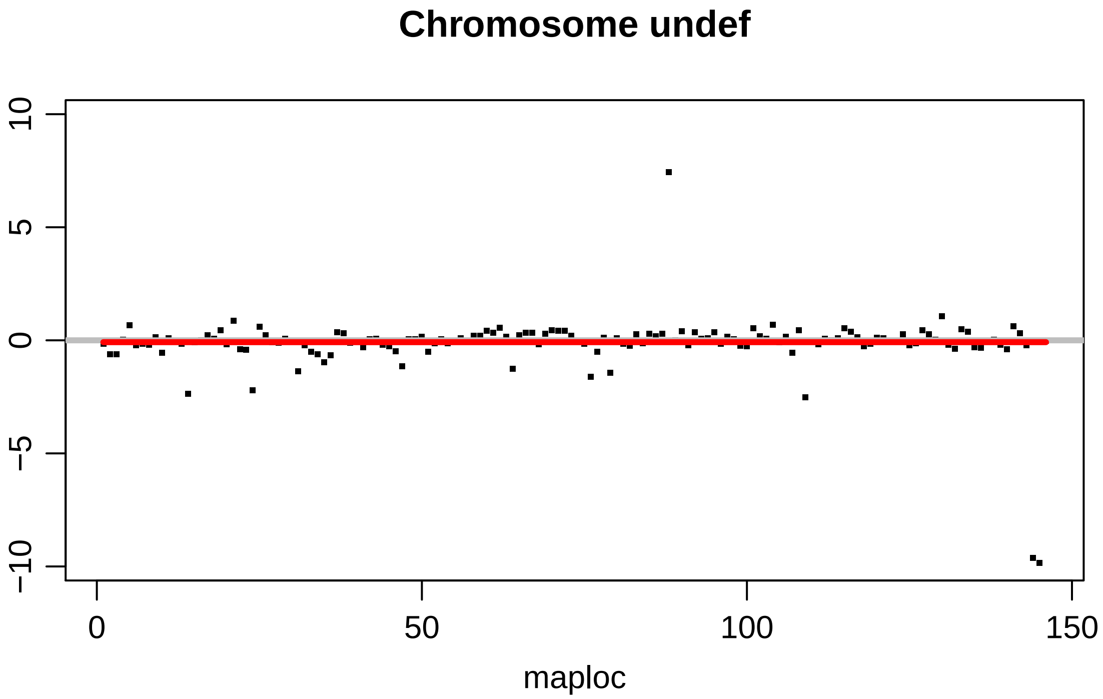


```
## Segplot might not work because of special characters in the sample names. Use only A-Z,a-z and 0-9!
##  There is a hidden function cn.mops:::.replaceNames that replaces the names in the "CNVDetectionResul
```

press_002_R_2012_09_03_22_20_58_Sequoia_SN1.18.withoutCYP_32_Run_15_hg

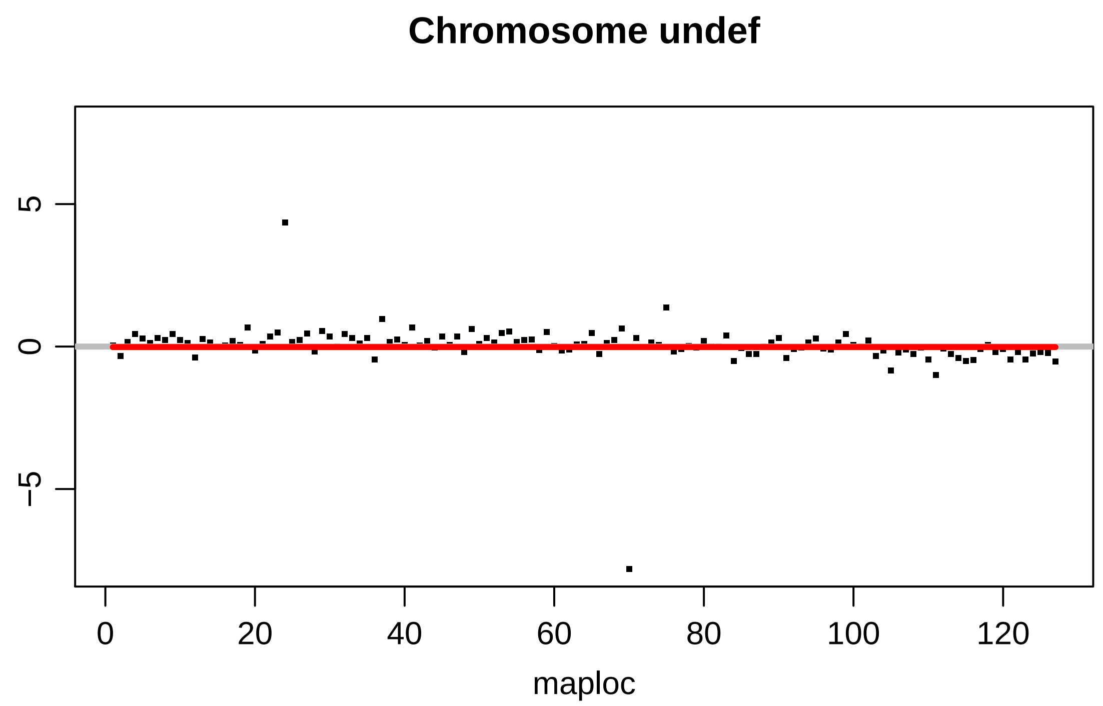


```
## Segplot might not work because of special characters in the sample names. Use only A-Z,a-z and 0-9!
##  There is a hidden function cn.mops:::.replaceNames that replaces the names in the "CNVDetectionResul
```

press_003_R_2012_09_03_22_20_58_Sequoia_SN1.18.withoutCYP_32_Run_15_hg

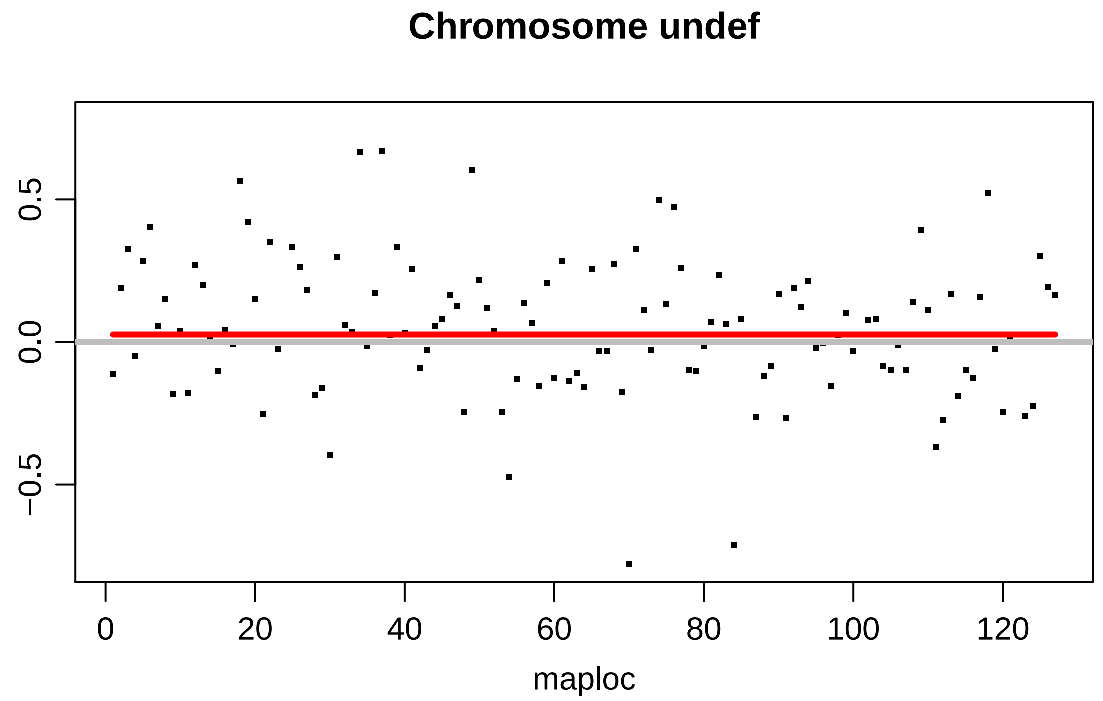

```
## Segplot might not work because of special characters in the sample names. Use only A-Z,a-z and 0-9!
##  There is a hidden function cn.mops:::.replaceNames that replaces the names in the "CNVDetectionResul
```

press_004_R_2012_09_03_22_20_58_Sequoia_SN1.18.withoutCYP_32_Run_15_hg

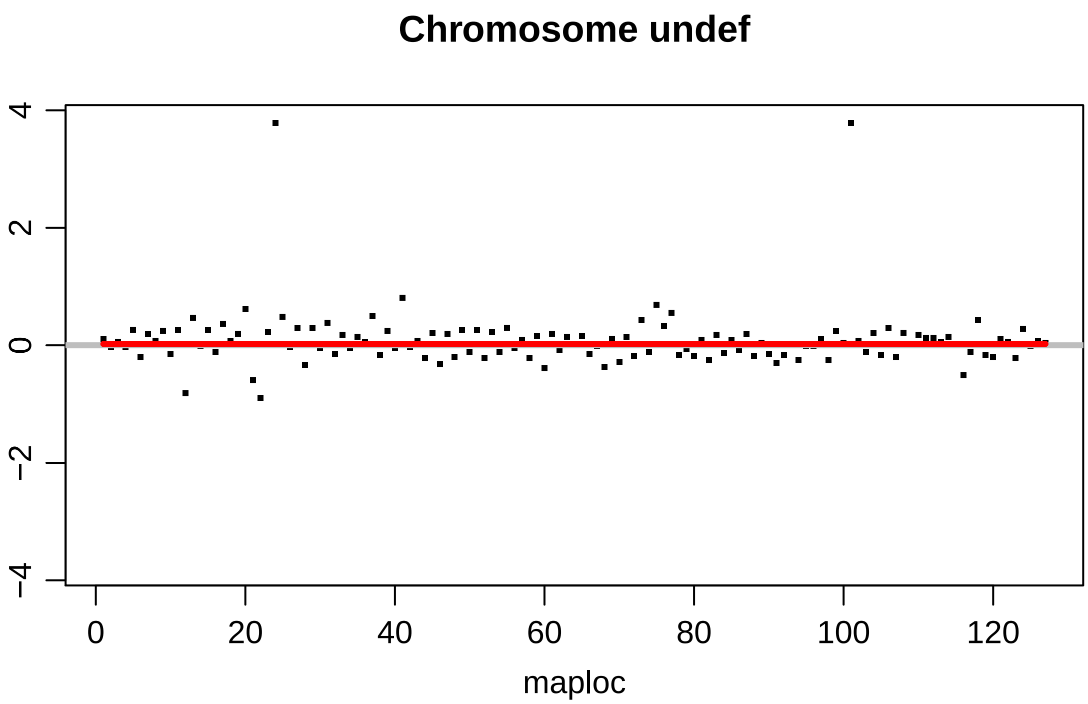


```
## Segplot might not work because of special characters in the sample names. Use only A-Z,a-z and 0-9!
##  There is a hidden function cn.mops:::.replaceNames that replaces the names in the "CNVDetectionResul
```

press_005_R_2012_09_03_22_20_58_Sequoia_SN1.18.withoutCYP_32_Run_15_hg

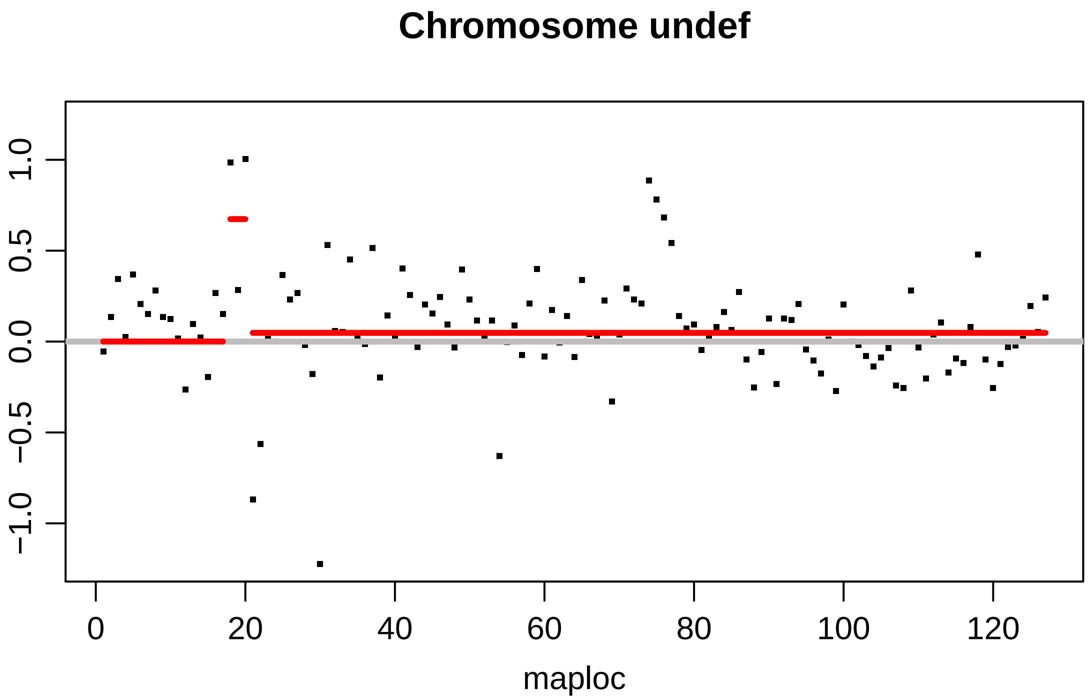


```
## Segplot might not work because of special characters in the sample names. Use only A-Z,a-z and 0-9!
##  There is a hidden function cn.mops:::.replaceNames that replaces the names in the "CNVDetectionResu
```

press_006_R_2012_09_03_22_20_58_Sequoia_SN1.18.withoutCYP_32_Run_15_hg

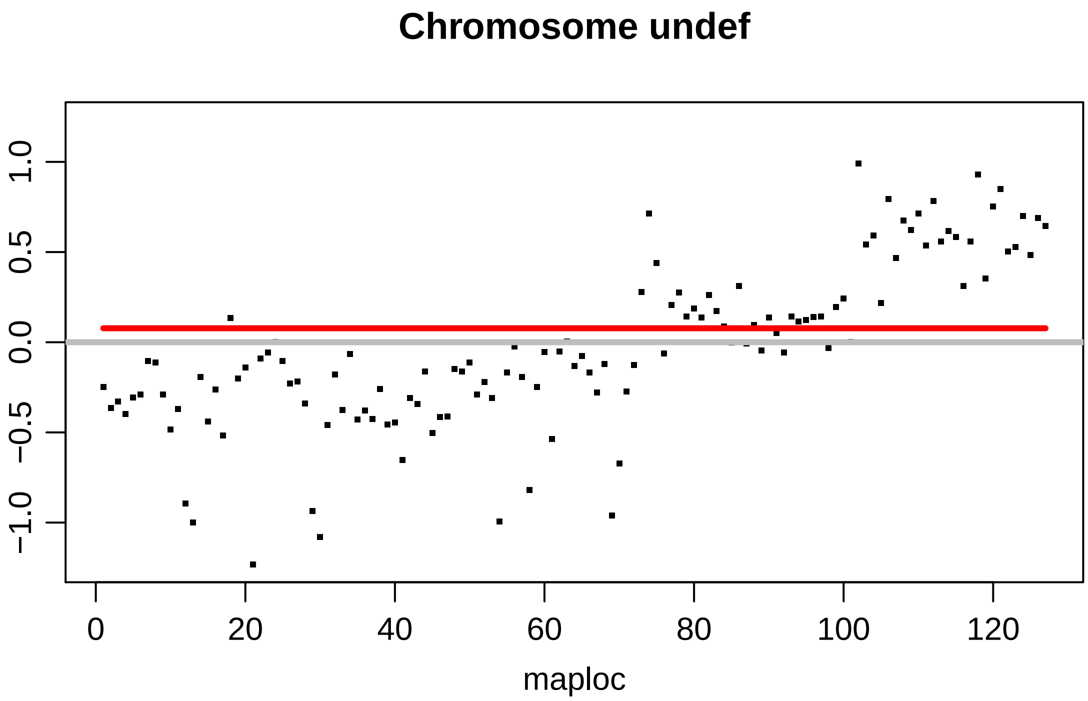

```
## Segplot might not work because of special characters in the sample names. Use only A-Z,a-z and 0-9!
##  There is a hidden function cn.mops:::.replaceNames that replaces the names in the "CNVDetectionResul
```

press_007_R_2012_09_03_22_20_58_Sequoia_SN1.18.withoutCYP_32_Run_15_hg

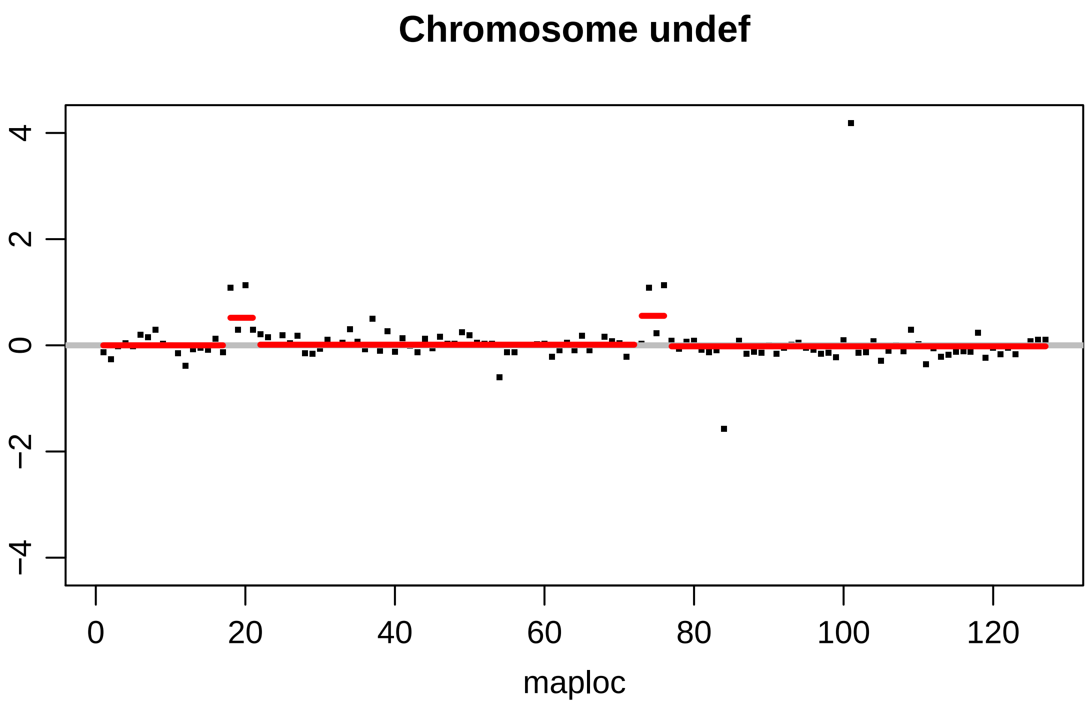


```
## Segplot might not work because of special characters in the sample names. Use only A-Z,a-z and 0-9!
##  There is a hidden function cn.mops:::.replaceNames that replaces the names in the "CNVDetectionResul
```

press_012_R_2012_09_03_22_20_58_Sequoia_SN1.18.withoutCYP_32_Run_15_hg

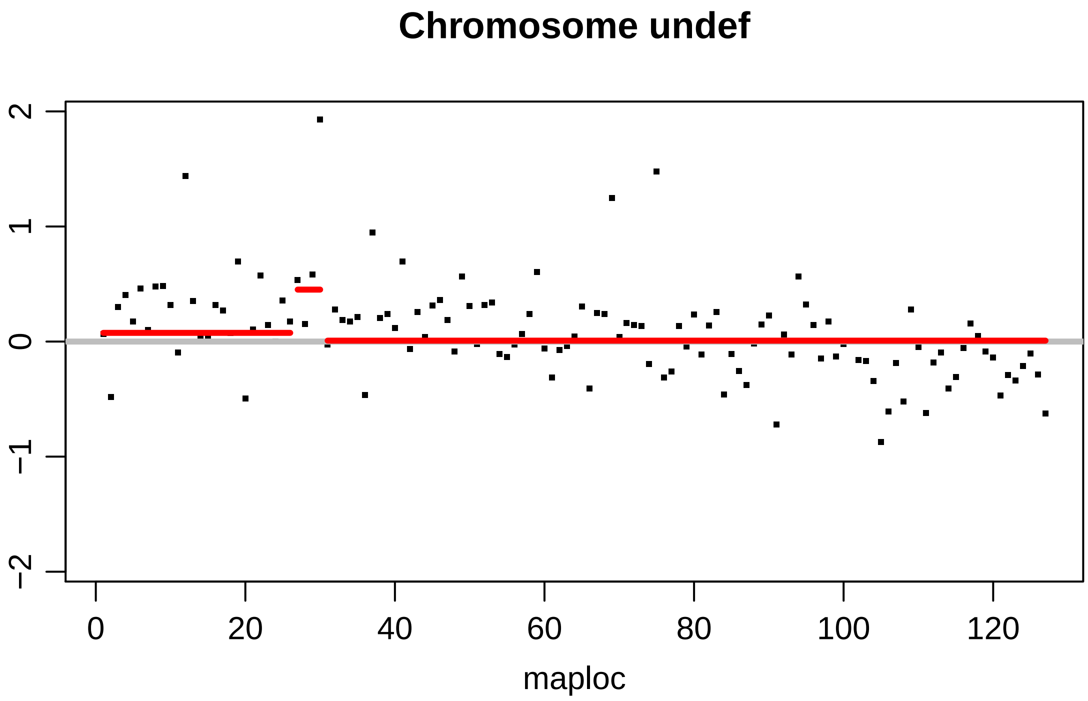


```
## Segplot might not work because of special characters in the sample names. Use only A-Z,a-z and 0-9!
##  There is a hidden function cn.mops:::.replaceNames that replaces the names in the "CNVDetectionResul
```

press_013_R_2012_09_03_22_20_58_Sequoia_SN1.18.withoutCYP_32_Run_15_hg

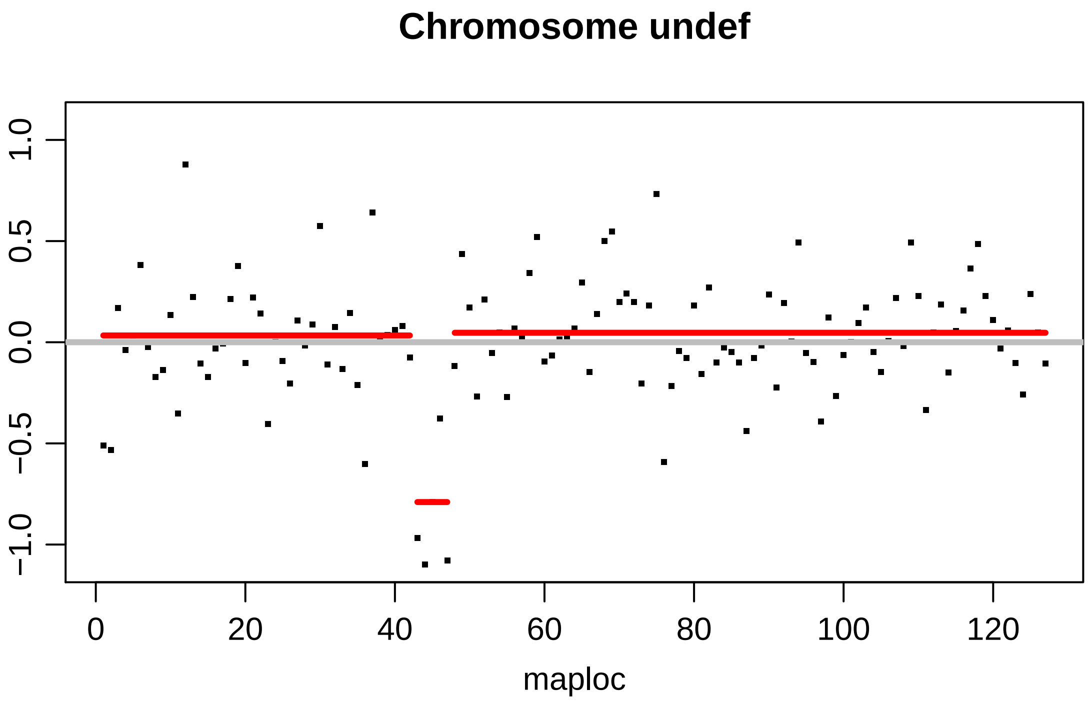

```
## Segplot might not work because of special characters in the sample names. Use only A-Z,a-z and 0-9!
##  There is a hidden function cn.mops:::.replaceNames that replaces the names in the "CNVDetectionResul
```

press_014_R_2012_09_03_22_20_58_Sequoia_SN1.18.withoutCYP_32_Run_15_hg

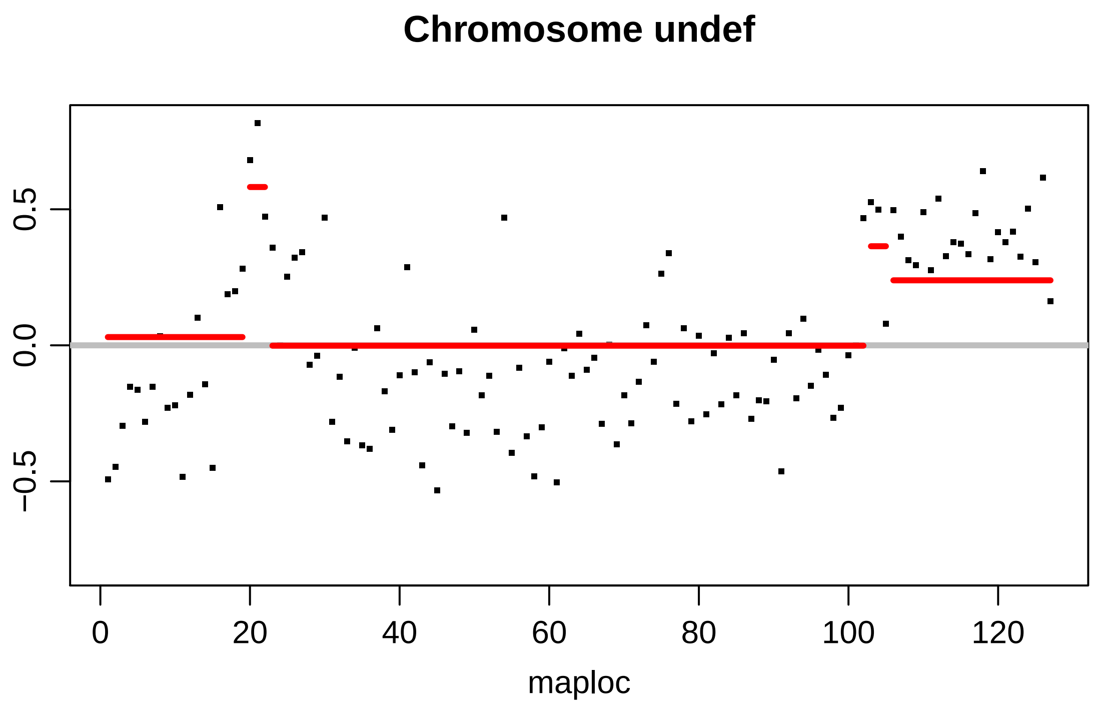


```
## Segplot might not work because of special characters in the sample names. Use only A-Z,a-z and 0-9!
##  There is a hidden function cn.mops:::.replaceNames that replaces the names in the "CNVDetectionResul
```

press_015_R_2012_09_03_22_20_58_Sequoia_SN1.18.withoutCYP_32_Run_15_hg

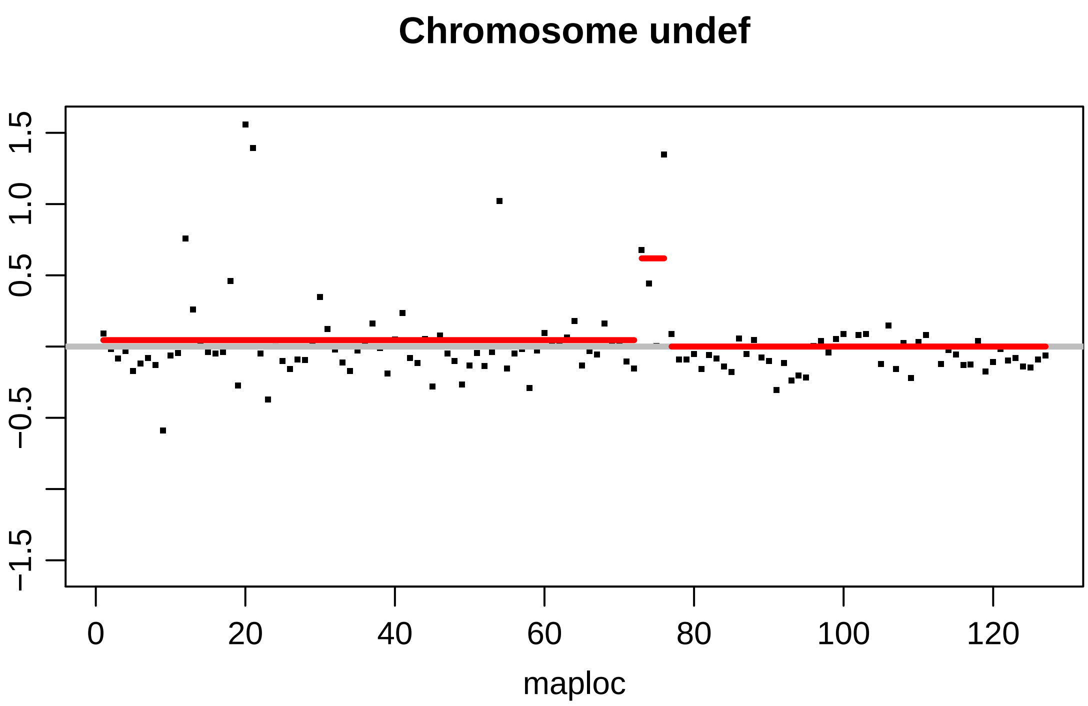


```
## Segplot might not work because of special characters in the sample names. Use only A-Z,a-z and 0-9!
##  There is a hidden function cn.mops:::.replaceNames that replaces the names in the "CNVDetectionResu
```

press_016_R_2012_09_03_22_20_58_Sequoia_SN1.18.withoutCYP_32_Run_15_hg

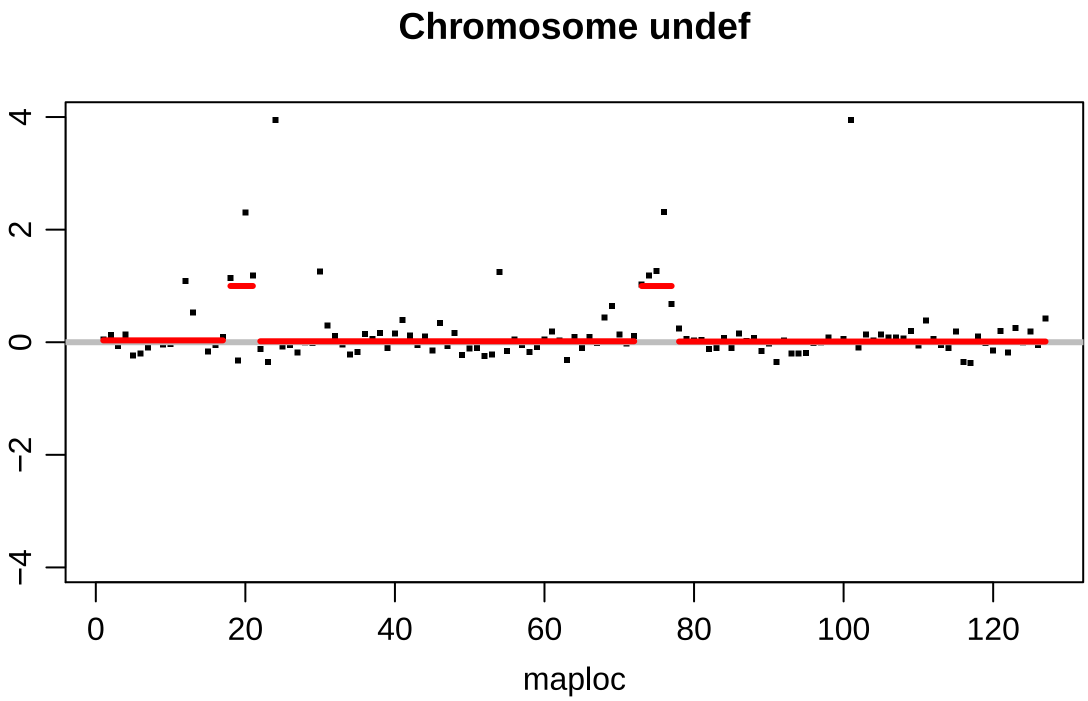

```
## Segplot might not work because of special characters in the sample names. Use only A-Z,a-z and 0-9!
##  There is a hidden function cn.mops:::.replaceNames that replaces the names in the "CNVDetectionResul
```

press_017_R_2012_09_03_22_20_58_Sequoia_SN1.18.withoutCYP_32_Run_15_hg

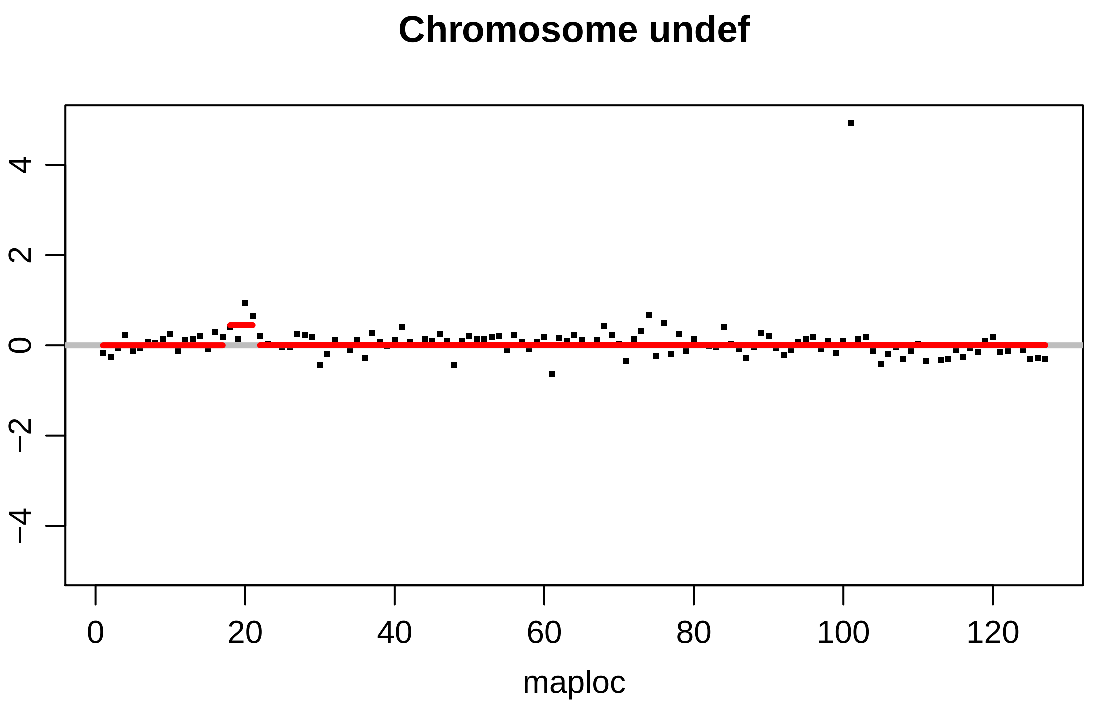


```
## Segplot might not work because of special characters in the sample names. Use only A-Z,a-z and 0-9!
##  There is a hidden function cn.mops:::.replaceNames that replaces the names in the "CNVDetectionResul
```

press_018_R_2012_09_03_22_20_58_Sequoia_SN1.18.withoutCYP_32_Run_15_hg

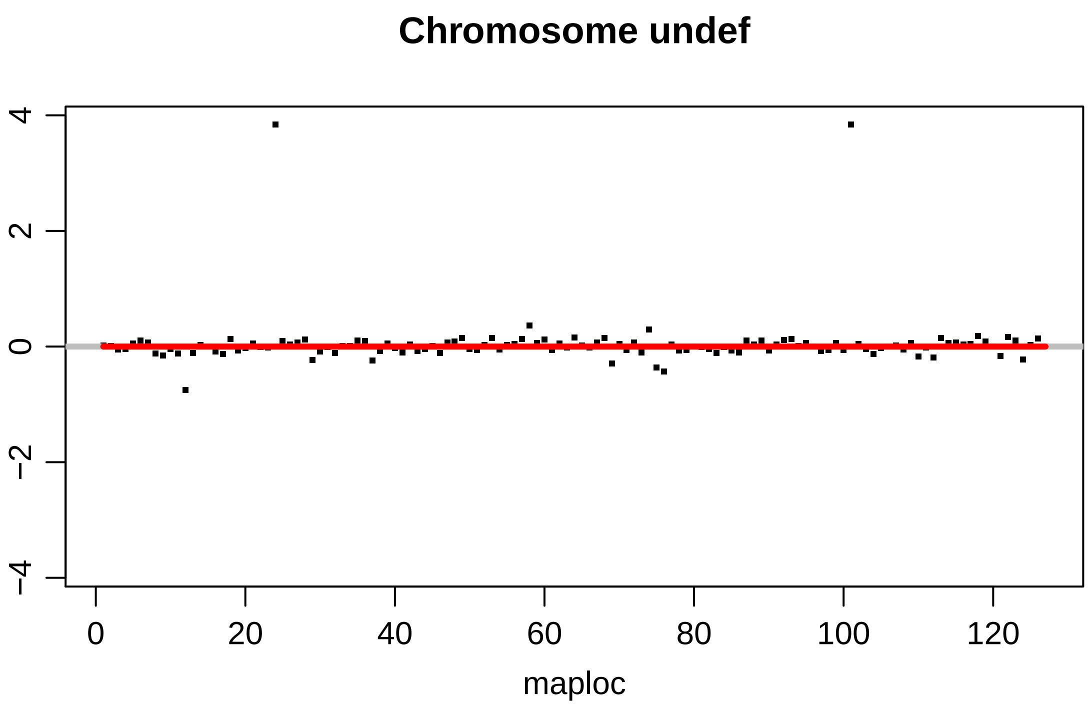


```
## Segplot might not work because of special characters in the sample names. Use only A-Z,a-z and 0-9!
##  There is a hidden function cn.mops:::.replaceNames that replaces the names in the "CNVDetectionResul
```

press_019_R_2012_09_03_22_20_58_Sequoia_SN1.18.withoutCYP_32_Run_15_hg

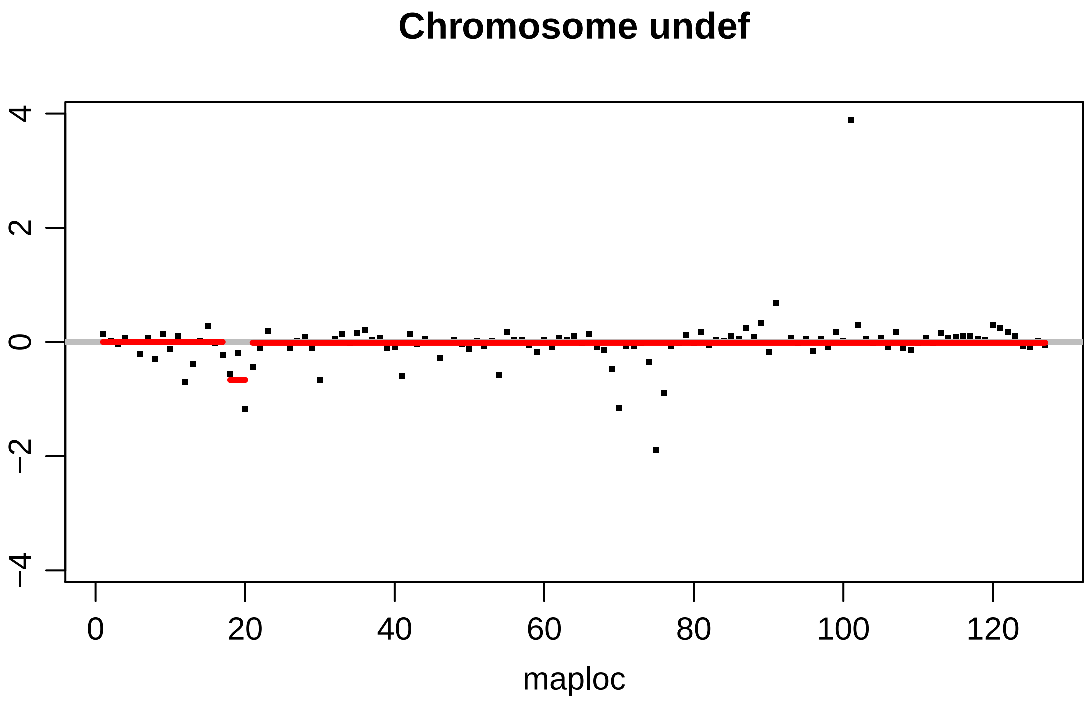

```
## Segplot might not work because of special characters in the sample names. Use only A-Z,a-z and 0-9!
##  There is a hidden function cn.mops:::.replaceNames that replaces the names in the "CNVDetectionResul
```

press_020_R_2012_09_03_22_20_58_Sequoia_SN1.18.withoutCYP_32_Run_15_hg

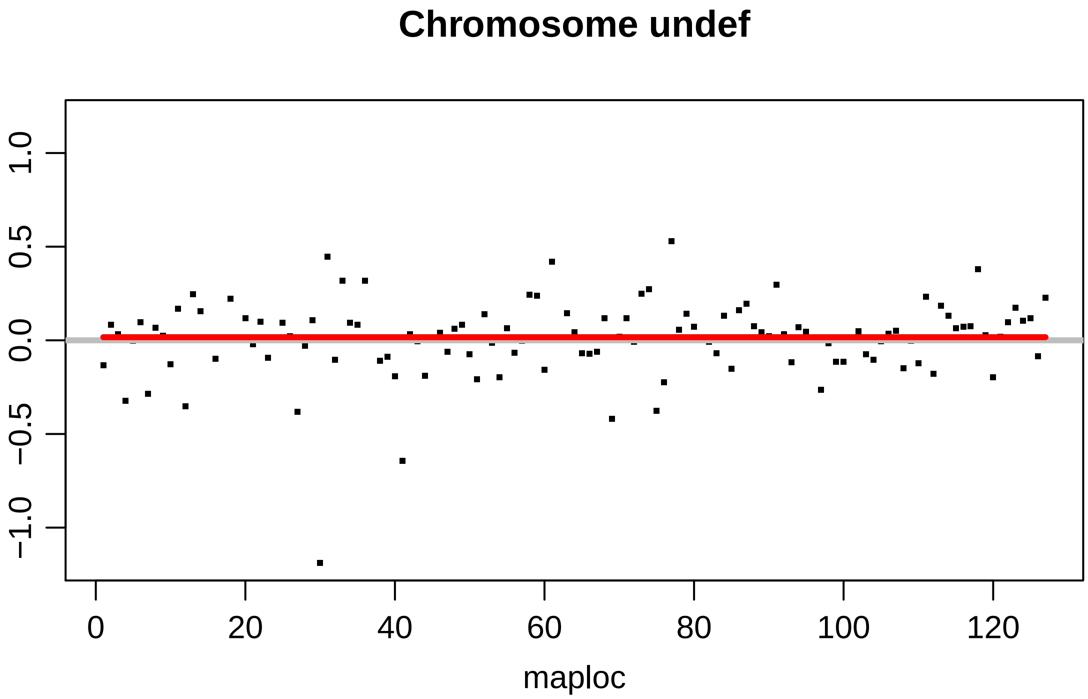


```
## Segplot might not work because of special characters in the sample names. Use only A-Z,a-z and 0-9!
##  There is a hidden function cn.mops:::.replaceNames that replaces the names in the "CNVDetectionResul
```

press_021_R_2012_09_03_22_20_58_Sequoia_SN1.18.withoutCYP_32_Run_15_hg

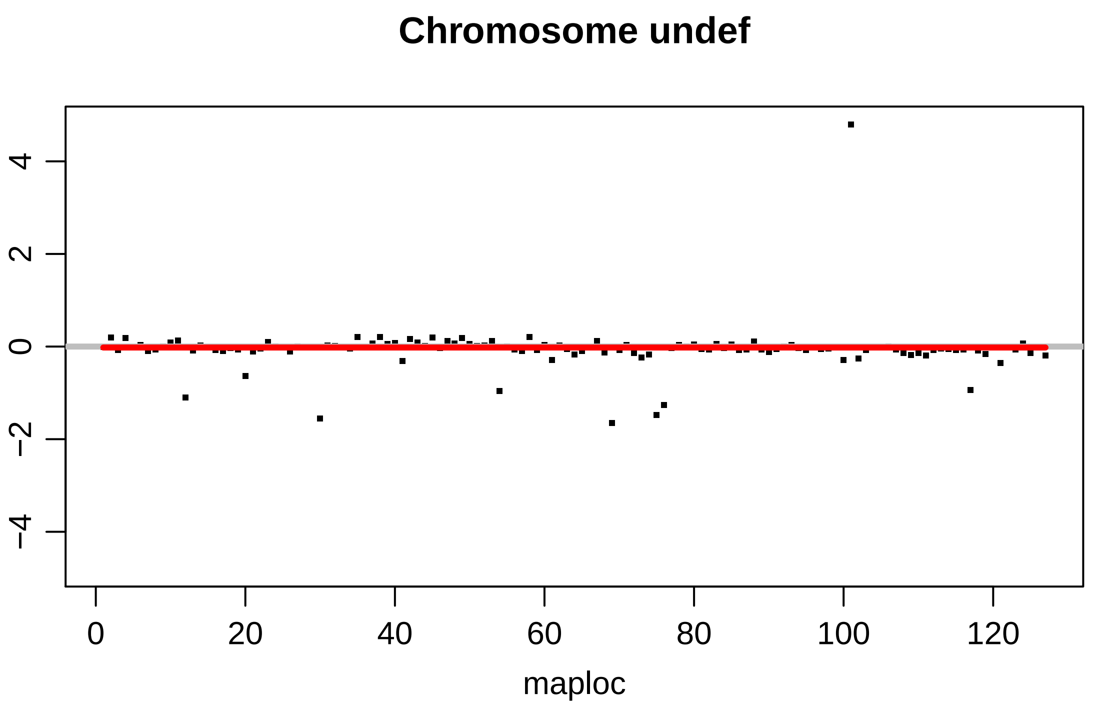


```
## Segplot might not work because of special characters in the sample names. Use only A-Z,a-z and 0-9!
##  There is a hidden function cn.mops:::.replaceNames that replaces the names in the "CNVDetectionResul
```

press_022_R_2012_09_03_22_20_58_Sequoia_SN1.18.withoutCYP_32_Run_15_hg

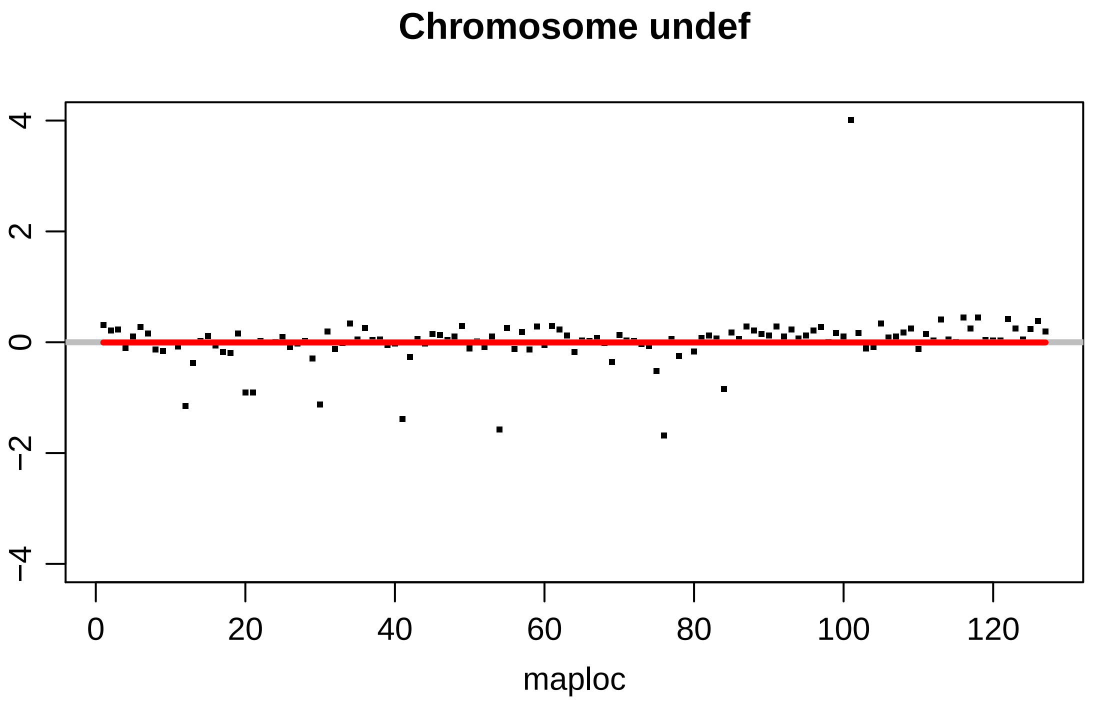

```
## Segplot might not work because of special characters in the sample names. Use only A-Z,a-z and 0-9!
##  There is a hidden function cn.mops:::.replaceNames that replaces the names in the "CNVDetectionResul
```

press_023_R_2012_09_03_22_20_58_Sequoia_SN1.18.withoutCYP_32_Run_15_hg

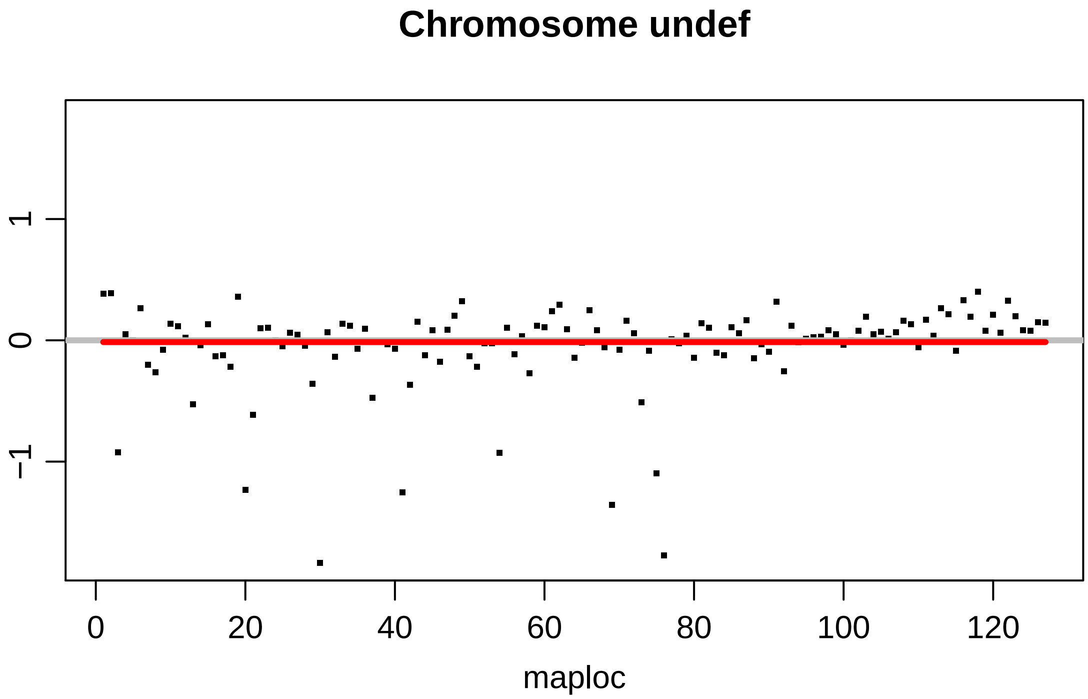


```
## Segplot might not work because of special characters in the sample names. Use only A-Z,a-z and 0-9!
##  There is a hidden function cn.mops:::.replaceNames that replaces the names in the "CNVDetectionResul
```

press_024_R_2012_09_03_22_20_58_Sequoia_SN1.18.withoutCYP_32_Run_15_hg

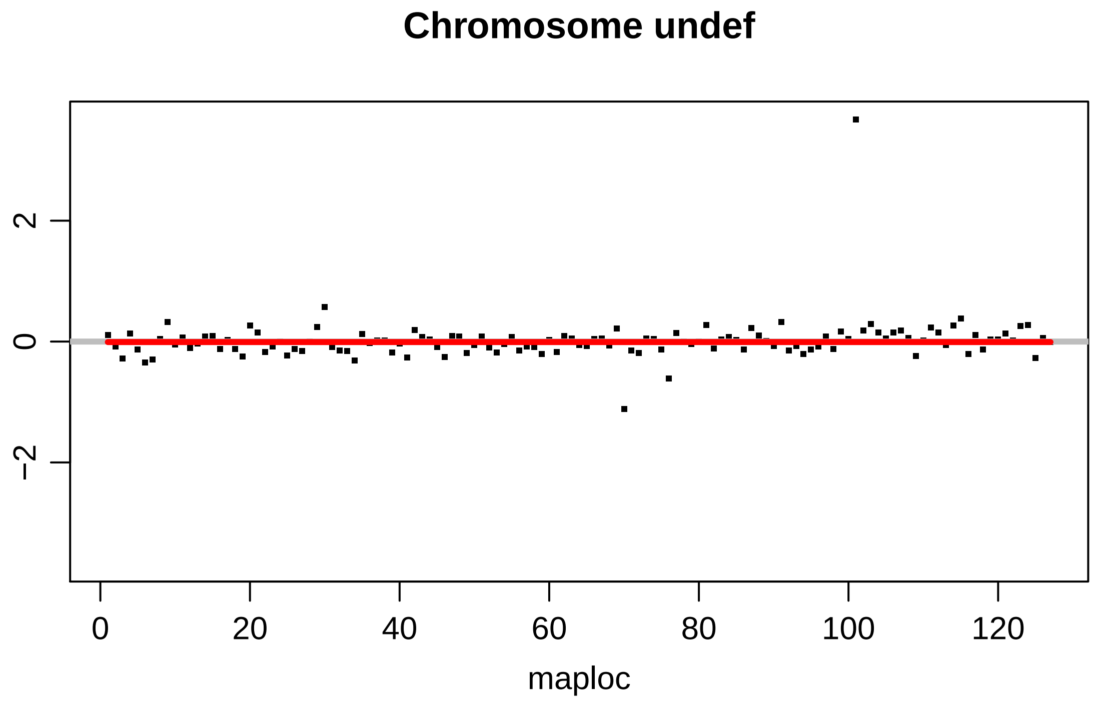


```
## Segplot might not work because of special characters in the sample names. Use only A-Z,a-z and 0-9!
##  There is a hidden function cn.mops:::.replaceNames that replaces the names in the "CNVDetectionResul
```

press_025_R_2012_09_03_22_20_58_Sequoia_SN1.18.withoutCYP_32_Run_15_hg

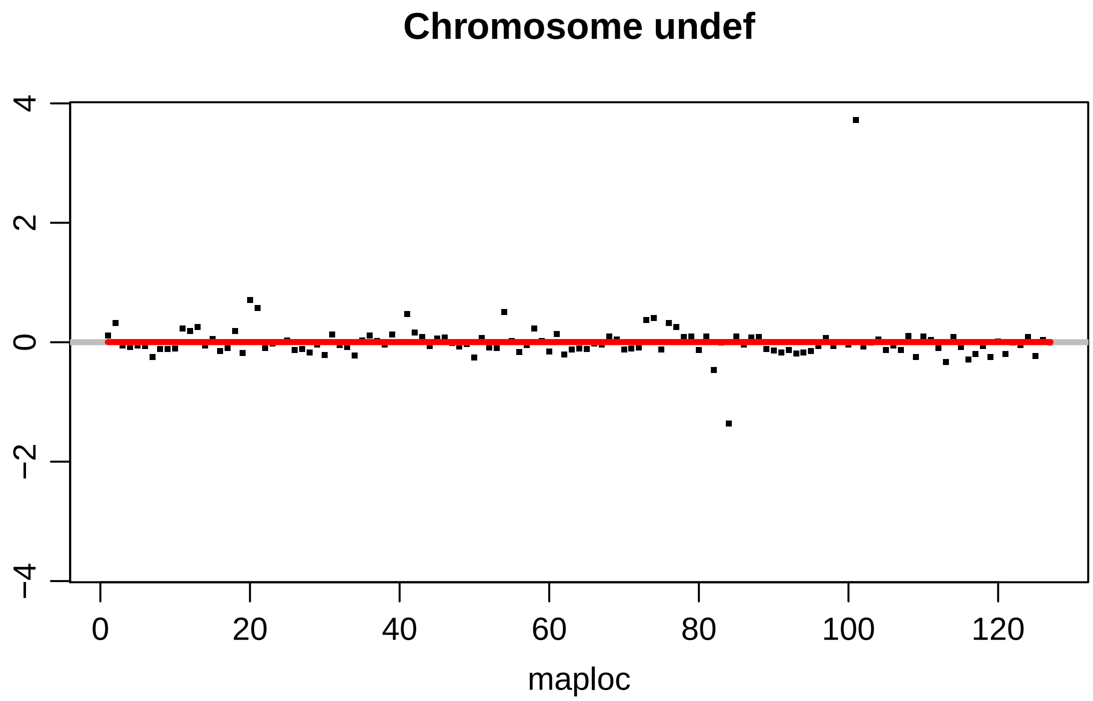

```
## Segplot might not work because of special characters in the sample names. Use only A-Z,a-z and 0-9!
##  There is a hidden function cn.mops:::.replaceNames that replaces the names in the "CNVDetectionResul
```

press_026_R_2012_09_03_22_20_58_Sequoia_SN1.18.withoutCYP_32_Run_15_hg

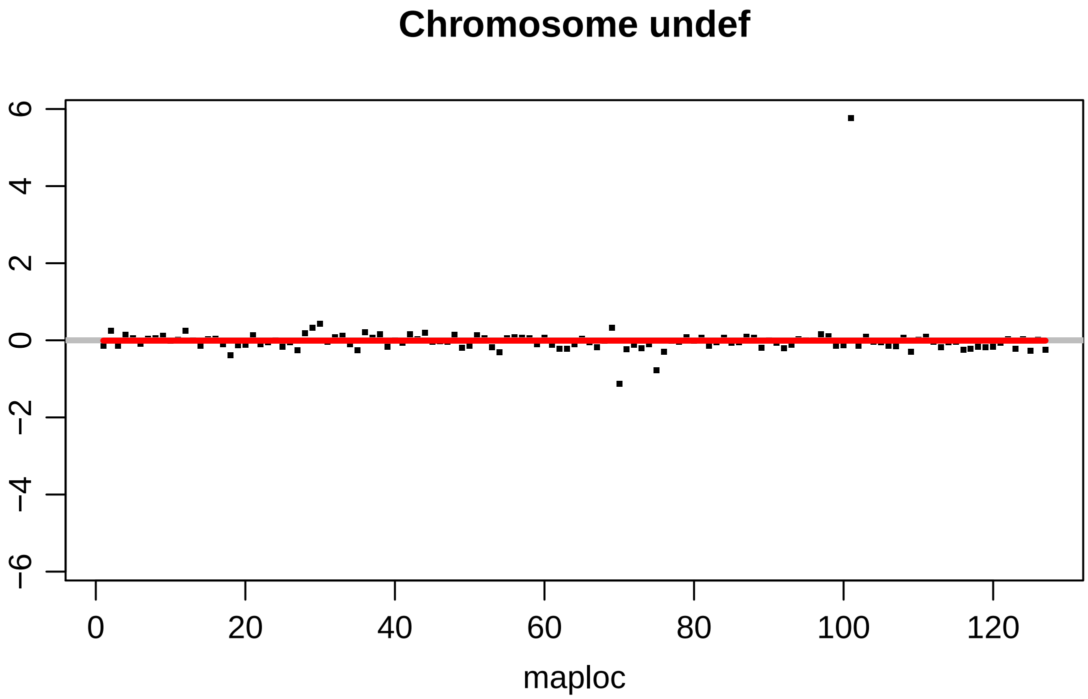


```
## Segplot might not work because of special characters in the sample names. Use only A-Z,a-z and 0-9!
##  There is a hidden function cn.mops:::.replaceNames that replaces the names in the "CNVDetectionResul
```

press_027_R_2012_09_03_22_20_58_Sequoia_SN1.18.withoutCYP_32_Run_15_hg

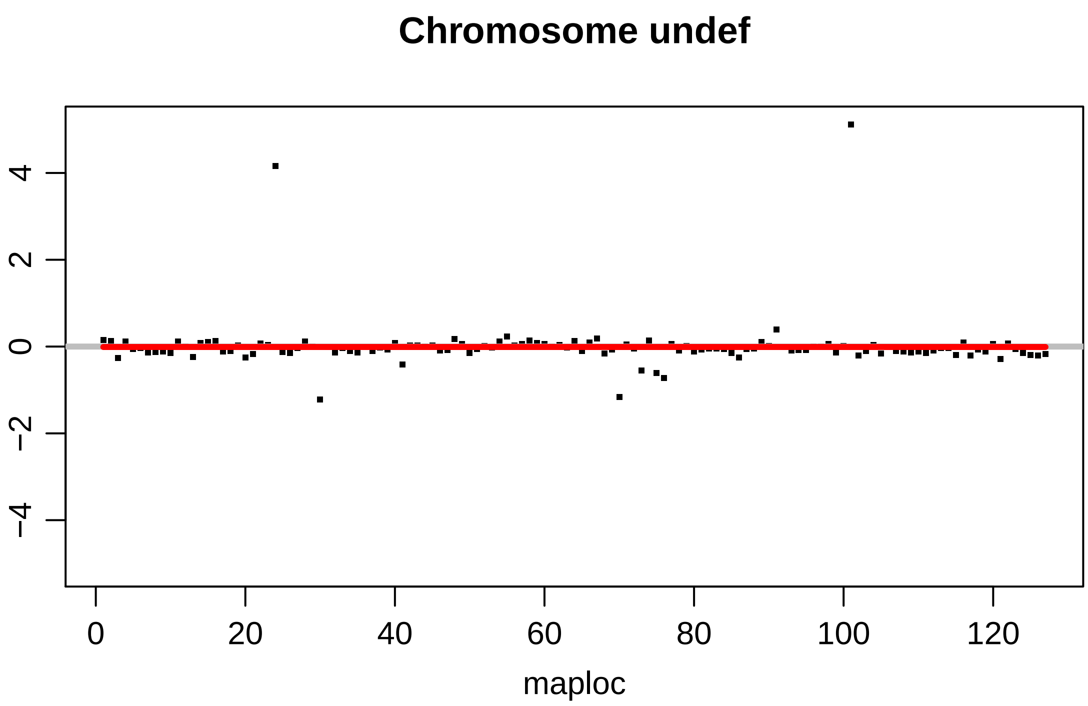


```
## Segplot might not work because of special characters in the sample names. Use only A-Z,a-z and 0-9!
##  There is a hidden function cn.mops:::.replaceNames that replaces the names in the "CNVDetectionResul
```

press_028_R_2012_09_03_22_20_58_Sequoia_SN1.18.withoutCYP_32_Run_15_hg

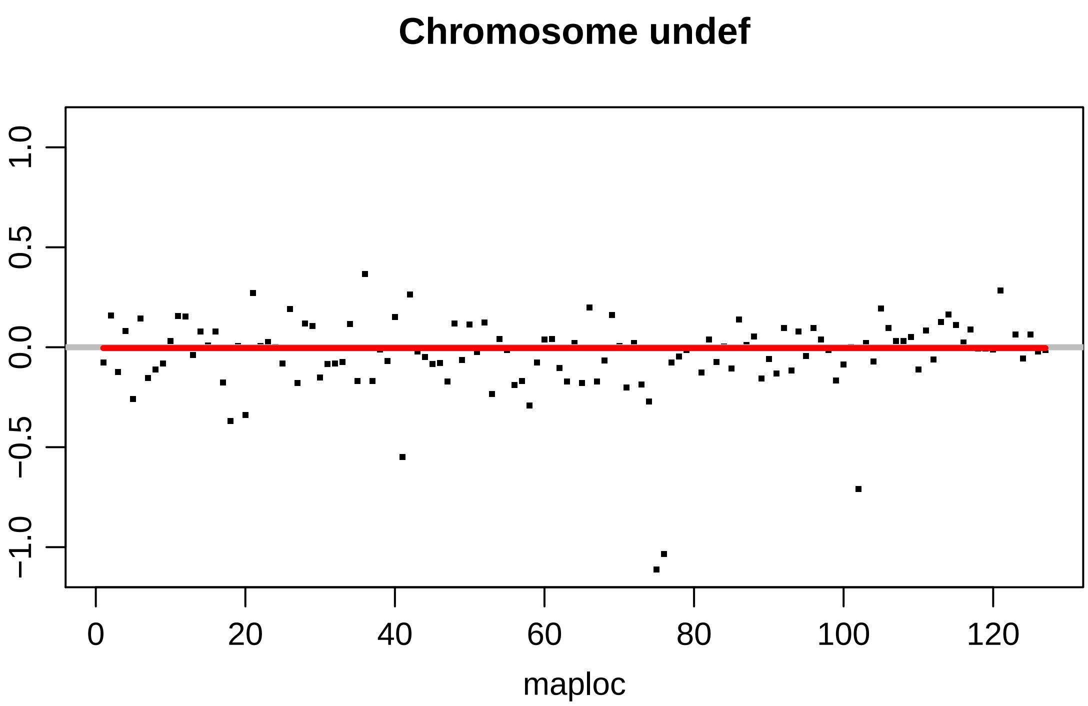

```
## Segplot might not work because of special characters in the sample names. Use only A-Z,a-z and 0-9!
##  There is a hidden function cn.mops:::.replaceNames that replaces the names in the "CNVDetectionResul
```

press_029_R_2012_09_03_22_20_58_Sequoia_SN1.18.withoutCYP_32_Run_15_hg

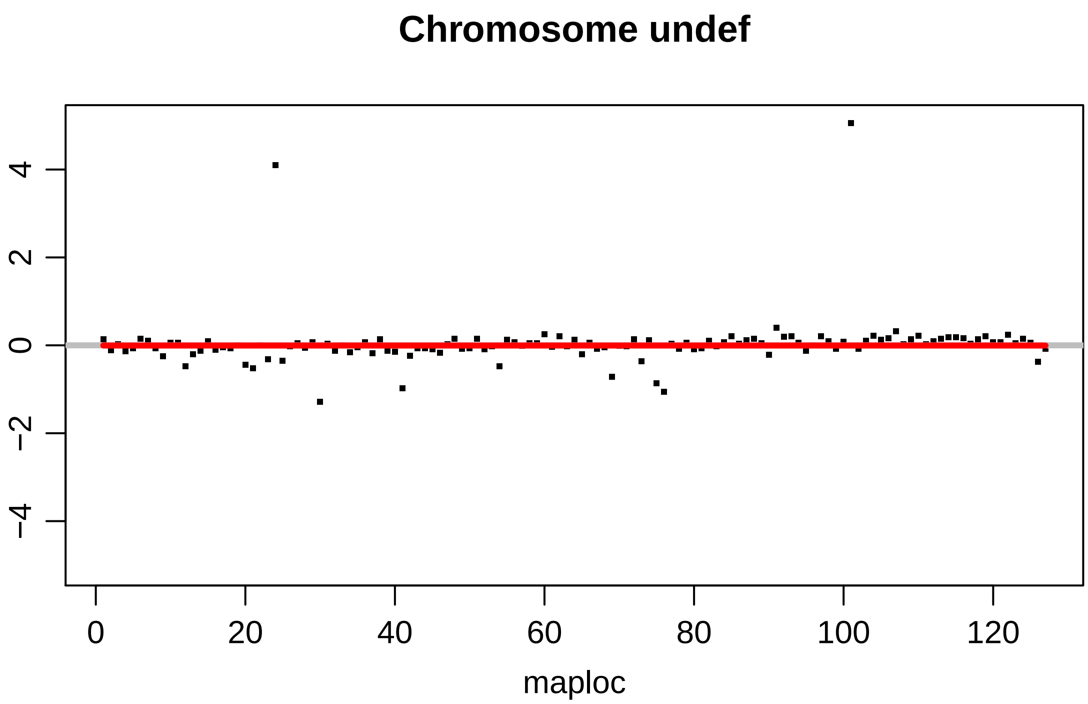


```
## Segplot might not work because of special characters in the sample names. Use only A-Z,a-z and 0-9!
##  There is a hidden function cn.mops:::.replaceNames that replaces the names in the "CNVDetectionResul
```

press_030_R_2012_09_03_22_20_58_Sequoia_SN1.18.withoutCYP_32_Run_15_hg

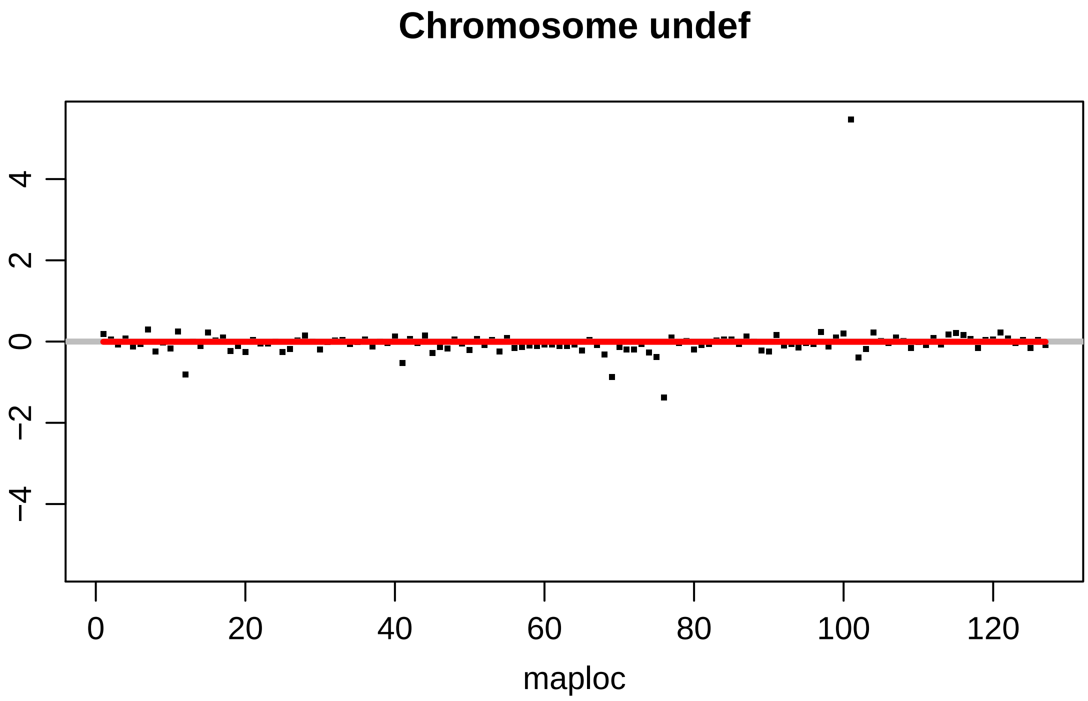


```
## Segplot might not work because of special characters in the sample names. Use only A-Z,a-z and 0-9!
##  There is a hidden function cn.mops:::.replaceNames that replaces the names in the "CNVDetectionResul
```

press_031_R_2012_09_03_22_20_58_Sequoia_SN1.18.withoutCYP_32_Run_15_hg

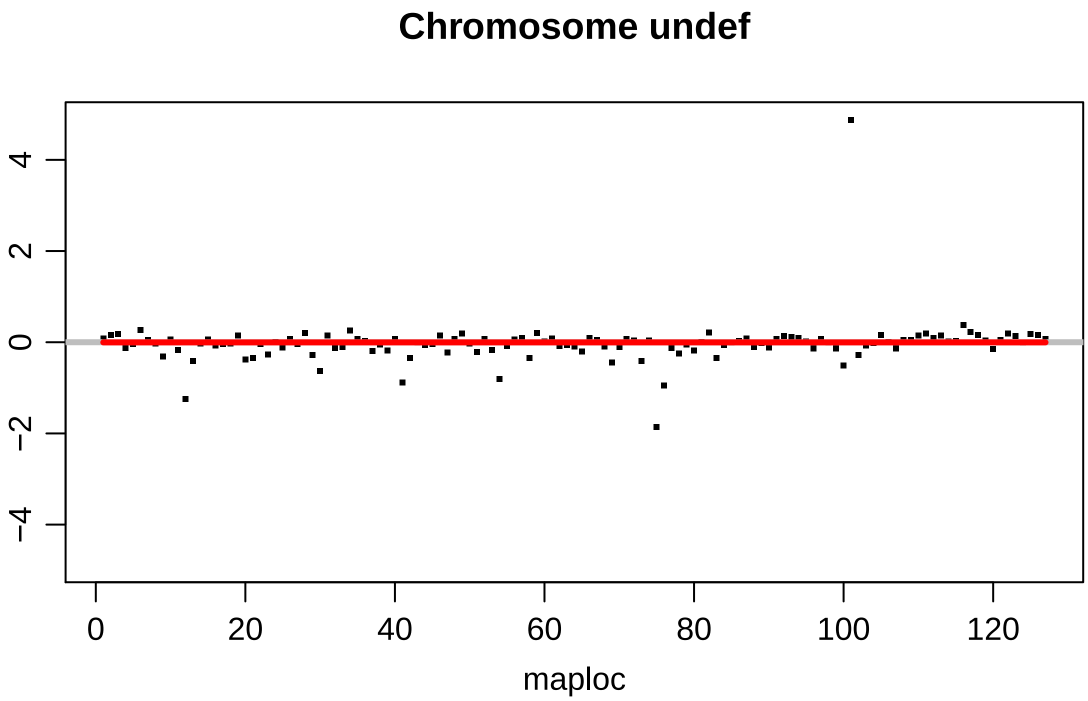

```
##
## CNV regions:
## GRanges object with 2 ranges and 27 metadata columns:
##       seqnames    ranges strand |
##          <Rle> <IRanges>  <Rle> |
##   [1]    undef  [18, 22]      * |
##   [2]    undef  [73, 77]      * |
##       Case_IonXpress_002_R_2012_09_03_22_20_58_Sequoia_SN1.18.withoutCYP_32_Run_15_hg19_v3.sam
##                                                                                      <factor>
##   [1]                                                                                     CN2
##   [2]                                                                                     CN2
##       Case_IonXpress_003_R_2012_09_03_22_20_58_Sequoia_SN1.18.withoutCYP_32_Run_15_hg19_v3.sam
##                                                                                      <factor>
##   [1]                                                                                     CN2
##   [2]                                                                                     CN2
##       Case_IonXpress_004_R_2012_09_03_22_20_58_Sequoia_SN1.18.withoutCYP_32_Run_15_hg19_v3.sam
##                                                                                      <factor>
##   [1]                                                                                     CN2
##   [2]                                                                                     CN2
##       Case_IonXpress_005_R_2012_09_03_22_20_58_Sequoia_SN1.18.withoutCYP_32_Run_15_hg19_v3.sam
##                                                                                      <factor>
##   [1]                                                                                     CN3
##   [2]                                                                                     CN2
##       Case_IonXpress_006_R_2012_09_03_22_20_58_Sequoia_SN1.18.withoutCYP_32_Run_15_hg19_v3.sam
##                                                                                      <factor>
##   [1]                                                                                     CN2
##   [2]                                                                                     CN2
##       Case_IonXpress_007_R_2012_09_03_22_20_58_Sequoia_SN1.18.withoutCYP_32_Run_15_hg19_v3.sam
##                                                                                      <factor>
##   [1]                                                                                     CN3
##   [2]                                                                                     CN4
##       Case_IonXpress_012_R_2012_09_03_22_20_58_Sequoia_SN1.18.withoutCYP_32_Run_15_hg19_v3.sam
##                                                                                      <factor>
##   [1]                                                                                     CN2
##   [2]                                                                                     CN2
##       Case_IonXpress_013_R_2012_09_03_22_20_58_Sequoia_SN1.18.withoutCYP_32_Run_15_hg19_v3.sam
##                                                                                      <factor>
##   [1]                                                                                     CN2
##   [2]                                                                                     CN2
##       Case_IonXpress_014_R_2012_09_03_22_20_58_Sequoia_SN1.18.withoutCYP_32_Run_15_hg19_v3.sam
##                                                                                      <factor>
##   [1]                                                                                     CN4
##   [2]                                                                                     CN2
##       Case_IonXpress_015_R_2012_09_03_22_20_58_Sequoia_SN1.18.withoutCYP_32_Run_15_hg19_v3.sam
##                                                                                      <factor>
##   [1]                                                                                     CN2
##   [2]                                                                                     CN3
##       Case_IonXpress_016_R_2012_09_03_22_20_58_Sequoia_SN1.18.withoutCYP_32_Run_15_hg19_v3.sam
##                                                                                      <factor>
##   [1]                                                                                     CN4
##   [2]                                                                                     CN4
##       Case_IonXpress_017_R_2012_09_03_22_20_58_Sequoia_SN1.18.withoutCYP_32_Run_15_hg19_v3.sam
##                                                                                      <factor>
##   [1]                                                                                     CN2
```

```
##  [2]                                                                    CN2
##     Case_IonXpress_018_R_2012_09_03_22_20_58_Sequoia_SN1.18.withoutCYP_32_Run_15_hg19_v3.sam
##                                                                    <factor>
##  [1]                                                                    CN2
##  [2]                                                                    CN2
##     Case_IonXpress_019_R_2012_09_03_22_20_58_Sequoia_SN1.18.withoutCYP_32_Run_15_hg19_v3.sam
##                                                                    <factor>
##  [1]                                                                    CN2
##  [2]                                                                    CN2
##     Case_IonXpress_020_R_2012_09_03_22_20_58_Sequoia_SN1.18.withoutCYP_32_Run_15_hg19_v3.sam
##                                                                    <factor>
##  [1]                                                                    CN2
##  [2]                                                                    CN2
##     Case_IonXpress_021_R_2012_09_03_22_20_58_Sequoia_SN1.18.withoutCYP_32_Run_15_hg19_v3.sam
##                                                                    <factor>
##  [1]                                                                    CN2
##  [2]                                                                    CN2
##     Case_IonXpress_022_R_2012_09_03_22_20_58_Sequoia_SN1.18.withoutCYP_32_Run_15_hg19_v3.sam
##                                                                    <factor>
##  [1]                                                                    CN2
##  [2]                                                                    CN2
##     Case_IonXpress_023_R_2012_09_03_22_20_58_Sequoia_SN1.18.withoutCYP_32_Run_15_hg19_v3.sam
##                                                                    <factor>
##  [1]                                                                    CN2
##  [2]                                                                    CN2
##     Case_IonXpress_024_R_2012_09_03_22_20_58_Sequoia_SN1.18.withoutCYP_32_Run_15_hg19_v3.sam
##                                                                    <factor>
##  [1]                                                                    CN2
##  [2]                                                                    CN2
##     Case_IonXpress_025_R_2012_09_03_22_20_58_Sequoia_SN1.18.withoutCYP_32_Run_15_hg19_v3.sam
##                                                                    <factor>
##  [1]                                                                    CN2
##  [2]                                                                    CN2
##     Case_IonXpress_026_R_2012_09_03_22_20_58_Sequoia_SN1.18.withoutCYP_32_Run_15_hg19_v3.sam
##                                                                    <factor>
##  [1]                                                                    CN2
##  [2]                                                                    CN2
##     Case_IonXpress_027_R_2012_09_03_22_20_58_Sequoia_SN1.18.withoutCYP_32_Run_15_hg19_v3.sam
##                                                                    <factor>
##  [1]                                                                    CN2
##  [2]                                                                    CN2
##     Case_IonXpress_028_R_2012_09_03_22_20_58_Sequoia_SN1.18.withoutCYP_32_Run_15_hg19_v3.sam
##                                                                    <factor>
##  [1]                                                                    CN2
##  [2]                                                                    CN2
##     Case_IonXpress_029_R_2012_09_03_22_20_58_Sequoia_SN1.18.withoutCYP_32_Run_15_hg19_v3.sam
##                                                                    <factor>
##  [1]                                                                    CN2
##  [2]                                                                    CN2
##     Case_IonXpress_030_R_2012_09_03_22_20_58_Sequoia_SN1.18.withoutCYP_32_Run_15_hg19_v3.sam
##                                                                    <factor>
##  [1]                                                                    CN2
##  [2]                                                                    CN2
##     Case_IonXpress_031_R_2012_09_03_22_20_58_Sequoia_SN1.18.withoutCYP_32_Run_15_hg19_v3.sam
```

```
##                                                                                  <factor>
##   [1]                                                                                 CN2
##   [2]                                                                                 CN2
##       Case_IonXpress_032_R_2012_09_03_22_20_58_Sequoia_SN1.18.withoutCYP_32_Run_15_hg19_v3.sam
##                                                                                  <factor>
##   [1]                                                                                 CN2
##   [2]                                                                                 CN2
##   -------
##   seqinfo: 1 sequence from an unspecified genome; no seqlengths
##
## Individual CNVs:
## GRanges object with 7 ranges and 4 metadata columns:
##       seqnames    ranges strand |
##          <Rle> <IRanges>  <Rle> |
##   [1]    undef  [18, 20]      * |
##   [2]    undef  [18, 21]      * |
##   [3]    undef  [73, 76]      * |
##   [4]    undef  [20, 22]      * |
##   [5]    undef  [73, 76]      * |
##   [6]    undef  [18, 21]      * |
##   [7]    undef  [73, 77]      * |
##                                                                                sampleName
##                                                                                  <factor>
##   [1] Case_IonXpress_005_R_2012_09_03_22_20_58_Sequoia_SN1.18.withoutCYP_32_Run_15_hg19_v3.sam
##   [2] Case_IonXpress_007_R_2012_09_03_22_20_58_Sequoia_SN1.18.withoutCYP_32_Run_15_hg19_v3.sam
##   [3] Case_IonXpress_007_R_2012_09_03_22_20_58_Sequoia_SN1.18.withoutCYP_32_Run_15_hg19_v3.sam
##   [4] Case_IonXpress_014_R_2012_09_03_22_20_58_Sequoia_SN1.18.withoutCYP_32_Run_15_hg19_v3.sam
##   [5] Case_IonXpress_015_R_2012_09_03_22_20_58_Sequoia_SN1.18.withoutCYP_32_Run_15_hg19_v3.sam
##   [6] Case_IonXpress_016_R_2012_09_03_22_20_58_Sequoia_SN1.18.withoutCYP_32_Run_15_hg19_v3.sam
##   [7] Case_IonXpress_016_R_2012_09_03_22_20_58_Sequoia_SN1.18.withoutCYP_32_Run_15_hg19_v3.sam
##          median      mean          CN
##       <numeric> <numeric> <character>
##   [1] 1.0000000 0.6736438         CN3
##   [2] 0.5185096 0.5189176         CN3
##   [3] 0.5057875 0.5552463         CN4
##   [4] 0.5933714 0.5818118         CN4
##   [5] 0.5875588 0.6190583         CN3
##   [6] 0.9982188 0.9991073         CN4
##   [7] 0.9760317 0.9984034         CN4
##   -------
##   seqinfo: 1 sequence from an unspecified genome; no seqlengths
## [1] "/Users/gdemidov/Downloads/doc/Run_16_fin_05_qc.xls"

## Normalizing...

## Starting local modeling, please be patient...

## Reference sequence:  undef

## Starting segmentation algorithm...

## Using "fastseg" for segmentation.
```

```
## No CNVs detected. Try changing "normalization", "priorImpact" or "thresholds".
```

```
## [1] ""
## [1] "/Users/gdemidov/Downloads/doc/Run_16_fin_05_qc.xls"
## [1] ""
```

```
## Segplot might not work because of special characters in the sample names. Use only A-Z,a-z and 0-9!
##  There is a hidden function cn.mops:::.replaceNames that replaces the names in the "CNVDetectionResul
```

press_032_R_2012_09_03_22_20_58_Sequoia_SN1.18.withoutCYP_32_Run_15_hg

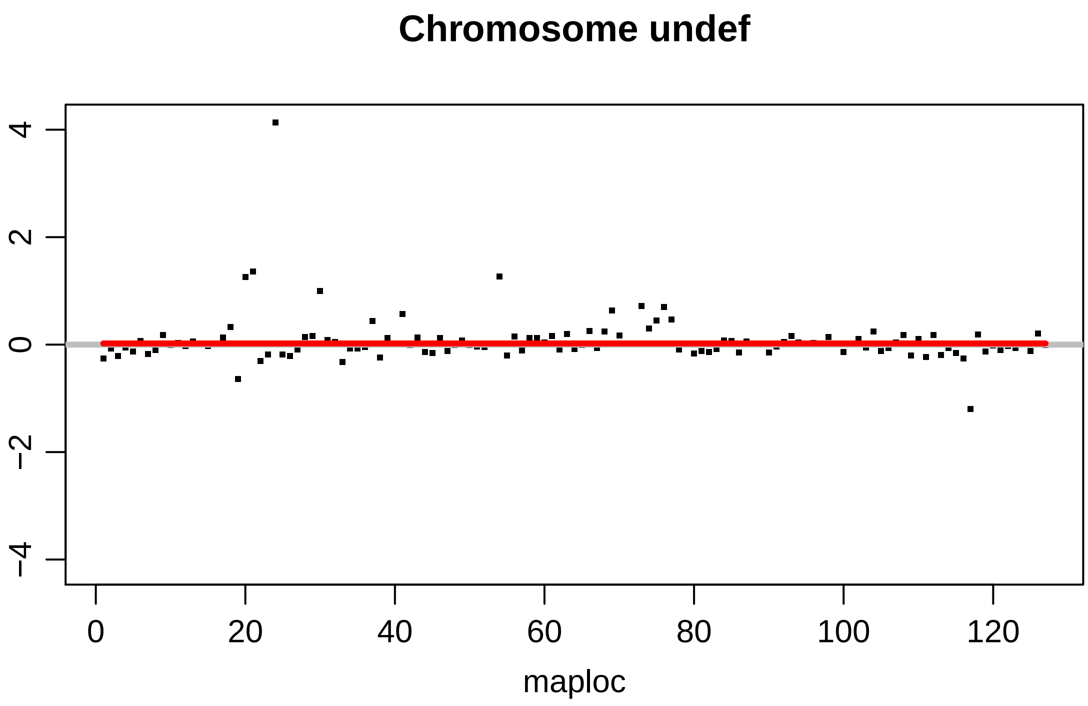


```
## Segplot might not work because of special characters in the sample names. Use only A-Z,a-z and 0-9!
##  There is a hidden function cn.mops:::.replaceNames that replaces the names in the "CNVDetectionResul
```

press_002_R_2012_09_04_02_34_22_Sequoia_SN1.19.withoutCYP_48_Run_16_hg

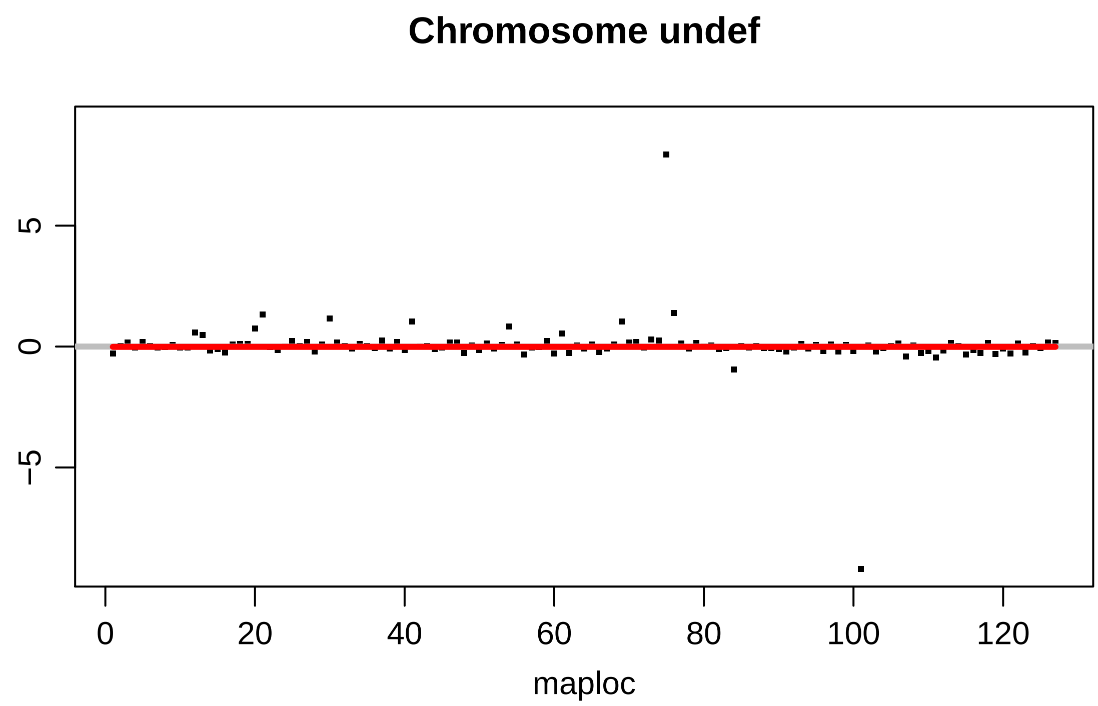


```
## Segplot might not work because of special characters in the sample names. Use only A-Z,a-z and 0-9!
##  There is a hidden function cn.mops:::.replaceNames that replaces the names in the "CNVDetectionResul
```

press_003_R_2012_09_04_02_34_22_Sequoia_SN1.19.withoutCYP_48_Run_16_hg

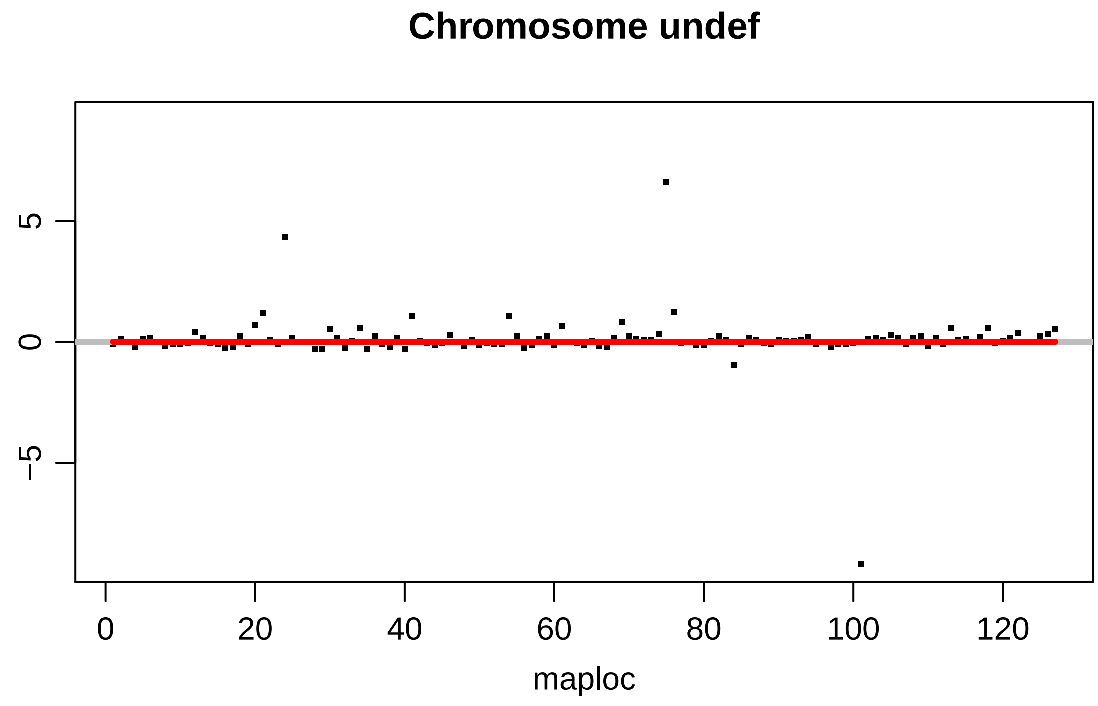

```
## Segplot might not work because of special characters in the sample names. Use only A-Z,a-z and 0-9!
##  There is a hidden function cn.mops:::.replaceNames that replaces the names in the "CNVDetectionResul
```

press_004_R_2012_09_04_02_34_22_Sequoia_SN1.19.withoutCYP_48_Run_16_hg

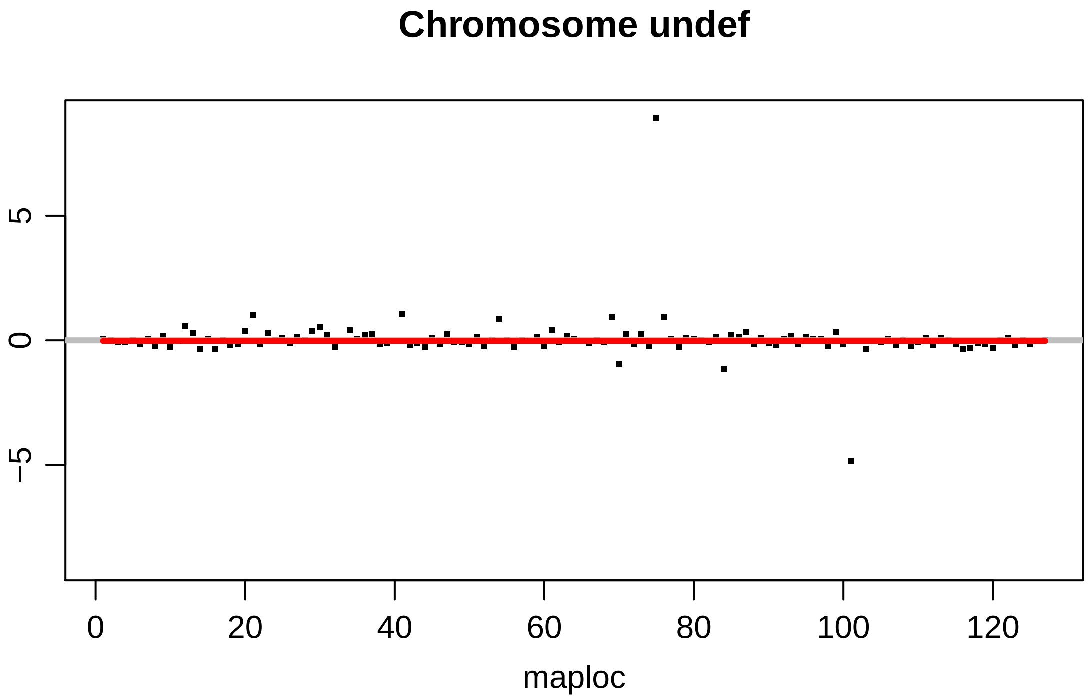


```
## Segplot might not work because of special characters in the sample names. Use only A-Z,a-z and 0-9!
##  There is a hidden function cn.mops:::.replaceNames that replaces the names in the "CNVDetectionResul
```

press_005_R_2012_09_04_02_34_22_Sequoia_SN1.19.withoutCYP_48_Run_16_hg

Chromosome undef

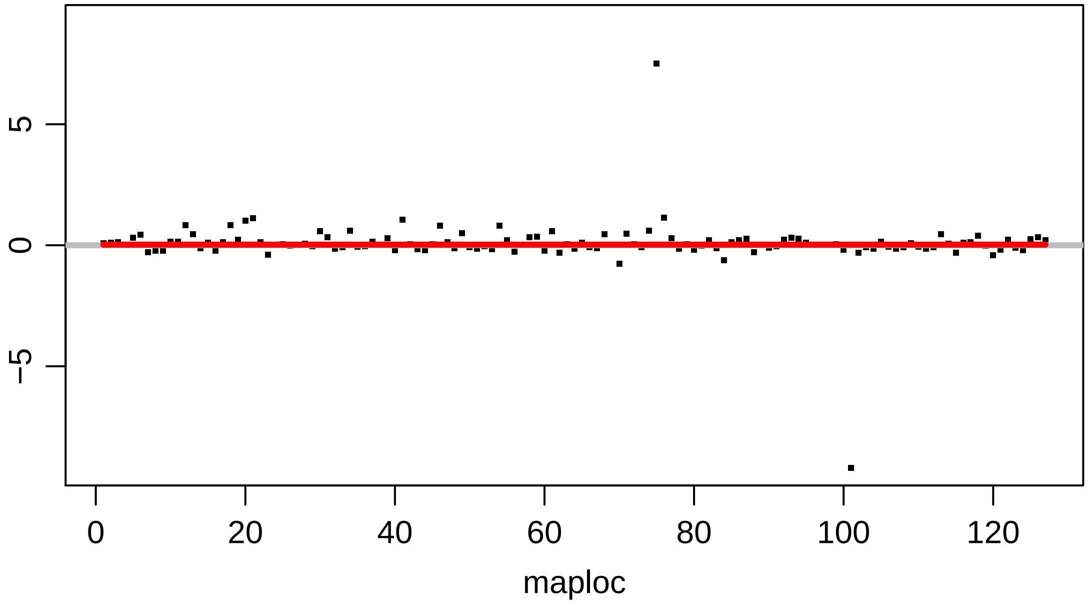


```
## Segplot might not work because of special characters in the sample names. Use only A-Z,a-z and 0-9!
##  There is a hidden function cn.mops:::.replaceNames that replaces the names in the "CNVDetectionResul
```

press_006_R_2012_09_04_02_34_22_Sequoia_SN1.19.withoutCYP_48_Run_16_hg

Chromosome undef

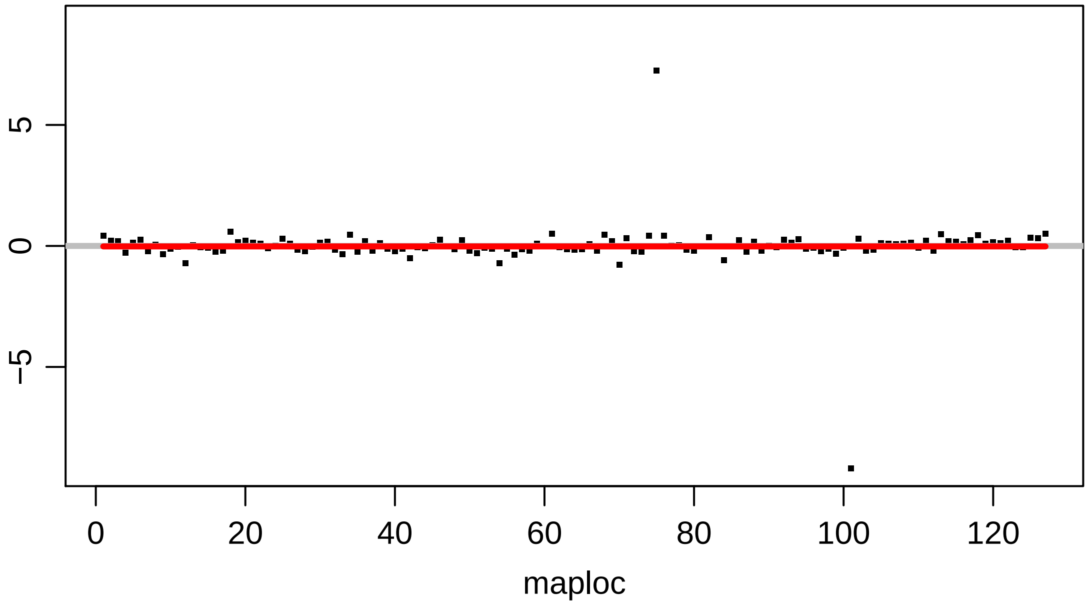

```
## Segplot might not work because of special characters in the sample names. Use only A-Z,a-z and 0-9!
##  There is a hidden function cn.mops:::.replaceNames that replaces the names in the "CNVDetectionResul
```

press_007_R_2012_09_04_02_34_22_Sequoia_SN1.19.withoutCYP_48_Run_16_hg

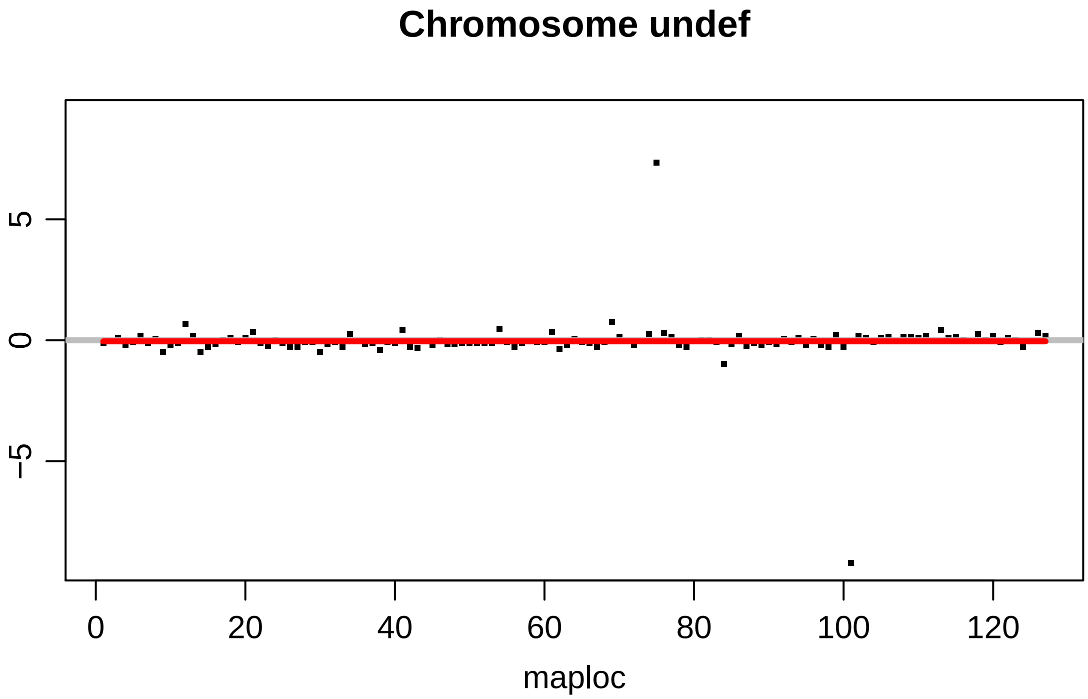


```
## Segplot might not work because of special characters in the sample names. Use only A-Z,a-z and 0-9!
##  There is a hidden function cn.mops:::.replaceNames that replaces the names in the "CNVDetectionResul
```

press_008_R_2012_09_04_02_34_22_Sequoia_SN1.19.withoutCYP_48_Run_16_hg

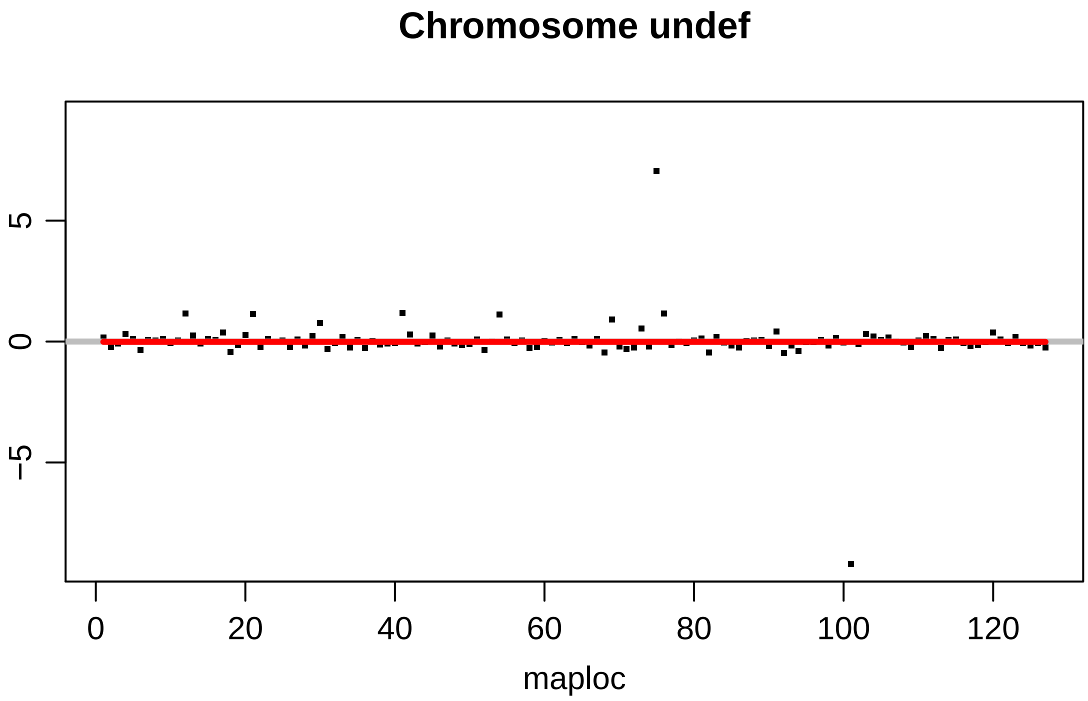


```
## Segplot might not work because of special characters in the sample names. Use only A-Z,a-z and 0-9!
##  There is a hidden function cn.mops:::.replaceNames that replaces the names in the "CNVDetectionResul
```

press_009_R_2012_09_04_02_34_22_Sequoia_SN1.19.withoutCYP_48_Run_16_hg

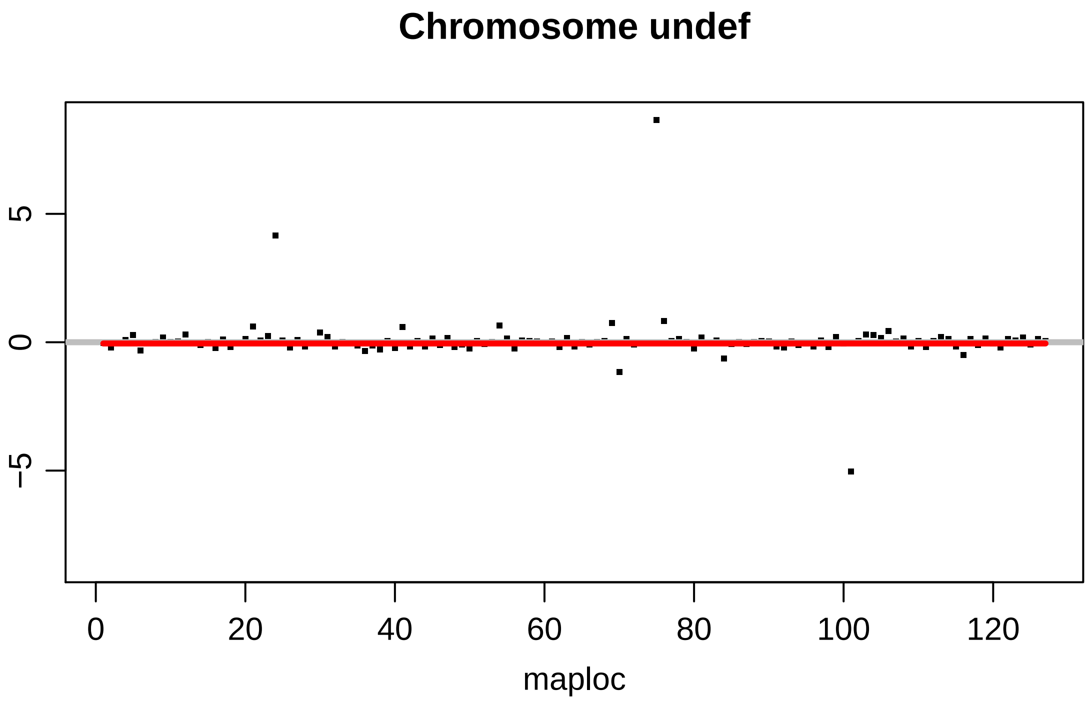

```
## Segplot might not work because of special characters in the sample names. Use only A-Z,a-z and 0-9!
##  There is a hidden function cn.mops:::.replaceNames that replaces the names in the "CNVDetectionResul
```

press_010_R_2012_09_04_02_34_22_Sequoia_SN1.19.withoutCYP_48_Run_16_hg

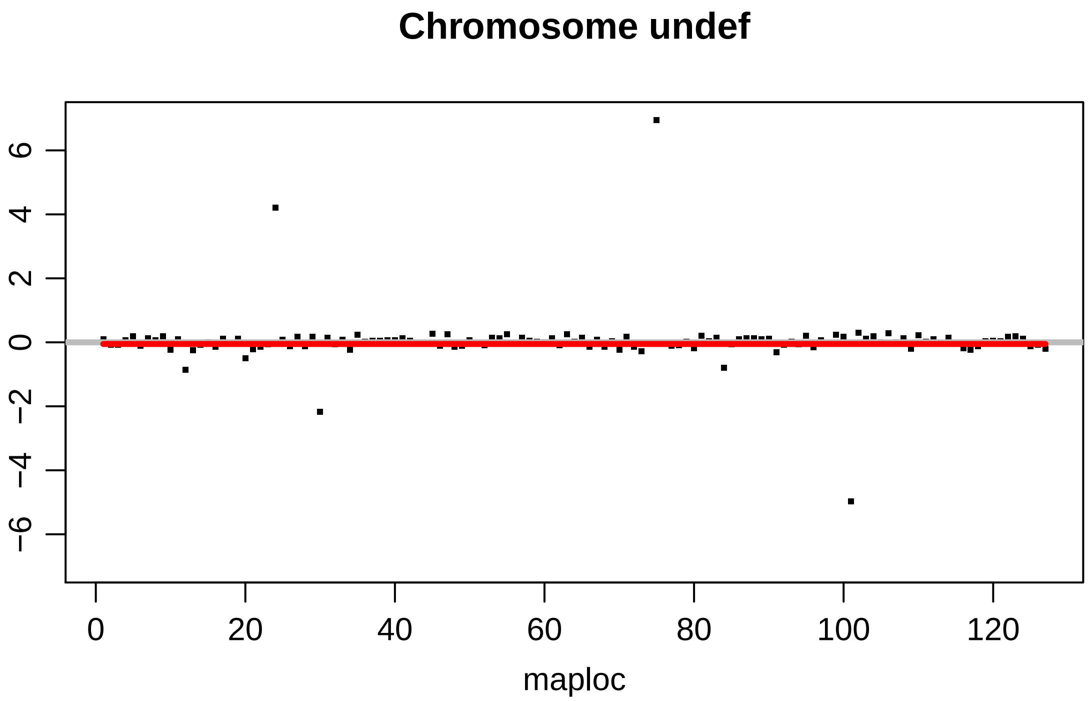


```
## Segplot might not work because of special characters in the sample names. Use only A-Z,a-z and 0-9!
##  There is a hidden function cn.mops:::.replaceNames that replaces the names in the "CNVDetectionResul
```

press_011_R_2012_09_04_02_34_22_Sequoia_SN1.19.withoutCYP_48_Run_16_hg

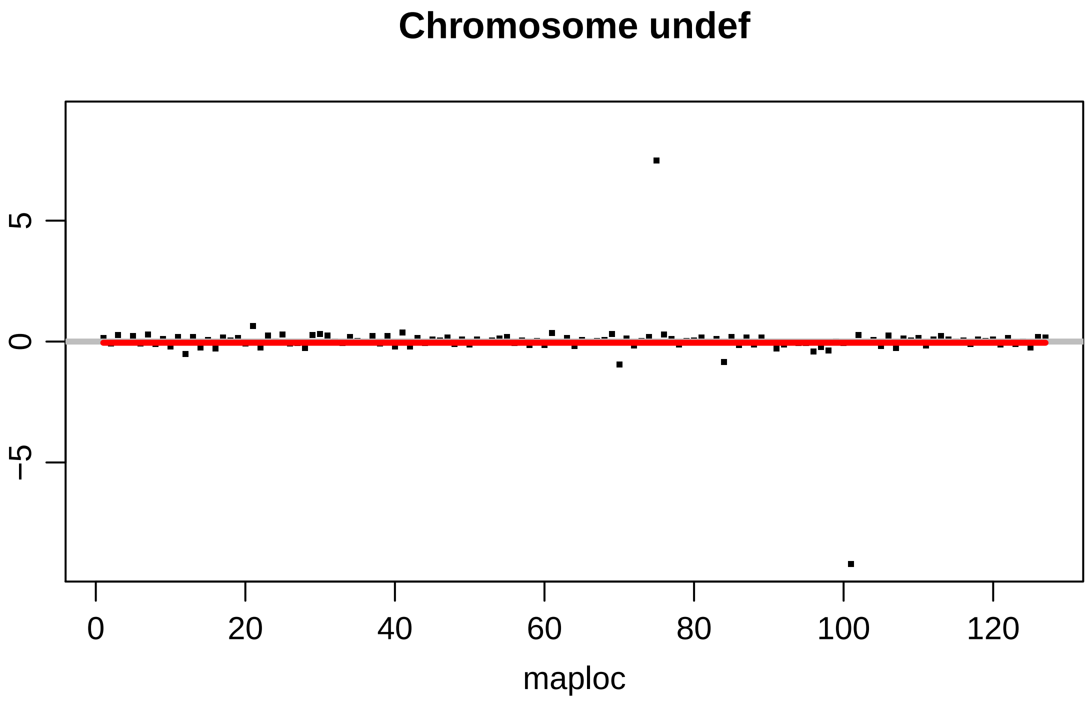


```
## Segplot might not work because of special characters in the sample names. Use only A-Z,a-z and 0-9!
##  There is a hidden function cn.mops:::.replaceNames that replaces the names in the "CNVDetectionResul
```

press_012_R_2012_09_04_02_34_22_Sequoia_SN1.19.withoutCYP_48_Run_16_hg

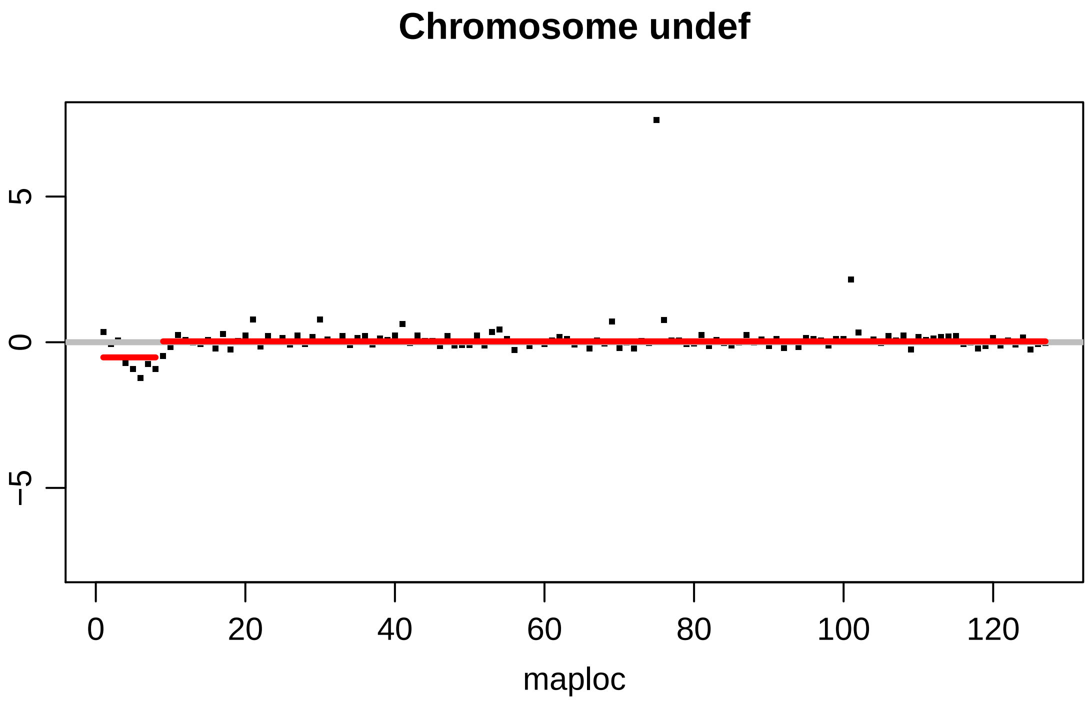

```
## Segplot might not work because of special characters in the sample names. Use only A-Z,a-z and 0-9!
##  There is a hidden function cn.mops:::.replaceNames that replaces the names in the "CNVDetectionResul
```

press_013_R_2012_09_04_02_34_22_Sequoia_SN1.19.withoutCYP_48_Run_16_hg

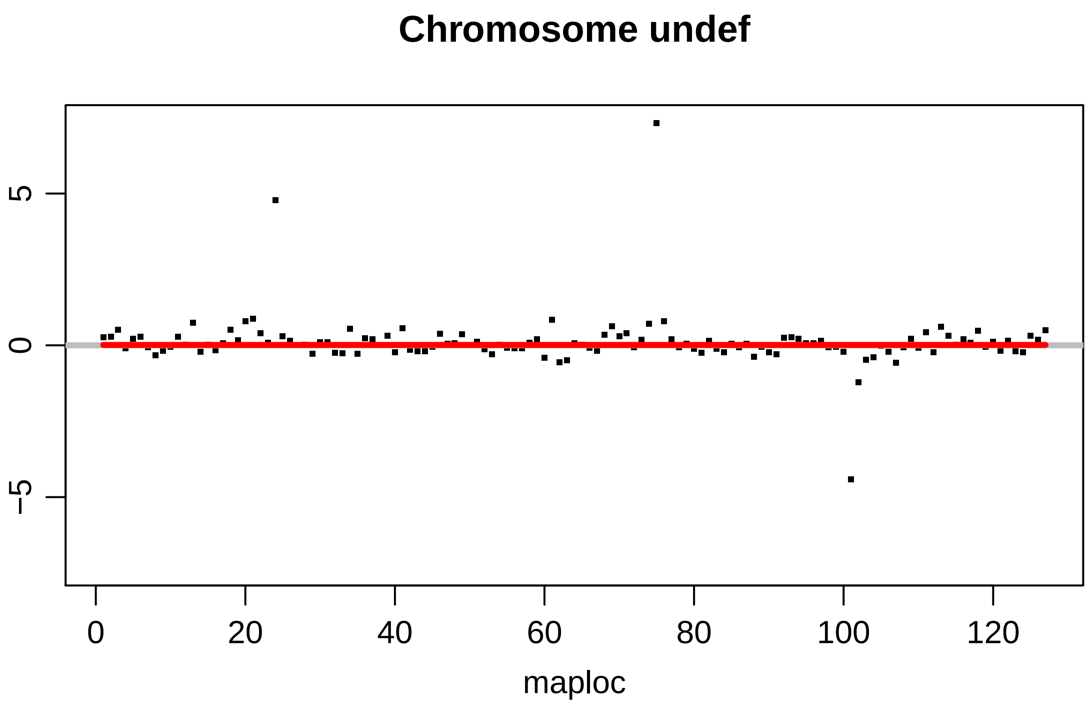


```
## Segplot might not work because of special characters in the sample names. Use only A-Z,a-z and 0-9!
##  There is a hidden function cn.mops:::.replaceNames that replaces the names in the "CNVDetectionResul
```

press_014_R_2012_09_04_02_34_22_Sequoia_SN1.19.withoutCYP_48_Run_16_hg

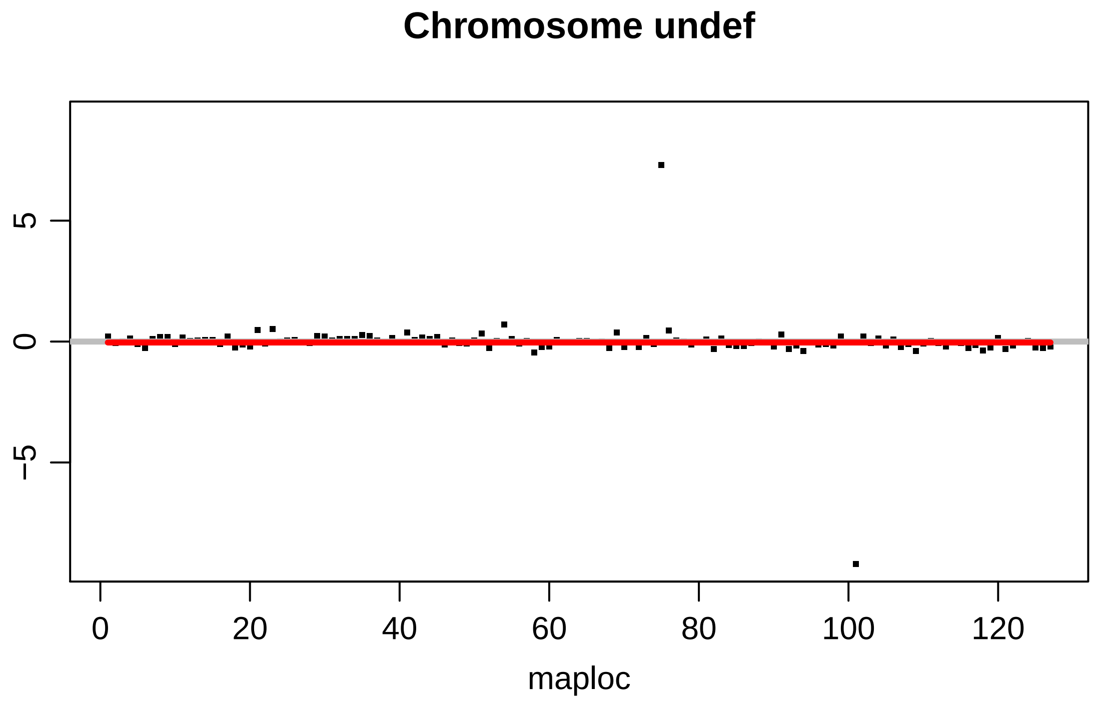


```
## Segplot might not work because of special characters in the sample names. Use only A-Z,a-z and 0-9!
##  There is a hidden function cn.mops:::.replaceNames that replaces the names in the "CNVDetectionResul
```

press_015_R_2012_09_04_02_34_22_Sequoia_SN1.19.withoutCYP_48_Run_16_hg

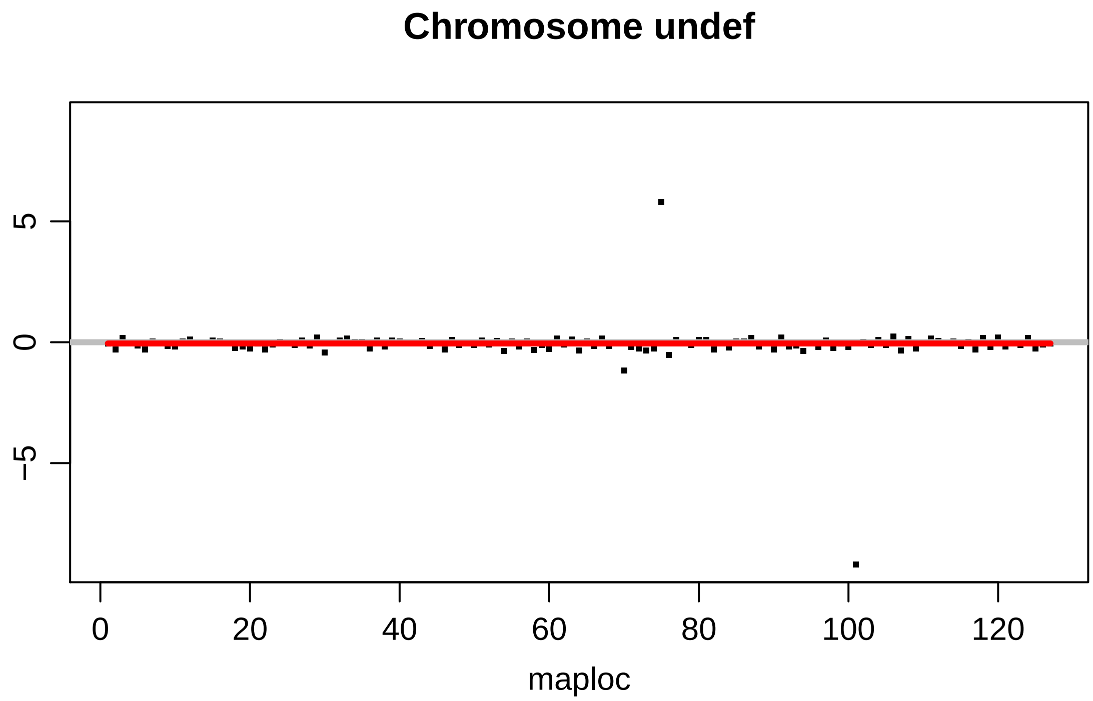

```
## Segplot might not work because of special characters in the sample names. Use only A-Z,a-z and 0-9!
##  There is a hidden function cn.mops:::.replaceNames that replaces the names in the "CNVDetectionResul
```

press_016_R_2012_09_04_02_34_22_Sequoia_SN1.19.withoutCYP_48_Run_16_hg

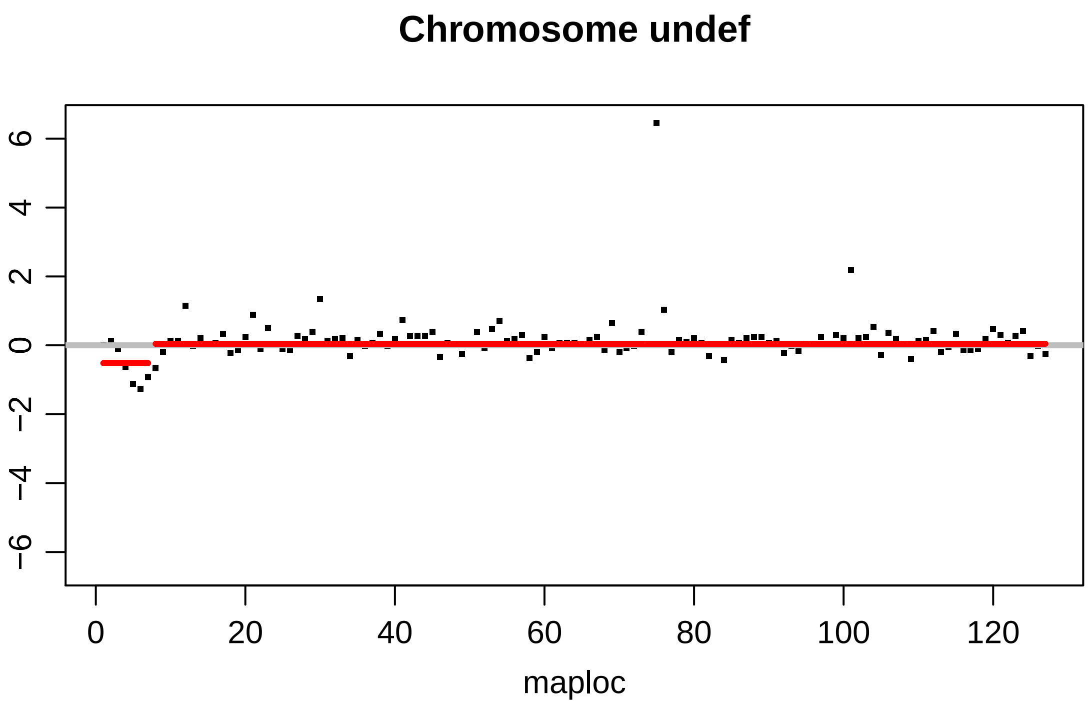


```
## Segplot might not work because of special characters in the sample names. Use only A-Z,a-z and 0-9!
##  There is a hidden function cn.mops:::.replaceNames that replaces the names in the "CNVDetectionResul
```

press_017_R_2012_09_04_02_34_22_Sequoia_SN1.19.withoutCYP_48_Run_16_hg

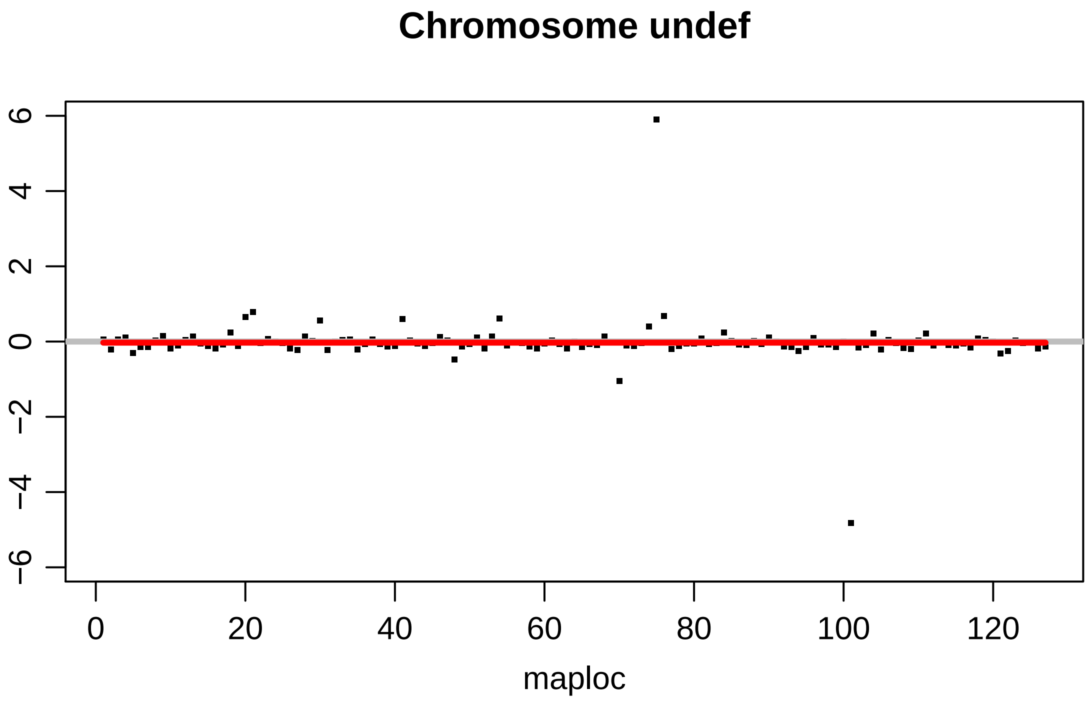


```
## Segplot might not work because of special characters in the sample names. Use only A-Z,a-z and 0-9!
##  There is a hidden function cn.mops:::.replaceNames that replaces the names in the "CNVDetectionResul
```

press_018_R_2012_09_04_02_34_22_Sequoia_SN1.19.withoutCYP_48_Run_16_hg

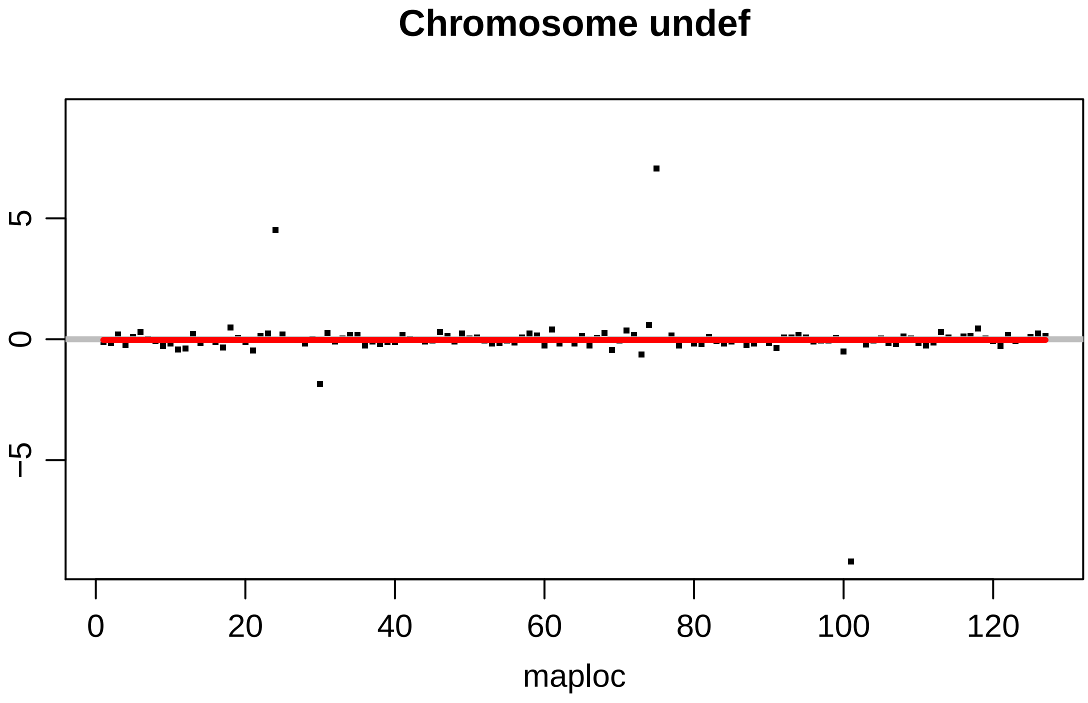

```
## Segplot might not work because of special characters in the sample names. Use only A-Z,a-z and 0-9!
##  There is a hidden function cn.mops:::.replaceNames that replaces the names in the "CNVDetectionResul
```

press_019_R_2012_09_04_02_34_22_Sequoia_SN1.19.withoutCYP_48_Run_16_hg

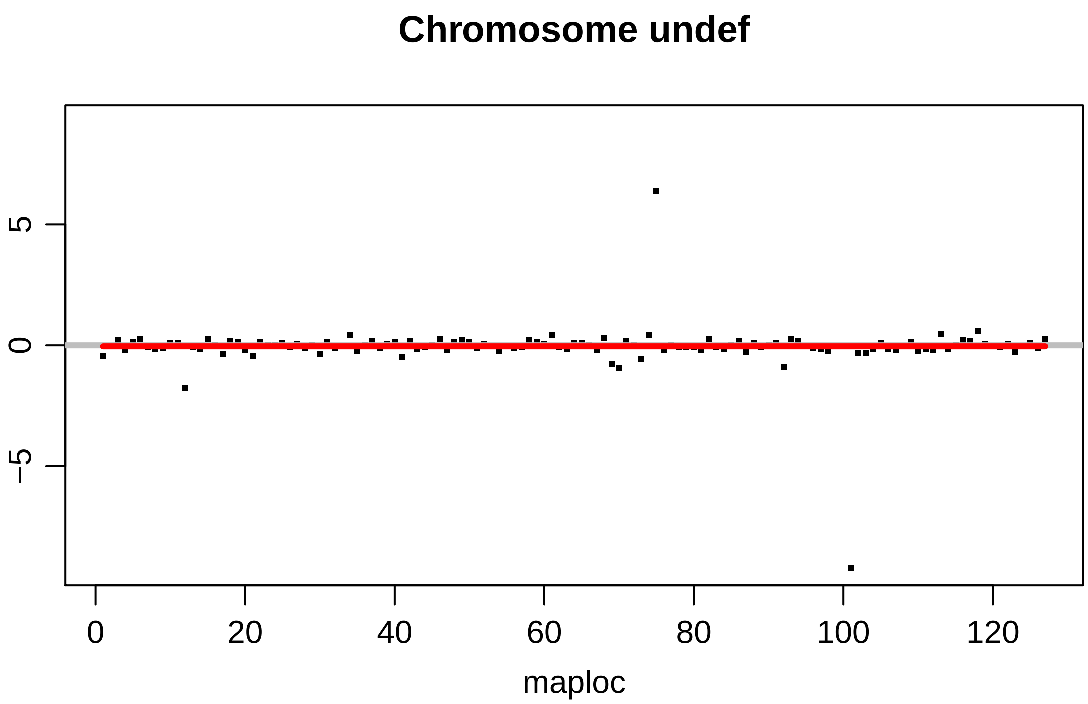


```
## Segplot might not work because of special characters in the sample names. Use only A-Z,a-z and 0-9!
##  There is a hidden function cn.mops:::.replaceNames that replaces the names in the "CNVDetectionResul
```

press_020_R_2012_09_04_02_34_22_Sequoia_SN1.19.withoutCYP_48_Run_16_hg

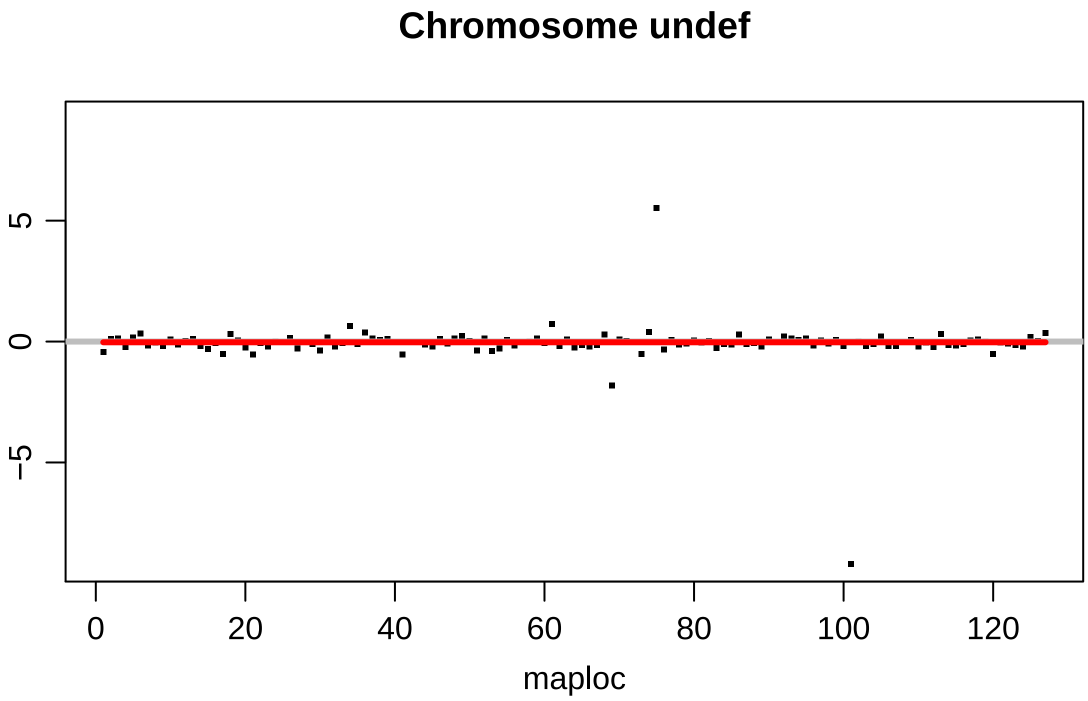


```
## Segplot might not work because of special characters in the sample names. Use only A-Z,a-z and 0-9!
##  There is a hidden function cn.mops:::.replaceNames that replaces the names in the "CNVDetectionResul
```

press_021_R_2012_09_04_02_34_22_Sequoia_SN1.19.withoutCYP_48_Run_16_hg

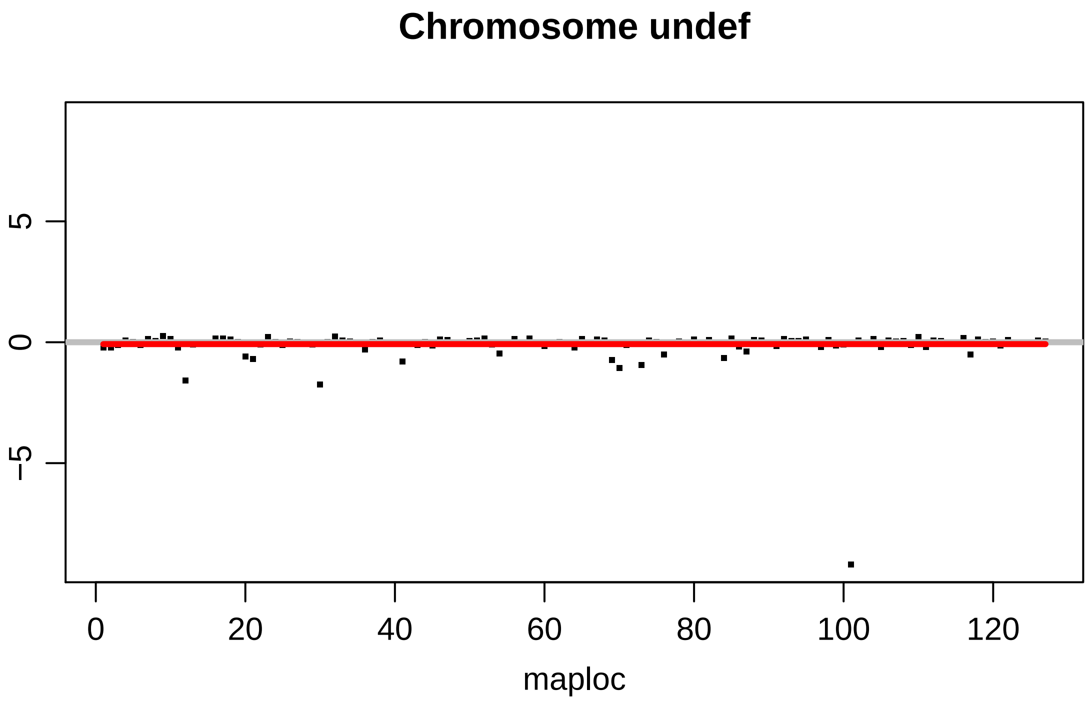

```
## Segplot might not work because of special characters in the sample names. Use only A-Z,a-z and 0-9!
##  There is a hidden function cn.mops:::.replaceNames that replaces the names in the "CNVDetectionResul
```

press_022_R_2012_09_04_02_34_22_Sequoia_SN1.19.withoutCYP_48_Run_16_hg

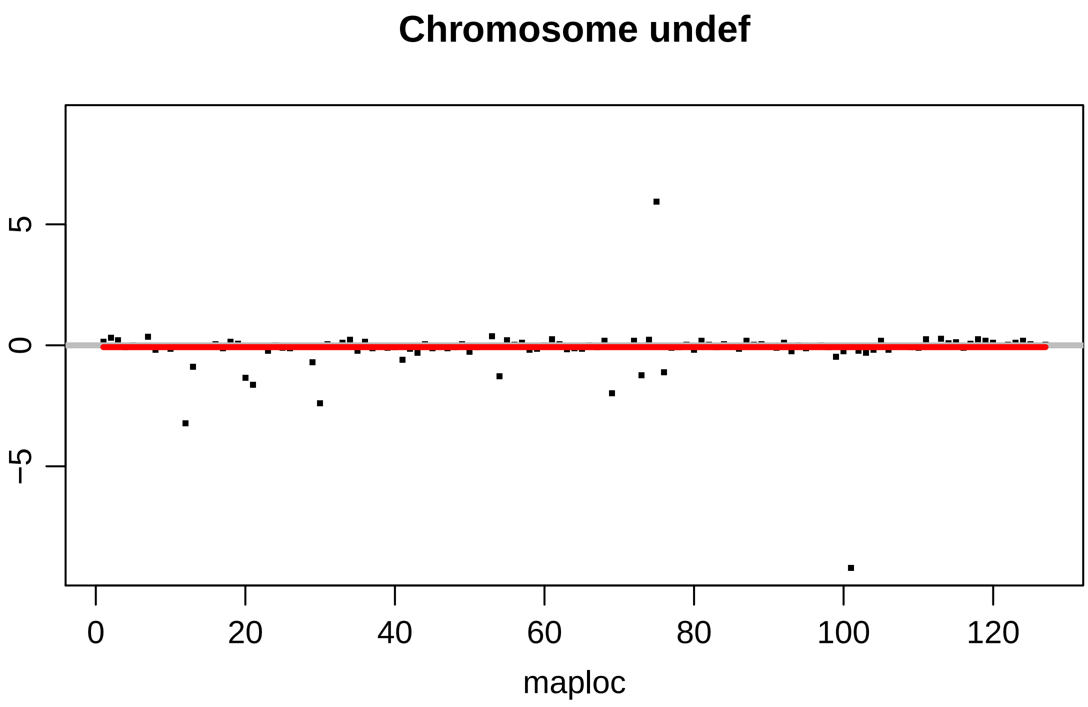


```
## Segplot might not work because of special characters in the sample names. Use only A-Z,a-z and 0-9!
##  There is a hidden function cn.mops:::.replaceNames that replaces the names in the "CNVDetectionResul
```

press_023_R_2012_09_04_02_34_22_Sequoia_SN1.19.withoutCYP_48_Run_16_hg

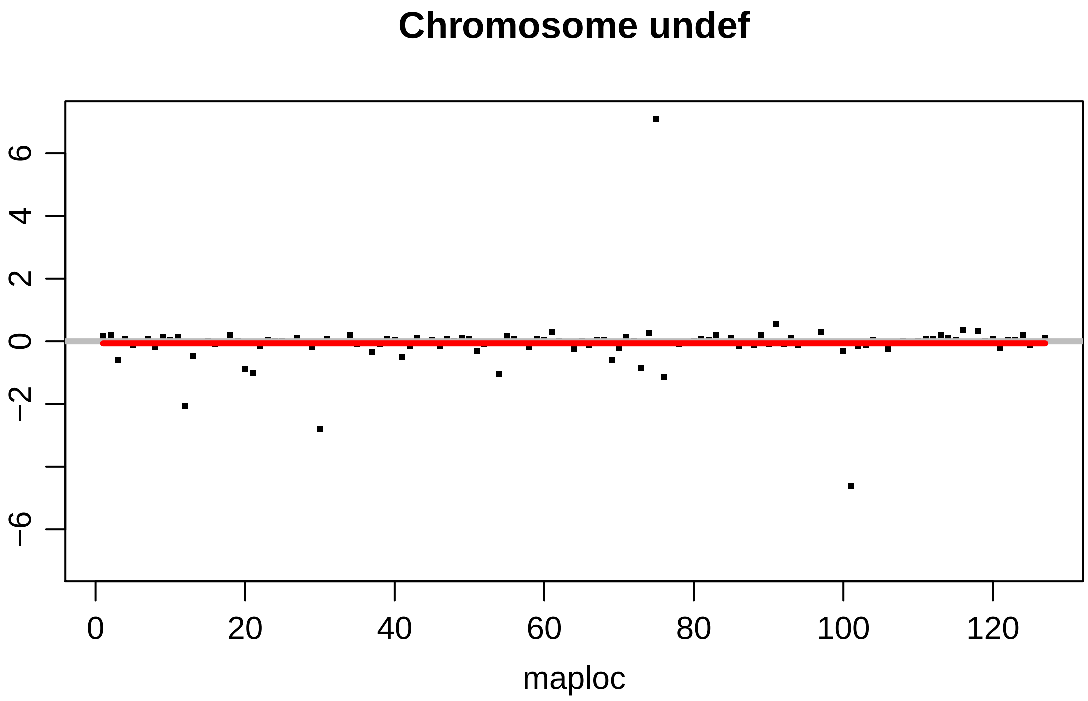


```
## Segplot might not work because of special characters in the sample names. Use only A-Z,a-z and 0-9!
##  There is a hidden function cn.mops:::.replaceNames that replaces the names in the "CNVDetectionResul
```

press_024_R_2012_09_04_02_34_22_Sequoia_SN1.19.withoutCYP_48_Run_16_hg

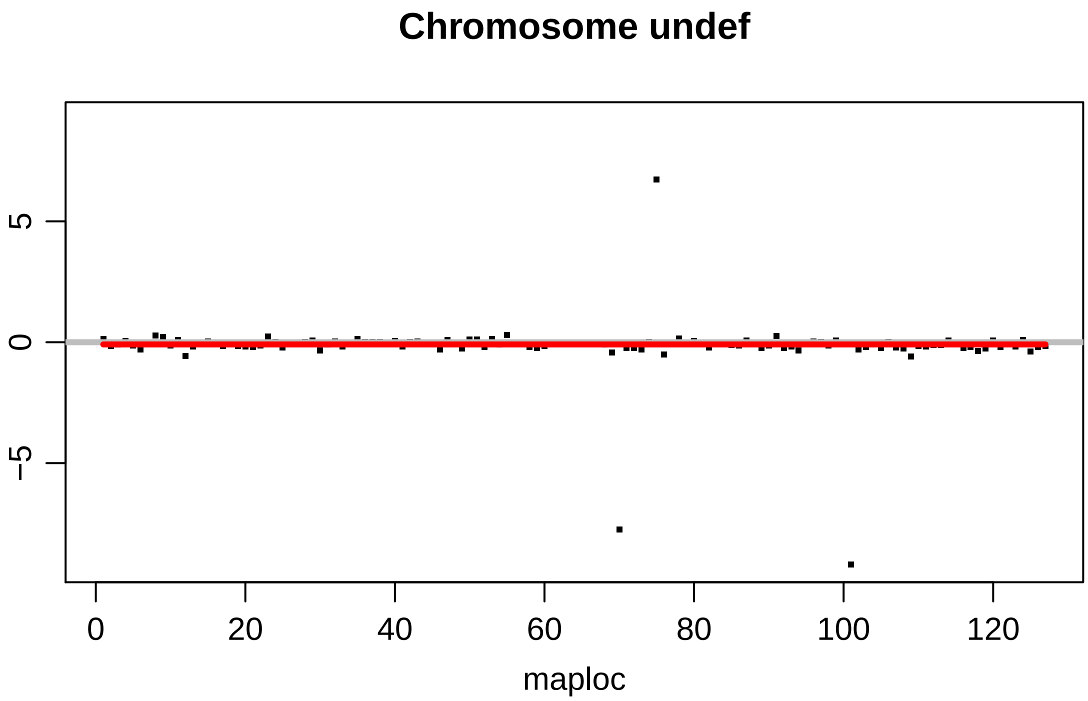

```
## Segplot might not work because of special characters in the sample names. Use only A-Z,a-z and 0-9!
##  There is a hidden function cn.mops:::.replaceNames that replaces the names in the "CNVDetectionResul
```

press_025_R_2012_09_04_02_34_22_Sequoia_SN1.19.withoutCYP_48_Run_16_hg

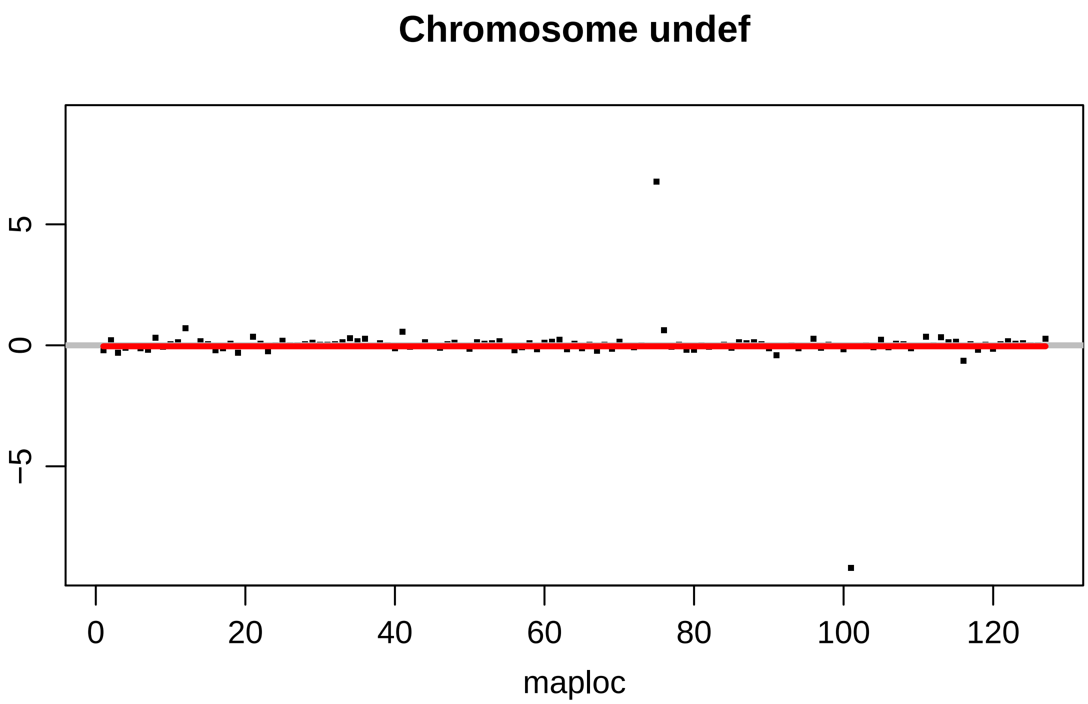


```
## Segplot might not work because of special characters in the sample names. Use only A-Z,a-z and 0-9!
##  There is a hidden function cn.mops:::.replaceNames that replaces the names in the "CNVDetectionResul
```

press_026_R_2012_09_04_02_34_22_Sequoia_SN1.19.withoutCYP_48_Run_16_hg

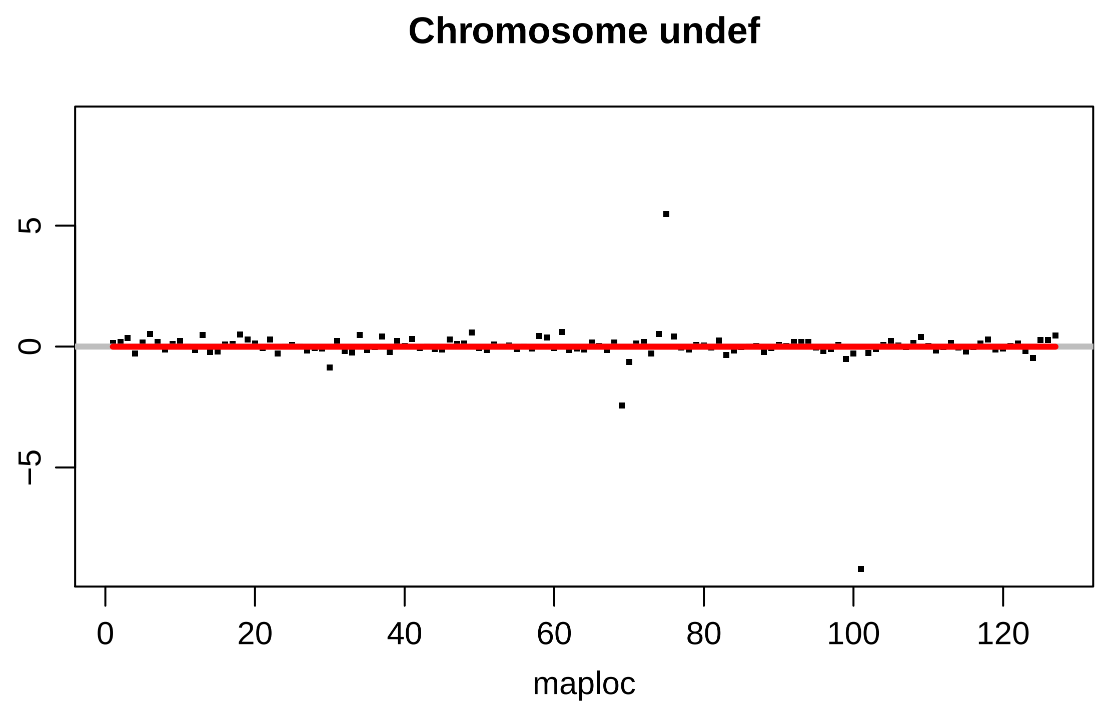


```
## Segplot might not work because of special characters in the sample names. Use only A-Z,a-z and 0-9!
##  There is a hidden function cn.mops:::.replaceNames that replaces the names in the "CNVDetectionResu
```

press_027_R_2012_09_04_02_34_22_Sequoia_SN1.19.withoutCYP_48_Run_16_hg

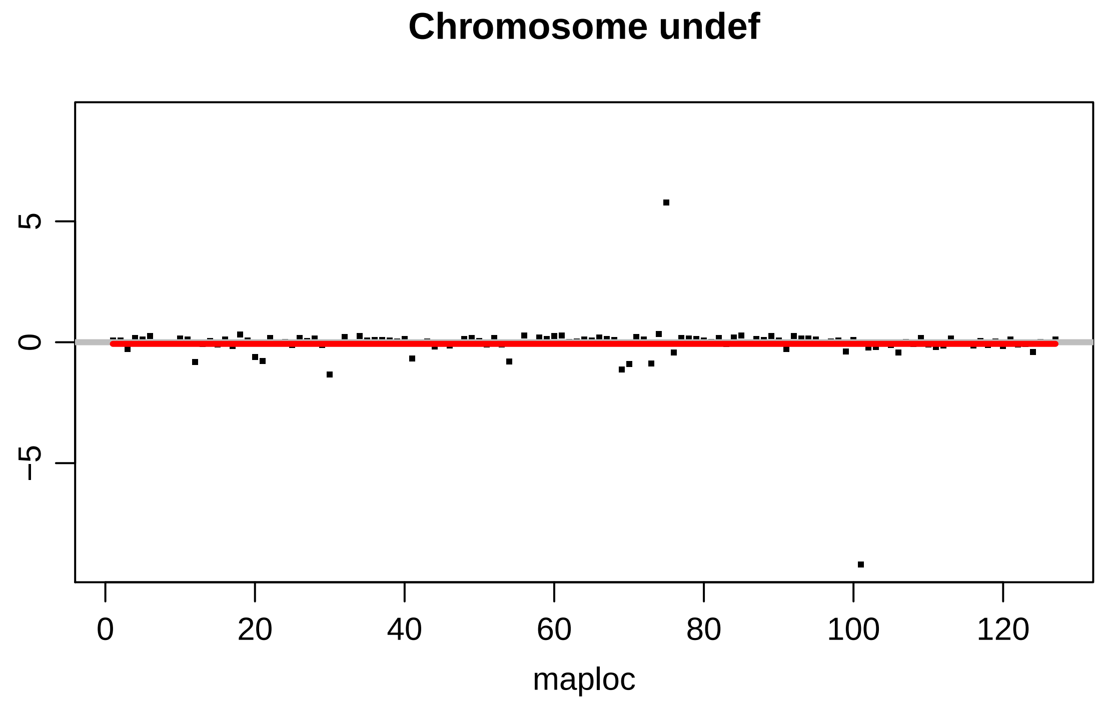

```
## Segplot might not work because of special characters in the sample names. Use only A-Z,a-z and 0-9!
##  There is a hidden function cn.mops:::.replaceNames that replaces the names in the "CNVDetectionResul
```

press_028_R_2012_09_04_02_34_22_Sequoia_SN1.19.withoutCYP_48_Run_16_hg

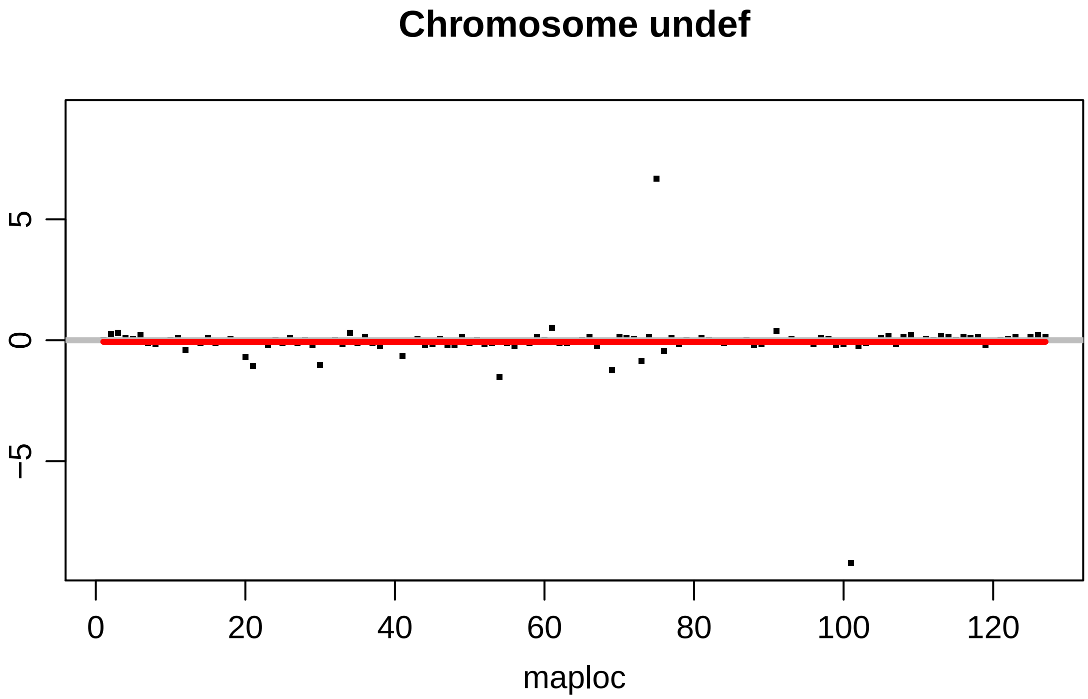


```
## Segplot might not work because of special characters in the sample names. Use only A-Z,a-z and 0-9!
##  There is a hidden function cn.mops:::.replaceNames that replaces the names in the "CNVDetectionResul
```

press_029_R_2012_09_04_02_34_22_Sequoia_SN1.19.withoutCYP_48_Run_16_hg

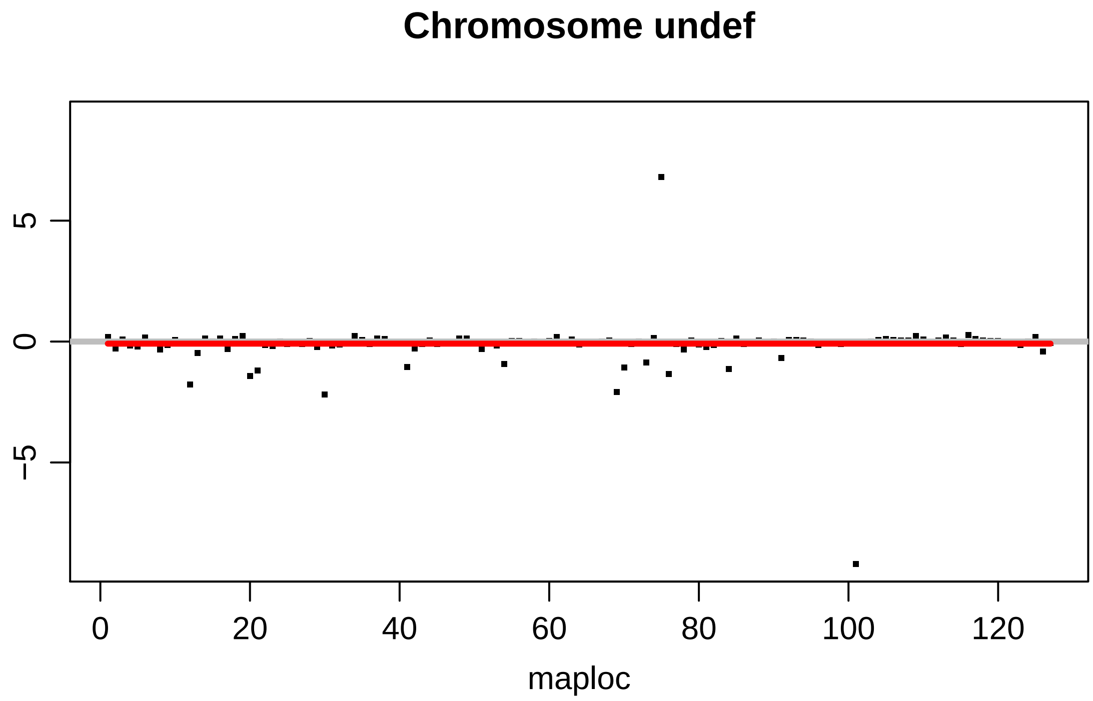


```
## Segplot might not work because of special characters in the sample names. Use only A-Z,a-z and 0-9!
##  There is a hidden function cn.mops:::.replaceNames that replaces the names in the "CNVDetectionResul
```

press_030_R_2012_09_04_02_34_22_Sequoia_SN1.19.withoutCYP_48_Run_16_hg

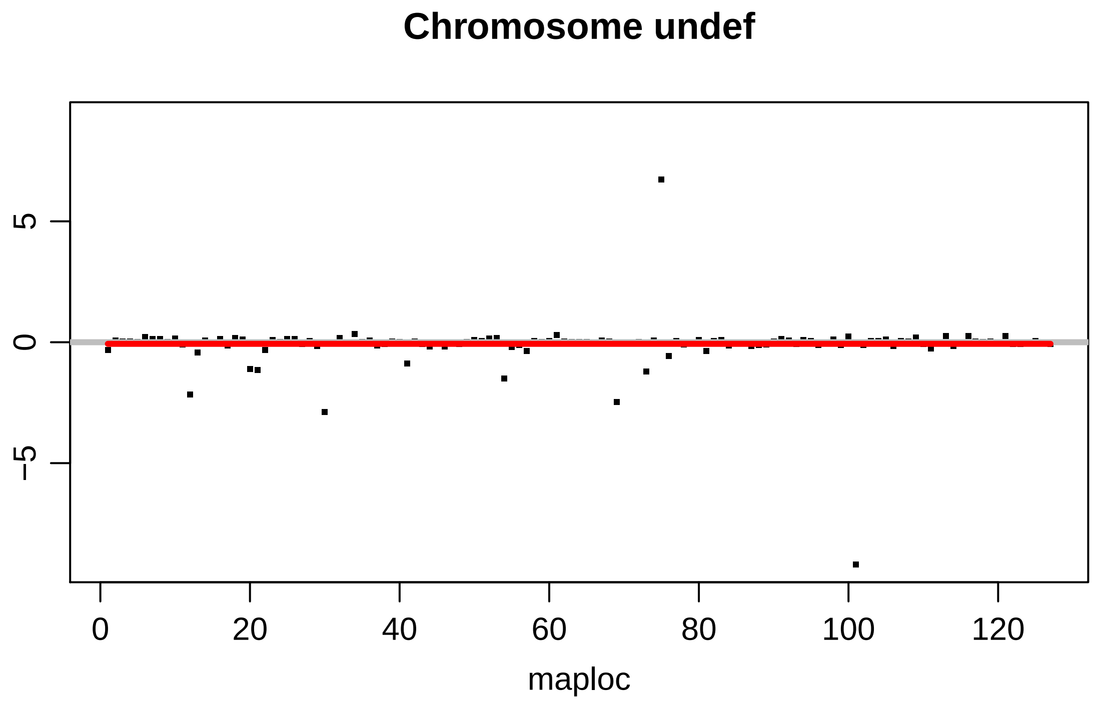

```
## Segplot might not work because of special characters in the sample names. Use only A-Z,a-z and 0-9!
##  There is a hidden function cn.mops:::.replaceNames that replaces the names in the "CNVDetectionResul
```

press_031_R_2012_09_04_02_34_22_Sequoia_SN1.19.withoutCYP_48_Run_16_hg

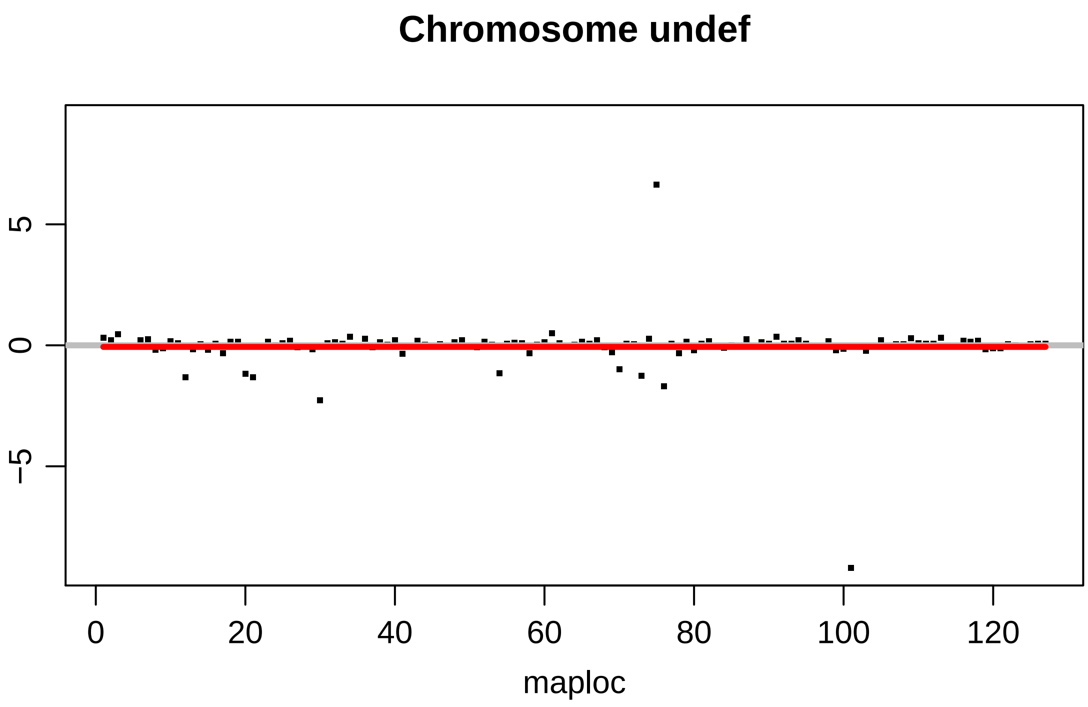


```
## Segplot might not work because of special characters in the sample names. Use only A-Z,a-z and 0-9!
##  There is a hidden function cn.mops:::.replaceNames that replaces the names in the "CNVDetectionResul
```

press_032_R_2012_09_04_02_34_22_Sequoia_SN1.19.withoutCYP_48_Run_16_hg

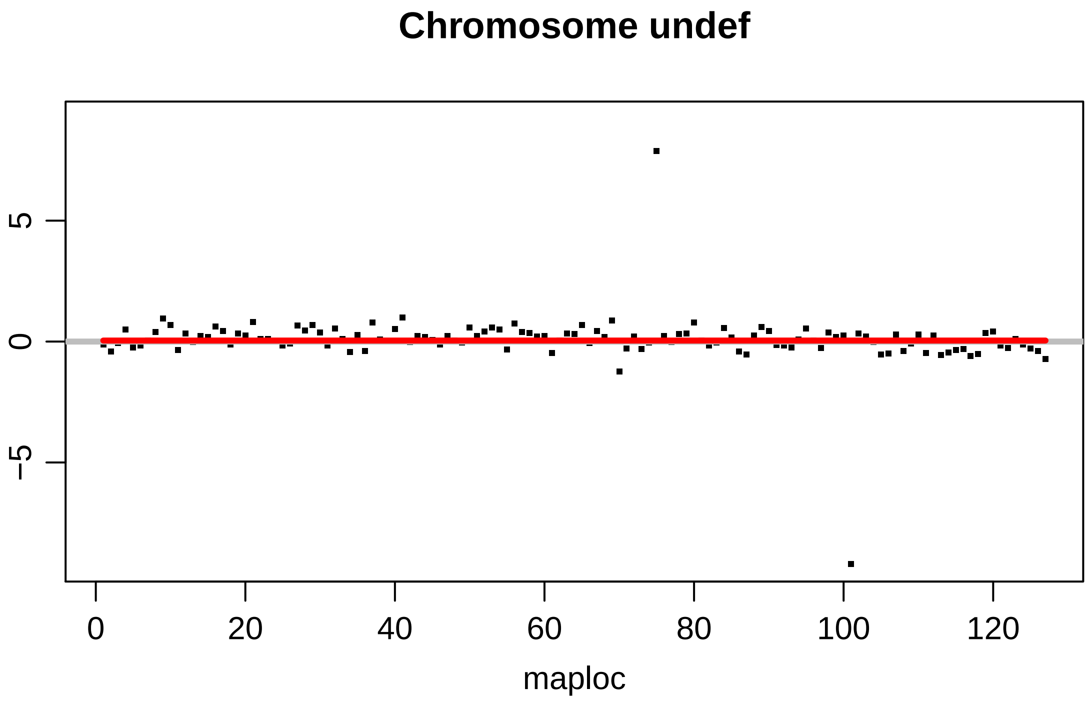


```
## Segplot might not work because of special characters in the sample names. Use only A-Z,a-z and 0-9!
##  There is a hidden function cn.mops:::.replaceNames that replaces the names in the "CNVDetectionResul
```

press_033_R_2012_09_04_02_34_22_Sequoia_SN1.19.withoutCYP_48_Run_16_hg

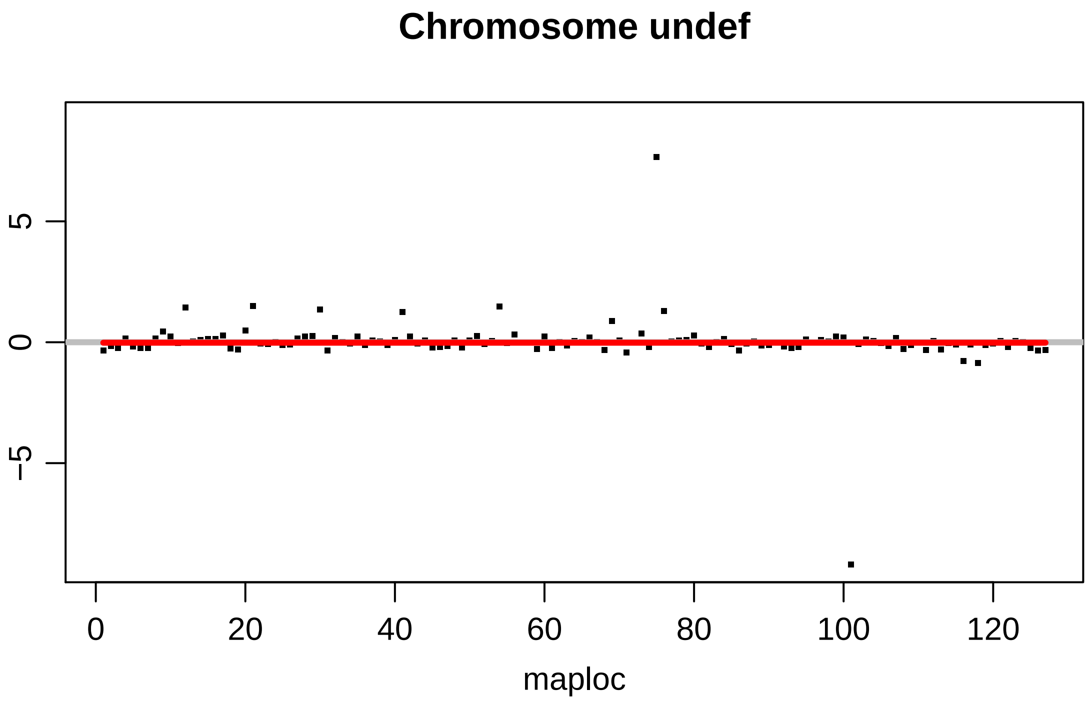

```
## Segplot might not work because of special characters in the sample names. Use only A-Z,a-z and 0-9!
##  There is a hidden function cn.mops:::.replaceNames that replaces the names in the "CNVDetectionResul
```

press_034_R_2012_09_04_02_34_22_Sequoia_SN1.19.withoutCYP_48_Run_16_hg

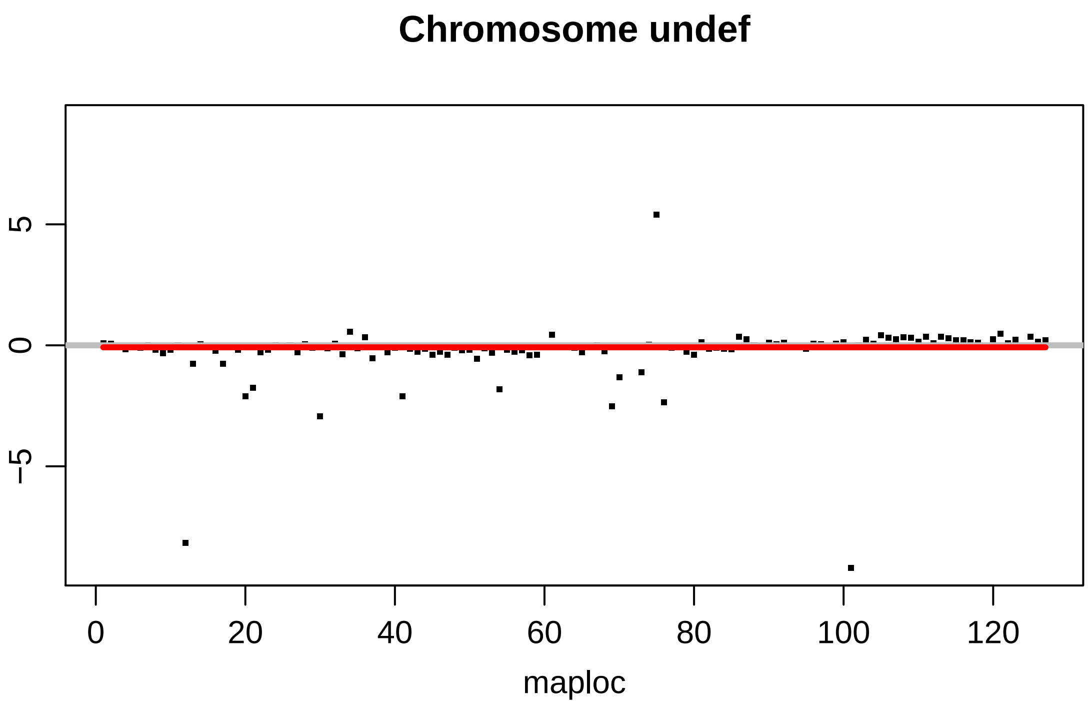


```
## Segplot might not work because of special characters in the sample names. Use only A-Z,a-z and 0-9!
##  There is a hidden function cn.mops:::.replaceNames that replaces the names in the "CNVDetectionResul
```

press_035_R_2012_09_04_02_34_22_Sequoia_SN1.19.withoutCYP_48_Run_16_hg

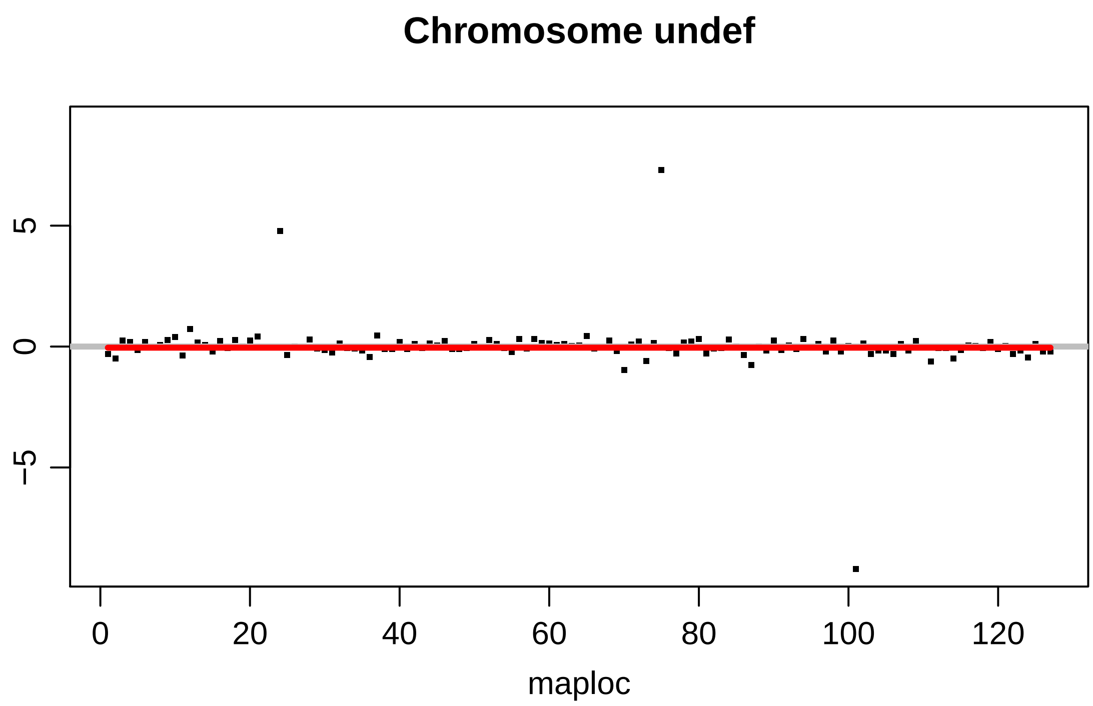


```
## Segplot might not work because of special characters in the sample names. Use only A-Z,a-z and 0-9!
##  There is a hidden function cn.mops:::.replaceNames that replaces the names in the "CNVDetectionResul
```

press_036_R_2012_09_04_02_34_22_Sequoia_SN1.19.withoutCYP_48_Run_16_hg

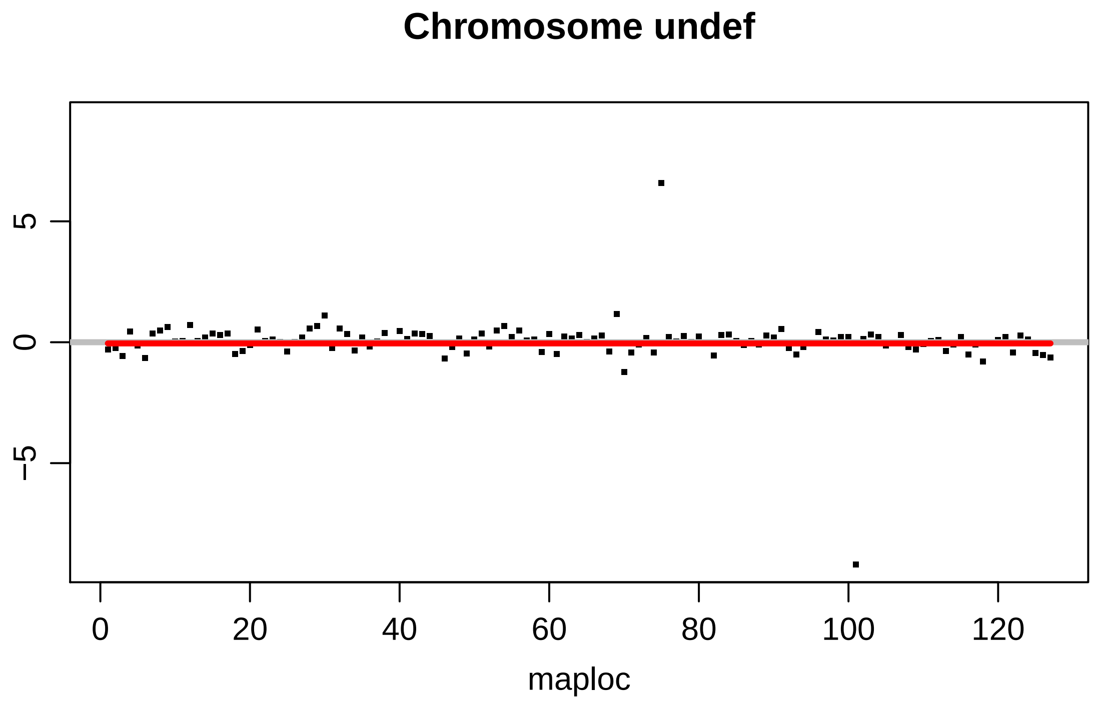

```
## Segplot might not work because of special characters in the sample names. Use only A-Z,a-z and 0-9!
##  There is a hidden function cn.mops:::.replaceNames that replaces the names in the "CNVDetectionResul
```

press_037_R_2012_09_04_02_34_22_Sequoia_SN1.19.withoutCYP_48_Run_16_hg

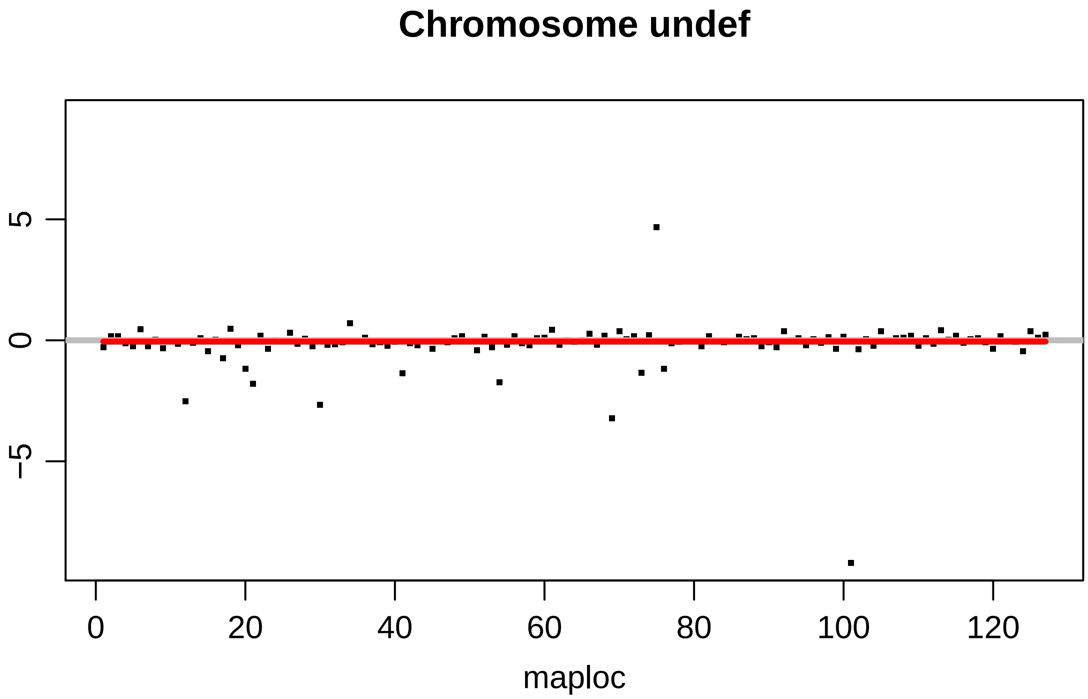


```
## Segplot might not work because of special characters in the sample names. Use only A-Z,a-z and 0-9!
##  There is a hidden function cn.mops:::.replaceNames that replaces the names in the "CNVDetectionResul
```

press_038_R_2012_09_04_02_34_22_Sequoia_SN1.19.withoutCYP_48_Run_16_hg

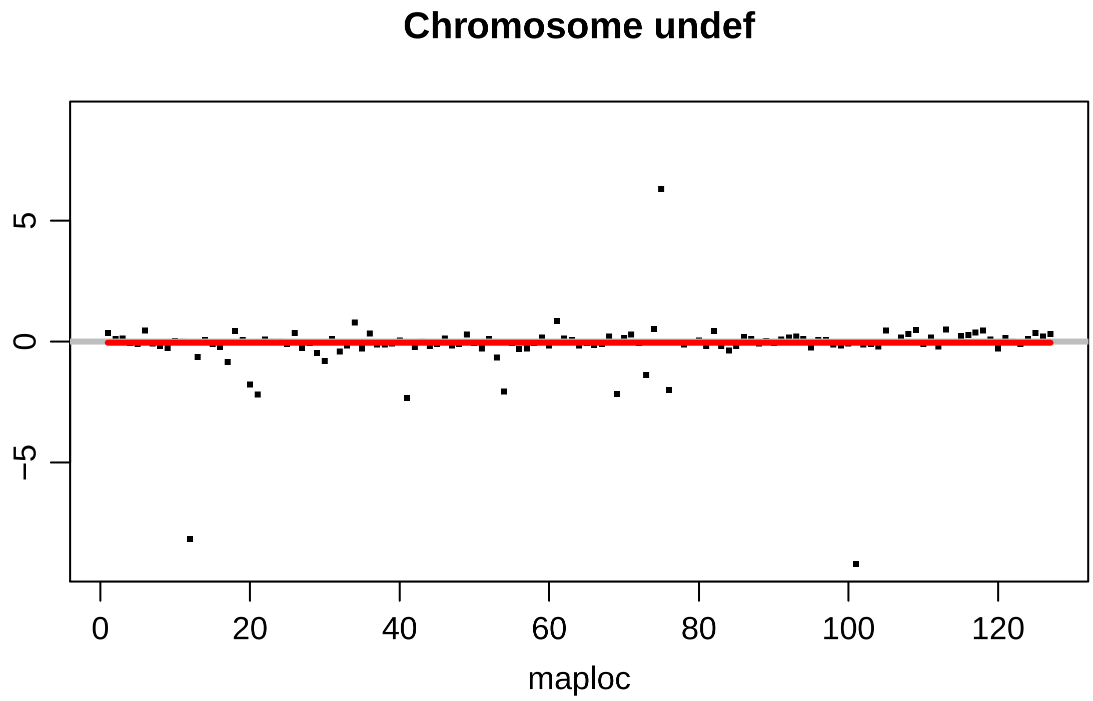


```
## Segplot might not work because of special characters in the sample names. Use only A-Z,a-z and 0-9!
##  There is a hidden function cn.mops:::.replaceNames that replaces the names in the "CNVDetectionResul
```

press_039_R_2012_09_04_02_34_22_Sequoia_SN1.19.withoutCYP_48_Run_16_hg

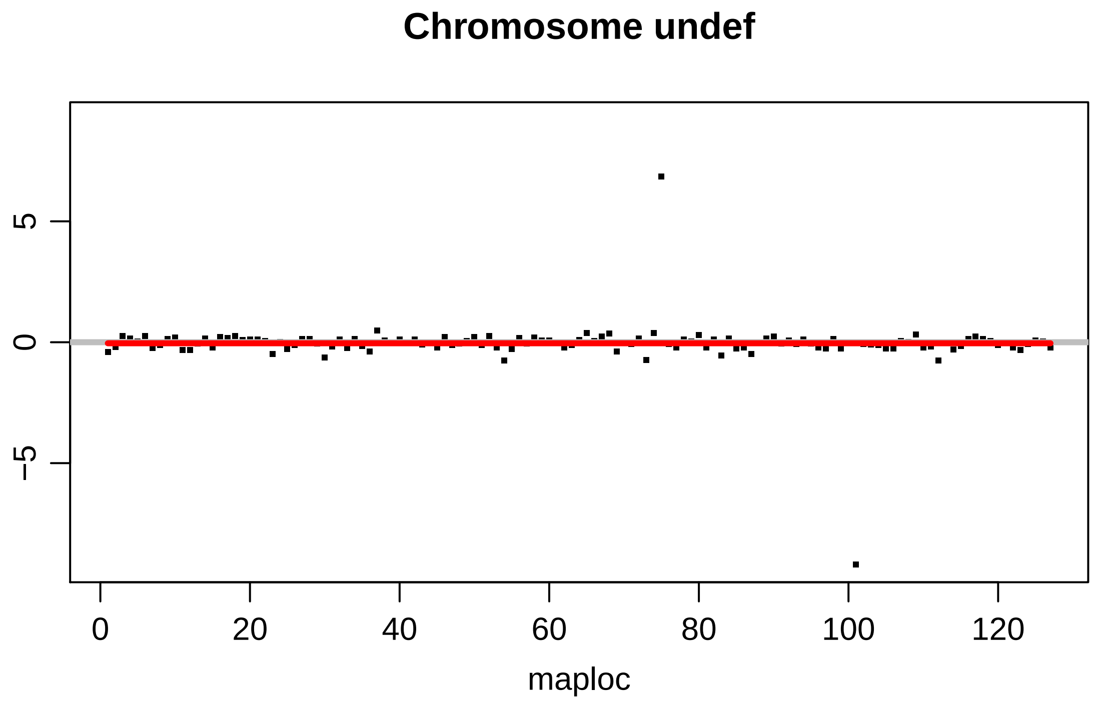

```
## Segplot might not work because of special characters in the sample names. Use only A-Z,a-z and 0-9!
##  There is a hidden function cn.mops:::.replaceNames that replaces the names in the "CNVDetectionResul
```

press_040_R_2012_09_04_02_34_22_Sequoia_SN1.19.withoutCYP_48_Run_16_hg

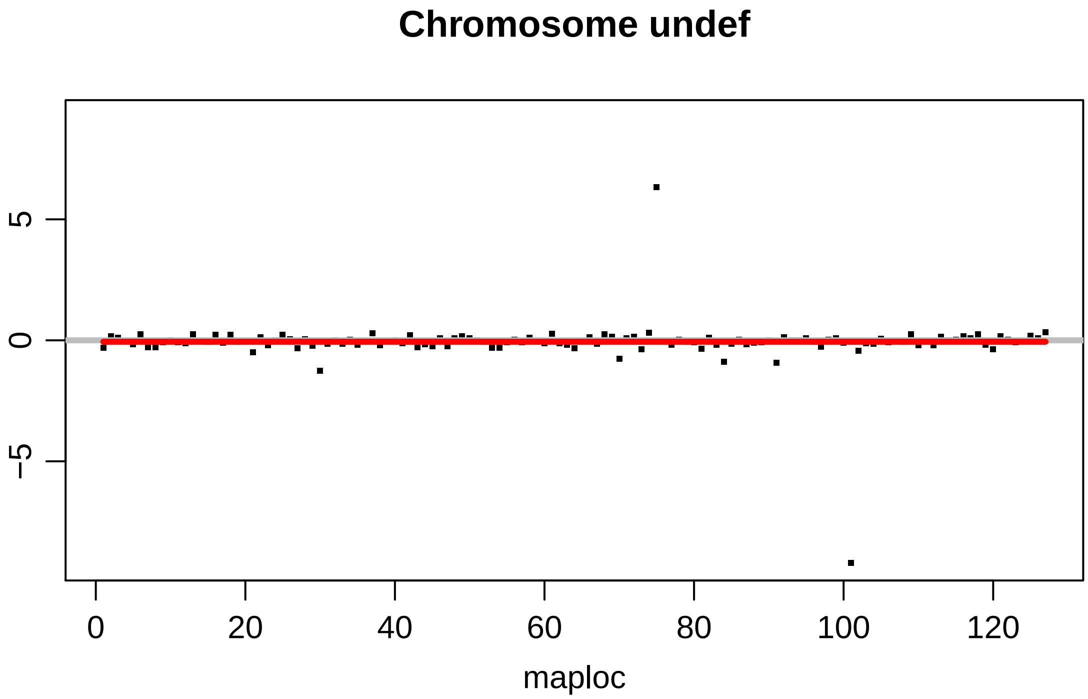


```
## Segplot might not work because of special characters in the sample names. Use only A-Z,a-z and 0-9!
##  There is a hidden function cn.mops:::.replaceNames that replaces the names in the "CNVDetectionResul
```

press_041_R_2012_09_04_02_34_22_Sequoia_SN1.19.withoutCYP_48_Run_16_hg

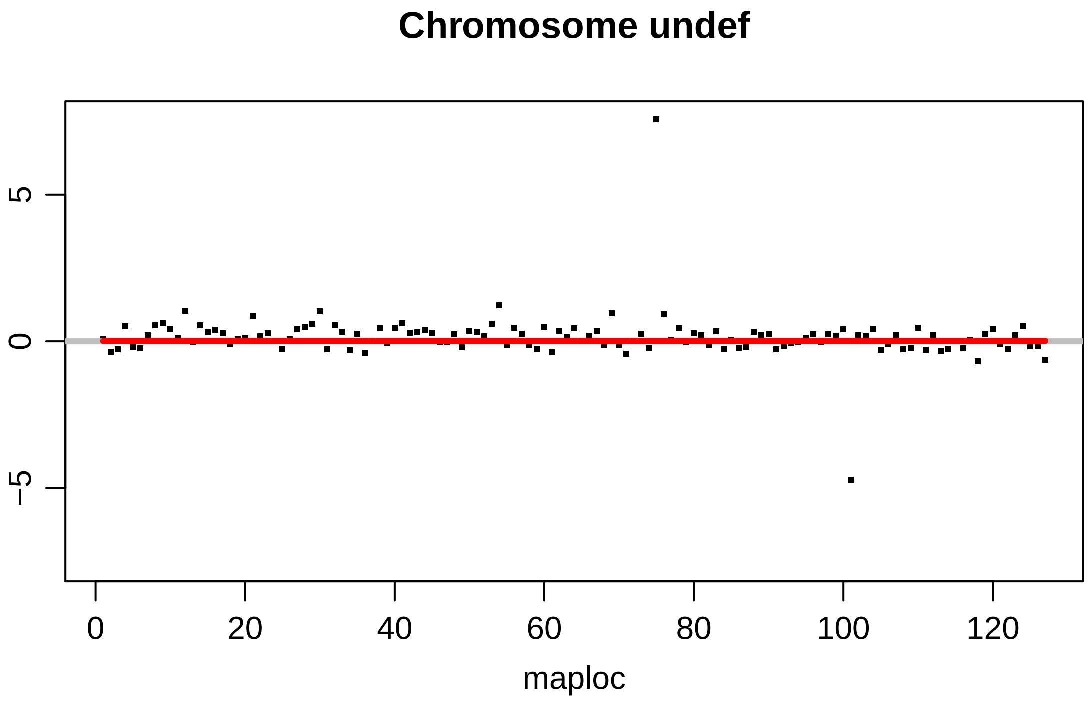


```
## Segplot might not work because of special characters in the sample names. Use only A-Z,a-z and 0-9!
##  There is a hidden function cn.mops:::.replaceNames that replaces the names in the "CNVDetectionResul
```

press_042_R_2012_09_04_02_34_22_Sequoia_SN1.19.withoutCYP_48_Run_16_hg

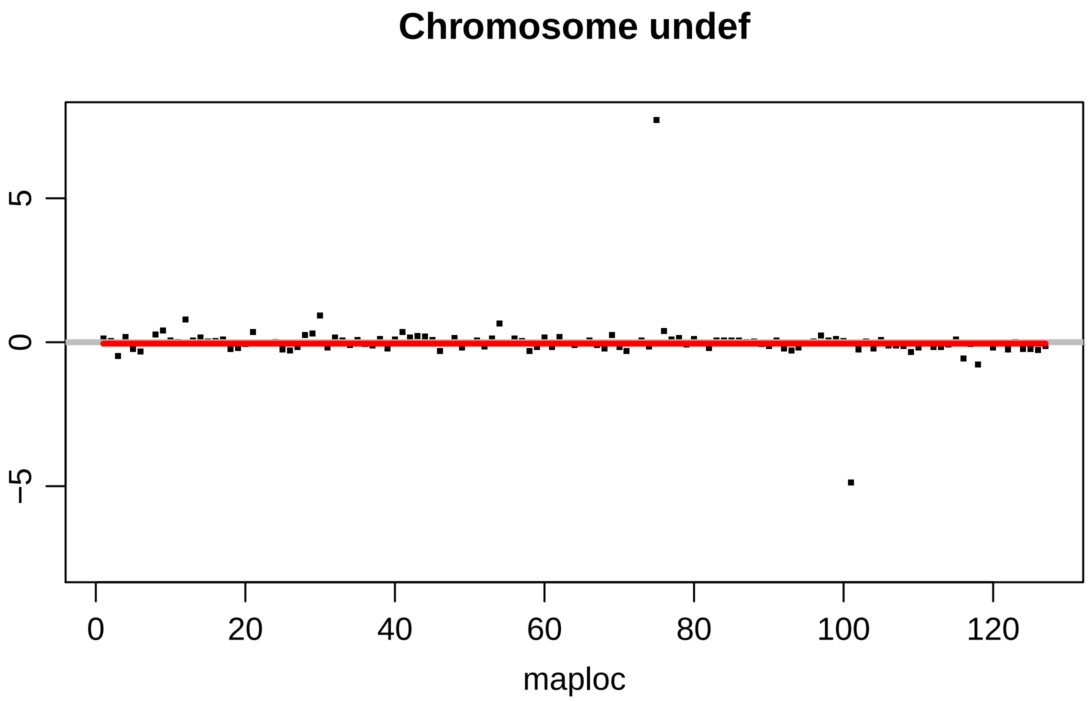

```
## Segplot might not work because of special characters in the sample names. Use only A-Z,a-z and 0-9!
##  There is a hidden function cn.mops:::.replaceNames that replaces the names in the "CNVDetectionResul
```

press_043_R_2012_09_04_02_34_22_Sequoia_SN1.19.withoutCYP_48_Run_16_hg

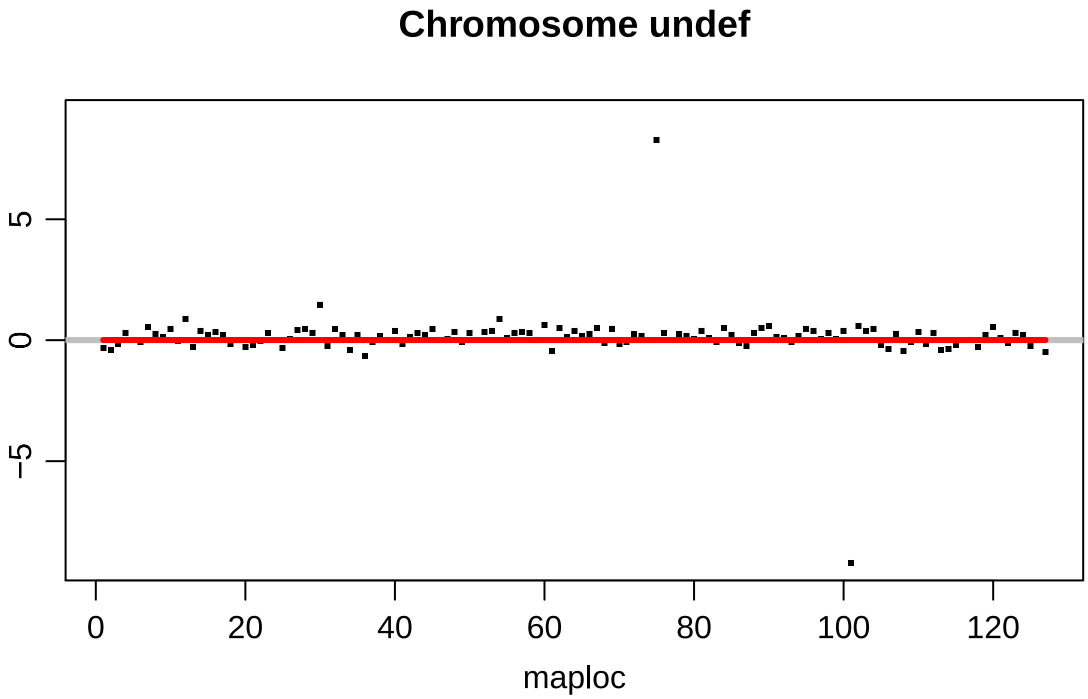


```
## Segplot might not work because of special characters in the sample names. Use only A-Z,a-z and 0-9!
##  There is a hidden function cn.mops:::.replaceNames that replaces the names in the "CNVDetectionResul
```

press_044_R_2012_09_04_02_34_22_Sequoia_SN1.19.withoutCYP_48_Run_16_hg

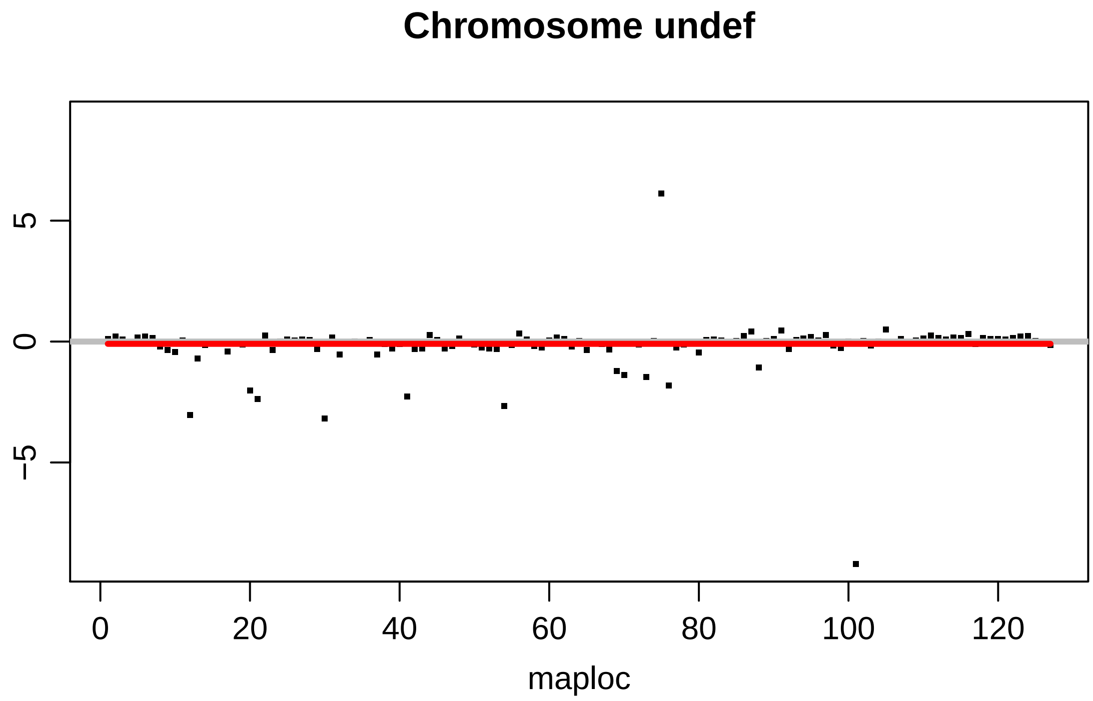


```
## Segplot might not work because of special characters in the sample names. Use only A-Z,a-z and 0-9!
##  There is a hidden function cn.mops:::.replaceNames that replaces the names in the "CNVDetectionResul
```

press_045_R_2012_09_04_02_34_22_Sequoia_SN1.19.withoutCYP_48_Run_16_hg

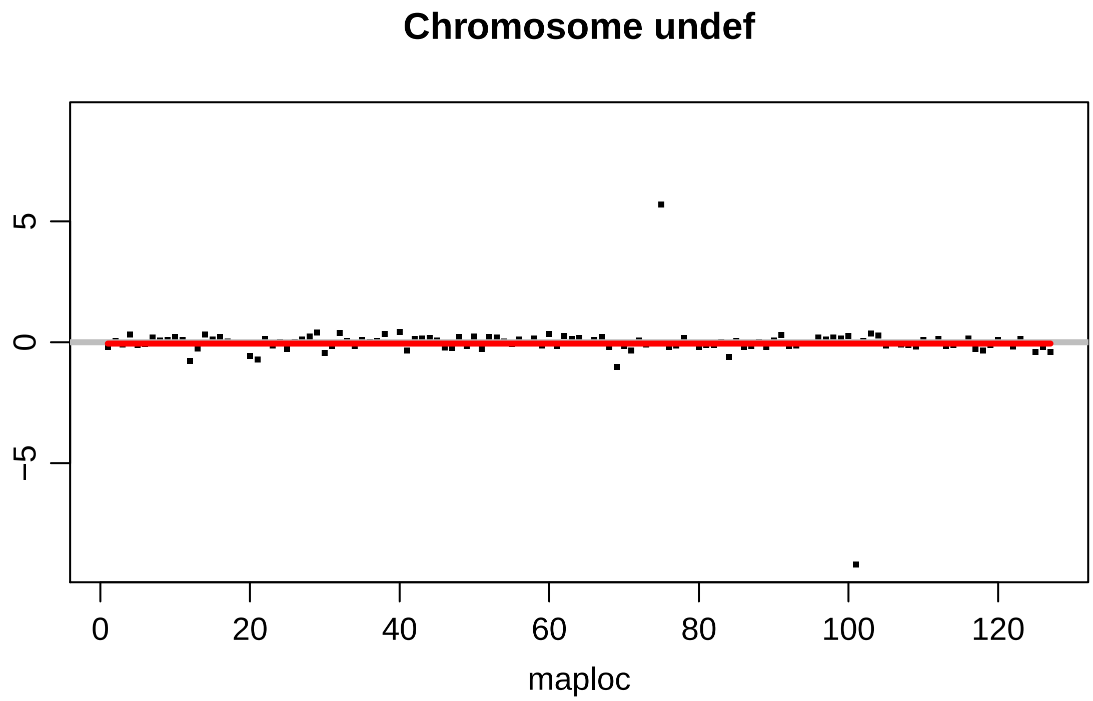

```
## Segplot might not work because of special characters in the sample names. Use only A-Z,a-z and 0-9!
##  There is a hidden function cn.mops:::.replaceNames that replaces the names in the "CNVDetectionResul
```

press_046_R_2012_09_04_02_34_22_Sequoia_SN1.19.withoutCYP_48_Run_16_hg

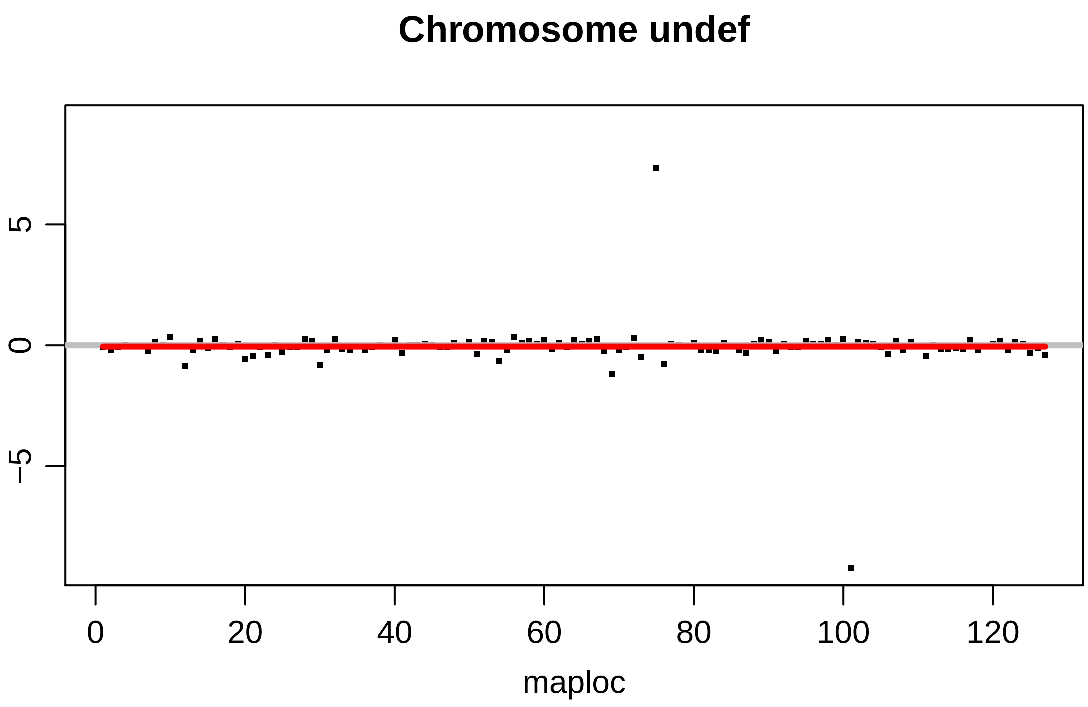


```
## Segplot might not work because of special characters in the sample names. Use only A-Z,a-z and 0-9!
##  There is a hidden function cn.mops:::.replaceNames that replaces the names in the "CNVDetectionResul
```

press_047_R_2012_09_04_02_34_22_Sequoia_SN1.19.withoutCYP_48_Run_16_hg

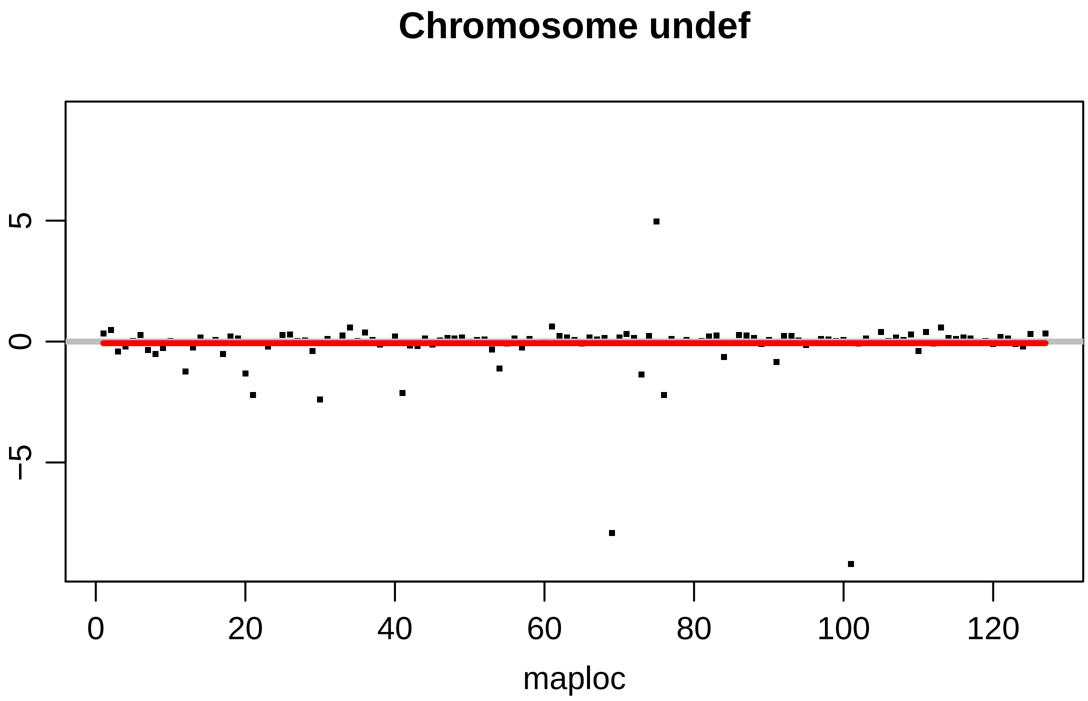


```
## [1] "/Users/gdemidov/Downloads/doc/Run_17_fin_05_qc.xls"

## Normalizing...

## Starting local modeling, please be patient...

## Reference sequence:  undef

## Starting segmentation algorithm...

## Using "fastseg" for segmentation.

## [1] ""
## [1] "/Users/gdemidov/Downloads/doc/Run_17_fin_05_qc.xls"
## [1] ""

## Segplot might not work because of special characters in the sample names. Use only A-Z,a-z and 0-9!
##  There is a hidden function cn.mops:::.replaceNames that replaces the names in the "CNVDetectionResul
```

press_048_R_2012_09_04_02_34_22_Sequoia_SN1.19.withoutCYP_48_Run_16_hg

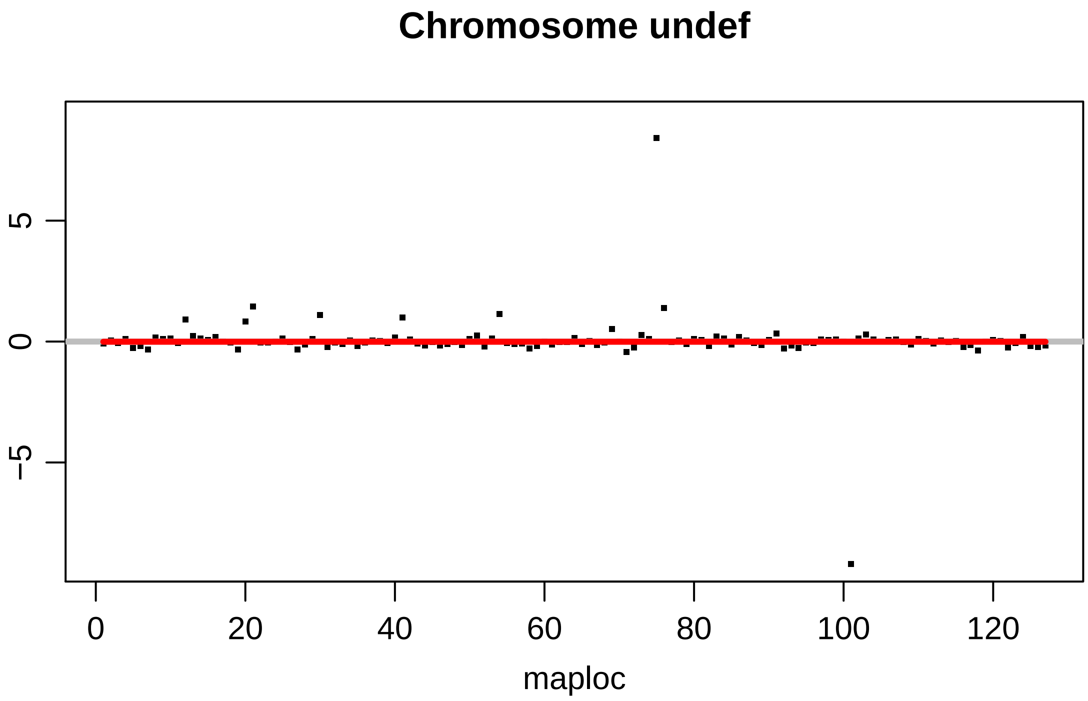


```
## Segplot might not work because of special characters in the sample names. Use only A-Z,a-z and 0-9!
##  There is a hidden function cn.mops:::.replaceNames that replaces the names in the "CNVDetectionResul
```

se_IonXpress_001_R_2012_09_07_19_18_37_Sequoia_SN1.21_Run_17_hg19_v3.s

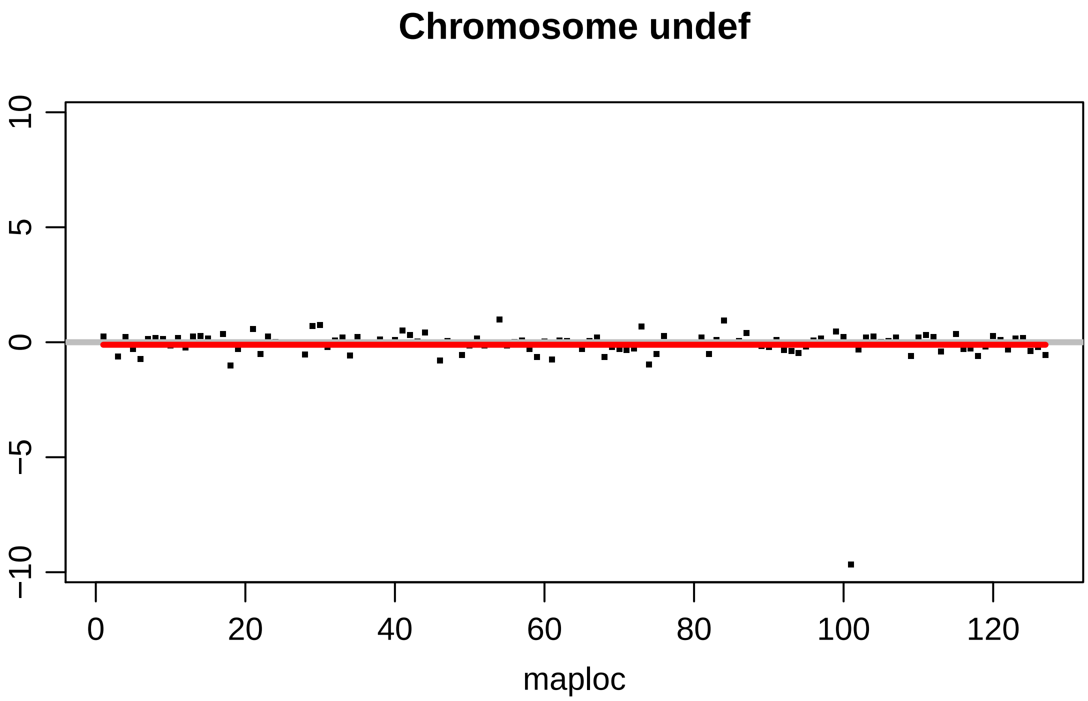

```
## Segplot might not work because of special characters in the sample names. Use only A-Z,a-z and 0-9!
##  There is a hidden function cn.mops:::.replaceNames that replaces the names in the "CNVDetectionResul
```

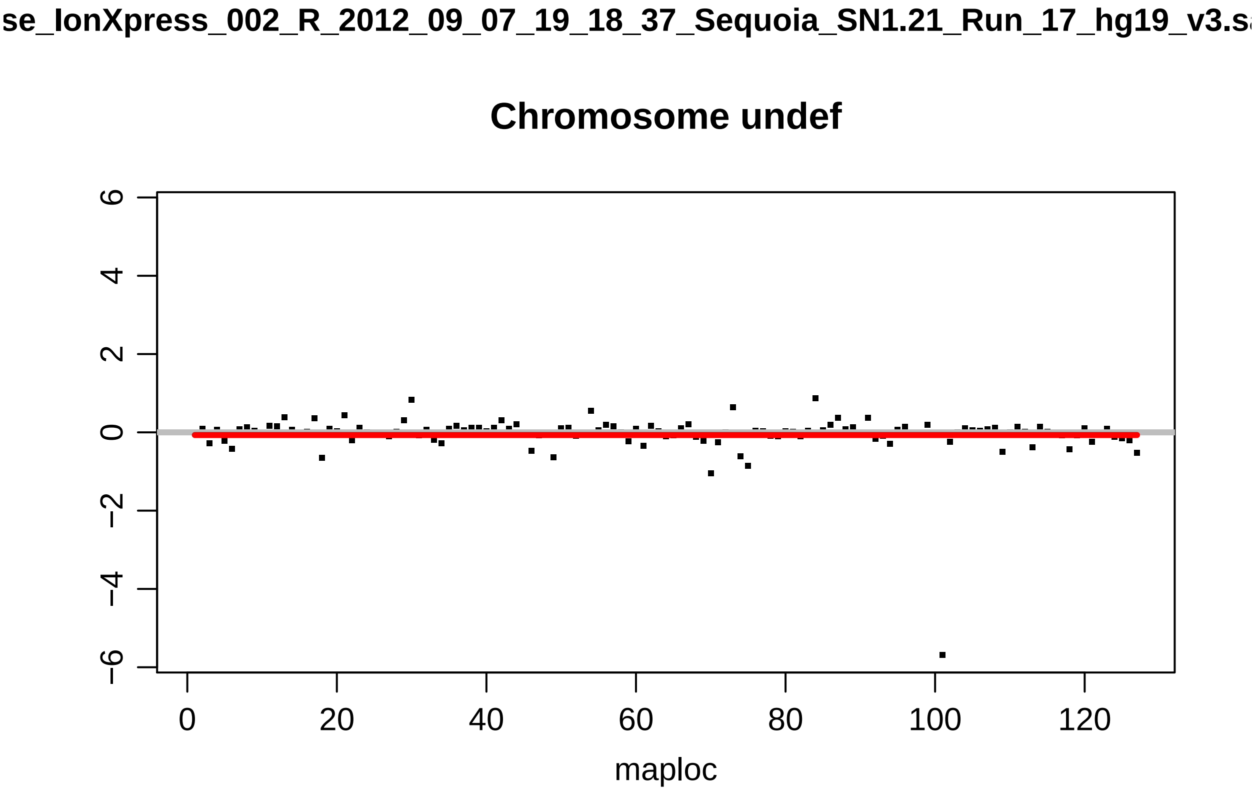


```
## Segplot might not work because of special characters in the sample names. Use only A-Z,a-z and 0-9!
##  There is a hidden function cn.mops:::.replaceNames that replaces the names in the "CNVDetectionResul
```

se_IonXpress_003_R_2012_09_07_19_18_37_Sequoia_SN1.21_Run_17_hg19_v3.s

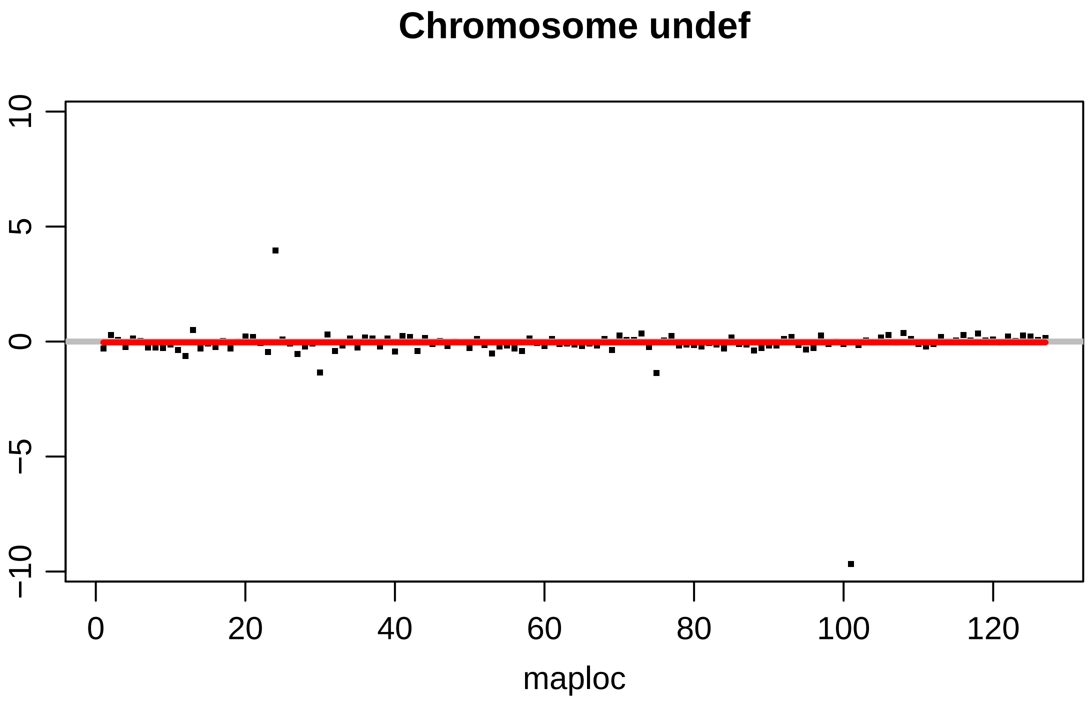


```
## Segplot might not work because of special characters in the sample names. Use only A-Z,a-z and 0-9!
##  There is a hidden function cn.mops:::.replaceNames that replaces the names in the "CNVDetectionResul
```

se_IonXpress_004_R_2012_09_07_19_18_37_Sequoia_SN1.21_Run_17_hg19_v3.s

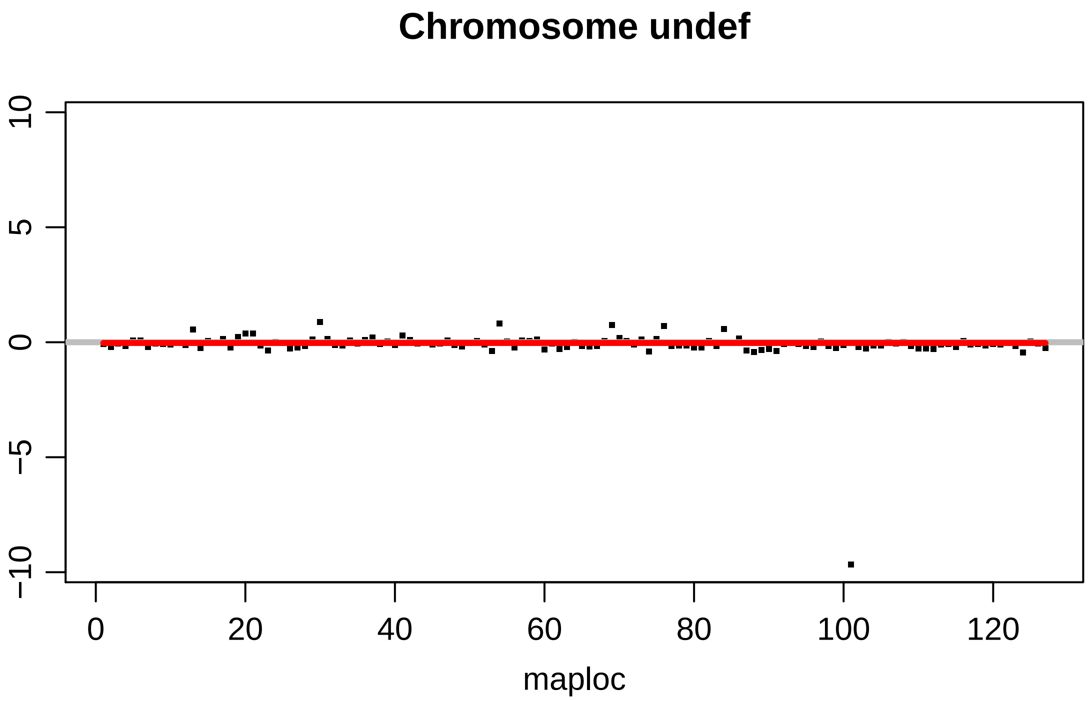

```
## Segplot might not work because of special characters in the sample names. Use only A-Z,a-z and 0-9!
##  There is a hidden function cn.mops:::.replaceNames that replaces the names in the "CNVDetectionResul
```

se_IonXpress_006_R_2012_09_07_19_18_37_Sequoia_SN1.21_Run_17_hg19_v3.s

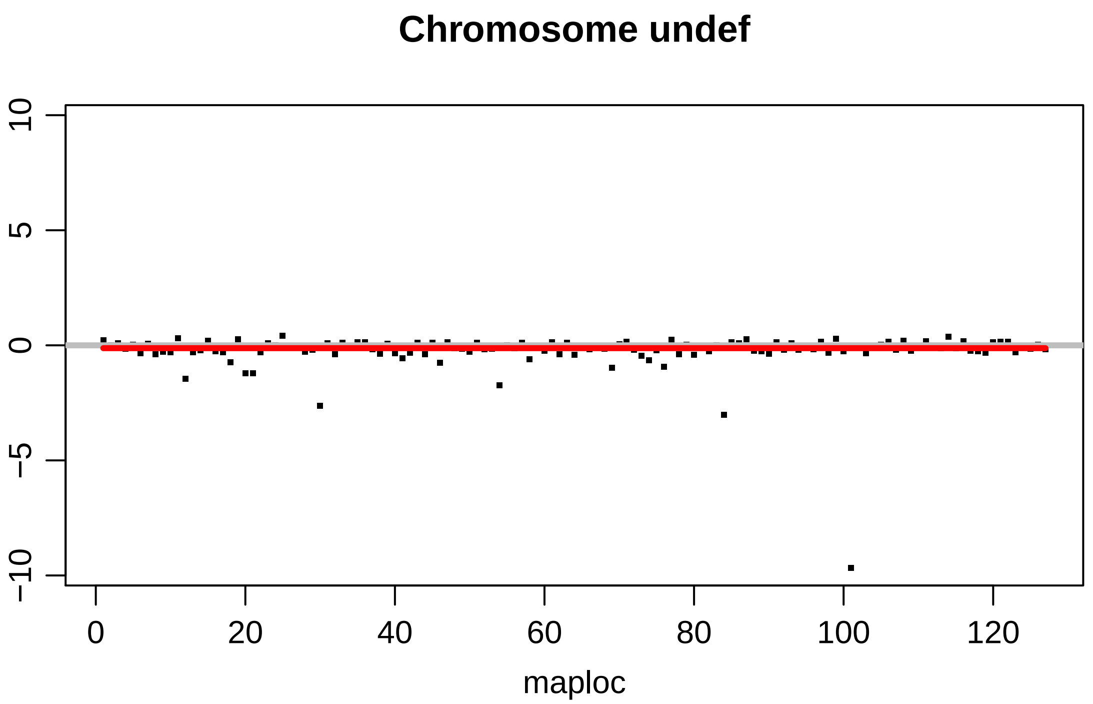


```
## Segplot might not work because of special characters in the sample names. Use only A-Z,a-z and 0-9!
##  There is a hidden function cn.mops:::.replaceNames that replaces the names in the "CNVDetectionResul
```

se_IonXpress_007_R_2012_09_07_19_18_37_Sequoia_SN1.21_Run_17_hg19_v3.s

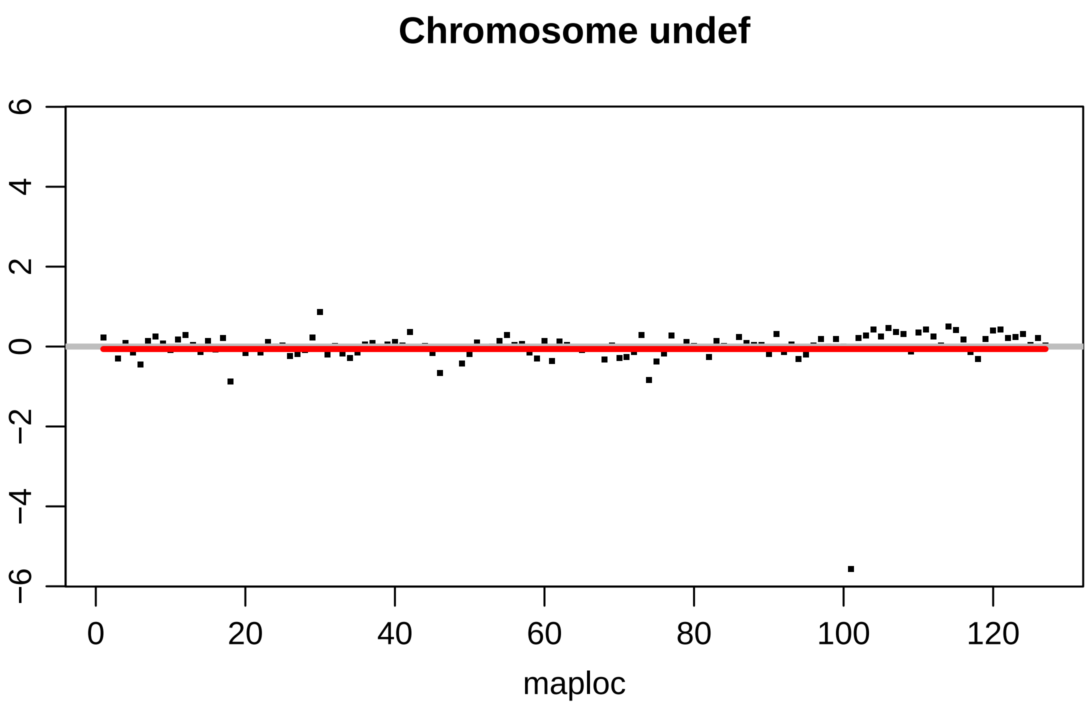


```
## Segplot might not work because of special characters in the sample names. Use only A-Z,a-z and 0-9!
##  There is a hidden function cn.mops:::.replaceNames that replaces the names in the "CNVDetectionResu
```

se_IonXpress_008_R_2012_09_07_19_18_37_Sequoia_SN1.21_Run_17_hg19_v3.s

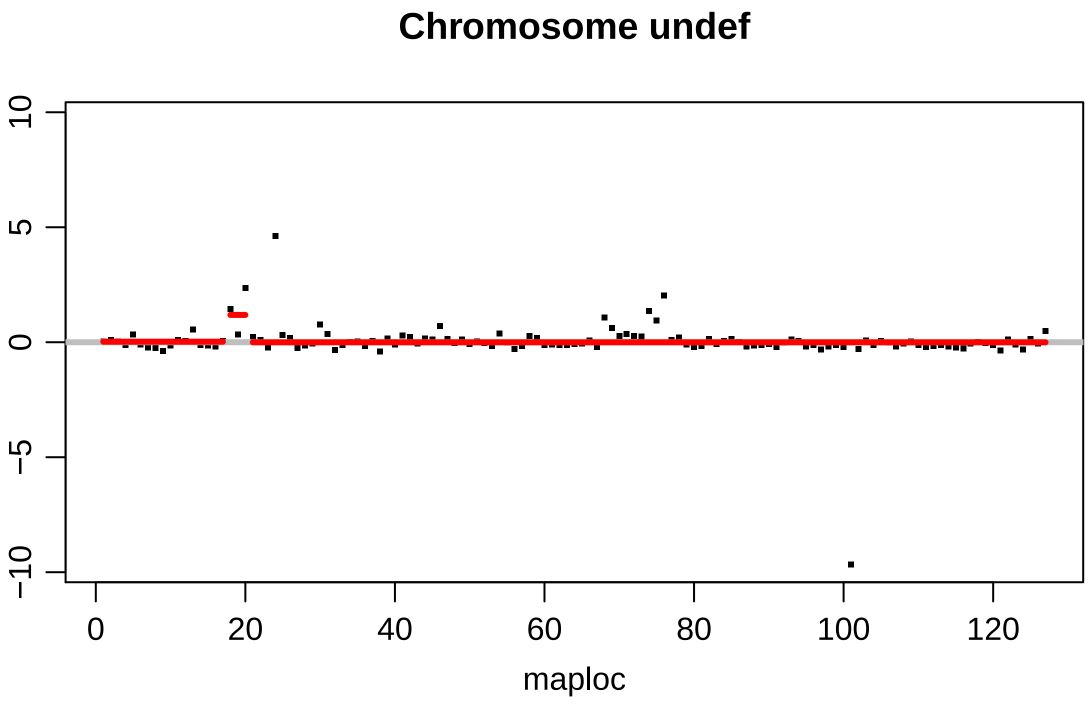

```
## Segplot might not work because of special characters in the sample names. Use only A-Z,a-z and 0-9!
##  There is a hidden function cn.mops:::.replaceNames that replaces the names in the "CNVDetectionResul
```

se_IonXpress_009_R_2012_09_07_19_18_37_Sequoia_SN1.21_Run_17_hg19_v3.s

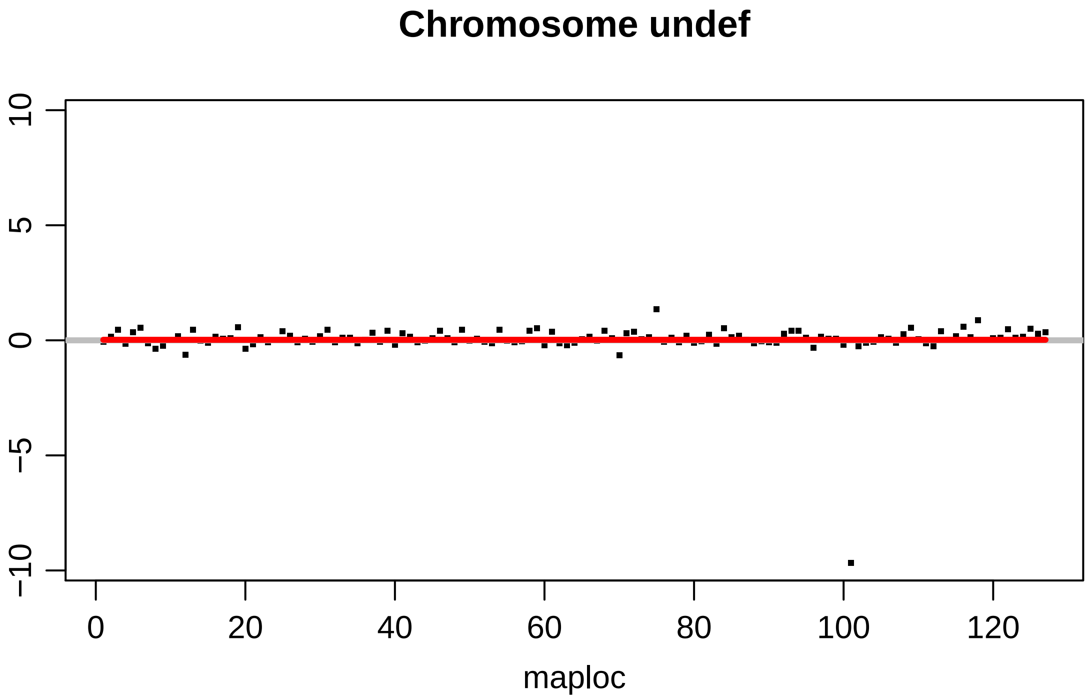


```
## Segplot might not work because of special characters in the sample names. Use only A-Z,a-z and 0-9!
##  There is a hidden function cn.mops:::.replaceNames that replaces the names in the "CNVDetectionResul
```

se_IonXpress_011_R_2012_09_07_19_18_37_Sequoia_SN1.21_Run_17_hg19_v3.s

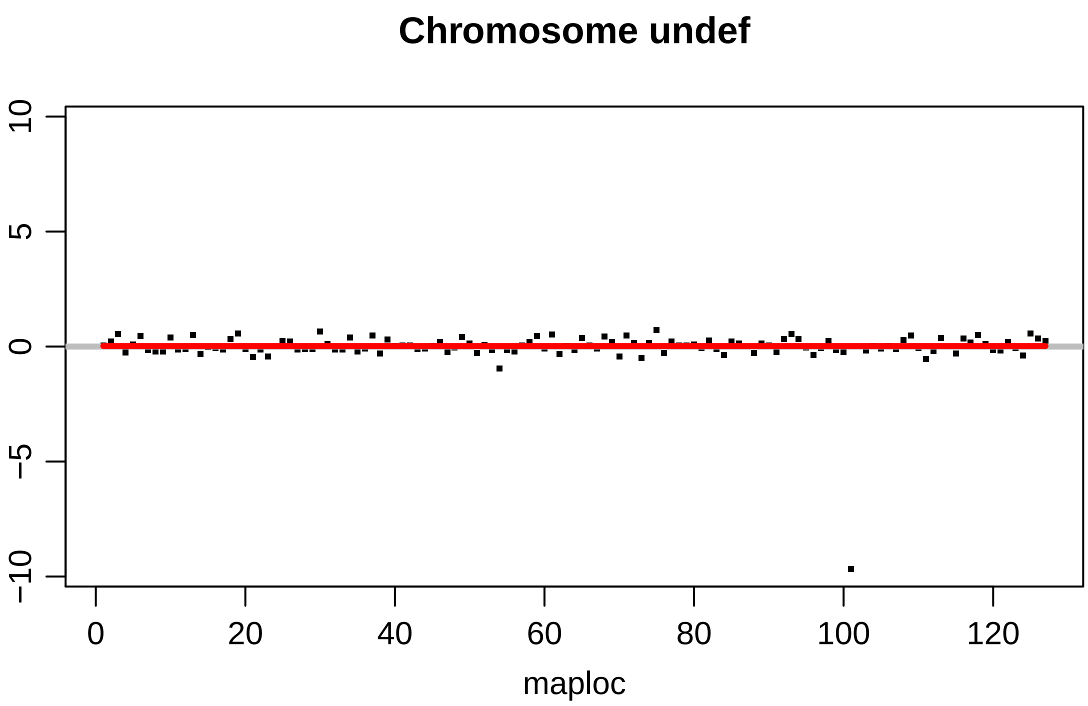


```
## Segplot might not work because of special characters in the sample names. Use only A-Z,a-z and 0-9!
##  There is a hidden function cn.mops:::.replaceNames that replaces the names in the "CNVDetectionResul
```

se_IonXpress_013_R_2012_09_07_19_18_37_Sequoia_SN1.21_Run_17_hg19_v3.s

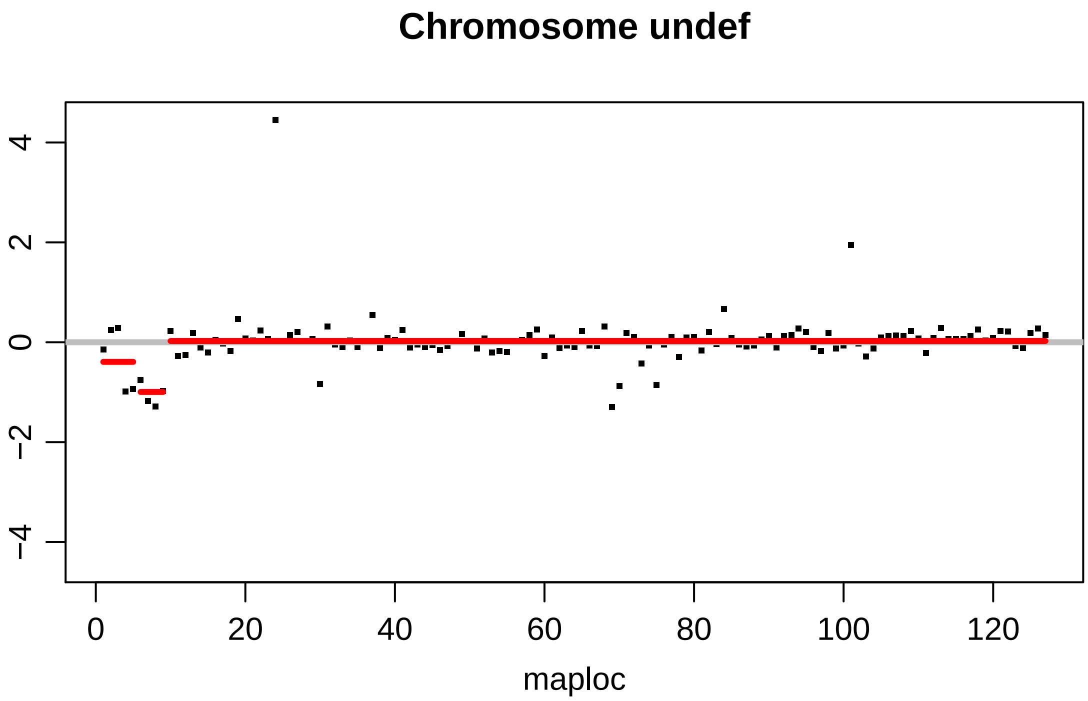

```
## Segplot might not work because of special characters in the sample names. Use only A-Z,a-z and 0-9!
##  There is a hidden function cn.mops:::.replaceNames that replaces the names in the "CNVDetectionResul
```

se_IonXpress_014_R_2012_09_07_19_18_37_Sequoia_SN1.21_Run_17_hg19_v3.s

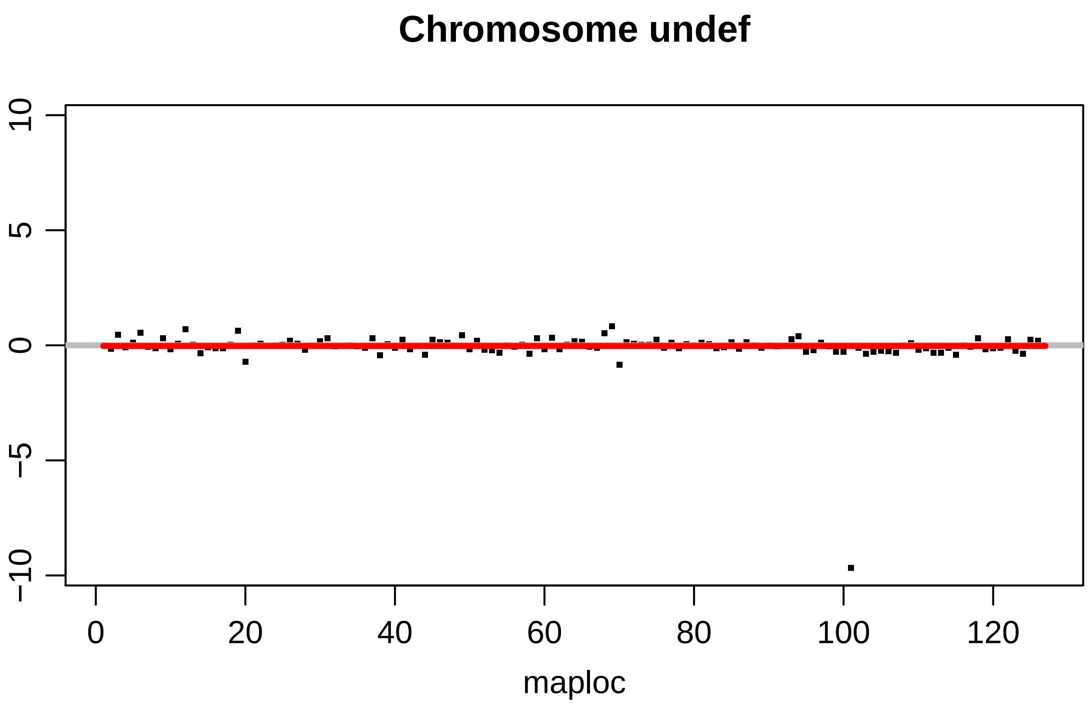


```
## Segplot might not work because of special characters in the sample names. Use only A-Z,a-z and 0-9!
##  There is a hidden function cn.mops:::.replaceNames that replaces the names in the "CNVDetectionResul
```

se_IonXpress_015_R_2012_09_07_19_18_37_Sequoia_SN1.21_Run_17_hg19_v3.s

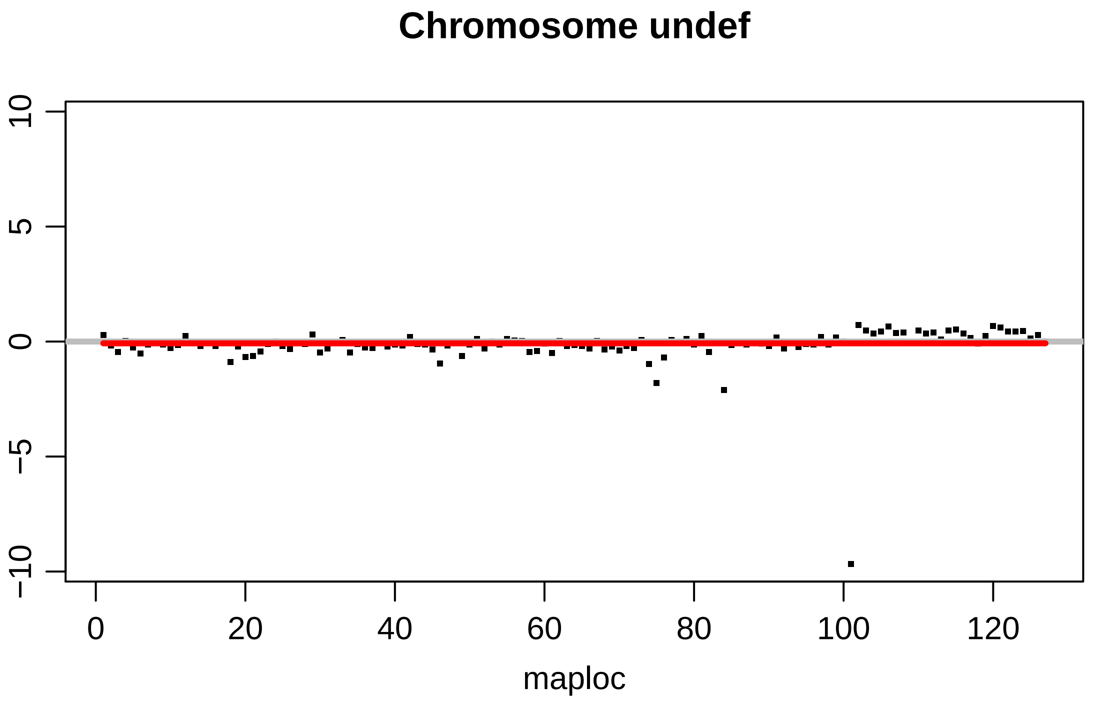


```
## Segplot might not work because of special characters in the sample names. Use only A-Z,a-z and 0-9!
##  There is a hidden function cn.mops:::.replaceNames that replaces the names in the "CNVDetectionResu
```

se_IonXpress_016_R_2012_09_07_19_18_37_Sequoia_SN1.21_Run_17_hg19_v3.s

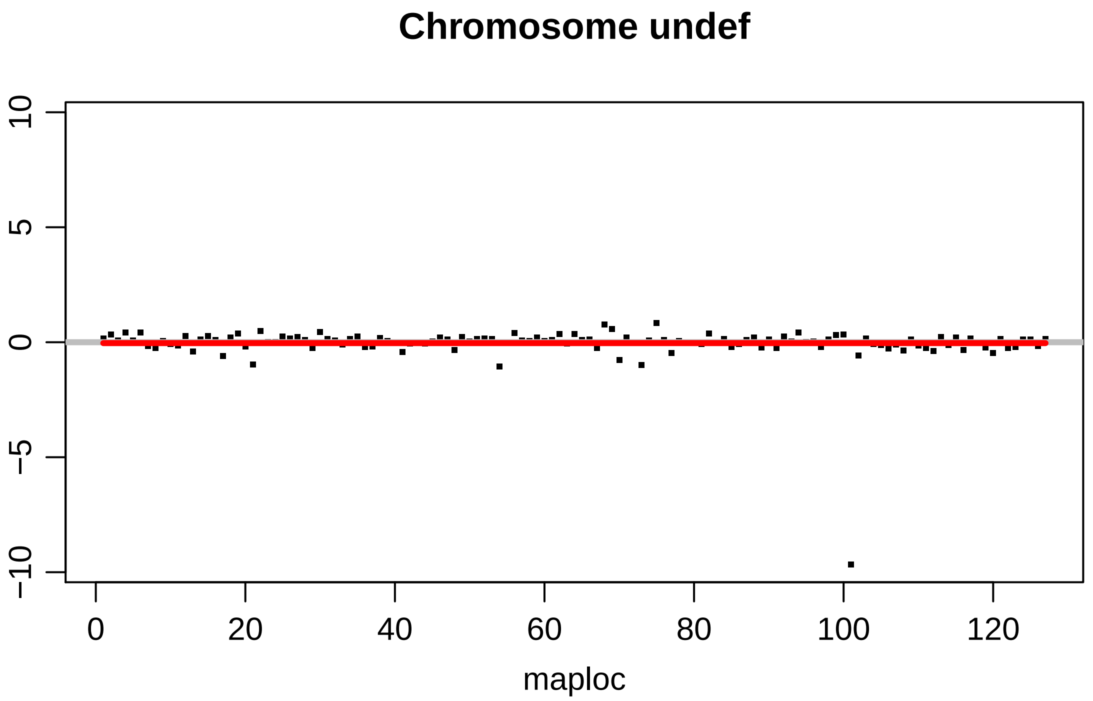

```
## Segplot might not work because of special characters in the sample names. Use only A-Z,a-z and 0-9!
##  There is a hidden function cn.mops:::.replaceNames that replaces the names in the "CNVDetectionResul
```

se_IonXpress_017_R_2012_09_07_19_18_37_Sequoia_SN1.21_Run_17_hg19_v3.s

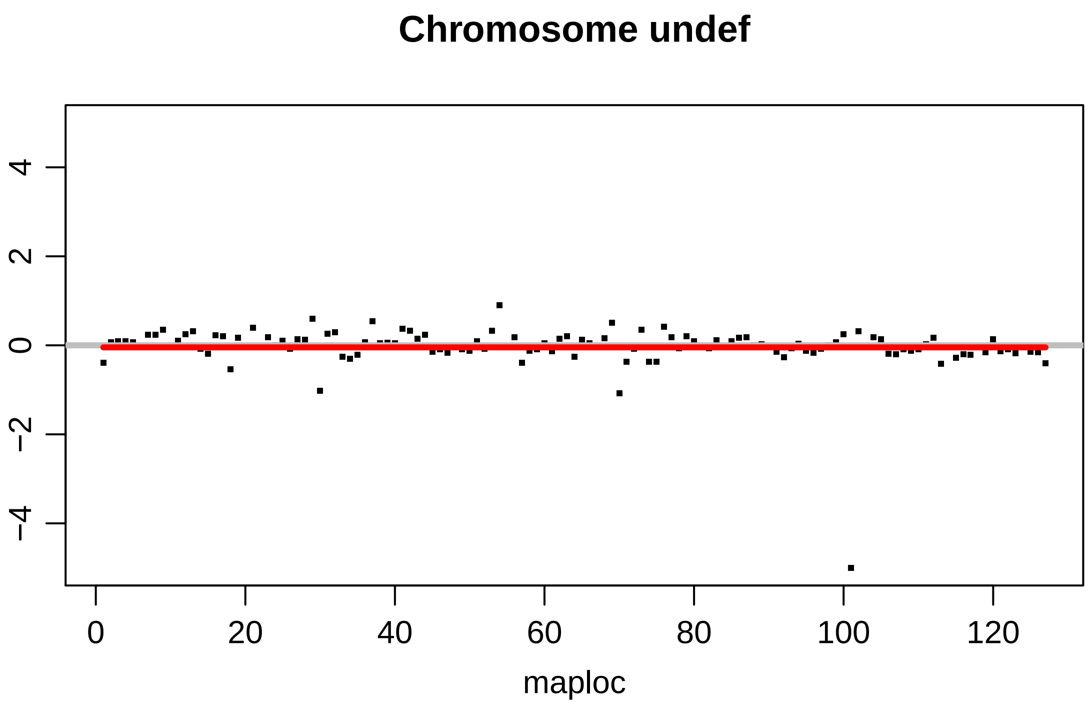


```
## Segplot might not work because of special characters in the sample names. Use only A-Z,a-z and 0-9!
##  There is a hidden function cn.mops:::.replaceNames that replaces the names in the "CNVDetectionResul
```

se_IonXpress_018_R_2012_09_07_19_18_37_Sequoia_SN1.21_Run_17_hg19_v3.s

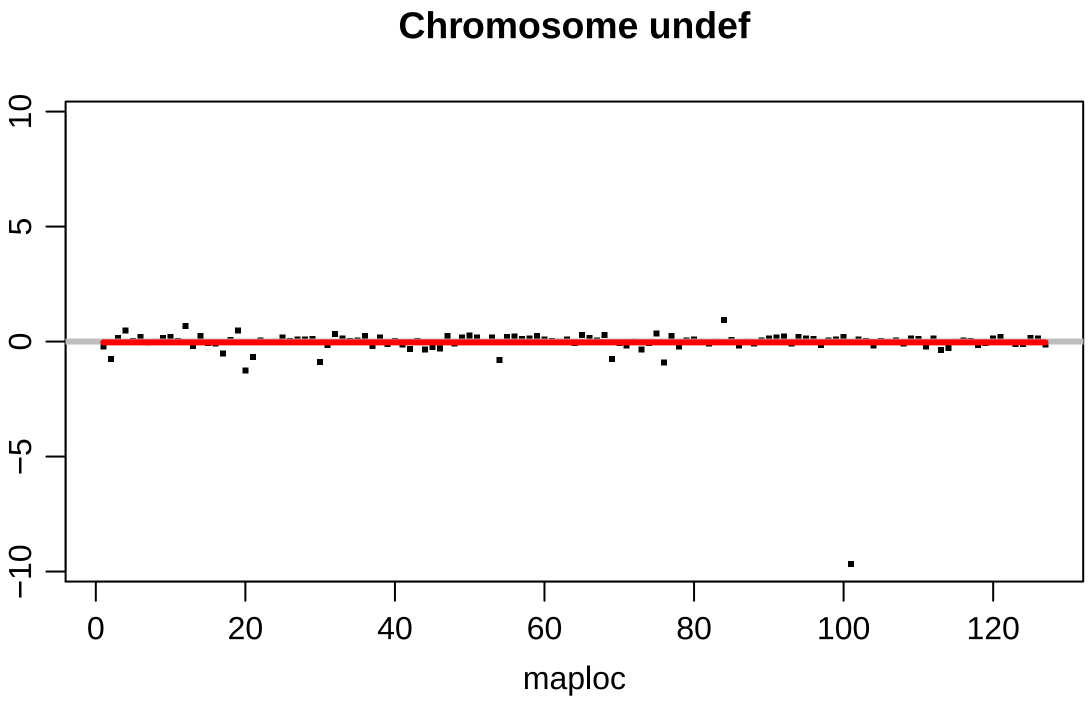


```
## Segplot might not work because of special characters in the sample names. Use only A-Z,a-z and 0-9!
##  There is a hidden function cn.mops:::.replaceNames that replaces the names in the "CNVDetectionResu
```

se_IonXpress_019_R_2012_09_07_19_18_37_Sequoia_SN1.21_Run_17_hg19_v3.s

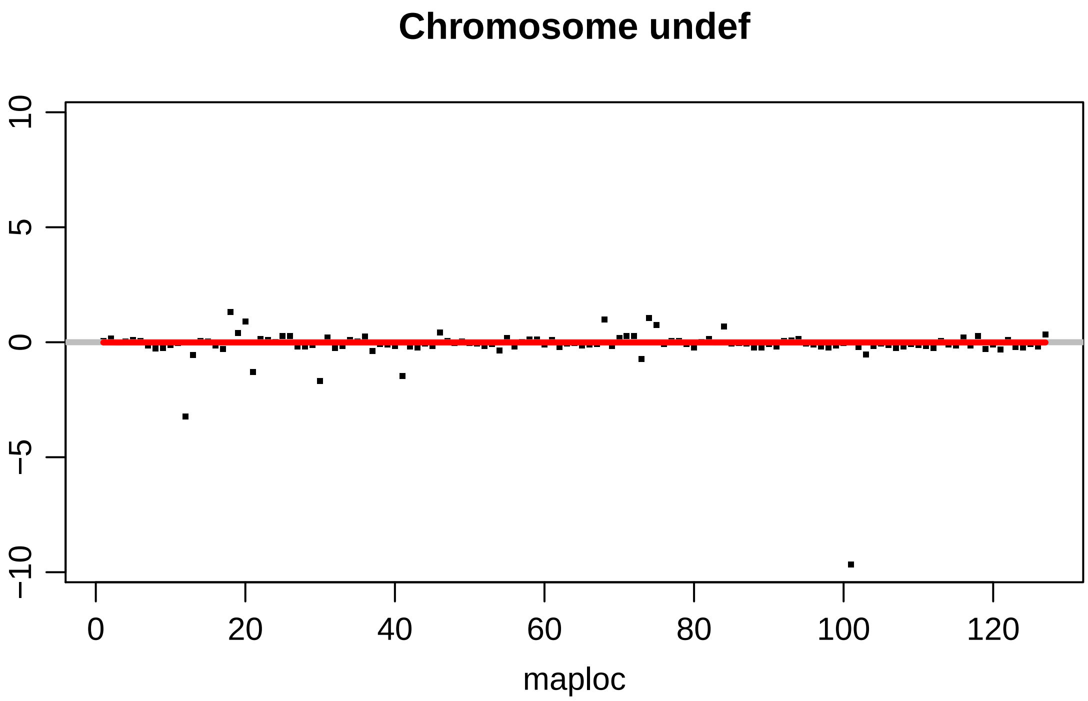

```
## Segplot might not work because of special characters in the sample names. Use only A-Z,a-z and 0-9!
##  There is a hidden function cn.mops:::.replaceNames that replaces the names in the "CNVDetectionResul
```

se_IonXpress_020_R_2012_09_07_19_18_37_Sequoia_SN1.21_Run_17_hg19_v3.s

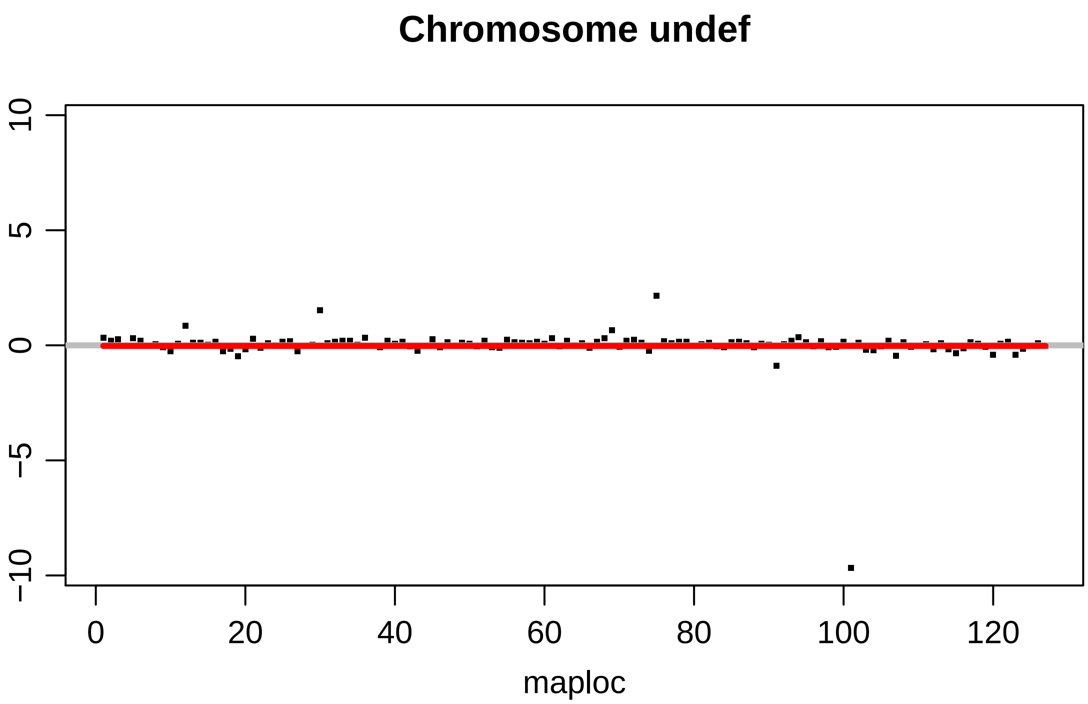


```
## Segplot might not work because of special characters in the sample names. Use only A-Z,a-z and 0-9!
##  There is a hidden function cn.mops:::.replaceNames that replaces the names in the "CNVDetectionResul
```

se_IonXpress_021_R_2012_09_07_19_18_37_Sequoia_SN1.21_Run_17_hg19_v3.s

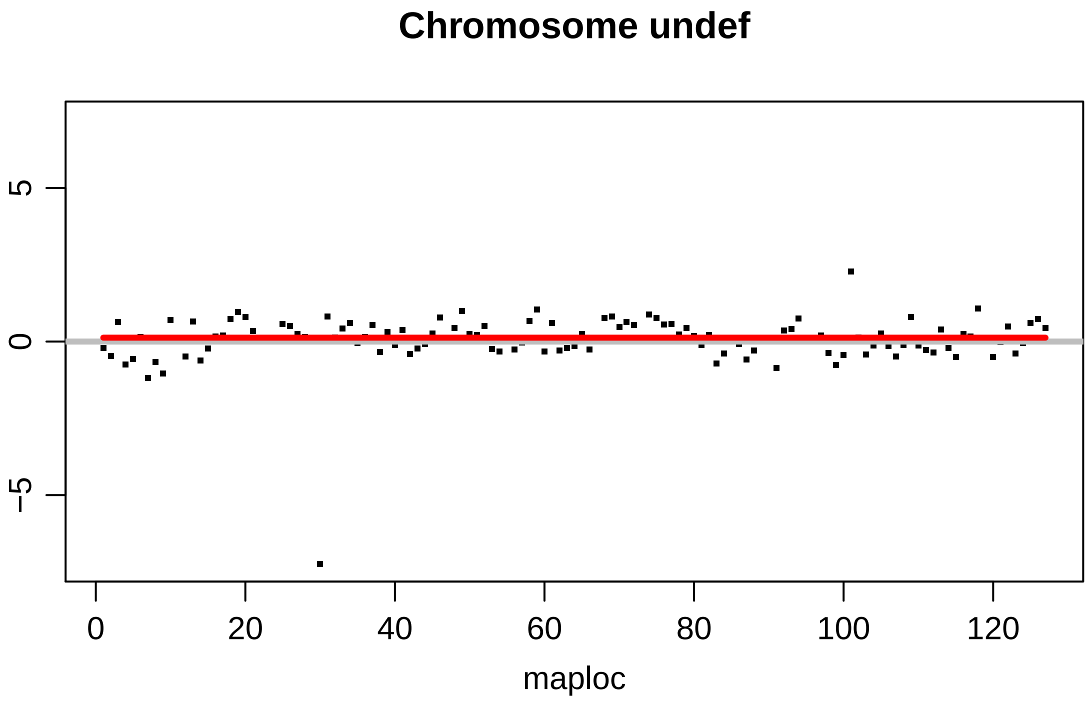


```
## Segplot might not work because of special characters in the sample names. Use only A-Z,a-z and 0-9!
##  There is a hidden function cn.mops:::.replaceNames that replaces the names in the "CNVDetectionResu
```

se_IonXpress_023_R_2012_09_07_19_18_37_Sequoia_SN1.21_Run_17_hg19_v3.s

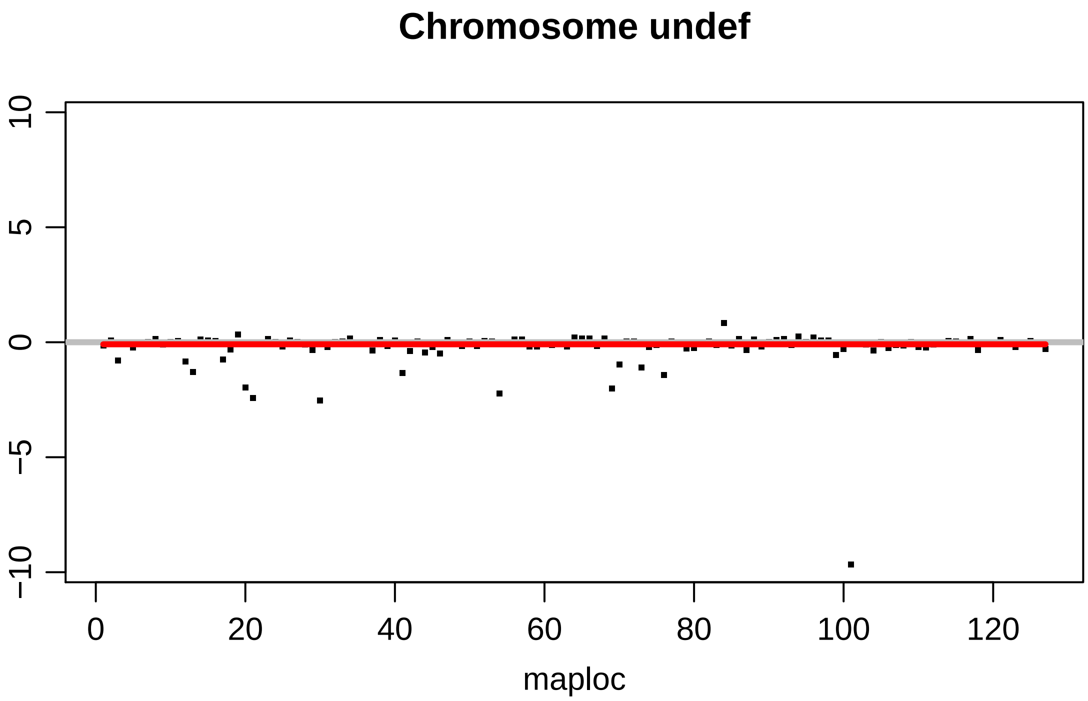

```
## Segplot might not work because of special characters in the sample names. Use only A-Z,a-z and 0-9!
##  There is a hidden function cn.mops:::.replaceNames that replaces the names in the "CNVDetectionResul
```

se_IonXpress_024_R_2012_09_07_19_18_37_Sequoia_SN1.21_Run_17_hg19_v3.s

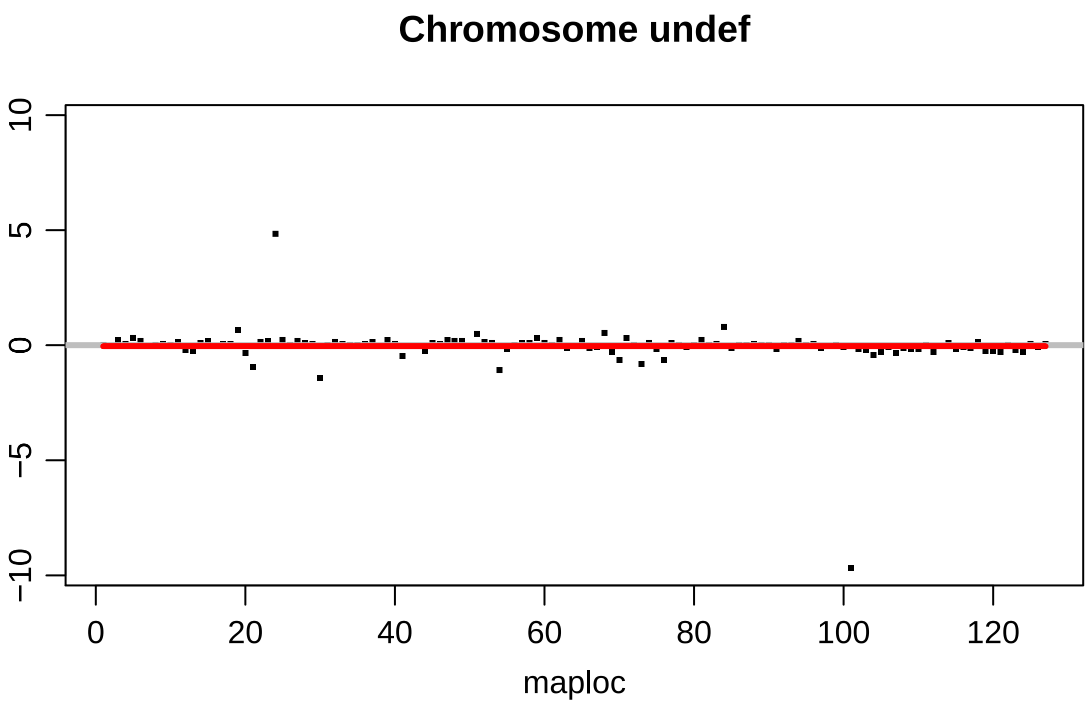


```
## Segplot might not work because of special characters in the sample names. Use only A-Z,a-z and 0-9!
##  There is a hidden function cn.mops:::.replaceNames that replaces the names in the "CNVDetectionResul
```

se_IonXpress_025_R_2012_09_07_19_18_37_Sequoia_SN1.21_Run_17_hg19_v3.s

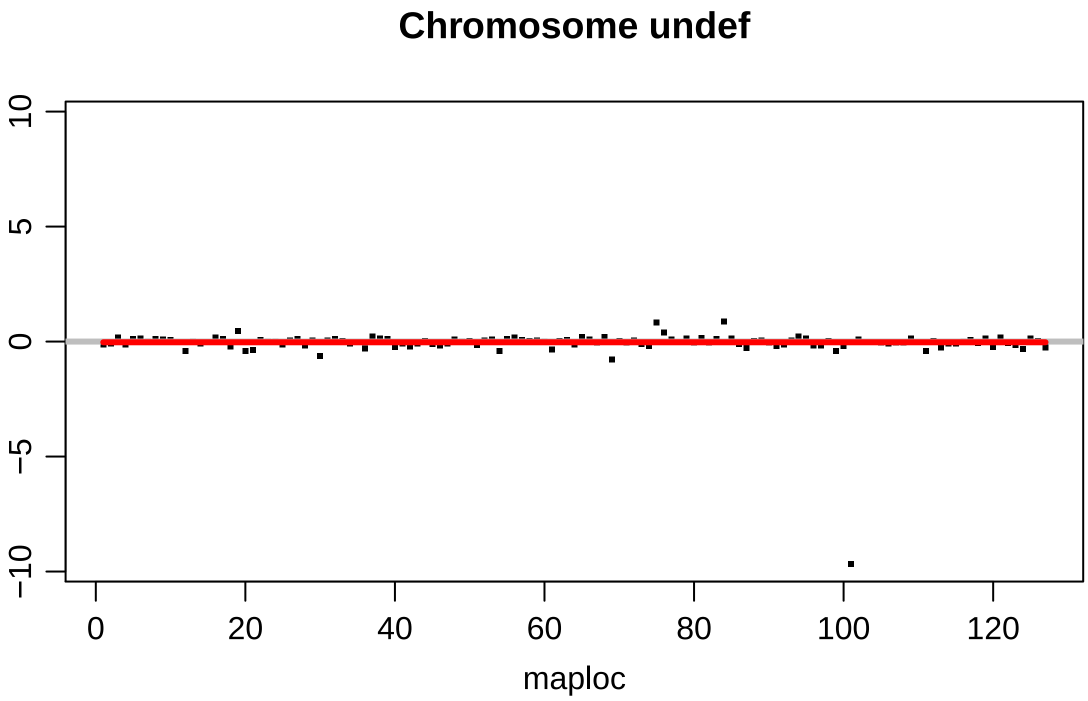


```
## Segplot might not work because of special characters in the sample names. Use only A-Z,a-z and 0-9!
##  There is a hidden function cn.mops:::.replaceNames that replaces the names in the "CNVDetectionResul
```

se_IonXpress_026_R_2012_09_07_19_18_37_Sequoia_SN1.21_Run_17_hg19_v3.s

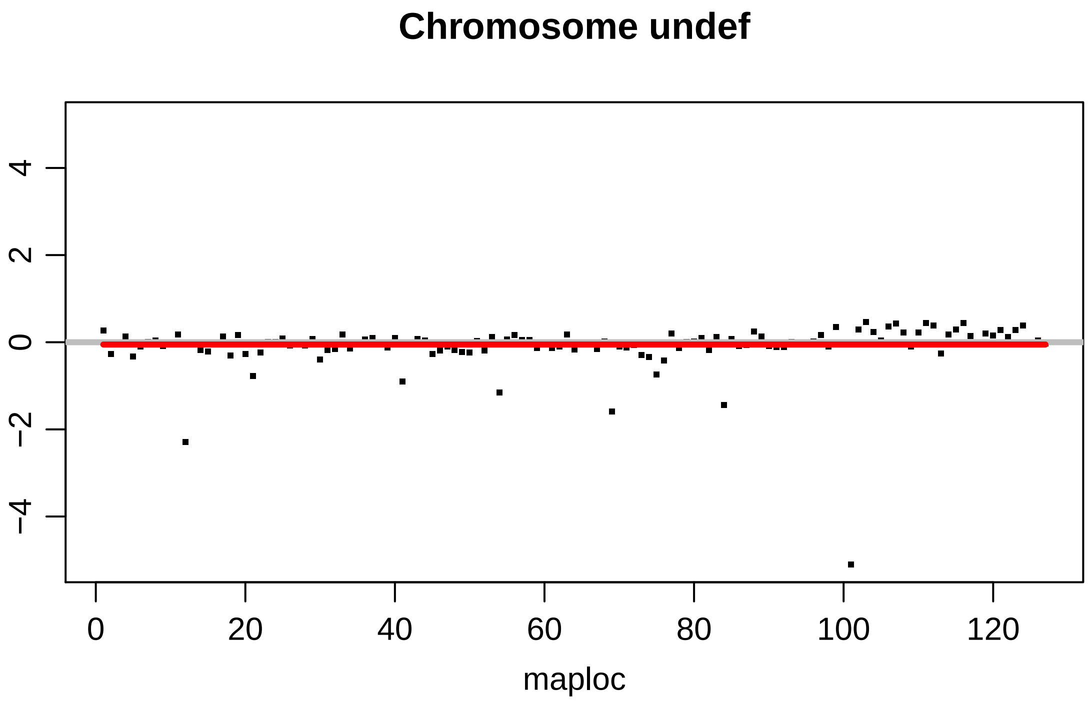

```
## Segplot might not work because of special characters in the sample names. Use only A-Z,a-z and 0-9!
##  There is a hidden function cn.mops:::.replaceNames that replaces the names in the "CNVDetectionResul
```

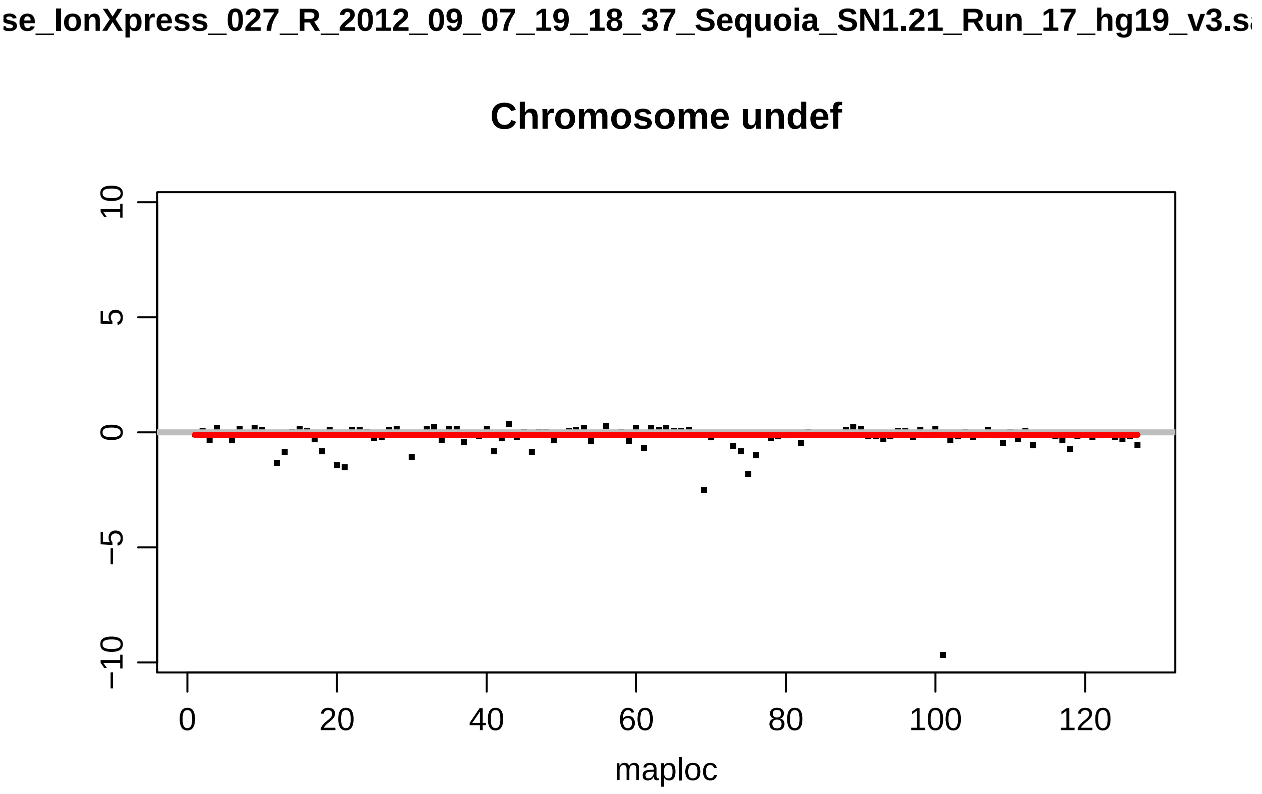


```
## Segplot might not work because of special characters in the sample names. Use only A-Z,a-z and 0-9!
##  There is a hidden function cn.mops:::.replaceNames that replaces the names in the "CNVDetectionResul
```

se_IonXpress_028_R_2012_09_07_19_18_37_Sequoia_SN1.21_Run_17_hg19_v3.s

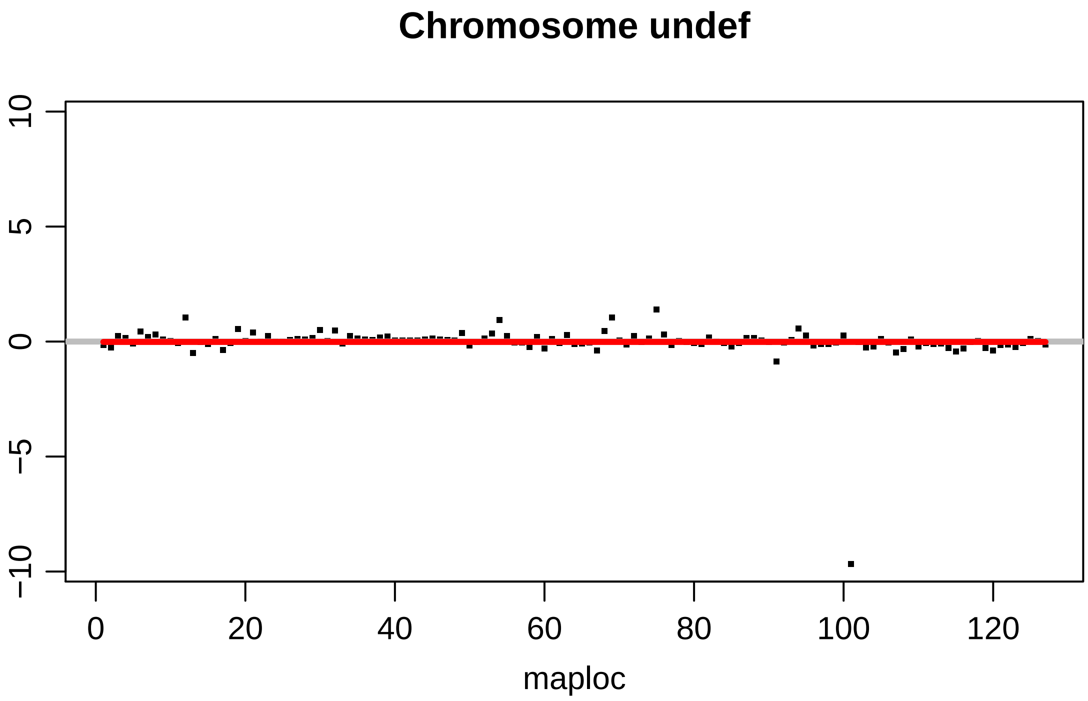


```
## Segplot might not work because of special characters in the sample names. Use only A-Z,a-z and 0-9!
##  There is a hidden function cn.mops:::.replaceNames that replaces the names in the "CNVDetectionResul
```

se_IonXpress_029_R_2012_09_07_19_18_37_Sequoia_SN1.21_Run_17_hg19_v3.s

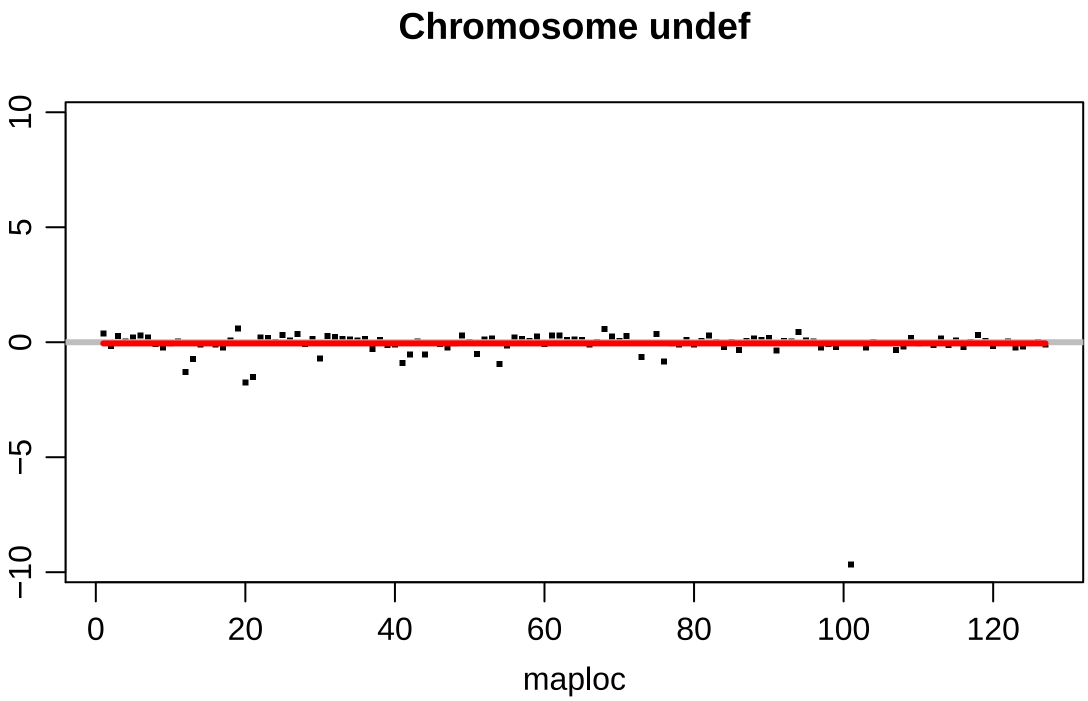

```
## Segplot might not work because of special characters in the sample names. Use only A-Z,a-z and 0-9!
##  There is a hidden function cn.mops:::.replaceNames that replaces the names in the "CNVDetectionResul
```

se_IonXpress_030_R_2012_09_07_19_18_37_Sequoia_SN1.21_Run_17_hg19_v3.s

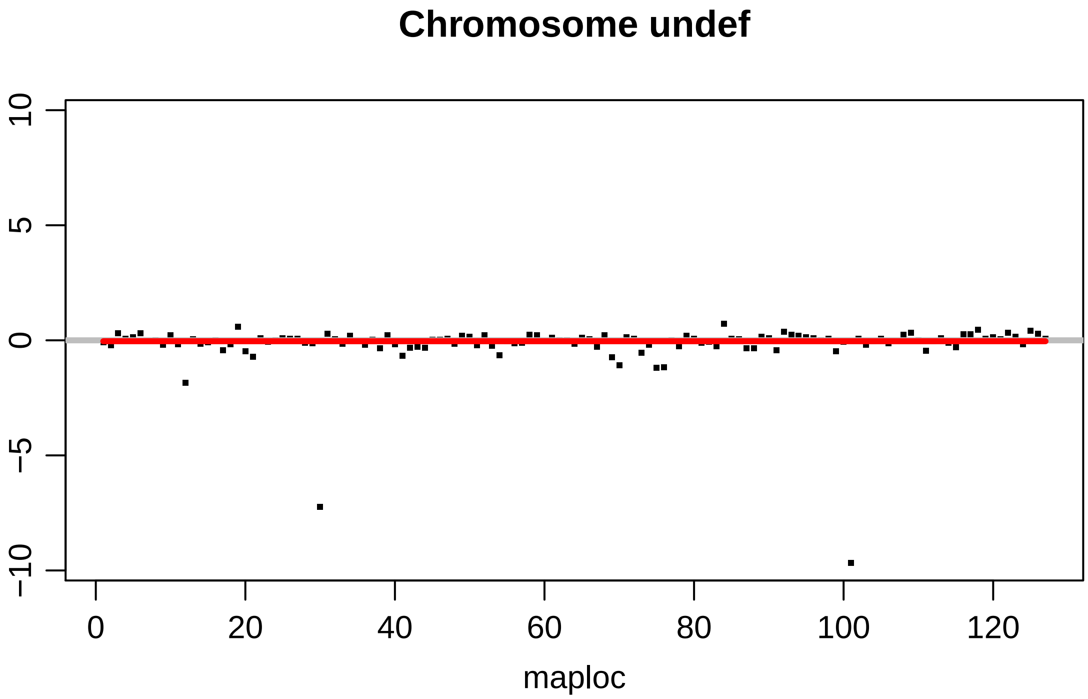


```
## Segplot might not work because of special characters in the sample names. Use only A-Z,a-z and 0-9!
##  There is a hidden function cn.mops:::.replaceNames that replaces the names in the "CNVDetectionResul
```

se_IonXpress_031_R_2012_09_07_19_18_37_Sequoia_SN1.21_Run_17_hg19_v3.s

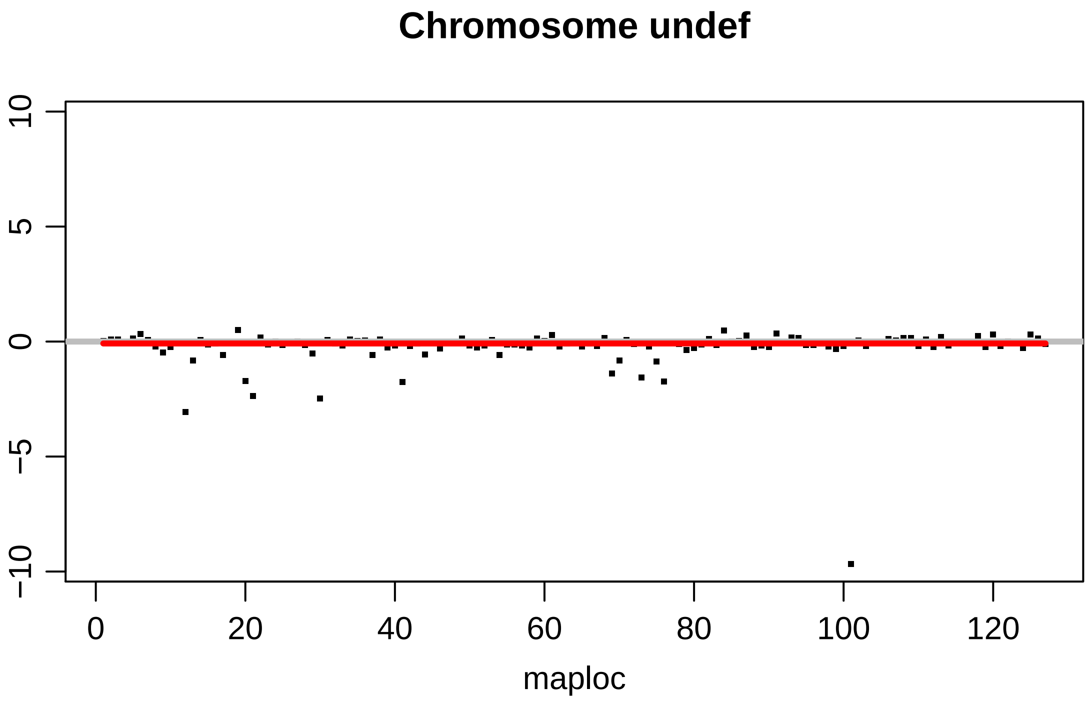


```
## Segplot might not work because of special characters in the sample names. Use only A-Z,a-z and 0-9!
##  There is a hidden function cn.mops:::.replaceNames that replaces the names in the "CNVDetectionResul
```

se_IonXpress_032_R_2012_09_07_19_18_37_Sequoia_SN1.21_Run_17_hg19_v3.s

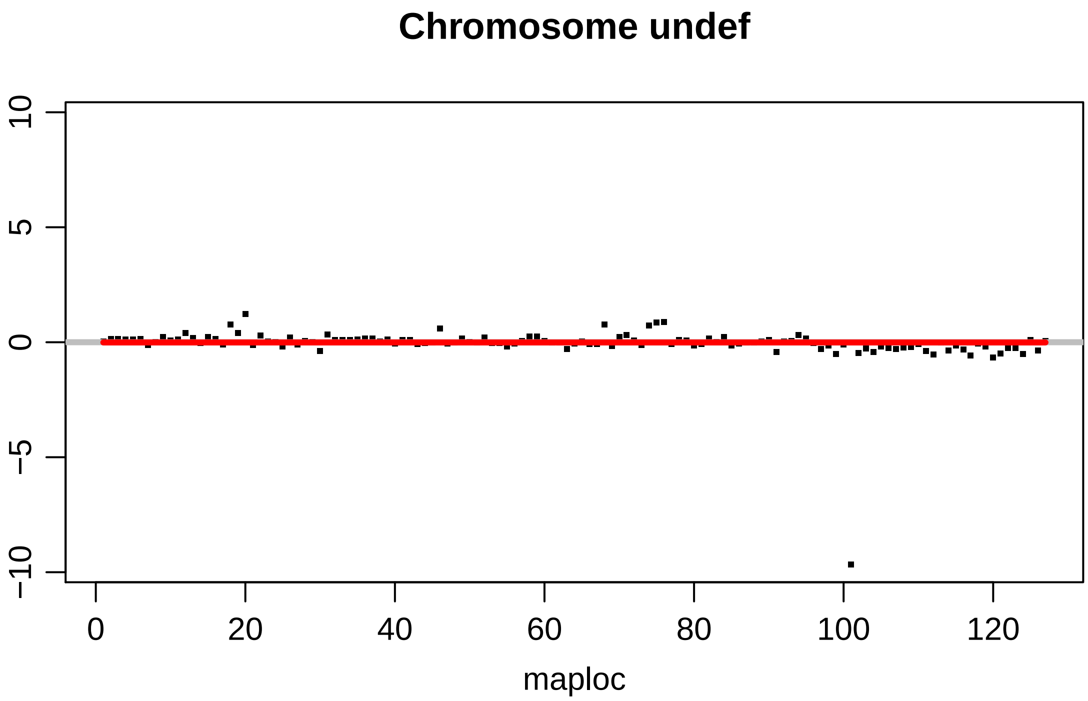

```
## Segplot might not work because of special characters in the sample names. Use only A-Z,a-z and 0-9!
##  There is a hidden function cn.mops:::.replaceNames that replaces the names in the "CNVDetectionResul
```

se_IonXpress_033_R_2012_09_07_19_18_37_Sequoia_SN1.21_Run_17_hg19_v3.s

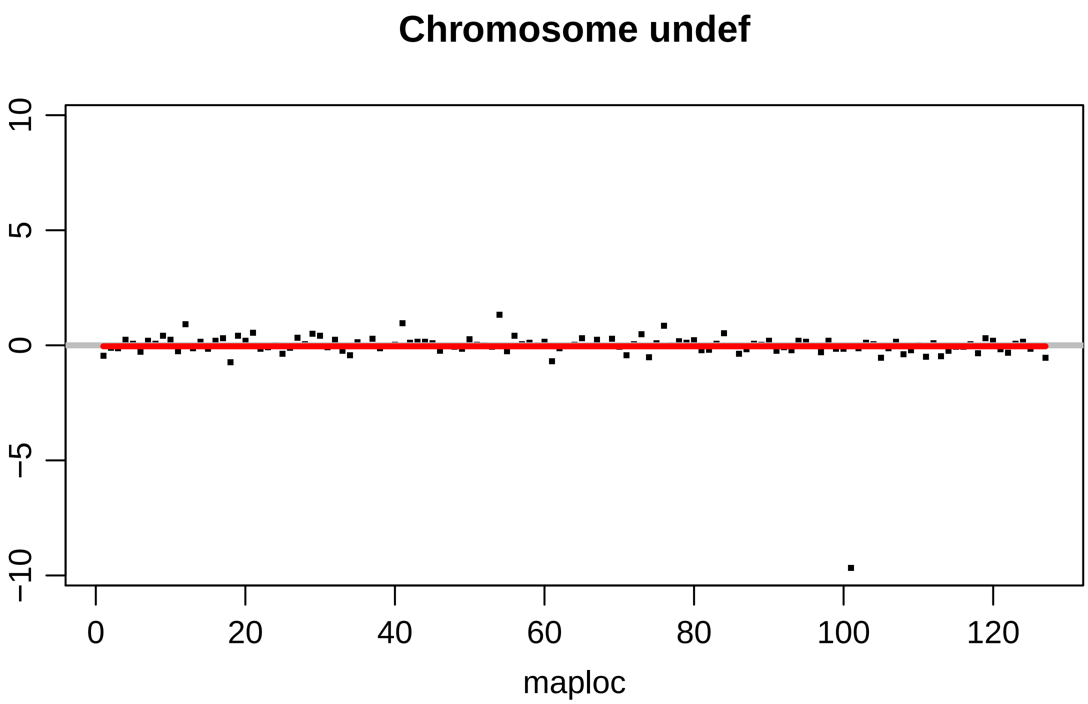


```
## Segplot might not work because of special characters in the sample names. Use only A-Z,a-z and 0-9!
##  There is a hidden function cn.mops:::.replaceNames that replaces the names in the "CNVDetectionResul
```

se_IonXpress_034_R_2012_09_07_19_18_37_Sequoia_SN1.21_Run_17_hg19_v3.s

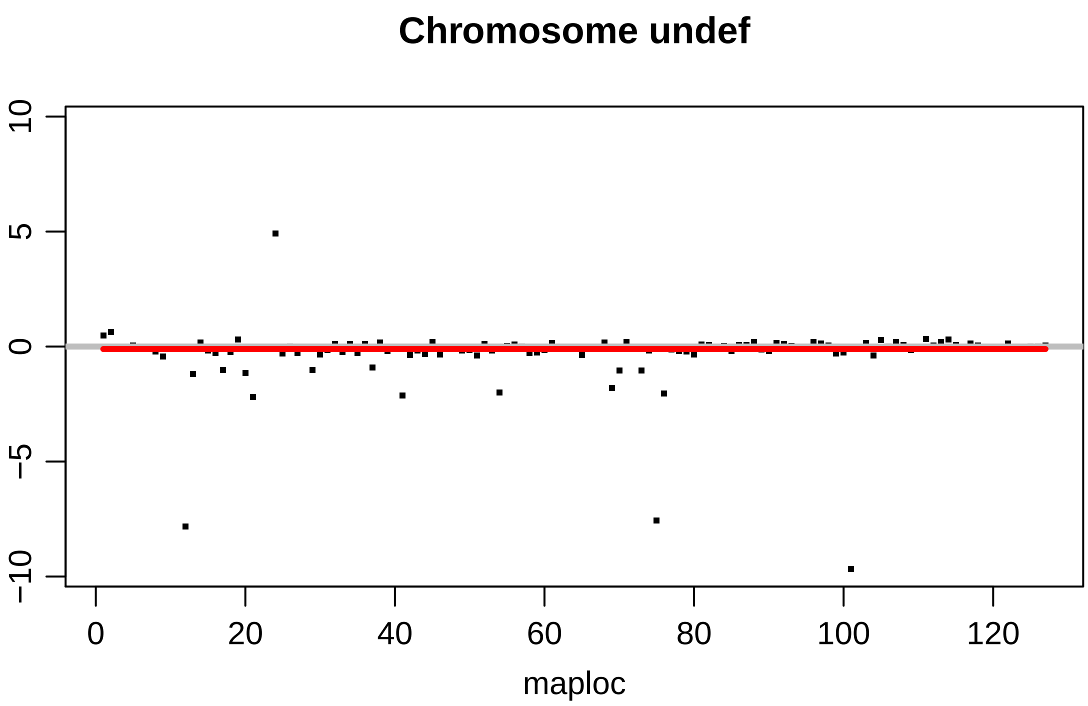


```
## Segplot might not work because of special characters in the sample names. Use only A-Z,a-z and 0-9!
##  There is a hidden function cn.mops:::.replaceNames that replaces the names in the "CNVDetectionResu
```

se_IonXpress_036_R_2012_09_07_19_18_37_Sequoia_SN1.21_Run_17_hg19_v3.s

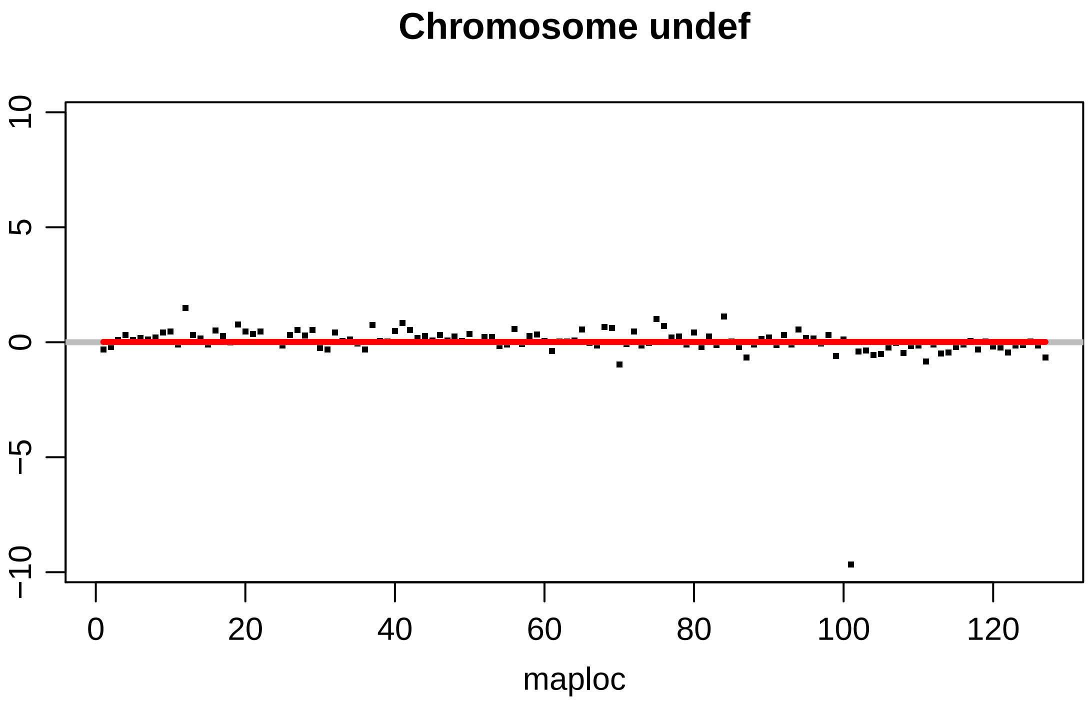

```
## Segplot might not work because of special characters in the sample names. Use only A-Z,a-z and 0-9!
##  There is a hidden function cn.mops:::.replaceNames that replaces the names in the "CNVDetectionResul
```

se_IonXpress_037_R_2012_09_07_19_18_37_Sequoia_SN1.21_Run_17_hg19_v3.s

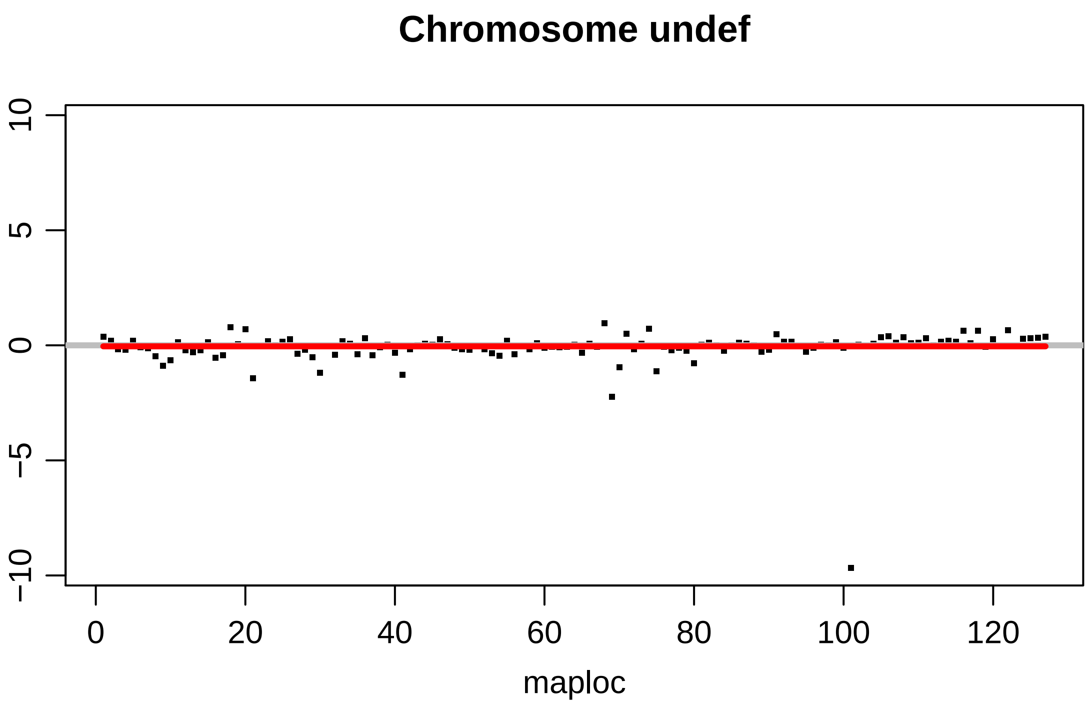


```
## Segplot might not work because of special characters in the sample names. Use only A-Z,a-z and 0-9!
##  There is a hidden function cn.mops:::.replaceNames that replaces the names in the "CNVDetectionResul
```

se_IonXpress_038_R_2012_09_07_19_18_37_Sequoia_SN1.21_Run_17_hg19_v3.s

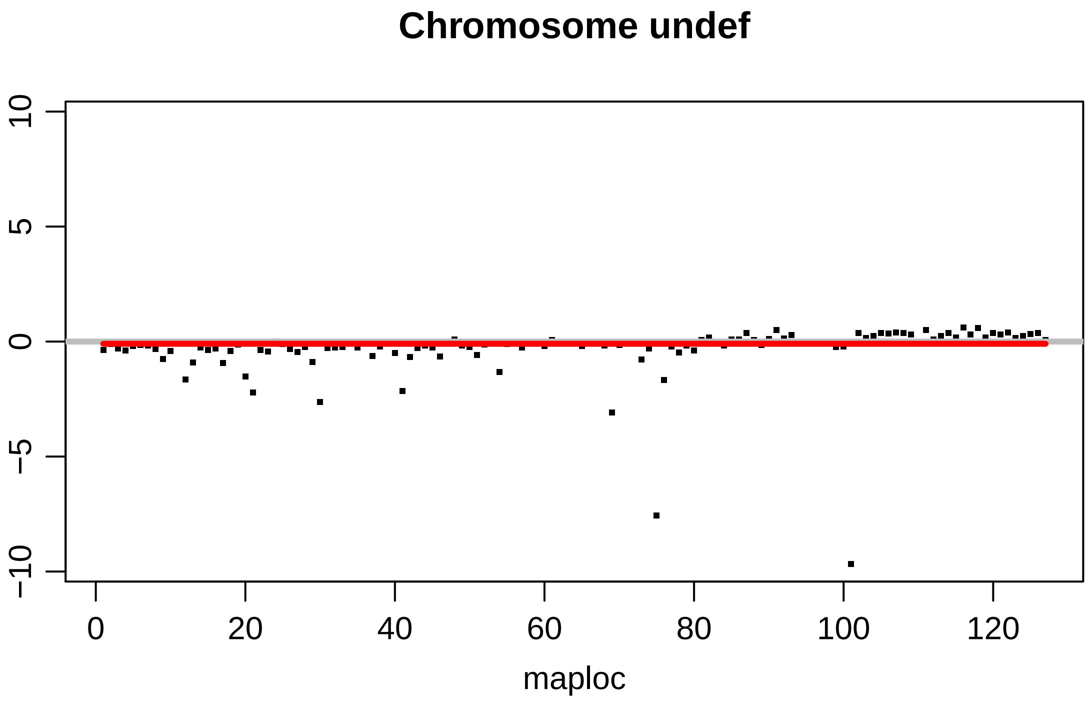


```
## Segplot might not work because of special characters in the sample names. Use only A-Z,a-z and 0-9!
##  There is a hidden function cn.mops:::.replaceNames that replaces the names in the "CNVDetectionResul
```

se_IonXpress_039_R_2012_09_07_19_18_37_Sequoia_SN1.21_Run_17_hg19_v3.s

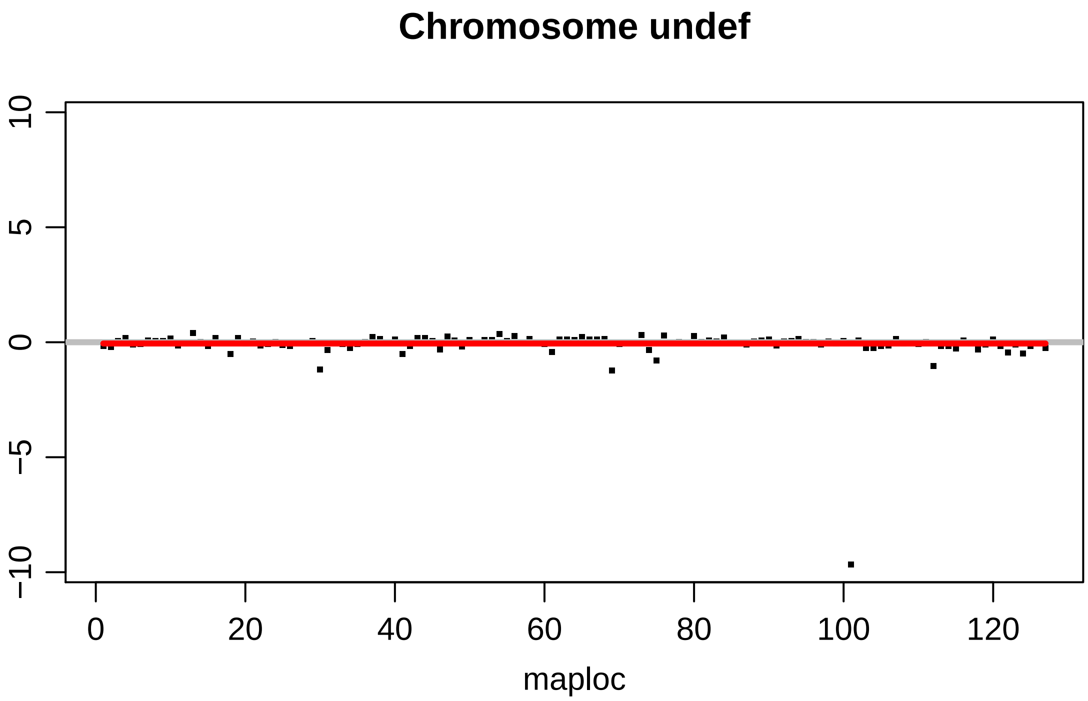

```
## Segplot might not work because of special characters in the sample names. Use only A-Z,a-z and 0-9!
##  There is a hidden function cn.mops:::.replaceNames that replaces the names in the "CNVDetectionResul
```

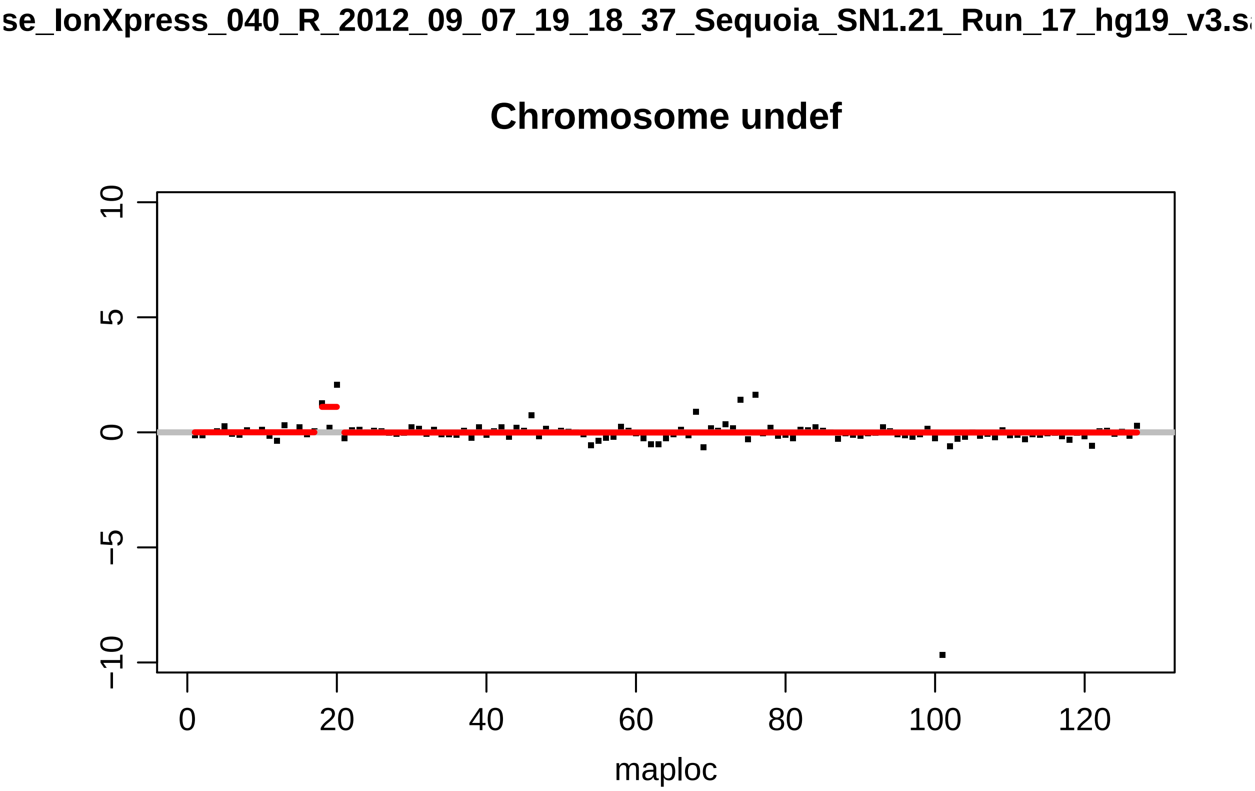


```
## Segplot might not work because of special characters in the sample names. Use only A-Z,a-z and 0-9!
##  There is a hidden function cn.mops:::.replaceNames that replaces the names in the "CNVDetectionResul
```

se_IonXpress_041_R_2012_09_07_19_18_37_Sequoia_SN1.21_Run_17_hg19_v3.s

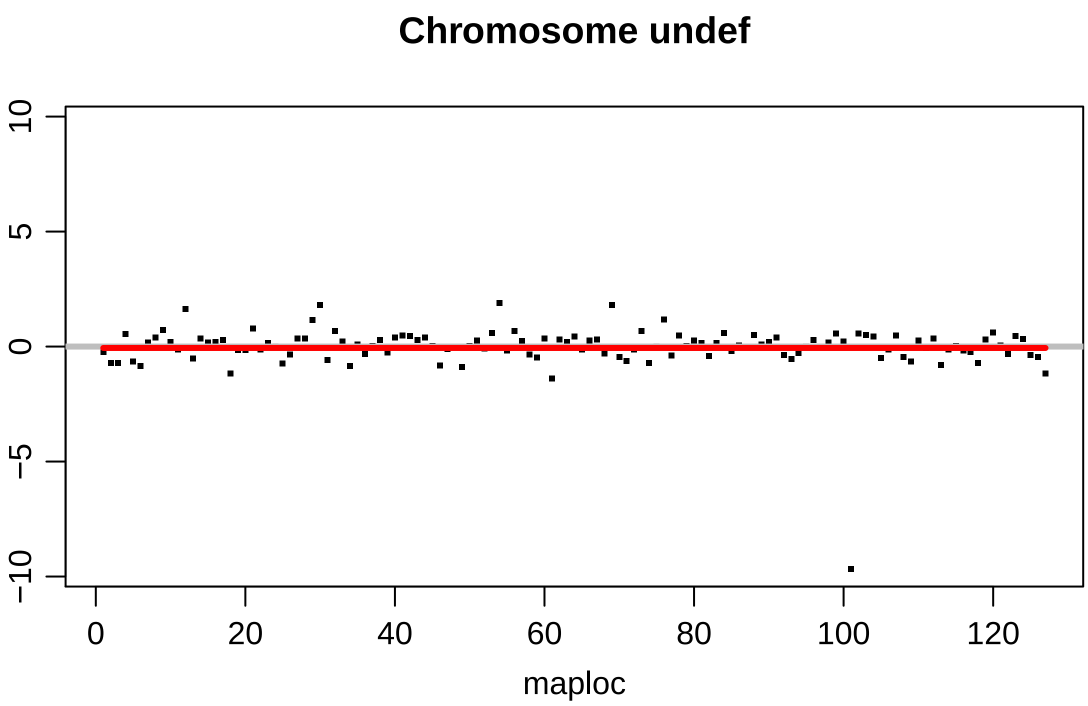


```
## Segplot might not work because of special characters in the sample names. Use only A-Z,a-z and 0-9!
##  There is a hidden function cn.mops:::.replaceNames that replaces the names in the "CNVDetectionResul
```

se_IonXpress_042_R_2012_09_07_19_18_37_Sequoia_SN1.21_Run_17_hg19_v3.s

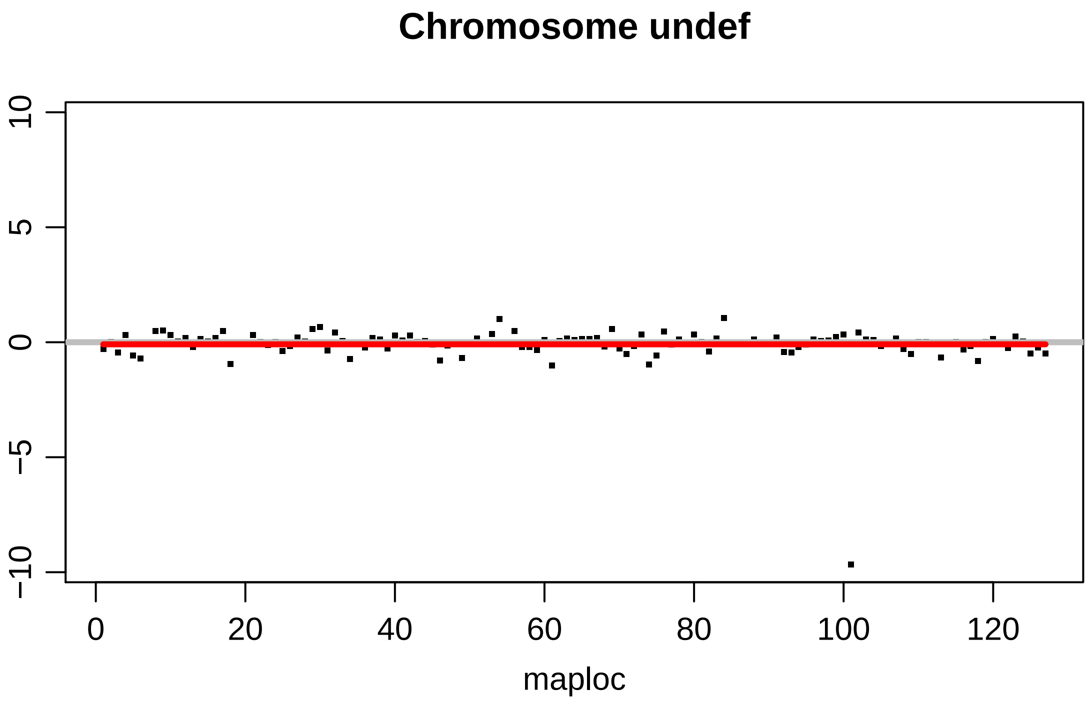

```
## Segplot might not work because of special characters in the sample names. Use only A-Z,a-z and 0-9!
##  There is a hidden function cn.mops:::.replaceNames that replaces the names in the "CNVDetectionResul
```

se_IonXpress_043_R_2012_09_07_19_18_37_Sequoia_SN1.21_Run_17_hg19_v3.s

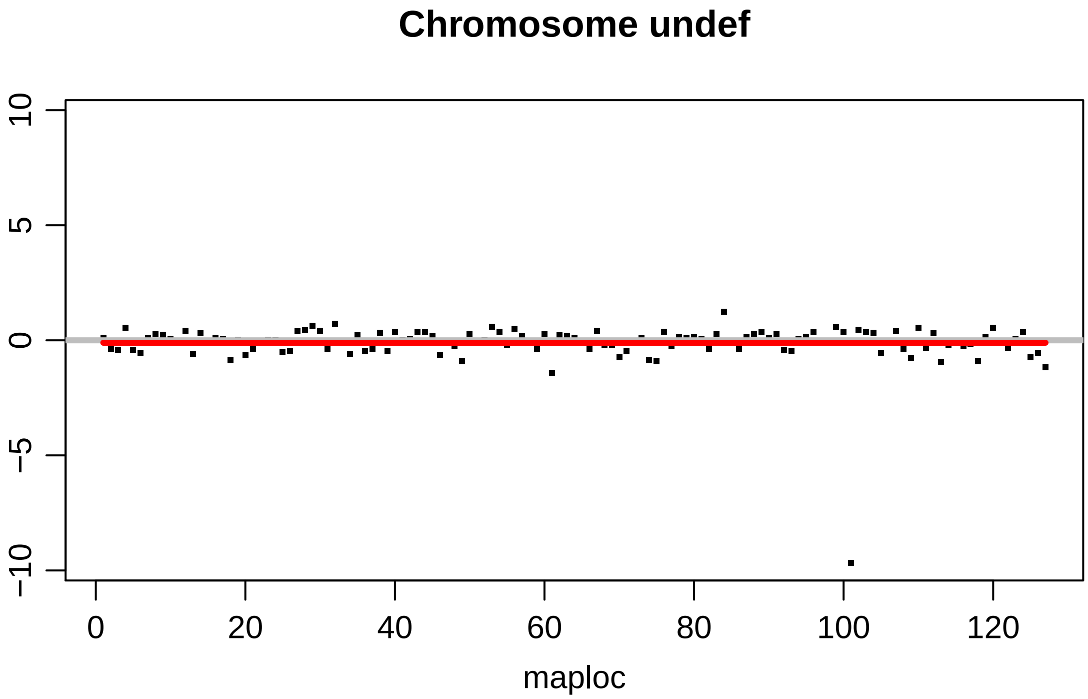


```
## Segplot might not work because of special characters in the sample names. Use only A-Z,a-z and 0-9!
##  There is a hidden function cn.mops:::.replaceNames that replaces the names in the "CNVDetectionResul
```

se_IonXpress_044_R_2012_09_07_19_18_37_Sequoia_SN1.21_Run_17_hg19_v3.sa

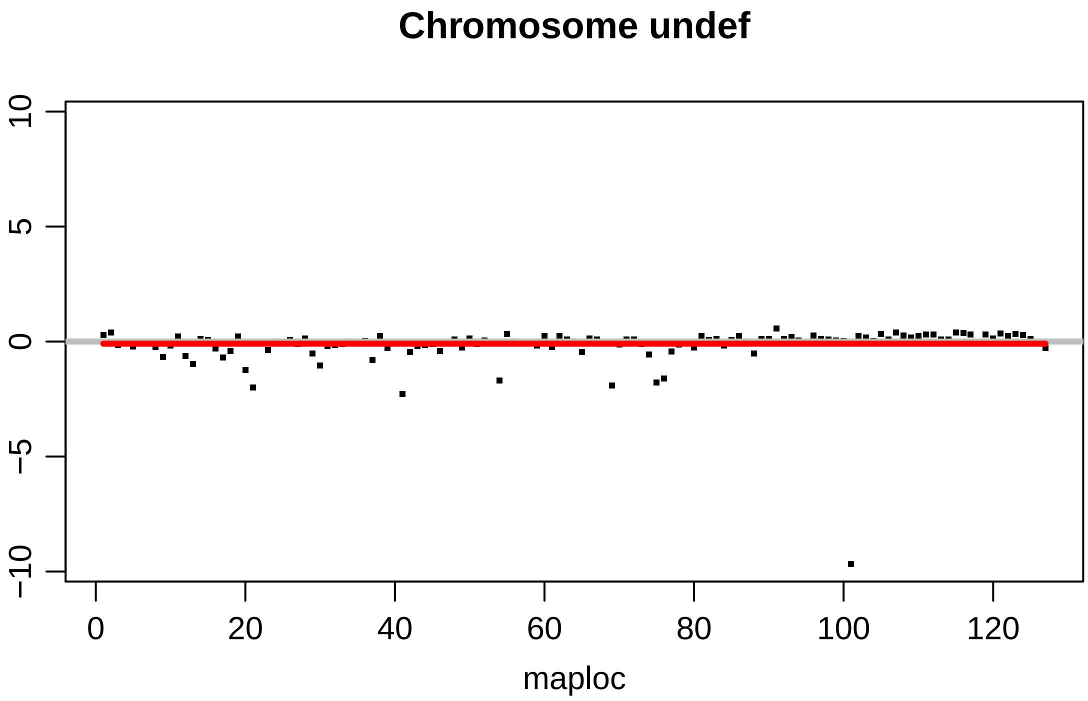


```
## Segplot might not work because of special characters in the sample names. Use only A-Z,a-z and 0-9!
##  There is a hidden function cn.mops:::.replaceNames that replaces the names in the "CNVDetectionResul
```

se_IonXpress_045_R_2012_09_07_19_18_37_Sequoia_SN1.21_Run_17_hg19_v3.sa

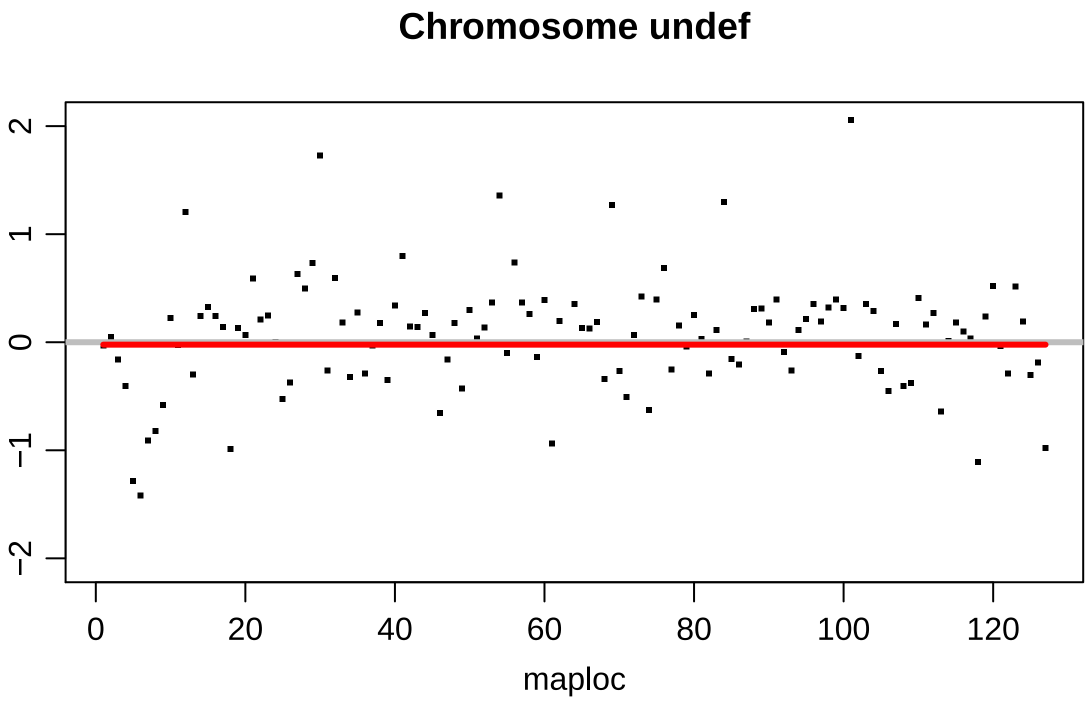

```
## Segplot might not work because of special characters in the sample names. Use only A-Z,a-z and 0-9!
##  There is a hidden function cn.mops:::.replaceNames that replaces the names in the "CNVDetectionResul
```

se_IonXpress_046_R_2012_09_07_19_18_37_Sequoia_SN1.21_Run_17_hg19_v3.s

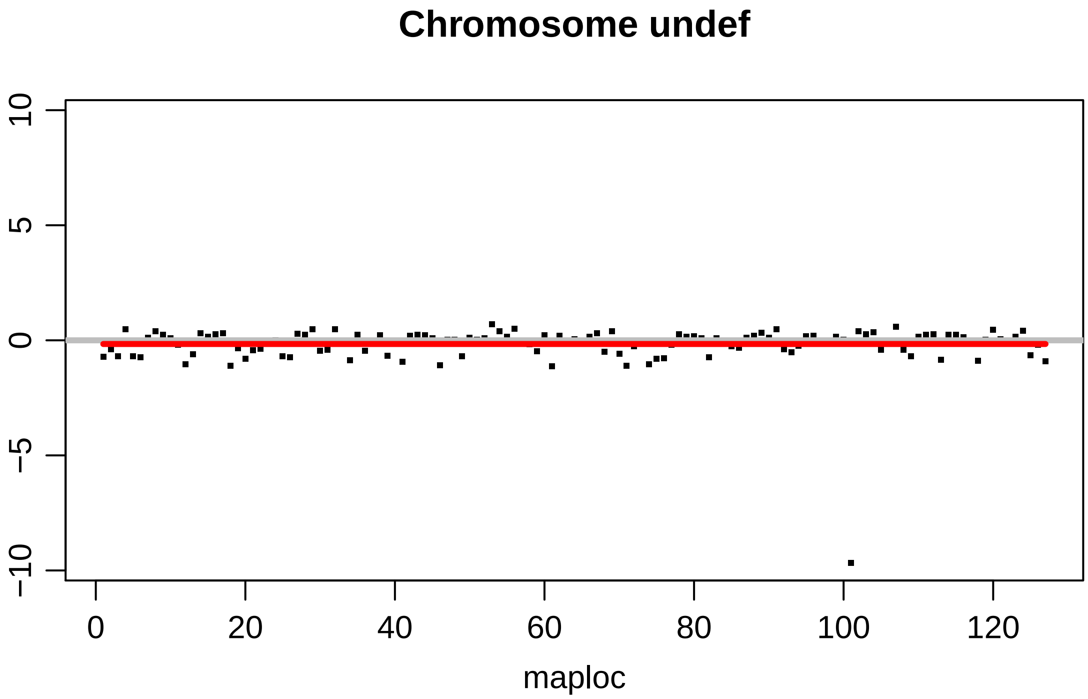


```
## Segplot might not work because of special characters in the sample names. Use only A-Z,a-z and 0-9!
##  There is a hidden function cn.mops:::.replaceNames that replaces the names in the "CNVDetectionResul
```

se_IonXpress_047_R_2012_09_07_19_18_37_Sequoia_SN1.21_Run_17_hg19_v3.s

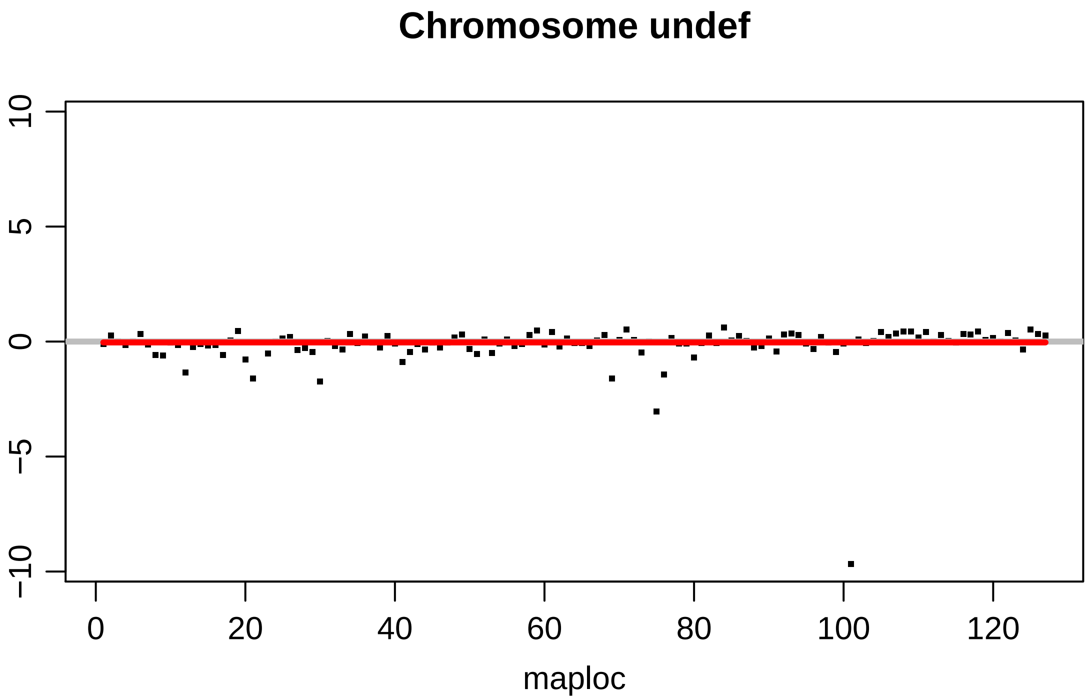


```
##
## CNV regions:
## GRanges object with 2 ranges and 43 metadata columns:
##       seqnames    ranges strand |
##          <Rle> <IRanges>  <Rle> |
##   [1]    undef  [ 6,  9]      * |
##   [2]    undef  [18, 20]      * |
##       Case_IonXpress_001_R_2012_09_07_19_18_37_Sequoia_SN1.21_Run_17_hg19_v3.sam
##                                                                         <factor>
##   [1]                                                                        CN2
##   [2]                                                                        CN2
##       Case_IonXpress_002_R_2012_09_07_19_18_37_Sequoia_SN1.21_Run_17_hg19_v3.sam
##                                                                         <factor>
##   [1]                                                                        CN2
##   [2]                                                                        CN2
##       Case_IonXpress_003_R_2012_09_07_19_18_37_Sequoia_SN1.21_Run_17_hg19_v3.sam
##                                                                         <factor>
##   [1]                                                                        CN2
##   [2]                                                                        CN2
##       Case_IonXpress_004_R_2012_09_07_19_18_37_Sequoia_SN1.21_Run_17_hg19_v3.sam
##                                                                         <factor>
##   [1]                                                                        CN2
##   [2]                                                                        CN2
##       Case_IonXpress_006_R_2012_09_07_19_18_37_Sequoia_SN1.21_Run_17_hg19_v3.sam
##                                                                         <factor>
##   [1]                                                                        CN2
##   [2]                                                                        CN2
##       Case_IonXpress_007_R_2012_09_07_19_18_37_Sequoia_SN1.21_Run_17_hg19_v3.sam
```

```
##                                                                     <factor>
##   [1]                                                                    CN2
##   [2]                                                                    CN2
##       Case_IonXpress_008_R_2012_09_07_19_18_37_Sequoia_SN1.21_Run_17_hg19_v3.sam
##                                                                     <factor>
##   [1]                                                                    CN2
##   [2]                                                                    CN3
##       Case_IonXpress_009_R_2012_09_07_19_18_37_Sequoia_SN1.21_Run_17_hg19_v3.sam
##                                                                     <factor>
##   [1]                                                                    CN2
##   [2]                                                                    CN2
##       Case_IonXpress_011_R_2012_09_07_19_18_37_Sequoia_SN1.21_Run_17_hg19_v3.sam
##                                                                     <factor>
##   [1]                                                                    CN2
##   [2]                                                                    CN2
##       Case_IonXpress_013_R_2012_09_07_19_18_37_Sequoia_SN1.21_Run_17_hg19_v3.sam
##                                                                     <factor>
##   [1]                                                                    CN1
##   [2]                                                                    CN2
##       Case_IonXpress_014_R_2012_09_07_19_18_37_Sequoia_SN1.21_Run_17_hg19_v3.sam
##                                                                     <factor>
##   [1]                                                                    CN2
##   [2]                                                                    CN2
##       Case_IonXpress_015_R_2012_09_07_19_18_37_Sequoia_SN1.21_Run_17_hg19_v3.sam
##                                                                     <factor>
##   [1]                                                                    CN2
##   [2]                                                                    CN2
##       Case_IonXpress_016_R_2012_09_07_19_18_37_Sequoia_SN1.21_Run_17_hg19_v3.sam
##                                                                     <factor>
##   [1]                                                                    CN2
##   [2]                                                                    CN2
##       Case_IonXpress_017_R_2012_09_07_19_18_37_Sequoia_SN1.21_Run_17_hg19_v3.sam
##                                                                     <factor>
##   [1]                                                                    CN2
##   [2]                                                                    CN2
##       Case_IonXpress_018_R_2012_09_07_19_18_37_Sequoia_SN1.21_Run_17_hg19_v3.sam
##                                                                     <factor>
##   [1]                                                                    CN2
##   [2]                                                                    CN2
##       Case_IonXpress_019_R_2012_09_07_19_18_37_Sequoia_SN1.21_Run_17_hg19_v3.sam
##                                                                     <factor>
##   [1]                                                                    CN2
##   [2]                                                                    CN2
##       Case_IonXpress_020_R_2012_09_07_19_18_37_Sequoia_SN1.21_Run_17_hg19_v3.sam
##                                                                     <factor>
##   [1]                                                                    CN2
##   [2]                                                                    CN2
##       Case_IonXpress_021_R_2012_09_07_19_18_37_Sequoia_SN1.21_Run_17_hg19_v3.sam
##                                                                     <factor>
##   [1]                                                                    CN2
##   [2]                                                                    CN2
##       Case_IonXpress_023_R_2012_09_07_19_18_37_Sequoia_SN1.21_Run_17_hg19_v3.sam
##                                                                     <factor>
##   [1]                                                                    CN2
```

```
##   [2]                                                                   CN2
##       Case_IonXpress_024_R_2012_09_07_19_18_37_Sequoia_SN1.21_Run_17_hg19_v3.sam
##                                                                    <factor>
##   [1]                                                                   CN2
##   [2]                                                                   CN2
##       Case_IonXpress_025_R_2012_09_07_19_18_37_Sequoia_SN1.21_Run_17_hg19_v3.sam
##                                                                    <factor>
##   [1]                                                                   CN2
##   [2]                                                                   CN2
##       Case_IonXpress_026_R_2012_09_07_19_18_37_Sequoia_SN1.21_Run_17_hg19_v3.sam
##                                                                    <factor>
##   [1]                                                                   CN2
##   [2]                                                                   CN2
##       Case_IonXpress_027_R_2012_09_07_19_18_37_Sequoia_SN1.21_Run_17_hg19_v3.sam
##                                                                    <factor>
##   [1]                                                                   CN2
##   [2]                                                                   CN2
##       Case_IonXpress_028_R_2012_09_07_19_18_37_Sequoia_SN1.21_Run_17_hg19_v3.sam
##                                                                    <factor>
##   [1]                                                                   CN2
##   [2]                                                                   CN2
##       Case_IonXpress_029_R_2012_09_07_19_18_37_Sequoia_SN1.21_Run_17_hg19_v3.sam
##                                                                    <factor>
##   [1]                                                                   CN2
##   [2]                                                                   CN2
##       Case_IonXpress_030_R_2012_09_07_19_18_37_Sequoia_SN1.21_Run_17_hg19_v3.sam
##                                                                    <factor>
##   [1]                                                                   CN2
##   [2]                                                                   CN2
##       Case_IonXpress_031_R_2012_09_07_19_18_37_Sequoia_SN1.21_Run_17_hg19_v3.sam
##                                                                    <factor>
##   [1]                                                                   CN2
##   [2]                                                                   CN2
##       Case_IonXpress_032_R_2012_09_07_19_18_37_Sequoia_SN1.21_Run_17_hg19_v3.sam
##                                                                    <factor>
##   [1]                                                                   CN2
##   [2]                                                                   CN2
##       Case_IonXpress_033_R_2012_09_07_19_18_37_Sequoia_SN1.21_Run_17_hg19_v3.sam
##                                                                    <factor>
##   [1]                                                                   CN2
##   [2]                                                                   CN2
##       Case_IonXpress_034_R_2012_09_07_19_18_37_Sequoia_SN1.21_Run_17_hg19_v3.sam
##                                                                    <factor>
##   [1]                                                                   CN2
##   [2]                                                                   CN2
##       Case_IonXpress_036_R_2012_09_07_19_18_37_Sequoia_SN1.21_Run_17_hg19_v3.sam
##                                                                    <factor>
##   [1]                                                                   CN2
##   [2]                                                                   CN2
##       Case_IonXpress_037_R_2012_09_07_19_18_37_Sequoia_SN1.21_Run_17_hg19_v3.sam
##                                                                    <factor>
##   [1]                                                                   CN2
##   [2]                                                                   CN2
##       Case_IonXpress_038_R_2012_09_07_19_18_37_Sequoia_SN1.21_Run_17_hg19_v3.sam
```

```
##                                                                        <factor>
##   [1]                                                                      CN2
##   [2]                                                                      CN2
##      Case_IonXpress_039_R_2012_09_07_19_18_37_Sequoia_SN1.21_Run_17_hg19_v3.sam
##                                                                        <factor>
##   [1]                                                                      CN2
##   [2]                                                                      CN2
##      Case_IonXpress_040_R_2012_09_07_19_18_37_Sequoia_SN1.21_Run_17_hg19_v3.sam
##                                                                        <factor>
##   [1]                                                                      CN2
##   [2]                                                                      CN3
##      Case_IonXpress_041_R_2012_09_07_19_18_37_Sequoia_SN1.21_Run_17_hg19_v3.sam
##                                                                        <factor>
##   [1]                                                                      CN2
##   [2]                                                                      CN2
##      Case_IonXpress_042_R_2012_09_07_19_18_37_Sequoia_SN1.21_Run_17_hg19_v3.sam
##                                                                        <factor>
##   [1]                                                                      CN2
##   [2]                                                                      CN2
##      Case_IonXpress_043_R_2012_09_07_19_18_37_Sequoia_SN1.21_Run_17_hg19_v3.sam
##                                                                        <factor>
##   [1]                                                                      CN2
##   [2]                                                                      CN2
##      Case_IonXpress_044_R_2012_09_07_19_18_37_Sequoia_SN1.21_Run_17_hg19_v3.sam
##                                                                        <factor>
##   [1]                                                                      CN2
##   [2]                                                                      CN2
##      Case_IonXpress_045_R_2012_09_07_19_18_37_Sequoia_SN1.21_Run_17_hg19_v3.sam
##                                                                        <factor>
##   [1]                                                                      CN2
##   [2]                                                                      CN2
##      Case_IonXpress_046_R_2012_09_07_19_18_37_Sequoia_SN1.21_Run_17_hg19_v3.sam
##                                                                        <factor>
##   [1]                                                                      CN2
##   [2]                                                                      CN2
##      Case_IonXpress_047_R_2012_09_07_19_18_37_Sequoia_SN1.21_Run_17_hg19_v3.sam
##                                                                        <factor>
##   [1]                                                                      CN2
##   [2]                                                                      CN2
##      Case_IonXpress_048_R_2012_09_07_19_18_37_Sequoia_SN1.21_Run_17_hg19_v3.sam
##                                                                        <factor>
##   [1]                                                                      CN2
##   [2]                                                                      CN2
##   -------
##   seqinfo: 1 sequence from an unspecified genome; no seqlengths
##
## Individual CNVs:
## GRanges object with 3 ranges and 4 metadata columns:
##      seqnames    ranges strand |
##         <Rle> <IRanges>  <Rle> |
##   [1]    undef  [18, 20]      * |
##   [2]    undef  [ 6,  9]      * |
##   [3]    undef  [18, 20]      * |
##                                                                      sampleName
```

```
##                                                                            <factor>
##   [1] Case_IonXpress_008_R_2012_09_07_19_18_37_Sequoia_SN1.21_Run_17_hg19_v3.sam
##   [2] Case_IonXpress_013_R_2012_09_07_19_18_37_Sequoia_SN1.21_Run_17_hg19_v3.sam
##   [3] Case_IonXpress_040_R_2012_09_07_19_18_37_Sequoia_SN1.21_Run_17_hg19_v3.sam
##           median       mean           CN
##        <numeric>  <numeric> <character>
##   [1]  1.3219281  1.1873997         CN3
##   [2] -0.9952071 -0.9953233         CN1
##   [3]  1.3219281  1.1077005         CN3
##   -------
##   seqinfo: 1 sequence from an unspecified genome; no seqlengths
## [1] "/Users/gdemidov/Downloads/doc/Run_18_fin_05_qc.xls"

## Normalizing...

## Starting local modeling, please be patient...

## Reference sequence:  undef

## Starting segmentation algorithm...

## Using "fastseg" for segmentation.

## [1] ""
## [1] "/Users/gdemidov/Downloads/doc/Run_18_fin_05_qc.xls"
## [1] ""

## Segplot might not work because of special characters in the sample names. Use only A-Z,a-z and 0-9!
##  There is a hidden function cn.mops:::.replaceNames that replaces the names in the "CNVDetectionResul
```

se_IonXpress_048_R_2012_09_07_19_18_37_Sequoia_SN1.21_Run_17_hg19_v3.s

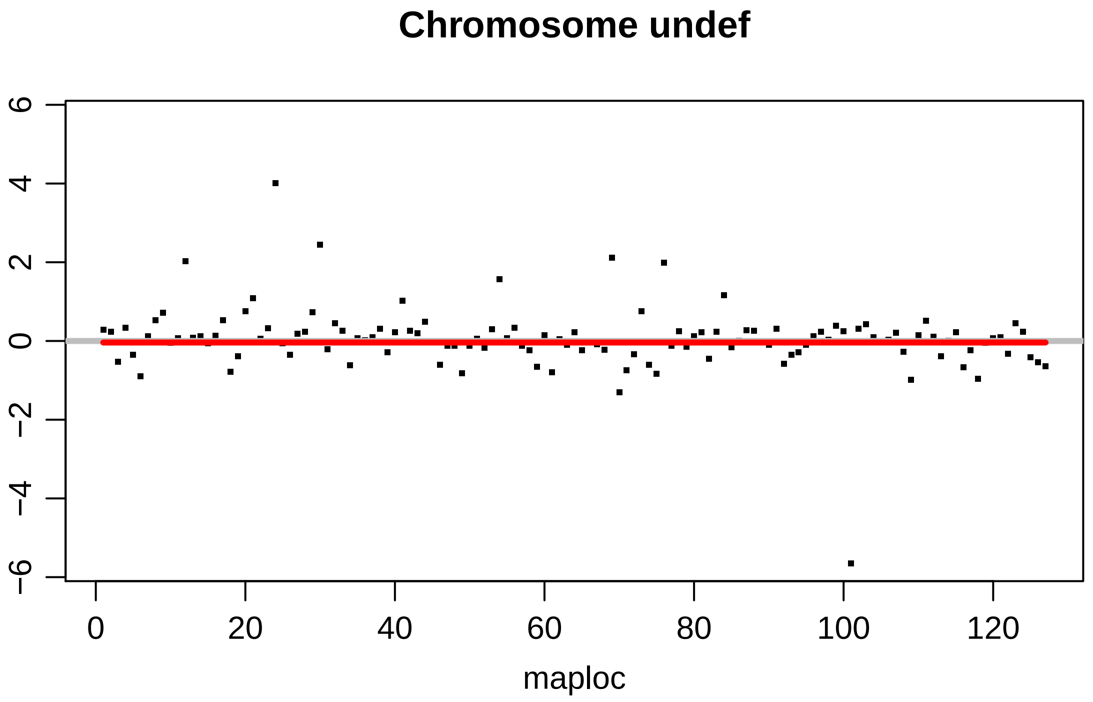

```
## Segplot might not work because of special characters in the sample names. Use only A-Z,a-z and 0-9!
##  There is a hidden function cn.mops:::.replaceNames that replaces the names in the "CNVDetectionResul
```

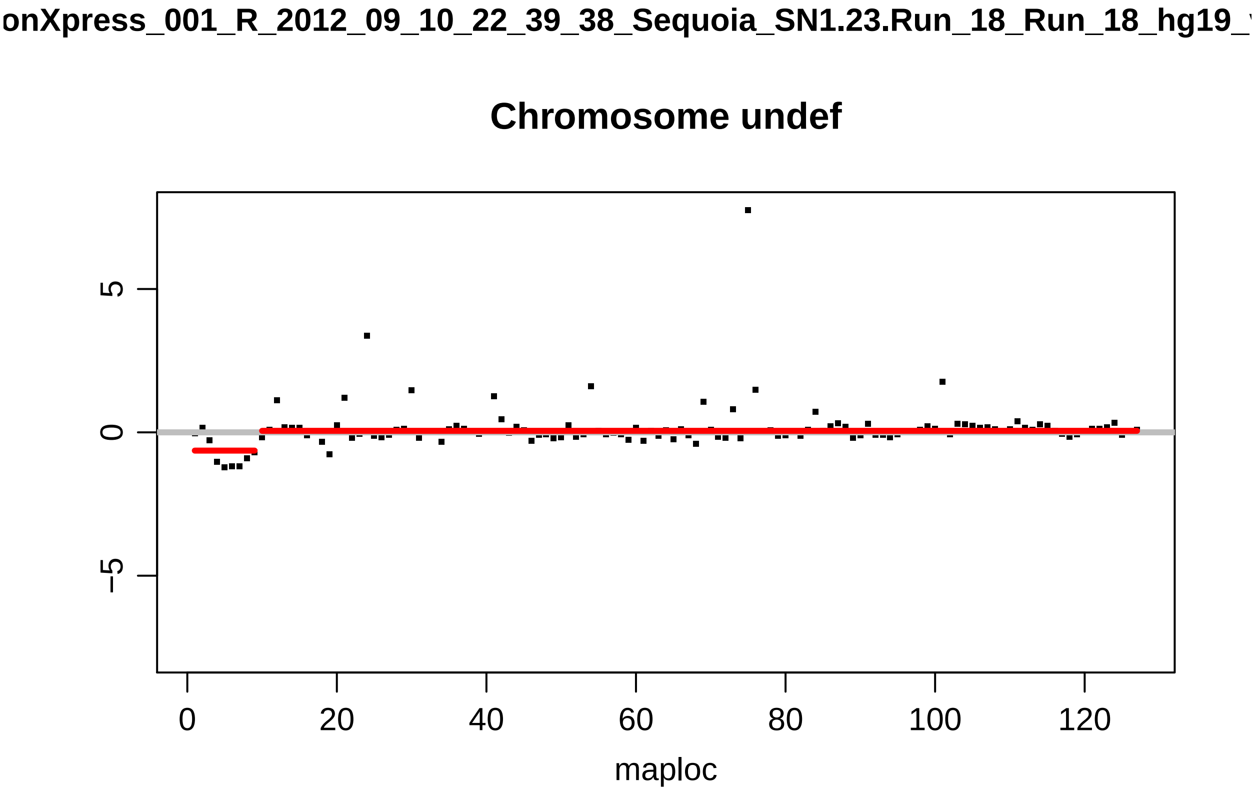


```
## Segplot might not work because of special characters in the sample names. Use only A-Z,a-z and 0-9!
##  There is a hidden function cn.mops:::.replaceNames that replaces the names in the "CNVDetectionResul
```

onXpress_002_R_2012_09_10_22_39_38_Sequoia_SN1.23.Run_18_Run_18_hg19_

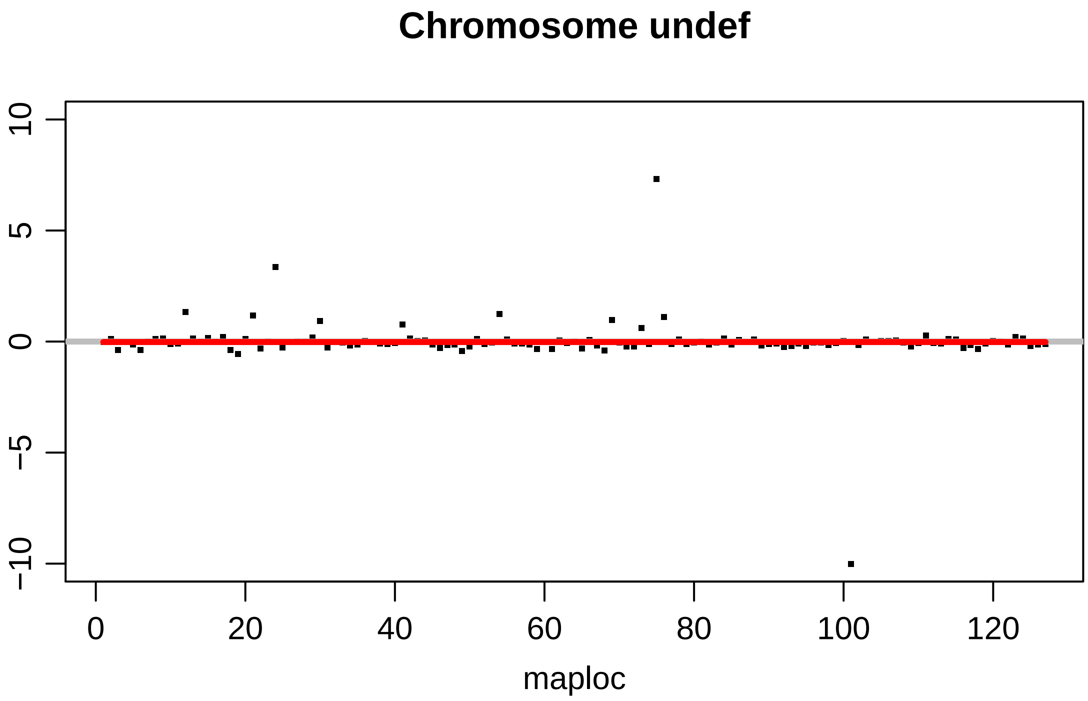


```
## Segplot might not work because of special characters in the sample names. Use only A-Z,a-z and 0-9!
##  There is a hidden function cn.mops:::.replaceNames that replaces the names in the "CNVDetectionResul
```

onXpress_003_R_2012_09_10_22_39_38_Sequoia_SN1.23.Run_18_Run_18_hg19_

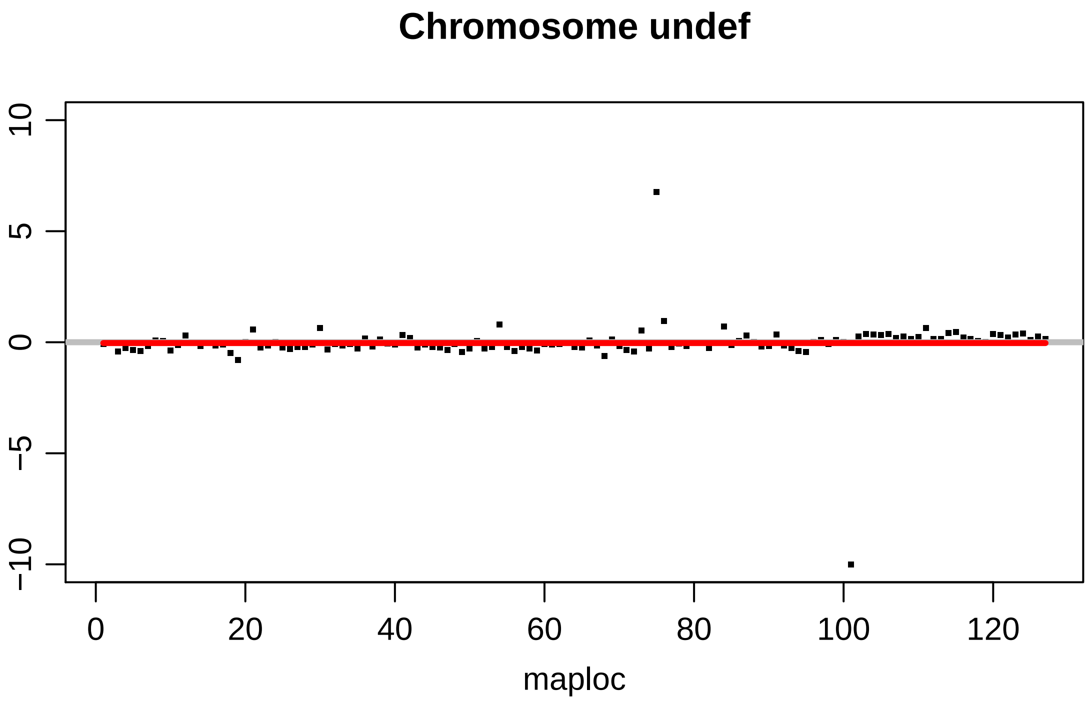

```
## Segplot might not work because of special characters in the sample names. Use only A-Z,a-z and 0-9!
##  There is a hidden function cn.mops:::.replaceNames that replaces the names in the "CNVDetectionResul
```

onXpress_004_R_2012_09_10_22_39_38_Sequoia_SN1.23.Run_18_Run_18_hg19_

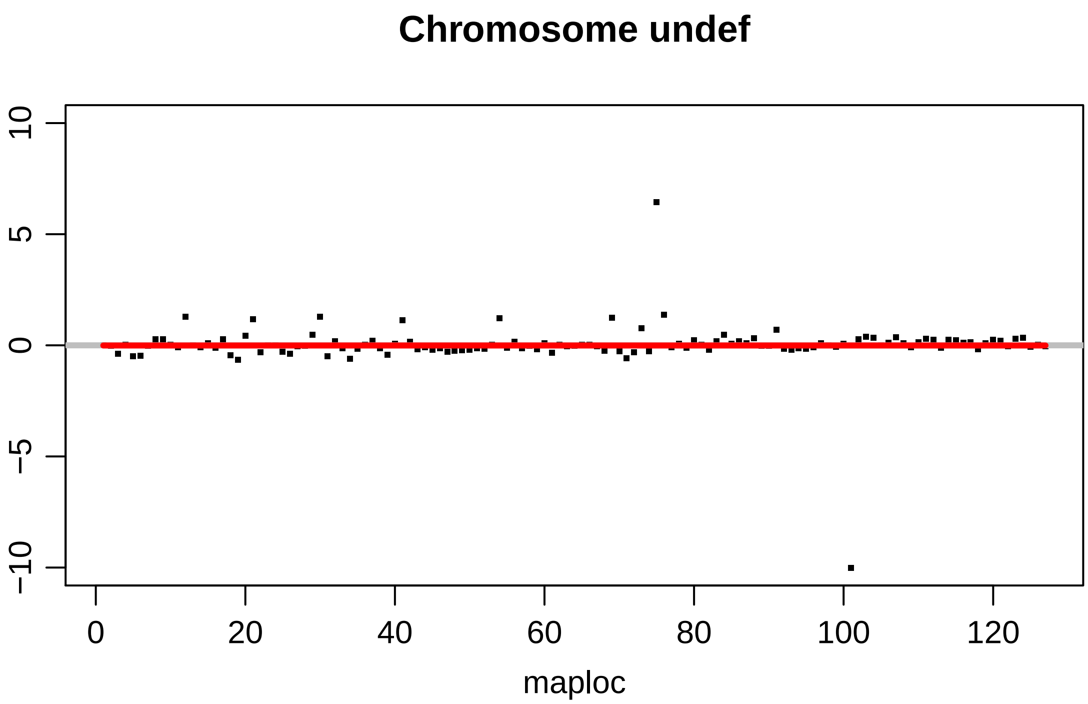


```
## Segplot might not work because of special characters in the sample names. Use only A-Z,a-z and 0-9!
##  There is a hidden function cn.mops:::.replaceNames that replaces the names in the "CNVDetectionResul
```

onXpress_005_R_2012_09_10_22_39_38_Sequoia_SN1.23.Run_18_Run_18_hg19_

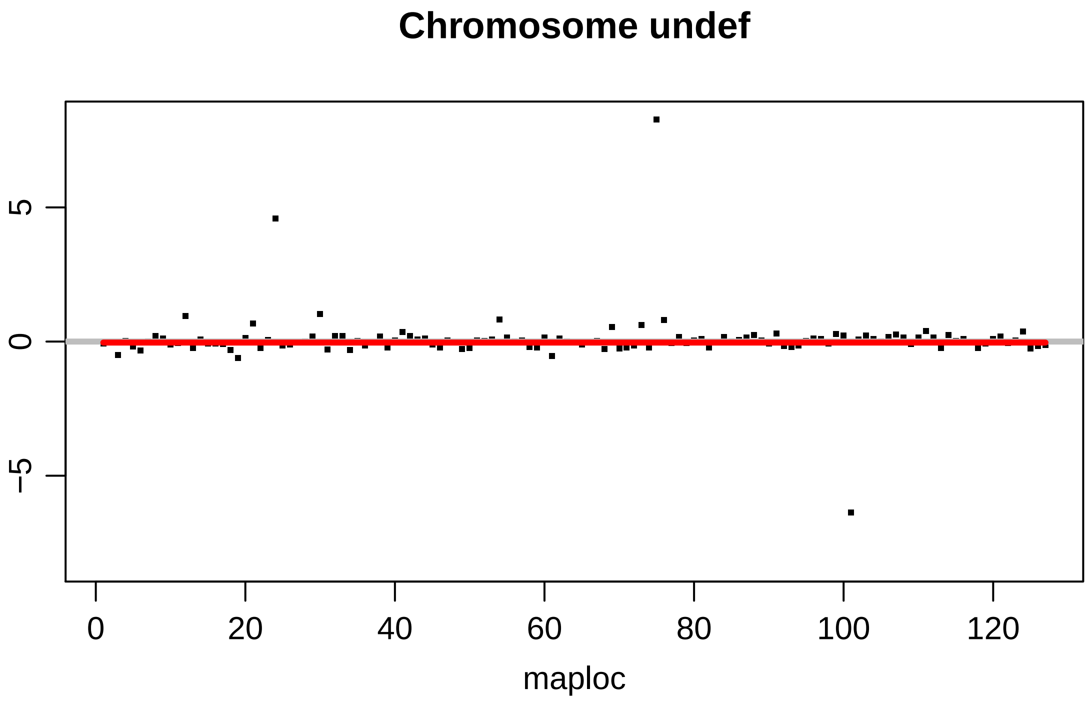


```
## Segplot might not work because of special characters in the sample names. Use only A-Z,a-z and 0-9!
##  There is a hidden function cn.mops:::.replaceNames that replaces the names in the "CNVDetectionResul
```

onXpress_006_R_2012_09_10_22_39_38_Sequoia_SN1.23.Run_18_Run_18_hg19_

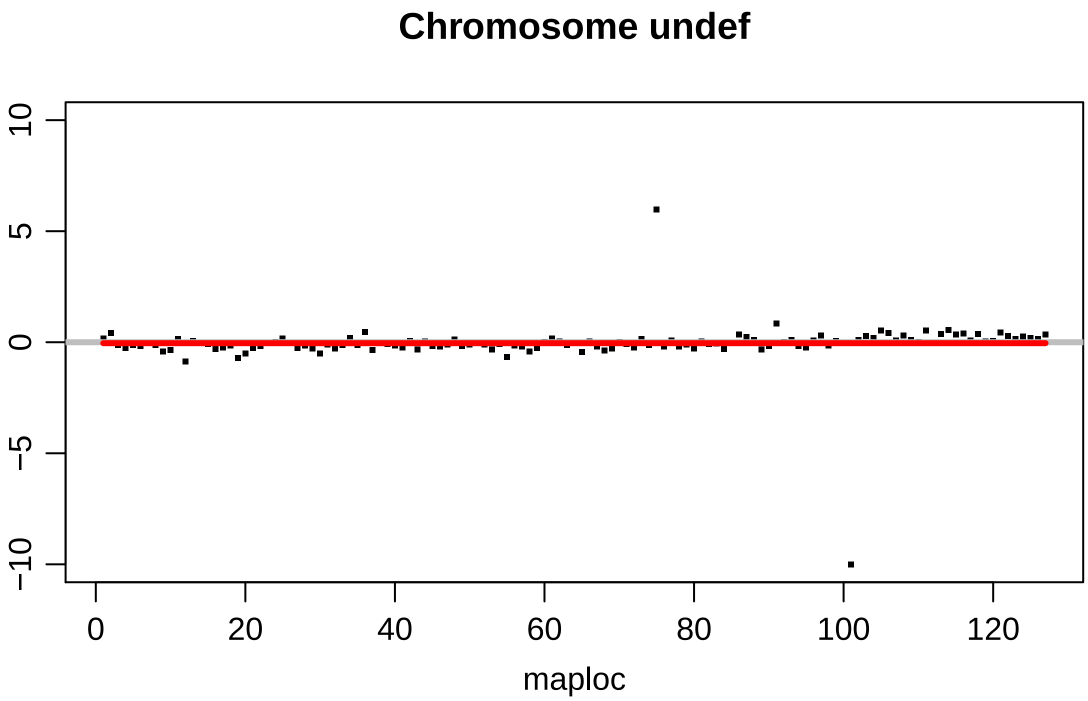

```
## Segplot might not work because of special characters in the sample names. Use only A-Z,a-z and 0-9!
##  There is a hidden function cn.mops:::.replaceNames that replaces the names in the "CNVDetectionResul
```

onXpress_007_R_2012_09_10_22_39_38_Sequoia_SN1.23.Run_18_Run_18_hg19_

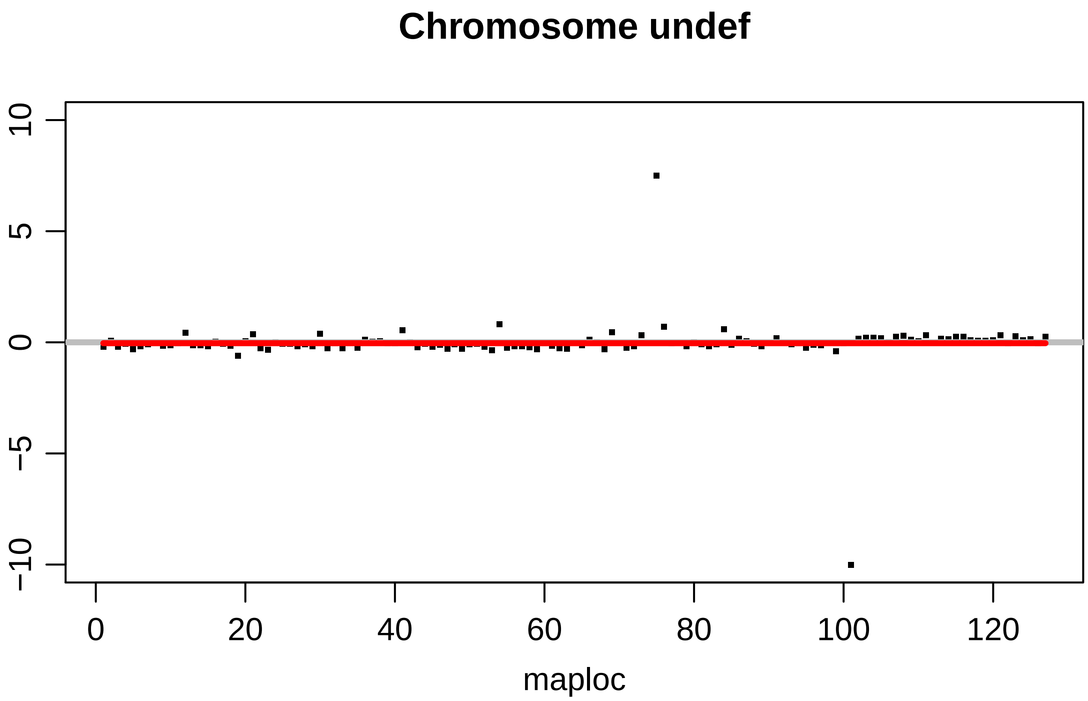


```
## Segplot might not work because of special characters in the sample names. Use only A-Z,a-z and 0-9!
##  There is a hidden function cn.mops:::.replaceNames that replaces the names in the "CNVDetectionResul
```

onXpress_008_R_2012_09_10_22_39_38_Sequoia_SN1.23.Run_18_Run_18_hg19_

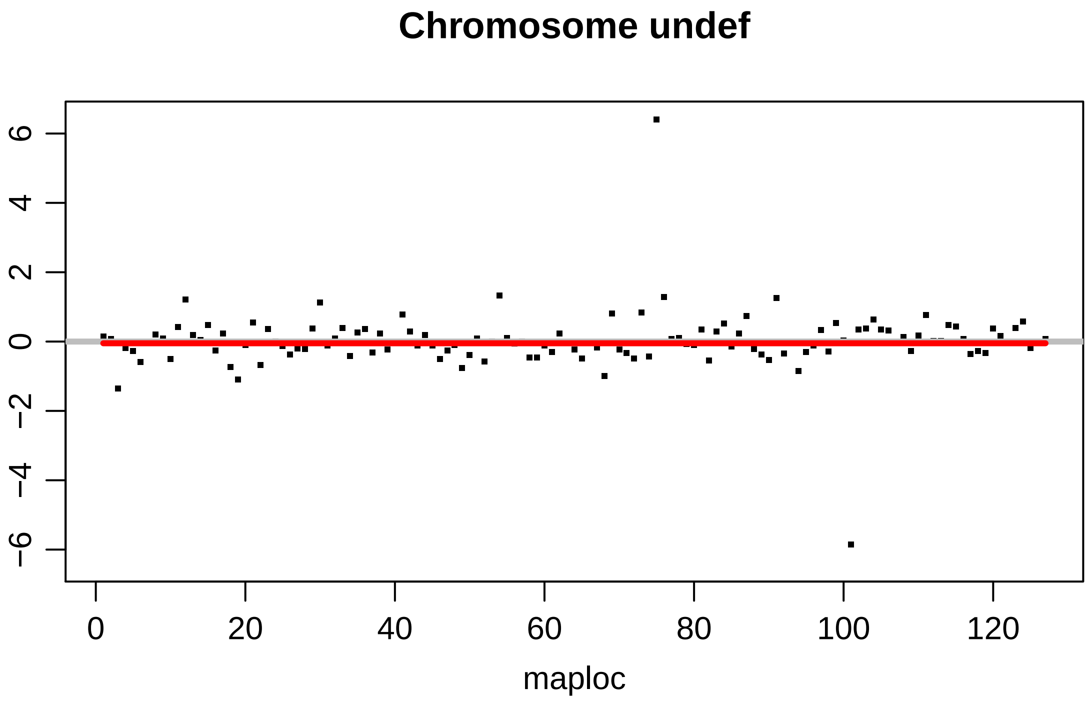

Segplot might not work because of special characters in the sample names. Use only A-Z,a-z and 0-9!
There is a hidden function cn.mops:::.replaceNames that replaces the names in the "CNVDetectionResul

onXpress_009_R_2012_09_10_22_39_38_Sequoia_SN1.23.Run_18_Run_18_hg19_

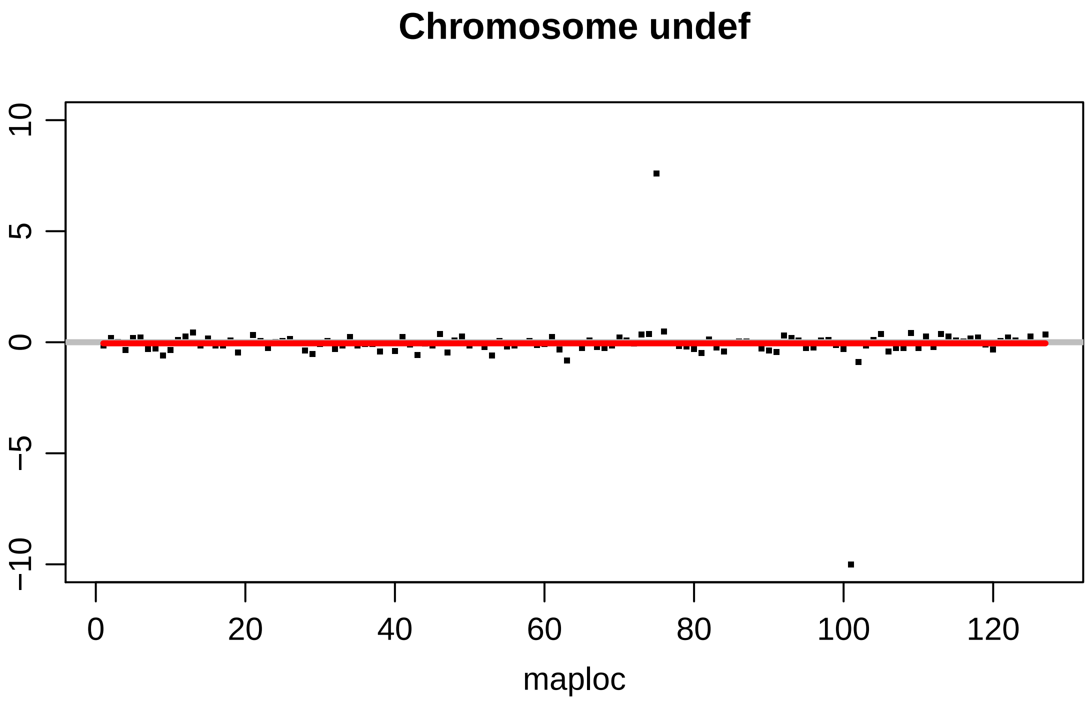

```
## Segplot might not work because of special characters in the sample names. Use only A-Z,a-z and 0-9!
##  There is a hidden function cn.mops:::.replaceNames that replaces the names in the "CNVDetectionResul
```

onXpress_010_R_2012_09_10_22_39_38_Sequoia_SN1.23.Run_18_Run_18_hg19_

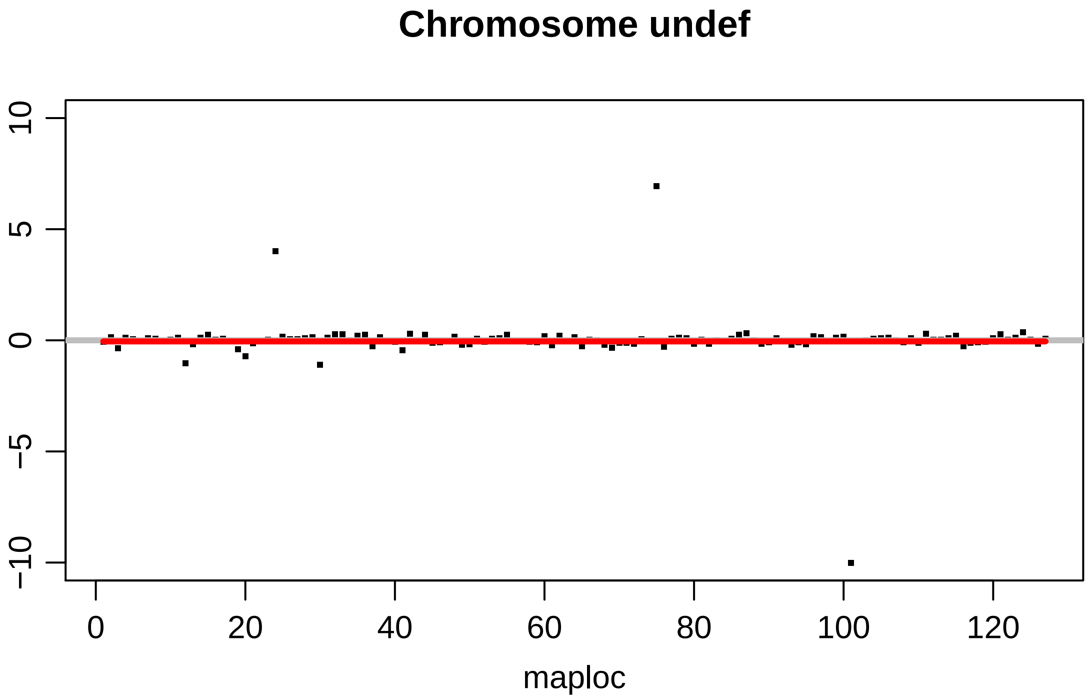


```
## Segplot might not work because of special characters in the sample names. Use only A-Z,a-z and 0-9!
##  There is a hidden function cn.mops:::.replaceNames that replaces the names in the "CNVDetectionResul
```

onXpress_011_R_2012_09_10_22_39_38_Sequoia_SN1.23.Run_18_Run_18_hg19_

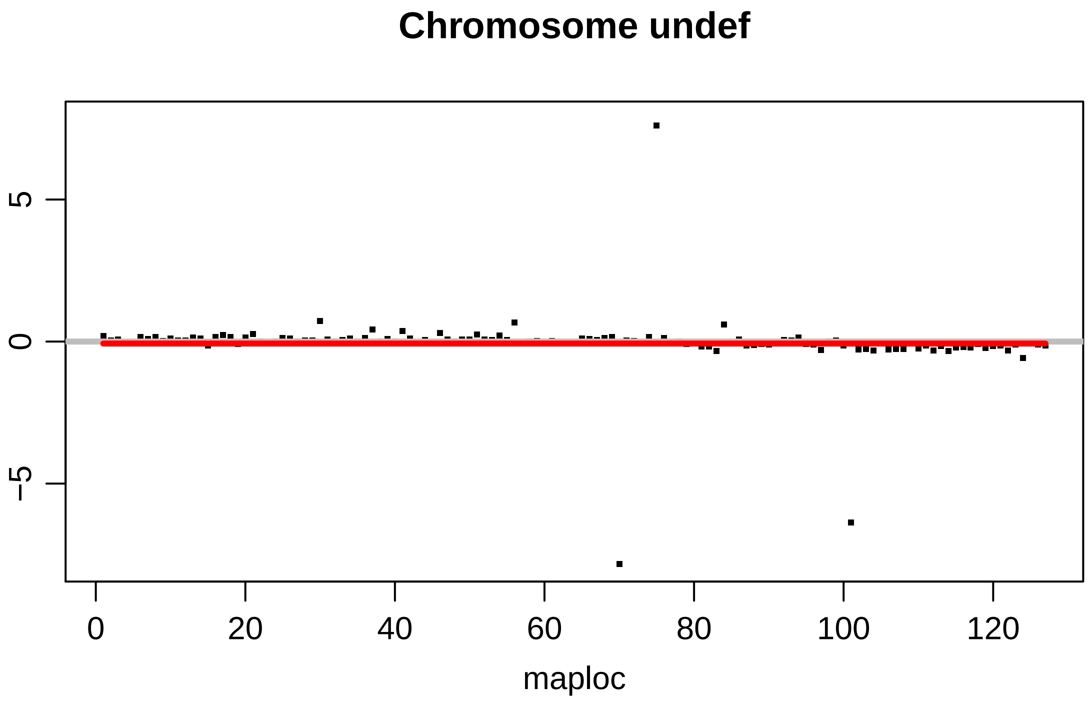


```
## Segplot might not work because of special characters in the sample names. Use only A-Z,a-z and 0-9!
##  There is a hidden function cn.mops:::.replaceNames that replaces the names in the "CNVDetectionResul
```

onXpress_012_R_2012_09_10_22_39_38_Sequoia_SN1.23.Run_18_Run_18_hg19_

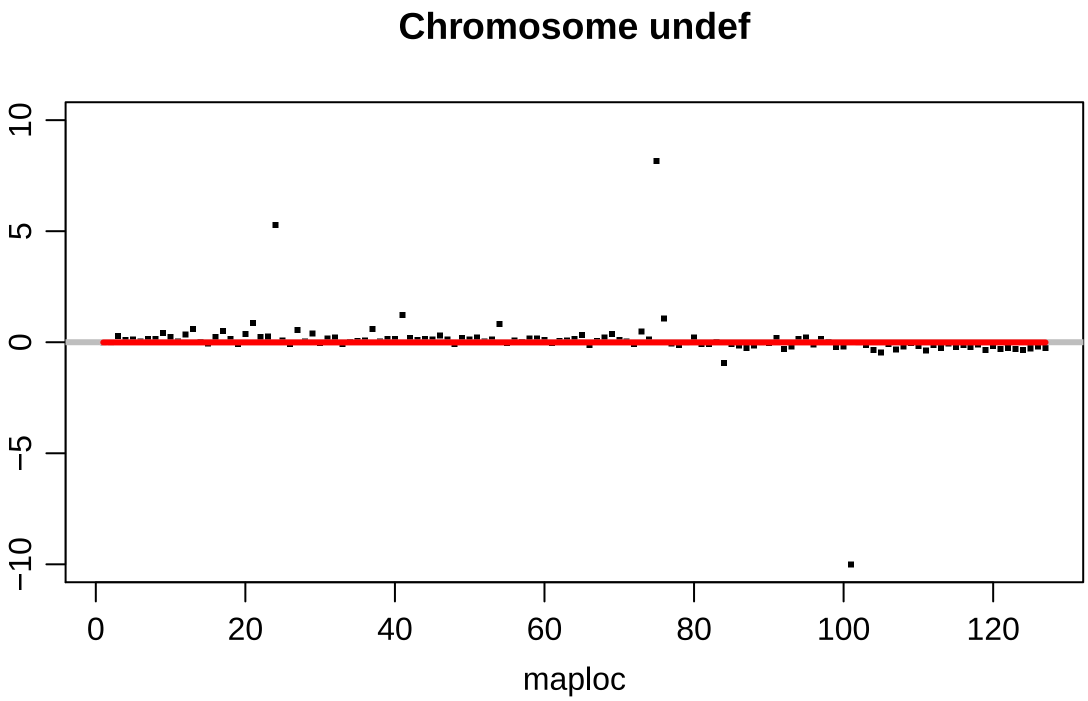

```
## Segplot might not work because of special characters in the sample names. Use only A-Z,a-z and 0-9!
##  There is a hidden function cn.mops:::.replaceNames that replaces the names in the "CNVDetectionResul
```

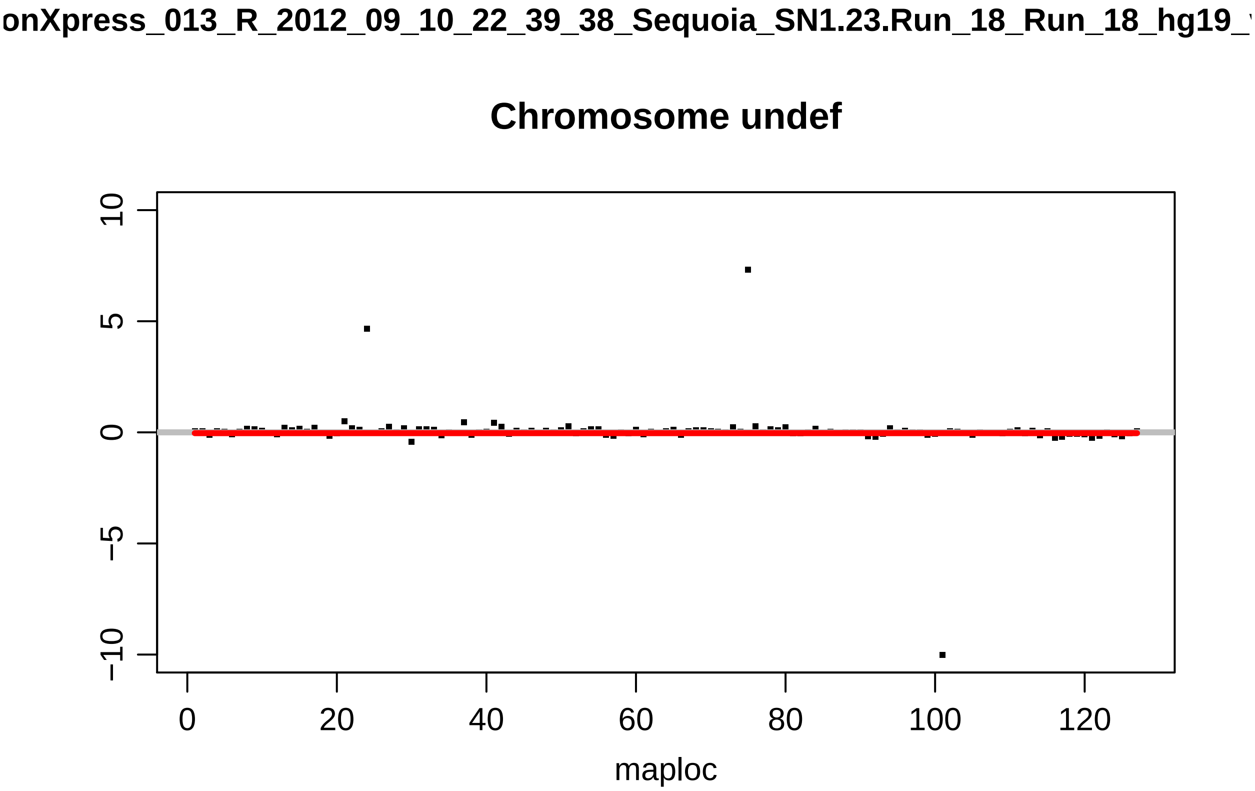


```
## Segplot might not work because of special characters in the sample names. Use only A-Z,a-z and 0-9!
##  There is a hidden function cn.mops:::.replaceNames that replaces the names in the "CNVDetectionResul
```

onXpress_015_R_2012_09_10_22_39_38_Sequoia_SN1.23.Run_18_Run_18_hg19_

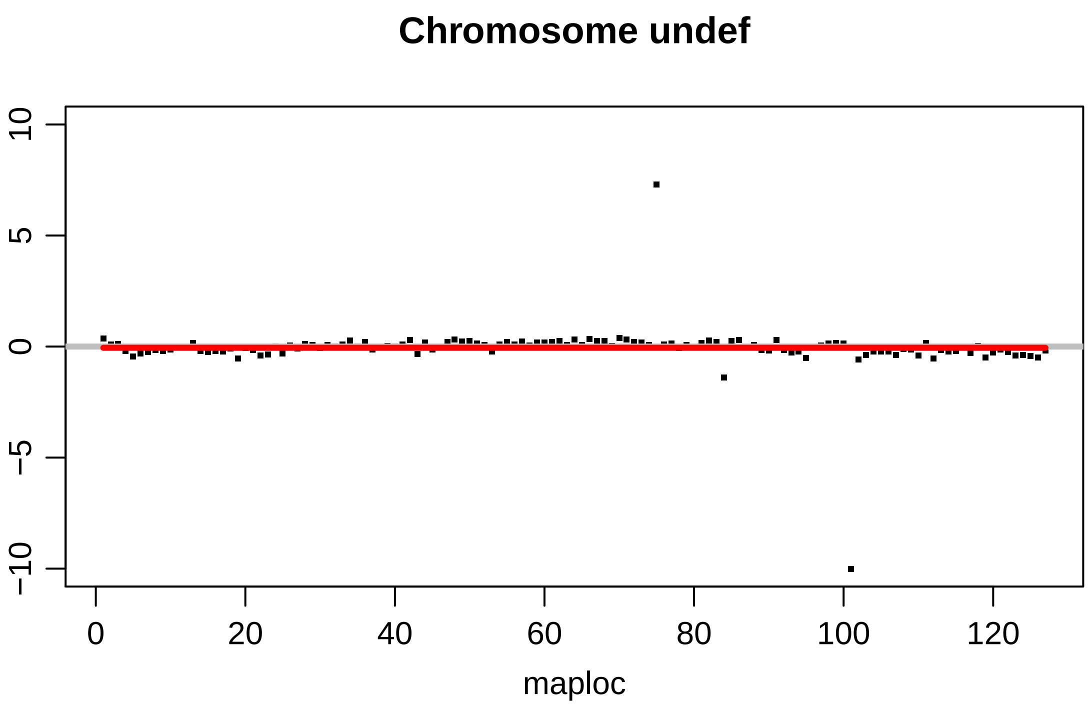


```
## Segplot might not work because of special characters in the sample names. Use only A-Z,a-z and 0-9!
##  There is a hidden function cn.mops:::.replaceNames that replaces the names in the "CNVDetectionResul
```

onXpress_016_R_2012_09_10_22_39_38_Sequoia_SN1.23.Run_18_Run_18_hg19_

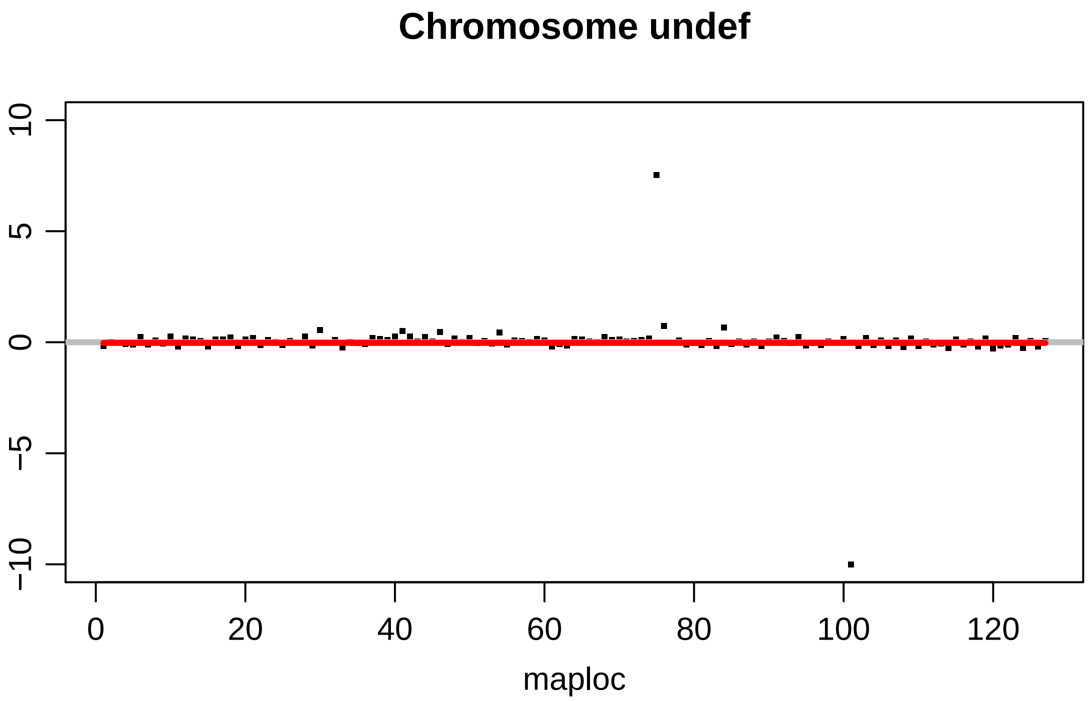

```
## Segplot might not work because of special characters in the sample names. Use only A-Z,a-z and 0-9!
##  There is a hidden function cn.mops:::.replaceNames that replaces the names in the "CNVDetectionResul
```

onXpress_017_R_2012_09_10_22_39_38_Sequoia_SN1.23.Run_18_Run_18_hg19_

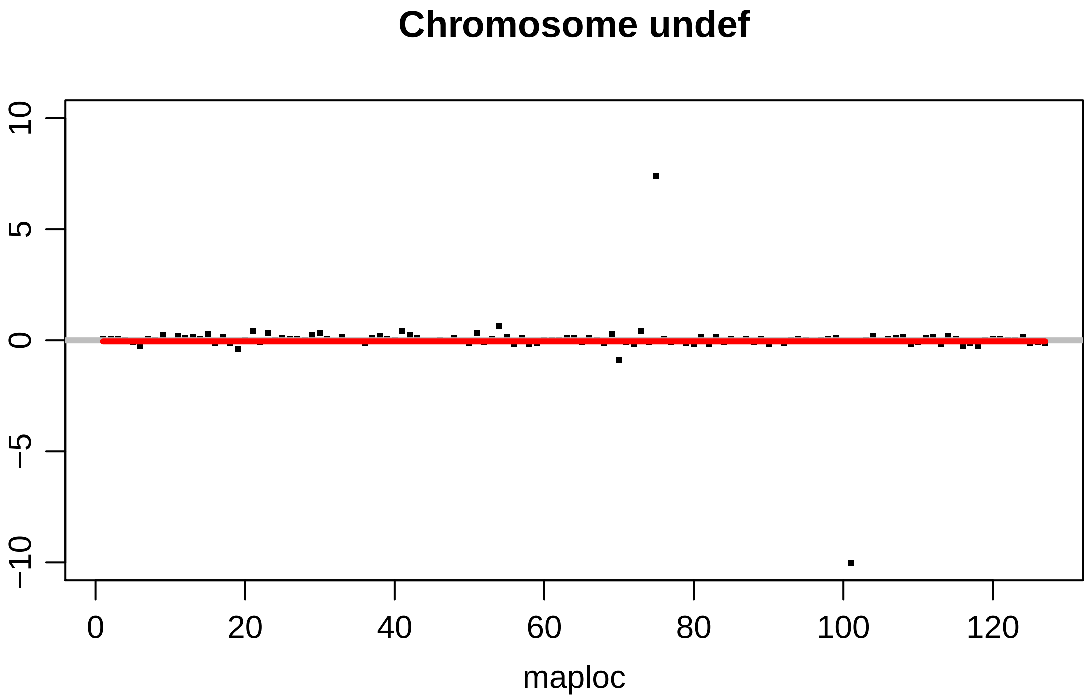


```
## Segplot might not work because of special characters in the sample names. Use only A-Z,a-z and 0-9!
##  There is a hidden function cn.mops:::.replaceNames that replaces the names in the "CNVDetectionResul
```

onXpress_018_R_2012_09_10_22_39_38_Sequoia_SN1.23.Run_18_Run_18_hg19_

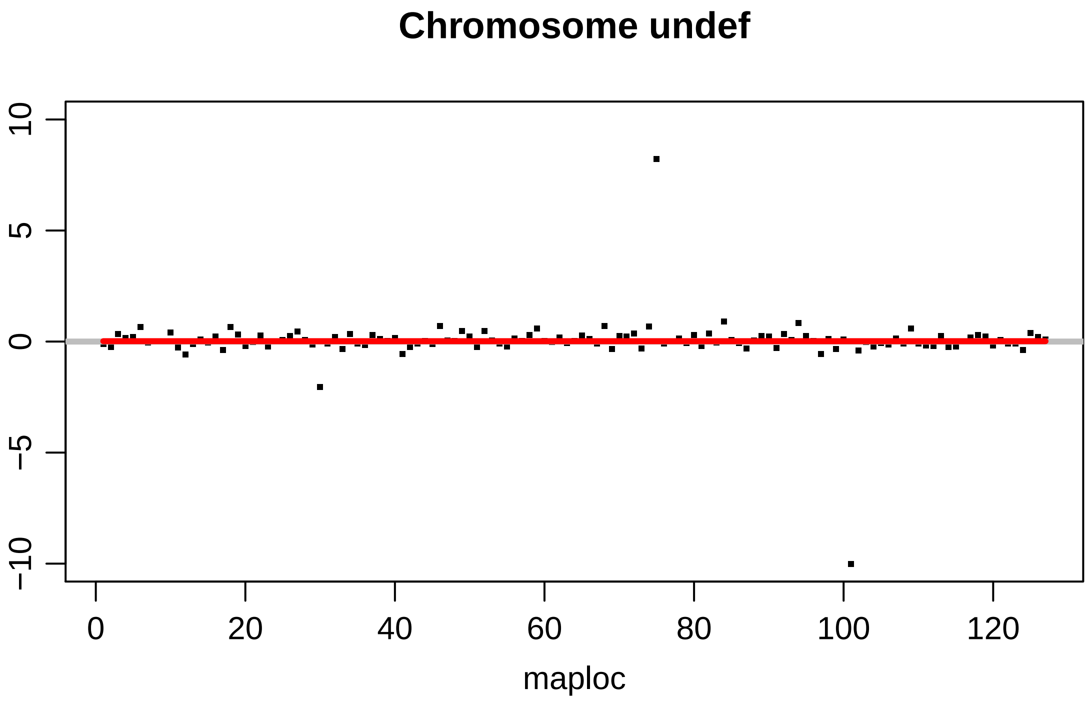


```
## Segplot might not work because of special characters in the sample names. Use only A-Z,a-z and 0-9!
##  There is a hidden function cn.mops:::.replaceNames that replaces the names in the "CNVDetectionResul
```

onXpress_020_R_2012_09_10_22_39_38_Sequoia_SN1.23.Run_18_Run_18_hg19_

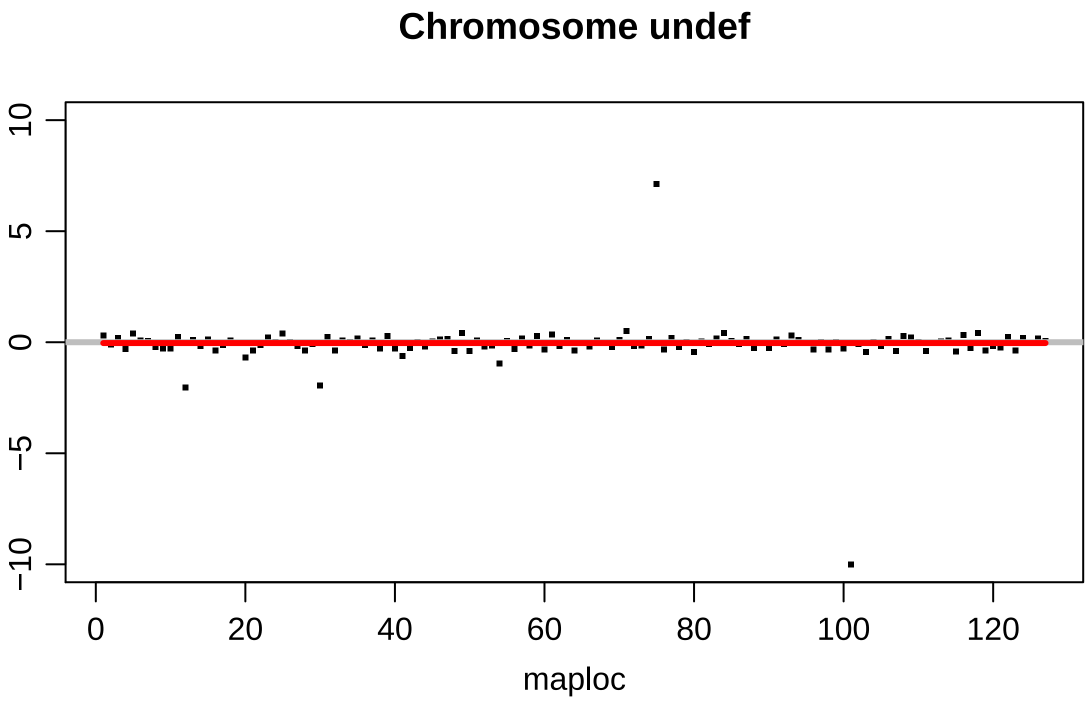

```
## Segplot might not work because of special characters in the sample names. Use only A-Z,a-z and 0-9!
##  There is a hidden function cn.mops:::.replaceNames that replaces the names in the "CNVDetectionResul
```

onXpress_021_R_2012_09_10_22_39_38_Sequoia_SN1.23.Run_18_Run_18_hg19_

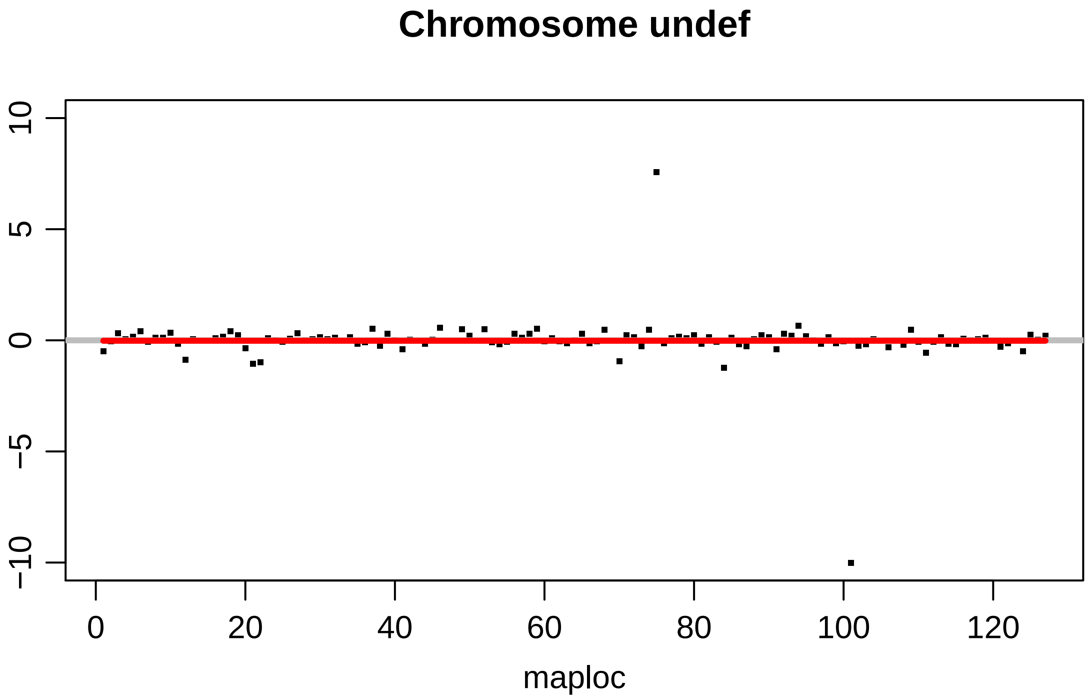


```
## Segplot might not work because of special characters in the sample names. Use only A-Z,a-z and 0-9!
##  There is a hidden function cn.mops:::.replaceNames that replaces the names in the "CNVDetectionResul
```

onXpress_022_R_2012_09_10_22_39_38_Sequoia_SN1.23.Run_18_Run_18_hg19_

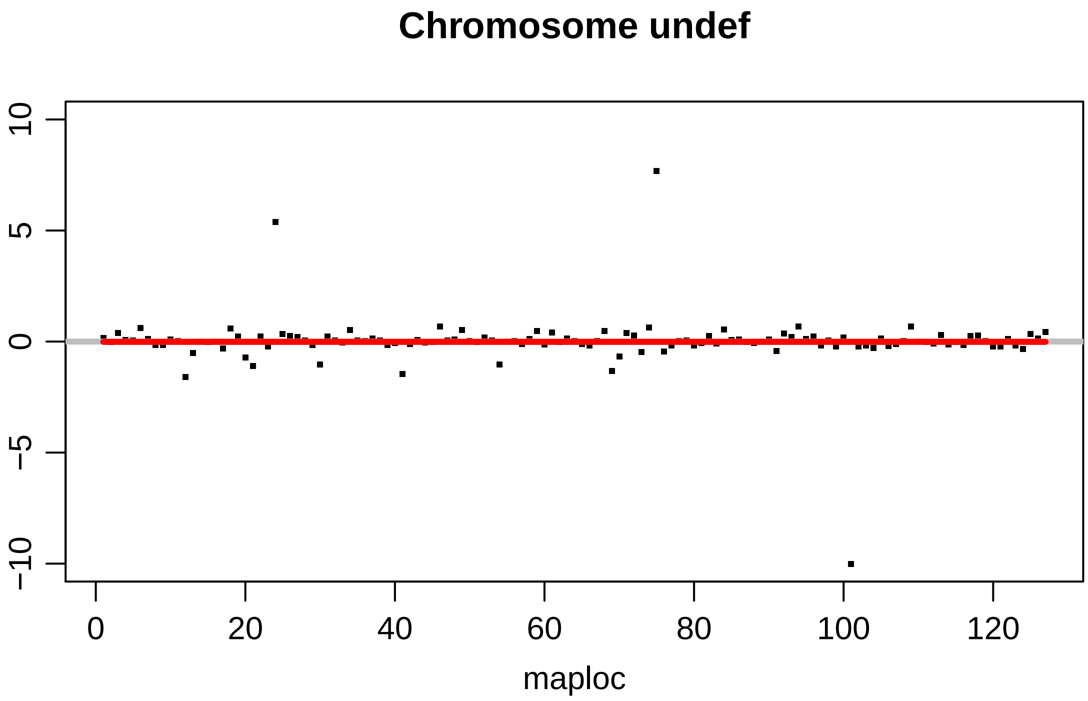


```
## Segplot might not work because of special characters in the sample names. Use only A-Z,a-z and 0-9!
##  There is a hidden function cn.mops:::.replaceNames that replaces the names in the "CNVDetectionResul
```

onXpress_023_R_2012_09_10_22_39_38_Sequoia_SN1.23.Run_18_Run_18_hg19_

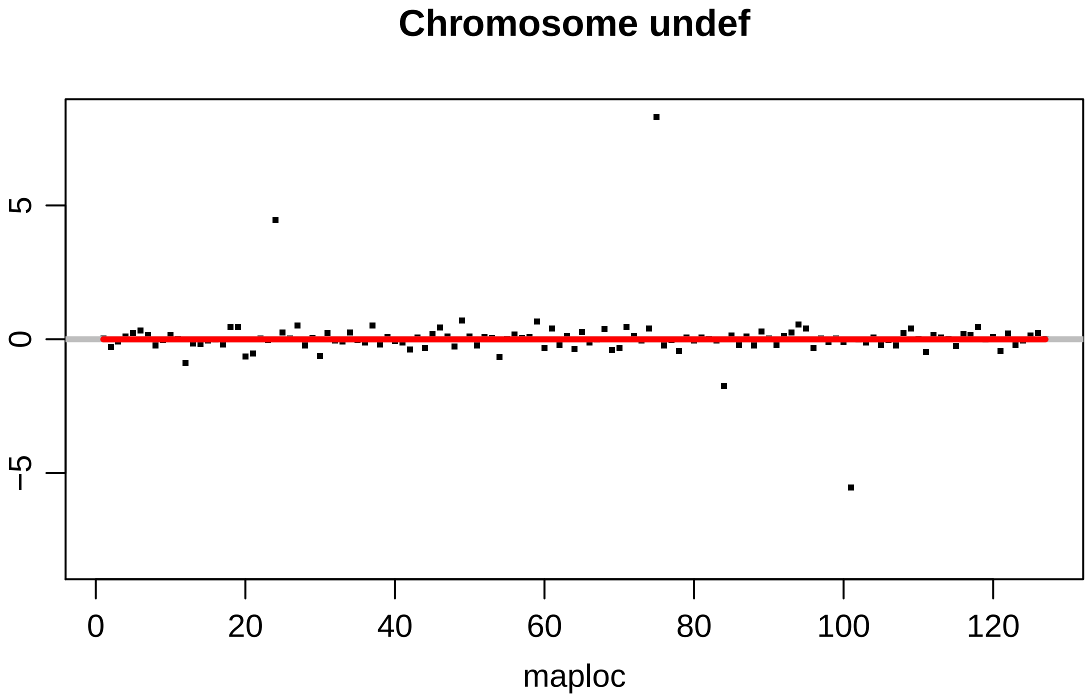

```
## Segplot might not work because of special characters in the sample names. Use only A-Z,a-z and 0-9!
##  There is a hidden function cn.mops:::.replaceNames that replaces the names in the "CNVDetectionResul
```

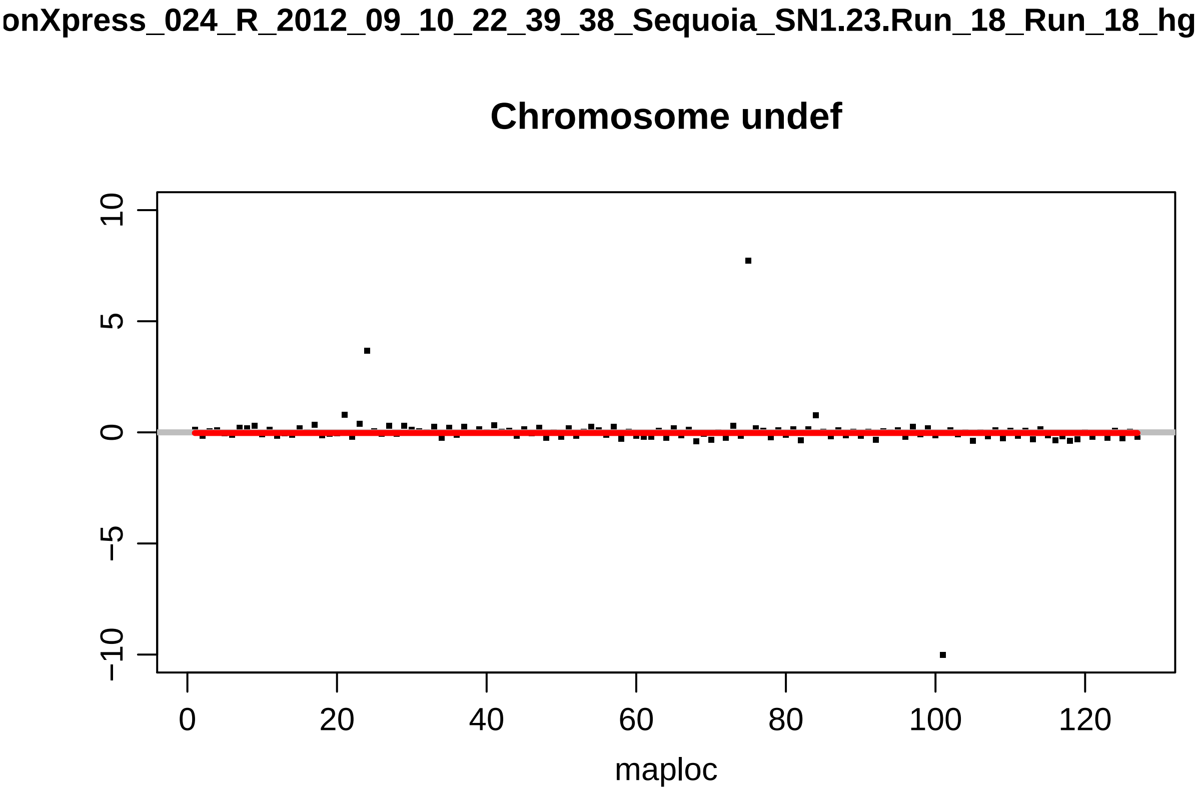


```
## Segplot might not work because of special characters in the sample names. Use only A-Z,a-z and 0-9!
##  There is a hidden function cn.mops:::.replaceNames that replaces the names in the "CNVDetectionResul
```

onXpress_025_R_2012_09_10_22_39_38_Sequoia_SN1.23.Run_18_Run_18_hg19_

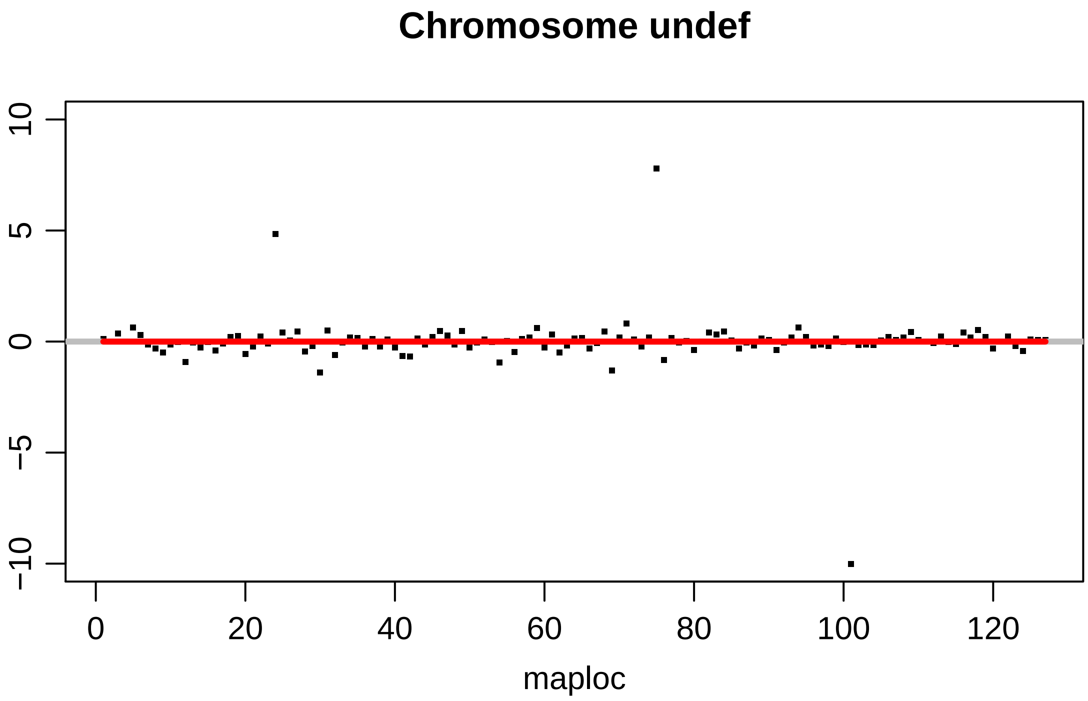


```
## Segplot might not work because of special characters in the sample names. Use only A-Z,a-z and 0-9!
##  There is a hidden function cn.mops:::.replaceNames that replaces the names in the "CNVDetectionResul
```

onXpress_026_R_2012_09_10_22_39_38_Sequoia_SN1.23.Run_18_Run_18_hg19_

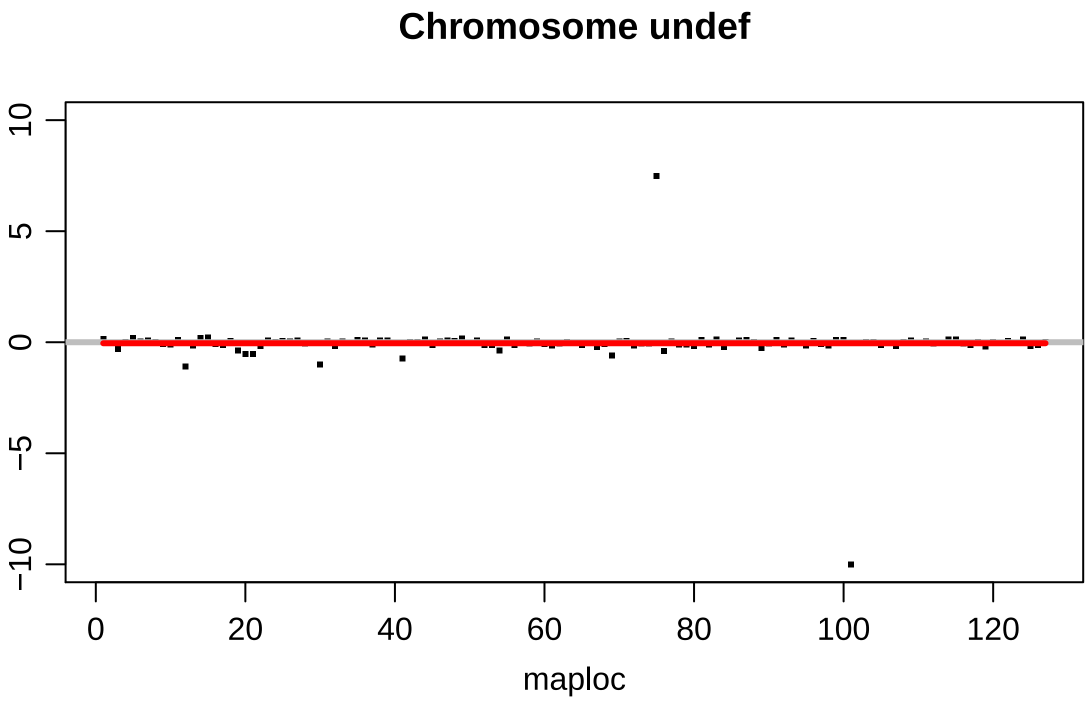

```
## Segplot might not work because of special characters in the sample names. Use only A-Z,a-z and 0-9!
##  There is a hidden function cn.mops:::.replaceNames that replaces the names in the "CNVDetectionResul
```

onXpress_027_R_2012_09_10_22_39_38_Sequoia_SN1.23.Run_18_Run_18_hg19_

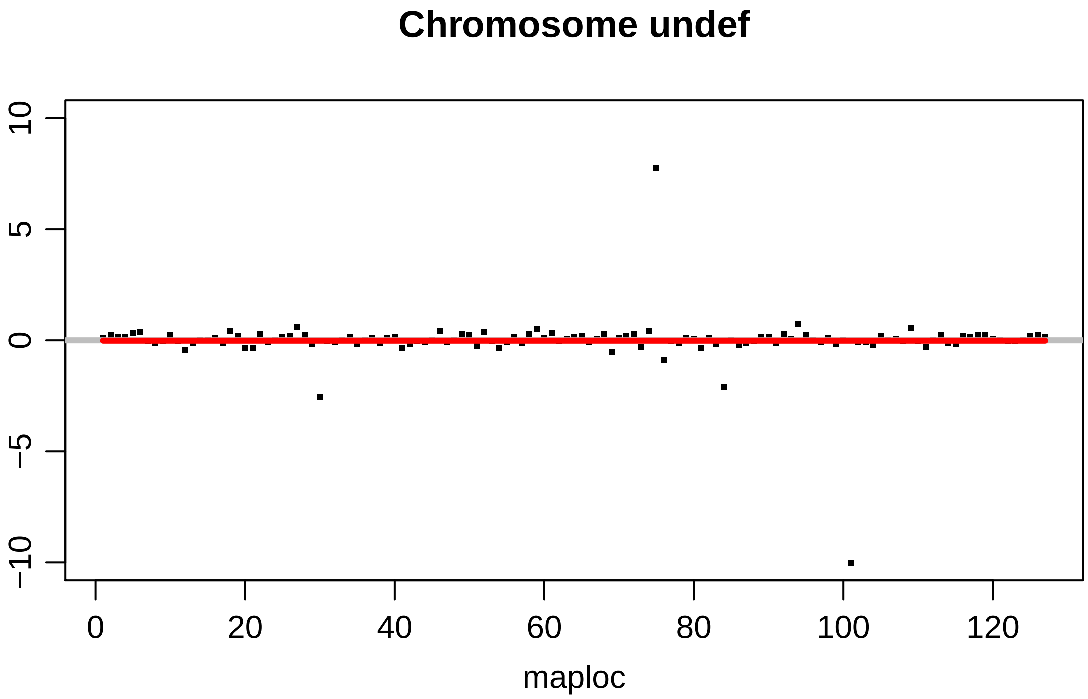


```
## Segplot might not work because of special characters in the sample names. Use only A-Z,a-z and 0-9!
##  There is a hidden function cn.mops:::.replaceNames that replaces the names in the "CNVDetectionResul
```

onXpress_028_R_2012_09_10_22_39_38_Sequoia_SN1.23.Run_18_Run_18_hg19_

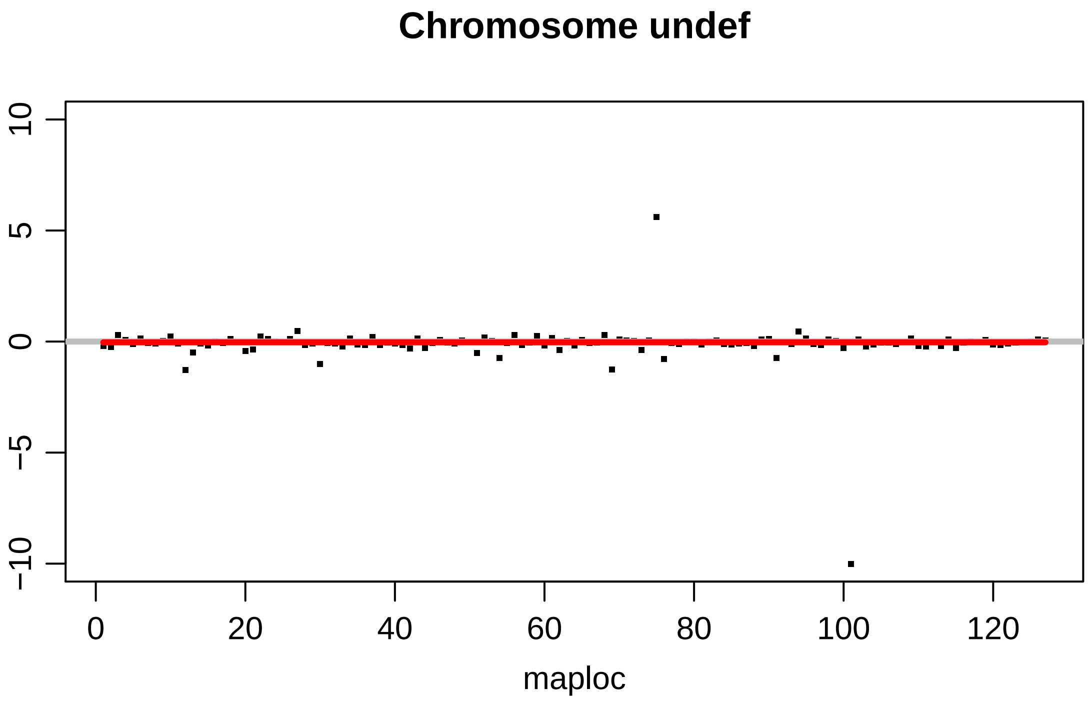


```
## Segplot might not work because of special characters in the sample names. Use only A-Z,a-z and 0-9!
##  There is a hidden function cn.mops:::.replaceNames that replaces the names in the "CNVDetectionResul
```

onXpress_029_R_2012_09_10_22_39_38_Sequoia_SN1.23.Run_18_Run_18_hg19_

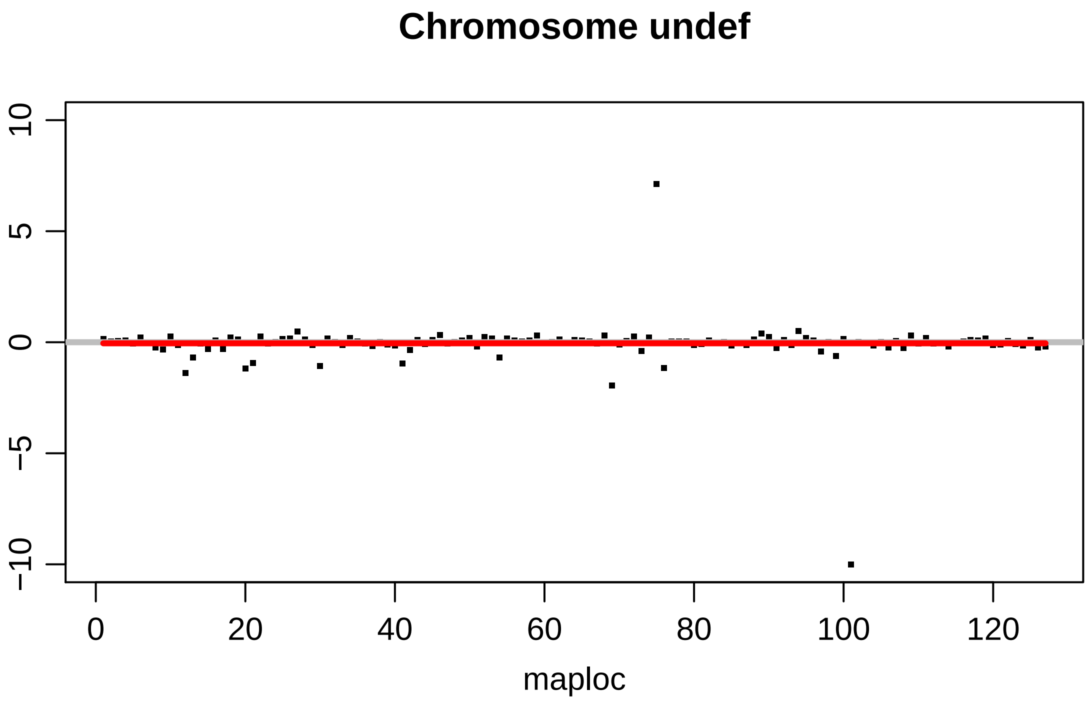

```
## Segplot might not work because of special characters in the sample names. Use only A-Z,a-z and 0-9!
##  There is a hidden function cn.mops:::.replaceNames that replaces the names in the "CNVDetectionResul
```

onXpress_030_R_2012_09_10_22_39_38_Sequoia_SN1.23.Run_18_Run_18_hg19_

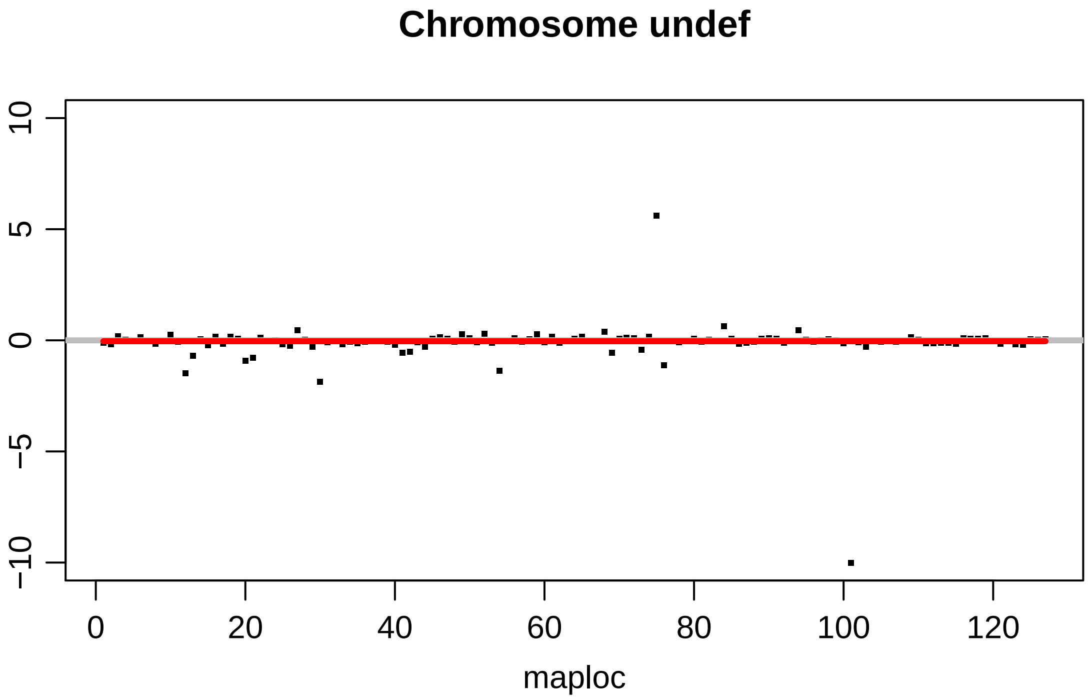


```
## Segplot might not work because of special characters in the sample names. Use only A-Z,a-z and 0-9!
##  There is a hidden function cn.mops:::.replaceNames that replaces the names in the "CNVDetectionResul
```

onXpress_031_R_2012_09_10_22_39_38_Sequoia_SN1.23.Run_18_Run_18_hg19_

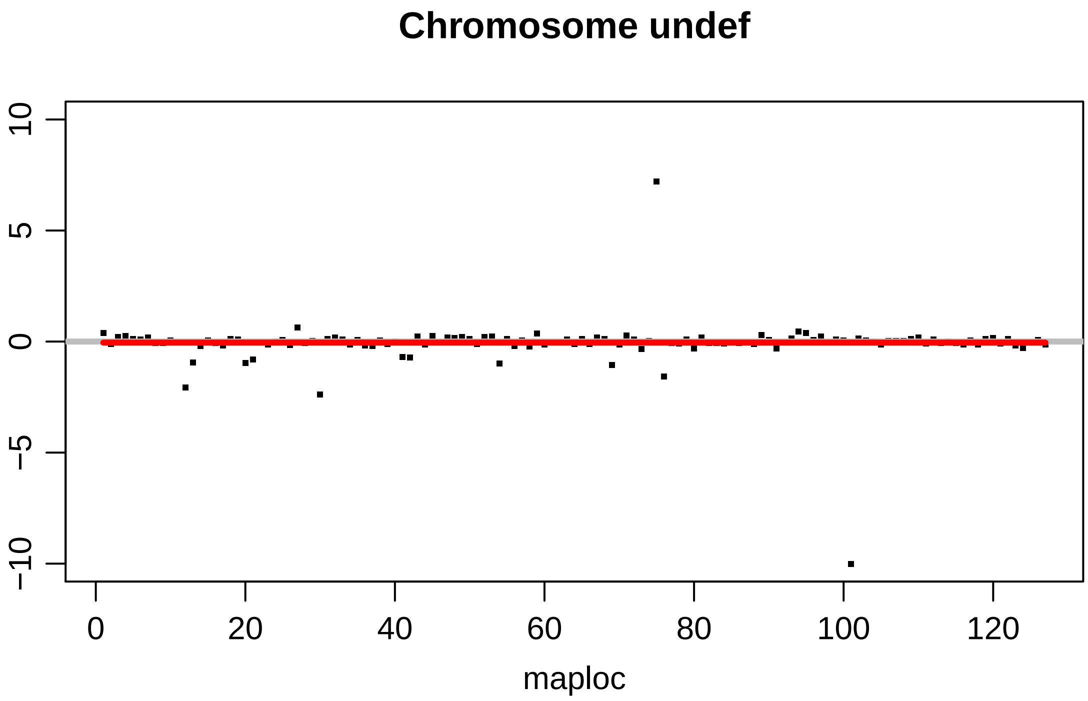


```
## Segplot might not work because of special characters in the sample names. Use only A-Z,a-z and 0-9!
##  There is a hidden function cn.mops:::.replaceNames that replaces the names in the "CNVDetectionResul
```

onXpress_032_R_2012_09_10_22_39_38_Sequoia_SN1.23.Run_18_Run_18_hg19_

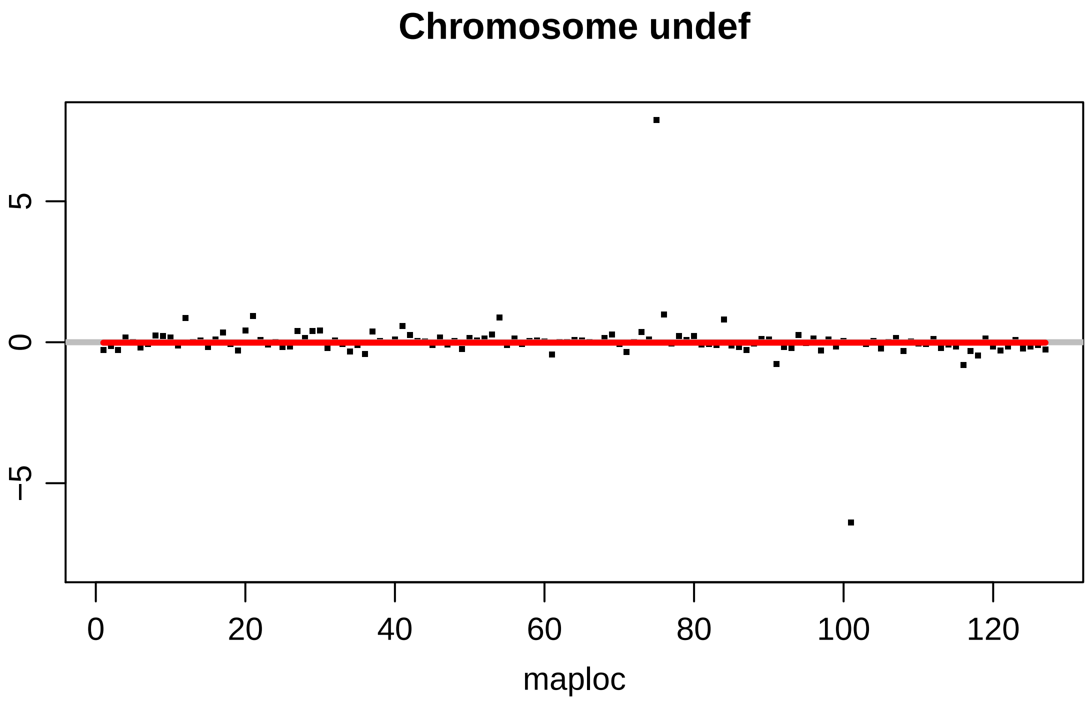

```
## Segplot might not work because of special characters in the sample names. Use only A-Z,a-z and 0-9!
##  There is a hidden function cn.mops:::.replaceNames that replaces the names in the "CNVDetectionResul
```

onXpress_041_R_2012_09_10_22_39_38_Sequoia_SN1.23.Run_18_Run_18_hg19_

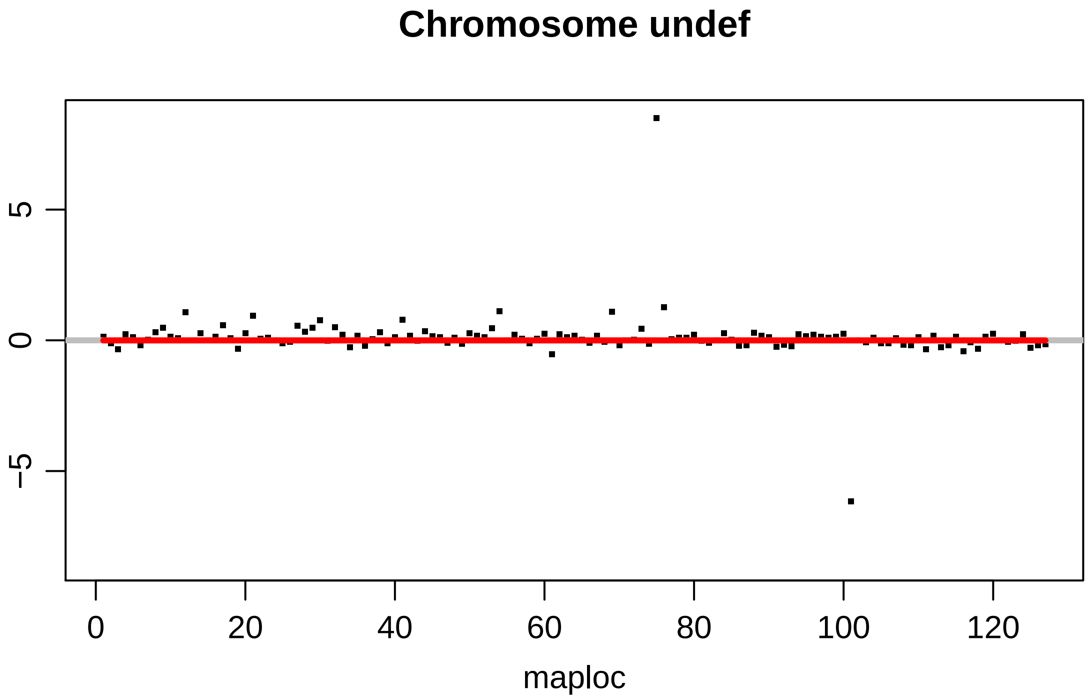


```
## Segplot might not work because of special characters in the sample names. Use only A-Z,a-z and 0-9!
##  There is a hidden function cn.mops:::.replaceNames that replaces the names in the "CNVDetectionResul
```

onXpress_042_R_2012_09_10_22_39_38_Sequoia_SN1.23.Run_18_Run_18_hg19_

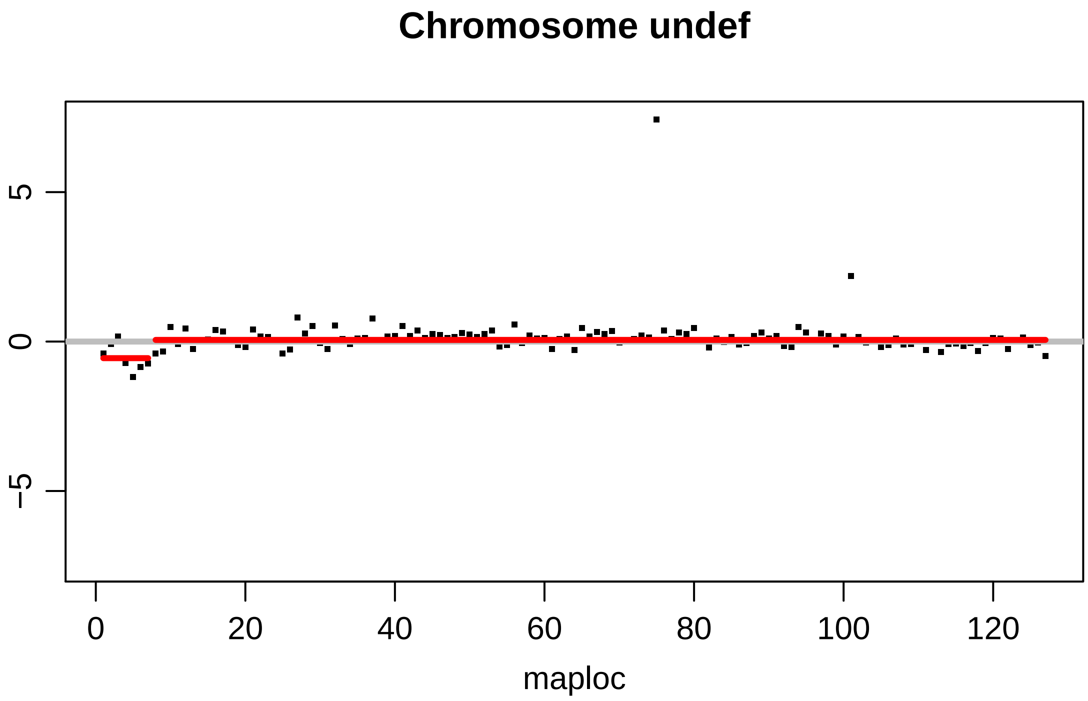


```
## Segplot might not work because of special characters in the sample names. Use only A-Z,a-z and 0-9!
##  There is a hidden function cn.mops:::.replaceNames that replaces the names in the "CNVDetectionResul
```

onXpress_043_R_2012_09_10_22_39_38_Sequoia_SN1.23.Run_18_Run_18_hg19_

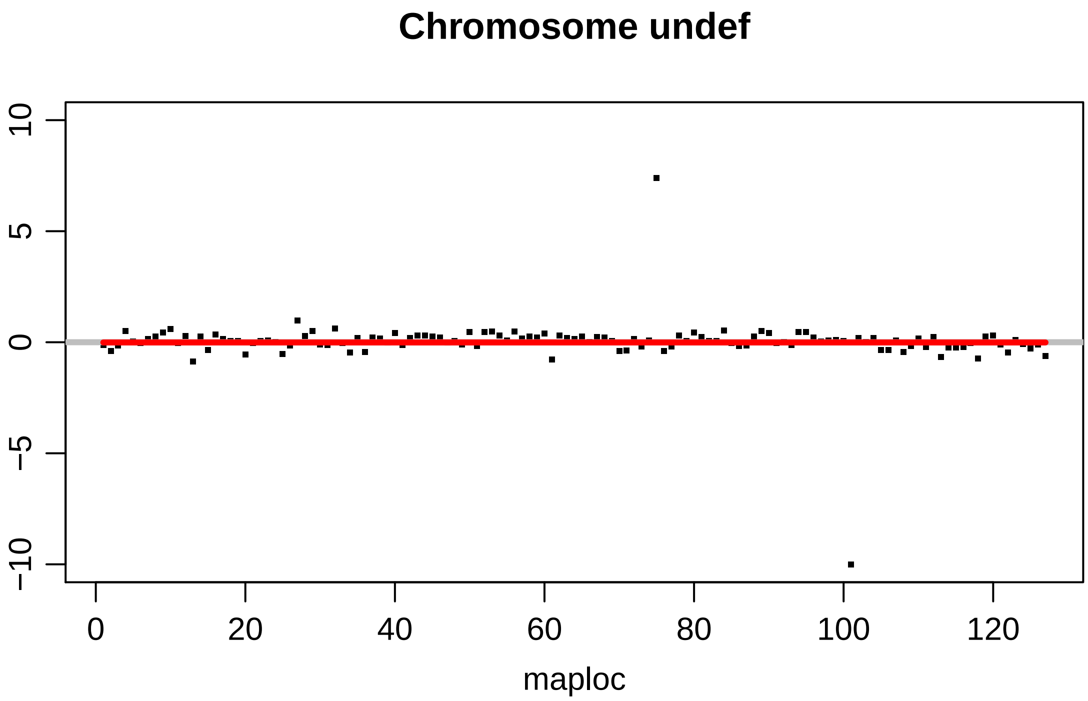

```
## Segplot might not work because of special characters in the sample names. Use only A-Z,a-z and 0-9!
##  There is a hidden function cn.mops:::.replaceNames that replaces the names in the "CNVDetectionResu
```

onXpress_044_R_2012_09_10_22_39_38_Sequoia_SN1.23.Run_18_Run_18_hg19_

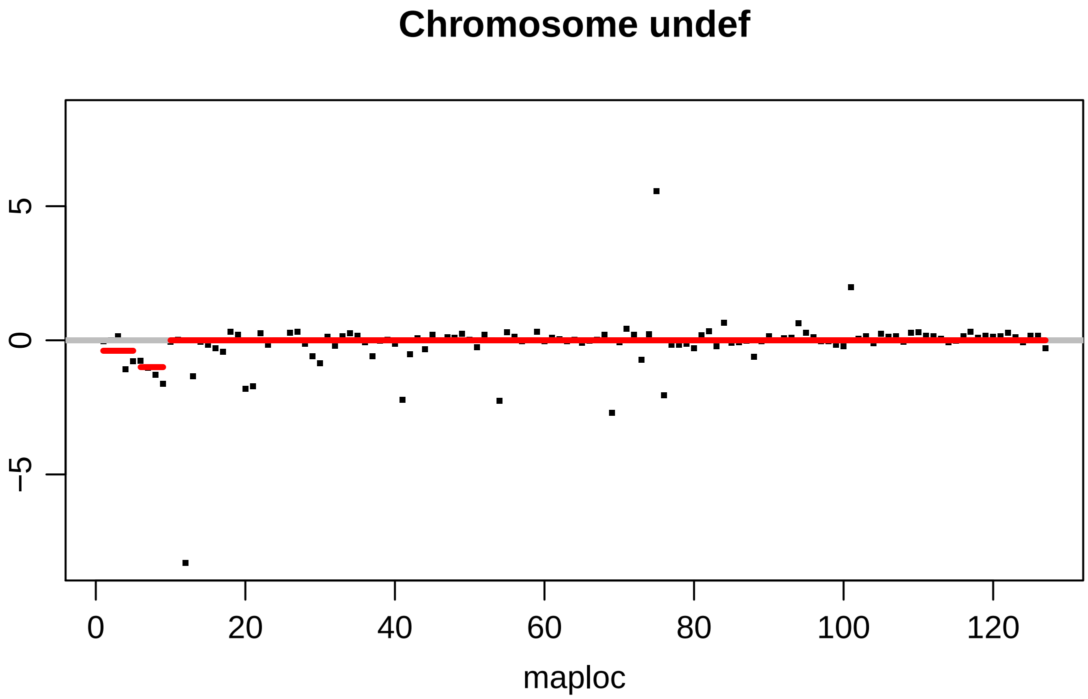


```
## Segplot might not work because of special characters in the sample names. Use only A-Z,a-z and 0-9!
##  There is a hidden function cn.mops:::.replaceNames that replaces the names in the "CNVDetectionResu
```

onXpress_045_R_2012_09_10_22_39_38_Sequoia_SN1.23.Run_18_Run_18_hg19_

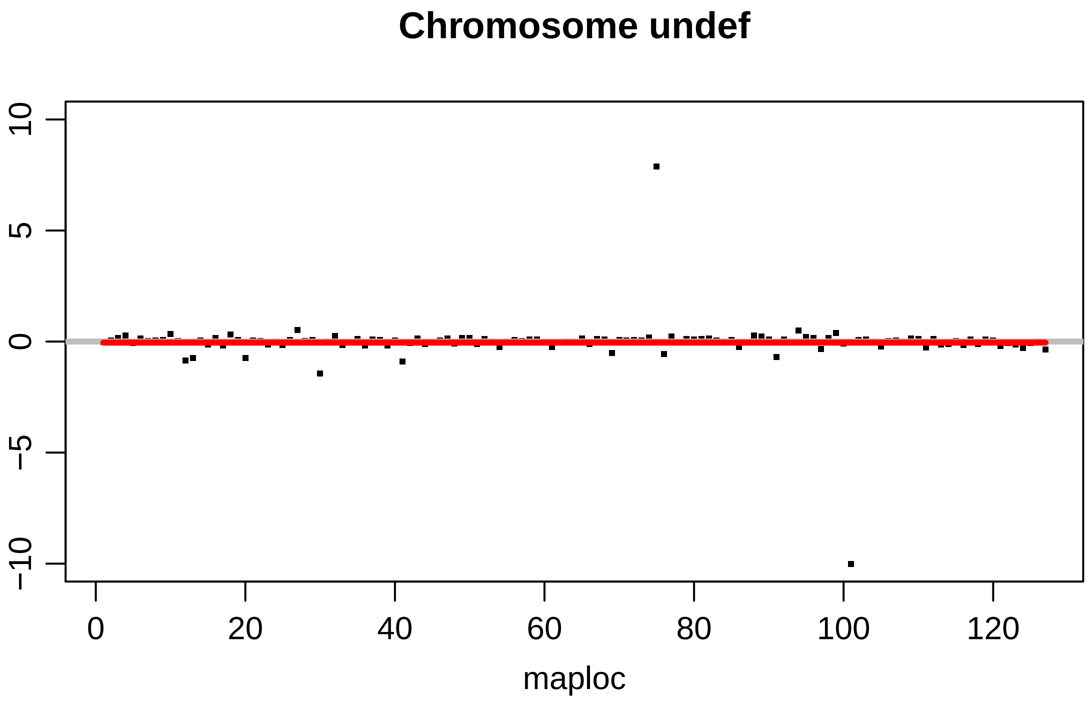


```
## Segplot might not work because of special characters in the sample names. Use only A-Z,a-z and 0-9!
##  There is a hidden function cn.mops:::.replaceNames that replaces the names in the "CNVDetectionResu
```

onXpress_046_R_2012_09_10_22_39_38_Sequoia_SN1.23.Run_18_Run_18_hg19_

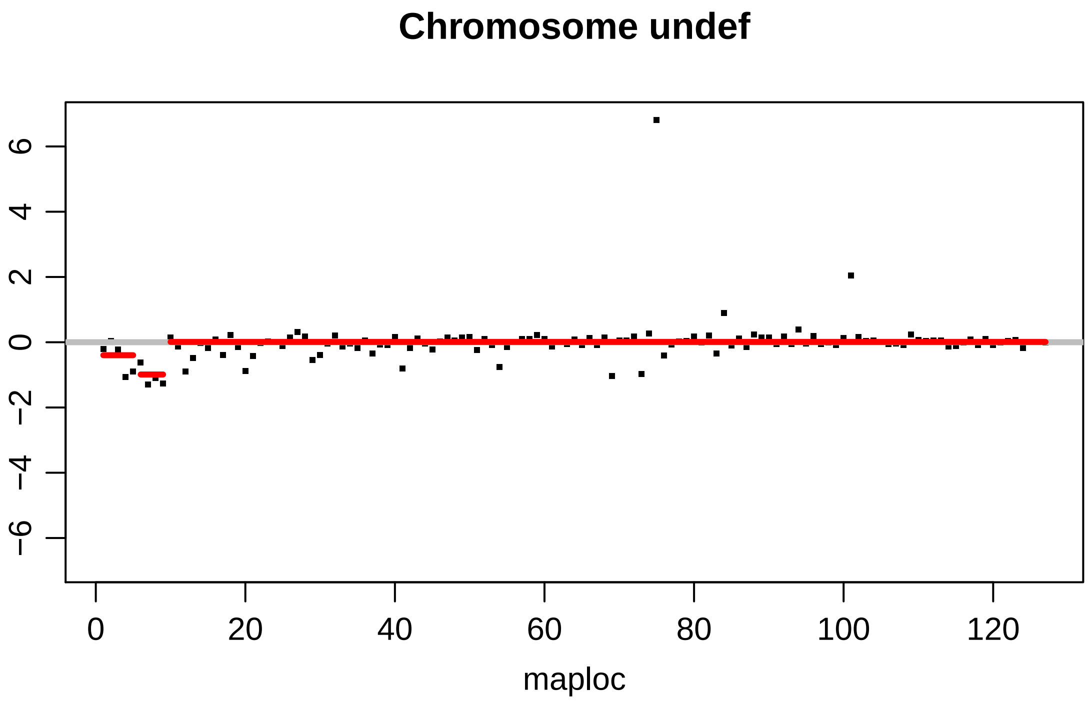

```
## Segplot might not work because of special characters in the sample names. Use only A-Z,a-z and 0-9!
##  There is a hidden function cn.mops:::.replaceNames that replaces the names in the "CNVDetectionResul
```

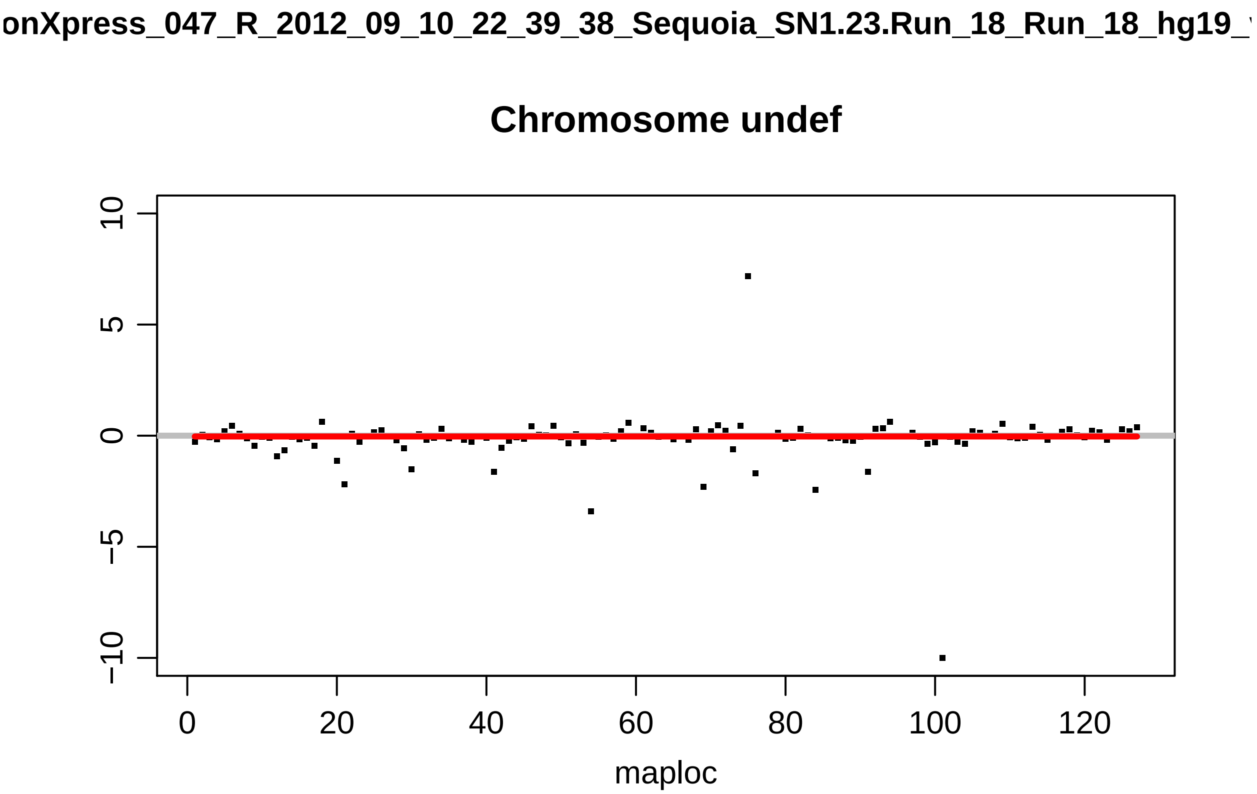


```
##
## CNV regions:
## GRanges object with 1 range and 38 metadata columns:
##       seqnames    ranges strand |
##          <Rle> <IRanges>  <Rle> |
##   [1]    undef    [6, 9]      * |
##       Case_IonXpress_001_R_2012_09_10_22_39_38_Sequoia_SN1.23.Run_18_Run_18_hg19_v3.sam
##                                                                                <factor>
##   [1]                                                                               CN2
##       Case_IonXpress_002_R_2012_09_10_22_39_38_Sequoia_SN1.23.Run_18_Run_18_hg19_v3.sam
##                                                                                <factor>
##   [1]                                                                               CN2
##       Case_IonXpress_003_R_2012_09_10_22_39_38_Sequoia_SN1.23.Run_18_Run_18_hg19_v3.sam
##                                                                                <factor>
##   [1]                                                                               CN2
##       Case_IonXpress_004_R_2012_09_10_22_39_38_Sequoia_SN1.23.Run_18_Run_18_hg19_v3.sam
##                                                                                <factor>
##   [1]                                                                               CN2
##       Case_IonXpress_005_R_2012_09_10_22_39_38_Sequoia_SN1.23.Run_18_Run_18_hg19_v3.sam
##                                                                                <factor>
##   [1]                                                                               CN2
##       Case_IonXpress_006_R_2012_09_10_22_39_38_Sequoia_SN1.23.Run_18_Run_18_hg19_v3.sam
##                                                                                <factor>
##   [1]                                                                               CN2
##       Case_IonXpress_007_R_2012_09_10_22_39_38_Sequoia_SN1.23.Run_18_Run_18_hg19_v3.sam
```

```
##                                                                                  <factor>
##    [1]                                                                               CN2
##        Case_IonXpress_008_R_2012_09_10_22_39_38_Sequoia_SN1.23.Run_18_Run_18_hg19_v3.sam
##                                                                                  <factor>
##    [1]                                                                               CN2
##        Case_IonXpress_009_R_2012_09_10_22_39_38_Sequoia_SN1.23.Run_18_Run_18_hg19_v3.sam
##                                                                                  <factor>
##    [1]                                                                               CN2
##        Case_IonXpress_010_R_2012_09_10_22_39_38_Sequoia_SN1.23.Run_18_Run_18_hg19_v3.sam
##                                                                                  <factor>
##    [1]                                                                               CN2
##        Case_IonXpress_011_R_2012_09_10_22_39_38_Sequoia_SN1.23.Run_18_Run_18_hg19_v3.sam
##                                                                                  <factor>
##    [1]                                                                               CN2
##        Case_IonXpress_012_R_2012_09_10_22_39_38_Sequoia_SN1.23.Run_18_Run_18_hg19_v3.sam
##                                                                                  <factor>
##    [1]                                                                               CN2
##        Case_IonXpress_013_R_2012_09_10_22_39_38_Sequoia_SN1.23.Run_18_Run_18_hg19_v3.sam
##                                                                                  <factor>
##    [1]                                                                               CN2
##        Case_IonXpress_015_R_2012_09_10_22_39_38_Sequoia_SN1.23.Run_18_Run_18_hg19_v3.sam
##                                                                                  <factor>
##    [1]                                                                               CN2
##        Case_IonXpress_016_R_2012_09_10_22_39_38_Sequoia_SN1.23.Run_18_Run_18_hg19_v3.sam
##                                                                                  <factor>
##    [1]                                                                               CN2
##        Case_IonXpress_017_R_2012_09_10_22_39_38_Sequoia_SN1.23.Run_18_Run_18_hg19_v3.sam
##                                                                                  <factor>
##    [1]                                                                               CN2
##        Case_IonXpress_018_R_2012_09_10_22_39_38_Sequoia_SN1.23.Run_18_Run_18_hg19_v3.sam
##                                                                                  <factor>
##    [1]                                                                               CN2
##        Case_IonXpress_020_R_2012_09_10_22_39_38_Sequoia_SN1.23.Run_18_Run_18_hg19_v3.sam
##                                                                                  <factor>
##    [1]                                                                               CN2
##        Case_IonXpress_021_R_2012_09_10_22_39_38_Sequoia_SN1.23.Run_18_Run_18_hg19_v3.sam
##                                                                                  <factor>
##    [1]                                                                               CN2
##        Case_IonXpress_022_R_2012_09_10_22_39_38_Sequoia_SN1.23.Run_18_Run_18_hg19_v3.sam
##                                                                                  <factor>
##    [1]                                                                               CN2
##        Case_IonXpress_023_R_2012_09_10_22_39_38_Sequoia_SN1.23.Run_18_Run_18_hg19_v3.sam
##                                                                                  <factor>
##    [1]                                                                               CN2
##        Case_IonXpress_024_R_2012_09_10_22_39_38_Sequoia_SN1.23.Run_18_Run_18_hg19_v3.sam
##                                                                                  <factor>
##    [1]                                                                               CN2
##        Case_IonXpress_025_R_2012_09_10_22_39_38_Sequoia_SN1.23.Run_18_Run_18_hg19_v3.sam
##                                                                                  <factor>
##    [1]                                                                               CN2
##        Case_IonXpress_026_R_2012_09_10_22_39_38_Sequoia_SN1.23.Run_18_Run_18_hg19_v3.sam
##                                                                                  <factor>
##    [1]                                                                               CN2
##        Case_IonXpress_027_R_2012_09_10_22_39_38_Sequoia_SN1.23.Run_18_Run_18_hg19_v3.sam
```

```
##                                                                        <factor>
##   [1]                                                                      CN2
##       Case_IonXpress_028_R_2012_09_10_22_39_38_Sequoia_SN1.23.Run_18_Run_18_hg19_v3.sam
##                                                                        <factor>
##   [1]                                                                      CN2
##       Case_IonXpress_029_R_2012_09_10_22_39_38_Sequoia_SN1.23.Run_18_Run_18_hg19_v3.sam
##                                                                        <factor>
##   [1]                                                                      CN2
##       Case_IonXpress_030_R_2012_09_10_22_39_38_Sequoia_SN1.23.Run_18_Run_18_hg19_v3.sam
##                                                                        <factor>
##   [1]                                                                      CN2
##       Case_IonXpress_031_R_2012_09_10_22_39_38_Sequoia_SN1.23.Run_18_Run_18_hg19_v3.sam
##                                                                        <factor>
##   [1]                                                                      CN2
##       Case_IonXpress_032_R_2012_09_10_22_39_38_Sequoia_SN1.23.Run_18_Run_18_hg19_v3.sam
##                                                                        <factor>
##   [1]                                                                      CN2
##       Case_IonXpress_041_R_2012_09_10_22_39_38_Sequoia_SN1.23.Run_18_Run_18_hg19_v3.sam
##                                                                        <factor>
##   [1]                                                                      CN2
##       Case_IonXpress_042_R_2012_09_10_22_39_38_Sequoia_SN1.23.Run_18_Run_18_hg19_v3.sam
##                                                                        <factor>
##   [1]                                                                      CN2
##       Case_IonXpress_043_R_2012_09_10_22_39_38_Sequoia_SN1.23.Run_18_Run_18_hg19_v3.sam
##                                                                        <factor>
##   [1]                                                                      CN2
##       Case_IonXpress_044_R_2012_09_10_22_39_38_Sequoia_SN1.23.Run_18_Run_18_hg19_v3.sam
##                                                                        <factor>
##   [1]                                                                      CN1
##       Case_IonXpress_045_R_2012_09_10_22_39_38_Sequoia_SN1.23.Run_18_Run_18_hg19_v3.sam
##                                                                        <factor>
##   [1]                                                                      CN2
##       Case_IonXpress_046_R_2012_09_10_22_39_38_Sequoia_SN1.23.Run_18_Run_18_hg19_v3.sam
##                                                                        <factor>
##   [1]                                                                      CN1
##       Case_IonXpress_047_R_2012_09_10_22_39_38_Sequoia_SN1.23.Run_18_Run_18_hg19_v3.sam
##                                                                        <factor>
##   [1]                                                                      CN2
##       Case_IonXpress_048_R_2012_09_10_22_39_38_Sequoia_SN1.23.Run_18_Run_18_hg19_v3.sam
##                                                                        <factor>
##   [1]                                                                      CN2
##   -------
##   seqinfo: 1 sequence from an unspecified genome; no seqlengths
##
## Individual CNVs:
## GRanges object with 2 ranges and 4 metadata columns:
##       seqnames    ranges strand |
##          <Rle> <IRanges>  <Rle> |
##   [1]    undef    [6, 9]      * |
##   [2]    undef    [6, 9]      * |
##                                                                      sampleName
##                                                                        <factor>
##   [1] Case_IonXpress_044_R_2012_09_10_22_39_38_Sequoia_SN1.23.Run_18_Run_18_hg19_v3.sam
##   [2] Case_IonXpress_046_R_2012_09_10_22_39_38_Sequoia_SN1.23.Run_18_Run_18_hg19_v3.sam
```

```
##           median       mean          CN
##        <numeric>  <numeric> <character>
##   [1] -0.9995223 -0.9992444         CN1
##   [2] -0.9979590 -0.9890494         CN1
##   -------
##   seqinfo: 1 sequence from an unspecified genome; no seqlengths
## [1] "/Users/gdemidov/Downloads/doc/Run_19_fin_05_qc.xls"

## Normalizing...

## Starting local modeling, please be patient...

## Reference sequence:  undef

## Starting segmentation algorithm...

## Using "fastseg" for segmentation.

## [1] ""
## [1] "/Users/gdemidov/Downloads/doc/Run_19_fin_05_qc.xls"
## [1] ""

## Segplot might not work because of special characters in the sample names. Use only A-Z,a-z and 0-9!
##  There is a hidden function cn.mops:::.replaceNames that replaces the names in the "CNVDetectionResul
```

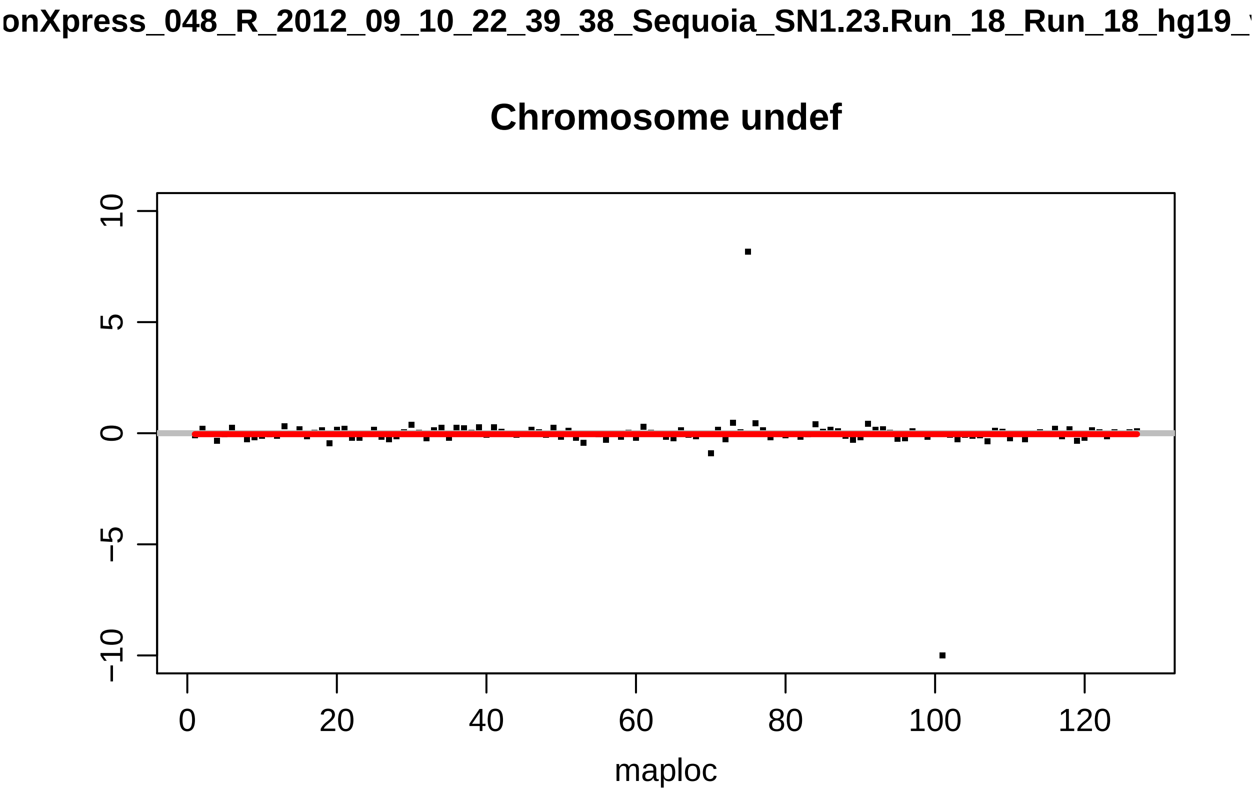


```
## Segplot might not work because of special characters in the sample names. Use only A-Z,a-z and 0-9!
##  There is a hidden function cn.mops:::.replaceNames that replaces the names in the "CNVDetectionResul
```

onXpress_001_R_2012_09_12_03_13_43_Sequoia_SN1.25.Run_19_Run_19_hg19_

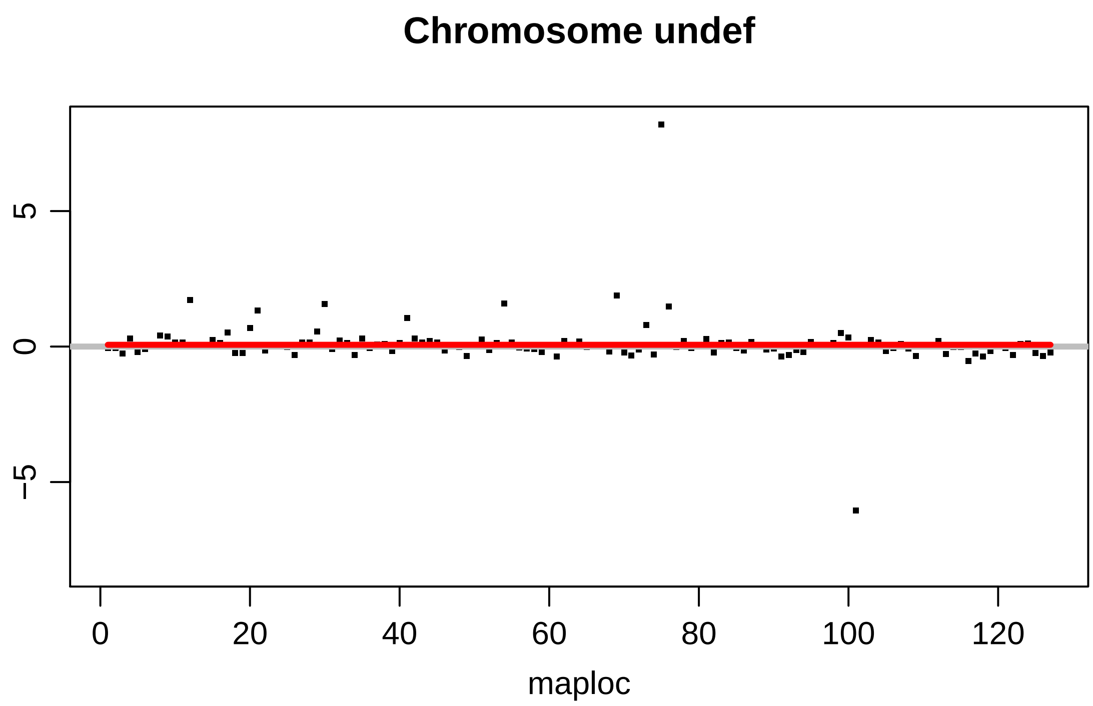


```
## Segplot might not work because of special characters in the sample names. Use only A-Z,a-z and 0-9!
##  There is a hidden function cn.mops:::.replaceNames that replaces the names in the "CNVDetectionResul
```

onXpress_002_R_2012_09_12_03_13_43_Sequoia_SN1.25.Run_19_Run_19_hg19_

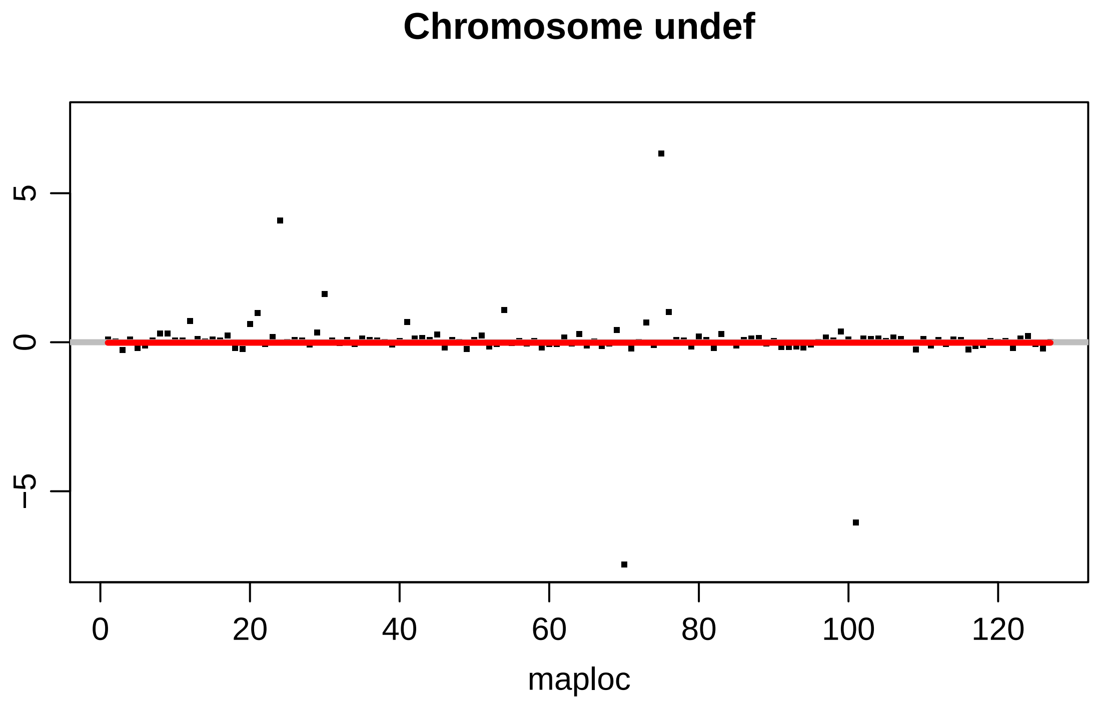

```
## Segplot might not work because of special characters in the sample names. Use only A-Z,a-z and 0-9!
##  There is a hidden function cn.mops:::.replaceNames that replaces the names in the "CNVDetectionResul
```

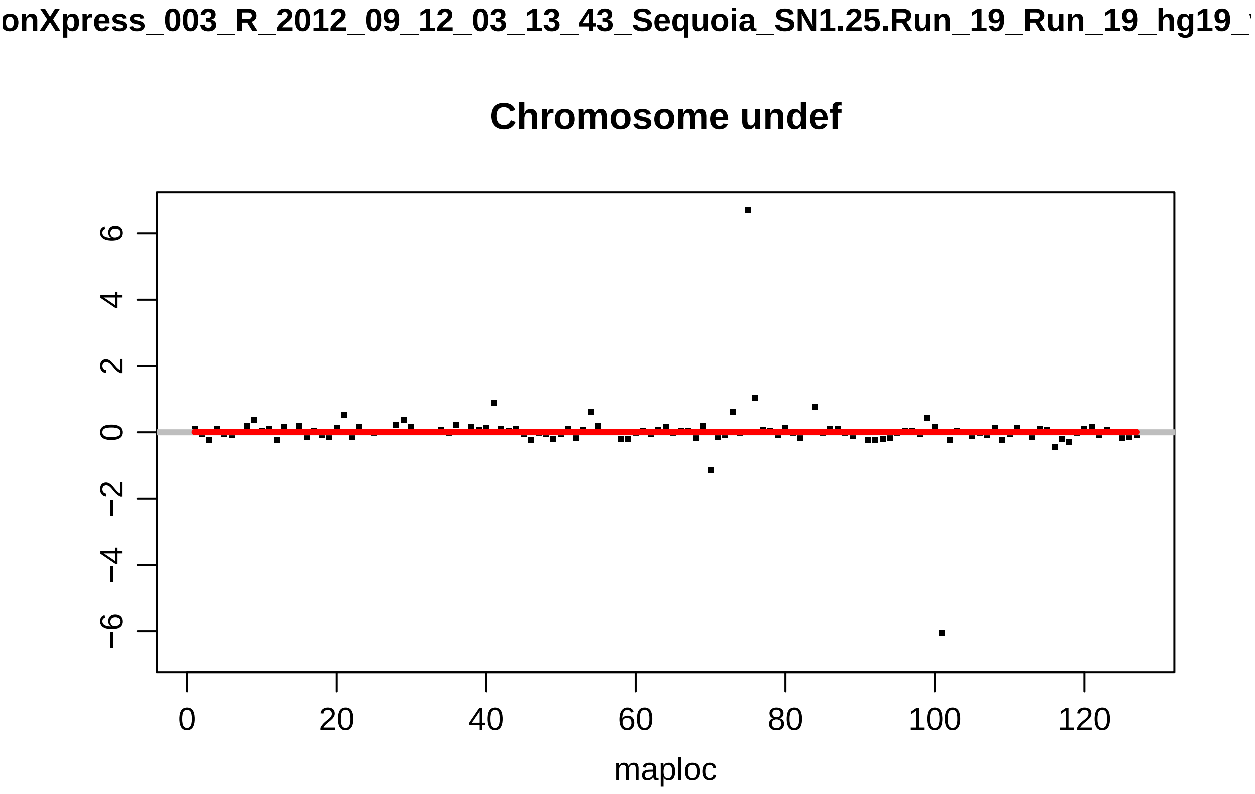


```
## Segplot might not work because of special characters in the sample names. Use only A-Z,a-z and 0-9!
##  There is a hidden function cn.mops:::.replaceNames that replaces the names in the "CNVDetectionResul
```

onXpress_004_R_2012_09_12_03_13_43_Sequoia_SN1.25.Run_19_Run_19_hg19_

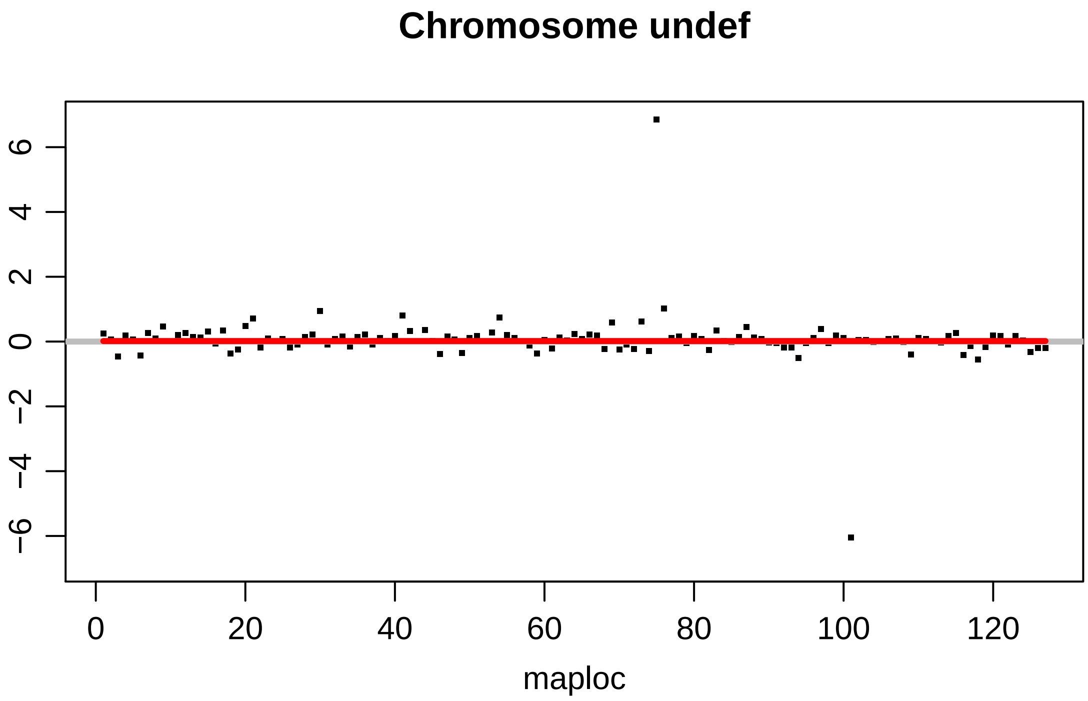


```
## Segplot might not work because of special characters in the sample names. Use only A-Z,a-z and 0-9!
##  There is a hidden function cn.mops:::.replaceNames that replaces the names in the "CNVDetectionResul
```

onXpress_005_R_2012_09_12_03_13_43_Sequoia_SN1.25.Run_19_Run_19_hg19_

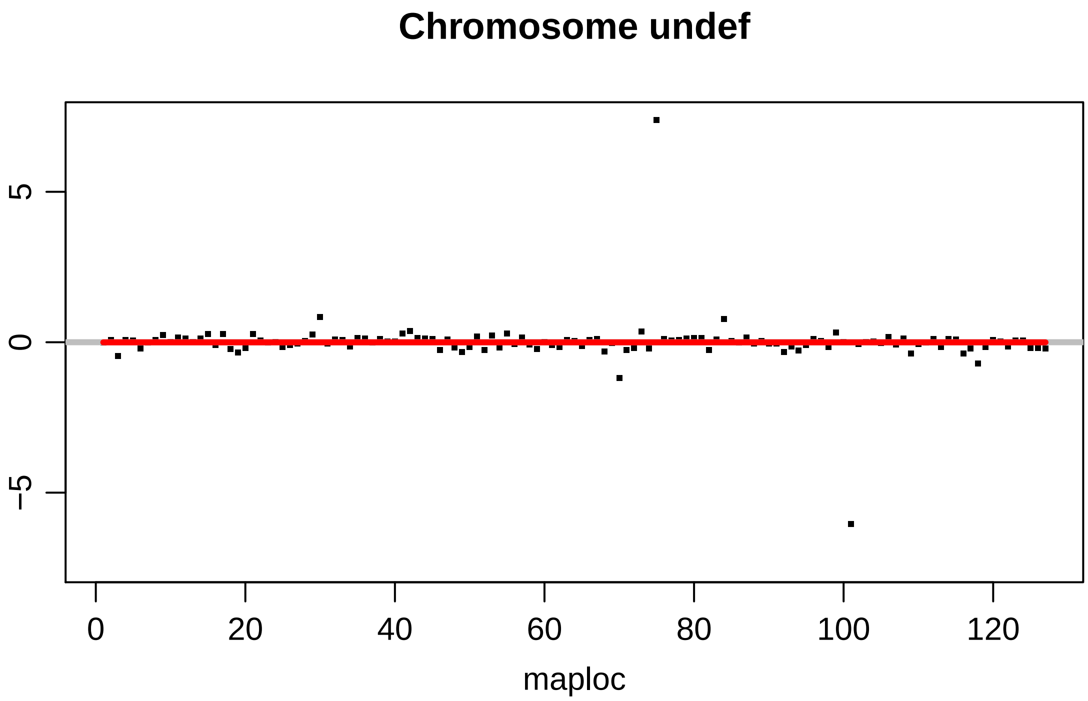

```
## Segplot might not work because of special characters in the sample names. Use only A-Z,a-z and 0-9!
##  There is a hidden function cn.mops:::.replaceNames that replaces the names in the "CNVDetectionResul
```

onXpress_006_R_2012_09_12_03_13_43_Sequoia_SN1.25.Run_19_Run_19_hg19_

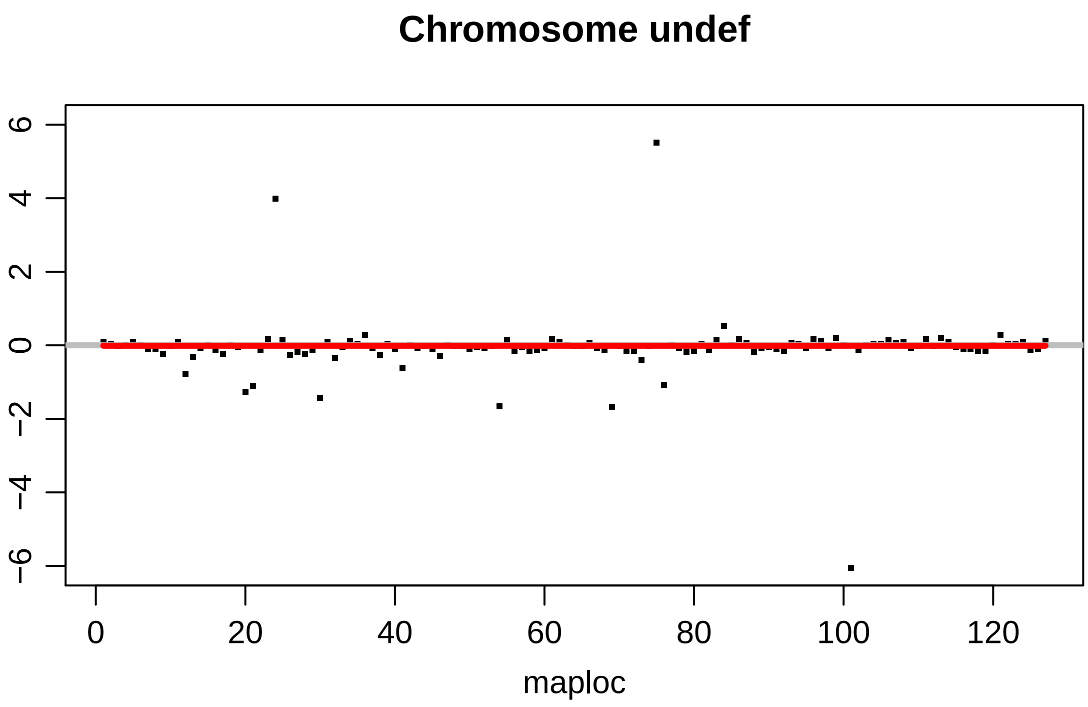


```
## Segplot might not work because of special characters in the sample names. Use only A-Z,a-z and 0-9!
##  There is a hidden function cn.mops:::.replaceNames that replaces the names in the "CNVDetectionResul
```

onXpress_007_R_2012_09_12_03_13_43_Sequoia_SN1.25.Run_19_Run_19_hg19_

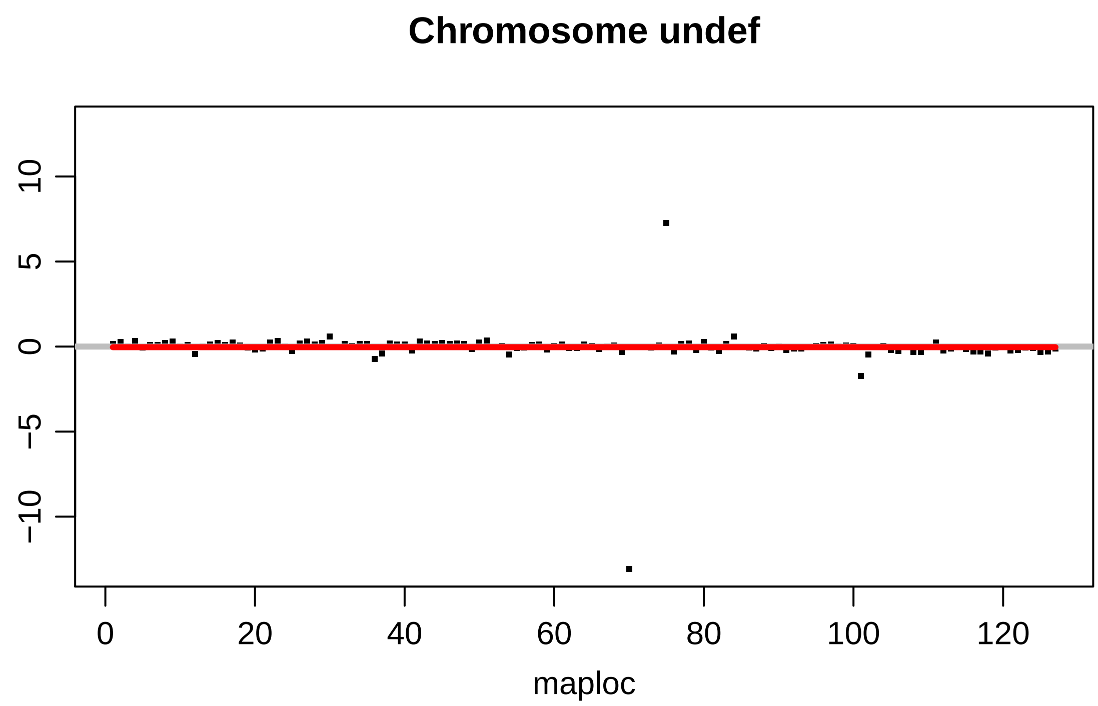


```
## Segplot might not work because of special characters in the sample names. Use only A-Z,a-z and 0-9!
##  There is a hidden function cn.mops:::.replaceNames that replaces the names in the "CNVDetectionResul
```

onXpress_008_R_2012_09_12_03_13_43_Sequoia_SN1.25.Run_19_Run_19_hg19_

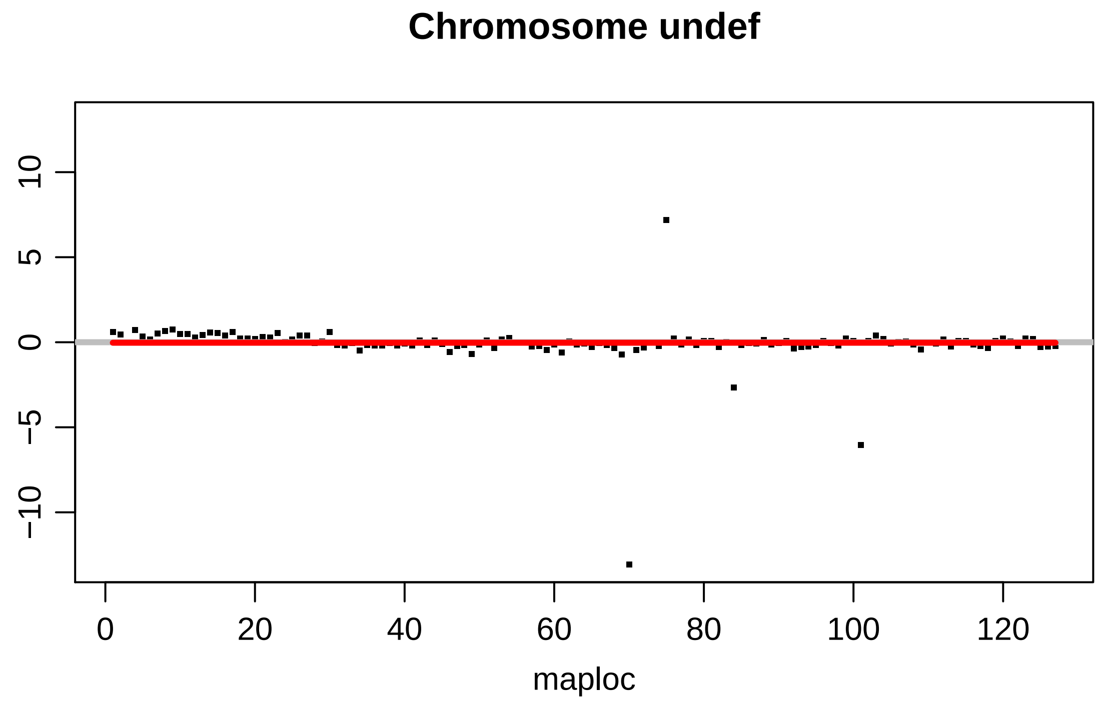

```
## Segplot might not work because of special characters in the sample names. Use only A-Z,a-z and 0-9!
##  There is a hidden function cn.mops:::.replaceNames that replaces the names in the "CNVDetectionResul
```

onXpress_009_R_2012_09_12_03_13_43_Sequoia_SN1.25.Run_19_Run_19_hg19_

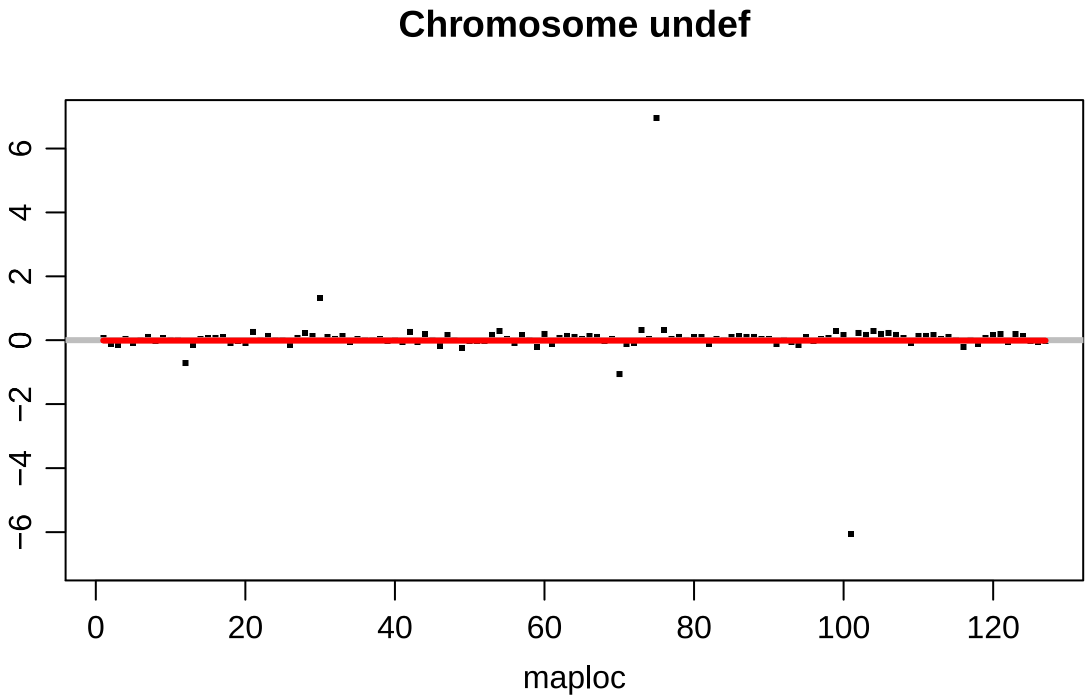


```
## Segplot might not work because of special characters in the sample names. Use only A-Z,a-z and 0-9!
##  There is a hidden function cn.mops:::.replaceNames that replaces the names in the "CNVDetectionResul
```

onXpress_010_R_2012_09_12_03_13_43_Sequoia_SN1.25.Run_19_Run_19_hg19_

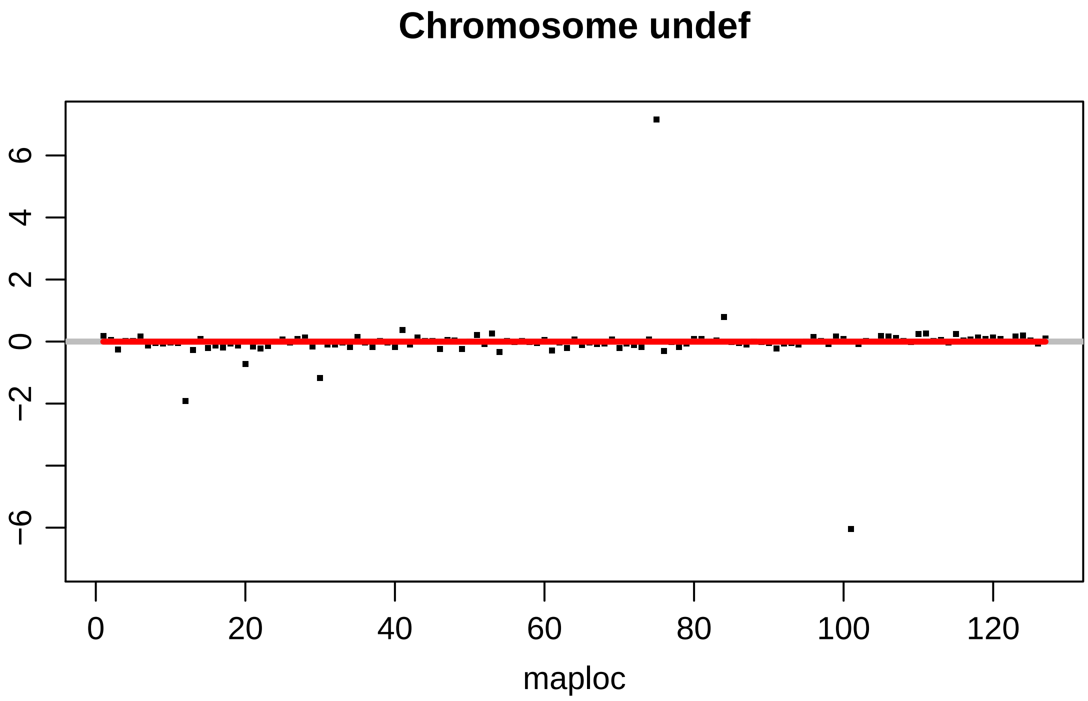


```
## Segplot might not work because of special characters in the sample names. Use only A-Z,a-z and 0-9!
##  There is a hidden function cn.mops:::.replaceNames that replaces the names in the "CNVDetectionResul
```

onXpress_011_R_2012_09_12_03_13_43_Sequoia_SN1.25.Run_19_Run_19_hg19_

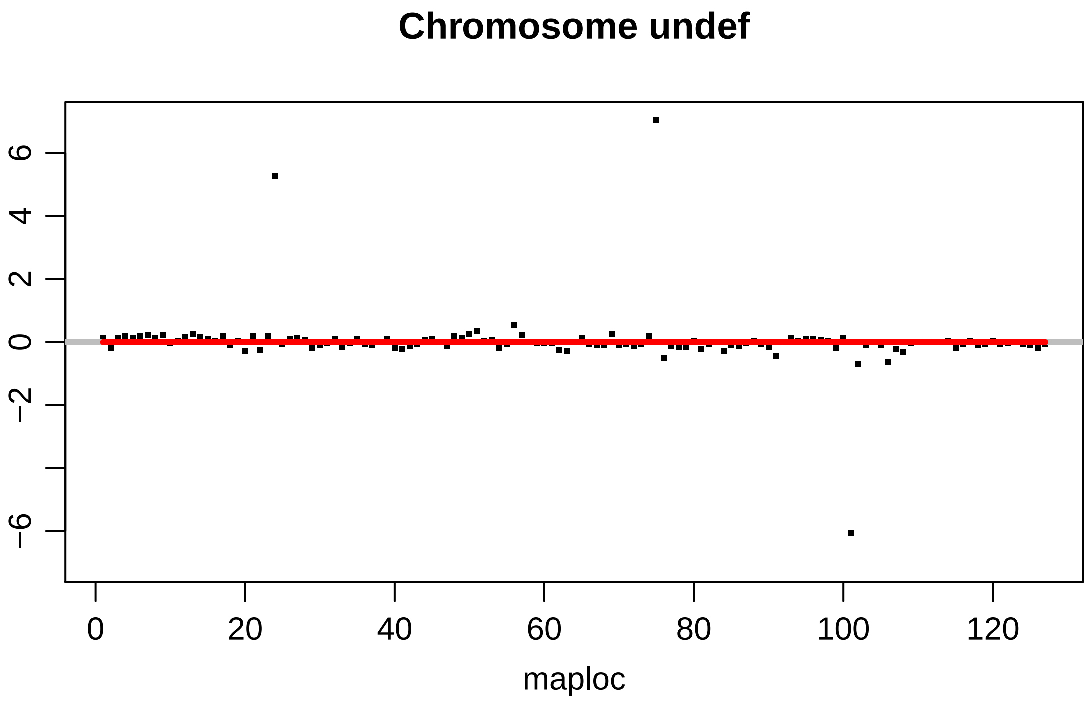

```
## Segplot might not work because of special characters in the sample names. Use only A-Z,a-z and 0-9!
##  There is a hidden function cn.mops:::.replaceNames that replaces the names in the "CNVDetectionResul
```

onXpress_012_R_2012_09_12_03_13_43_Sequoia_SN1.25.Run_19_Run_19_hg19_

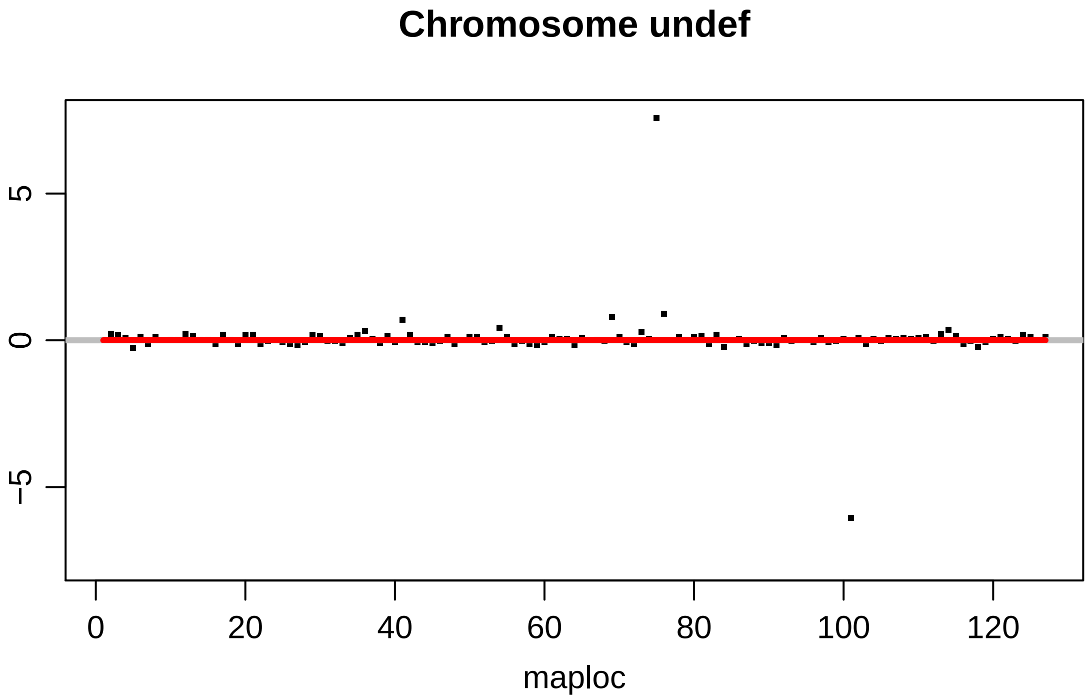


```
## Segplot might not work because of special characters in the sample names. Use only A-Z,a-z and 0-9!
##  There is a hidden function cn.mops:::.replaceNames that replaces the names in the "CNVDetectionResul
```

onXpress_013_R_2012_09_12_03_13_43_Sequoia_SN1.25.Run_19_Run_19_hg19_

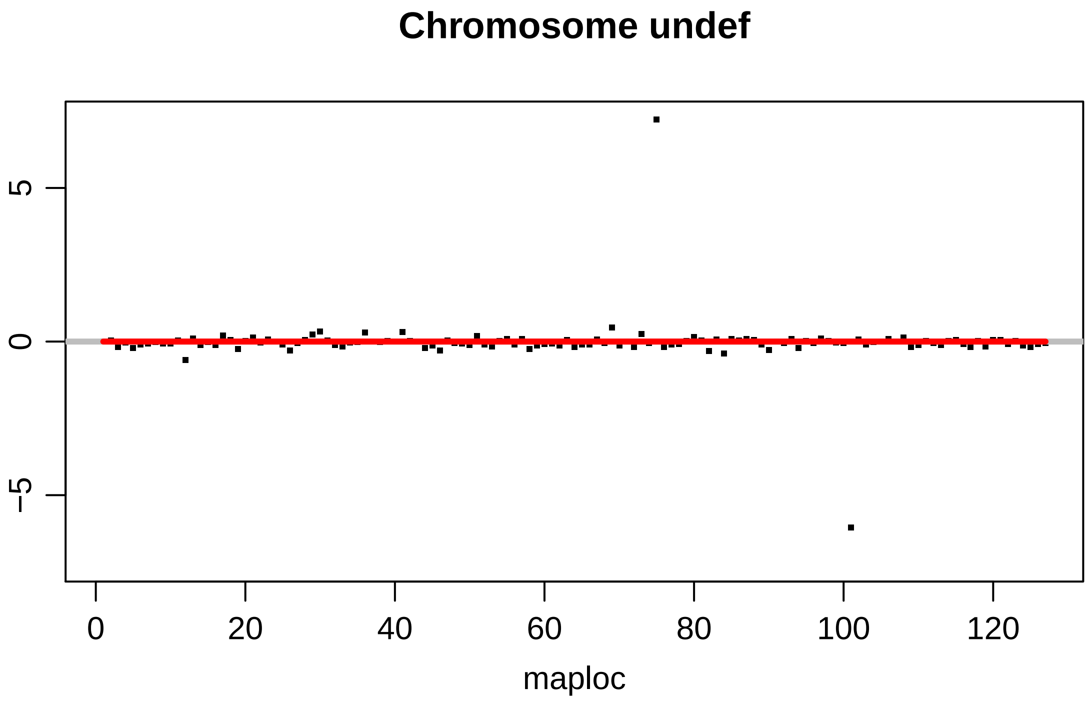


```
## Segplot might not work because of special characters in the sample names. Use only A-Z,a-z and 0-9!
##  There is a hidden function cn.mops:::.replaceNames that replaces the names in the "CNVDetectionResul
```

onXpress_014_R_2012_09_12_03_13_43_Sequoia_SN1.25.Run_19_Run_19_hg19_

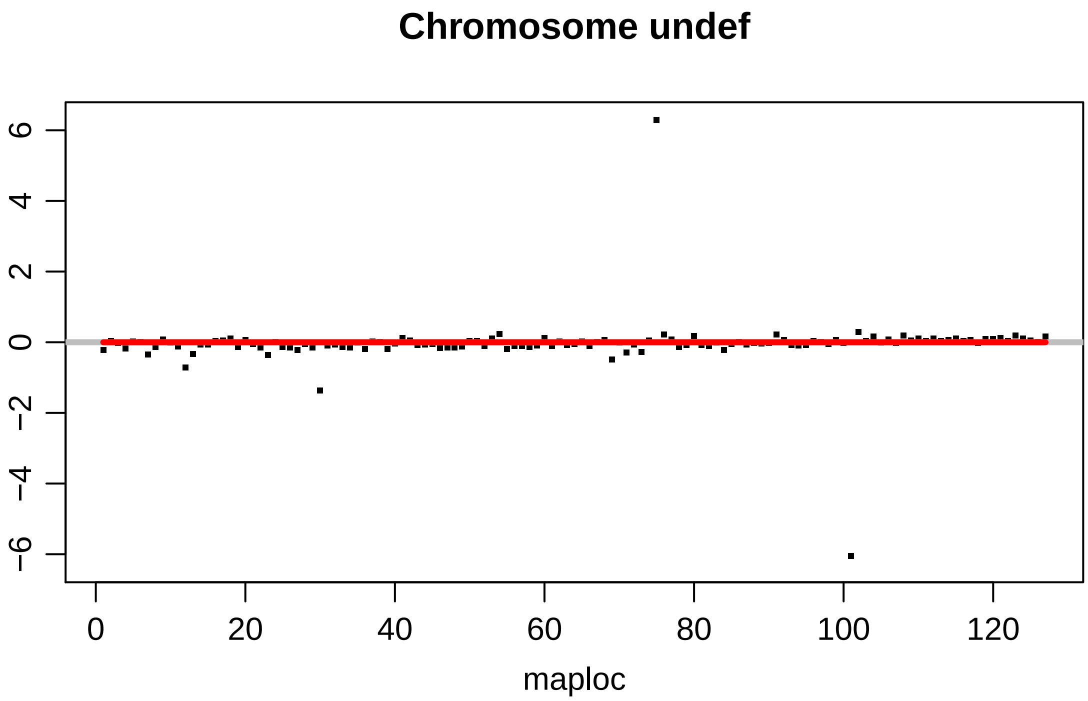

```
## Segplot might not work because of special characters in the sample names. Use only A-Z,a-z and 0-9!
##  There is a hidden function cn.mops:::.replaceNames that replaces the names in the "CNVDetectionResul
```

onXpress_015_R_2012_09_12_03_13_43_Sequoia_SN1.25.Run_19_Run_19_hg19_

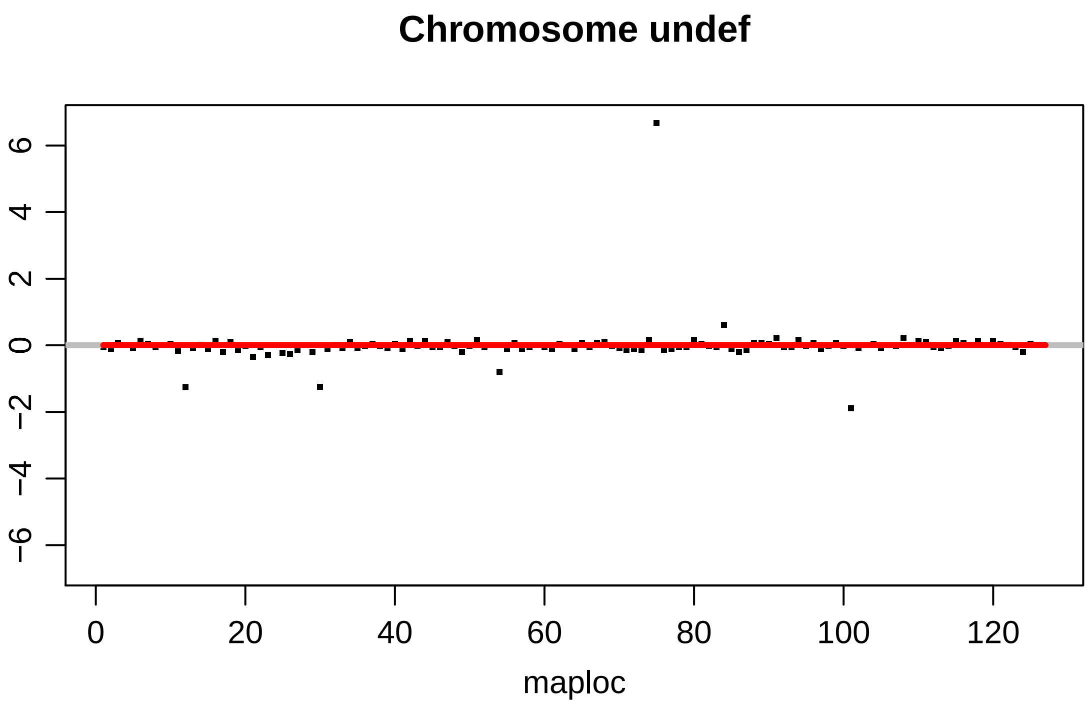


```
## Segplot might not work because of special characters in the sample names. Use only A-Z,a-z and 0-9!
##  There is a hidden function cn.mops:::.replaceNames that replaces the names in the "CNVDetectionResul
```

onXpress_016_R_2012_09_12_03_13_43_Sequoia_SN1.25.Run_19_Run_19_hg19_

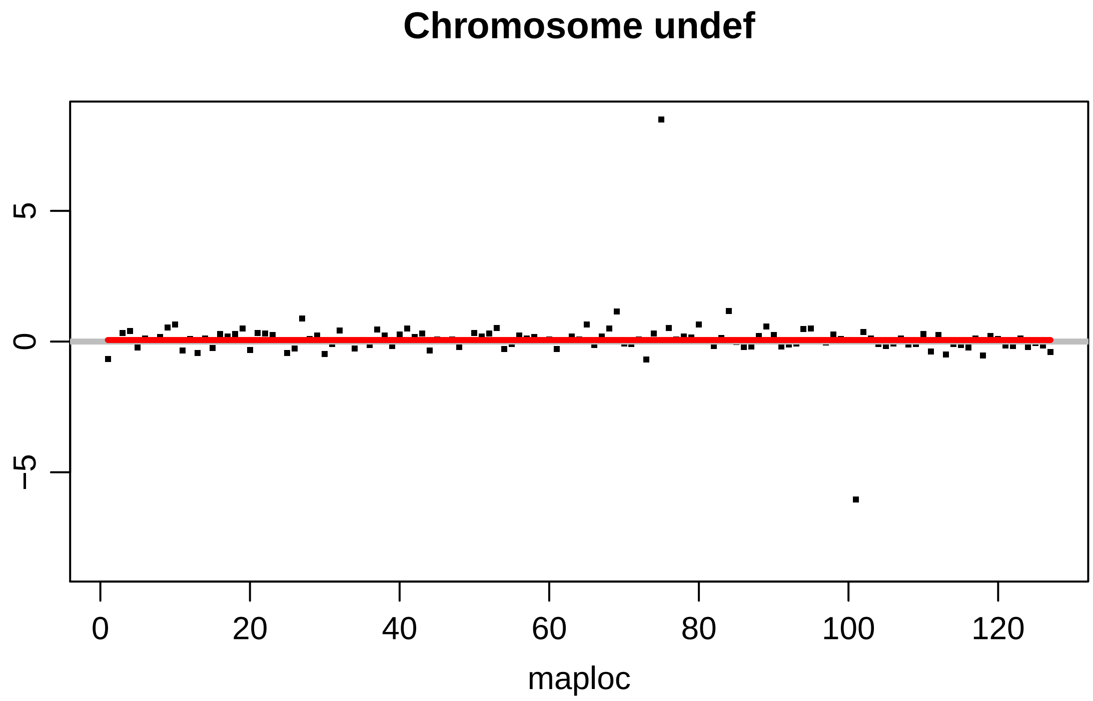


```
## Segplot might not work because of special characters in the sample names. Use only A-Z,a-z and 0-9!
##  There is a hidden function cn.mops:::.replaceNames that replaces the names in the "CNVDetectionResul
```

onXpress_017_R_2012_09_12_03_13_43_Sequoia_SN1.25.Run_19_Run_19_hg19_

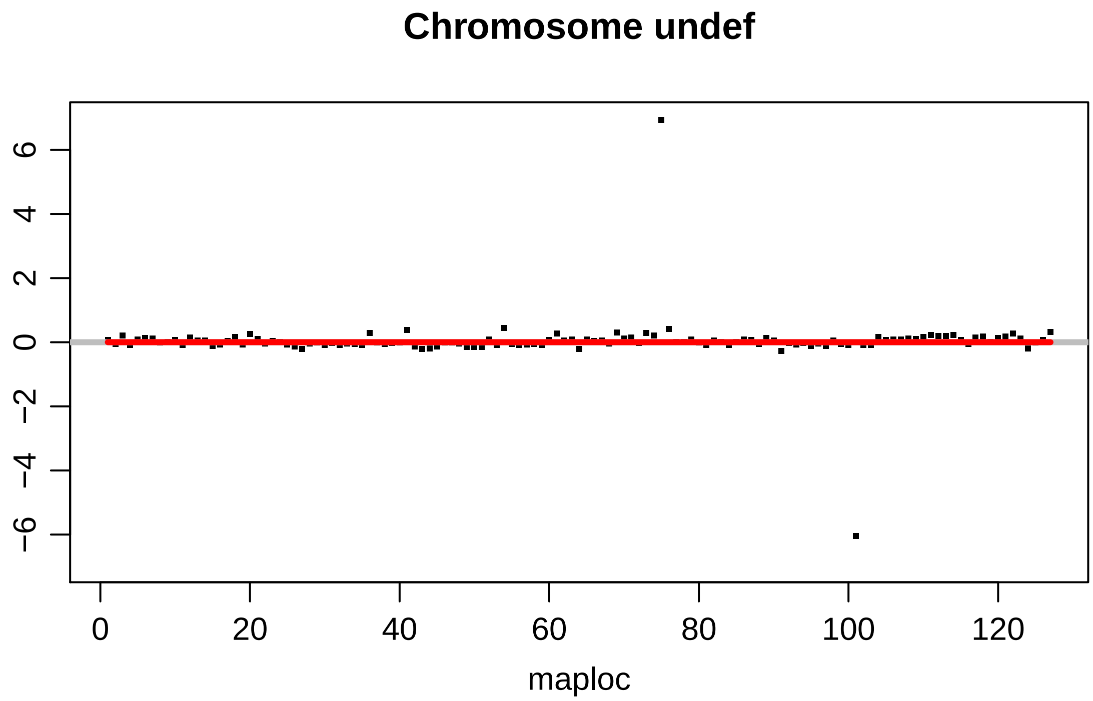

```
## Segplot might not work because of special characters in the sample names. Use only A-Z,a-z and 0-9!
##  There is a hidden function cn.mops:::.replaceNames that replaces the names in the "CNVDetectionResul
```

onXpress_018_R_2012_09_12_03_13_43_Sequoia_SN1.25.Run_19_Run_19_hg19_

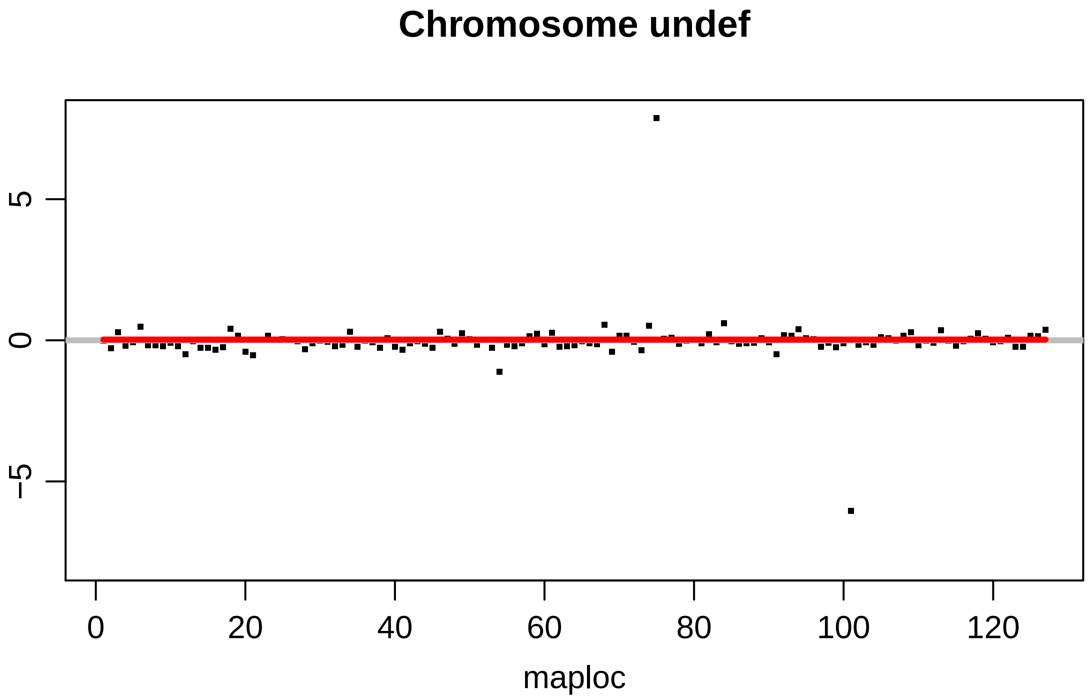


```
## Segplot might not work because of special characters in the sample names. Use only A-Z,a-z and 0-9!
##  There is a hidden function cn.mops:::.replaceNames that replaces the names in the "CNVDetectionResul
```

onXpress_019_R_2012_09_12_03_13_43_Sequoia_SN1.25.Run_19_Run_19_hg19_

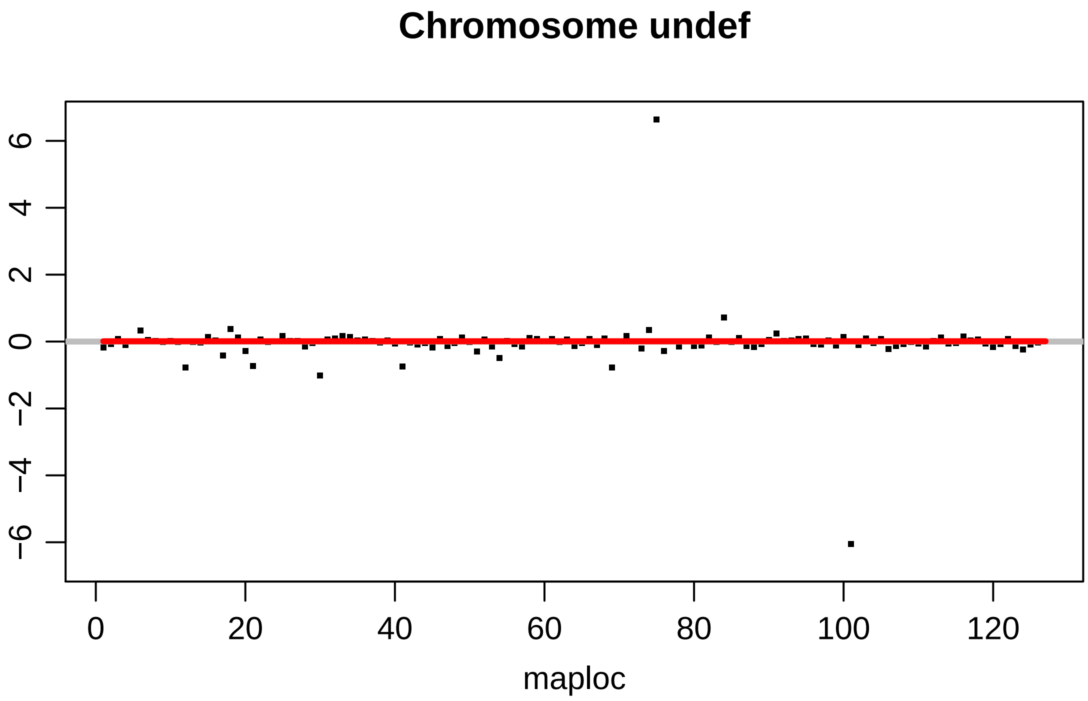


```
## Segplot might not work because of special characters in the sample names. Use only A-Z,a-z and 0-9!
##  There is a hidden function cn.mops:::.replaceNames that replaces the names in the "CNVDetectionResul
```

onXpress_020_R_2012_09_12_03_13_43_Sequoia_SN1.25.Run_19_Run_19_hg19_

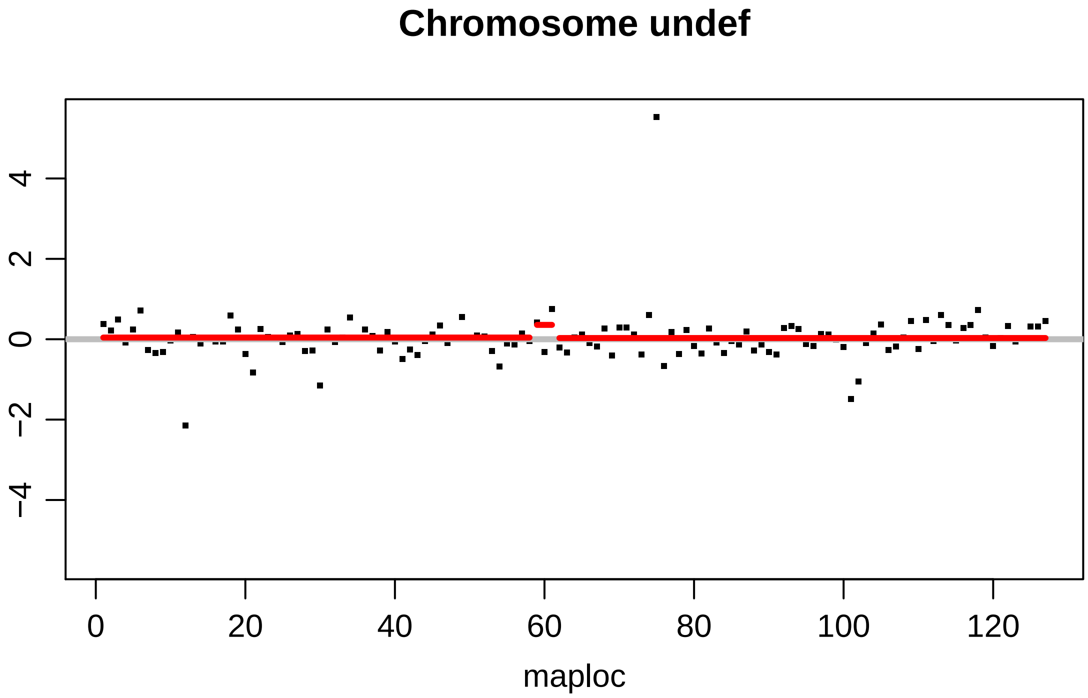

```
## Segplot might not work because of special characters in the sample names. Use only A-Z,a-z and 0-9!
##  There is a hidden function cn.mops:::.replaceNames that replaces the names in the "CNVDetectionResul
```

onXpress_021_R_2012_09_12_03_13_43_Sequoia_SN1.25.Run_19_Run_19_hg19_

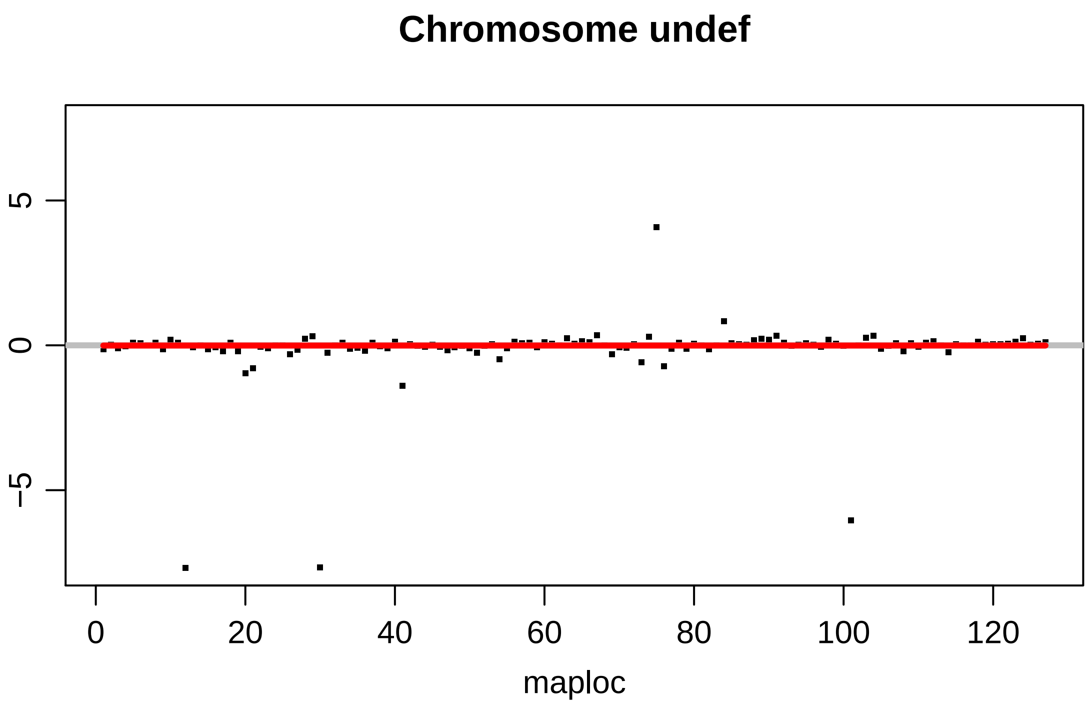


```
## Segplot might not work because of special characters in the sample names. Use only A-Z,a-z and 0-9!
##  There is a hidden function cn.mops:::.replaceNames that replaces the names in the "CNVDetectionResul
```

onXpress_022_R_2012_09_12_03_13_43_Sequoia_SN1.25.Run_19_Run_19_hg19_

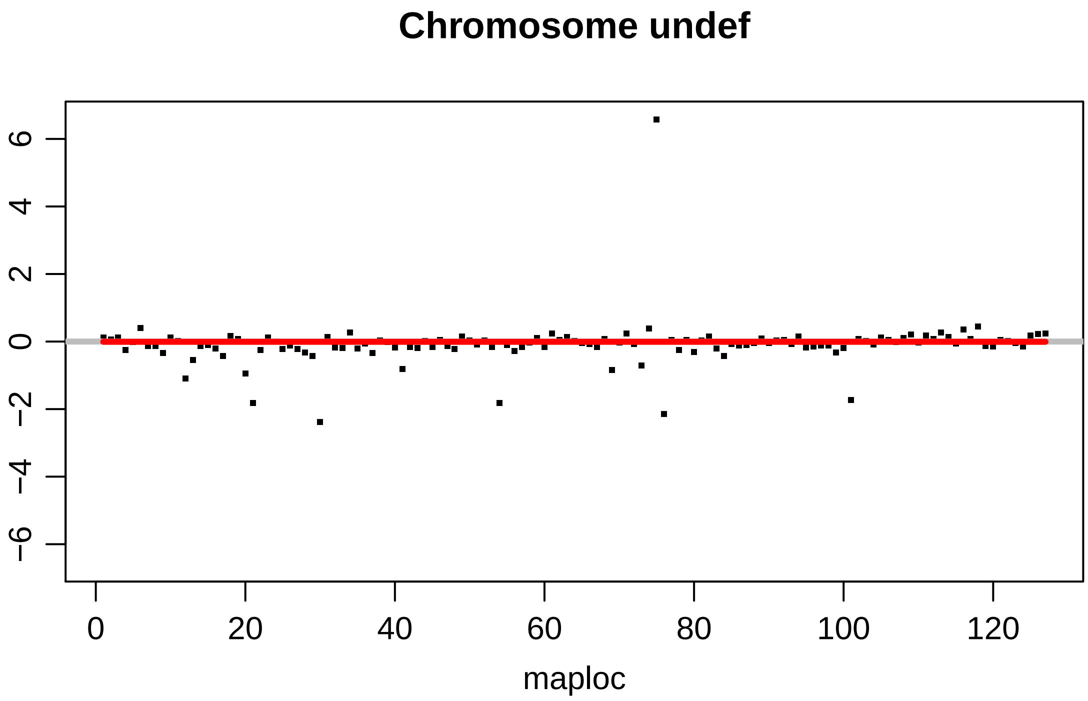


```
## Segplot might not work because of special characters in the sample names. Use only A-Z,a-z and 0-9!
##  There is a hidden function cn.mops:::.replaceNames that replaces the names in the "CNVDetectionResul
```

onXpress_023_R_2012_09_12_03_13_43_Sequoia_SN1.25.Run_19_Run_19_hg19_

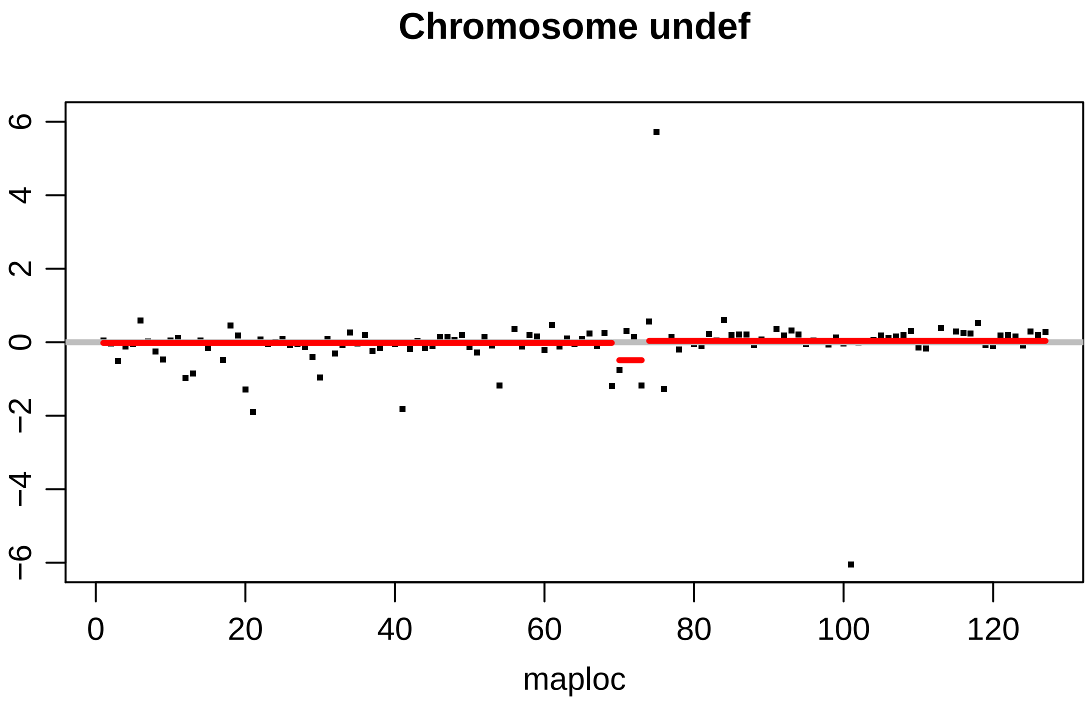

```
## Segplot might not work because of special characters in the sample names. Use only A-Z,a-z and 0-9!
##  There is a hidden function cn.mops:::.replaceNames that replaces the names in the "CNVDetectionResul
```

onXpress_024_R_2012_09_12_03_13_43_Sequoia_SN1.25.Run_19_Run_19_hg19_

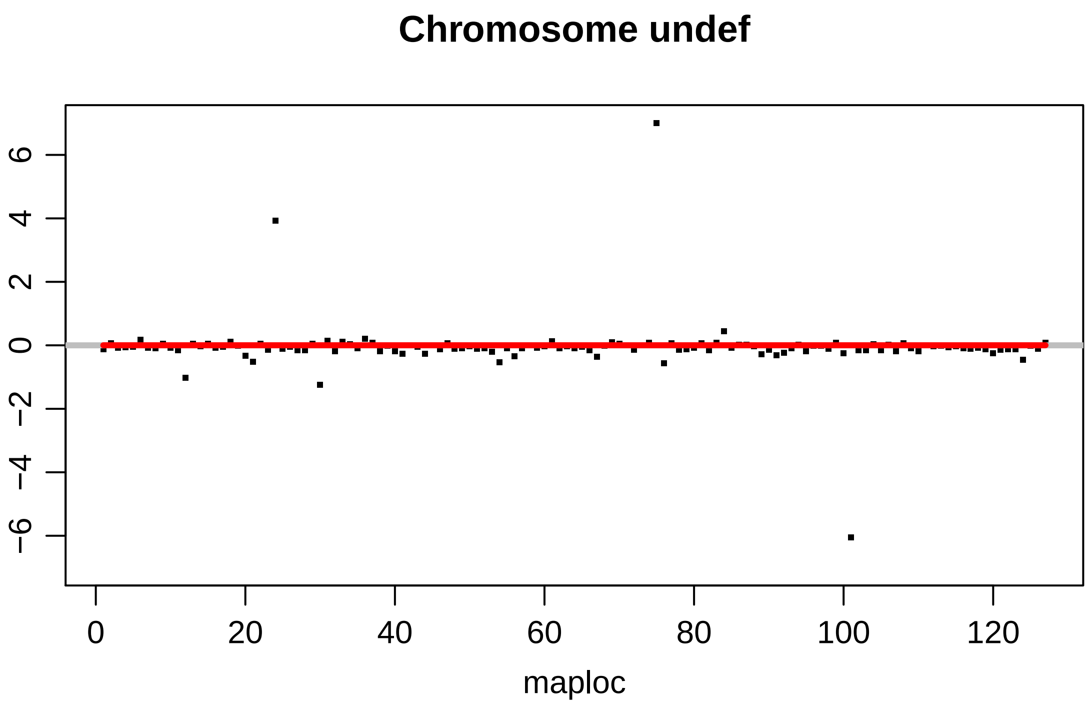


```
## Segplot might not work because of special characters in the sample names. Use only A-Z,a-z and 0-9!
##  There is a hidden function cn.mops:::.replaceNames that replaces the names in the "CNVDetectionResul
```

onXpress_025_R_2012_09_12_03_13_43_Sequoia_SN1.25.Run_19_Run_19_hg19_

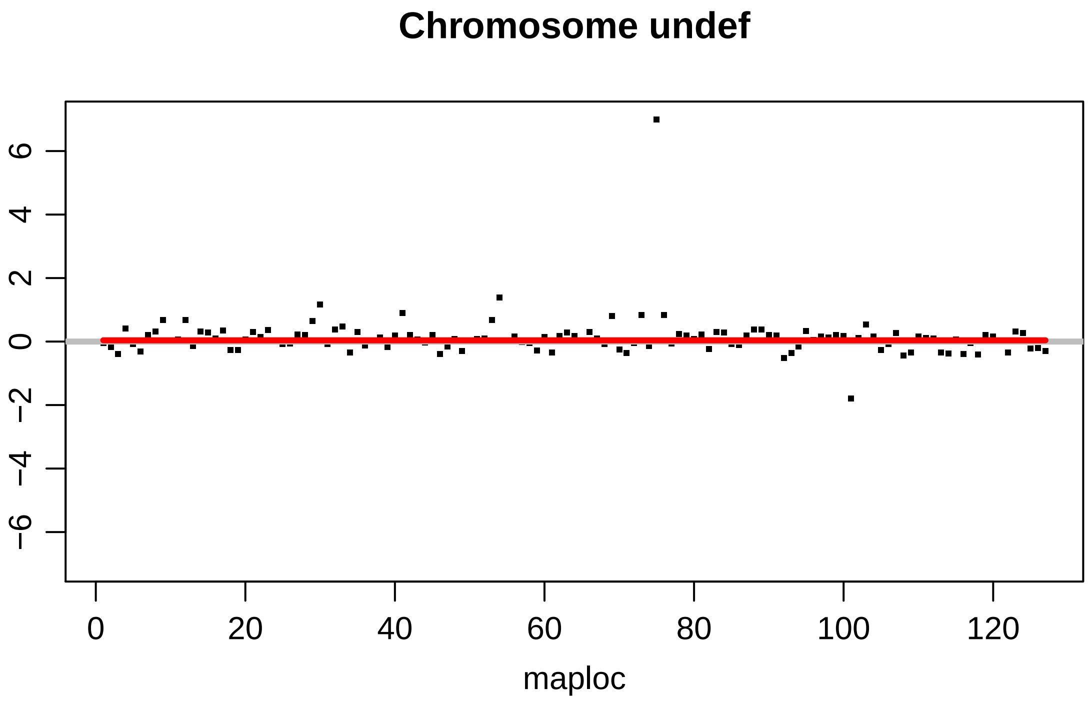


```
## Segplot might not work because of special characters in the sample names. Use only A-Z,a-z and 0-9!
##  There is a hidden function cn.mops:::.replaceNames that replaces the names in the "CNVDetectionResul
```

onXpress_026_R_2012_09_12_03_13_43_Sequoia_SN1.25.Run_19_Run_19_hg19_

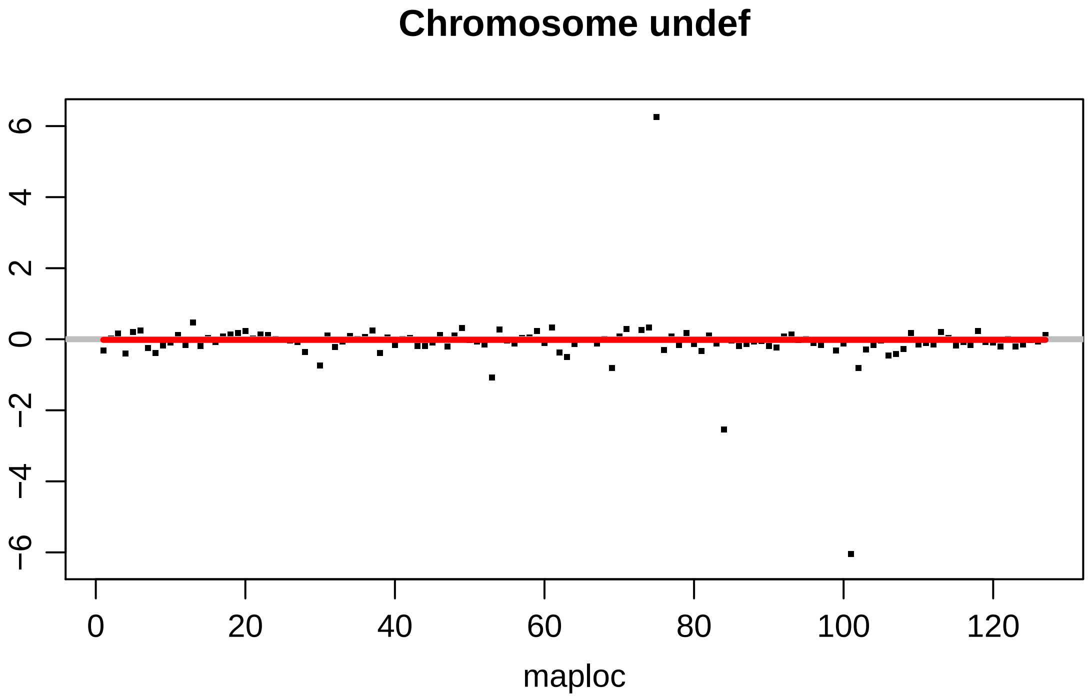

```
## Segplot might not work because of special characters in the sample names. Use only A-Z,a-z and 0-9!
##  There is a hidden function cn.mops:::.replaceNames that replaces the names in the "CNVDetectionResul
```

onXpress_027_R_2012_09_12_03_13_43_Sequoia_SN1.25.Run_19_Run_19_hg19_

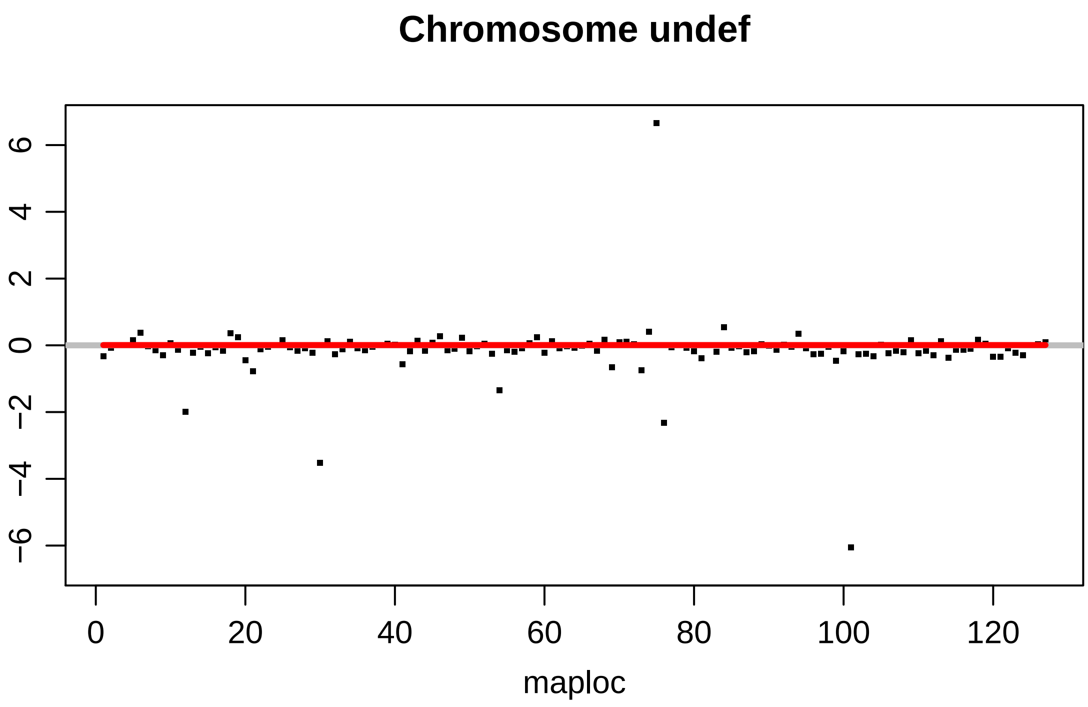


```
## Segplot might not work because of special characters in the sample names. Use only A-Z,a-z and 0-9!
##  There is a hidden function cn.mops:::.replaceNames that replaces the names in the "CNVDetectionResul
```

onXpress_028_R_2012_09_12_03_13_43_Sequoia_SN1.25.Run_19_Run_19_hg19_

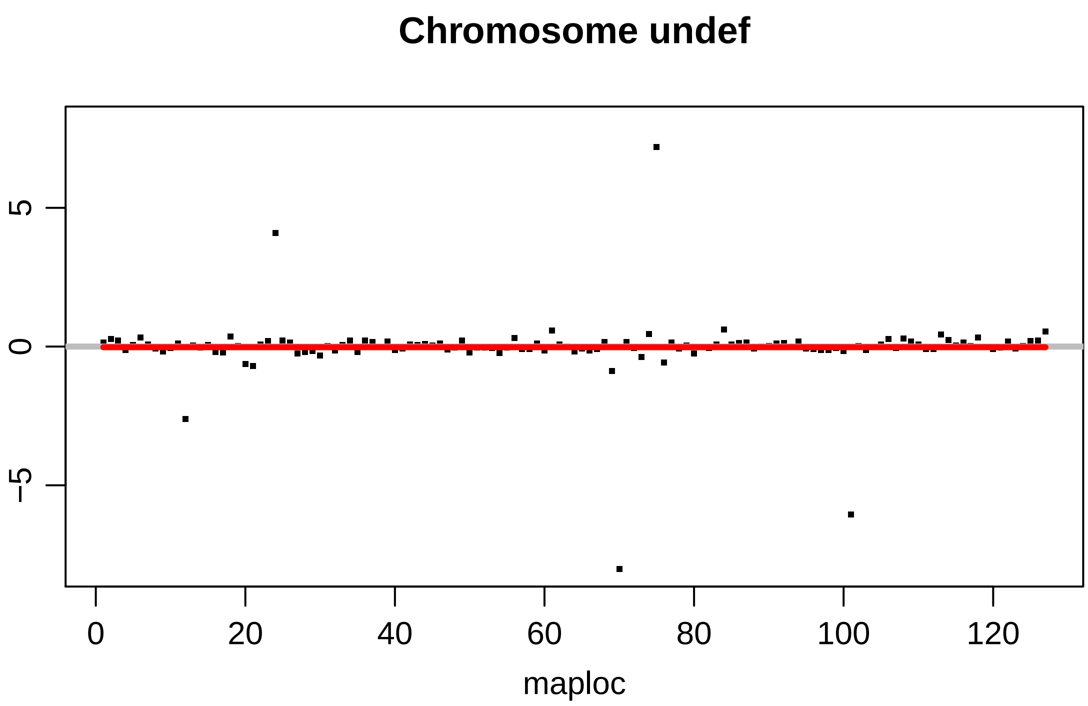


```
## Segplot might not work because of special characters in the sample names. Use only A-Z,a-z and 0-9!
##  There is a hidden function cn.mops:::.replaceNames that replaces the names in the "CNVDetectionResul
```

onXpress_029_R_2012_09_12_03_13_43_Sequoia_SN1.25.Run_19_Run_19_hg19_

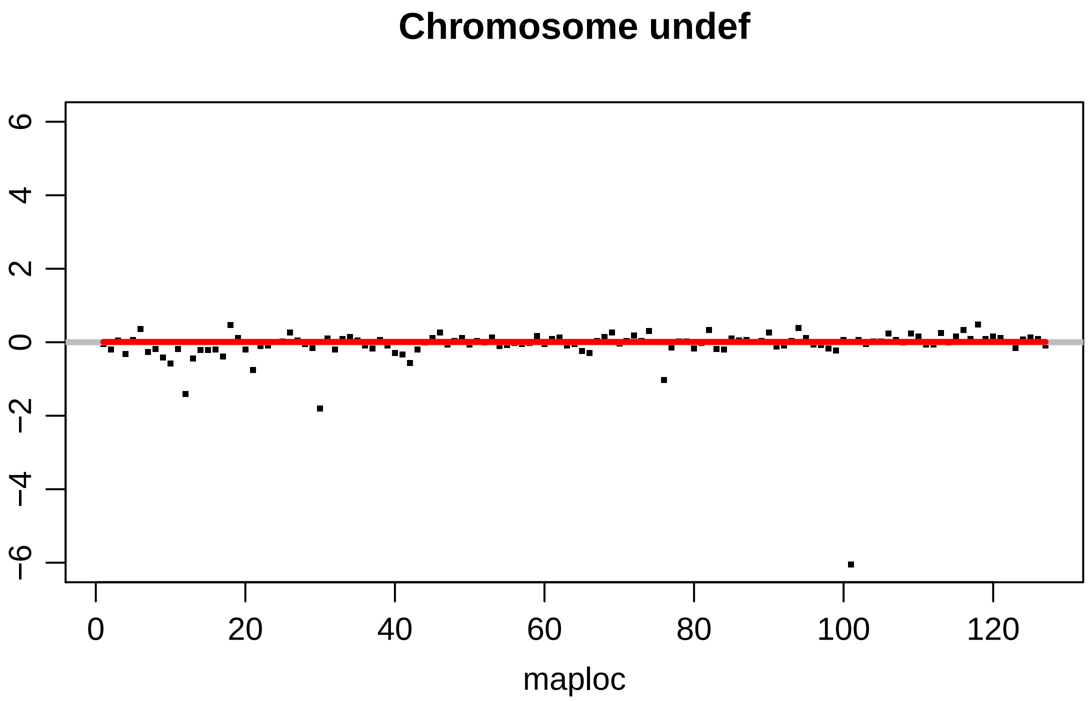

```
## Segplot might not work because of special characters in the sample names. Use only A-Z,a-z and 0-9!
##  There is a hidden function cn.mops:::.replaceNames that replaces the names in the "CNVDetectionResul
```

onXpress_030_R_2012_09_12_03_13_43_Sequoia_SN1.25.Run_19_Run_19_hg19_

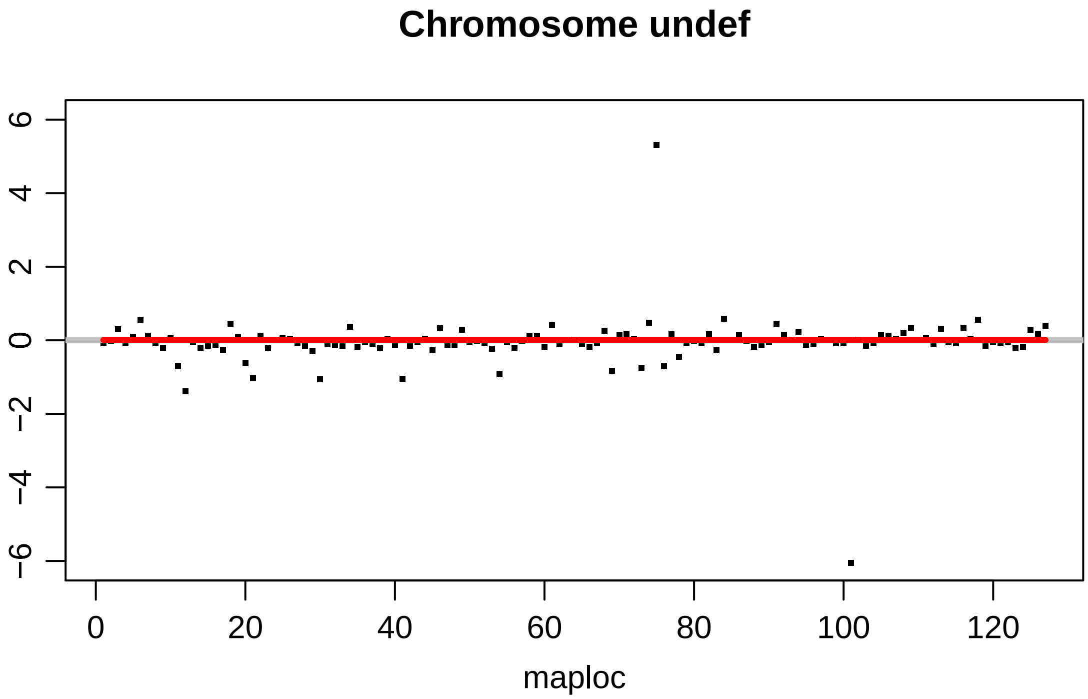


```
## Segplot might not work because of special characters in the sample names. Use only A-Z,a-z and 0-9!
##  There is a hidden function cn.mops:::.replaceNames that replaces the names in the "CNVDetectionResul
```

onXpress_031_R_2012_09_12_03_13_43_Sequoia_SN1.25.Run_19_Run_19_hg19_

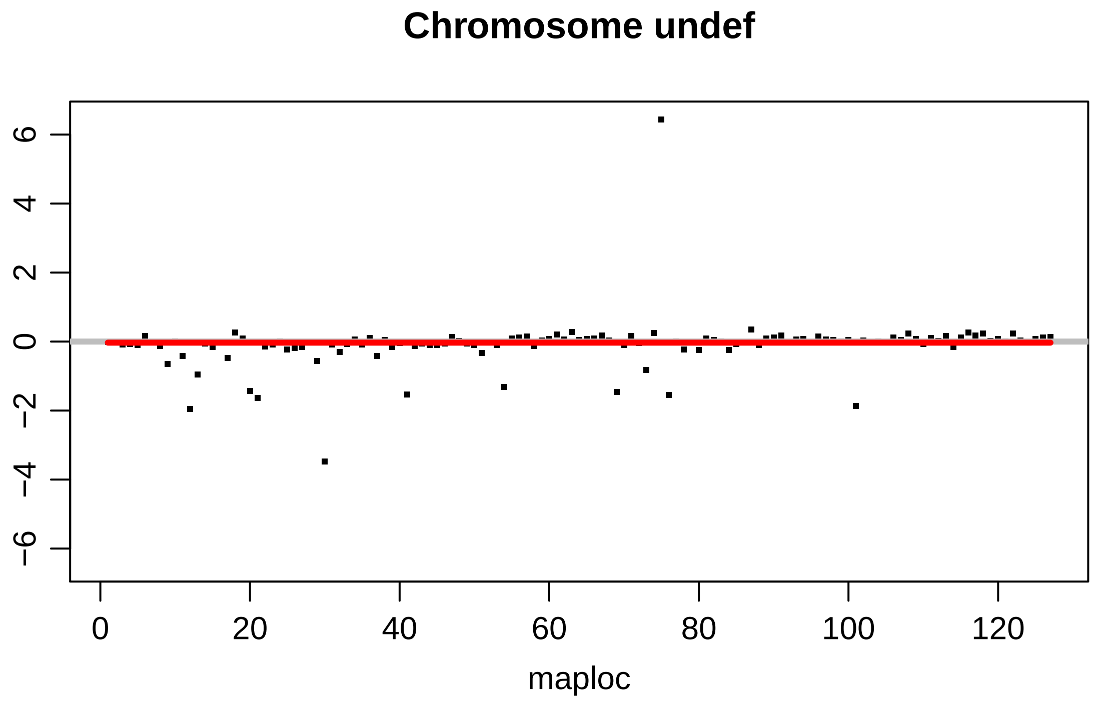


```
## Segplot might not work because of special characters in the sample names. Use only A-Z,a-z and 0-9!
##  There is a hidden function cn.mops:::.replaceNames that replaces the names in the "CNVDetectionResu
```

onXpress_032_R_2012_09_12_03_13_43_Sequoia_SN1.25.Run_19_Run_19_hg19_

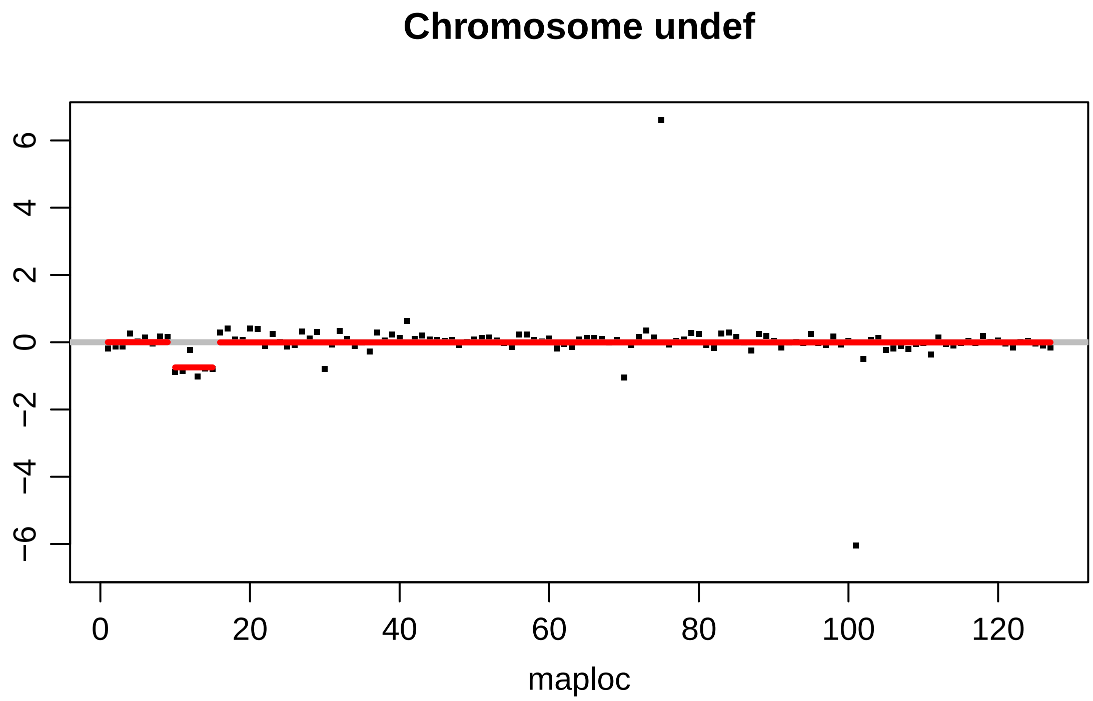

```
## Segplot might not work because of special characters in the sample names. Use only A-Z,a-z and 0-9!
##  There is a hidden function cn.mops:::.replaceNames that replaces the names in the "CNVDetectionResul
```

onXpress_033_R_2012_09_12_03_13_43_Sequoia_SN1.25.Run_19_Run_19_hg19_

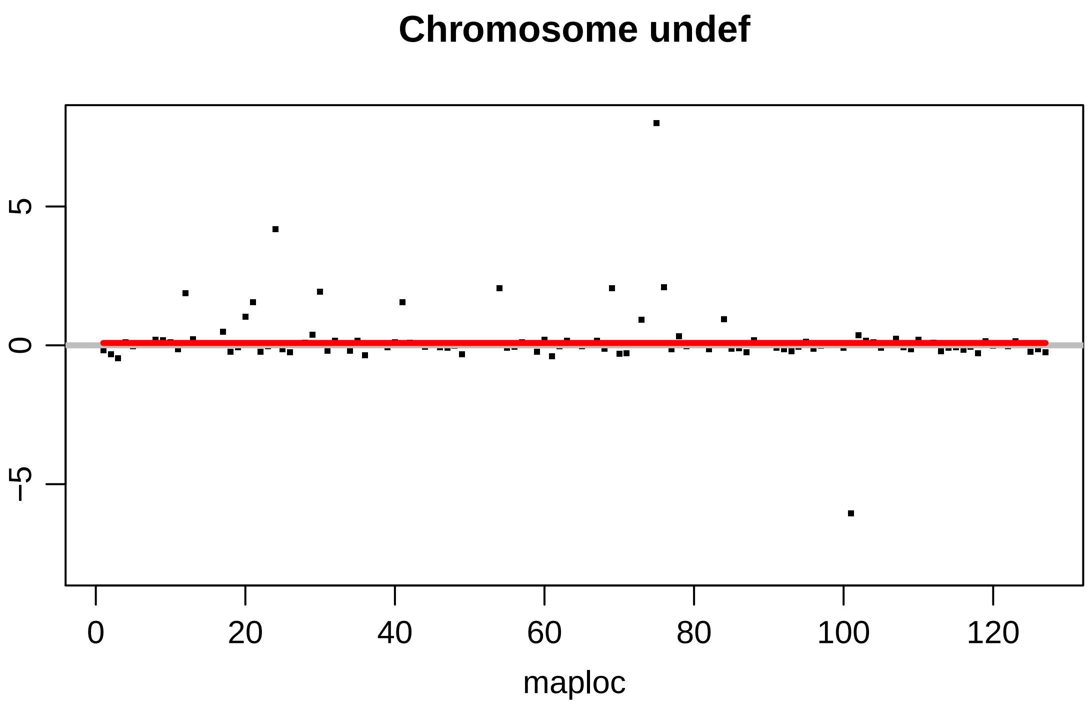


```
## Segplot might not work because of special characters in the sample names. Use only A-Z,a-z and 0-9!
##  There is a hidden function cn.mops:::.replaceNames that replaces the names in the "CNVDetectionResul
```

onXpress_034_R_2012_09_12_03_13_43_Sequoia_SN1.25.Run_19_Run_19_hg19_

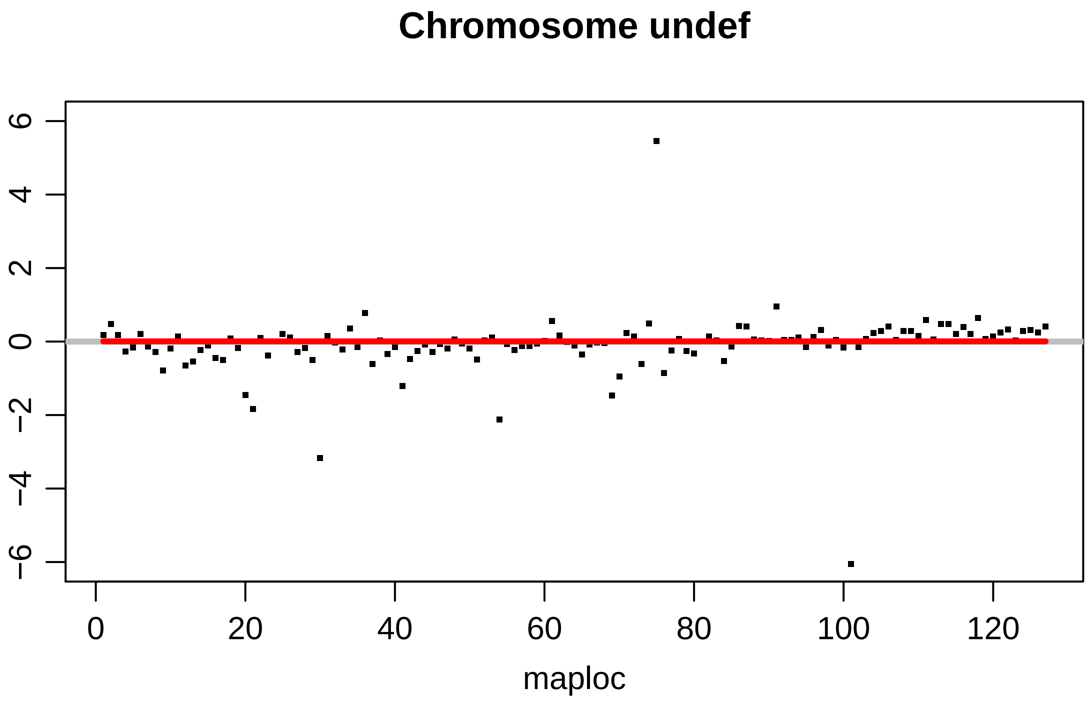


```
## Segplot might not work because of special characters in the sample names. Use only A-Z,a-z and 0-9!
##  There is a hidden function cn.mops:::.replaceNames that replaces the names in the "CNVDetectionResul
```

onXpress_035_R_2012_09_12_03_13_43_Sequoia_SN1.25.Run_19_Run_19_hg19_

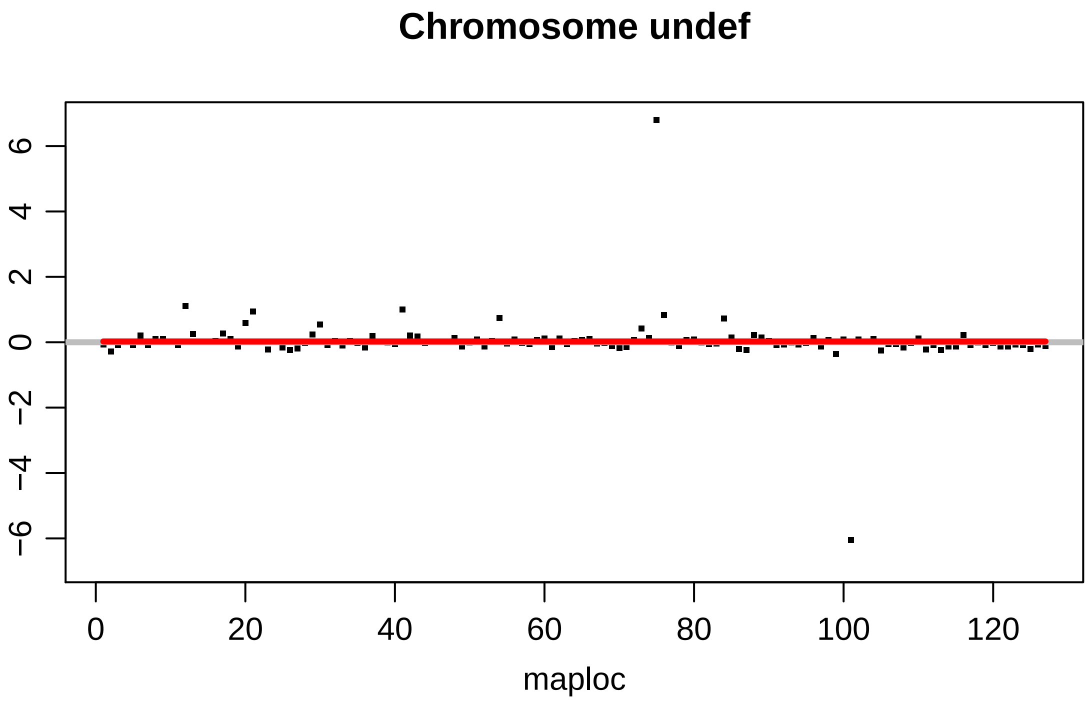

```
## Segplot might not work because of special characters in the sample names. Use only A-Z,a-z and 0-9!
##  There is a hidden function cn.mops:::.replaceNames that replaces the names in the "CNVDetectionResul
```

onXpress_036_R_2012_09_12_03_13_43_Sequoia_SN1.25.Run_19_Run_19_hg19_

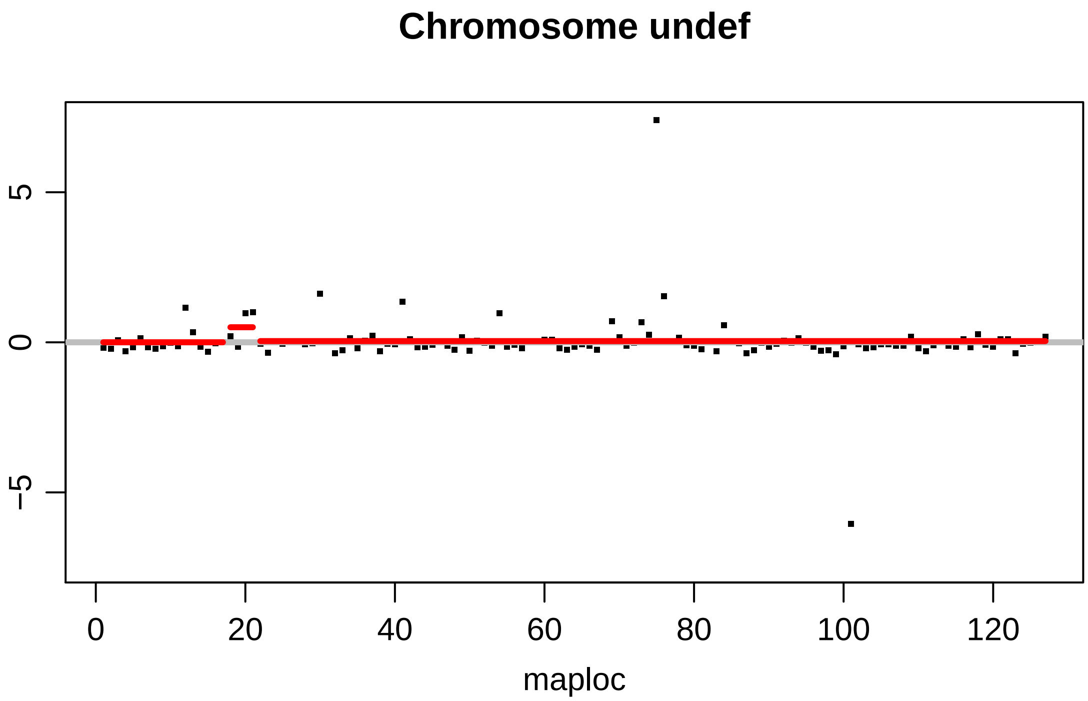


```
## Segplot might not work because of special characters in the sample names. Use only A-Z,a-z and 0-9!
##  There is a hidden function cn.mops:::.replaceNames that replaces the names in the "CNVDetectionResul
```

onXpress_037_R_2012_09_12_03_13_43_Sequoia_SN1.25.Run_19_Run_19_hg19_

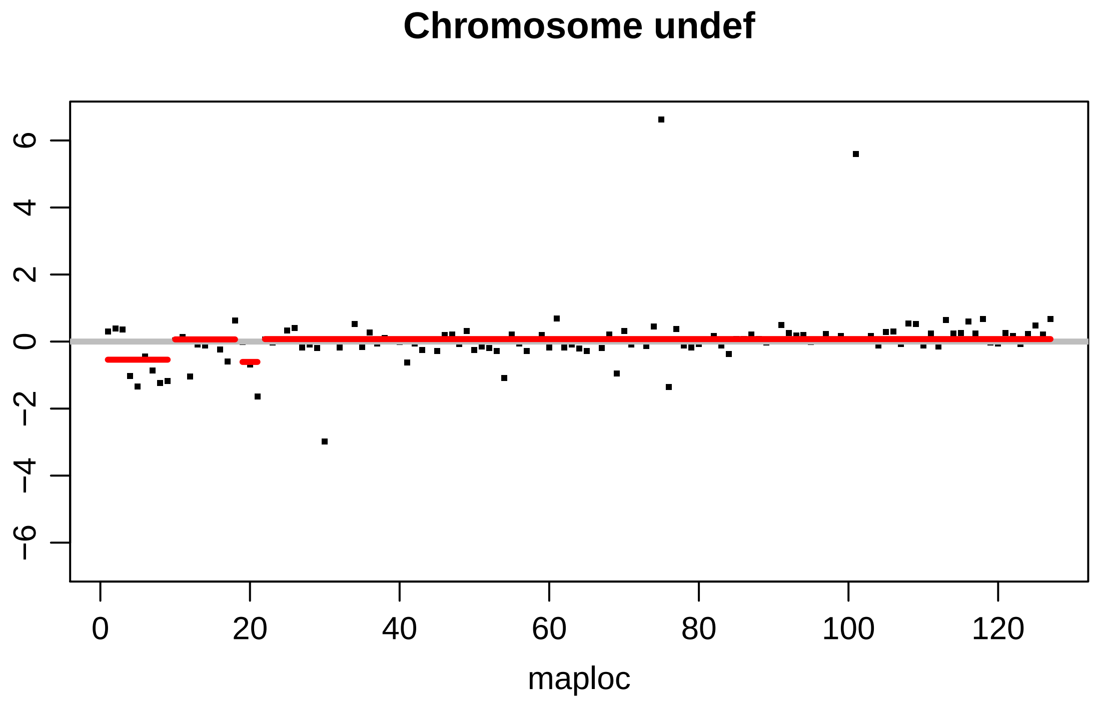


```
## Segplot might not work because of special characters in the sample names. Use only A-Z,a-z and 0-9!
##  There is a hidden function cn.mops:::.replaceNames that replaces the names in the "CNVDetectionResul
```

onXpress_038_R_2012_09_12_03_13_43_Sequoia_SN1.25.Run_19_Run_19_hg19_

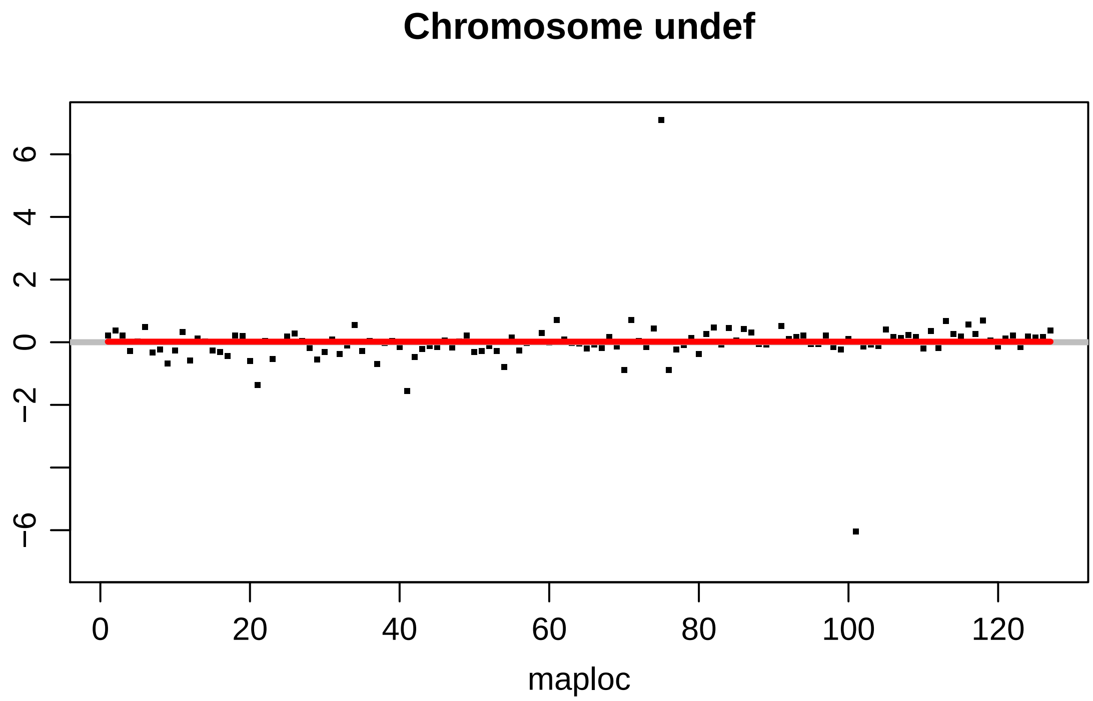

```
## Segplot might not work because of special characters in the sample names. Use only A-Z,a-z and 0-9!
##  There is a hidden function cn.mops:::.replaceNames that replaces the names in the "CNVDetectionResul
```

onXpress_039_R_2012_09_12_03_13_43_Sequoia_SN1.25.Run_19_Run_19_hg19_

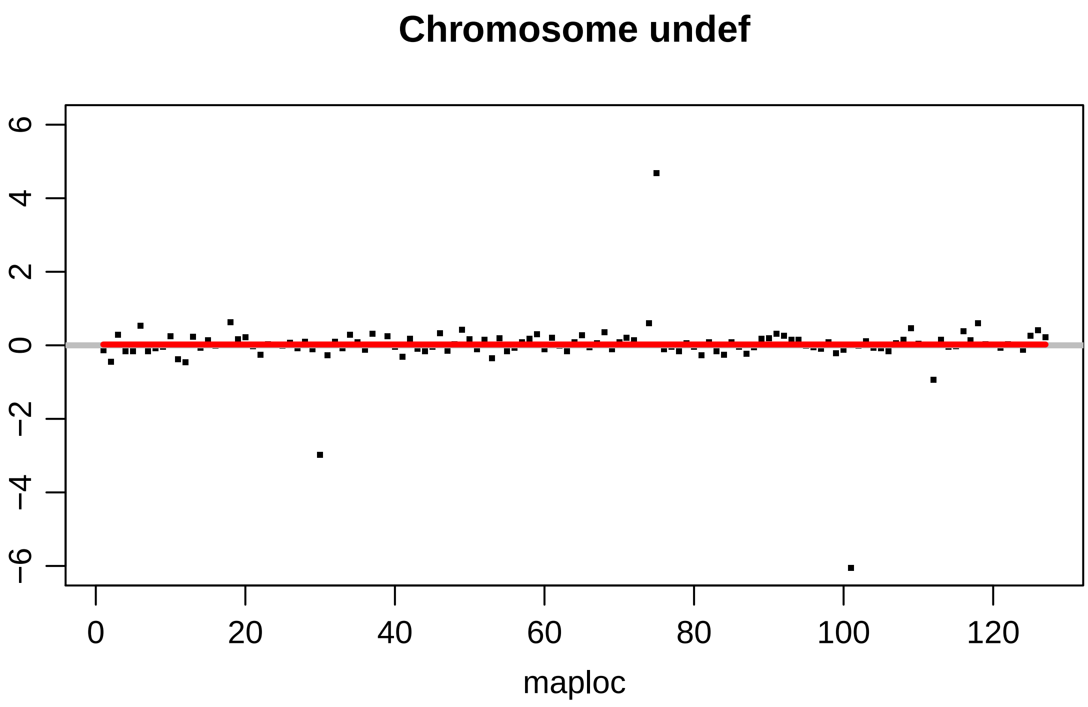


```
## Segplot might not work because of special characters in the sample names. Use only A-Z,a-z and 0-9!
##  There is a hidden function cn.mops:::.replaceNames that replaces the names in the "CNVDetectionResul
```

onXpress_040_R_2012_09_12_03_13_43_Sequoia_SN1.25.Run_19_Run_19_hg19_

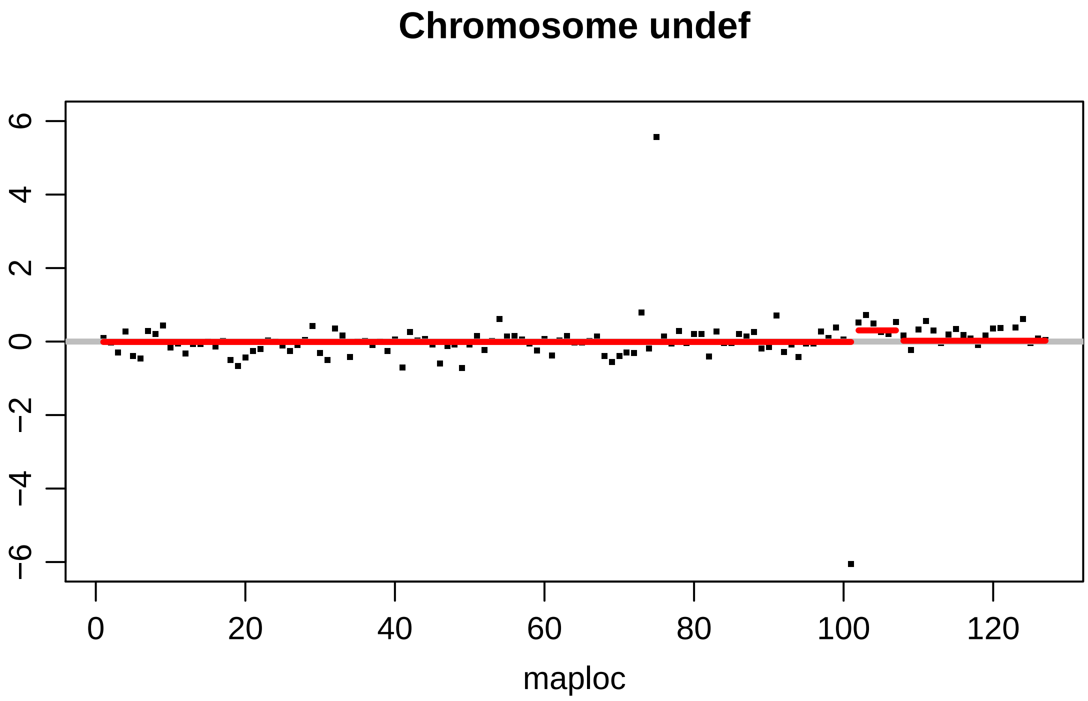


```
## Segplot might not work because of special characters in the sample names. Use only A-Z,a-z and 0-9!
##  There is a hidden function cn.mops:::.replaceNames that replaces the names in the "CNVDetectionResul
```

onXpress_041_R_2012_09_12_03_13_43_Sequoia_SN1.25.Run_19_Run_19_hg19_

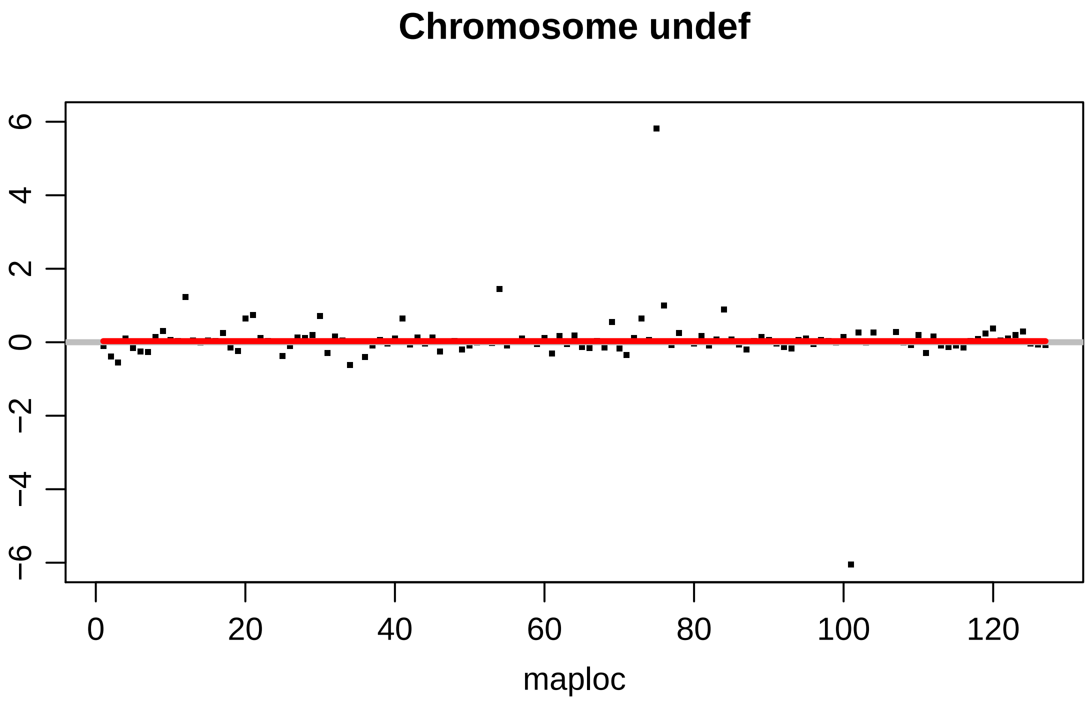

```
## Segplot might not work because of special characters in the sample names. Use only A-Z,a-z and 0-9!
##  There is a hidden function cn.mops:::.replaceNames that replaces the names in the "CNVDetectionResul
```

onXpress_042_R_2012_09_12_03_13_43_Sequoia_SN1.25.Run_19_Run_19_hg19_

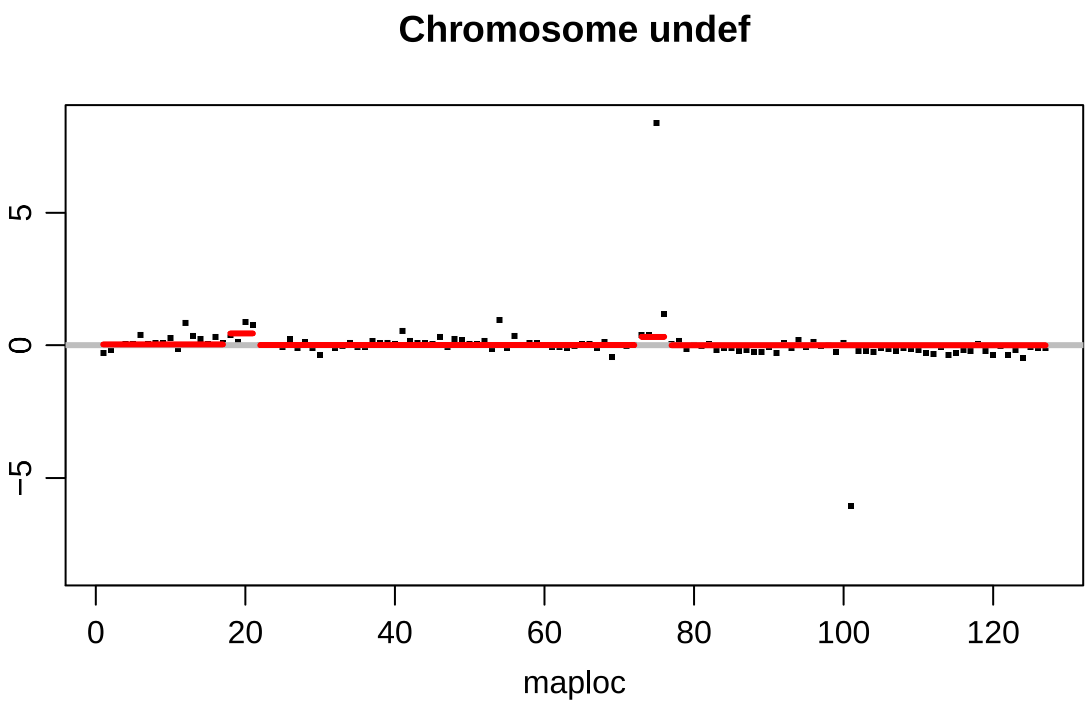


```
## Segplot might not work because of special characters in the sample names. Use only A-Z,a-z and 0-9!
##  There is a hidden function cn.mops:::.replaceNames that replaces the names in the "CNVDetectionResul
```

onXpress_043_R_2012_09_12_03_13_43_Sequoia_SN1.25.Run_19_Run_19_hg19_

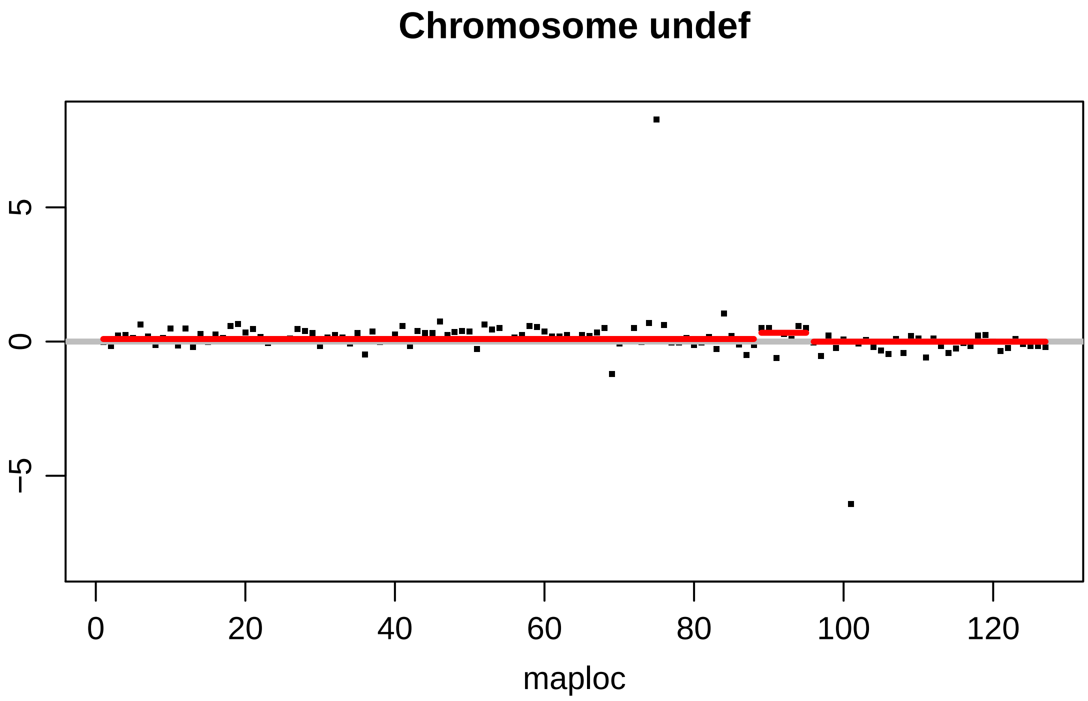


```
## Segplot might not work because of special characters in the sample names. Use only A-Z,a-z and 0-9!
##  There is a hidden function cn.mops:::.replaceNames that replaces the names in the "CNVDetectionResul
```

onXpress_044_R_2012_09_12_03_13_43_Sequoia_SN1.25.Run_19_Run_19_hg19_

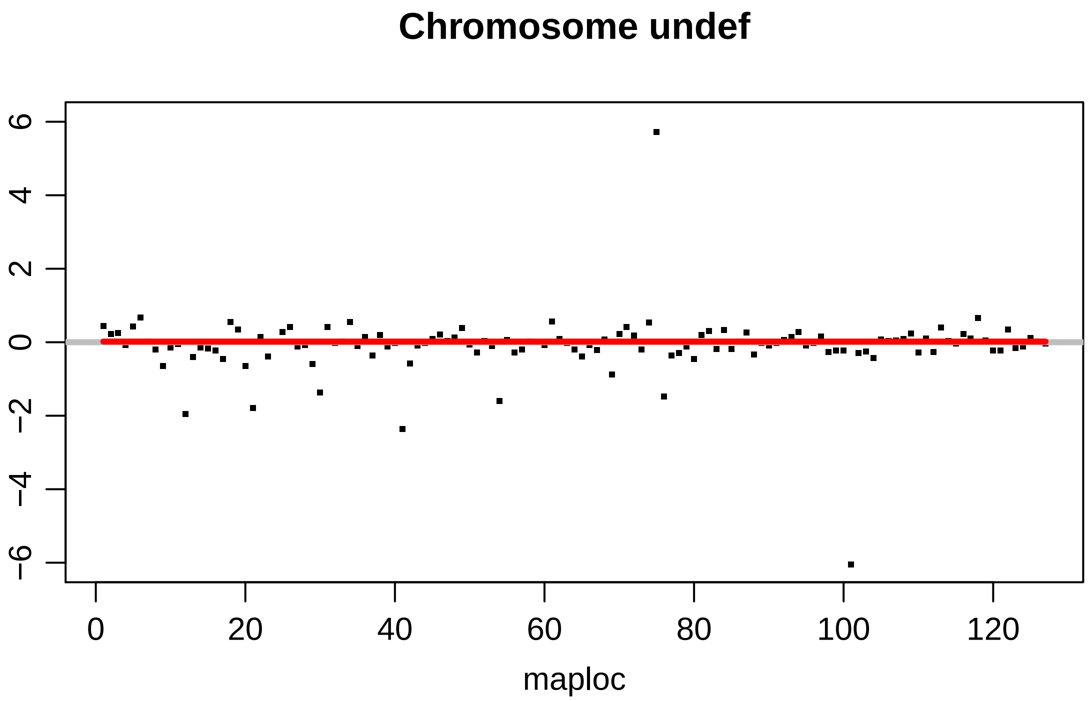

```
## Segplot might not work because of special characters in the sample names. Use only A-Z,a-z and 0-9!
##  There is a hidden function cn.mops:::.replaceNames that replaces the names in the "CNVDetectionResul
```

onXpress_045_R_2012_09_12_03_13_43_Sequoia_SN1.25.Run_19_Run_19_hg19_

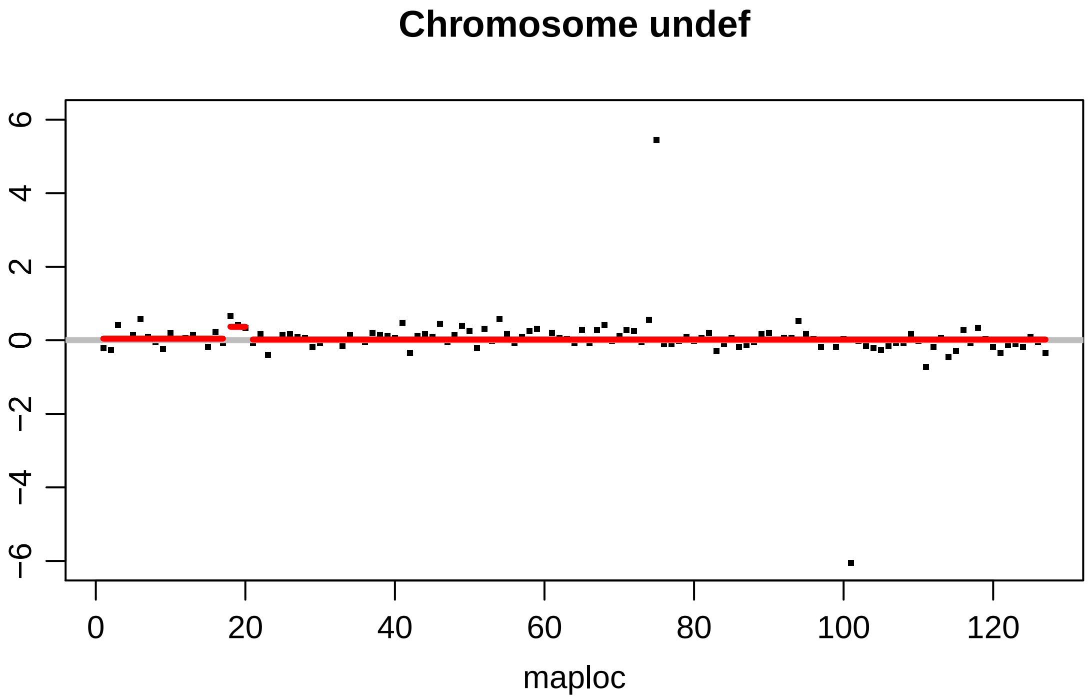


```
## Segplot might not work because of special characters in the sample names. Use only A-Z,a-z and 0-9!
##  There is a hidden function cn.mops:::.replaceNames that replaces the names in the "CNVDetectionResul
```

onXpress_046_R_2012_09_12_03_13_43_Sequoia_SN1.25.Run_19_Run_19_hg19_

Chromosome undef

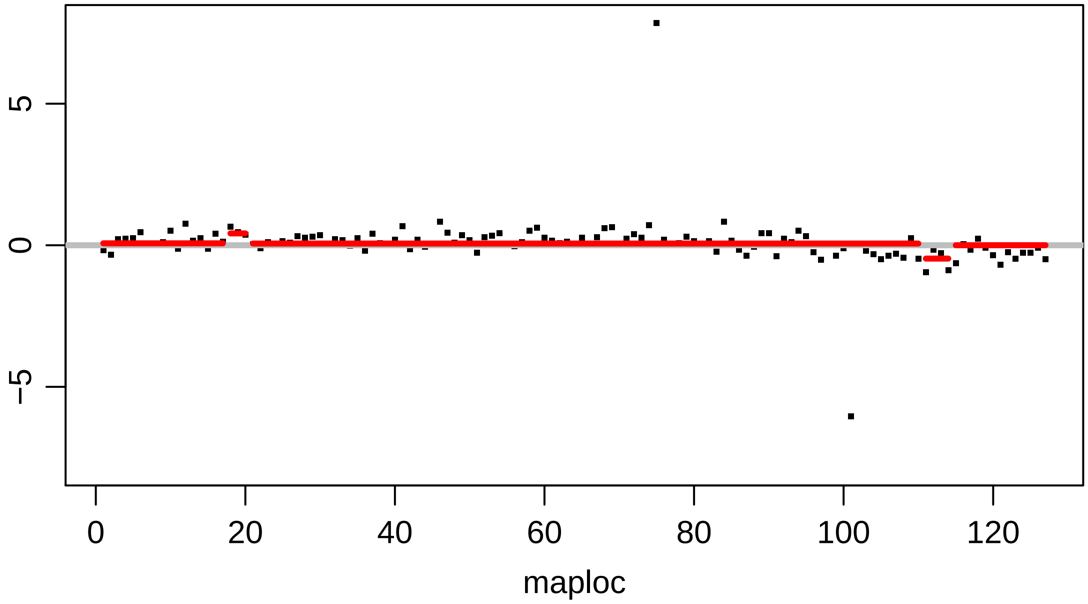


```
## Segplot might not work because of special characters in the sample names. Use only A-Z,a-z and 0-9!
##  There is a hidden function cn.mops:::.replaceNames that replaces the names in the "CNVDetectionResul
```

onXpress_047_R_2012_09_12_03_13_43_Sequoia_SN1.25.Run_19_Run_19_hg19_

Chromosome undef

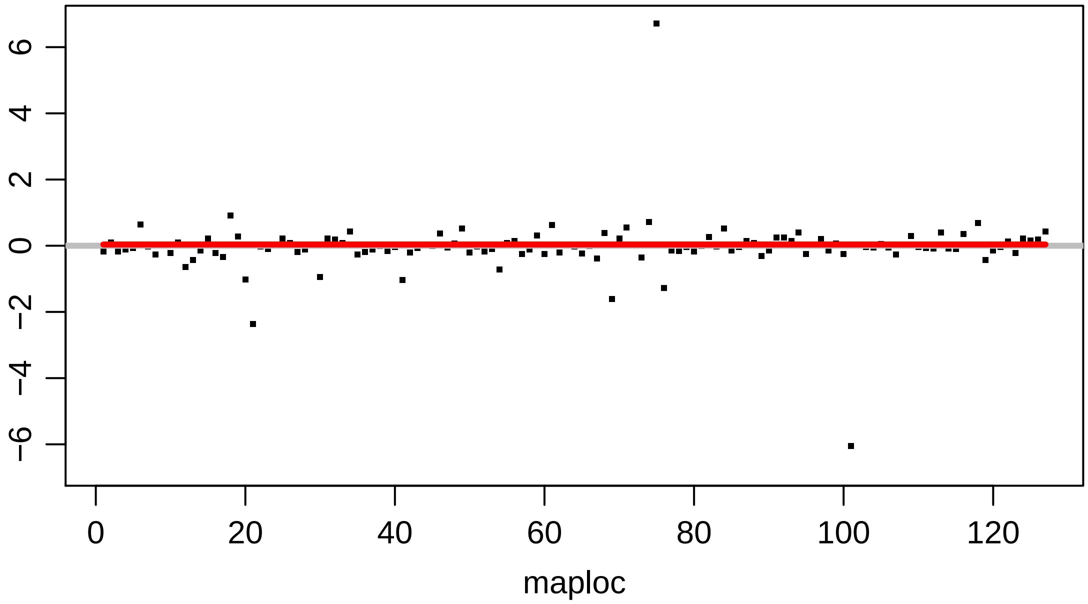

```
##
## CNV regions:
## GRanges object with 1 range and 48 metadata columns:
##       seqnames    ranges strand |
##          <Rle> <IRanges>  <Rle> |
##   [1]    undef  [18, 21]      * |
##       Case_IonXpress_001_R_2012_09_12_03_13_43_Sequoia_SN1.25.Run_19_Run_19_hg19_v3.sam
##                                                                                <factor>
##   [1]                                                                               CN2
##       Case_IonXpress_002_R_2012_09_12_03_13_43_Sequoia_SN1.25.Run_19_Run_19_hg19_v3.sam
##                                                                                <factor>
##   [1]                                                                               CN2
##       Case_IonXpress_003_R_2012_09_12_03_13_43_Sequoia_SN1.25.Run_19_Run_19_hg19_v3.sam
##                                                                                <factor>
##   [1]                                                                               CN2
##       Case_IonXpress_004_R_2012_09_12_03_13_43_Sequoia_SN1.25.Run_19_Run_19_hg19_v3.sam
##                                                                                <factor>
##   [1]                                                                               CN2
##       Case_IonXpress_005_R_2012_09_12_03_13_43_Sequoia_SN1.25.Run_19_Run_19_hg19_v3.sam
##                                                                                <factor>
##   [1]                                                                               CN2
##       Case_IonXpress_006_R_2012_09_12_03_13_43_Sequoia_SN1.25.Run_19_Run_19_hg19_v3.sam
##                                                                                <factor>
##   [1]                                                                               CN2
##       Case_IonXpress_007_R_2012_09_12_03_13_43_Sequoia_SN1.25.Run_19_Run_19_hg19_v3.sam
##                                                                                <factor>
##   [1]                                                                               CN2
##       Case_IonXpress_008_R_2012_09_12_03_13_43_Sequoia_SN1.25.Run_19_Run_19_hg19_v3.sam
##                                                                                <factor>
##   [1]                                                                               CN2
##       Case_IonXpress_009_R_2012_09_12_03_13_43_Sequoia_SN1.25.Run_19_Run_19_hg19_v3.sam
##                                                                                <factor>
##   [1]                                                                               CN2
##       Case_IonXpress_010_R_2012_09_12_03_13_43_Sequoia_SN1.25.Run_19_Run_19_hg19_v3.sam
##                                                                                <factor>
##   [1]                                                                               CN2
##       Case_IonXpress_011_R_2012_09_12_03_13_43_Sequoia_SN1.25.Run_19_Run_19_hg19_v3.sam
##                                                                                <factor>
##   [1]                                                                               CN2
##       Case_IonXpress_012_R_2012_09_12_03_13_43_Sequoia_SN1.25.Run_19_Run_19_hg19_v3.sam
##                                                                                <factor>
##   [1]                                                                               CN2
##       Case_IonXpress_013_R_2012_09_12_03_13_43_Sequoia_SN1.25.Run_19_Run_19_hg19_v3.sam
##                                                                                <factor>
##   [1]                                                                               CN2
##       Case_IonXpress_014_R_2012_09_12_03_13_43_Sequoia_SN1.25.Run_19_Run_19_hg19_v3.sam
##                                                                                <factor>
##   [1]                                                                               CN2
##       Case_IonXpress_015_R_2012_09_12_03_13_43_Sequoia_SN1.25.Run_19_Run_19_hg19_v3.sam
##                                                                                <factor>
##   [1]                                                                               CN2
##       Case_IonXpress_016_R_2012_09_12_03_13_43_Sequoia_SN1.25.Run_19_Run_19_hg19_v3.sam
##                                                                                <factor>
##   [1]                                                                               CN2
```

```
##       Case_IonXpress_017_R_2012_09_12_03_13_43_Sequoia_SN1.25.Run_19_Run_19_hg19_v3.sam
##                                                                                <factor>
##   [1]                                                                               CN2
##       Case_IonXpress_018_R_2012_09_12_03_13_43_Sequoia_SN1.25.Run_19_Run_19_hg19_v3.sam
##                                                                                <factor>
##   [1]                                                                               CN2
##       Case_IonXpress_019_R_2012_09_12_03_13_43_Sequoia_SN1.25.Run_19_Run_19_hg19_v3.sam
##                                                                                <factor>
##   [1]                                                                               CN2
##       Case_IonXpress_020_R_2012_09_12_03_13_43_Sequoia_SN1.25.Run_19_Run_19_hg19_v3.sam
##                                                                                <factor>
##   [1]                                                                               CN2
##       Case_IonXpress_021_R_2012_09_12_03_13_43_Sequoia_SN1.25.Run_19_Run_19_hg19_v3.sam
##                                                                                <factor>
##   [1]                                                                               CN2
##       Case_IonXpress_022_R_2012_09_12_03_13_43_Sequoia_SN1.25.Run_19_Run_19_hg19_v3.sam
##                                                                                <factor>
##   [1]                                                                               CN2
##       Case_IonXpress_023_R_2012_09_12_03_13_43_Sequoia_SN1.25.Run_19_Run_19_hg19_v3.sam
##                                                                                <factor>
##   [1]                                                                               CN2
##       Case_IonXpress_024_R_2012_09_12_03_13_43_Sequoia_SN1.25.Run_19_Run_19_hg19_v3.sam
##                                                                                <factor>
##   [1]                                                                               CN2
##       Case_IonXpress_025_R_2012_09_12_03_13_43_Sequoia_SN1.25.Run_19_Run_19_hg19_v3.sam
##                                                                                <factor>
##   [1]                                                                               CN2
##       Case_IonXpress_026_R_2012_09_12_03_13_43_Sequoia_SN1.25.Run_19_Run_19_hg19_v3.sam
##                                                                                <factor>
##   [1]                                                                               CN2
##       Case_IonXpress_027_R_2012_09_12_03_13_43_Sequoia_SN1.25.Run_19_Run_19_hg19_v3.sam
##                                                                                <factor>
##   [1]                                                                               CN2
##       Case_IonXpress_028_R_2012_09_12_03_13_43_Sequoia_SN1.25.Run_19_Run_19_hg19_v3.sam
##                                                                                <factor>
##   [1]                                                                               CN2
##       Case_IonXpress_029_R_2012_09_12_03_13_43_Sequoia_SN1.25.Run_19_Run_19_hg19_v3.sam
##                                                                                <factor>
##   [1]                                                                               CN2
##       Case_IonXpress_030_R_2012_09_12_03_13_43_Sequoia_SN1.25.Run_19_Run_19_hg19_v3.sam
##                                                                                <factor>
##   [1]                                                                               CN2
##       Case_IonXpress_031_R_2012_09_12_03_13_43_Sequoia_SN1.25.Run_19_Run_19_hg19_v3.sam
##                                                                                <factor>
##   [1]                                                                               CN2
##       Case_IonXpress_032_R_2012_09_12_03_13_43_Sequoia_SN1.25.Run_19_Run_19_hg19_v3.sam
##                                                                                <factor>
##   [1]                                                                               CN2
##       Case_IonXpress_033_R_2012_09_12_03_13_43_Sequoia_SN1.25.Run_19_Run_19_hg19_v3.sam
##                                                                                <factor>
##   [1]                                                                               CN2
##       Case_IonXpress_034_R_2012_09_12_03_13_43_Sequoia_SN1.25.Run_19_Run_19_hg19_v3.sam
##                                                                                <factor>
##   [1]                                                                               CN2
```

```
##       Case_IonXpress_035_R_2012_09_12_03_13_43_Sequoia_SN1.25.Run_19_Run_19_hg19_v3.sam
##                                                                                <factor>
##   [1]                                                                               CN2
##       Case_IonXpress_036_R_2012_09_12_03_13_43_Sequoia_SN1.25.Run_19_Run_19_hg19_v3.sam
##                                                                                <factor>
##   [1]                                                                               CN3
##       Case_IonXpress_037_R_2012_09_12_03_13_43_Sequoia_SN1.25.Run_19_Run_19_hg19_v3.sam
##                                                                                <factor>
##   [1]                                                                               CN2
##       Case_IonXpress_038_R_2012_09_12_03_13_43_Sequoia_SN1.25.Run_19_Run_19_hg19_v3.sam
##                                                                                <factor>
##   [1]                                                                               CN2
##       Case_IonXpress_039_R_2012_09_12_03_13_43_Sequoia_SN1.25.Run_19_Run_19_hg19_v3.sam
##                                                                                <factor>
##   [1]                                                                               CN2
##       Case_IonXpress_040_R_2012_09_12_03_13_43_Sequoia_SN1.25.Run_19_Run_19_hg19_v3.sam
##                                                                                <factor>
##   [1]                                                                               CN2
##       Case_IonXpress_041_R_2012_09_12_03_13_43_Sequoia_SN1.25.Run_19_Run_19_hg19_v3.sam
##                                                                                <factor>
##   [1]                                                                               CN2
##       Case_IonXpress_042_R_2012_09_12_03_13_43_Sequoia_SN1.25.Run_19_Run_19_hg19_v3.sam
##                                                                                <factor>
##   [1]                                                                               CN2
##       Case_IonXpress_043_R_2012_09_12_03_13_43_Sequoia_SN1.25.Run_19_Run_19_hg19_v3.sam
##                                                                                <factor>
##   [1]                                                                               CN2
##       Case_IonXpress_044_R_2012_09_12_03_13_43_Sequoia_SN1.25.Run_19_Run_19_hg19_v3.sam
##                                                                                <factor>
##   [1]                                                                               CN2
##       Case_IonXpress_045_R_2012_09_12_03_13_43_Sequoia_SN1.25.Run_19_Run_19_hg19_v3.sam
##                                                                                <factor>
##   [1]                                                                               CN2
##       Case_IonXpress_046_R_2012_09_12_03_13_43_Sequoia_SN1.25.Run_19_Run_19_hg19_v3.sam
##                                                                                <factor>
##   [1]                                                                               CN2
##       Case_IonXpress_047_R_2012_09_12_03_13_43_Sequoia_SN1.25.Run_19_Run_19_hg19_v3.sam
##                                                                                <factor>
##   [1]                                                                               CN2
##       Case_IonXpress_048_R_2012_09_12_03_13_43_Sequoia_SN1.25.Run_19_Run_19_hg19_v3.sam
##                                                                                <factor>
##   [1]                                                                               CN4
##   -------
##   seqinfo: 1 sequence from an unspecified genome; no seqlengths
##
## Individual CNVs:
## GRanges object with 2 ranges and 4 metadata columns:
##       seqnames    ranges strand |
##          <Rle> <IRanges>  <Rle> |
##   [1]    undef  [18, 21]      * |
##   [2]    undef  [18, 21]      * |
##                                                                              sampleName
##                                                                                <factor>
##   [1] Case_IonXpress_036_R_2012_09_12_03_13_43_Sequoia_SN1.25.Run_19_Run_19_hg19_v3.sam
```

```
##   [2] Case_IonXpress_048_R_2012_09_12_03_13_43_Sequoia_SN1.25.Run_19_Run_19_hg19_v3.sam
##          median      mean          CN
##       <numeric> <numeric> <character>
##   [1] 0.4873263 0.5019180         CN3
##   [2] 1.0675175 0.9517635         CN4
##   -------
##   seqinfo: 1 sequence from an unspecified genome; no seqlengths
## [1] "/Users/gdemidov/Downloads/doc/Run_20_fin_05_qc.xls"

## Normalizing...

## Starting local modeling, please be patient...

## Reference sequence:  undef

## Starting segmentation algorithm...

## Using "fastseg" for segmentation.

## No CNVs detected. Try changing "normalization", "priorImpact" or "thresholds".

## [1] ""
## [1] "/Users/gdemidov/Downloads/doc/Run_20_fin_05_qc.xls"
## [1] ""

## Segplot might not work because of special characters in the sample names. Use only A-Z,a-z and 0-9!
##  There is a hidden function cn.mops:::.replaceNames that replaces the names in the "CNVDetectionResul
```

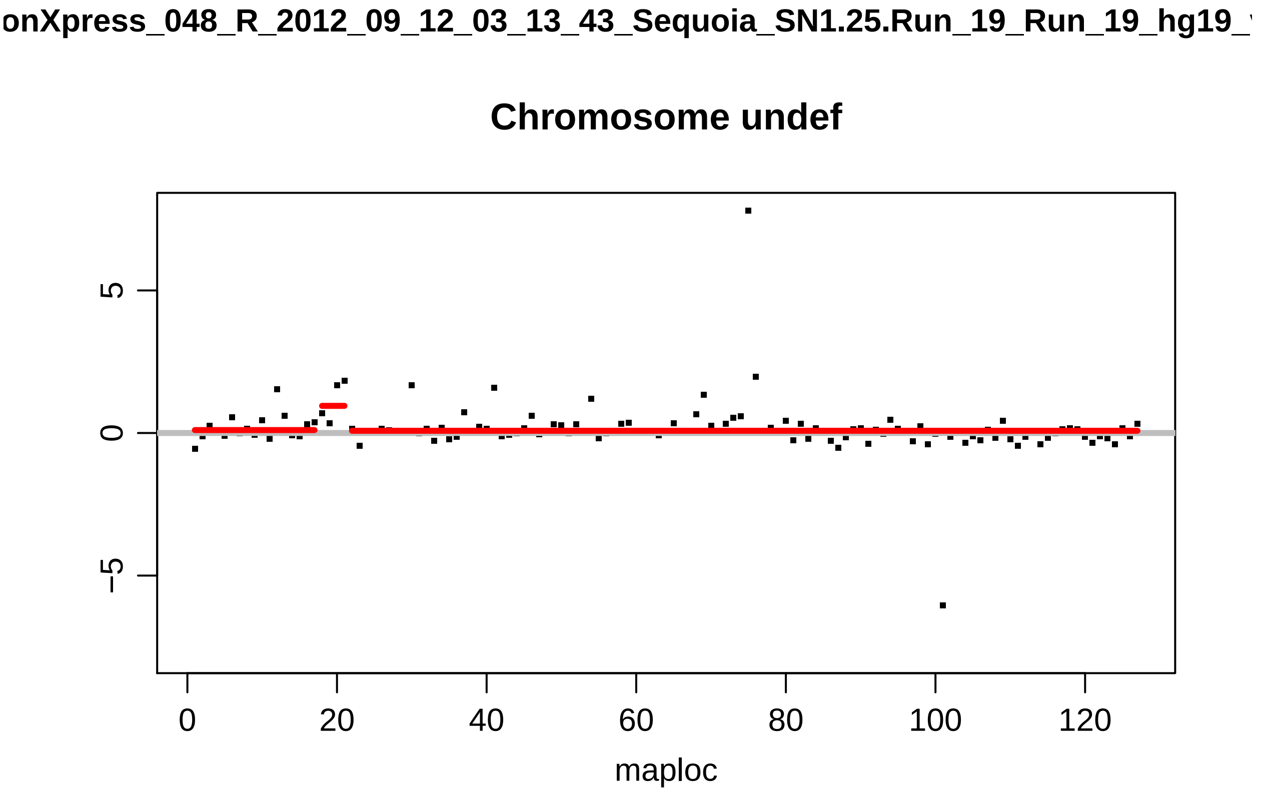

```
## Segplot might not work because of special characters in the sample names. Use only A-Z,a-z and 0-9!
##  There is a hidden function cn.mops:::.replaceNames that replaces the names in the "CNVDetectionResul
```

onXpress_002_R_2012_09_12_07_50_54_Sequoia_SN1.26.Run_20_Run_20_hg19_

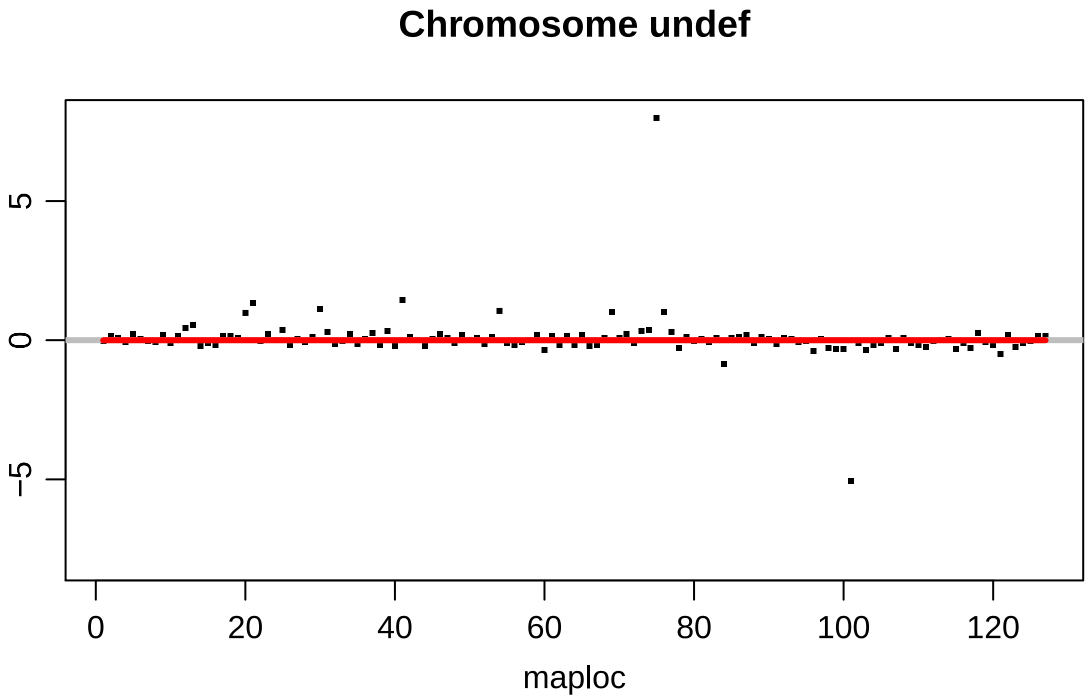


```
## Segplot might not work because of special characters in the sample names. Use only A-Z,a-z and 0-9!
##  There is a hidden function cn.mops:::.replaceNames that replaces the names in the "CNVDetectionResul
```

onXpress_003_R_2012_09_12_07_50_54_Sequoia_SN1.26.Run_20_Run_20_hg19_

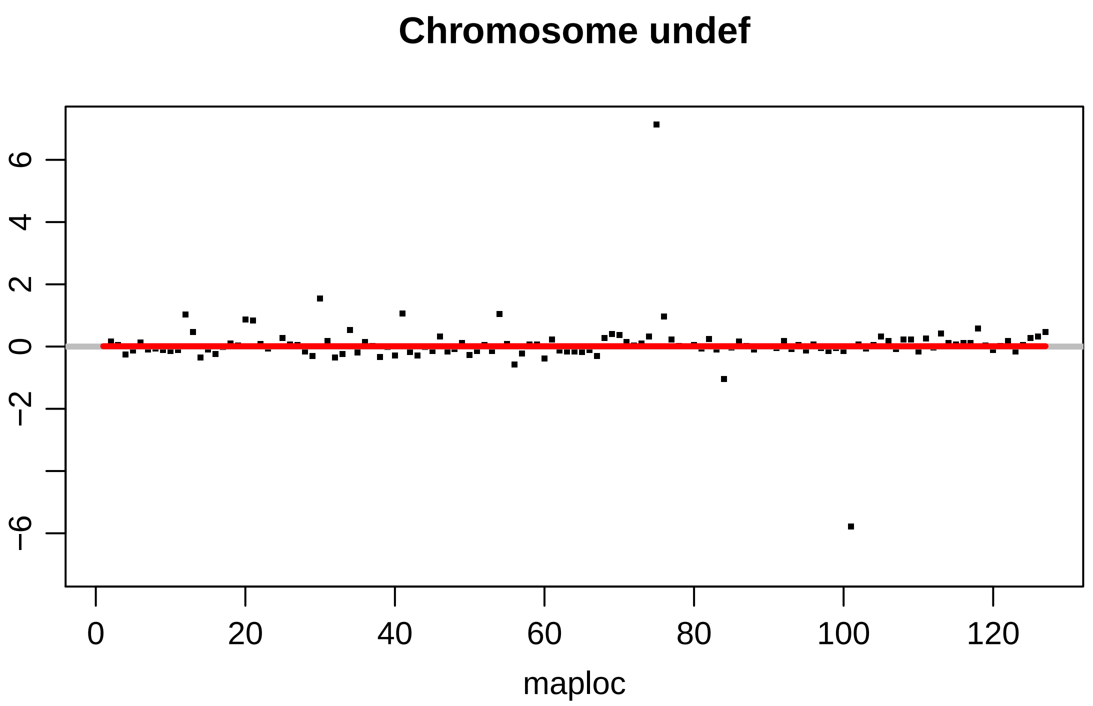


```
## Segplot might not work because of special characters in the sample names. Use only A-Z,a-z and 0-9!
##  There is a hidden function cn.mops:::.replaceNames that replaces the names in the "CNVDetectionResul
```

onXpress_004_R_2012_09_12_07_50_54_Sequoia_SN1.26.Run_20_Run_20_hg19_

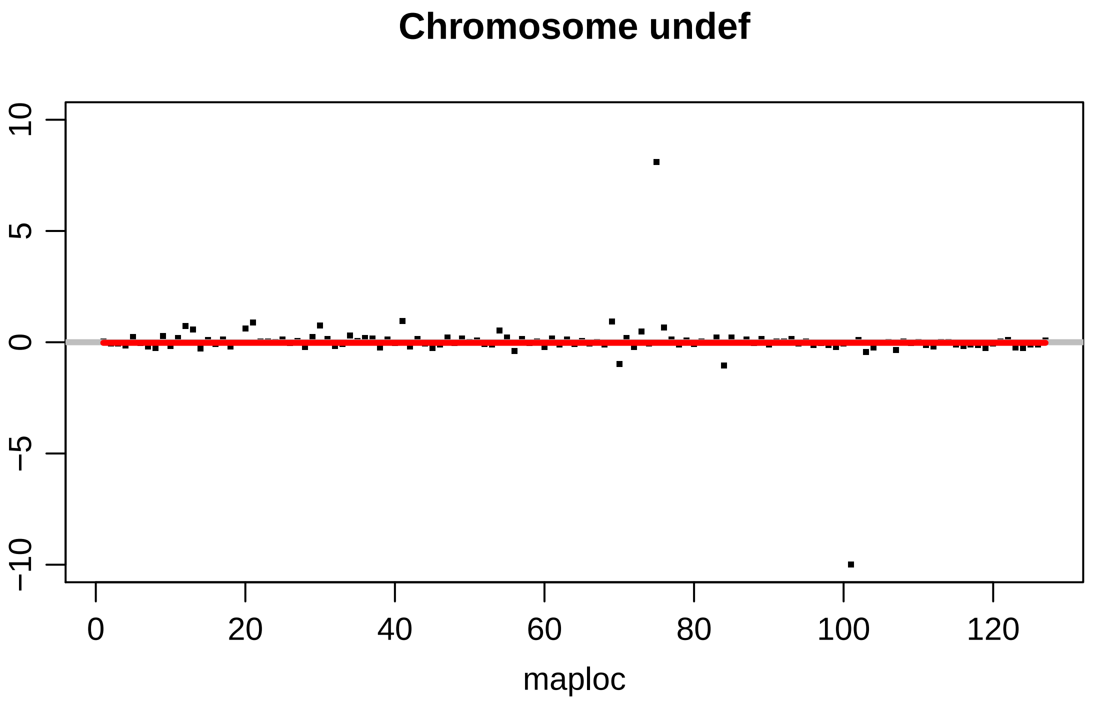

```
## Segplot might not work because of special characters in the sample names. Use only A-Z,a-z and 0-9!
##  There is a hidden function cn.mops:::.replaceNames that replaces the names in the "CNVDetectionResul
```

onXpress_005_R_2012_09_12_07_50_54_Sequoia_SN1.26.Run_20_Run_20_hg19_

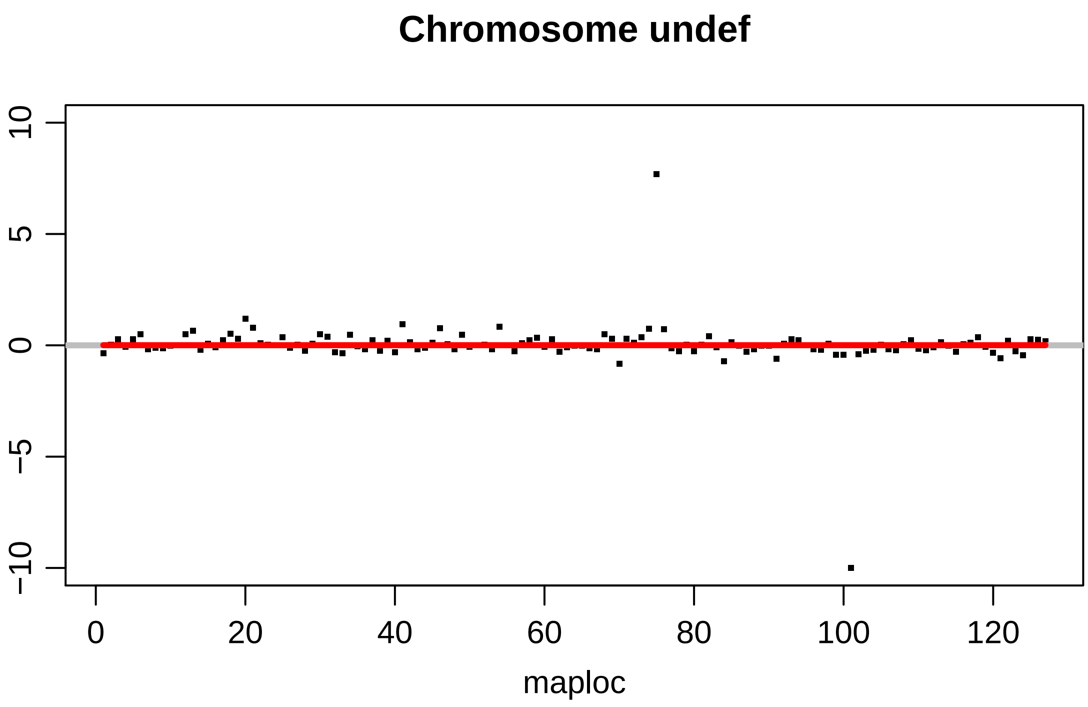


```
## Segplot might not work because of special characters in the sample names. Use only A-Z,a-z and 0-9!
##  There is a hidden function cn.mops:::.replaceNames that replaces the names in the "CNVDetectionResul
```

onXpress_006_R_2012_09_12_07_50_54_Sequoia_SN1.26.Run_20_Run_20_hg19_

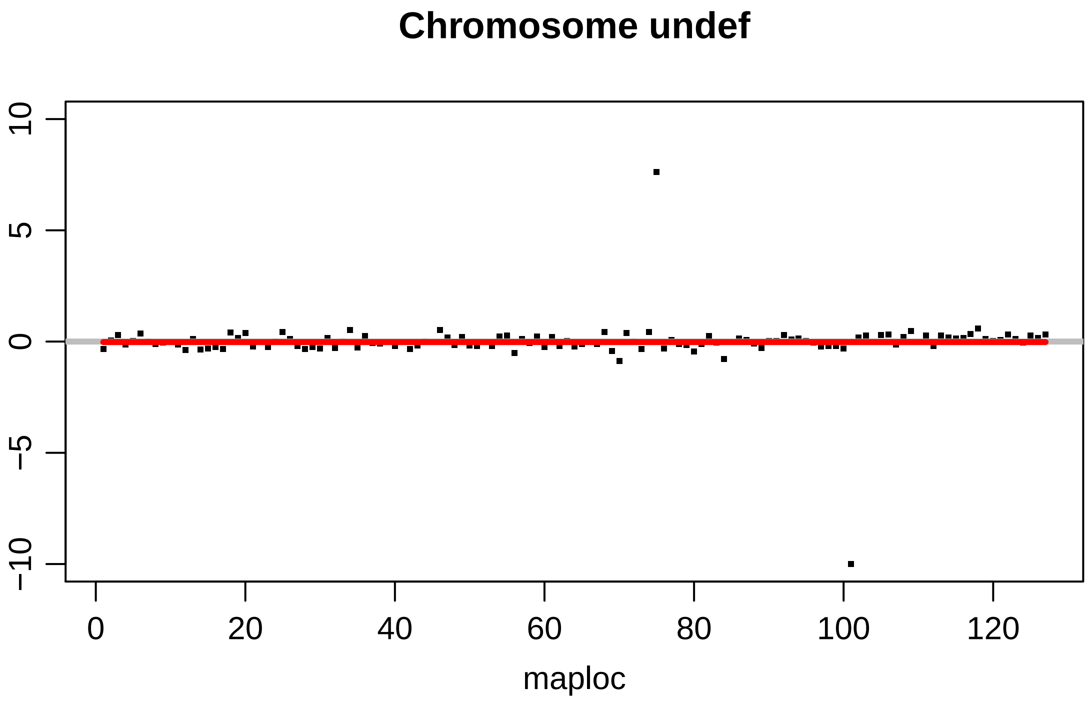


```
## Segplot might not work because of special characters in the sample names. Use only A-Z,a-z and 0-9!
##  There is a hidden function cn.mops:::.replaceNames that replaces the names in the "CNVDetectionResul
```

onXpress_007_R_2012_09_12_07_50_54_Sequoia_SN1.26.Run_20_Run_20_hg19_

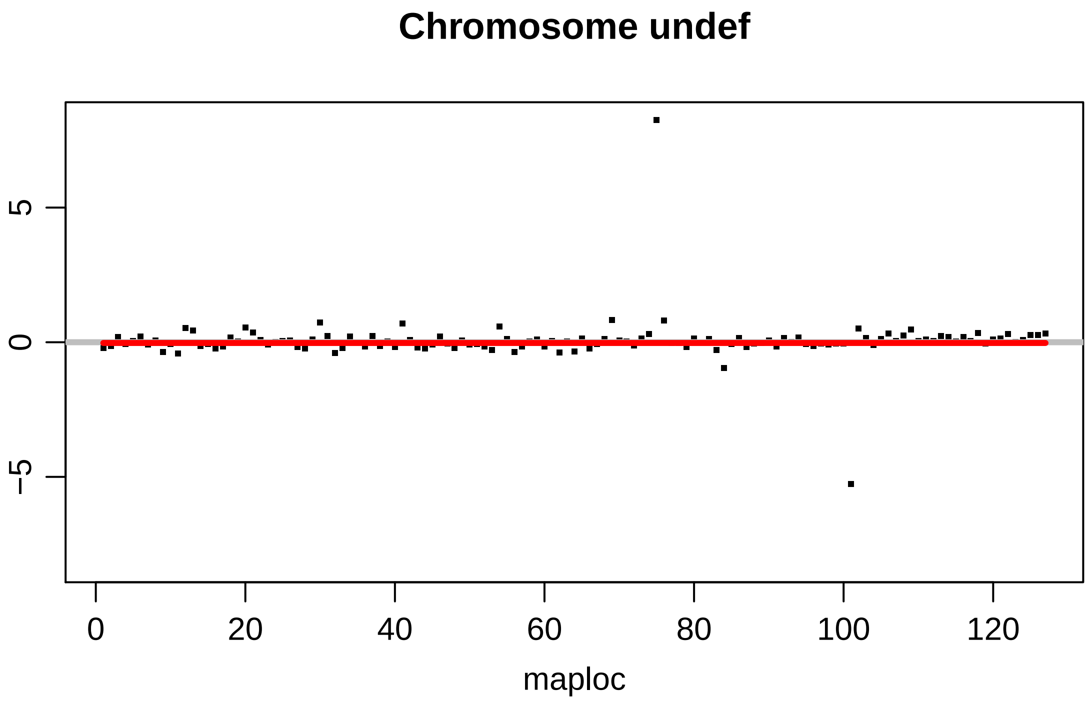

```
## Segplot might not work because of special characters in the sample names. Use only A-Z,a-z and 0-9!
##  There is a hidden function cn.mops:::.replaceNames that replaces the names in the "CNVDetectionResul
```

onXpress_008_R_2012_09_12_07_50_54_Sequoia_SN1.26.Run_20_Run_20_hg19_

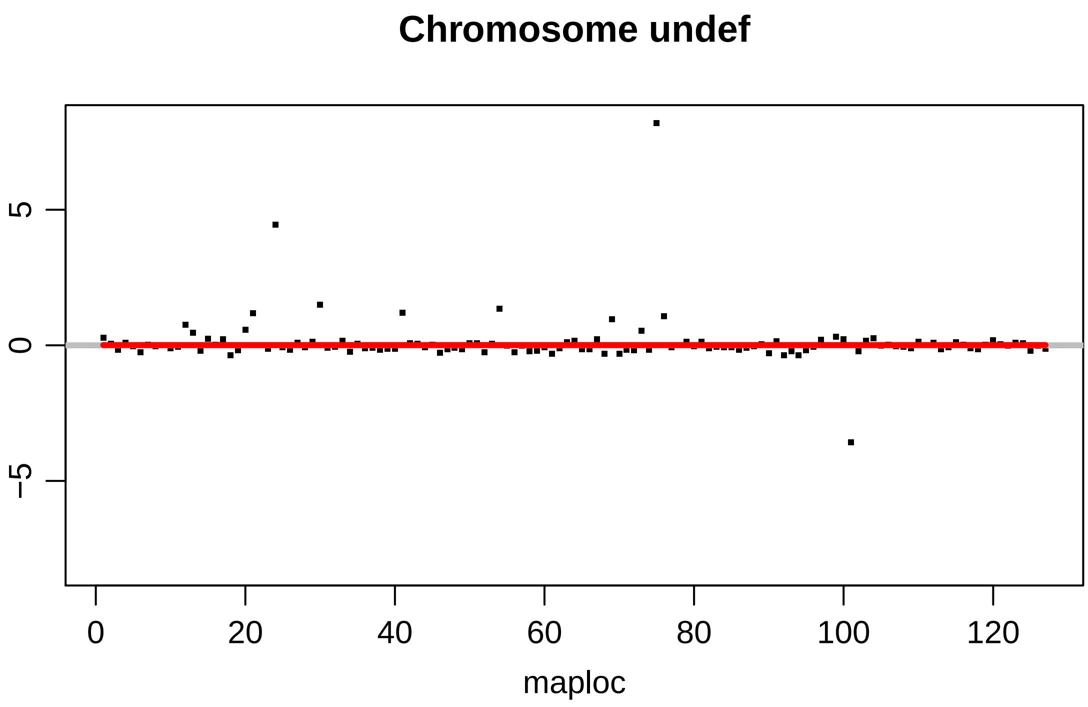


```
## Segplot might not work because of special characters in the sample names. Use only A-Z,a-z and 0-9!
##  There is a hidden function cn.mops:::.replaceNames that replaces the names in the "CNVDetectionResul
```

onXpress_009_R_2012_09_12_07_50_54_Sequoia_SN1.26.Run_20_Run_20_hg19_

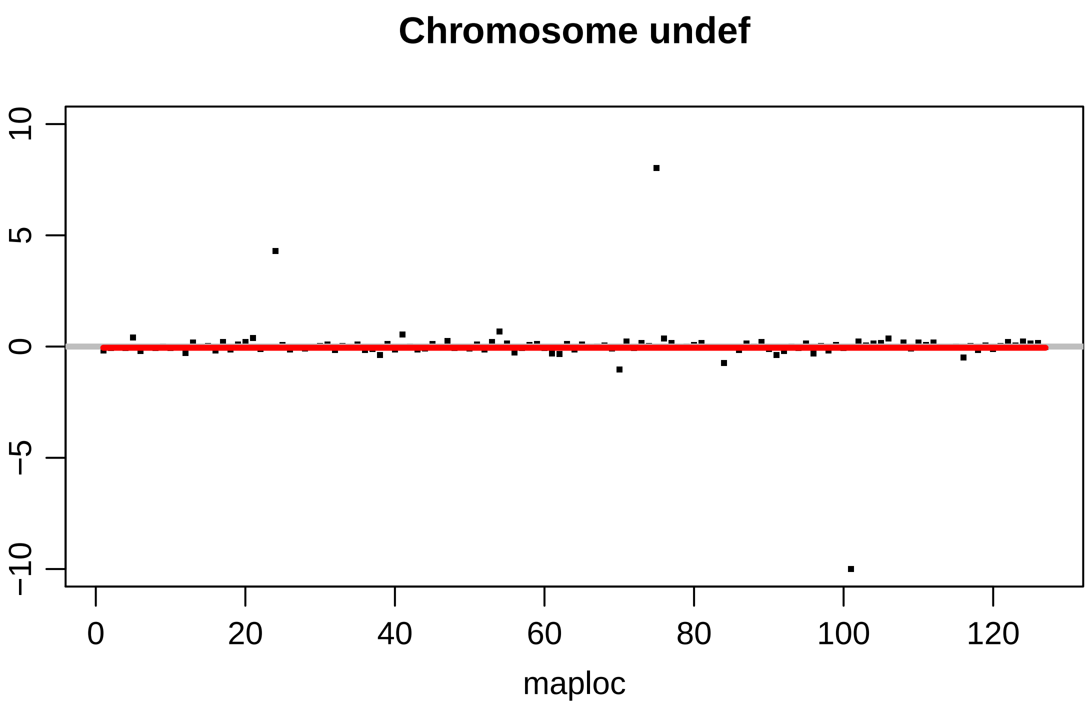


```
## Segplot might not work because of special characters in the sample names. Use only A-Z,a-z and 0-9!
##  There is a hidden function cn.mops:::.replaceNames that replaces the names in the "CNVDetectionResul
```

onXpress_010_R_2012_09_12_07_50_54_Sequoia_SN1.26.Run_20_Run_20_hg19_

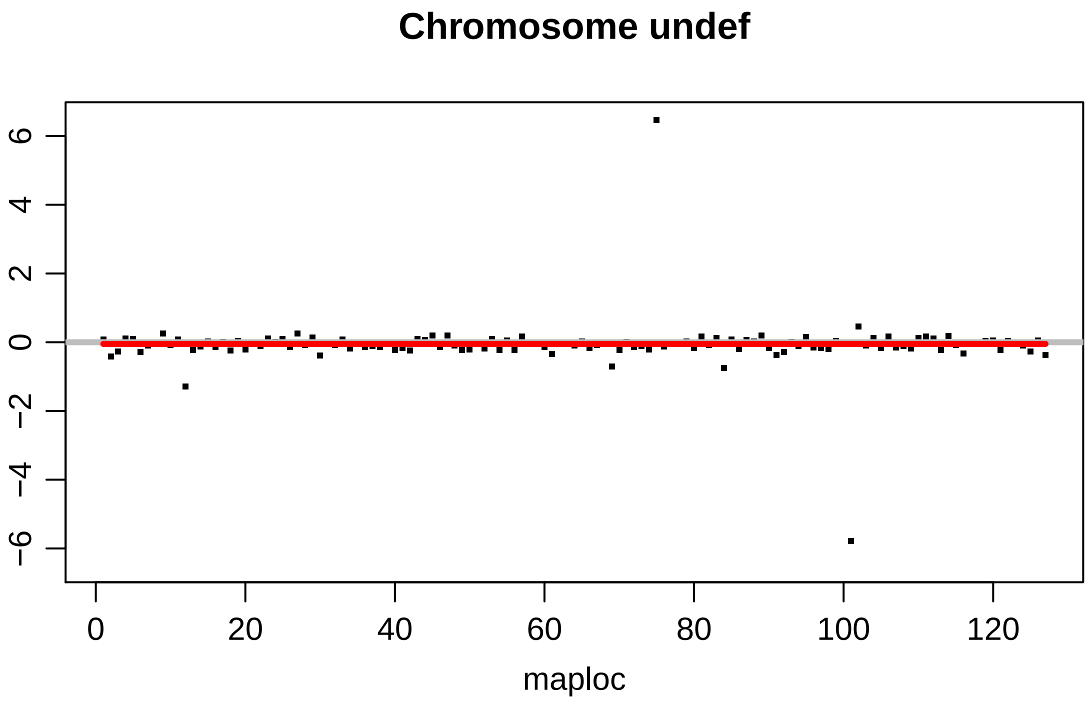

```
## Segplot might not work because of special characters in the sample names. Use only A-Z,a-z and 0-9!
##  There is a hidden function cn.mops:::.replaceNames that replaces the names in the "CNVDetectionResul
```

onXpress_011_R_2012_09_12_07_50_54_Sequoia_SN1.26.Run_20_Run_20_hg19_

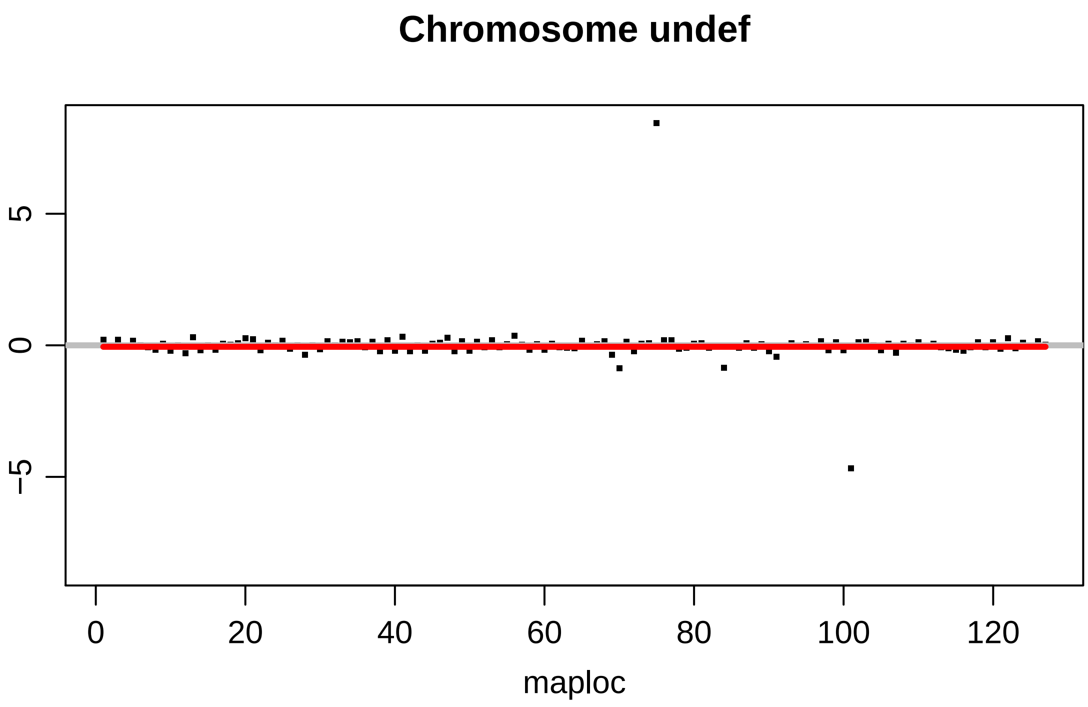


```
## Segplot might not work because of special characters in the sample names. Use only A-Z,a-z and 0-9!
##  There is a hidden function cn.mops:::.replaceNames that replaces the names in the "CNVDetectionResul
```

onXpress_012_R_2012_09_12_07_50_54_Sequoia_SN1.26.Run_20_Run_20_hg19_

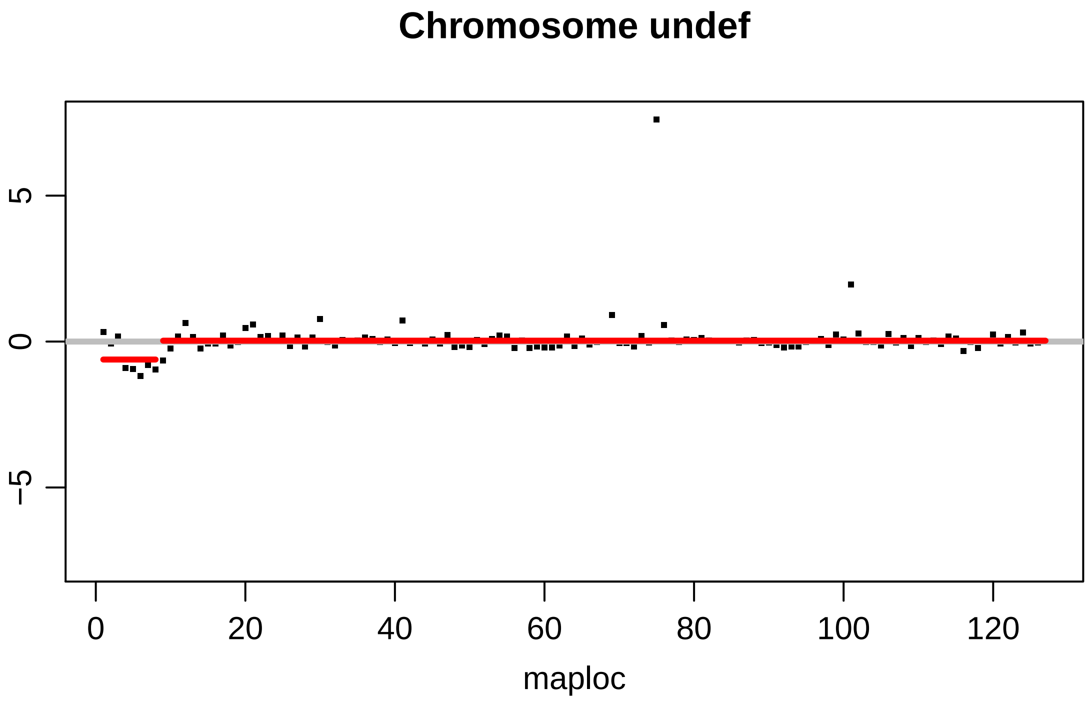


```
## Segplot might not work because of special characters in the sample names. Use only A-Z,a-z and 0-9!
##  There is a hidden function cn.mops:::.replaceNames that replaces the names in the "CNVDetectionResul
```

onXpress_013_R_2012_09_12_07_50_54_Sequoia_SN1.26.Run_20_Run_20_hg19_

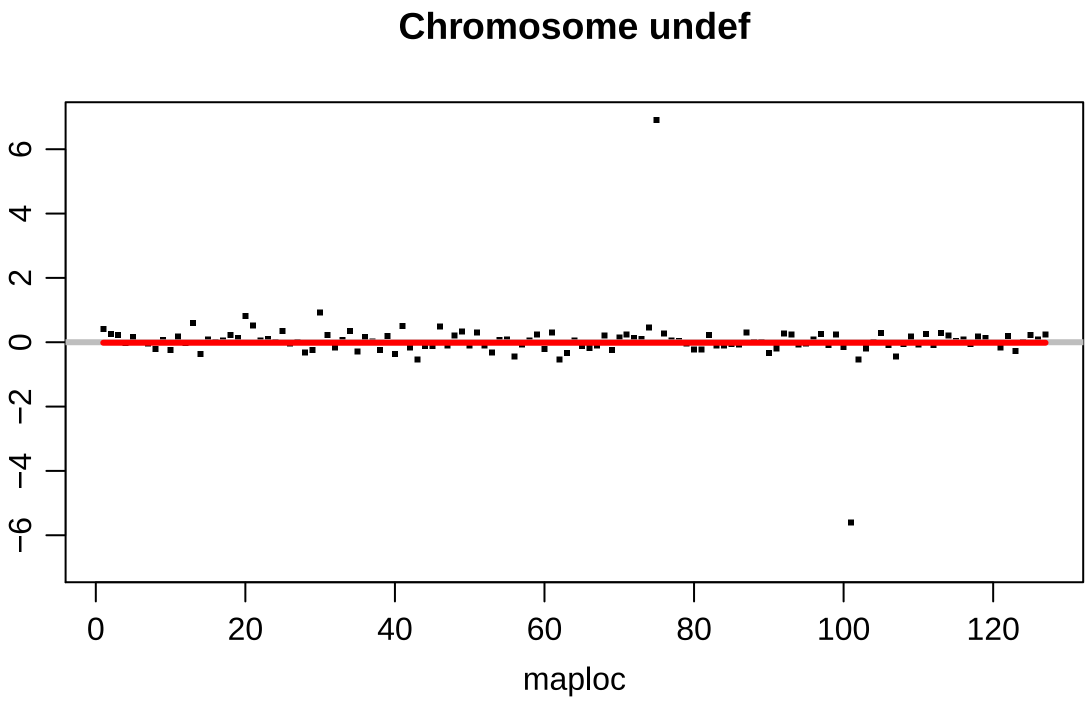

```
## Segplot might not work because of special characters in the sample names. Use only A-Z,a-z and 0-9!
##  There is a hidden function cn.mops:::.replaceNames that replaces the names in the "CNVDetectionResul
```

onXpress_014_R_2012_09_12_07_50_54_Sequoia_SN1.26.Run_20_Run_20_hg19_

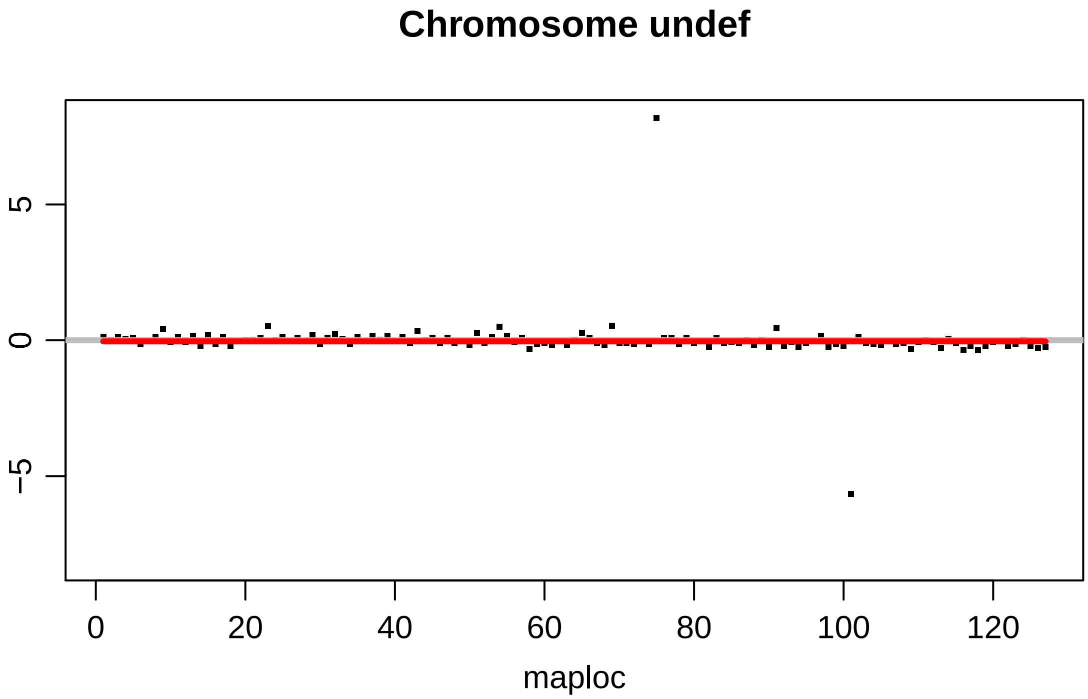


```
## Segplot might not work because of special characters in the sample names. Use only A-Z,a-z and 0-9!
##  There is a hidden function cn.mops:::.replaceNames that replaces the names in the "CNVDetectionResul
```

onXpress_015_R_2012_09_12_07_50_54_Sequoia_SN1.26.Run_20_Run_20_hg19_

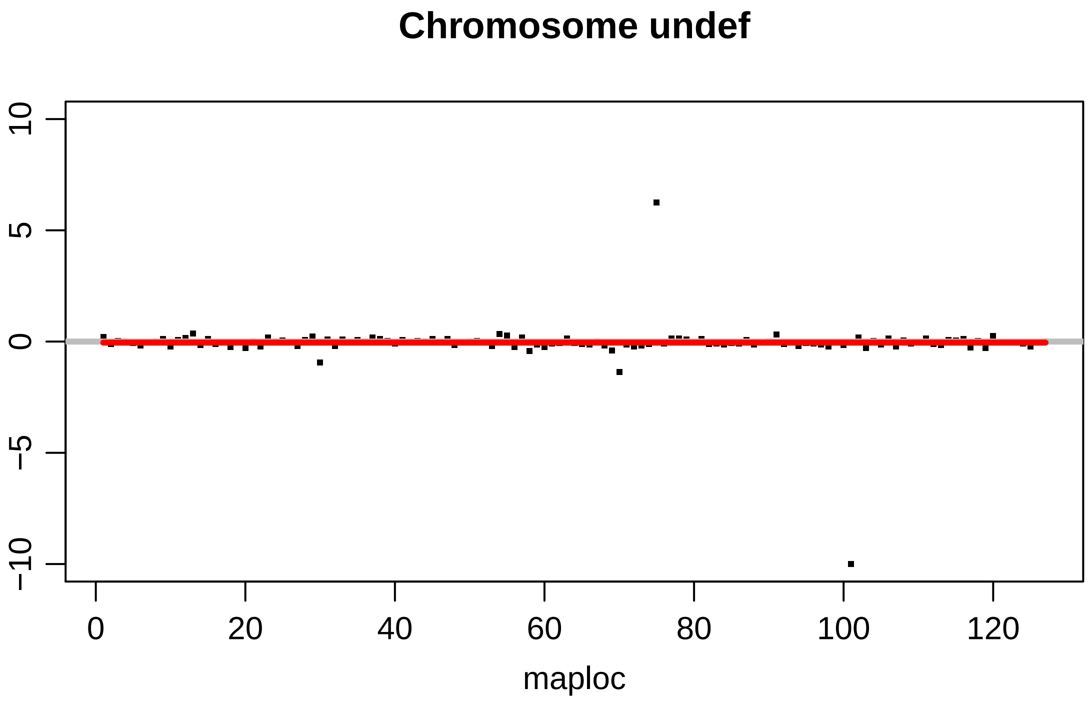


```
## Segplot might not work because of special characters in the sample names. Use only A-Z,a-z and 0-9!
##  There is a hidden function cn.mops:::.replaceNames that replaces the names in the "CNVDetectionResul
```

onXpress_016_R_2012_09_12_07_50_54_Sequoia_SN1.26.Run_20_Run_20_hg19_

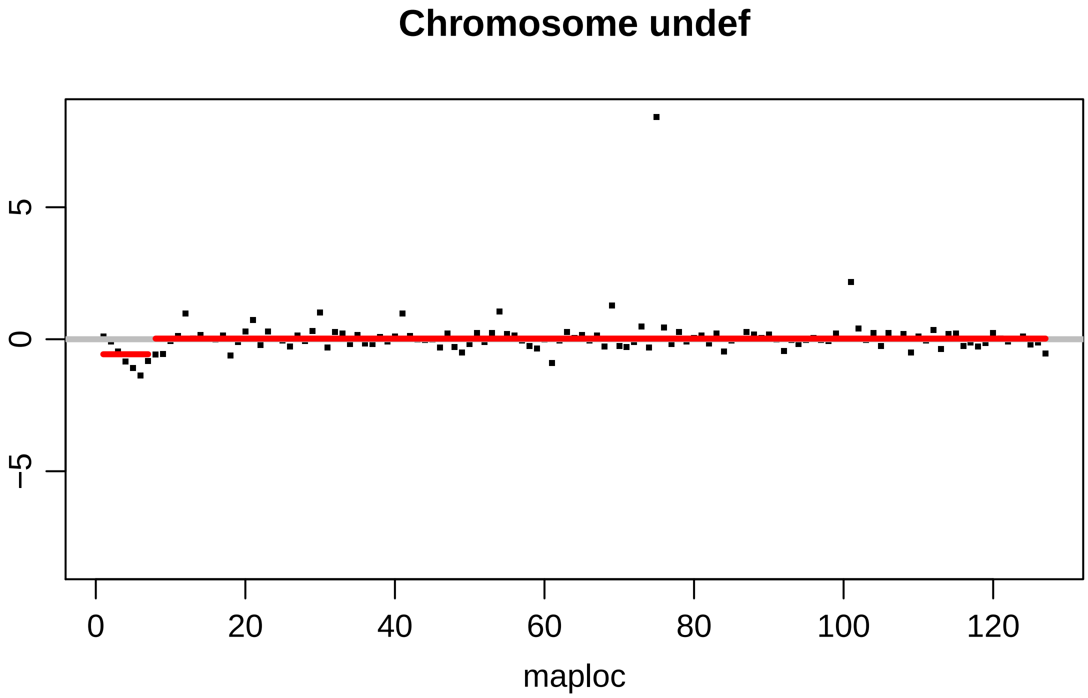

```
## Segplot might not work because of special characters in the sample names. Use only A-Z,a-z and 0-9!
##  There is a hidden function cn.mops:::.replaceNames that replaces the names in the "CNVDetectionResul
```

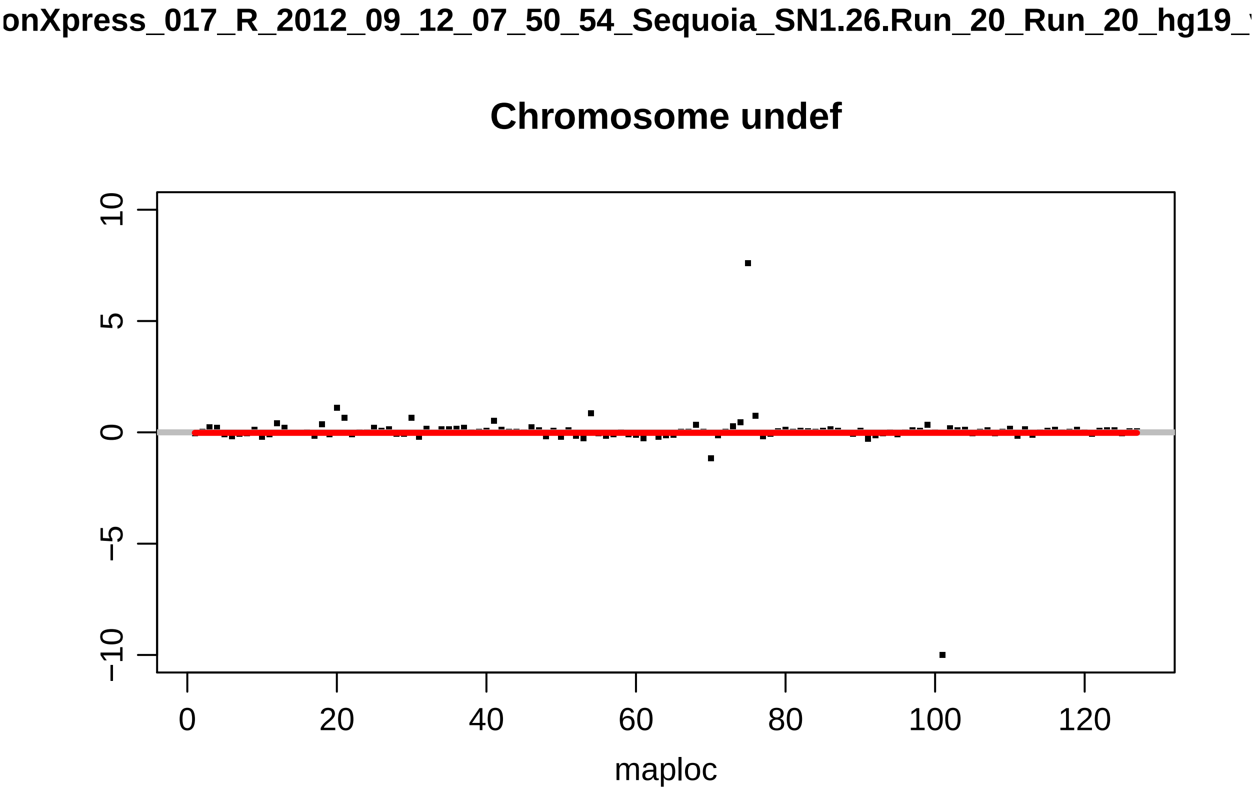


```
## Segplot might not work because of special characters in the sample names. Use only A-Z,a-z and 0-9!
##  There is a hidden function cn.mops:::.replaceNames that replaces the names in the "CNVDetectionResul
```

onXpress_018_R_2012_09_12_07_50_54_Sequoia_SN1.26.Run_20_Run_20_hg19_

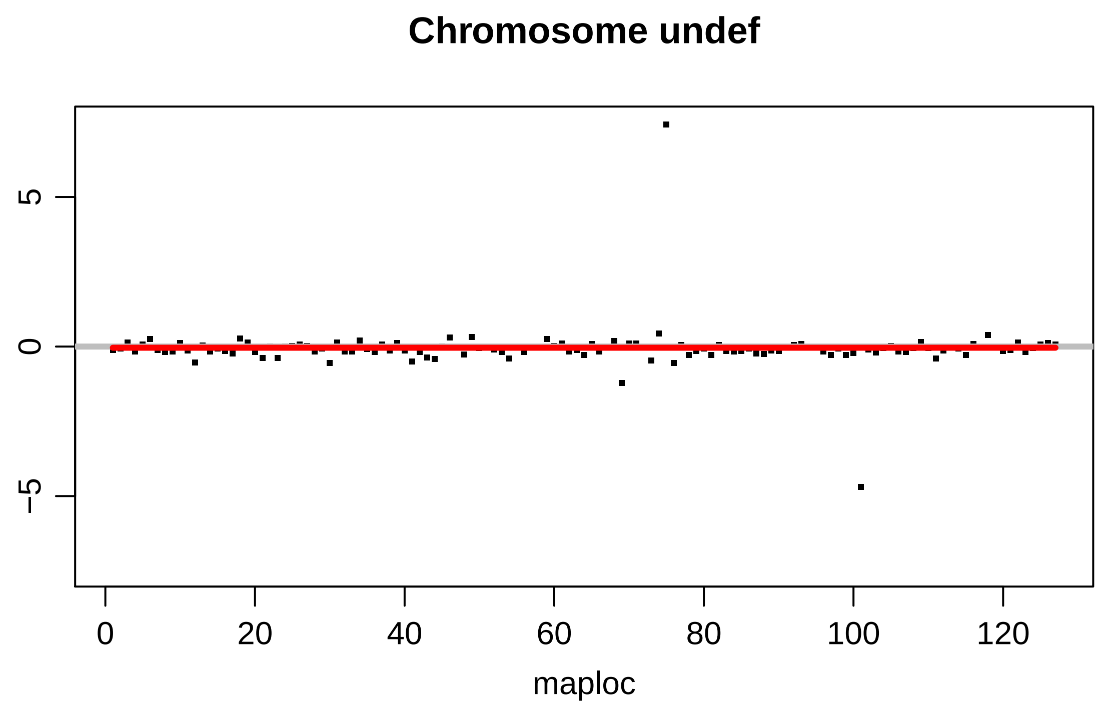


```
## Segplot might not work because of special characters in the sample names. Use only A-Z,a-z and 0-9!
##  There is a hidden function cn.mops:::.replaceNames that replaces the names in the "CNVDetectionResul
```

onXpress_019_R_2012_09_12_07_50_54_Sequoia_SN1.26.Run_20_Run_20_hg19_

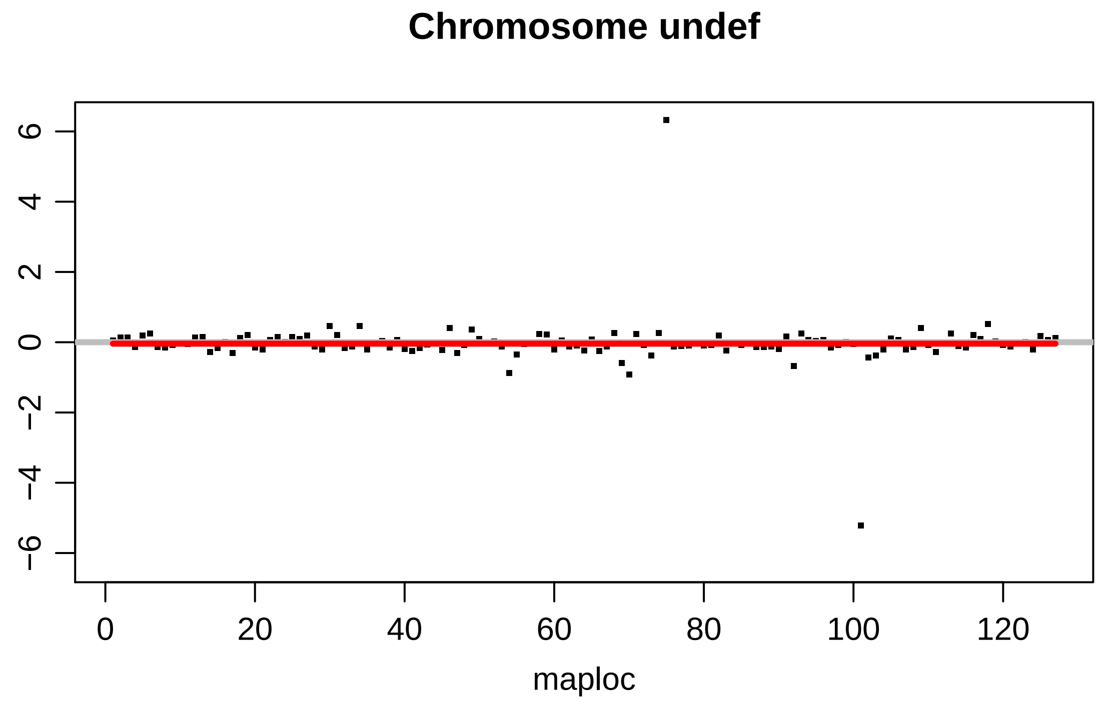

```
## Segplot might not work because of special characters in the sample names. Use only A-Z,a-z and 0-9!
##  There is a hidden function cn.mops:::.replaceNames that replaces the names in the "CNVDetectionResul
```

onXpress_020_R_2012_09_12_07_50_54_Sequoia_SN1.26.Run_20_Run_20_hg19_

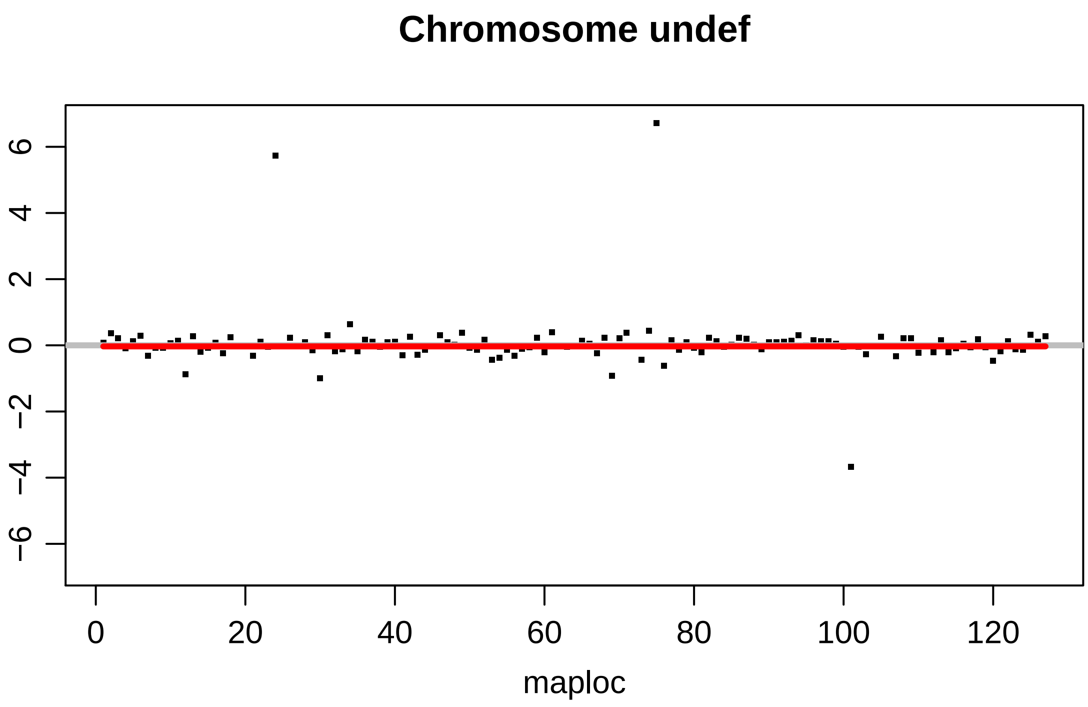


```
## Segplot might not work because of special characters in the sample names. Use only A-Z,a-z and 0-9!
##  There is a hidden function cn.mops:::.replaceNames that replaces the names in the "CNVDetectionResul
```

onXpress_021_R_2012_09_12_07_50_54_Sequoia_SN1.26.Run_20_Run_20_hg19_

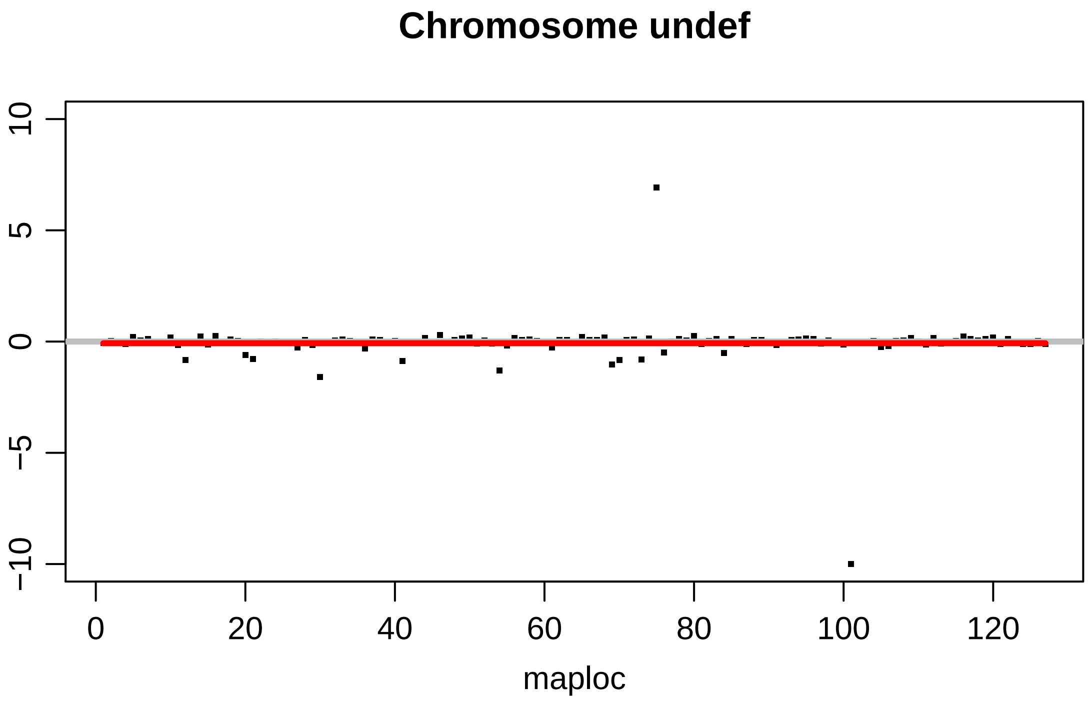


```
## Segplot might not work because of special characters in the sample names. Use only A-Z,a-z and 0-9!
##  There is a hidden function cn.mops:::.replaceNames that replaces the names in the "CNVDetectionResul
```

onXpress_022_R_2012_09_12_07_50_54_Sequoia_SN1.26.Run_20_Run_20_hg19_

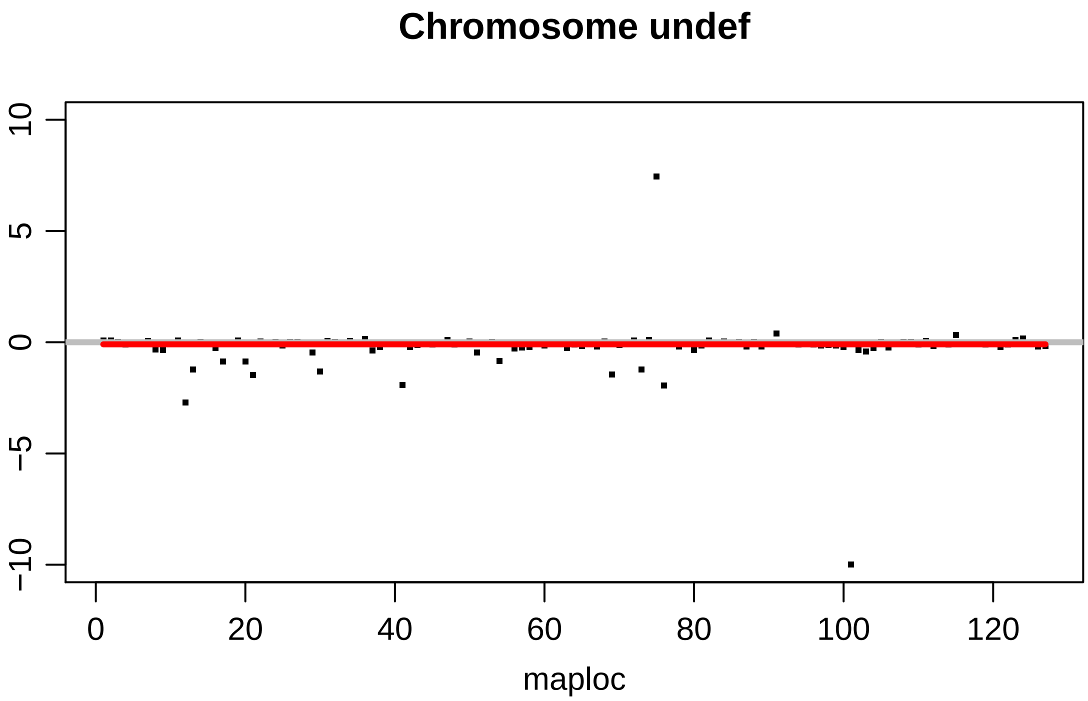

```
## Segplot might not work because of special characters in the sample names. Use only A-Z,a-z and 0-9!
##  There is a hidden function cn.mops:::.replaceNames that replaces the names in the "CNVDetectionResul
```

onXpress_023_R_2012_09_12_07_50_54_Sequoia_SN1.26.Run_20_Run_20_hg19_

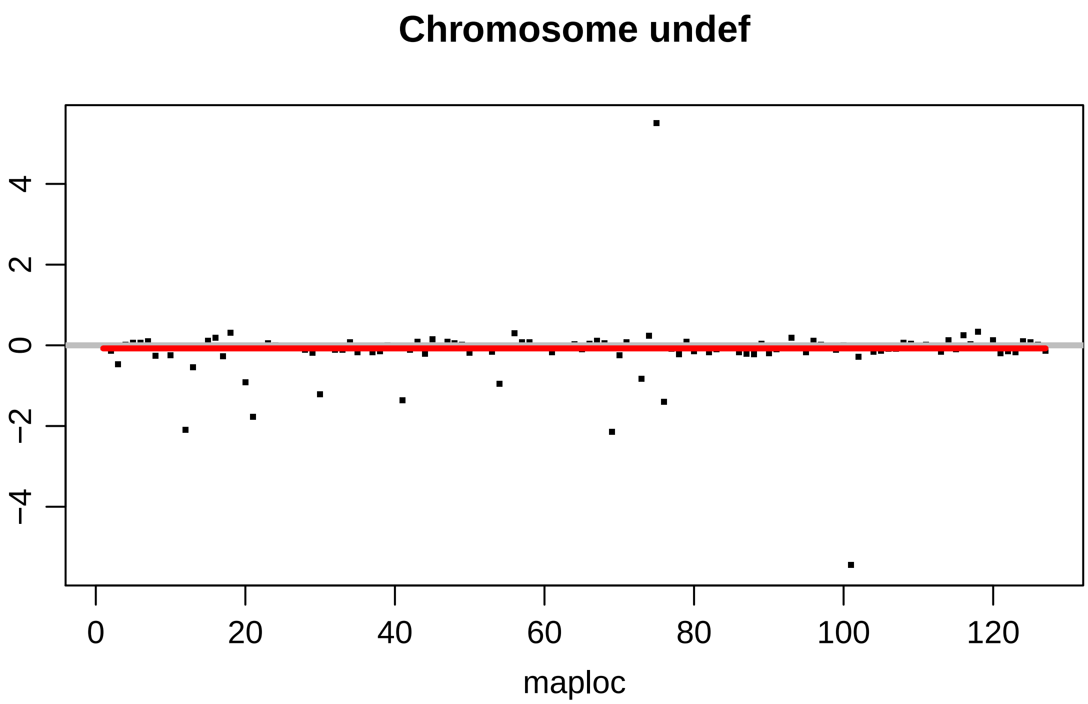


```
## Segplot might not work because of special characters in the sample names. Use only A-Z,a-z and 0-9!
##  There is a hidden function cn.mops:::.replaceNames that replaces the names in the "CNVDetectionResul
```

onXpress_024_R_2012_09_12_07_50_54_Sequoia_SN1.26.Run_20_Run_20_hg19_

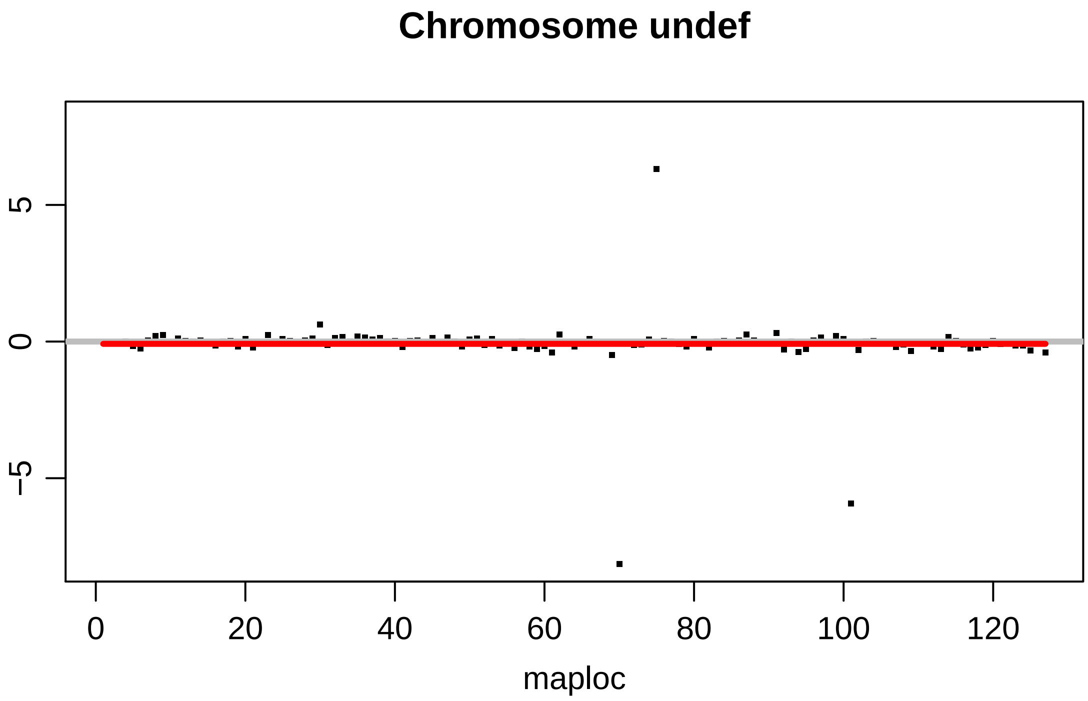


```
## Segplot might not work because of special characters in the sample names. Use only A-Z,a-z and 0-9!
##  There is a hidden function cn.mops:::.replaceNames that replaces the names in the "CNVDetectionResul
```

onXpress_025_R_2012_09_12_07_50_54_Sequoia_SN1.26.Run_20_Run_20_hg19_

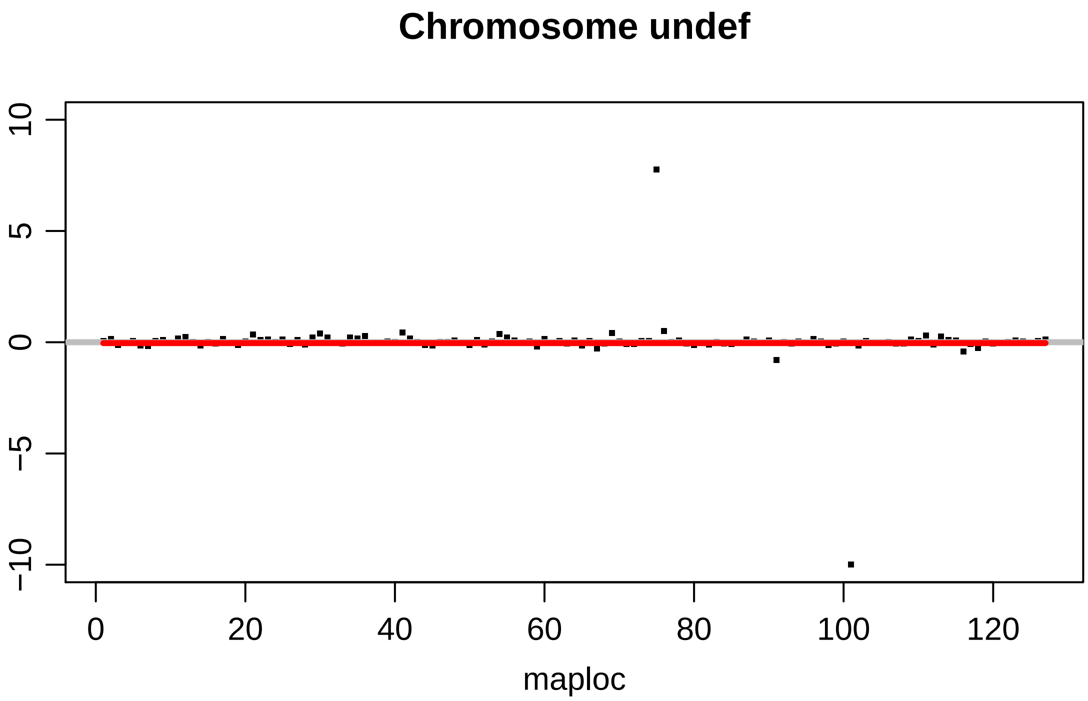

```
## Segplot might not work because of special characters in the sample names. Use only A-Z,a-z and 0-9!
##  There is a hidden function cn.mops:::.replaceNames that replaces the names in the "CNVDetectionResul
```

onXpress_026_R_2012_09_12_07_50_54_Sequoia_SN1.26.Run_20_Run_20_hg19_

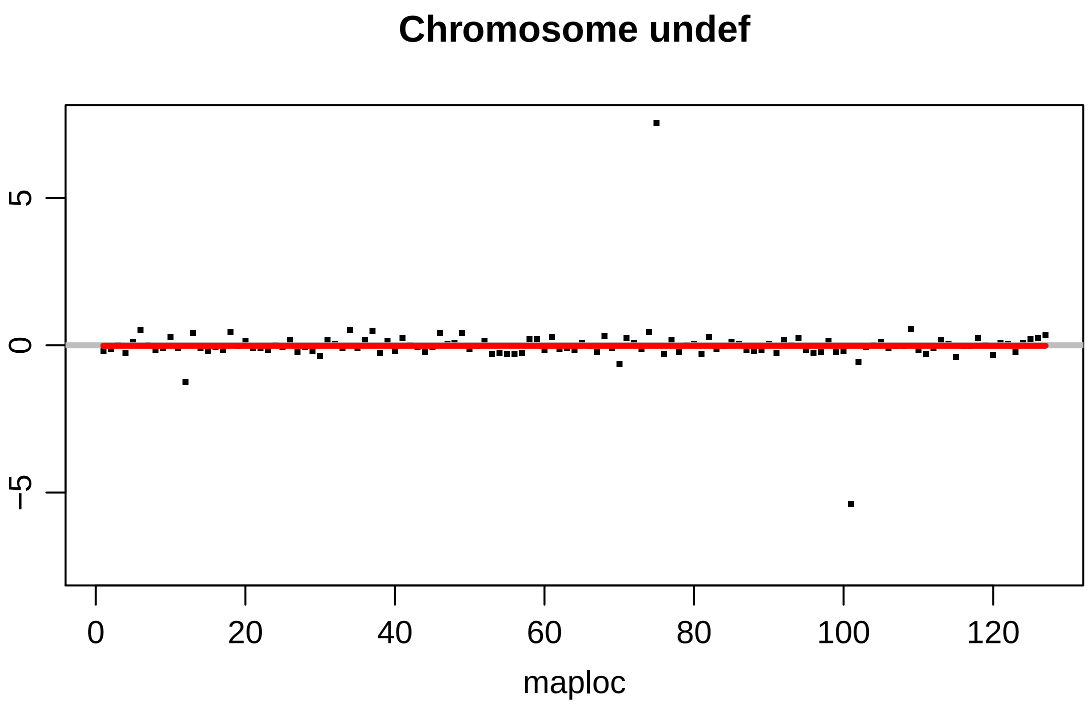


```
## Segplot might not work because of special characters in the sample names. Use only A-Z,a-z and 0-9!
##  There is a hidden function cn.mops:::.replaceNames that replaces the names in the "CNVDetectionResul
```

onXpress_027_R_2012_09_12_07_50_54_Sequoia_SN1.26.Run_20_Run_20_hg19_

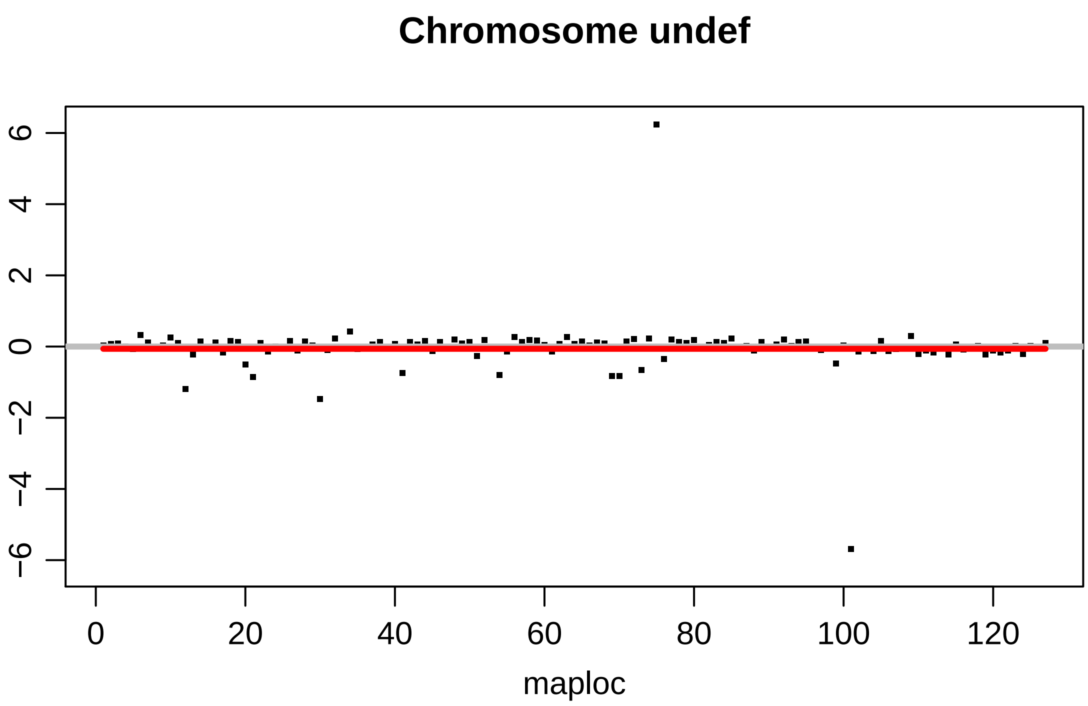


```
## Segplot might not work because of special characters in the sample names. Use only A-Z,a-z and 0-9!
##  There is a hidden function cn.mops:::.replaceNames that replaces the names in the "CNVDetectionResul
```

onXpress_028_R_2012_09_12_07_50_54_Sequoia_SN1.26.Run_20_Run_20_hg19_

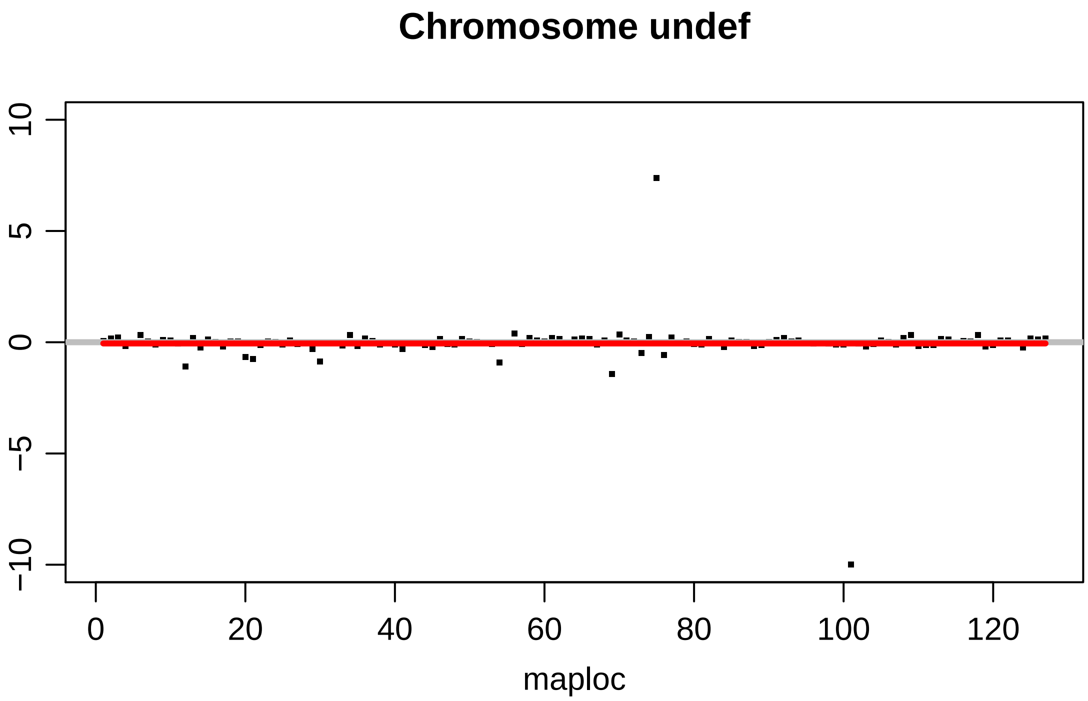

```
## Segplot might not work because of special characters in the sample names. Use only A-Z,a-z and 0-9!
##  There is a hidden function cn.mops:::.replaceNames that replaces the names in the "CNVDetectionResul
```

onXpress_029_R_2012_09_12_07_50_54_Sequoia_SN1.26.Run_20_Run_20_hg19_

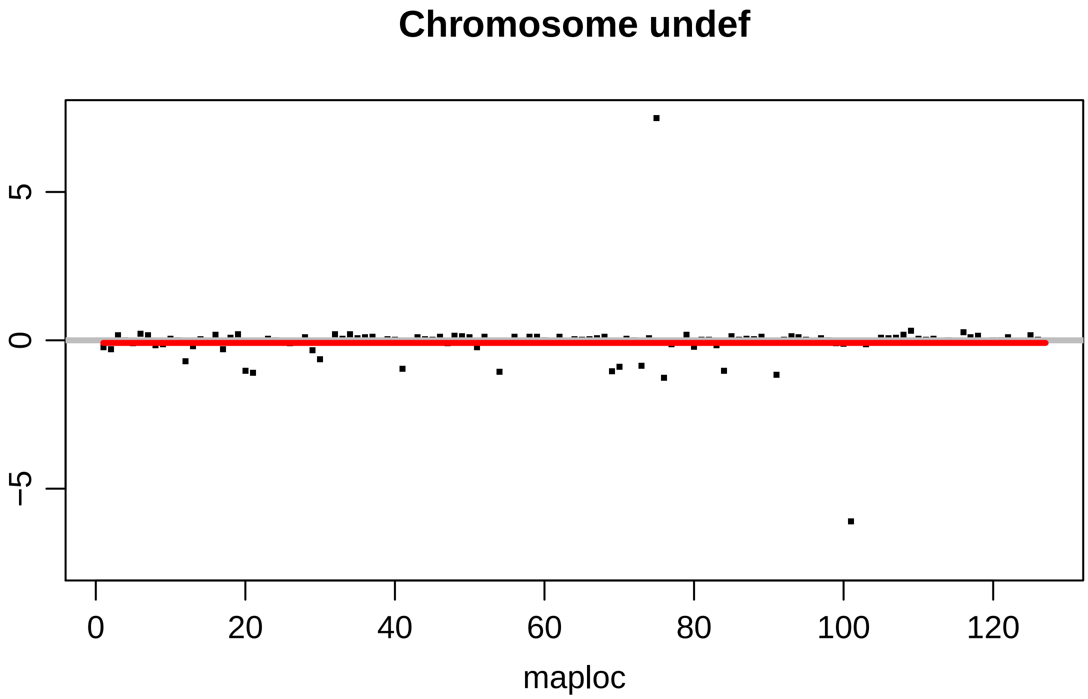


```
## Segplot might not work because of special characters in the sample names. Use only A-Z,a-z and 0-9!
##  There is a hidden function cn.mops:::.replaceNames that replaces the names in the "CNVDetectionResul
```

onXpress_030_R_2012_09_12_07_50_54_Sequoia_SN1.26.Run_20_Run_20_hg19_

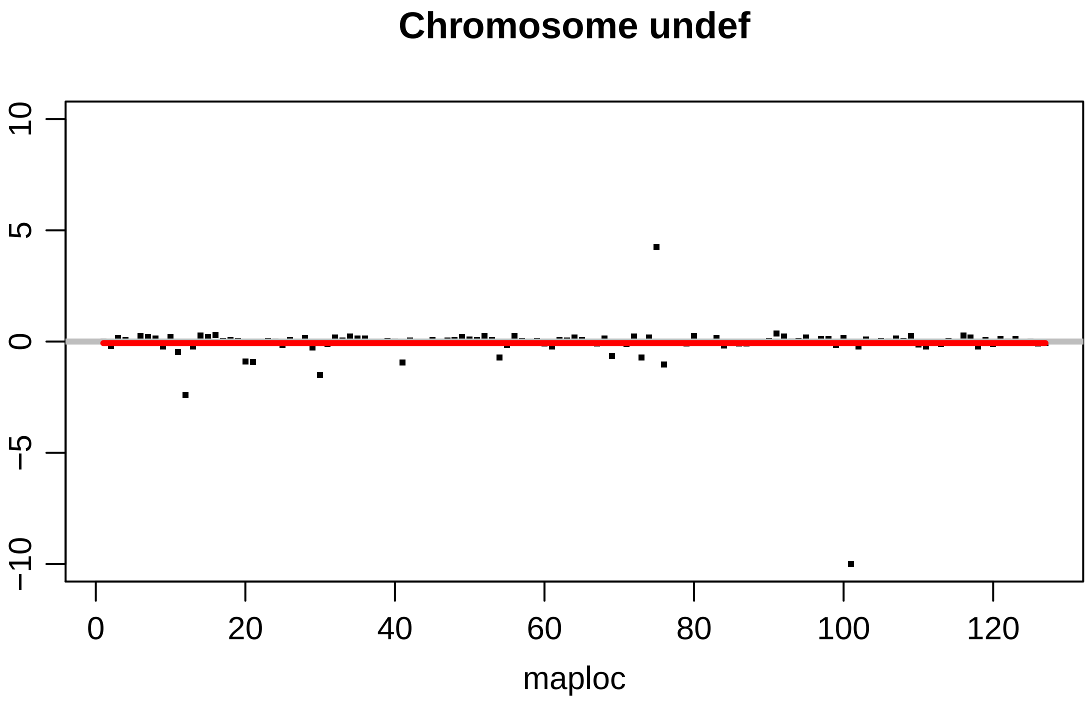


```
## Segplot might not work because of special characters in the sample names. Use only A-Z,a-z and 0-9!
##  There is a hidden function cn.mops:::.replaceNames that replaces the names in the "CNVDetectionResul
```

onXpress_031_R_2012_09_12_07_50_54_Sequoia_SN1.26.Run_20_Run_20_hg19_

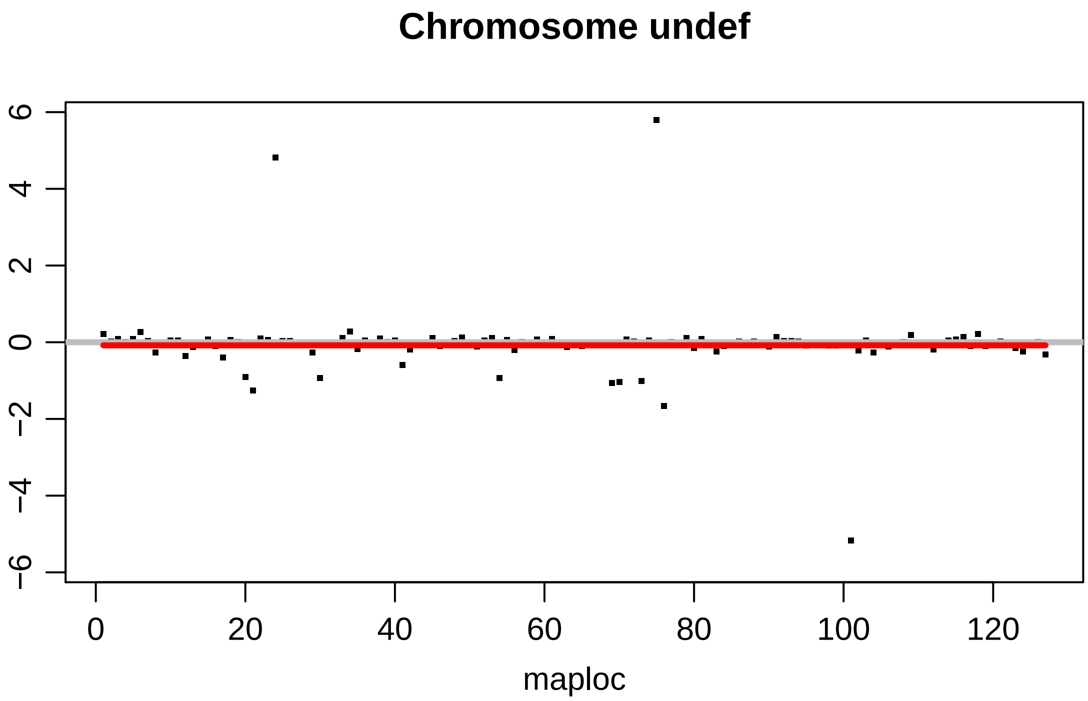

```
## Segplot might not work because of special characters in the sample names. Use only A-Z,a-z and 0-9!
##  There is a hidden function cn.mops:::.replaceNames that replaces the names in the "CNVDetectionResul
```

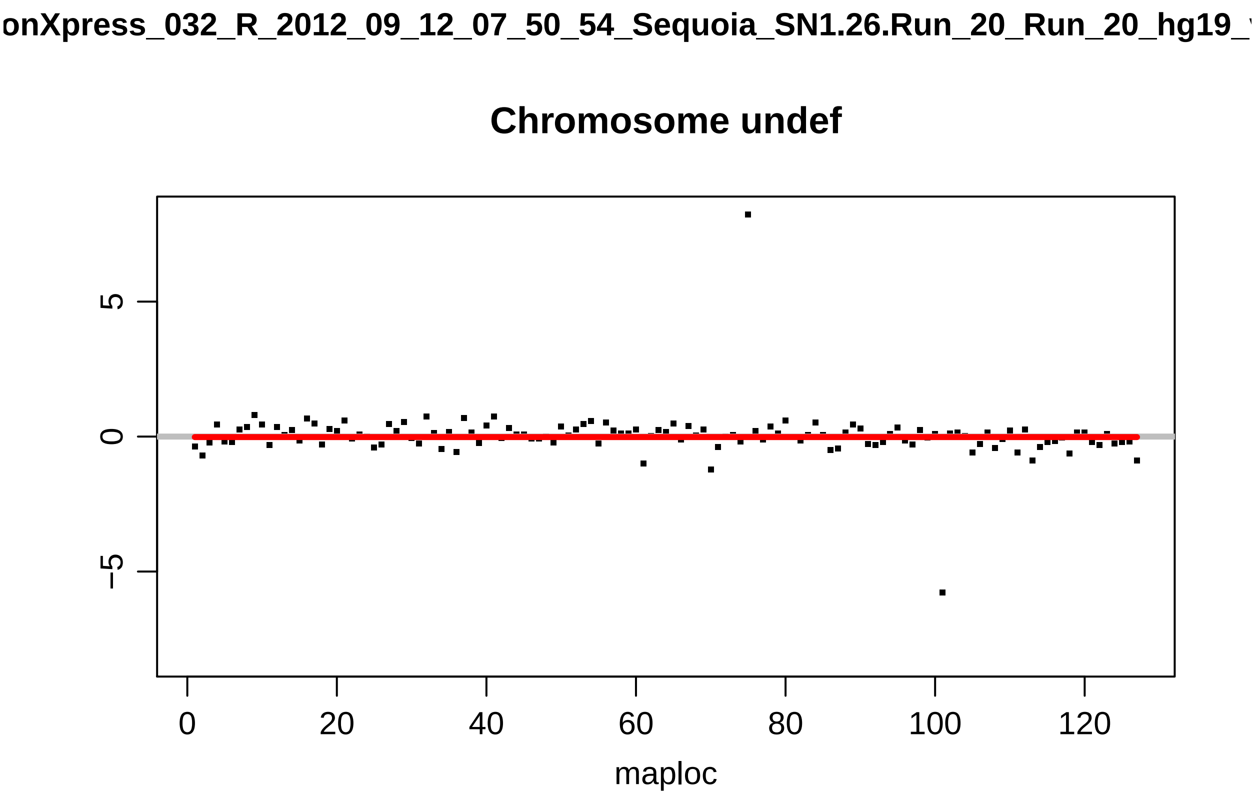


```
## Segplot might not work because of special characters in the sample names. Use only A-Z,a-z and 0-9!
##  There is a hidden function cn.mops:::.replaceNames that replaces the names in the "CNVDetectionResul
```

onXpress_033_R_2012_09_12_07_50_54_Sequoia_SN1.26.Run_20_Run_20_hg19_

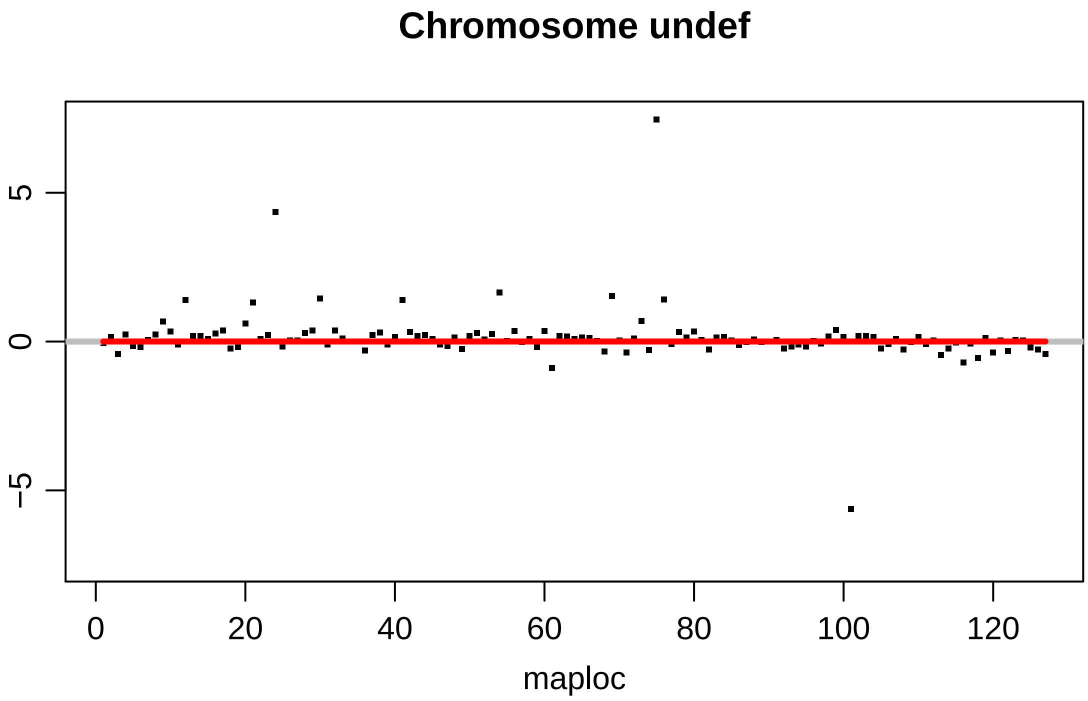


```
## Segplot might not work because of special characters in the sample names. Use only A-Z,a-z and 0-9!
##  There is a hidden function cn.mops:::.replaceNames that replaces the names in the "CNVDetectionResul
```

onXpress_034_R_2012_09_12_07_50_54_Sequoia_SN1.26.Run_20_Run_20_hg19_

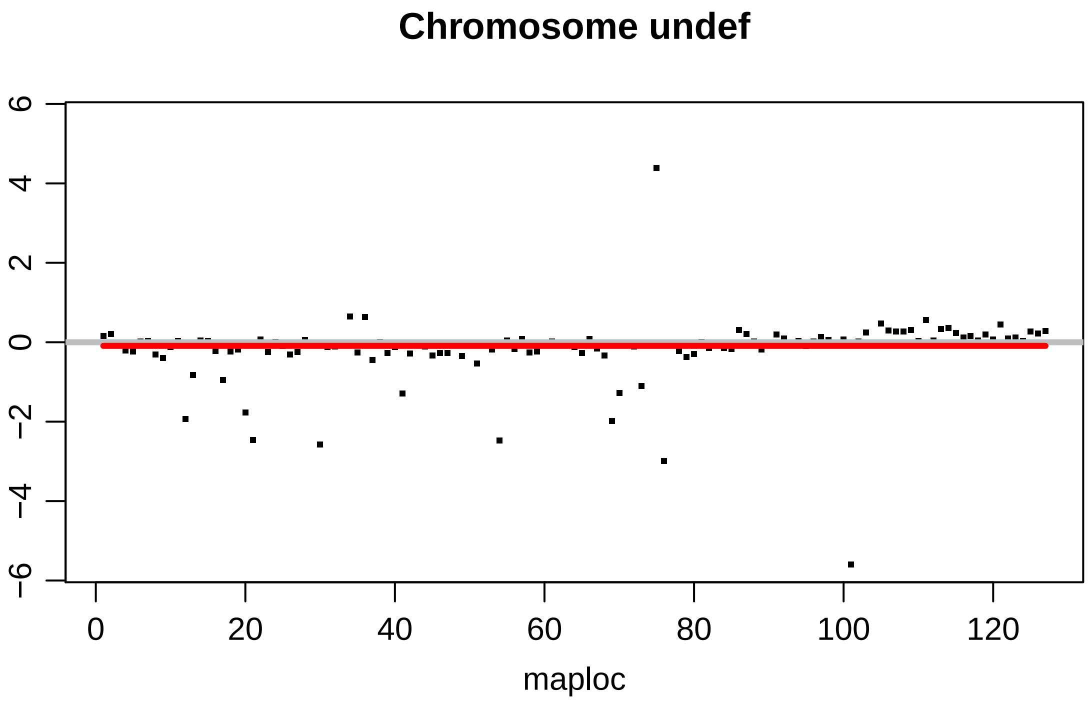

```
## Segplot might not work because of special characters in the sample names. Use only A-Z,a-z and 0-9!
##  There is a hidden function cn.mops:::.replaceNames that replaces the names in the "CNVDetectionResul
```

onXpress_035_R_2012_09_12_07_50_54_Sequoia_SN1.26.Run_20_Run_20_hg19_

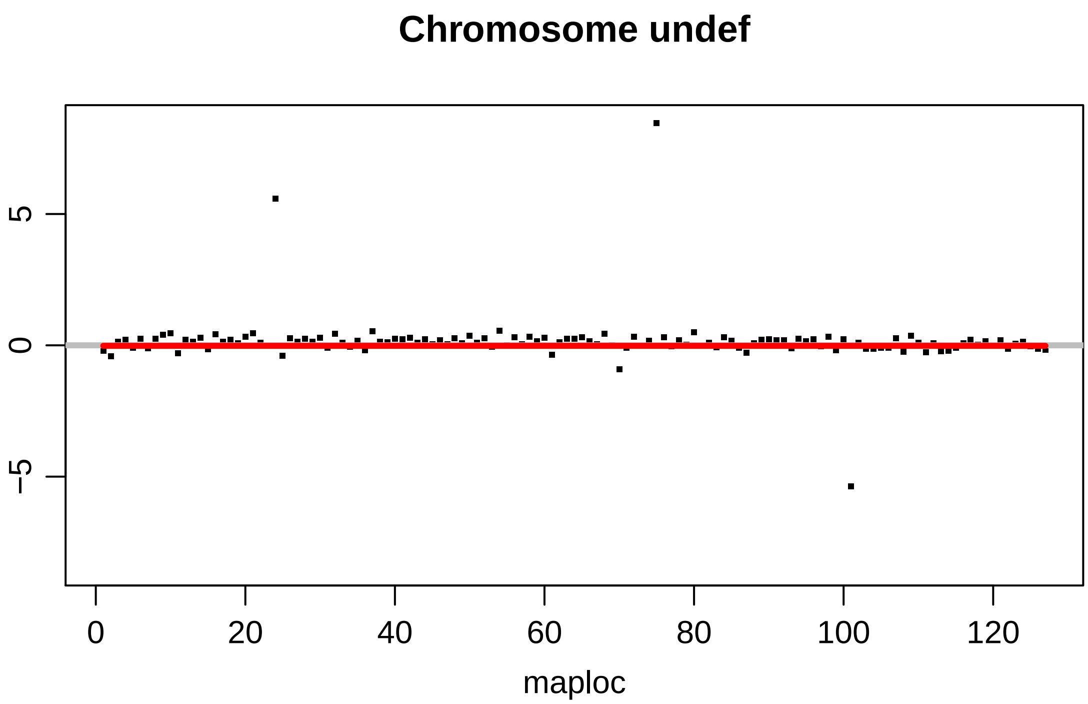


```
## Segplot might not work because of special characters in the sample names. Use only A-Z,a-z and 0-9!
##  There is a hidden function cn.mops:::.replaceNames that replaces the names in the "CNVDetectionResul
```

onXpress_036_R_2012_09_12_07_50_54_Sequoia_SN1.26.Run_20_Run_20_hg19_

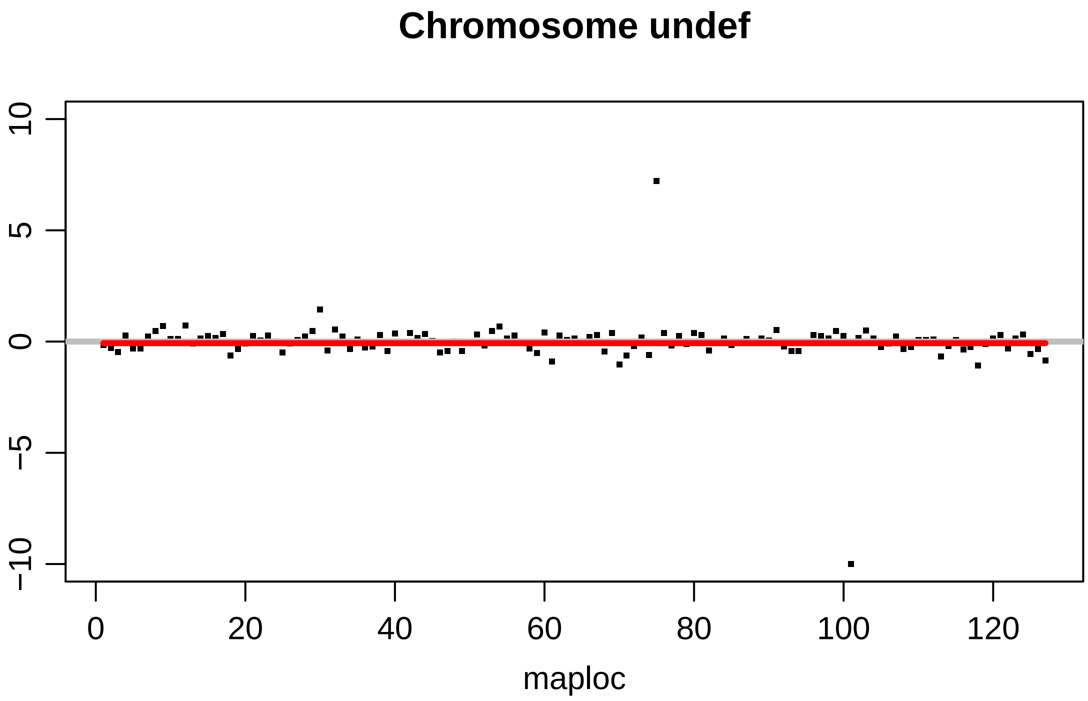


```
## Segplot might not work because of special characters in the sample names. Use only A-Z,a-z and 0-9!
##  There is a hidden function cn.mops:::.replaceNames that replaces the names in the "CNVDetectionResul
```

onXpress_037_R_2012_09_12_07_50_54_Sequoia_SN1.26.Run_20_Run_20_hg19_

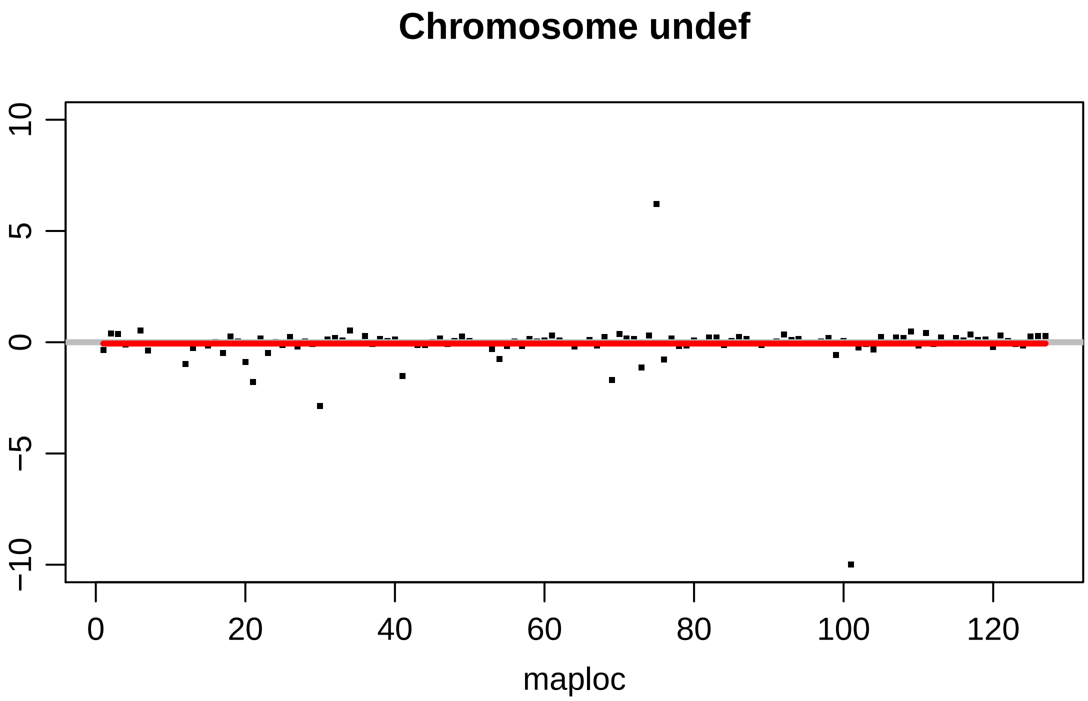

```
## Segplot might not work because of special characters in the sample names. Use only A-Z,a-z and 0-9!
##  There is a hidden function cn.mops:::.replaceNames that replaces the names in the "CNVDetectionResul
```

onXpress_038_R_2012_09_12_07_50_54_Sequoia_SN1.26.Run_20_Run_20_hg19_

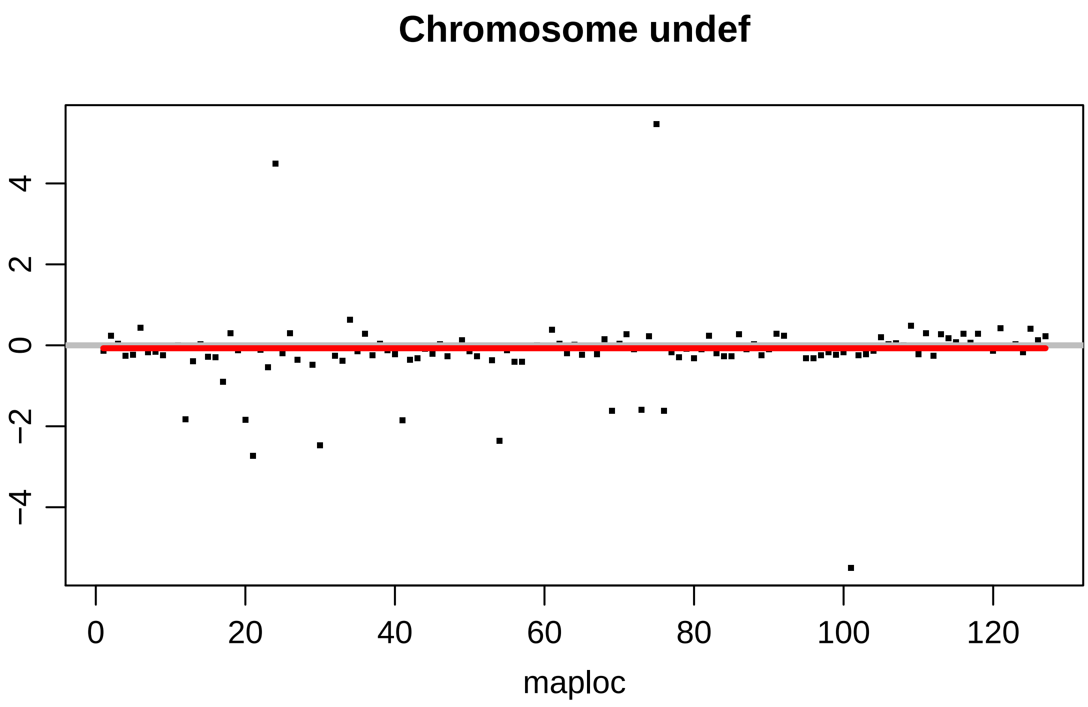


```
## Segplot might not work because of special characters in the sample names. Use only A-Z,a-z and 0-9!
##  There is a hidden function cn.mops:::.replaceNames that replaces the names in the "CNVDetectionResul
```

onXpress_039_R_2012_09_12_07_50_54_Sequoia_SN1.26.Run_20_Run_20_hg19_

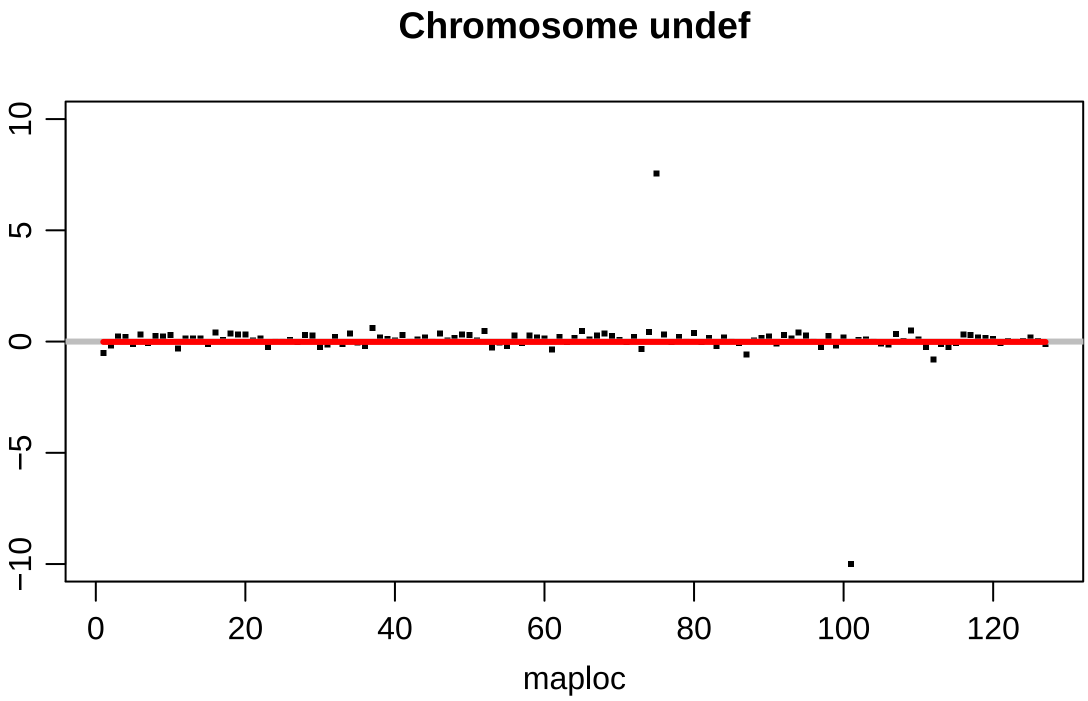


```
## Segplot might not work because of special characters in the sample names. Use only A-Z,a-z and 0-9!
##  There is a hidden function cn.mops:::.replaceNames that replaces the names in the "CNVDetectionResul
```

onXpress_040_R_2012_09_12_07_50_54_Sequoia_SN1.26.Run_20_Run_20_hg19_

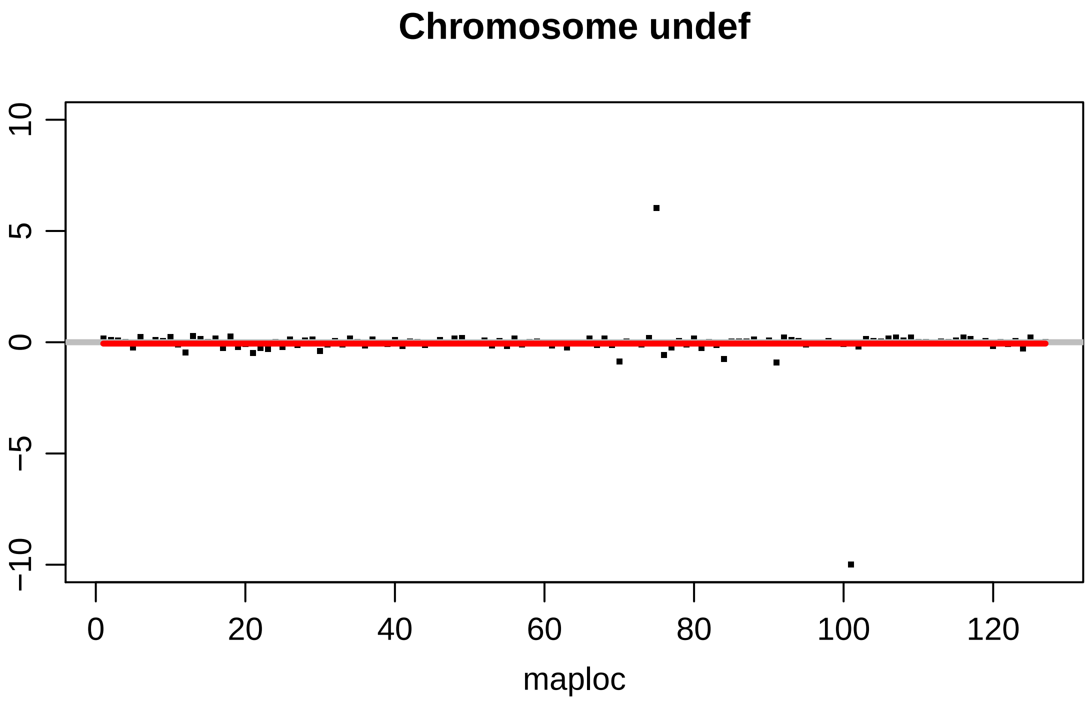

```
## Segplot might not work because of special characters in the sample names. Use only A-Z,a-z and 0-9!
##  There is a hidden function cn.mops:::.replaceNames that replaces the names in the "CNVDetectionResul
```

onXpress_041_R_2012_09_12_07_50_54_Sequoia_SN1.26.Run_20_Run_20_hg19_

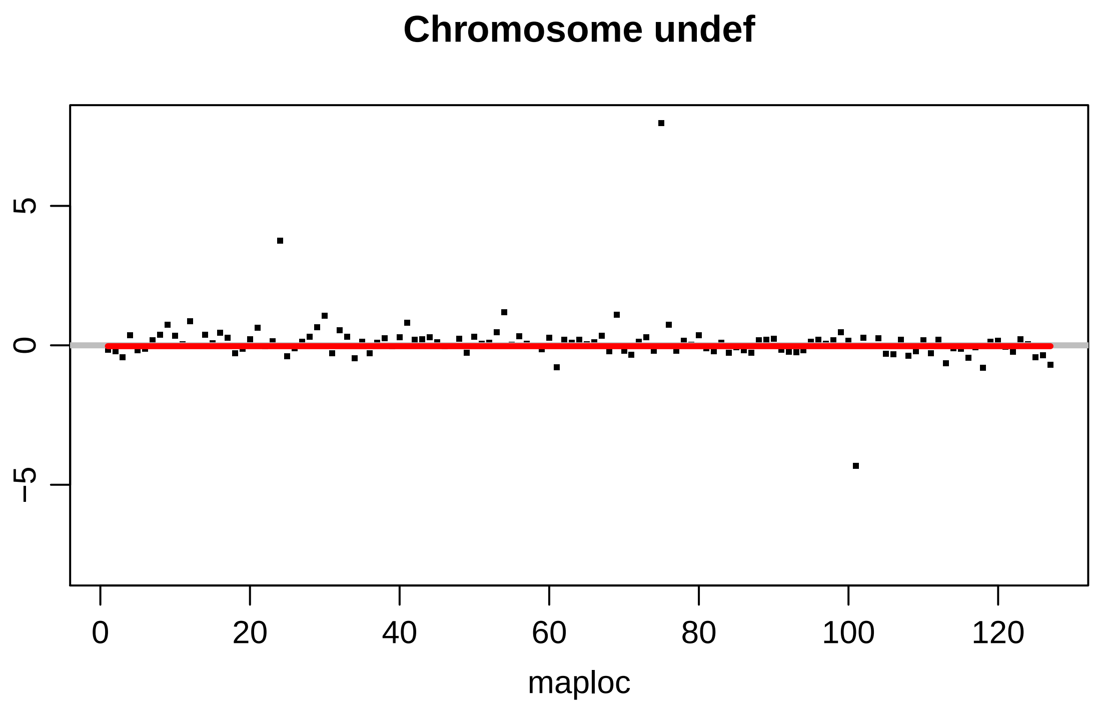


```
## Segplot might not work because of special characters in the sample names. Use only A-Z,a-z and 0-9!
##  There is a hidden function cn.mops:::.replaceNames that replaces the names in the "CNVDetectionResul
```

onXpress_042_R_2012_09_12_07_50_54_Sequoia_SN1.26.Run_20_Run_20_hg19_

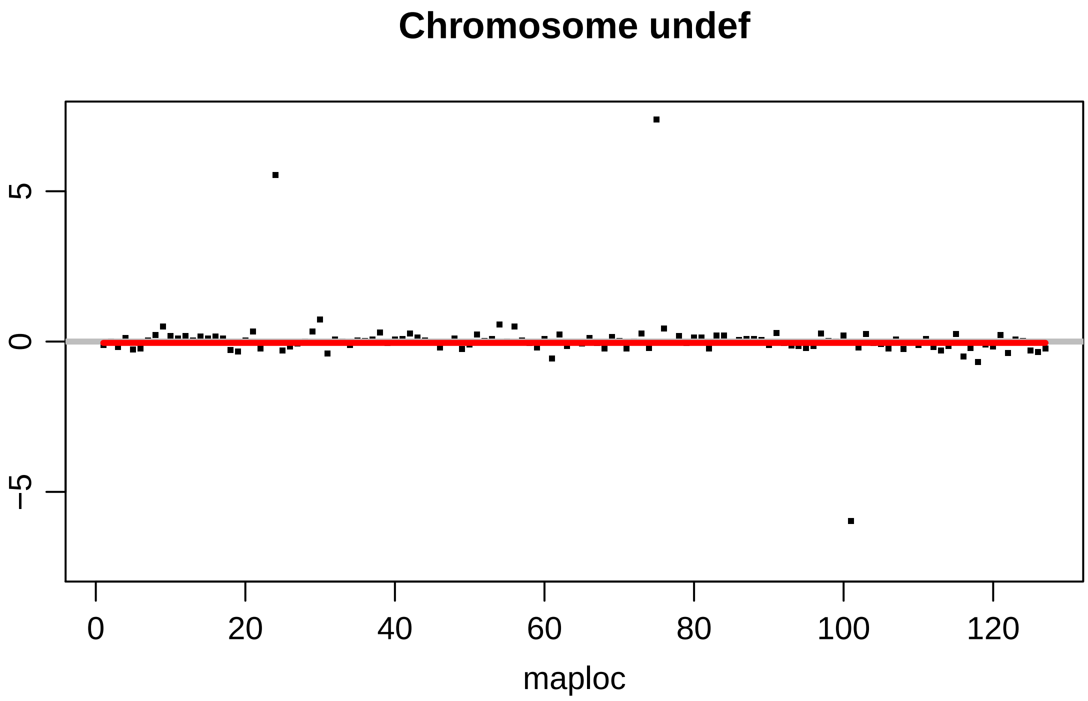


```
## Segplot might not work because of special characters in the sample names. Use only A-Z,a-z and 0-9!
##  There is a hidden function cn.mops:::.replaceNames that replaces the names in the "CNVDetectionResul
```

onXpress_043_R_2012_09_12_07_50_54_Sequoia_SN1.26.Run_20_Run_20_hg19_

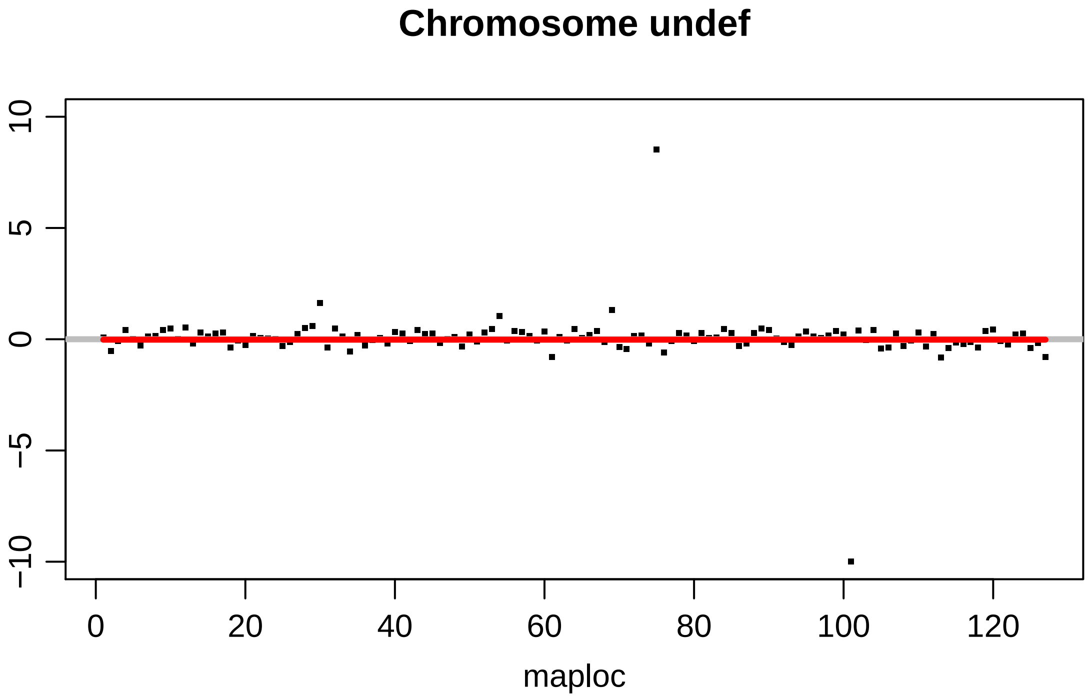

```
## Segplot might not work because of special characters in the sample names. Use only A-Z,a-z and 0-9!
##  There is a hidden function cn.mops:::.replaceNames that replaces the names in the "CNVDetectionResul
```

onXpress_044_R_2012_09_12_07_50_54_Sequoia_SN1.26.Run_20_Run_20_hg19_

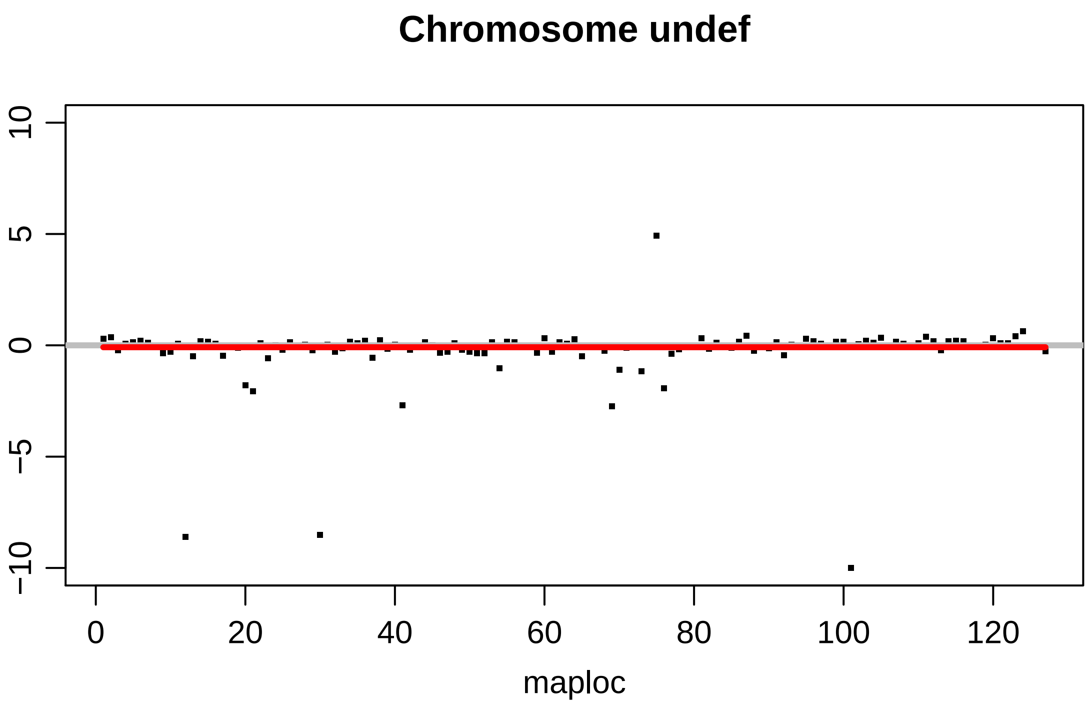


```
## Segplot might not work because of special characters in the sample names. Use only A-Z,a-z and 0-9!
##  There is a hidden function cn.mops:::.replaceNames that replaces the names in the "CNVDetectionResul
```

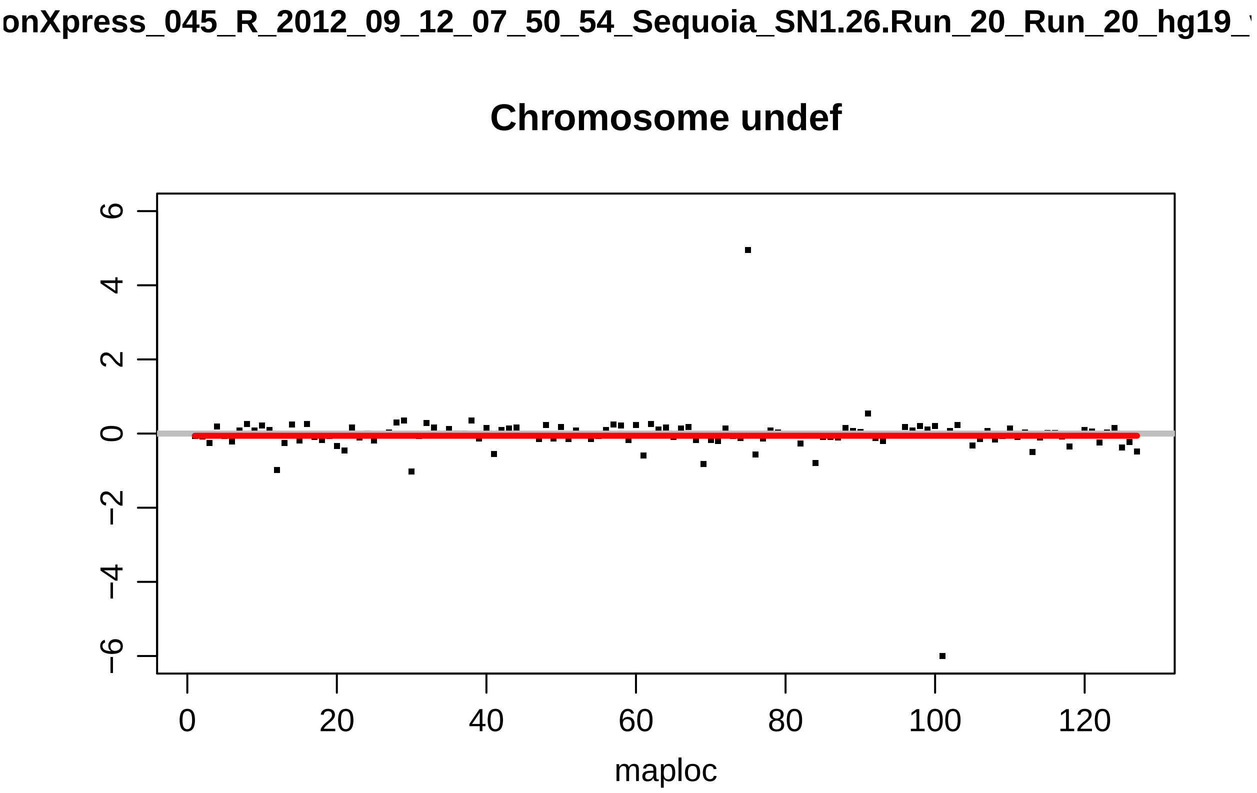


```
## Segplot might not work because of special characters in the sample names. Use only A-Z,a-z and 0-9!
##  There is a hidden function cn.mops:::.replaceNames that replaces the names in the "CNVDetectionResul
```

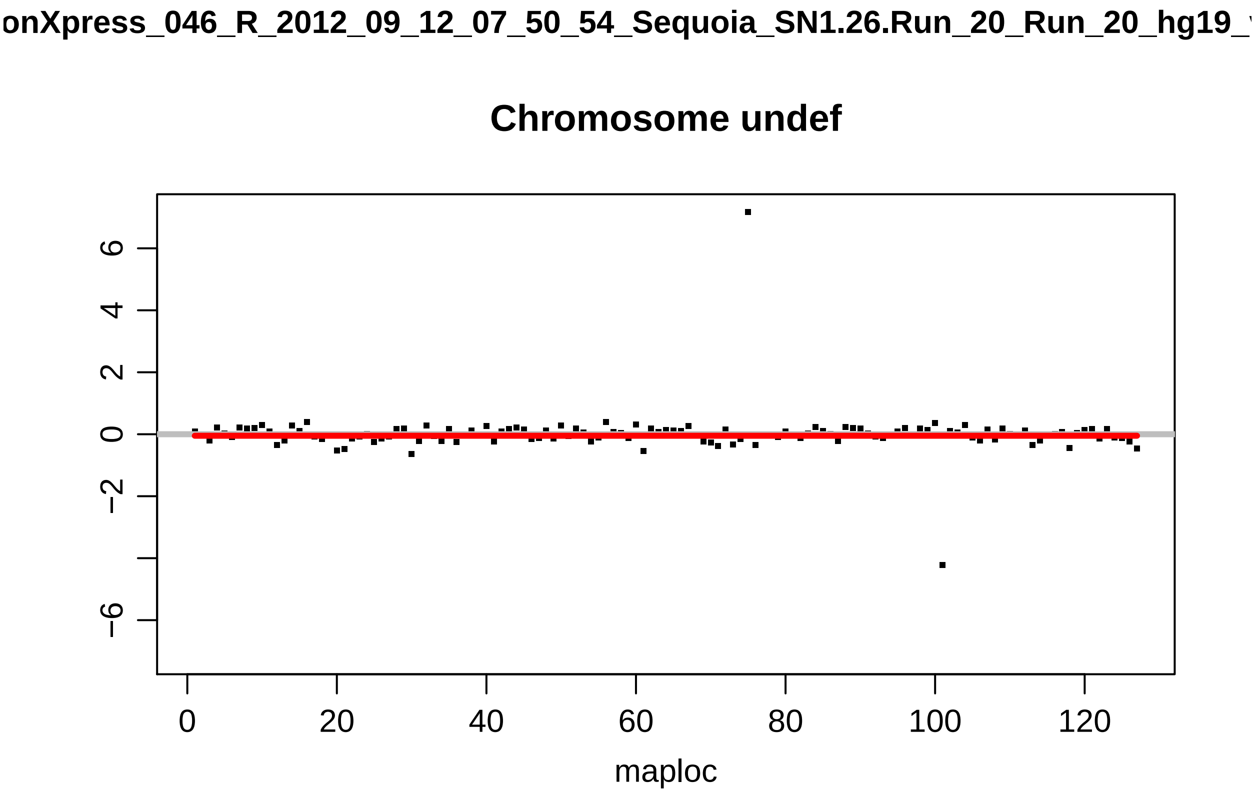

```
## Segplot might not work because of special characters in the sample names. Use only A-Z,a-z and 0-9!
##  There is a hidden function cn.mops:::.replaceNames that replaces the names in the "CNVDetectionResul
```

onXpress_047_R_2012_09_12_07_50_54_Sequoia_SN1.26.Run_20_Run_20_hg19_

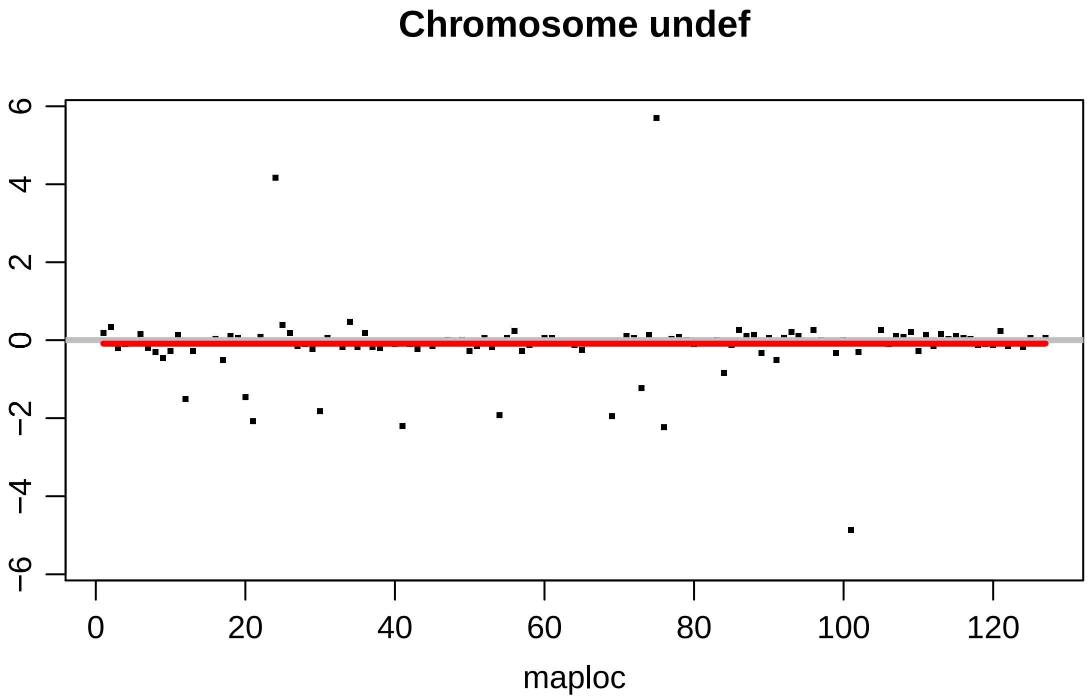


```
## [1] "/Users/gdemidov/Downloads/doc/Run_first_run_fin_05_qc.xls"

## Normalizing...

## Starting local modeling, please be patient...

## Reference sequence:  undef

## Starting segmentation algorithm...

## Using "fastseg" for segmentation.

## [1] ""
## [1] "/Users/gdemidov/Downloads/doc/Run_first_run_fin_05_qc.xls"
## [1] ""

## Segplot might not work because of special characters in the sample names. Use only A-Z,a-z and 0-9!
##  There is a hidden function cn.mops:::.replaceNames that replaces the names in the "CNVDetectionResul
```

onXpress_048_R_2012_09_12_07_50_54_Sequoia_SN1.26.Run_20_Run_20_hg19_

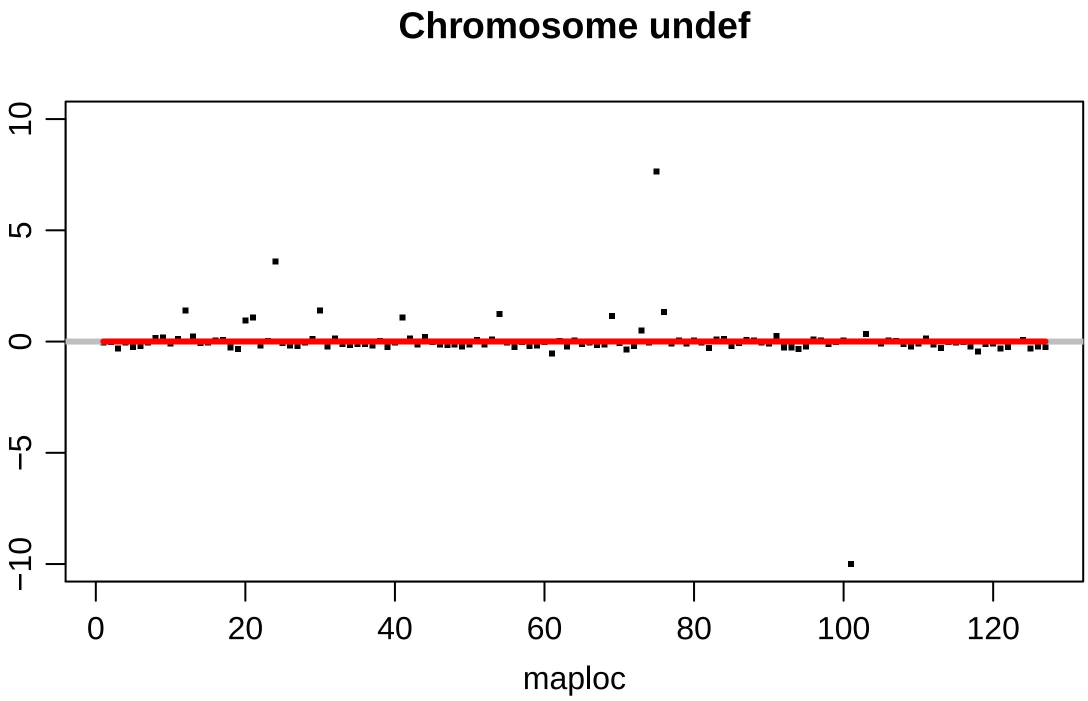


```
## Segplot might not work because of special characters in the sample names. Use only A-Z,a-z and 0-9!
##  There is a hidden function cn.mops:::.replaceNames that replaces the names in the "CNVDetectionResul
```

Case_IP001.G1.sam

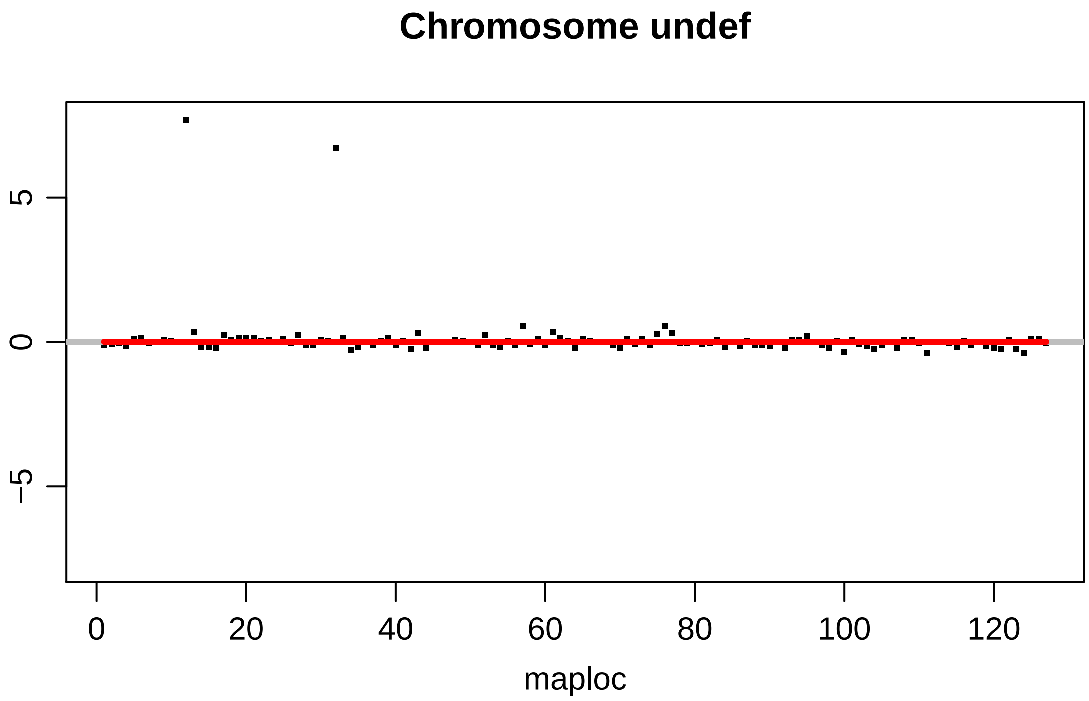

```
## Segplot might not work because of special characters in the sample names. Use only A-Z,a-z and 0-9!
##  There is a hidden function cn.mops:::.replaceNames that replaces the names in the "CNVDetectionResul
```

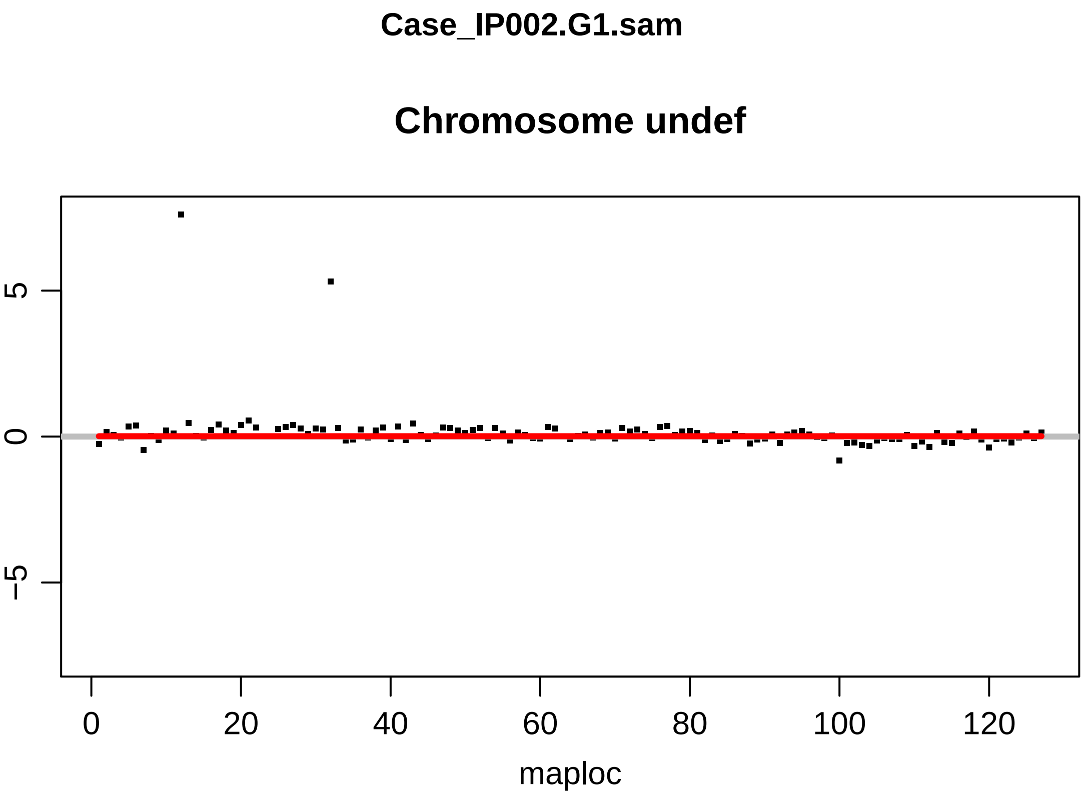


```
## Segplot might not work because of special characters in the sample names. Use only A-Z,a-z and 0-9!
##  There is a hidden function cn.mops:::.replaceNames that replaces the names in the "CNVDetectionResul
```

Case_IP003.G1.sam

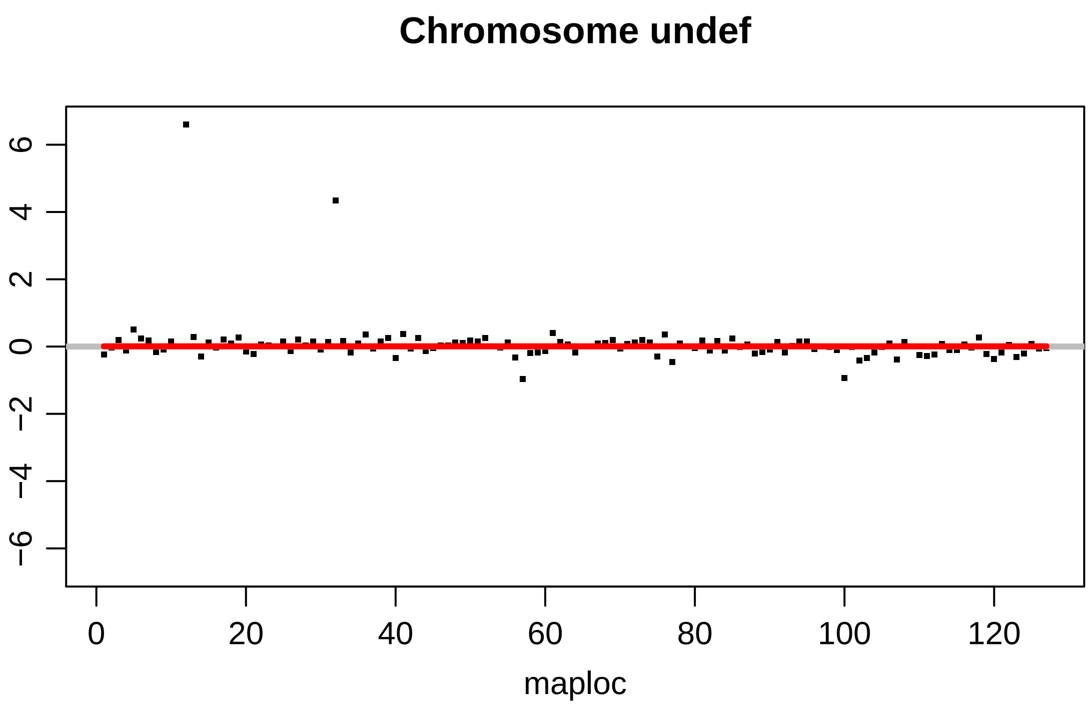


```
## Segplot might not work because of special characters in the sample names. Use only A-Z,a-z and 0-9!
##  There is a hidden function cn.mops:::.replaceNames that replaces the names in the "CNVDetectionResul
```

Case_IP004.G1.sam

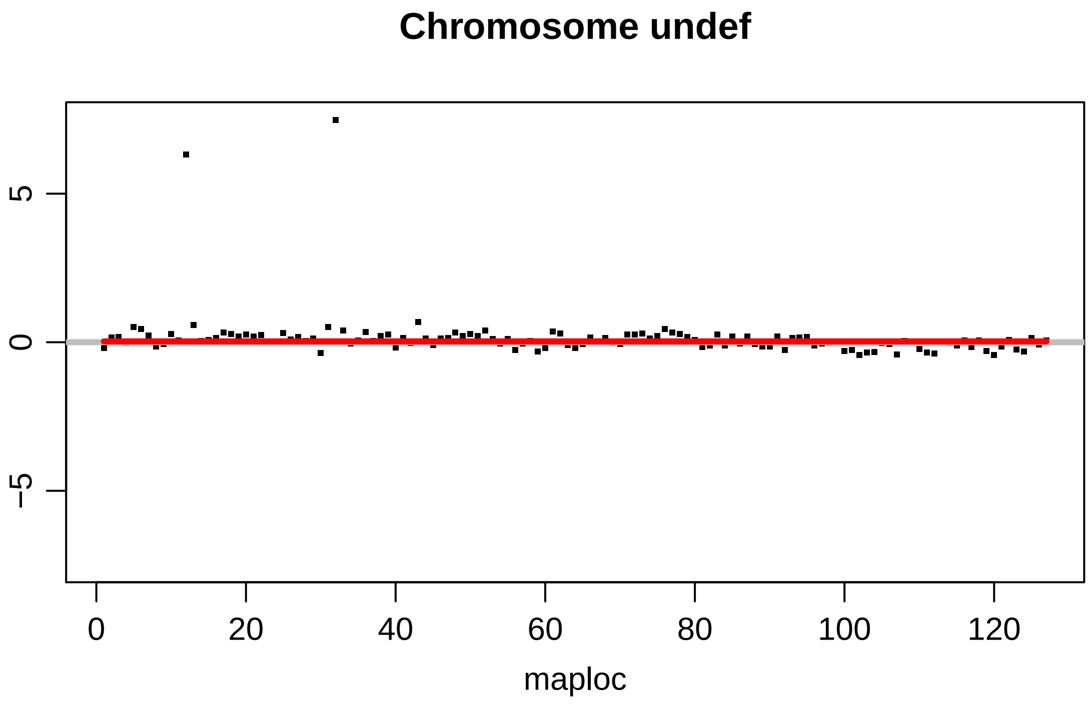

```
## Segplot might not work because of special characters in the sample names. Use only A-Z,a-z and 0-9!
##  There is a hidden function cn.mops:::.replaceNames that replaces the names in the "CNVDetectionResul
```

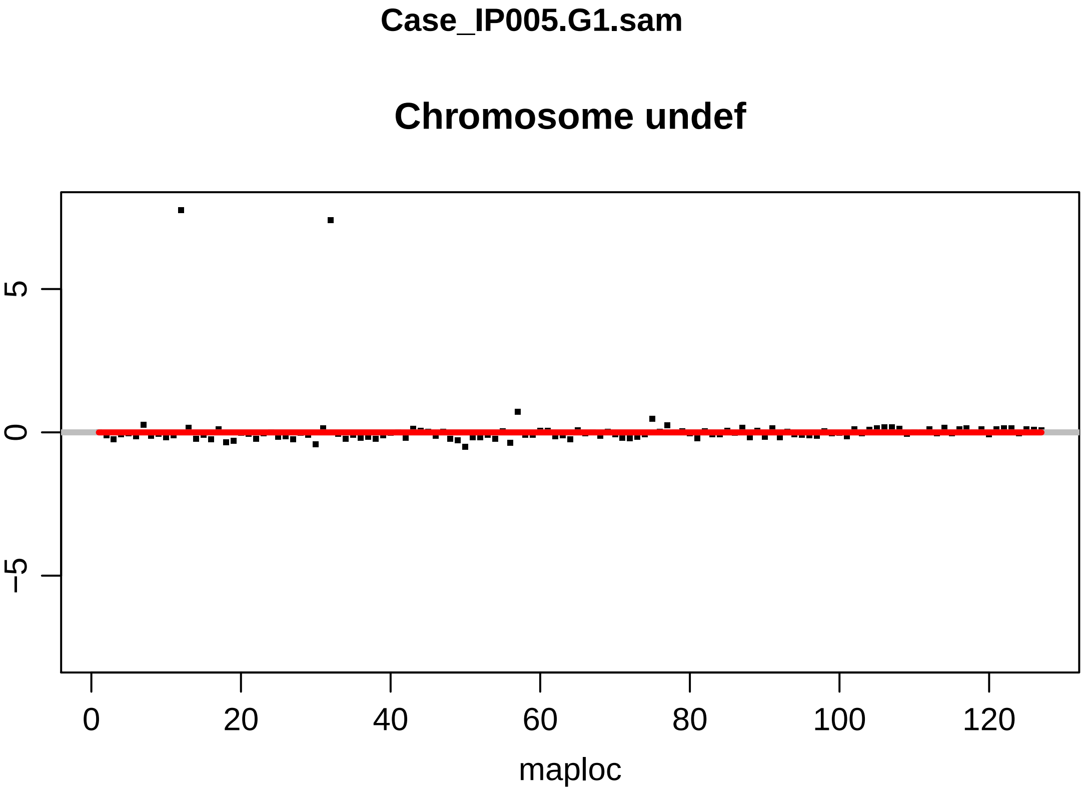


```
## Segplot might not work because of special characters in the sample names. Use only A-Z,a-z and 0-9!
##  There is a hidden function cn.mops:::.replaceNames that replaces the names in the "CNVDetectionResul
```

Case_IP006.G1.sam

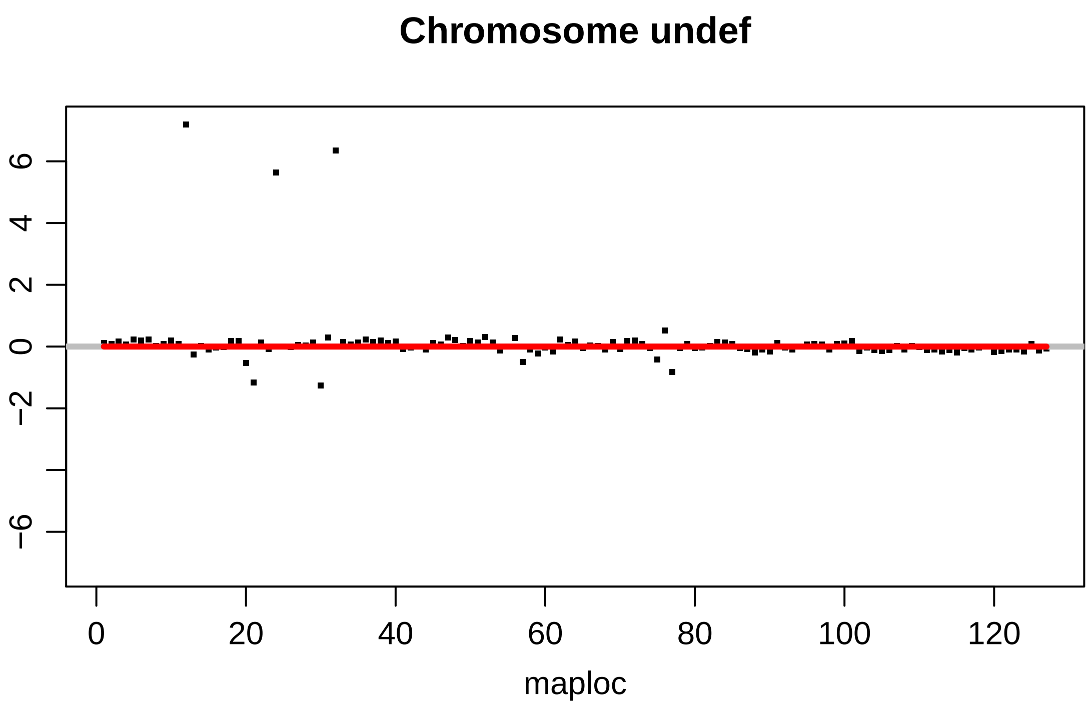


```
## Segplot might not work because of special characters in the sample names. Use only A-Z,a-z and 0-9!
##  There is a hidden function cn.mops:::.replaceNames that replaces the names in the "CNVDetectionResul
```

Case_IP007.G1.sam

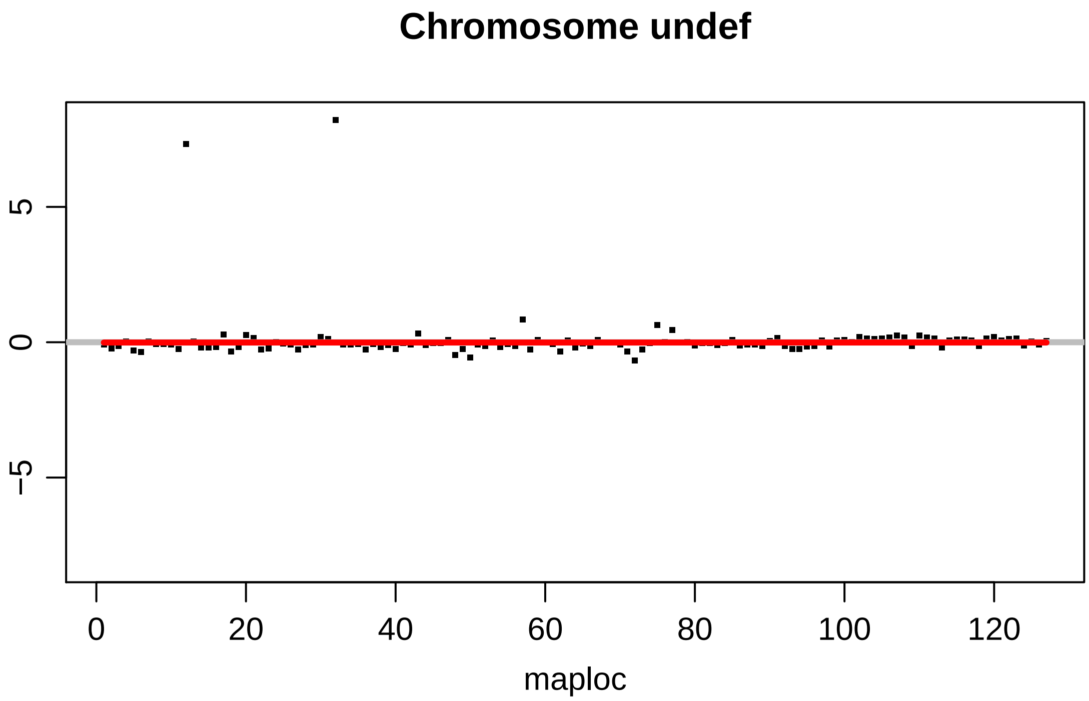

```
## Segplot might not work because of special characters in the sample names. Use only A-Z,a-z and 0-9!
##  There is a hidden function cn.mops:::.replaceNames that replaces the names in the "CNVDetectionResul
```

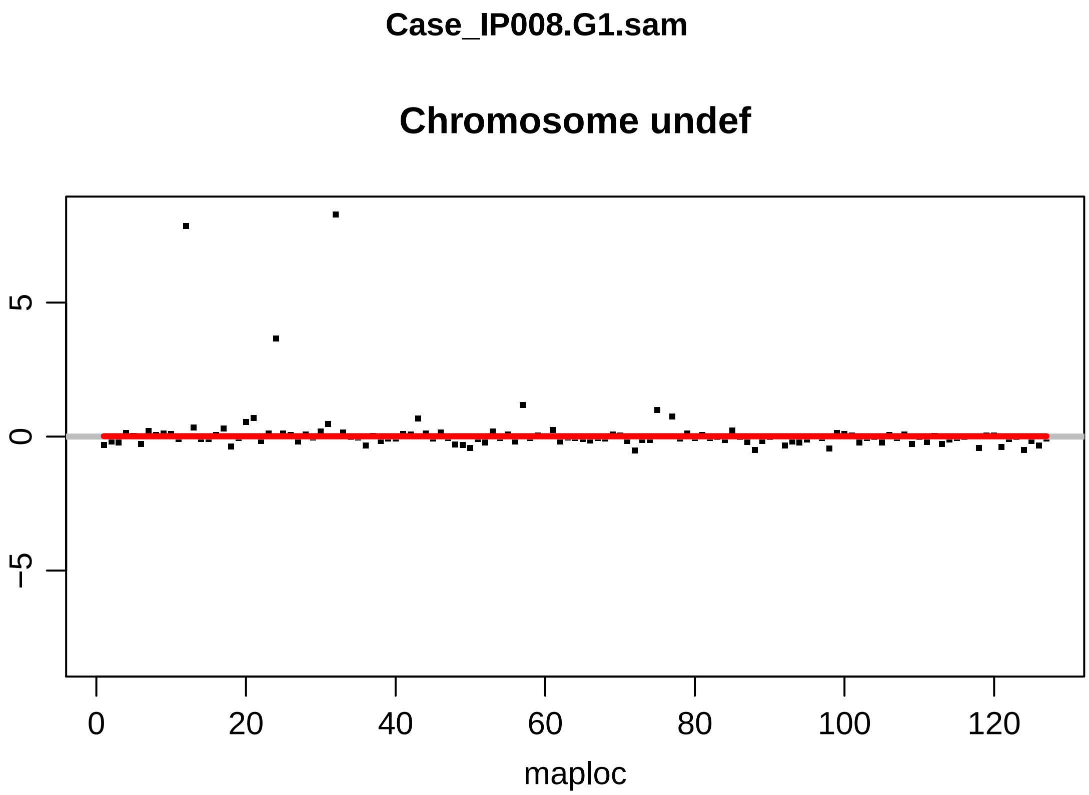


```
## Segplot might not work because of special characters in the sample names. Use only A-Z,a-z and 0-9!
##  There is a hidden function cn.mops:::.replaceNames that replaces the names in the "CNVDetectionResul
```

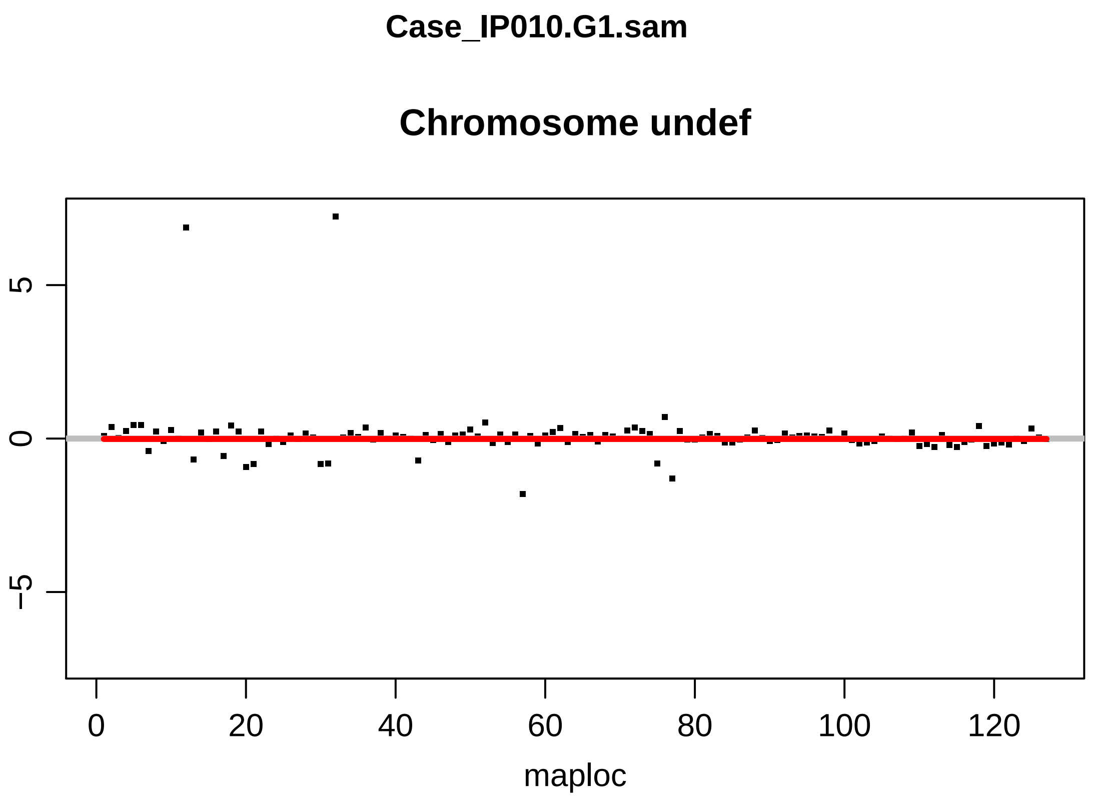


```
## Segplot might not work because of special characters in the sample names. Use only A-Z,a-z and 0-9!
##  There is a hidden function cn.mops:::.replaceNames that replaces the names in the "CNVDetectionResul
```

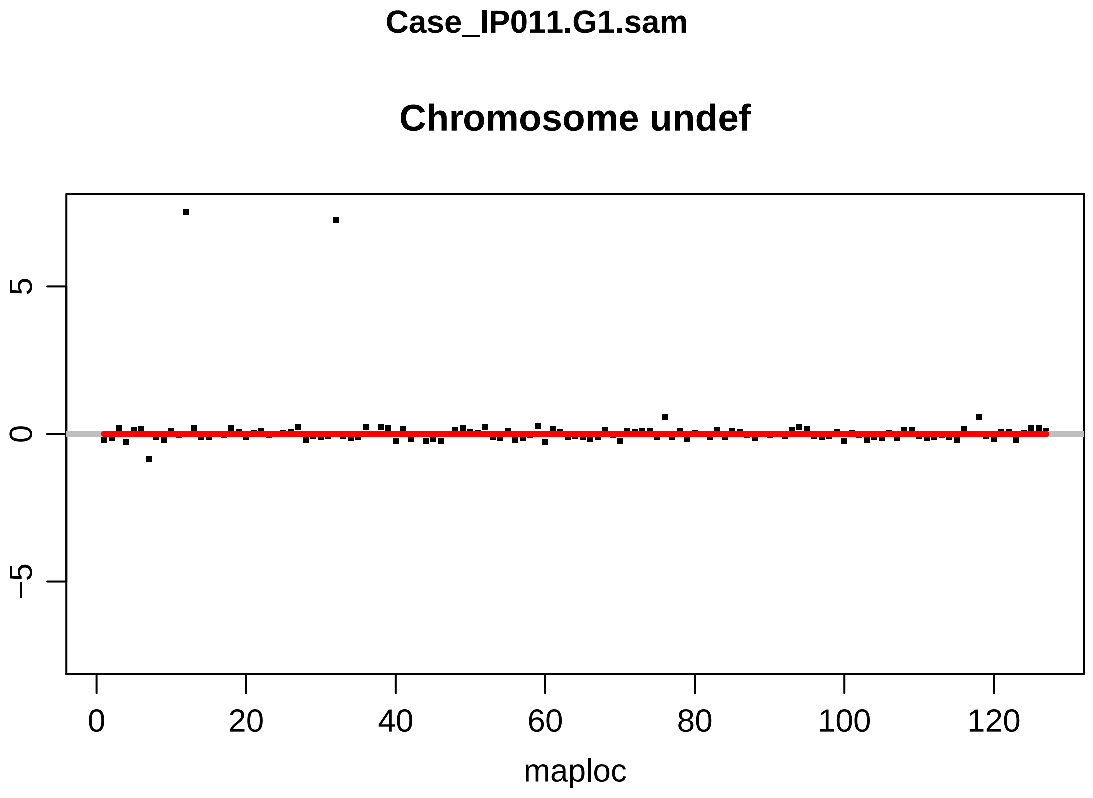

```
## Segplot might not work because of special characters in the sample names. Use only A-Z,a-z and 0-9!
##  There is a hidden function cn.mops:::.replaceNames that replaces the names in the "CNVDetectionResul
```

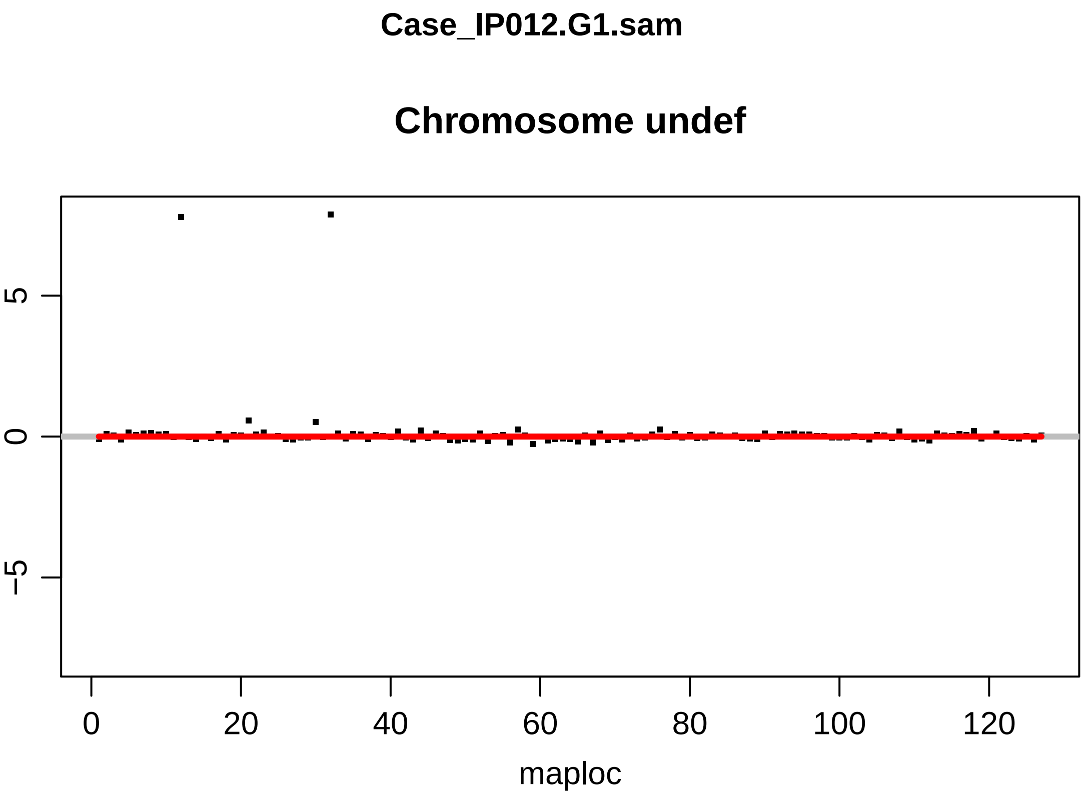


```
## Segplot might not work because of special characters in the sample names. Use only A-Z,a-z and 0-9!
##  There is a hidden function cn.mops:::.replaceNames that replaces the names in the "CNVDetectionResul
```

Case_IP013.G1.sam

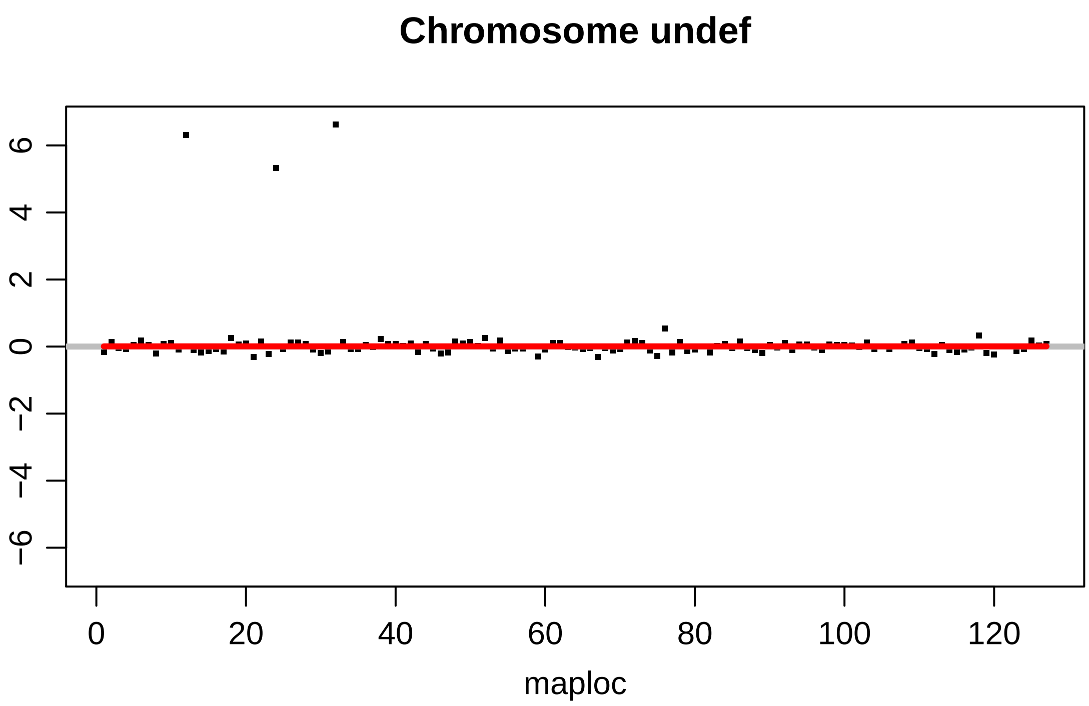


```
## Segplot might not work because of special characters in the sample names. Use only A-Z,a-z and 0-9!
##  There is a hidden function cn.mops:::.replaceNames that replaces the names in the "CNVDetectionResul
```

Case_IP014.G1.sam

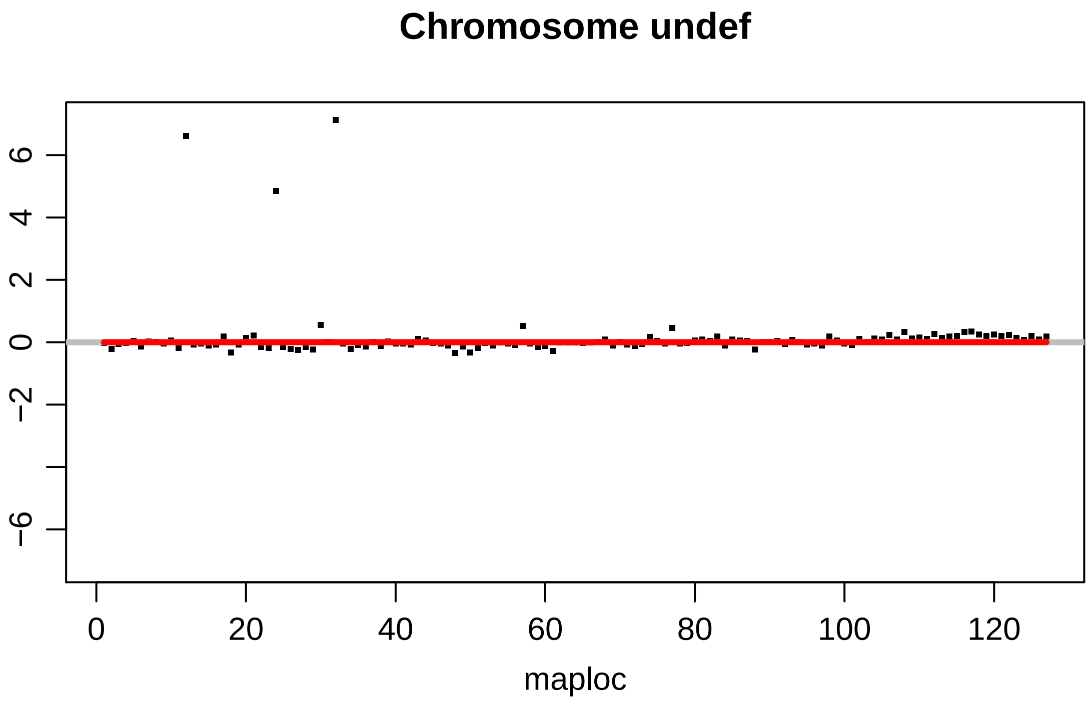

```
## Segplot might not work because of special characters in the sample names. Use only A-Z,a-z and 0-9!
##  There is a hidden function cn.mops:::.replaceNames that replaces the names in the "CNVDetectionResul
```

Case_IP015.G1.sam

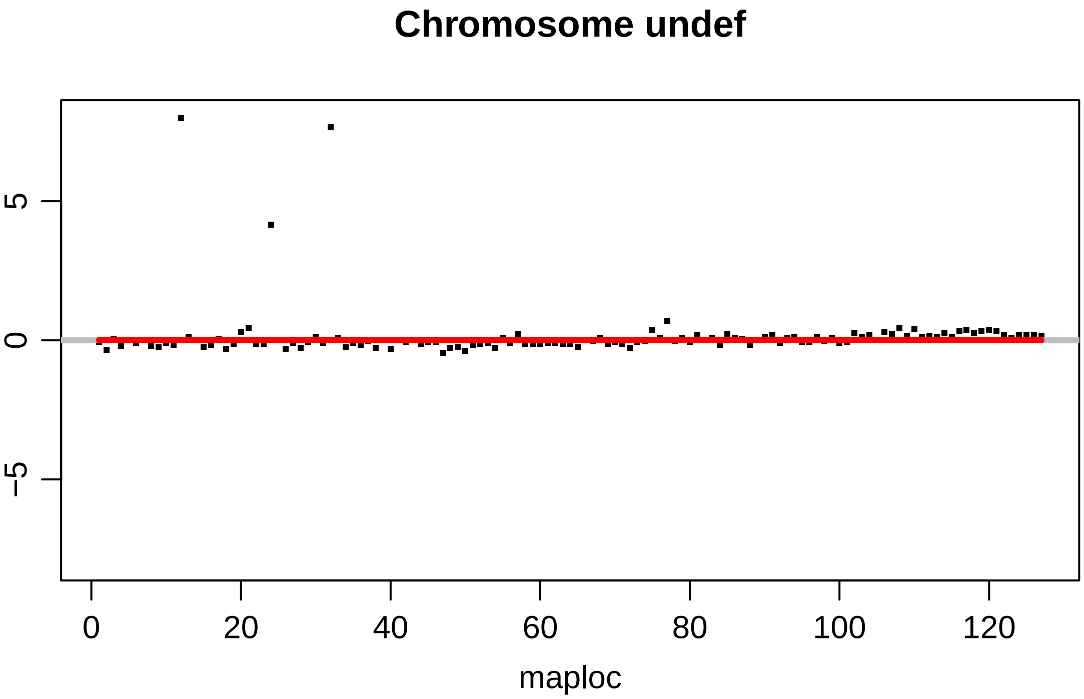


```
## Segplot might not work because of special characters in the sample names. Use only A-Z,a-z and 0-9!
##  There is a hidden function cn.mops:::.replaceNames that replaces the names in the "CNVDetectionResul
```

Case_IP016.G1.sam

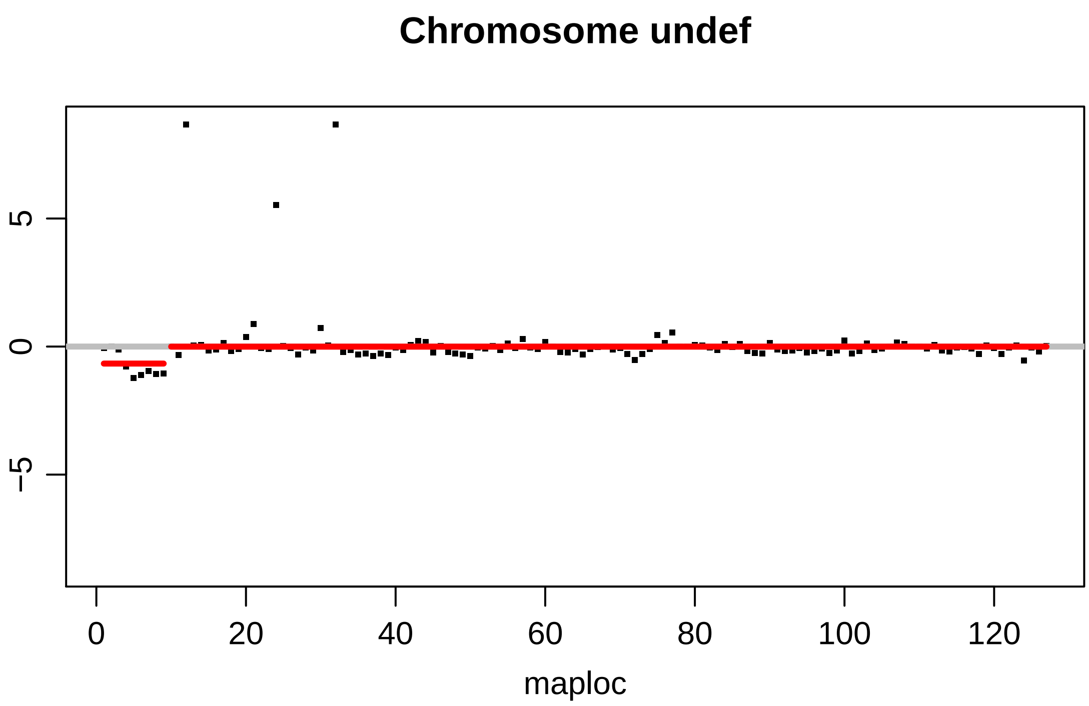


```
## Segplot might not work because of special characters in the sample names. Use only A-Z,a-z and 0-9!
##  There is a hidden function cn.mops:::.replaceNames that replaces the names in the "CNVDetectionResul
```

Case_IP017.G1.sam

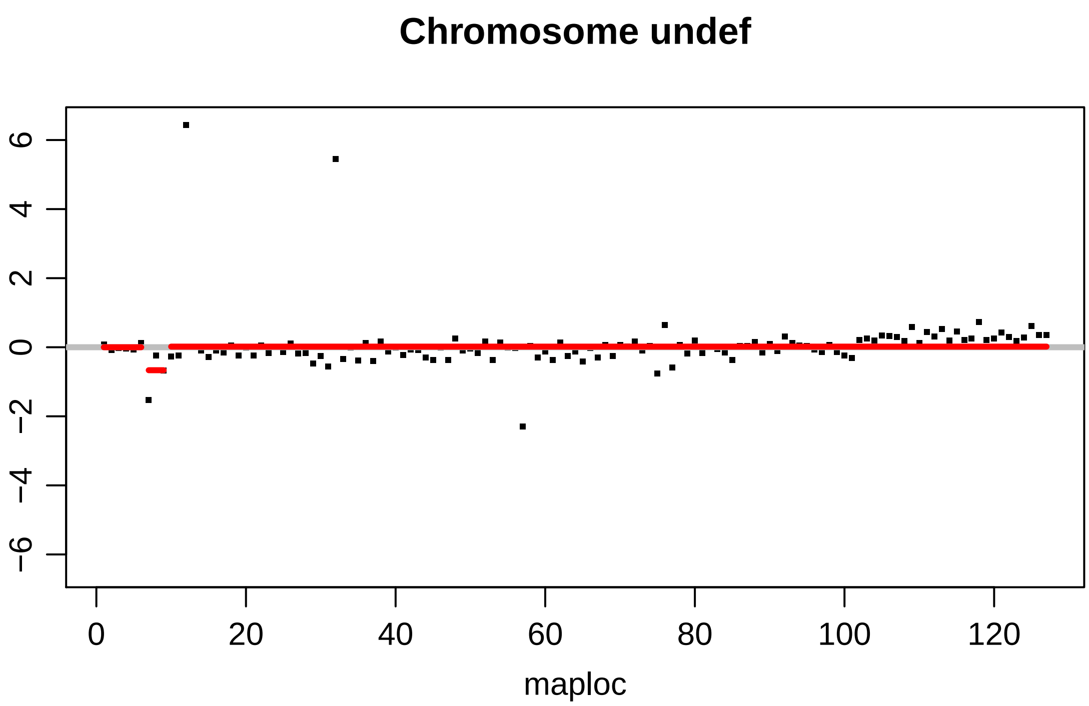

```
## Segplot might not work because of special characters in the sample names. Use only A-Z,a-z and 0-9!
##  There is a hidden function cn.mops:::.replaceNames that replaces the names in the "CNVDetectionResul
```

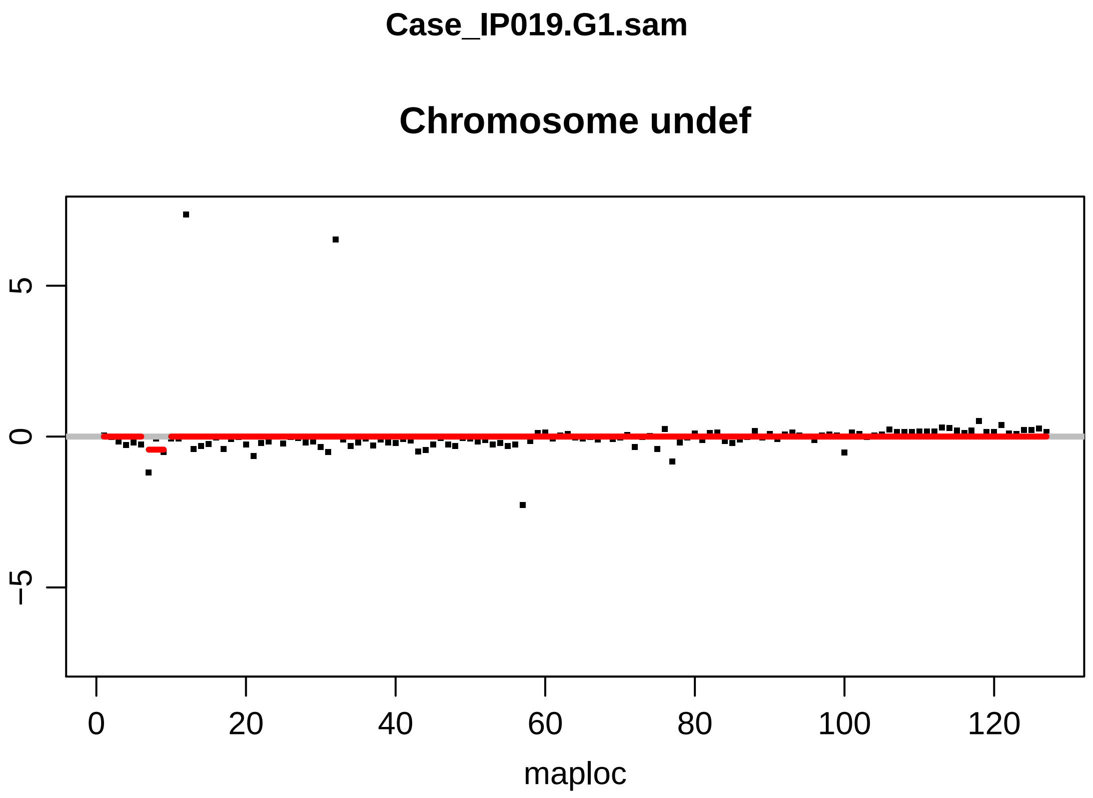


```
## Segplot might not work because of special characters in the sample names. Use only A-Z,a-z and 0-9!
##  There is a hidden function cn.mops:::.replaceNames that replaces the names in the "CNVDetectionResul
```

Case_IP020.G1.sam

Chromosome undef

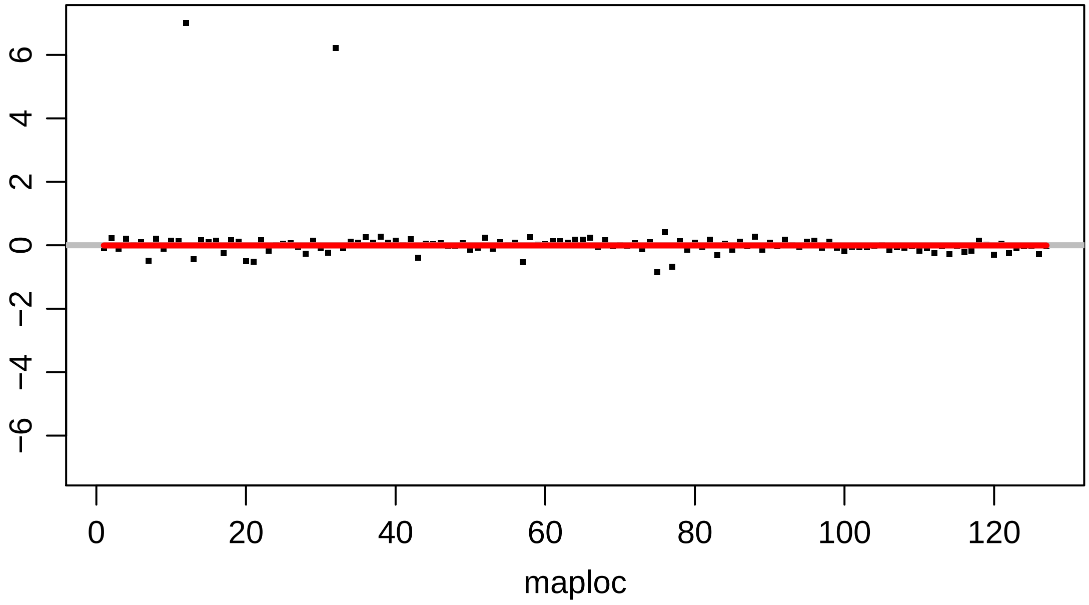


```
## Segplot might not work because of special characters in the sample names. Use only A-Z,a-z and 0-9!
##  There is a hidden function cn.mops:::.replaceNames that replaces the names in the "CNVDetectionResul
```

Case_IP022.G1.sam

Chromosome undef

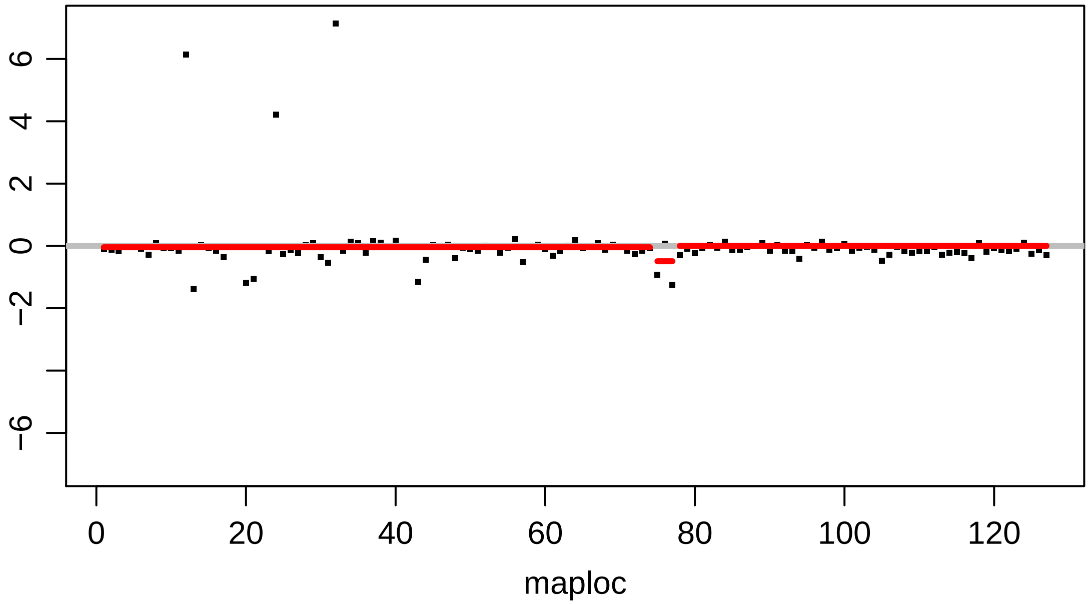

```
## Segplot might not work because of special characters in the sample names. Use only A-Z,a-z and 0-9!
##  There is a hidden function cn.mops:::.replaceNames that replaces the names in the "CNVDetectionResul
```

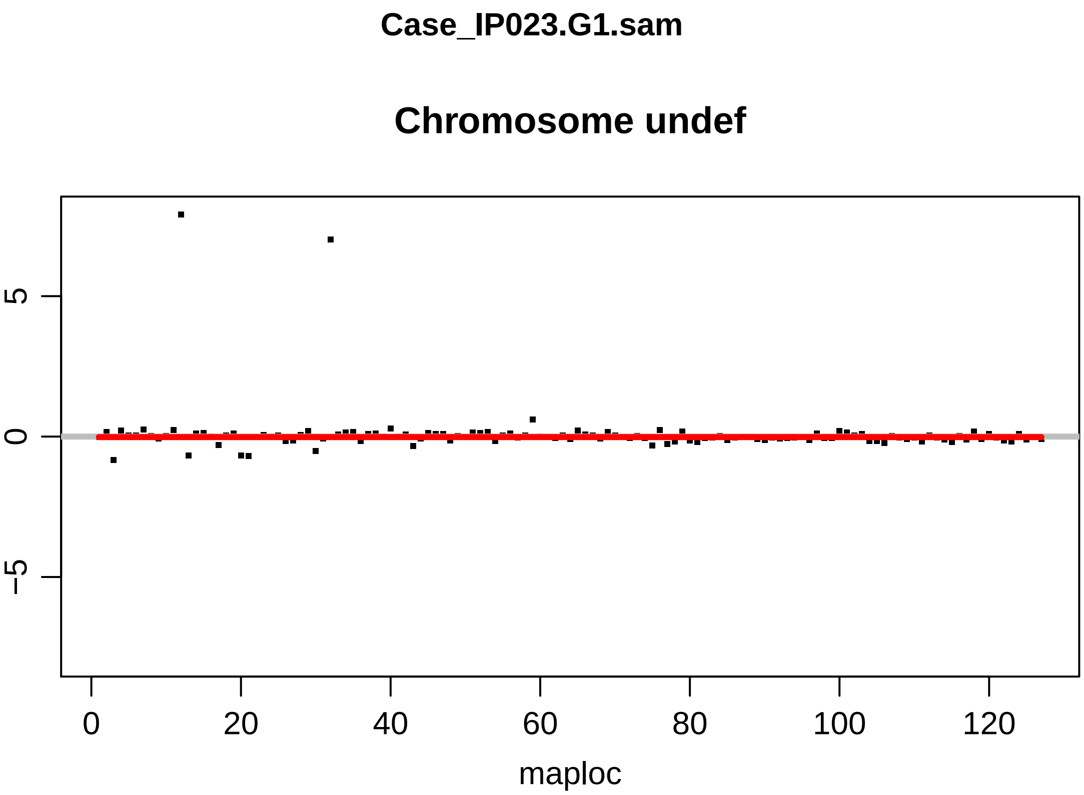


```
## Segplot might not work because of special characters in the sample names. Use only A-Z,a-z and 0-9!
##  There is a hidden function cn.mops:::.replaceNames that replaces the names in the "CNVDetectionResul
```

Case_IP024.G1.sam

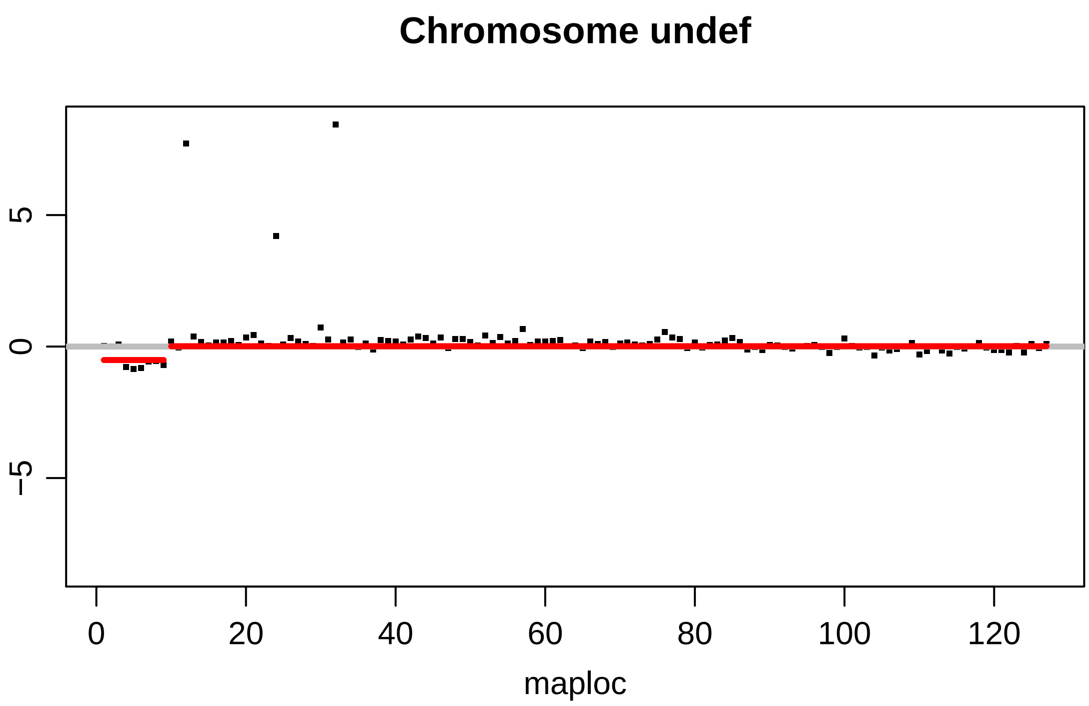


```
## Segplot might not work because of special characters in the sample names. Use only A-Z,a-z and 0-9!
##  There is a hidden function cn.mops:::.replaceNames that replaces the names in the "CNVDetectionResul
```

Case_IP025.G1.sam

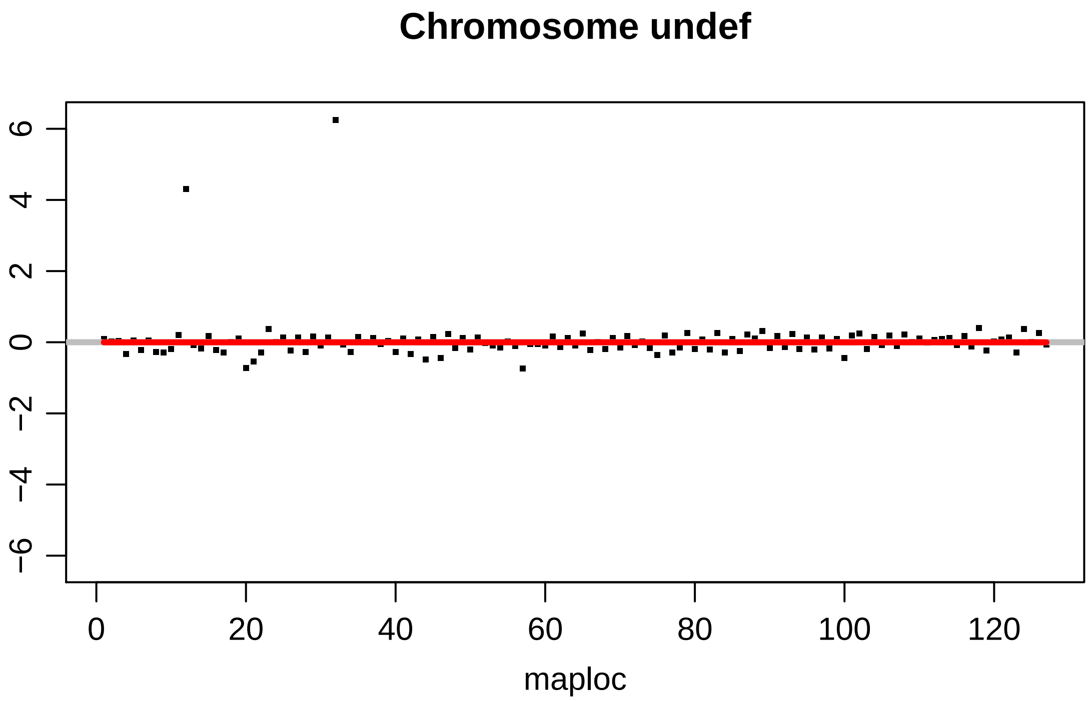

```
## Segplot might not work because of special characters in the sample names. Use only A-Z,a-z and 0-9!
##  There is a hidden function cn.mops:::.replaceNames that replaces the names in the "CNVDetectionResul
```

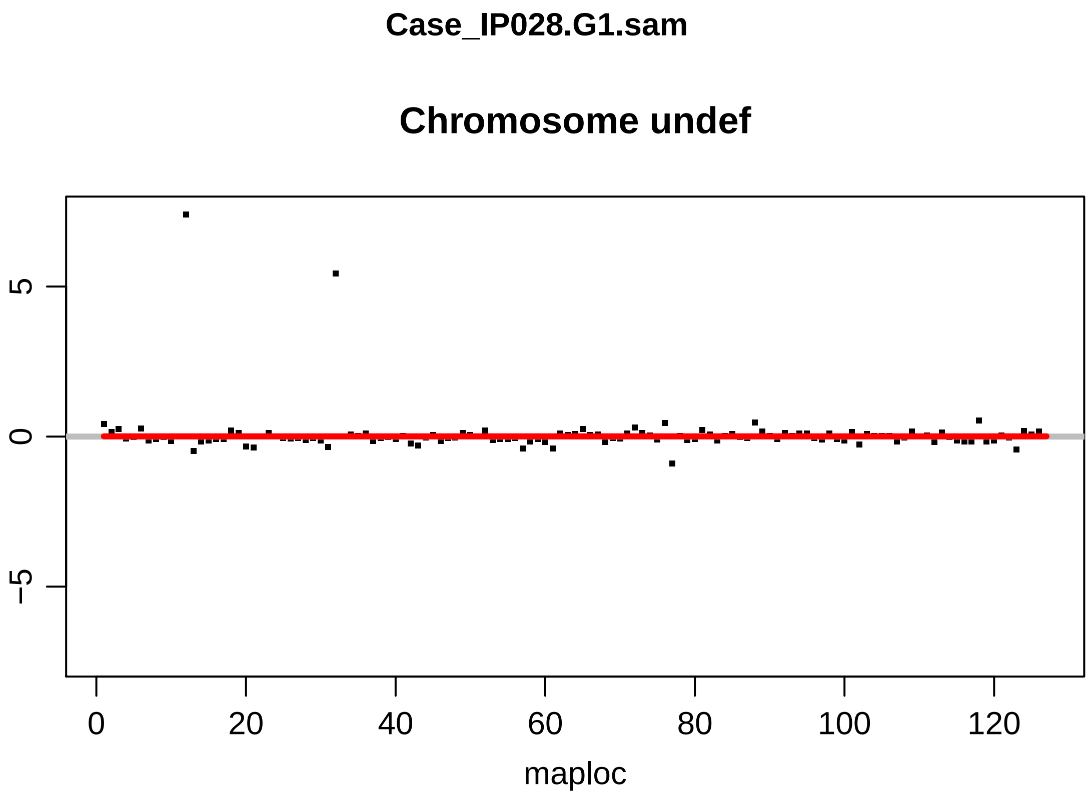


```
## Segplot might not work because of special characters in the sample names. Use only A-Z,a-z and 0-9!
##  There is a hidden function cn.mops:::.replaceNames that replaces the names in the "CNVDetectionResul
```

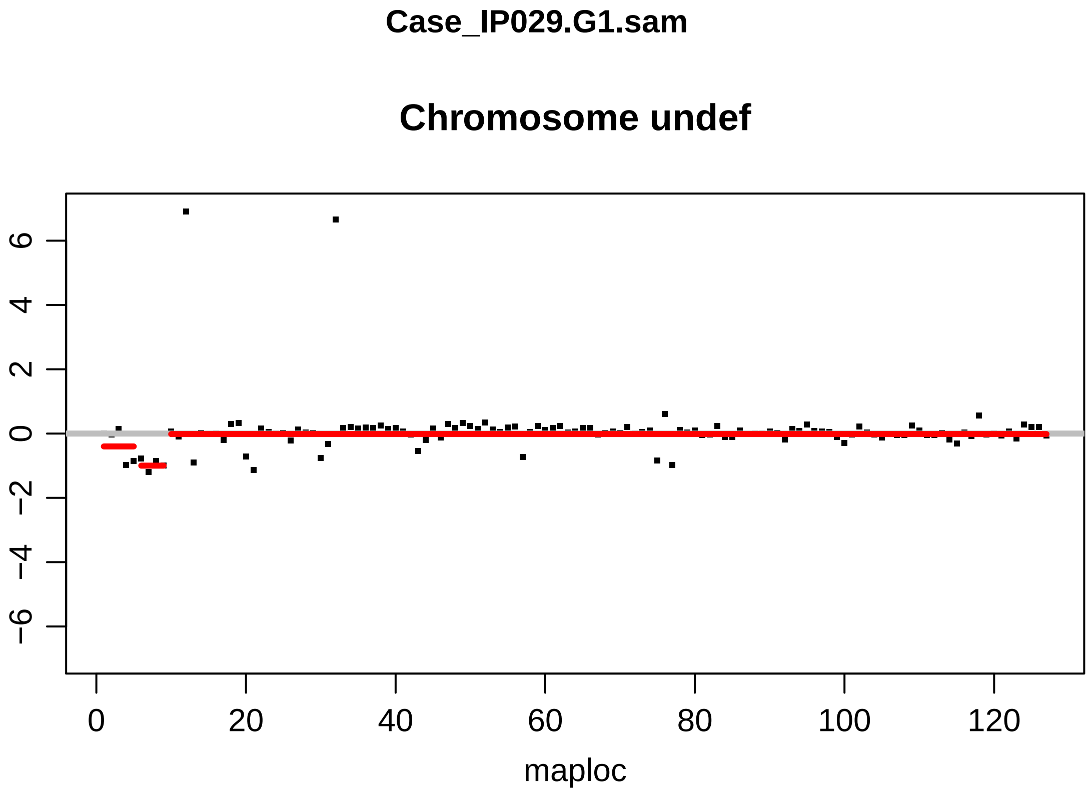


```
## Segplot might not work because of special characters in the sample names. Use only A-Z,a-z and 0-9!
##  There is a hidden function cn.mops:::.replaceNames that replaces the names in the "CNVDetectionResul
```

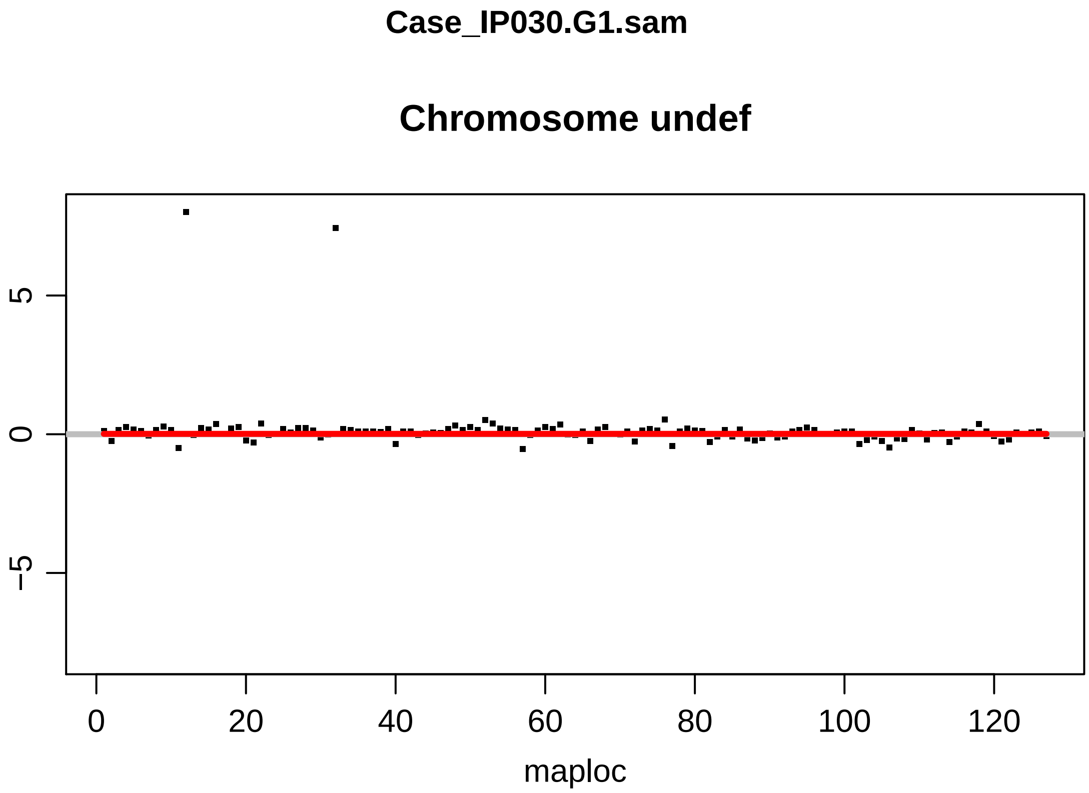

```
## Segplot might not work because of special characters in the sample names. Use only A-Z,a-z and 0-9!
##  There is a hidden function cn.mops:::.replaceNames that replaces the names in the "CNVDetectionResul
```

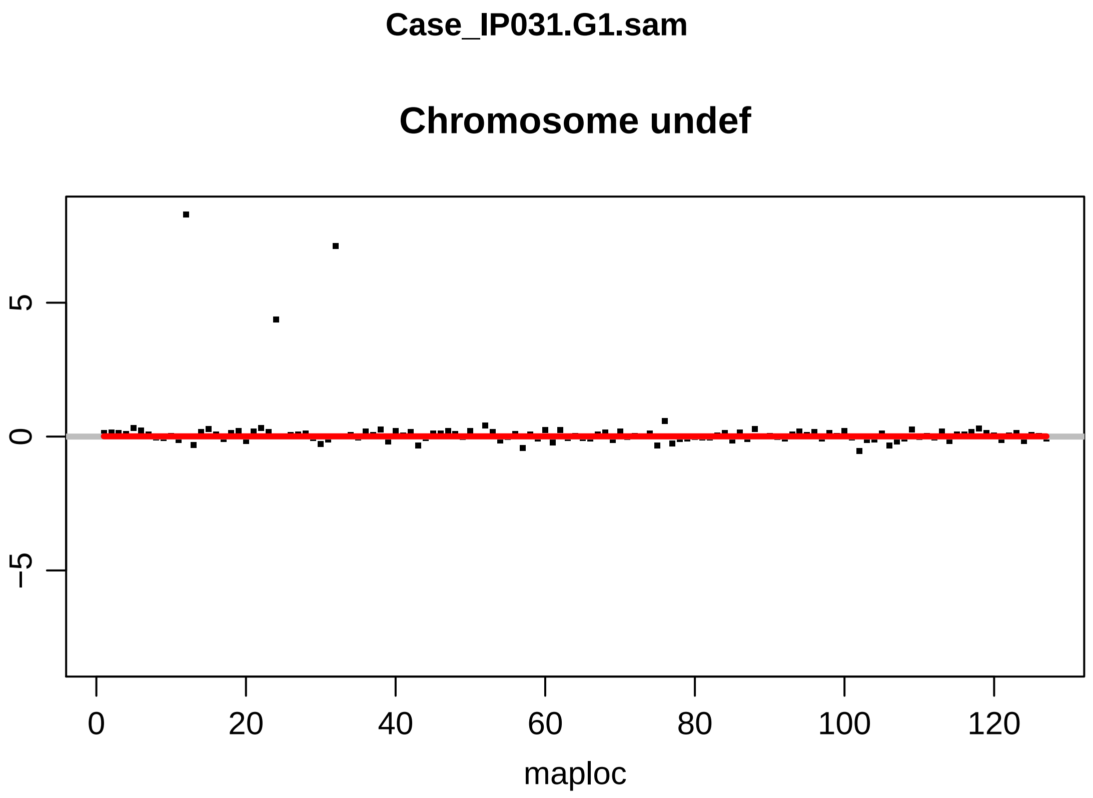


```
## Segplot might not work because of special characters in the sample names. Use only A-Z,a-z and 0-9!
##  There is a hidden function cn.mops:::.replaceNames that replaces the names in the "CNVDetectionResul
```

Case_IP032.G1.sam

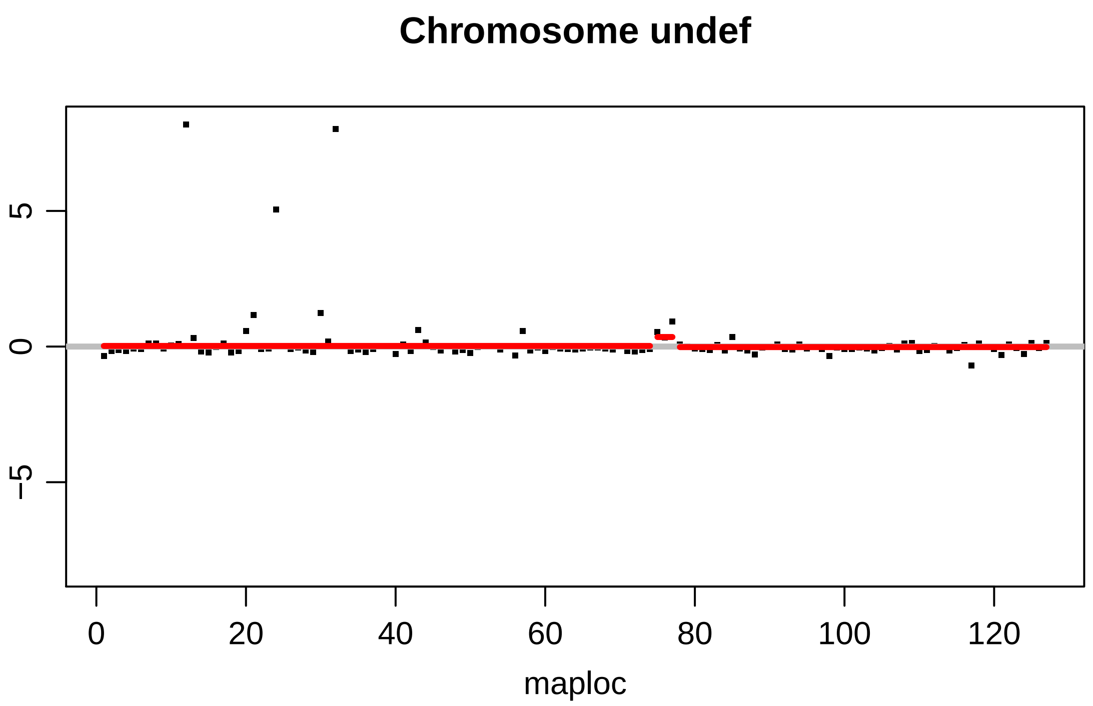


```
## Segplot might not work because of special characters in the sample names. Use only A-Z,a-z and 0-9!
##  There is a hidden function cn.mops:::.replaceNames that replaces the names in the "CNVDetectionResul
```

Case_IP033.G1.sam

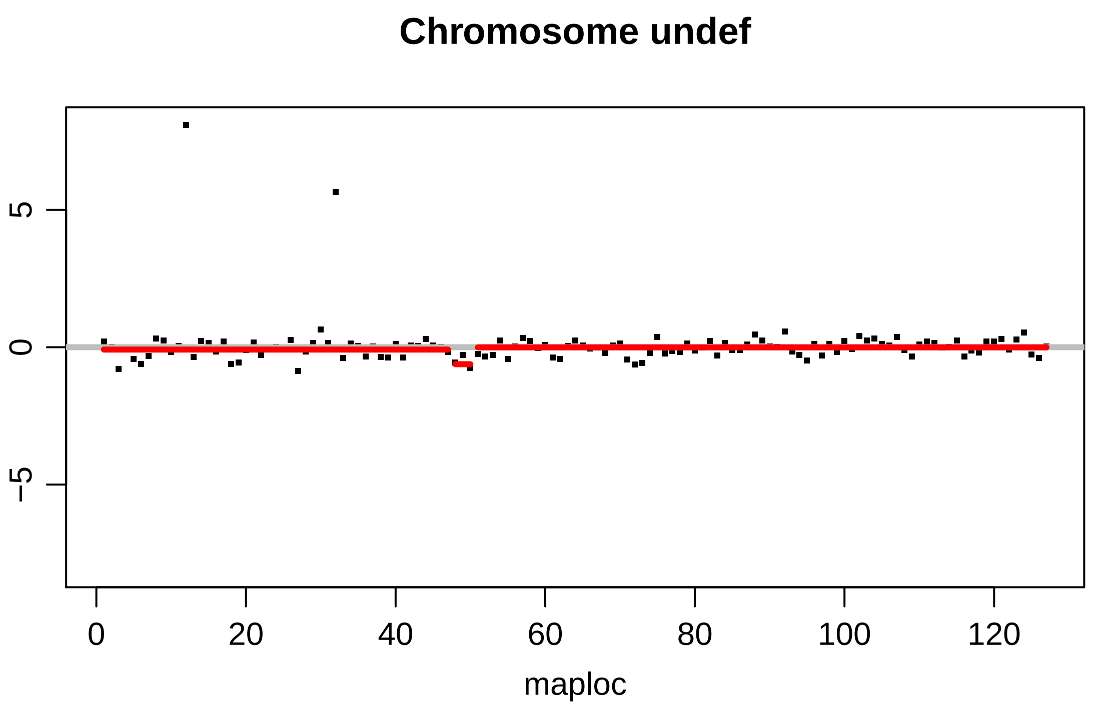

```
## Segplot might not work because of special characters in the sample names. Use only A-Z,a-z and 0-9!
##  There is a hidden function cn.mops:::.replaceNames that replaces the names in the "CNVDetectionResul
```

Case_IP035.G1.sam

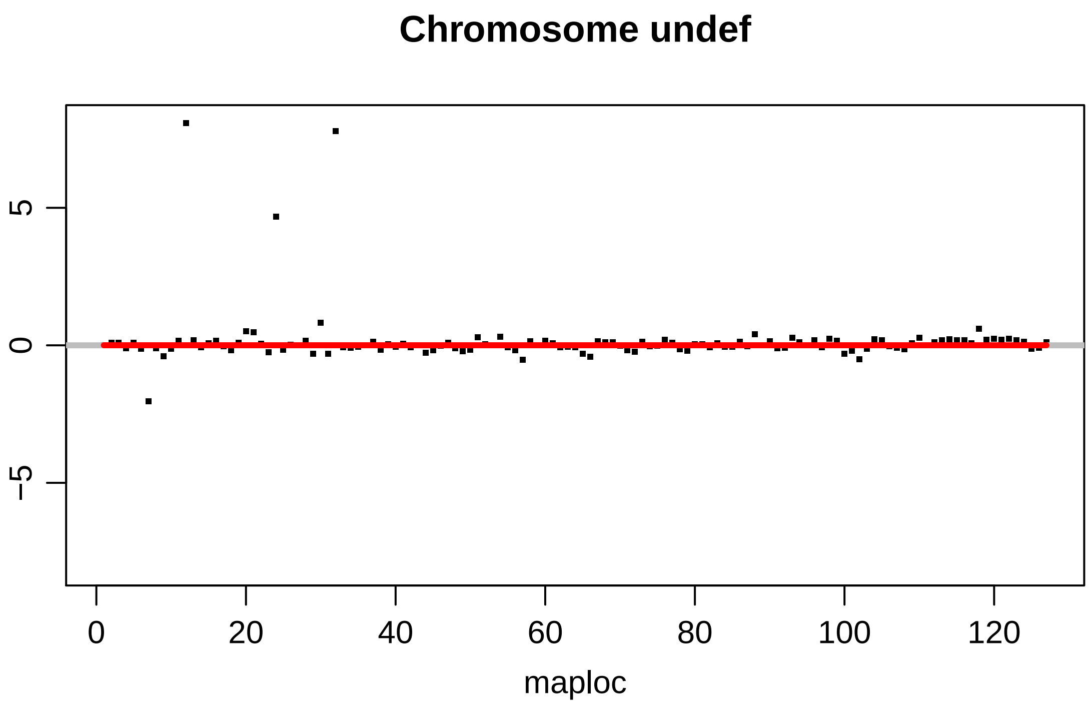


```
## Segplot might not work because of special characters in the sample names. Use only A-Z,a-z and 0-9!
##  There is a hidden function cn.mops:::.replaceNames that replaces the names in the "CNVDetectionResul
```

Case_IP036.G1.sam

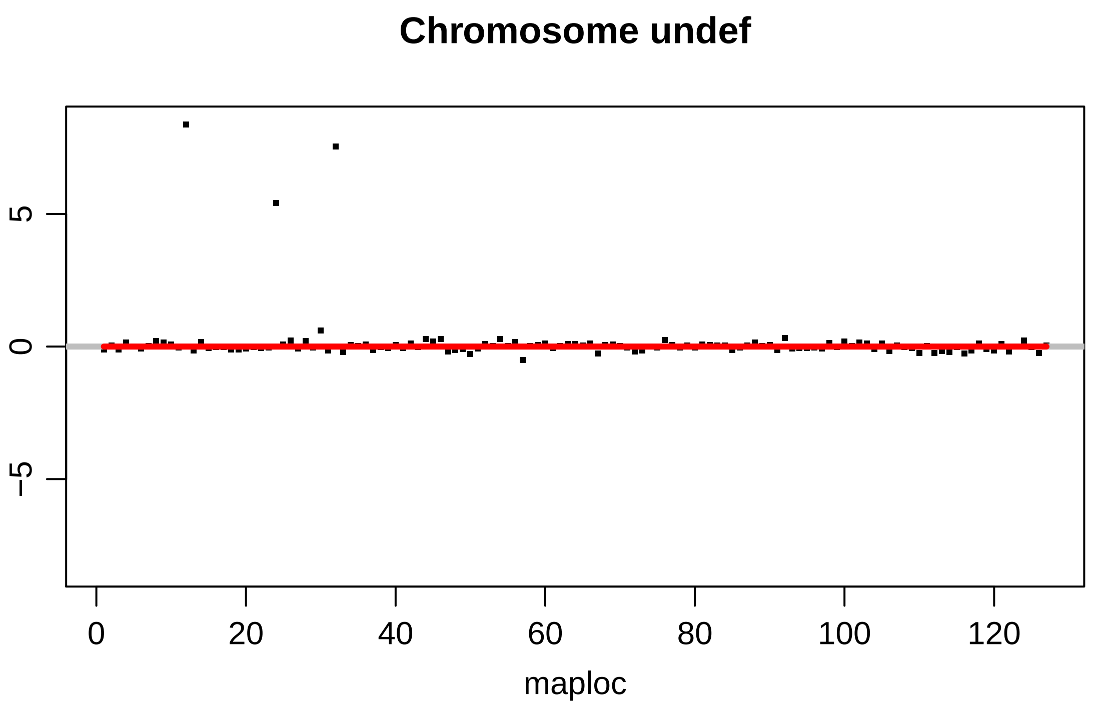


```
## Segplot might not work because of special characters in the sample names. Use only A-Z,a-z and 0-9!
##  There is a hidden function cn.mops:::.replaceNames that replaces the names in the "CNVDetectionResul
```

Case_IP037.G1.sam

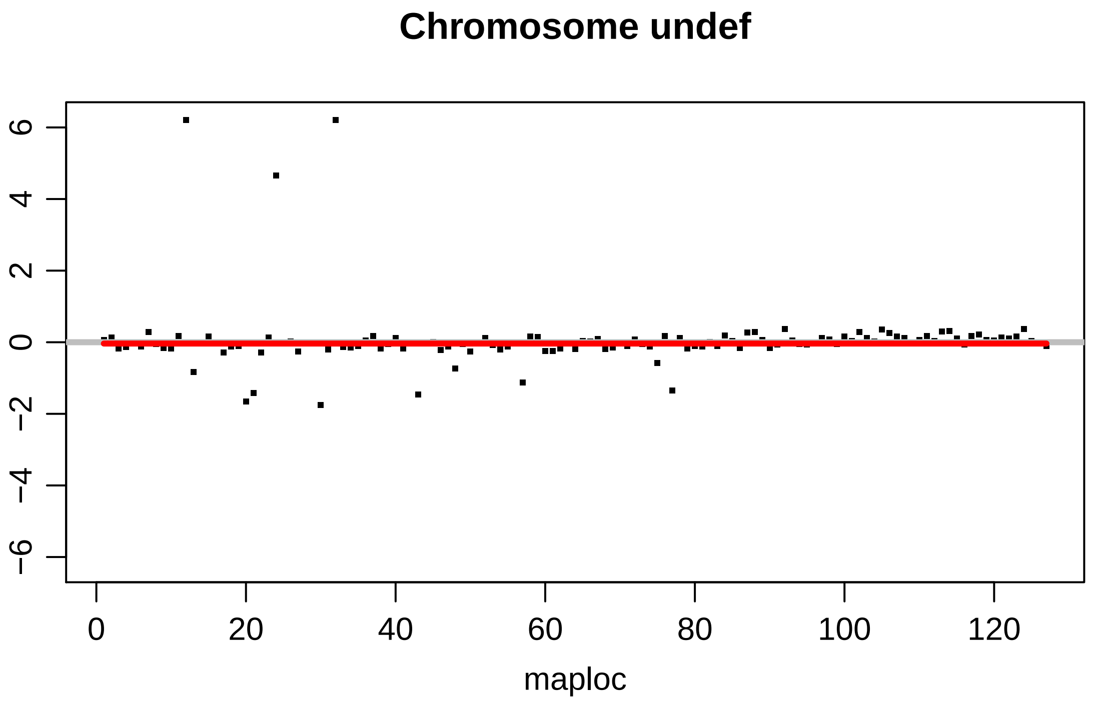

```
## Segplot might not work because of special characters in the sample names. Use only A-Z,a-z and 0-9!
##  There is a hidden function cn.mops:::.replaceNames that replaces the names in the "CNVDetectionResul
```

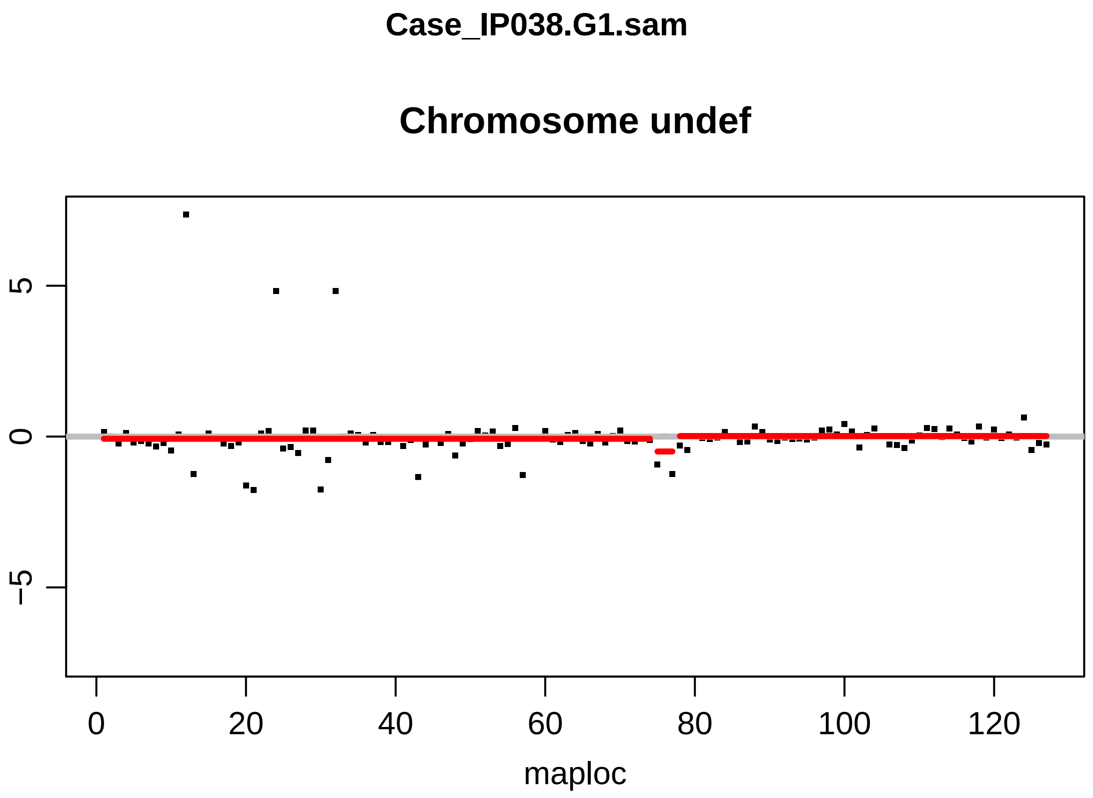


```
## Segplot might not work because of special characters in the sample names. Use only A-Z,a-z and 0-9!
##  There is a hidden function cn.mops:::.replaceNames that replaces the names in the "CNVDetectionResul
```

Case_IP039.G1.sam

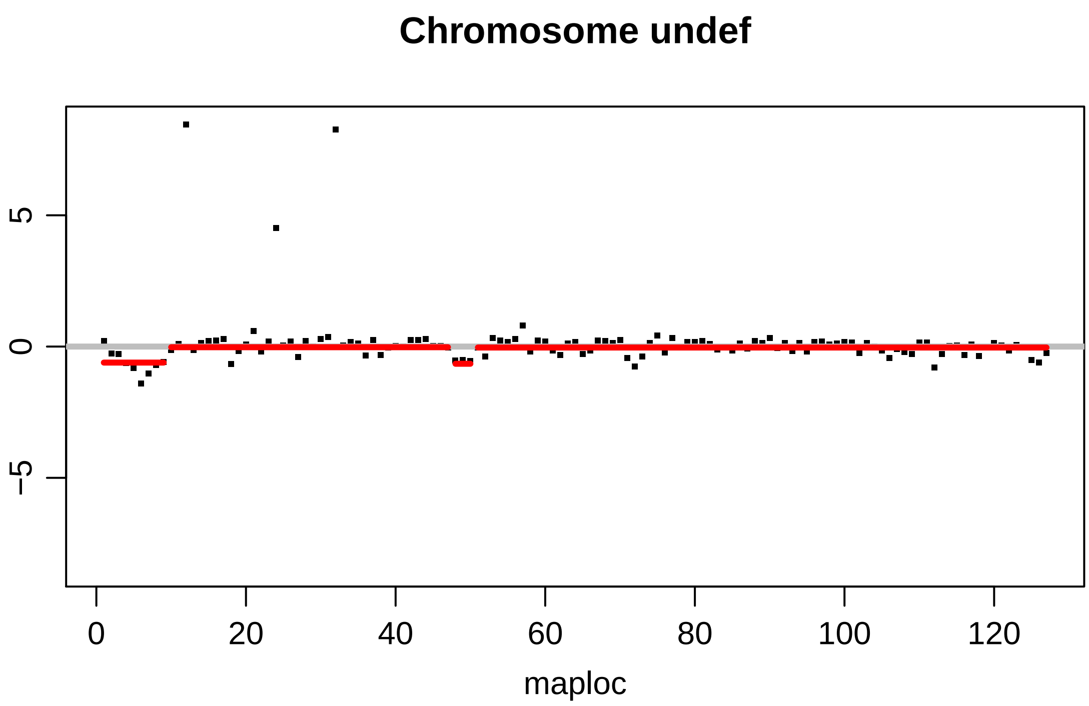


```
## Segplot might not work because of special characters in the sample names. Use only A-Z,a-z and 0-9!
##  There is a hidden function cn.mops:::.replaceNames that replaces the names in the "CNVDetectionResul
```

Case_IP040.G1.sam

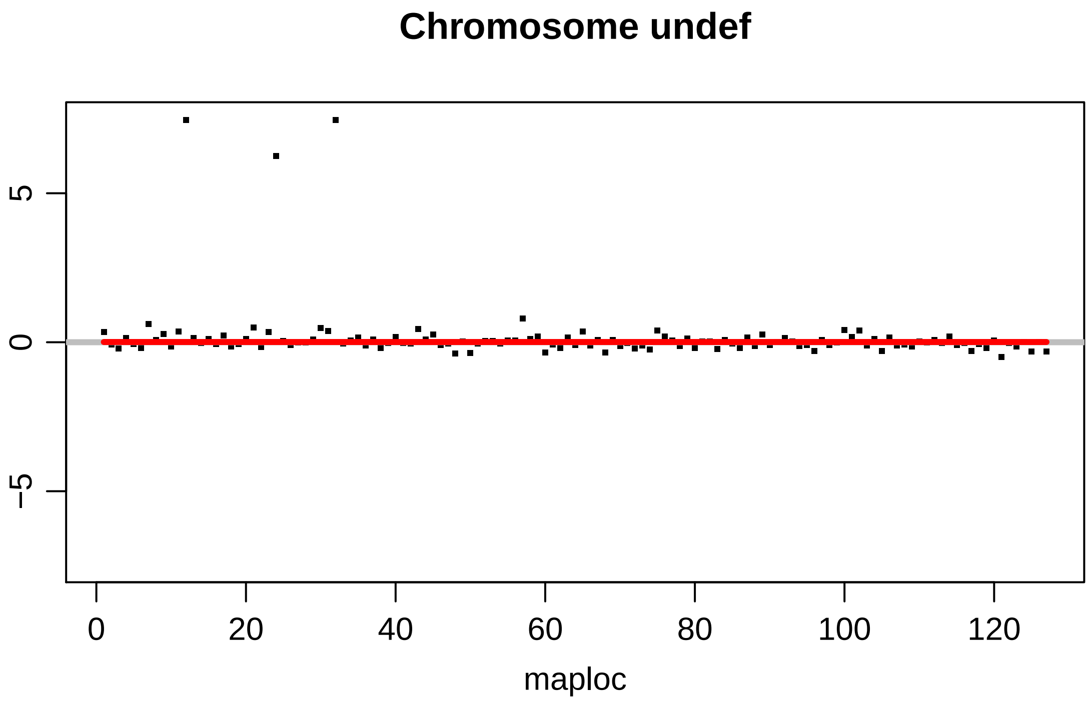

```
## Segplot might not work because of special characters in the sample names. Use only A-Z,a-z and 0-9!
##  There is a hidden function cn.mops:::.replaceNames that replaces the names in the "CNVDetectionResul
```

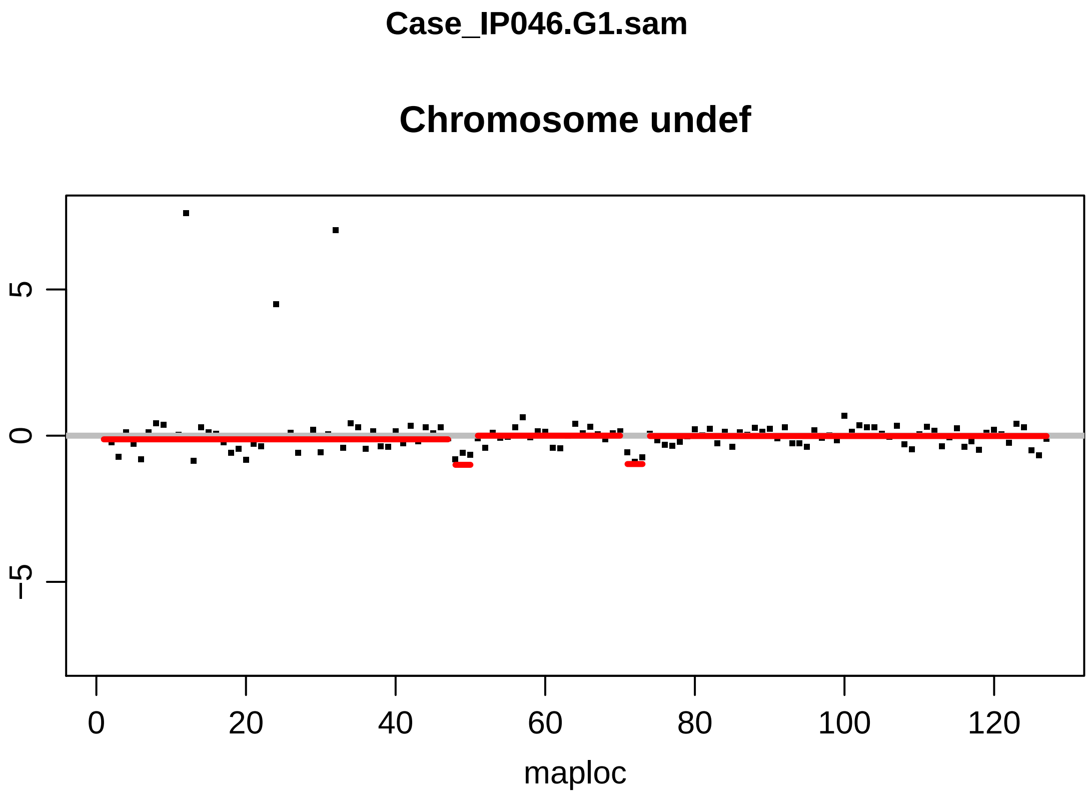


```
##
## CNV regions:
## GRanges object with 3 ranges and 36 metadata columns:
##       seqnames    ranges strand | Case_IP001.G1.sam Case_IP002.G1.sam
##          <Rle> <IRanges>  <Rle> |          <factor>          <factor>
##   [1]    undef  [ 6,  9]      * |               CN2               CN2
##   [2]    undef  [48, 50]      * |               CN2               CN2
##   [3]    undef  [71, 73]      * |               CN2               CN2
##       Case_IP003.G1.sam Case_IP004.G1.sam Case_IP005.G1.sam
##                <factor>          <factor>          <factor>
##   [1]               CN2               CN2               CN2
##   [2]               CN2               CN2               CN2
##   [3]               CN2               CN2               CN2
##       Case_IP006.G1.sam Case_IP007.G1.sam Case_IP008.G1.sam
##                <factor>          <factor>          <factor>
##   [1]               CN2               CN2               CN2
##   [2]               CN2               CN2               CN2
##   [3]               CN2               CN2               CN2
##       Case_IP010.G1.sam Case_IP011.G1.sam Case_IP012.G1.sam
##                <factor>          <factor>          <factor>
##   [1]               CN2               CN2               CN2
##   [2]               CN2               CN2               CN2
##   [3]               CN2               CN2               CN2
##       Case_IP013.G1.sam Case_IP014.G1.sam Case_IP015.G1.sam
##                <factor>          <factor>          <factor>
```

```
##   [1]               CN2               CN2               CN2
##   [2]               CN2               CN2               CN2
##   [3]               CN2               CN2               CN2
##       Case_IP016.G1.sam Case_IP017.G1.sam Case_IP019.G1.sam
##                <factor>          <factor>          <factor>
##   [1]               CN2               CN2               CN2
##   [2]               CN2               CN2               CN2
##   [3]               CN2               CN2               CN2
##       Case_IP020.G1.sam Case_IP022.G1.sam Case_IP023.G1.sam
##                <factor>          <factor>          <factor>
##   [1]               CN2               CN2               CN2
##   [2]               CN2               CN2               CN2
##   [3]               CN2               CN2               CN2
##       Case_IP024.G1.sam Case_IP025.G1.sam Case_IP028.G1.sam
##                <factor>          <factor>          <factor>
##   [1]               CN2               CN2               CN2
##   [2]               CN2               CN2               CN2
##   [3]               CN2               CN2               CN2
##       Case_IP029.G1.sam Case_IP030.G1.sam Case_IP031.G1.sam
##                <factor>          <factor>          <factor>
##   [1]               CN1               CN2               CN2
##   [2]               CN2               CN2               CN2
##   [3]               CN2               CN2               CN2
##       Case_IP032.G1.sam Case_IP033.G1.sam Case_IP035.G1.sam
##                <factor>          <factor>          <factor>
##   [1]               CN2               CN2               CN2
##   [2]               CN2               CN2               CN2
##   [3]               CN2               CN2               CN2
##       Case_IP036.G1.sam Case_IP037.G1.sam Case_IP038.G1.sam
##                <factor>          <factor>          <factor>
##   [1]               CN2               CN2               CN2
##   [2]               CN2               CN2               CN2
##   [3]               CN2               CN2               CN2
##       Case_IP039.G1.sam Case_IP040.G1.sam Case_IP046.G1.sam
##                <factor>          <factor>          <factor>
##   [1]               CN2               CN2               CN2
##   [2]               CN2               CN2               CN1
##   [3]               CN2               CN2               CN1
##       Case_IP047.G1.sam
##                <factor>
##   [1]               CN2
##   [2]               CN1
##   [3]               CN2
##   -------
##   seqinfo: 1 sequence from an unspecified genome; no seqlengths
##
## Individual CNVs:
## GRanges object with 4 ranges and 4 metadata columns:
##       seqnames    ranges strand |        sampleName     median       mean
##          <Rle> <IRanges>  <Rle> |          <factor>  <numeric>  <numeric>
##   [1]    undef  [ 6,  9]      * | Case_IP029.G1.sam -0.9998080 -0.9974585
##   [2]    undef  [48, 50]      * | Case_IP046.G1.sam -0.9996777 -0.9961643
##   [3]    undef  [71, 73]      * | Case_IP046.G1.sam -0.9999995 -0.9699700
##   [4]    undef  [48, 50]      * | Case_IP047.G1.sam -0.9999246 -0.9996100
```

```
##                CN
##       <character>
##   [1]         CN1
##   [2]         CN1
##   [3]         CN1
##   [4]         CN1
##   -------
##   seqinfo: 1 sequence from an unspecified genome; no seqlengths
## [1] "/Users/gdemidov/Downloads/doc/Run_fourth_run_fin_05_qc.xls"

## Normalizing...

## Starting local modeling, please be patient...

## Reference sequence:  undef

## Starting segmentation algorithm...

## Using "fastseg" for segmentation.

## [1] ""
## [1] "/Users/gdemidov/Downloads/doc/Run_fourth_run_fin_05_qc.xls"
## [1] ""

## Segplot might not work because of special characters in the sample names. Use only A-Z,a-z and 0-9!
##  There is a hidden function cn.mops:::.replaceNames that replaces the names in the "CNVDetectionResul
```

Case_IP047.G1.sam

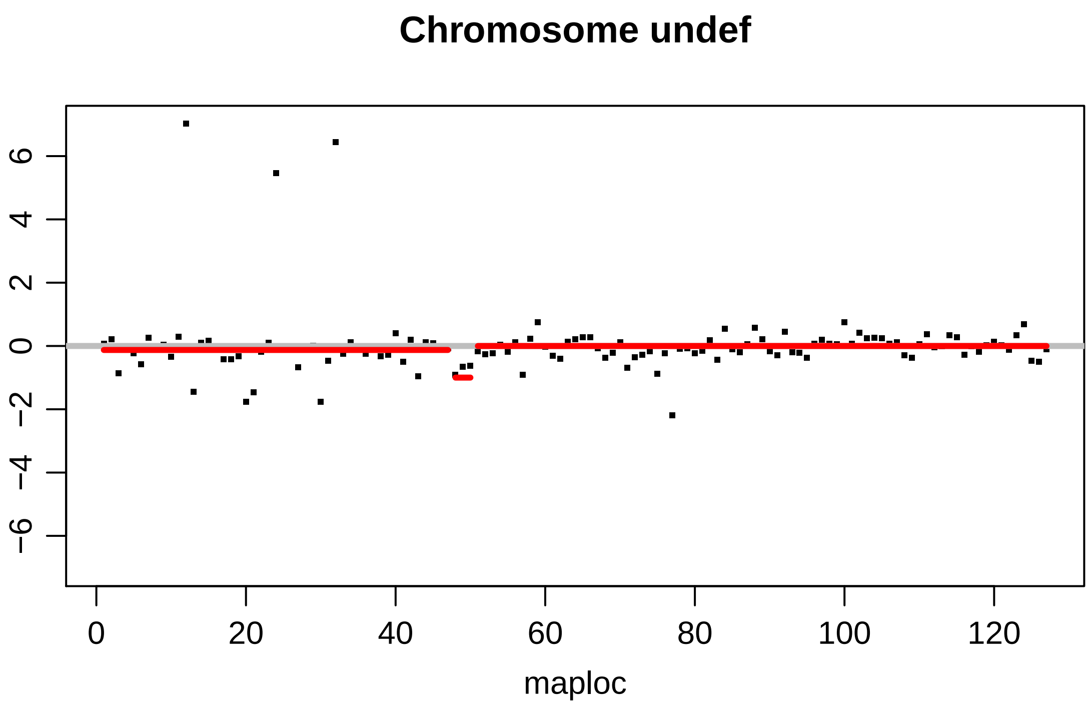

```
## Segplot might not work because of special characters in the sample names. Use only A-Z,a-z and 0-9!
##  There is a hidden function cn.mops:::.replaceNames that replaces the names in the "CNVDetectionResul
```

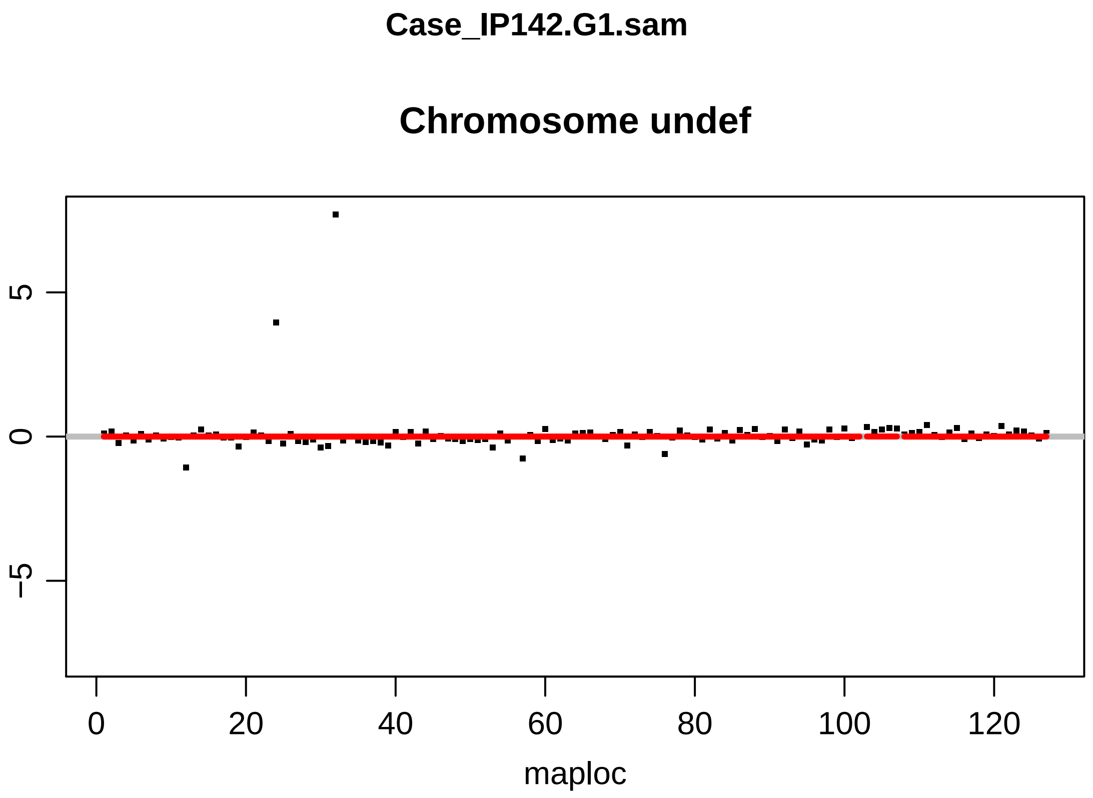


```
## Segplot might not work because of special characters in the sample names. Use only A-Z,a-z and 0-9!
##  There is a hidden function cn.mops:::.replaceNames that replaces the names in the "CNVDetectionResul
```

Case_IP143.G1.sam

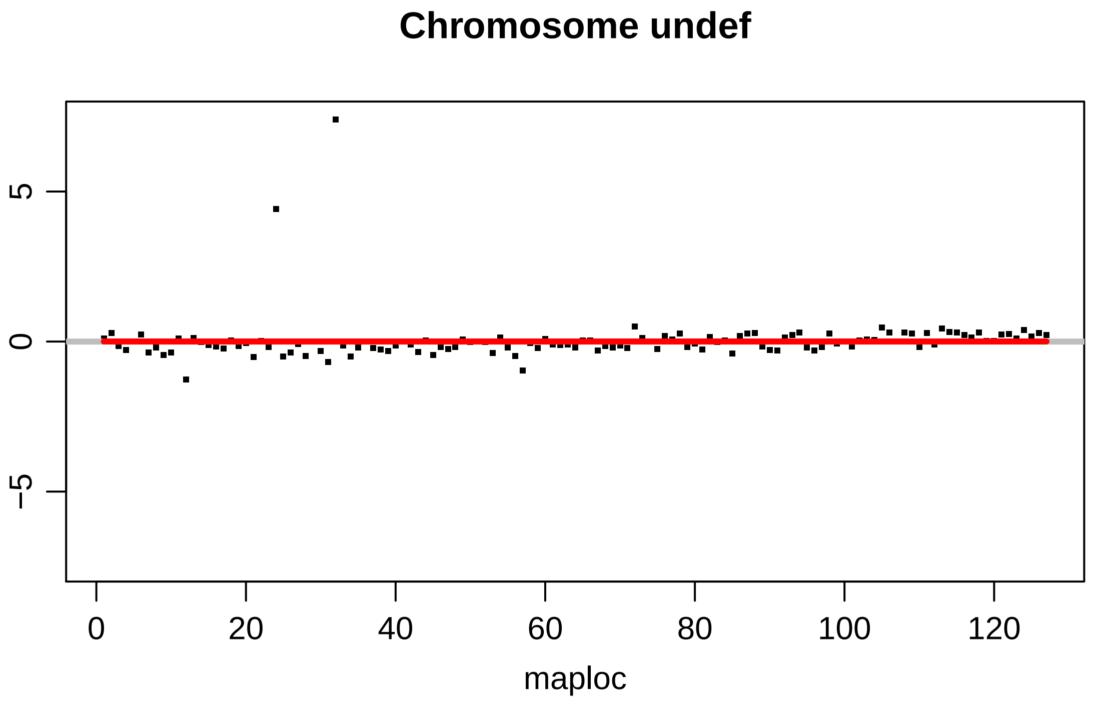


```
## Segplot might not work because of special characters in the sample names. Use only A-Z,a-z and 0-9!
##  There is a hidden function cn.mops:::.replaceNames that replaces the names in the "CNVDetectionResul
```

Case_IP144.G1.sam

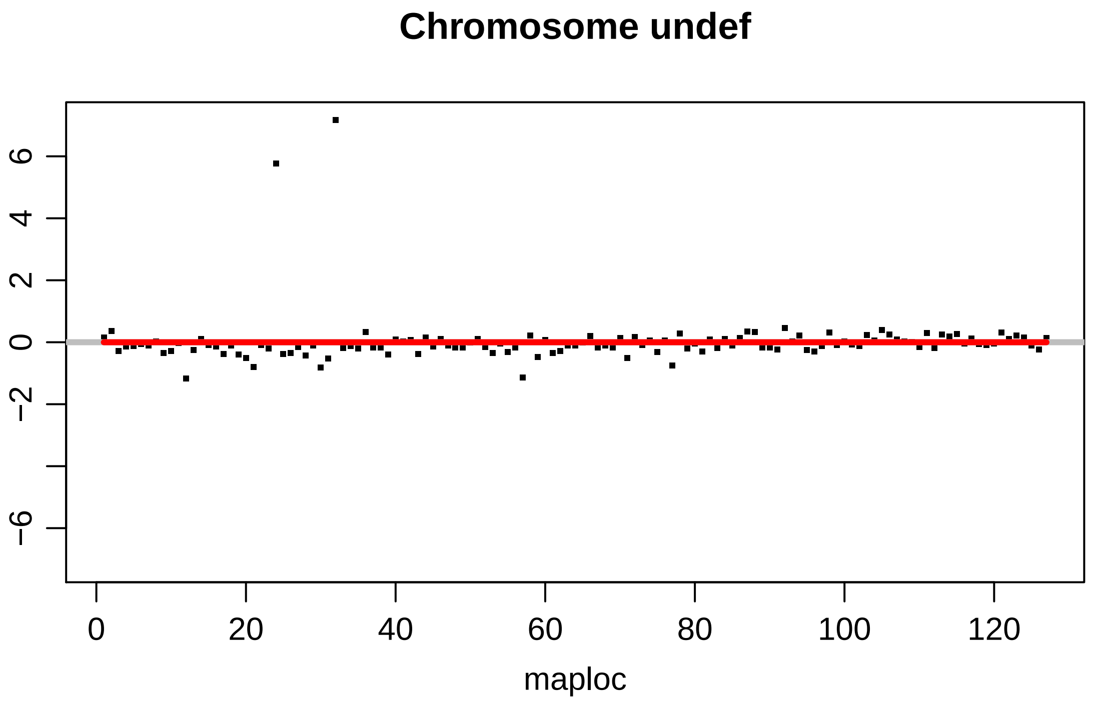

```
## Segplot might not work because of special characters in the sample names. Use only A-Z,a-z and 0-9!
##  There is a hidden function cn.mops:::.replaceNames that replaces the names in the "CNVDetectionResul
```

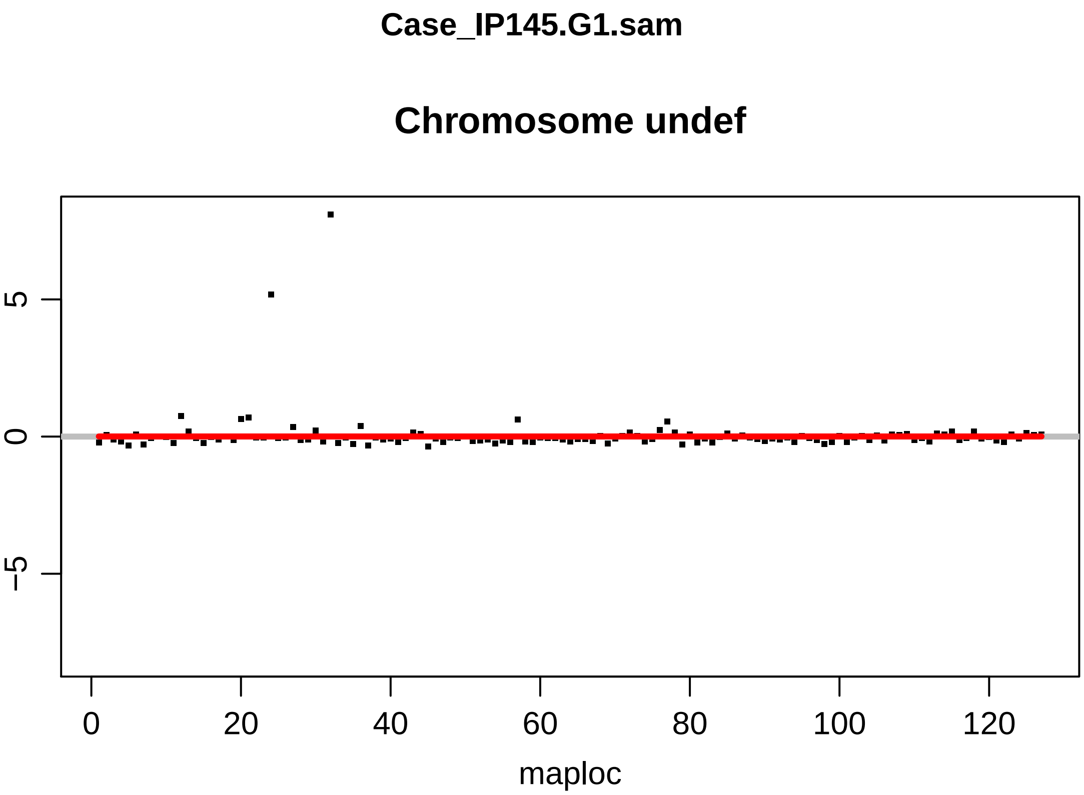


```
## Segplot might not work because of special characters in the sample names. Use only A-Z,a-z and 0-9!
##  There is a hidden function cn.mops:::.replaceNames that replaces the names in the "CNVDetectionResul
```

Case_IP146.G1.sam

Chromosome undef

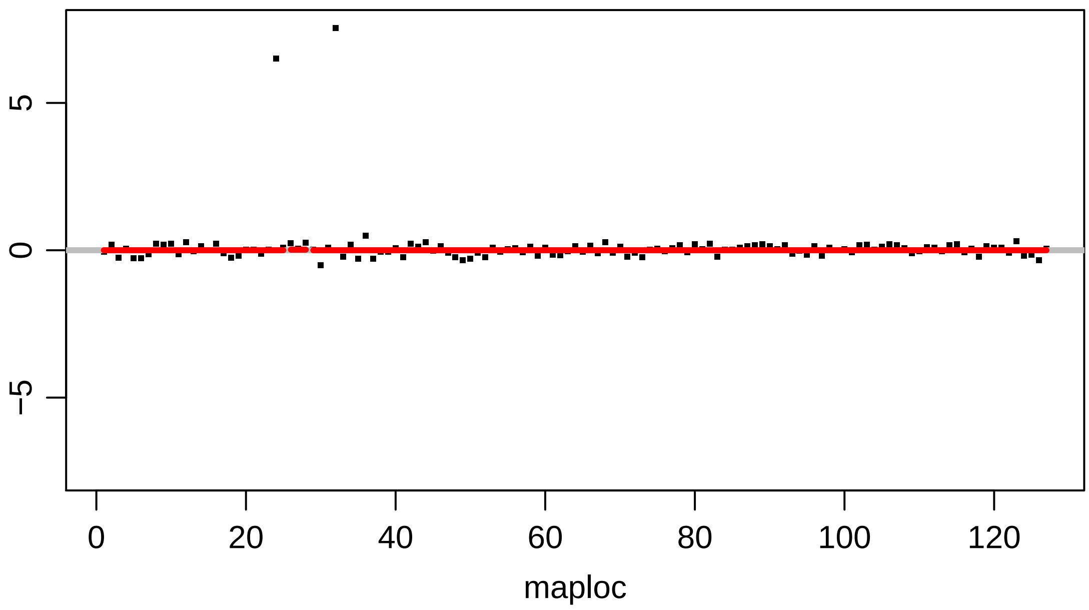


```
## Segplot might not work because of special characters in the sample names. Use only A-Z,a-z and 0-9!
##  There is a hidden function cn.mops:::.replaceNames that replaces the names in the "CNVDetectionResul[illegible]
```

Case_IP148.G1.sam

Chromosome undef

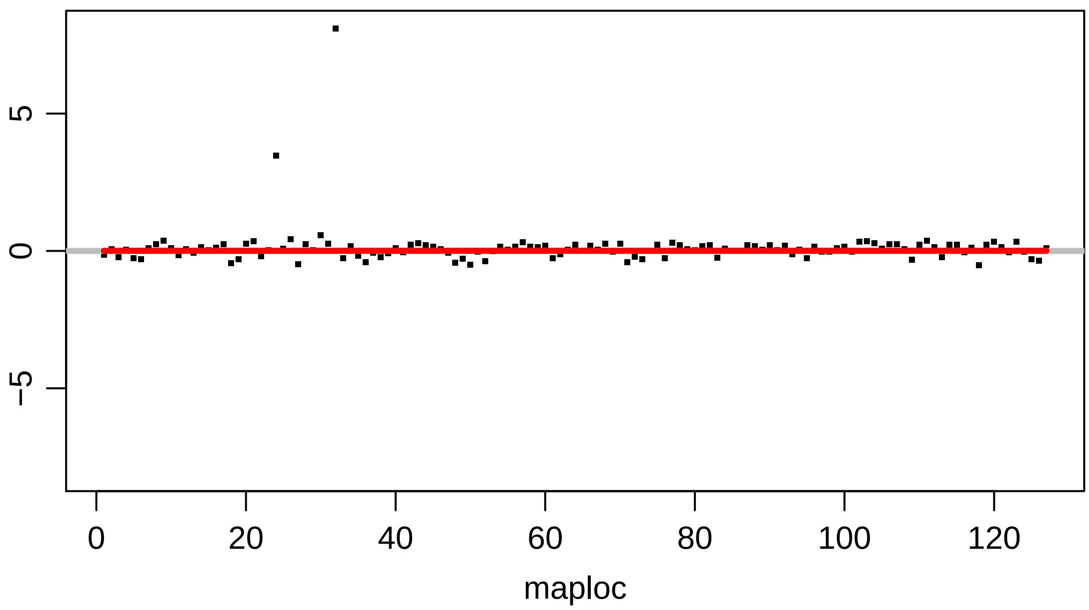

```
## Segplot might not work because of special characters in the sample names. Use only A-Z,a-z and 0-9!
##  There is a hidden function cn.mops:::.replaceNames that replaces the names in the "CNVDetectionResul
```

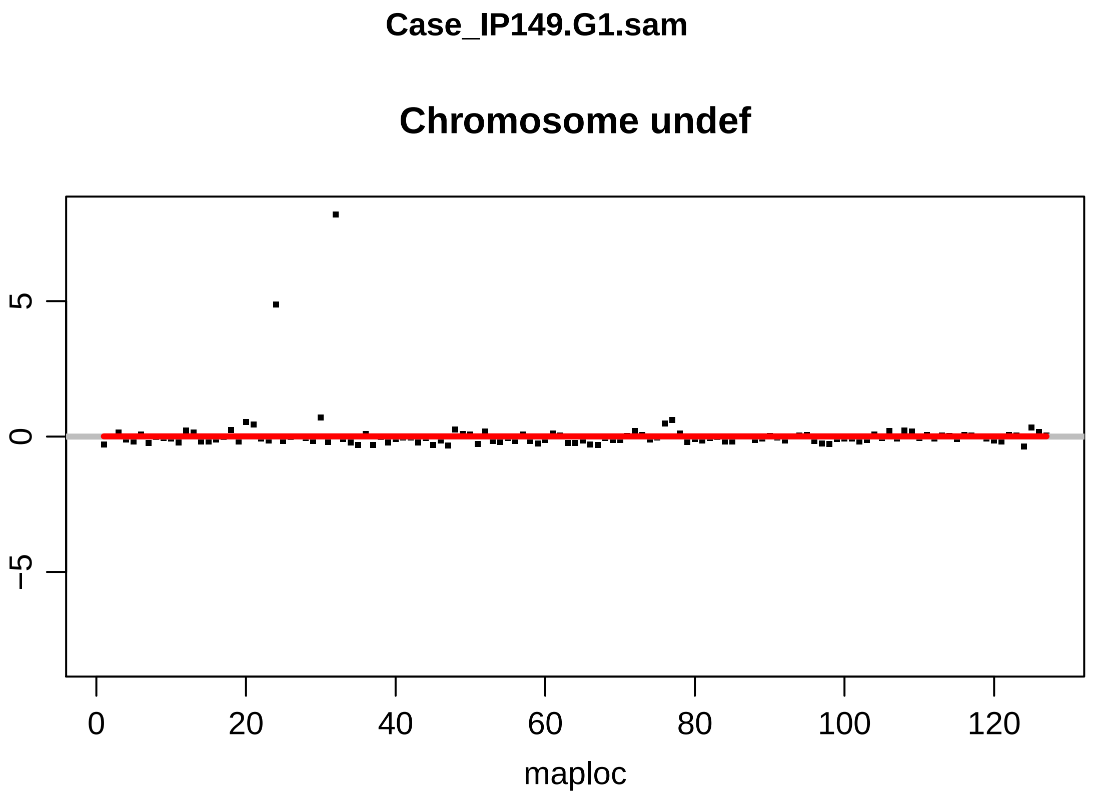


```
## Segplot might not work because of special characters in the sample names. Use only A-Z,a-z and 0-9!
##  There is a hidden function cn.mops:::.replaceNames that replaces the names in the "CNVDetectionResul
```

Case_IP150.G1.sam

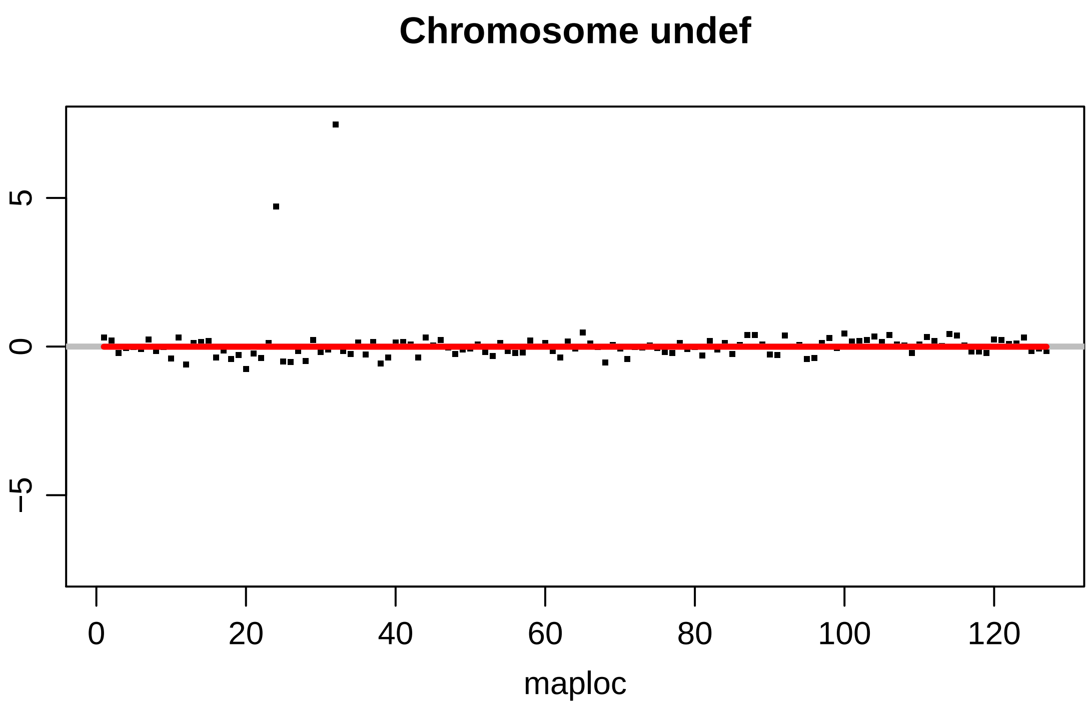


```
## Segplot might not work because of special characters in the sample names. Use only A-Z,a-z and 0-9!
##  There is a hidden function cn.mops:::.replaceNames that replaces the names in the "CNVDetectionResul
```

Case_IP151.G1.sam

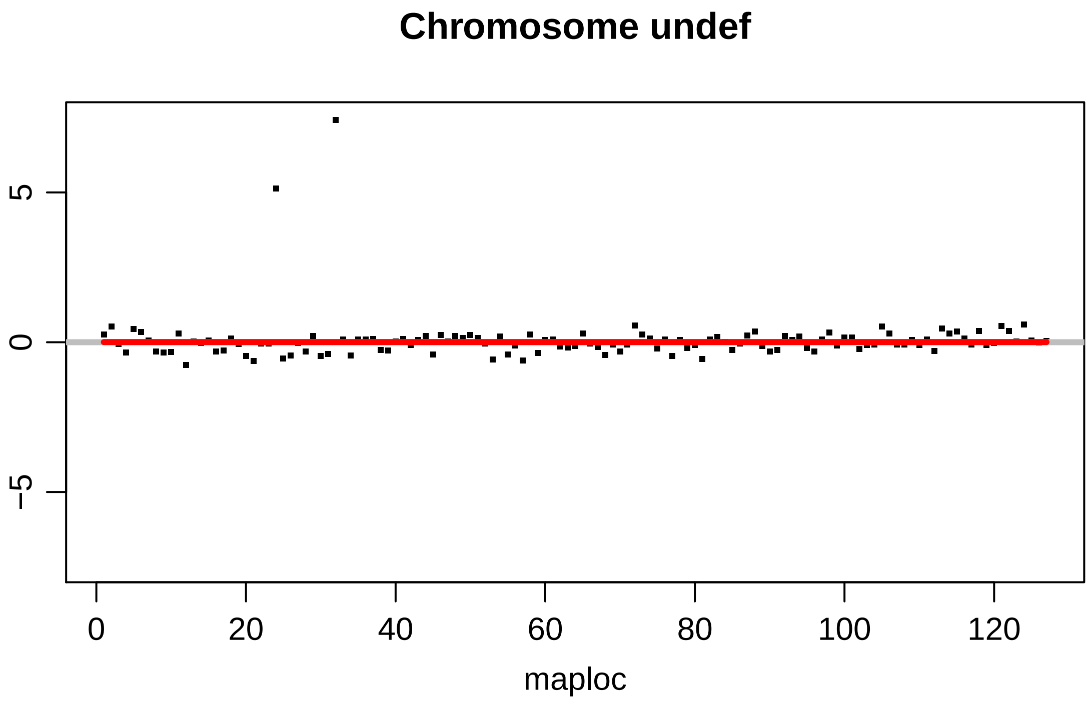

```
## Segplot might not work because of special characters in the sample names. Use only A-Z,a-z and 0-9!
##  There is a hidden function cn.mops:::.replaceNames that replaces the names in the "CNVDetectionResul
```

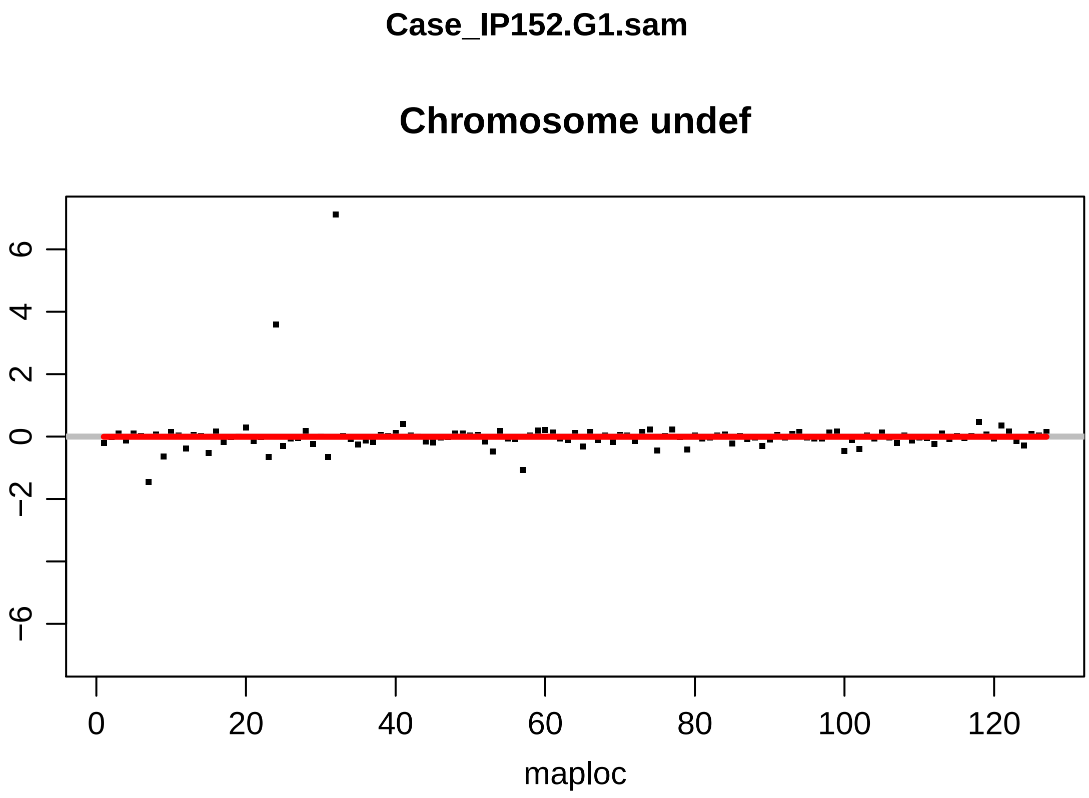


```
## Segplot might not work because of special characters in the sample names. Use only A-Z,a-z and 0-9!
##  There is a hidden function cn.mops:::.replaceNames that replaces the names in the "CNVDetectionResul
```

Case_IP154.G1.sam

Chromosome undef

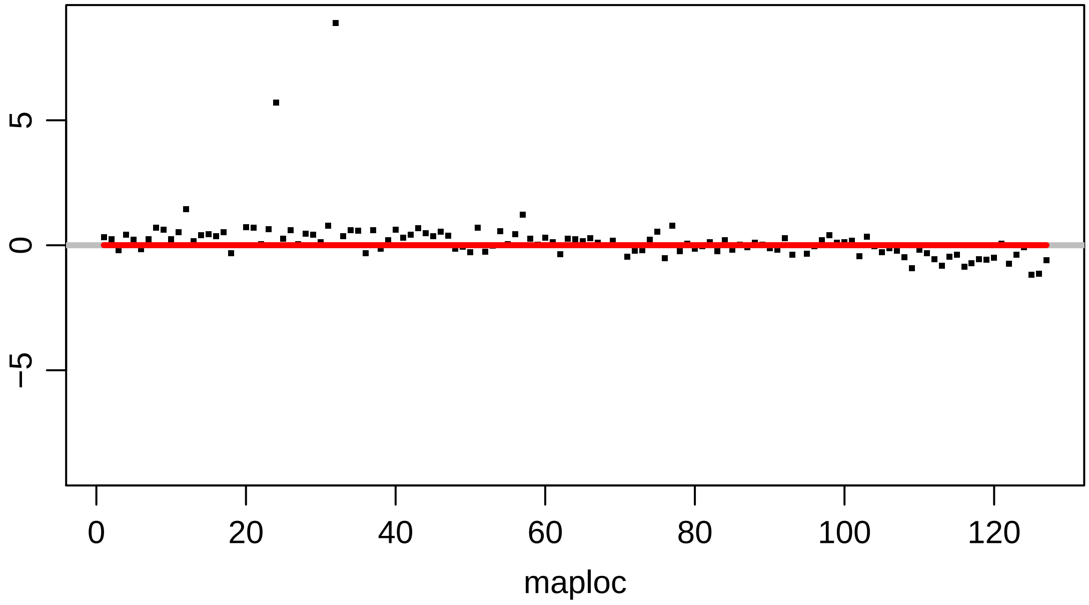


```
## Segplot might not work because of special characters in the sample names. Use only A-Z,a-z and 0-9!
##  There is a hidden function cn.mops:::.replaceNames that replaces the names in the "CNVDetectionResul
```

Case_IP155.G2.sam

Chromosome undef

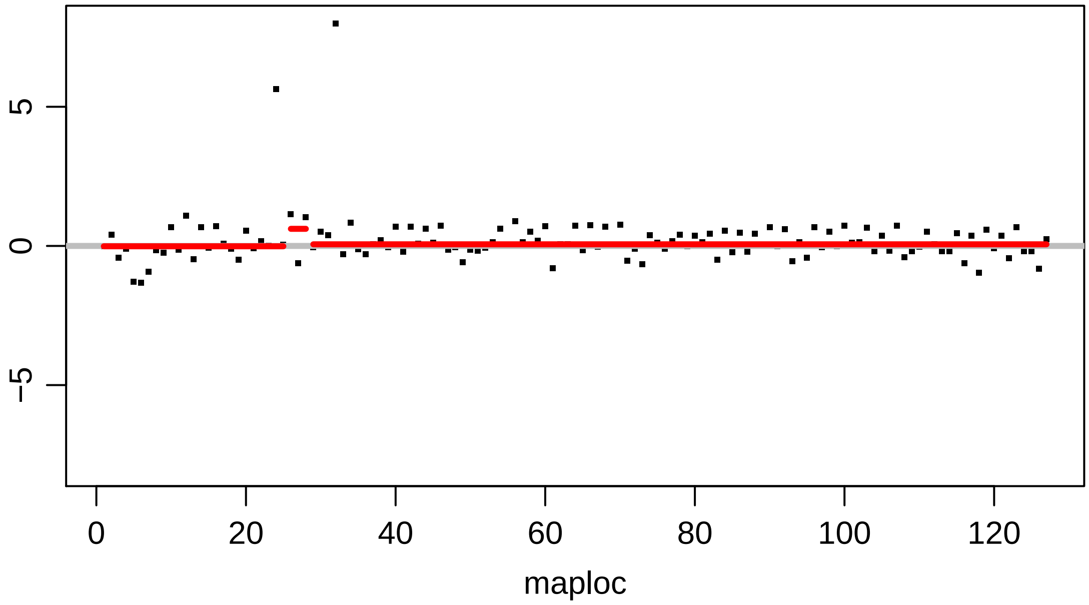

```
## Segplot might not work because of special characters in the sample names. Use only A-Z,a-z and 0-9!
##  There is a hidden function cn.mops:::.replaceNames that replaces the names in the "CNVDetectionResul
```

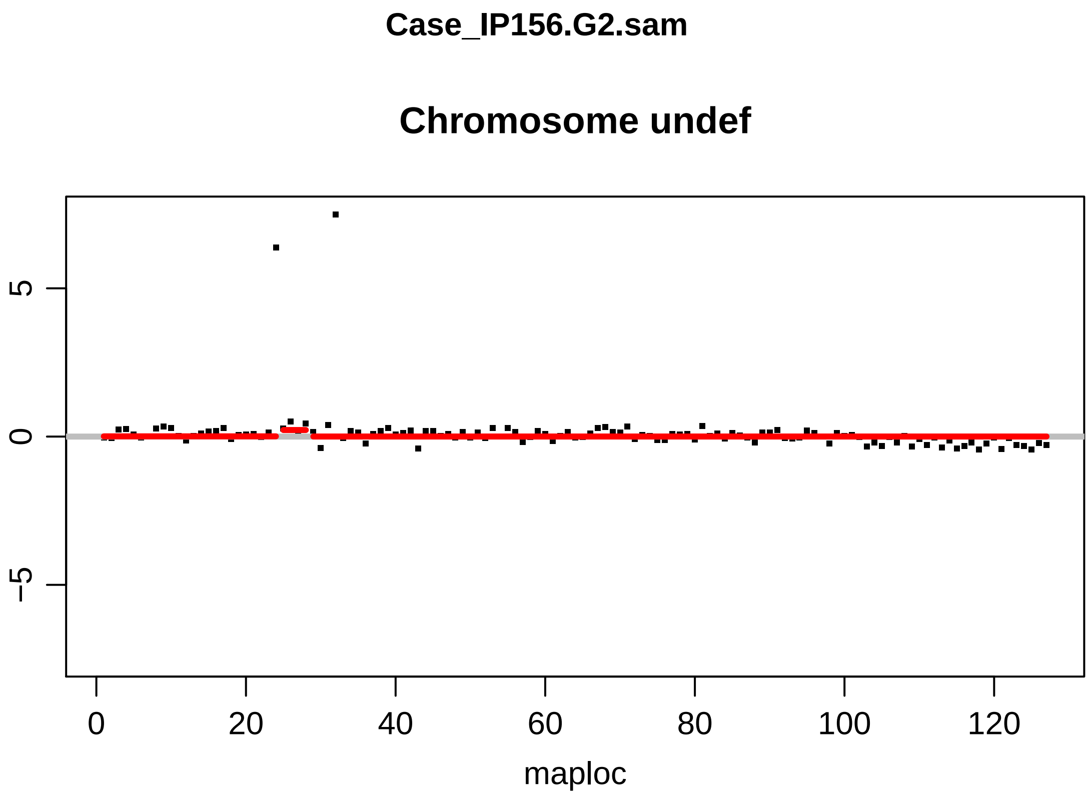


```
## Segplot might not work because of special characters in the sample names. Use only A-Z,a-z and 0-9!
##  There is a hidden function cn.mops:::.replaceNames that replaces the names in the "CNVDetectionResul
```

Case_IP158.G2.sam

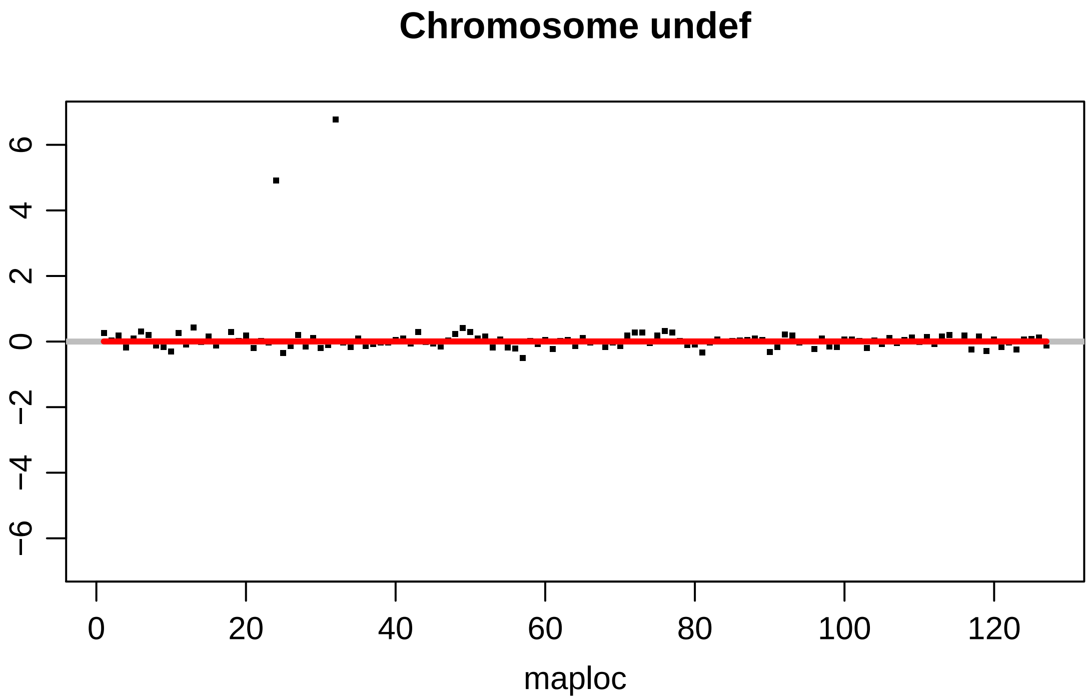


```
## Segplot might not work because of special characters in the sample names. Use only A-Z,a-z and 0-9!
##  There is a hidden function cn.mops:::.replaceNames that replaces the names in the "CNVDetectionResul
```

Case_IP160.G2.sam

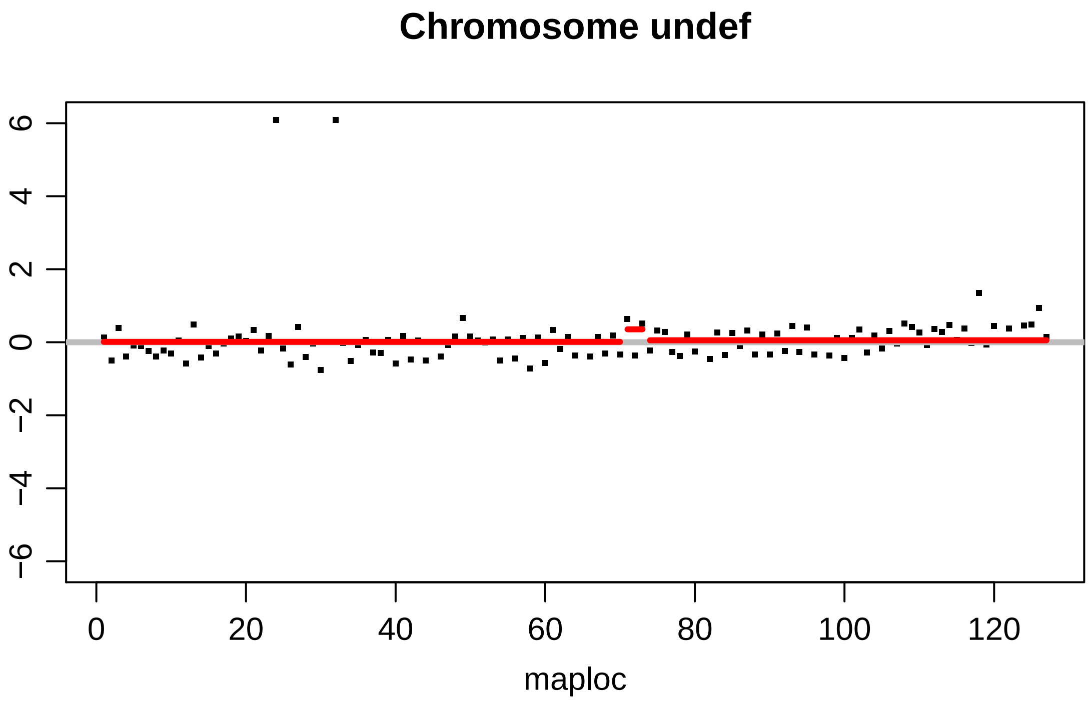

```
## Segplot might not work because of special characters in the sample names. Use only A-Z,a-z and 0-9!
##  There is a hidden function cn.mops:::.replaceNames that replaces the names in the "CNVDetectionResul
```

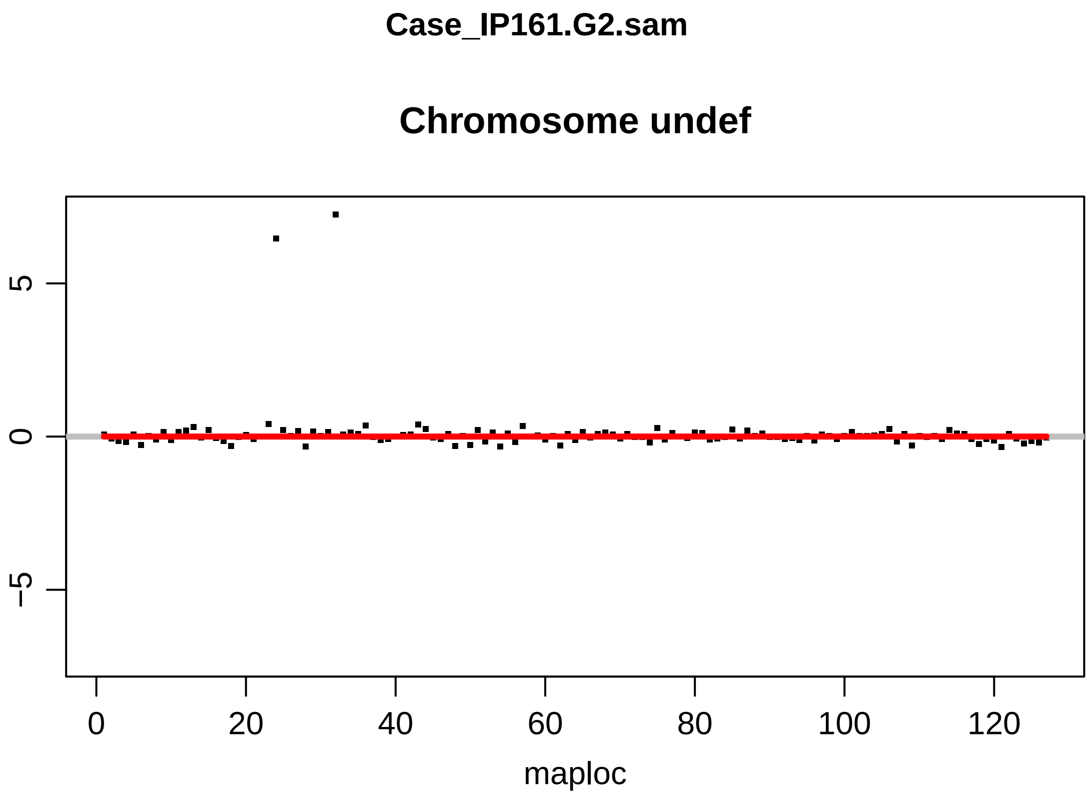


```
## Segplot might not work because of special characters in the sample names. Use only A-Z,a-z and 0-9!
##  There is a hidden function cn.mops:::.replaceNames that replaces the names in the "CNVDetectionResul
```

Case_IP162.G2.sam

Chromosome undef

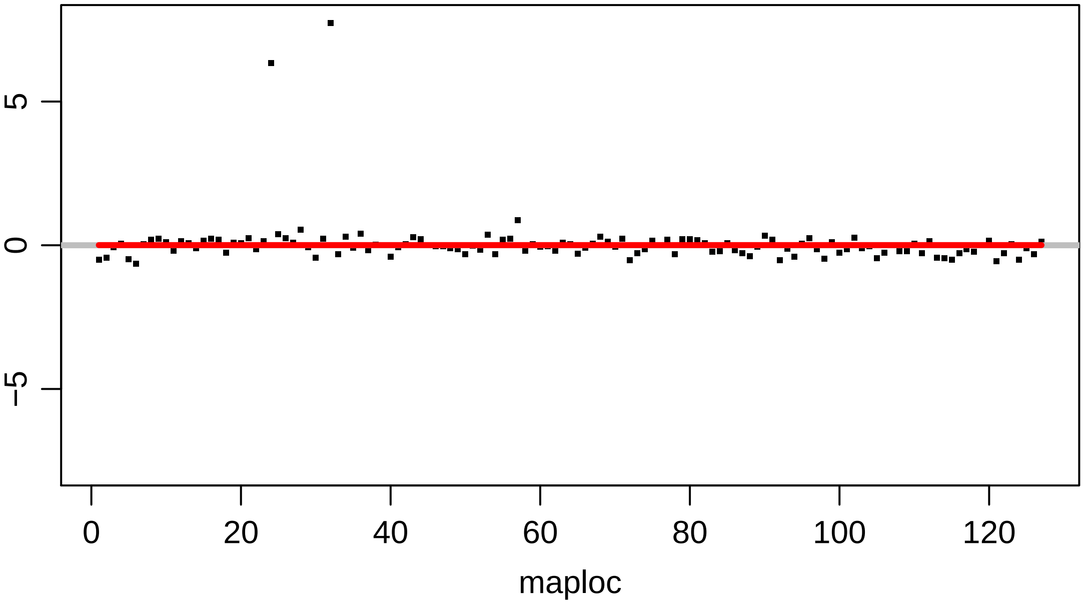


```
## Segplot might not work because of special characters in the sample names. Use only A-Z,a-z and 0-9!
##  There is a hidden function cn.mops:::.replaceNames that replaces the names in the "CNVDetectionResul
```

Case_IP164.G2.sam

Chromosome undef

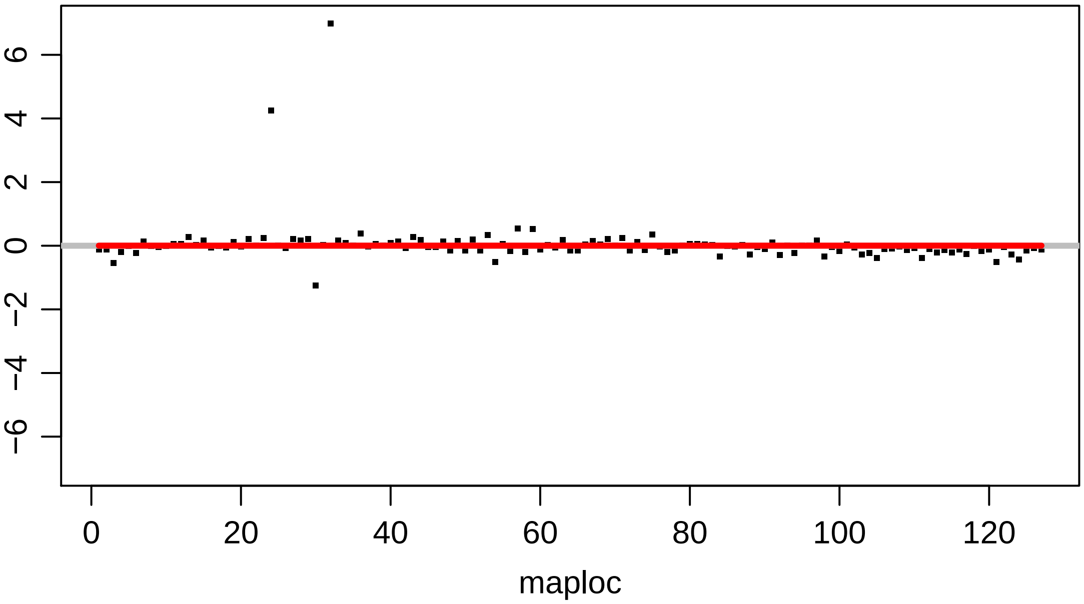

```
## Segplot might not work because of special characters in the sample names. Use only A-Z,a-z and 0-9!
##  There is a hidden function cn.mops:::.replaceNames that replaces the names in the "CNVDetectionResul
```

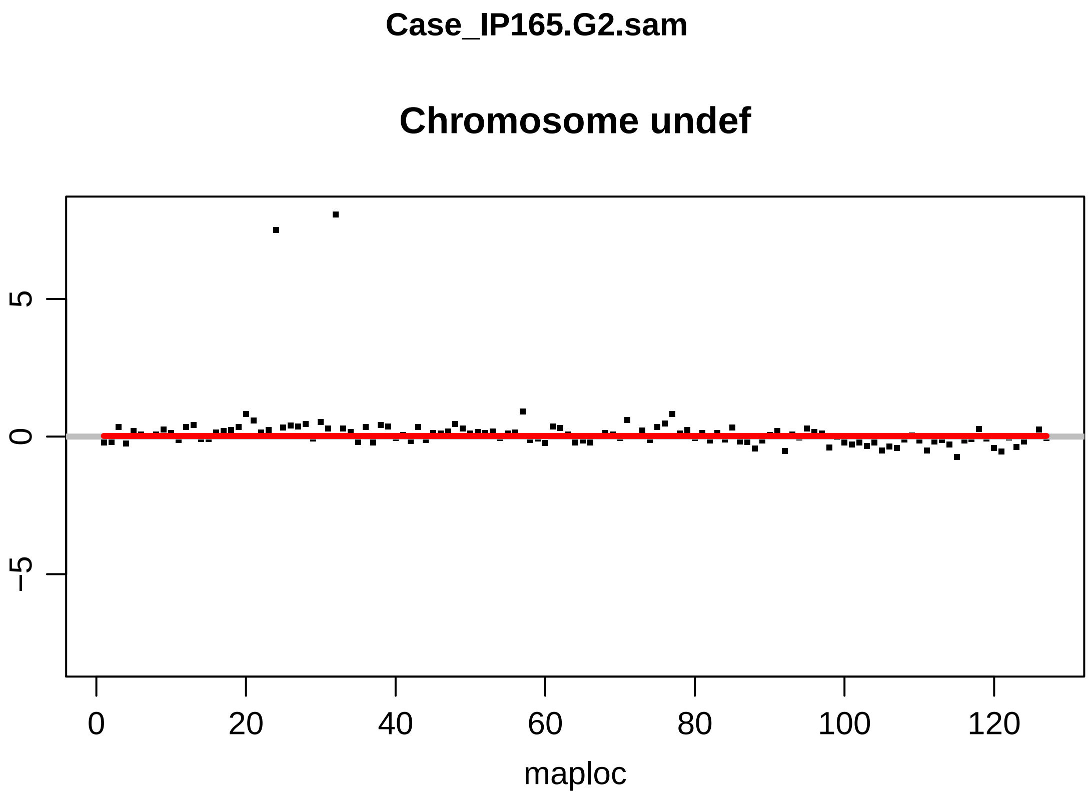


```
## Segplot might not work because of special characters in the sample names. Use only A-Z,a-z and 0-9!
##  There is a hidden function cn.mops:::.replaceNames that replaces the names in the "CNVDetectionResul
```

Case_IP167.G2.sam

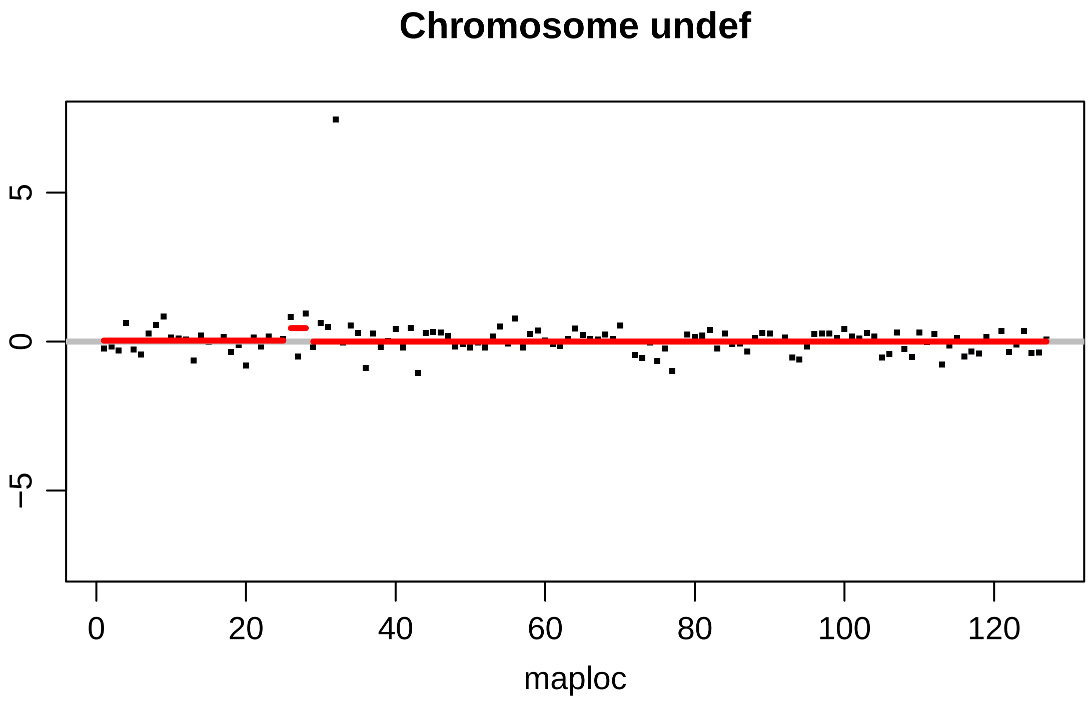


```
## Segplot might not work because of special characters in the sample names. Use only A-Z,a-z and 0-9!
##  There is a hidden function cn.mops:::.replaceNames that replaces the names in the "CNVDetectionResul
```

Case_IP168.G2.sam

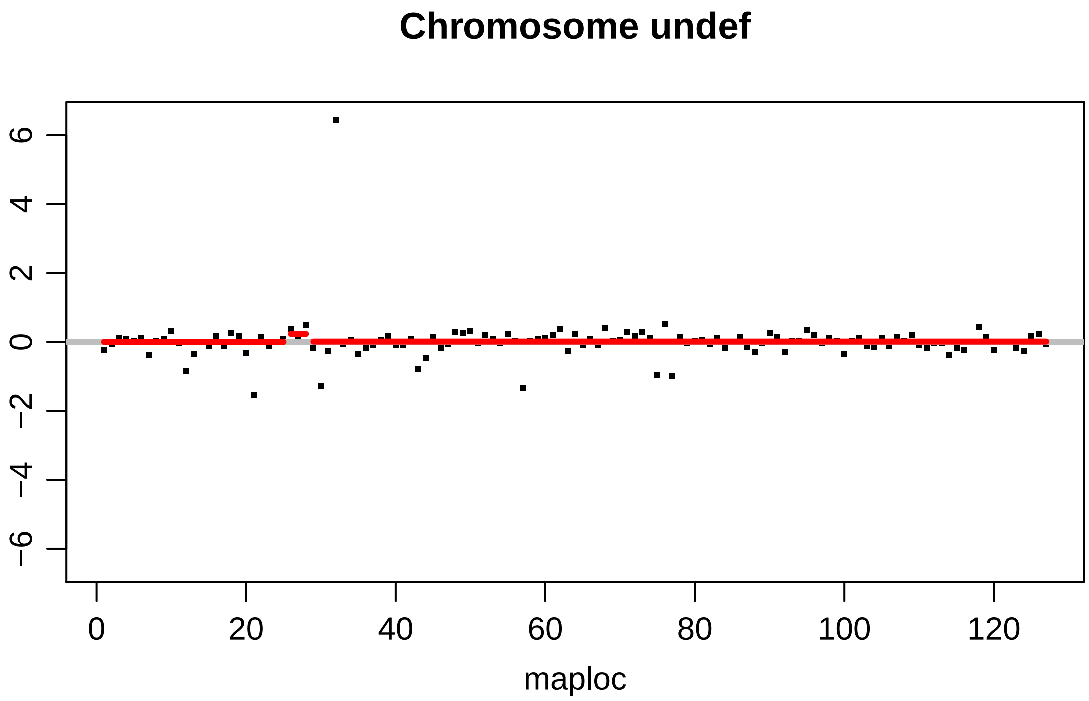

```
## Segplot might not work because of special characters in the sample names. Use only A-Z,a-z and 0-9!
##  There is a hidden function cn.mops:::.replaceNames that replaces the names in the "CNVDetectionResul
```

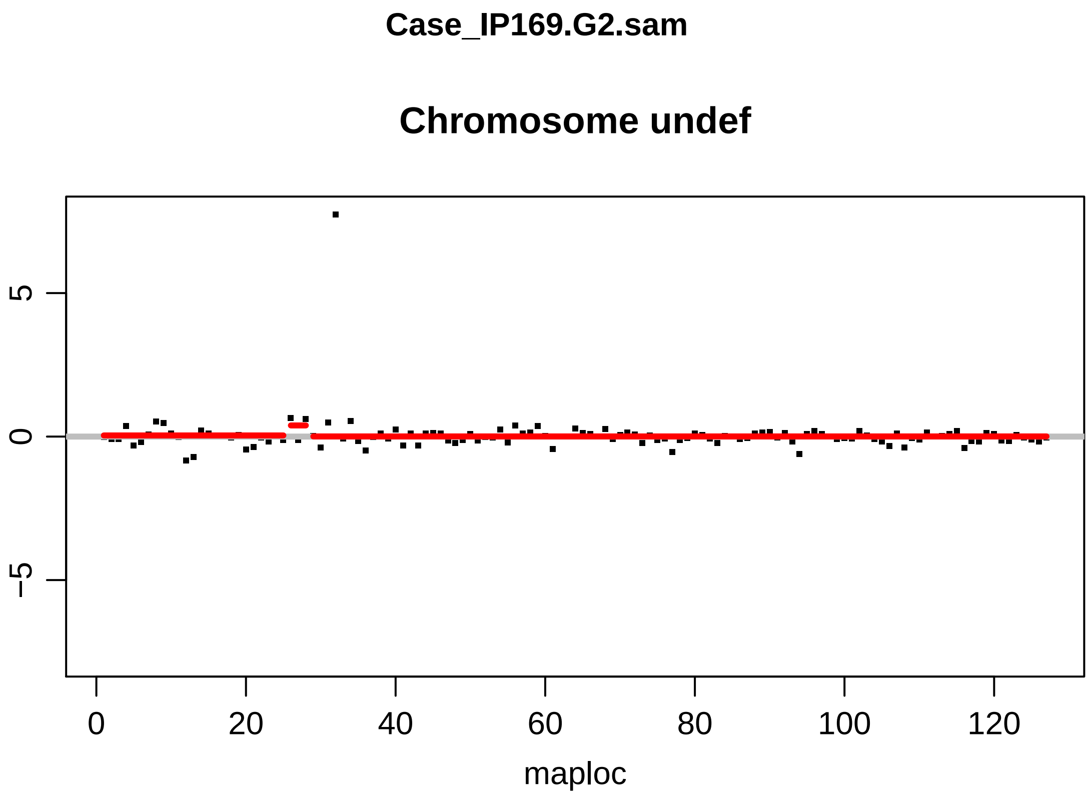


```
## Segplot might not work because of special characters in the sample names. Use only A-Z,a-z and 0-9!
##  There is a hidden function cn.mops:::.replaceNames that replaces the names in the "CNVDetectionResul
```

Case_IP170.G2.sam

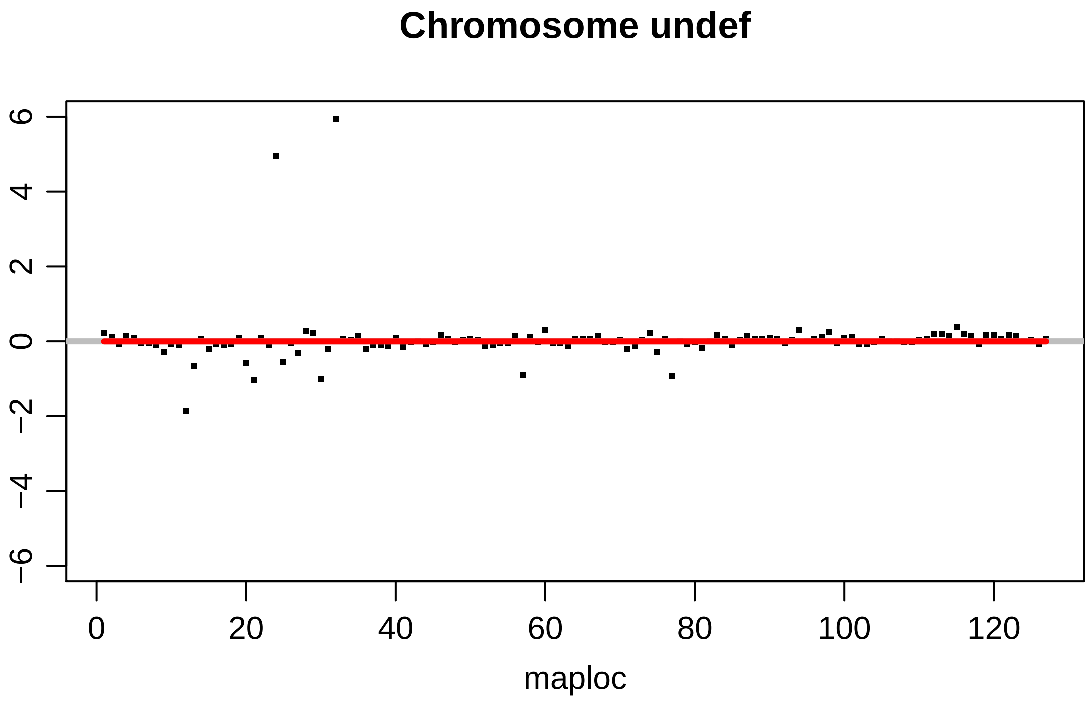


```
## Segplot might not work because of special characters in the sample names. Use only A-Z,a-z and 0-9!
##  There is a hidden function cn.mops:::.replaceNames that replaces the names in the "CNVDetectionResul
```

Case_IP171.G2.sam

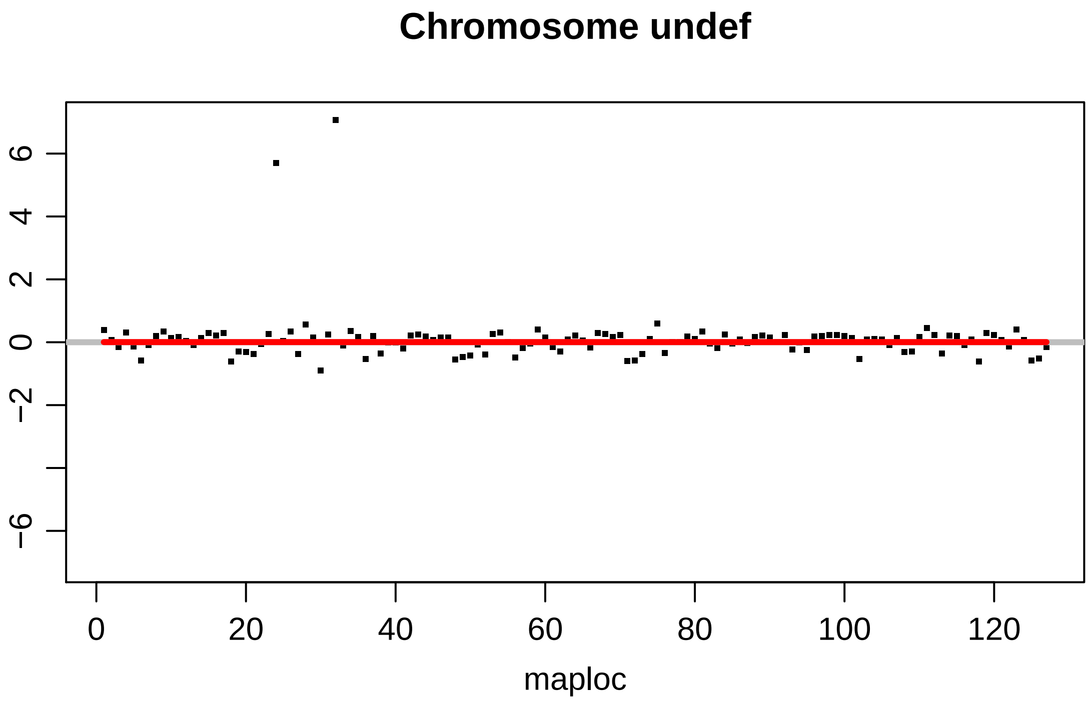

```
## Segplot might not work because of special characters in the sample names. Use only A-Z,a-z and 0-9!
##  There is a hidden function cn.mops:::.replaceNames that replaces the names in the "CNVDetectionResul
```

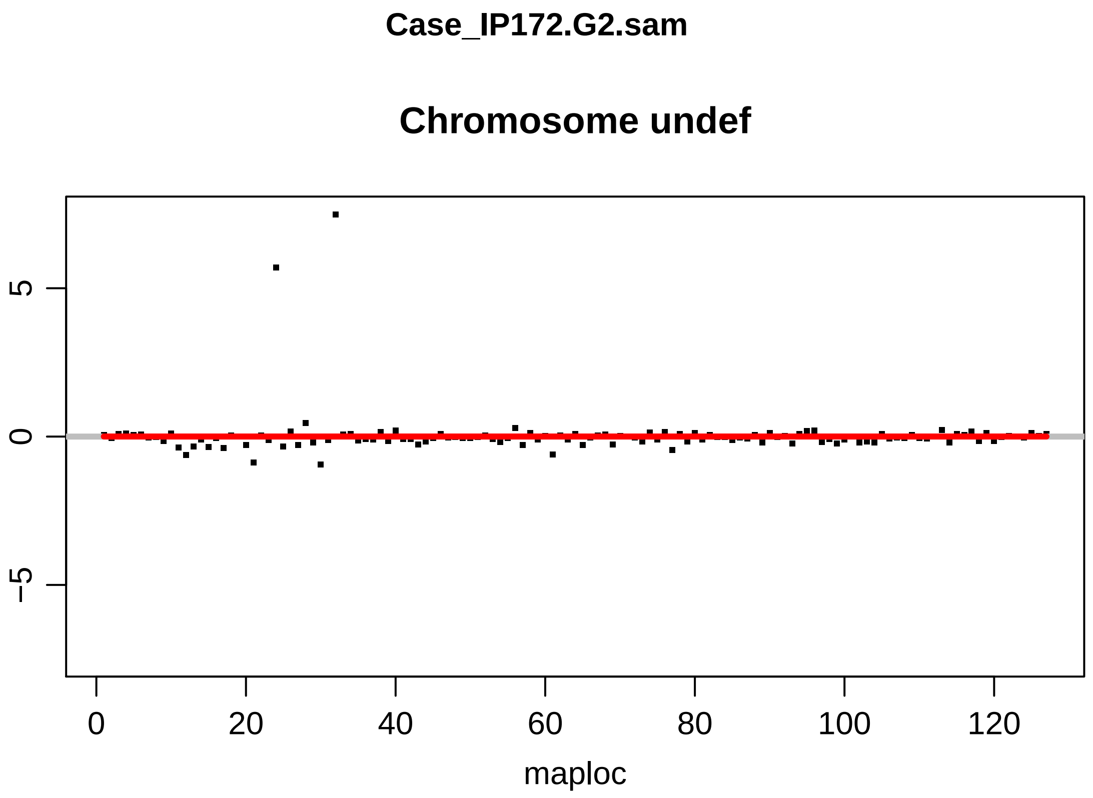


```
## Segplot might not work because of special characters in the sample names. Use only A-Z,a-z and 0-9!
##  There is a hidden function cn.mops:::.replaceNames that replaces the names in the "CNVDetectionResul
```

Case_IP173.G2.sam

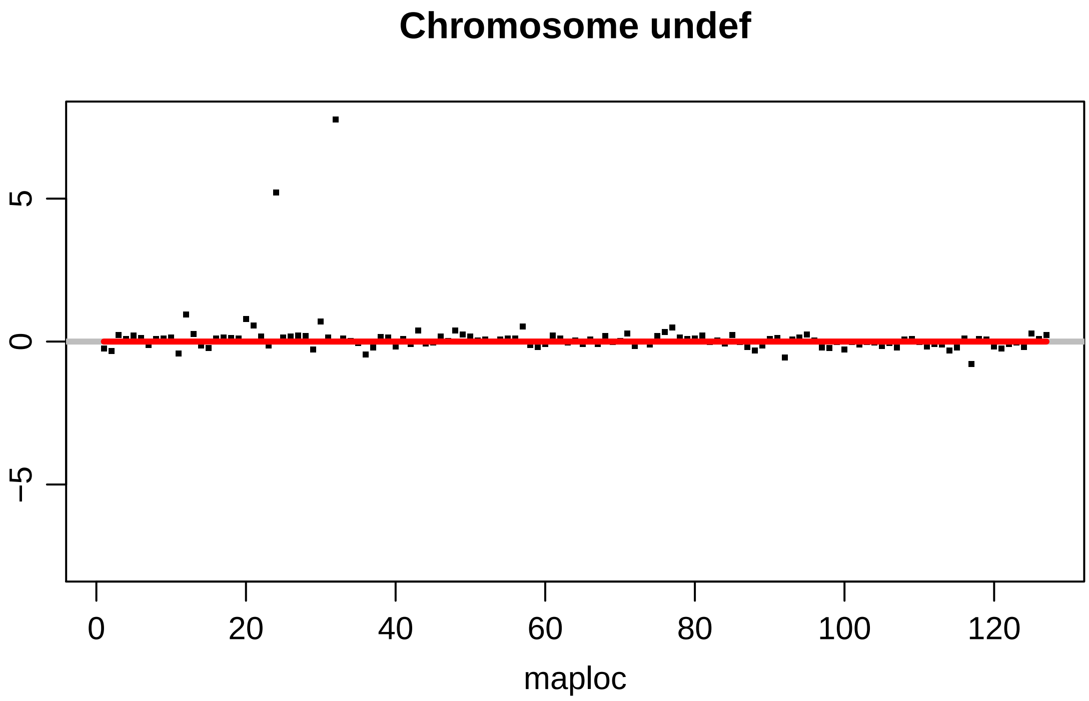


```
## Segplot might not work because of special characters in the sample names. Use only A-Z,a-z and 0-9!
##  There is a hidden function cn.mops:::.replaceNames that replaces the names in the "CNVDetectionResul
```

Case_IP174.G2.sam

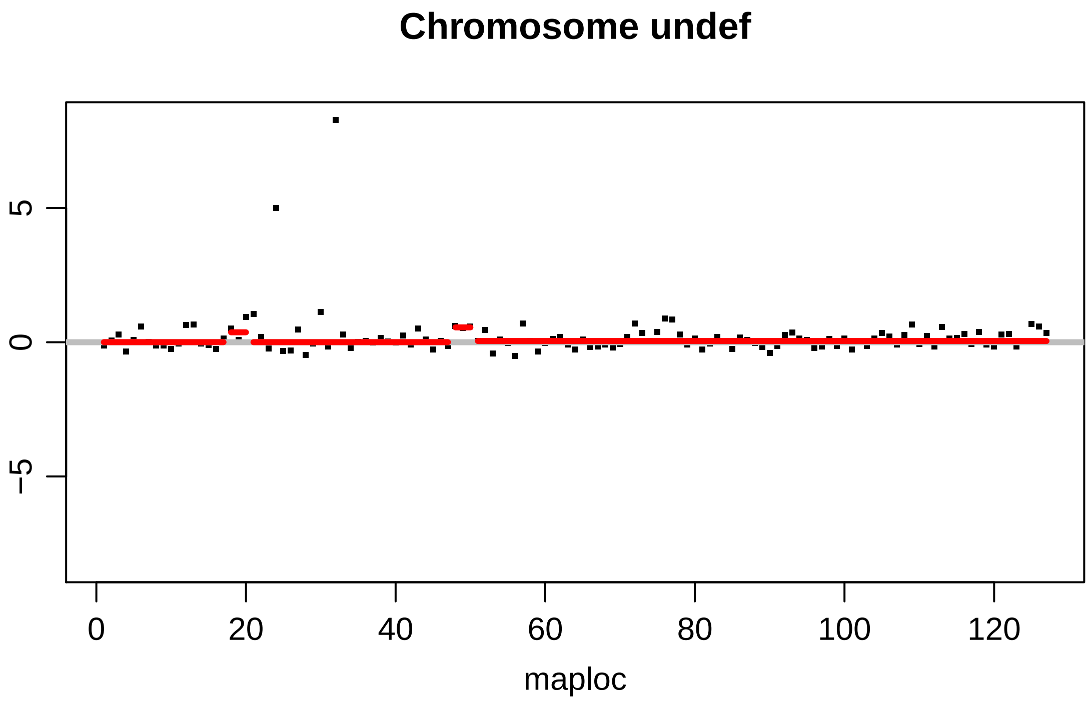

```
## Segplot might not work because of special characters in the sample names. Use only A-Z,a-z and 0-9!
##  There is a hidden function cn.mops:::.replaceNames that replaces the names in the "CNVDetectionResul
```

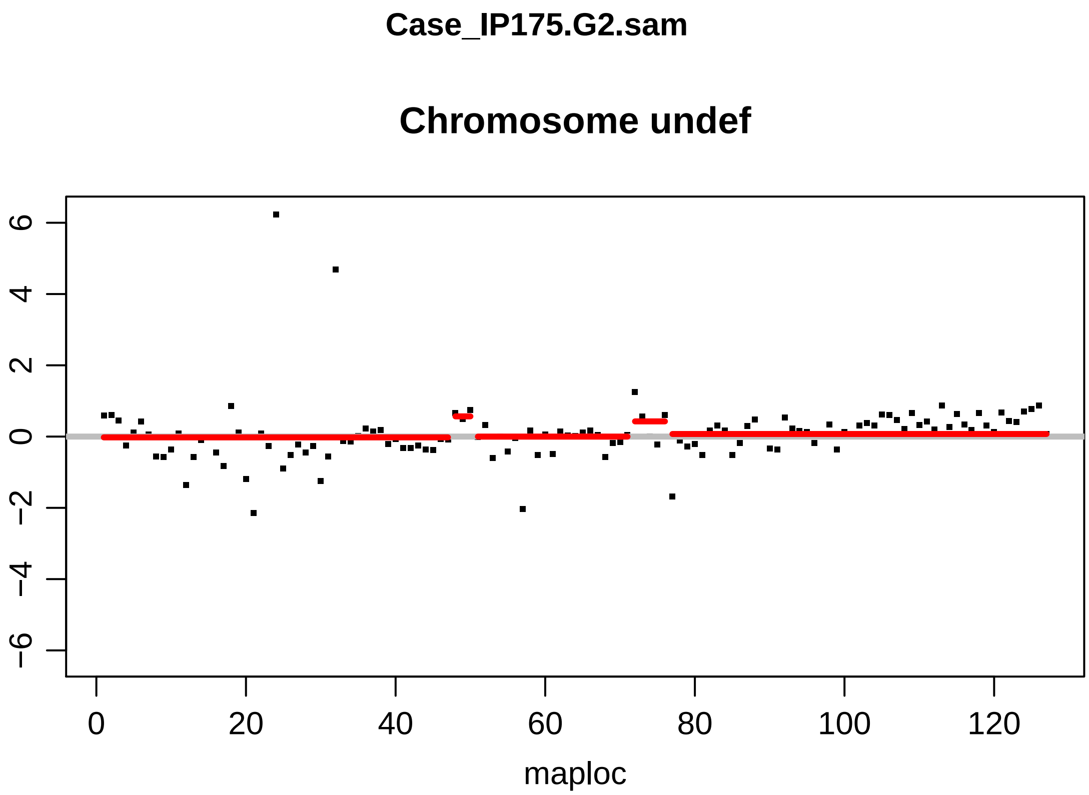


```
## Segplot might not work because of special characters in the sample names. Use only A-Z,a-z and 0-9!
##  There is a hidden function cn.mops:::.replaceNames that replaces the names in the "CNVDetectionResul
```

Case_IP176.G2.sam

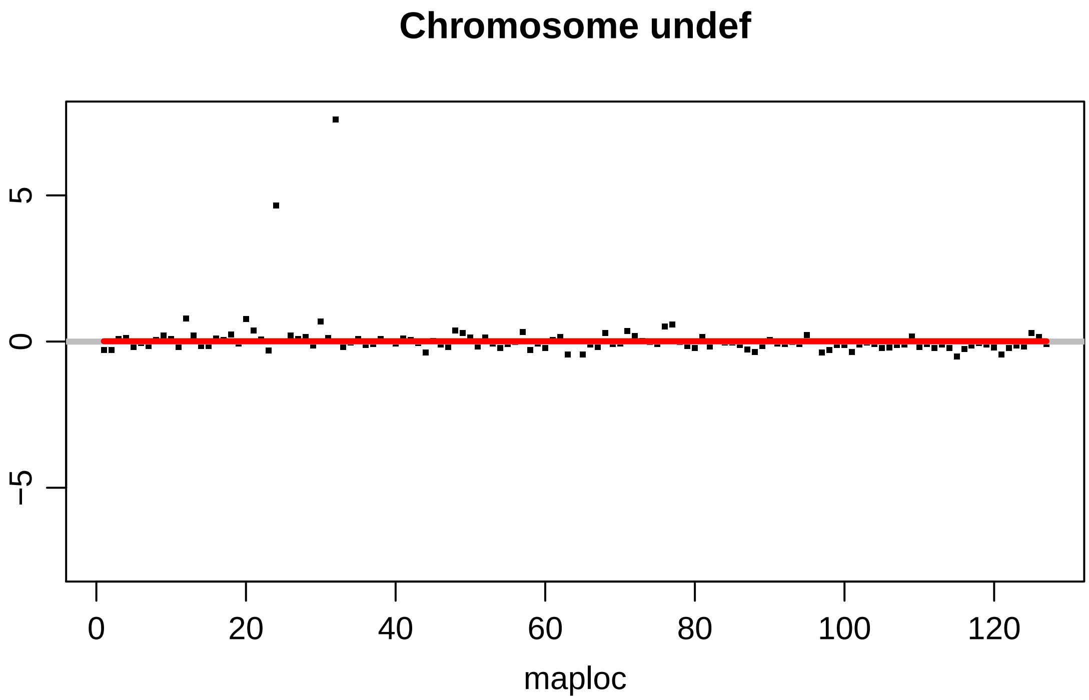


```
## Segplot might not work because of special characters in the sample names. Use only A-Z,a-z and 0-9!
##  There is a hidden function cn.mops:::.replaceNames that replaces the names in the "CNVDetectionResu
```

Case_IP177.G2.sam

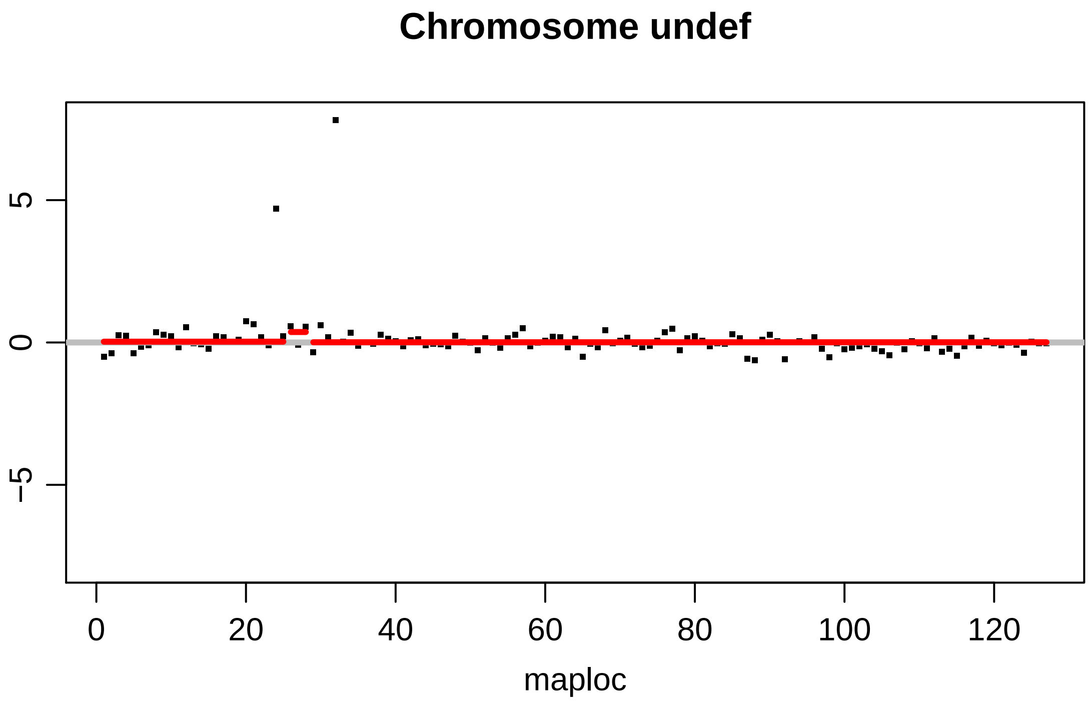

```
## Segplot might not work because of special characters in the sample names. Use only A-Z,a-z and 0-9!
##  There is a hidden function cn.mops:::.replaceNames that replaces the names in the "CNVDetectionResul
```

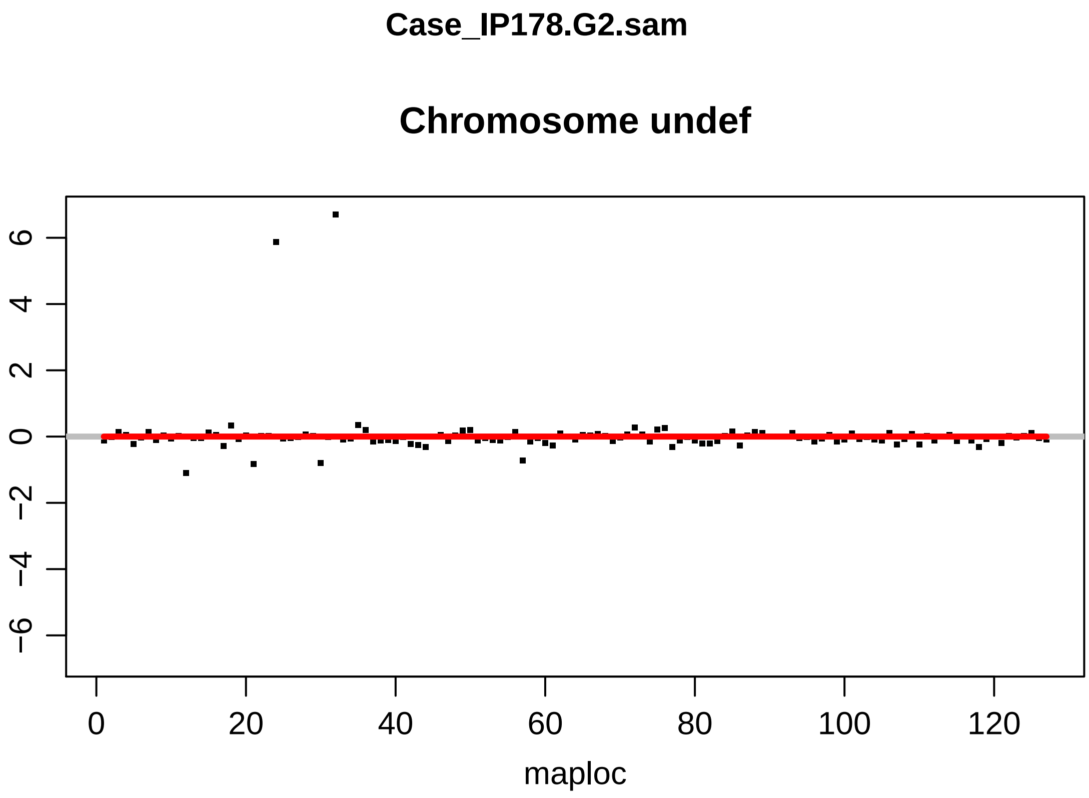


```
## Segplot might not work because of special characters in the sample names. Use only A-Z,a-z and 0-9!
##  There is a hidden function cn.mops:::.replaceNames that replaces the names in the "CNVDetectionResul
```

Case_IP179.G2.sam

Chromosome undef

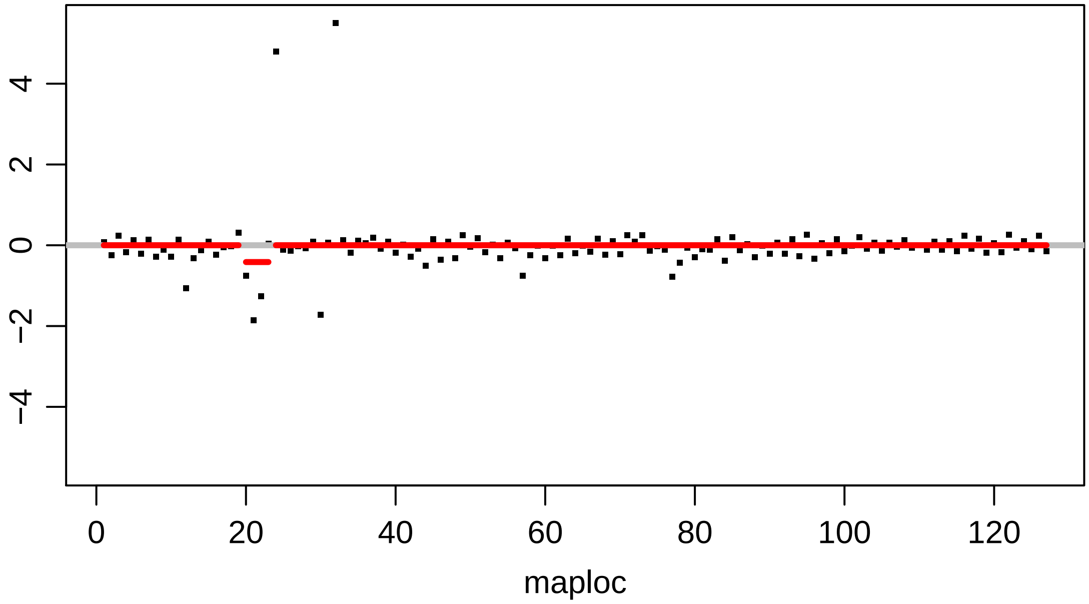


```
## Segplot might not work because of special characters in the sample names. Use only A-Z,a-z and 0-9!
##  There is a hidden function cn.mops:::.replaceNames that replaces the names in the "CNVDetectionResu
```

Case_IP181.G2.sam

Chromosome undef

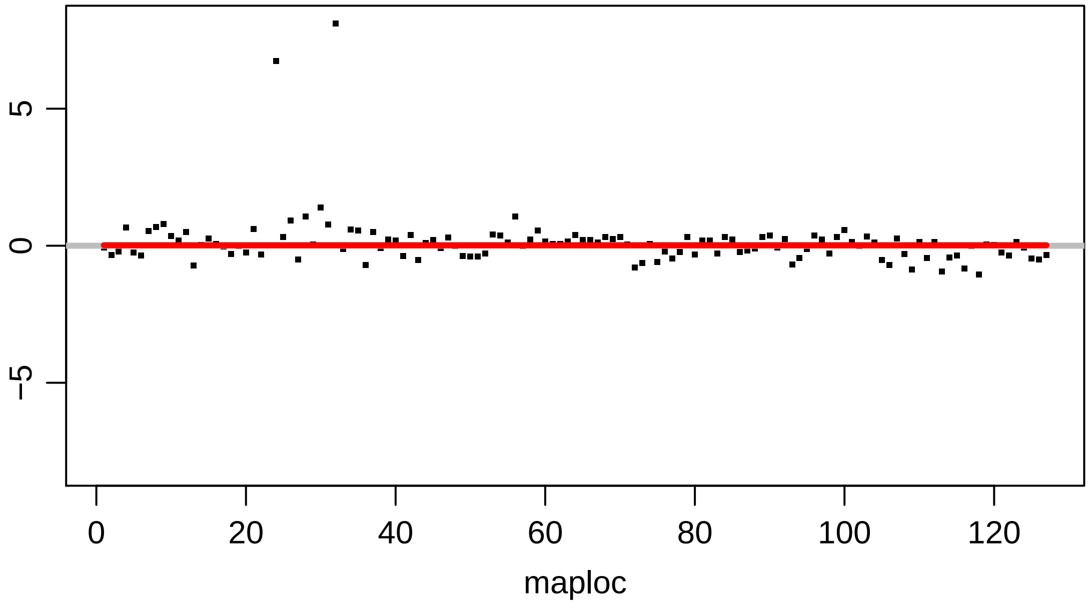

```
## Segplot might not work because of special characters in the sample names. Use only A-Z,a-z and 0-9!
##  There is a hidden function cn.mops:::.replaceNames that replaces the names in the "CNVDetectionResul
```

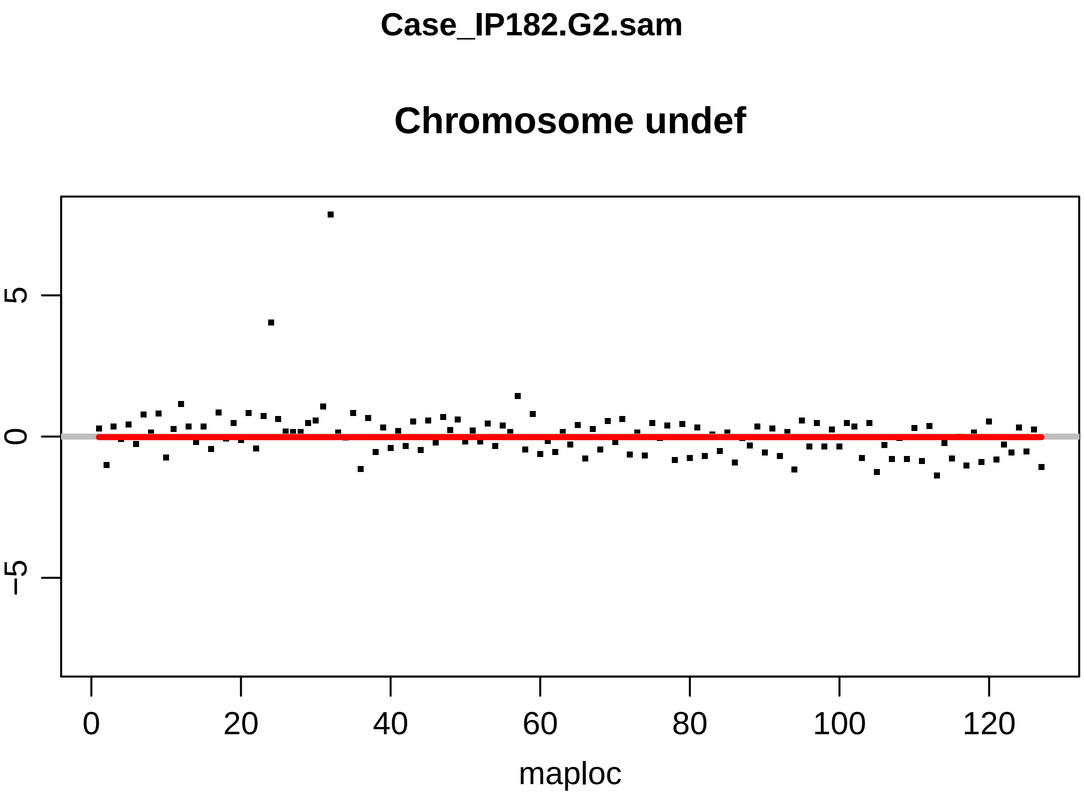


```
## Segplot might not work because of special characters in the sample names. Use only A-Z,a-z and 0-9!
##  There is a hidden function cn.mops:::.replaceNames that replaces the names in the "CNVDetectionResul
```

Case_IP183.G2.sam

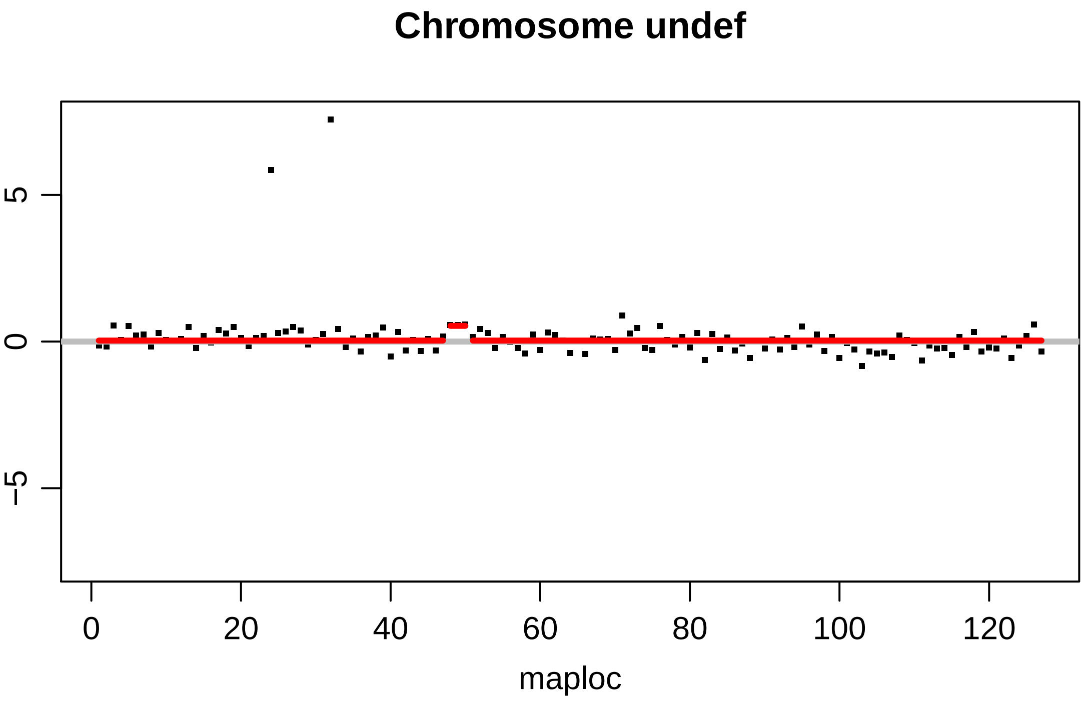


```
## Segplot might not work because of special characters in the sample names. Use only A-Z,a-z and 0-9!
##  There is a hidden function cn.mops:::.replaceNames that replaces the names in the "CNVDetectionResul
```

Case_IP185.G2.sam

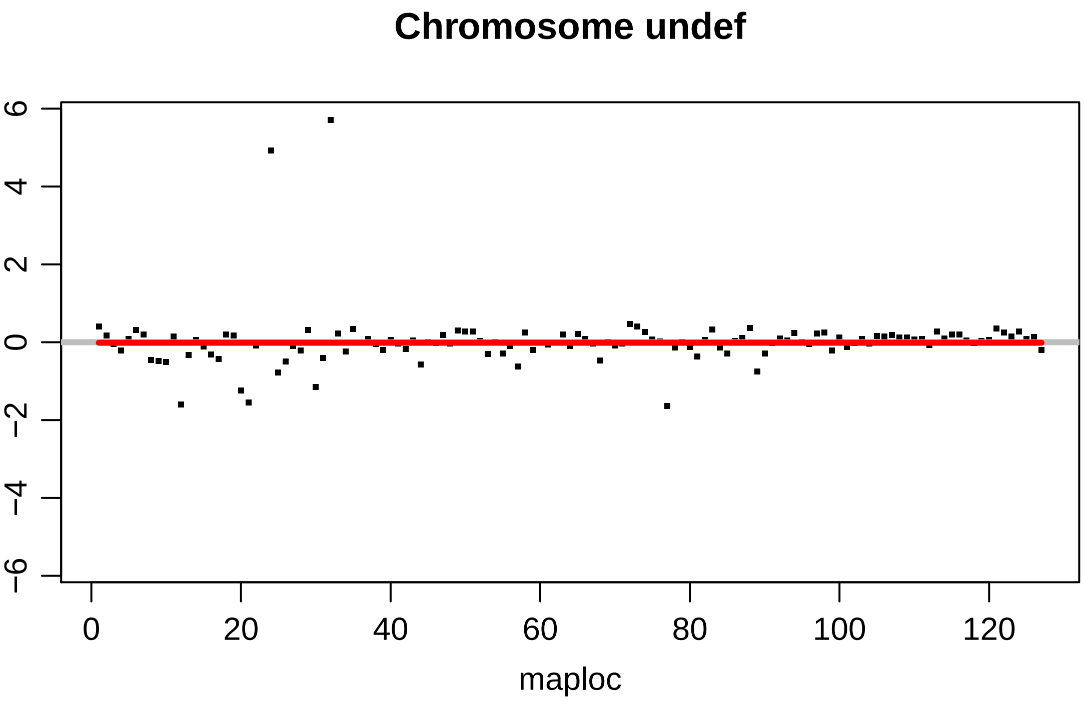

```
## Segplot might not work because of special characters in the sample names. Use only A-Z,a-z and 0-9!
##  There is a hidden function cn.mops:::.replaceNames that replaces the names in the "CNVDetectionResul
```

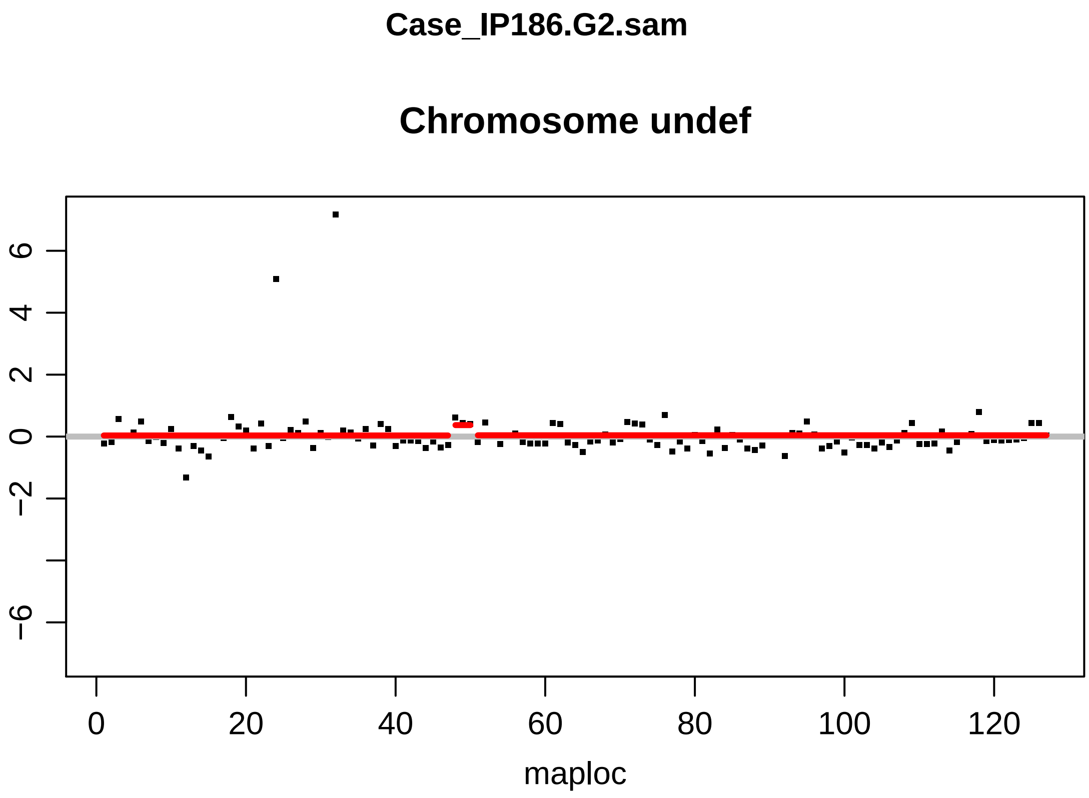


```
## Segplot might not work because of special characters in the sample names. Use only A-Z,a-z and 0-9!
##  There is a hidden function cn.mops:::.replaceNames that replaces the names in the "CNVDetectionResul
```

Case_IP187.G2.sam

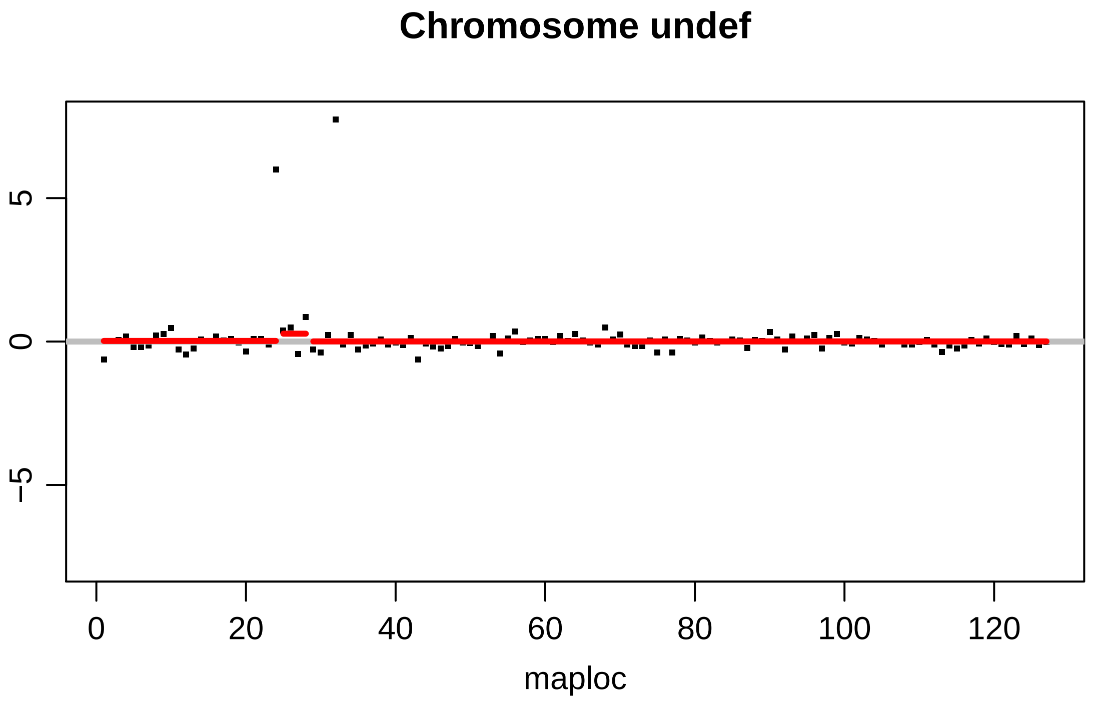


```
##
## CNV regions:
## GRanges object with 2 ranges and 39 metadata columns:
##       seqnames    ranges strand | Case_IP142.G1.sam Case_IP143.G1.sam
##          <Rle> <IRanges>  <Rle> |          <factor>          <factor>
##   [1]    undef  [26, 28]      * |               CN2               CN2
##   [2]    undef  [48, 50]      * |               CN2               CN2
##       Case_IP144.G1.sam Case_IP145.G1.sam Case_IP146.G1.sam
##                <factor>          <factor>          <factor>
##   [1]               CN2               CN2               CN2
##   [2]               CN2               CN2               CN2
##       Case_IP148.G1.sam Case_IP149.G1.sam Case_IP150.G1.sam
##                <factor>          <factor>          <factor>
##   [1]               CN2               CN2               CN2
##   [2]               CN2               CN2               CN2
##       Case_IP151.G1.sam Case_IP152.G1.sam Case_IP154.G1.sam
##                <factor>          <factor>          <factor>
##   [1]               CN2               CN2               CN2
##   [2]               CN2               CN2               CN2
##       Case_IP155.G2.sam Case_IP156.G2.sam Case_IP158.G2.sam
##                <factor>          <factor>          <factor>
##   [1]               CN3               CN2               CN2
##   [2]               CN2               CN2               CN2
##       Case_IP160.G2.sam Case_IP161.G2.sam Case_IP162.G2.sam
##                <factor>          <factor>          <factor>
##   [1]               CN2               CN2               CN2
##   [2]               CN2               CN2               CN2
##       Case_IP164.G2.sam Case_IP165.G2.sam Case_IP167.G2.sam
```

```
##                <factor>          <factor>          <factor>
##   [1]               CN2               CN2               CN2
##   [2]               CN2               CN2               CN2
##       Case_IP168.G2.sam Case_IP169.G2.sam Case_IP170.G2.sam
##                <factor>          <factor>          <factor>
##   [1]               CN2               CN2               CN2
##   [2]               CN2               CN2               CN2
##       Case_IP171.G2.sam Case_IP172.G2.sam Case_IP173.G2.sam
##                <factor>          <factor>          <factor>
##   [1]               CN2               CN2               CN2
##   [2]               CN2               CN2               CN2
##       Case_IP174.G2.sam Case_IP175.G2.sam Case_IP176.G2.sam
##                <factor>          <factor>          <factor>
##   [1]               CN2               CN2               CN2
##   [2]               CN3               CN3               CN2
##       Case_IP177.G2.sam Case_IP178.G2.sam Case_IP179.G2.sam
##                <factor>          <factor>          <factor>
##   [1]               CN2               CN2               CN2
##   [2]               CN2               CN2               CN2
##       Case_IP181.G2.sam Case_IP182.G2.sam Case_IP183.G2.sam
##                <factor>          <factor>          <factor>
##   [1]               CN2               CN2               CN2
##   [2]               CN2               CN2               CN3
##       Case_IP185.G2.sam Case_IP186.G2.sam Case_IP187.G2.sam
##                <factor>          <factor>          <factor>
##   [1]               CN2               CN2               CN2
##   [2]               CN2               CN2               CN2
##       Case_IP188.G2.sam
##                <factor>
##   [1]               CN2
##   [2]               CN2
##   -------
##   seqinfo: 1 sequence from an unspecified genome; no seqlengths
##
## Individual CNVs:
## GRanges object with 4 ranges and 4 metadata columns:
##       seqnames    ranges strand |        sampleName    median      mean
##          <Rle> <IRanges>  <Rle> |          <factor> <numeric> <numeric>
##   [1]    undef  [26, 28]      * | Case_IP155.G2.sam 0.8476066 0.6145870
##   [2]    undef  [48, 50]      * | Case_IP174.G2.sam 0.5534209 0.5542399
##   [3]    undef  [48, 50]      * | Case_IP175.G2.sam 0.5732298 0.5670166
##   [4]    undef  [48, 50]      * | Case_IP183.G2.sam 0.5193413 0.5356356
##                CN
##       <character>
##   [1]         CN3
##   [2]         CN3
##   [3]         CN3
##   [4]         CN3
##   -------
##   seqinfo: 1 sequence from an unspecified genome; no seqlengths
## [1] "/Users/gdemidov/Downloads/doc/Run_second_run_fin_05_qc.xls"

## Normalizing...
```

```
## Starting local modeling, please be patient...

## Reference sequence:  undef

## Starting segmentation algorithm...

## Using "fastseg" for segmentation.

## [1] ""
## [1] "/Users/gdemidov/Downloads/doc/Run_second_run_fin_05_qc.xls"
## [1] ""

## Segplot might not work because of special characters in the sample names. Use only A-Z,a-z and 0-9!
##  There is a hidden function cn.mops:::.replaceNames that replaces the names in the "CNVDetectionResul
```

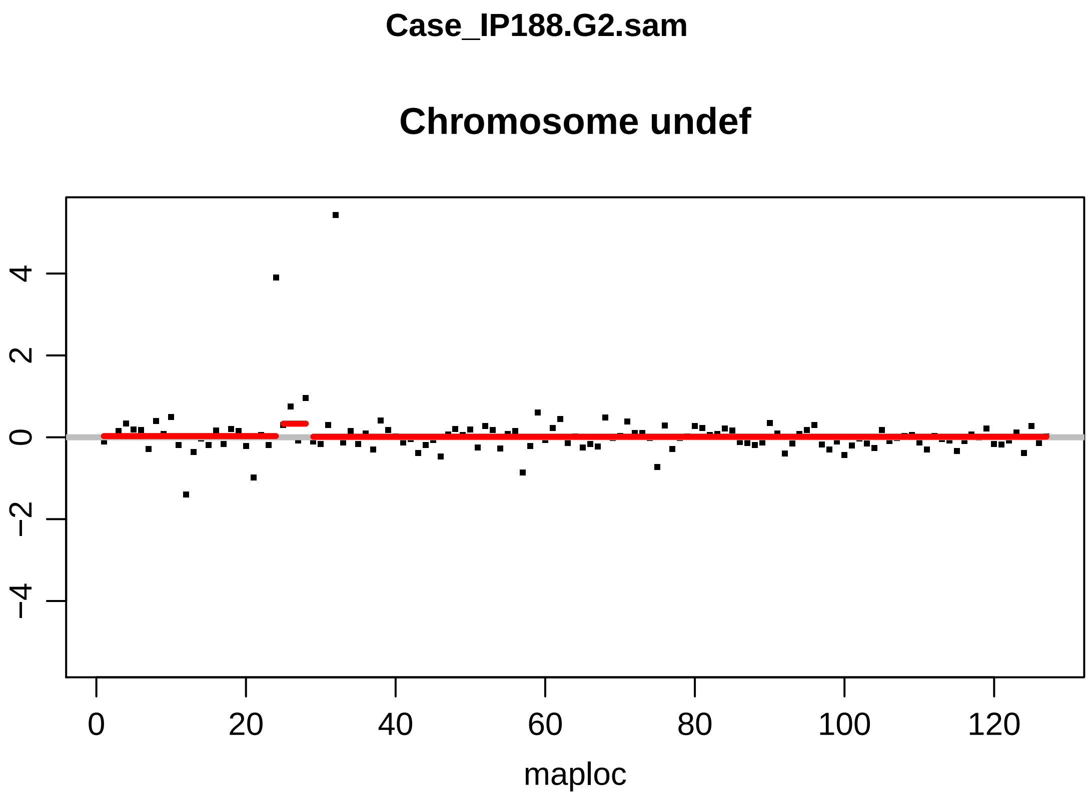


```
## Segplot might not work because of special characters in the sample names. Use only A-Z,a-z and 0-9!
##  There is a hidden function cn.mops:::.replaceNames that replaces the names in the "CNVDetectionResul
```

Case_IP048.G1.sam

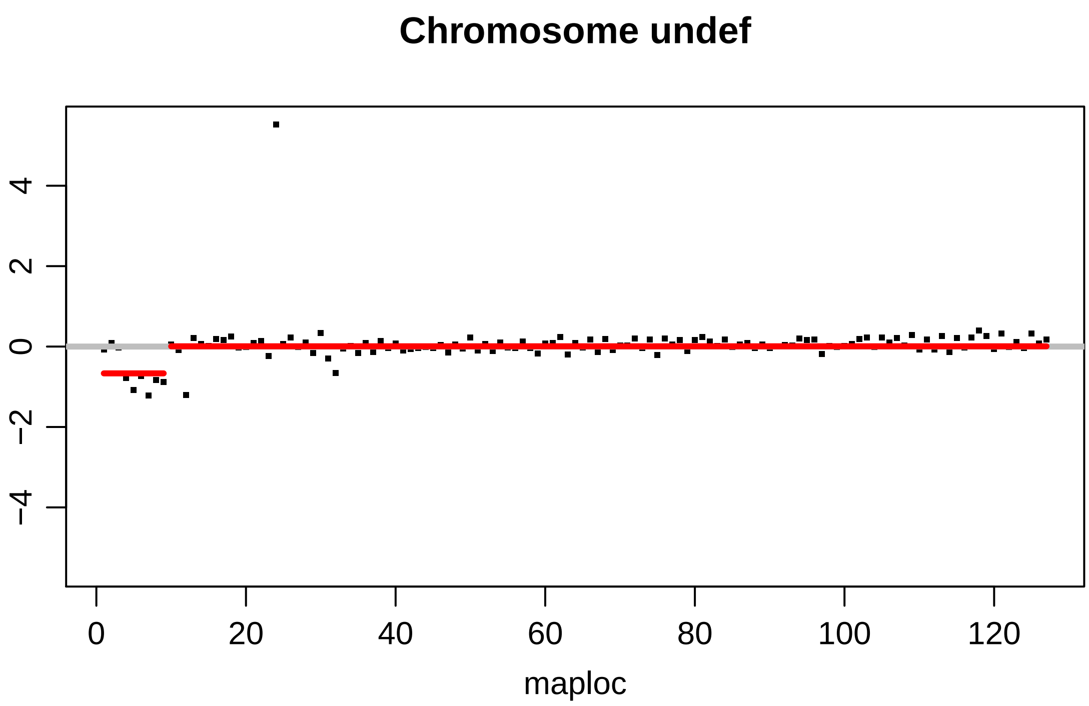


```
## Segplot might not work because of special characters in the sample names. Use only A-Z,a-z and 0-9!
##  There is a hidden function cn.mops:::.replaceNames that replaces the names in the "CNVDetectionResul
```

Case_IP049.G1.sam

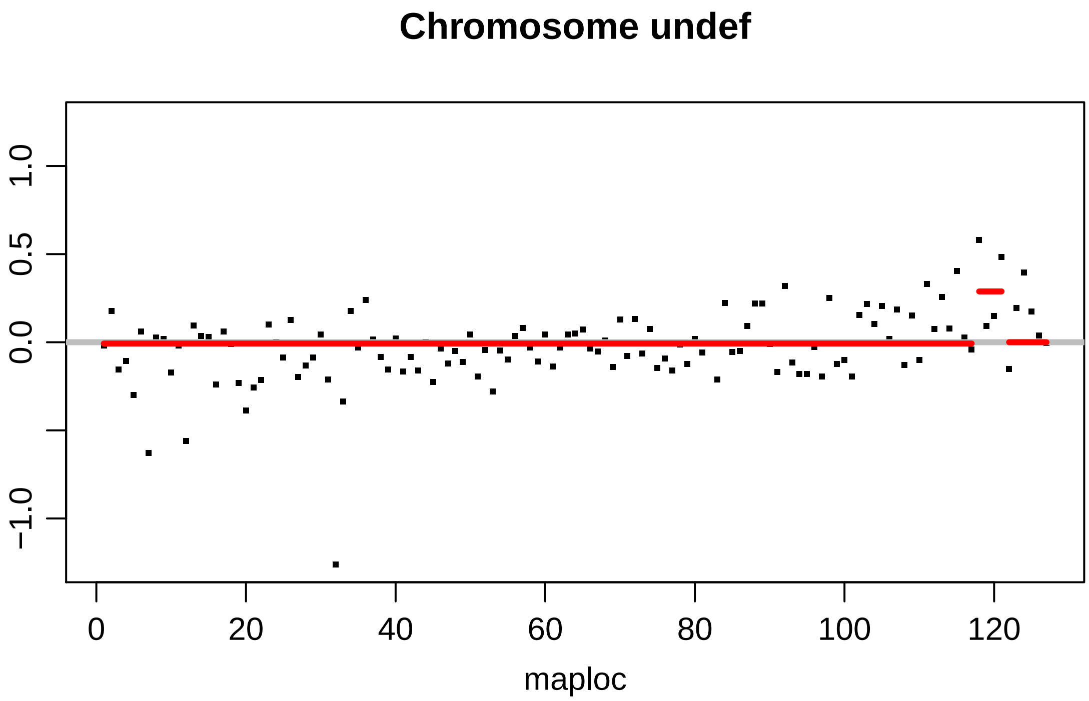

```
## Segplot might not work because of special characters in the sample names. Use only A-Z,a-z and 0-9!
##  There is a hidden function cn.mops:::.replaceNames that replaces the names in the "CNVDetectionResul
```

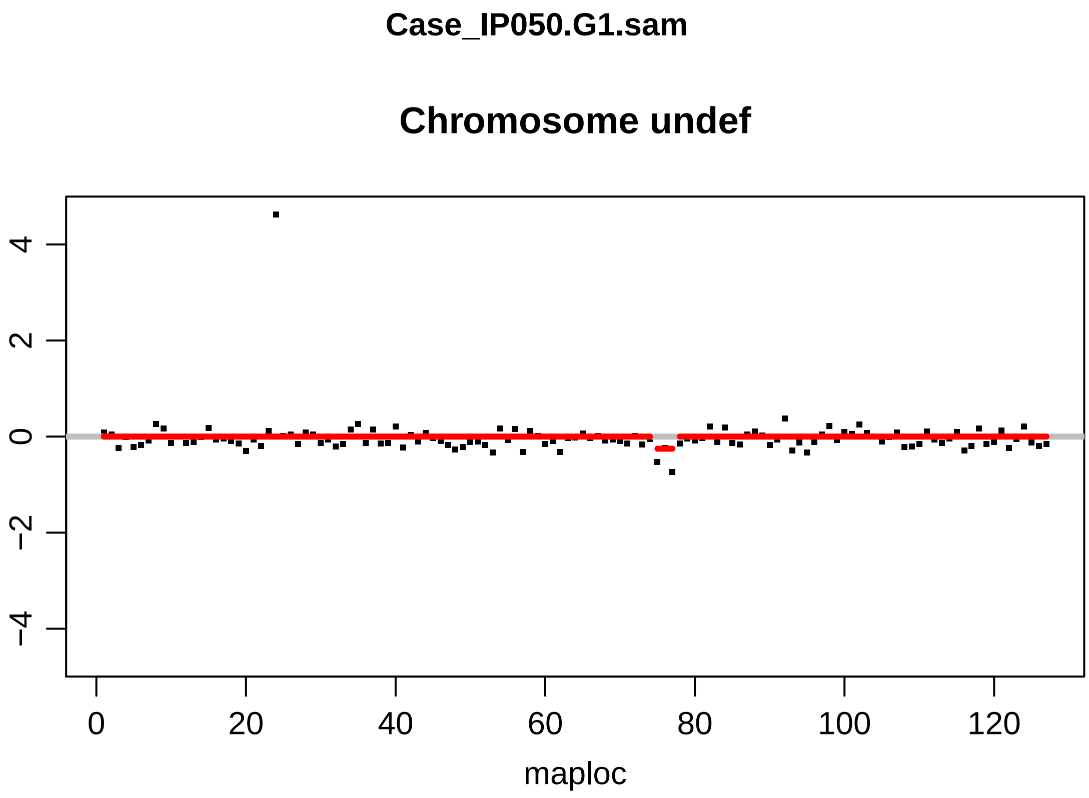


```
## Segplot might not work because of special characters in the sample names. Use only A-Z,a-z and 0-9!
##  There is a hidden function cn.mops:::.replaceNames that replaces the names in the "CNVDetectionResul
```

Case_IP051.G1.sam

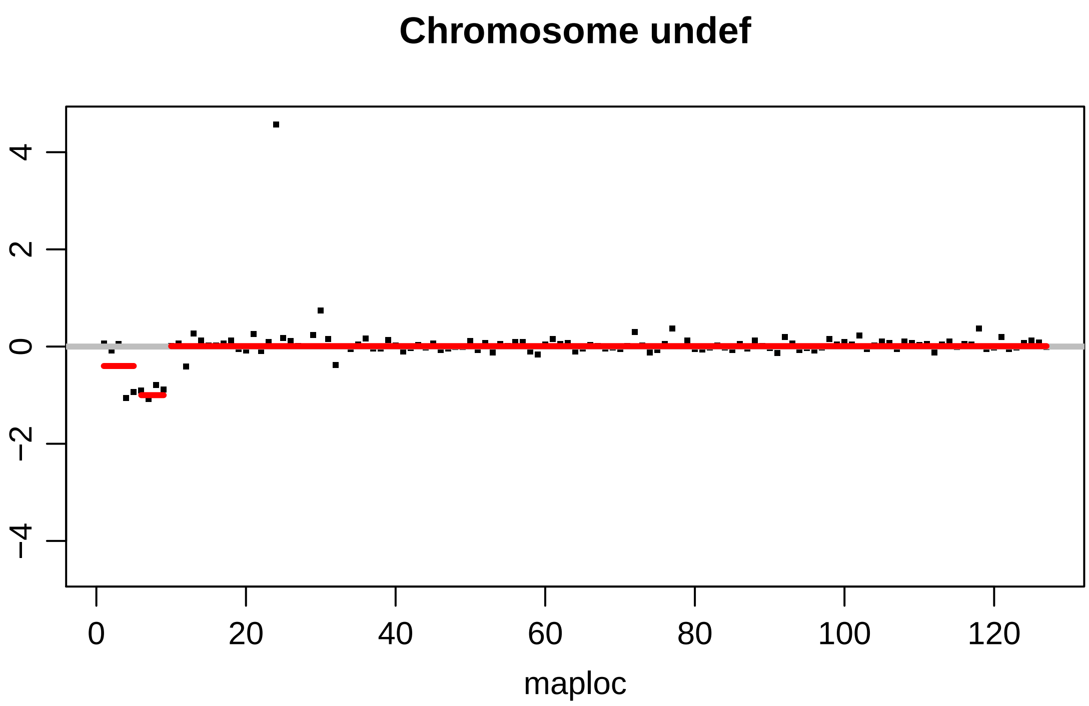


```
## Segplot might not work because of special characters in the sample names. Use only A-Z,a-z and 0-9!
##  There is a hidden function cn.mops:::.replaceNames that replaces the names in the "CNVDetectionResul
```

Case_IP052.G1.sam

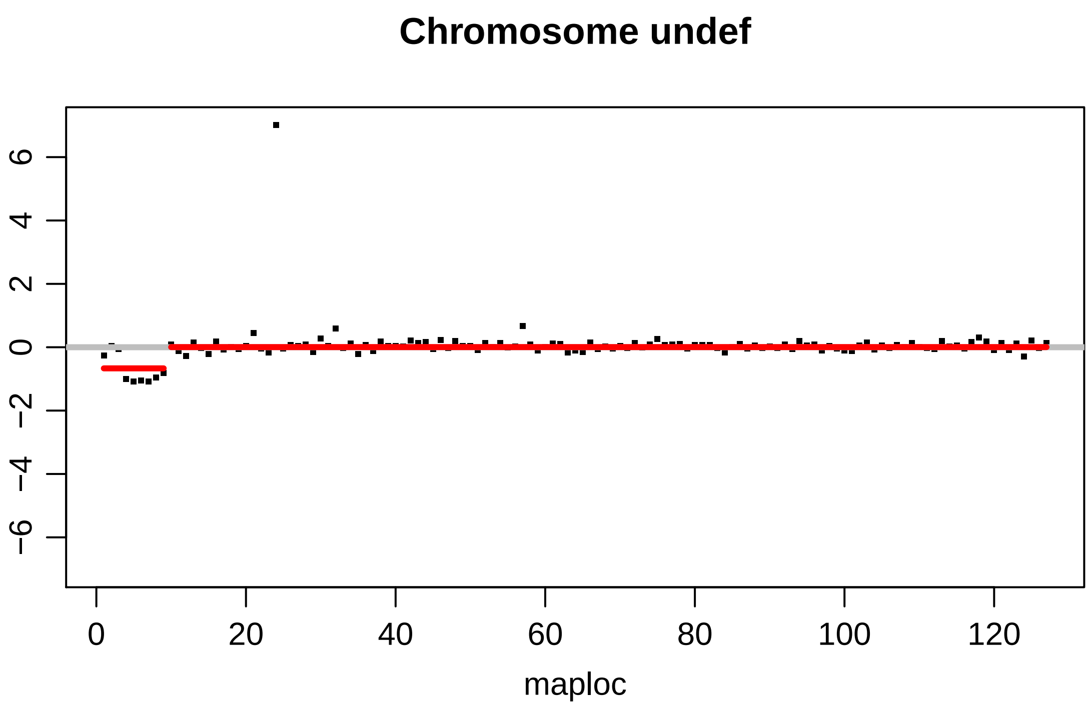

```
## Segplot might not work because of special characters in the sample names. Use only A-Z,a-z and 0-9!
##  There is a hidden function cn.mops:::.replaceNames that replaces the names in the "CNVDetectionResul
```

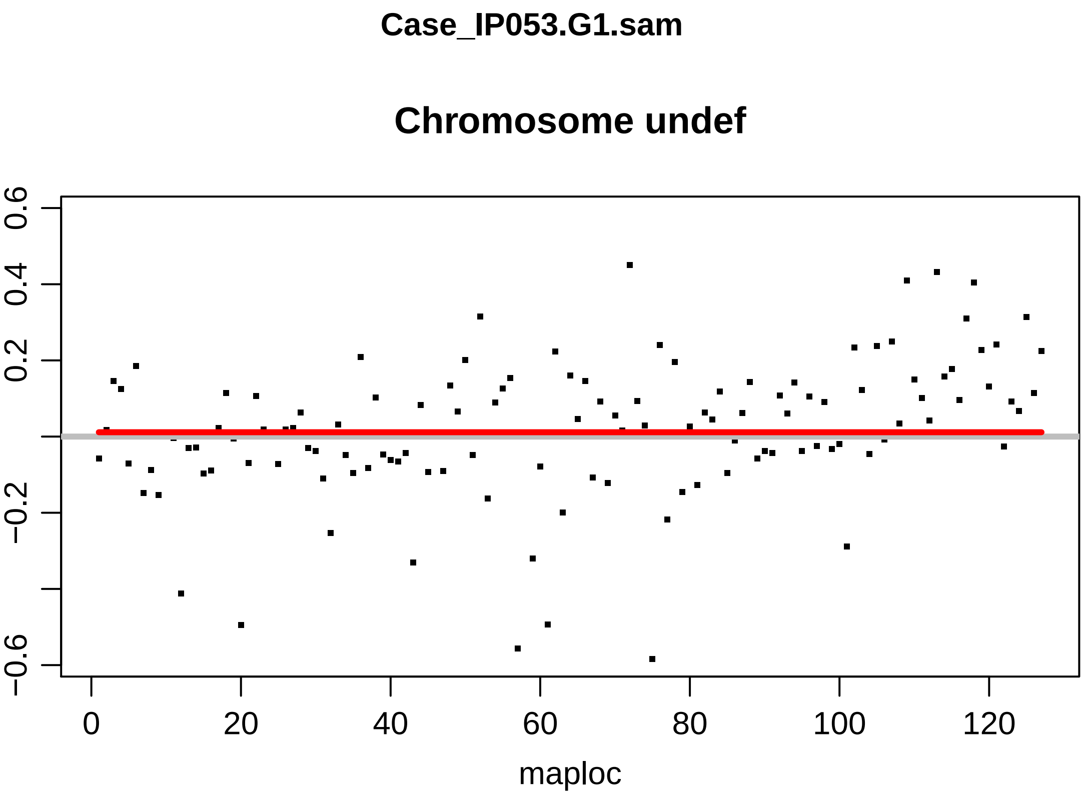


```
## Segplot might not work because of special characters in the sample names. Use only A-Z,a-z and 0-9!
##  There is a hidden function cn.mops:::.replaceNames that replaces the names in the "CNVDetectionResul
```

Case_IP054.G1.sam

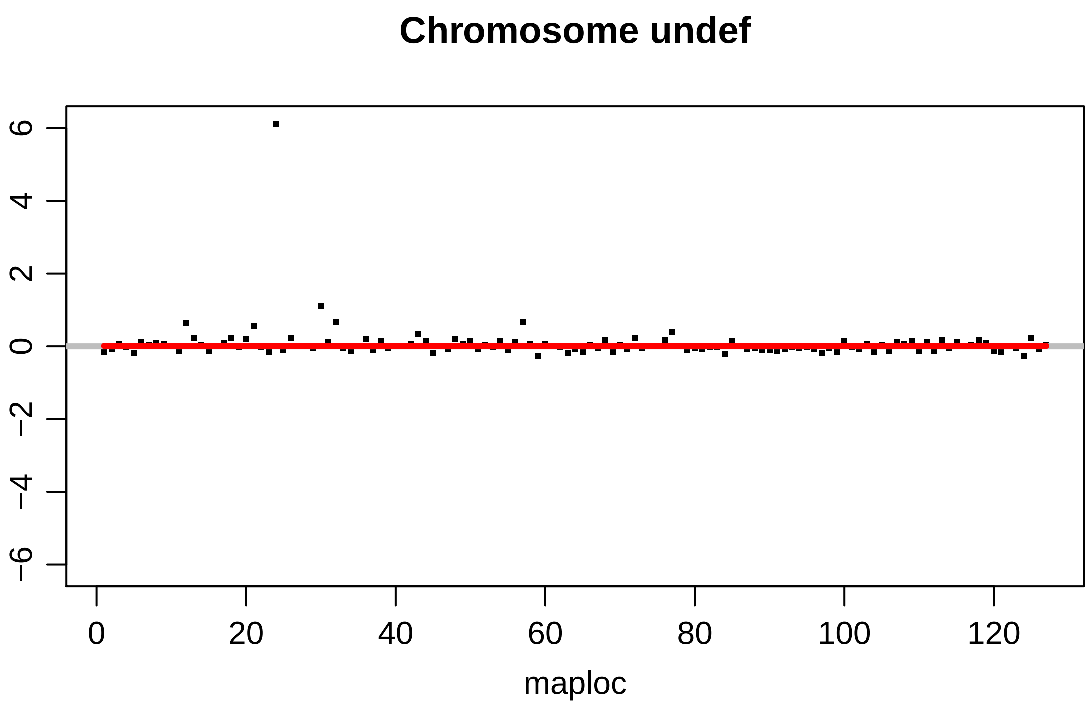


```
## Segplot might not work because of special characters in the sample names. Use only A-Z,a-z and 0-9!
##  There is a hidden function cn.mops:::.replaceNames that replaces the names in the "CNVDetectionResul
```

Case_IP055.G1.sam

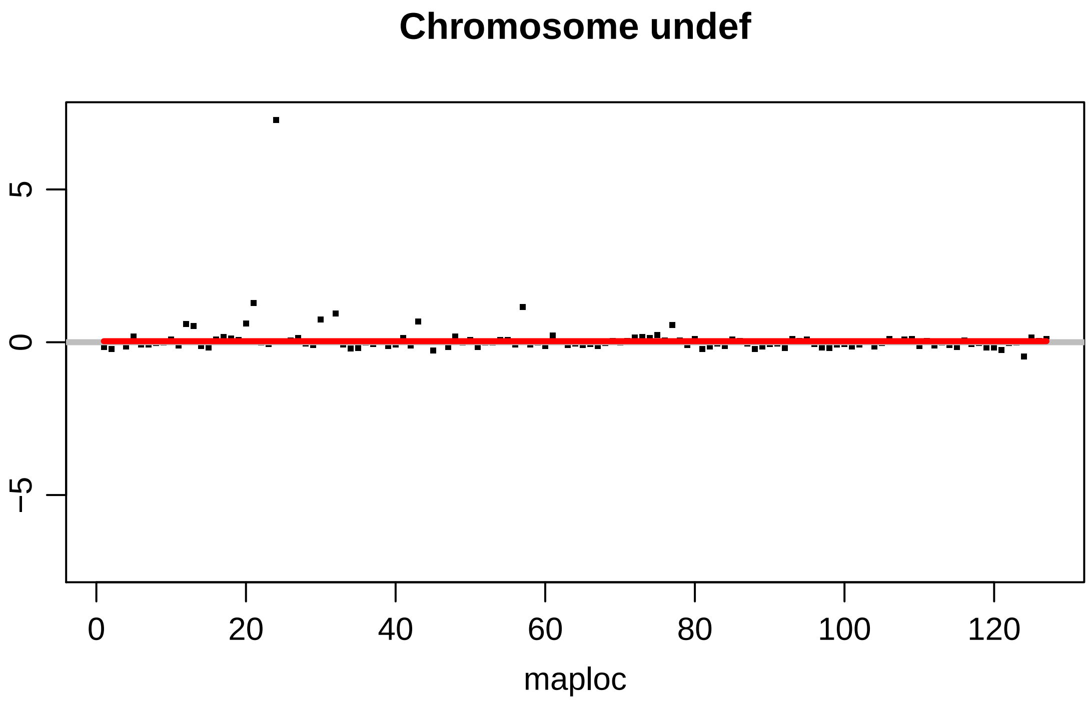

```
## Segplot might not work because of special characters in the sample names. Use only A-Z,a-z and 0-9!
##  There is a hidden function cn.mops:::.replaceNames that replaces the names in the "CNVDetectionResul
```

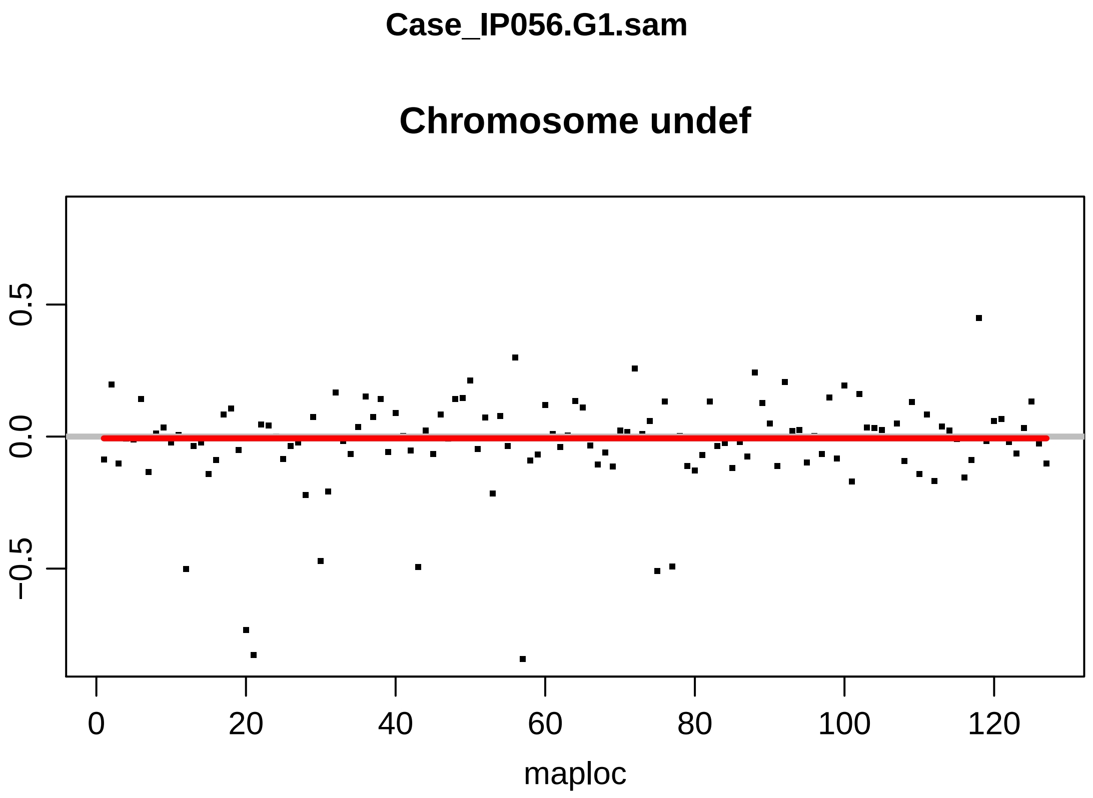


```
## Segplot might not work because of special characters in the sample names. Use only A-Z,a-z and 0-9!
##  There is a hidden function cn.mops:::.replaceNames that replaces the names in the "CNVDetectionResul
```

Case_IP057.G1.sam

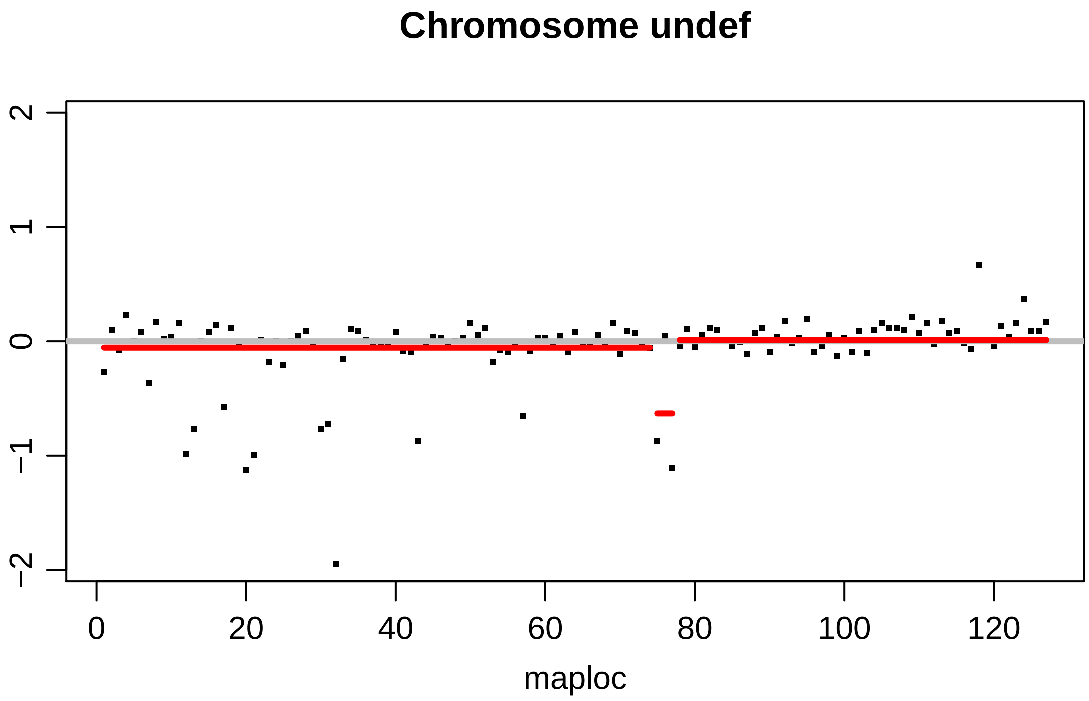


```
## Segplot might not work because of special characters in the sample names. Use only A-Z,a-z and 0-9!
##  There is a hidden function cn.mops:::.replaceNames that replaces the names in the "CNVDetectionResul
```

Case_IP058.G1.sam

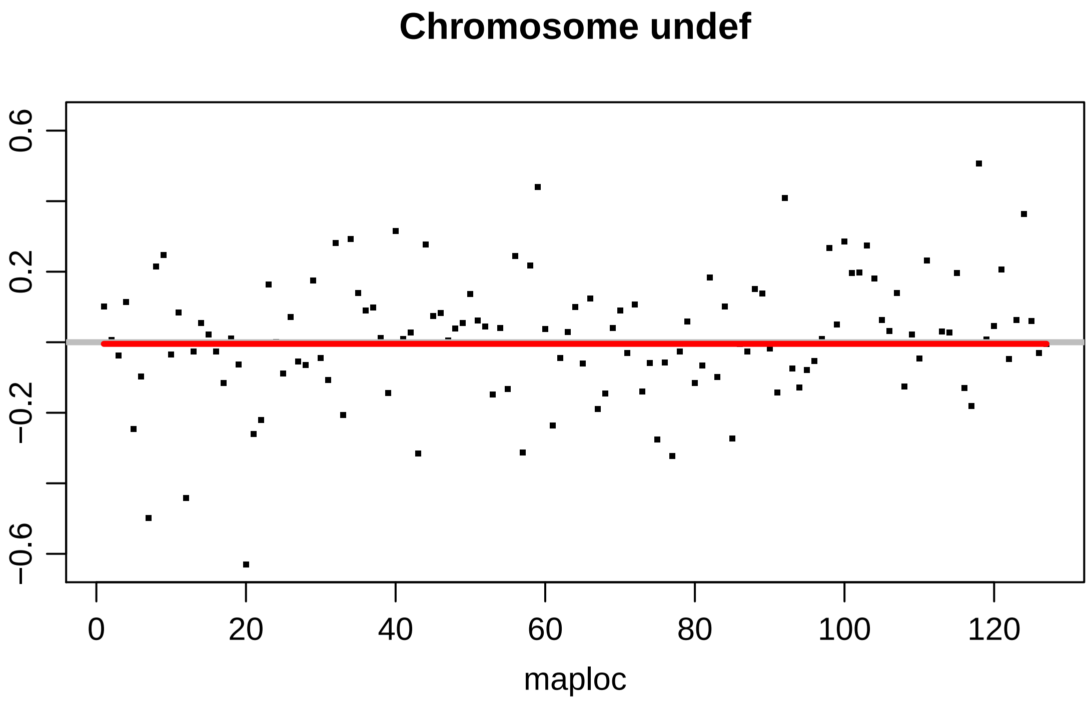

```
## Segplot might not work because of special characters in the sample names. Use only A-Z,a-z and 0-9!
##  There is a hidden function cn.mops:::.replaceNames that replaces the names in the "CNVDetectionResul
```

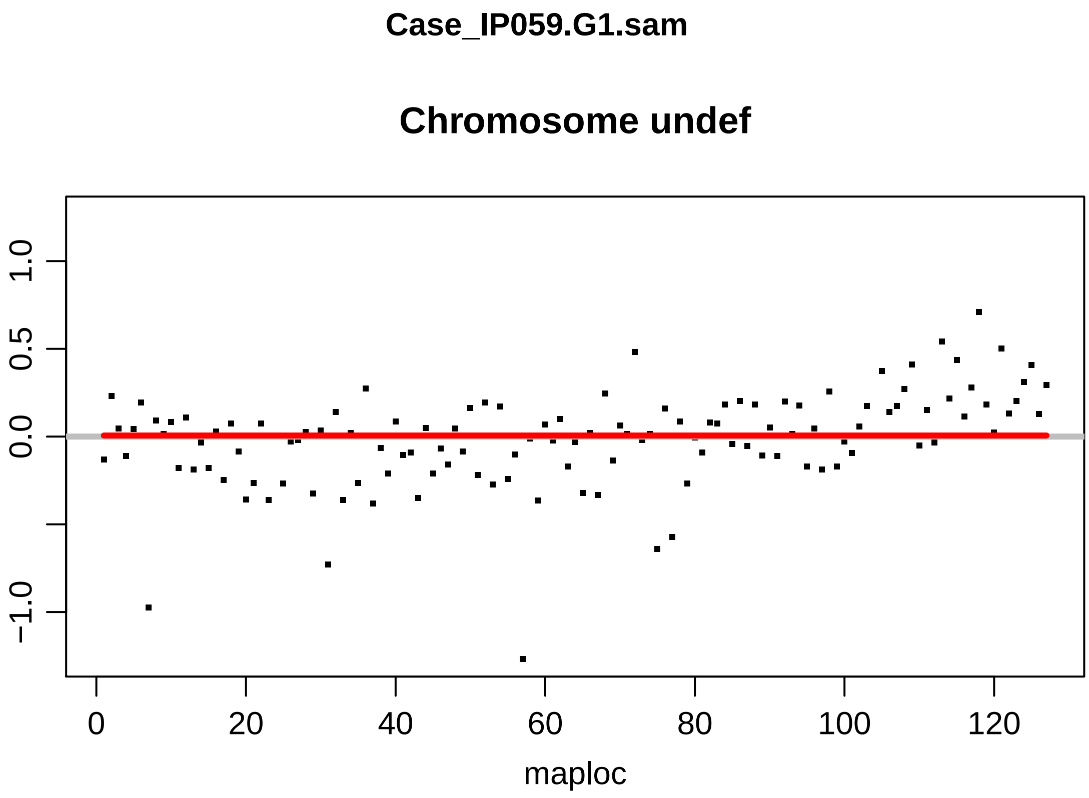


```
## Segplot might not work because of special characters in the sample names. Use only A-Z,a-z and 0-9!
##  There is a hidden function cn.mops:::.replaceNames that replaces the names in the "CNVDetectionResul
```

Case_IP060.G1.sam

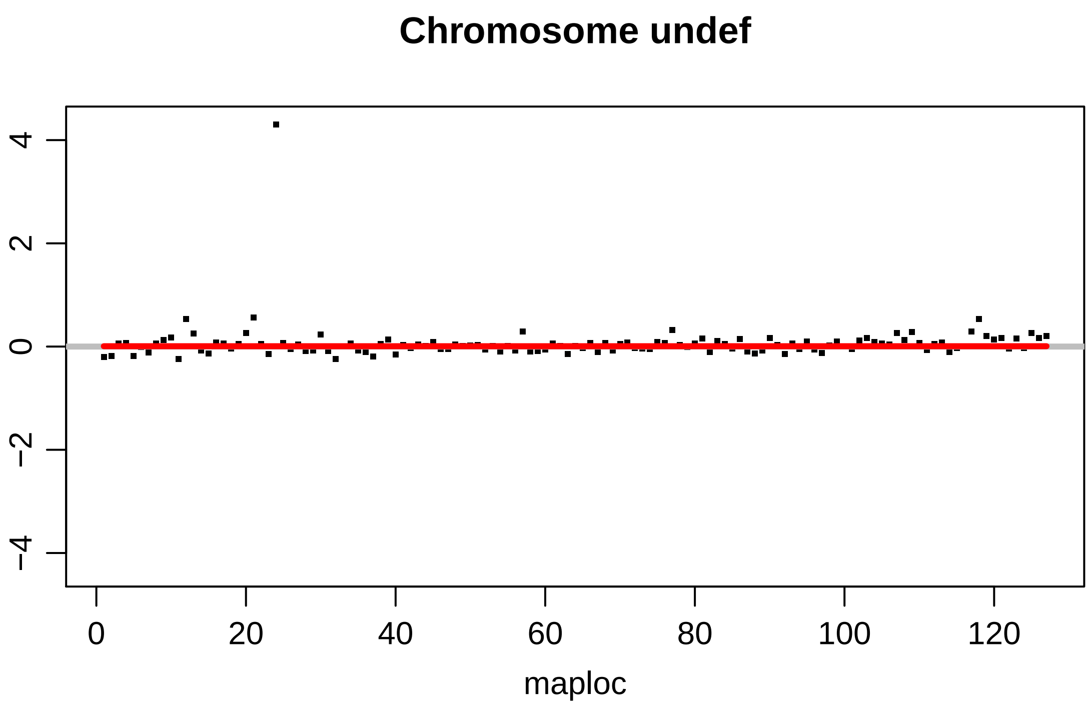


```
## Segplot might not work because of special characters in the sample names. Use only A-Z,a-z and 0-9!
##  There is a hidden function cn.mops:::.replaceNames that replaces the names in the "CNVDetectionResul
```

Case_IP061.G1.sam

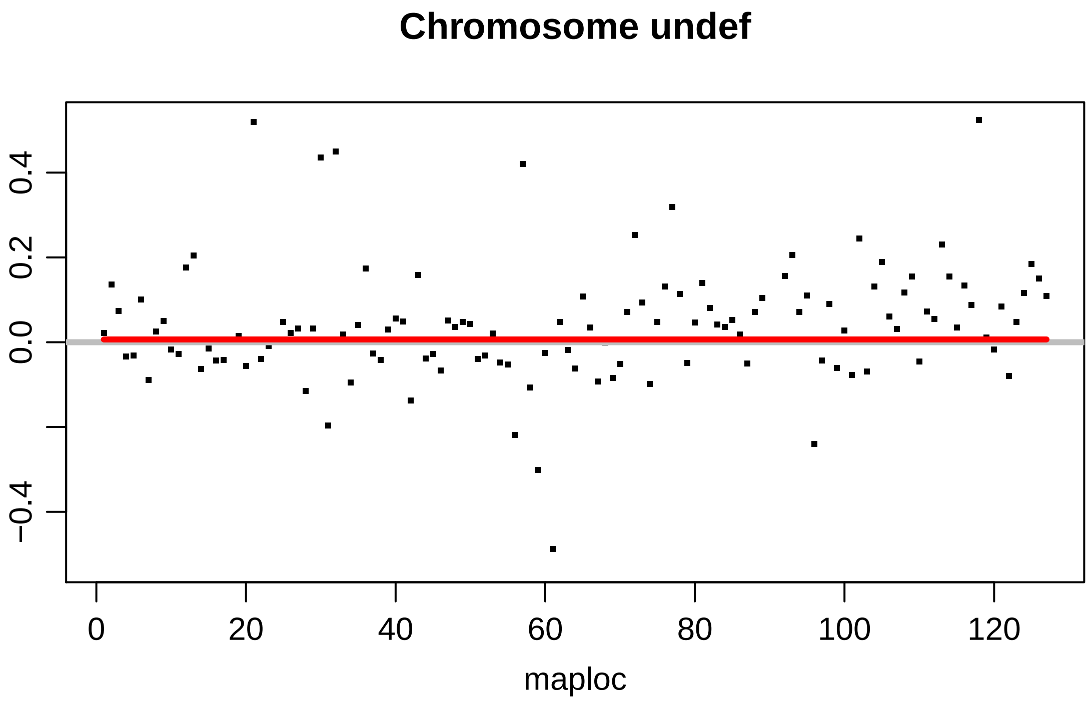

```
## Segplot might not work because of special characters in the sample names. Use only A-Z,a-z and 0-9!
##  There is a hidden function cn.mops:::.replaceNames that replaces the names in the "CNVDetectionResul
```

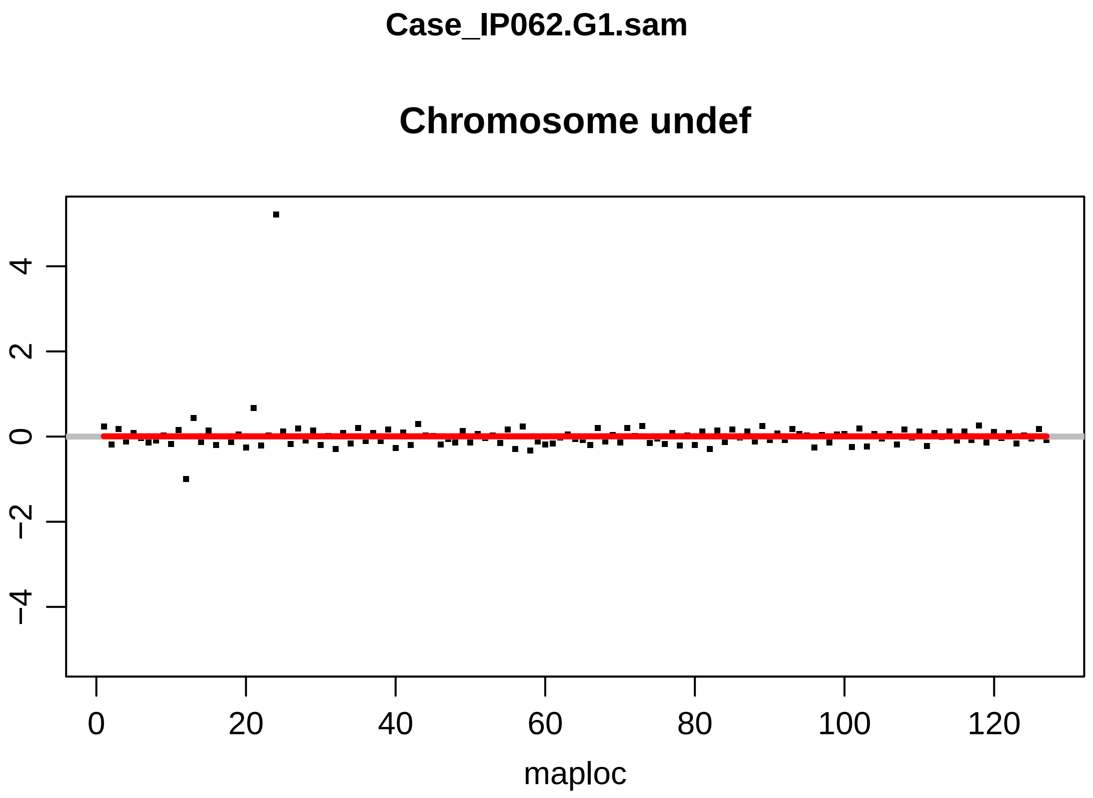


```
## Segplot might not work because of special characters in the sample names. Use only A-Z,a-z and 0-9!
##  There is a hidden function cn.mops:::.replaceNames that replaces the names in the "CNVDetectionResul
```

Case_IP063.G1.sam

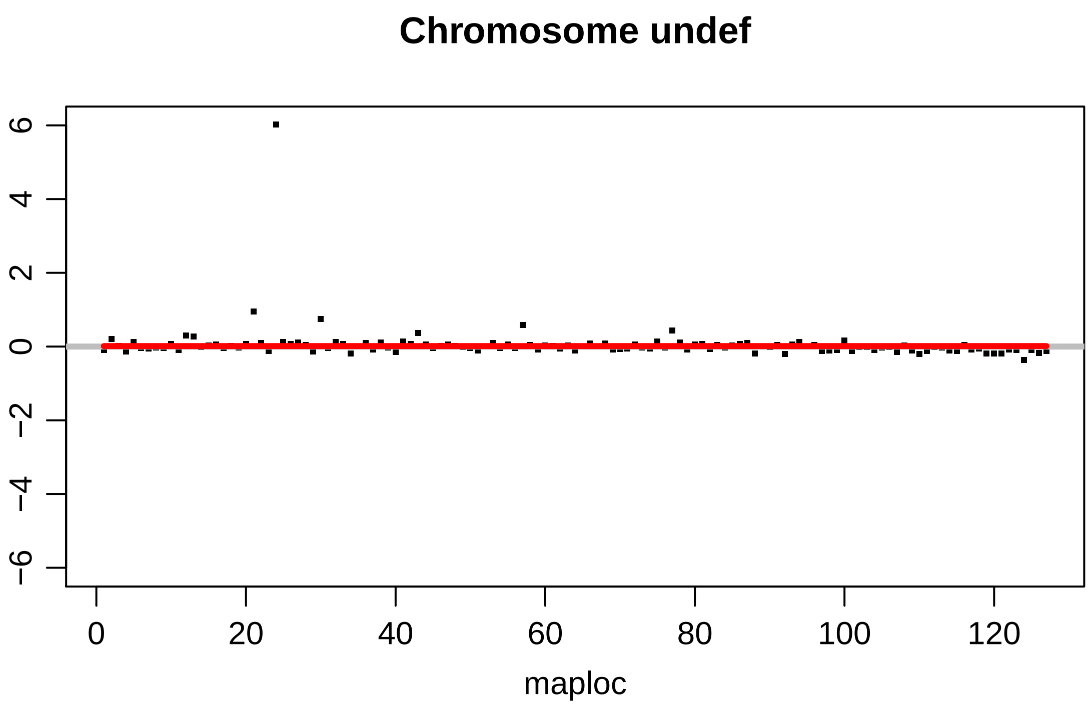


```
## Segplot might not work because of special characters in the sample names. Use only A-Z,a-z and 0-9!
##  There is a hidden function cn.mops:::.replaceNames that replaces the names in the "CNVDetectionResul
```

Case_IP065.G1.sam

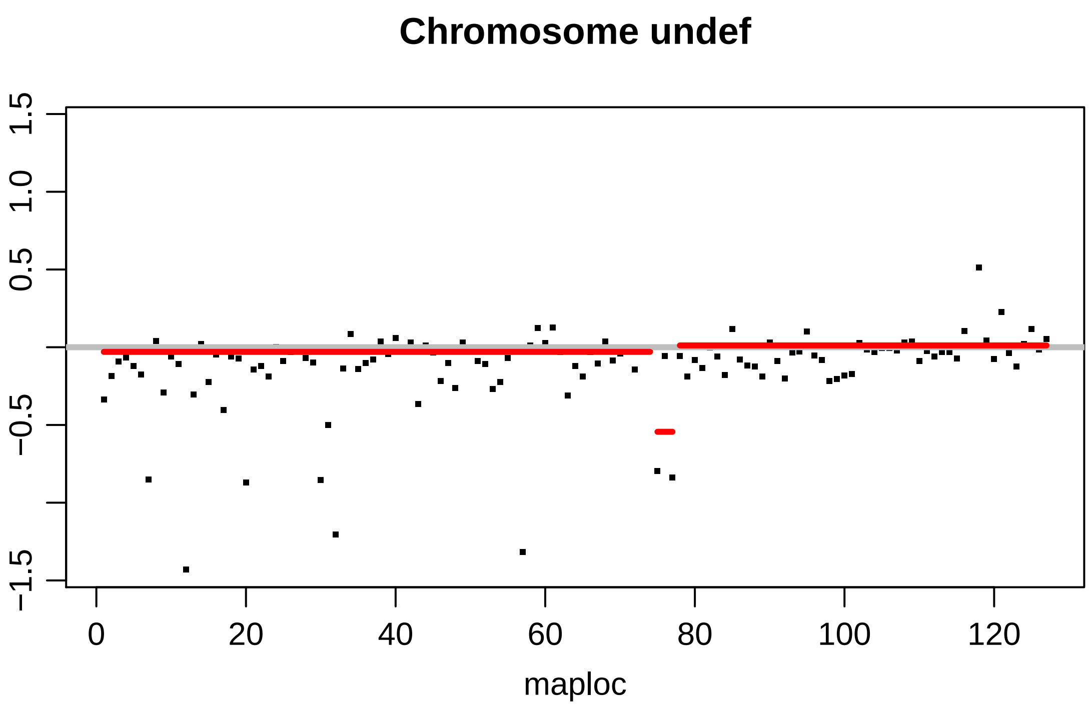

```
## Segplot might not work because of special characters in the sample names. Use only A-Z,a-z and 0-9!
##  There is a hidden function cn.mops:::.replaceNames that replaces the names in the "CNVDetectionResul
```

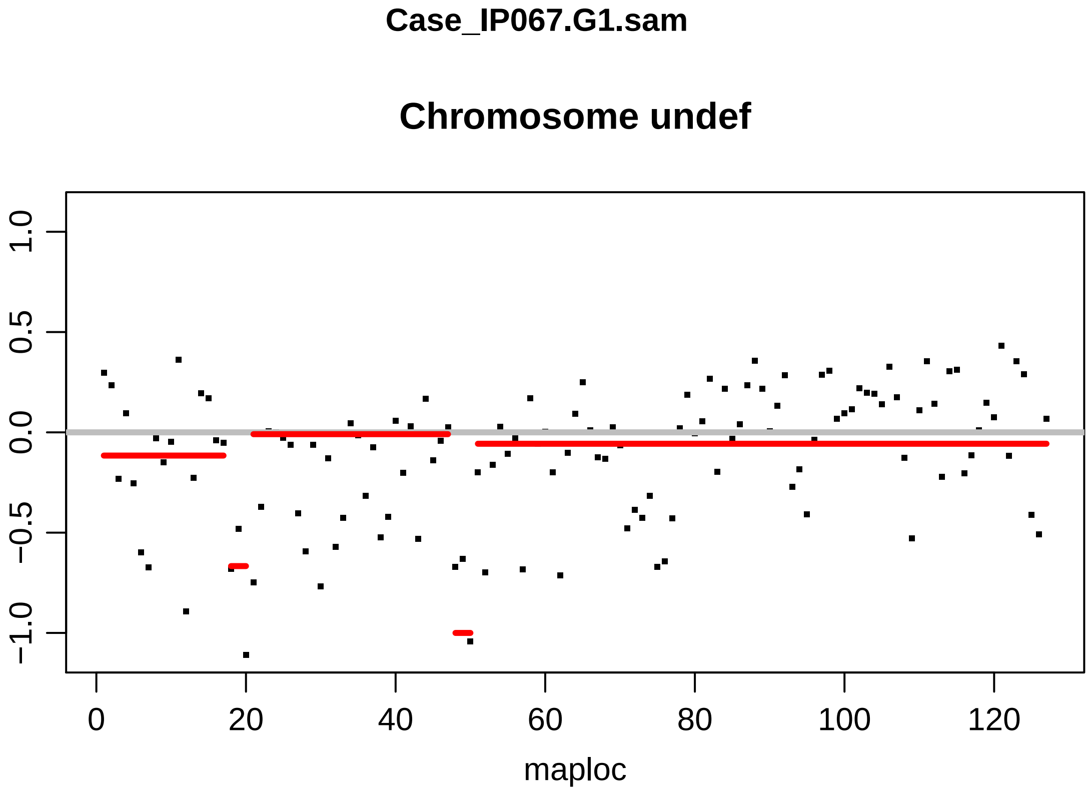


```
## Segplot might not work because of special characters in the sample names. Use only A-Z,a-z and 0-9!
##  There is a hidden function cn.mops:::.replaceNames that replaces the names in the "CNVDetectionResul
```

Case_IP068.G1.sam

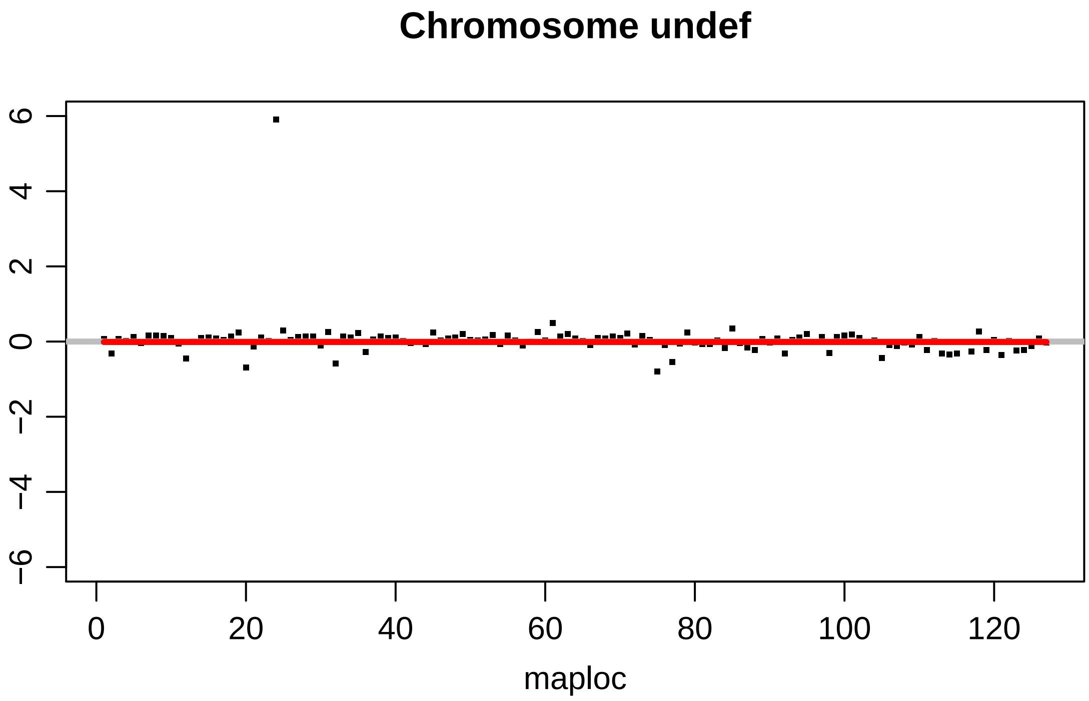


```
## Segplot might not work because of special characters in the sample names. Use only A-Z,a-z and 0-9!
##  There is a hidden function cn.mops:::.replaceNames that replaces the names in the "CNVDetectionResul
```

Case_IP069.G1.sam

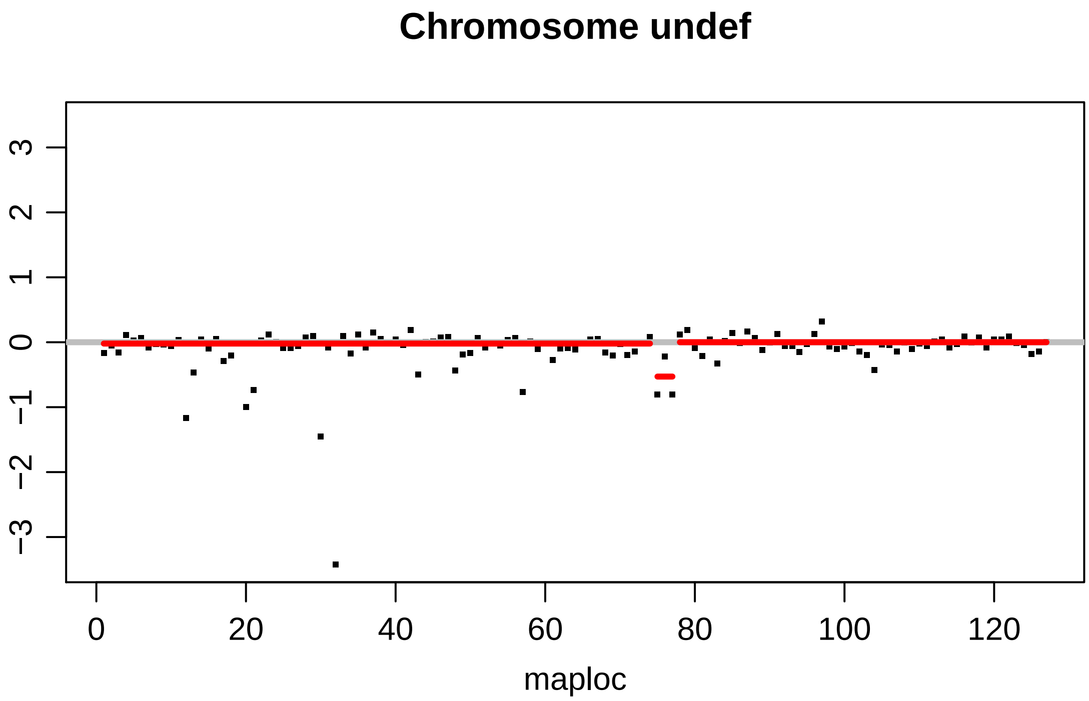

```
## Segplot might not work because of special characters in the sample names. Use only A-Z,a-z and 0-9!
##  There is a hidden function cn.mops:::.replaceNames that replaces the names in the "CNVDetectionResul
```

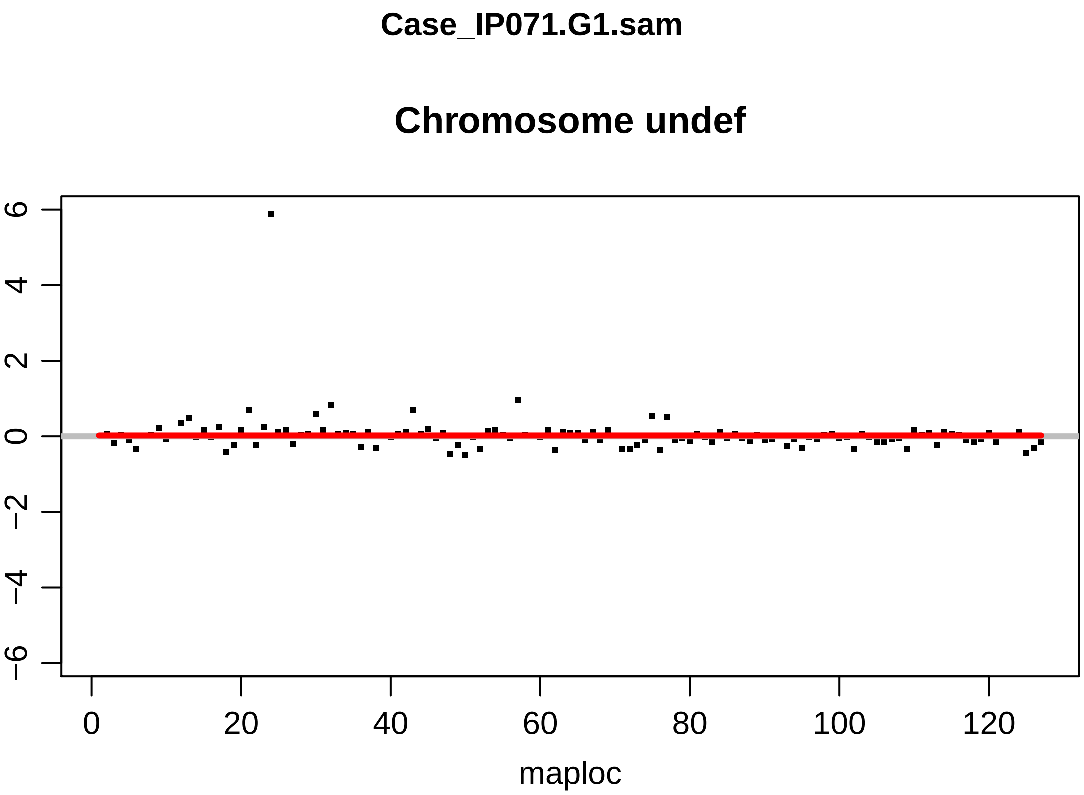


```
## Segplot might not work because of special characters in the sample names. Use only A-Z,a-z and 0-9!
##  There is a hidden function cn.mops:::.replaceNames that replaces the names in the "CNVDetectionResul
```

Case_IP072.G1.sam

Chromosome undef

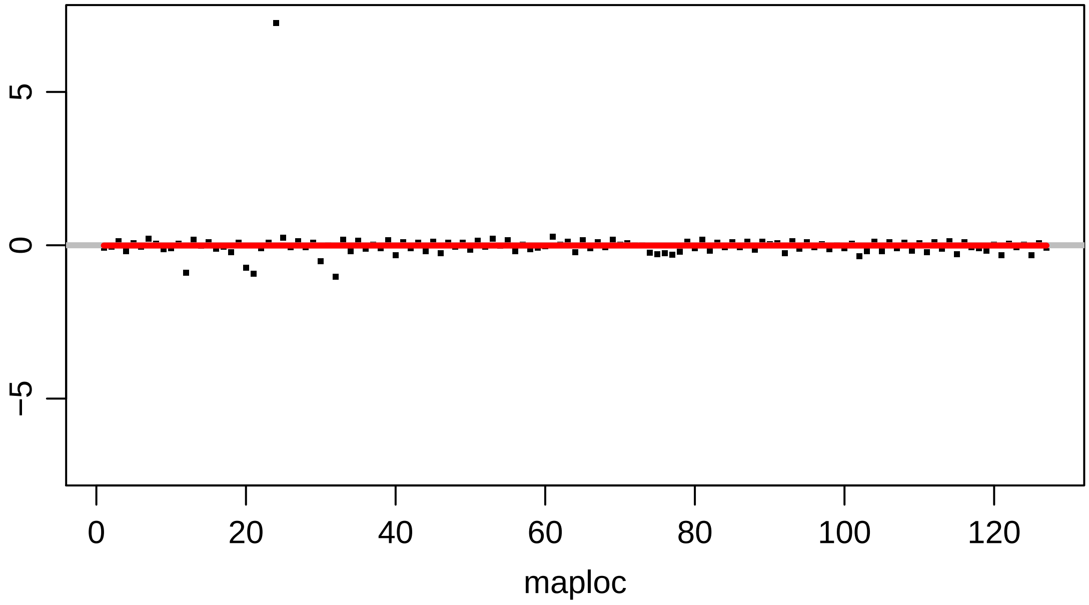


```
## Segplot might not work because of special characters in the sample names. Use only A-Z,a-z and 0-9!
##  There is a hidden function cn.mops:::.replaceNames that replaces the names in the "CNVDetectionResul
```

Case_IP073.G1.sam

Chromosome undef

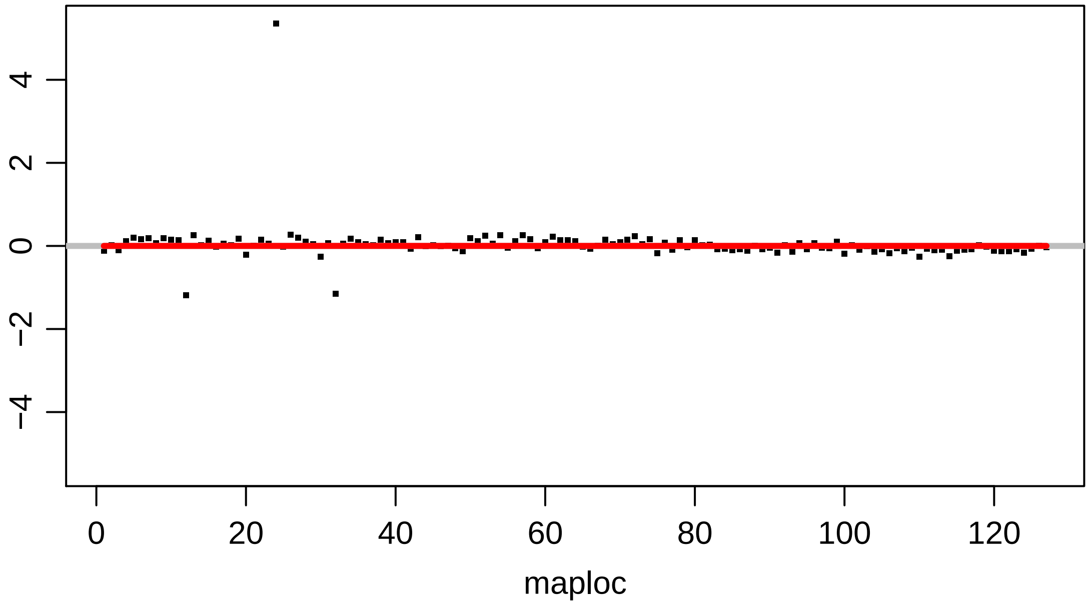

```
## Segplot might not work because of special characters in the sample names. Use only A-Z,a-z and 0-9!
##  There is a hidden function cn.mops:::.replaceNames that replaces the names in the "CNVDetectionResul
```

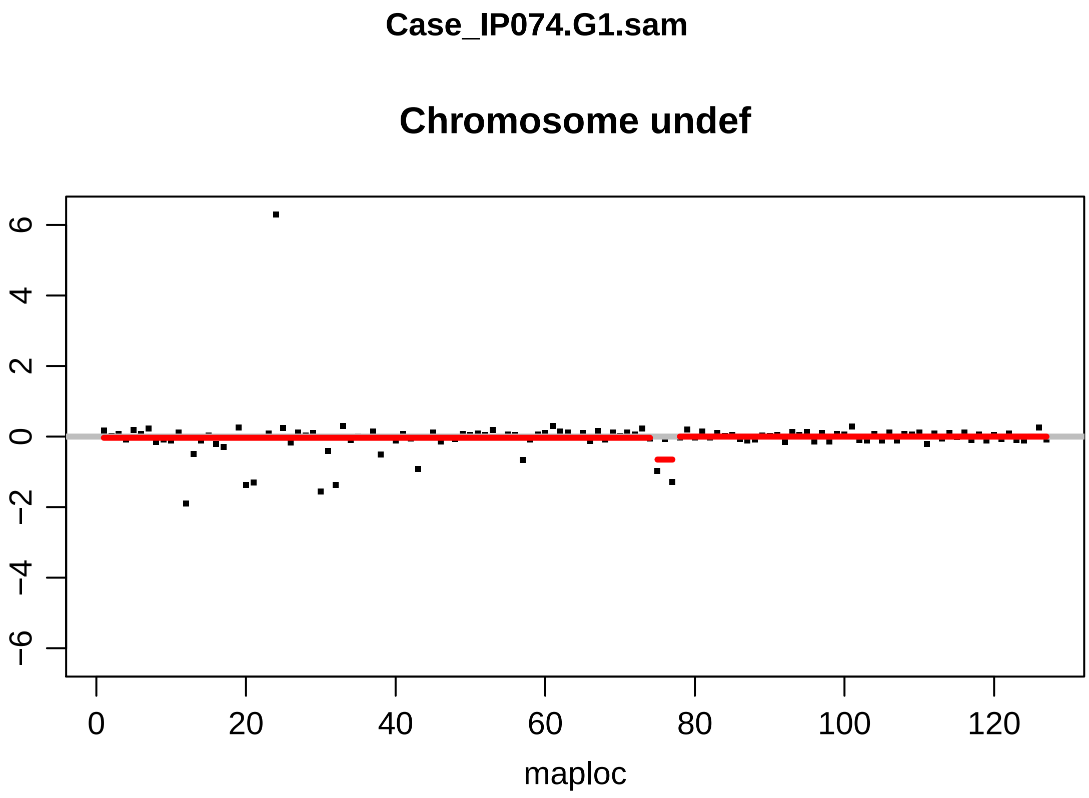


```
## Segplot might not work because of special characters in the sample names. Use only A-Z,a-z and 0-9!
##  There is a hidden function cn.mops:::.replaceNames that replaces the names in the "CNVDetectionResul
```

Case_IP075.G1.sam

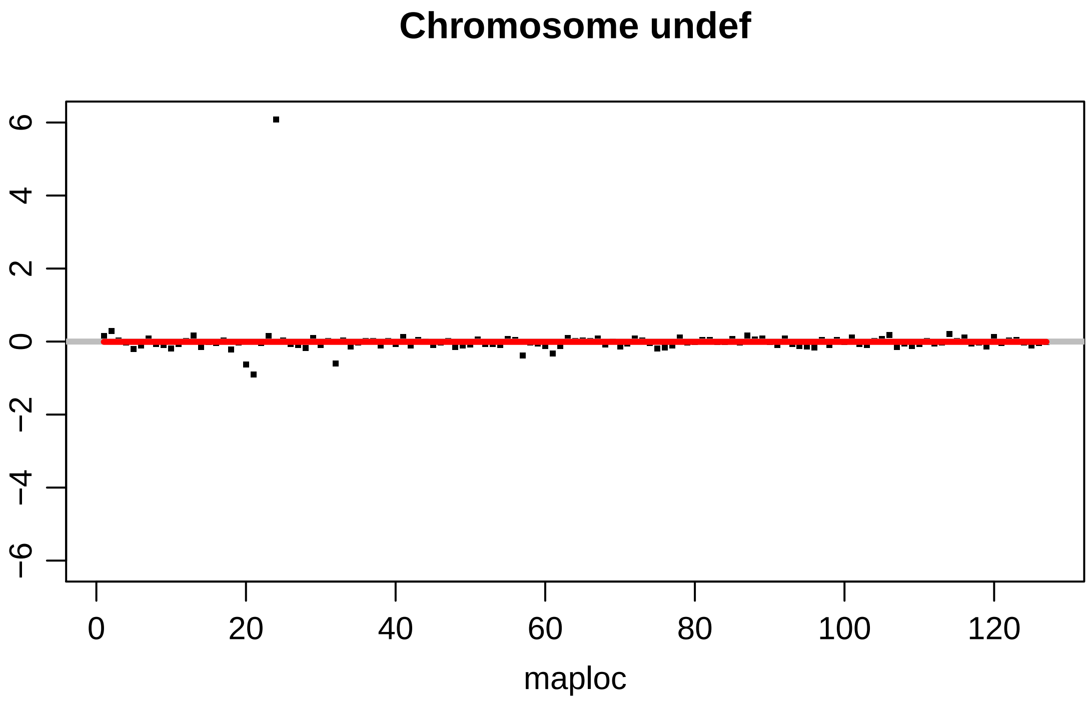


```
## Segplot might not work because of special characters in the sample names. Use only A-Z,a-z and 0-9!
##  There is a hidden function cn.mops:::.replaceNames that replaces the names in the "CNVDetectionResul
```

Case_IP076.G1.sam

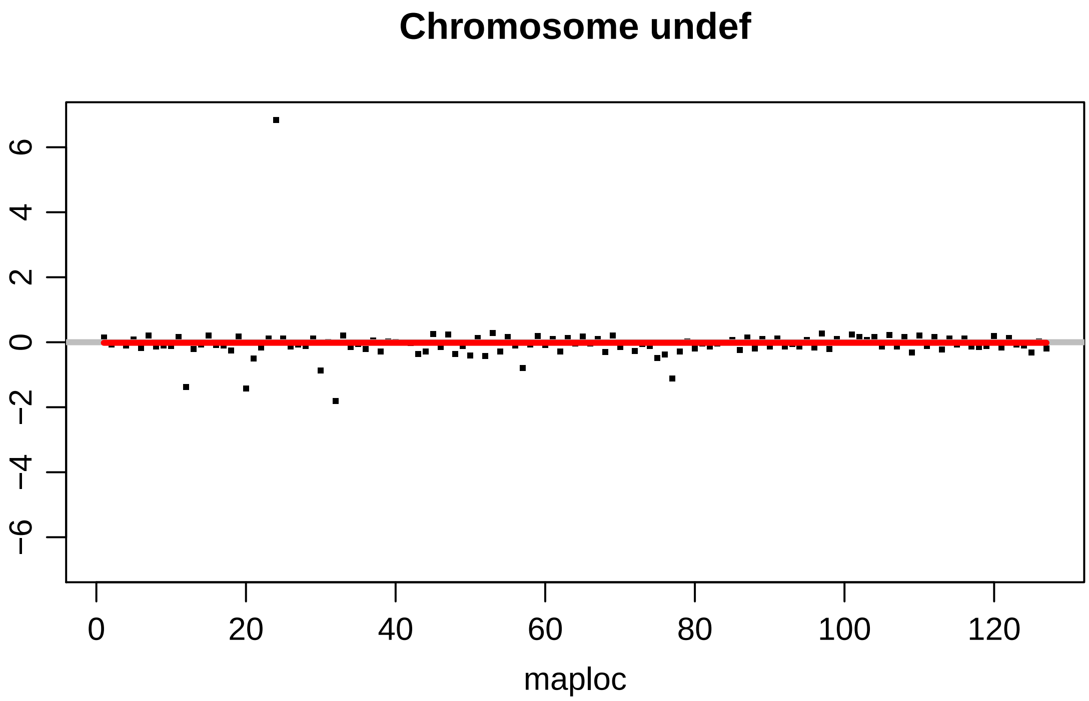

```
## Segplot might not work because of special characters in the sample names. Use only A-Z,a-z and 0-9!
##  There is a hidden function cn.mops:::.replaceNames that replaces the names in the "CNVDetectionResul
```

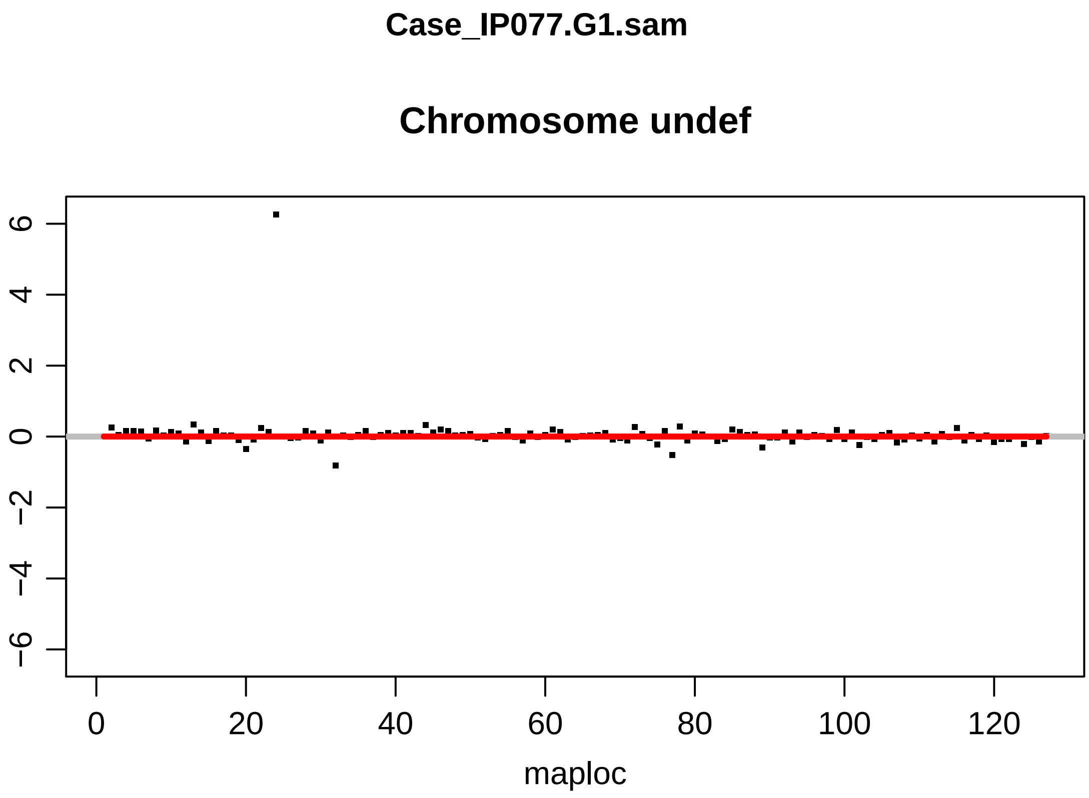


```
## Segplot might not work because of special characters in the sample names. Use only A-Z,a-z and 0-9!
##  There is a hidden function cn.mops:::.replaceNames that replaces the names in the "CNVDetectionResul
```

Case_IP078.G1.sam

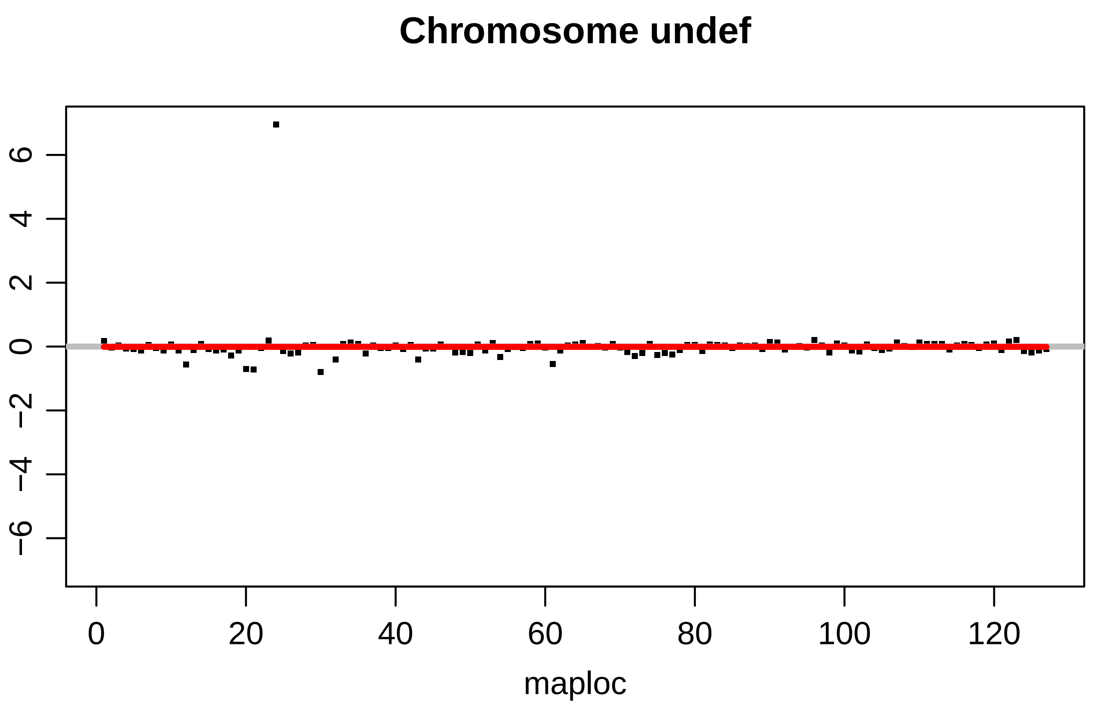


```
## Segplot might not work because of special characters in the sample names. Use only A-Z,a-z and 0-9!
##  There is a hidden function cn.mops:::.replaceNames that replaces the names in the "CNVDetectionResul
```

Case_IP079.G1.sam

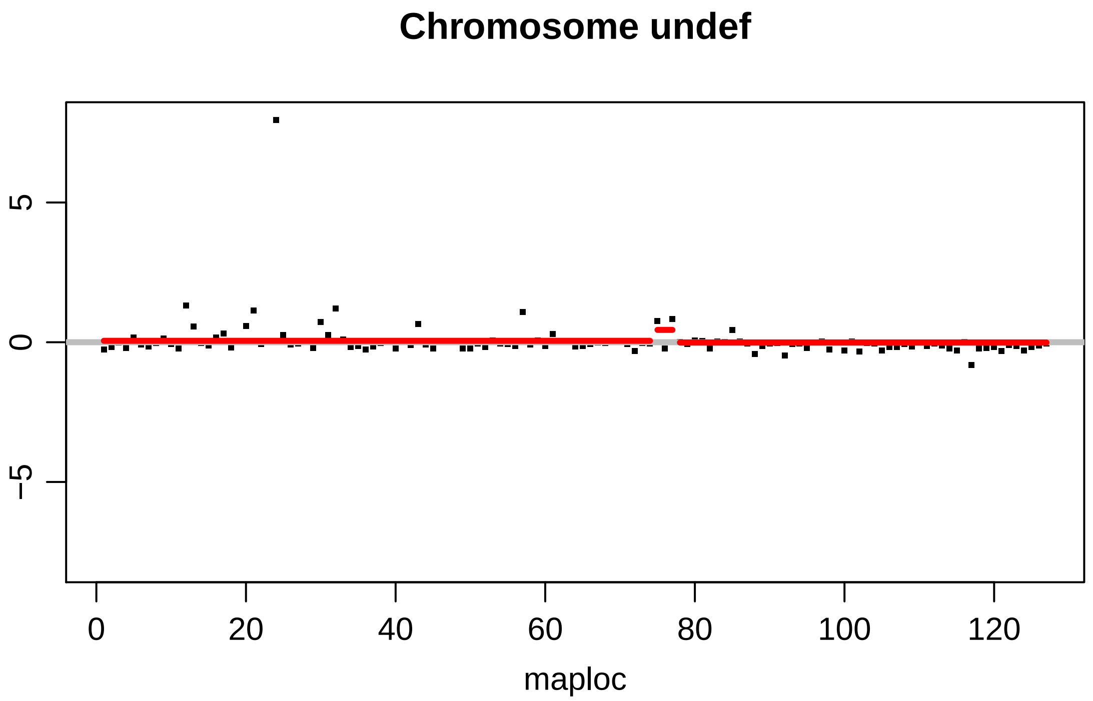

```
## Segplot might not work because of special characters in the sample names. Use only A-Z,a-z and 0-9!
##  There is a hidden function cn.mops:::.replaceNames that replaces the names in the "CNVDetectionResul
```

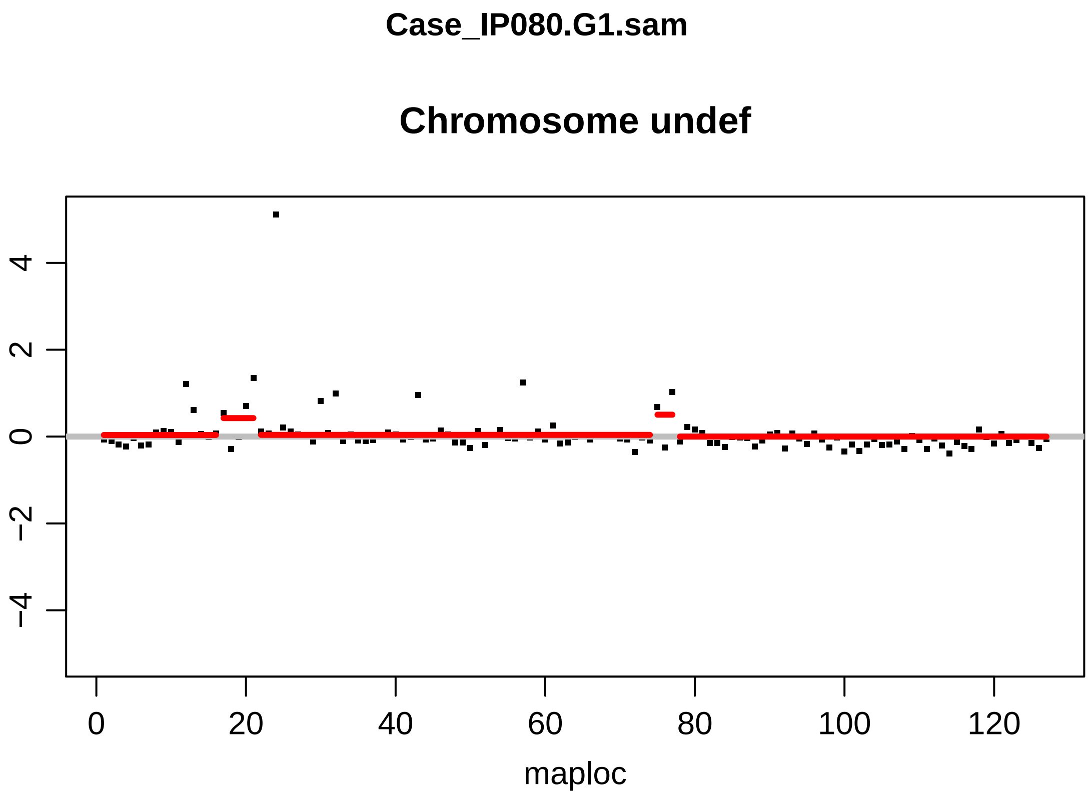


```
## Segplot might not work because of special characters in the sample names. Use only A-Z,a-z and 0-9!
##  There is a hidden function cn.mops:::.replaceNames that replaces the names in the "CNVDetectionResul
```

Case_IP083.G1.sam

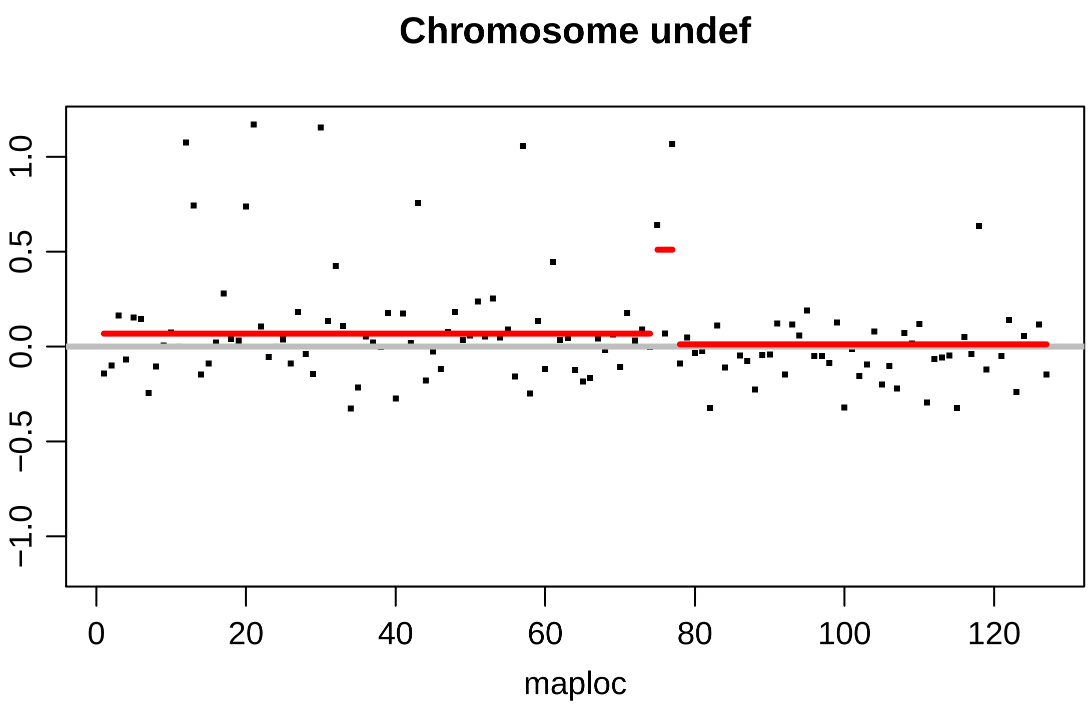


```
## Segplot might not work because of special characters in the sample names. Use only A-Z,a-z and 0-9!
##  There is a hidden function cn.mops:::.replaceNames that replaces the names in the "CNVDetectionResul
```

Case_IP084.G1.sam

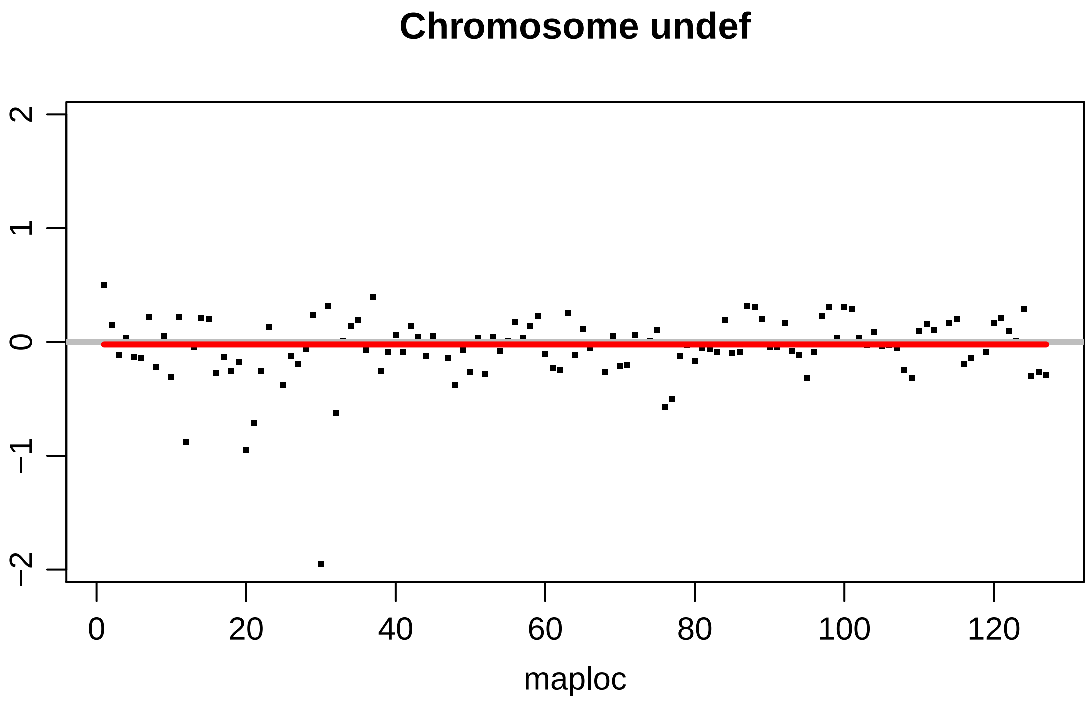

```
## Segplot might not work because of special characters in the sample names. Use only A-Z,a-z and 0-9!
##  There is a hidden function cn.mops:::.replaceNames that replaces the names in the "CNVDetectionResul
```

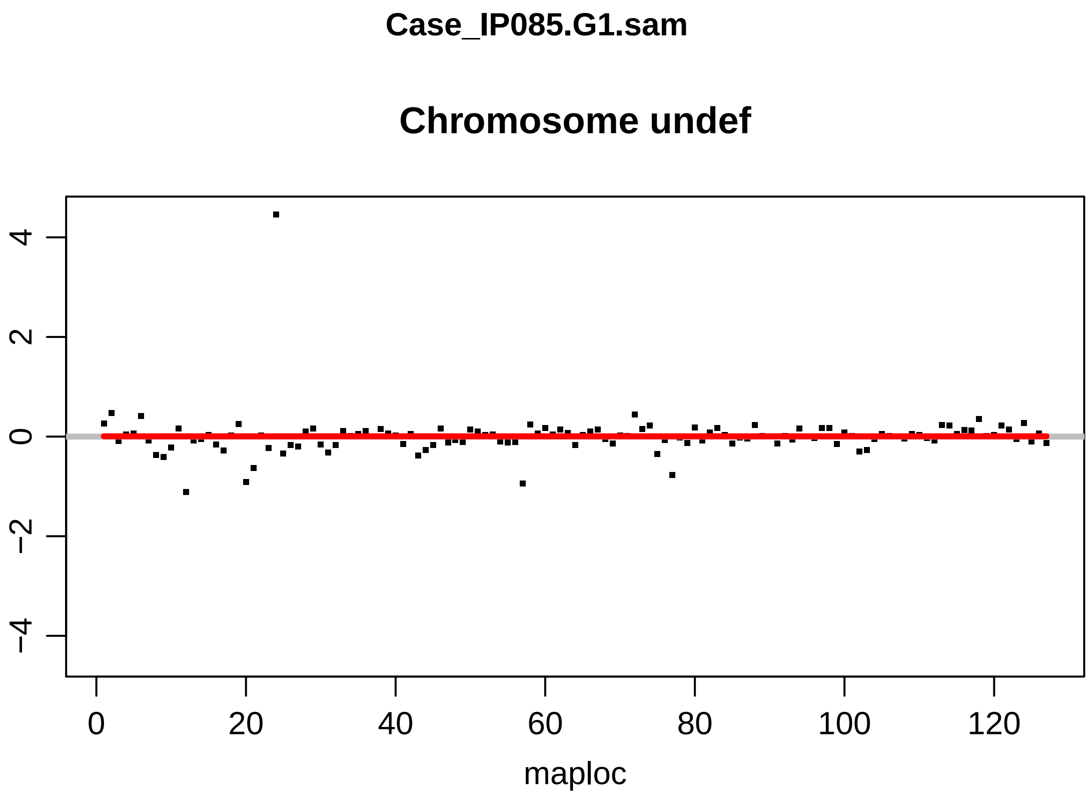


```
## Segplot might not work because of special characters in the sample names. Use only A-Z,a-z and 0-9!
##  There is a hidden function cn.mops:::.replaceNames that replaces the names in the "CNVDetectionResul
```

Case_IP086.G1.sam

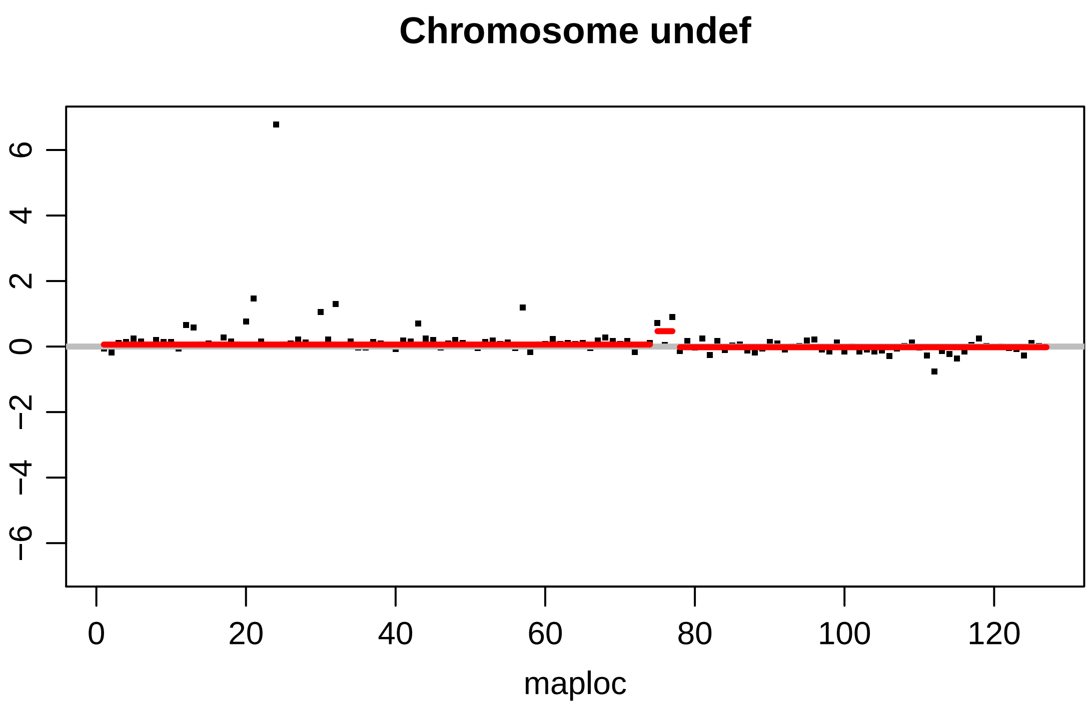


```
## Segplot might not work because of special characters in the sample names. Use only A-Z,a-z and 0-9!
##  There is a hidden function cn.mops:::.replaceNames that replaces the names in the "CNVDetectionResul
```

Case_IP087.G1.sam

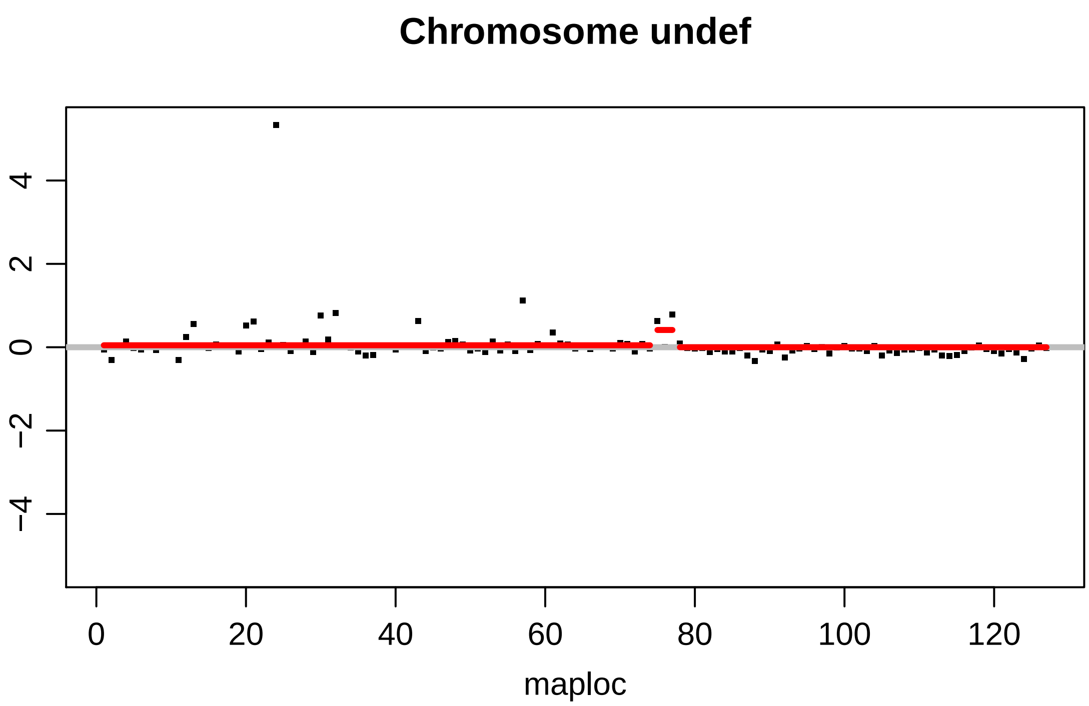

```
## Segplot might not work because of special characters in the sample names. Use only A-Z,a-z and 0-9!
##  There is a hidden function cn.mops:::.replaceNames that replaces the names in the "CNVDetectionResul
```

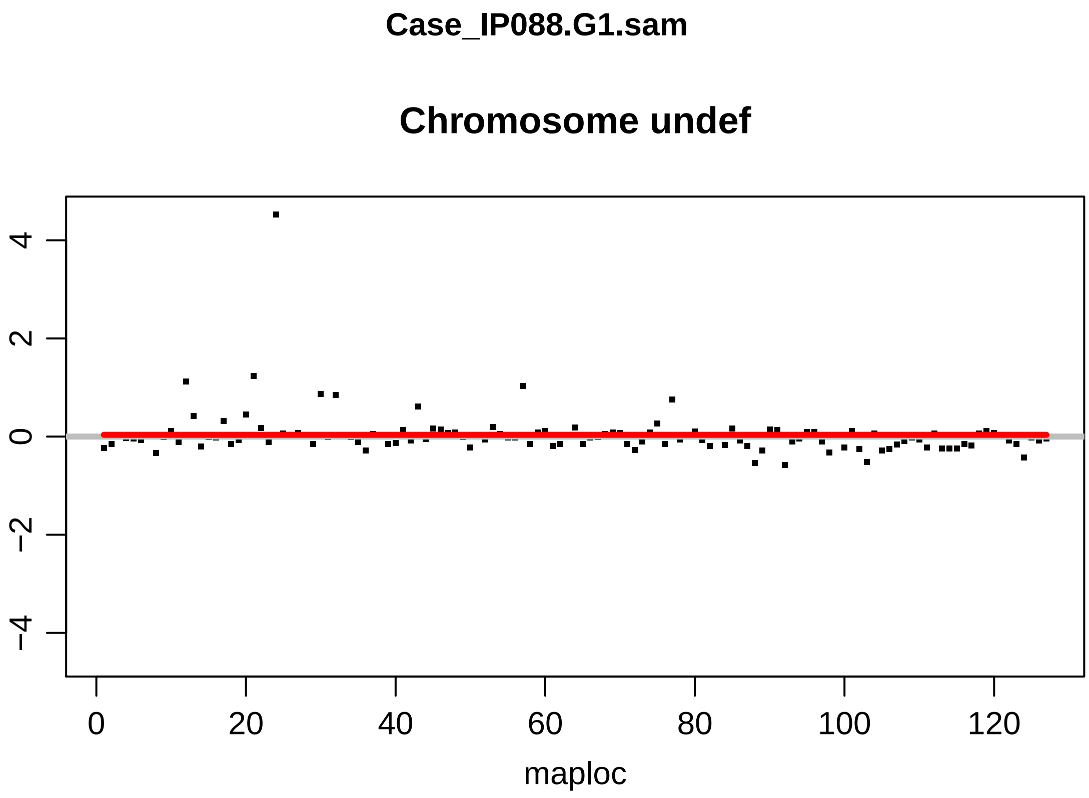


```
## Segplot might not work because of special characters in the sample names. Use only A-Z,a-z and 0-9!
##  There is a hidden function cn.mops:::.replaceNames that replaces the names in the "CNVDetectionResul
```

Case_IP089.G1.sam

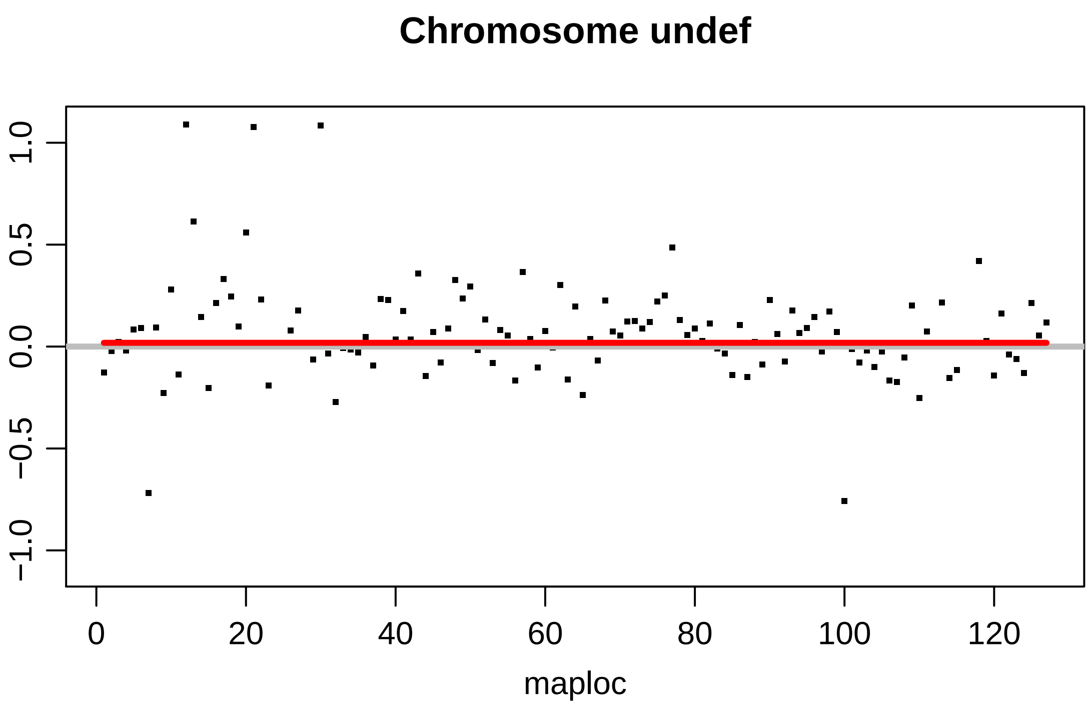


```
## Segplot might not work because of special characters in the sample names. Use only A-Z,a-z and 0-9!
##  There is a hidden function cn.mops:::.replaceNames that replaces the names in the "CNVDetectionResul
```

Case_IP092.G1.sam

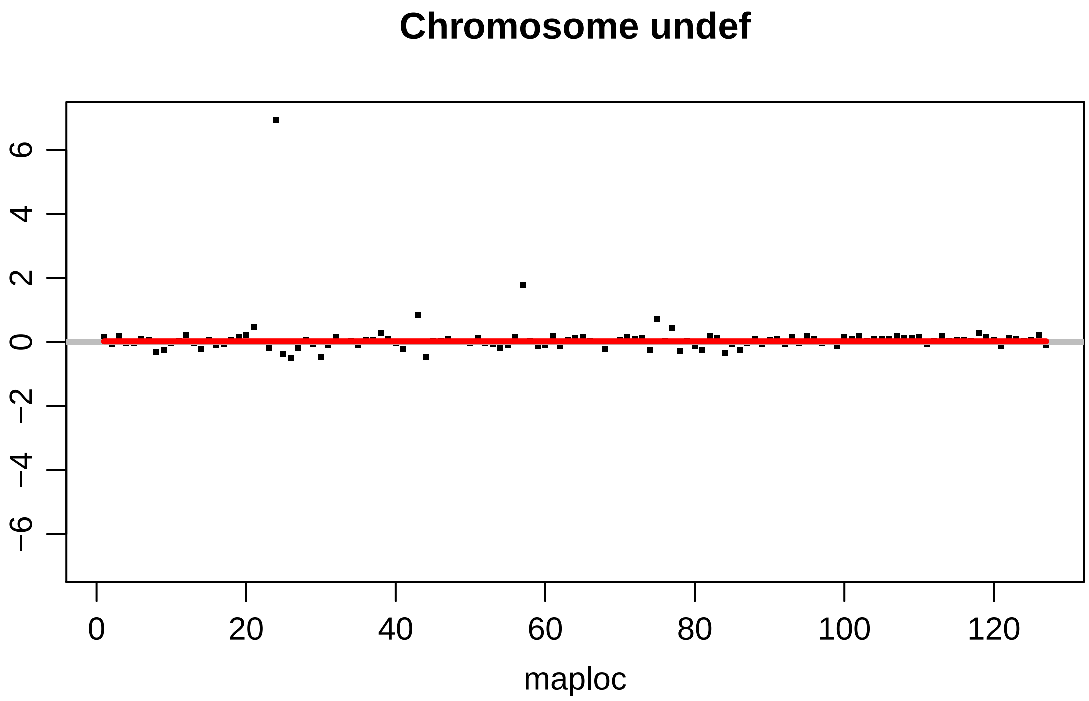

```
##
## CNV regions:
## GRanges object with 3 ranges and 39 metadata columns:
##       seqnames    ranges strand | Case_IP048.G1.sam Case_IP049.G1.sam
##          <Rle> <IRanges>  <Rle> |          <factor>          <factor>
##   [1]    undef  [ 6,  9]      * |               CN2               CN2
##   [2]    undef  [48, 50]      * |               CN2               CN2
##   [3]    undef  [75, 77]      * |               CN2               CN2
##       Case_IP050.G1.sam Case_IP051.G1.sam Case_IP052.G1.sam
##                <factor>          <factor>          <factor>
##   [1]               CN2               CN1               CN2
##   [2]               CN2               CN2               CN2
##   [3]               CN2               CN2               CN2
##       Case_IP053.G1.sam Case_IP054.G1.sam Case_IP055.G1.sam
##                <factor>          <factor>          <factor>
##   [1]               CN2               CN2               CN2
##   [2]               CN2               CN2               CN2
##   [3]               CN2               CN2               CN2
##       Case_IP056.G1.sam Case_IP057.G1.sam Case_IP058.G1.sam
##                <factor>          <factor>          <factor>
##   [1]               CN2               CN2               CN2
##   [2]               CN2               CN2               CN2
##   [3]               CN2               CN2               CN2
##       Case_IP059.G1.sam Case_IP060.G1.sam Case_IP061.G1.sam
##                <factor>          <factor>          <factor>
##   [1]               CN2               CN2               CN2
##   [2]               CN2               CN2               CN2
##   [3]               CN2               CN2               CN2
##       Case_IP062.G1.sam Case_IP063.G1.sam Case_IP065.G1.sam
##                <factor>          <factor>          <factor>
##   [1]               CN2               CN2               CN2
##   [2]               CN2               CN2               CN2
##   [3]               CN2               CN2               CN2
##       Case_IP067.G1.sam Case_IP068.G1.sam Case_IP069.G1.sam
##                <factor>          <factor>          <factor>
##   [1]               CN2               CN2               CN2
##   [2]               CN1               CN2               CN2
##   [3]               CN2               CN2               CN2
##       Case_IP071.G1.sam Case_IP072.G1.sam Case_IP073.G1.sam
##                <factor>          <factor>          <factor>
##   [1]               CN2               CN2               CN2
##   [2]               CN2               CN2               CN2
##   [3]               CN2               CN2               CN2
##       Case_IP074.G1.sam Case_IP075.G1.sam Case_IP076.G1.sam
##                <factor>          <factor>          <factor>
##   [1]               CN2               CN2               CN2
##   [2]               CN2               CN2               CN2
##   [3]               CN2               CN2               CN2
##       Case_IP077.G1.sam Case_IP078.G1.sam Case_IP079.G1.sam
##                <factor>          <factor>          <factor>
##   [1]               CN2               CN2               CN2
##   [2]               CN2               CN2               CN2
##   [3]               CN2               CN2               CN2
##       Case_IP080.G1.sam Case_IP083.G1.sam Case_IP084.G1.sam
```

```
##                <factor>          <factor>          <factor>
##   [1]               CN2               CN2               CN2
##   [2]               CN2               CN2               CN2
##   [3]               CN3               CN3               CN2
##       Case_IP085.G1.sam Case_IP086.G1.sam Case_IP087.G1.sam
##                <factor>          <factor>          <factor>
##   [1]               CN2               CN2               CN2
##   [2]               CN2               CN2               CN2
##   [3]               CN2               CN2               CN2
##       Case_IP088.G1.sam Case_IP089.G1.sam Case_IP092.G1.sam
##                <factor>          <factor>          <factor>
##   [1]               CN2               CN2               CN2
##   [2]               CN2               CN2               CN2
##   [3]               CN2               CN2               CN2
##       Case_IP094.G1.sam
##                <factor>
##   [1]               CN2
##   [2]               CN2
##   [3]               CN2
##   -------
##   seqinfo: 1 sequence from an unspecified genome; no seqlengths
##
## Individual CNVs:
## GRanges object with 4 ranges and 4 metadata columns:
##       seqnames    ranges strand |        sampleName     median       mean
##          <Rle> <IRanges>  <Rle> |          <factor>  <numeric>  <numeric>
##   [1]    undef  [ 6,  9]      * | Case_IP051.G1.sam -0.9999993 -0.9999003
##   [2]    undef  [48, 50]      * | Case_IP067.G1.sam -0.9999952 -0.9999648
##   [3]    undef  [75, 77]      * | Case_IP080.G1.sam  0.5840101  0.5041360
##   [4]    undef  [75, 77]      * | Case_IP083.G1.sam  0.5825900  0.5106382
##                CN
##       <character>
##   [1]         CN1
##   [2]         CN1
##   [3]         CN3
##   [4]         CN3
##   -------
##   seqinfo: 1 sequence from an unspecified genome; no seqlengths
## [1] "/Users/gdemidov/Downloads/doc/Run_SN1_27_fin_05_qc.xls"

## Normalizing...

## Starting local modeling, please be patient...

## Reference sequence:  undef

## Starting segmentation algorithm...

## Using "fastseg" for segmentation.

## [1] ""
## [1] "/Users/gdemidov/Downloads/doc/Run_SN1_27_fin_05_qc.xls"
## [1] ""
```

```
## Segplot might not work because of special characters in the sample names. Use only A-Z,a-z and 0-9!
##  There is a hidden function cn.mops:::.replaceNames that replaces the names in the "CNVDetectionResul
```

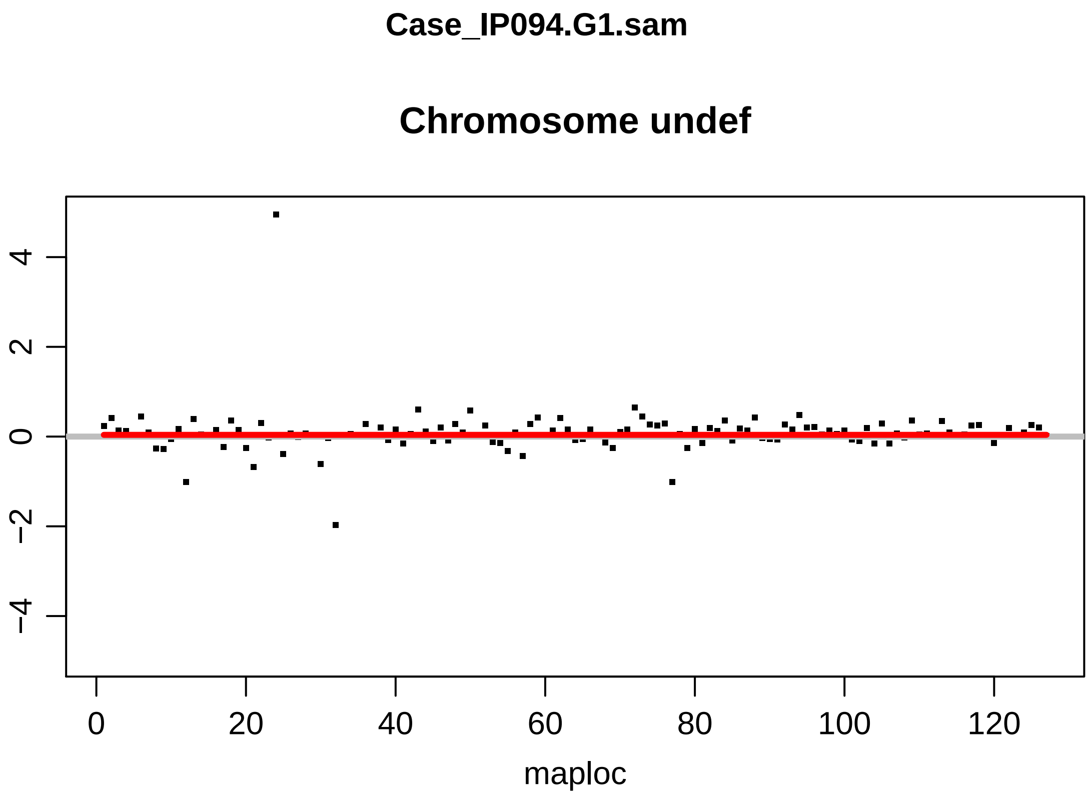


```
## Segplot might not work because of special characters in the sample names. Use only A-Z,a-z and 0-9!
##  There is a hidden function cn.mops:::.replaceNames that replaces the names in the "CNVDetectionResul
```

s_002_R_2012_09_13_10_57_38_Sequoia_SN1.27.Run_21_Auto_Sequoia_SN1.27.I

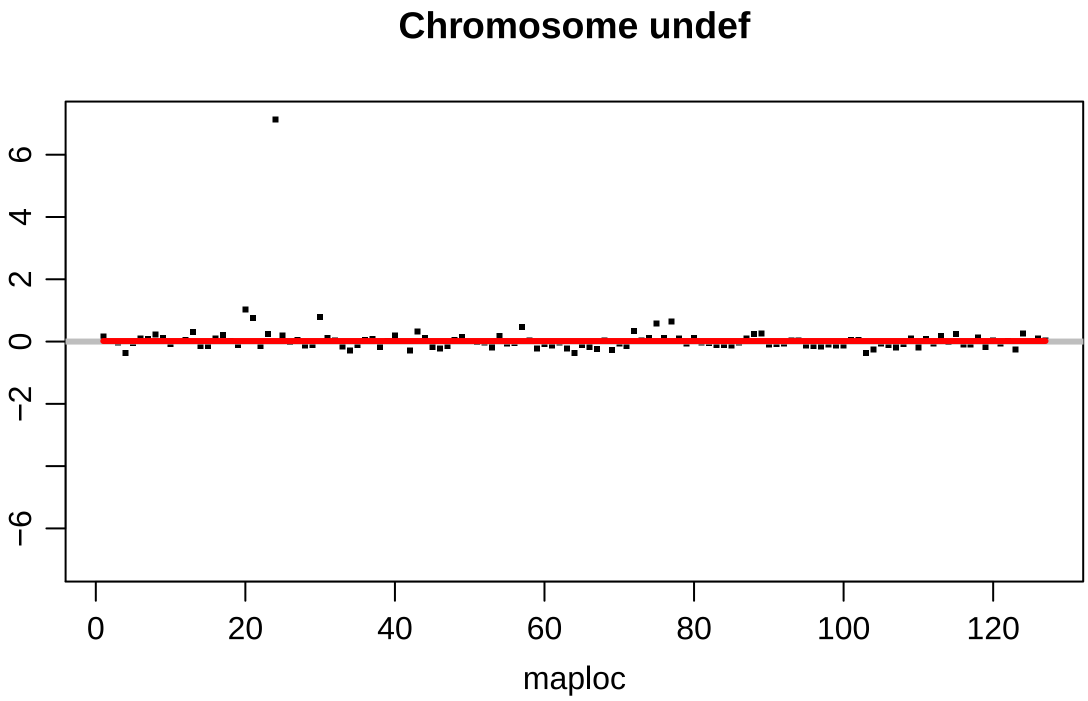


```
## Segplot might not work because of special characters in the sample names. Use only A-Z,a-z and 0-9!
##  There is a hidden function cn.mops:::.replaceNames that replaces the names in the "CNVDetectionResul
```

s_004_R_2012_09_13_10_57_38_Sequoia_SN1.27.Run_21_Auto_Sequoia_SN1.27.I

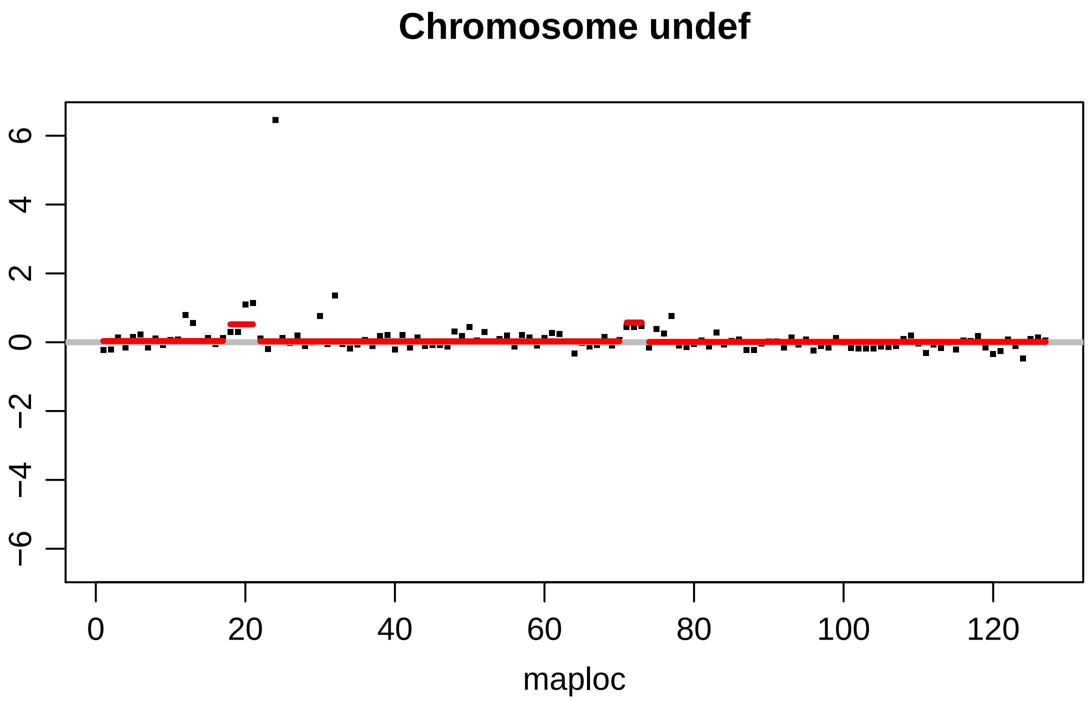

```
## Segplot might not work because of special characters in the sample names. Use only A-Z,a-z and 0-9!
##  There is a hidden function cn.mops:::.replaceNames that replaces the names in the "CNVDetectionResul
```

s_005_R_2012_09_13_10_57_38_Sequoia_SN1.27.Run_21_Auto_Sequoia_SN1.27.I

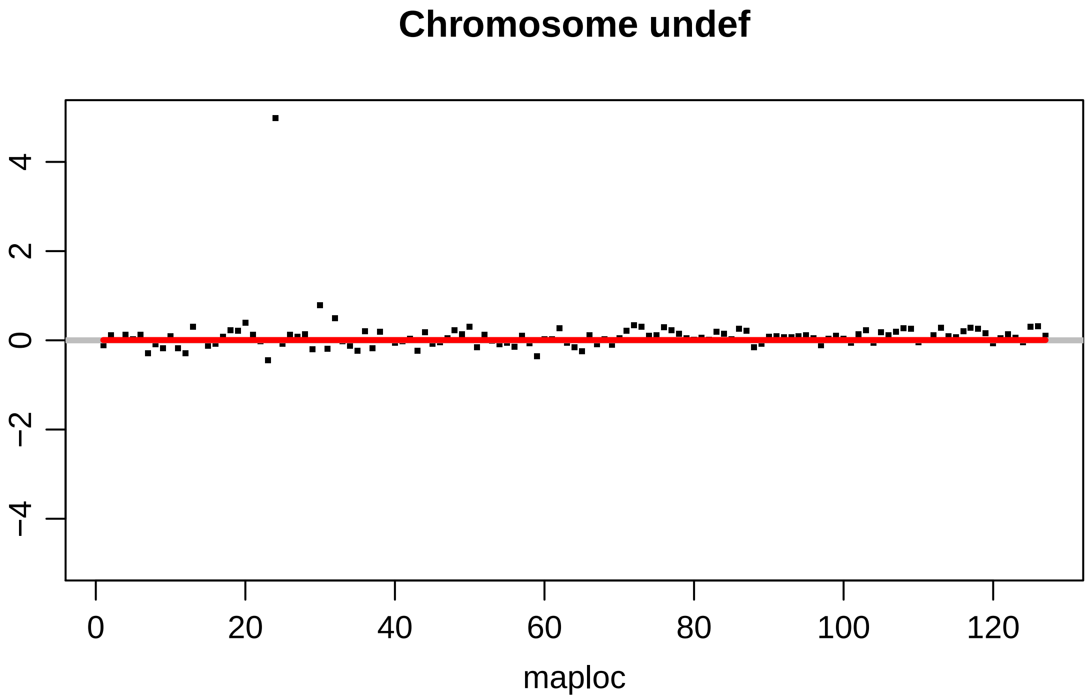


```
## Segplot might not work because of special characters in the sample names. Use only A-Z,a-z and 0-9!
##  There is a hidden function cn.mops:::.replaceNames that replaces the names in the "CNVDetectionResul
```

s_006_R_2012_09_13_10_57_38_Sequoia_SN1.27.Run_21_Auto_Sequoia_SN1.27.I

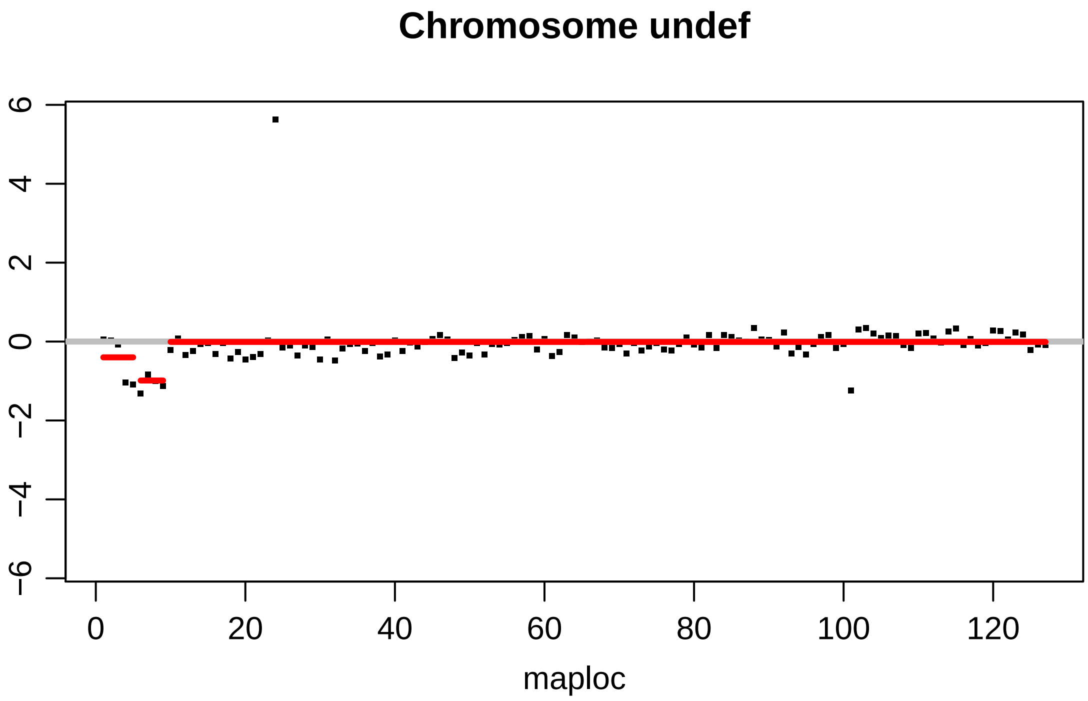


```
## Segplot might not work because of special characters in the sample names. Use only A-Z,a-z and 0-9!
##  There is a hidden function cn.mops:::.replaceNames that replaces the names in the "CNVDetectionResul
```

s_007_R_2012_09_13_10_57_38_Sequoia_SN1.27.Run_21_Auto_Sequoia_SN1.27.I

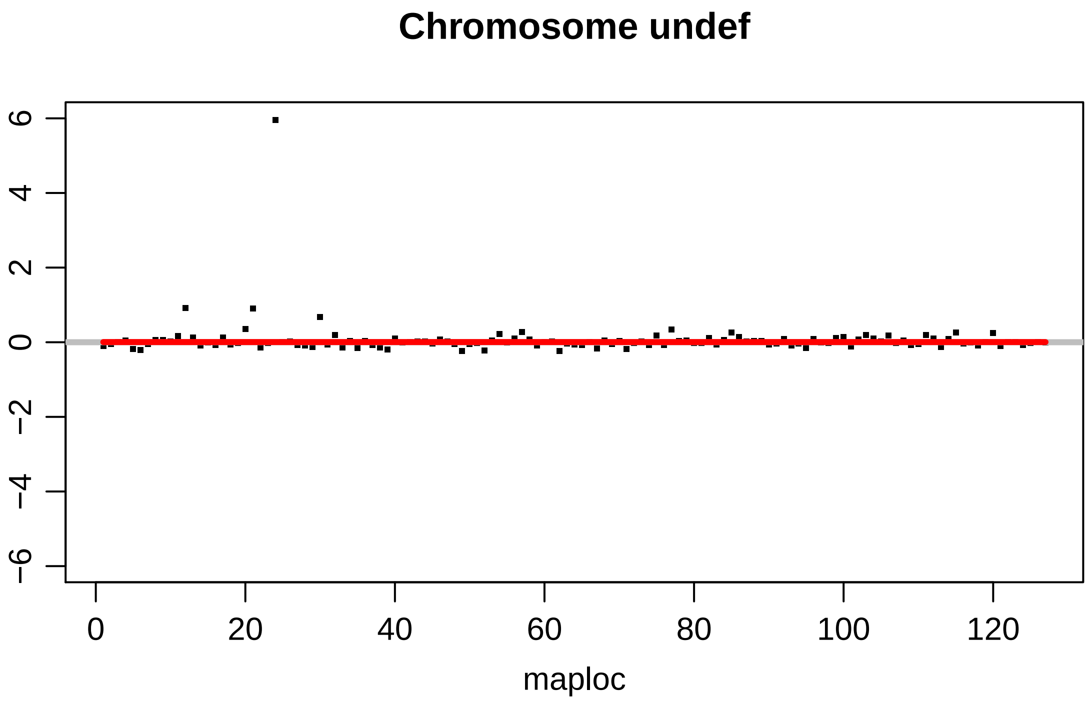

```
## Segplot might not work because of special characters in the sample names. Use only A-Z,a-z and 0-9!
##  There is a hidden function cn.mops:::.replaceNames that replaces the names in the "CNVDetectionResul
```

s_009_R_2012_09_13_10_57_38_Sequoia_SN1.27.Run_21_Auto_Sequoia_SN1.27.I

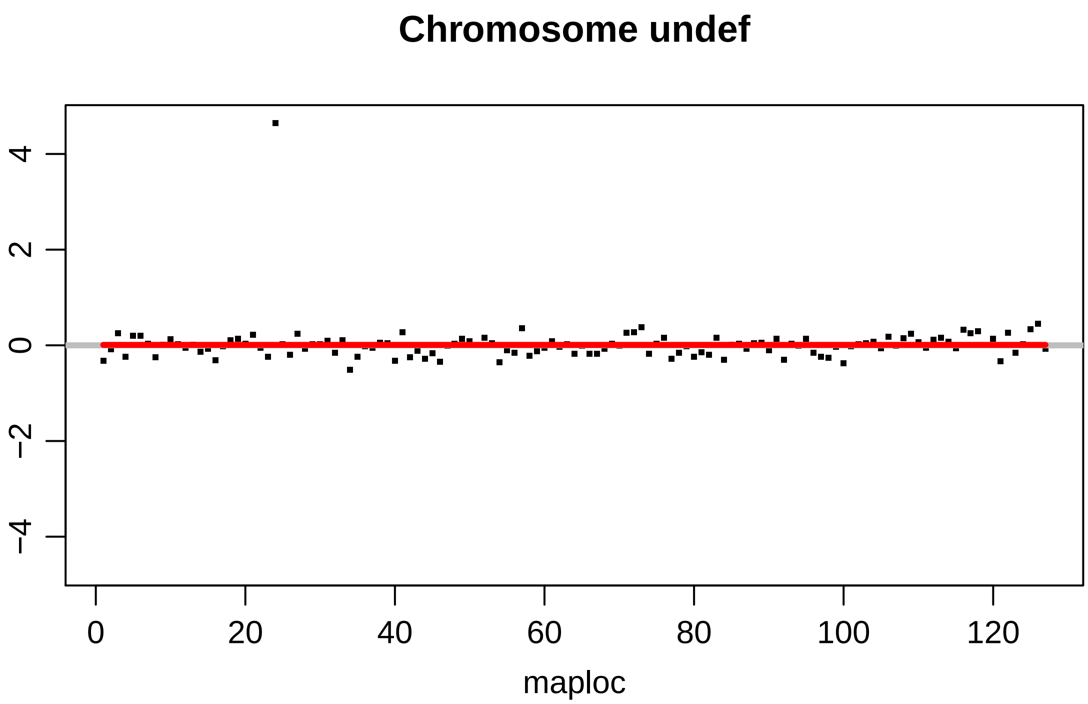


```
## Segplot might not work because of special characters in the sample names. Use only A-Z,a-z and 0-9!
##  There is a hidden function cn.mops:::.replaceNames that replaces the names in the "CNVDetectionResul
```

s_010_R_2012_09_13_10_57_38_Sequoia_SN1.27.Run_21_Auto_Sequoia_SN1.27.I

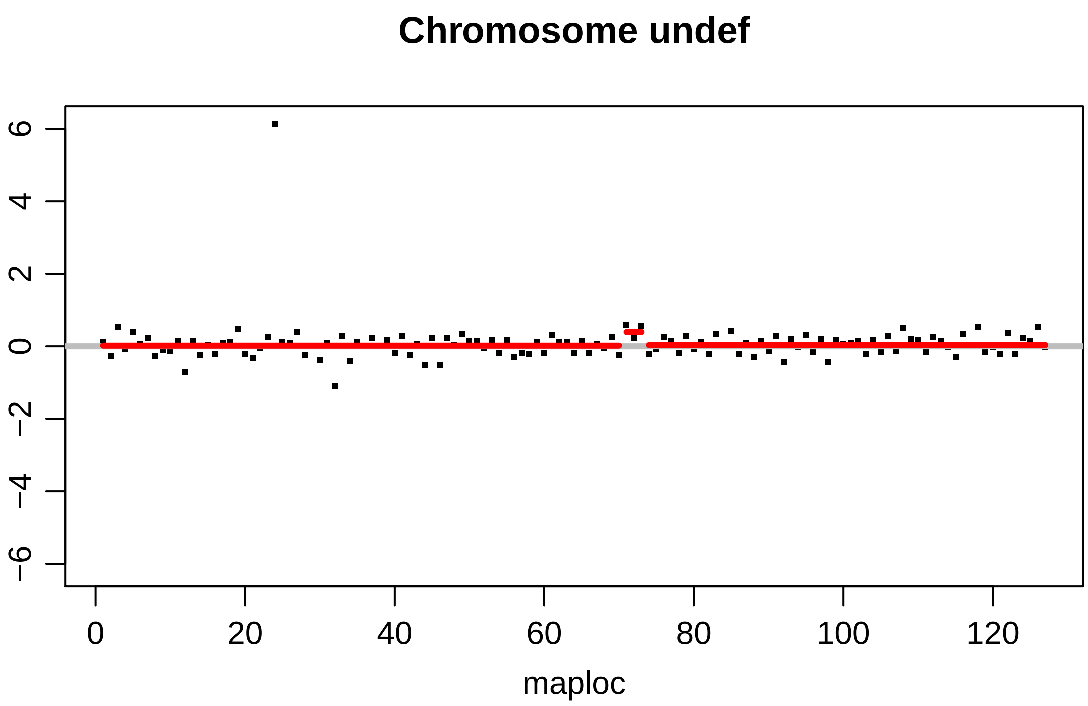


```
## Segplot might not work because of special characters in the sample names. Use only A-Z,a-z and 0-9!
##  There is a hidden function cn.mops:::.replaceNames that replaces the names in the "CNVDetectionResul
```

s_011_R_2012_09_13_10_57_38_Sequoia_SN1.27.Run_21_Auto_Sequoia_SN1.27.I

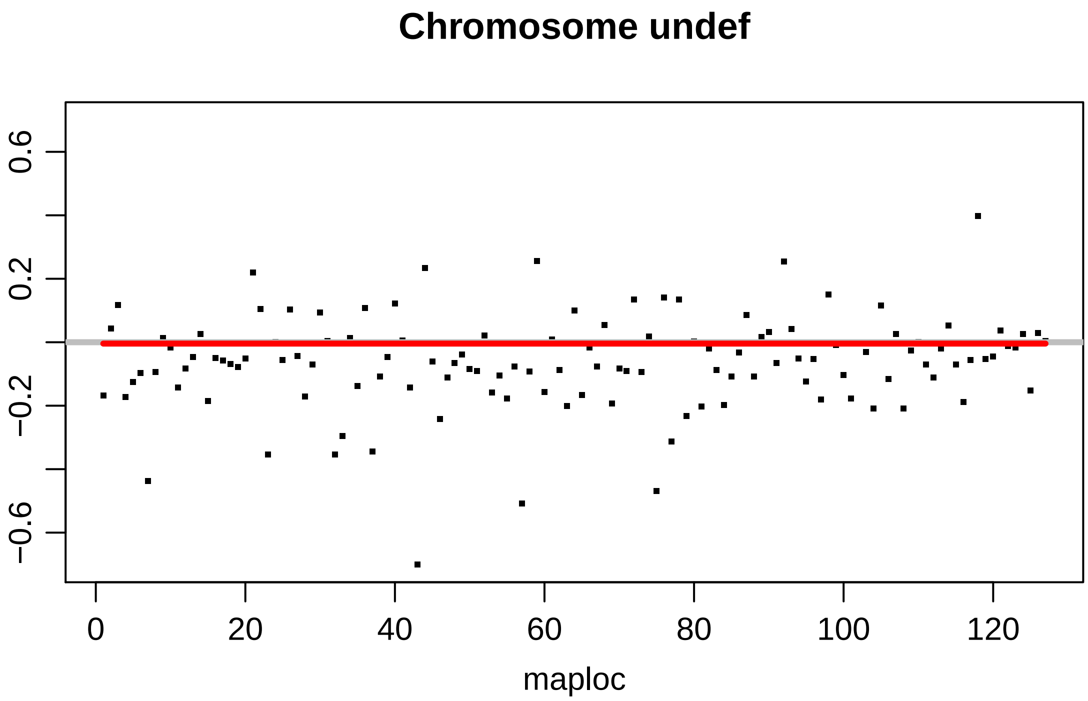

```
## Segplot might not work because of special characters in the sample names. Use only A-Z,a-z and 0-9!
##  There is a hidden function cn.mops:::.replaceNames that replaces the names in the "CNVDetectionResul
```

s_012_R_2012_09_13_10_57_38_Sequoia_SN1.27.Run_21_Auto_Sequoia_SN1.27.R

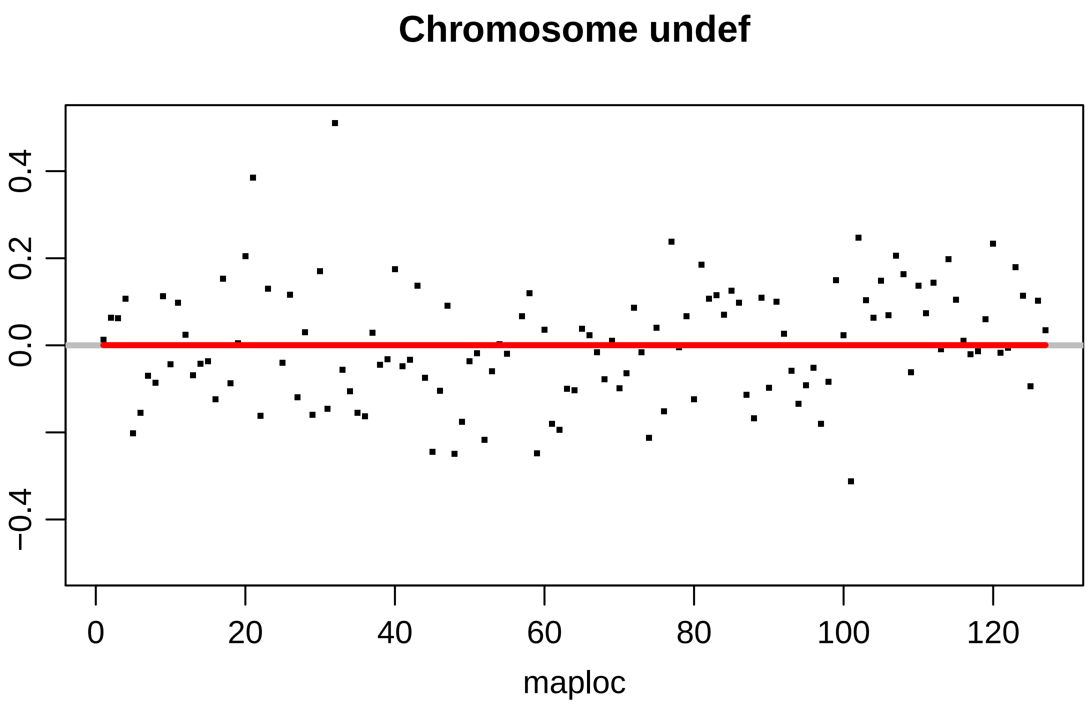


```
## Segplot might not work because of special characters in the sample names. Use only A-Z,a-z and 0-9!
##  There is a hidden function cn.mops:::.replaceNames that replaces the names in the "CNVDetectionResul
```

s_013_R_2012_09_13_10_57_38_Sequoia_SN1.27.Run_21_Auto_Sequoia_SN1.27.I

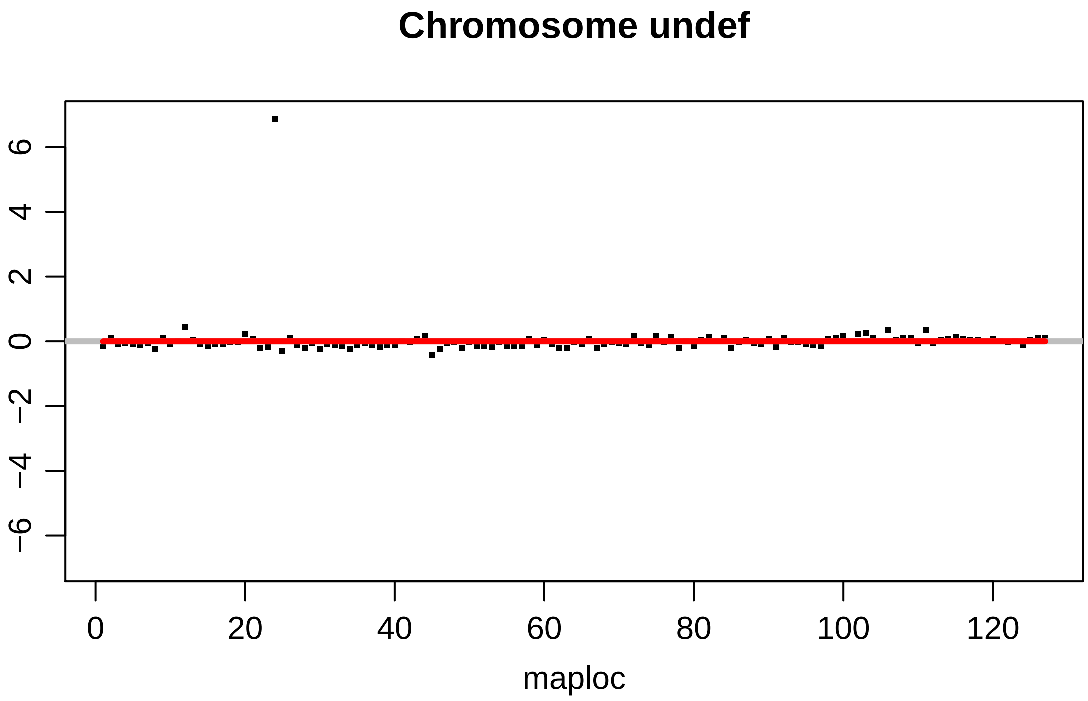


```
## Segplot might not work because of special characters in the sample names. Use only A-Z,a-z and 0-9!
##  There is a hidden function cn.mops:::.replaceNames that replaces the names in the "CNVDetectionResul
```

s_014_R_2012_09_13_10_57_38_Sequoia_SN1.27.Run_21_Auto_Sequoia_SN1.27.I

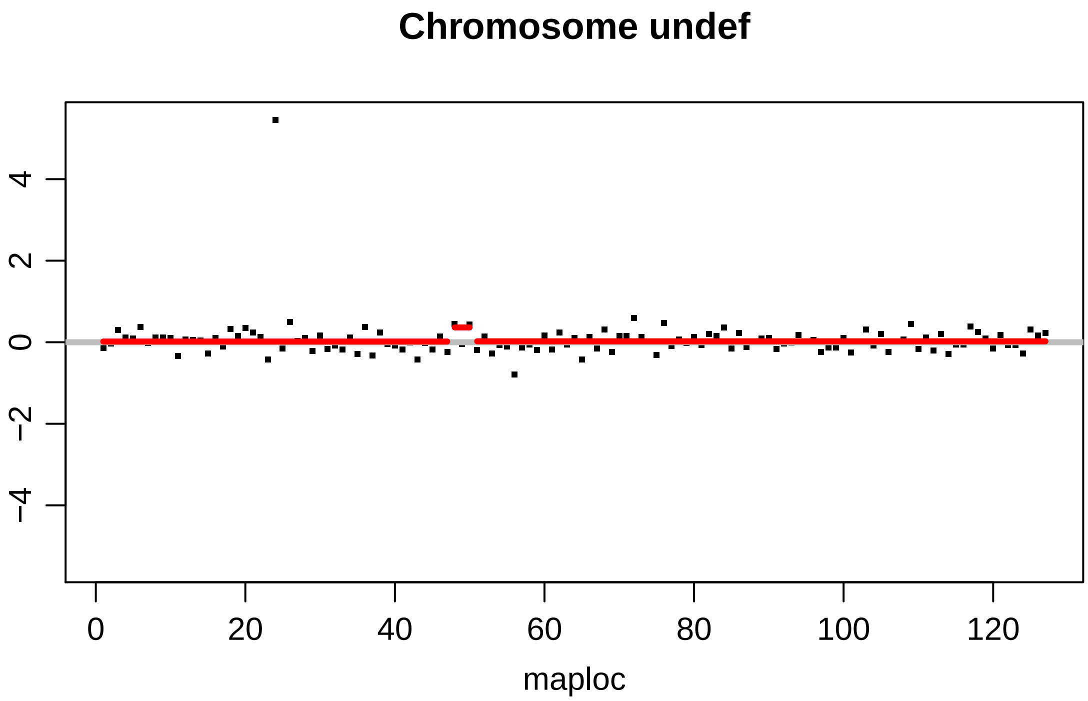

```
## Segplot might not work because of special characters in the sample names. Use only A-Z,a-z and 0-9!
##  There is a hidden function cn.mops:::.replaceNames that replaces the names in the "CNVDetectionResul
```

s_015_R_2012_09_13_10_57_38_Sequoia_SN1.27.Run_21_Auto_Sequoia_SN1.27.I

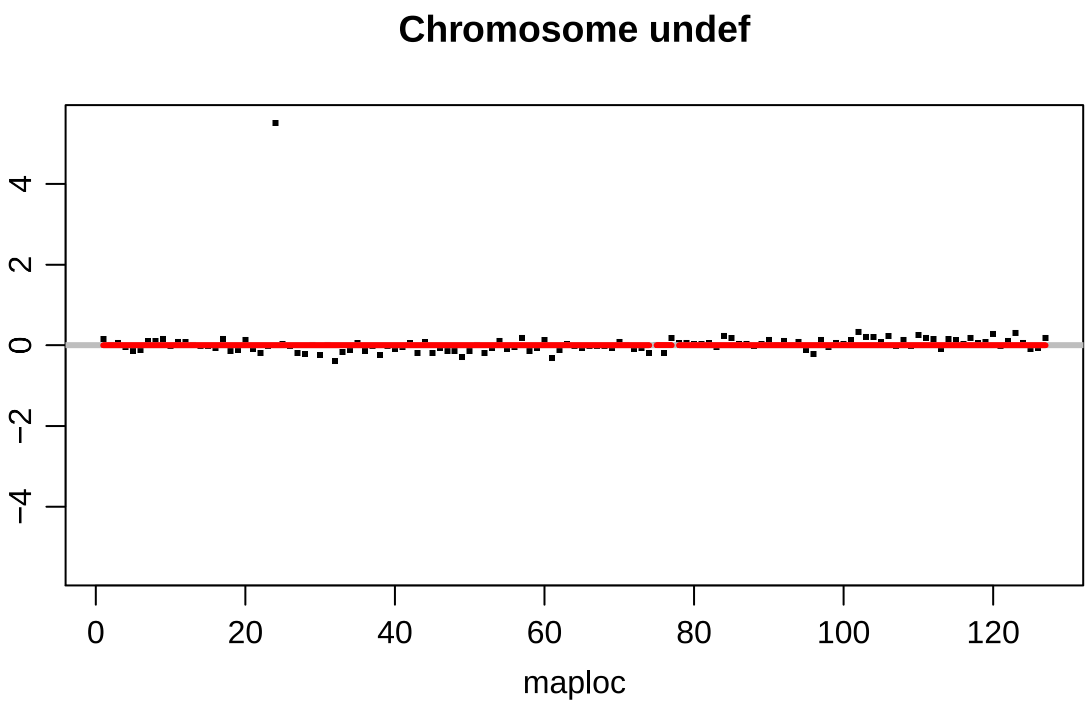


```
## Segplot might not work because of special characters in the sample names. Use only A-Z,a-z and 0-9!
##  There is a hidden function cn.mops:::.replaceNames that replaces the names in the "CNVDetectionResul
```

s_016_R_2012_09_13_10_57_38_Sequoia_SN1.27.Run_21_Auto_Sequoia_SN1.27.I

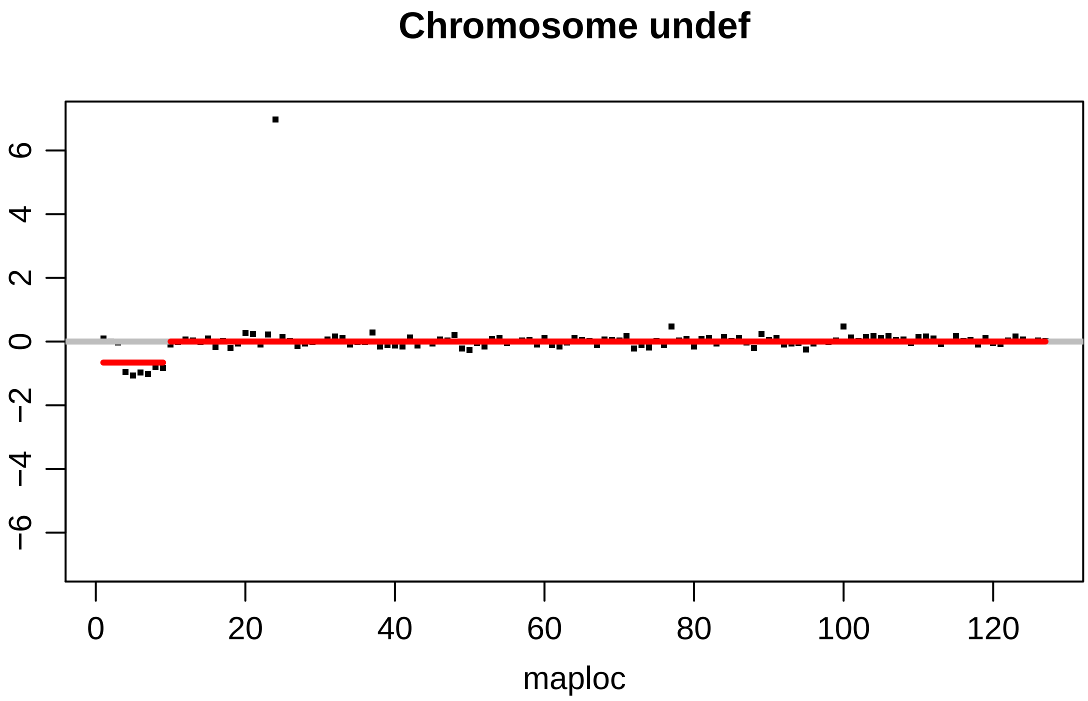


```
## Segplot might not work because of special characters in the sample names. Use only A-Z,a-z and 0-9!
##  There is a hidden function cn.mops:::.replaceNames that replaces the names in the "CNVDetectionResul
```

s_017_R_2012_09_13_10_57_38_Sequoia_SN1.27.Run_21_Auto_Sequoia_SN1.27.I

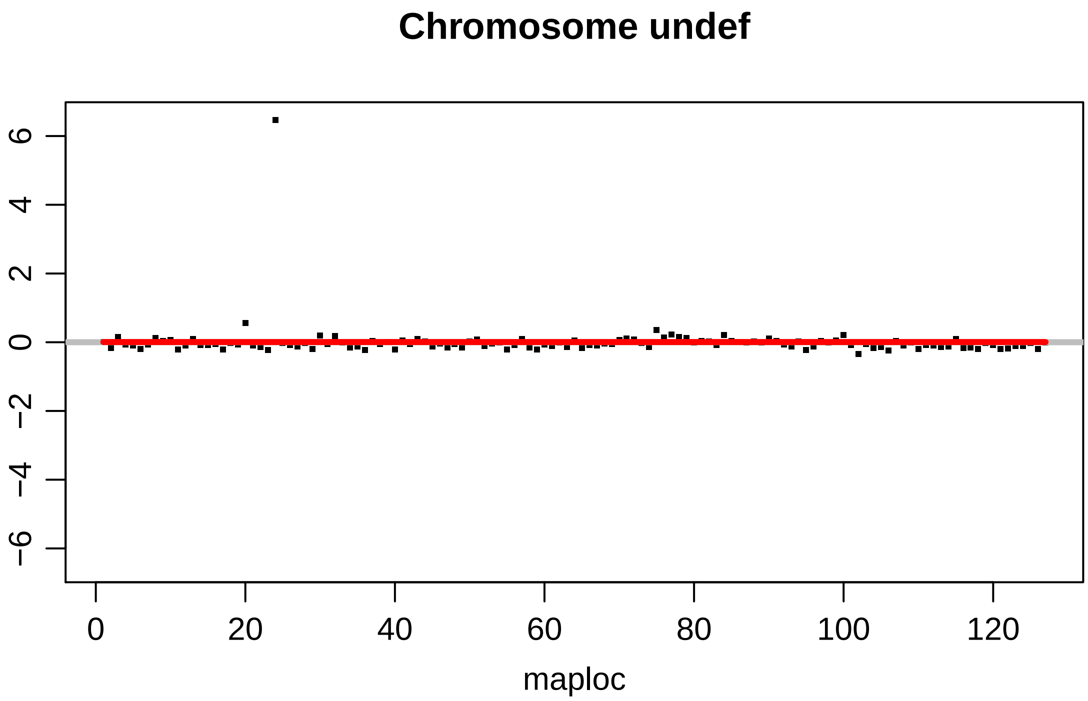

```
## Segplot might not work because of special characters in the sample names. Use only A-Z,a-z and 0-9!
##  There is a hidden function cn.mops:::.replaceNames that replaces the names in the "CNVDetectionResul
```

s_018_R_2012_09_13_10_57_38_Sequoia_SN1.27.Run_21_Auto_Sequoia_SN1.27.

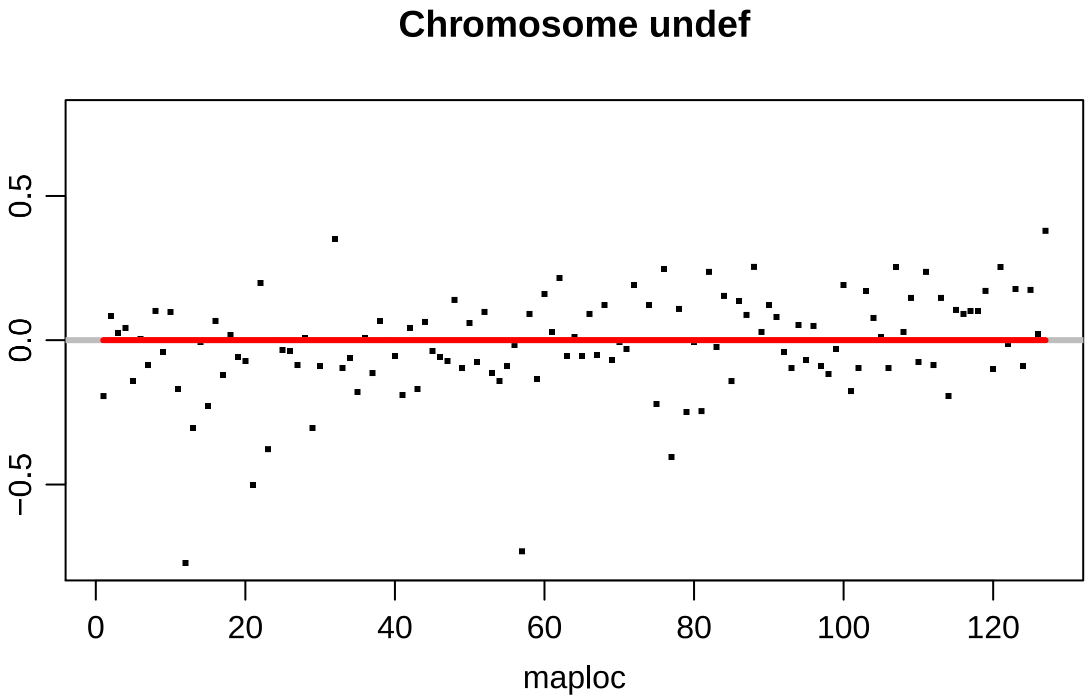


```
## Segplot might not work because of special characters in the sample names. Use only A-Z,a-z and 0-9!
##  There is a hidden function cn.mops:::.replaceNames that replaces the names in the "CNVDetectionResul
```

s_019_R_2012_09_13_10_57_38_Sequoia_SN1.27.Run_21_Auto_Sequoia_SN1.27.I

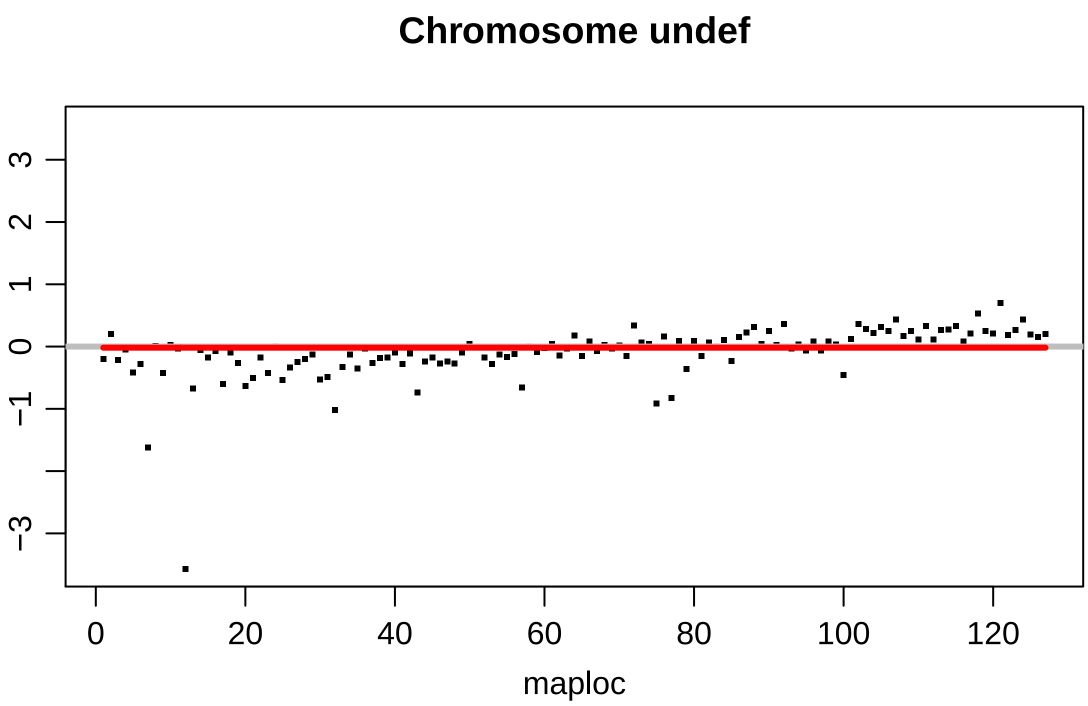


```
## Segplot might not work because of special characters in the sample names. Use only A-Z,a-z and 0-9!
##  There is a hidden function cn.mops:::.replaceNames that replaces the names in the "CNVDetectionResul
```

s_020_R_2012_09_13_10_57_38_Sequoia_SN1.27.Run_21_Auto_Sequoia_SN1.27.I

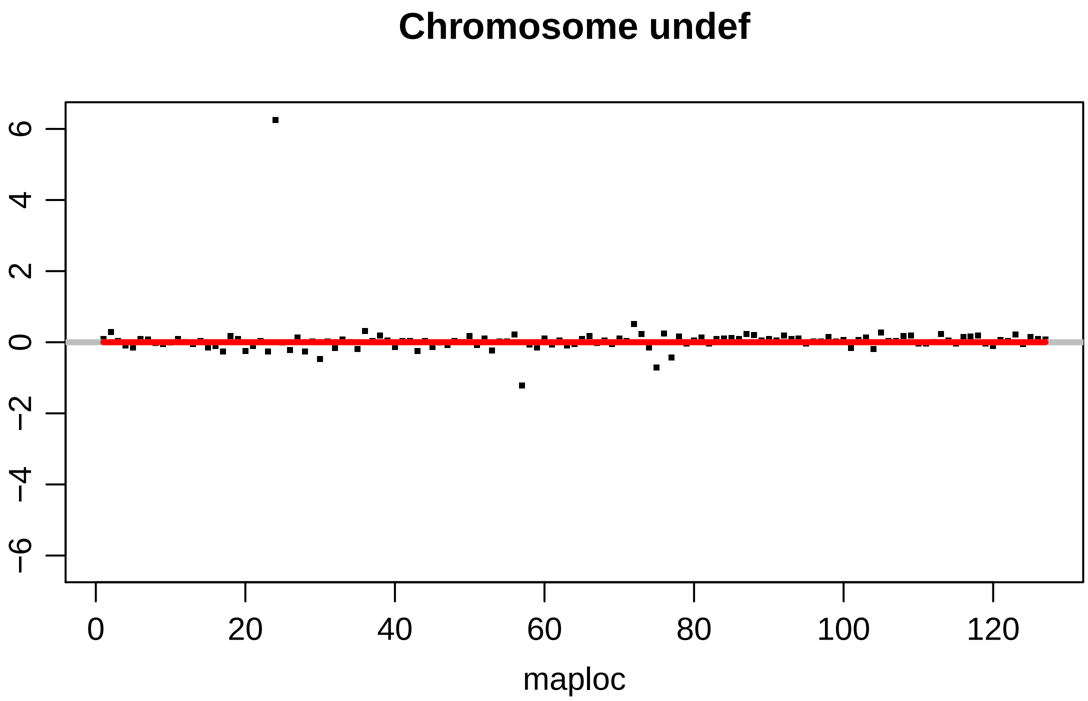

```
## Segplot might not work because of special characters in the sample names. Use only A-Z,a-z and 0-9!
##  There is a hidden function cn.mops:::.replaceNames that replaces the names in the "CNVDetectionResul
```

s_021_R_2012_09_13_10_57_38_Sequoia_SN1.27.Run_21_Auto_Sequoia_SN1.27.I

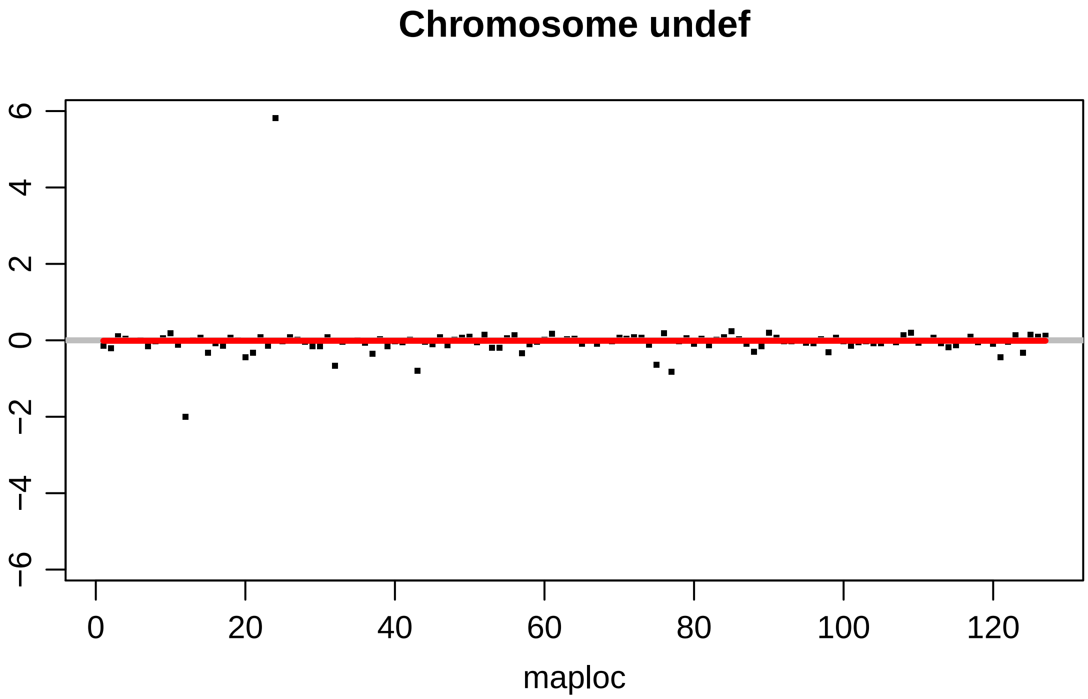


```
## Segplot might not work because of special characters in the sample names. Use only A-Z,a-z and 0-9!
##  There is a hidden function cn.mops:::.replaceNames that replaces the names in the "CNVDetectionResul
```

s_022_R_2012_09_13_10_57_38_Sequoia_SN1.27.Run_21_Auto_Sequoia_SN1.27.I

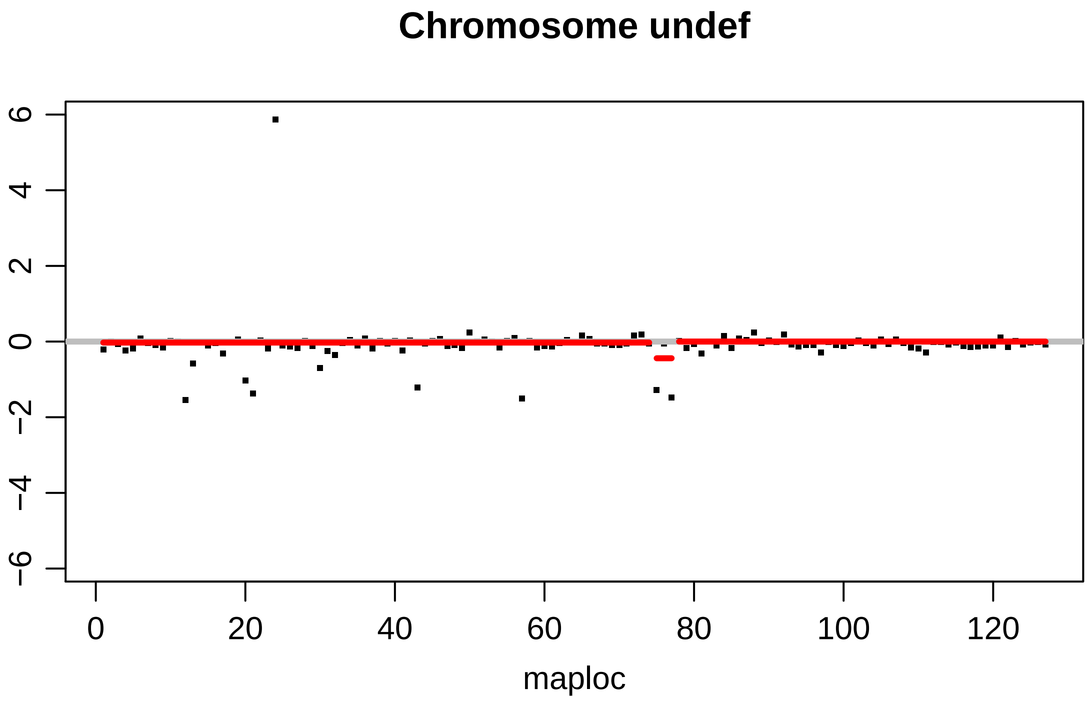


```
## Segplot might not work because of special characters in the sample names. Use only A-Z,a-z and 0-9!
##  There is a hidden function cn.mops:::.replaceNames that replaces the names in the "CNVDetectionResul
```

s_023_R_2012_09_13_10_57_38_Sequoia_SN1.27.Run_21_Auto_Sequoia_SN1.27.I

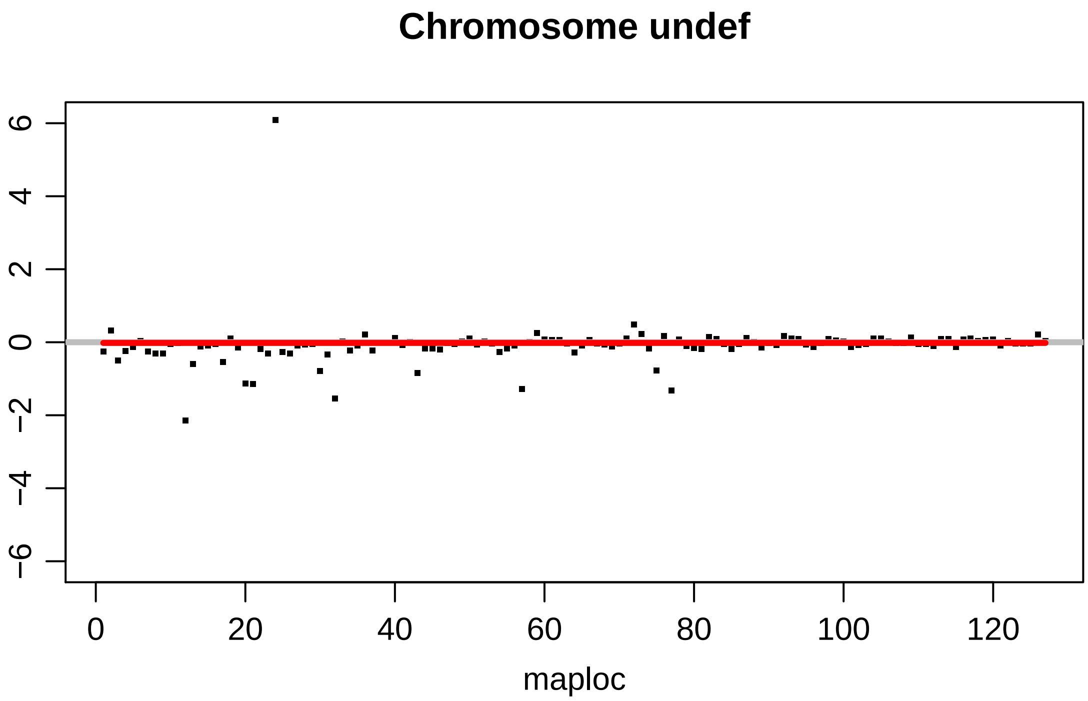

```
## Segplot might not work because of special characters in the sample names. Use only A-Z,a-z and 0-9!
##  There is a hidden function cn.mops:::.replaceNames that replaces the names in the "CNVDetectionResul
```

s_024_R_2012_09_13_10_57_38_Sequoia_SN1.27.Run_21_Auto_Sequoia_SN1.27.I

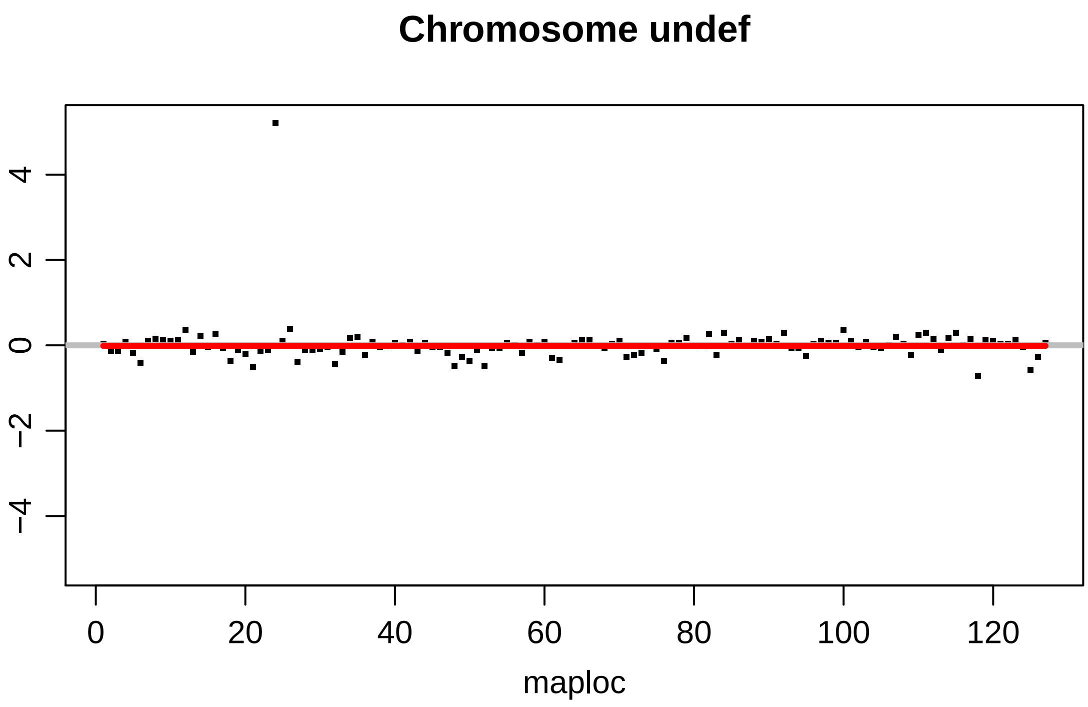


```
## Segplot might not work because of special characters in the sample names. Use only A-Z,a-z and 0-9!
##  There is a hidden function cn.mops:::.replaceNames that replaces the names in the "CNVDetectionResul
```

s_025_R_2012_09_13_10_57_38_Sequoia_SN1.27.Run_21_Auto_Sequoia_SN1.27.I

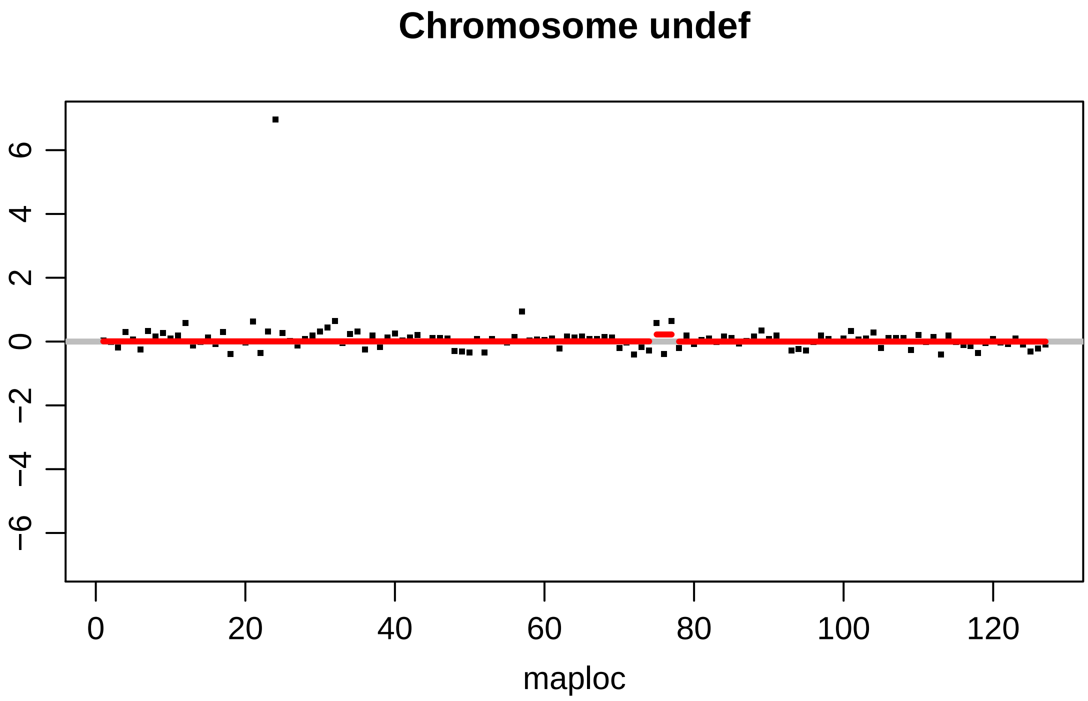


```
## Segplot might not work because of special characters in the sample names. Use only A-Z,a-z and 0-9!
##  There is a hidden function cn.mops:::.replaceNames that replaces the names in the "CNVDetectionResul
```

s_026_R_2012_09_13_10_57_38_Sequoia_SN1.27.Run_21_Auto_Sequoia_SN1.27.I

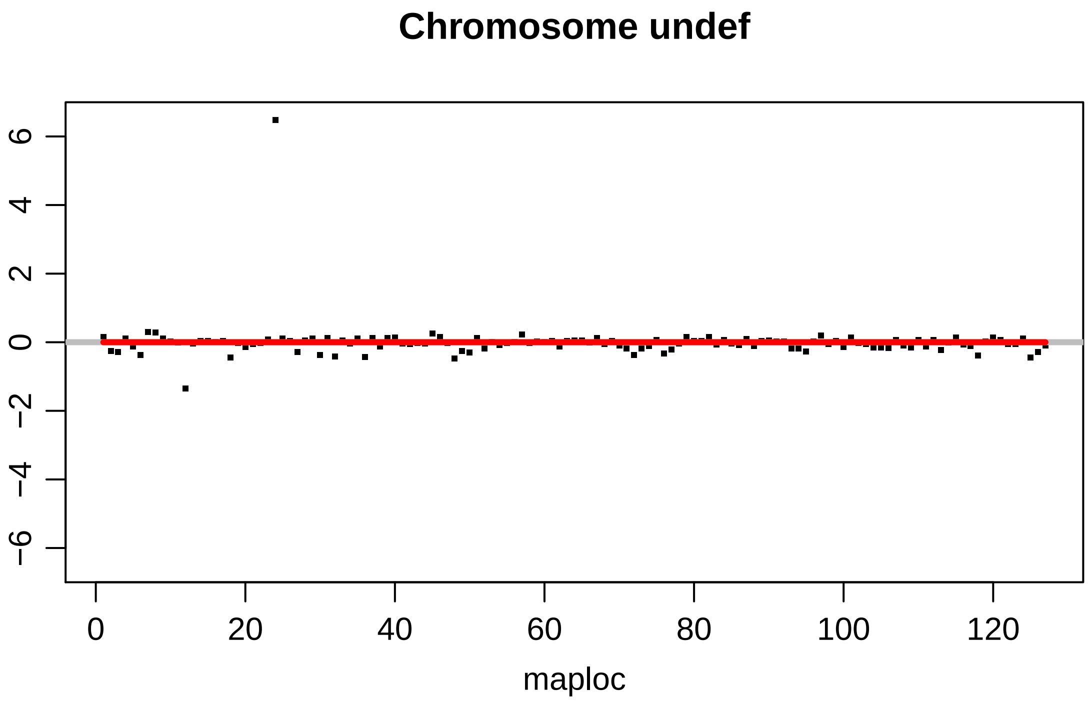

```
## Segplot might not work because of special characters in the sample names. Use only A-Z,a-z and 0-9!
##  There is a hidden function cn.mops:::.replaceNames that replaces the names in the "CNVDetectionResul
```

s_027_R_2012_09_13_10_57_38_Sequoia_SN1.27.Run_21_Auto_Sequoia_SN1.27.I

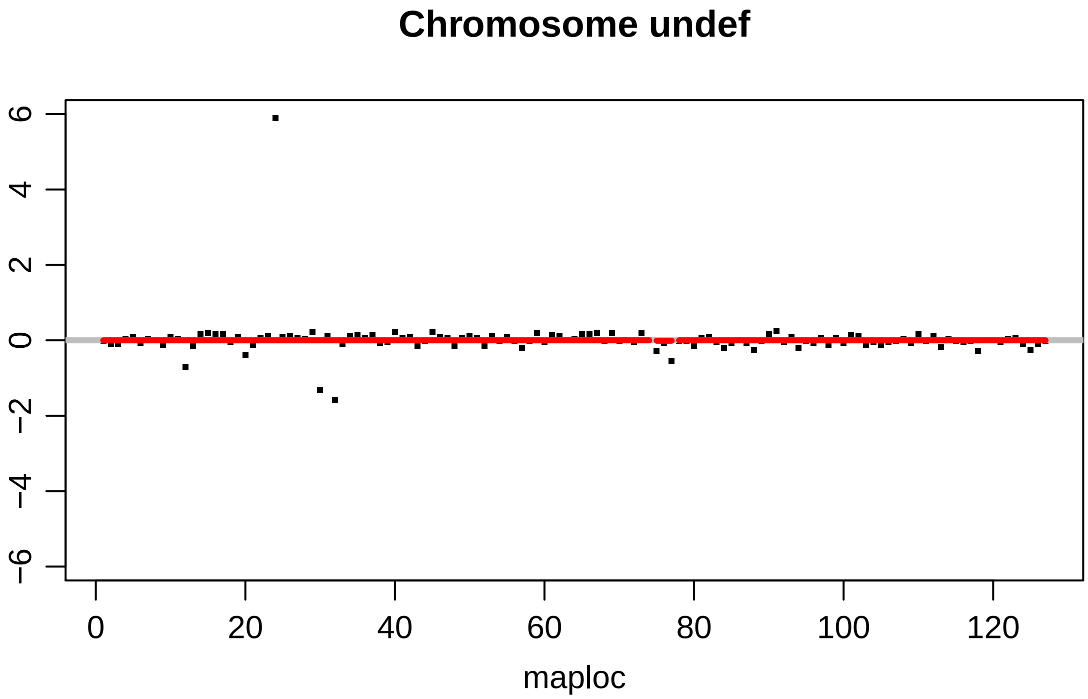


```
## Segplot might not work because of special characters in the sample names. Use only A-Z,a-z and 0-9!
##  There is a hidden function cn.mops:::.replaceNames that replaces the names in the "CNVDetectionResul
```

s_028_R_2012_09_13_10_57_38_Sequoia_SN1.27.Run_21_Auto_Sequoia_SN1.27.I

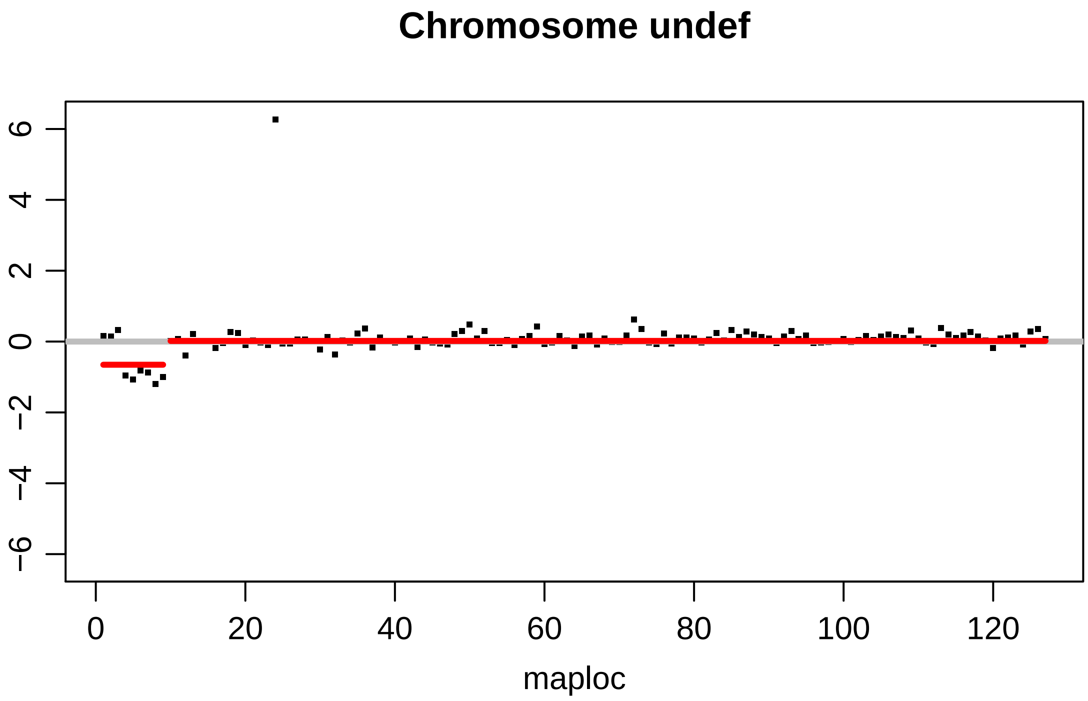


```
## Segplot might not work because of special characters in the sample names. Use only A-Z,a-z and 0-9!
##  There is a hidden function cn.mops:::.replaceNames that replaces the names in the "CNVDetectionResul
```

s_029_R_2012_09_13_10_57_38_Sequoia_SN1.27.Run_21_Auto_Sequoia_SN1.27.I

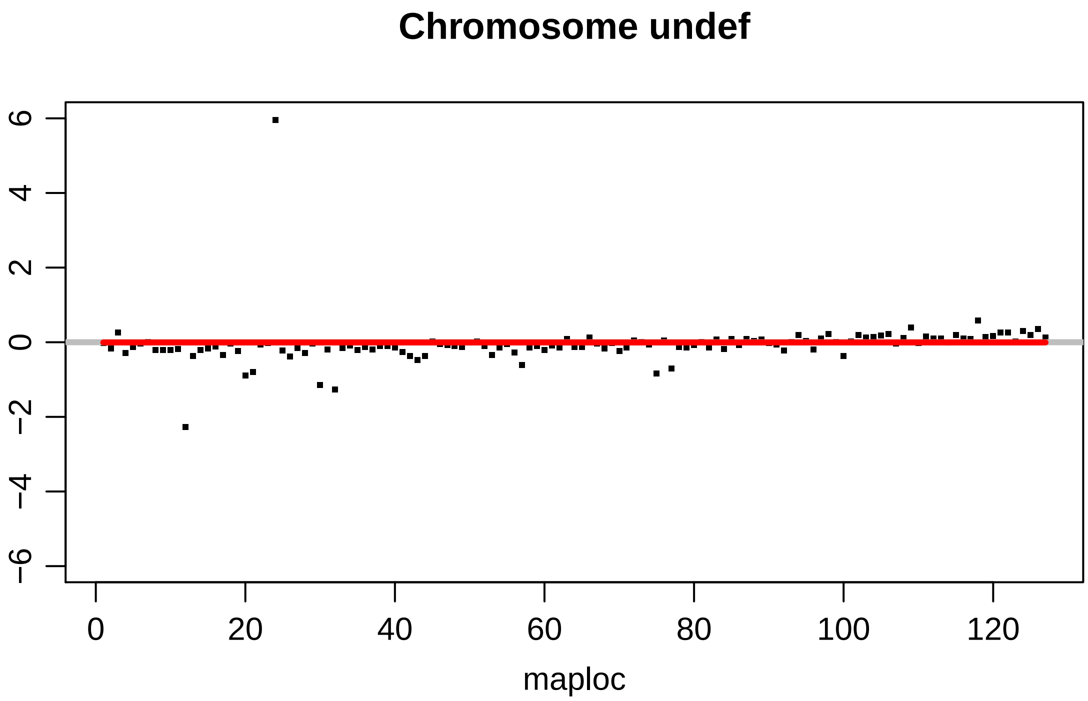

```
## Segplot might not work because of special characters in the sample names. Use only A-Z,a-z and 0-9!
##  There is a hidden function cn.mops:::.replaceNames that replaces the names in the "CNVDetectionResul
```

s_030_R_2012_09_13_10_57_38_Sequoia_SN1.27.Run_21_Auto_Sequoia_SN1.27.I

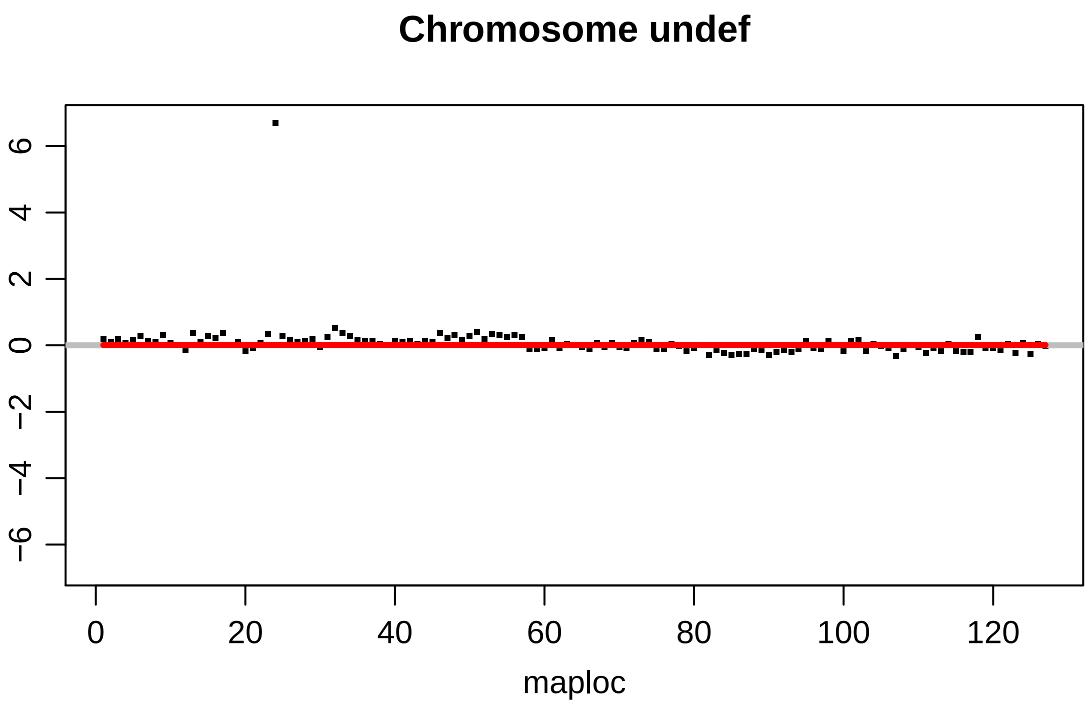


```
## Segplot might not work because of special characters in the sample names. Use only A-Z,a-z and 0-9!
##  There is a hidden function cn.mops:::.replaceNames that replaces the names in the "CNVDetectionResul
```

s_031_R_2012_09_13_10_57_38_Sequoia_SN1.27.Run_21_Auto_Sequoia_SN1.27.I

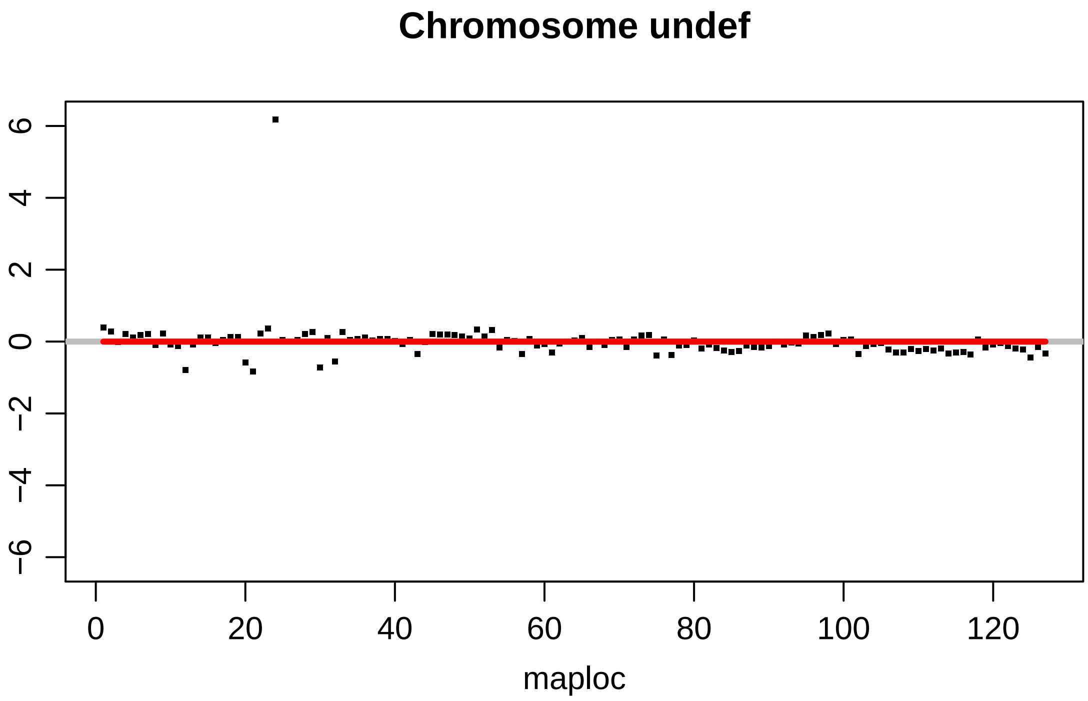


```
## Segplot might not work because of special characters in the sample names. Use only A-Z,a-z and 0-9!
##  There is a hidden function cn.mops:::.replaceNames that replaces the names in the "CNVDetectionResul
```

s_032_R_2012_09_13_10_57_38_Sequoia_SN1.27.Run_21_Auto_Sequoia_SN1.27.I

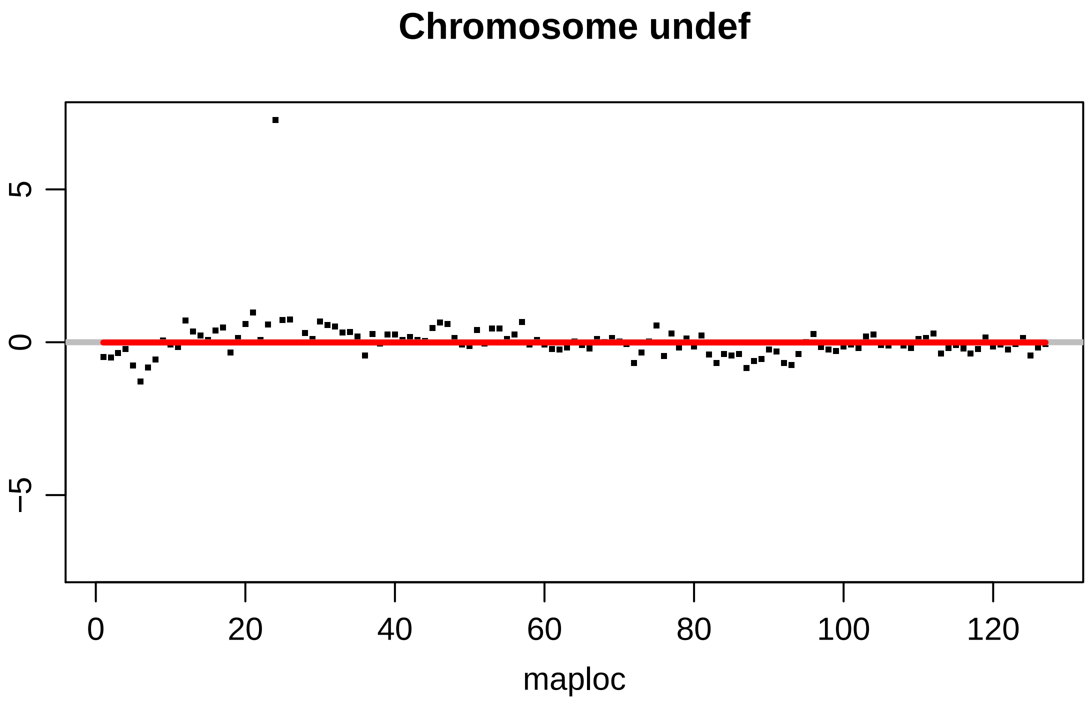

```
## Segplot might not work because of special characters in the sample names. Use only A-Z,a-z and 0-9!
##  There is a hidden function cn.mops:::.replaceNames that replaces the names in the "CNVDetectionResul
```

s_033_R_2012_09_13_10_57_38_Sequoia_SN1.27.Run_21_Auto_Sequoia_SN1.27.I

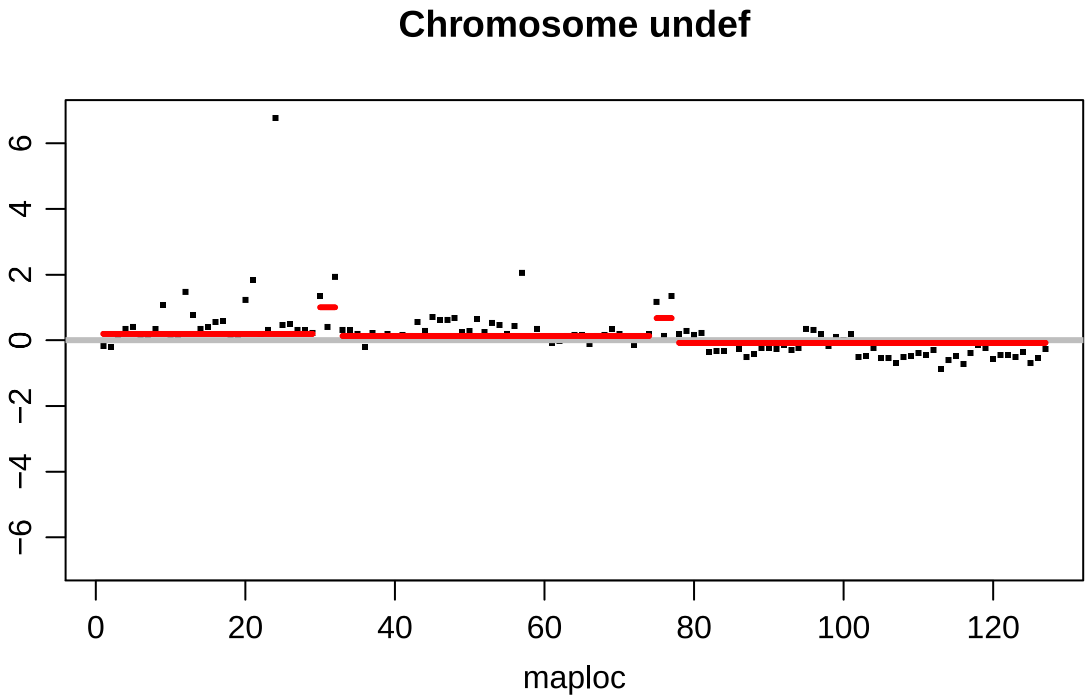


```
## Segplot might not work because of special characters in the sample names. Use only A-Z,a-z and 0-9!
##  There is a hidden function cn.mops:::.replaceNames that replaces the names in the "CNVDetectionResul
```

s_034_R_2012_09_13_10_57_38_Sequoia_SN1.27.Run_21_Auto_Sequoia_SN1.27.I

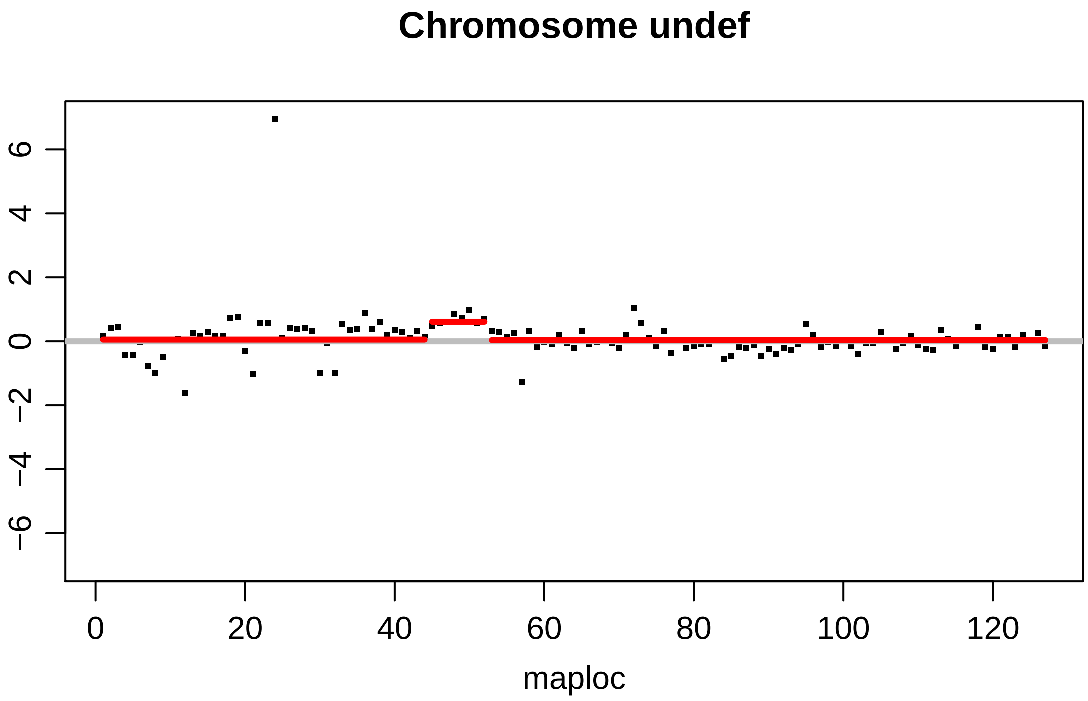


```
## Segplot might not work because of special characters in the sample names. Use only A-Z,a-z and 0-9!
##  There is a hidden function cn.mops:::.replaceNames that replaces the names in the "CNVDetectionResul
```

s_035_R_2012_09_13_10_57_38_Sequoia_SN1.27.Run_21_Auto_Sequoia_SN1.27.I

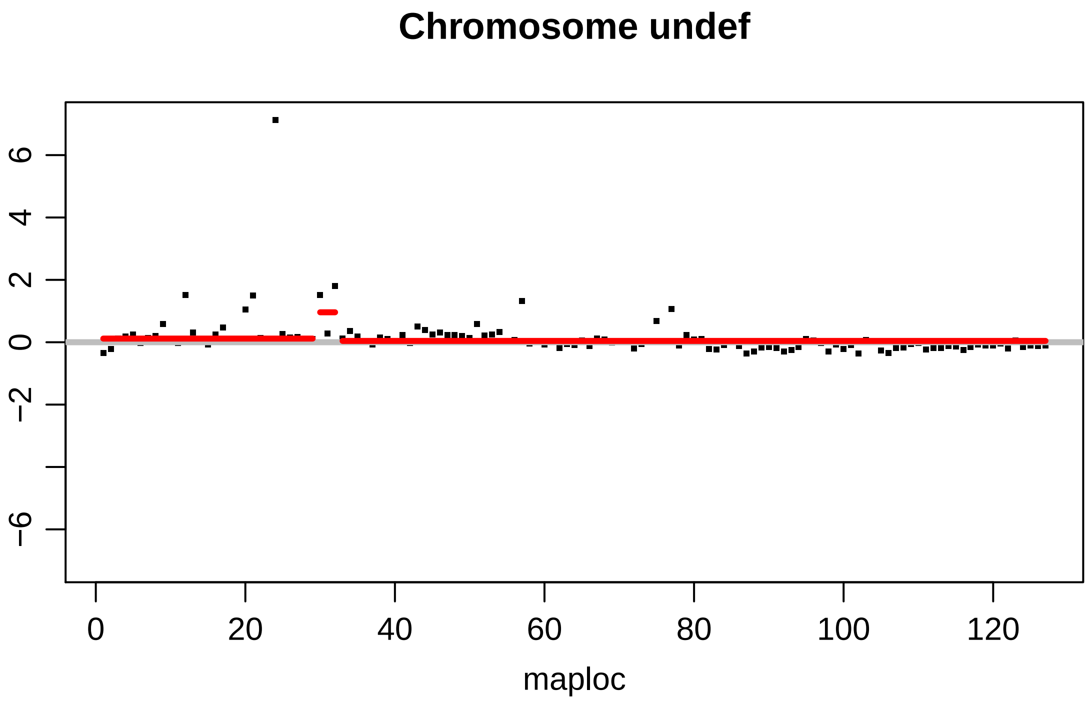

```
## Segplot might not work because of special characters in the sample names. Use only A-Z,a-z and 0-9!
##  There is a hidden function cn.mops:::.replaceNames that replaces the names in the "CNVDetectionResul
```

s_036_R_2012_09_13_10_57_38_Sequoia_SN1.27.Run_21_Auto_Sequoia_SN1.27.I

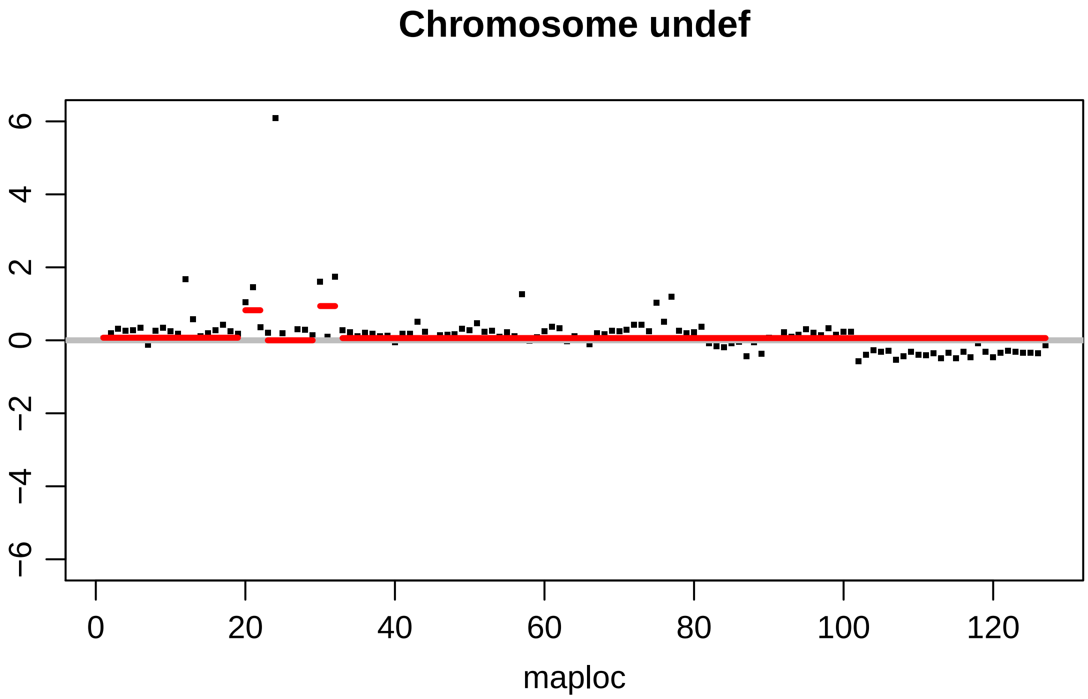


```
## Segplot might not work because of special characters in the sample names. Use only A-Z,a-z and 0-9!
##  There is a hidden function cn.mops:::.replaceNames that replaces the names in the "CNVDetectionResul
```

s_037_R_2012_09_13_10_57_38_Sequoia_SN1.27.Run_21_Auto_Sequoia_SN1.27.I

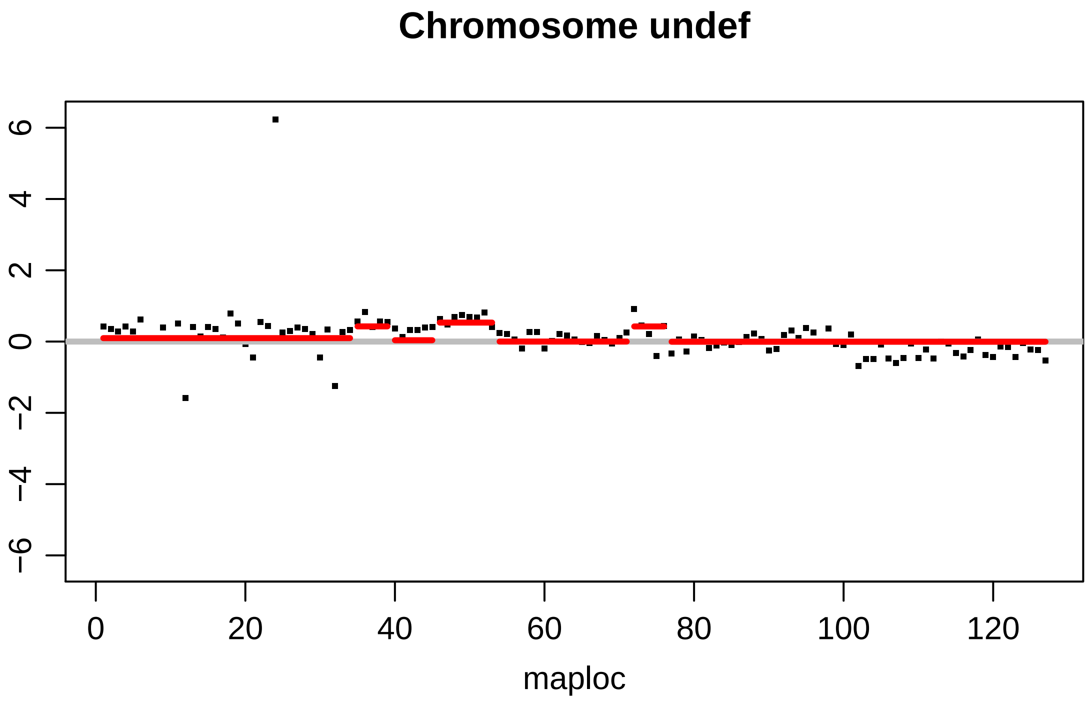


```
## Segplot might not work because of special characters in the sample names. Use only A-Z,a-z and 0-9!
##  There is a hidden function cn.mops:::.replaceNames that replaces the names in the "CNVDetectionResul
```

s_038_R_2012_09_13_10_57_38_Sequoia_SN1.27.Run_21_Auto_Sequoia_SN1.27.I

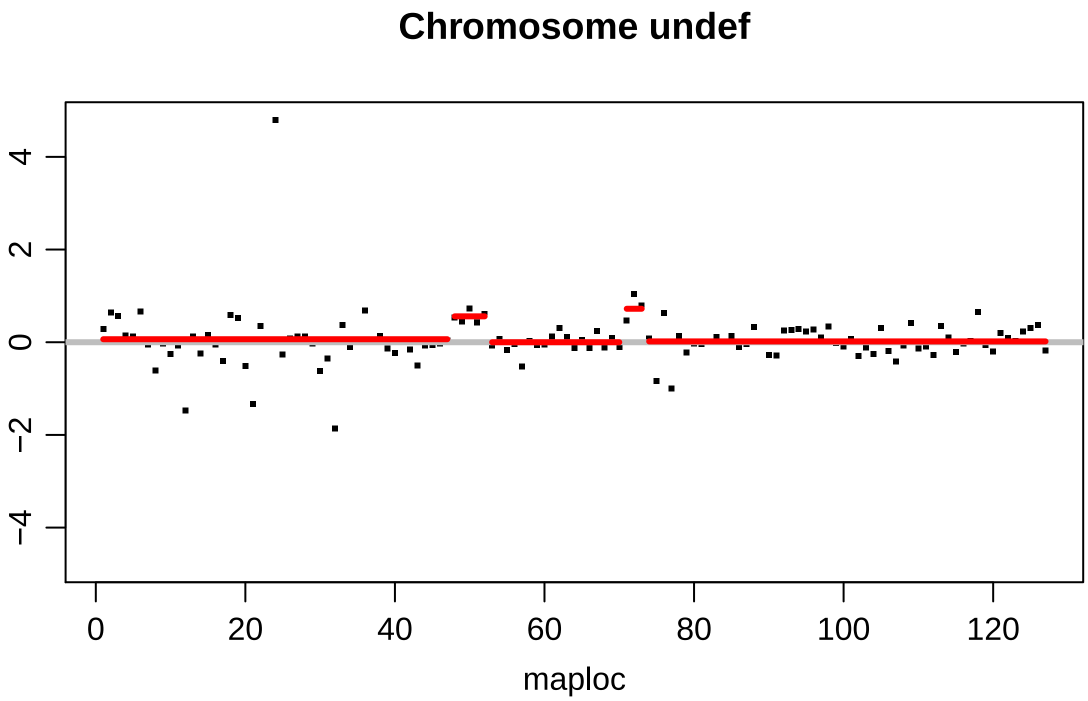

```
## Segplot might not work because of special characters in the sample names. Use only A-Z,a-z and 0-9!
##  There is a hidden function cn.mops:::.replaceNames that replaces the names in the "CNVDetectionResul
```

s_039_R_2012_09_13_10_57_38_Sequoia_SN1.27.Run_21_Auto_Sequoia_SN1.27.I

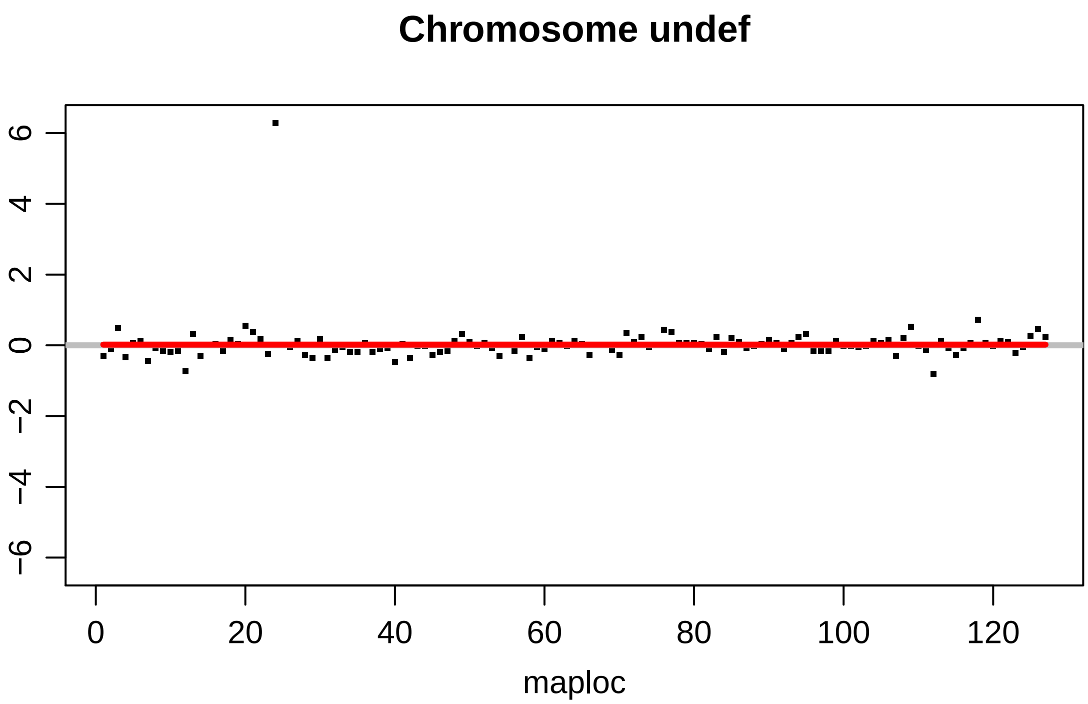


```
## Segplot might not work because of special characters in the sample names. Use only A-Z,a-z and 0-9!
##  There is a hidden function cn.mops:::.replaceNames that replaces the names in the "CNVDetectionResul
```

s_040_R_2012_09_13_10_57_38_Sequoia_SN1.27.Run_21_Auto_Sequoia_SN1.27.I

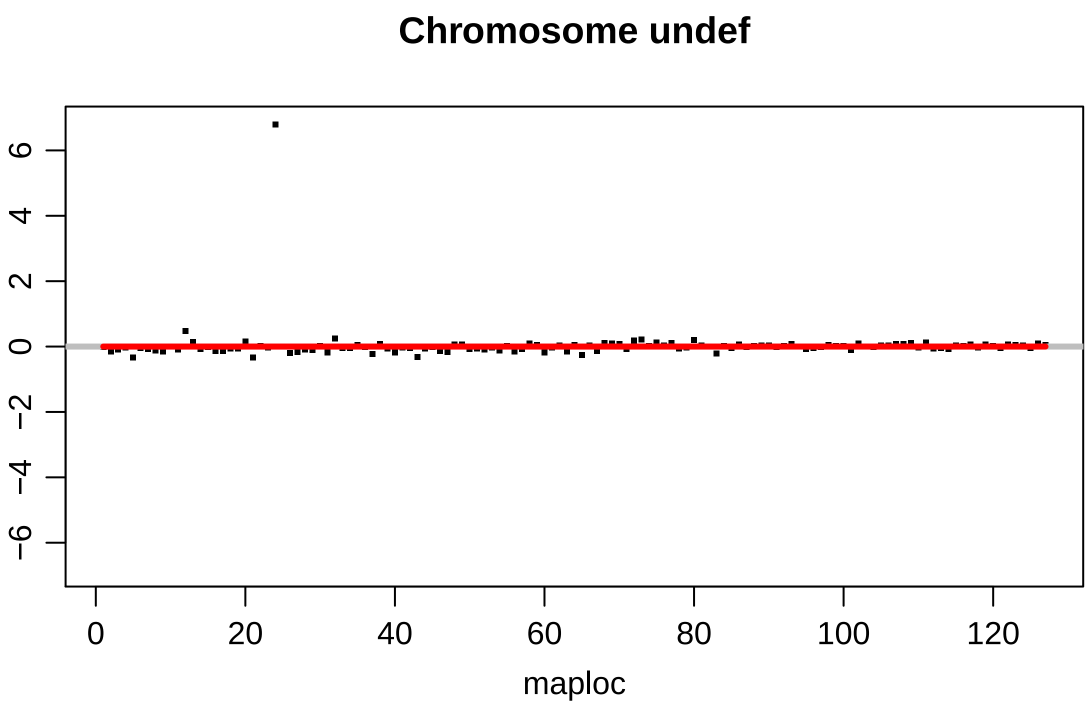


```
## Segplot might not work because of special characters in the sample names. Use only A-Z,a-z and 0-9!
##  There is a hidden function cn.mops:::.replaceNames that replaces the names in the "CNVDetectionResul
```

s_041_R_2012_09_13_10_57_38_Sequoia_SN1.27.Run_21_Auto_Sequoia_SN1.27.I

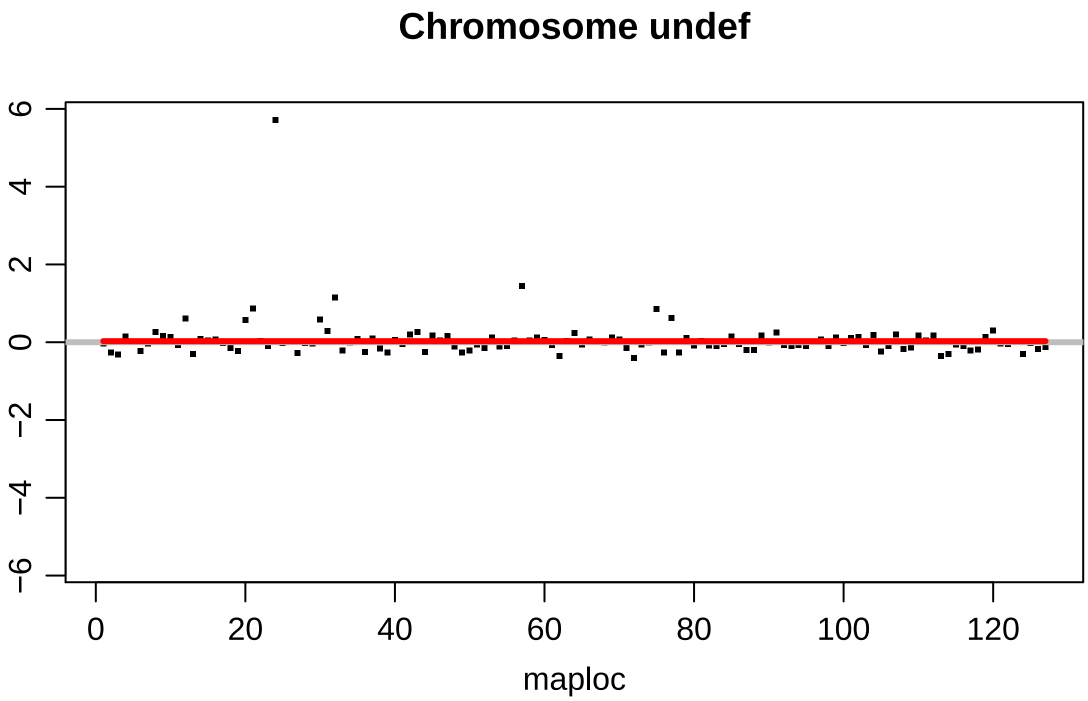

```
## Segplot might not work because of special characters in the sample names. Use only A-Z,a-z and 0-9!
##  There is a hidden function cn.mops:::.replaceNames that replaces the names in the "CNVDetectionResul
```

s_042_R_2012_09_13_10_57_38_Sequoia_SN1.27.Run_21_Auto_Sequoia_SN1.27.F

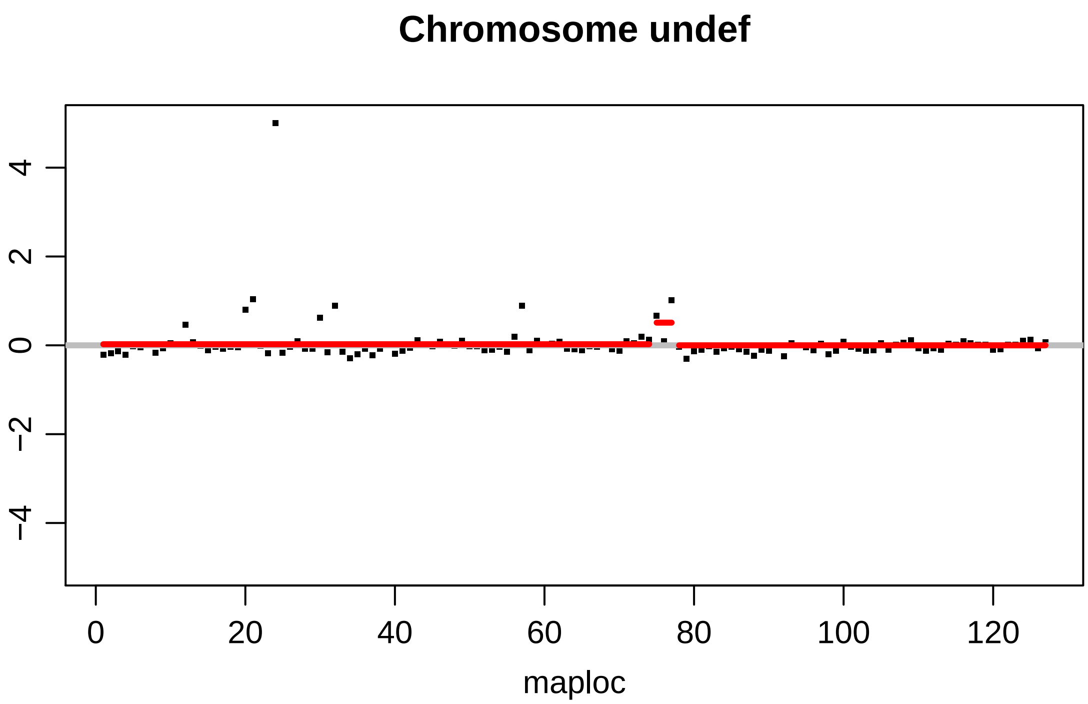


```
## Segplot might not work because of special characters in the sample names. Use only A-Z,a-z and 0-9!
##  There is a hidden function cn.mops:::.replaceNames that replaces the names in the "CNVDetectionResul
```

s_044_R_2012_09_13_10_57_38_Sequoia_SN1.27.Run_21_Auto_Sequoia_SN1.27.F

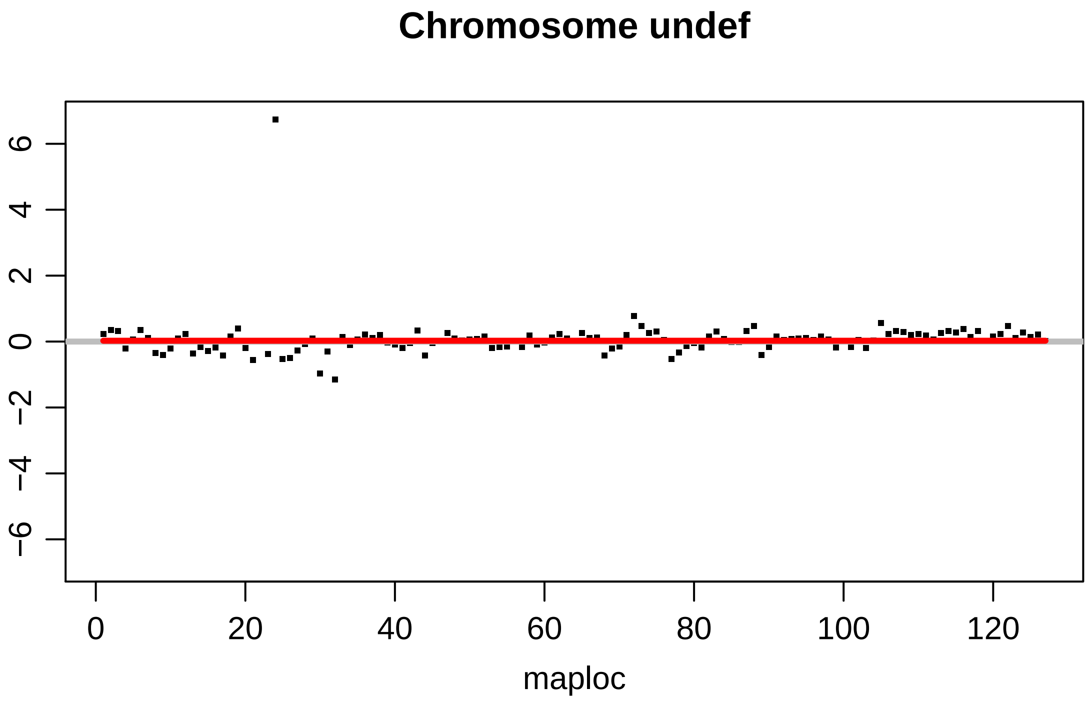


```
## Segplot might not work because of special characters in the sample names. Use only A-Z,a-z and 0-9!
##  There is a hidden function cn.mops:::.replaceNames that replaces the names in the "CNVDetectionResul
```

s_045_R_2012_09_13_10_57_38_Sequoia_SN1.27.Run_21_Auto_Sequoia_SN1.27.F

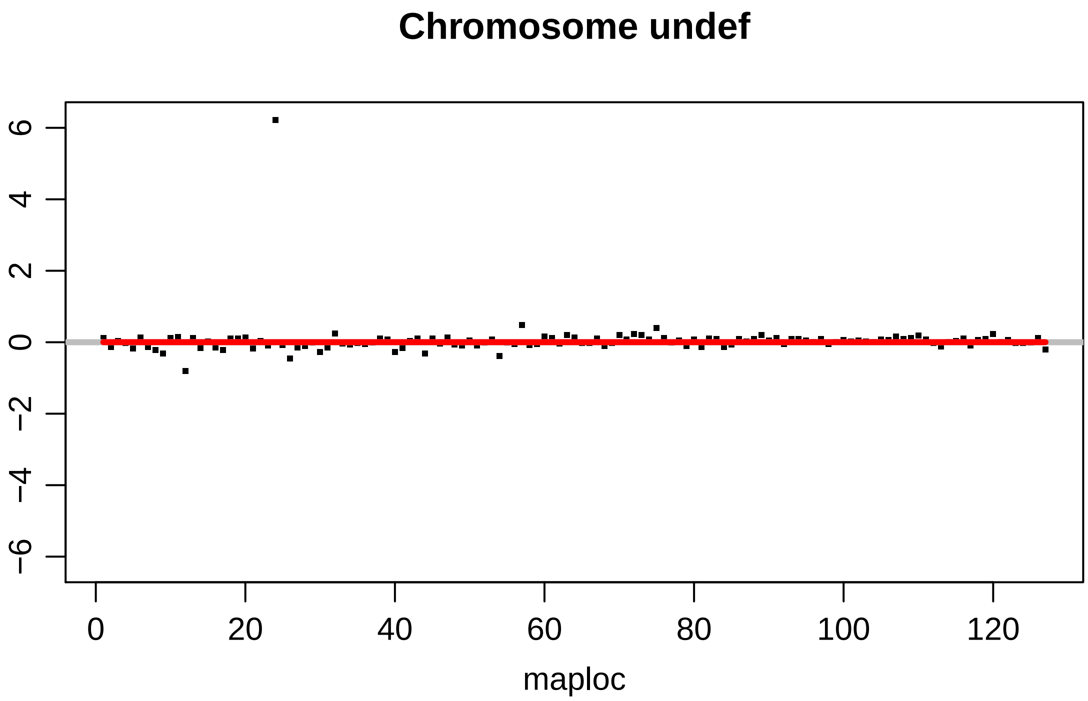

```
## Segplot might not work because of special characters in the sample names. Use only A-Z,a-z and 0-9!
##  There is a hidden function cn.mops:::.replaceNames that replaces the names in the "CNVDetectionResul
```

s_046_R_2012_09_13_10_57_38_Sequoia_SN1.27.Run_21_Auto_Sequoia_SN1.27.I

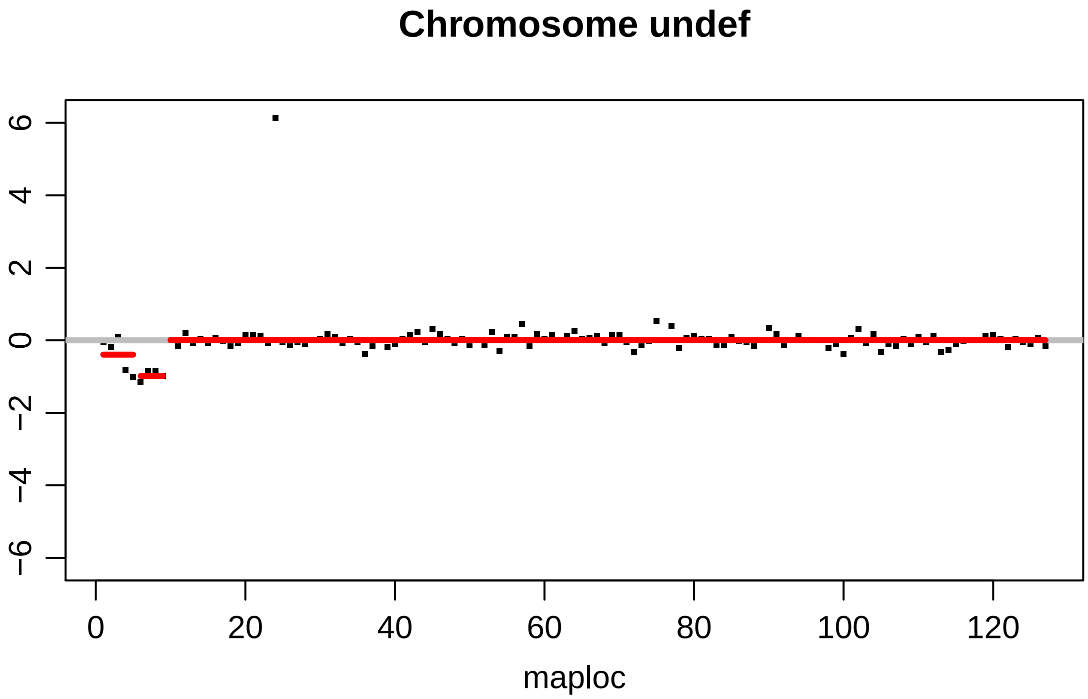


```
## Segplot might not work because of special characters in the sample names. Use only A-Z,a-z and 0-9!
##  There is a hidden function cn.mops:::.replaceNames that replaces the names in the "CNVDetectionResul
```

s_047_R_2012_09_13_10_57_38_Sequoia_SN1.27.Run_21_Auto_Sequoia_SN1.27.I

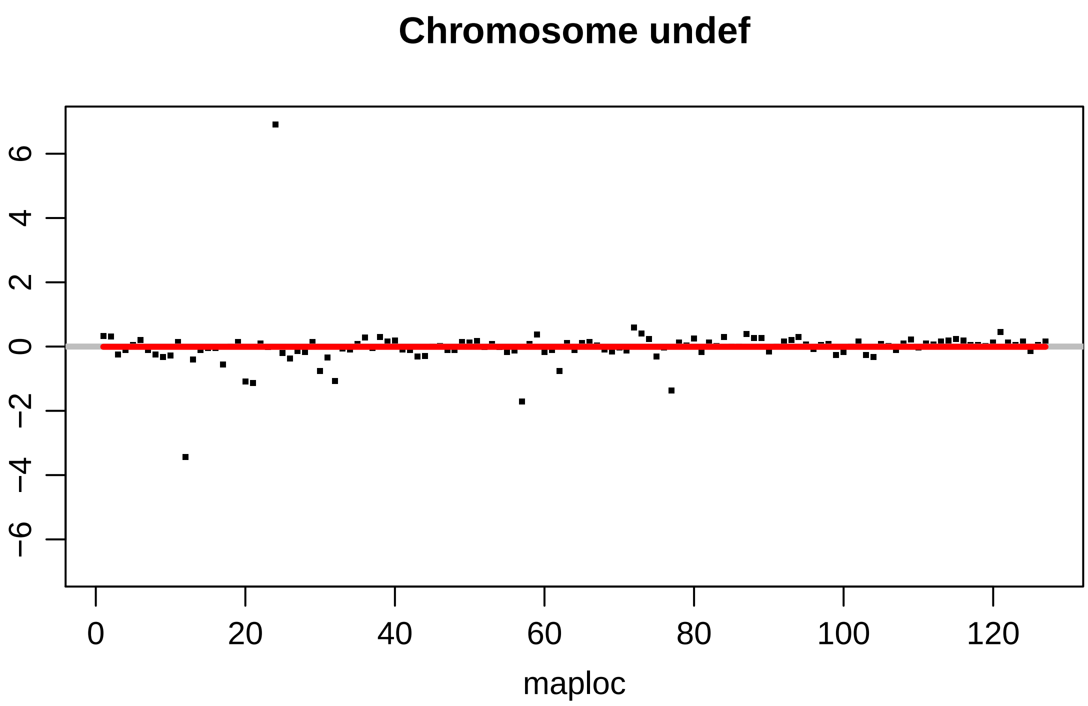


```
##
## CNV regions:
## GRanges object with 6 ranges and 44 metadata columns:
##       seqnames    ranges strand |
##          <Rle> <IRanges>  <Rle> |
##   [1]    undef  [ 6,  9]      * |
##   [2]    undef  [18, 22]      * |
##   [3]    undef  [30, 32]      * |
##   [4]    undef  [45, 53]      * |
##   [5]    undef  [71, 73]      * |
##   [6]    undef  [75, 77]      * |
##       Case_IonXpress_002_R_2012_09_13_10_57_38_Sequoia_SN1.27.Run_21_Auto_Sequoia_SN1.27.Run_21_54.sa
##                                                                                                <facto
##   [1]                                                                                               Cl
##   [2]                                                                                               Cl
##   [3]                                                                                               Cl
##   [4]                                                                                               Cl
##   [5]                                                                                               Cl
##   [6]                                                                                               Cl
##       Case_IonXpress_004_R_2012_09_13_10_57_38_Sequoia_SN1.27.Run_21_Auto_Sequoia_SN1.27.Run_21_54.sa
##                                                                                                <facto
##   [1]                                                                                               Cl
##   [2]                                                                                               Cl
##   [3]                                                                                               Cl
##   [4]                                                                                               Cl
##   [5]                                                                                               Cl
##   [6]                                                                                               Cl
##       Case_IonXpress_005_R_2012_09_13_10_57_38_Sequoia_SN1.27.Run_21_Auto_Sequoia_SN1.27.Run_21_54.sa
```

```
##                                                                                     <facto
##   [1]                                                                                   Cl
##   [2]                                                                                   Cl
##   [3]                                                                                   Cl
##   [4]                                                                                   Cl
##   [5]                                                                                   Cl
##   [6]                                                                                   Cl
##      Case_IonXpress_006_R_2012_09_13_10_57_38_Sequoia_SN1.27.Run_21_Auto_Sequoia_SN1.27.Run_21_54.sa
##                                                                                     <facto
##   [1]                                                                                   Cl
##   [2]                                                                                   Cl
##   [3]                                                                                   Cl
##   [4]                                                                                   Cl
##   [5]                                                                                   Cl
##   [6]                                                                                   Cl
##      Case_IonXpress_007_R_2012_09_13_10_57_38_Sequoia_SN1.27.Run_21_Auto_Sequoia_SN1.27.Run_21_54.sa
##                                                                                     <facto
##   [1]                                                                                   Cl
##   [2]                                                                                   Cl
##   [3]                                                                                   Cl
##   [4]                                                                                   Cl
##   [5]                                                                                   Cl
##   [6]                                                                                   Cl
##      Case_IonXpress_009_R_2012_09_13_10_57_38_Sequoia_SN1.27.Run_21_Auto_Sequoia_SN1.27.Run_21_54.sa
##                                                                                     <facto
##   [1]                                                                                   Cl
##   [2]                                                                                   Cl
##   [3]                                                                                   Cl
##   [4]                                                                                   Cl
##   [5]                                                                                   Cl
##   [6]                                                                                   Cl
##      Case_IonXpress_010_R_2012_09_13_10_57_38_Sequoia_SN1.27.Run_21_Auto_Sequoia_SN1.27.Run_21_54.sa
##                                                                                     <facto
##   [1]                                                                                   Cl
##   [2]                                                                                   Cl
##   [3]                                                                                   Cl
##   [4]                                                                                   Cl
##   [5]                                                                                   Cl
##   [6]                                                                                   Cl
##      Case_IonXpress_011_R_2012_09_13_10_57_38_Sequoia_SN1.27.Run_21_Auto_Sequoia_SN1.27.Run_21_54.sa
##                                                                                     <facto
##   [1]                                                                                   Cl
##   [2]                                                                                   Cl
##   [3]                                                                                   Cl
##   [4]                                                                                   Cl
##   [5]                                                                                   Cl
##   [6]                                                                                   Cl
##      Case_IonXpress_012_R_2012_09_13_10_57_38_Sequoia_SN1.27.Run_21_Auto_Sequoia_SN1.27.Run_21_54.sa
##                                                                                     <facto
##   [1]                                                                                   Cl
##   [2]                                                                                   Cl
##   [3]                                                                                   Cl
##   [4]                                                                                   Cl
##   [5]                                                                                   Cl
```

```
## [6] Cl
## Case_IonXpress_013_R_2012_09_13_10_57_38_Sequoia_SN1.27.Run_21_Auto_Sequoia_SN1.27.Run_21_54.sa
## <facto
## [1] Cl
## [2] Cl
## [3] Cl
## [4] Cl
## [5] Cl
## [6] Cl
## Case_IonXpress_014_R_2012_09_13_10_57_38_Sequoia_SN1.27.Run_21_Auto_Sequoia_SN1.27.Run_21_54.sa
## <facto
## [1] Cl
## [2] Cl
## [3] Cl
## [4] Cl
## [5] Cl
## [6] Cl
## Case_IonXpress_015_R_2012_09_13_10_57_38_Sequoia_SN1.27.Run_21_Auto_Sequoia_SN1.27.Run_21_54.sa
## <facto
## [1] Cl
## [2] Cl
## [3] Cl
## [4] Cl
## [5] Cl
## [6] Cl
## Case_IonXpress_016_R_2012_09_13_10_57_38_Sequoia_SN1.27.Run_21_Auto_Sequoia_SN1.27.Run_21_54.sa
## <facto
## [1] Cl
## [2] Cl
## [3] Cl
## [4] Cl
## [5] Cl
## [6] Cl
## Case_IonXpress_017_R_2012_09_13_10_57_38_Sequoia_SN1.27.Run_21_Auto_Sequoia_SN1.27.Run_21_54.sa
## <facto
## [1] Cl
## [2] Cl
## [3] Cl
## [4] Cl
## [5] Cl
## [6] Cl
## Case_IonXpress_018_R_2012_09_13_10_57_38_Sequoia_SN1.27.Run_21_Auto_Sequoia_SN1.27.Run_21_54.sa
## <facto
## [1] Cl
## [2] Cl
## [3] Cl
## [4] Cl
## [5] Cl
## [6] Cl
## Case_IonXpress_019_R_2012_09_13_10_57_38_Sequoia_SN1.27.Run_21_Auto_Sequoia_SN1.27.Run_21_54.sa
## <facto
## [1] Cl
## [2] Cl
## [3] Cl
```

```
## [4]                                                                                                    Cl
## [5]                                                                                                    Cl
## [6]                                                                                                    Cl
##      Case_IonXpress_020_R_2012_09_13_10_57_38_Sequoia_SN1.27.Run_21_Auto_Sequoia_SN1.27.Run_21_54.sa
##                                                                                                   <facto
## [1]                                                                                                    Cl
## [2]                                                                                                    Cl
## [3]                                                                                                    Cl
## [4]                                                                                                    Cl
## [5]                                                                                                    Cl
## [6]                                                                                                    Cl
##      Case_IonXpress_021_R_2012_09_13_10_57_38_Sequoia_SN1.27.Run_21_Auto_Sequoia_SN1.27.Run_21_54.sa
##                                                                                                   <facto
## [1]                                                                                                    Cl
## [2]                                                                                                    Cl
## [3]                                                                                                    Cl
## [4]                                                                                                    Cl
## [5]                                                                                                    Cl
## [6]                                                                                                    Cl
##      Case_IonXpress_022_R_2012_09_13_10_57_38_Sequoia_SN1.27.Run_21_Auto_Sequoia_SN1.27.Run_21_54.sa
##                                                                                                   <facto
## [1]                                                                                                    Cl
## [2]                                                                                                    Cl
## [3]                                                                                                    Cl
## [4]                                                                                                    Cl
## [5]                                                                                                    Cl
## [6]                                                                                                    Cl
##      Case_IonXpress_023_R_2012_09_13_10_57_38_Sequoia_SN1.27.Run_21_Auto_Sequoia_SN1.27.Run_21_54.sa
##                                                                                                   <facto
## [1]                                                                                                    Cl
## [2]                                                                                                    Cl
## [3]                                                                                                    Cl
## [4]                                                                                                    Cl
## [5]                                                                                                    Cl
## [6]                                                                                                    Cl
##      Case_IonXpress_024_R_2012_09_13_10_57_38_Sequoia_SN1.27.Run_21_Auto_Sequoia_SN1.27.Run_21_54.sa
##                                                                                                   <facto
## [1]                                                                                                    Cl
## [2]                                                                                                    Cl
## [3]                                                                                                    Cl
## [4]                                                                                                    Cl
## [5]                                                                                                    Cl
## [6]                                                                                                    Cl
##      Case_IonXpress_025_R_2012_09_13_10_57_38_Sequoia_SN1.27.Run_21_Auto_Sequoia_SN1.27.Run_21_54.sa
##                                                                                                   <facto
## [1]                                                                                                    Cl
## [2]                                                                                                    Cl
## [3]                                                                                                    Cl
## [4]                                                                                                    Cl
## [5]                                                                                                    Cl
## [6]                                                                                                    Cl
##      Case_IonXpress_026_R_2012_09_13_10_57_38_Sequoia_SN1.27.Run_21_Auto_Sequoia_SN1.27.Run_21_54.sa
##                                                                                                   <facto
## [1]                                                                                                    Cl
```

```
## [2] Cl
## [3] Cl
## [4] Cl
## [5] Cl
## [6] Cl
## Case_IonXpress_027_R_2012_09_13_10_57_38_Sequoia_SN1.27.Run_21_Auto_Sequoia_SN1.27.Run_21_54.sa
## <facto
## [1] Cl
## [2] Cl
## [3] Cl
## [4] Cl
## [5] Cl
## [6] Cl
## Case_IonXpress_028_R_2012_09_13_10_57_38_Sequoia_SN1.27.Run_21_Auto_Sequoia_SN1.27.Run_21_54.sa
## <facto
## [1] Cl
## [2] Cl
## [3] Cl
## [4] Cl
## [5] Cl
## [6] Cl
## Case_IonXpress_029_R_2012_09_13_10_57_38_Sequoia_SN1.27.Run_21_Auto_Sequoia_SN1.27.Run_21_54.sa
## <facto
## [1] Cl
## [2] Cl
## [3] Cl
## [4] Cl
## [5] Cl
## [6] Cl
## Case_IonXpress_030_R_2012_09_13_10_57_38_Sequoia_SN1.27.Run_21_Auto_Sequoia_SN1.27.Run_21_54.sa
## <facto
## [1] Cl
## [2] Cl
## [3] Cl
## [4] Cl
## [5] Cl
## [6] Cl
## Case_IonXpress_031_R_2012_09_13_10_57_38_Sequoia_SN1.27.Run_21_Auto_Sequoia_SN1.27.Run_21_54.sa
## <facto
## [1] Cl
## [2] Cl
## [3] Cl
## [4] Cl
## [5] Cl
## [6] Cl
## Case_IonXpress_032_R_2012_09_13_10_57_38_Sequoia_SN1.27.Run_21_Auto_Sequoia_SN1.27.Run_21_54.sa
## <facto
## [1] Cl
## [2] Cl
## [3] Cl
## [4] Cl
## [5] Cl
## [6] Cl
## Case_IonXpress_033_R_2012_09_13_10_57_38_Sequoia_SN1.27.Run_21_Auto_Sequoia_SN1.27.Run_21_54.sa
```

```
##                                                                                                              <facto
##   [1]                                                                                                            Cl
##   [2]                                                                                                            Cl
##   [3]                                                                                                            Cl
##   [4]                                                                                                            Cl
##   [5]                                                                                                            Cl
##   [6]                                                                                                            Cl
##       Case_IonXpress_034_R_2012_09_13_10_57_38_Sequoia_SN1.27.Run_21_Auto_Sequoia_SN1.27.Run_21_54.sa
##                                                                                                              <facto
##   [1]                                                                                                            Cl
##   [2]                                                                                                            Cl
##   [3]                                                                                                            Cl
##   [4]                                                                                                            Cl
##   [5]                                                                                                            Cl
##   [6]                                                                                                            Cl
##       Case_IonXpress_035_R_2012_09_13_10_57_38_Sequoia_SN1.27.Run_21_Auto_Sequoia_SN1.27.Run_21_54.sa
##                                                                                                              <facto
##   [1]                                                                                                            Cl
##   [2]                                                                                                            Cl
##   [3]                                                                                                            Cl
##   [4]                                                                                                            Cl
##   [5]                                                                                                            Cl
##   [6]                                                                                                            Cl
##       Case_IonXpress_036_R_2012_09_13_10_57_38_Sequoia_SN1.27.Run_21_Auto_Sequoia_SN1.27.Run_21_54.sa
##                                                                                                              <facto
##   [1]                                                                                                            Cl
##   [2]                                                                                                            Cl
##   [3]                                                                                                            Cl
##   [4]                                                                                                            Cl
##   [5]                                                                                                            Cl
##   [6]                                                                                                            Cl
##       Case_IonXpress_037_R_2012_09_13_10_57_38_Sequoia_SN1.27.Run_21_Auto_Sequoia_SN1.27.Run_21_54.sa
##                                                                                                              <facto
##   [1]                                                                                                            Cl
##   [2]                                                                                                            Cl
##   [3]                                                                                                            Cl
##   [4]                                                                                                            Cl
##   [5]                                                                                                            Cl
##   [6]                                                                                                            Cl
##       Case_IonXpress_038_R_2012_09_13_10_57_38_Sequoia_SN1.27.Run_21_Auto_Sequoia_SN1.27.Run_21_54.sa
##                                                                                                              <facto
##   [1]                                                                                                            Cl
##   [2]                                                                                                            Cl
##   [3]                                                                                                            Cl
##   [4]                                                                                                            Cl
##   [5]                                                                                                            Cl
##   [6]                                                                                                            Cl
##       Case_IonXpress_039_R_2012_09_13_10_57_38_Sequoia_SN1.27.Run_21_Auto_Sequoia_SN1.27.Run_21_54.sa
##                                                                                                              <facto
##   [1]                                                                                                            Cl
##   [2]                                                                                                            Cl
##   [3]                                                                                                            Cl
##   [4]                                                                                                            Cl
##   [5]                                                                                                            Cl
```

```
##   [6]                                                                                                   C]
##        Case_IonXpress_040_R_2012_09_13_10_57_38_Sequoia_SN1.27.Run_21_Auto_Sequoia_SN1.27.Run_21_54.sa
##                                                                                                   <facto
##   [1]                                                                                                   C]
##   [2]                                                                                                   C]
##   [3]                                                                                                   C]
##   [4]                                                                                                   C]
##   [5]                                                                                                   C]
##   [6]                                                                                                   C]
##        Case_IonXpress_041_R_2012_09_13_10_57_38_Sequoia_SN1.27.Run_21_Auto_Sequoia_SN1.27.Run_21_54.sa
##                                                                                                   <facto
##   [1]                                                                                                   C]
##   [2]                                                                                                   C]
##   [3]                                                                                                   C]
##   [4]                                                                                                   C]
##   [5]                                                                                                   C]
##   [6]                                                                                                   C]
##        Case_IonXpress_042_R_2012_09_13_10_57_38_Sequoia_SN1.27.Run_21_Auto_Sequoia_SN1.27.Run_21_54.sa
##                                                                                                   <facto
##   [1]                                                                                                   C]
##   [2]                                                                                                   C]
##   [3]                                                                                                   C]
##   [4]                                                                                                   C]
##   [5]                                                                                                   C]
##   [6]                                                                                                   C]
##        Case_IonXpress_044_R_2012_09_13_10_57_38_Sequoia_SN1.27.Run_21_Auto_Sequoia_SN1.27.Run_21_54.sa
##                                                                                                   <facto
##   [1]                                                                                                   C]
##   [2]                                                                                                   C]
##   [3]                                                                                                   C]
##   [4]                                                                                                   C]
##   [5]                                                                                                   C]
##   [6]                                                                                                   C]
##        Case_IonXpress_045_R_2012_09_13_10_57_38_Sequoia_SN1.27.Run_21_Auto_Sequoia_SN1.27.Run_21_54.sa
##                                                                                                   <facto
##   [1]                                                                                                   C]
##   [2]                                                                                                   C]
##   [3]                                                                                                   C]
##   [4]                                                                                                   C]
##   [5]                                                                                                   C]
##   [6]                                                                                                   C]
##        Case_IonXpress_046_R_2012_09_13_10_57_38_Sequoia_SN1.27.Run_21_Auto_Sequoia_SN1.27.Run_21_54.sa
##                                                                                                   <facto
##   [1]                                                                                                   C]
##   [2]                                                                                                   C]
##   [3]                                                                                                   C]
##   [4]                                                                                                   C]
##   [5]                                                                                                   C]
##   [6]                                                                                                   C]
##        Case_IonXpress_047_R_2012_09_13_10_57_38_Sequoia_SN1.27.Run_21_Auto_Sequoia_SN1.27.Run_21_54.sa
##                                                                                                   <facto
##   [1]                                                                                                   C]
##   [2]                                                                                                   C]
##   [3]                                                                                                   C]
```

```
##   [4]                                                                                                    Cl
##   [5]                                                                                                    Cl
##   [6]                                                                                                    Cl
##      Case_IonXpress_048_R_2012_09_13_10_57_38_Sequoia_SN1.27.Run_21_Auto_Sequoia_SN1.27.Run_21_54.sa
##                                                                                                 <facto
##   [1]                                                                                                    Cl
##   [2]                                                                                                    Cl
##   [3]                                                                                                    Cl
##   [4]                                                                                                    Cl
##   [5]                                                                                                    Cl
##   [6]                                                                                                    Cl
##   -------
##   seqinfo: 1 sequence from an unspecified genome; no seqlengths
##
## Individual CNVs:
## GRanges object with 16 ranges and 4 metadata columns:
##        seqnames    ranges strand   |
##           <Rle> <IRanges>  <Rle>   |
##    [1]    undef  [18, 21]      *   |
##    [2]    undef  [71, 73]      *   |
##    [3]    undef  [ 6,  9]      *   |
##    [4]    undef  [30, 32]      *   |
##    [5]    undef  [75, 77]      *   |
##    ...      ...       ...    ... ...
##   [12]    undef  [71, 73]      *   |
##   [13]    undef  [75, 77]      *   |
##   [14]    undef  [ 6,  9]      *   |
##   [15]    undef  [30, 32]      *   |
##   [16]    undef  [75, 77]      *   |
##                                                                                                sampleNa
##                                                                                                 <facto
##    [1] Case_IonXpress_004_R_2012_09_13_10_57_38_Sequoia_SN1.27.Run_21_Auto_Sequoia_SN1.27.Run_21_54.s
##    [2] Case_IonXpress_004_R_2012_09_13_10_57_38_Sequoia_SN1.27.Run_21_Auto_Sequoia_SN1.27.Run_21_54.s
##    [3] Case_IonXpress_006_R_2012_09_13_10_57_38_Sequoia_SN1.27.Run_21_Auto_Sequoia_SN1.27.Run_21_54.s
##    [4] Case_IonXpress_033_R_2012_09_13_10_57_38_Sequoia_SN1.27.Run_21_Auto_Sequoia_SN1.27.Run_21_54.s
##    [5] Case_IonXpress_033_R_2012_09_13_10_57_38_Sequoia_SN1.27.Run_21_Auto_Sequoia_SN1.27.Run_21_54.s
##    ...
##   [12] Case_IonXpress_038_R_2012_09_13_10_57_38_Sequoia_SN1.27.Run_21_Auto_Sequoia_SN1.27.Run_21_54.s
##   [13] Case_IonXpress_042_R_2012_09_13_10_57_38_Sequoia_SN1.27.Run_21_Auto_Sequoia_SN1.27.Run_21_54.s
##   [14] Case_IonXpress_046_R_2012_09_13_10_57_38_Sequoia_SN1.27.Run_21_Auto_Sequoia_SN1.27.Run_21_54.s
##   [15] Case_IonXpress_048_R_2012_09_13_10_57_38_Sequoia_SN1.27.Run_21_Auto_Sequoia_SN1.27.Run_21_54.s
##   [16] Case_IonXpress_048_R_2012_09_13_10_57_38_Sequoia_SN1.27.Run_21_Auto_Sequoia_SN1.27.Run_21_54.s
##            median       mean          CN
##         <numeric>  <numeric> <character>
##    [1]  0.5109941  0.5206174         CN3
##    [2]  0.5695447  0.5726523         CN3
##    [3] -0.9981794 -0.9876948         CN1
##    [4]  1.2827508  1.0043294         CN3
##    [5]  0.9965487  0.6755617         CN3
##    ...        ...        ...         ...
##   [12]  0.5849625  0.7219261         CN4
##   [13]  0.5892001  0.5107656         CN3
##   [14] -0.9853496 -0.9845733         CN1
##   [15]  1.3371342  1.0146842         CN3
```

```
##   [16]  0.8715429  0.6490333         CN3
##   -------
##   seqinfo: 1 sequence from an unspecified genome; no seqlengths
## [1] "/Users/gdemidov/Downloads/doc/Run_SN1_28_fin_05_qc.xls"

## Normalizing...

## Starting local modeling, please be patient...

## Reference sequence:  undef

## Starting segmentation algorithm...

## Using "fastseg" for segmentation.

## [1] ""
## [1] "/Users/gdemidov/Downloads/doc/Run_SN1_28_fin_05_qc.xls"
## [1] ""

## Segplot might not work because of special characters in the sample names. Use only A-Z,a-z and 0-9!
##  There is a hidden function cn.mops:::.replaceNames that replaces the names in the "CNVDetectionResu
```

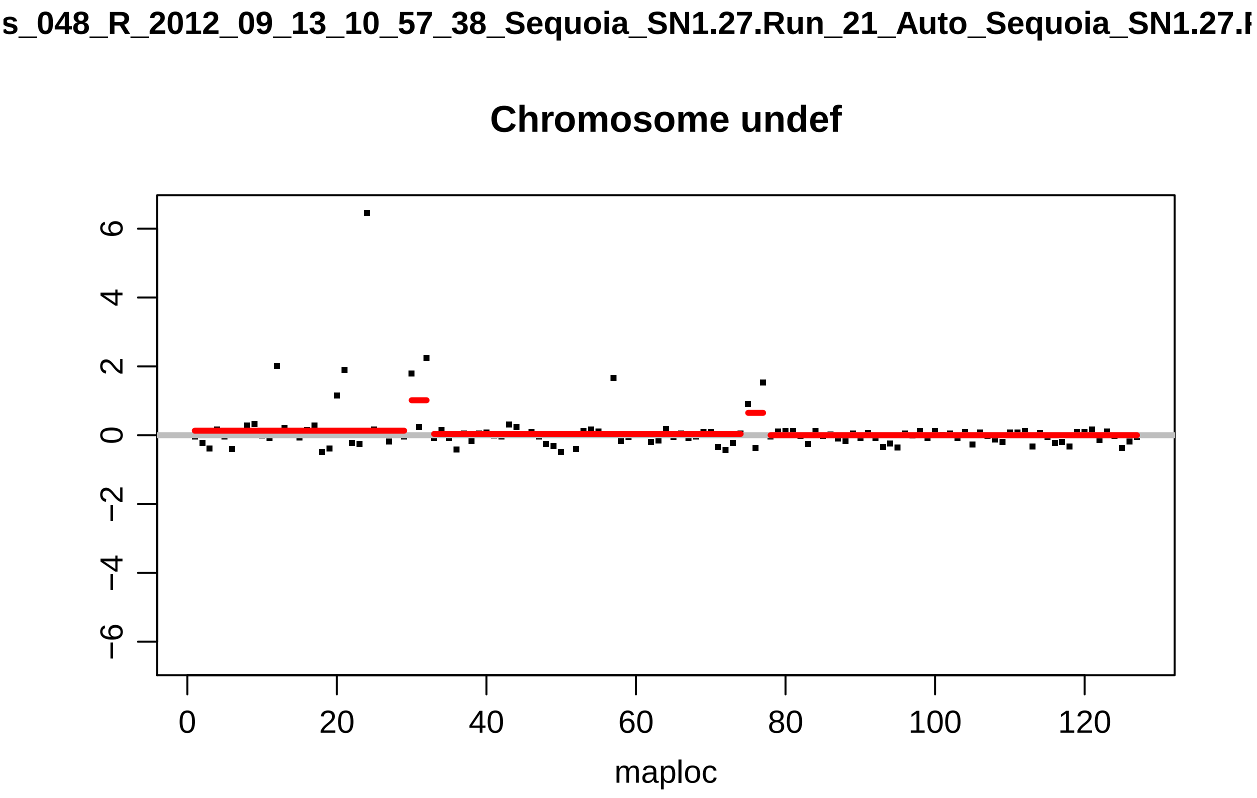


```
## Segplot might not work because of special characters in the sample names. Use only A-Z,a-z and 0-9!
##  There is a hidden function cn.mops:::.replaceNames that replaces the names in the "CNVDetectionResu
```

s_001_R_2012_09_13_15_10_42_Sequoia_SN1.28.Run_22_Auto_Sequoia_SN1.28.I

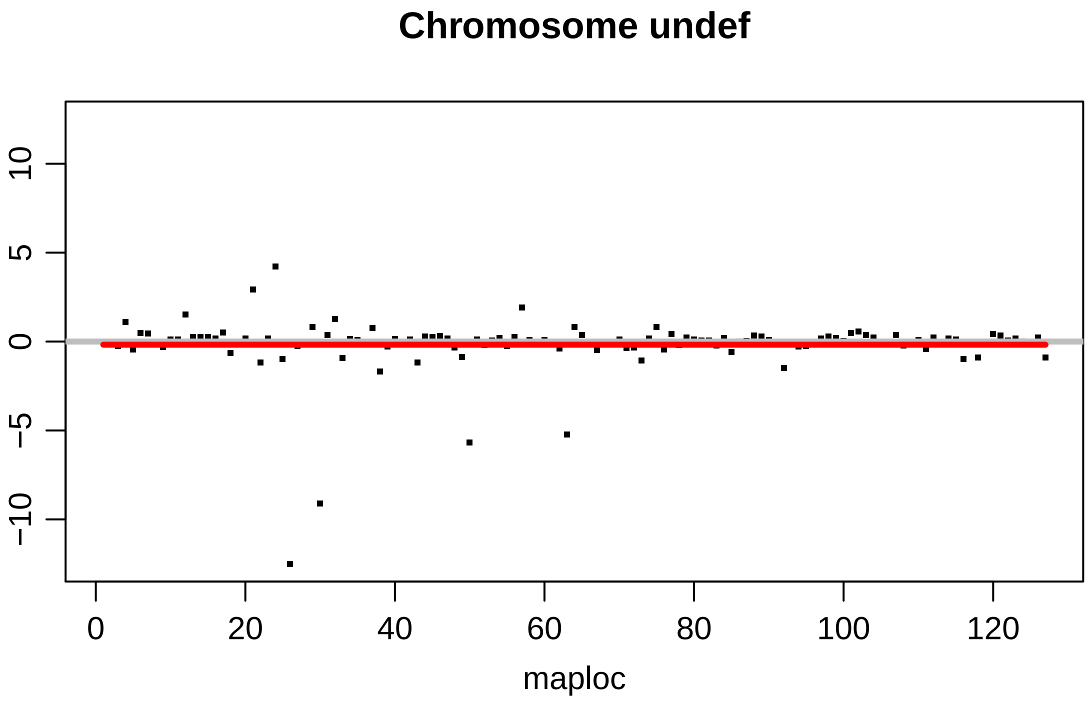


```
## Segplot might not work because of special characters in the sample names. Use only A-Z,a-z and 0-9!
##  There is a hidden function cn.mops:::.replaceNames that replaces the names in the "CNVDetectionResul
```

s_014_R_2012_09_13_15_10_42_Sequoia_SN1.28.Run_22_Auto_Sequoia_SN1.28.I

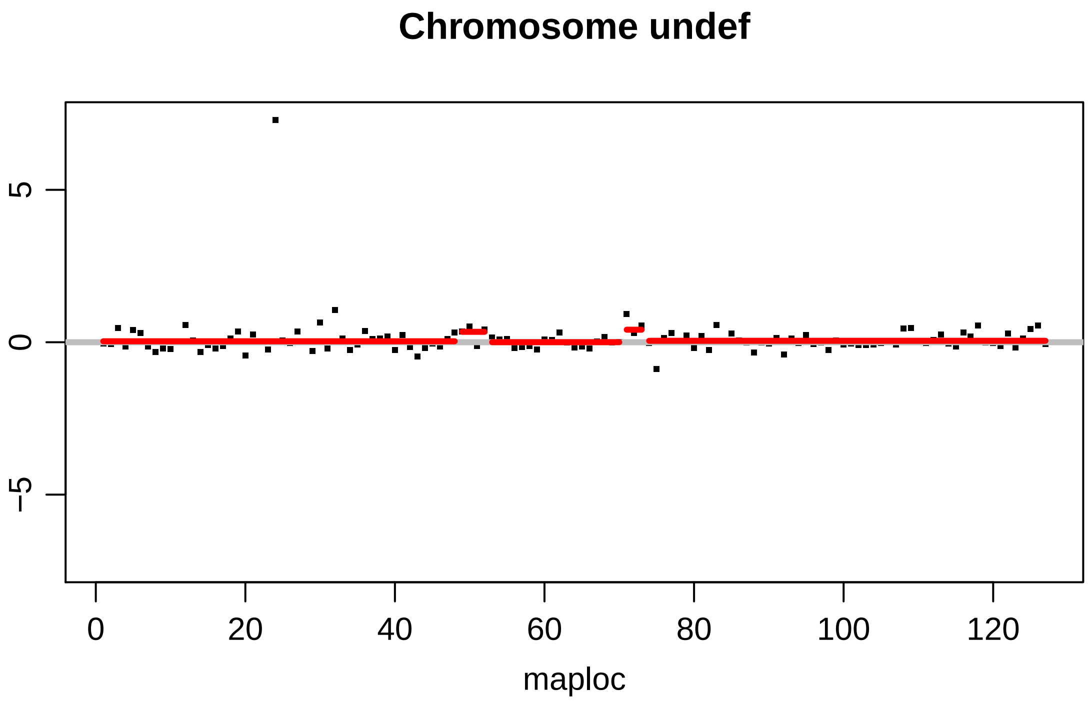

```
## Segplot might not work because of special characters in the sample names. Use only A-Z,a-z and 0-9!
##  There is a hidden function cn.mops:::.replaceNames that replaces the names in the "CNVDetectionResul
```

s_015_R_2012_09_13_15_10_42_Sequoia_SN1.28.Run_22_Auto_Sequoia_SN1.28.I

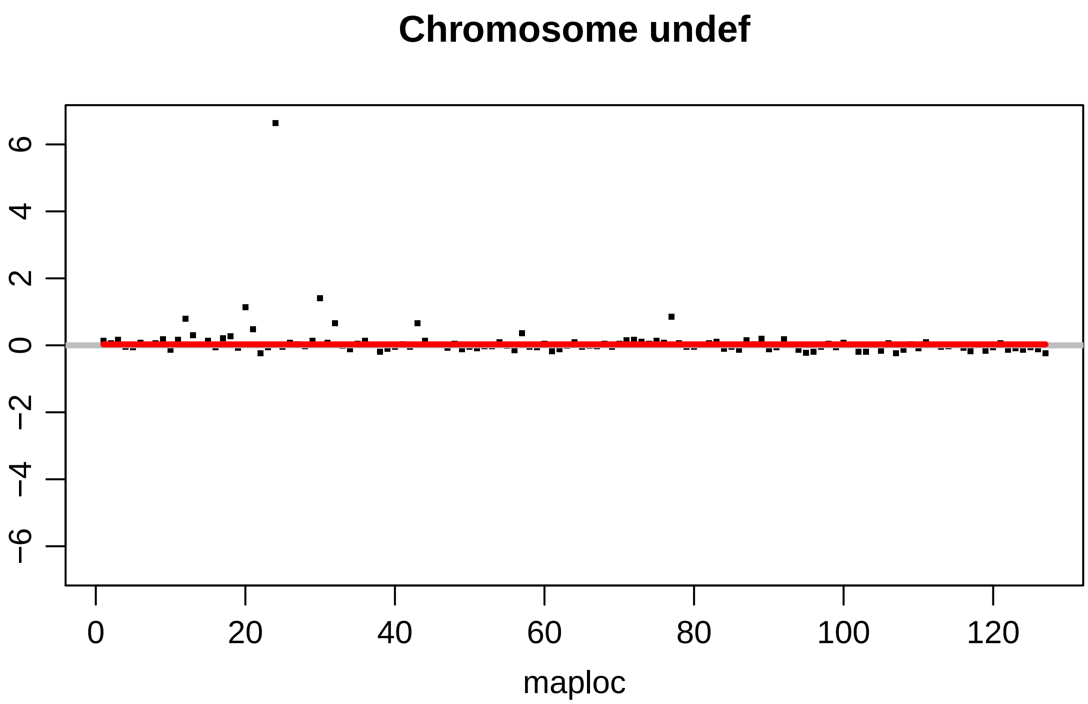


```
## Segplot might not work because of special characters in the sample names. Use only A-Z,a-z and 0-9!
##  There is a hidden function cn.mops:::.replaceNames that replaces the names in the "CNVDetectionResul
```

s_016_R_2012_09_13_15_10_42_Sequoia_SN1.28.Run_22_Auto_Sequoia_SN1.28.I

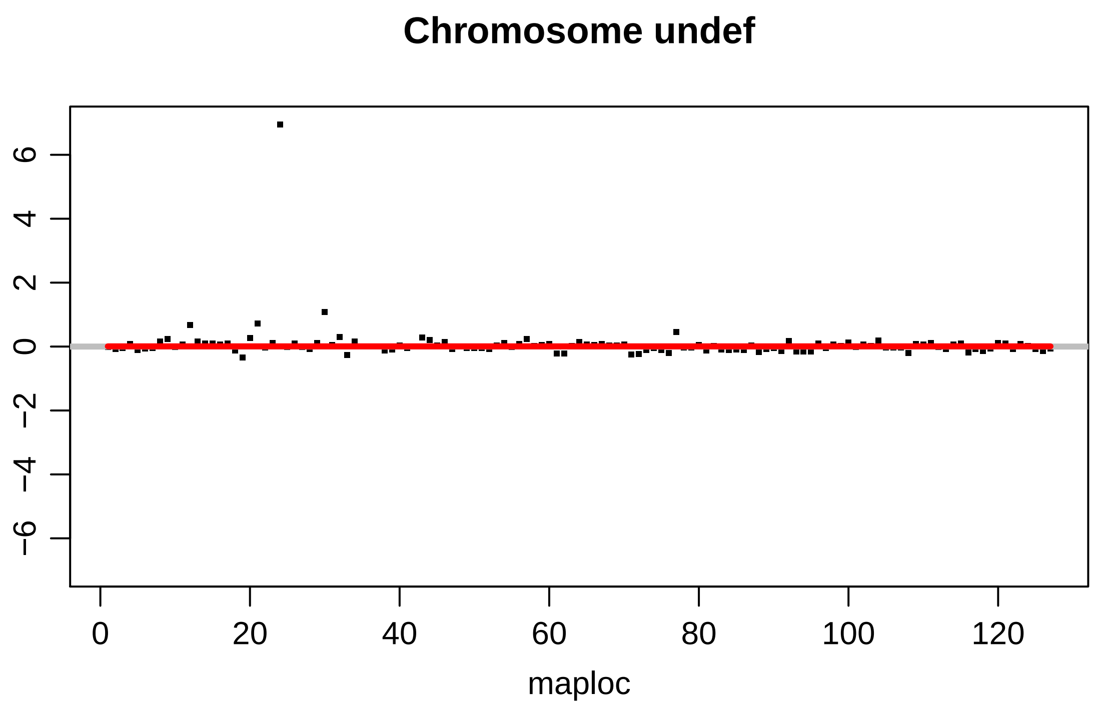


```
## Segplot might not work because of special characters in the sample names. Use only A-Z,a-z and 0-9!
##  There is a hidden function cn.mops:::.replaceNames that replaces the names in the "CNVDetectionResul
```

s_017_R_2012_09_13_15_10_42_Sequoia_SN1.28.Run_22_Auto_Sequoia_SN1.28.I

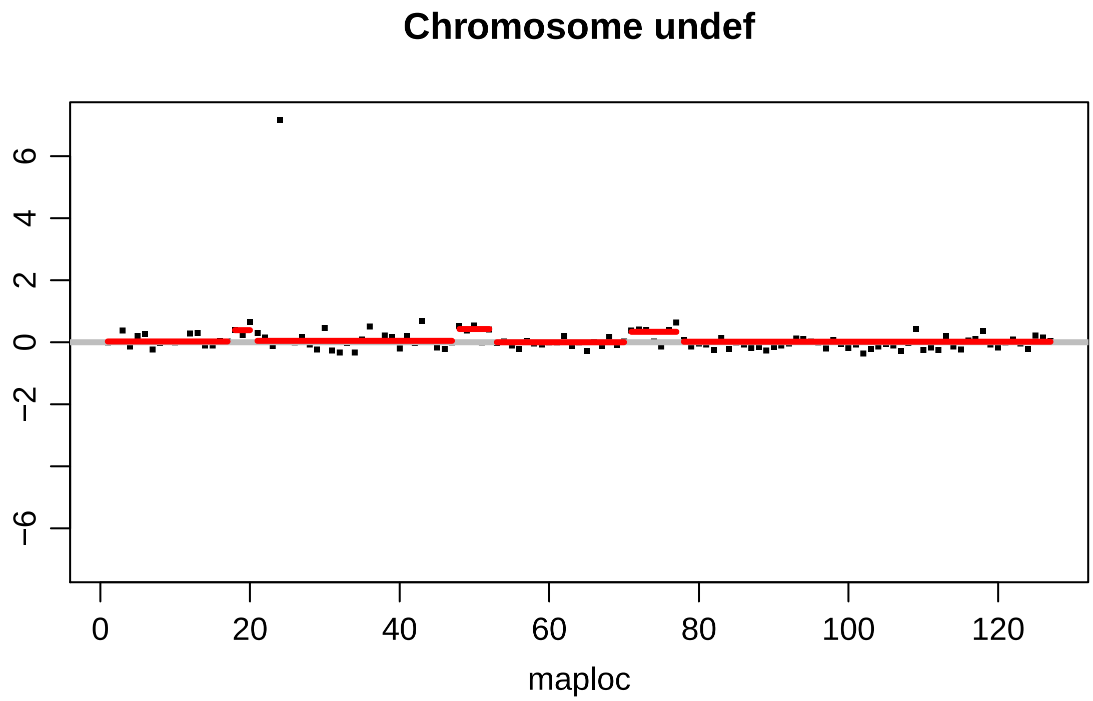

```
## Segplot might not work because of special characters in the sample names. Use only A-Z,a-z and 0-9!
##  There is a hidden function cn.mops:::.replaceNames that replaces the names in the "CNVDetectionResul
```

s_018_R_2012_09_13_15_10_42_Sequoia_SN1.28.Run_22_Auto_Sequoia_SN1.28.I

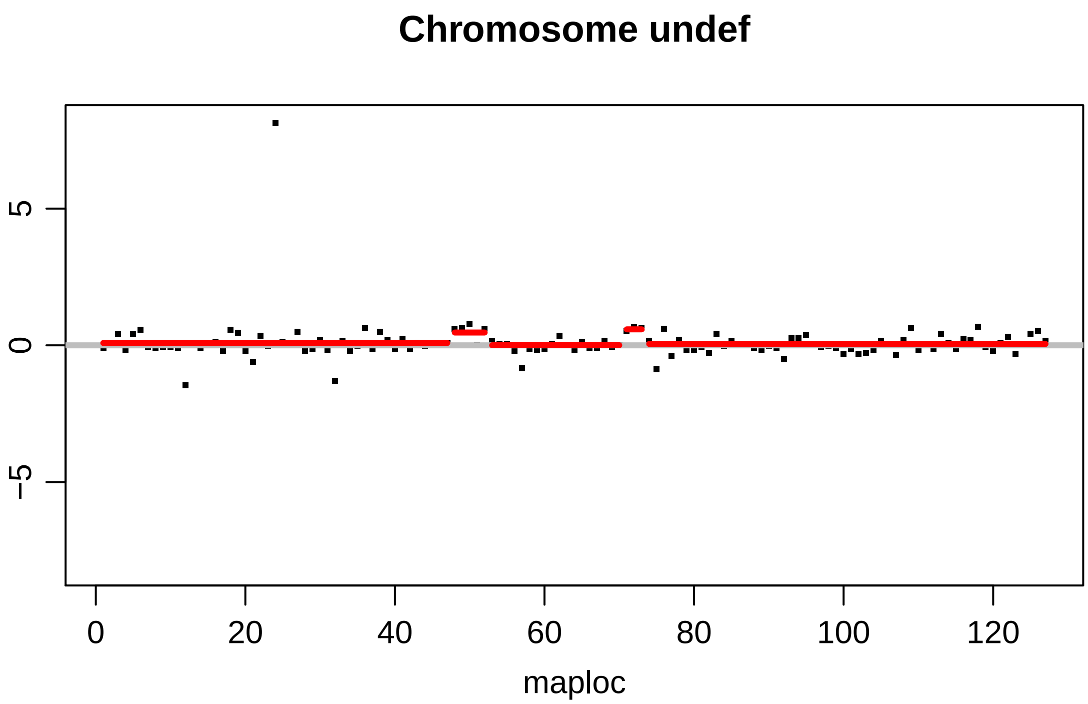


```
## Segplot might not work because of special characters in the sample names. Use only A-Z,a-z and 0-9!
##  There is a hidden function cn.mops:::.replaceNames that replaces the names in the "CNVDetectionResul
```

s_019_R_2012_09_13_15_10_42_Sequoia_SN1.28.Run_22_Auto_Sequoia_SN1.28.I

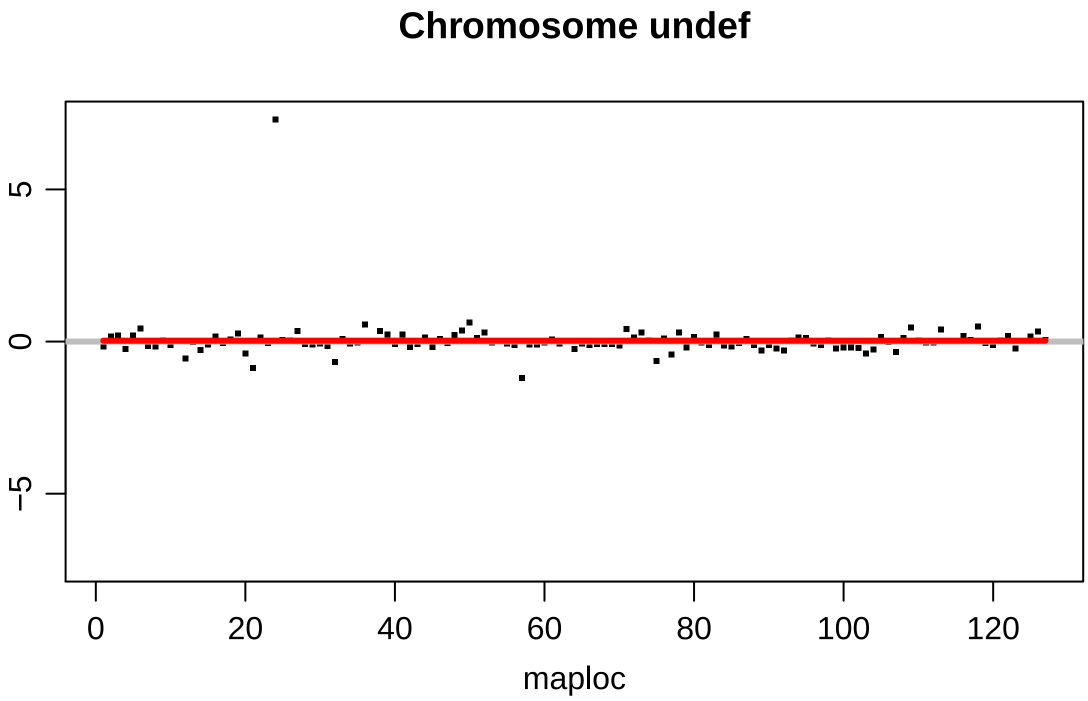


```
## Segplot might not work because of special characters in the sample names. Use only A-Z,a-z and 0-9!
##  There is a hidden function cn.mops:::.replaceNames that replaces the names in the "CNVDetectionResul
```

s_020_R_2012_09_13_15_10_42_Sequoia_SN1.28.Run_22_Auto_Sequoia_SN1.28.I

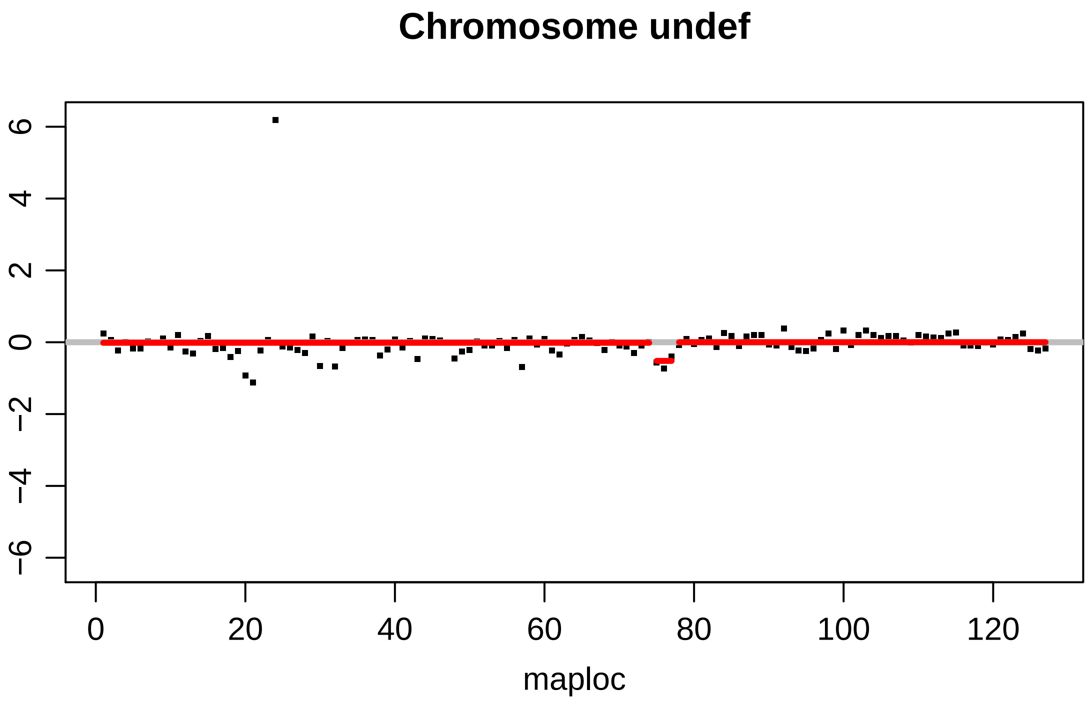

```
## Segplot might not work because of special characters in the sample names. Use only A-Z,a-z and 0-9!
##  There is a hidden function cn.mops:::.replaceNames that replaces the names in the "CNVDetectionResul
```

s_021_R_2012_09_13_15_10_42_Sequoia_SN1.28.Run_22_Auto_Sequoia_SN1.28.I

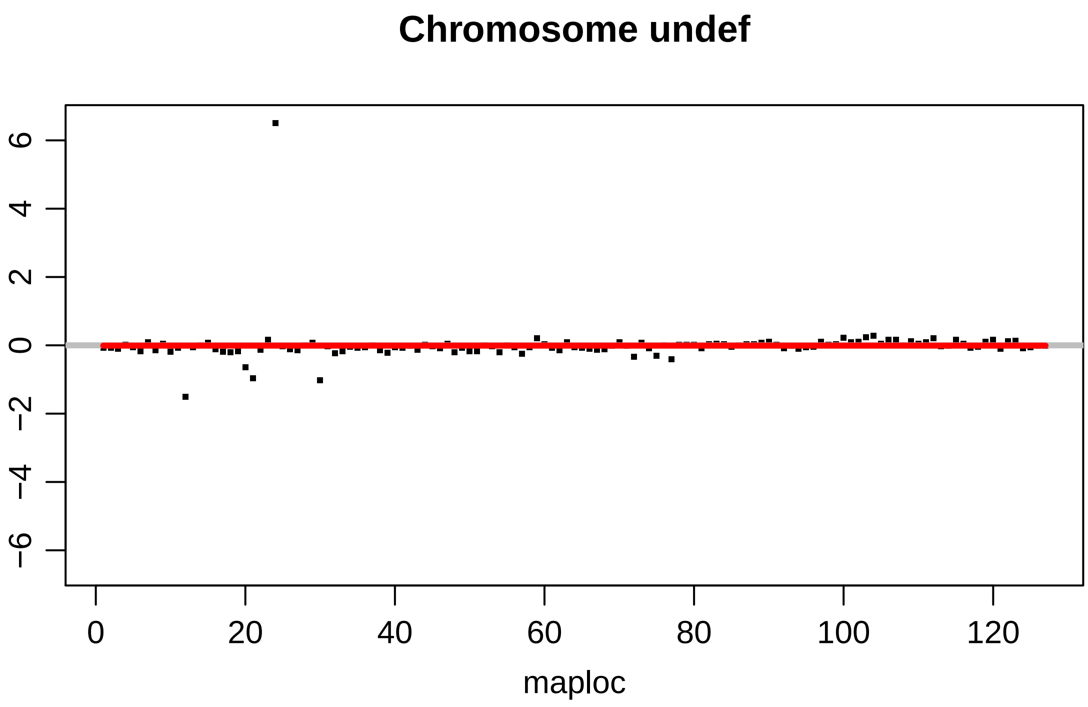


```
## Segplot might not work because of special characters in the sample names. Use only A-Z,a-z and 0-9!
##  There is a hidden function cn.mops:::.replaceNames that replaces the names in the "CNVDetectionResul
```

s_022_R_2012_09_13_15_10_42_Sequoia_SN1.28.Run_22_Auto_Sequoia_SN1.28.I

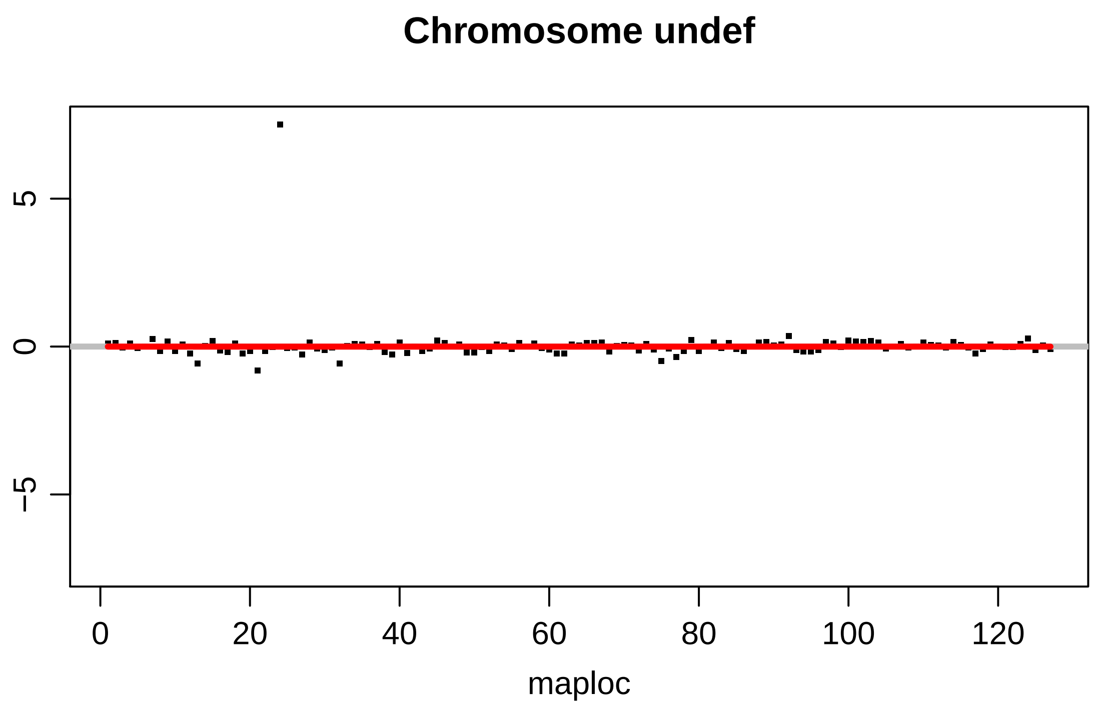


```
## Segplot might not work because of special characters in the sample names. Use only A-Z,a-z and 0-9!
##  There is a hidden function cn.mops:::.replaceNames that replaces the names in the "CNVDetectionResul
```

s_024_R_2012_09_13_15_10_42_Sequoia_SN1.28.Run_22_Auto_Sequoia_SN1.28.I

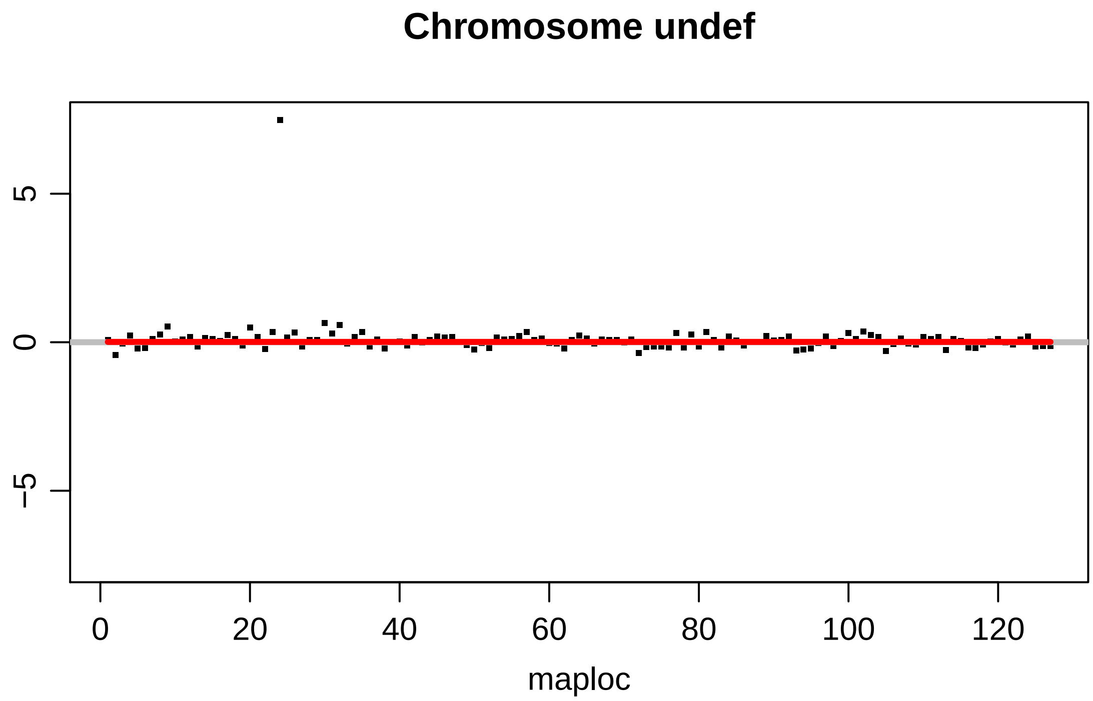

```
## Segplot might not work because of special characters in the sample names. Use only A-Z,a-z and 0-9!
##  There is a hidden function cn.mops:::.replaceNames that replaces the names in the "CNVDetectionResul
```

s_025_R_2012_09_13_15_10_42_Sequoia_SN1.28.Run_22_Auto_Sequoia_SN1.28.I

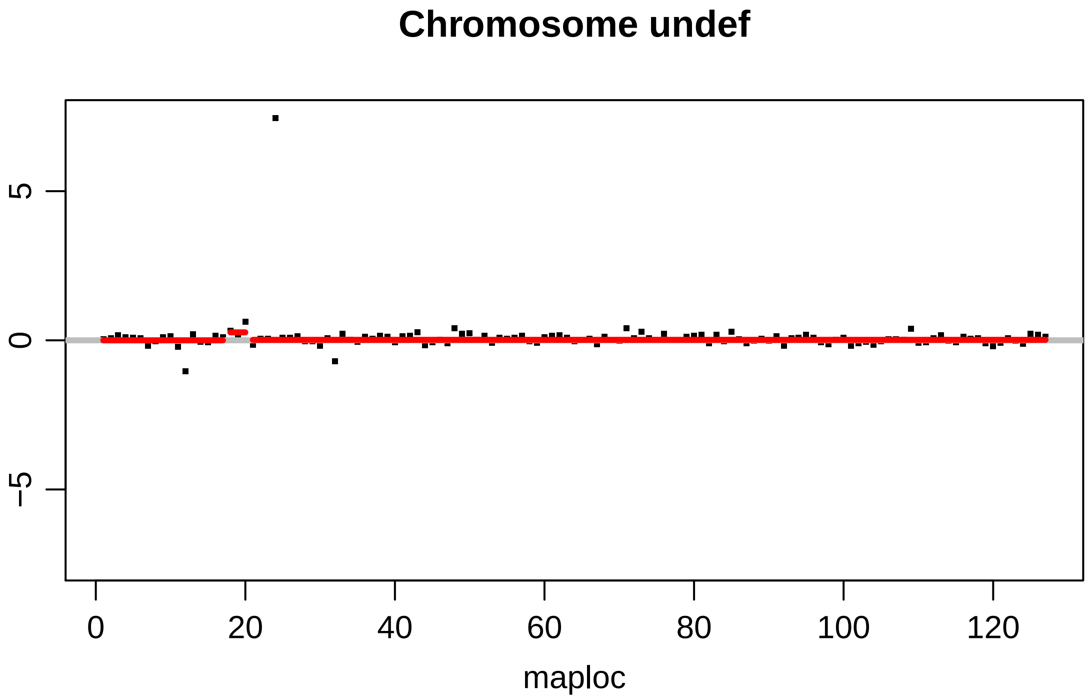


```
## Segplot might not work because of special characters in the sample names. Use only A-Z,a-z and 0-9!
##  There is a hidden function cn.mops:::.replaceNames that replaces the names in the "CNVDetectionResul
```

s_026_R_2012_09_13_15_10_42_Sequoia_SN1.28.Run_22_Auto_Sequoia_SN1.28.I

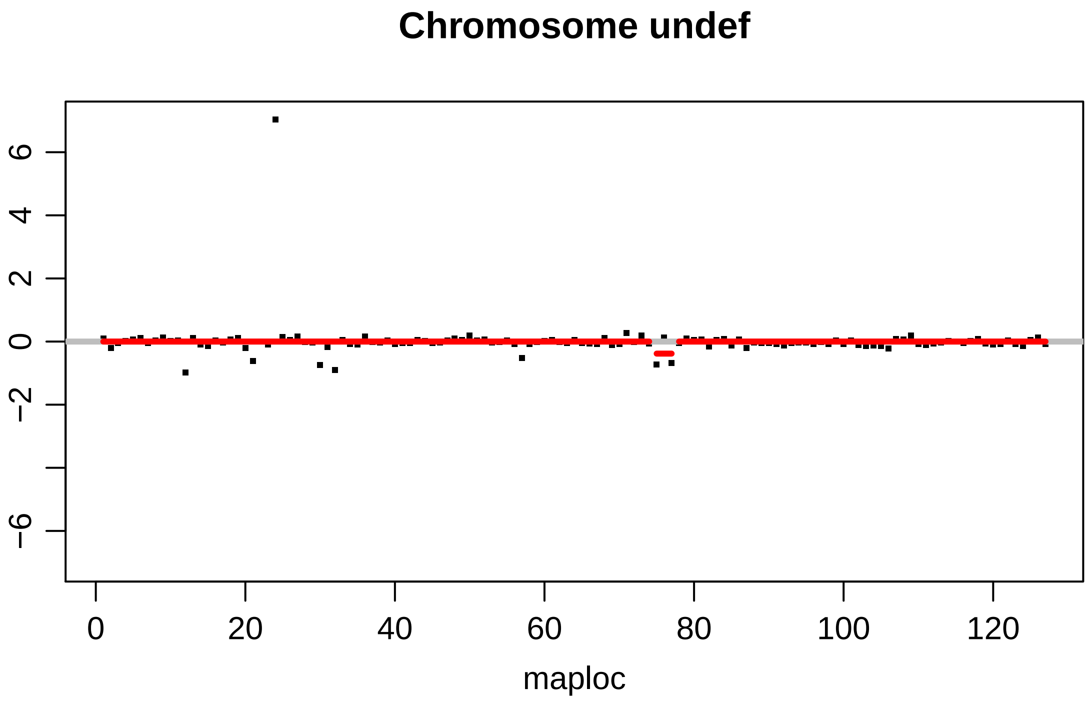


```
## Segplot might not work because of special characters in the sample names. Use only A-Z,a-z and 0-9!
##  There is a hidden function cn.mops:::.replaceNames that replaces the names in the "CNVDetectionResul
```

s_027_R_2012_09_13_15_10_42_Sequoia_SN1.28.Run_22_Auto_Sequoia_SN1.28.I

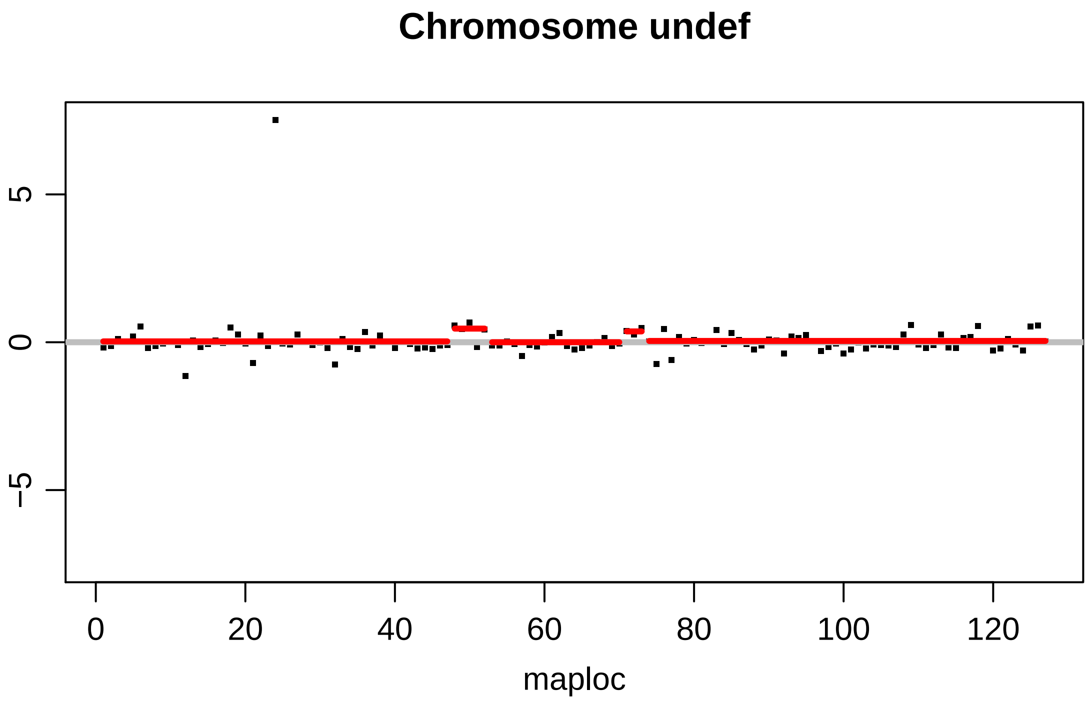

```
## Segplot might not work because of special characters in the sample names. Use only A-Z,a-z and 0-9!
##  There is a hidden function cn.mops:::.replaceNames that replaces the names in the "CNVDetectionResul
```

s_028_R_2012_09_13_15_10_42_Sequoia_SN1.28.Run_22_Auto_Sequoia_SN1.28.I

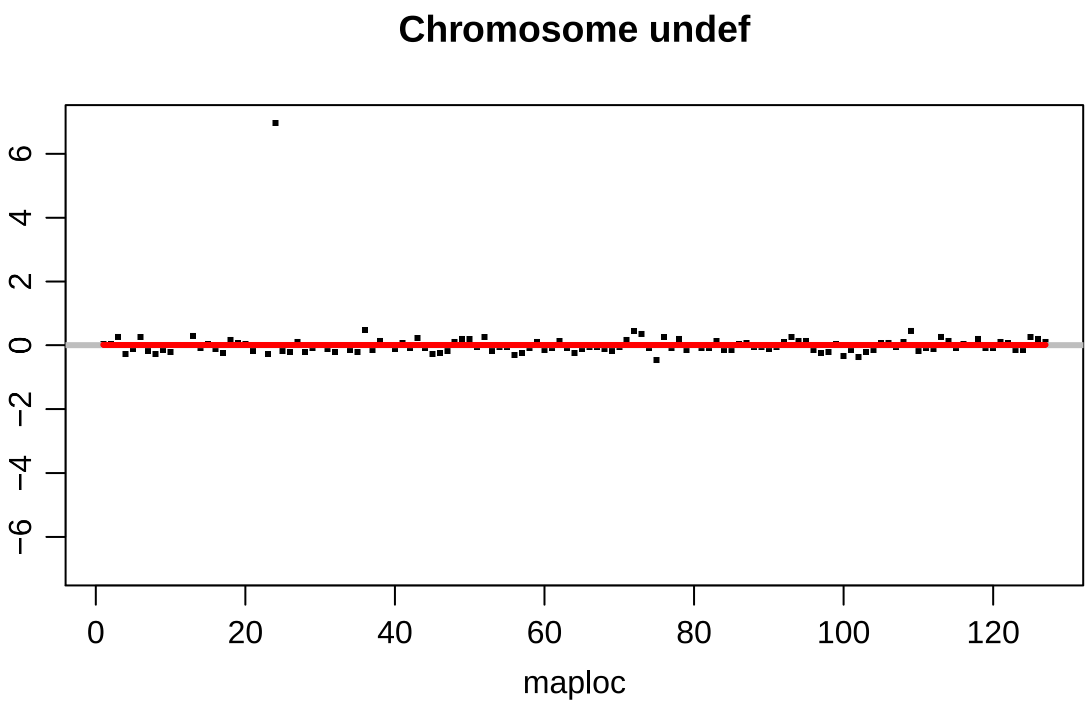


```
## Segplot might not work because of special characters in the sample names. Use only A-Z,a-z and 0-9!
##  There is a hidden function cn.mops:::.replaceNames that replaces the names in the "CNVDetectionResul
```

s_029_R_2012_09_13_15_10_42_Sequoia_SN1.28.Run_22_Auto_Sequoia_SN1.28.I

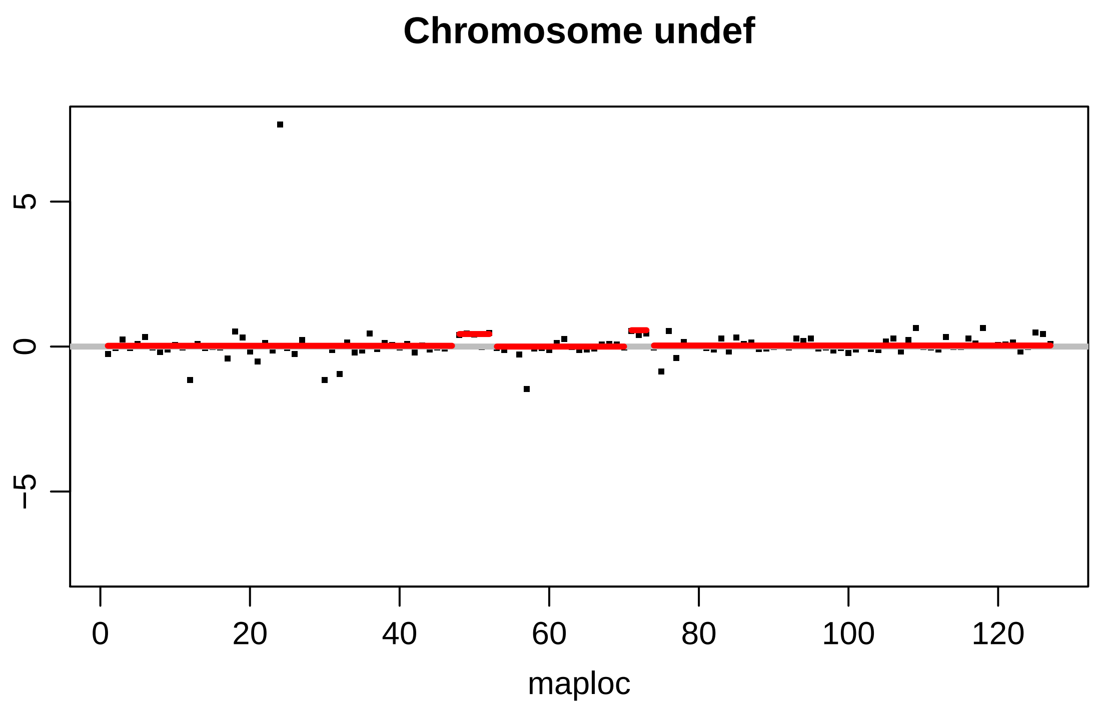


```
## Segplot might not work because of special characters in the sample names. Use only A-Z,a-z and 0-9!
##  There is a hidden function cn.mops:::.replaceNames that replaces the names in the "CNVDetectionResul
```

s_030_R_2012_09_13_15_10_42_Sequoia_SN1.28.Run_22_Auto_Sequoia_SN1.28.I

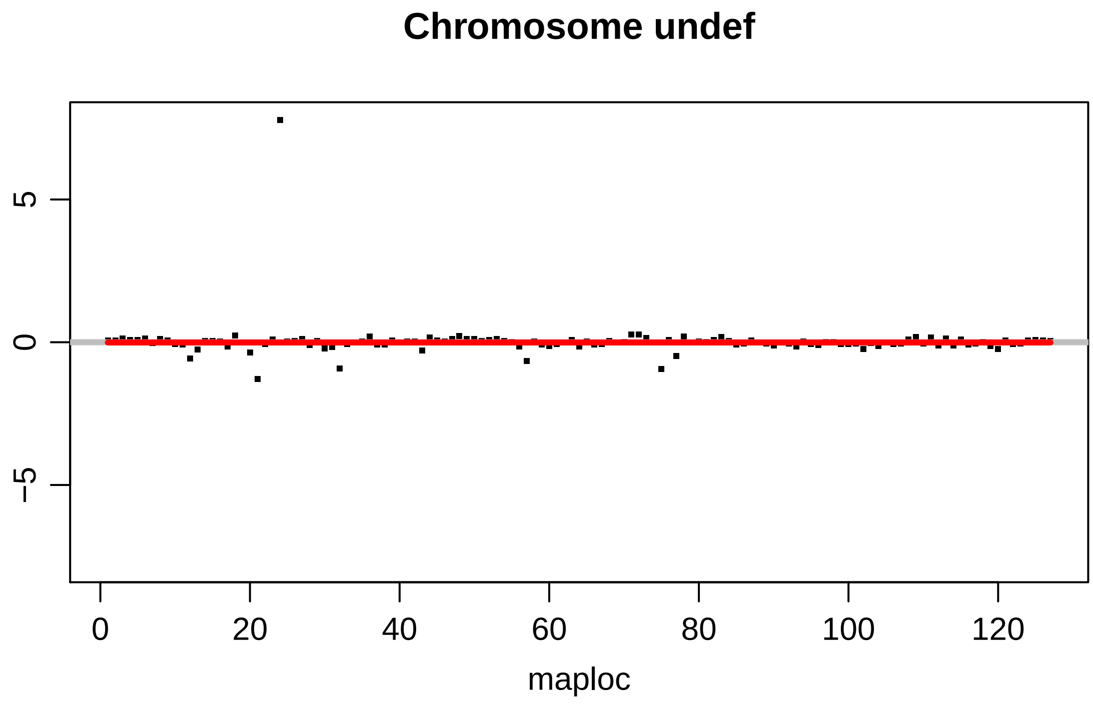

```
## Segplot might not work because of special characters in the sample names. Use only A-Z,a-z and 0-9!
##  There is a hidden function cn.mops:::.replaceNames that replaces the names in the "CNVDetectionResul
```

s_031_R_2012_09_13_15_10_42_Sequoia_SN1.28.Run_22_Auto_Sequoia_SN1.28.I

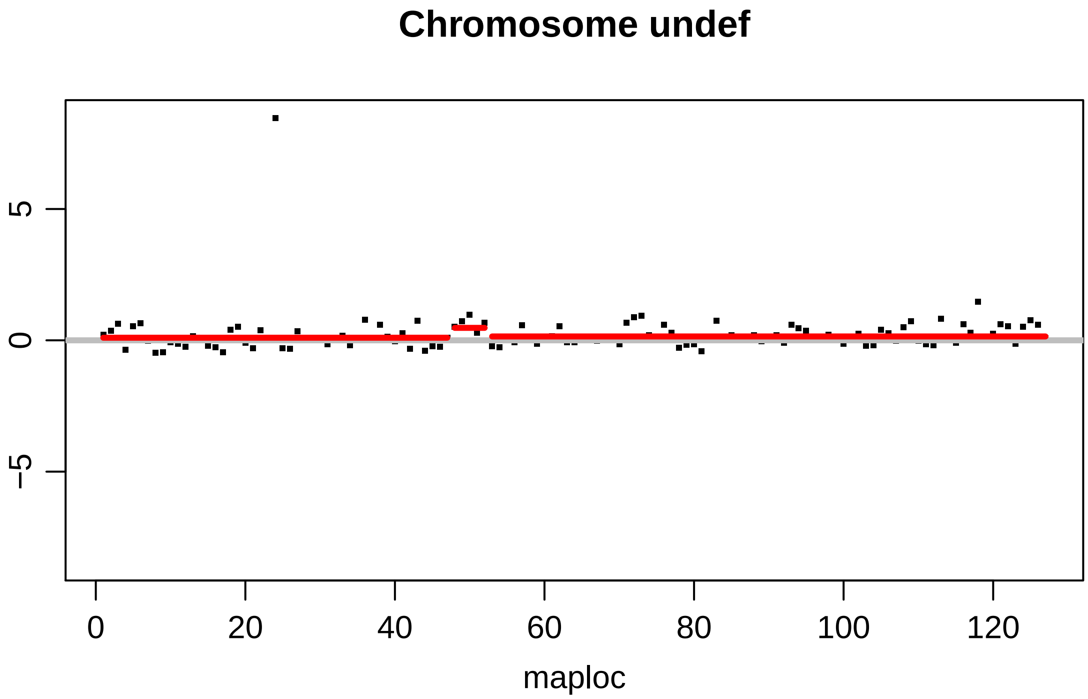


```
## Segplot might not work because of special characters in the sample names. Use only A-Z,a-z and 0-9!
##  There is a hidden function cn.mops:::.replaceNames that replaces the names in the "CNVDetectionResul
```

s_032_R_2012_09_13_15_10_42_Sequoia_SN1.28.Run_22_Auto_Sequoia_SN1.28.I

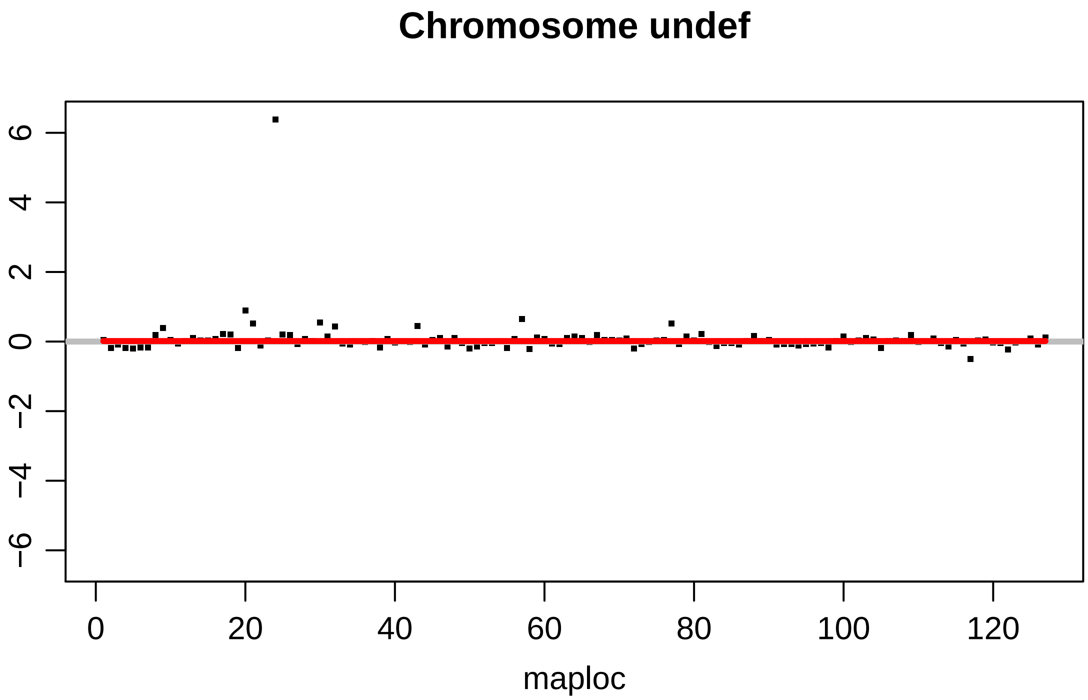


```
## Segplot might not work because of special characters in the sample names. Use only A-Z,a-z and 0-9!
##  There is a hidden function cn.mops:::.replaceNames that replaces the names in the "CNVDetectionResul
```

s_033_R_2012_09_13_15_10_42_Sequoia_SN1.28.Run_22_Auto_Sequoia_SN1.28.I

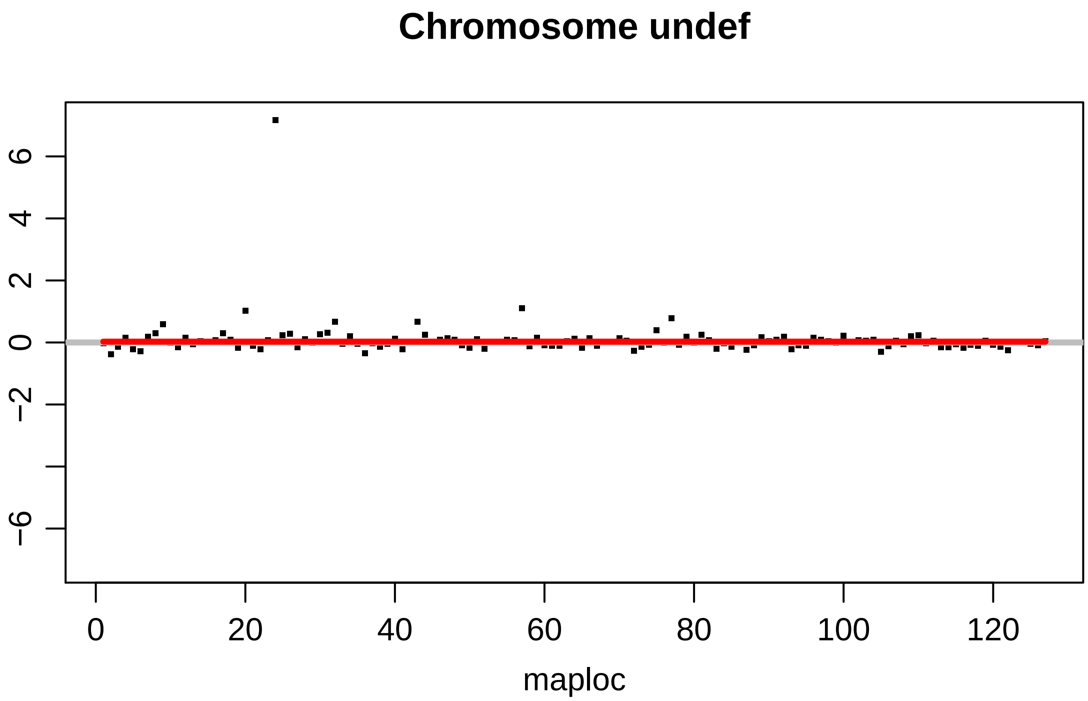

```
## Segplot might not work because of special characters in the sample names. Use only A-Z,a-z and 0-9!
##  There is a hidden function cn.mops:::.replaceNames that replaces the names in the "CNVDetectionResul
```

s_034_R_2012_09_13_15_10_42_Sequoia_SN1.28.Run_22_Auto_Sequoia_SN1.28.I

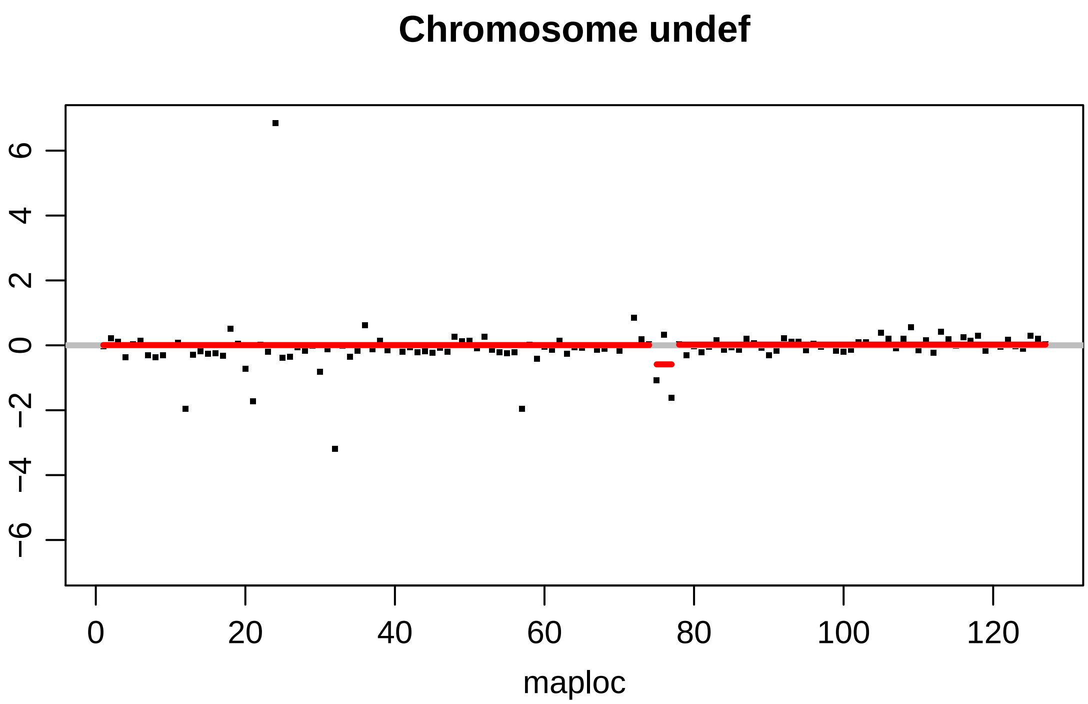


```
## Segplot might not work because of special characters in the sample names. Use only A-Z,a-z and 0-9!
##  There is a hidden function cn.mops:::.replaceNames that replaces the names in the "CNVDetectionResul
```

s_035_R_2012_09_13_15_10_42_Sequoia_SN1.28.Run_22_Auto_Sequoia_SN1.28.I

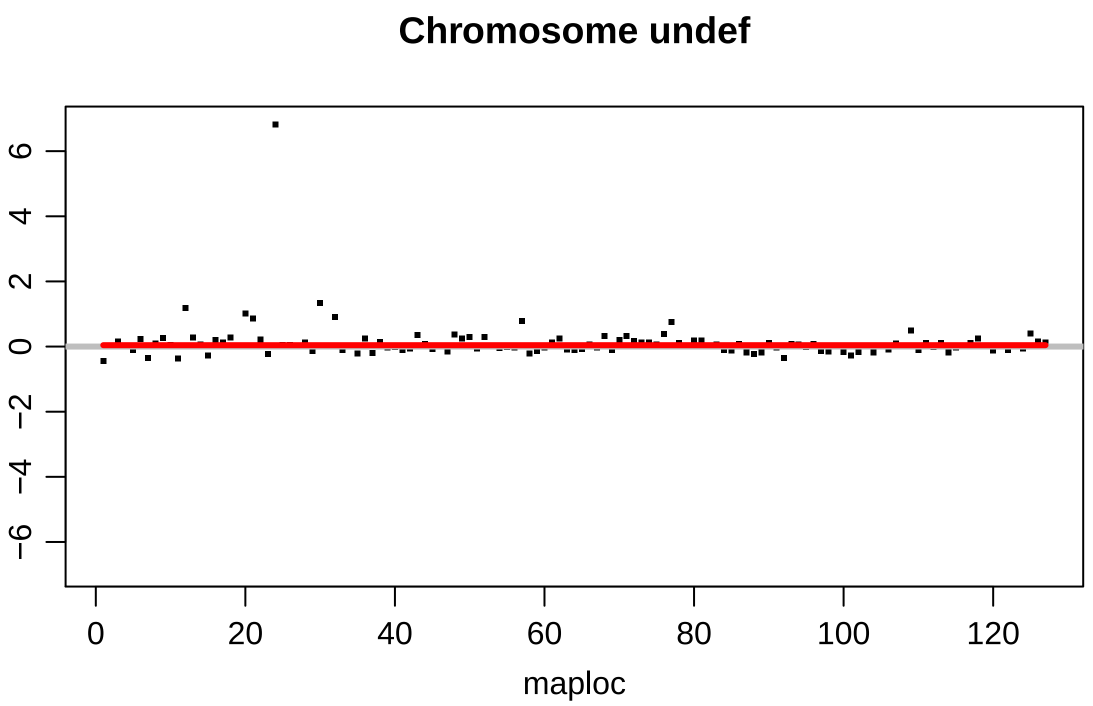


```
## Segplot might not work because of special characters in the sample names. Use only A-Z,a-z and 0-9!
##  There is a hidden function cn.mops:::.replaceNames that replaces the names in the "CNVDetectionResul
```

s_036_R_2012_09_13_15_10_42_Sequoia_SN1.28.Run_22_Auto_Sequoia_SN1.28.I

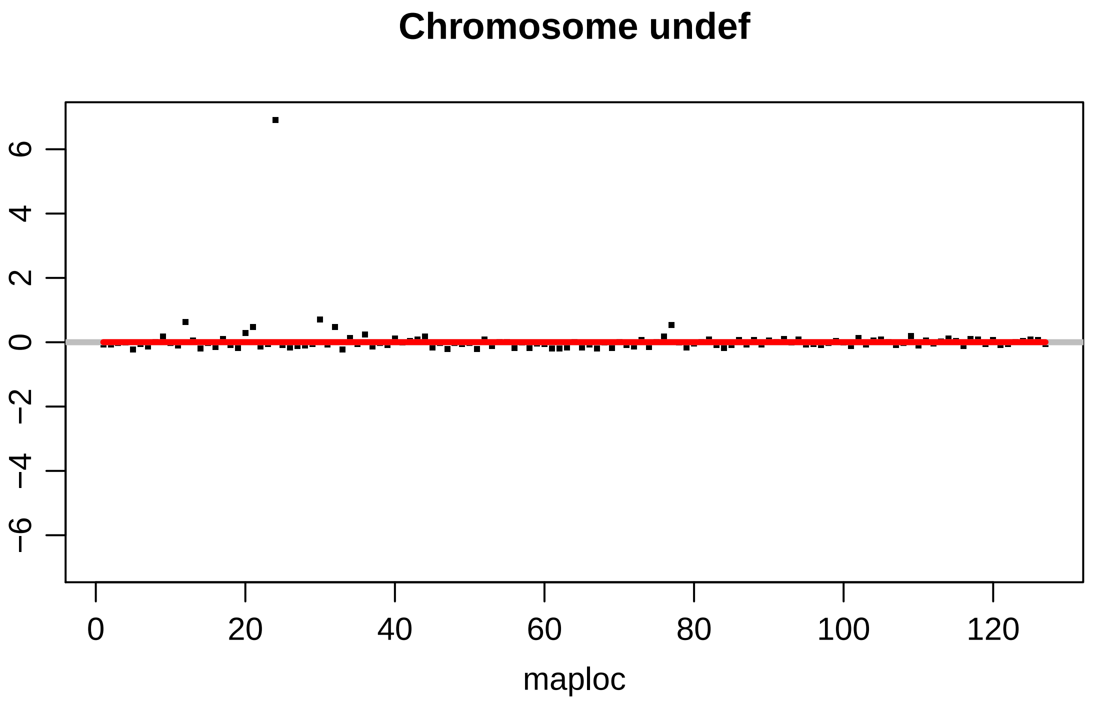

```
## Segplot might not work because of special characters in the sample names. Use only A-Z,a-z and 0-9!
##  There is a hidden function cn.mops:::.replaceNames that replaces the names in the "CNVDetectionResul
```

s_038_R_2012_09_13_15_10_42_Sequoia_SN1.28.Run_22_Auto_Sequoia_SN1.28.I

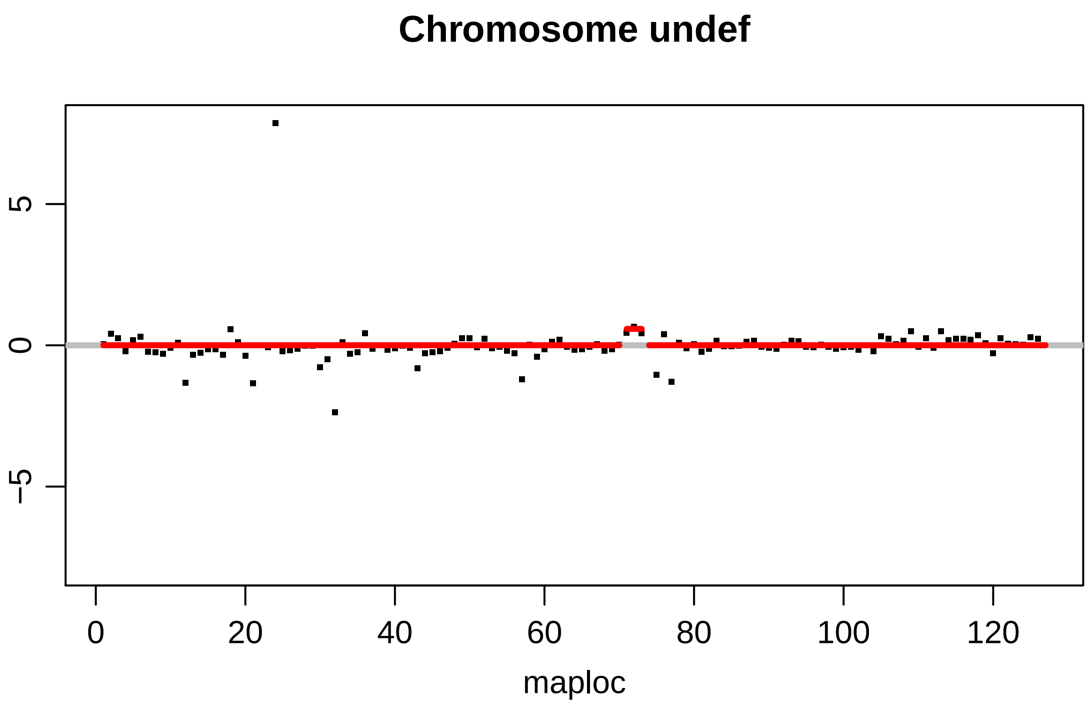


```
## Segplot might not work because of special characters in the sample names. Use only A-Z,a-z and 0-9!
##  There is a hidden function cn.mops:::.replaceNames that replaces the names in the "CNVDetectionResul
```

s_039_R_2012_09_13_15_10_42_Sequoia_SN1.28.Run_22_Auto_Sequoia_SN1.28.I

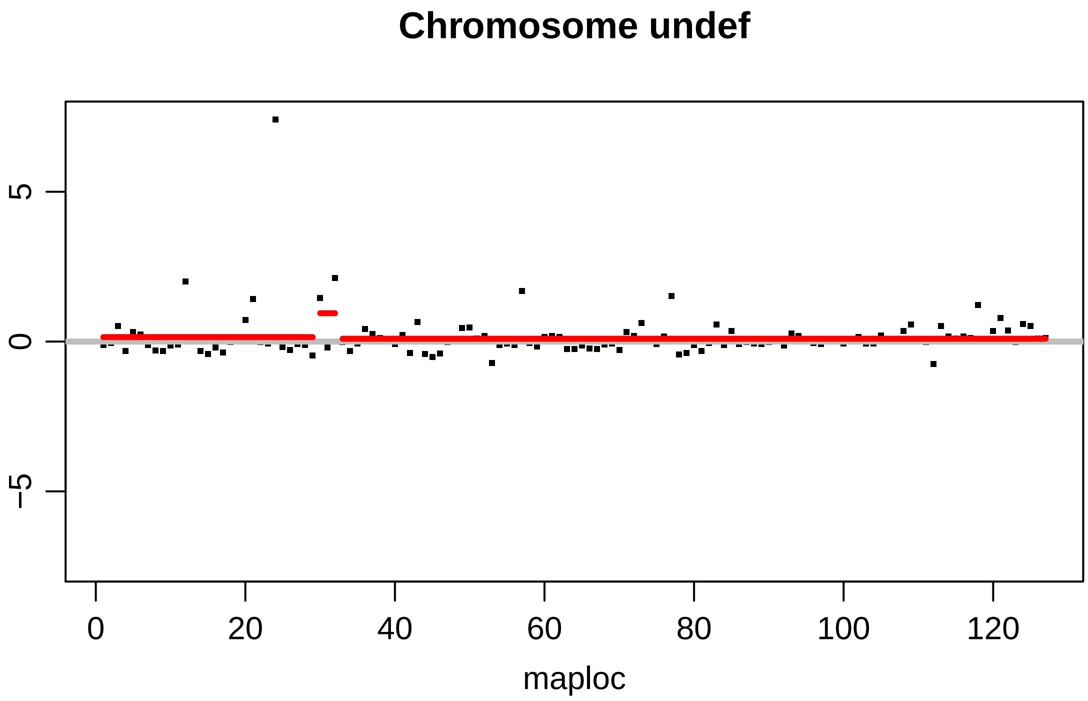


```
## Segplot might not work because of special characters in the sample names. Use only A-Z,a-z and 0-9!
##  There is a hidden function cn.mops:::.replaceNames that replaces the names in the "CNVDetectionResul
```

s_040_R_2012_09_13_15_10_42_Sequoia_SN1.28.Run_22_Auto_Sequoia_SN1.28.I

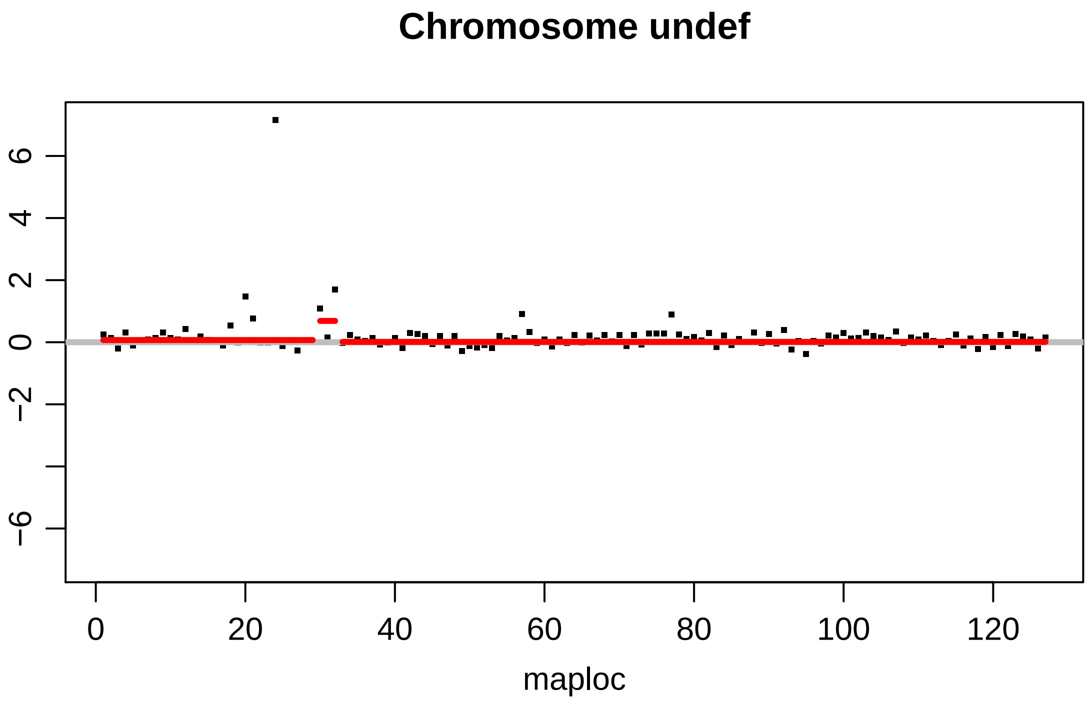

```
## Segplot might not work because of special characters in the sample names. Use only A-Z,a-z and 0-9!
##  There is a hidden function cn.mops:::.replaceNames that replaces the names in the "CNVDetectionResul
```

s_041_R_2012_09_13_15_10_42_Sequoia_SN1.28.Run_22_Auto_Sequoia_SN1.28.I

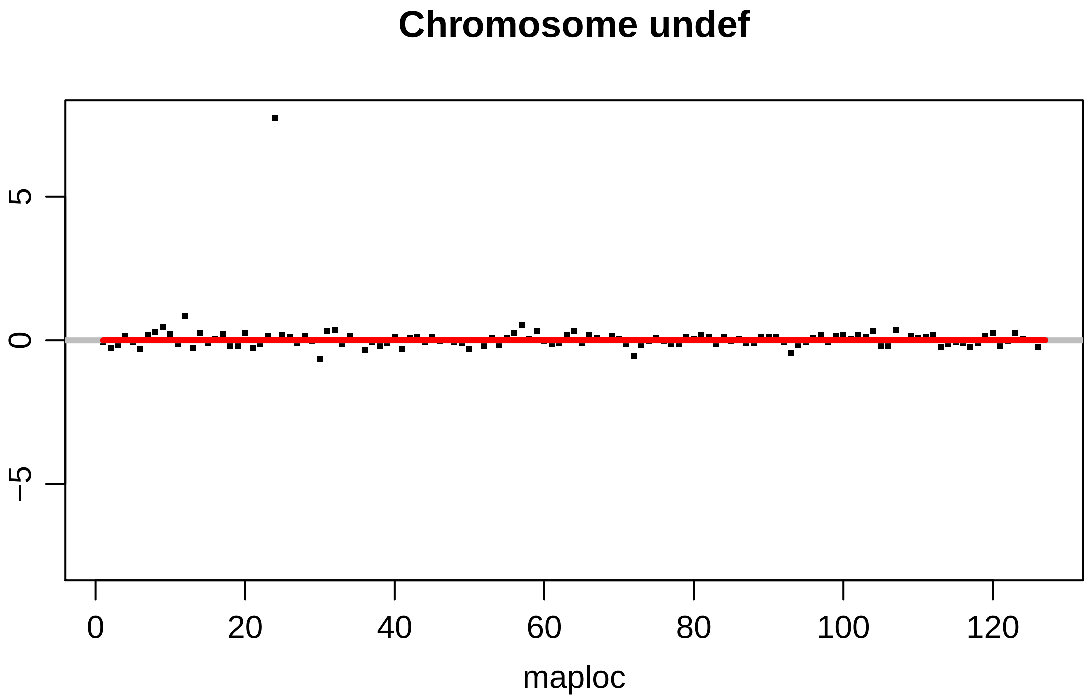


```
## Segplot might not work because of special characters in the sample names. Use only A-Z,a-z and 0-9!
##  There is a hidden function cn.mops:::.replaceNames that replaces the names in the "CNVDetectionResul
```

s_042_R_2012_09_13_15_10_42_Sequoia_SN1.28.Run_22_Auto_Sequoia_SN1.28.I

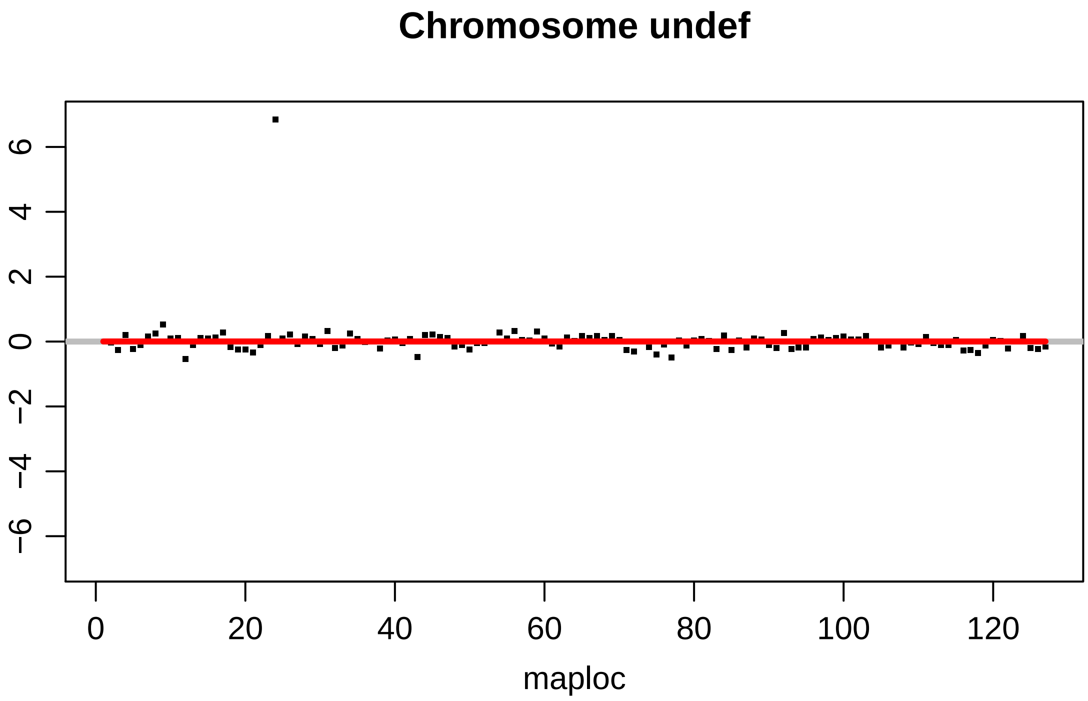


```
## Segplot might not work because of special characters in the sample names. Use only A-Z,a-z and 0-9!
##  There is a hidden function cn.mops:::.replaceNames that replaces the names in the "CNVDetectionResul
```

s_043_R_2012_09_13_15_10_42_Sequoia_SN1.28.Run_22_Auto_Sequoia_SN1.28.I

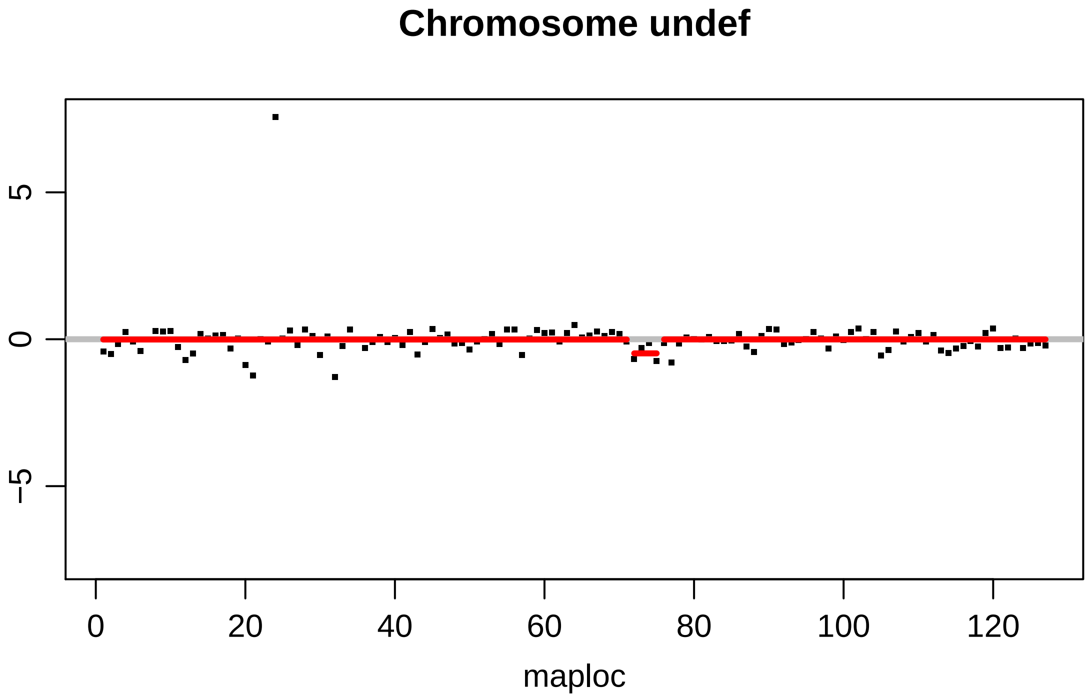

```
## Segplot might not work because of special characters in the sample names. Use only A-Z,a-z and 0-9!
##  There is a hidden function cn.mops:::.replaceNames that replaces the names in the "CNVDetectionResul
```

s_044_R_2012_09_13_15_10_42_Sequoia_SN1.28.Run_22_Auto_Sequoia_SN1.28.I

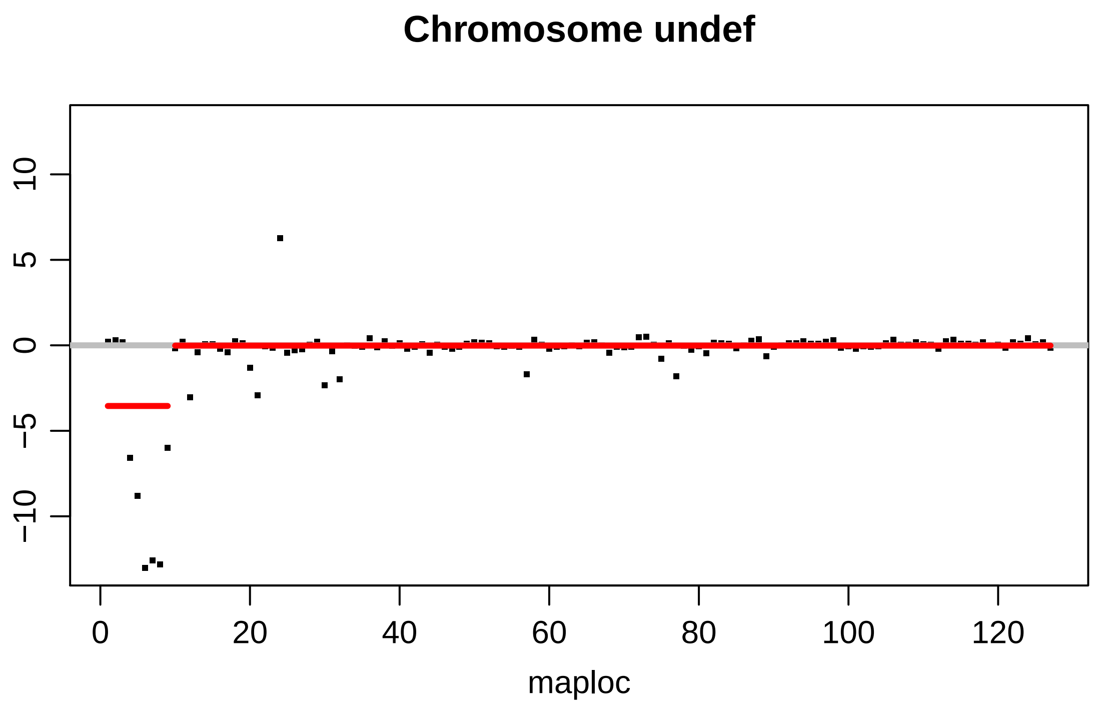


```
## Segplot might not work because of special characters in the sample names. Use only A-Z,a-z and 0-9!
##  There is a hidden function cn.mops:::.replaceNames that replaces the names in the "CNVDetectionResul
```

s_045_R_2012_09_13_15_10_42_Sequoia_SN1.28.Run_22_Auto_Sequoia_SN1.28.I

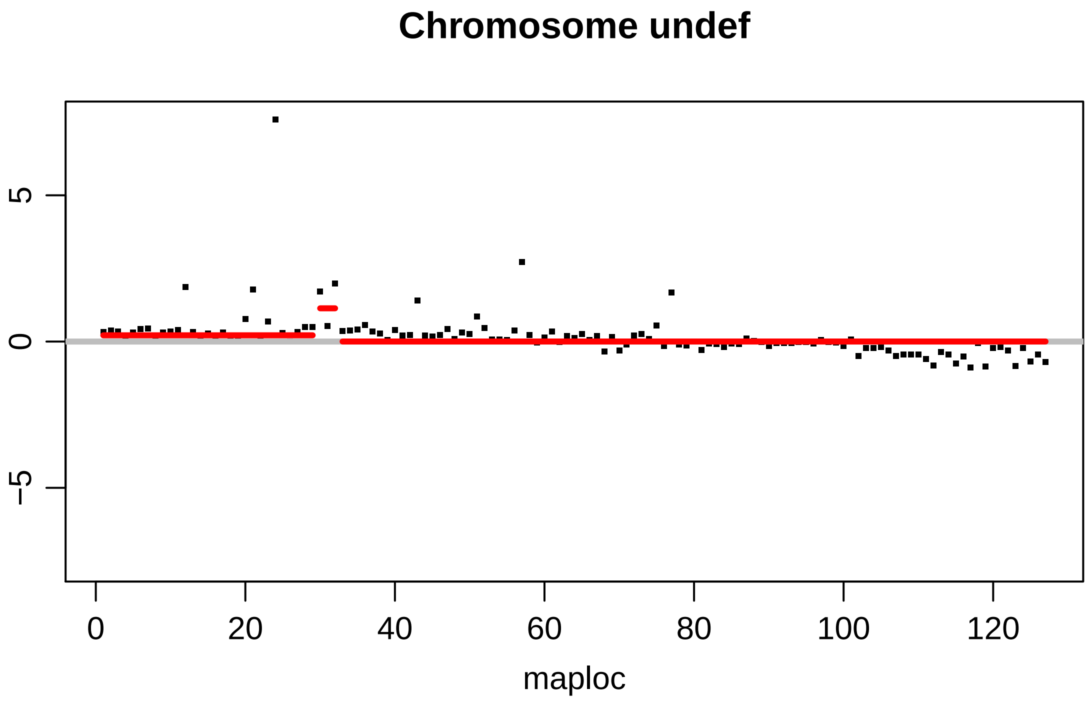


```
## Segplot might not work because of special characters in the sample names. Use only A-Z,a-z and 0-9!
##  There is a hidden function cn.mops:::.replaceNames that replaces the names in the "CNVDetectionResul
```

s_046_R_2012_09_13_15_10_42_Sequoia_SN1.28.Run_22_Auto_Sequoia_SN1.28.I

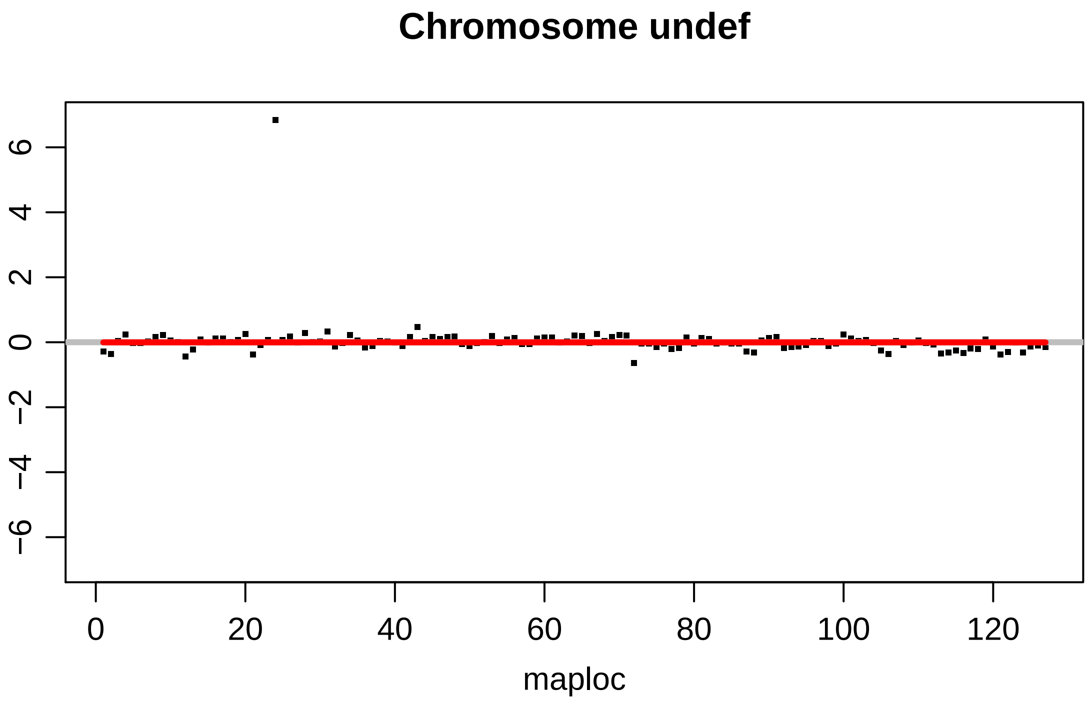

```
## Segplot might not work because of special characters in the sample names. Use only A-Z,a-z and 0-9!
##  There is a hidden function cn.mops:::.replaceNames that replaces the names in the "CNVDetectionResul
```

s_047_R_2012_09_13_15_10_42_Sequoia_SN1.28.Run_22_Auto_Sequoia_SN1.28.I

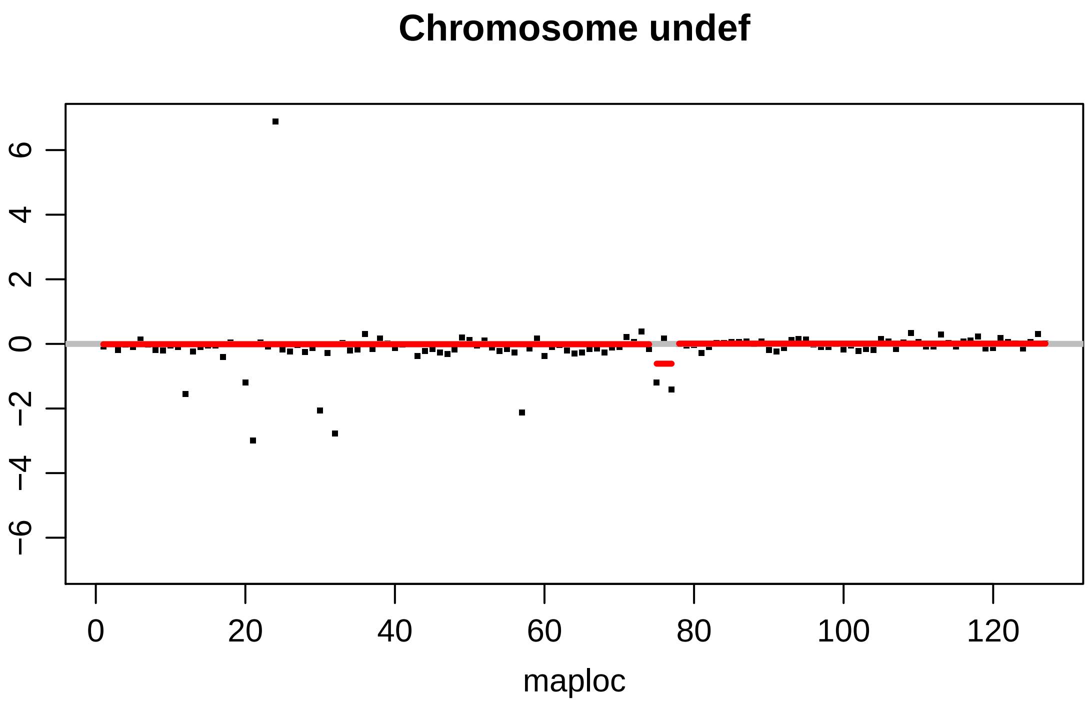


```
##
## CNV regions:
## GRanges object with 4 ranges and 34 metadata columns:
##       seqnames    ranges strand |
##          <Rle> <IRanges>  <Rle> |
##   [1]    undef  [ 1,  9]      * |
##   [2]    undef  [30, 32]      * |
##   [3]    undef  [71, 73]      * |
##   [4]    undef  [75, 77]      * |
##       Case_IonXpress_001_R_2012_09_13_15_10_42_Sequoia_SN1.28.Run_22_Auto_Sequoia_SN1.28.Run_22_55.sa
##                                                                                                <facto
##   [1]                                                                                              CN
##   [2]                                                                                              CN
##   [3]                                                                                              CN
##   [4]                                                                                              CN
##       Case_IonXpress_014_R_2012_09_13_15_10_42_Sequoia_SN1.28.Run_22_Auto_Sequoia_SN1.28.Run_22_55.sa
##                                                                                                <facto
##   [1]                                                                                              CN
##   [2]                                                                                              CN
##   [3]                                                                                              CN
##   [4]                                                                                              CN
##       Case_IonXpress_015_R_2012_09_13_15_10_42_Sequoia_SN1.28.Run_22_Auto_Sequoia_SN1.28.Run_22_55.sa
##                                                                                                <facto
##   [1]                                                                                              CN
##   [2]                                                                                              CN
```

```
##   [3]                                                                                 Cl
##   [4]                                                                                 Cl
##       Case_IonXpress_016_R_2012_09_13_15_10_42_Sequoia_SN1.28.Run_22_Auto_Sequoia_SN1.28.Run_22_55.sa
##                                                                                    <facto
##   [1]                                                                                 Cl
##   [2]                                                                                 Cl
##   [3]                                                                                 Cl
##   [4]                                                                                 Cl
##       Case_IonXpress_017_R_2012_09_13_15_10_42_Sequoia_SN1.28.Run_22_Auto_Sequoia_SN1.28.Run_22_55.sa
##                                                                                    <facto
##   [1]                                                                                 Cl
##   [2]                                                                                 Cl
##   [3]                                                                                 Cl
##   [4]                                                                                 Cl
##       Case_IonXpress_018_R_2012_09_13_15_10_42_Sequoia_SN1.28.Run_22_Auto_Sequoia_SN1.28.Run_22_55.sa
##                                                                                    <facto
##   [1]                                                                                 Cl
##   [2]                                                                                 Cl
##   [3]                                                                                 Cl
##   [4]                                                                                 Cl
##       Case_IonXpress_019_R_2012_09_13_15_10_42_Sequoia_SN1.28.Run_22_Auto_Sequoia_SN1.28.Run_22_55.sa
##                                                                                    <facto
##   [1]                                                                                 Cl
##   [2]                                                                                 Cl
##   [3]                                                                                 Cl
##   [4]                                                                                 Cl
##       Case_IonXpress_020_R_2012_09_13_15_10_42_Sequoia_SN1.28.Run_22_Auto_Sequoia_SN1.28.Run_22_55.sa
##                                                                                    <facto
##   [1]                                                                                 Cl
##   [2]                                                                                 Cl
##   [3]                                                                                 Cl
##   [4]                                                                                 Cl
##       Case_IonXpress_021_R_2012_09_13_15_10_42_Sequoia_SN1.28.Run_22_Auto_Sequoia_SN1.28.Run_22_55.sa
##                                                                                    <facto
##   [1]                                                                                 Cl
##   [2]                                                                                 Cl
##   [3]                                                                                 Cl
##   [4]                                                                                 Cl
##       Case_IonXpress_022_R_2012_09_13_15_10_42_Sequoia_SN1.28.Run_22_Auto_Sequoia_SN1.28.Run_22_55.sa
##                                                                                    <facto
##   [1]                                                                                 Cl
##   [2]                                                                                 Cl
##   [3]                                                                                 Cl
##   [4]                                                                                 Cl
##       Case_IonXpress_024_R_2012_09_13_15_10_42_Sequoia_SN1.28.Run_22_Auto_Sequoia_SN1.28.Run_22_55.sa
##                                                                                    <facto
##   [1]                                                                                 Cl
##   [2]                                                                                 Cl
##   [3]                                                                                 Cl
##   [4]                                                                                 Cl
##       Case_IonXpress_025_R_2012_09_13_15_10_42_Sequoia_SN1.28.Run_22_Auto_Sequoia_SN1.28.Run_22_55.sa
##                                                                                    <facto
##   [1]                                                                                 Cl
##   [2]                                                                                 Cl
```

```
##   [3]                                                                                                                           Cl
##   [4]                                                                                                                           Cl
##       Case_IonXpress_026_R_2012_09_13_15_10_42_Sequoia_SN1.28.Run_22_Auto_Sequoia_SN1.28.Run_22_55.sa
##                                                                                                                           <facto
##   [1]                                                                                                                           Cl
##   [2]                                                                                                                           Cl
##   [3]                                                                                                                           Cl
##   [4]                                                                                                                           Cl
##       Case_IonXpress_027_R_2012_09_13_15_10_42_Sequoia_SN1.28.Run_22_Auto_Sequoia_SN1.28.Run_22_55.sa
##                                                                                                                           <facto
##   [1]                                                                                                                           Cl
##   [2]                                                                                                                           Cl
##   [3]                                                                                                                           Cl
##   [4]                                                                                                                           Cl
##       Case_IonXpress_028_R_2012_09_13_15_10_42_Sequoia_SN1.28.Run_22_Auto_Sequoia_SN1.28.Run_22_55.sa
##                                                                                                                           <facto
##   [1]                                                                                                                           Cl
##   [2]                                                                                                                           Cl
##   [3]                                                                                                                           Cl
##   [4]                                                                                                                           Cl
##       Case_IonXpress_029_R_2012_09_13_15_10_42_Sequoia_SN1.28.Run_22_Auto_Sequoia_SN1.28.Run_22_55.sa
##                                                                                                                           <facto
##   [1]                                                                                                                           Cl
##   [2]                                                                                                                           Cl
##   [3]                                                                                                                           Cl
##   [4]                                                                                                                           Cl
##       Case_IonXpress_030_R_2012_09_13_15_10_42_Sequoia_SN1.28.Run_22_Auto_Sequoia_SN1.28.Run_22_55.sa
##                                                                                                                           <facto
##   [1]                                                                                                                           Cl
##   [2]                                                                                                                           Cl
##   [3]                                                                                                                           Cl
##   [4]                                                                                                                           Cl
##       Case_IonXpress_031_R_2012_09_13_15_10_42_Sequoia_SN1.28.Run_22_Auto_Sequoia_SN1.28.Run_22_55.sa
##                                                                                                                           <facto
##   [1]                                                                                                                           Cl
##   [2]                                                                                                                           Cl
##   [3]                                                                                                                           Cl
##   [4]                                                                                                                           Cl
##       Case_IonXpress_032_R_2012_09_13_15_10_42_Sequoia_SN1.28.Run_22_Auto_Sequoia_SN1.28.Run_22_55.sa
##                                                                                                                           <facto
##   [1]                                                                                                                           Cl
##   [2]                                                                                                                           Cl
##   [3]                                                                                                                           Cl
##   [4]                                                                                                                           Cl
##       Case_IonXpress_033_R_2012_09_13_15_10_42_Sequoia_SN1.28.Run_22_Auto_Sequoia_SN1.28.Run_22_55.sa
##                                                                                                                           <facto
##   [1]                                                                                                                           Cl
##   [2]                                                                                                                           Cl
##   [3]                                                                                                                           Cl
##   [4]                                                                                                                           Cl
##       Case_IonXpress_034_R_2012_09_13_15_10_42_Sequoia_SN1.28.Run_22_Auto_Sequoia_SN1.28.Run_22_55.sa
##                                                                                                                           <facto
##   [1]                                                                                                                           Cl
##   [2]                                                                                                                           Cl
```

```
##   [3]                                                                                                   Cl
##   [4]                                                                                                   Cl
##       Case_IonXpress_035_R_2012_09_13_15_10_42_Sequoia_SN1.28.Run_22_Auto_Sequoia_SN1.28.Run_22_55.sa
##                                                                                                     <facto
##   [1]                                                                                                   Cl
##   [2]                                                                                                   Cl
##   [3]                                                                                                   Cl
##   [4]                                                                                                   Cl
##       Case_IonXpress_036_R_2012_09_13_15_10_42_Sequoia_SN1.28.Run_22_Auto_Sequoia_SN1.28.Run_22_55.sa
##                                                                                                     <facto
##   [1]                                                                                                   Cl
##   [2]                                                                                                   Cl
##   [3]                                                                                                   Cl
##   [4]                                                                                                   Cl
##       Case_IonXpress_038_R_2012_09_13_15_10_42_Sequoia_SN1.28.Run_22_Auto_Sequoia_SN1.28.Run_22_55.sa
##                                                                                                     <facto
##   [1]                                                                                                   Cl
##   [2]                                                                                                   Cl
##   [3]                                                                                                   Cl
##   [4]                                                                                                   Cl
##       Case_IonXpress_039_R_2012_09_13_15_10_42_Sequoia_SN1.28.Run_22_Auto_Sequoia_SN1.28.Run_22_55.sa
##                                                                                                     <facto
##   [1]                                                                                                   Cl
##   [2]                                                                                                   Cl
##   [3]                                                                                                   Cl
##   [4]                                                                                                   Cl
##       Case_IonXpress_040_R_2012_09_13_15_10_42_Sequoia_SN1.28.Run_22_Auto_Sequoia_SN1.28.Run_22_55.sa
##                                                                                                     <facto
##   [1]                                                                                                   Cl
##   [2]                                                                                                   Cl
##   [3]                                                                                                   Cl
##   [4]                                                                                                   Cl
##       Case_IonXpress_041_R_2012_09_13_15_10_42_Sequoia_SN1.28.Run_22_Auto_Sequoia_SN1.28.Run_22_55.sa
##                                                                                                     <facto
##   [1]                                                                                                   Cl
##   [2]                                                                                                   Cl
##   [3]                                                                                                   Cl
##   [4]                                                                                                   Cl
##       Case_IonXpress_042_R_2012_09_13_15_10_42_Sequoia_SN1.28.Run_22_Auto_Sequoia_SN1.28.Run_22_55.sa
##                                                                                                     <facto
##   [1]                                                                                                   Cl
##   [2]                                                                                                   Cl
##   [3]                                                                                                   Cl
##   [4]                                                                                                   Cl
##       Case_IonXpress_043_R_2012_09_13_15_10_42_Sequoia_SN1.28.Run_22_Auto_Sequoia_SN1.28.Run_22_55.sa
##                                                                                                     <facto
##   [1]                                                                                                   Cl
##   [2]                                                                                                   Cl
##   [3]                                                                                                   Cl
##   [4]                                                                                                   Cl
##       Case_IonXpress_044_R_2012_09_13_15_10_42_Sequoia_SN1.28.Run_22_Auto_Sequoia_SN1.28.Run_22_55.sa
##                                                                                                     <facto
##   [1]                                                                                                   Cl
##   [2]                                                                                                   Cl
```

```
##   [3]                                                                                                         CN
##   [4]                                                                                                         CN
##       Case_IonXpress_045_R_2012_09_13_15_10_42_Sequoia_SN1.28.Run_22_Auto_Sequoia_SN1.28.Run_22_55.sa
##                                                                                                         <factor
##   [1]                                                                                                         CN
##   [2]                                                                                                         CN
##   [3]                                                                                                         CN
##   [4]                                                                                                         CN
##       Case_IonXpress_046_R_2012_09_13_15_10_42_Sequoia_SN1.28.Run_22_Auto_Sequoia_SN1.28.Run_22_55.sa
##                                                                                                         <factor
##   [1]                                                                                                         CN
##   [2]                                                                                                         CN
##   [3]                                                                                                         CN
##   [4]                                                                                                         CN
##       Case_IonXpress_047_R_2012_09_13_15_10_42_Sequoia_SN1.28.Run_22_Auto_Sequoia_SN1.28.Run_22_55.sa
##                                                                                                         <factor
##   [1]                                                                                                         CN
##   [2]                                                                                                         CN
##   [3]                                                                                                         CN
##   [4]                                                                                                         CN
##       Case_IonXpress_048_R_2012_09_13_15_10_42_Sequoia_SN1.28.Run_22_Auto_Sequoia_SN1.28.Run_22_55.sa
##                                                                                                         <factor
##   [1]                                                                                                         CN
##   [2]                                                                                                         CN
##   [3]                                                                                                         CN
##   [4]                                                                                                         CN
##   -------
##   seqinfo: 1 sequence from an unspecified genome; no seqlengths
##
## Individual CNVs:
## GRanges object with 8 ranges and 4 metadata columns:
##       seqnames    ranges strand |
##          <Rle> <IRanges>  <Rle> |
##   [1]    undef  [71, 73]      * |
##   [2]    undef  [71, 73]      * |
##   [3]    undef  [71, 73]      * |
##   [4]    undef  [30, 32]      * |
##   [5]    undef  [30, 32]      * |
##   [6]    undef  [ 1,  9]      * |
##   [7]    undef  [30, 32]      * |
##   [8]    undef  [75, 77]      * |
##                                                                                                       sampleNam
##                                                                                                         <factor
##   [1] Case_IonXpress_018_R_2012_09_13_15_10_42_Sequoia_SN1.28.Run_22_Auto_Sequoia_SN1.28.Run_22_55.sa
##   [2] Case_IonXpress_029_R_2012_09_13_15_10_42_Sequoia_SN1.28.Run_22_Auto_Sequoia_SN1.28.Run_22_55.sa
##   [3] Case_IonXpress_038_R_2012_09_13_15_10_42_Sequoia_SN1.28.Run_22_Auto_Sequoia_SN1.28.Run_22_55.sa
##   [4] Case_IonXpress_039_R_2012_09_13_15_10_42_Sequoia_SN1.28.Run_22_Auto_Sequoia_SN1.28.Run_22_55.sa
##   [5] Case_IonXpress_040_R_2012_09_13_15_10_42_Sequoia_SN1.28.Run_22_Auto_Sequoia_SN1.28.Run_22_55.sa
##   [6] Case_IonXpress_044_R_2012_09_13_15_10_42_Sequoia_SN1.28.Run_22_Auto_Sequoia_SN1.28.Run_22_55.sa
##   [7] Case_IonXpress_045_R_2012_09_13_15_10_42_Sequoia_SN1.28.Run_22_Auto_Sequoia_SN1.28.Run_22_55.sa
##   [8] Case_IonXpress_048_R_2012_09_13_15_10_42_Sequoia_SN1.28.Run_22_Auto_Sequoia_SN1.28.Run_22_55.sa
##           median       mean          CN
##        <numeric>  <numeric> <character>
##   [1]  0.5849624  0.5849369         CN3
```

```
##   [2]  0.5821153  0.5638646         CN3
##   [3]  0.5824591  0.5819343         CN3
##   [4]  1.1774289  0.9468308         CN3
##   [5]  0.6434855  0.6872528         CN3
##   [6] -5.3219281 -3.5478935         CN1
##   [7]  1.2685088  1.1372004         CN3
##   [8]  0.5626960  0.5536927         CN3
##   -------
##   seqinfo: 1 sequence from an unspecified genome; no seqlengths
## [1] "/Users/gdemidov/Downloads/doc/Run_SN1_41_CRG_fin_05_qc.xls"

## Normalizing...

## Starting local modeling, please be patient...

## Reference sequence:  undef

## Starting segmentation algorithm...

## Using "fastseg" for segmentation.

## [1] ""
## [1] "/Users/gdemidov/Downloads/doc/Run_SN1_41_CRG_fin_05_qc.xls"
## [1] ""

## Segplot might not work because of special characters in the sample names. Use only A-Z,a-z and 0-9!
##  There is a hidden function cn.mops:::.replaceNames that replaces the names in the "CNVDetectionResul
```

s_048_R_2012_09_13_15_10_42_Sequoia_SN1.28.Run_22_Auto_Sequoia_SN1.28.I

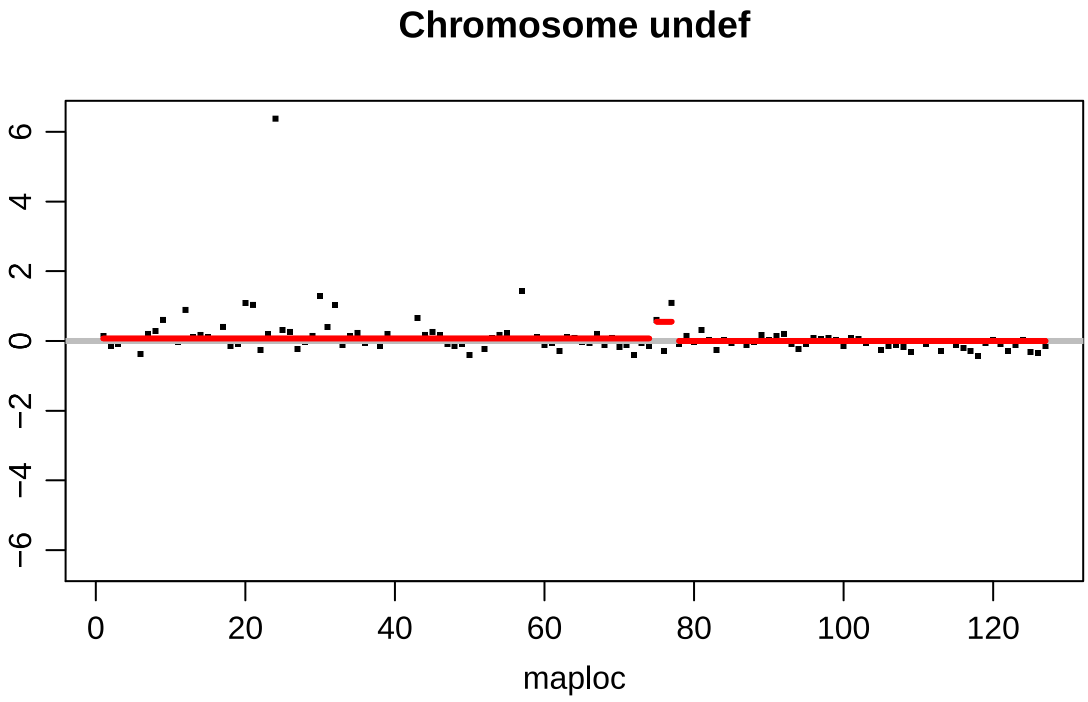

```
## Segplot might not work because of special characters in the sample names. Use only A-Z,a-z and 0-9!
##  There is a hidden function cn.mops:::.replaceNames that replaces the names in the "CNVDetectionResul
```

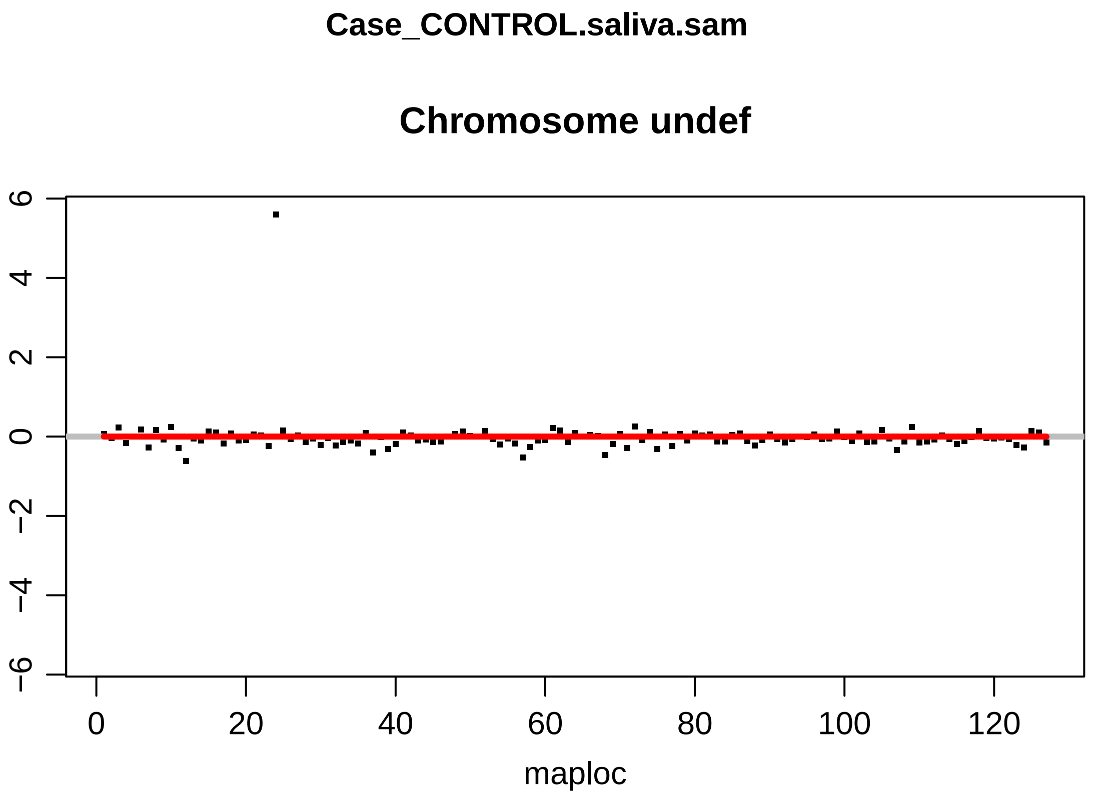


```
## Segplot might not work because of special characters in the sample names. Use only A-Z,a-z and 0-9!
##  There is a hidden function cn.mops:::.replaceNames that replaces the names in the "CNVDetectionResul
```

Case_L001.G1.sam

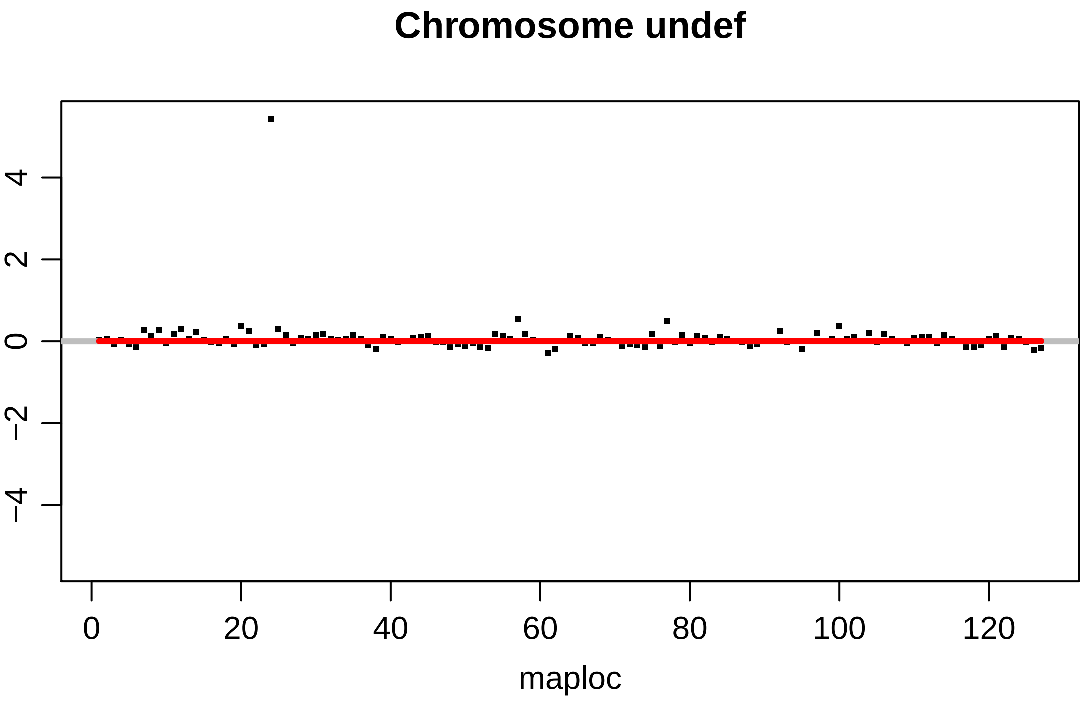


```
## Segplot might not work because of special characters in the sample names. Use only A-Z,a-z and 0-9!
##  There is a hidden function cn.mops:::.replaceNames that replaces the names in the "CNVDetectionResul
```

Case_L002.G1.sam

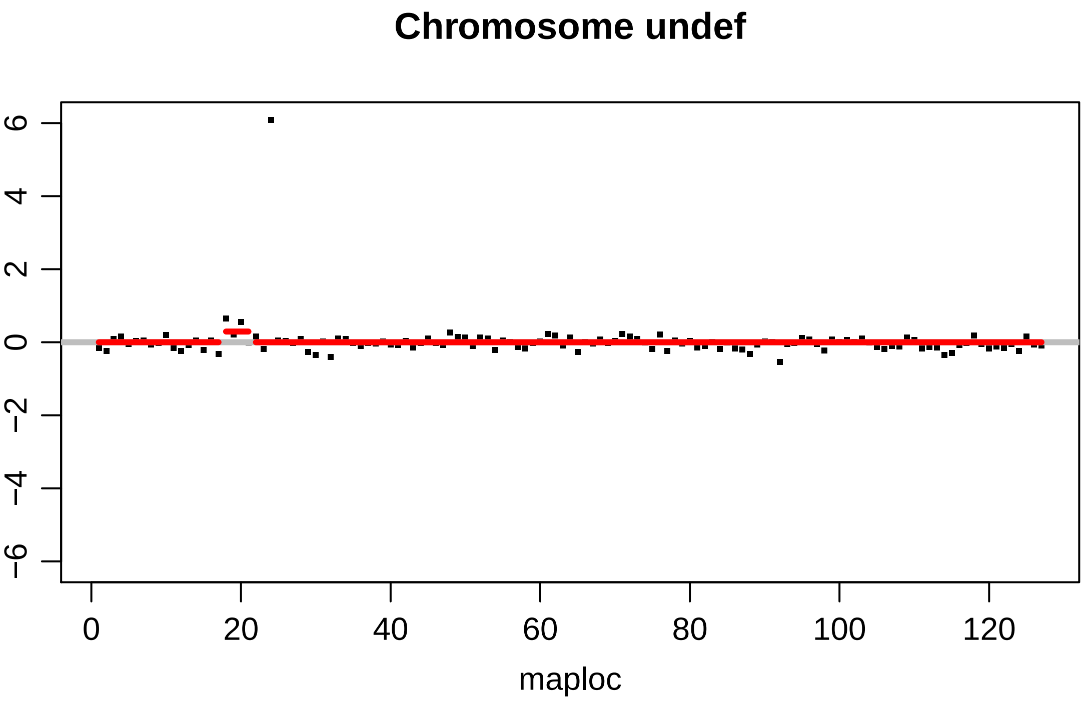

```
## Segplot might not work because of special characters in the sample names. Use only A-Z,a-z and 0-9!
##  There is a hidden function cn.mops:::.replaceNames that replaces the names in the "CNVDetectionResul
```

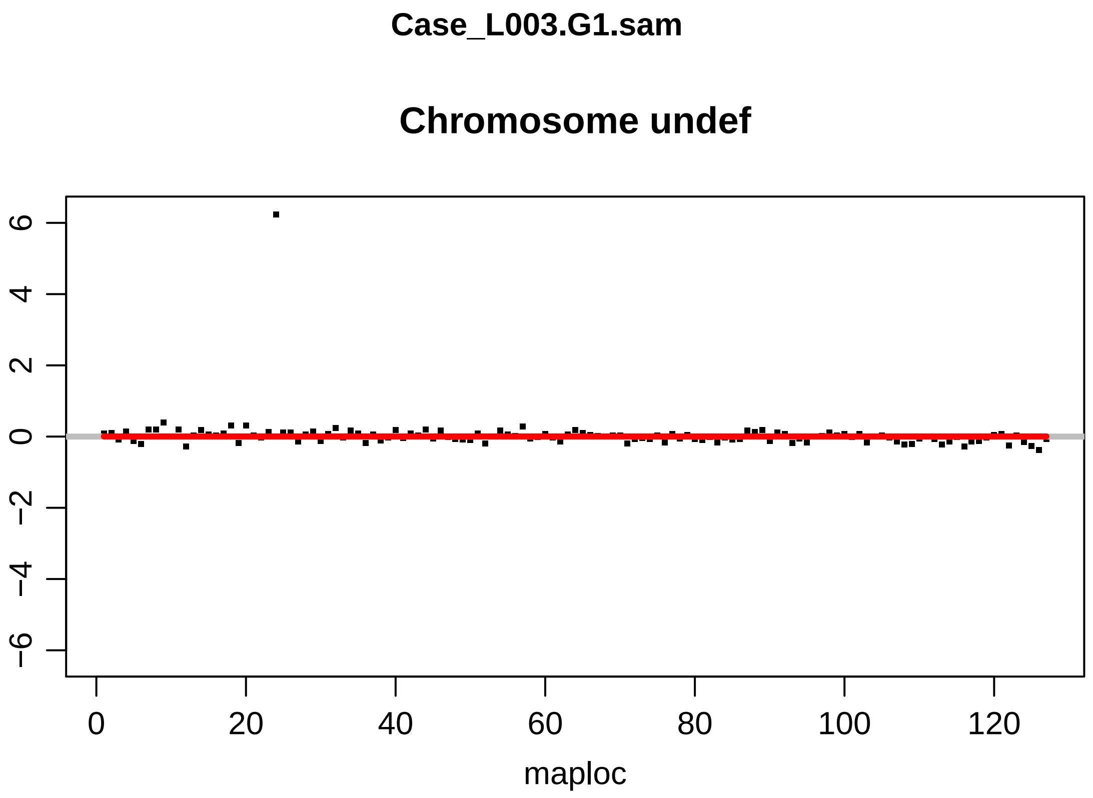


```
## Segplot might not work because of special characters in the sample names. Use only A-Z,a-z and 0-9!
##  There is a hidden function cn.mops:::.replaceNames that replaces the names in the "CNVDetectionResul
```

Case_L004.G1.sam

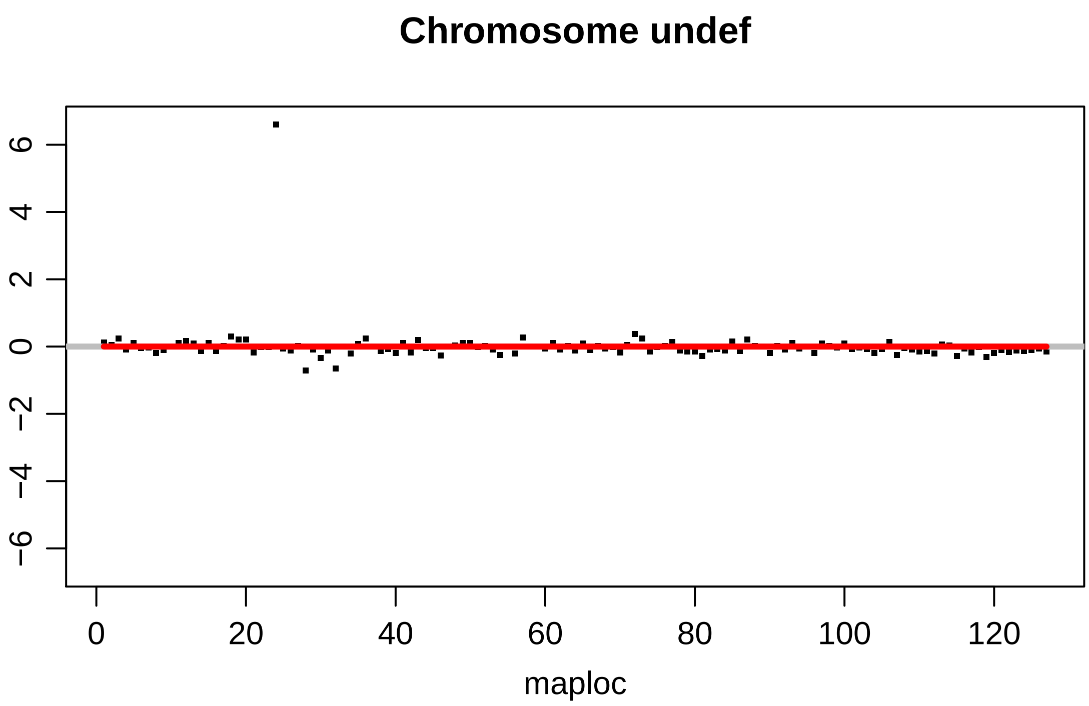


```
## Segplot might not work because of special characters in the sample names. Use only A-Z,a-z and 0-9!
##  There is a hidden function cn.mops:::.replaceNames that replaces the names in the "CNVDetectionResul
```

Case_L005.G1.sam

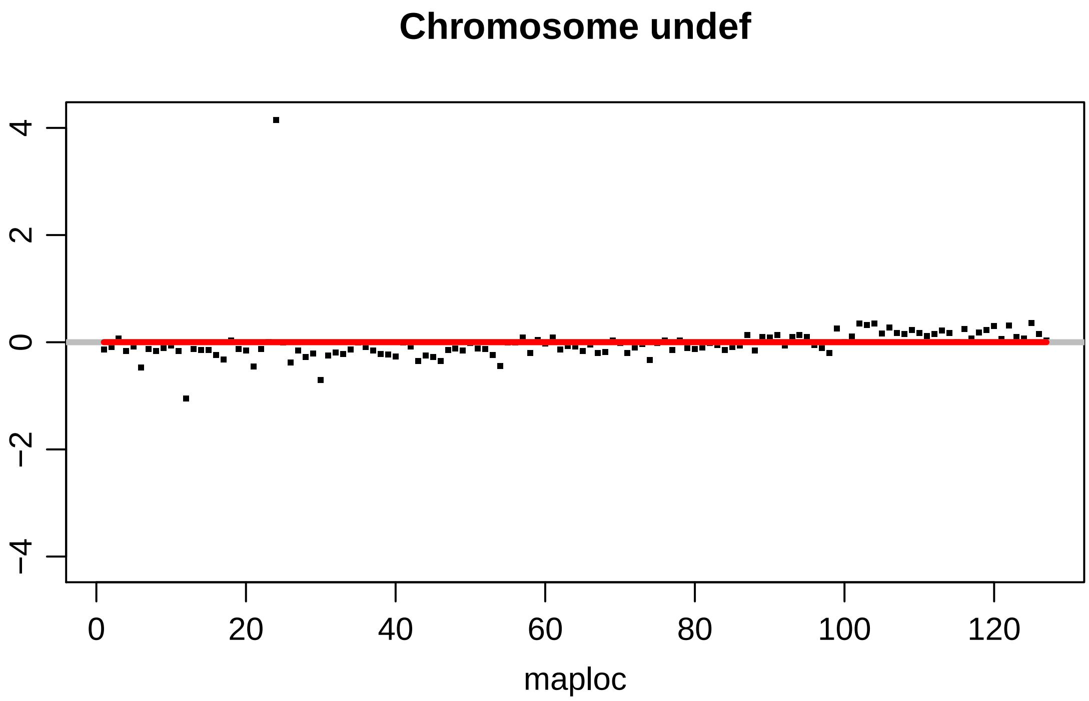

```
## Segplot might not work because of special characters in the sample names. Use only A-Z,a-z and 0-9!
##  There is a hidden function cn.mops:::.replaceNames that replaces the names in the "CNVDetectionResul
```

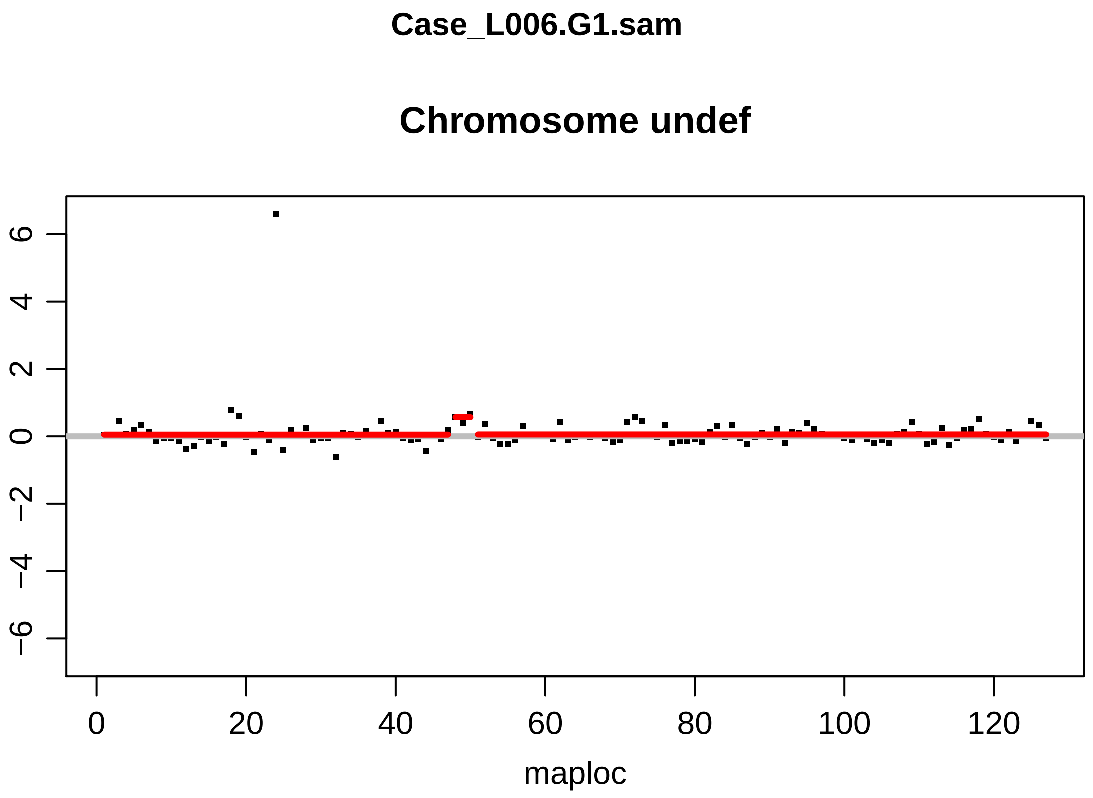


```
## Segplot might not work because of special characters in the sample names. Use only A-Z,a-z and 0-9!
##  There is a hidden function cn.mops:::.replaceNames that replaces the names in the "CNVDetectionResul
```

Case_L007.G1.sam

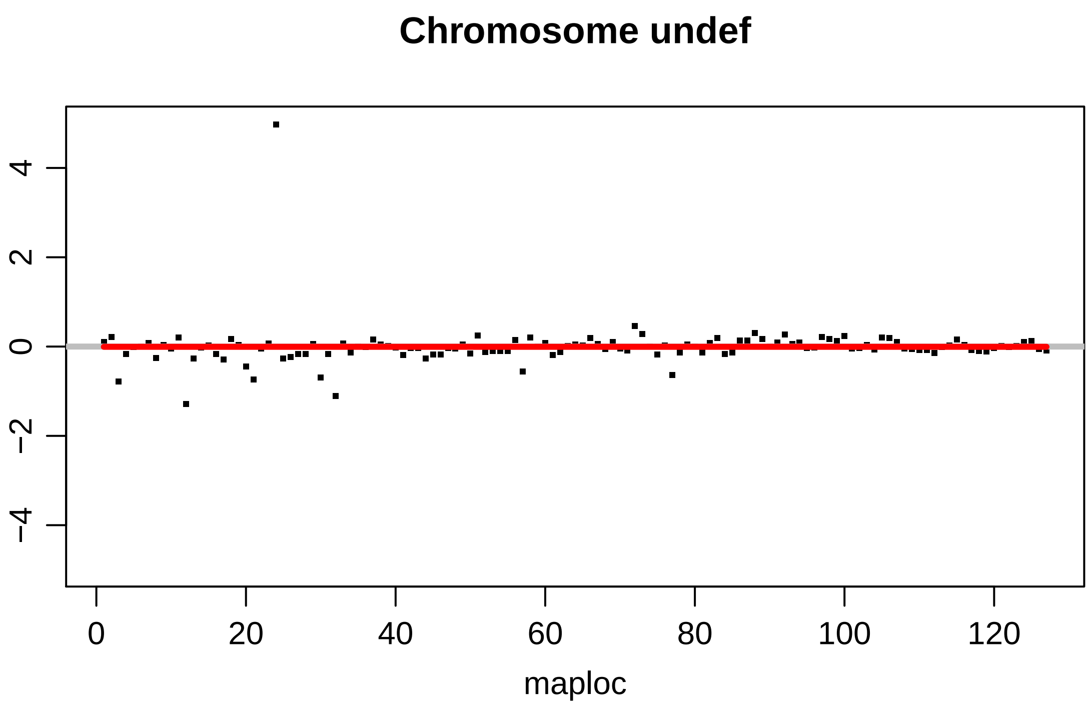


```
## Segplot might not work because of special characters in the sample names. Use only A-Z,a-z and 0-9!
##  There is a hidden function cn.mops:::.replaceNames that replaces the names in the "CNVDetectionResul
```

Case_L008.G1.sam

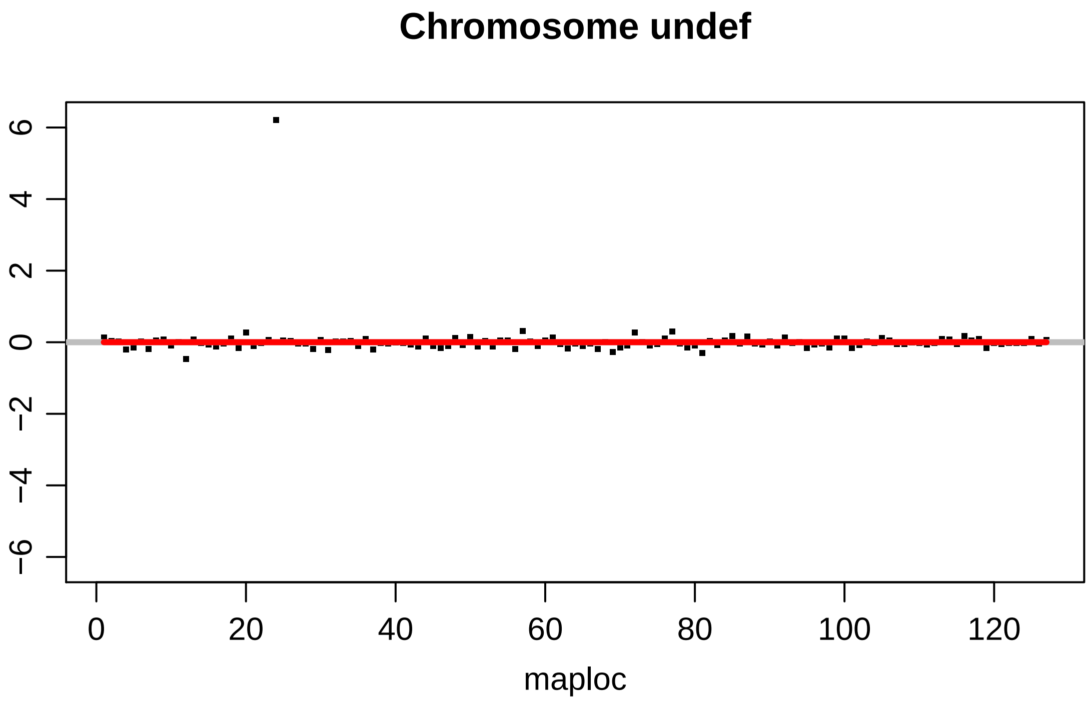

```
## Segplot might not work because of special characters in the sample names. Use only A-Z,a-z and 0-9!
##  There is a hidden function cn.mops:::.replaceNames that replaces the names in the "CNVDetectionResul
```

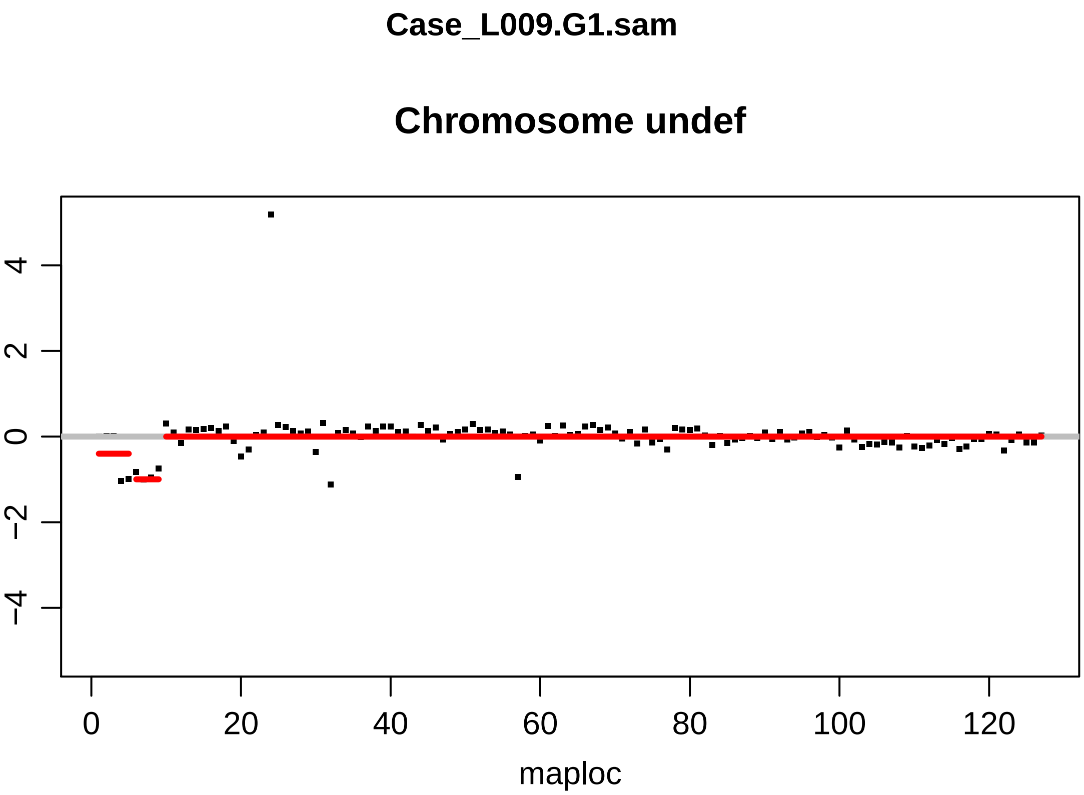


```
## Segplot might not work because of special characters in the sample names. Use only A-Z,a-z and 0-9!
##  There is a hidden function cn.mops:::.replaceNames that replaces the names in the "CNVDetectionResul
```

Case_L011.G1.sam

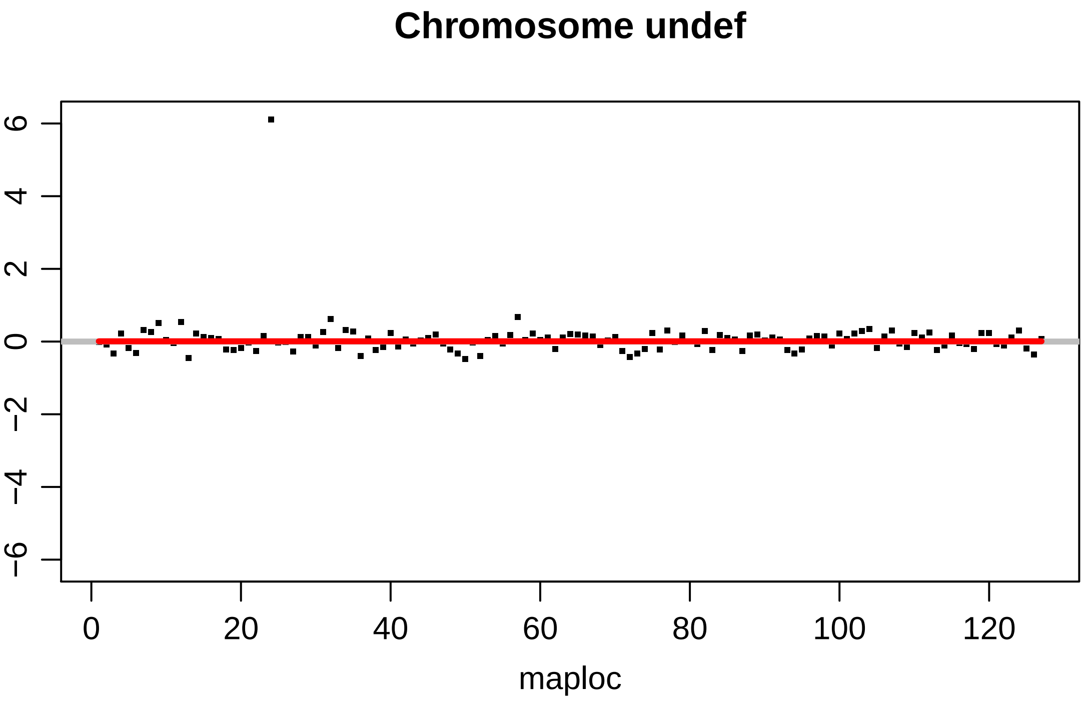


```
## Segplot might not work because of special characters in the sample names. Use only A-Z,a-z and 0-9!
##  There is a hidden function cn.mops:::.replaceNames that replaces the names in the "CNVDetectionResul
```

Case_L012.G1.sam

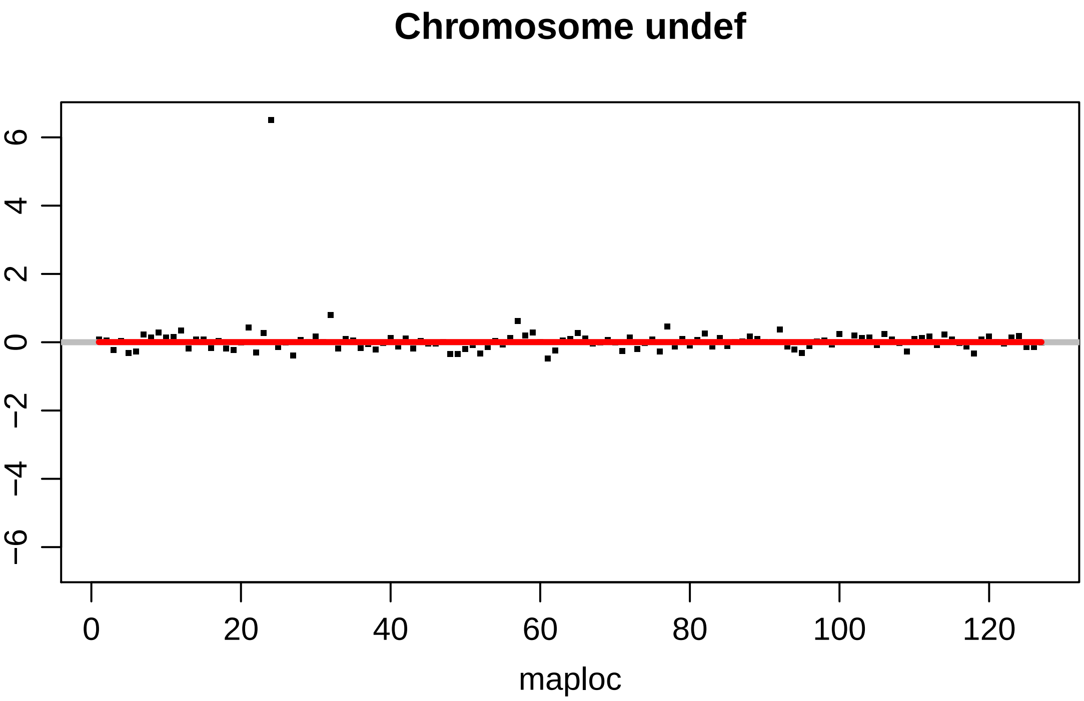

```
## Segplot might not work because of special characters in the sample names. Use only A-Z,a-z and 0-9!
##  There is a hidden function cn.mops:::.replaceNames that replaces the names in the "CNVDetectionResul
```

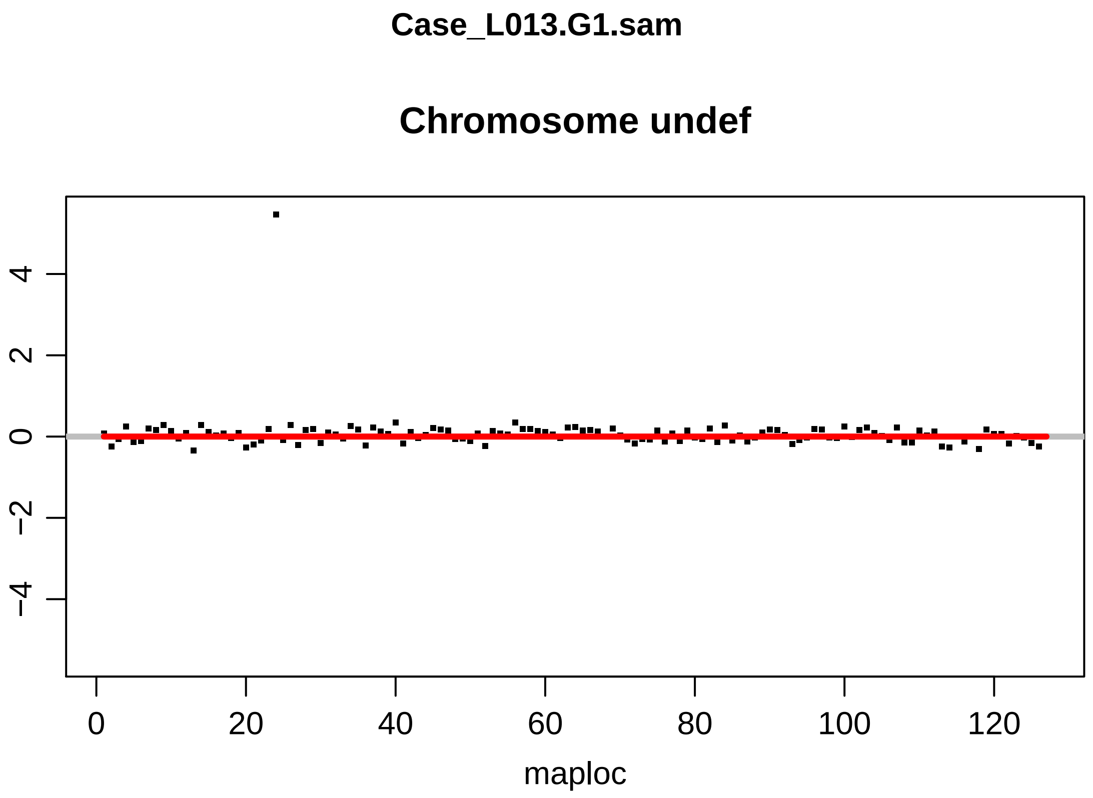


```
## Segplot might not work because of special characters in the sample names. Use only A-Z,a-z and 0-9!
##  There is a hidden function cn.mops:::.replaceNames that replaces the names in the "CNVDetectionResul
```

Case_L014.G1.sam

Chromosome undef

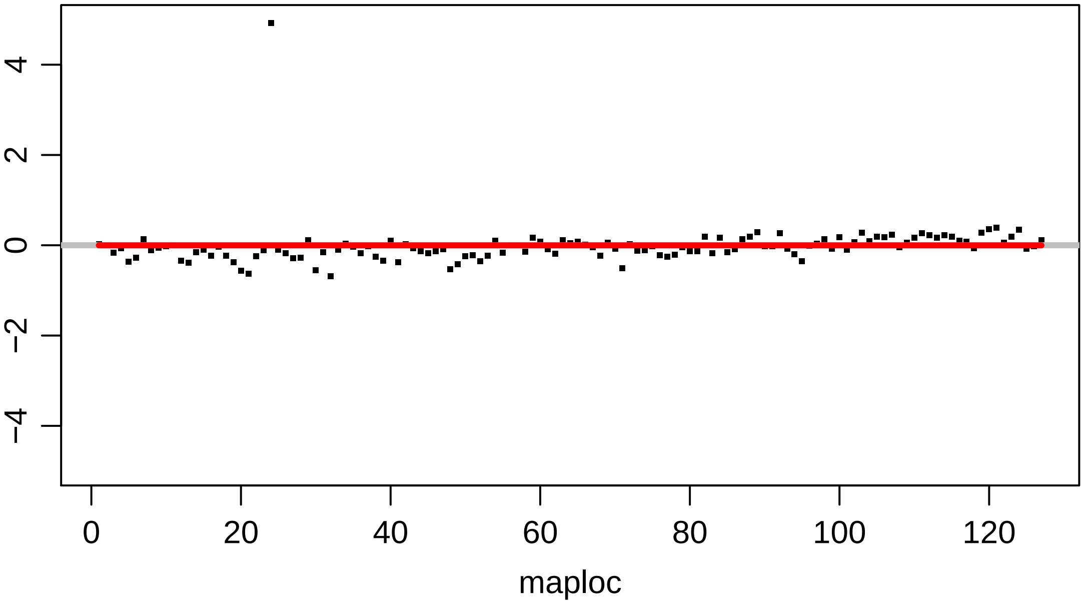


```
## Segplot might not work because of special characters in the sample names. Use only A-Z,a-z and 0-9!
##  There is a hidden function cn.mops:::.replaceNames that replaces the names in the "CNVDetectionResul
```

Case_L015.G1.sam

Chromosome undef

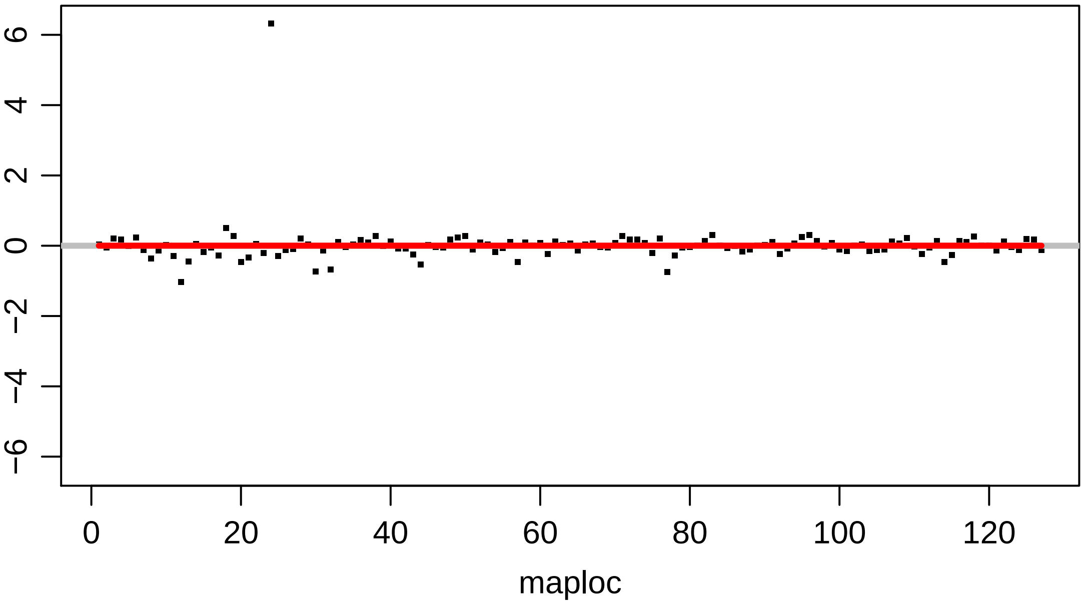

```
## Segplot might not work because of special characters in the sample names. Use only A-Z,a-z and 0-9!
##  There is a hidden function cn.mops:::.replaceNames that replaces the names in the "CNVDetectionResul
```

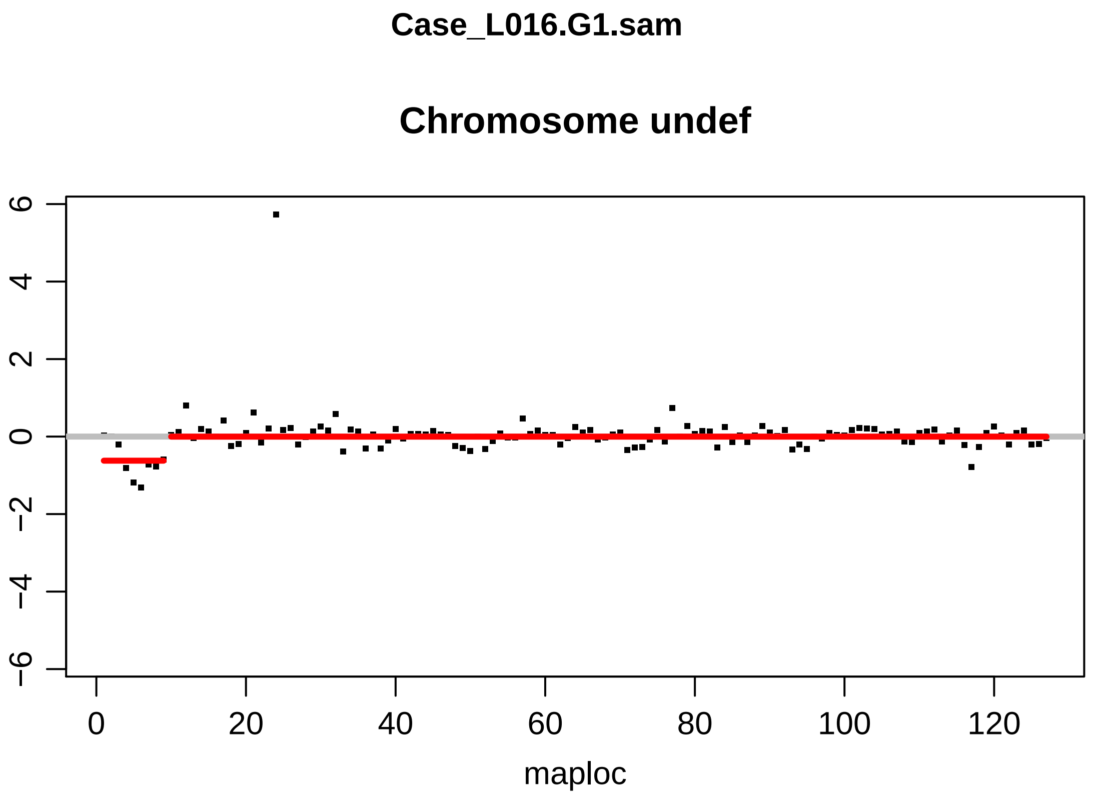


```
## Segplot might not work because of special characters in the sample names. Use only A-Z,a-z and 0-9!
##  There is a hidden function cn.mops:::.replaceNames that replaces the names in the "CNVDetectionResul
```

Case_L017.G1.sam

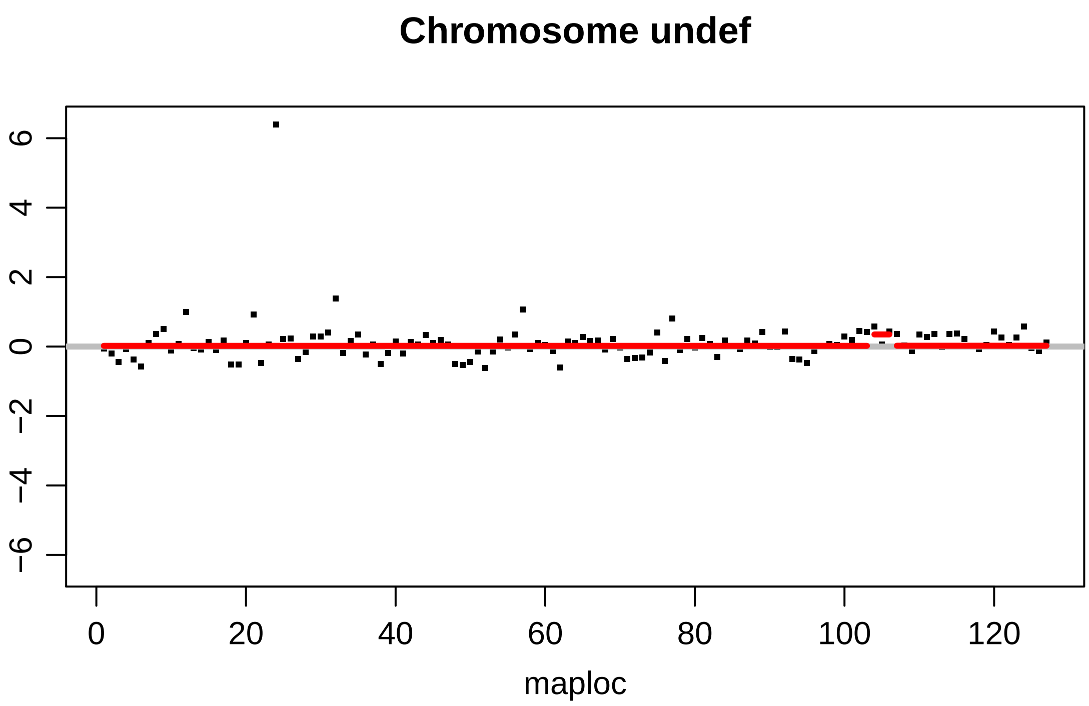


```
## Segplot might not work because of special characters in the sample names. Use only A-Z,a-z and 0-9!
##  There is a hidden function cn.mops:::.replaceNames that replaces the names in the "CNVDetectionResul
```

Case_L018.G1.sam

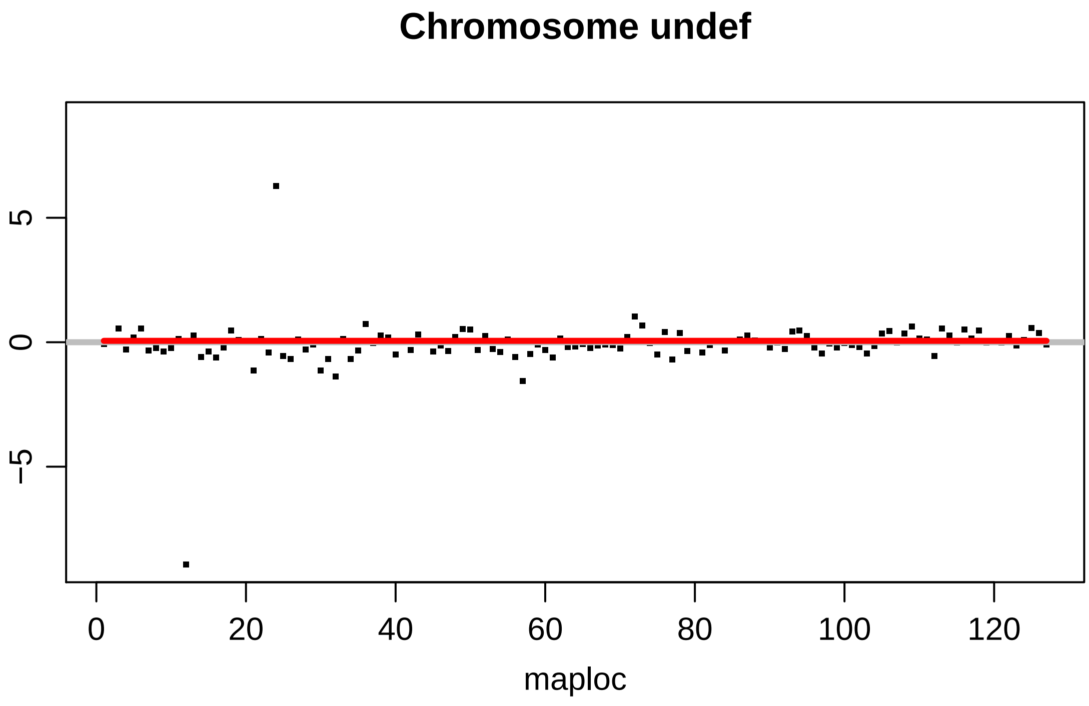

```
## Segplot might not work because of special characters in the sample names. Use only A-Z,a-z and 0-9!
##  There is a hidden function cn.mops:::.replaceNames that replaces the names in the "CNVDetectionResul
```

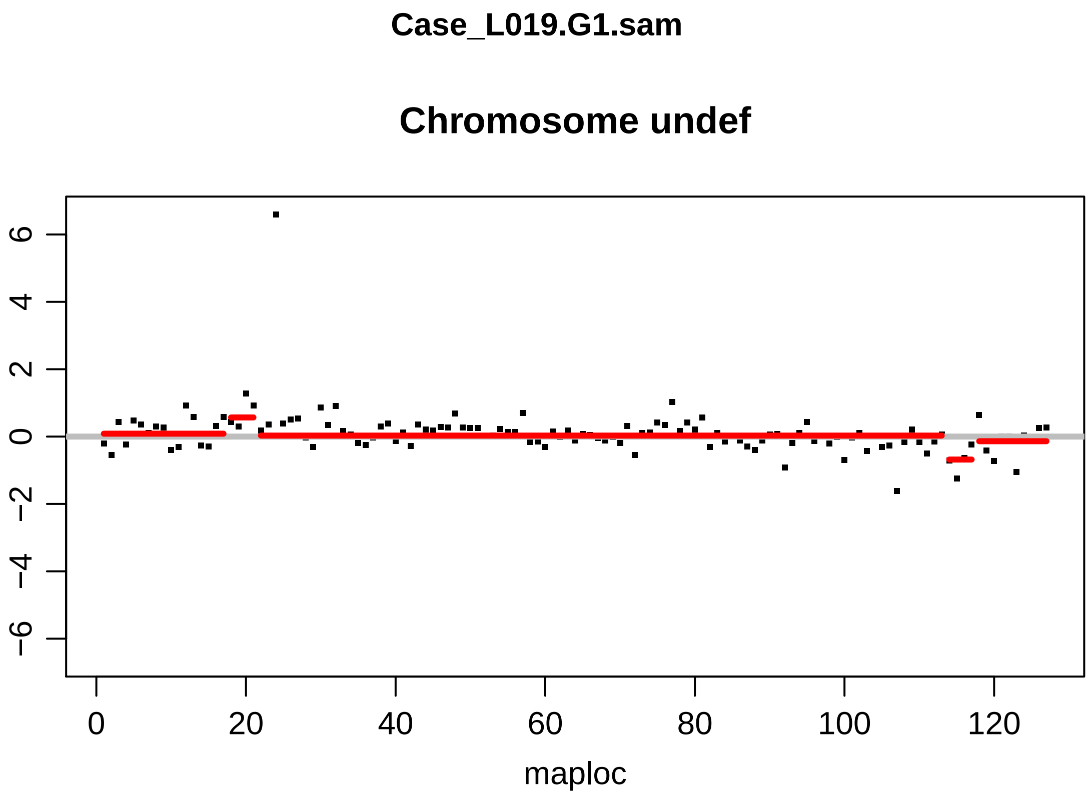


```
## Segplot might not work because of special characters in the sample names. Use only A-Z,a-z and 0-9!
##  There is a hidden function cn.mops:::.replaceNames that replaces the names in the "CNVDetectionResul
```

Case_L020.G1.sam

Chromosome undef

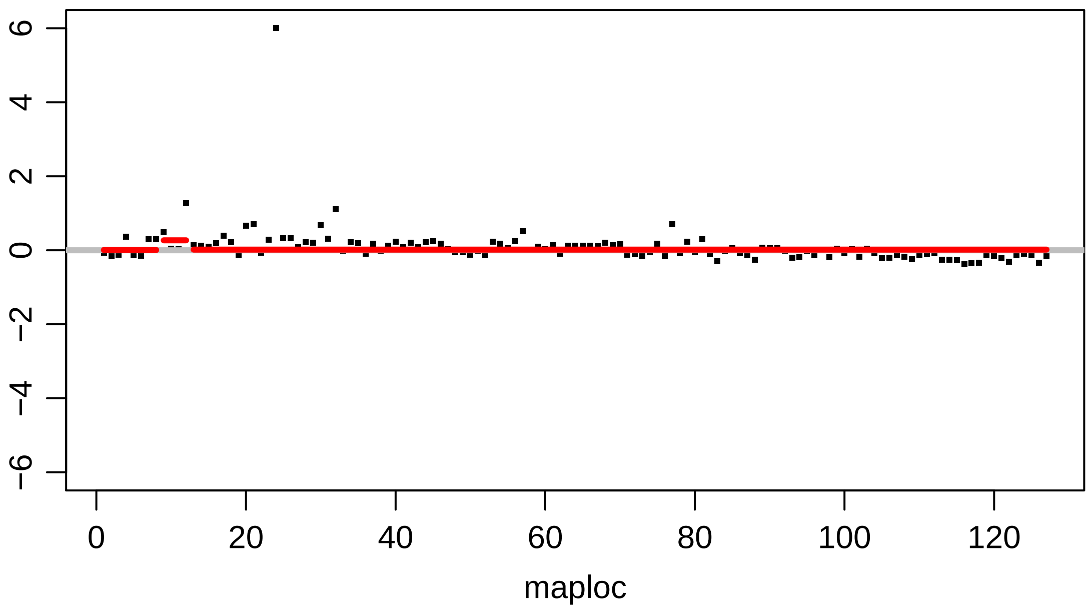


```
## Segplot might not work because of special characters in the sample names. Use only A-Z,a-z and 0-9!
##  There is a hidden function cn.mops:::.replaceNames that replaces the names in the "CNVDetectionResul
```

Case_L021.G1.sam

Chromosome undef

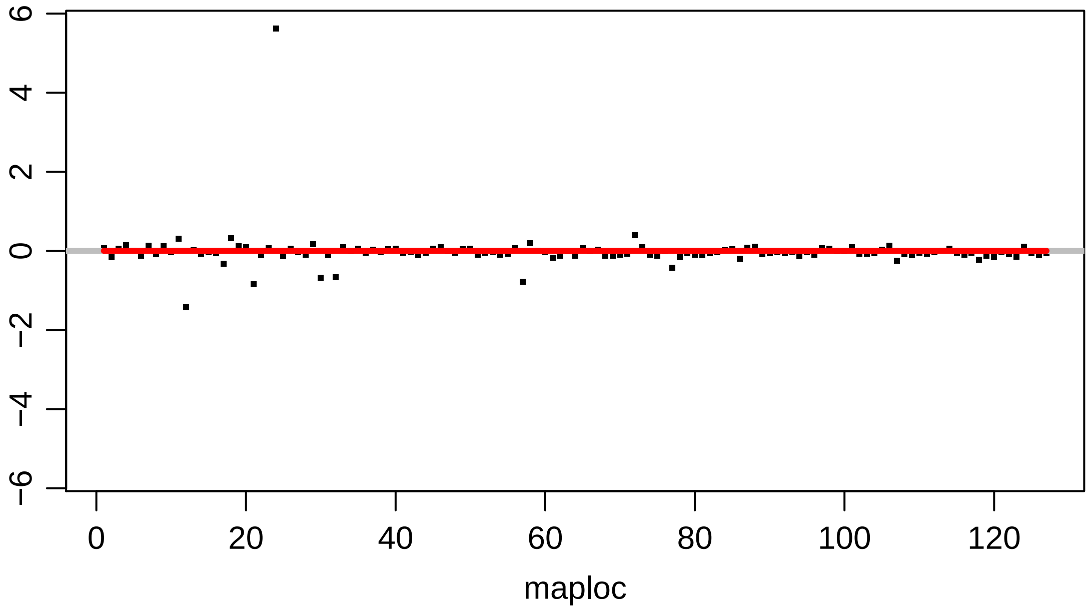

```
## Segplot might not work because of special characters in the sample names. Use only A-Z,a-z and 0-9!
##  There is a hidden function cn.mops:::.replaceNames that replaces the names in the "CNVDetectionResul
```

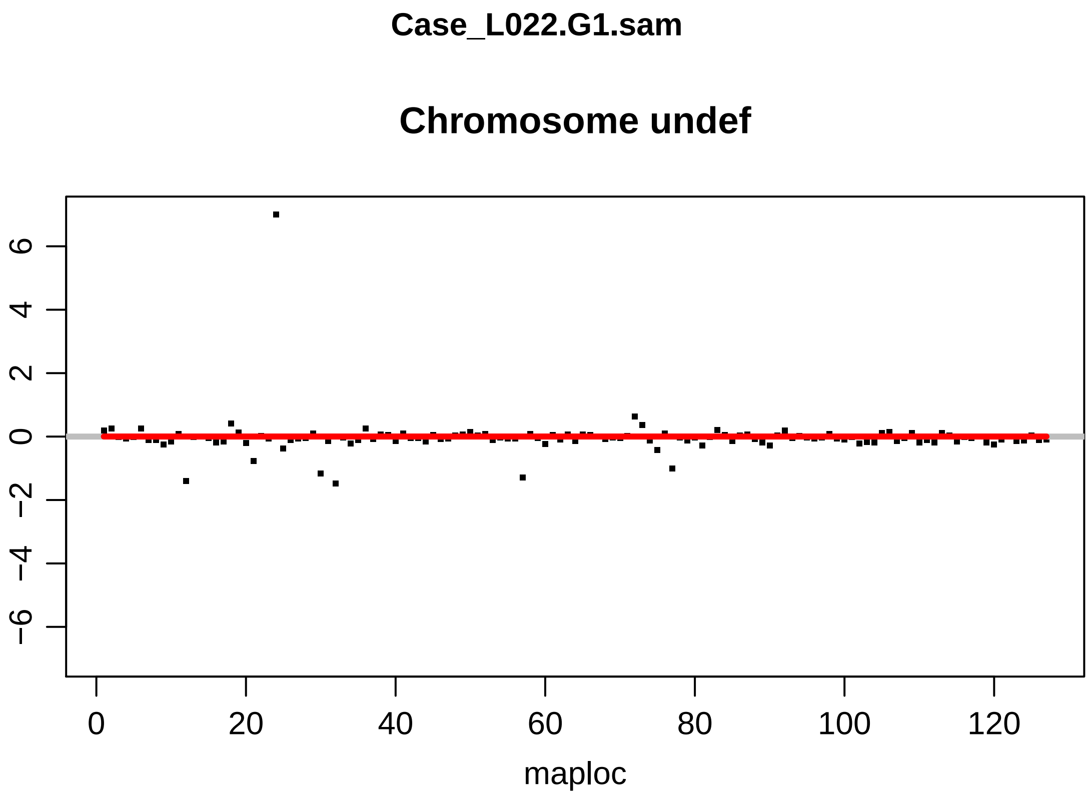


```
## Segplot might not work because of special characters in the sample names. Use only A-Z,a-z and 0-9!
##  There is a hidden function cn.mops:::.replaceNames that replaces the names in the "CNVDetectionResul
```

Case_L023.G1.sam

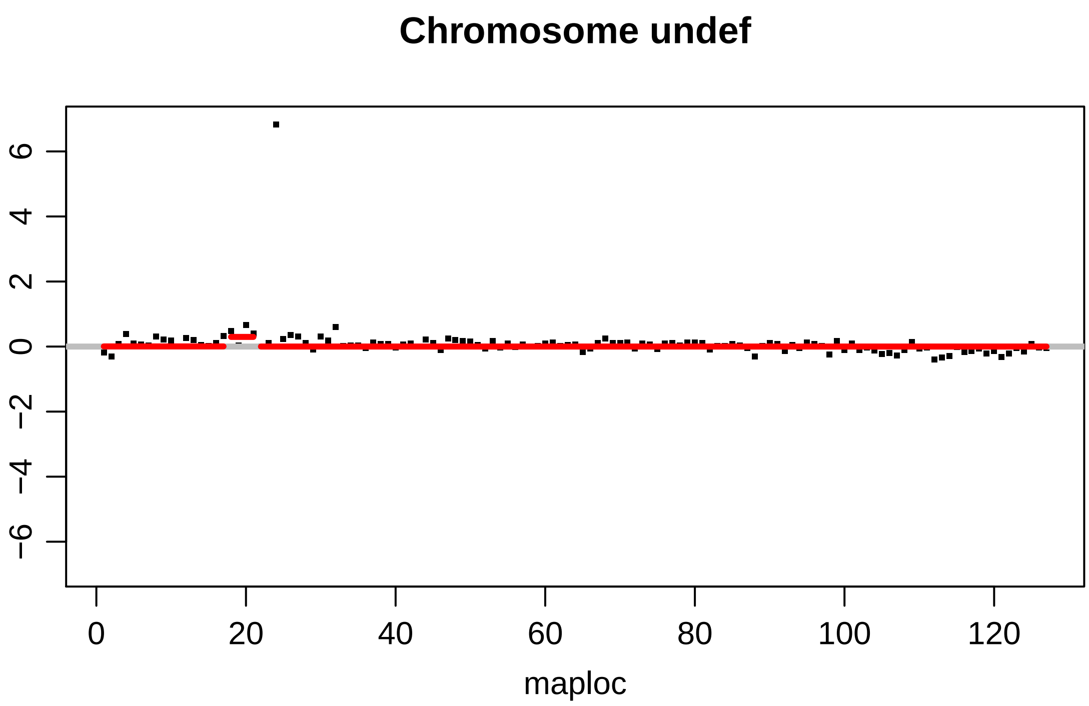


```
## Segplot might not work because of special characters in the sample names. Use only A-Z,a-z and 0-9!
##  There is a hidden function cn.mops:::.replaceNames that replaces the names in the "CNVDetectionResul
```

Case_L024.G1.sam

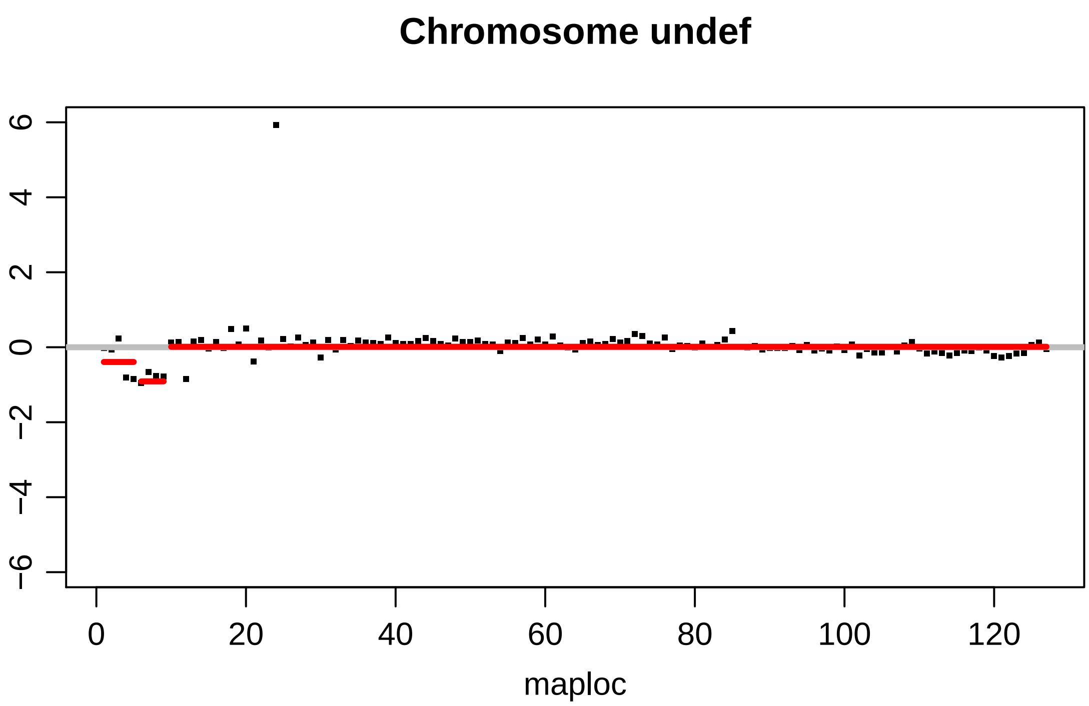

```
## Segplot might not work because of special characters in the sample names. Use only A-Z,a-z and 0-9!
##  There is a hidden function cn.mops:::.replaceNames that replaces the names in the "CNVDetectionResul
```

Case_L025.G1.sam

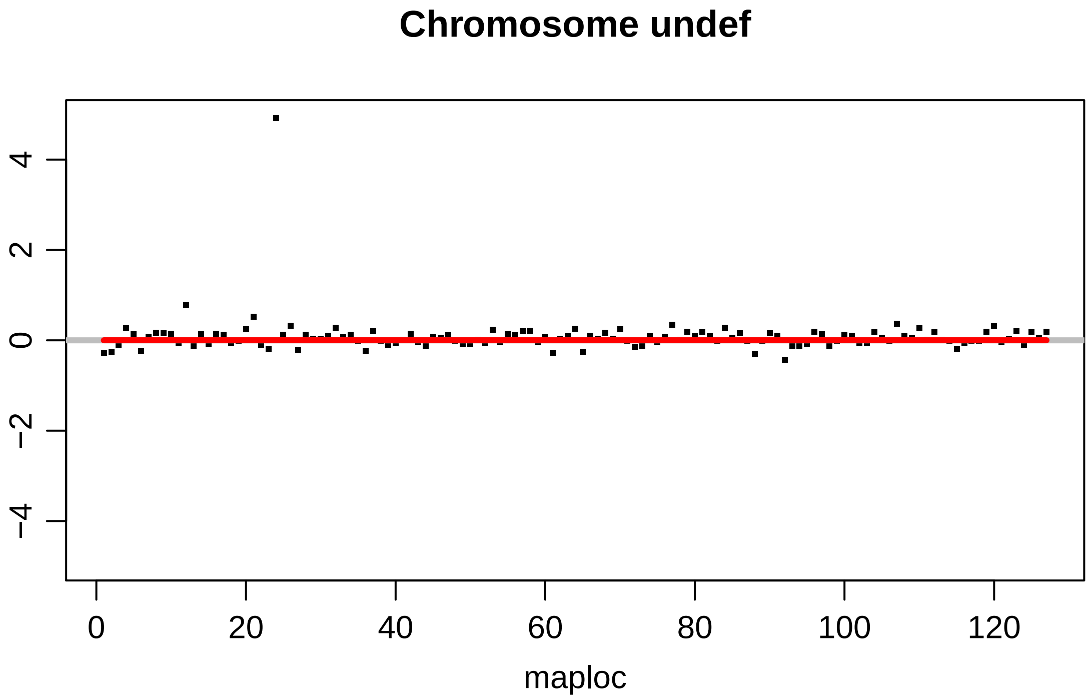


```
## Segplot might not work because of special characters in the sample names. Use only A-Z,a-z and 0-9!
##  There is a hidden function cn.mops:::.replaceNames that replaces the names in the "CNVDetectionResul
```

Case_L026.G1.sam

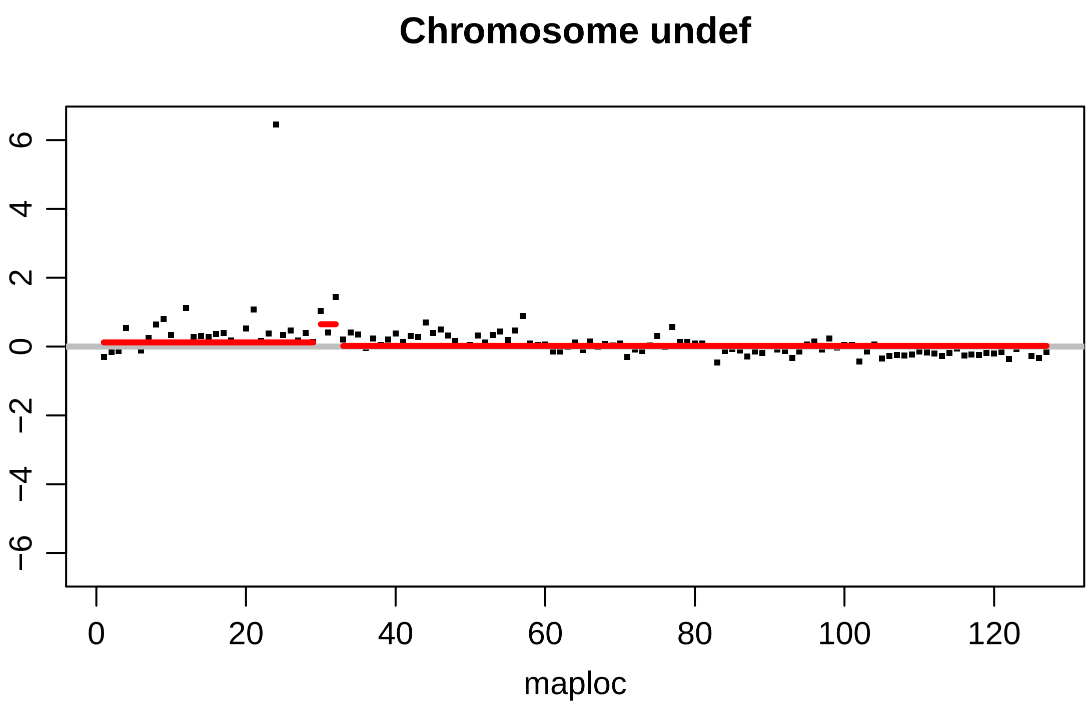


```
## Segplot might not work because of special characters in the sample names. Use only A-Z,a-z and 0-9!
##  There is a hidden function cn.mops:::.replaceNames that replaces the names in the "CNVDetectionResul
```

Case_L027.G1.sam

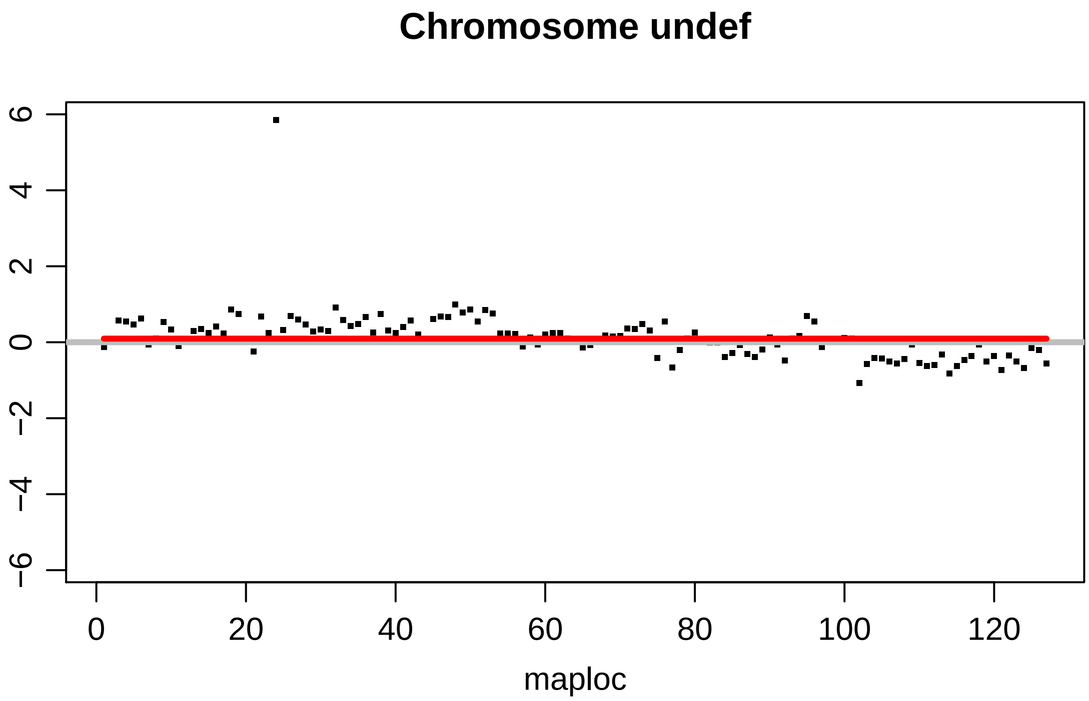

```
## Segplot might not work because of special characters in the sample names. Use only A-Z,a-z and 0-9!
##  There is a hidden function cn.mops:::.replaceNames that replaces the names in the "CNVDetectionResul
```

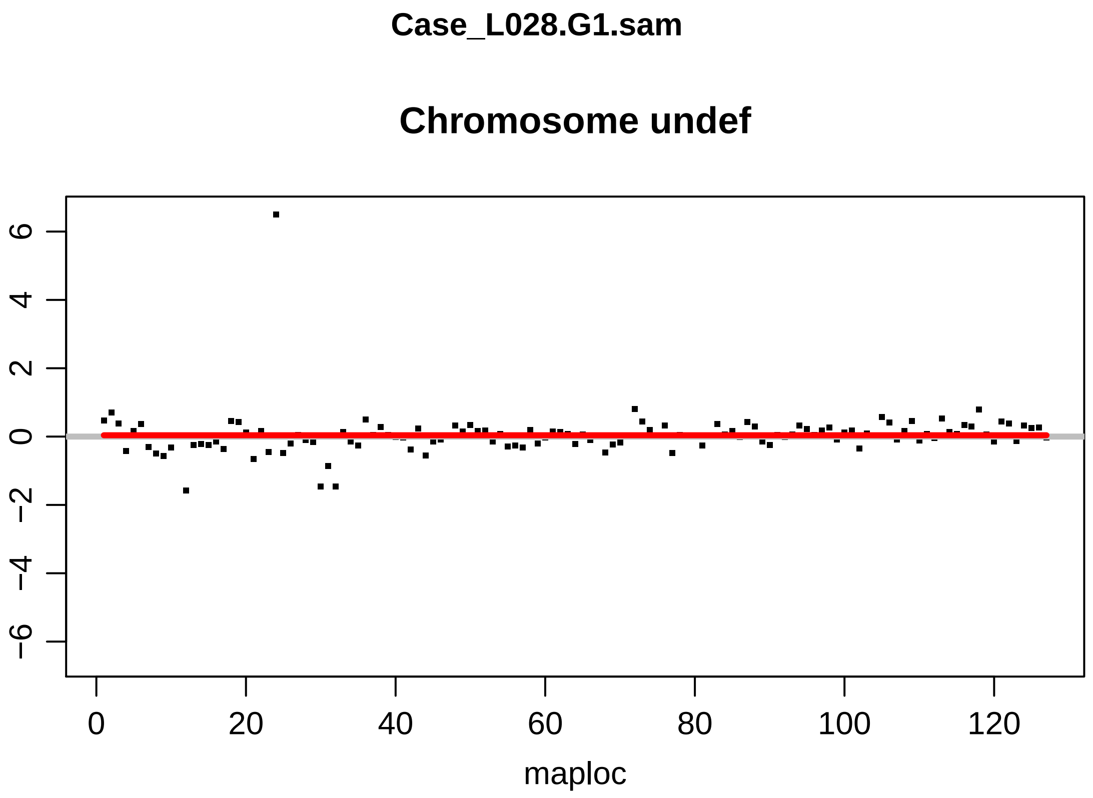


```
## Segplot might not work because of special characters in the sample names. Use only A-Z,a-z and 0-9!
##  There is a hidden function cn.mops:::.replaceNames that replaces the names in the "CNVDetectionResul
```

Case_L029.G1.sam

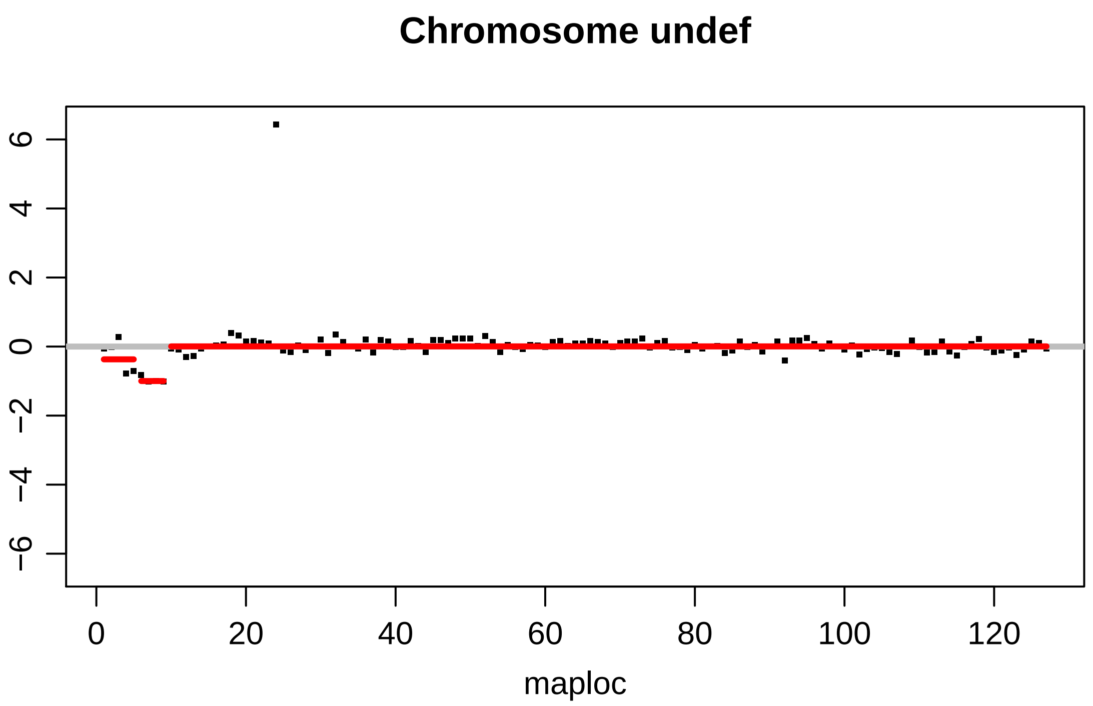


```
## Segplot might not work because of special characters in the sample names. Use only A-Z,a-z and 0-9!
##  There is a hidden function cn.mops:::.replaceNames that replaces the names in the "CNVDetectionResul
```

Case_L030.G1.sam

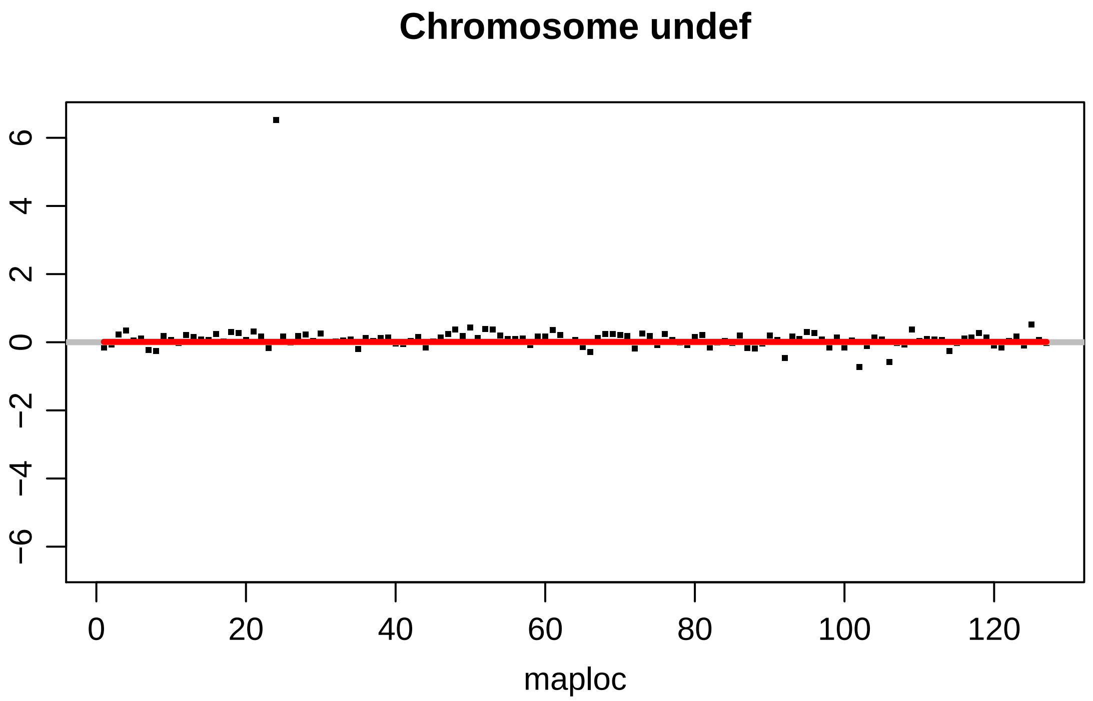

```
## Segplot might not work because of special characters in the sample names. Use only A-Z,a-z and 0-9!
##  There is a hidden function cn.mops:::.replaceNames that replaces the names in the "CNVDetectionResul
```

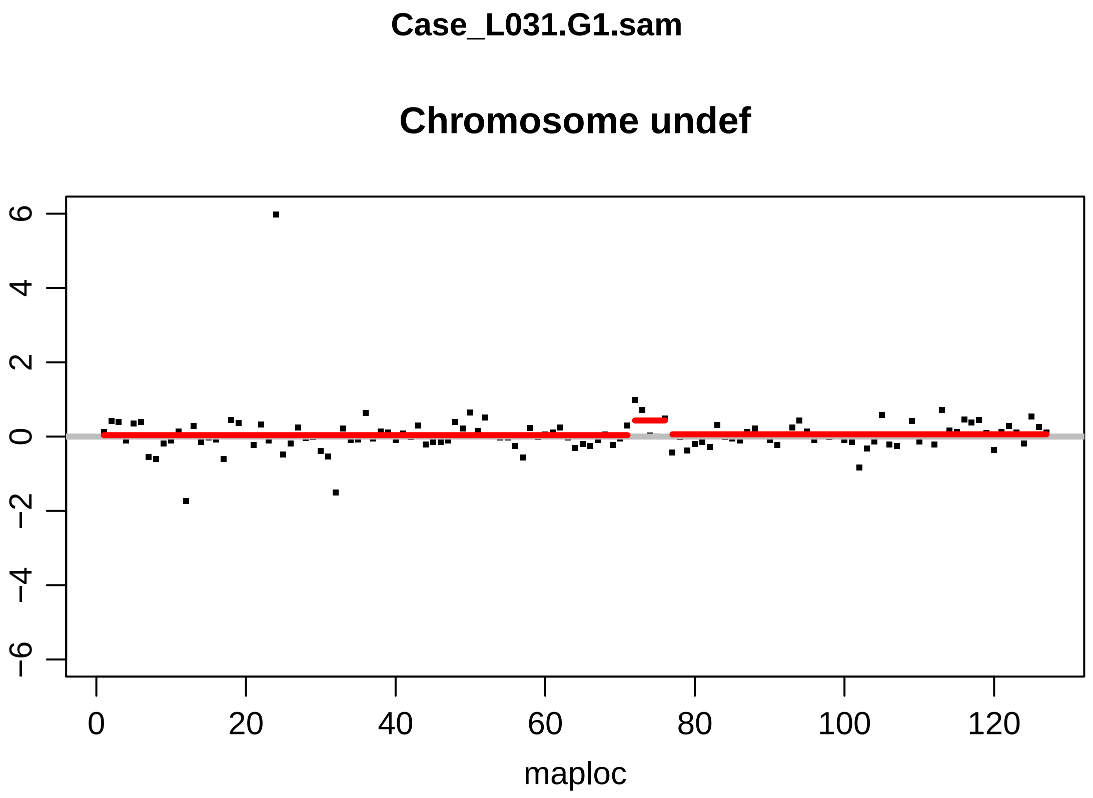


```
## Segplot might not work because of special characters in the sample names. Use only A-Z,a-z and 0-9!
##  There is a hidden function cn.mops:::.replaceNames that replaces the names in the "CNVDetectionResul
```

Case_L032.G1.sam

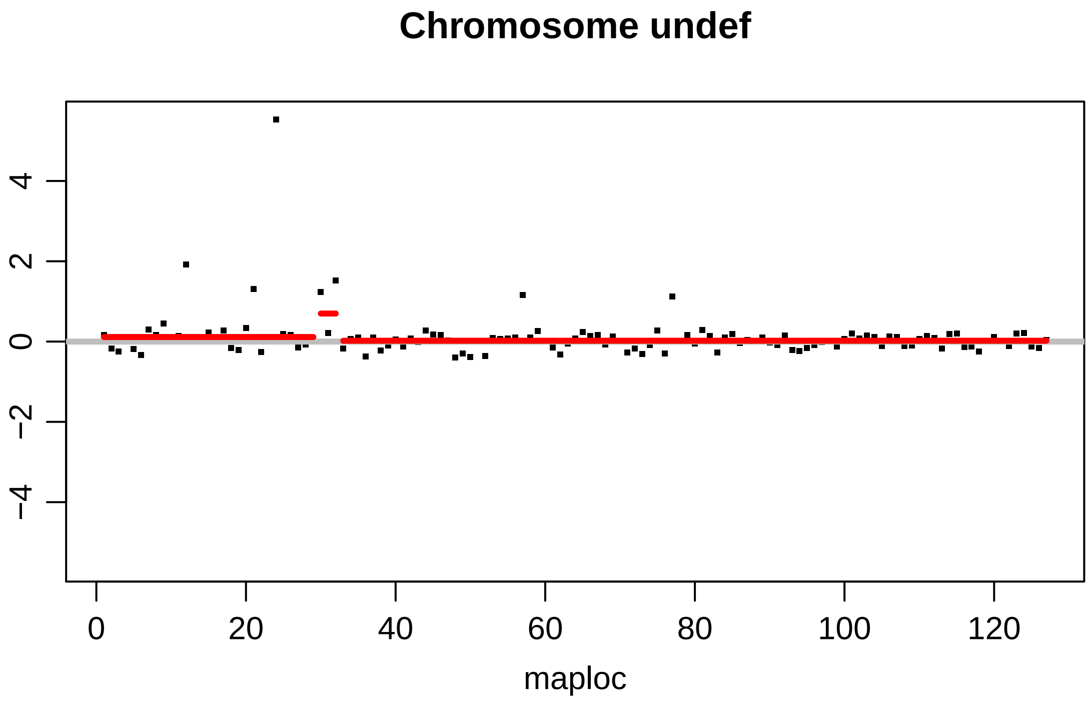


```
## Segplot might not work because of special characters in the sample names. Use only A-Z,a-z and 0-9!
##  There is a hidden function cn.mops:::.replaceNames that replaces the names in the "CNVDetectionResul
```

Case_L033.G1.sam

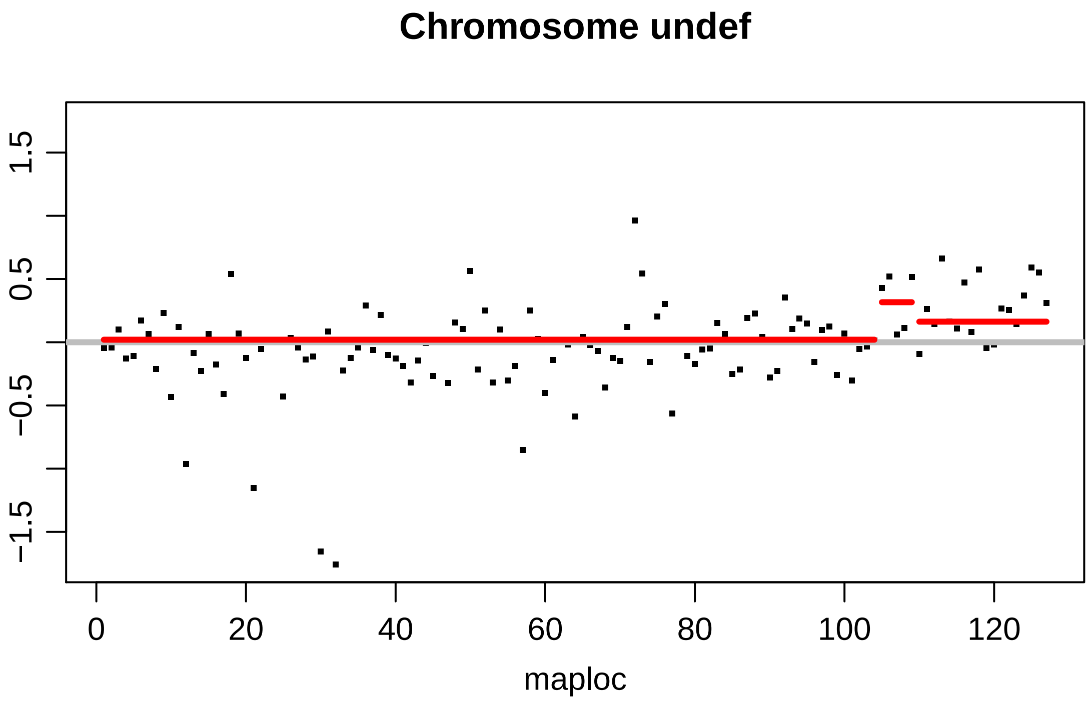

```
## Segplot might not work because of special characters in the sample names. Use only A-Z,a-z and 0-9!
##  There is a hidden function cn.mops:::.replaceNames that replaces the names in the "CNVDetectionResul
```

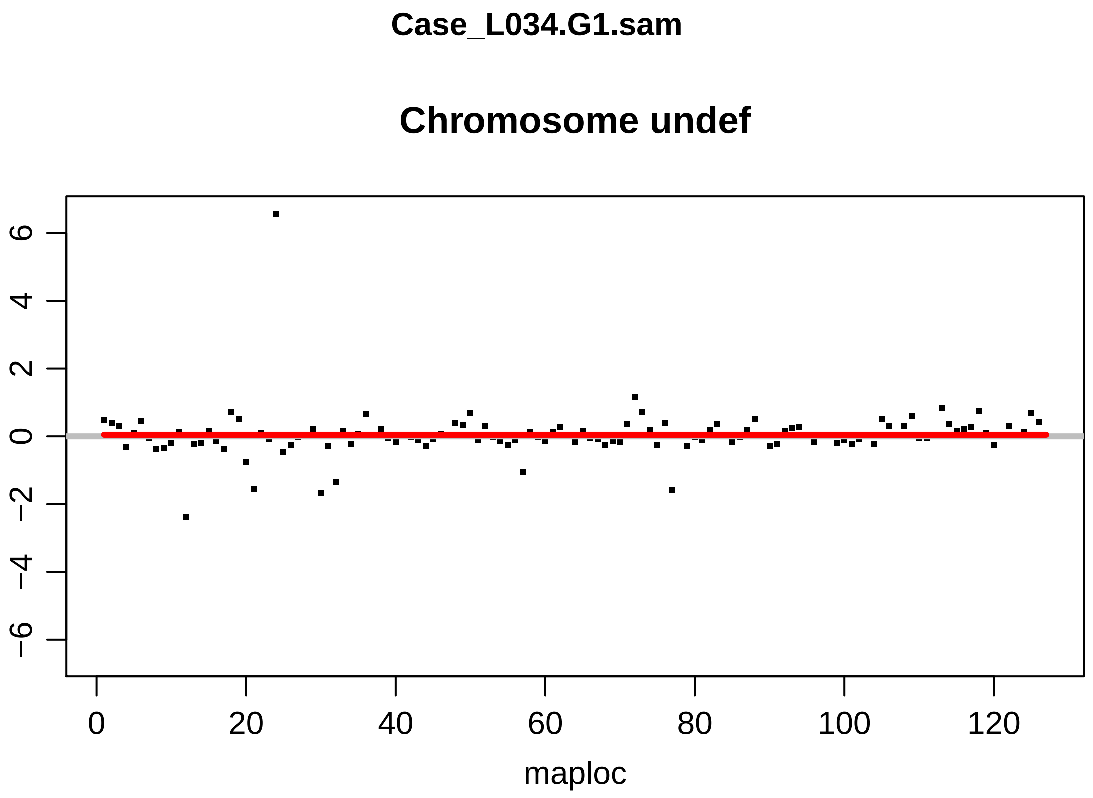


```
## Segplot might not work because of special characters in the sample names. Use only A-Z,a-z and 0-9!
##  There is a hidden function cn.mops:::.replaceNames that replaces the names in the "CNVDetectionResul
```

Case_L035.G1.sam

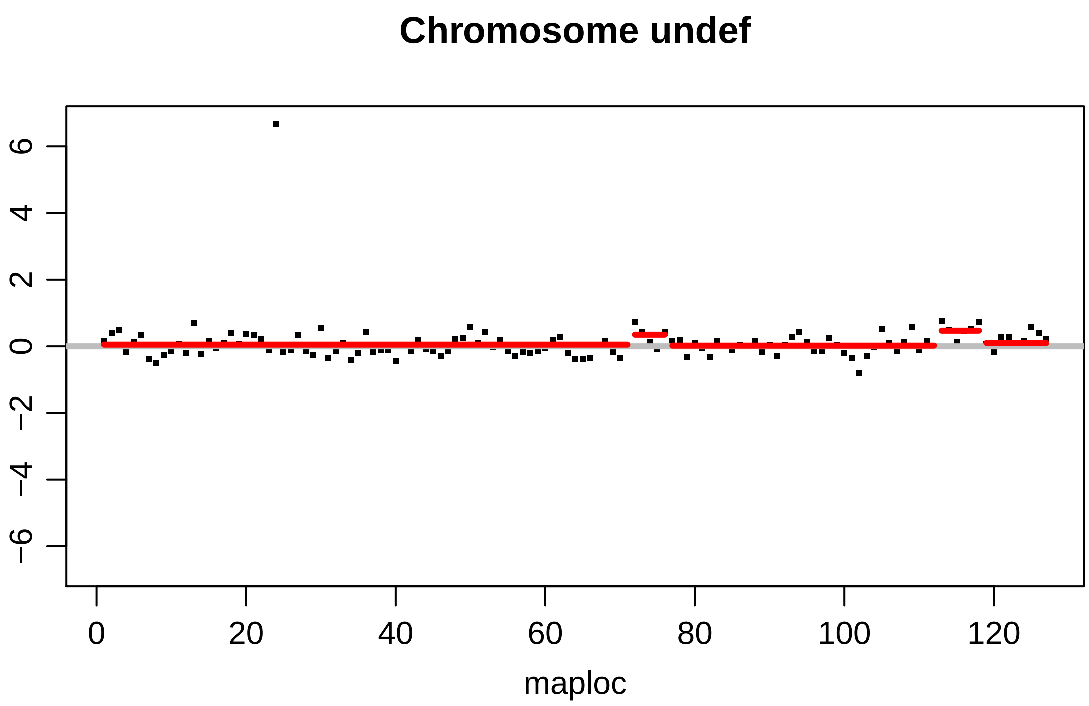


```
## Segplot might not work because of special characters in the sample names. Use only A-Z,a-z and 0-9!
##  There is a hidden function cn.mops:::.replaceNames that replaces the names in the "CNVDetectionResu[illegible]
```

Case_L036.G1.sam

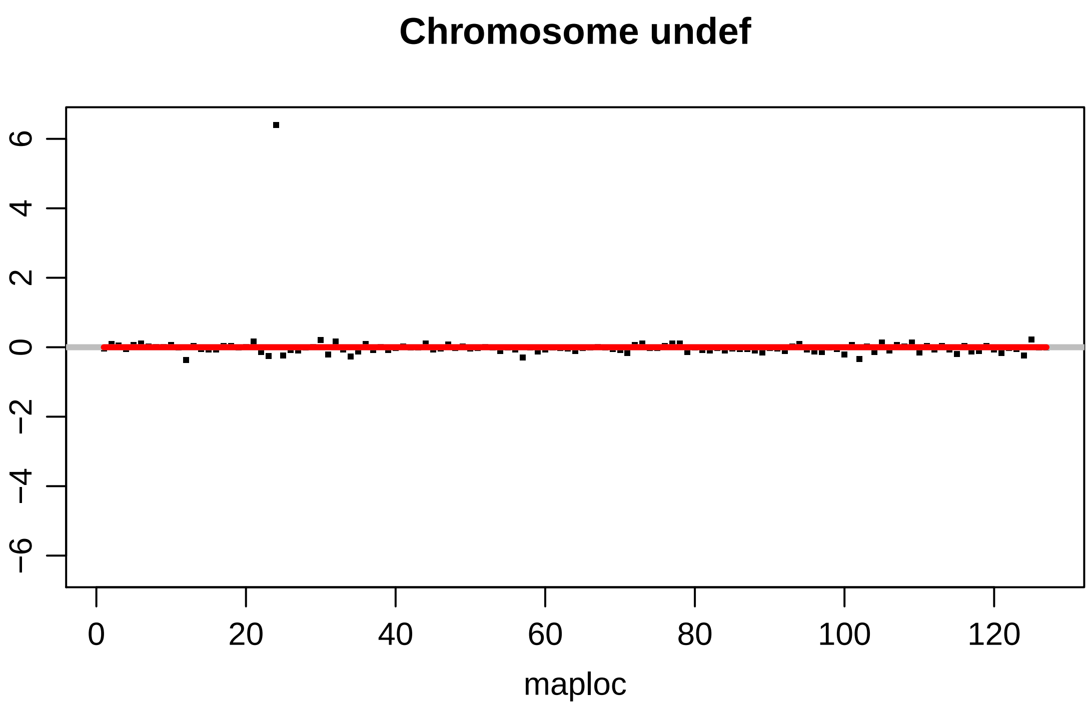

```
## Segplot might not work because of special characters in the sample names. Use only A-Z,a-z and 0-9!
##  There is a hidden function cn.mops:::.replaceNames that replaces the names in the "CNVDetectionResul
```

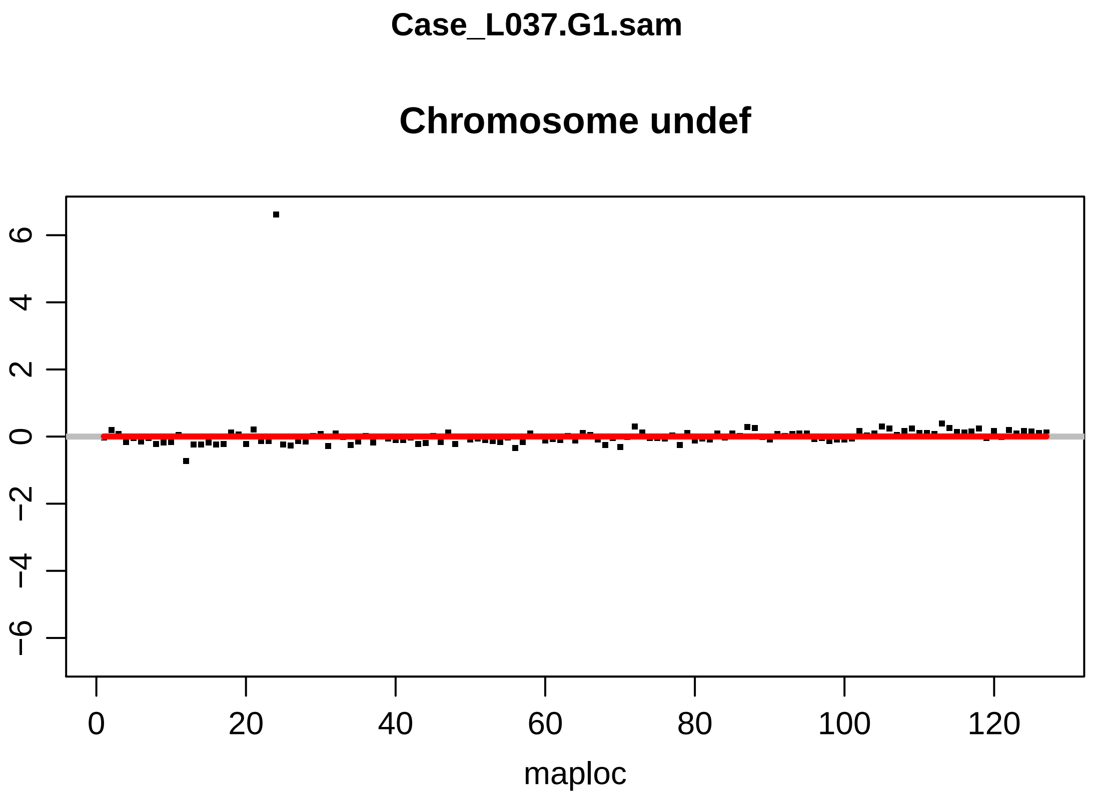


```
## Segplot might not work because of special characters in the sample names. Use only A-Z,a-z and 0-9!
##  There is a hidden function cn.mops:::.replaceNames that replaces the names in the "CNVDetectionResul
```

Case_L038.G1.sam

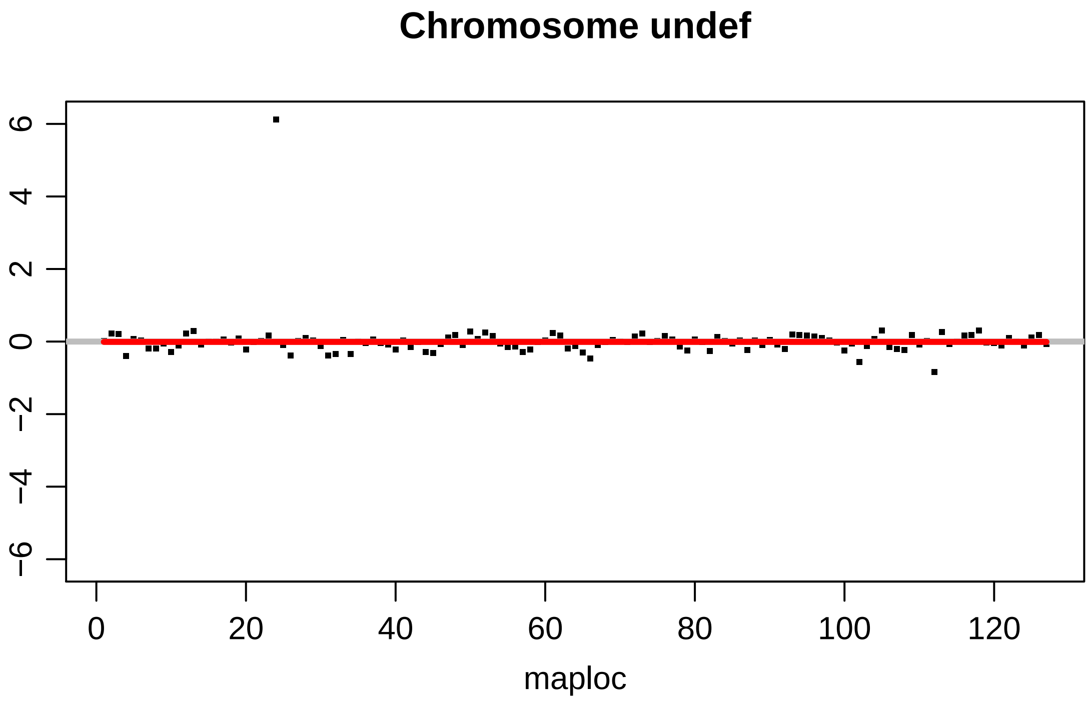


```
## Segplot might not work because of special characters in the sample names. Use only A-Z,a-z and 0-9!
##  There is a hidden function cn.mops:::.replaceNames that replaces the names in the "CNVDetectionResul
```

Case_L039.G1.sam

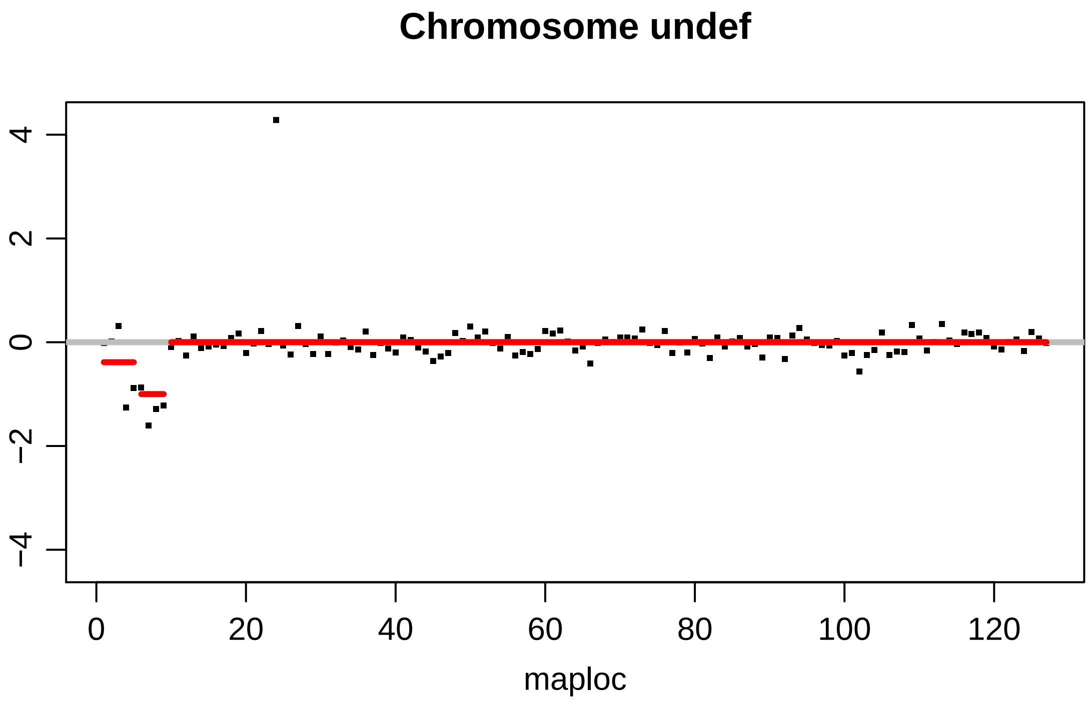

```
## Segplot might not work because of special characters in the sample names. Use only A-Z,a-z and 0-9!
##  There is a hidden function cn.mops:::.replaceNames that replaces the names in the "CNVDetectionResul
```

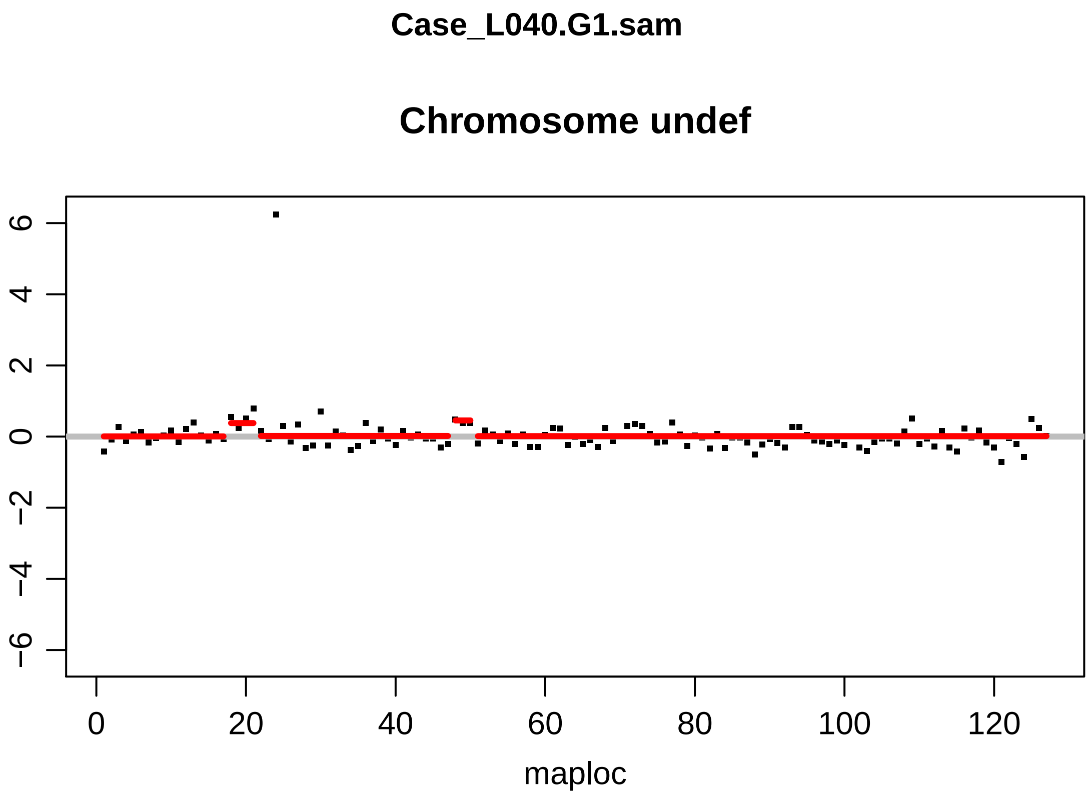


```
## Segplot might not work because of special characters in the sample names. Use only A-Z,a-z and 0-9!
##  There is a hidden function cn.mops:::.replaceNames that replaces the names in the "CNVDetectionResul
```

Case_L041.G1.sam

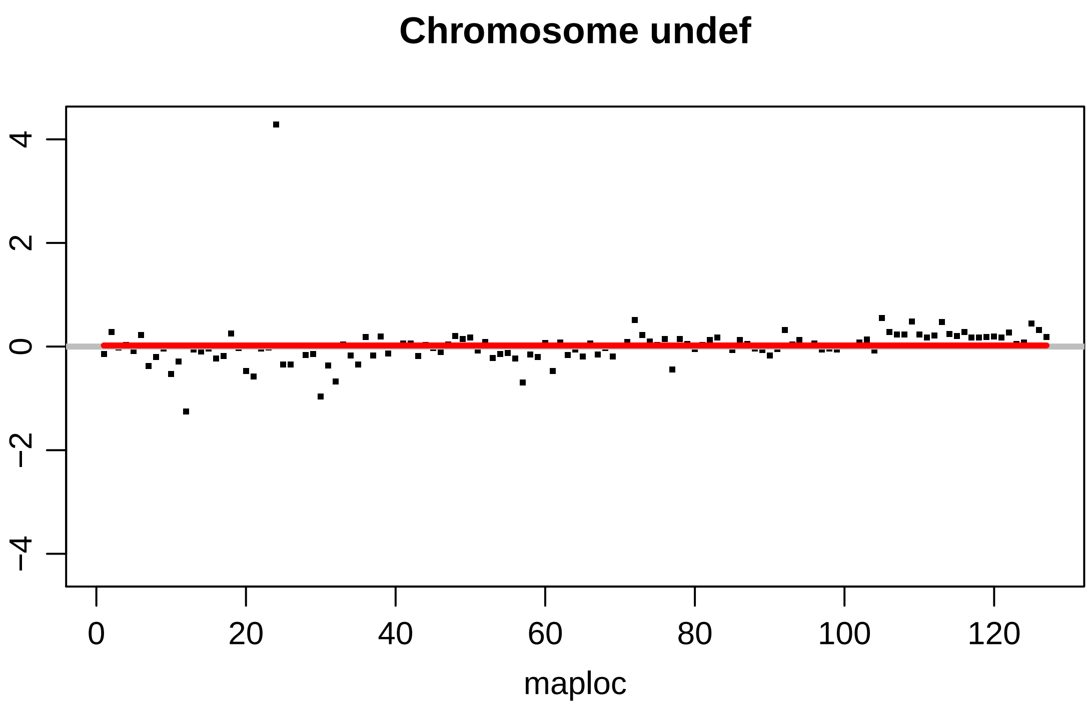


```
## Segplot might not work because of special characters in the sample names. Use only A-Z,a-z and 0-9!
##  There is a hidden function cn.mops:::.replaceNames that replaces the names in the "CNVDetectionResul
```

Case_L042.G1.sam

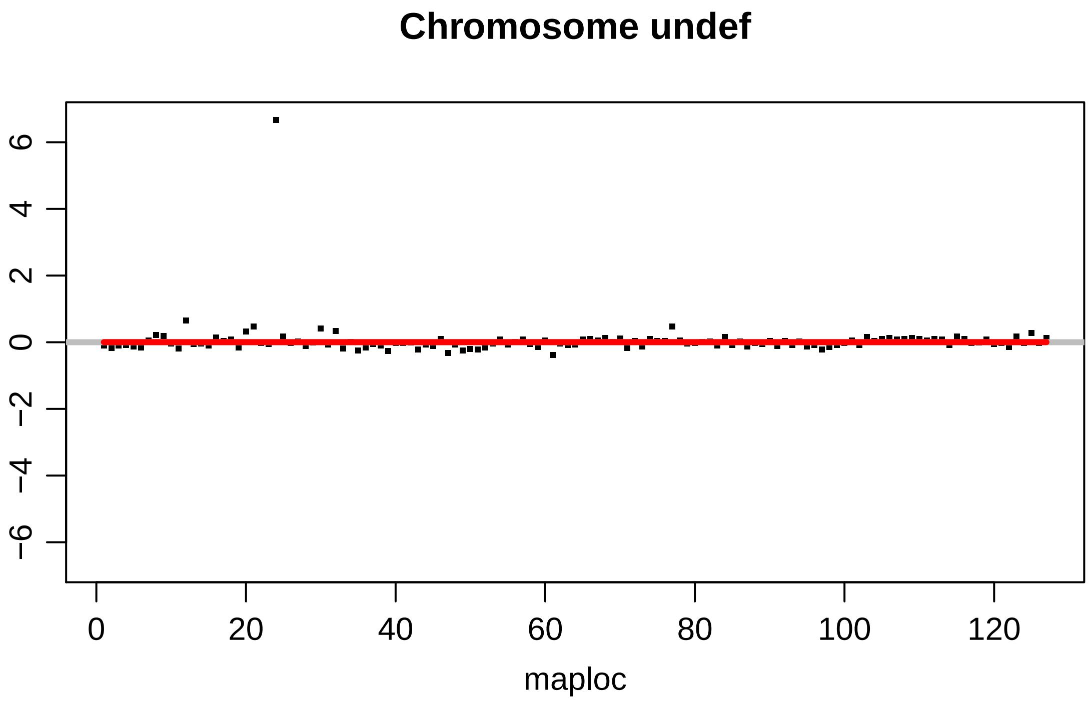

```
## Segplot might not work because of special characters in the sample names. Use only A-Z,a-z and 0-9!
##  There is a hidden function cn.mops:::.replaceNames that replaces the names in the "CNVDetectionResul
```

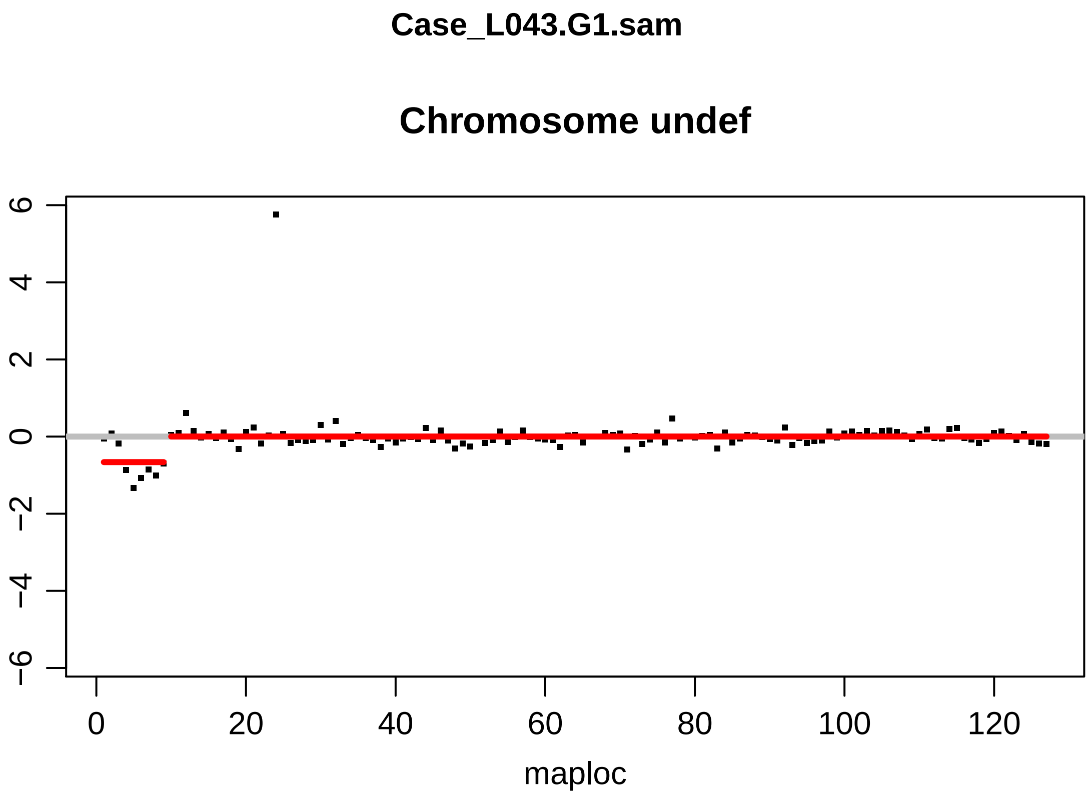


```
## Segplot might not work because of special characters in the sample names. Use only A-Z,a-z and 0-9!
##  There is a hidden function cn.mops:::.replaceNames that replaces the names in the "CNVDetectionResul
```

Case_L044.G1.sam

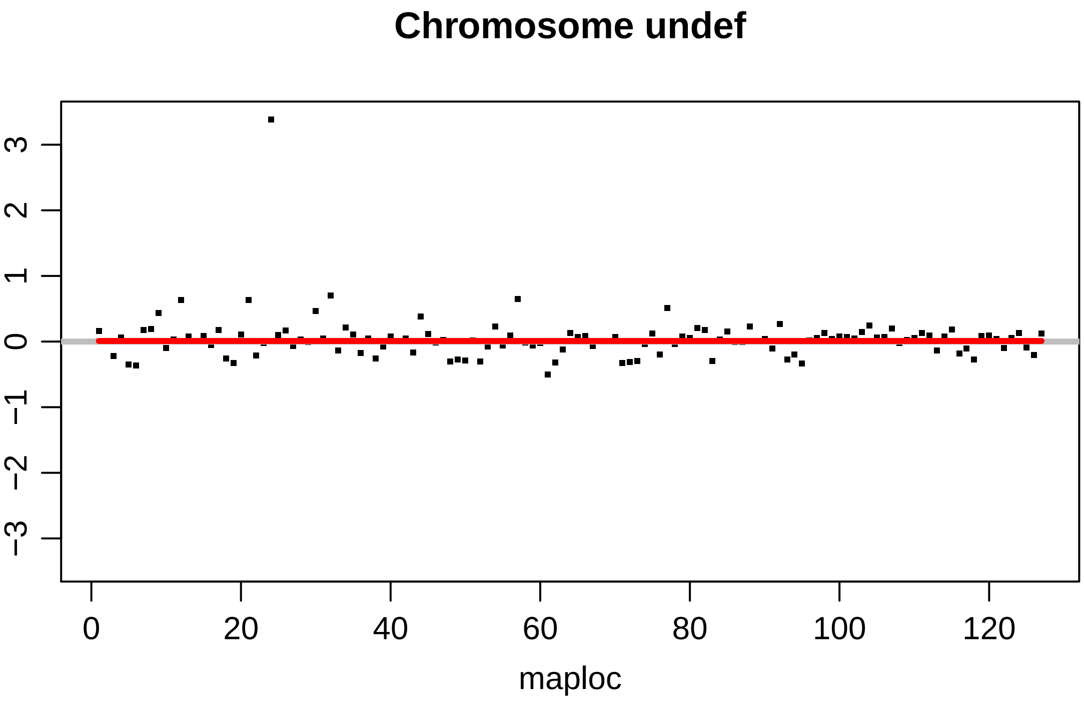


```
## Segplot might not work because of special characters in the sample names. Use only A-Z,a-z and 0-9!
##  There is a hidden function cn.mops:::.replaceNames that replaces the names in the "CNVDetectionResul
```

Case_L045.G1.sam

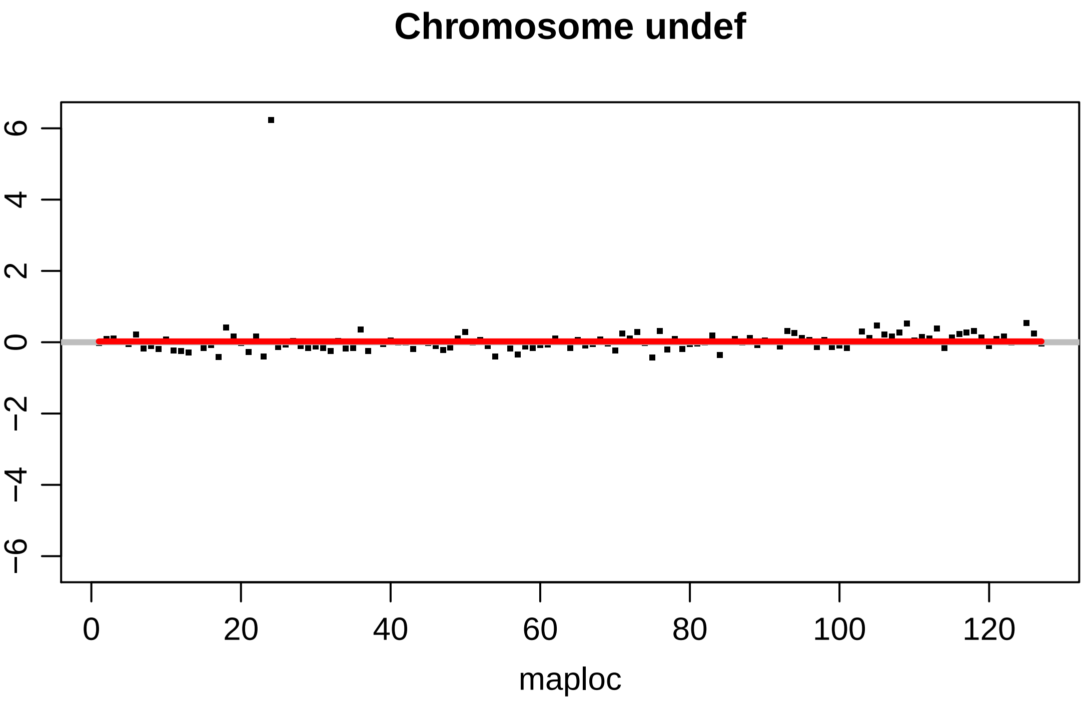

```
## Segplot might not work because of special characters in the sample names. Use only A-Z,a-z and 0-9!
##  There is a hidden function cn.mops:::.replaceNames that replaces the names in the "CNVDetectionResul
```

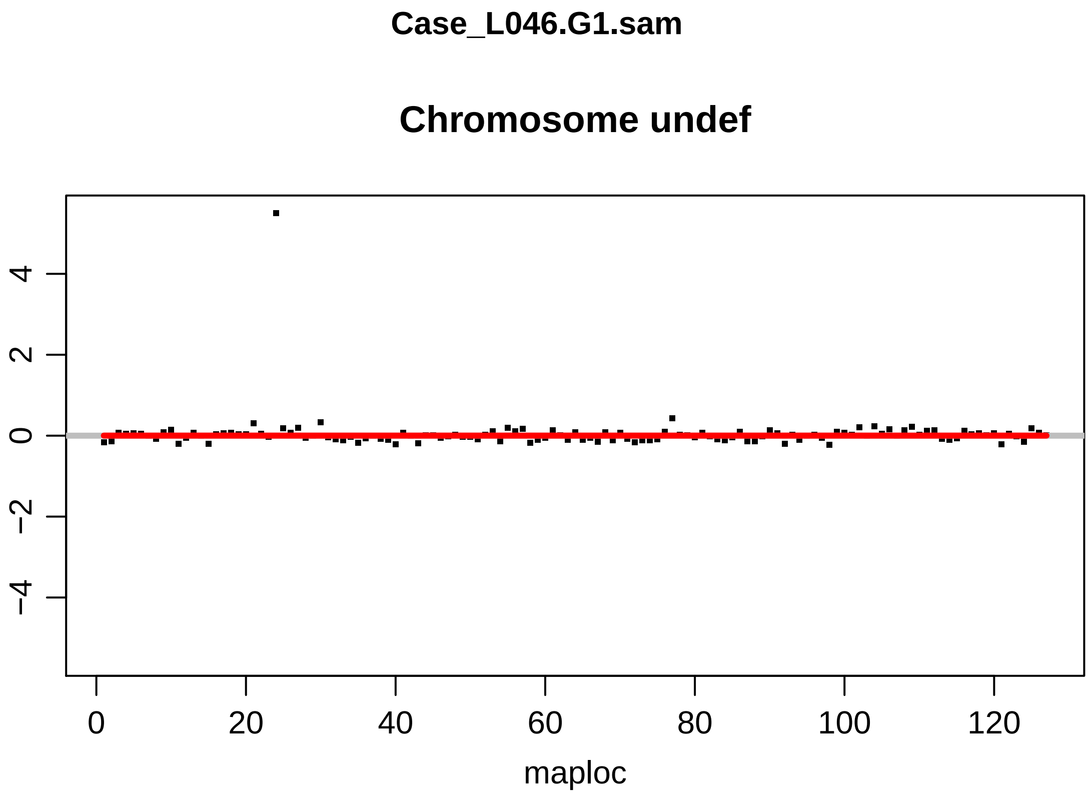


```
##
## CNV regions:
## GRanges object with 4 ranges and 47 metadata columns:
##       seqnames    ranges strand | Case_CONTROL.saliva.sam Case_L001.G1.sam
##          <Rle> <IRanges>  <Rle> |                <factor>         <factor>
##   [1]    undef  [ 6,  9]      * |                     CN2              CN2
##   [2]    undef  [18, 21]      * |                     CN2              CN2
##   [3]    undef  [30, 32]      * |                     CN2              CN2
##   [4]    undef  [48, 50]      * |                     CN2              CN2
##       Case_L002.G1.sam Case_L003.G1.sam Case_L004.G1.sam Case_L005.G1.sam
##               <factor>         <factor>         <factor>         <factor>
##   [1]              CN2              CN2              CN2              CN2
##   [2]              CN2              CN2              CN2              CN2
##   [3]              CN2              CN2              CN2              CN2
##   [4]              CN2              CN2              CN2              CN2
##       Case_L006.G1.sam Case_L007.G1.sam Case_L008.G1.sam Case_L009.G1.sam
##               <factor>         <factor>         <factor>         <factor>
##   [1]              CN2              CN2              CN2              CN1
##   [2]              CN2              CN2              CN2              CN2
##   [3]              CN2              CN2              CN2              CN2
##   [4]              CN3              CN2              CN2              CN2
##       Case_L011.G1.sam Case_L012.G1.sam Case_L013.G1.sam Case_L014.G1.sam
##               <factor>         <factor>         <factor>         <factor>
##   [1]              CN2              CN2              CN2              CN2
##   [2]              CN2              CN2              CN2              CN2
```

```
##    [3]                CN2                CN2                CN2                CN2
##    [4]                CN2                CN2                CN2                CN2
##        Case_L015.G1.sam Case_L016.G1.sam Case_L017.G1.sam Case_L018.G1.sam
##                <factor>           <factor>           <factor>           <factor>
##    [1]                CN2                CN2                CN2                CN2
##    [2]                CN2                CN2                CN2                CN2
##    [3]                CN2                CN2                CN2                CN2
##    [4]                CN2                CN2                CN2                CN2
##        Case_L019.G1.sam Case_L020.G1.sam Case_L021.G1.sam Case_L022.G1.sam
##                <factor>           <factor>           <factor>           <factor>
##    [1]                CN2                CN2                CN2                CN2
##    [2]                CN3                CN2                CN2                CN2
##    [3]                CN2                CN2                CN2                CN2
##    [4]                CN2                CN2                CN2                CN2
##        Case_L023.G1.sam Case_L024.G1.sam Case_L025.G1.sam Case_L026.G1.sam
##                <factor>           <factor>           <factor>           <factor>
##    [1]                CN2                CN1                CN2                CN2
##    [2]                CN2                CN2                CN2                CN2
##    [3]                CN2                CN2                CN2                CN3
##    [4]                CN2                CN2                CN2                CN2
##        Case_L027.G1.sam Case_L028.G1.sam Case_L029.G1.sam Case_L030.G1.sam
##                <factor>           <factor>           <factor>           <factor>
##    [1]                CN2                CN2                CN1                CN2
##    [2]                CN2                CN2                CN2                CN2
##    [3]                CN2                CN2                CN2                CN2
##    [4]                CN2                CN2                CN2                CN2
##        Case_L031.G1.sam Case_L032.G1.sam Case_L033.G1.sam Case_L034.G1.sam
##                <factor>           <factor>           <factor>           <factor>
##    [1]                CN2                CN2                CN2                CN2
##    [2]                CN2                CN2                CN2                CN2
##    [3]                CN2                CN3                CN2                CN2
##    [4]                CN2                CN2                CN2                CN2
##        Case_L035.G1.sam Case_L036.G1.sam Case_L037.G1.sam Case_L038.G1.sam
##                <factor>           <factor>           <factor>           <factor>
##    [1]                CN2                CN2                CN2                CN2
##    [2]                CN2                CN2                CN2                CN2
##    [3]                CN2                CN2                CN2                CN2
##    [4]                CN2                CN2                CN2                CN2
##        Case_L039.G1.sam Case_L040.G1.sam Case_L041.G1.sam Case_L042.G1.sam
##                <factor>           <factor>           <factor>           <factor>
##    [1]                CN1                CN2                CN2                CN2
##    [2]                CN2                CN2                CN2                CN2
##    [3]                CN2                CN2                CN2                CN2
##    [4]                CN2                CN2                CN2                CN2
##        Case_L043.G1.sam Case_L044.G1.sam Case_L045.G1.sam Case_L046.G1.sam
##                <factor>           <factor>           <factor>           <factor>
##    [1]                CN2                CN2                CN2                CN2
##    [2]                CN2                CN2                CN2                CN2
##    [3]                CN2                CN2                CN2                CN2
##    [4]                CN2                CN2                CN2                CN2
##        Case_L047.G1.sam
##                <factor>
##    [1]                CN2
##    [2]                CN2
```

```
##   [3]               CN2
##   [4]               CN2
##   -------
##   seqinfo: 1 sequence from an unspecified genome; no seqlengths
##
## Individual CNVs:
## GRanges object with 8 ranges and 4 metadata columns:
##       seqnames    ranges strand |       sampleName    median       mean
##          <Rle> <IRanges>  <Rle> |         <factor> <numeric>  <numeric>
##   [1]    undef  [48, 50]      * | Case_L006.G1.sam  0.5845758  0.5674124
##   [2]    undef  [ 6,  9]      * | Case_L009.G1.sam -0.9979046 -0.9977969
##   [3]    undef  [18, 21]      * | Case_L019.G1.sam  0.6215621  0.5683017
##   [4]    undef  [ 6,  9]      * | Case_L024.G1.sam -0.9815733 -0.9113304
##   [5]    undef  [30, 32]      * | Case_L026.G1.sam  0.8994016  0.6483700
##   [6]    undef  [ 6,  9]      * | Case_L029.G1.sam -0.9985869 -0.9986432
##   [7]    undef  [30, 32]      * | Case_L032.G1.sam  0.9936793  0.6973395
##   [8]    undef  [ 6,  9]      * | Case_L039.G1.sam -0.9999898 -0.9997927
##                CN
##       <character>
##   [1]         CN3
##   [2]         CN1
##   [3]         CN3
##   [4]         CN1
##   [5]         CN3
##   [6]         CN1
##   [7]         CN3
##   [8]         CN1
##   -------
##   seqinfo: 1 sequence from an unspecified genome; no seqlengths
## [1] "/Users/gdemidov/Downloads/doc/Run_SN1_41_fin_05_qc.xls"

## Normalizing...

## Starting local modeling, please be patient...

## Reference sequence:  undef

## Starting segmentation algorithm...

## Using "fastseg" for segmentation.

## [1] ""
## [1] "/Users/gdemidov/Downloads/doc/Run_SN1_41_fin_05_qc.xls"
## [1] ""

## Segplot might not work because of special characters in the sample names. Use only A-Z,a-z and 0-9!
##  There is a hidden function cn.mops:::.replaceNames that replaces the names in the "CNVDetectionResul
```

Case_L047.G1.sam

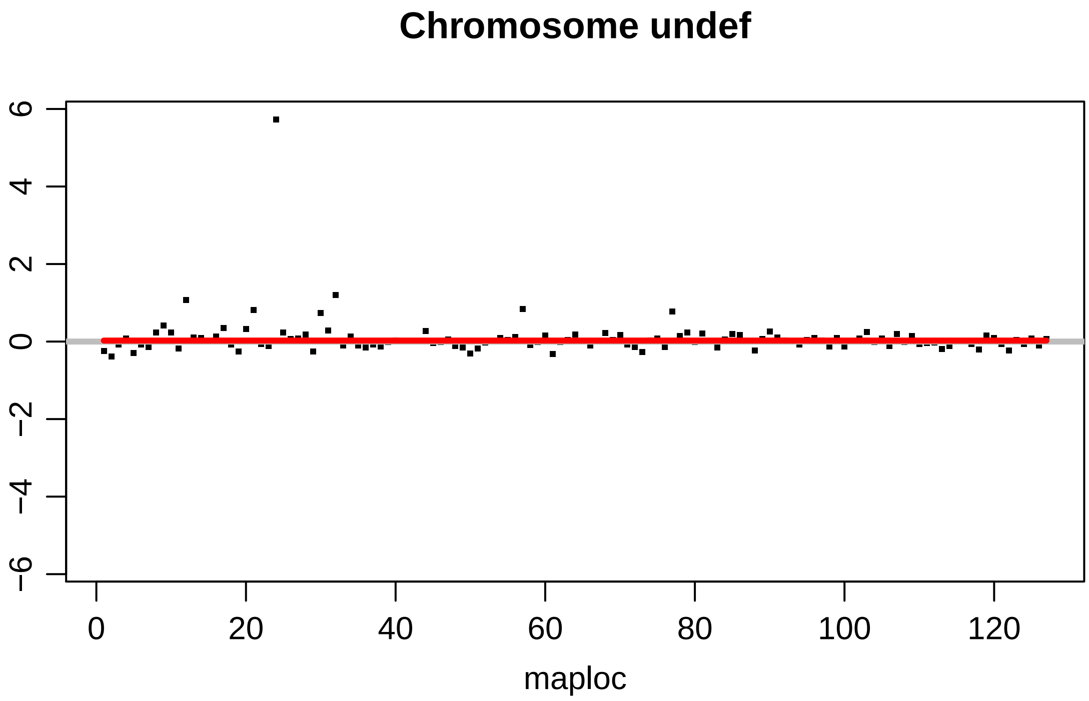


```
## Segplot might not work because of special characters in the sample names. Use only A-Z,a-z and 0-9!
##  There is a hidden function cn.mops:::.replaceNames that replaces the names in the "CNVDetectionResul
```

L_R_2014_03_03_21_49_55_Sequoia_SN1.41.Neonatal_29.10.2014_Hi.Q_non.mark_

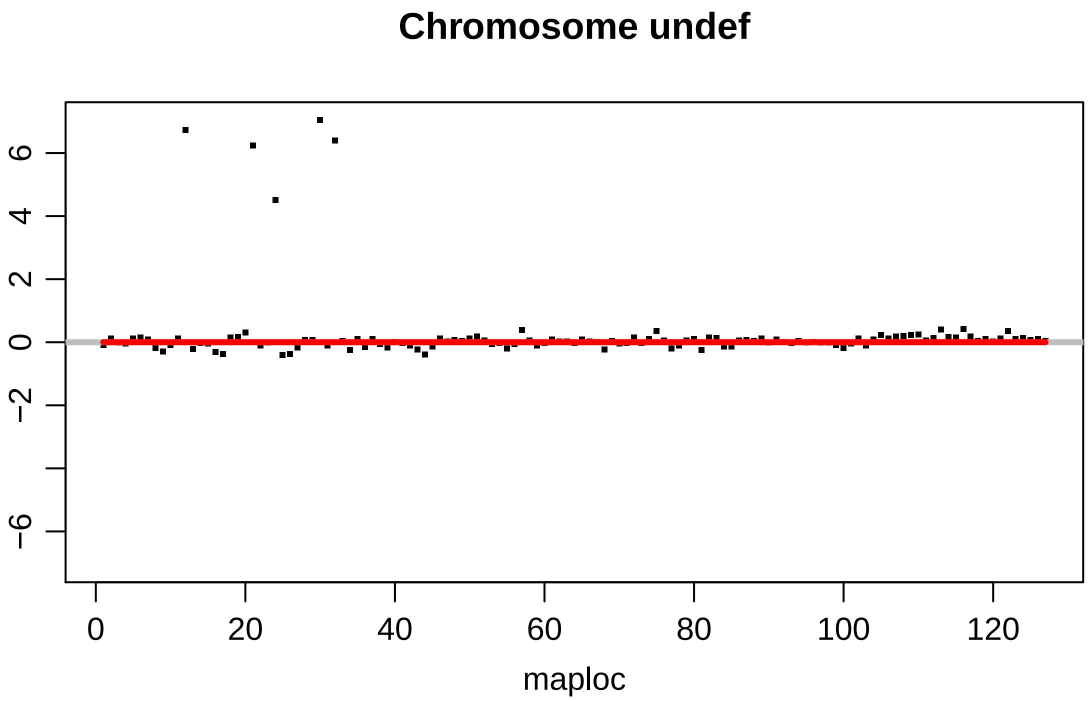

```
## Segplot might not work because of special characters in the sample names. Use only A-Z,a-z and 0-9!
##  There is a hidden function cn.mops:::.replaceNames that replaces the names in the "CNVDetectionResul
```

2_R_2014_03_03_21_49_55_Sequoia_SN1.41.Neonatal_29.10.2014_Hi.Q_non.mark_

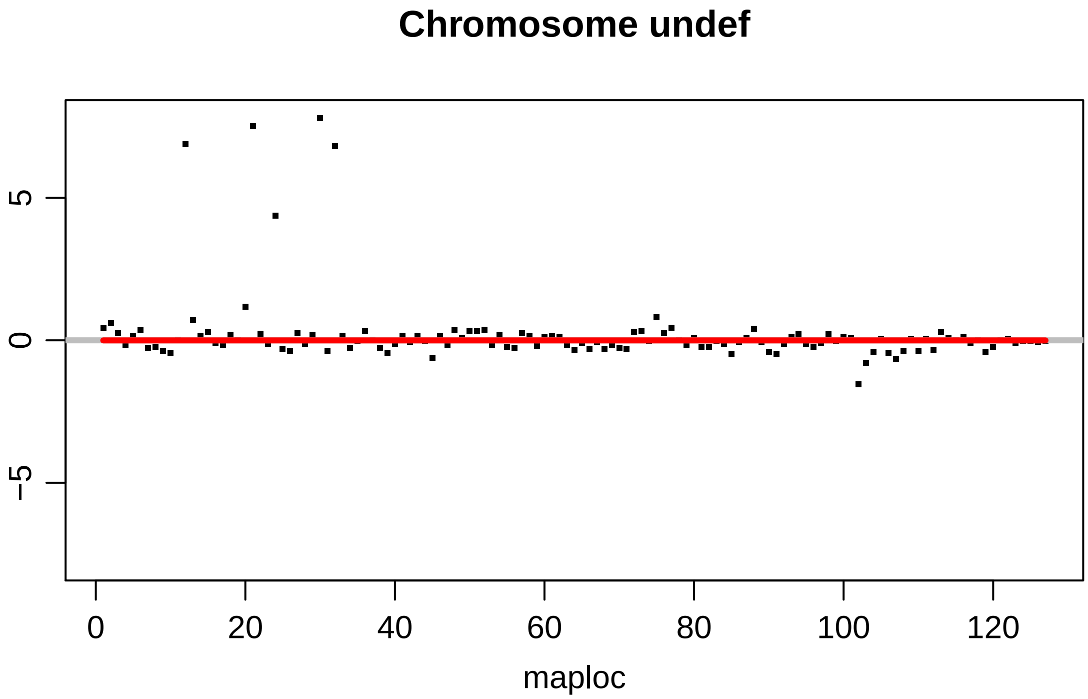


```
## Segplot might not work because of special characters in the sample names. Use only A-Z,a-z and 0-9!
##  There is a hidden function cn.mops:::.replaceNames that replaces the names in the "CNVDetectionResul
```

3_R_2014_03_03_21_49_55_Sequoia_SN1.41.Neonatal_29.10.2014_Hi.Q_non.mark_

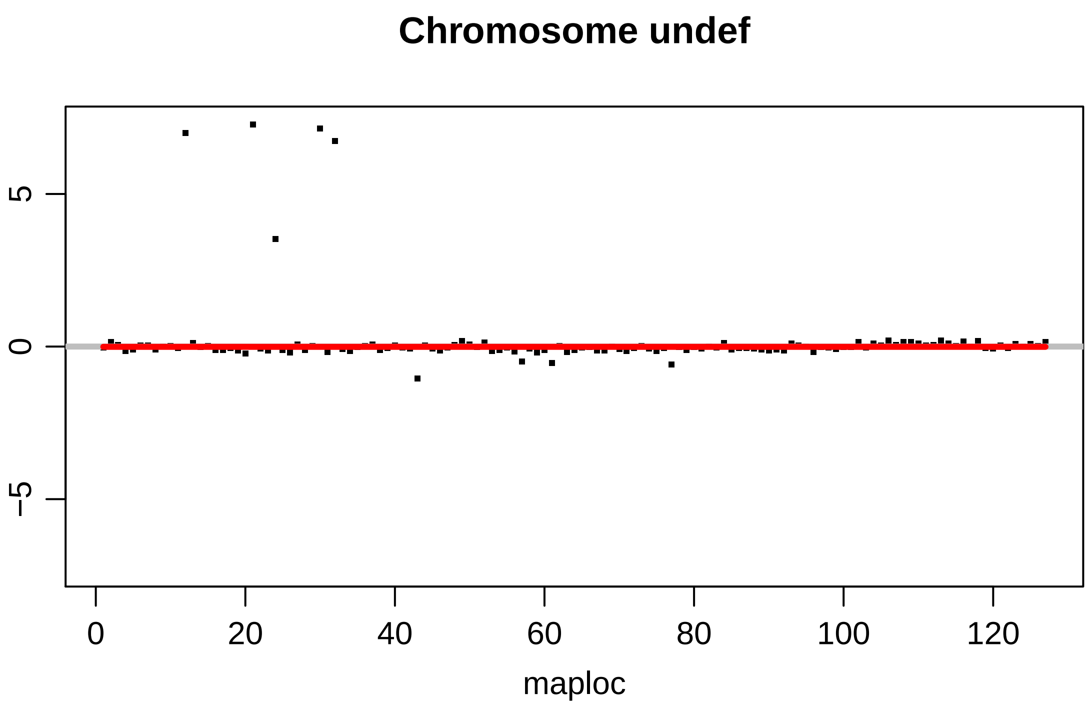


```
## Segplot might not work because of special characters in the sample names. Use only A-Z,a-z and 0-9!
##  There is a hidden function cn.mops:::.replaceNames that replaces the names in the "CNVDetectionResul
```

4_R_2014_03_03_21_49_55_Sequoia_SN1.41.Neonatal_29.10.2014_Hi.Q_non.mark_

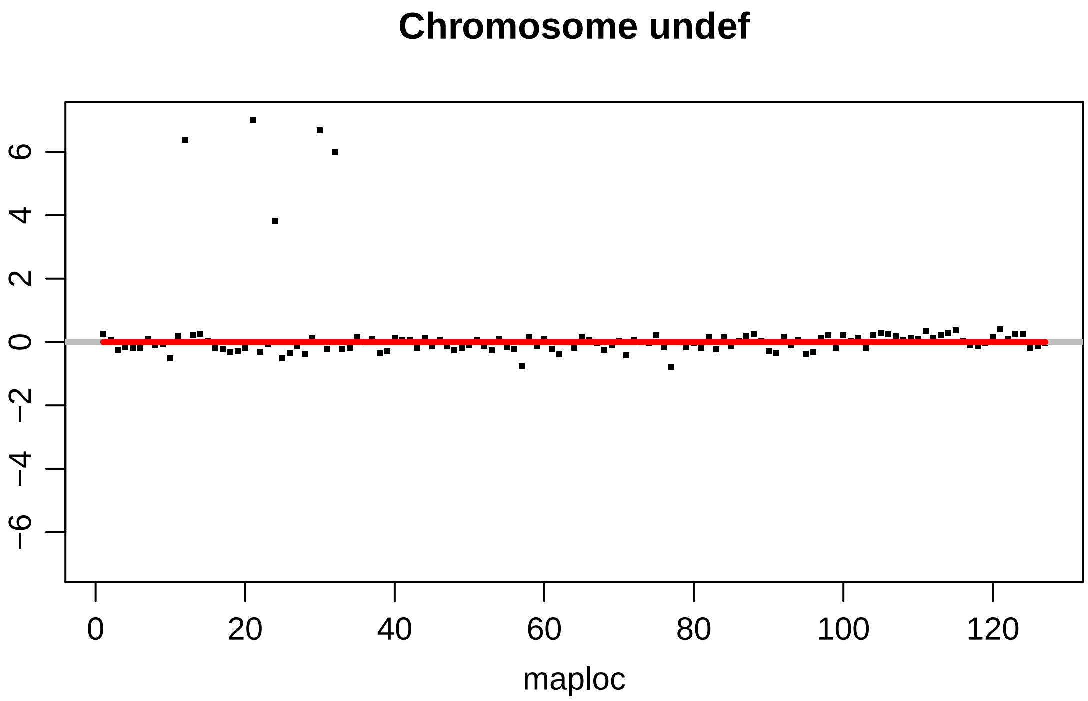

```
## Segplot might not work because of special characters in the sample names. Use only A-Z,a-z and 0-9!
##  There is a hidden function cn.mops:::.replaceNames that replaces the names in the "CNVDetectionResul
```

5_R_2014_03_03_21_49_55_Sequoia_SN1.41.Neonatal_29.10.2014_Hi.Q_non.mark_

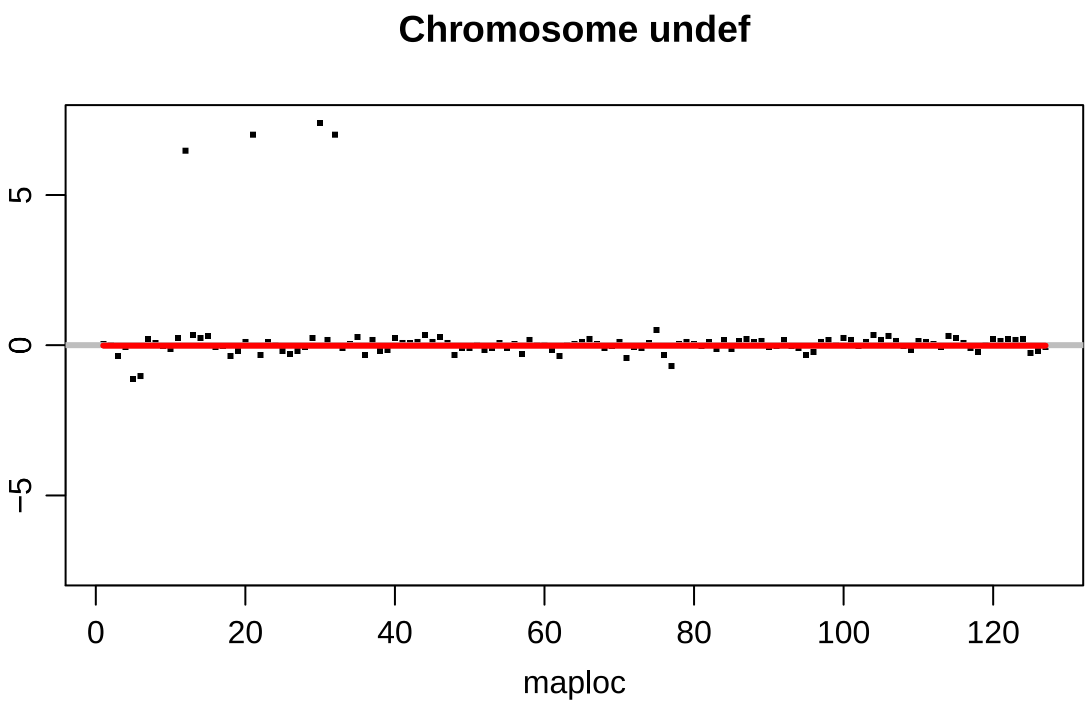


```
## Segplot might not work because of special characters in the sample names. Use only A-Z,a-z and 0-9!
##  There is a hidden function cn.mops:::.replaceNames that replaces the names in the "CNVDetectionResul
```

5_R_2014_03_03_21_49_55_Sequoia_SN1.41.Neonatal_29.10.2014_Hi.Q_non.mark_

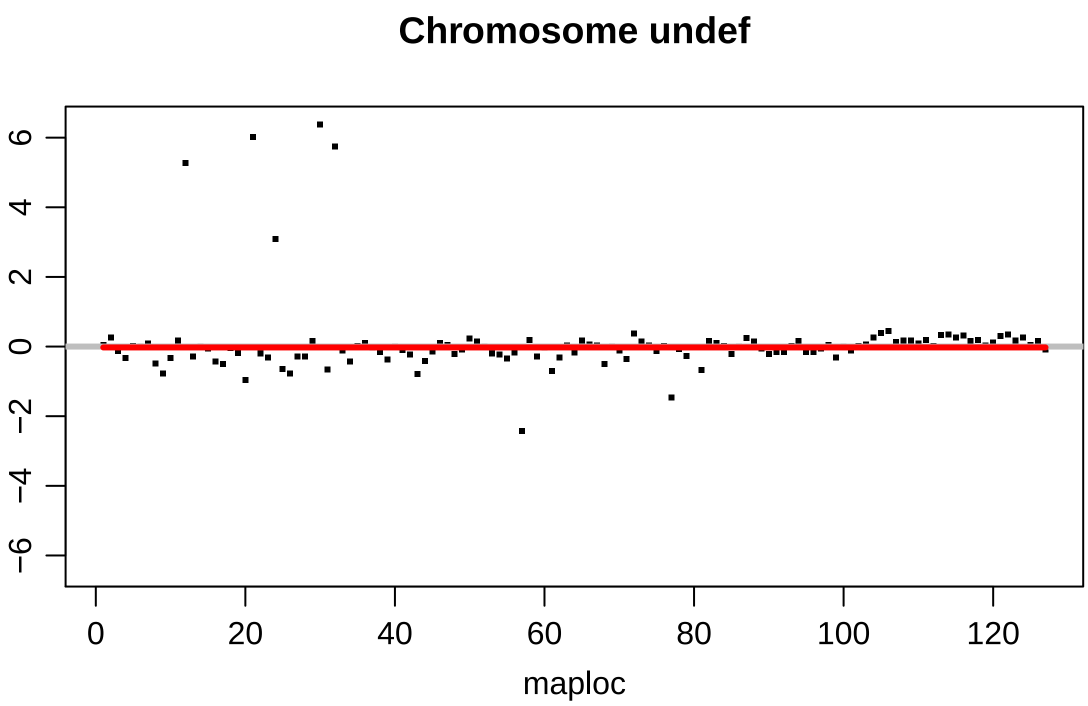


```
## Segplot might not work because of special characters in the sample names. Use only A-Z,a-z and 0-9!
##  There is a hidden function cn.mops:::.replaceNames that replaces the names in the "CNVDetectionResul
```

7_R_2014_03_03_21_49_55_Sequoia_SN1.41.Neonatal_29.10.2014_Hi.Q_non.mark_

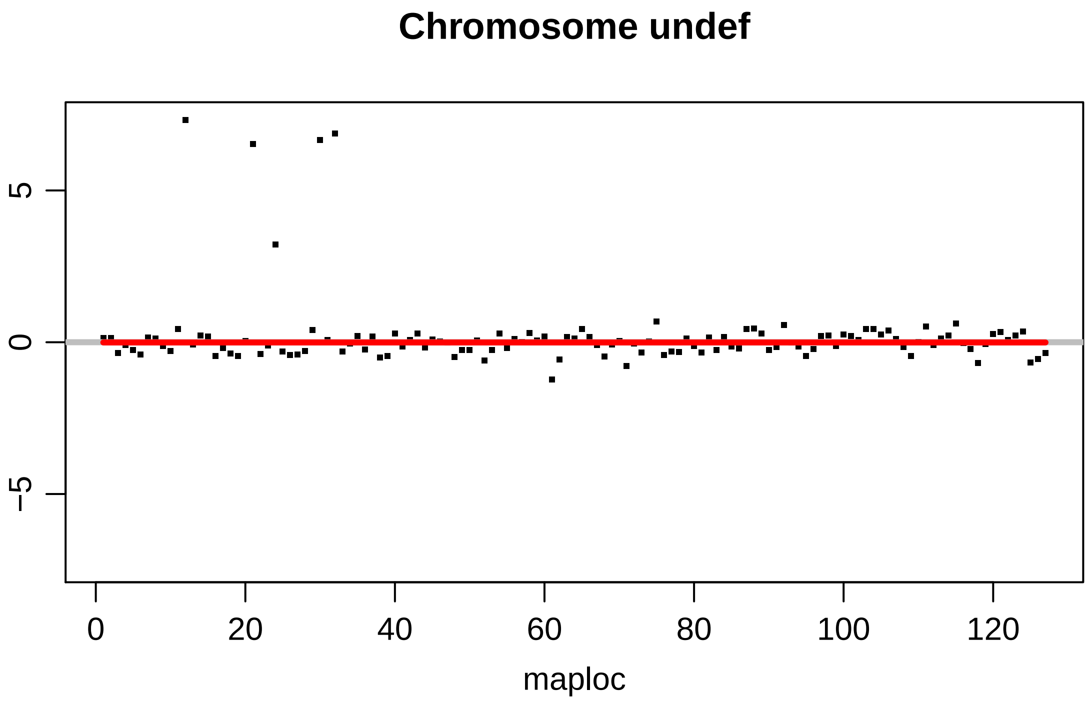

```
## Segplot might not work because of special characters in the sample names. Use only A-Z,a-z and 0-9!
##  There is a hidden function cn.mops:::.replaceNames that replaces the names in the "CNVDetectionResul
```

3_R_2014_03_03_21_49_55_Sequoia_SN1.41.Neonatal_29.10.2014_Hi.Q_non.mark_

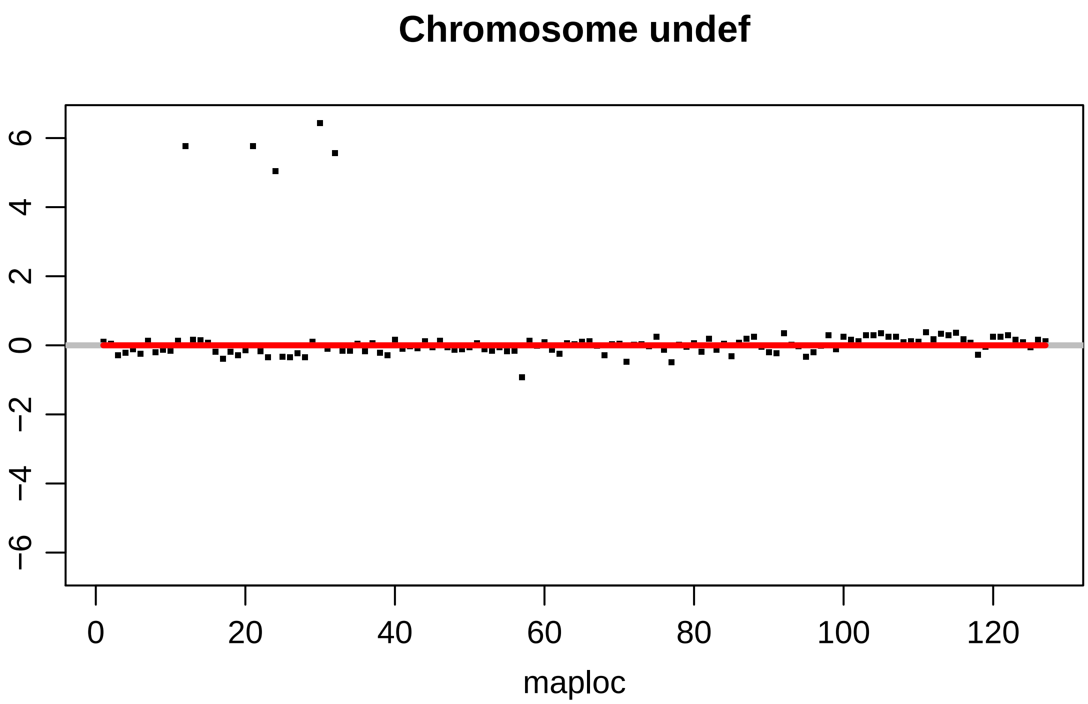


```
## Segplot might not work because of special characters in the sample names. Use only A-Z,a-z and 0-9!
##  There is a hidden function cn.mops:::.replaceNames that replaces the names in the "CNVDetectionResul
```

)_R_2014_03_03_21_49_55_Sequoia_SN1.41.Neonatal_29.10.2014_Hi.Q_non.mark_

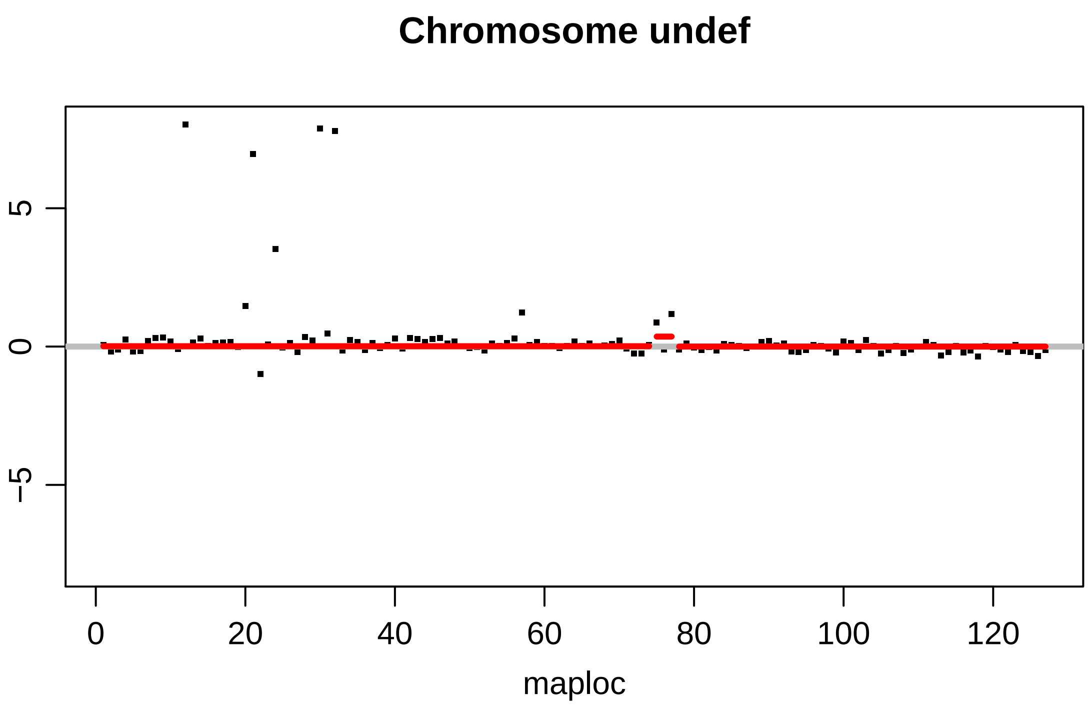


```
## Segplot might not work because of special characters in the sample names. Use only A-Z,a-z and 0-9!
##  There is a hidden function cn.mops:::.replaceNames that replaces the names in the "CNVDetectionResul
```

)_R_2014_03_03_21_49_55_Sequoia_SN1.41.Neonatal_29.10.2014_Hi.Q_non.mark_

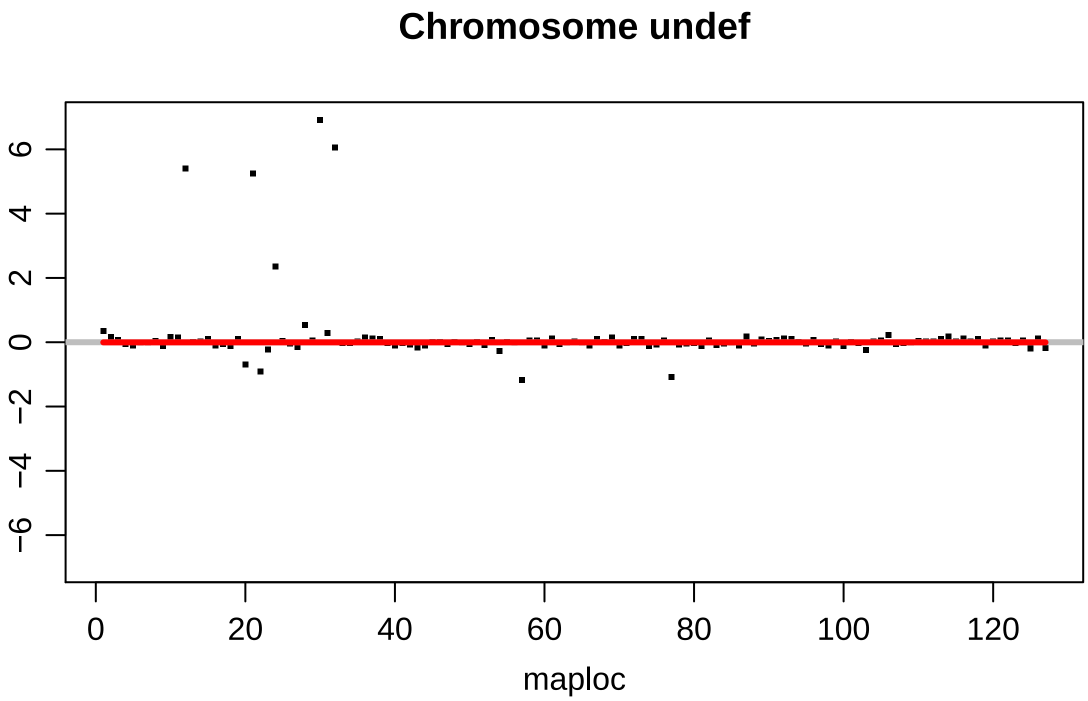

```
## Segplot might not work because of special characters in the sample names. Use only A-Z,a-z and 0-9!
##  There is a hidden function cn.mops:::.replaceNames that replaces the names in the "CNVDetectionResul
```

2_R_2014_03_03_21_49_55_Sequoia_SN1.41.Neonatal_29.10.2014_Hi.Q_non.mark_

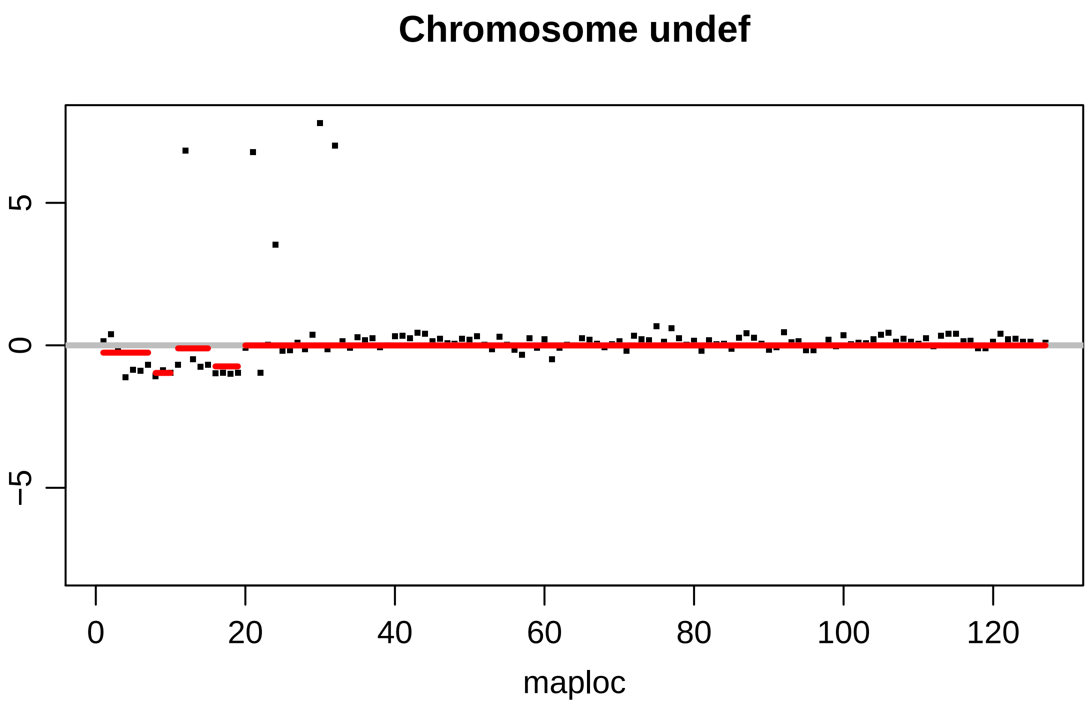


```
## Segplot might not work because of special characters in the sample names. Use only A-Z,a-z and 0-9!
##  There is a hidden function cn.mops:::.replaceNames that replaces the names in the "CNVDetectionResul
```

5_R_2014_03_03_21_49_55_Sequoia_SN1.41.Neonatal_29.10.2014_Hi.Q_non.mark_

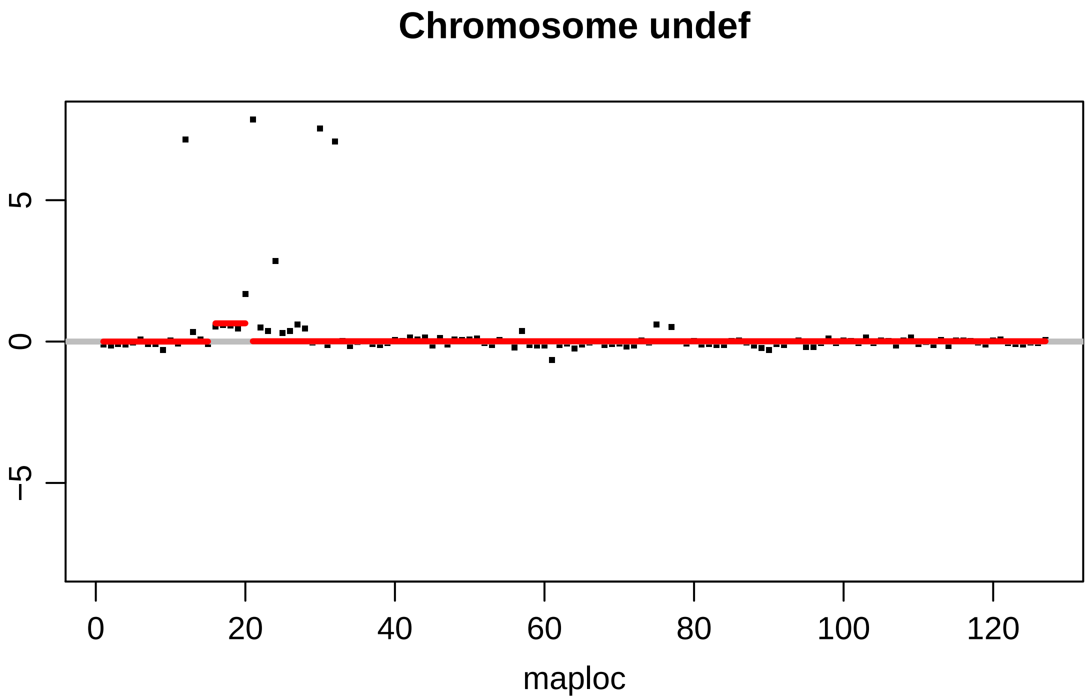


```
## Segplot might not work because of special characters in the sample names. Use only A-Z,a-z and 0-9!
##  There is a hidden function cn.mops:::.replaceNames that replaces the names in the "CNVDetectionResul
```

5_R_2014_03_03_21_49_55_Sequoia_SN1.41.Neonatal_29.10.2014_Hi.Q_non.mark_

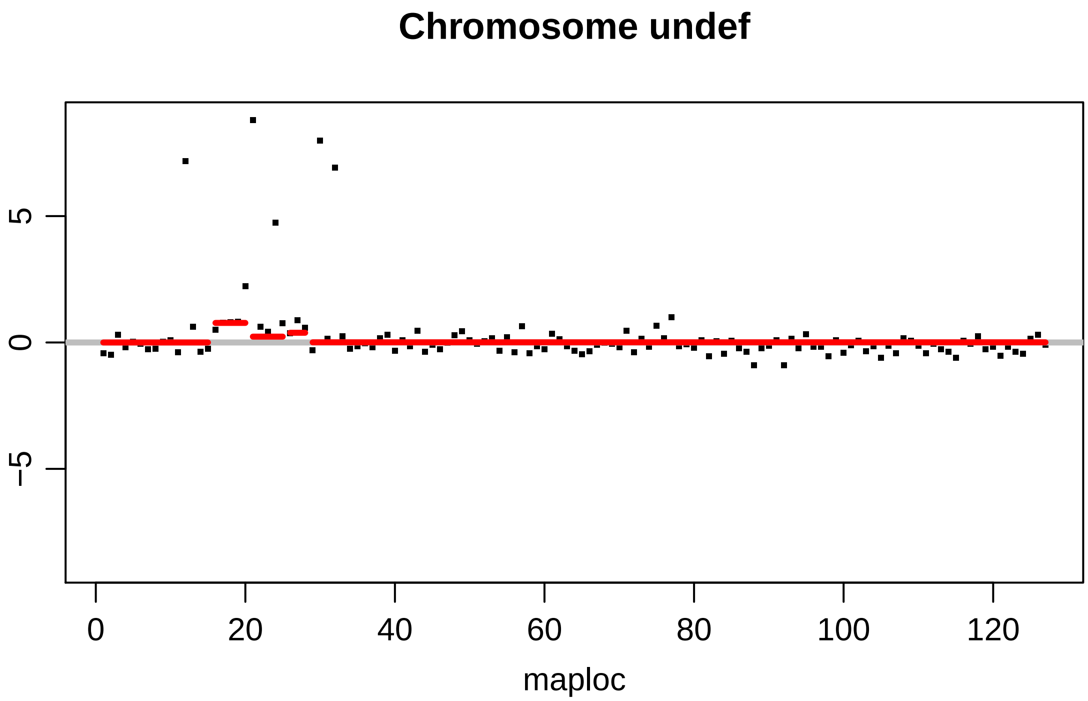

```
## Segplot might not work because of special characters in the sample names. Use only A-Z,a-z and 0-9!
##  There is a hidden function cn.mops:::.replaceNames that replaces the names in the "CNVDetectionResul
```

7_R_2014_03_03_21_49_55_Sequoia_SN1.41.Neonatal_29.10.2014_Hi.Q_non.mark_

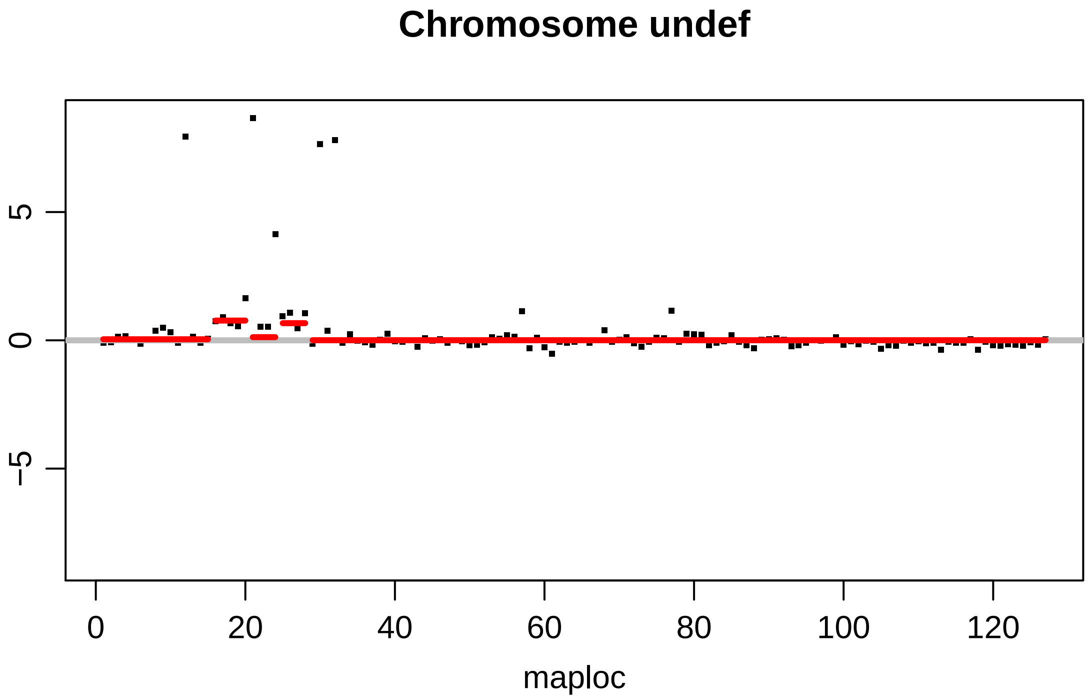


```
## Segplot might not work because of special characters in the sample names. Use only A-Z,a-z and 0-9!
##  There is a hidden function cn.mops:::.replaceNames that replaces the names in the "CNVDetectionResul
```

)_R_2014_03_03_21_49_55_Sequoia_SN1.41.Neonatal_29.10.2014_Hi.Q_non.mark_

Chromosome undef

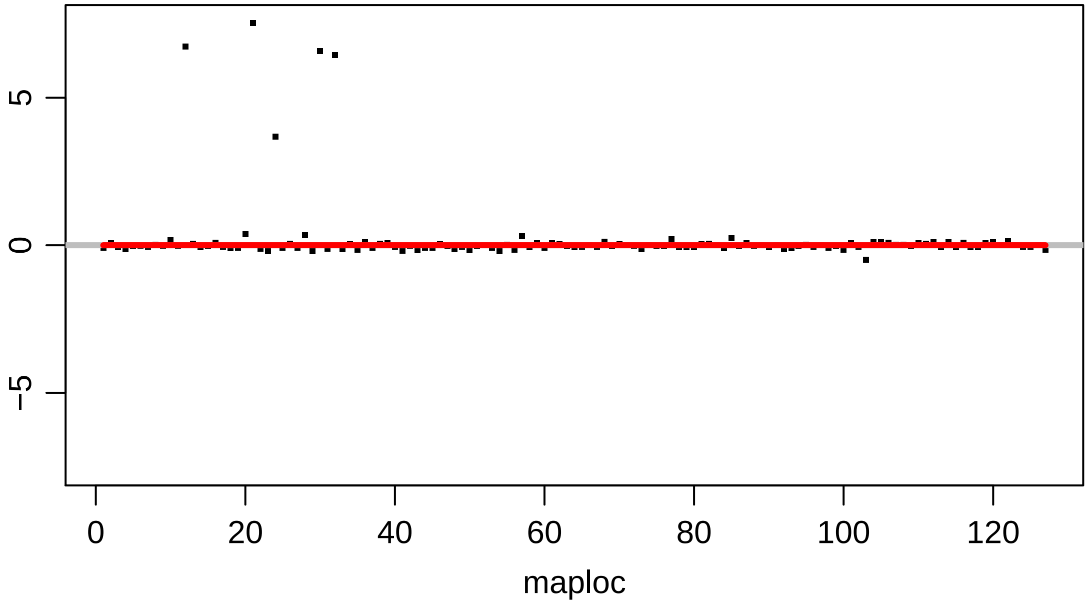


```
## Segplot might not work because of special characters in the sample names. Use only A-Z,a-z and 0-9!
##  There is a hidden function cn.mops:::.replaceNames that replaces the names in the "CNVDetectionResul
```

)_R_2014_03_03_21_49_55_Sequoia_SN1.41.Neonatal_29.10.2014_Hi.Q_non.mark_

Chromosome undef

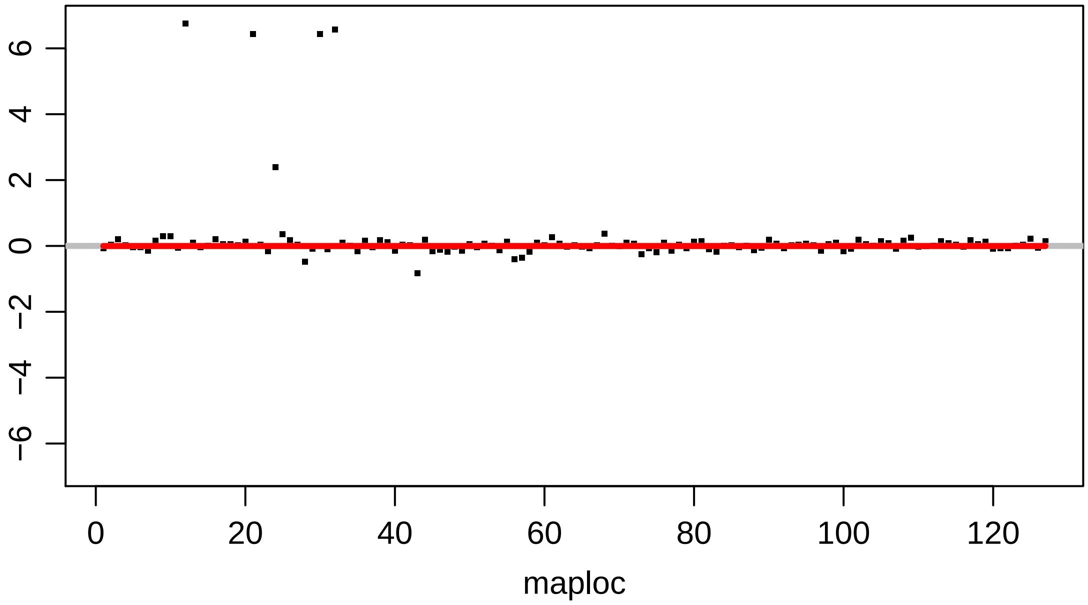

```
## Segplot might not work because of special characters in the sample names. Use only A-Z,a-z and 0-9!
##  There is a hidden function cn.mops:::.replaceNames that replaces the names in the "CNVDetectionResul
```

l_R_2014_03_03_21_49_55_Sequoia_SN1.41.Neonatal_29.10.2014_Hi.Q_non.mark_

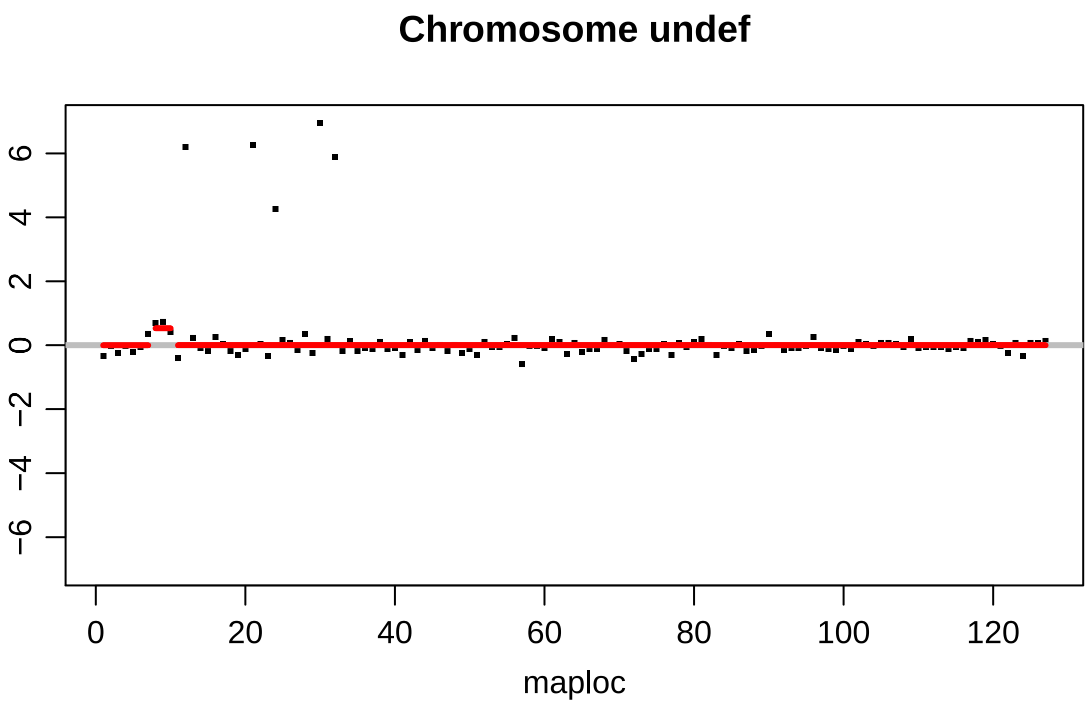


```
## Segplot might not work because of special characters in the sample names. Use only A-Z,a-z and 0-9!
##  There is a hidden function cn.mops:::.replaceNames that replaces the names in the "CNVDetectionResul
```

2_R_2014_03_03_21_49_55_Sequoia_SN1.41.Neonatal_29.10.2014_Hi.Q_non.mark_

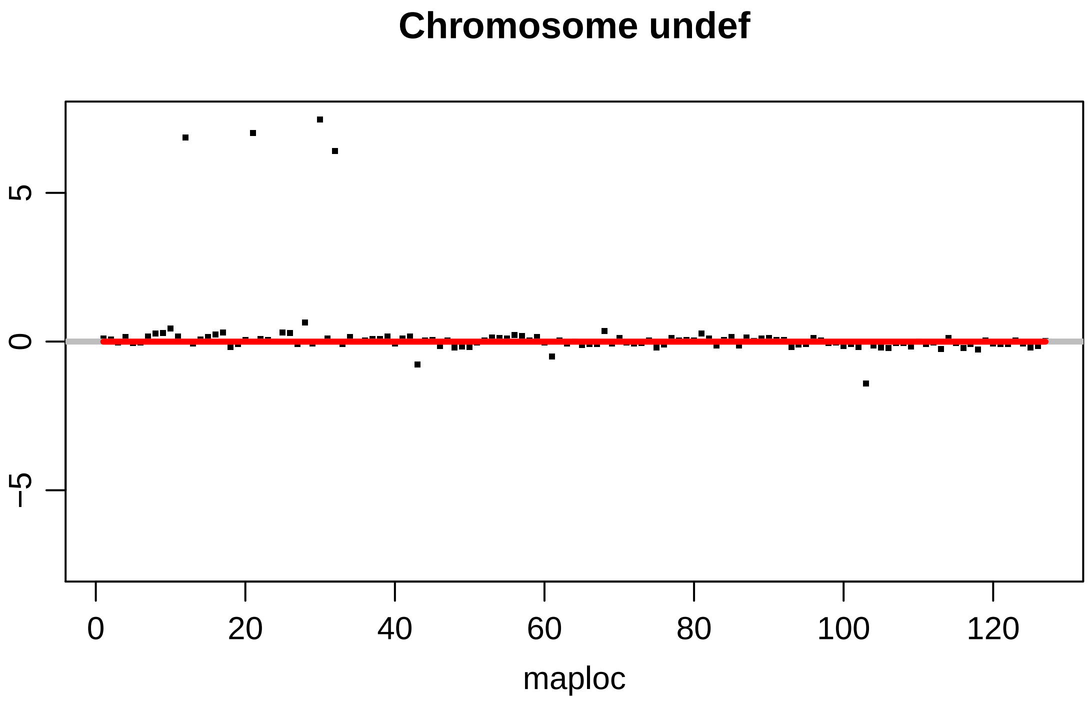


```
## Segplot might not work because of special characters in the sample names. Use only A-Z,a-z and 0-9!
##  There is a hidden function cn.mops:::.replaceNames that replaces the names in the "CNVDetectionResul
```

3_R_2014_03_03_21_49_55_Sequoia_SN1.41.Neonatal_29.10.2014_Hi.Q_non.mark_

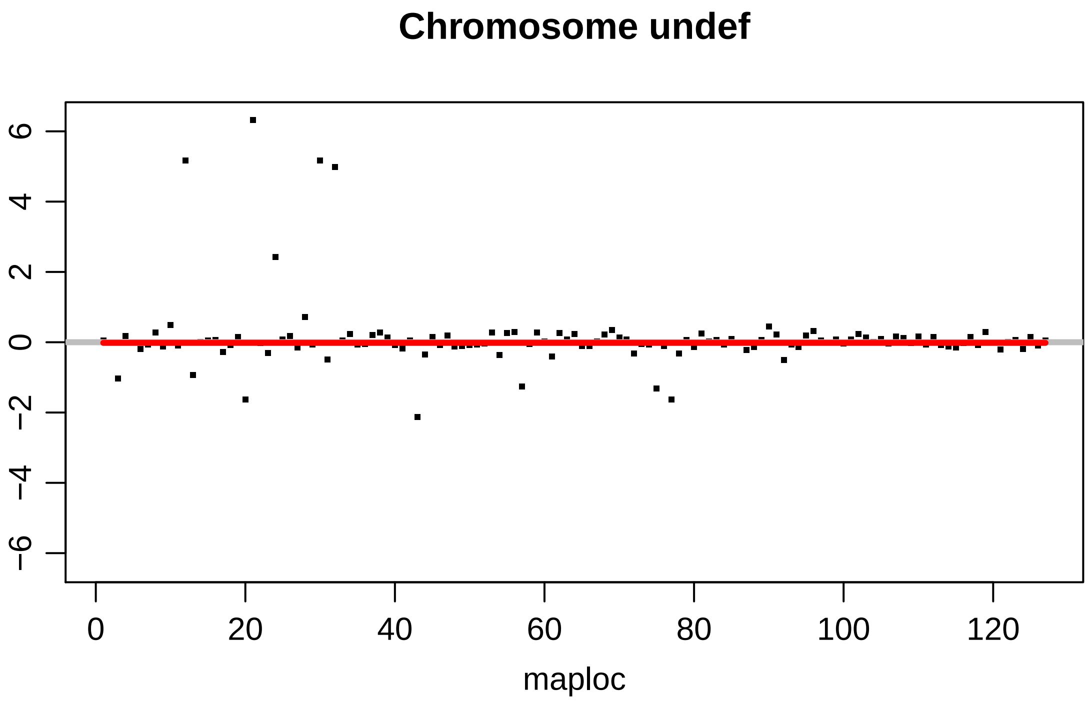

```
## Segplot might not work because of special characters in the sample names. Use only A-Z,a-z and 0-9!
##  There is a hidden function cn.mops:::.replaceNames that replaces the names in the "CNVDetectionResul
```

9_R_2014_03_03_21_49_55_Sequoia_SN1.41.Neonatal_29.10.2014_Hi.Q_non.mark_

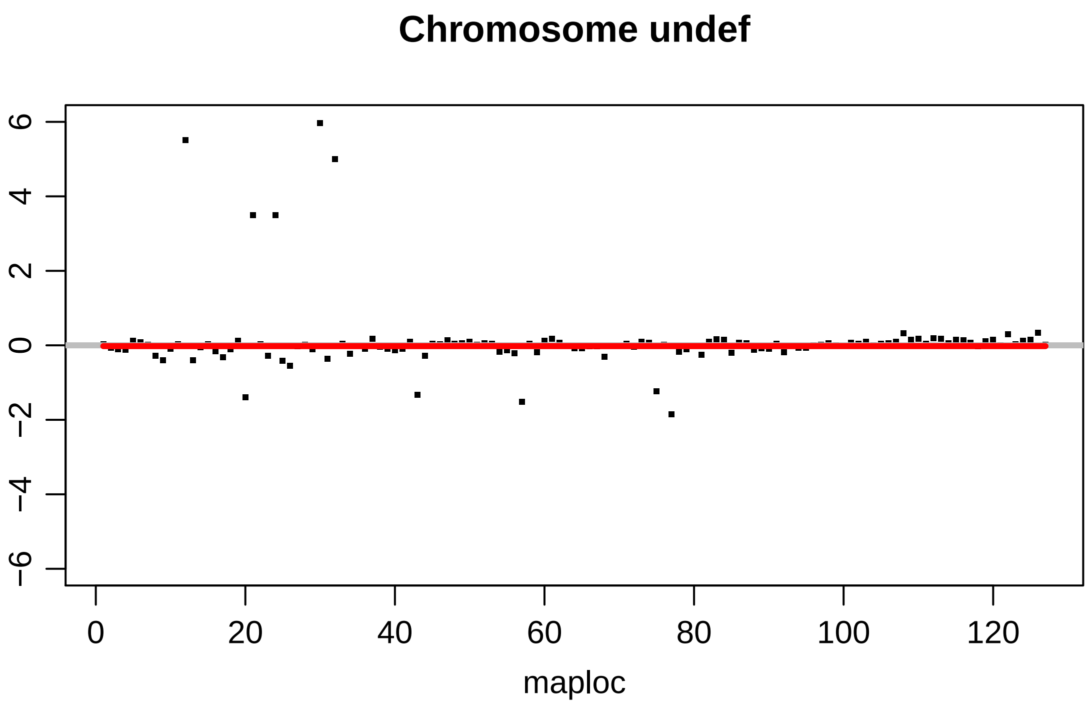


```
## Segplot might not work because of special characters in the sample names. Use only A-Z,a-z and 0-9!
##  There is a hidden function cn.mops:::.replaceNames that replaces the names in the "CNVDetectionResul
```

)_R_2014_03_03_21_49_55_Sequoia_SN1.41.Neonatal_29.10.2014_Hi.Q_non.mark_

Chromosome undef

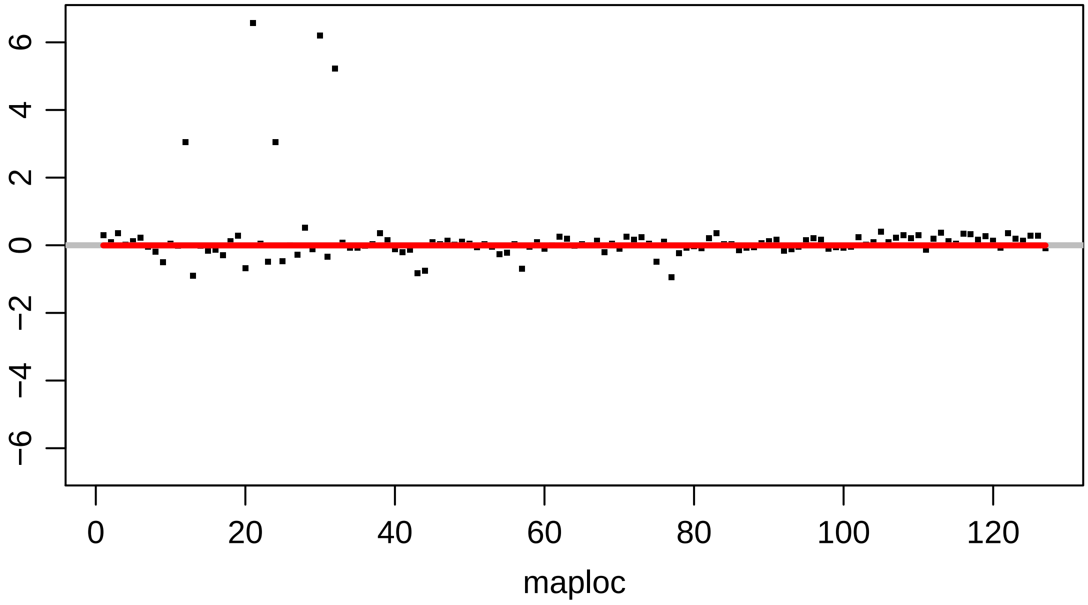


```
## Segplot might not work because of special characters in the sample names. Use only A-Z,a-z and 0-9!
##  There is a hidden function cn.mops:::.replaceNames that replaces the names in the "CNVDetectionResul
```

L_R_2014_03_03_21_49_55_Sequoia_SN1.41.Neonatal_29.10.2014_Hi.Q_non.mark_

Chromosome undef

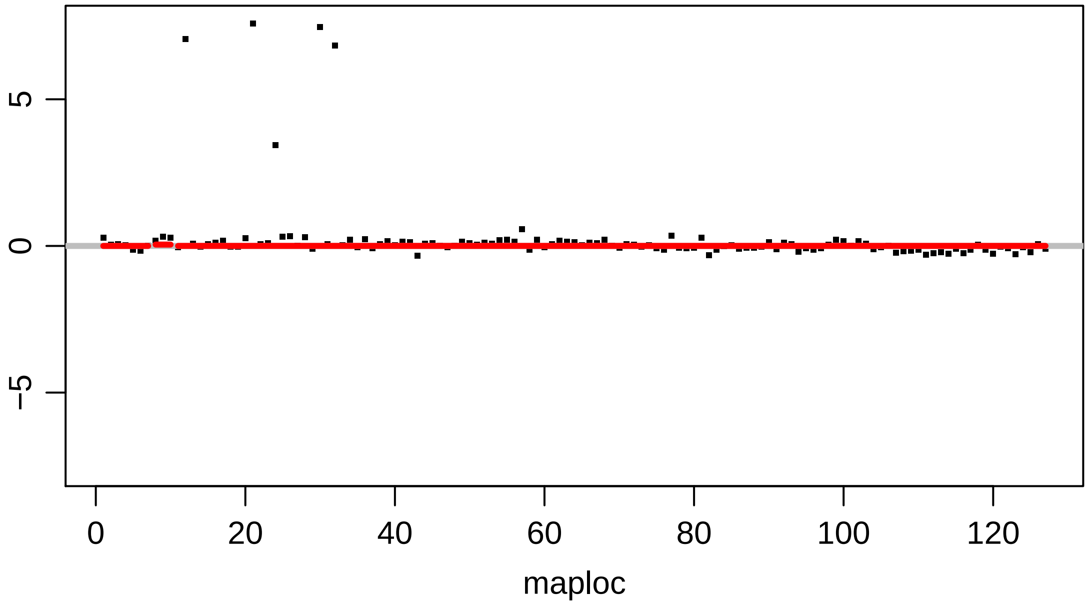

```
## Segplot might not work because of special characters in the sample names. Use only A-Z,a-z and 0-9!
##  There is a hidden function cn.mops:::.replaceNames that replaces the names in the "CNVDetectionResul
```

2_R_2014_03_03_21_49_55_Sequoia_SN1.41.Neonatal_29.10.2014_Hi.Q_non.mark_

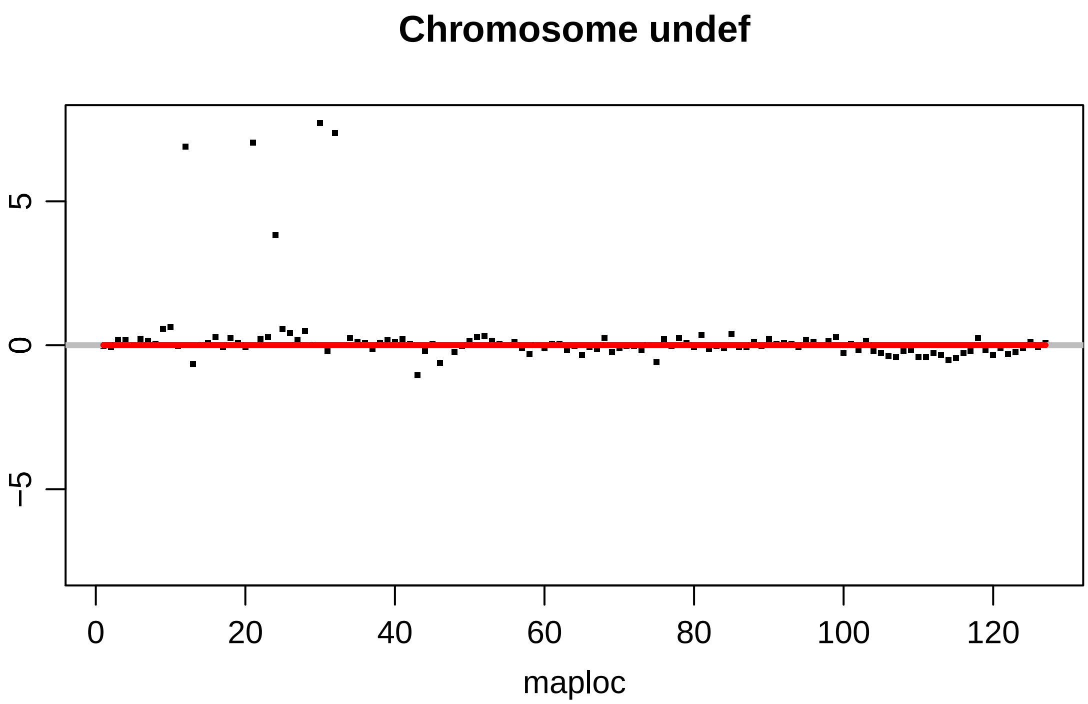


```
## Segplot might not work because of special characters in the sample names. Use only A-Z,a-z and 0-9!
##  There is a hidden function cn.mops:::.replaceNames that replaces the names in the "CNVDetectionResul
```

3_R_2014_03_03_21_49_55_Sequoia_SN1.41.Neonatal_29.10.2014_Hi.Q_non.mark_

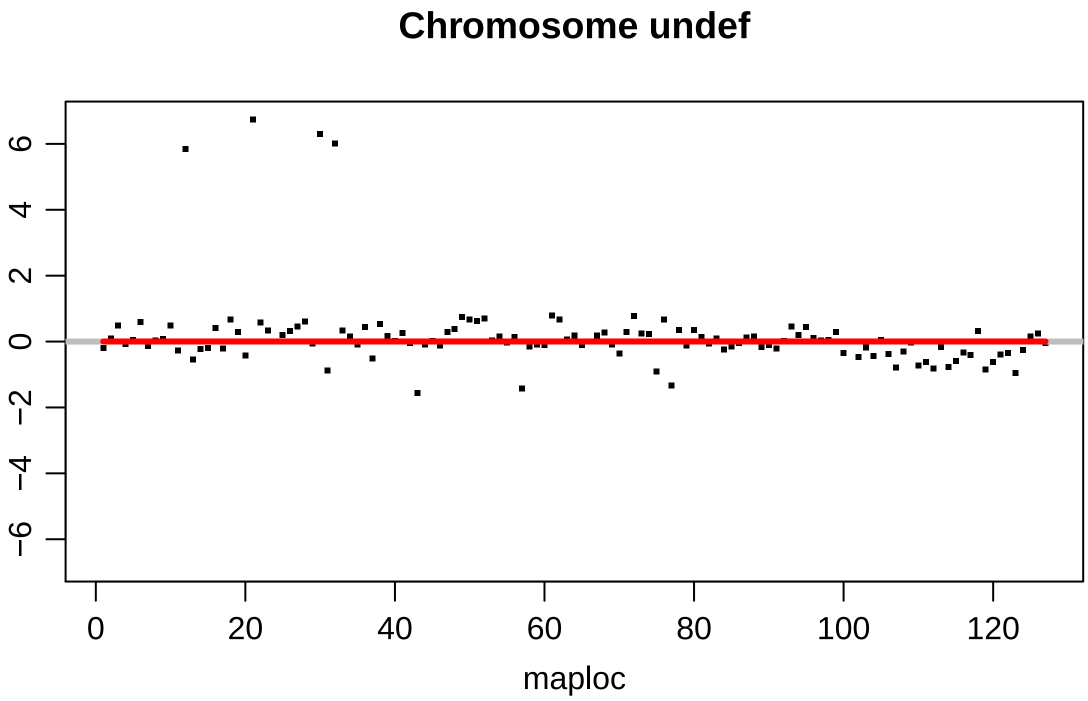


```
## Segplot might not work because of special characters in the sample names. Use only A-Z,a-z and 0-9!
##  There is a hidden function cn.mops:::.replaceNames that replaces the names in the "CNVDetectionResul
```

4_R_2014_03_03_21_49_55_Sequoia_SN1.41.Neonatal_29.10.2014_Hi.Q_non.mark_

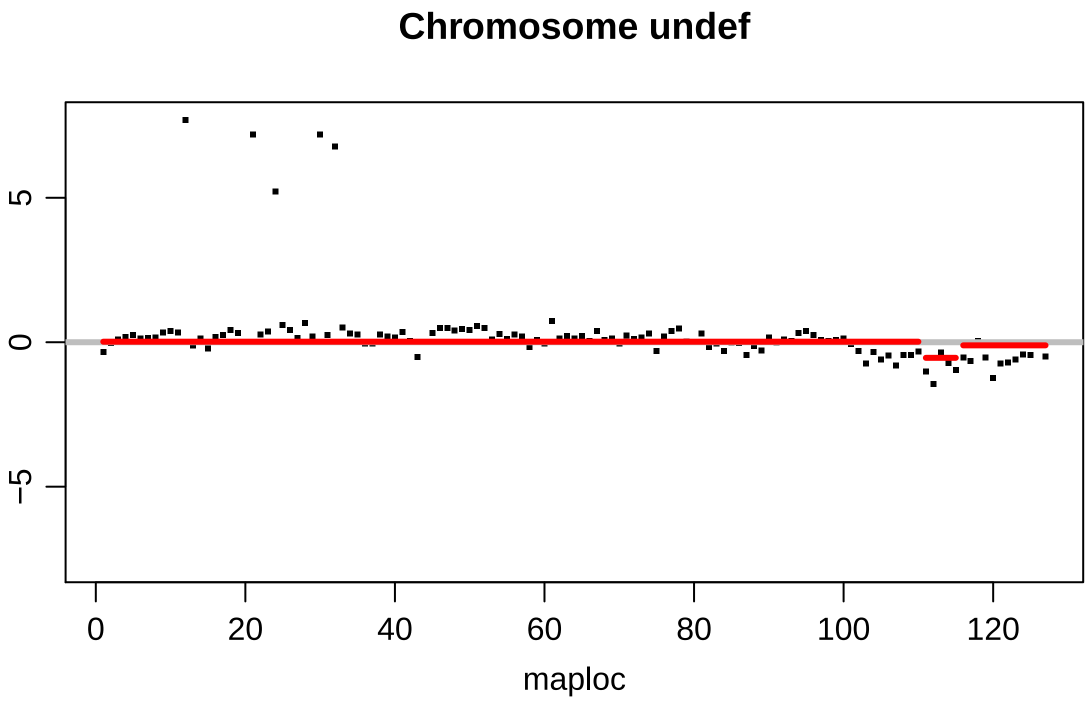

```
## Segplot might not work because of special characters in the sample names. Use only A-Z,a-z and 0-9!
##  There is a hidden function cn.mops:::.replaceNames that replaces the names in the "CNVDetectionResul
```

5_R_2014_03_03_21_49_55_Sequoia_SN1.41.Neonatal_29.10.2014_Hi.Q_non.mark_

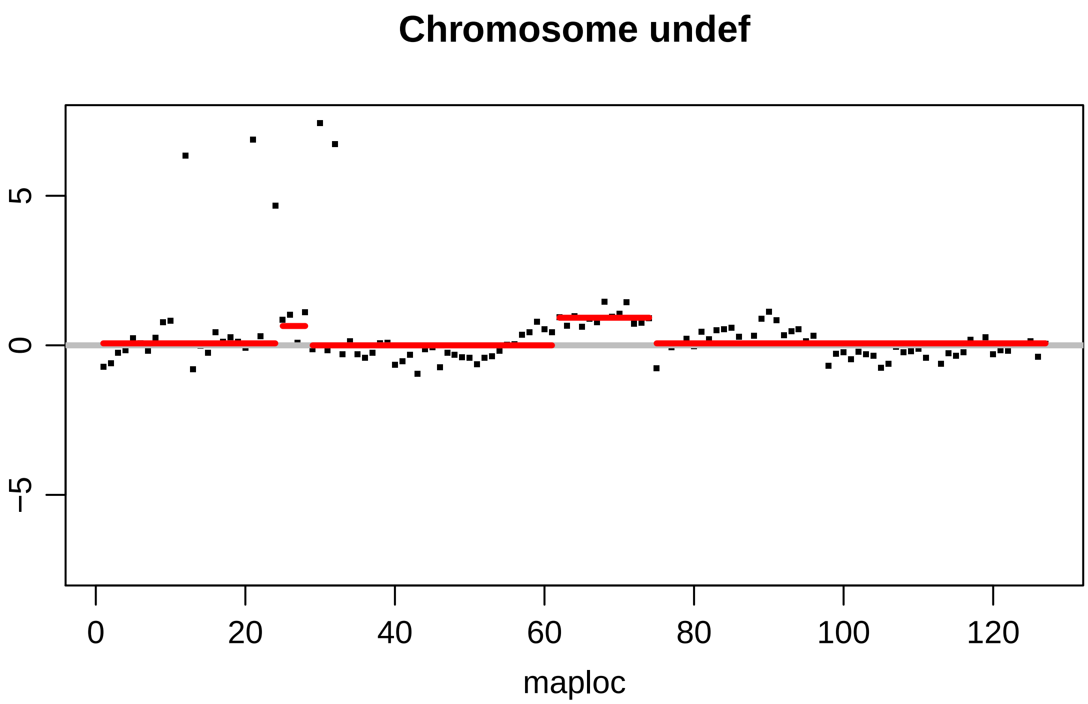


```
## Segplot might not work because of special characters in the sample names. Use only A-Z,a-z and 0-9!
##  There is a hidden function cn.mops:::.replaceNames that replaces the names in the "CNVDetectionResul
```

3_R_2014_03_03_21_49_55_Sequoia_SN1.41.Neonatal_29.10.2014_Hi.Q_non.mark_

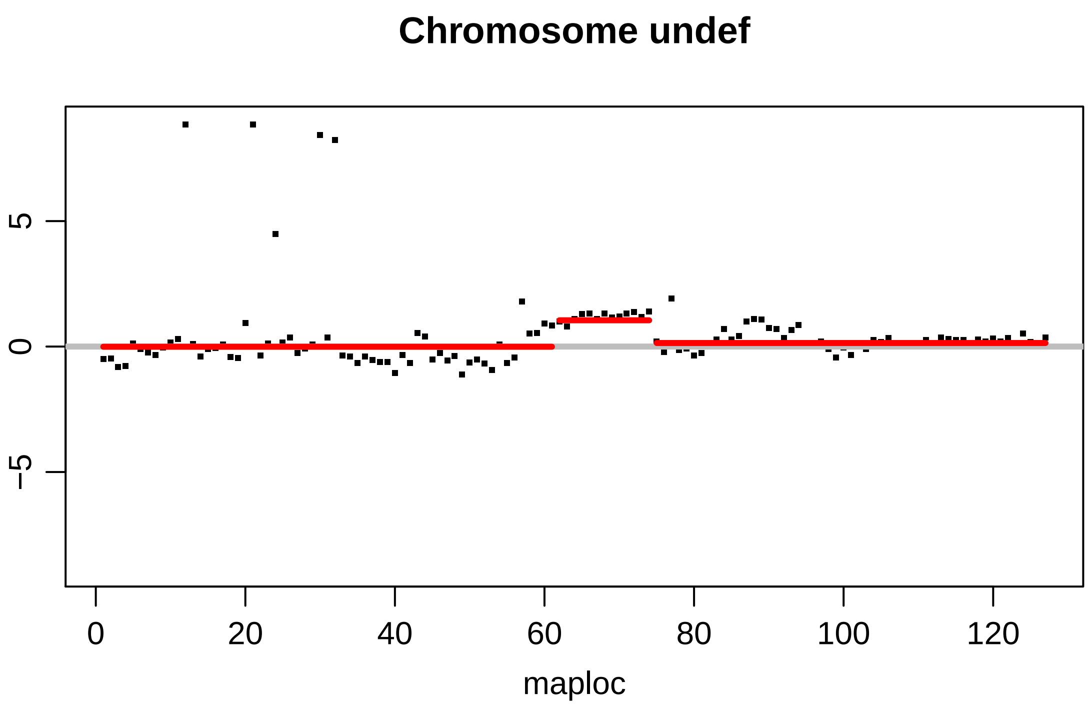


```
## Segplot might not work because of special characters in the sample names. Use only A-Z,a-z and 0-9!
##  There is a hidden function cn.mops:::.replaceNames that replaces the names in the "CNVDetectionResu
```

)_R_2014_03_03_21_49_55_Sequoia_SN1.41.Neonatal_29.10.2014_Hi.Q_non.mark_

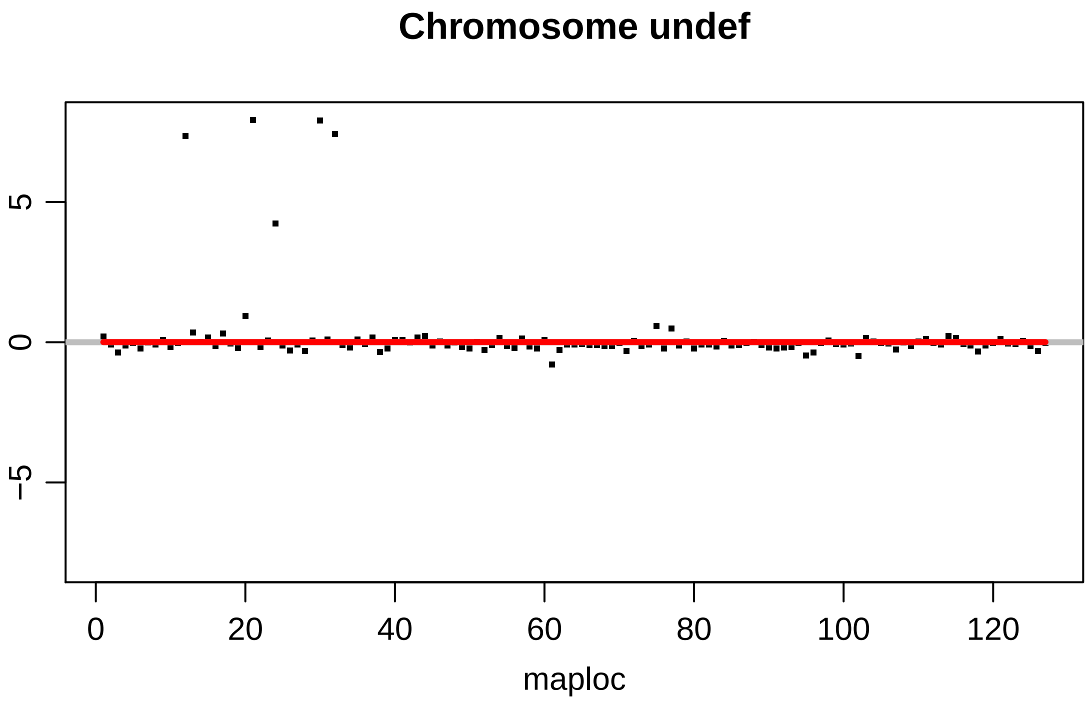

```
## Segplot might not work because of special characters in the sample names. Use only A-Z,a-z and 0-9!
##  There is a hidden function cn.mops:::.replaceNames that replaces the names in the "CNVDetectionResul
```

l_R_2014_03_03_21_49_55_Sequoia_SN1.41.Neonatal_29.10.2014_Hi.Q_non.mark_

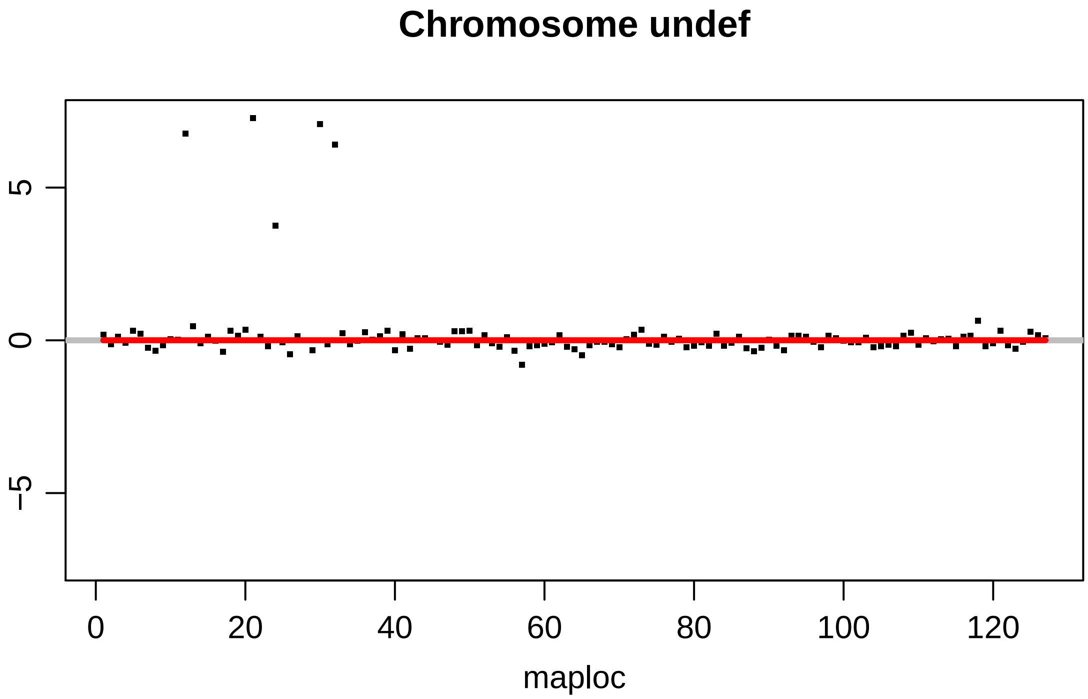


```
## Segplot might not work because of special characters in the sample names. Use only A-Z,a-z and 0-9!
##  There is a hidden function cn.mops:::.replaceNames that replaces the names in the "CNVDetectionResul
```

2_R_2014_03_03_21_49_55_Sequoia_SN1.41.Neonatal_29.10.2014_Hi.Q_non.mark_

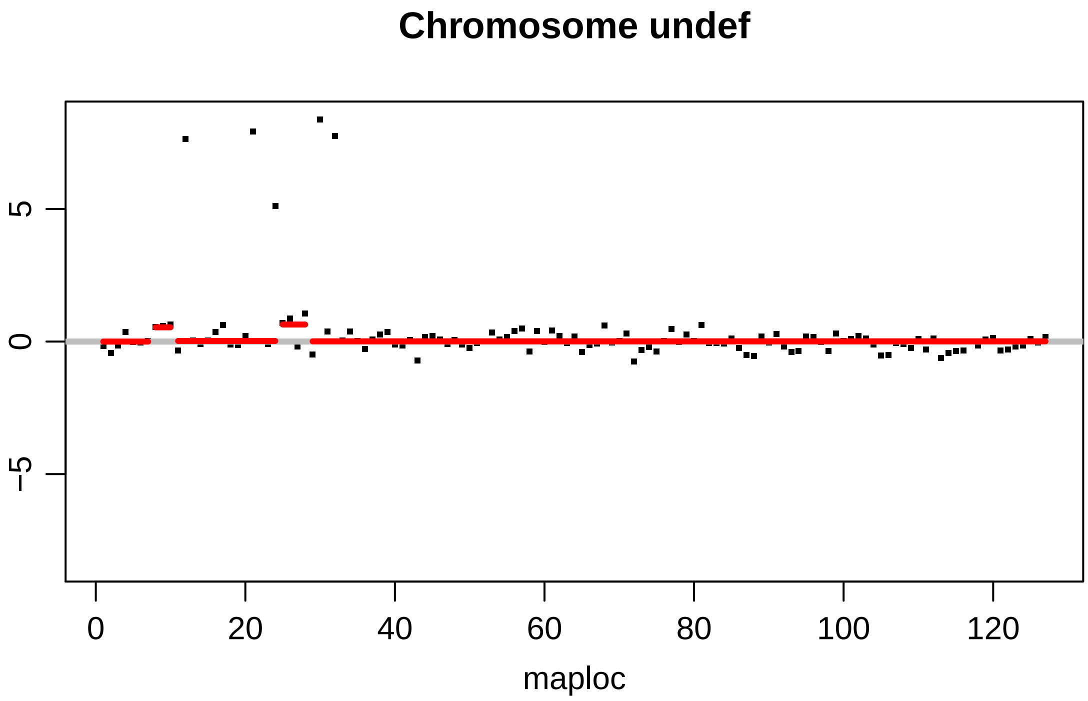


```
##
## CNV regions:
## GRanges object with 4 ranges and 31 metadata columns:
##       seqnames    ranges strand |
##          <Rle> <IRanges>  <Rle> |
##   [1]    undef  [ 8, 10]      * |
##   [2]    undef  [16, 20]      * |
##   [3]    undef  [25, 28]      * |
##   [4]    undef  [62, 74]      * |
##       Case_IonXpress_001_R_2014_03_03_21_49_55_Sequoia_SN1.41.Neonatal_29.10.2014_Hi.Q_non.mark_pcr_
##
##   [1]
##   [2]
##   [3]
##   [4]
##       Case_IonXpress_002_R_2014_03_03_21_49_55_Sequoia_SN1.41.Neonatal_29.10.2014_Hi.Q_non.mark_pcr_
##
##   [1]
##   [2]
##   [3]
##   [4]
##       Case_IonXpress_003_R_2014_03_03_21_49_55_Sequoia_SN1.41.Neonatal_29.10.2014_Hi.Q_non.mark_pcr_
##
##   [1]
##   [2]
##   [3]
##   [4]
##       Case_IonXpress_004_R_2014_03_03_21_49_55_Sequoia_SN1.41.Neonatal_29.10.2014_Hi.Q_non.mark_pcr_
```

```
##
##   [1]
##   [2]
##   [3]
##   [4]
##       Case_IonXpress_005_R_2014_03_03_21_49_55_Sequoia_SN1.41.Neonatal_29.10.2014_Hi.Q_non.mark_pcr_c
##
##   [1]
##   [2]
##   [3]
##   [4]
##       Case_IonXpress_006_R_2014_03_03_21_49_55_Sequoia_SN1.41.Neonatal_29.10.2014_Hi.Q_non.mark_pcr_c
##
##   [1]
##   [2]
##   [3]
##   [4]
##       Case_IonXpress_007_R_2014_03_03_21_49_55_Sequoia_SN1.41.Neonatal_29.10.2014_Hi.Q_non.mark_pcr_c
##
##   [1]
##   [2]
##   [3]
##   [4]
##       Case_IonXpress_008_R_2014_03_03_21_49_55_Sequoia_SN1.41.Neonatal_29.10.2014_Hi.Q_non.mark_pcr_c
##
##   [1]
##   [2]
##   [3]
##   [4]
##       Case_IonXpress_009_R_2014_03_03_21_49_55_Sequoia_SN1.41.Neonatal_29.10.2014_Hi.Q_non.mark_pcr_c
##
##   [1]
##   [2]
##   [3]
##   [4]
##       Case_IonXpress_010_R_2014_03_03_21_49_55_Sequoia_SN1.41.Neonatal_29.10.2014_Hi.Q_non.mark_pcr_c
##
##   [1]
##   [2]
##   [3]
##   [4]
##       Case_IonXpress_012_R_2014_03_03_21_49_55_Sequoia_SN1.41.Neonatal_29.10.2014_Hi.Q_non.mark_pcr_c
##
##   [1]
##   [2]
##   [3]
##   [4]
##       Case_IonXpress_015_R_2014_03_03_21_49_55_Sequoia_SN1.41.Neonatal_29.10.2014_Hi.Q_non.mark_pcr_c
##
##   [1]
##   [2]
##   [3]
##   [4]
##       Case_IonXpress_016_R_2014_03_03_21_49_55_Sequoia_SN1.41.Neonatal_29.10.2014_Hi.Q_non.mark_pcr_c
```

```
##
##   [1]
##   [2]
##   [3]
##   [4]
##       Case_IonXpress_017_R_2014_03_03_21_49_55_Sequoia_SN1.41.Neonatal_29.10.2014_Hi.Q_non.mark_pcr_
##
##   [1]
##   [2]
##   [3]
##   [4]
##       Case_IonXpress_019_R_2014_03_03_21_49_55_Sequoia_SN1.41.Neonatal_29.10.2014_Hi.Q_non.mark_pcr_
##
##   [1]
##   [2]
##   [3]
##   [4]
##       Case_IonXpress_020_R_2014_03_03_21_49_55_Sequoia_SN1.41.Neonatal_29.10.2014_Hi.Q_non.mark_pcr_
##
##   [1]
##   [2]
##   [3]
##   [4]
##       Case_IonXpress_021_R_2014_03_03_21_49_55_Sequoia_SN1.41.Neonatal_29.10.2014_Hi.Q_non.mark_pcr_
##
##   [1]
##   [2]
##   [3]
##   [4]
##       Case_IonXpress_022_R_2014_03_03_21_49_55_Sequoia_SN1.41.Neonatal_29.10.2014_Hi.Q_non.mark_pcr_
##
##   [1]
##   [2]
##   [3]
##   [4]
##       Case_IonXpress_023_R_2014_03_03_21_49_55_Sequoia_SN1.41.Neonatal_29.10.2014_Hi.Q_non.mark_pcr_
##
##   [1]
##   [2]
##   [3]
##   [4]
##       Case_IonXpress_049_R_2014_03_03_21_49_55_Sequoia_SN1.41.Neonatal_29.10.2014_Hi.Q_non.mark_pcr_
##
##   [1]
##   [2]
##   [3]
##   [4]
##       Case_IonXpress_050_R_2014_03_03_21_49_55_Sequoia_SN1.41.Neonatal_29.10.2014_Hi.Q_non.mark_pcr_
##
##   [1]
##   [2]
##   [3]
##   [4]
##       Case_IonXpress_051_R_2014_03_03_21_49_55_Sequoia_SN1.41.Neonatal_29.10.2014_Hi.Q_non.mark_pcr_
```

```
##
##   [1]
##   [2]
##   [3]
##   [4]
##       Case_IonXpress_052_R_2014_03_03_21_49_55_Sequoia_SN1.41.Neonatal_29.10.2014_Hi.Q_non.mark_pcr_c
##
##   [1]
##   [2]
##   [3]
##   [4]
##       Case_IonXpress_053_R_2014_03_03_21_49_55_Sequoia_SN1.41.Neonatal_29.10.2014_Hi.Q_non.mark_pcr_c
##
##   [1]
##   [2]
##   [3]
##   [4]
##       Case_IonXpress_054_R_2014_03_03_21_49_55_Sequoia_SN1.41.Neonatal_29.10.2014_Hi.Q_non.mark_pcr_c
##
##   [1]
##   [2]
##   [3]
##   [4]
##       Case_IonXpress_055_R_2014_03_03_21_49_55_Sequoia_SN1.41.Neonatal_29.10.2014_Hi.Q_non.mark_pcr_c
##
##   [1]
##   [2]
##   [3]
##   [4]
##       Case_IonXpress_058_R_2014_03_03_21_49_55_Sequoia_SN1.41.Neonatal_29.10.2014_Hi.Q_non.mark_pcr_c
##
##   [1]
##   [2]
##   [3]
##   [4]
##       Case_IonXpress_060_R_2014_03_03_21_49_55_Sequoia_SN1.41.Neonatal_29.10.2014_Hi.Q_non.mark_pcr_c
##
##   [1]
##   [2]
##   [3]
##   [4]
##       Case_IonXpress_061_R_2014_03_03_21_49_55_Sequoia_SN1.41.Neonatal_29.10.2014_Hi.Q_non.mark_pcr_c
##
##   [1]
##   [2]
##   [3]
##   [4]
##       Case_IonXpress_062_R_2014_03_03_21_49_55_Sequoia_SN1.41.Neonatal_29.10.2014_Hi.Q_non.mark_pcr_c
##
##   [1]
##   [2]
##   [3]
##   [4]
##       Case_IonXpress_063_R_2014_03_03_21_49_55_Sequoia_SN1.41.Neonatal_29.10.2014_Hi.Q_non.mark_pcr_c
```

```
##
##   [1]
##   [2]
##   [3]
##   [4]
##   -------
##   seqinfo: 1 sequence from an unspecified genome; no seqlengths
##
## Individual CNVs:
## GRanges object with 13 ranges and 4 metadata columns:
##        seqnames    ranges strand   |
##           <Rle> <IRanges>  <Rle>   |
##    [1]    undef  [ 8, 10]      *   |
##    [2]    undef  [16, 20]      *   |
##    [3]    undef  [16, 20]      *   |
##    [4]    undef  [16, 20]      *   |
##    [5]    undef  [25, 28]      *   |
##    ...      ...       ...    ... ...
##    [9]    undef  [62, 74]      *   |
##   [10]    undef  [ 8, 10]      *   |
##   [11]    undef  [25, 28]      *   |
##   [12]    undef  [ 8, 10]      *   |
##   [13]    undef  [25, 28]      *   |
##
##
##    [1] Case_IonXpress_012_R_2014_03_03_21_49_55_Sequoia_SN1.41.Neonatal_29.10.2014_Hi.Q_non.mark_pcr_
##    [2] Case_IonXpress_015_R_2014_03_03_21_49_55_Sequoia_SN1.41.Neonatal_29.10.2014_Hi.Q_non.mark_pcr_
##    [3] Case_IonXpress_016_R_2014_03_03_21_49_55_Sequoia_SN1.41.Neonatal_29.10.2014_Hi.Q_non.mark_pcr_
##    [4] Case_IonXpress_017_R_2014_03_03_21_49_55_Sequoia_SN1.41.Neonatal_29.10.2014_Hi.Q_non.mark_pcr_
##    [5] Case_IonXpress_017_R_2014_03_03_21_49_55_Sequoia_SN1.41.Neonatal_29.10.2014_Hi.Q_non.mark_pcr_
##    ...
##    [9] Case_IonXpress_058_R_2014_03_03_21_49_55_Sequoia_SN1.41.Neonatal_29.10.2014_Hi.Q_non.mark_pcr_
##   [10] Case_IonXpress_062_R_2014_03_03_21_49_55_Sequoia_SN1.41.Neonatal_29.10.2014_Hi.Q_non.mark_pcr_
##   [11] Case_IonXpress_062_R_2014_03_03_21_49_55_Sequoia_SN1.41.Neonatal_29.10.2014_Hi.Q_non.mark_pcr_
##   [12] Case_IonXpress_063_R_2014_03_03_21_49_55_Sequoia_SN1.41.Neonatal_29.10.2014_Hi.Q_non.mark_pcr_
##   [13] Case_IonXpress_063_R_2014_03_03_21_49_55_Sequoia_SN1.41.Neonatal_29.10.2014_Hi.Q_non.mark_pcr_
##           median       mean          CN
##        <numeric>  <numeric> <character>
##    [1] -0.9872893 -0.9689963         CN1
##    [2]  0.5405514  0.6452307         CN3
##    [3]  0.5848861  0.7738501         CN3
##    [4]  0.5826300  0.7701545         CN3
##    [5]  0.7884128  0.6692994         CN3
##    ...        ...        ...         ...
##    [9]  1.0000000  1.0464042         CN4
##   [10]  0.5553738  0.5367930         CN3
##   [11]  0.7882592  0.6426108         CN3
##   [12]  0.5844680  0.5753264         CN3
##   [13]  0.7912909  0.6458720         CN3
##   -------
##   seqinfo: 1 sequence from an unspecified genome; no seqlengths
## [1] "/Users/gdemidov/Downloads/doc/Run_SN1_42_CRG_fin_05_qc.xls"

## Normalizing...
```

```
## Starting local modeling, please be patient...

## Reference sequence:  undef

## Starting segmentation algorithm...

## Using "fastseg" for segmentation.

## [1] ""
## [1] "/Users/gdemidov/Downloads/doc/Run_SN1_42_CRG_fin_05_qc.xls"
## [1] ""

## Segplot might not work because of special characters in the sample names. Use only A-Z,a-z and 0-9!
##  There is a hidden function cn.mops:::.replaceNames that replaces the names in the "CNVDetectionResu
```

3_R_2014_03_03_21_49_55_Sequoia_SN1.41.Neonatal_29.10.2014_Hi.Q_non.mark_

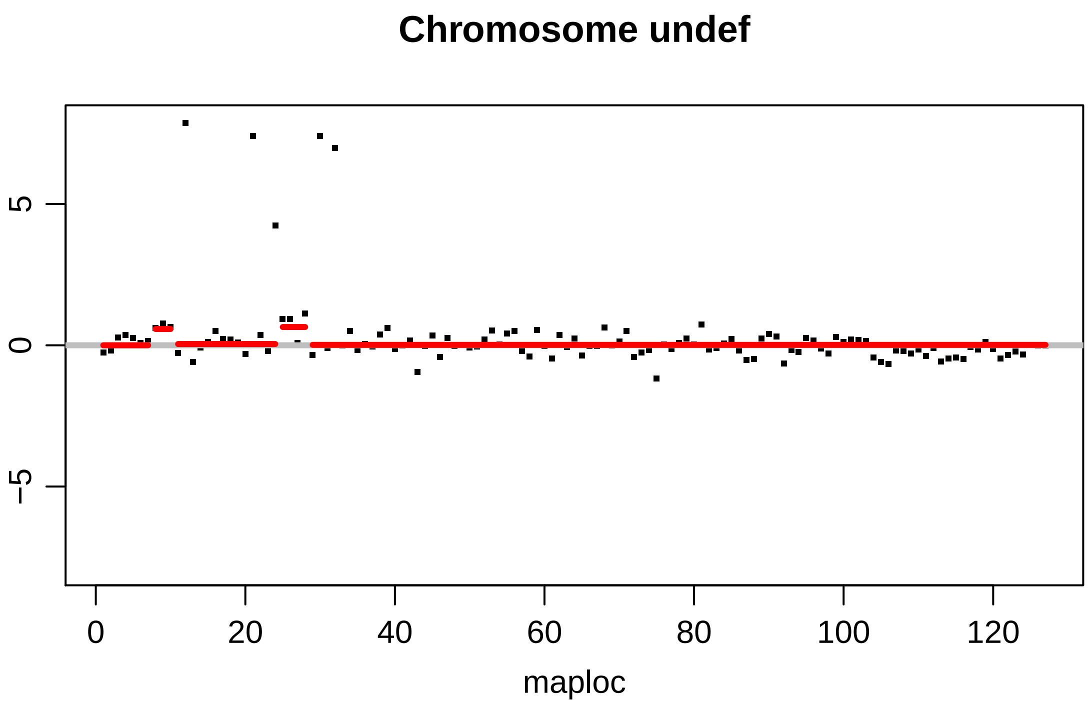


```
## Segplot might not work because of special characters in the sample names. Use only A-Z,a-z and 0-9!
##  There is a hidden function cn.mops:::.replaceNames that replaces the names in the "CNVDetectionResu
```

Case_CONTROL.blood.sam

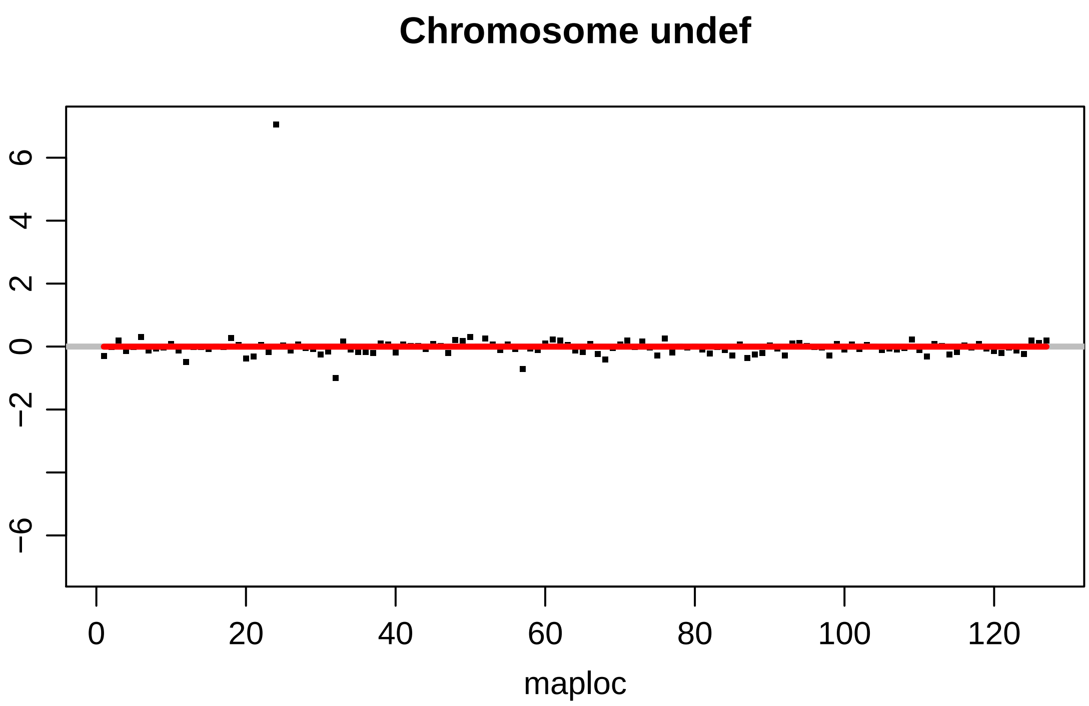


```
## Segplot might not work because of special characters in the sample names. Use only A-Z,a-z and 0-9!
##  There is a hidden function cn.mops:::.replaceNames that replaces the names in the "CNVDetectionResul
```

Case_L048.G1.sam

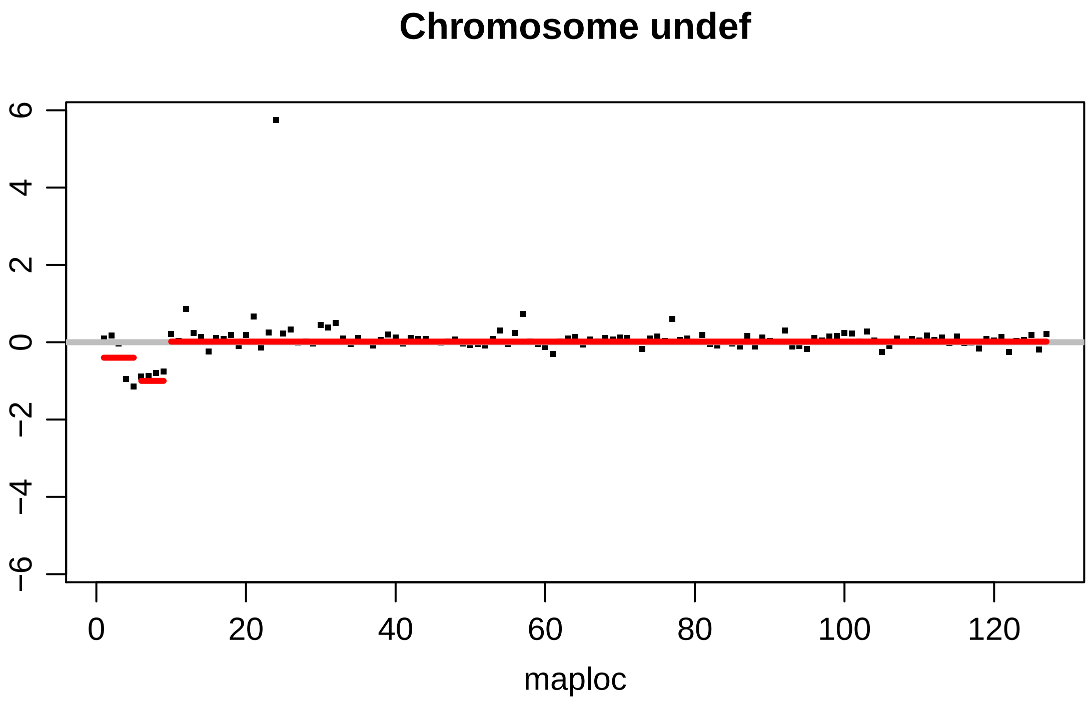

```
## Segplot might not work because of special characters in the sample names. Use only A-Z,a-z and 0-9!
##  There is a hidden function cn.mops:::.replaceNames that replaces the names in the "CNVDetectionResul
```

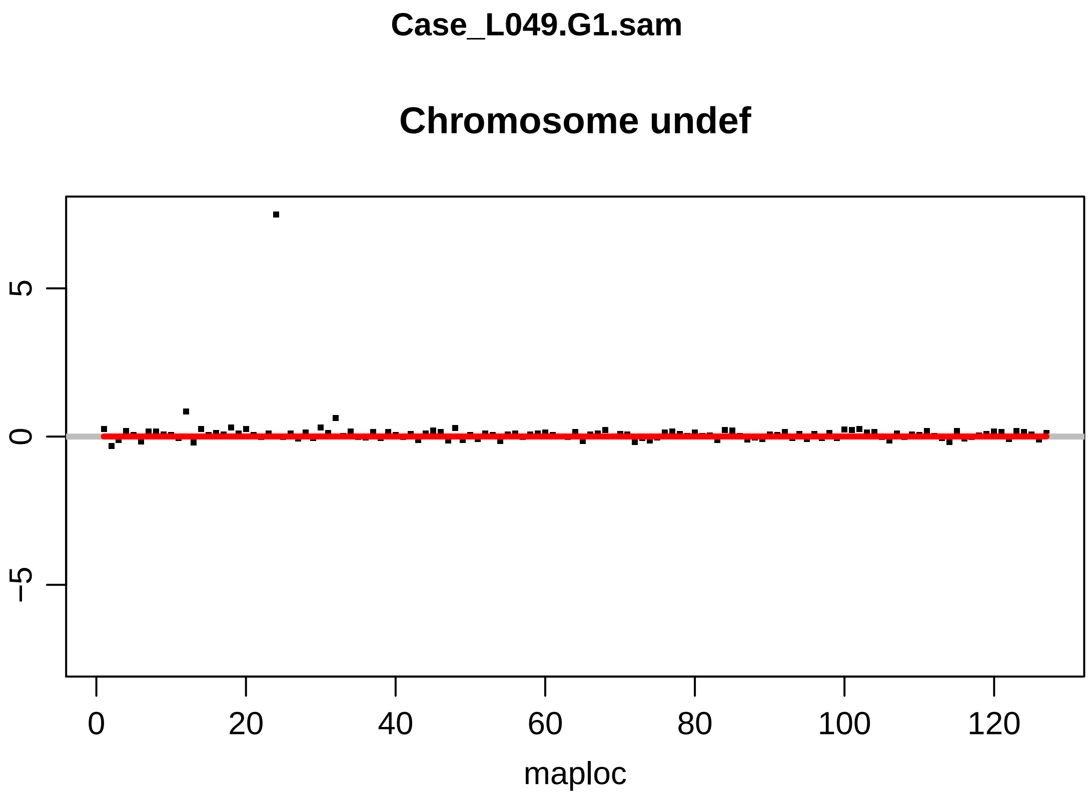


```
## Segplot might not work because of special characters in the sample names. Use only A-Z,a-z and 0-9!
##  There is a hidden function cn.mops:::.replaceNames that replaces the names in the "CNVDetectionResul
```

Case_L050.G1.sam

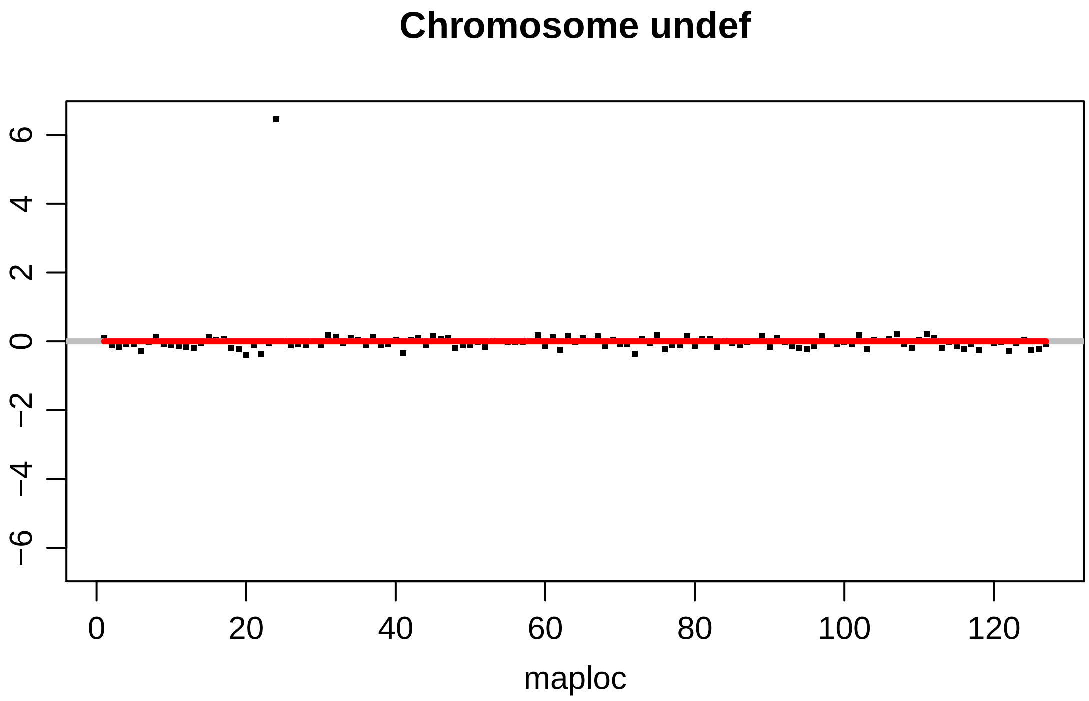


```
## Segplot might not work because of special characters in the sample names. Use only A-Z,a-z and 0-9!
##  There is a hidden function cn.mops:::.replaceNames that replaces the names in the "CNVDetectionResu
```

Case_L051.G1.sam

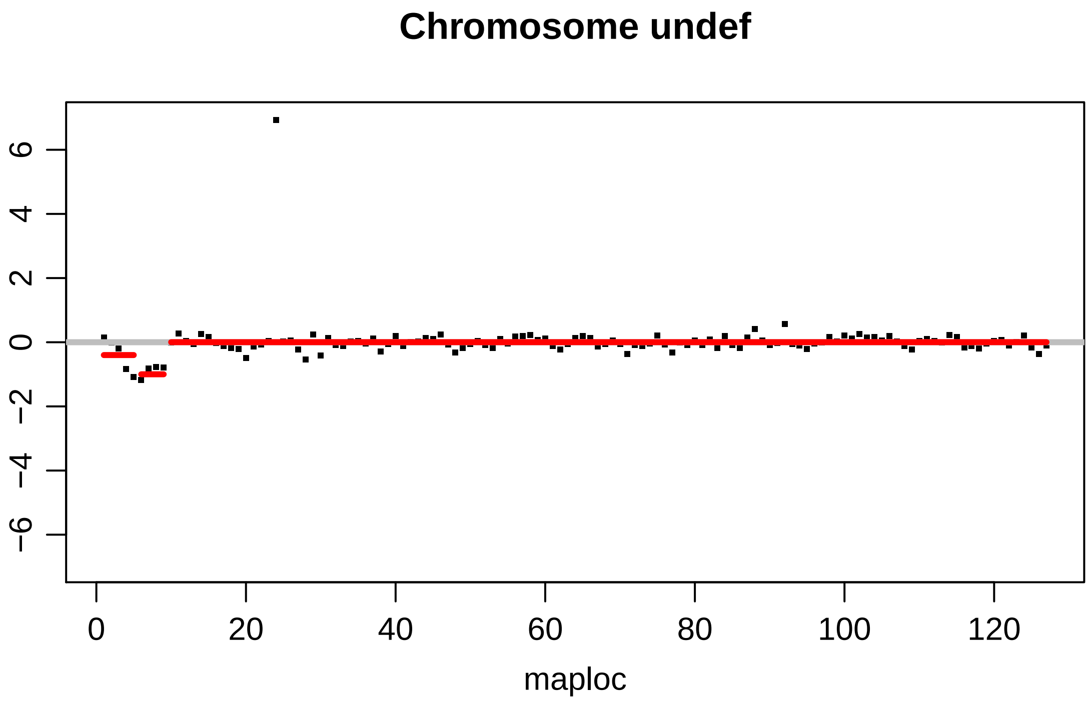

```
## Segplot might not work because of special characters in the sample names. Use only A-Z,a-z and 0-9!
##  There is a hidden function cn.mops:::.replaceNames that replaces the names in the "CNVDetectionResul
```

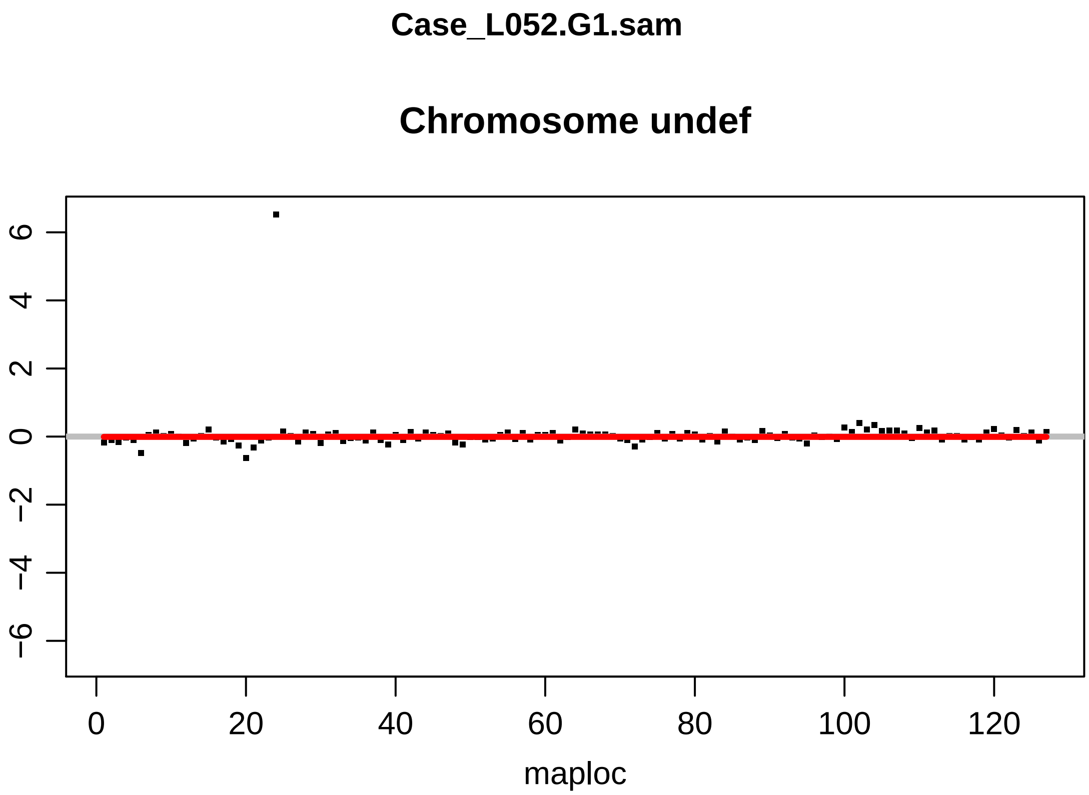


```
## Segplot might not work because of special characters in the sample names. Use only A-Z,a-z and 0-9!
##  There is a hidden function cn.mops:::.replaceNames that replaces the names in the "CNVDetectionResul
```

Case_L053.G1.sam

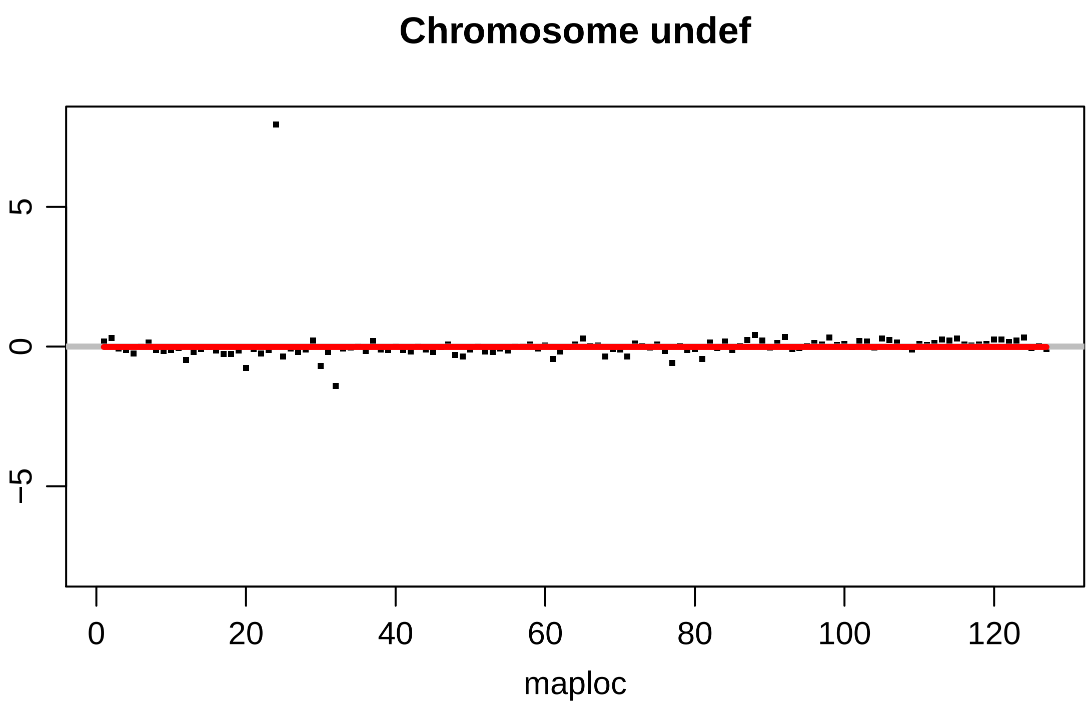


```
## Segplot might not work because of special characters in the sample names. Use only A-Z,a-z and 0-9!
##  There is a hidden function cn.mops:::.replaceNames that replaces the names in the "CNVDetectionResul
```

Case_L054.G1.sam

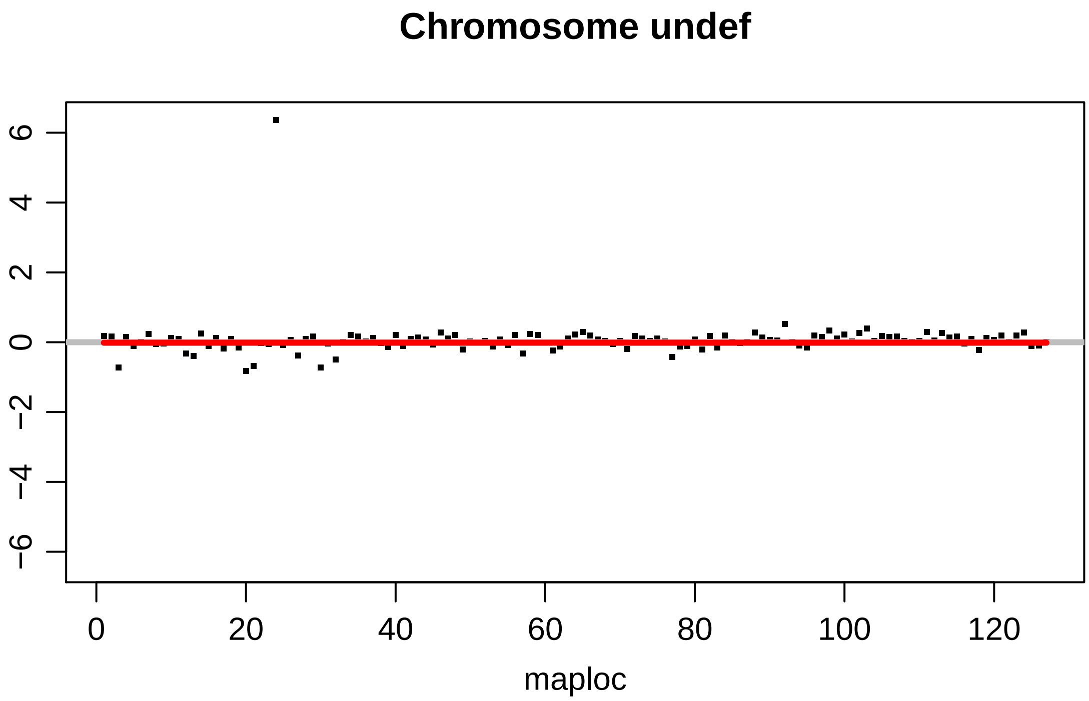

```
## Segplot might not work because of special characters in the sample names. Use only A-Z,a-z and 0-9!
##  There is a hidden function cn.mops:::.replaceNames that replaces the names in the "CNVDetectionResul
```

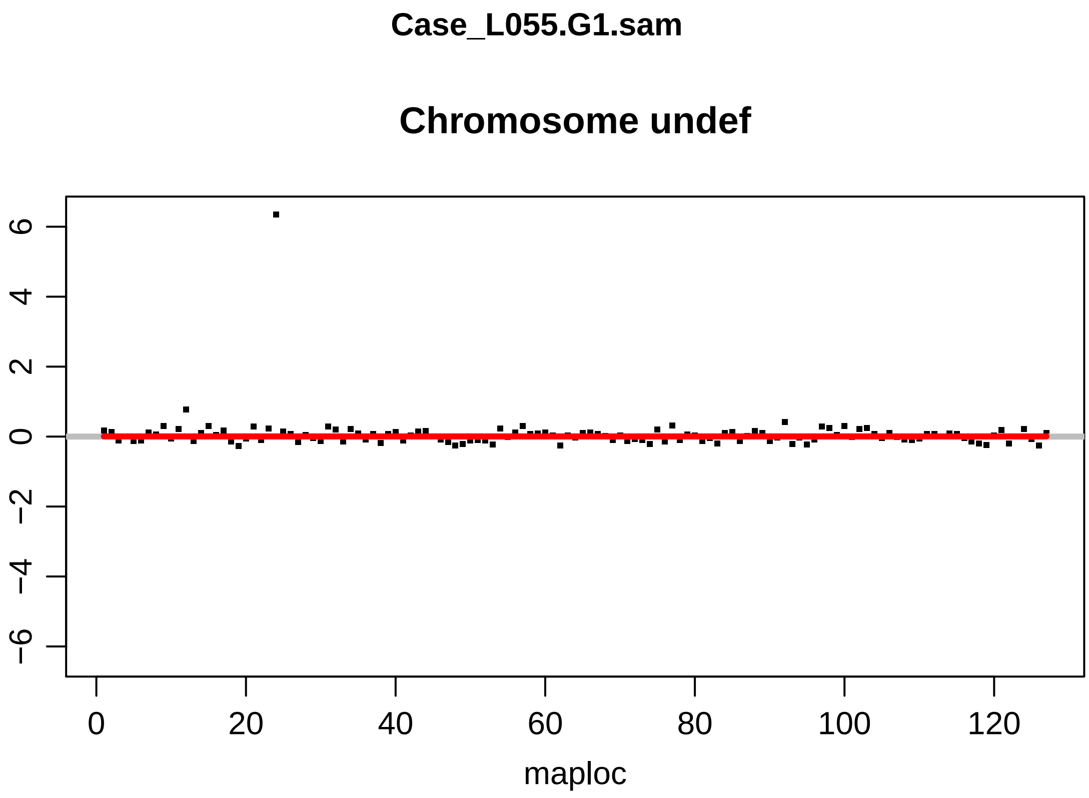


```
## Segplot might not work because of special characters in the sample names. Use only A-Z,a-z and 0-9!
##  There is a hidden function cn.mops:::.replaceNames that replaces the names in the "CNVDetectionResul
```

Case_L056.G1.sam

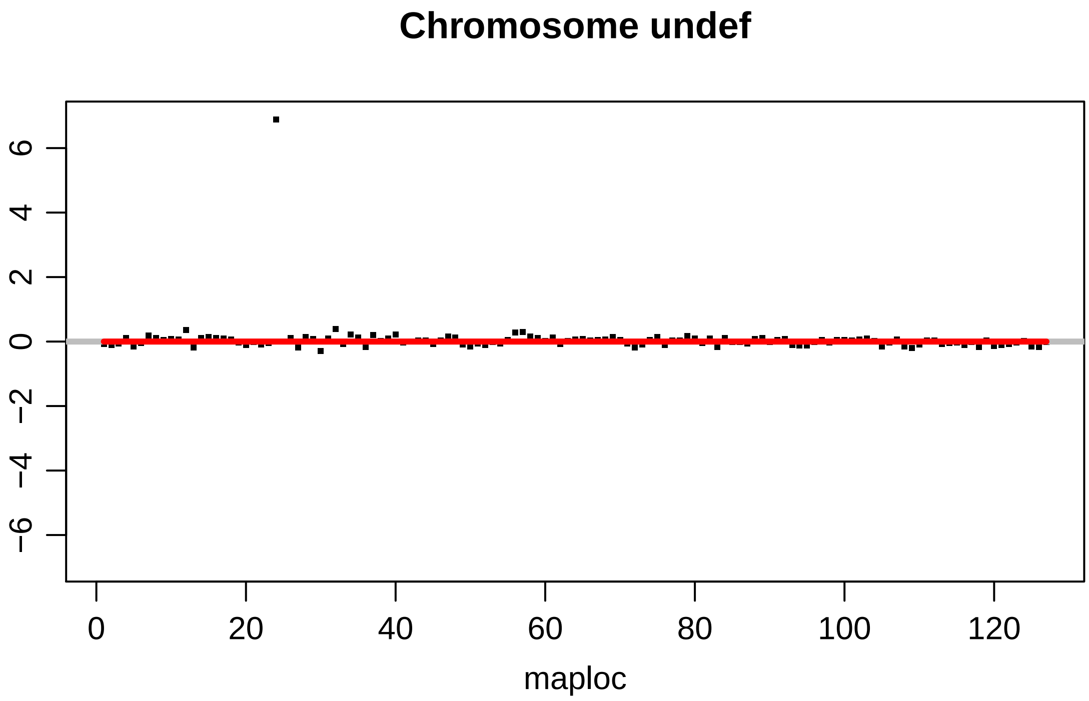


```
## Segplot might not work because of special characters in the sample names. Use only A-Z,a-z and 0-9!
##  There is a hidden function cn.mops:::.replaceNames that replaces the names in the "CNVDetectionResul
```

Case_L057.G1.sam

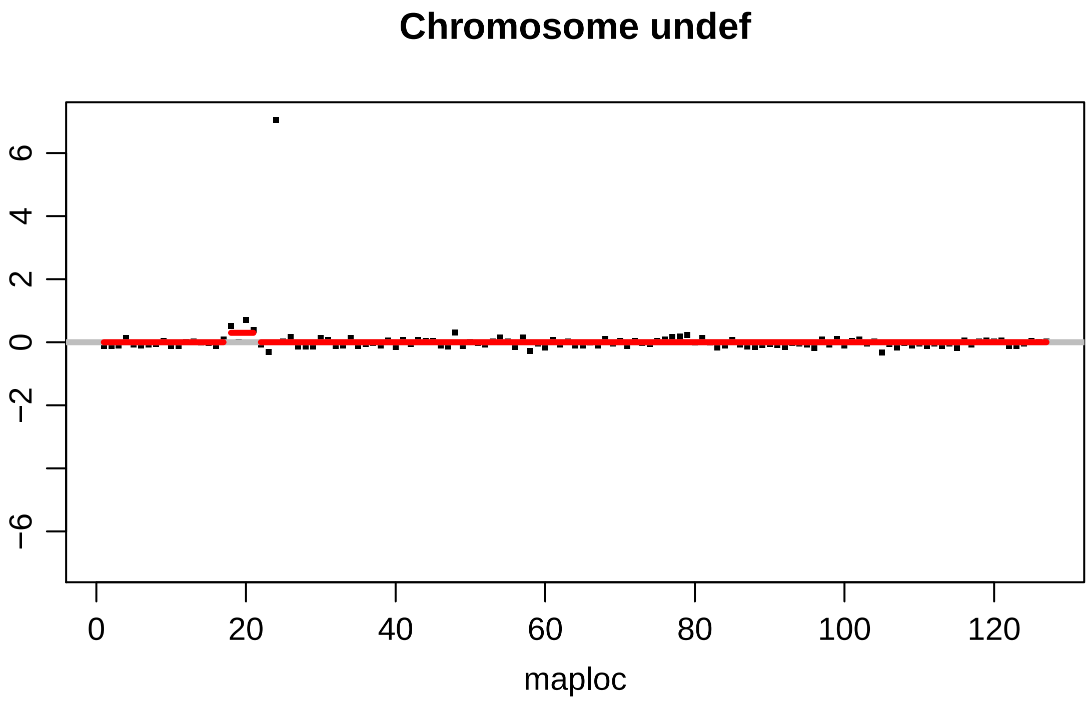

```
## Segplot might not work because of special characters in the sample names. Use only A-Z,a-z and 0-9!
##  There is a hidden function cn.mops:::.replaceNames that replaces the names in the "CNVDetectionResul
```

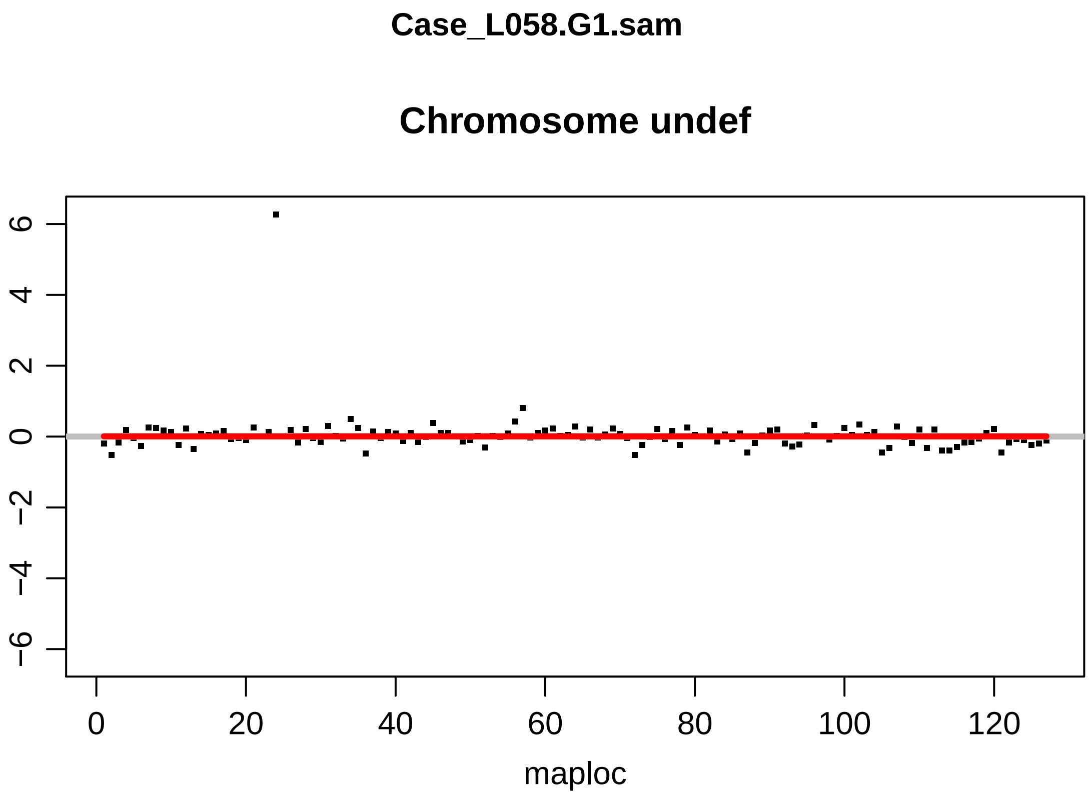


```
## Segplot might not work because of special characters in the sample names. Use only A-Z,a-z and 0-9!
##  There is a hidden function cn.mops:::.replaceNames that replaces the names in the "CNVDetectionResul
```

Case_L059.G1.sam

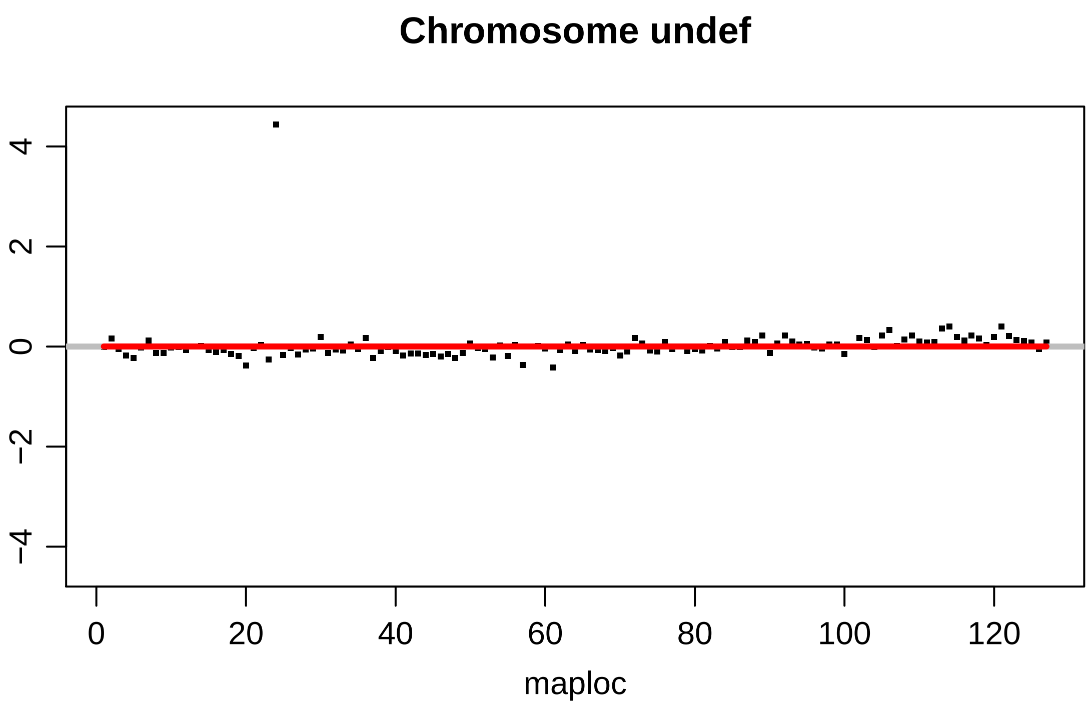


```
## Segplot might not work because of special characters in the sample names. Use only A-Z,a-z and 0-9!
##  There is a hidden function cn.mops:::.replaceNames that replaces the names in the "CNVDetectionResul
```

Case_L060.G1.sam

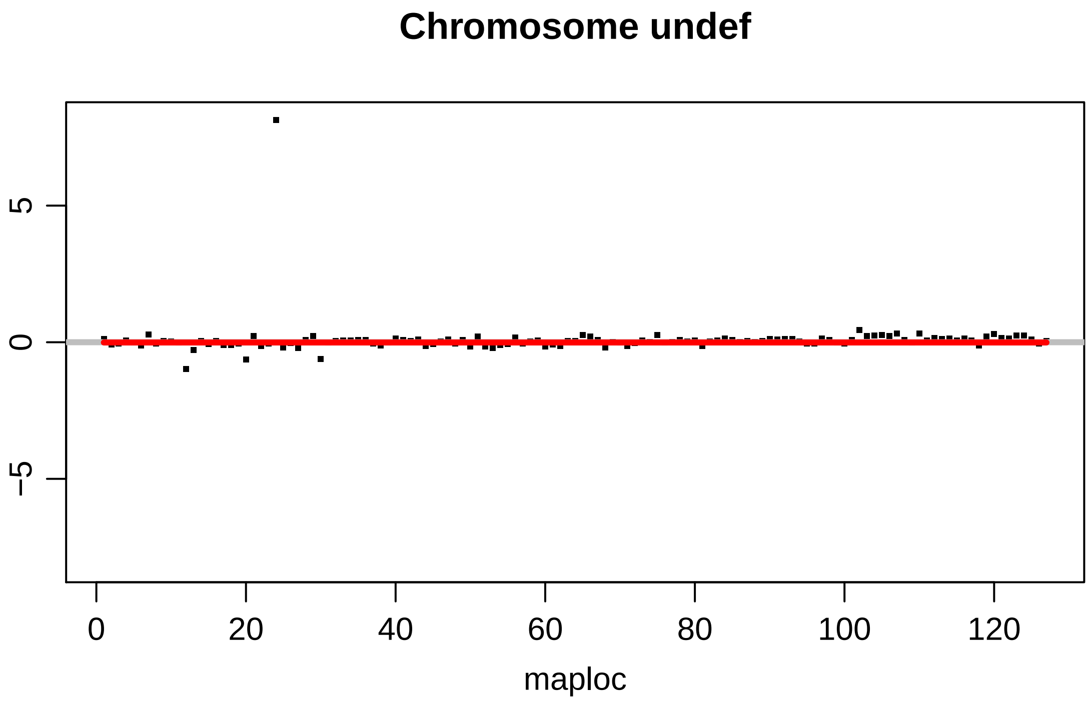

```
## Segplot might not work because of special characters in the sample names. Use only A-Z,a-z and 0-9!
##  There is a hidden function cn.mops:::.replaceNames that replaces the names in the "CNVDetectionResul
```

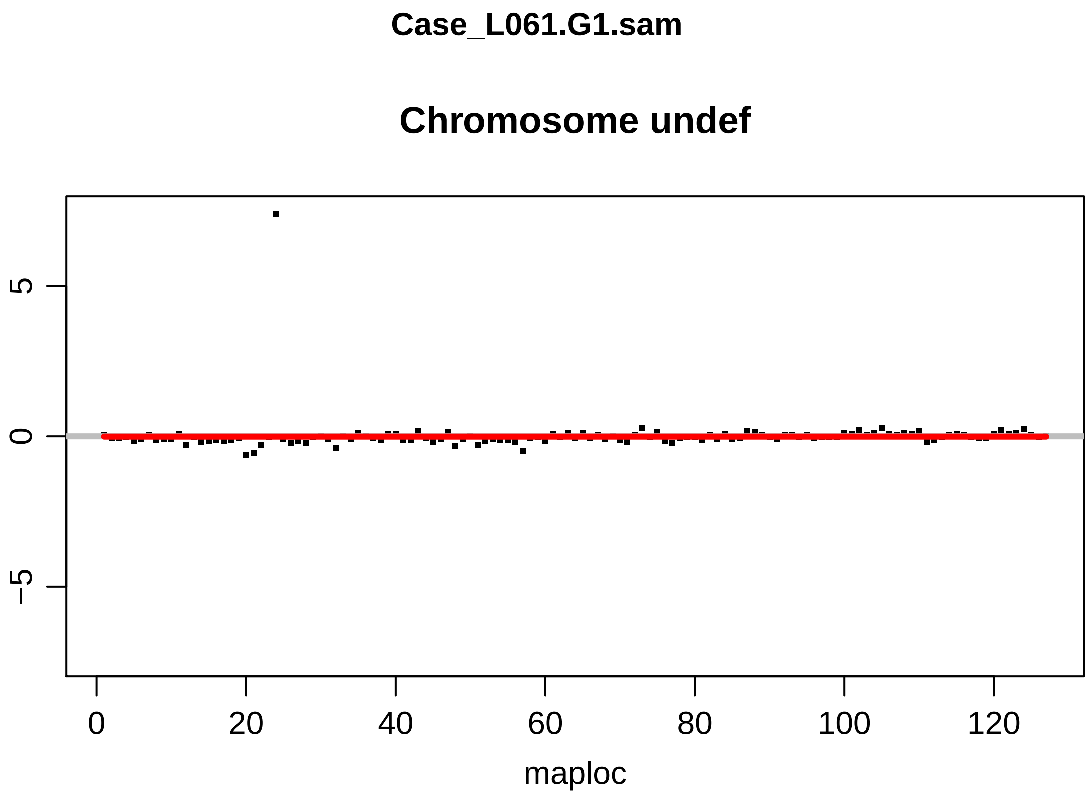


```
## Segplot might not work because of special characters in the sample names. Use only A-Z,a-z and 0-9!
##  There is a hidden function cn.mops:::.replaceNames that replaces the names in the "CNVDetectionResul
```

Case_L062.G1.sam

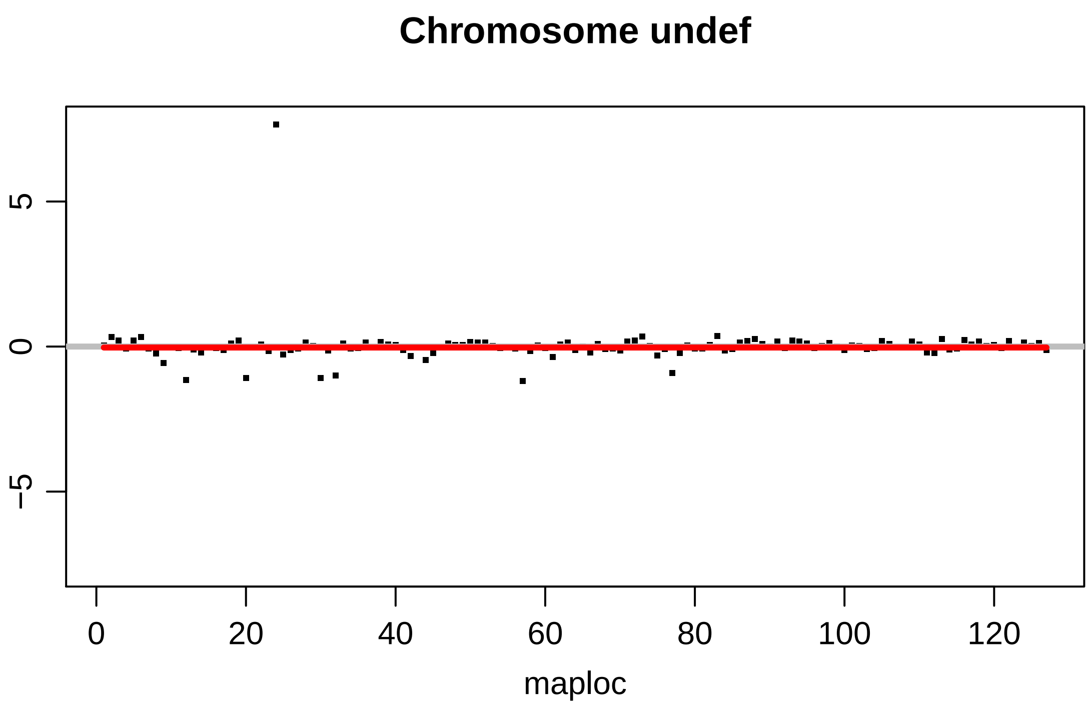


```
## Segplot might not work because of special characters in the sample names. Use only A-Z,a-z and 0-9!
##  There is a hidden function cn.mops:::.replaceNames that replaces the names in the "CNVDetectionResul
```

Case_L063.G1.sam

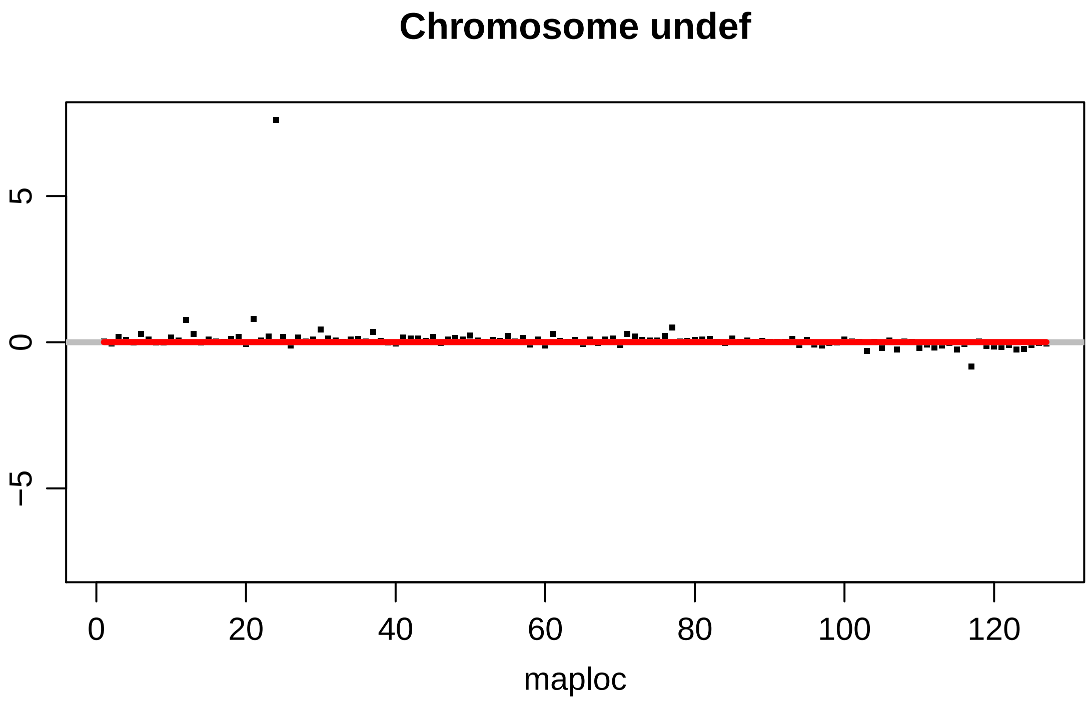

```
## Segplot might not work because of special characters in the sample names. Use only A-Z,a-z and 0-9!
##  There is a hidden function cn.mops:::.replaceNames that replaces the names in the "CNVDetectionResul
```

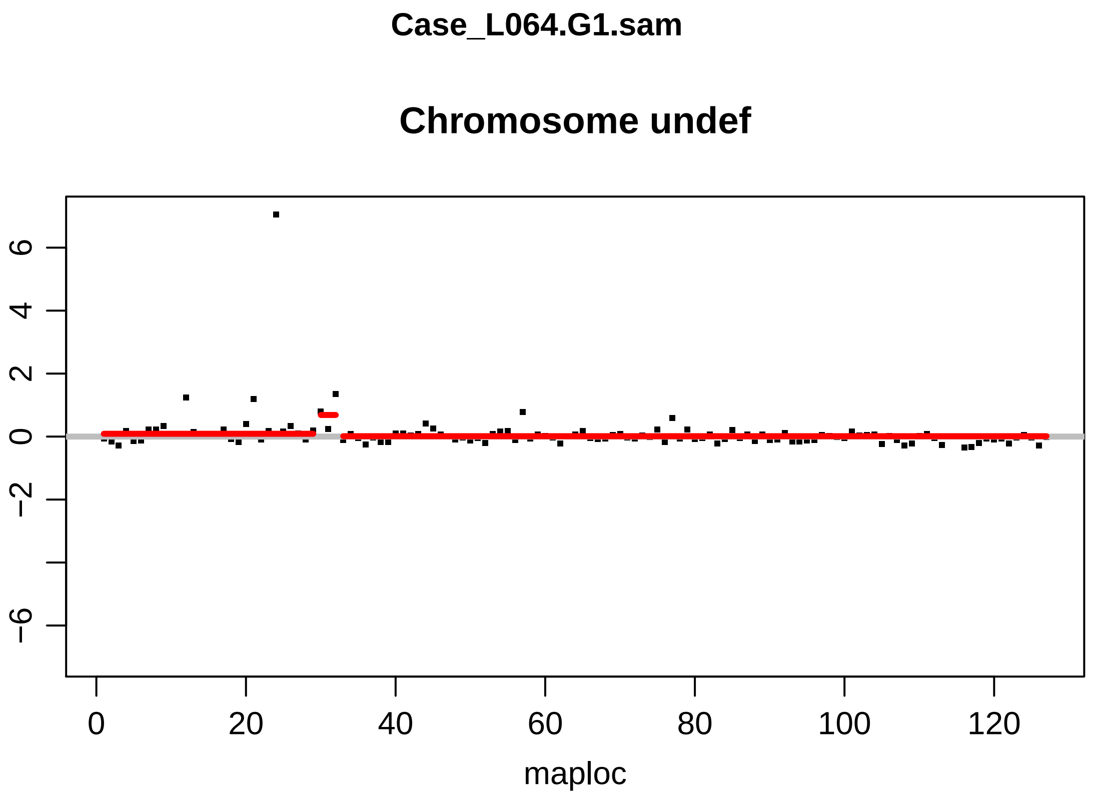


```
## Segplot might not work because of special characters in the sample names. Use only A-Z,a-z and 0-9!
##  There is a hidden function cn.mops:::.replaceNames that replaces the names in the "CNVDetectionResul
```

Case_L065.G1.sam

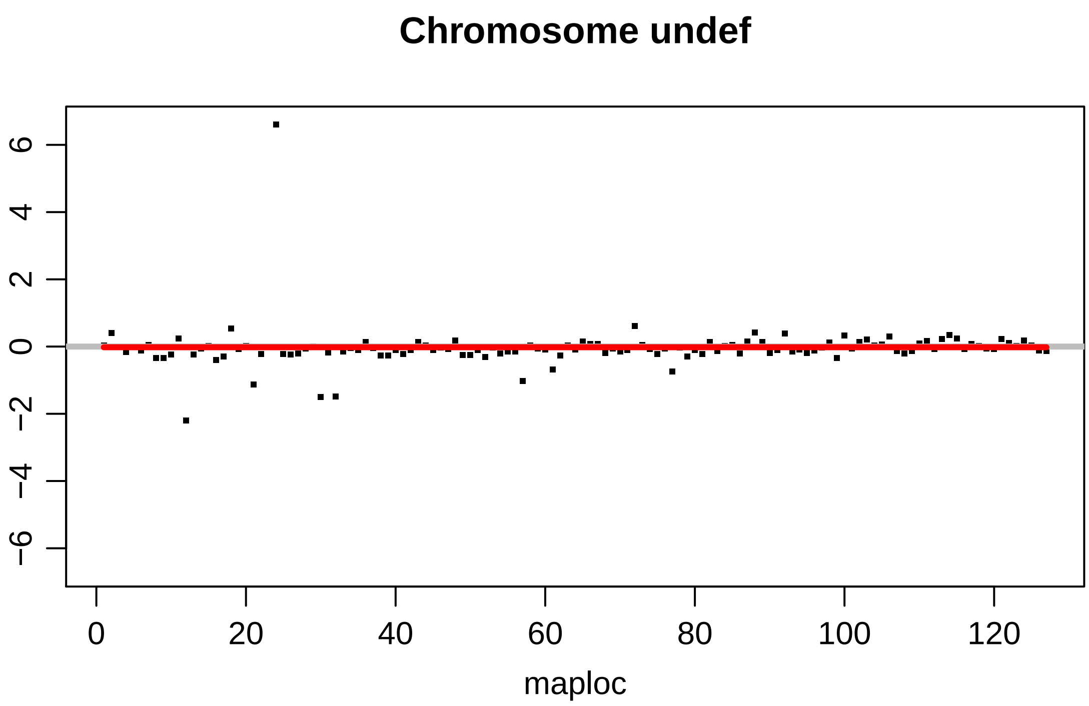


```
## Segplot might not work because of special characters in the sample names. Use only A-Z,a-z and 0-9!
##  There is a hidden function cn.mops:::.replaceNames that replaces the names in the "CNVDetectionResul
```

Case_L066.G1.sam

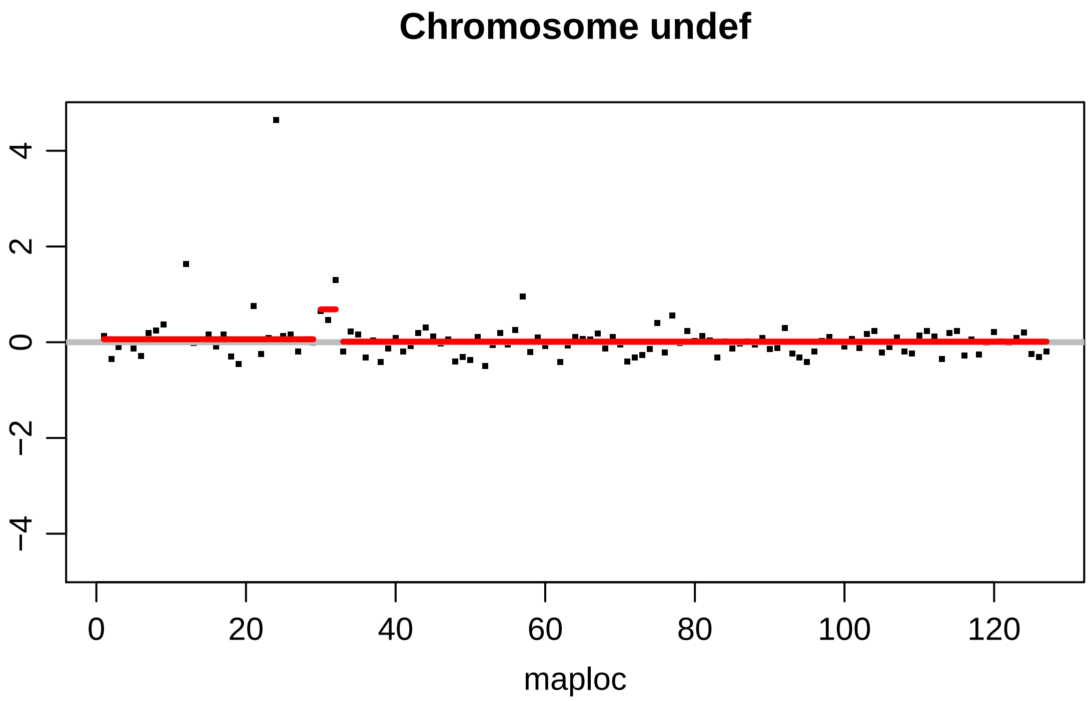

```
## Segplot might not work because of special characters in the sample names. Use only A-Z,a-z and 0-9!
##  There is a hidden function cn.mops:::.replaceNames that replaces the names in the "CNVDetectionResul
```

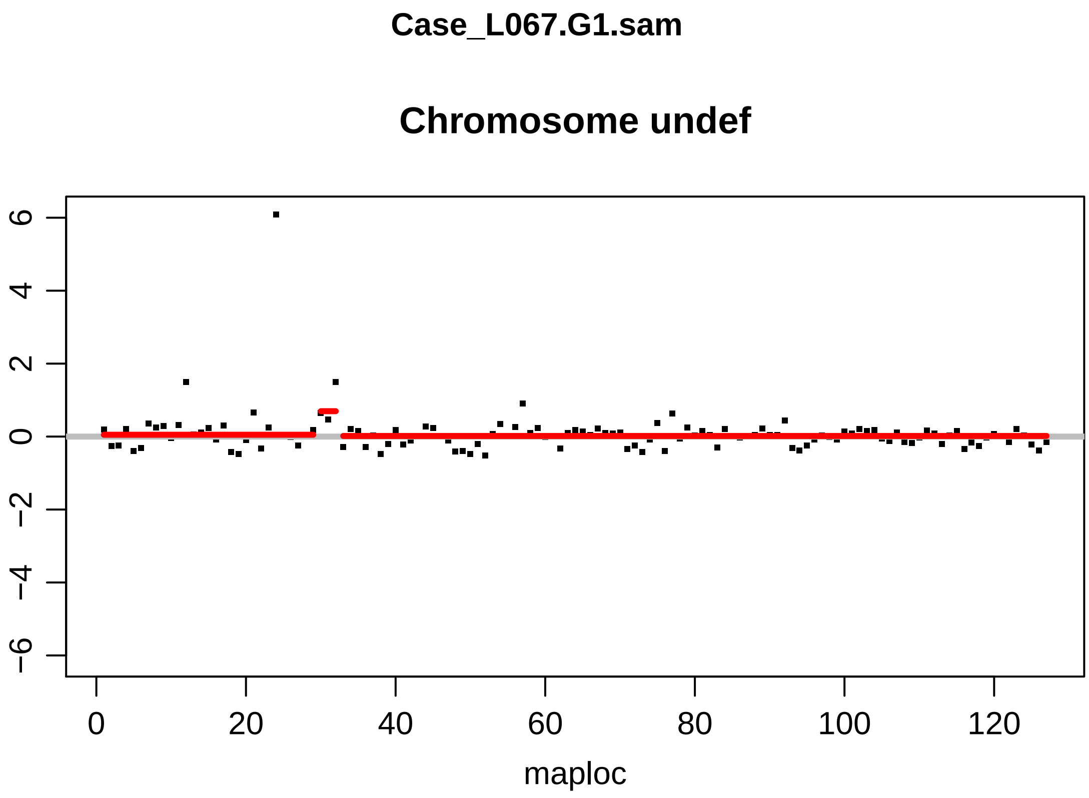


```
## Segplot might not work because of special characters in the sample names. Use only A-Z,a-z and 0-9!
##  There is a hidden function cn.mops:::.replaceNames that replaces the names in the "CNVDetectionResul
```

Case_L068.G1.sam

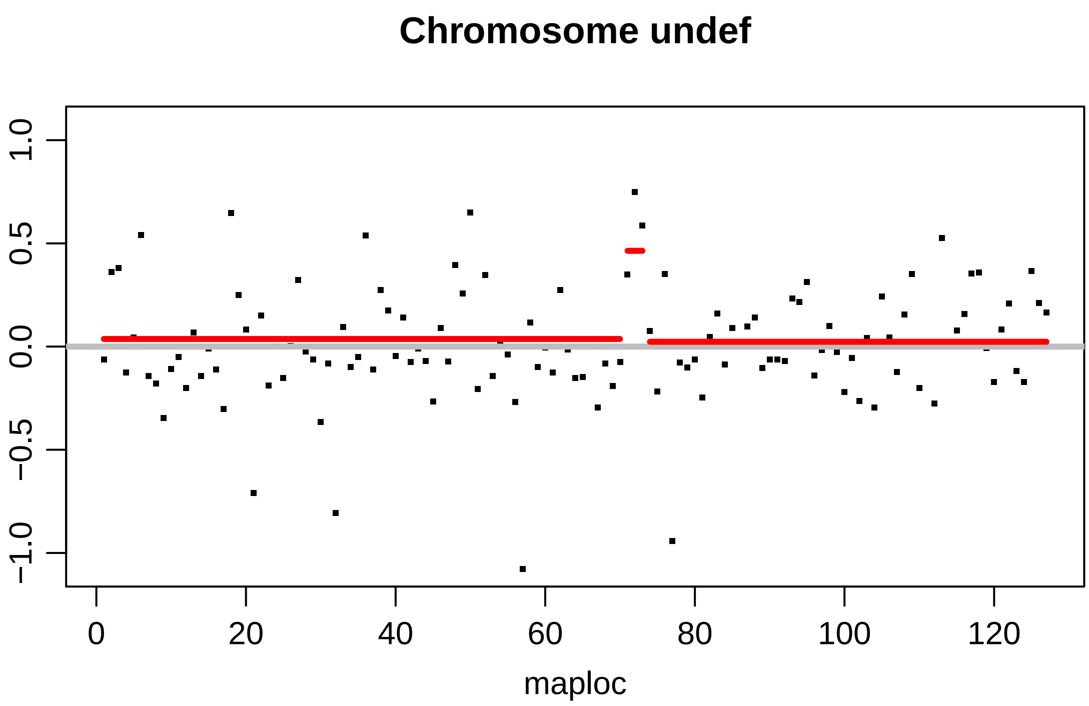


```
## Segplot might not work because of special characters in the sample names. Use only A-Z,a-z and 0-9!
##  There is a hidden function cn.mops:::.replaceNames that replaces the names in the "CNVDetectionResul
```

Case_L069.G1.sam

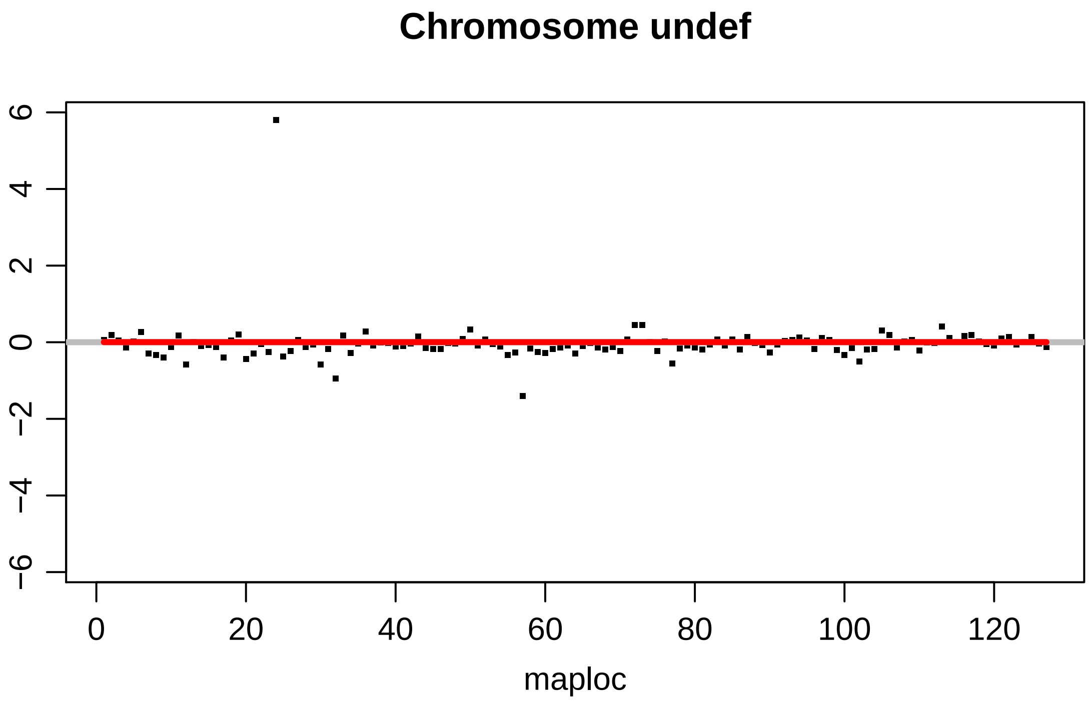

```
## Segplot might not work because of special characters in the sample names. Use only A-Z,a-z and 0-9!
##  There is a hidden function cn.mops:::.replaceNames that replaces the names in the "CNVDetectionResul
```

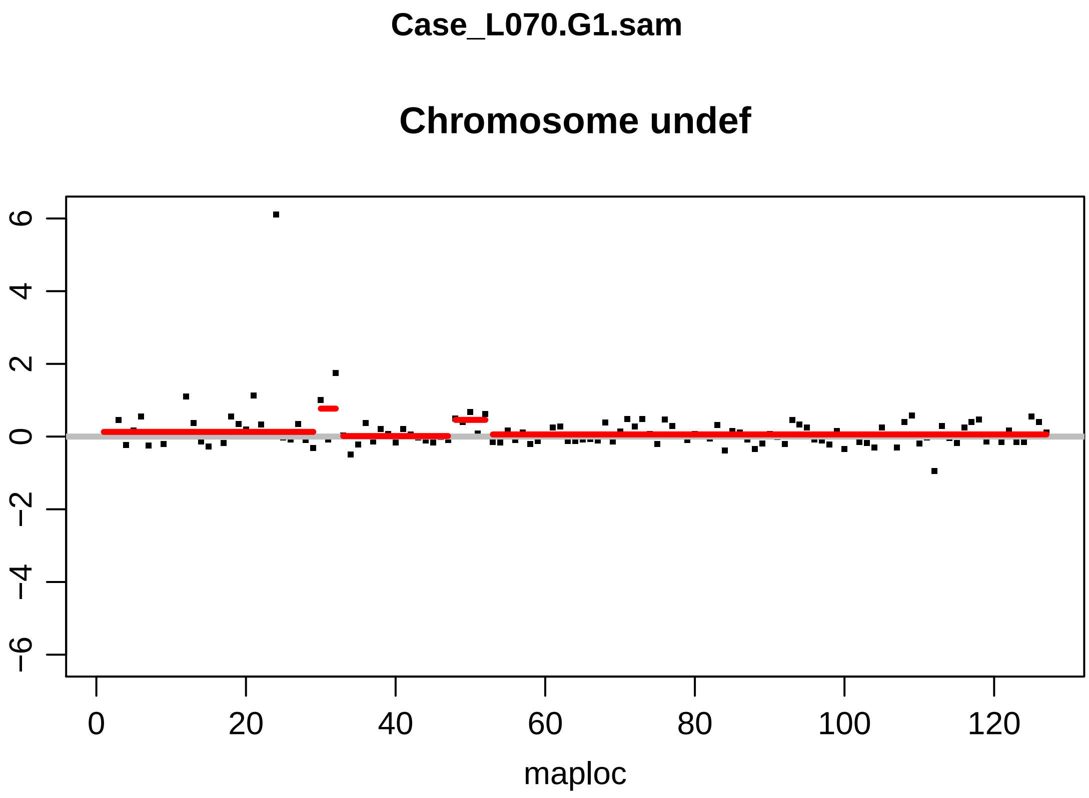


```
## Segplot might not work because of special characters in the sample names. Use only A-Z,a-z and 0-9!
##  There is a hidden function cn.mops:::.replaceNames that replaces the names in the "CNVDetectionResul
```

Case_L071.G1.sam

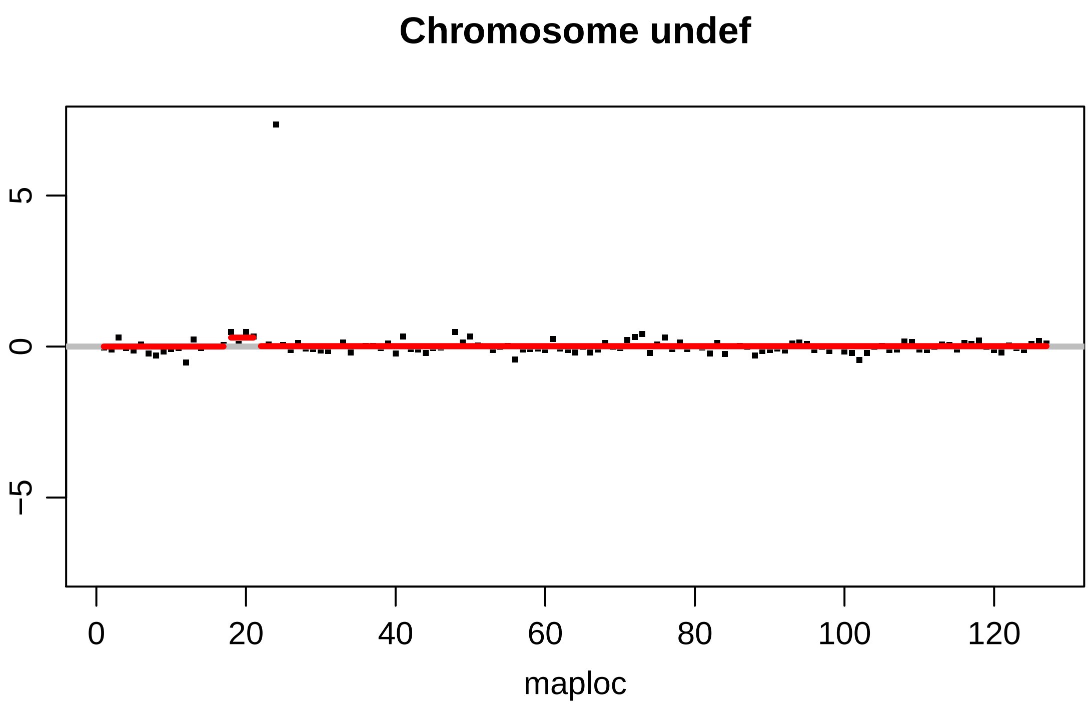


```
## Segplot might not work because of special characters in the sample names. Use only A-Z,a-z and 0-9!
##  There is a hidden function cn.mops:::.replaceNames that replaces the names in the "CNVDetectionResu[illegible]
```

Case_L072.G1.sam

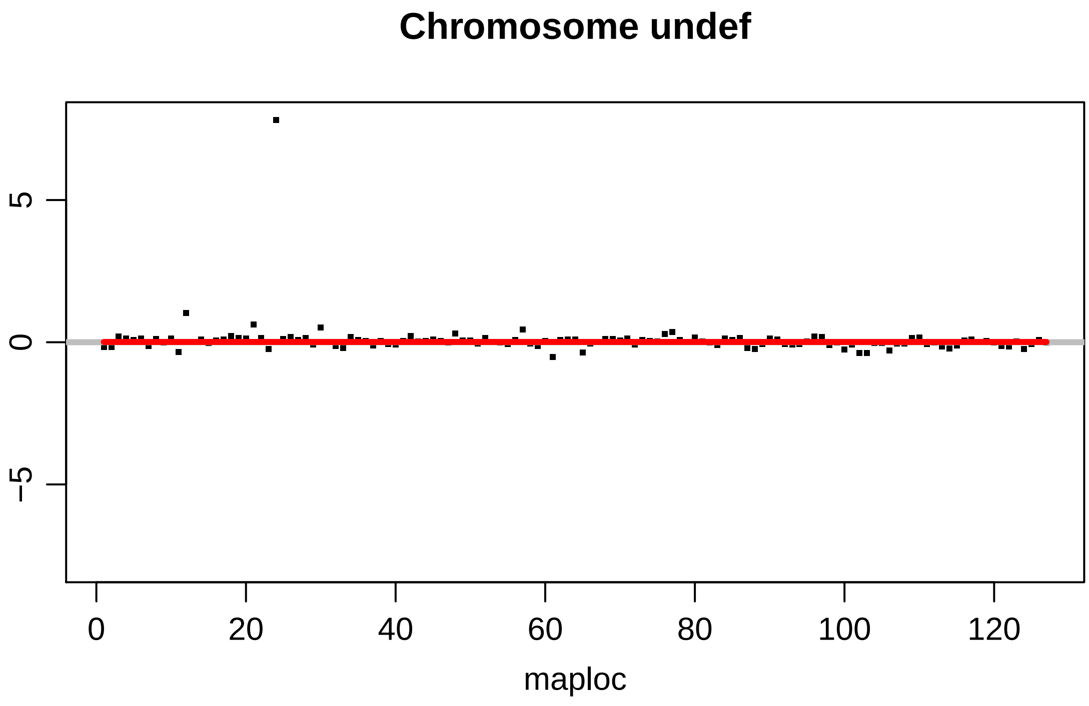

```
## Segplot might not work because of special characters in the sample names. Use only A-Z,a-z and 0-9!
##  There is a hidden function cn.mops:::.replaceNames that replaces the names in the "CNVDetectionResul
```

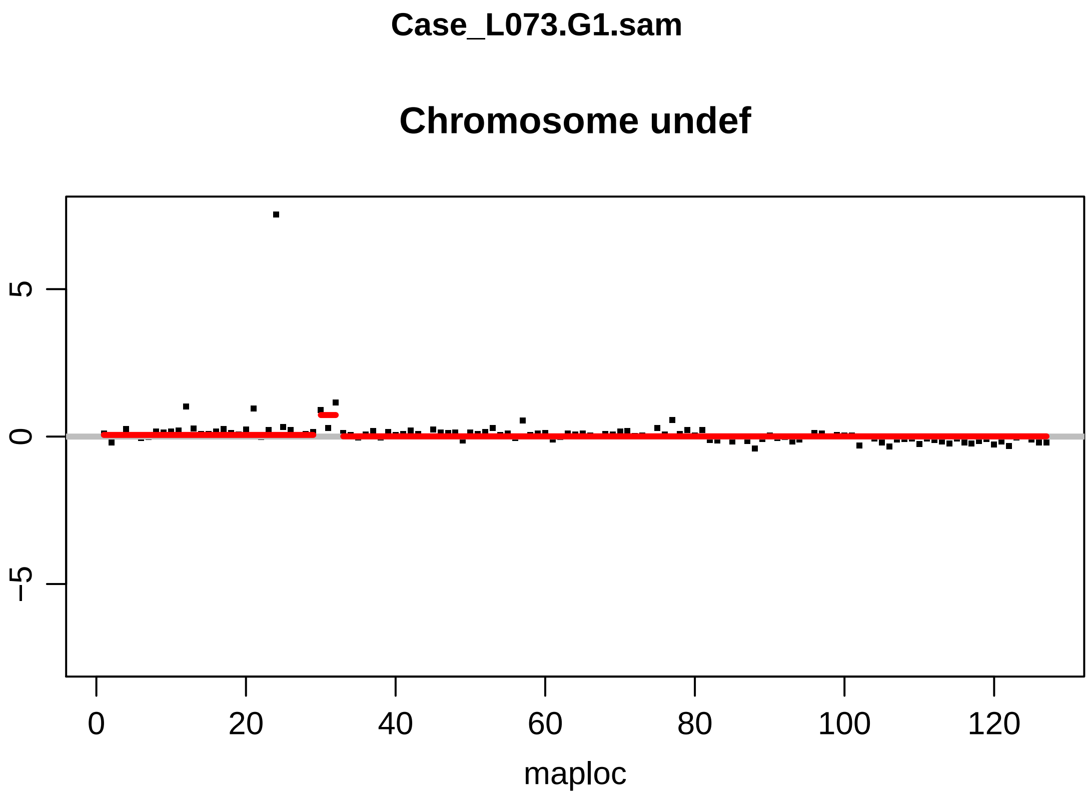


```
## Segplot might not work because of special characters in the sample names. Use only A-Z,a-z and 0-9!
##  There is a hidden function cn.mops:::.replaceNames that replaces the names in the "CNVDetectionResul
```

Case_L074.G1.sam

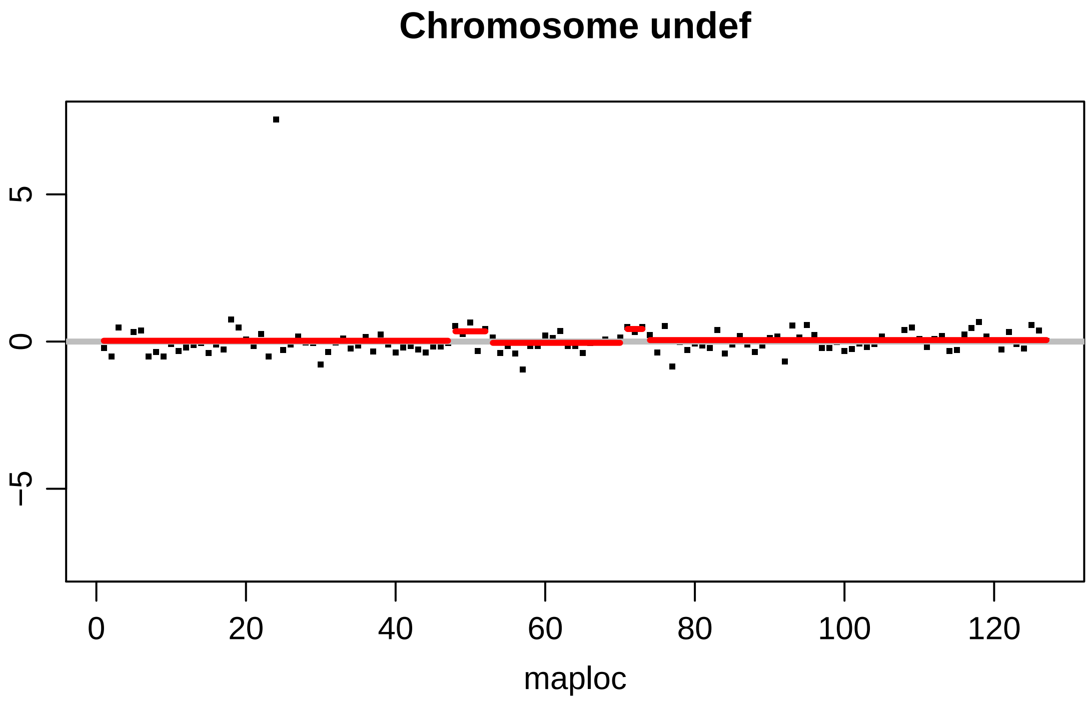


```
## Segplot might not work because of special characters in the sample names. Use only A-Z,a-z and 0-9!
##  There is a hidden function cn.mops:::.replaceNames that replaces the names in the "CNVDetectionResul
```

Case_L075.G1.sam

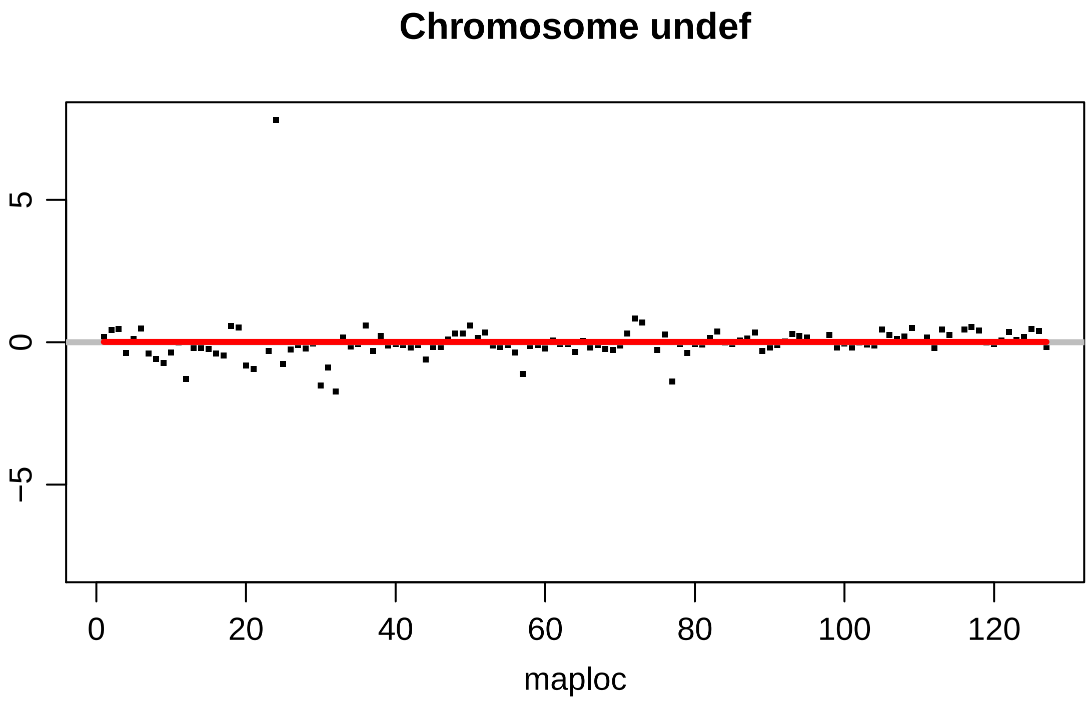

```
## Segplot might not work because of special characters in the sample names. Use only A-Z,a-z and 0-9!
##  There is a hidden function cn.mops:::.replaceNames that replaces the names in the "CNVDetectionResul
```

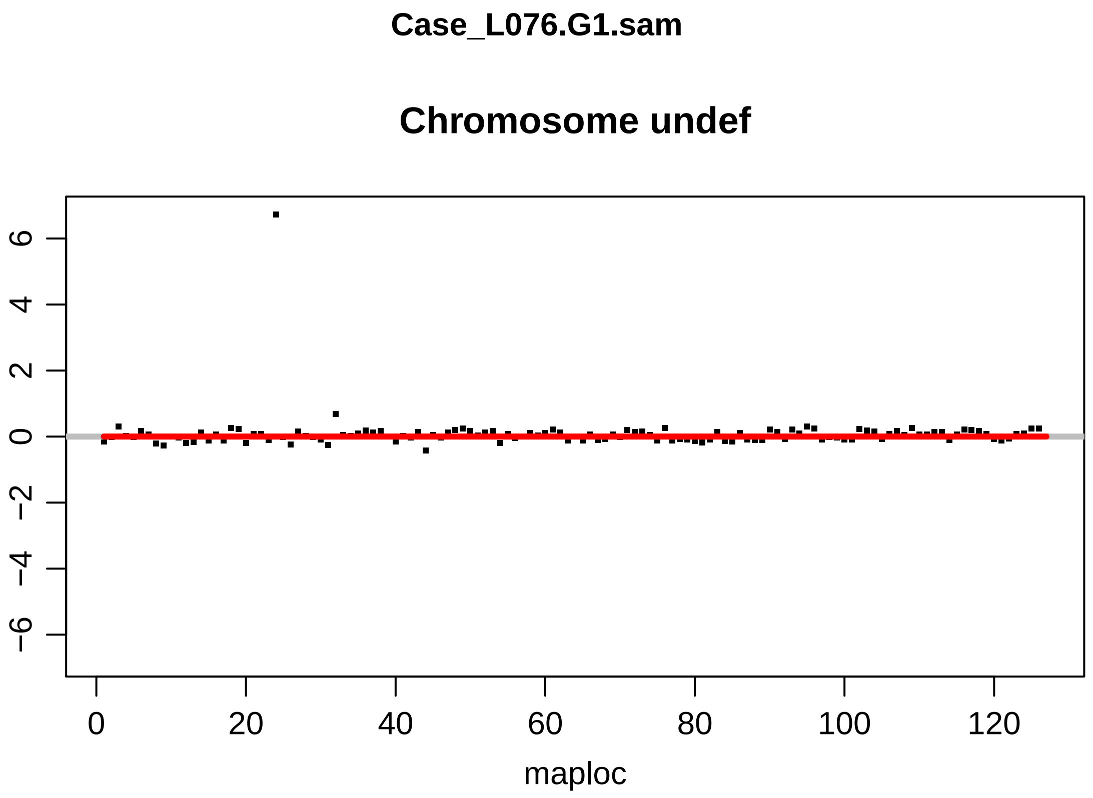


```
## Segplot might not work because of special characters in the sample names. Use only A-Z,a-z and 0-9!
##  There is a hidden function cn.mops:::.replaceNames that replaces the names in the "CNVDetectionResul
```

Case_L077.G1.sam

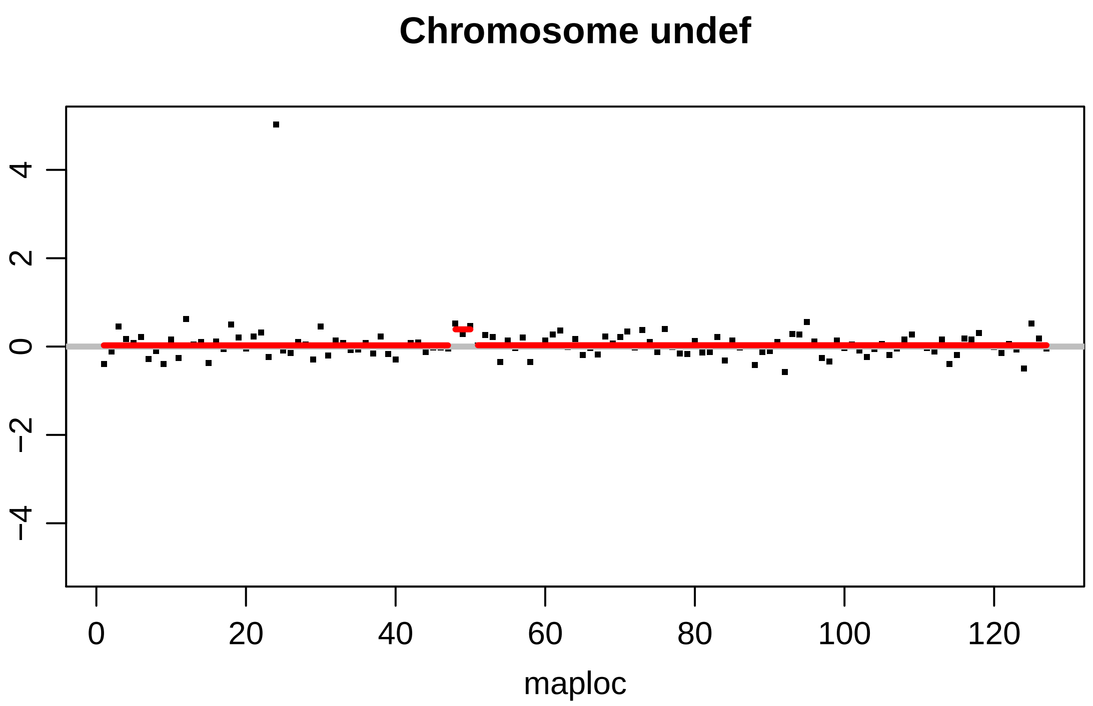


```
## Segplot might not work because of special characters in the sample names. Use only A-Z,a-z and 0-9!
##  There is a hidden function cn.mops:::.replaceNames that replaces the names in the "CNVDetectionResul
```

Case_L079.G1.sam

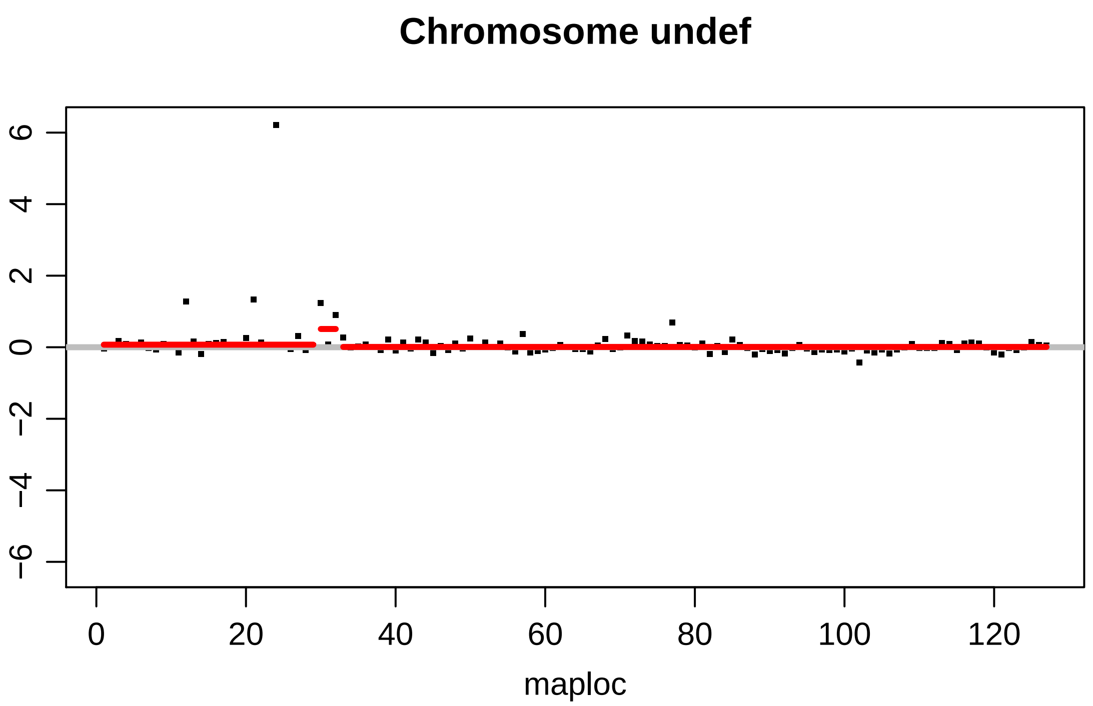

```
## Segplot might not work because of special characters in the sample names. Use only A-Z,a-z and 0-9!
##  There is a hidden function cn.mops:::.replaceNames that replaces the names in the "CNVDetectionResul
```

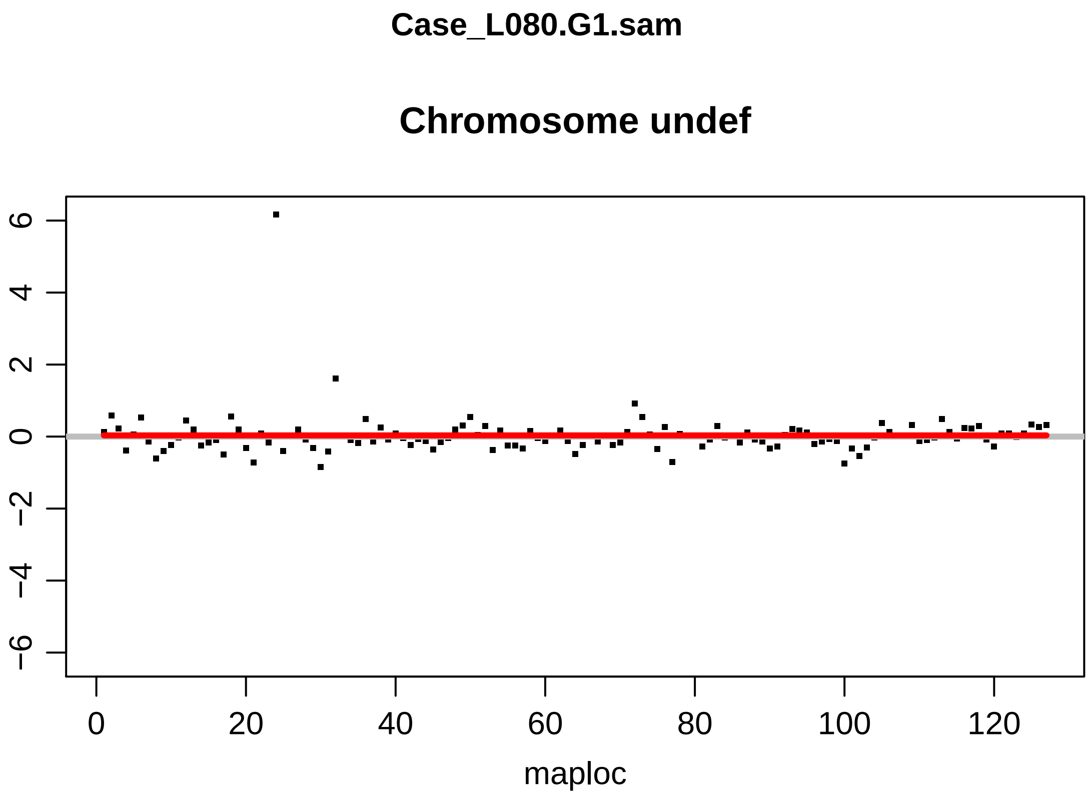


```
## Segplot might not work because of special characters in the sample names. Use only A-Z,a-z and 0-9!
##  There is a hidden function cn.mops:::.replaceNames that replaces the names in the "CNVDetectionResul
```

Case_L081.G1.sam

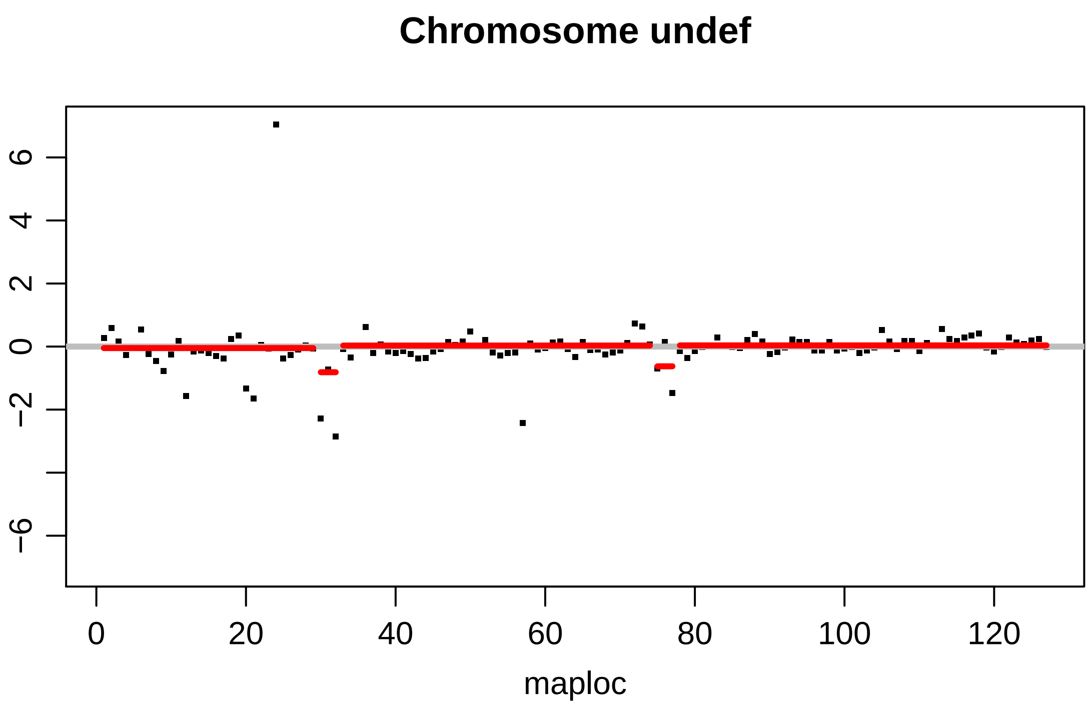


```
## Segplot might not work because of special characters in the sample names. Use only A-Z,a-z and 0-9!
##  There is a hidden function cn.mops:::.replaceNames that replaces the names in the "CNVDetectionResul
```

Case_L082.G1.sam

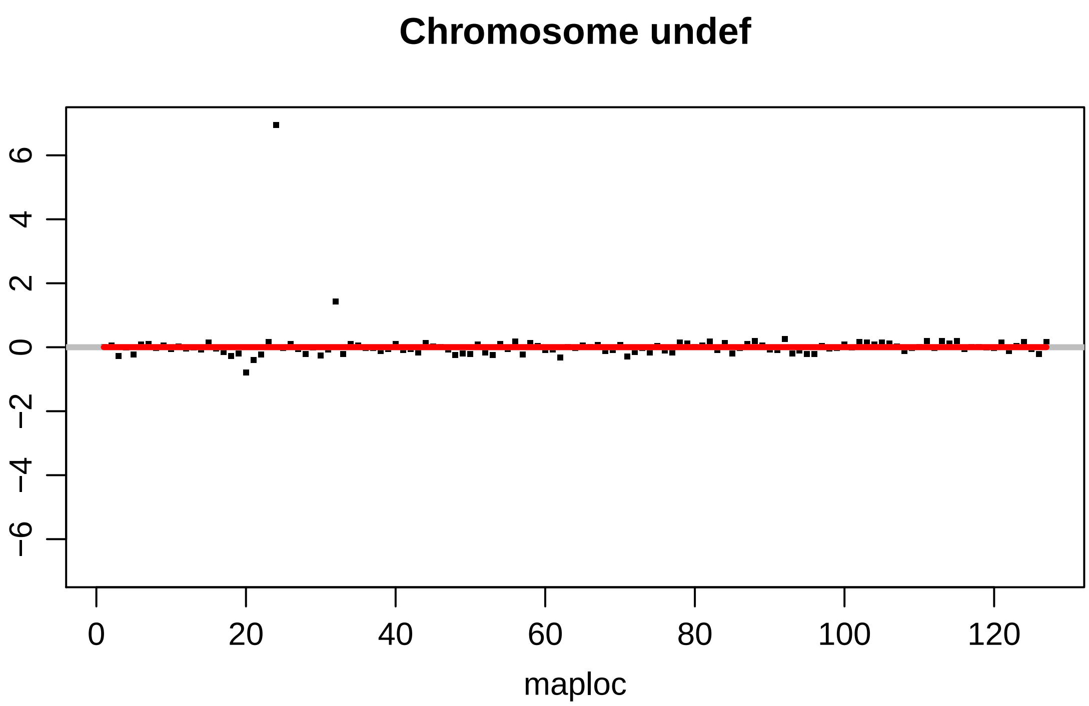

```
## Segplot might not work because of special characters in the sample names. Use only A-Z,a-z and 0-9!
##  There is a hidden function cn.mops:::.replaceNames that replaces the names in the "CNVDetectionResul
```

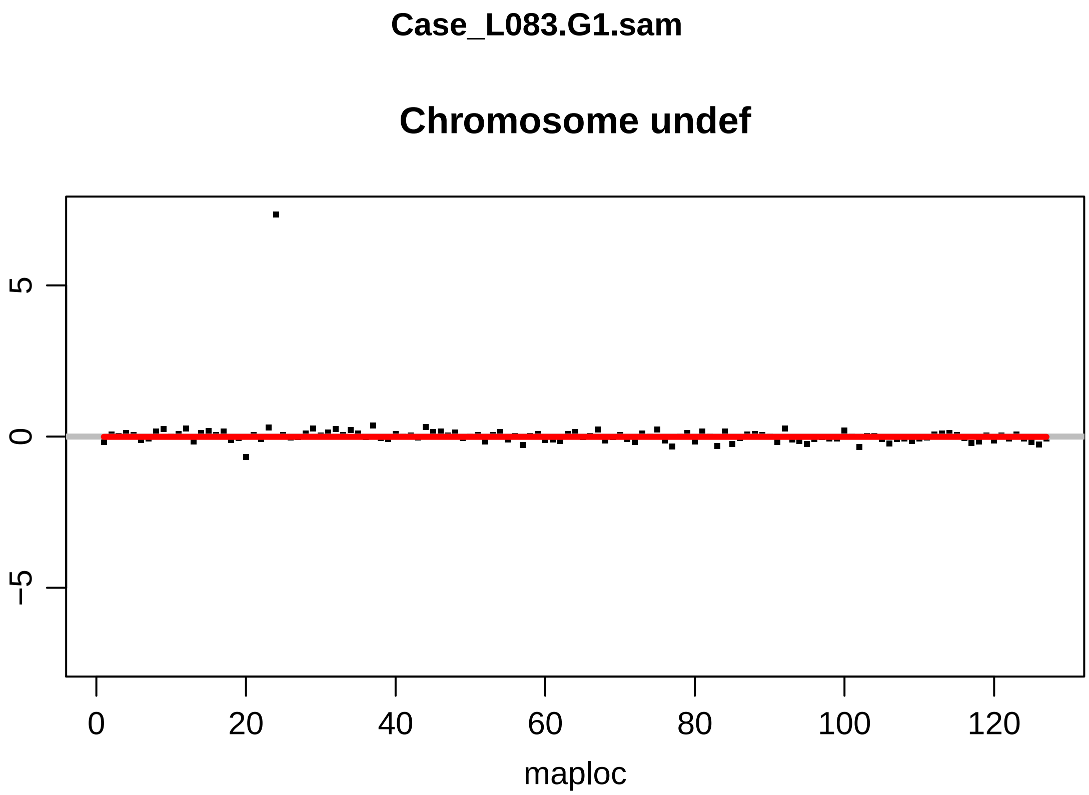


```
## Segplot might not work because of special characters in the sample names. Use only A-Z,a-z and 0-9!
##  There is a hidden function cn.mops:::.replaceNames that replaces the names in the "CNVDetectionResul
```

Case_L084.G1.sam

Chromosome undef

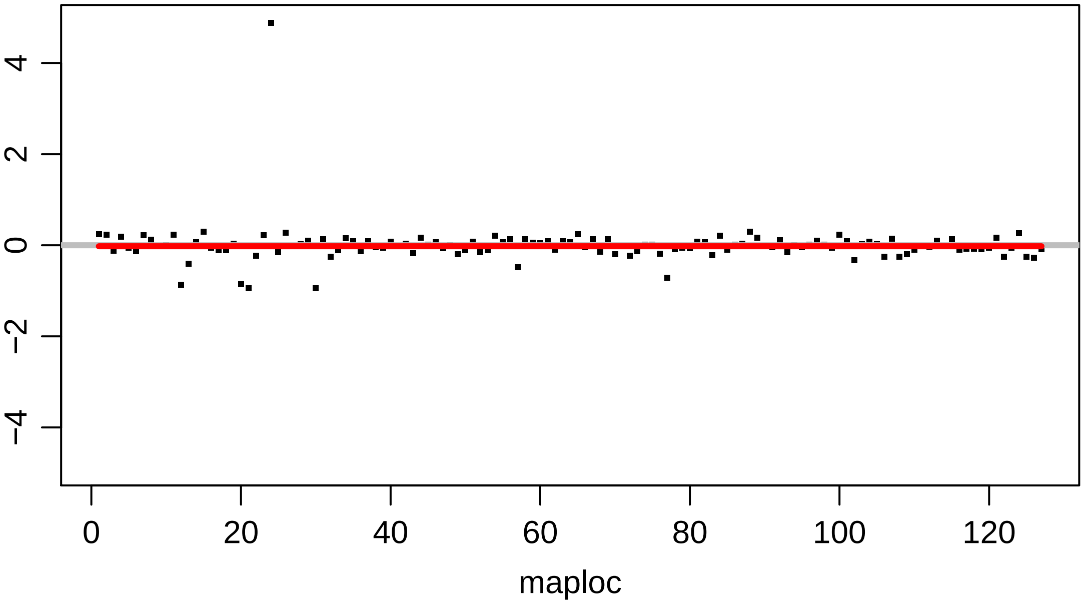


```
## Segplot might not work because of special characters in the sample names. Use only A-Z,a-z and 0-9!
##  There is a hidden function cn.mops:::.replaceNames that replaces the names in the "CNVDetectionResul
```

Case_L085.G1.sam

Chromosome undef

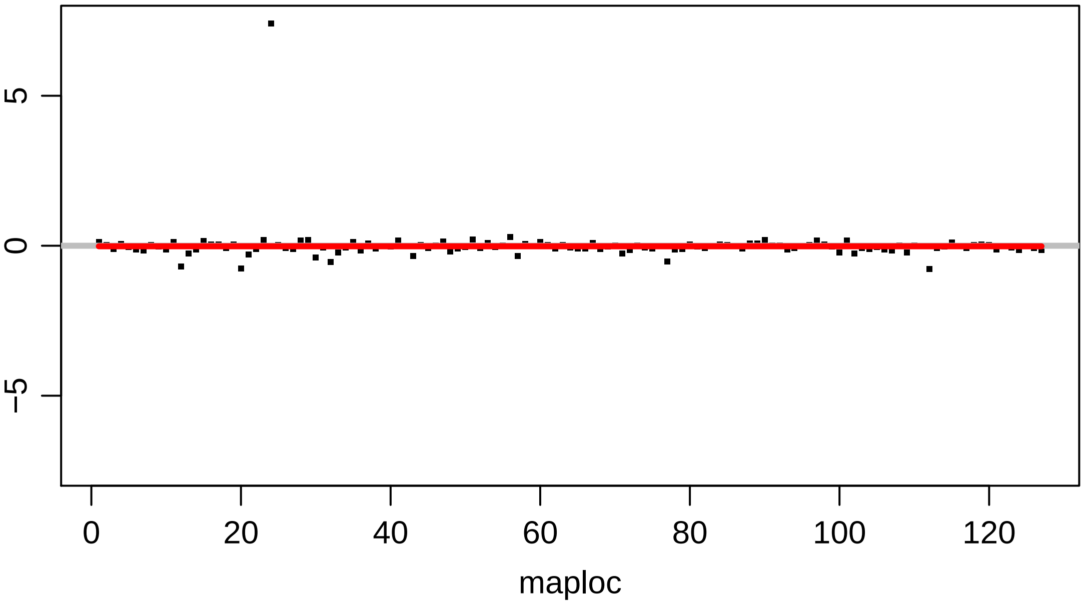

Segplot might not work because of special characters in the sample names. Use only A-Z,a-z and 0-9!
There is a hidden function cn.mops:::.replaceNames that replaces the names in the "CNVDetectionResul

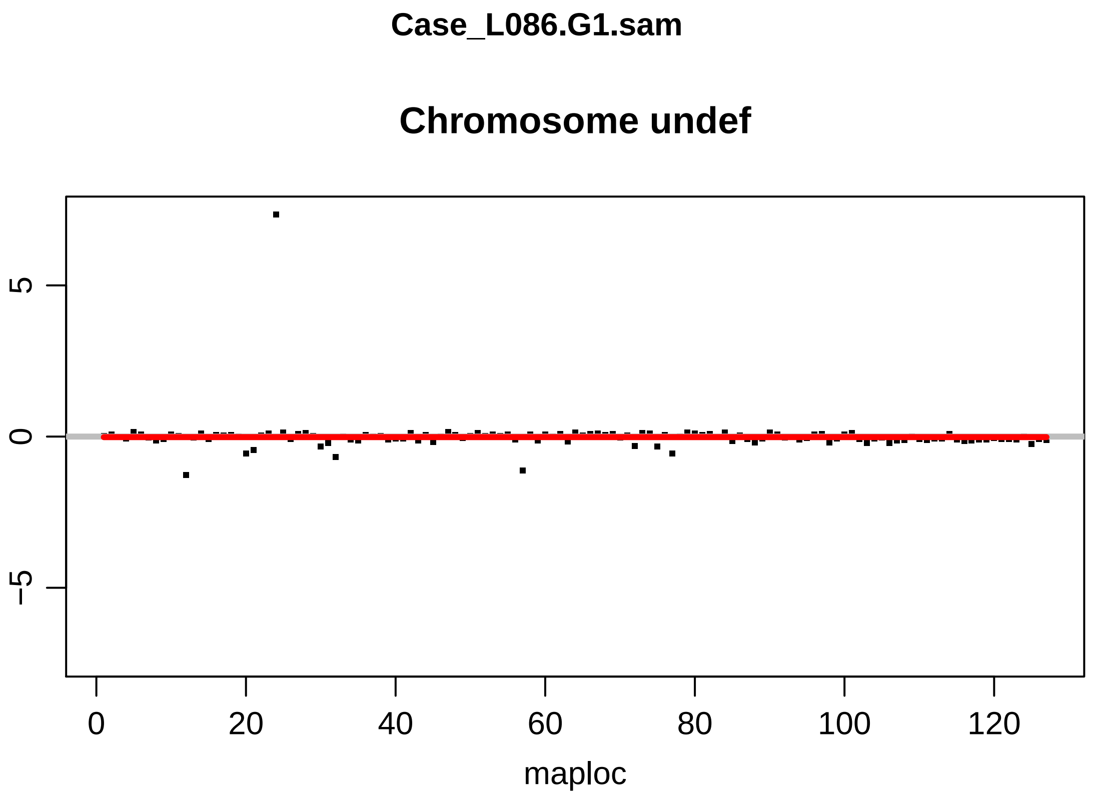

Segplot might not work because of special characters in the sample names. Use only A-Z,a-z and 0-9!
There is a hidden function cn.mops:::.replaceNames that replaces the names in the "CNVDetectionResul

Case_L087.G1.sam

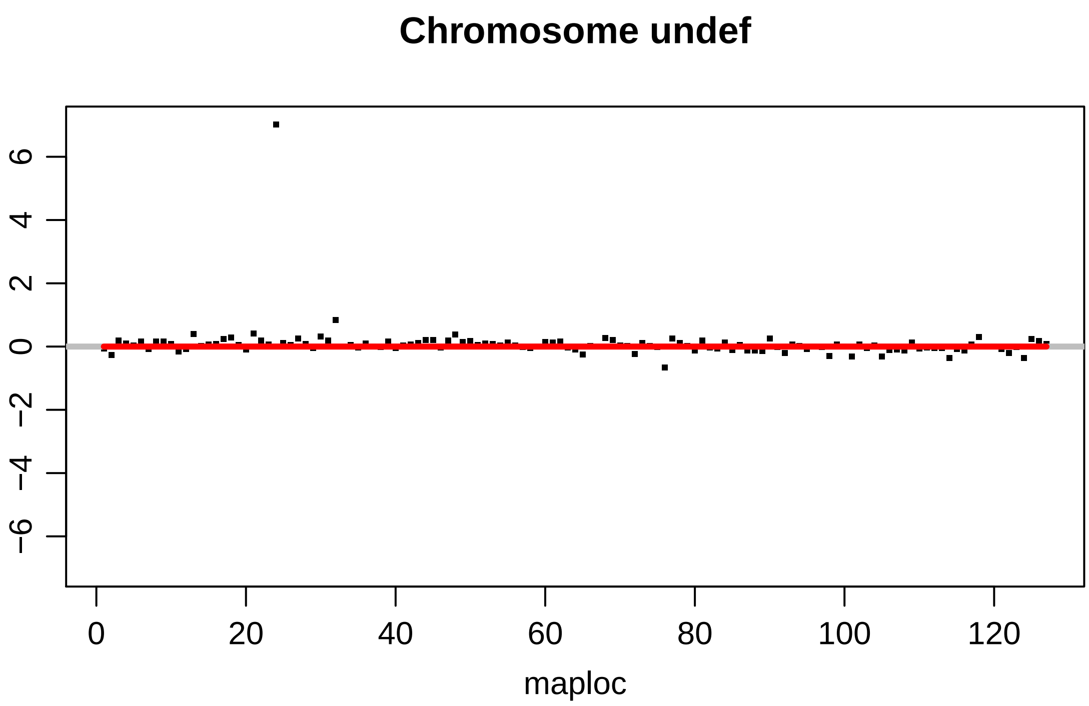


```
## Segplot might not work because of special characters in the sample names. Use only A-Z,a-z and 0-9!
##  There is a hidden function cn.mops:::.replaceNames that replaces the names in the "CNVDetectionResul
```

Case_L088.G1.sam

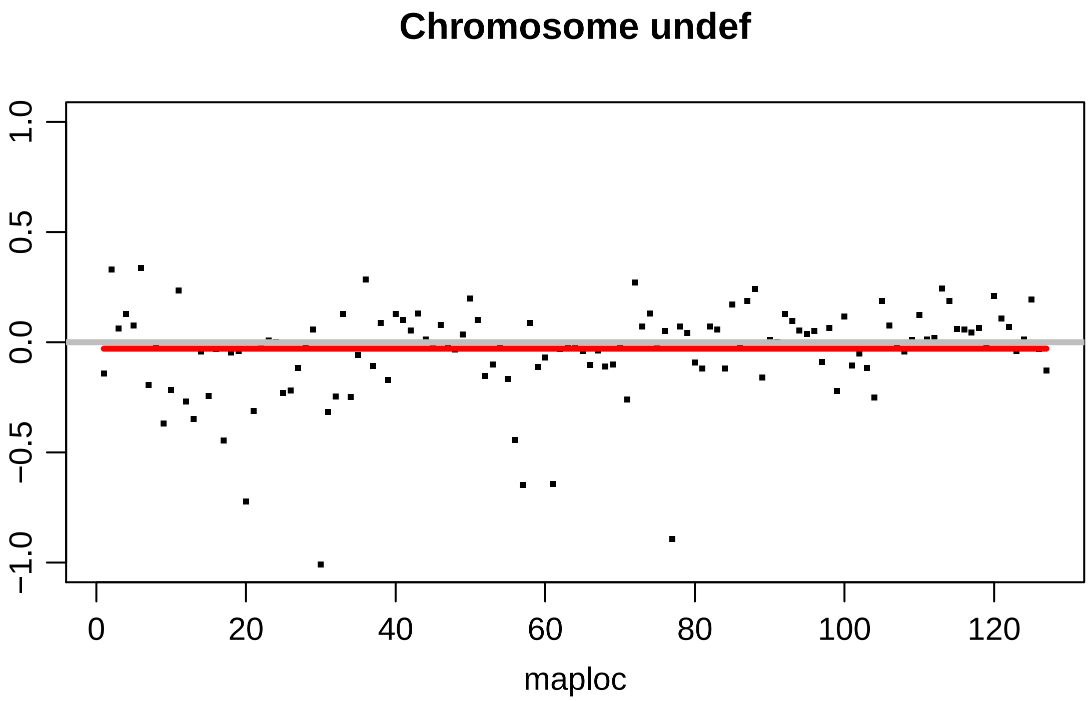

```
## Segplot might not work because of special characters in the sample names. Use only A-Z,a-z and 0-9!
##  There is a hidden function cn.mops:::.replaceNames that replaces the names in the "CNVDetectionResul
```

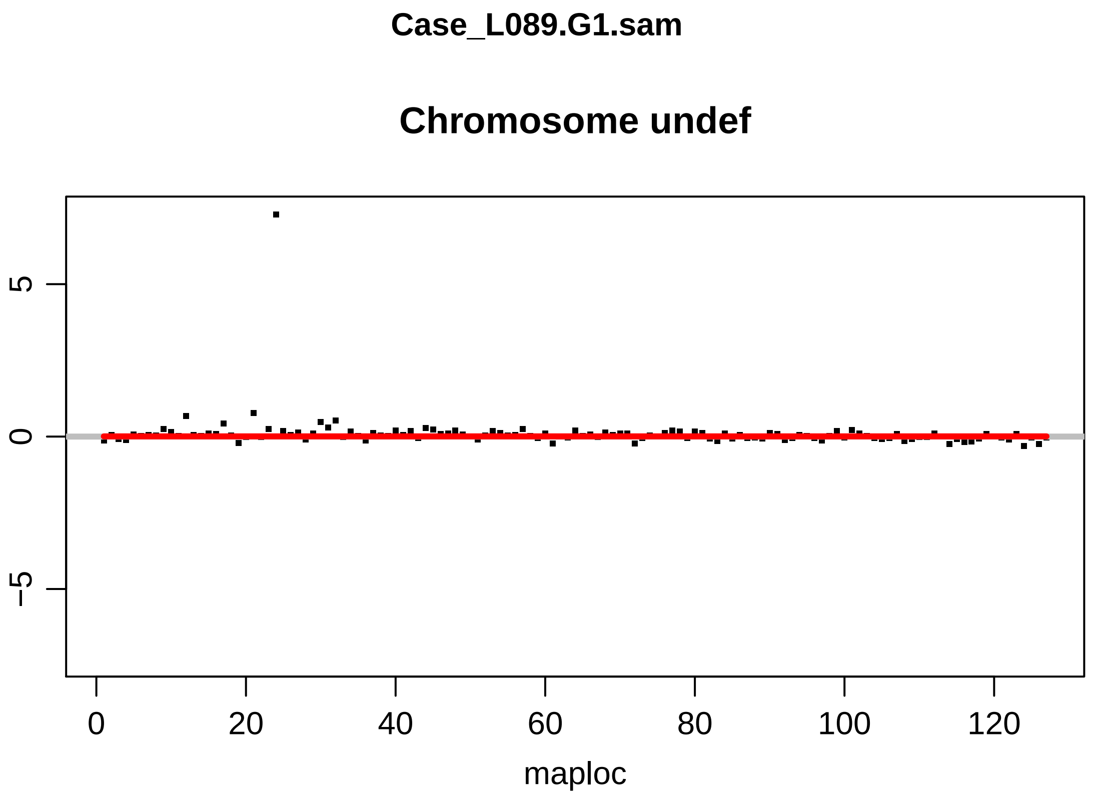


```
## Segplot might not work because of special characters in the sample names. Use only A-Z,a-z and 0-9!
##  There is a hidden function cn.mops:::.replaceNames that replaces the names in the "CNVDetectionResul
```

Case_L090.G1.sam

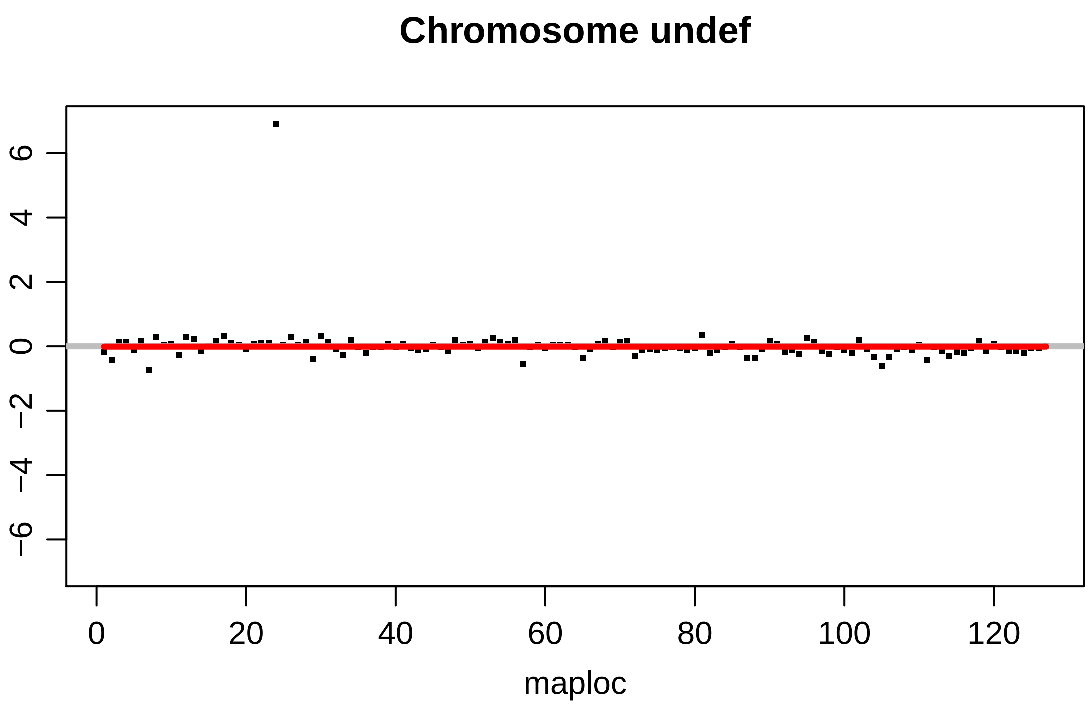


```
## Segplot might not work because of special characters in the sample names. Use only A-Z,a-z and 0-9!
##  There is a hidden function cn.mops:::.replaceNames that replaces the names in the "CNVDetectionResul
```

Case_L091.G1.sam

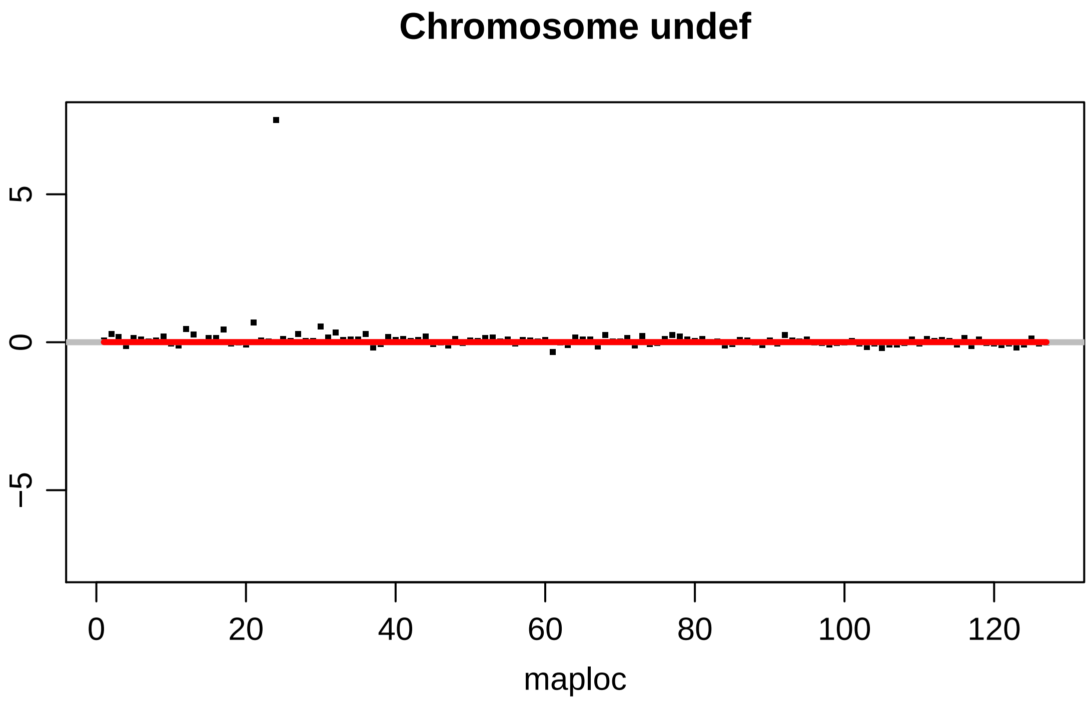

```
## Segplot might not work because of special characters in the sample names. Use only A-Z,a-z and 0-9!
##  There is a hidden function cn.mops:::.replaceNames that replaces the names in the "CNVDetectionResul
```

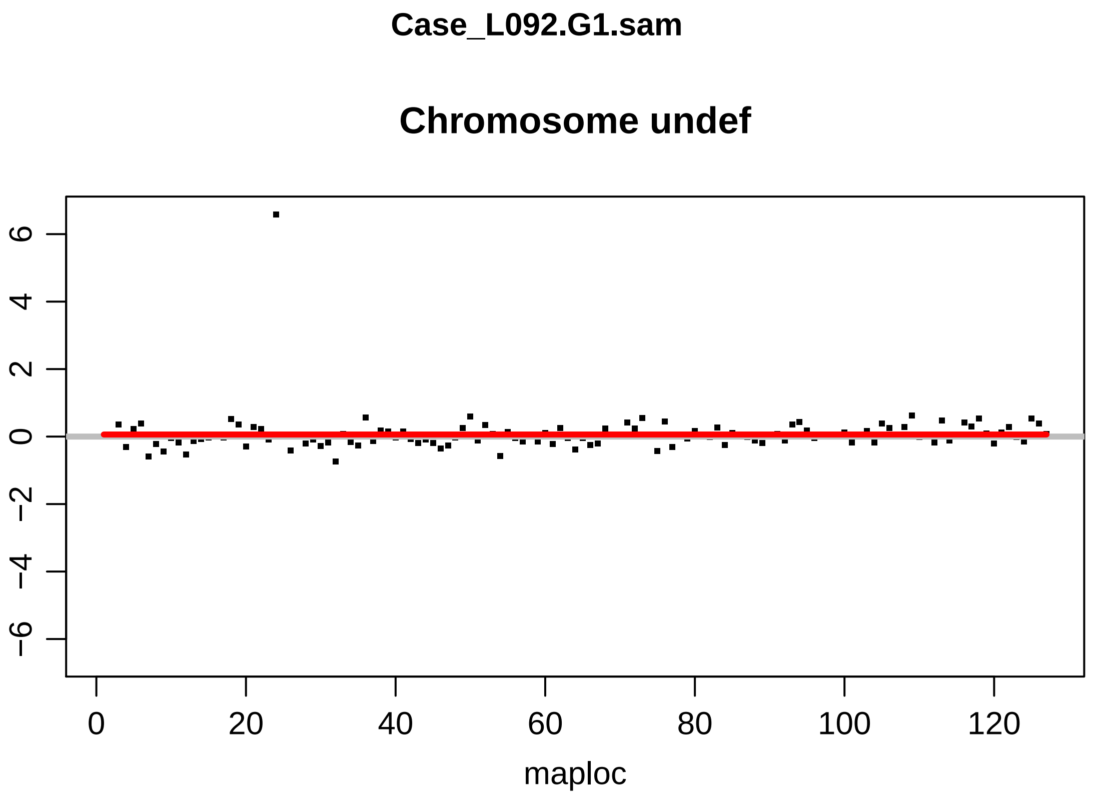


```
## Segplot might not work because of special characters in the sample names. Use only A-Z,a-z and 0-9!
##  There is a hidden function cn.mops:::.replaceNames that replaces the names in the "CNVDetectionResul
```

Case_L093.G1.sam

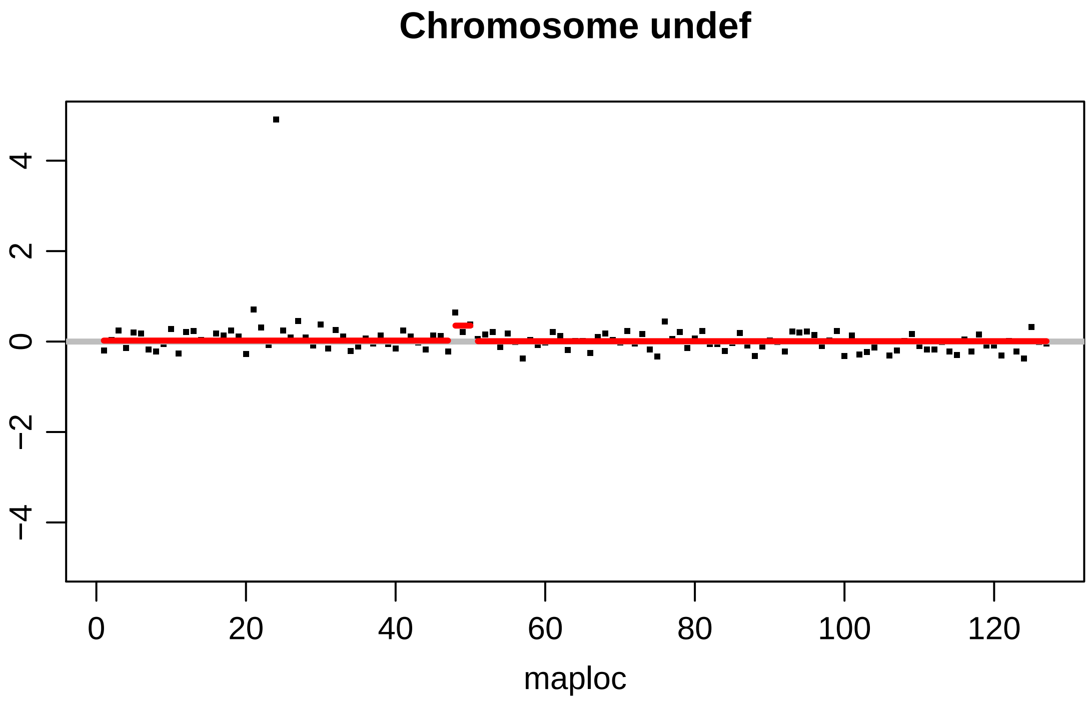


```
##
## CNV regions:
## GRanges object with 2 ranges and 47 metadata columns:
##       seqnames    ranges strand | Case_CONTROL.blood.sam Case_L048.G1.sam
##          <Rle> <IRanges>  <Rle> |               <factor>         <factor>
##   [1]    undef  [ 6,  9]      * |                    CN2              CN1
##   [2]    undef  [30, 32]      * |                    CN2              CN2
##       Case_L049.G1.sam Case_L050.G1.sam Case_L051.G1.sam Case_L052.G1.sam
##               <factor>         <factor>         <factor>         <factor>
##   [1]              CN2              CN2              CN1              CN2
##   [2]              CN2              CN2              CN2              CN2
##       Case_L053.G1.sam Case_L054.G1.sam Case_L055.G1.sam Case_L056.G1.sam
##               <factor>         <factor>         <factor>         <factor>
##   [1]              CN2              CN2              CN2              CN2
##   [2]              CN2              CN2              CN2              CN2
##       Case_L057.G1.sam Case_L058.G1.sam Case_L059.G1.sam Case_L060.G1.sam
##               <factor>         <factor>         <factor>         <factor>
##   [1]              CN2              CN2              CN2              CN2
##   [2]              CN2              CN2              CN2              CN2
##       Case_L061.G1.sam Case_L062.G1.sam Case_L063.G1.sam Case_L064.G1.sam
##               <factor>         <factor>         <factor>         <factor>
##   [1]              CN2              CN2              CN2              CN2
##   [2]              CN2              CN2              CN2              CN3
##       Case_L065.G1.sam Case_L066.G1.sam Case_L067.G1.sam Case_L068.G1.sam
##               <factor>         <factor>         <factor>         <factor>
##   [1]              CN2              CN2              CN2              CN2
##   [2]              CN2              CN3              CN3              CN2
##       Case_L069.G1.sam Case_L070.G1.sam Case_L071.G1.sam Case_L072.G1.sam
```

```
##              <factor>        <factor>        <factor>        <factor>
##   [1]             CN2             CN2             CN2             CN2
##   [2]             CN2             CN3             CN2             CN2
##       Case_L073.G1.sam Case_L074.G1.sam Case_L075.G1.sam Case_L076.G1.sam
##              <factor>        <factor>        <factor>        <factor>
##   [1]             CN2             CN2             CN2             CN2
##   [2]             CN3             CN2             CN2             CN2
##       Case_L077.G1.sam Case_L079.G1.sam Case_L080.G1.sam Case_L081.G1.sam
##              <factor>        <factor>        <factor>        <factor>
##   [1]             CN2             CN2             CN2             CN2
##   [2]             CN2             CN3             CN2             CN2
##       Case_L082.G1.sam Case_L083.G1.sam Case_L084.G1.sam Case_L085.G1.sam
##              <factor>        <factor>        <factor>        <factor>
##   [1]             CN2             CN2             CN2             CN2
##   [2]             CN2             CN2             CN2             CN2
##       Case_L086.G1.sam Case_L087.G1.sam Case_L088.G1.sam Case_L089.G1.sam
##              <factor>        <factor>        <factor>        <factor>
##   [1]             CN2             CN2             CN2             CN2
##   [2]             CN2             CN2             CN2             CN2
##       Case_L090.G1.sam Case_L091.G1.sam Case_L092.G1.sam Case_L093.G1.sam
##              <factor>        <factor>        <factor>        <factor>
##   [1]             CN2             CN2             CN2             CN2
##   [2]             CN2             CN2             CN2             CN2
##       Case_L094.G1.sam
##              <factor>
##   [1]             CN2
##   [2]             CN2
##   -------
##   seqinfo: 1 sequence from an unspecified genome; no seqlengths
##
## Individual CNVs:
## GRanges object with 8 ranges and 4 metadata columns:
##       seqnames    ranges strand |       sampleName     median       mean
##          <Rle> <IRanges>  <Rle> |         <factor>  <numeric>  <numeric>
##   [1]    undef  [ 6,  9]      * | Case_L048.G1.sam -0.9996685 -0.9995093
##   [2]    undef  [ 6,  9]      * | Case_L051.G1.sam -0.9990795 -0.9988412
##   [3]    undef  [30, 32]      * | Case_L064.G1.sam  0.7494421  0.6861013
##   [4]    undef  [30, 32]      * | Case_L066.G1.sam  0.4906219  0.6871242
##   [5]    undef  [30, 32]      * | Case_L067.G1.sam  0.4961909  0.6959176
##   [6]    undef  [30, 32]      * | Case_L070.G1.sam  0.9885304  0.7695707
##   [7]    undef  [30, 32]      * | Case_L073.G1.sam  0.9499858  0.7309261
##   [8]    undef  [30, 32]      * | Case_L079.G1.sam  0.5295616  0.5096933
##                 CN
##       <character>
##   [1]         CN1
##   [2]         CN1
##   [3]         CN3
##   [4]         CN3
##   [5]         CN3
##   [6]         CN3
##   [7]         CN3
##   [8]         CN3
##   -------
##   seqinfo: 1 sequence from an unspecified genome; no seqlengths
```

```
## [1] "/Users/gdemidov/Downloads/doc/Run_SN1_43_CRG_fin_05_qc.xls"

## Normalizing...

## Starting local modeling, please be patient...

## Reference sequence:  undef

## Starting segmentation algorithm...

## Using "fastseg" for segmentation.

## [1] ""
## [1] "/Users/gdemidov/Downloads/doc/Run_SN1_43_CRG_fin_05_qc.xls"
## [1] ""

## Segplot might not work because of special characters in the sample names. Use only A-Z,a-z and 0-9!
##  There is a hidden function cn.mops:::.replaceNames that replaces the names in the "CNVDetectionResul
```

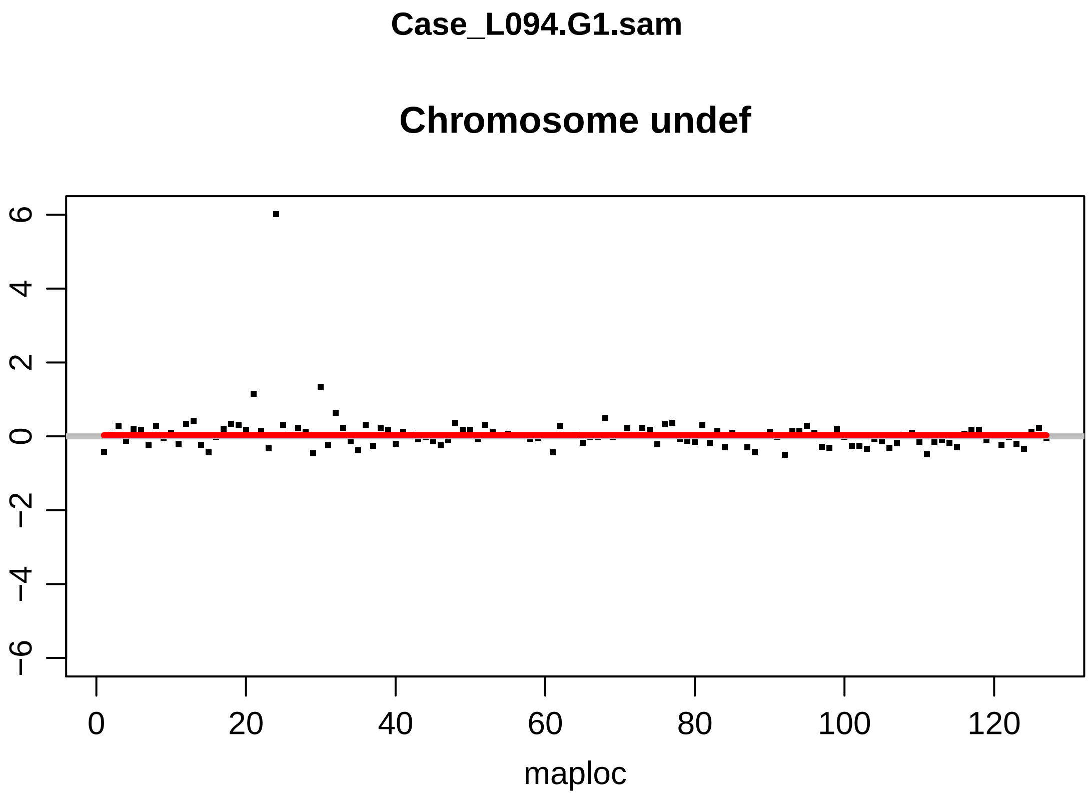


```
## Segplot might not work because of special characters in the sample names. Use only A-Z,a-z and 0-9!
##  There is a hidden function cn.mops:::.replaceNames that replaces the names in the "CNVDetectionResul
```

Case_CONTROL.L1.sam

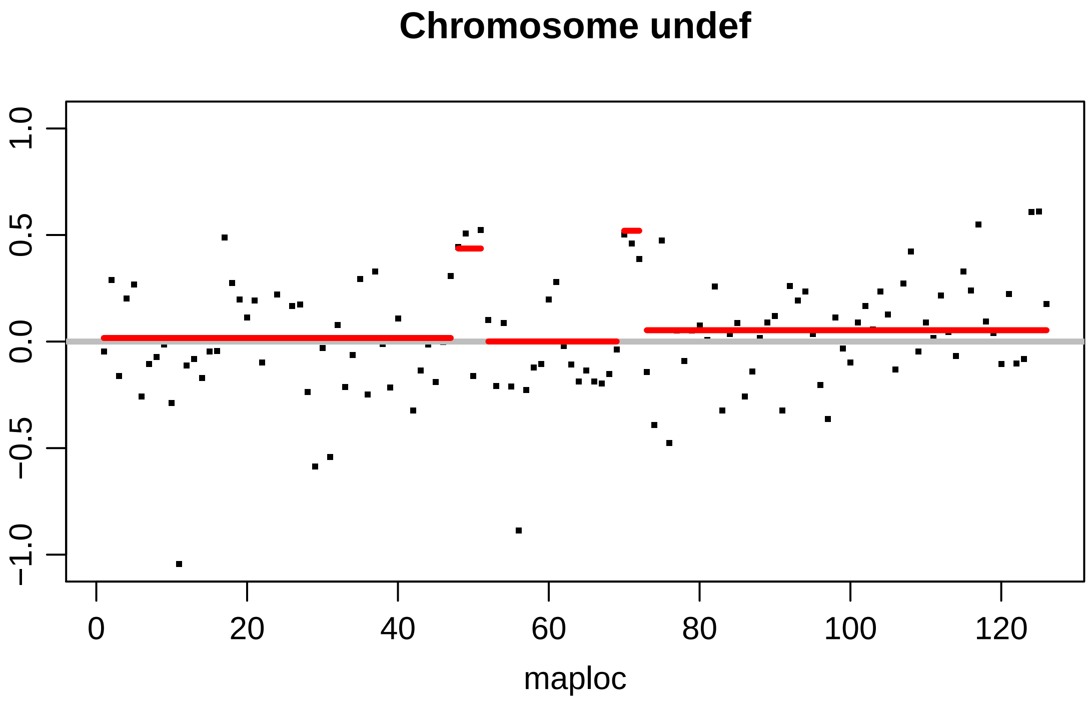


```
## Segplot might not work because of special characters in the sample names. Use only A-Z,a-z and 0-9!
##  There is a hidden function cn.mops:::.replaceNames that replaces the names in the "CNVDetectionResul
```

Case_L096.G1.sam

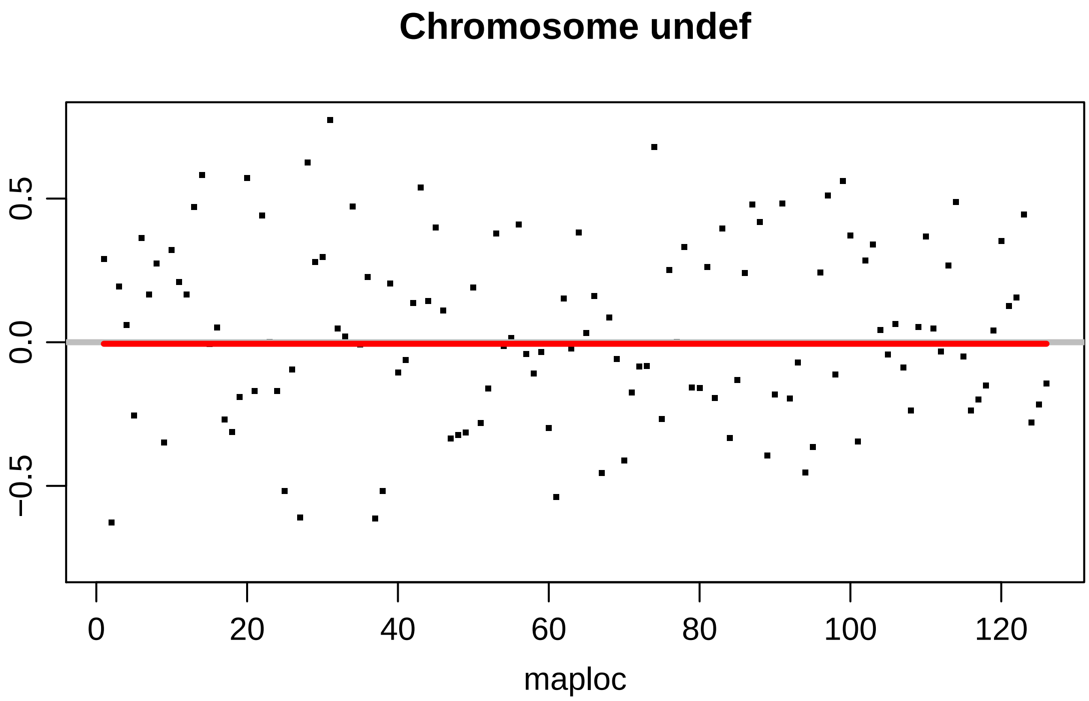

```
## Segplot might not work because of special characters in the sample names. Use only A-Z,a-z and 0-9!
##  There is a hidden function cn.mops:::.replaceNames that replaces the names in the "CNVDetectionResul
```

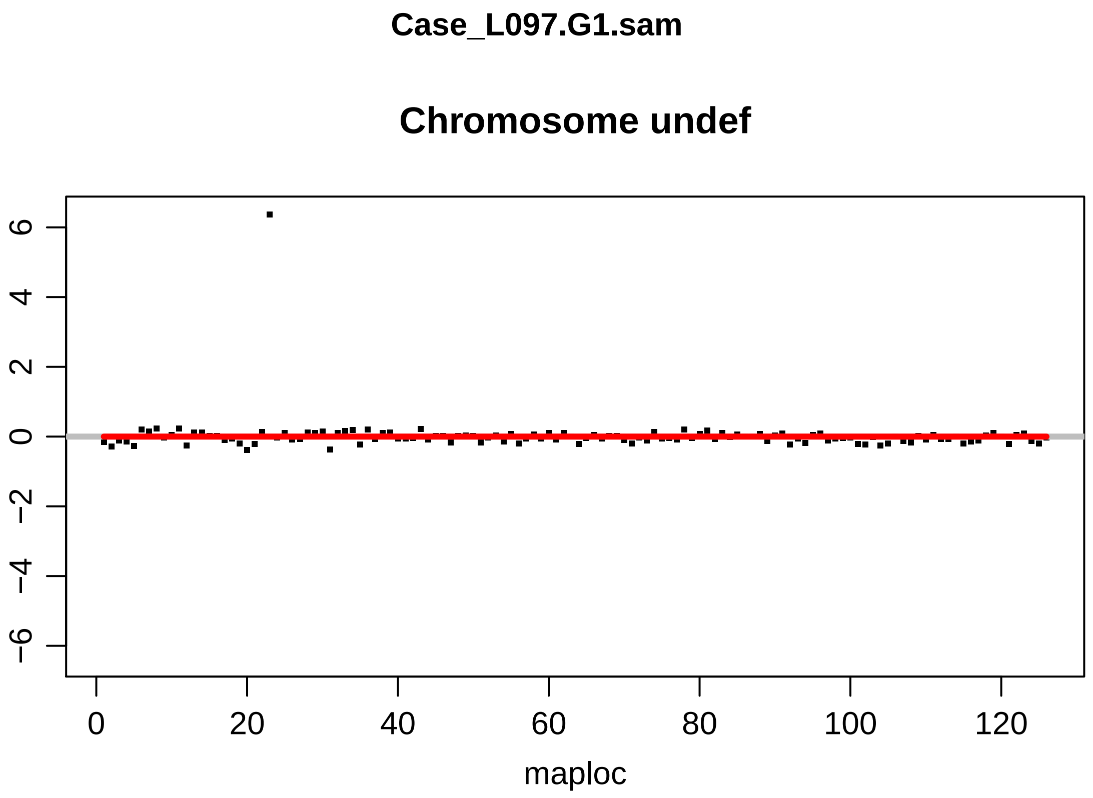


```
## Segplot might not work because of special characters in the sample names. Use only A-Z,a-z and 0-9!
##  There is a hidden function cn.mops:::.replaceNames that replaces the names in the "CNVDetectionResul
```

Case_L098.G1.sam

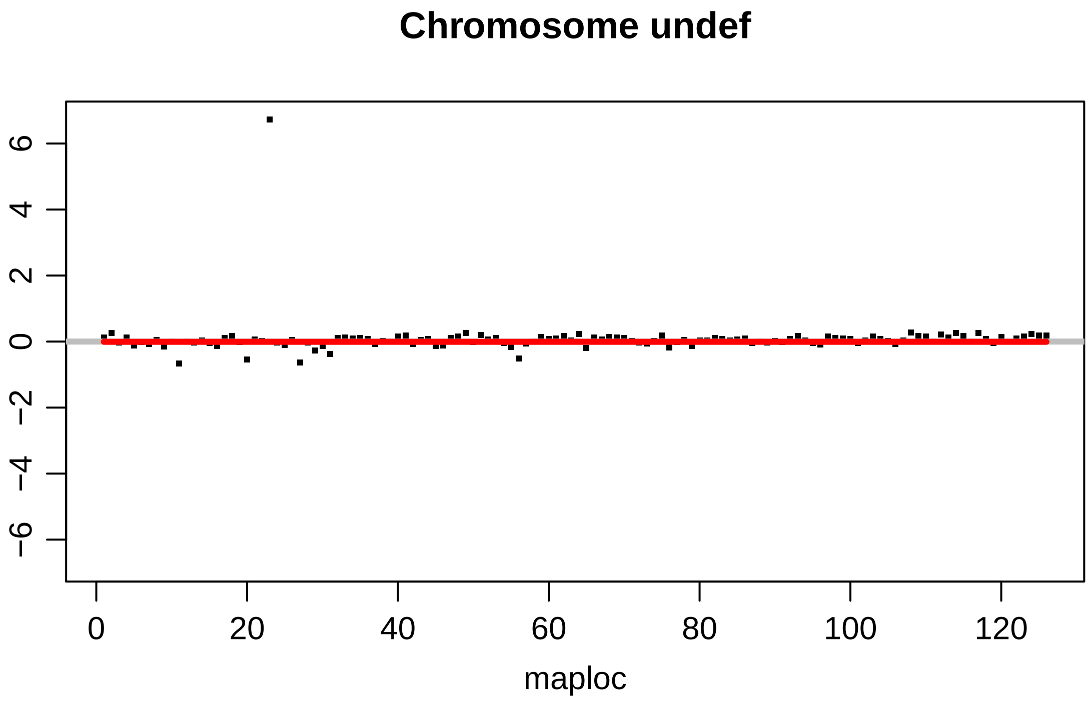


```
## Segplot might not work because of special characters in the sample names. Use only A-Z,a-z and 0-9!
##  There is a hidden function cn.mops:::.replaceNames that replaces the names in the "CNVDetectionResul
```

Case_L099.G1.sam

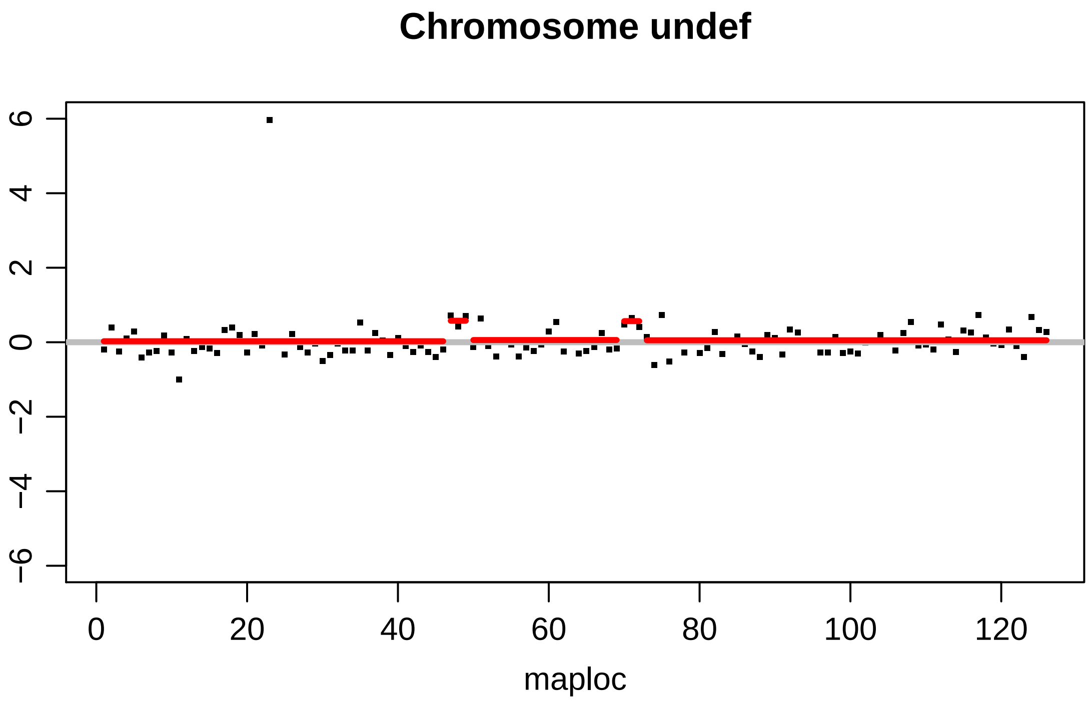

```
## Segplot might not work because of special characters in the sample names. Use only A-Z,a-z and 0-9!
##  There is a hidden function cn.mops:::.replaceNames that replaces the names in the "CNVDetectionResul
```

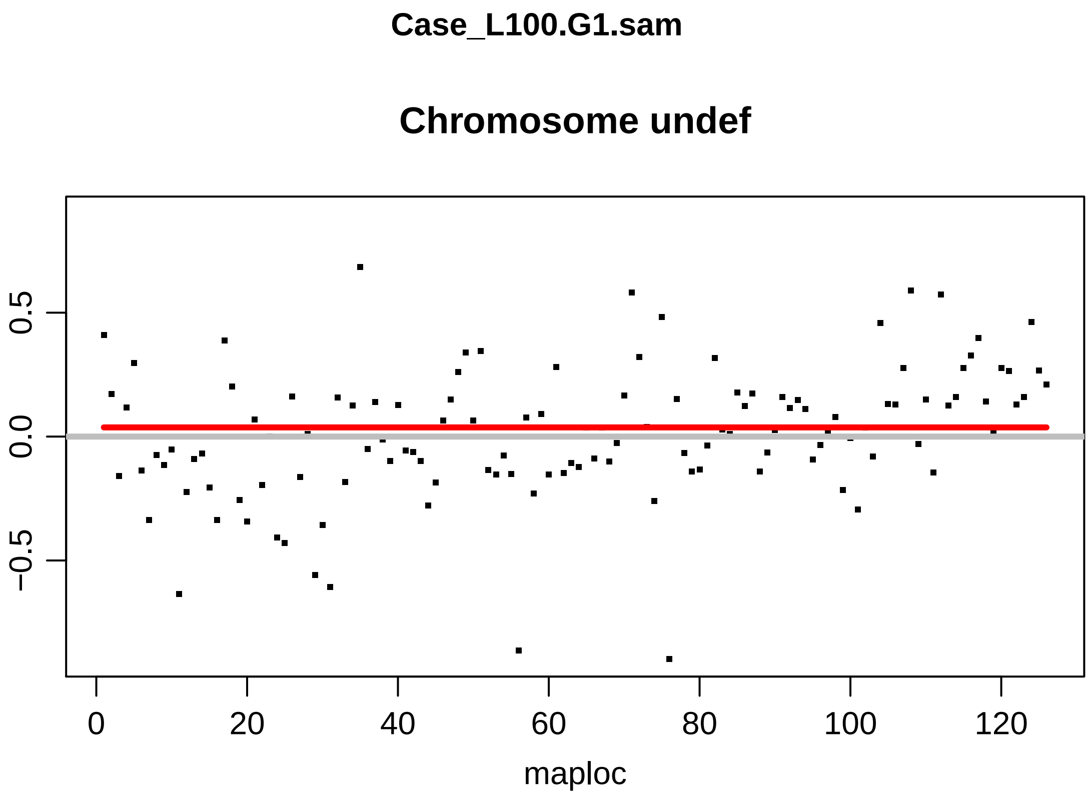


```
## Segplot might not work because of special characters in the sample names. Use only A-Z,a-z and 0-9!
##  There is a hidden function cn.mops:::.replaceNames that replaces the names in the "CNVDetectionResul
```

Case_L101.G1.sam

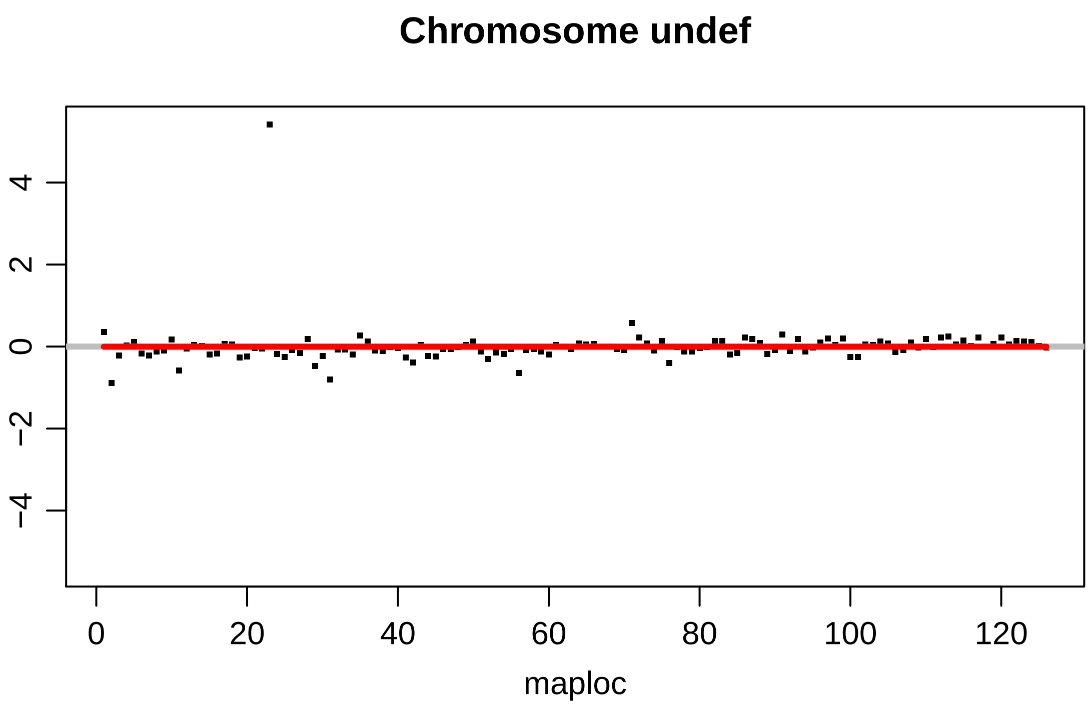


```
## Segplot might not work because of special characters in the sample names. Use only A-Z,a-z and 0-9!
##  There is a hidden function cn.mops:::.replaceNames that replaces the names in the "CNVDetectionResul
```

Case_L102.G1.sam

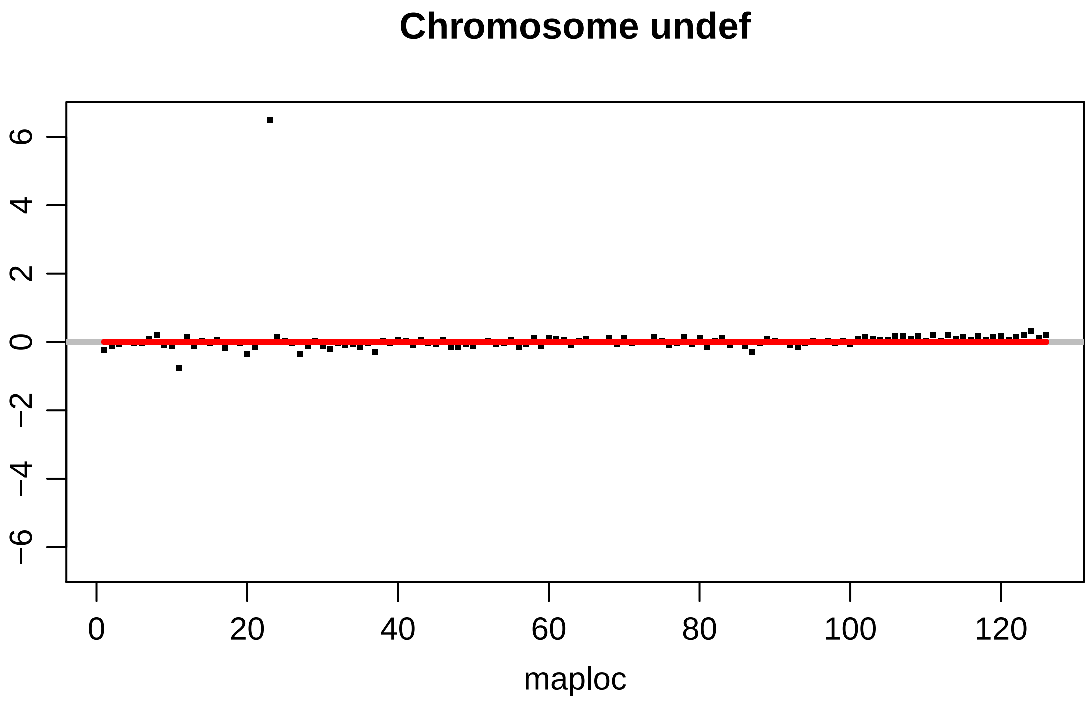

```
## Segplot might not work because of special characters in the sample names. Use only A-Z,a-z and 0-9!
##  There is a hidden function cn.mops:::.replaceNames that replaces the names in the "CNVDetectionResul
```

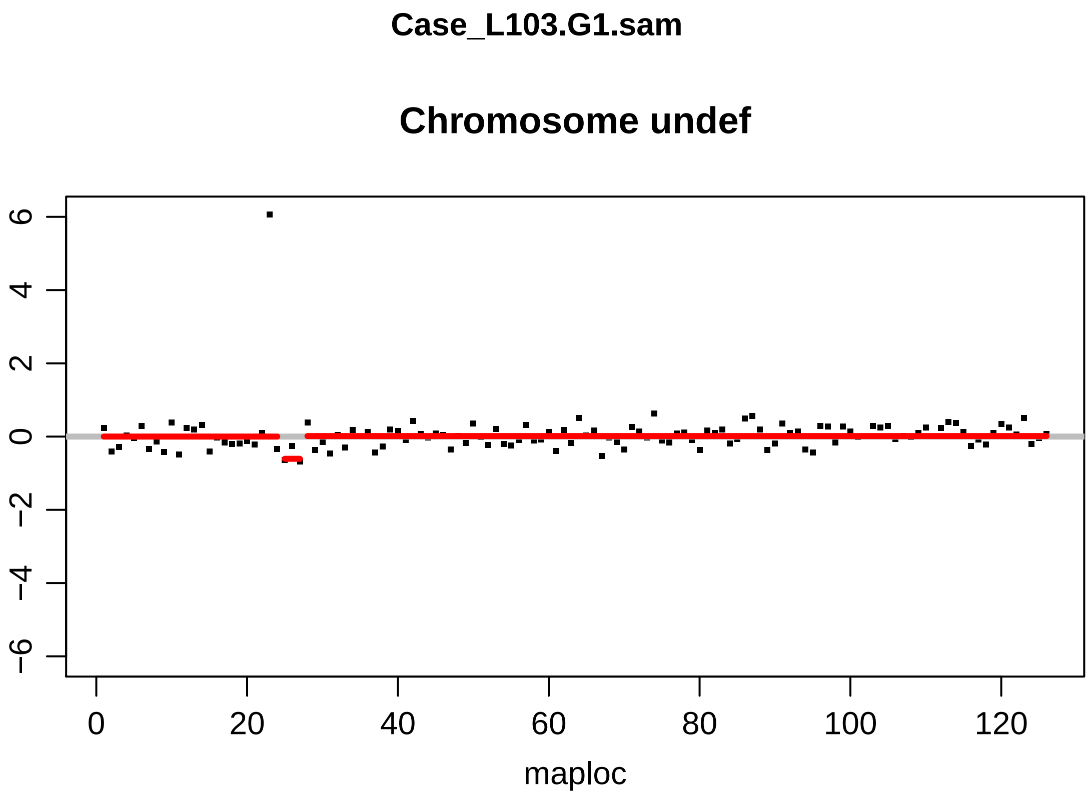


```
## Segplot might not work because of special characters in the sample names. Use only A-Z,a-z and 0-9!
##  There is a hidden function cn.mops:::.replaceNames that replaces the names in the "CNVDetectionResul
```

Case_L104.G1.sam

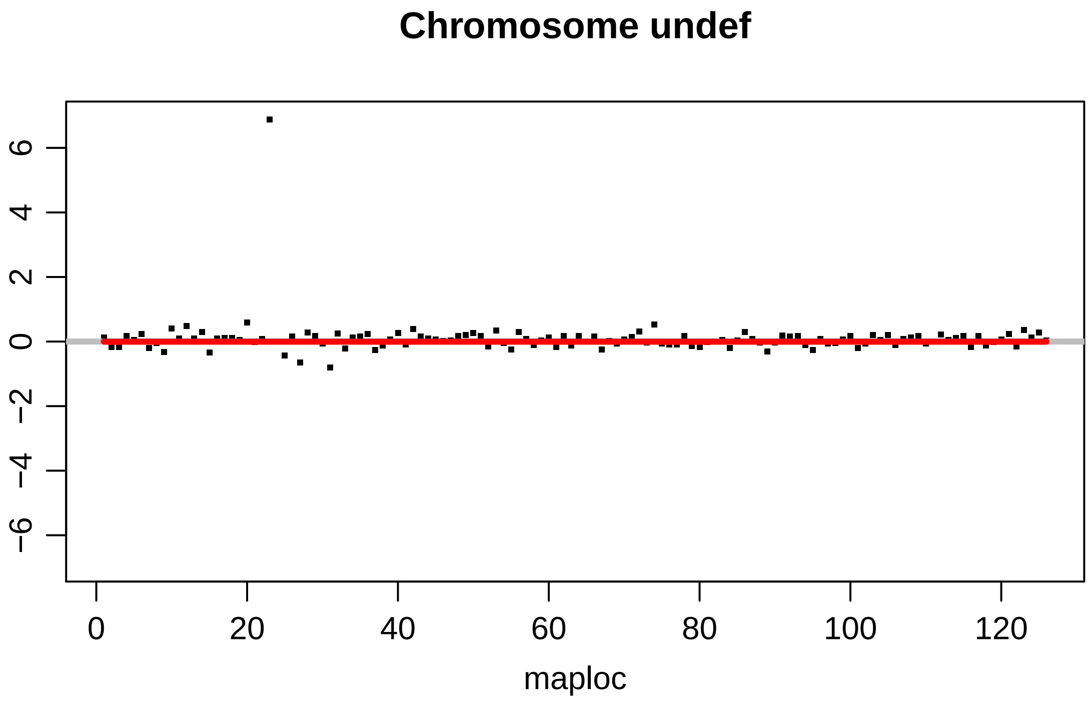


```
## Segplot might not work because of special characters in the sample names. Use only A-Z,a-z and 0-9!
##  There is a hidden function cn.mops:::.replaceNames that replaces the names in the "CNVDetectionResul
```

Case_L105.G1.sam

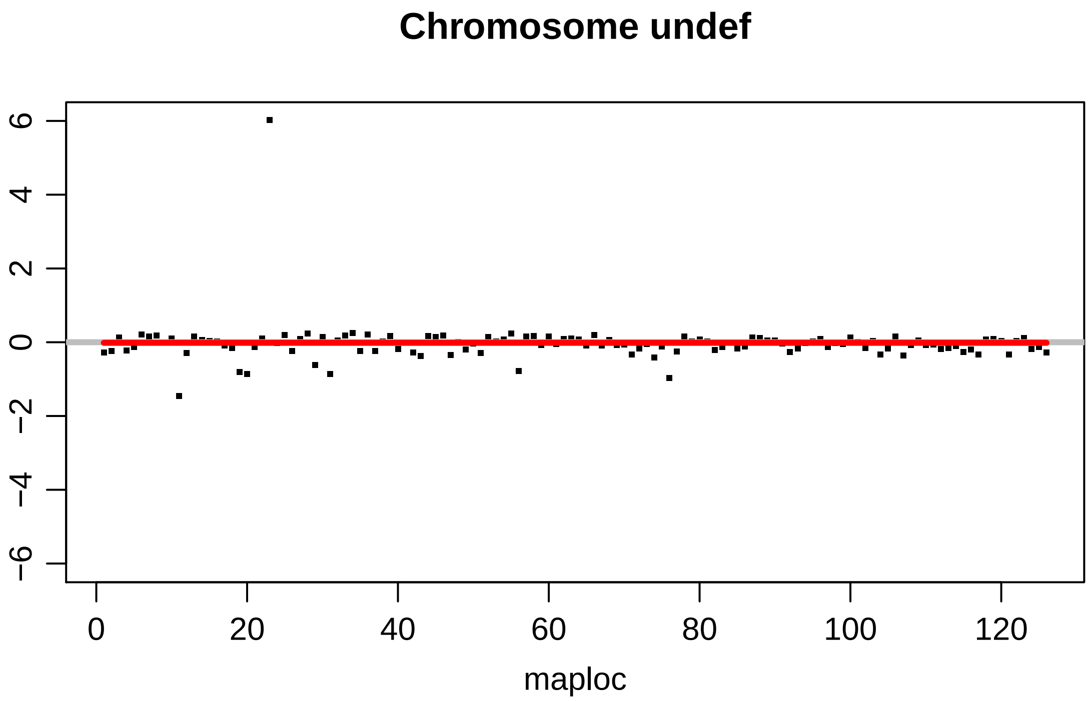

```
## Segplot might not work because of special characters in the sample names. Use only A-Z,a-z and 0-9!
##  There is a hidden function cn.mops:::.replaceNames that replaces the names in the "CNVDetectionResul
```

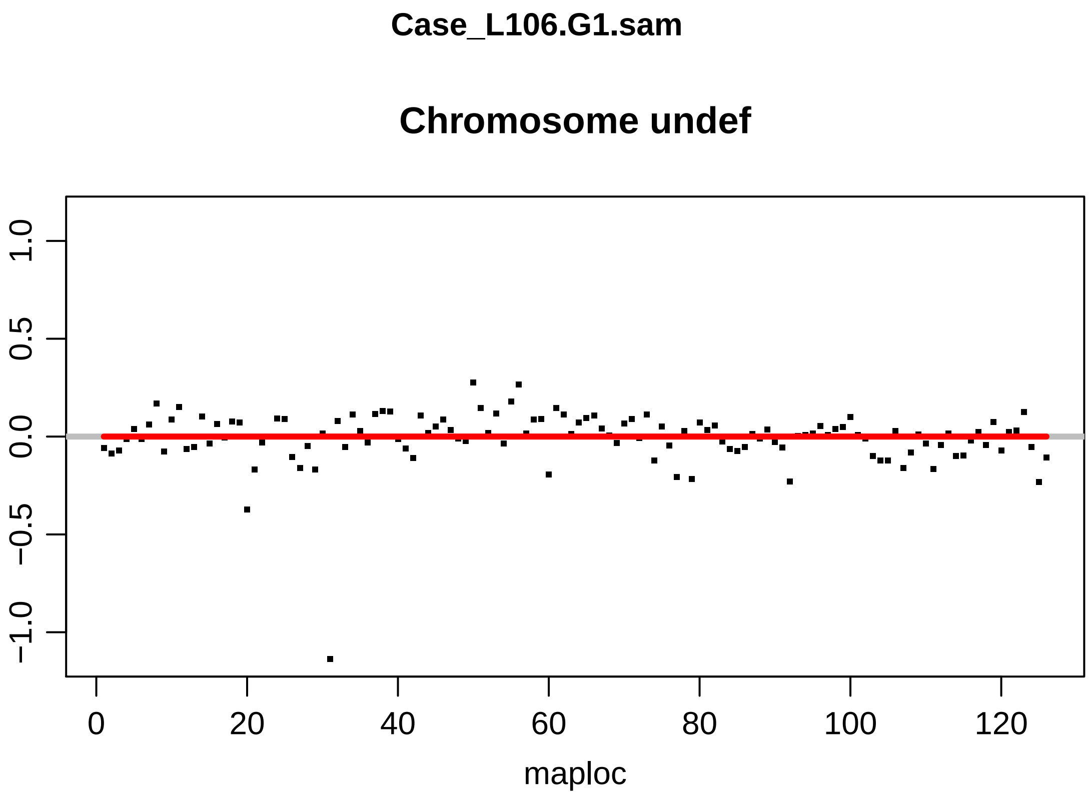


```
## Segplot might not work because of special characters in the sample names. Use only A-Z,a-z and 0-9!
##  There is a hidden function cn.mops:::.replaceNames that replaces the names in the "CNVDetectionResul
```

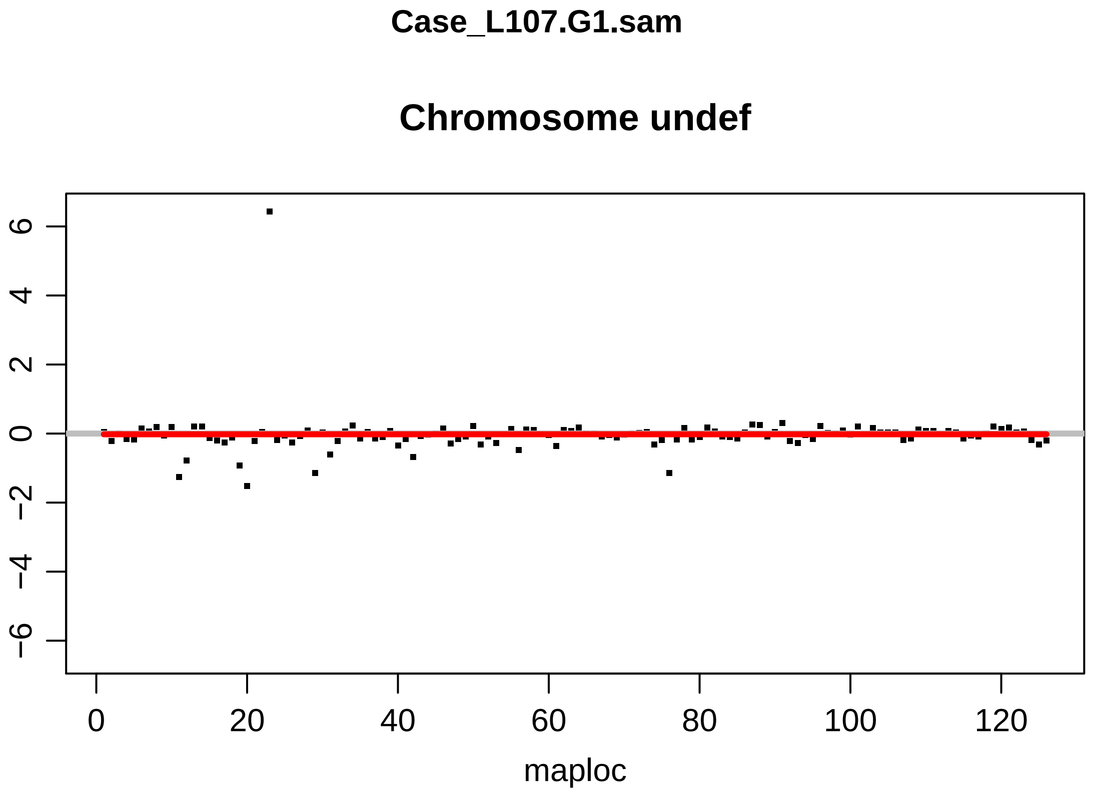


```
## Segplot might not work because of special characters in the sample names. Use only A-Z,a-z and 0-9!
##  There is a hidden function cn.mops:::.replaceNames that replaces the names in the "CNVDetectionResul
```

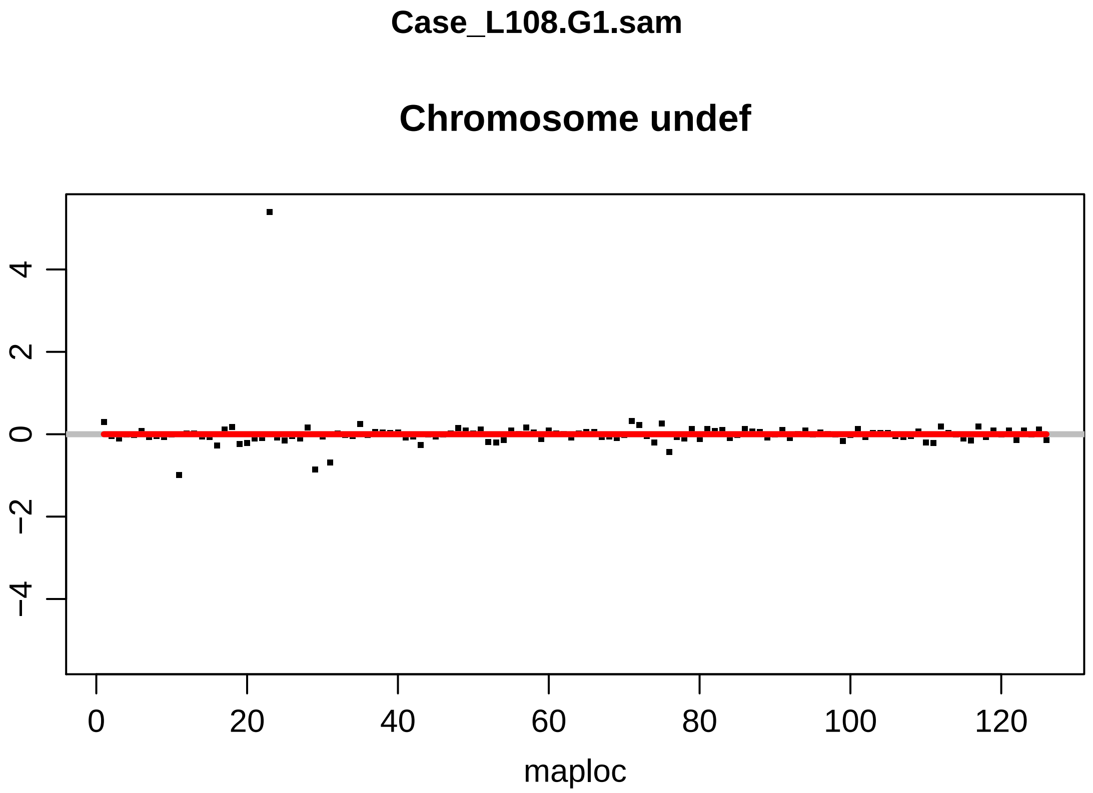

```
## Segplot might not work because of special characters in the sample names. Use only A-Z,a-z and 0-9!
##  There is a hidden function cn.mops:::.replaceNames that replaces the names in the "CNVDetectionResul
```

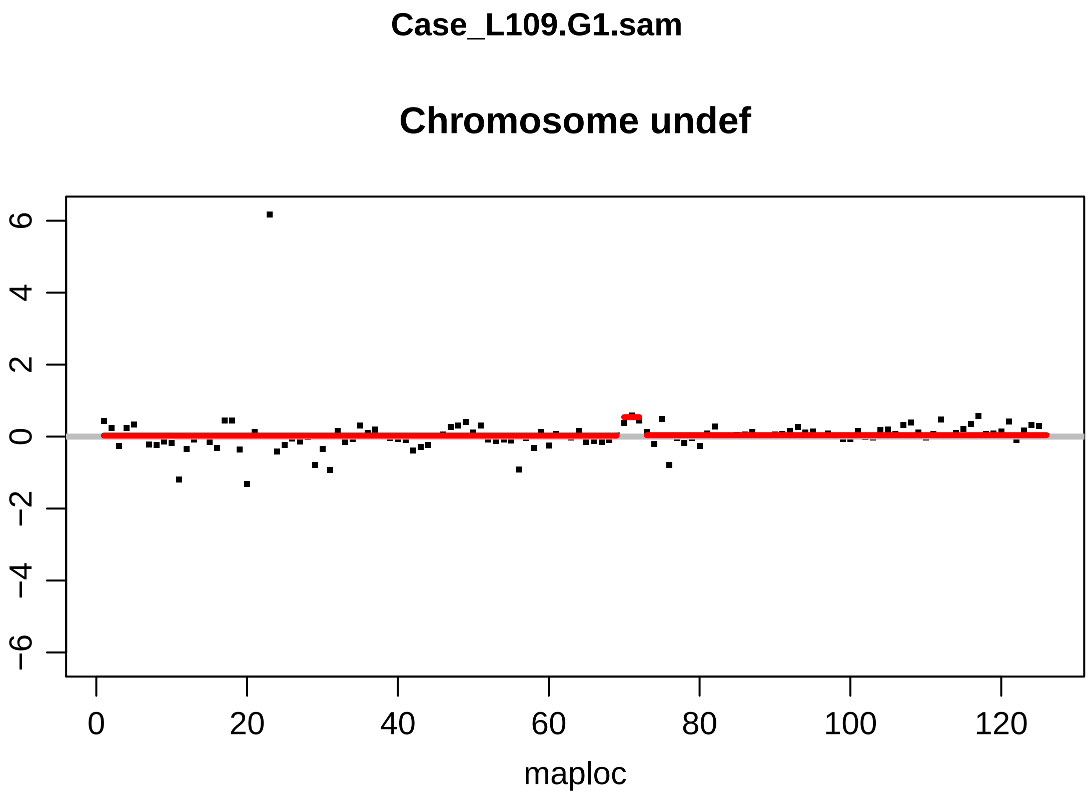


```
## Segplot might not work because of special characters in the sample names. Use only A-Z,a-z and 0-9!
##  There is a hidden function cn.mops:::.replaceNames that replaces the names in the "CNVDetectionResul
```

Case_L110.G1.sam

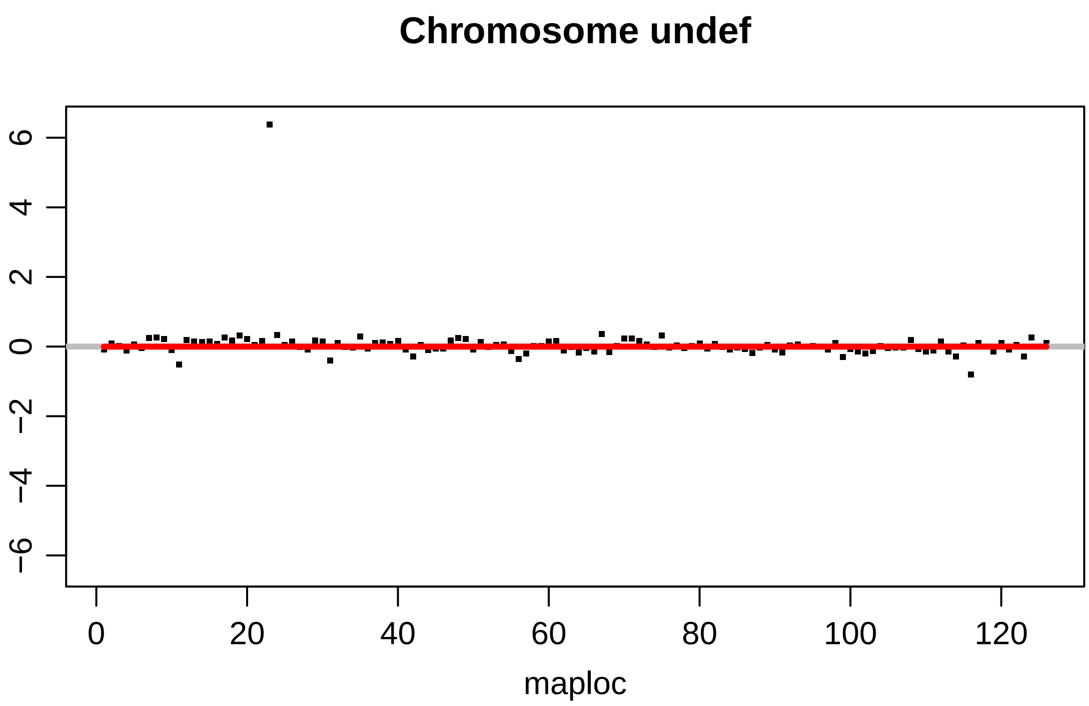


```
## Segplot might not work because of special characters in the sample names. Use only A-Z,a-z and 0-9!
##  There is a hidden function cn.mops:::.replaceNames that replaces the names in the "CNVDetectionResul
```

Case_L111.G1.sam

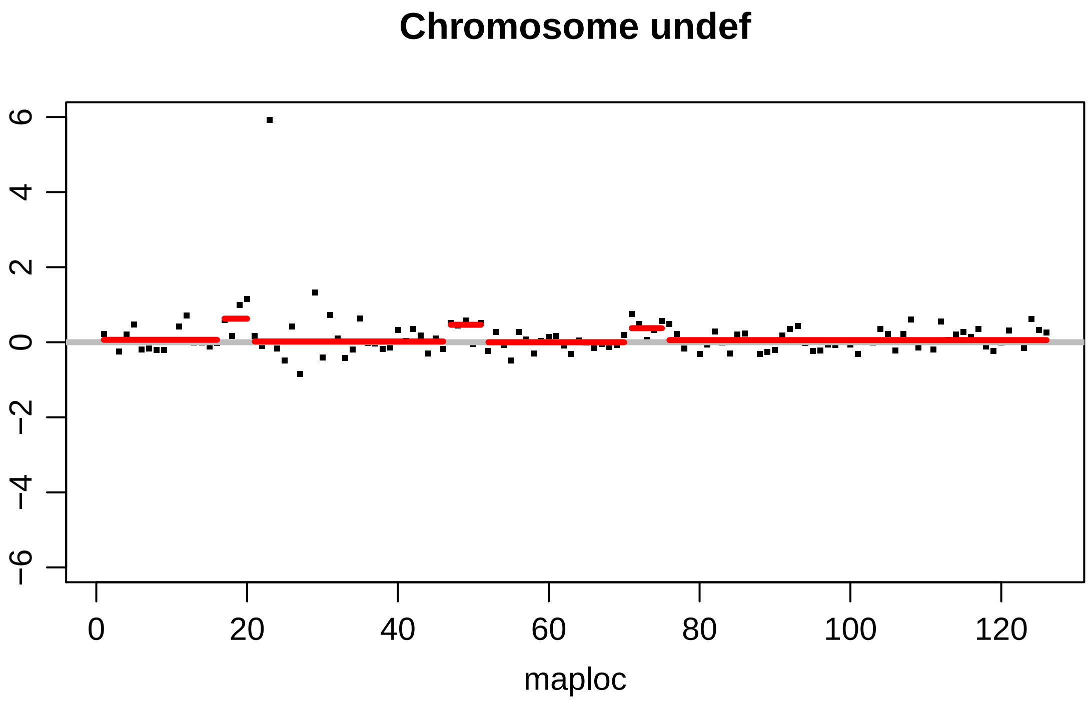

```
## Segplot might not work because of special characters in the sample names. Use only A-Z,a-z and 0-9!
##  There is a hidden function cn.mops:::.replaceNames that replaces the names in the "CNVDetectionResul
```

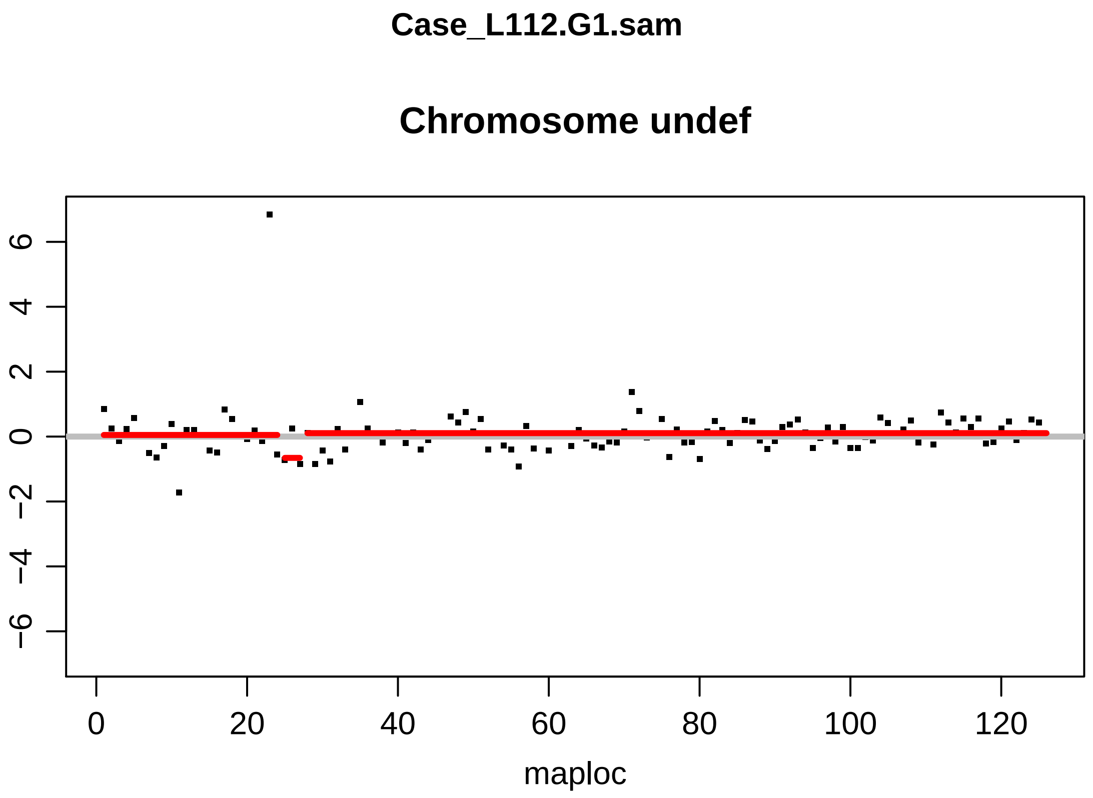


```
## Segplot might not work because of special characters in the sample names. Use only A-Z,a-z and 0-9!
##  There is a hidden function cn.mops:::.replaceNames that replaces the names in the "CNVDetectionResul
```

Case_L113.G1.sam

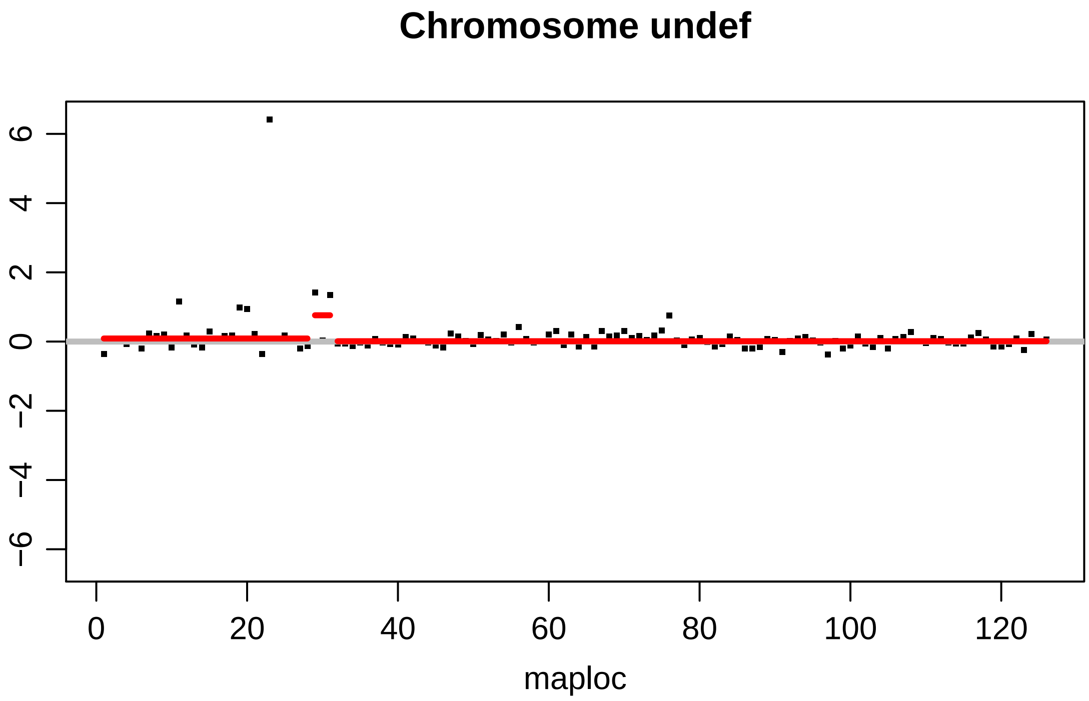


```
## Segplot might not work because of special characters in the sample names. Use only A-Z,a-z and 0-9!
##  There is a hidden function cn.mops:::.replaceNames that replaces the names in the "CNVDetectionResul
```

Case_L114.G1.sam

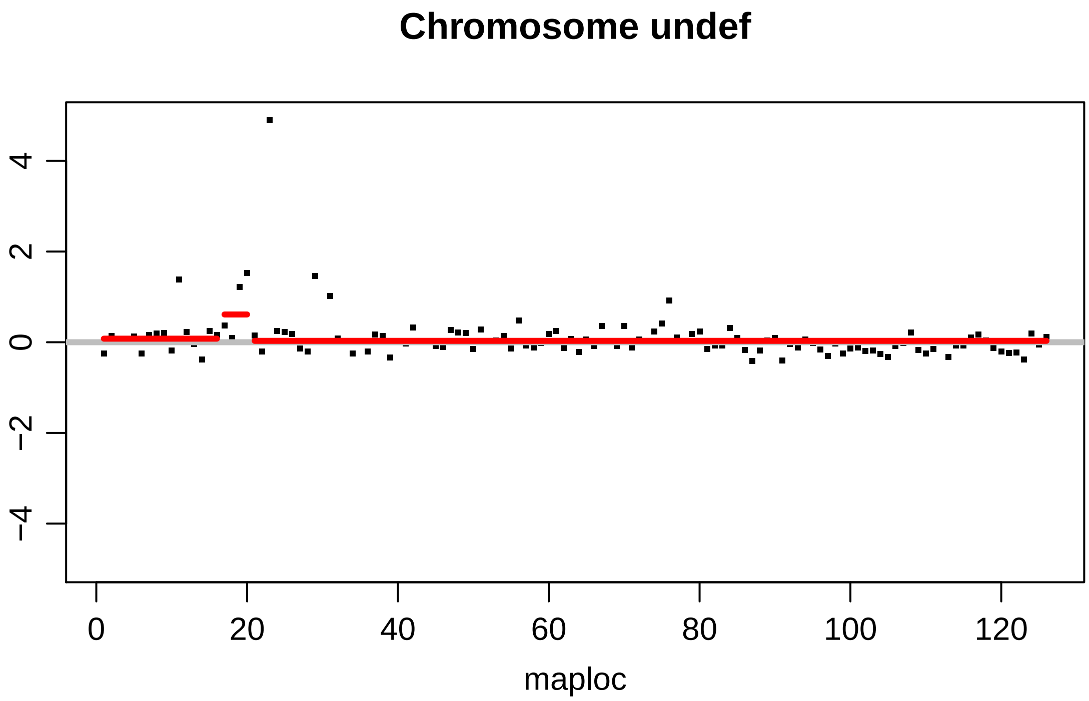

```
## Segplot might not work because of special characters in the sample names. Use only A-Z,a-z and 0-9!
##  There is a hidden function cn.mops:::.replaceNames that replaces the names in the "CNVDetectionResul
```

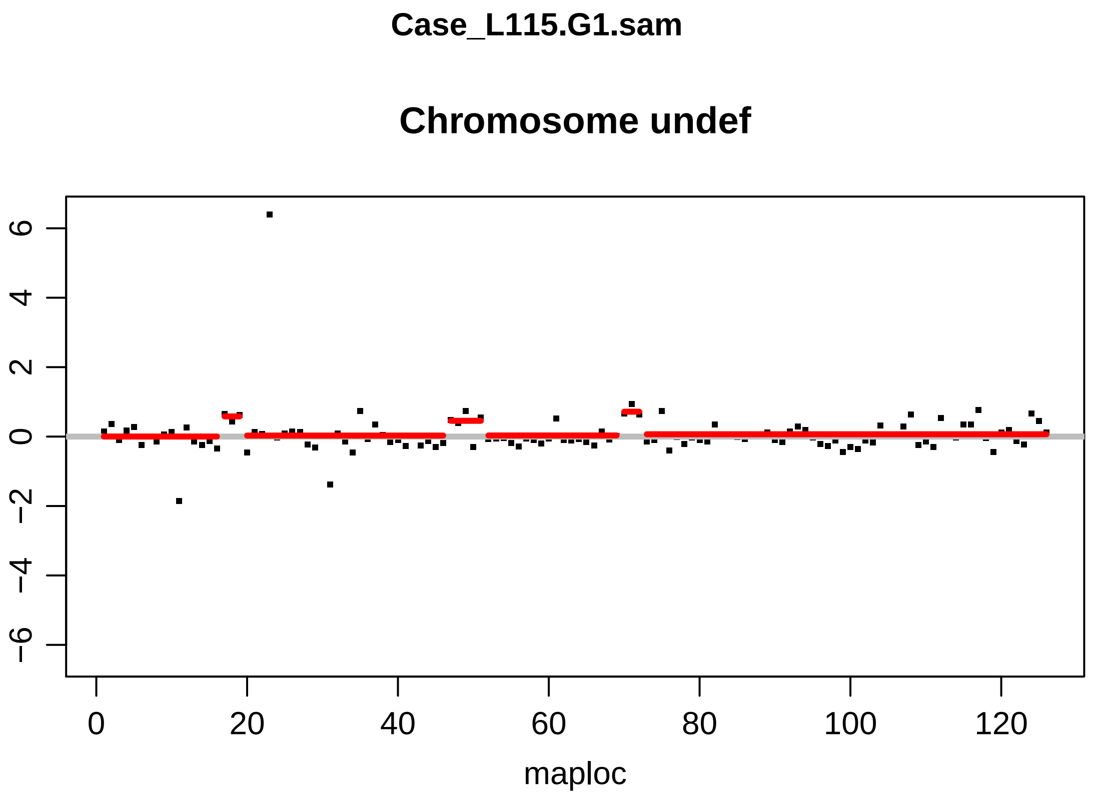


```
## Segplot might not work because of special characters in the sample names. Use only A-Z,a-z and 0-9!
##  There is a hidden function cn.mops:::.replaceNames that replaces the names in the "CNVDetectionResul
```

Case_L116.G1.sam

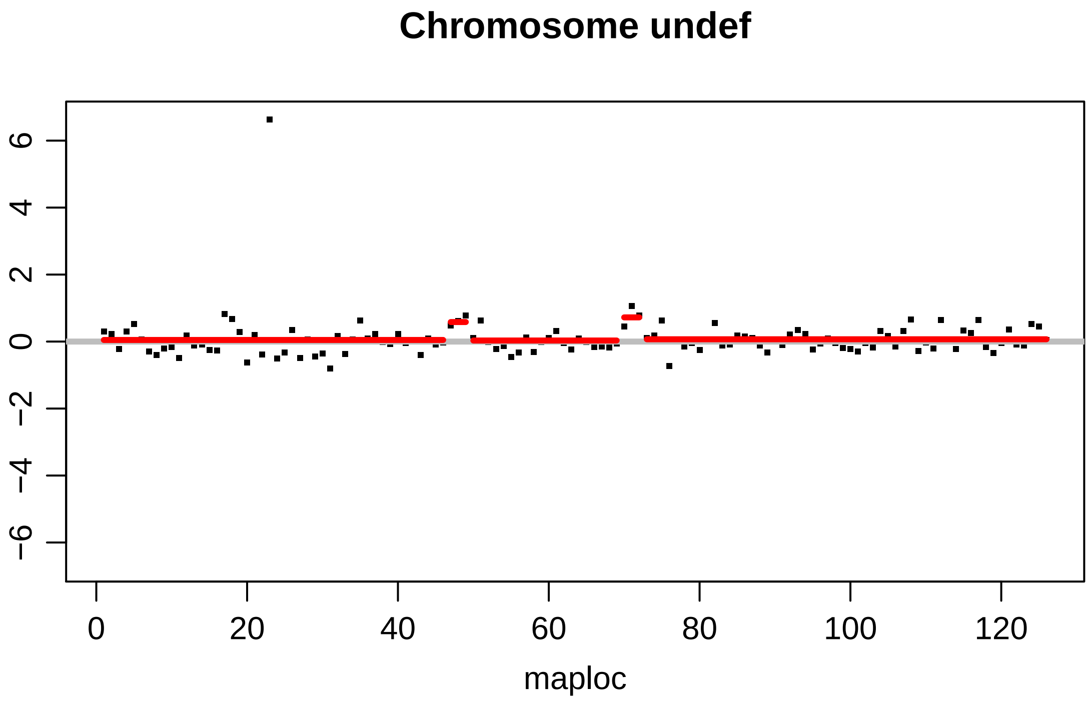


```
## Segplot might not work because of special characters in the sample names. Use only A-Z,a-z and 0-9!
##  There is a hidden function cn.mops:::.replaceNames that replaces the names in the "CNVDetectionResul
```

Case_L117.G1.sam

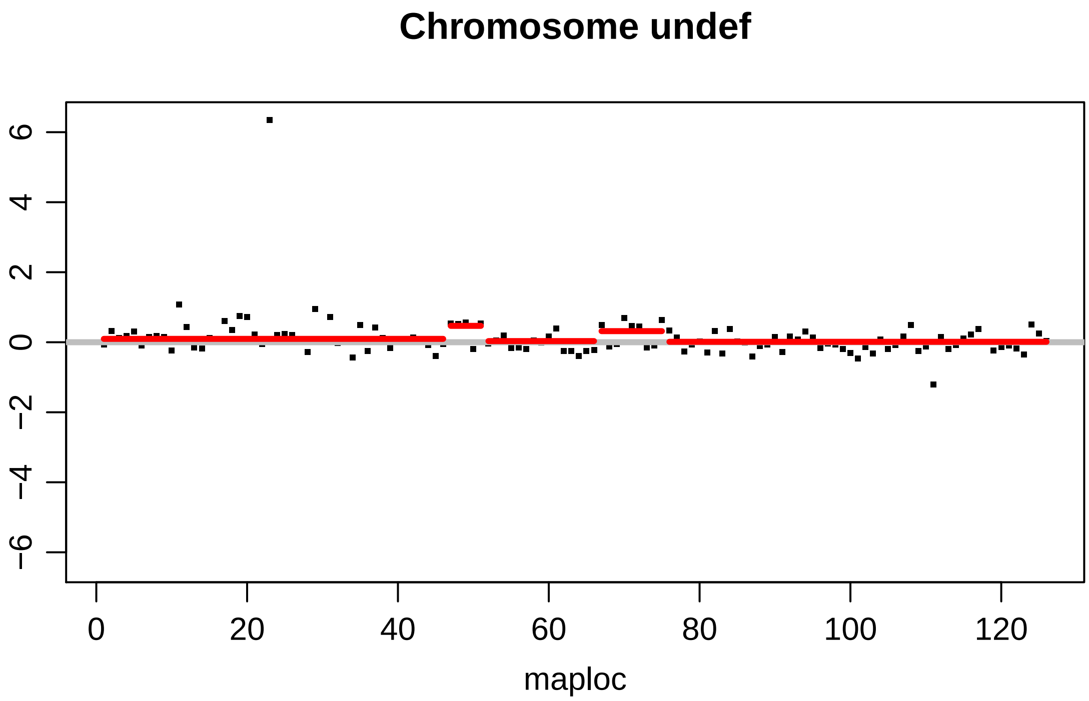

```
## Segplot might not work because of special characters in the sample names. Use only A-Z,a-z and 0-9!
##  There is a hidden function cn.mops:::.replaceNames that replaces the names in the "CNVDetectionResul
```

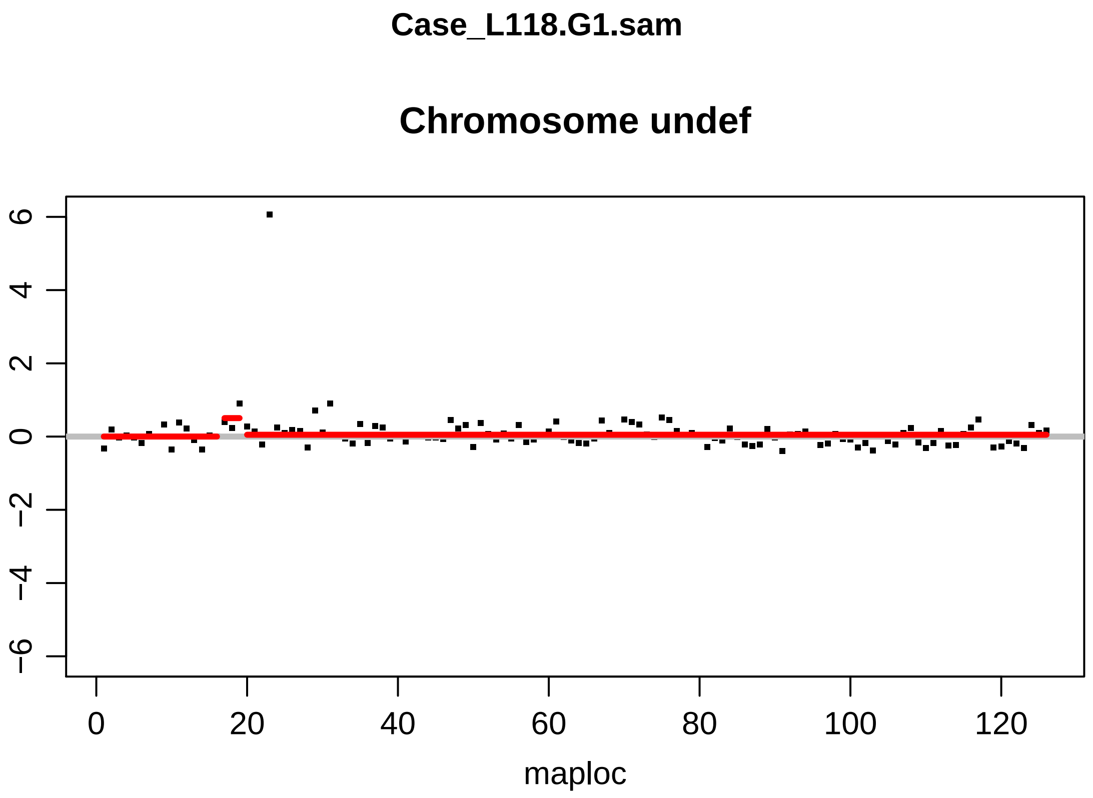


```
## Segplot might not work because of special characters in the sample names. Use only A-Z,a-z and 0-9!
##  There is a hidden function cn.mops:::.replaceNames that replaces the names in the "CNVDetectionResul
```

Case_L119.G1.sam

Chromosome undef

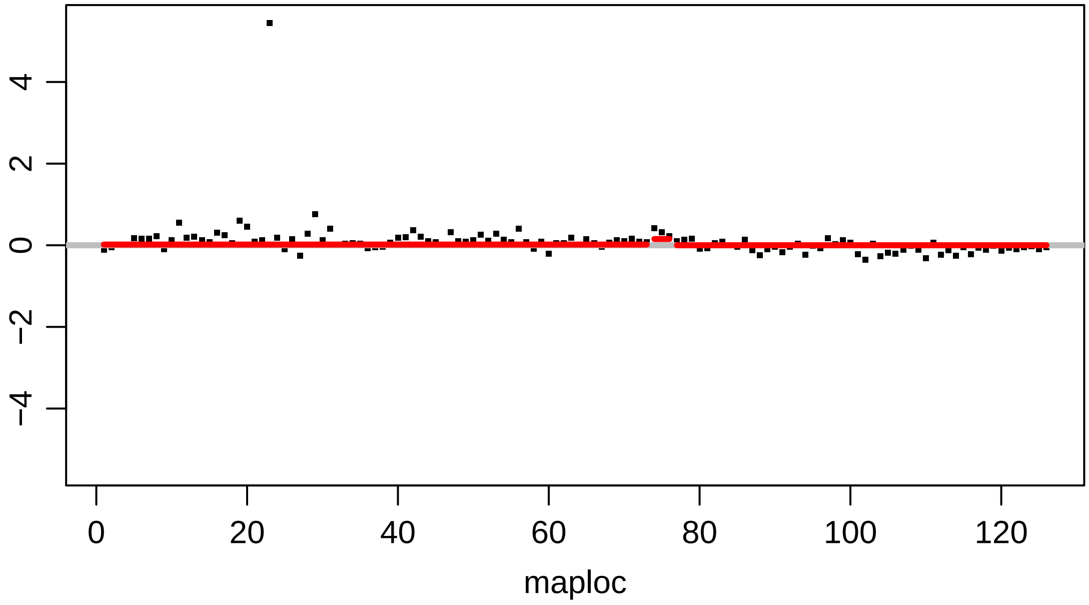


```
## Segplot might not work because of special characters in the sample names. Use only A-Z,a-z and 0-9!
##  There is a hidden function cn.mops:::.replaceNames that replaces the names in the "CNVDetectionResul
```

Case_L120.G1.sam

Chromosome undef

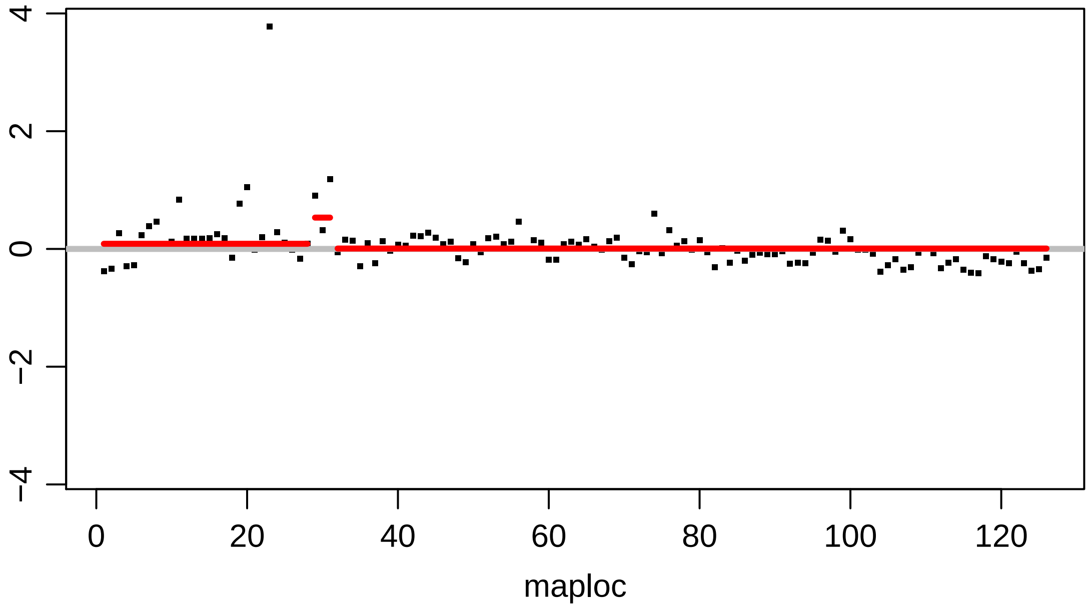

```
## Segplot might not work because of special characters in the sample names. Use only A-Z,a-z and 0-9!
##  There is a hidden function cn.mops:::.replaceNames that replaces the names in the "CNVDetectionResul
```

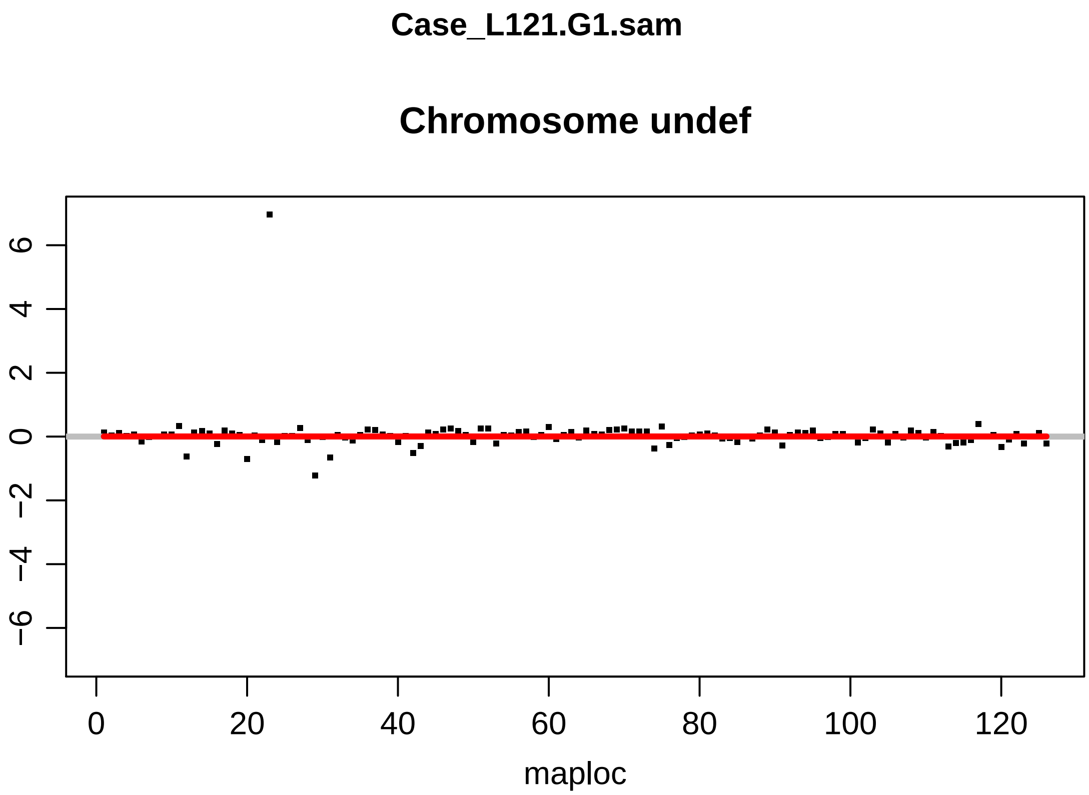


```
## Segplot might not work because of special characters in the sample names. Use only A-Z,a-z and 0-9!
##  There is a hidden function cn.mops:::.replaceNames that replaces the names in the "CNVDetectionResul
```

Case_L122.G1.sam

Chromosome undef

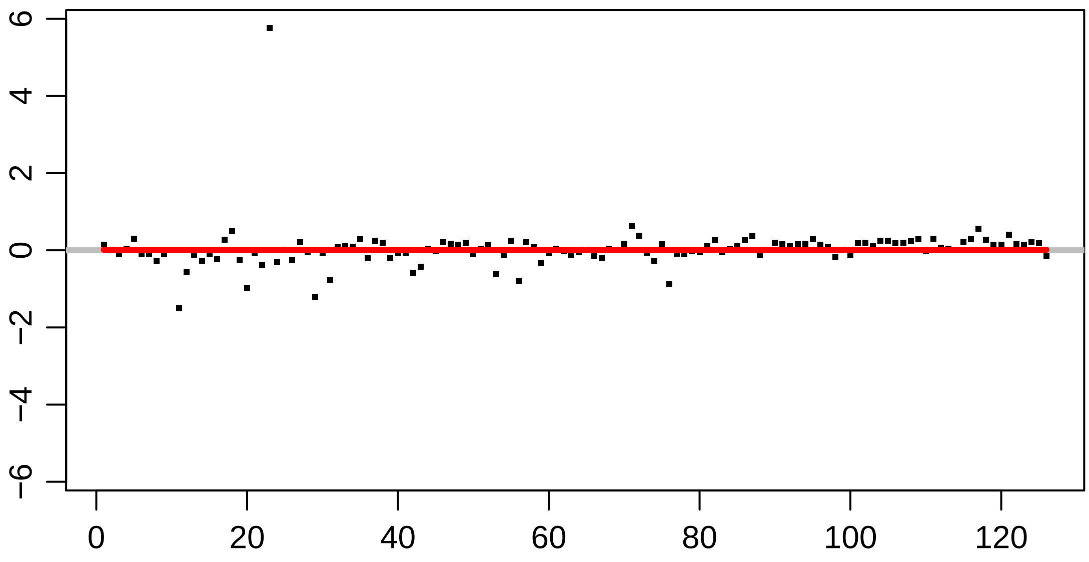


```
## Segplot might not work because of special characters in the sample names. Use only A-Z,a-z and 0-9!
##  There is a hidden function cn.mops:::.replaceNames that replaces the names in the "CNVDetectionResul
```

Case_L123.G1.sam

Chromosome undef

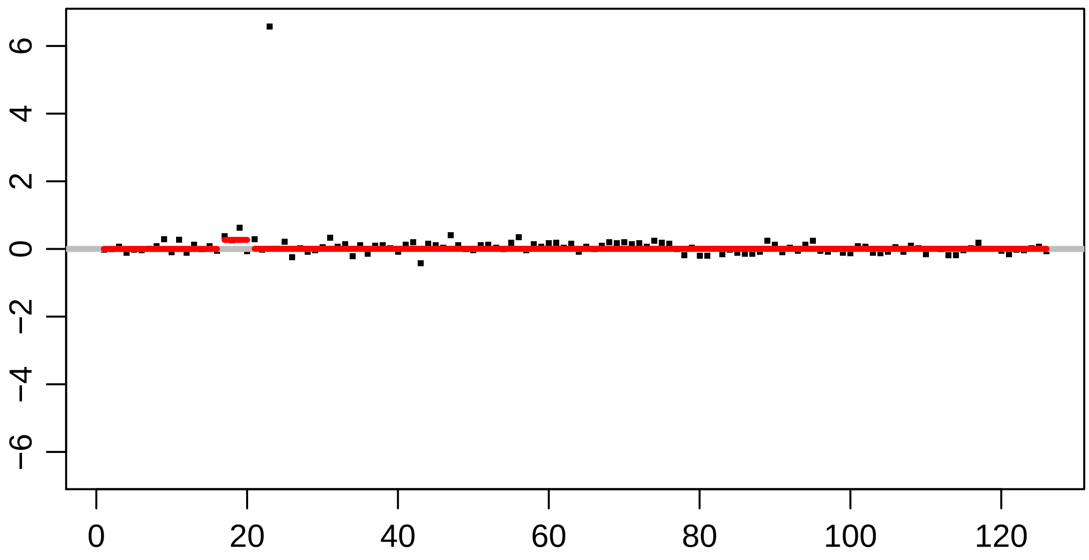

```
## Segplot might not work because of special characters in the sample names. Use only A-Z,a-z and 0-9!
##  There is a hidden function cn.mops:::.replaceNames that replaces the names in the "CNVDetectionResul
```

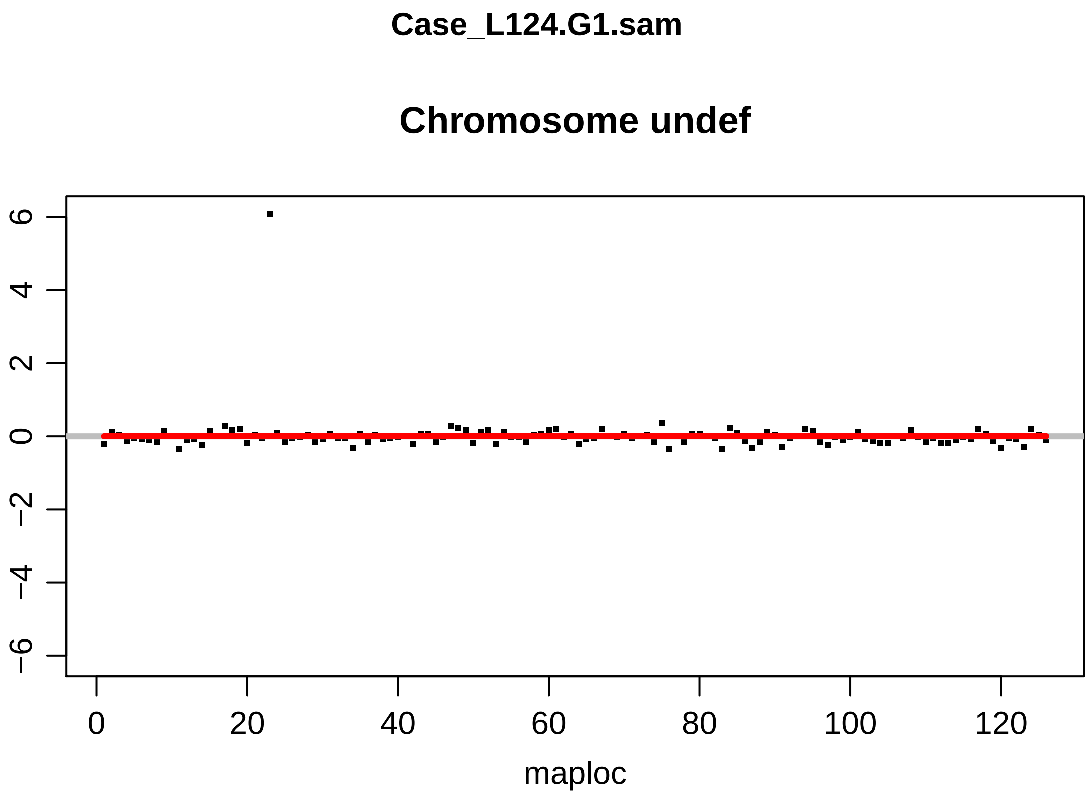


```
## Segplot might not work because of special characters in the sample names. Use only A-Z,a-z and 0-9!
##  There is a hidden function cn.mops:::.replaceNames that replaces the names in the "CNVDetectionResul
```

Case_L125.G1.sam

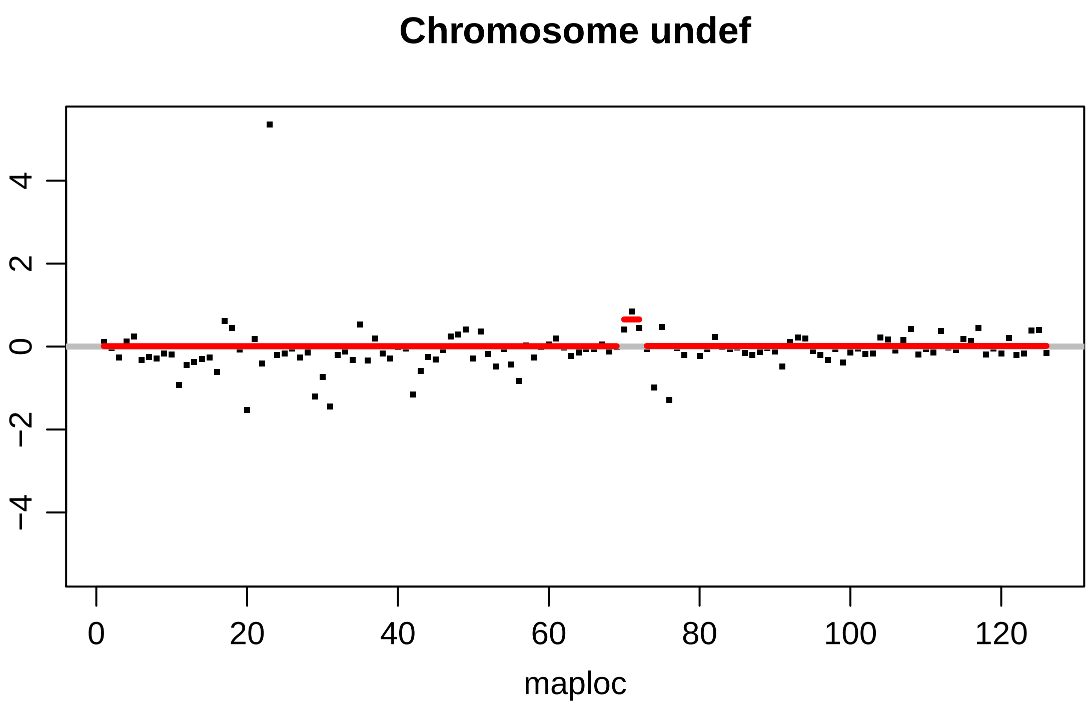


```
## Segplot might not work because of special characters in the sample names. Use only A-Z,a-z and 0-9!
##  There is a hidden function cn.mops:::.replaceNames that replaces the names in the "CNVDetectionResul
```

Case_L126.G1.sam

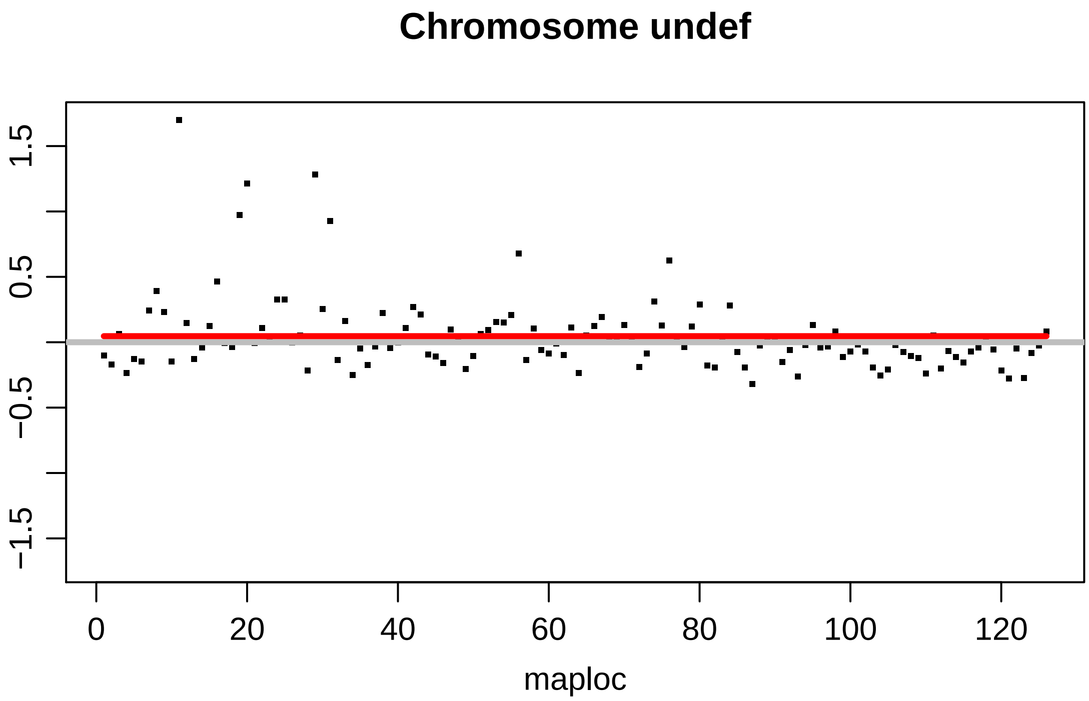

```
## Segplot might not work because of special characters in the sample names. Use only A-Z,a-z and 0-9!
##  There is a hidden function cn.mops:::.replaceNames that replaces the names in the "CNVDetectionResul
```

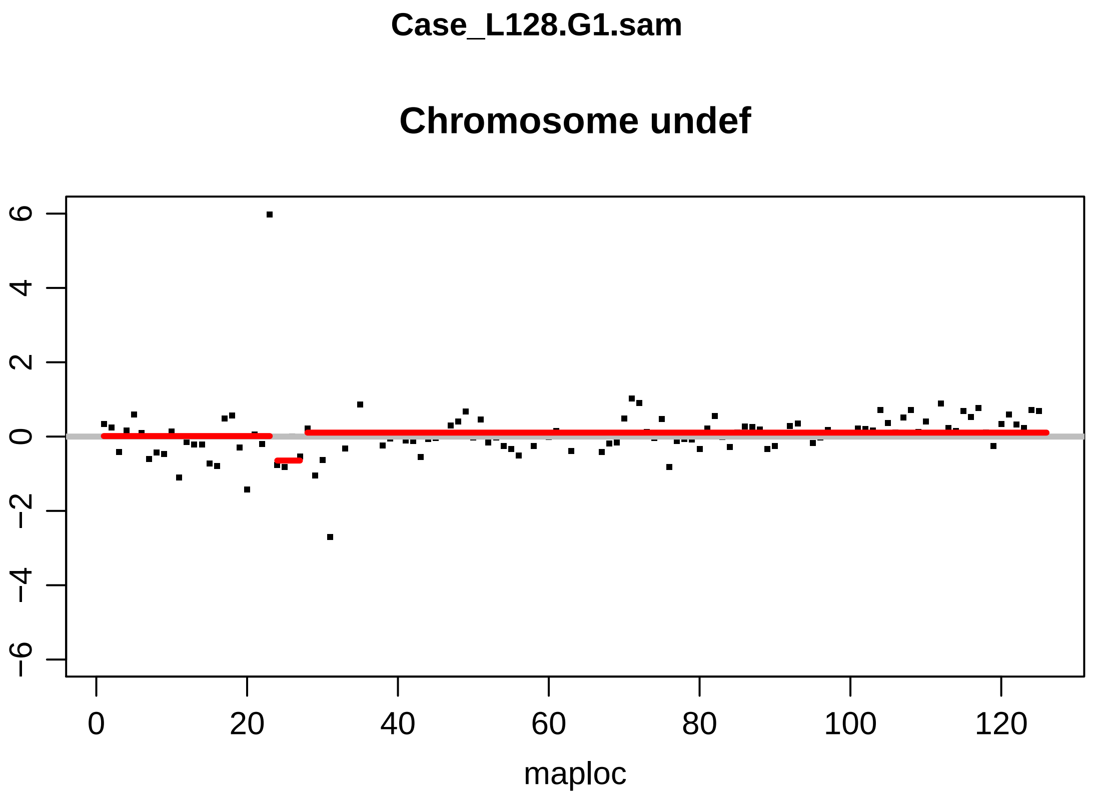


```
## Segplot might not work because of special characters in the sample names. Use only A-Z,a-z and 0-9!
##  There is a hidden function cn.mops:::.replaceNames that replaces the names in the "CNVDetectionResul
```

Case_L129.G1.sam

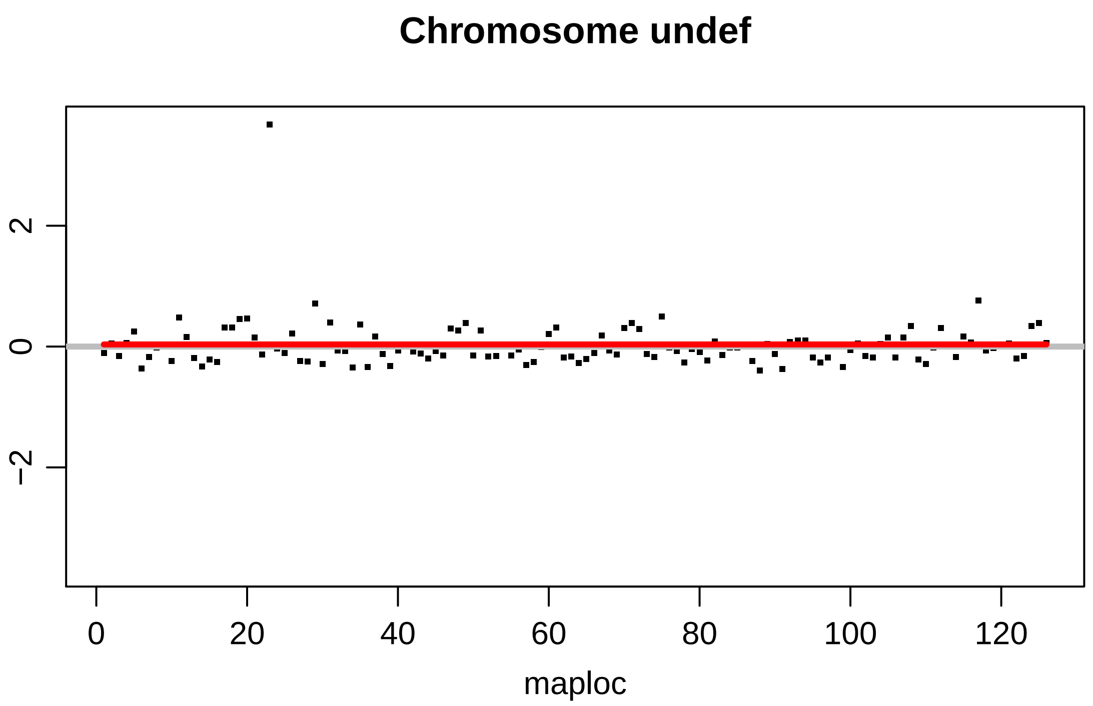


```
## Segplot might not work because of special characters in the sample names. Use only A-Z,a-z and 0-9!
##  There is a hidden function cn.mops:::.replaceNames that replaces the names in the "CNVDetectionResul
```

Case_L130.G1.sam

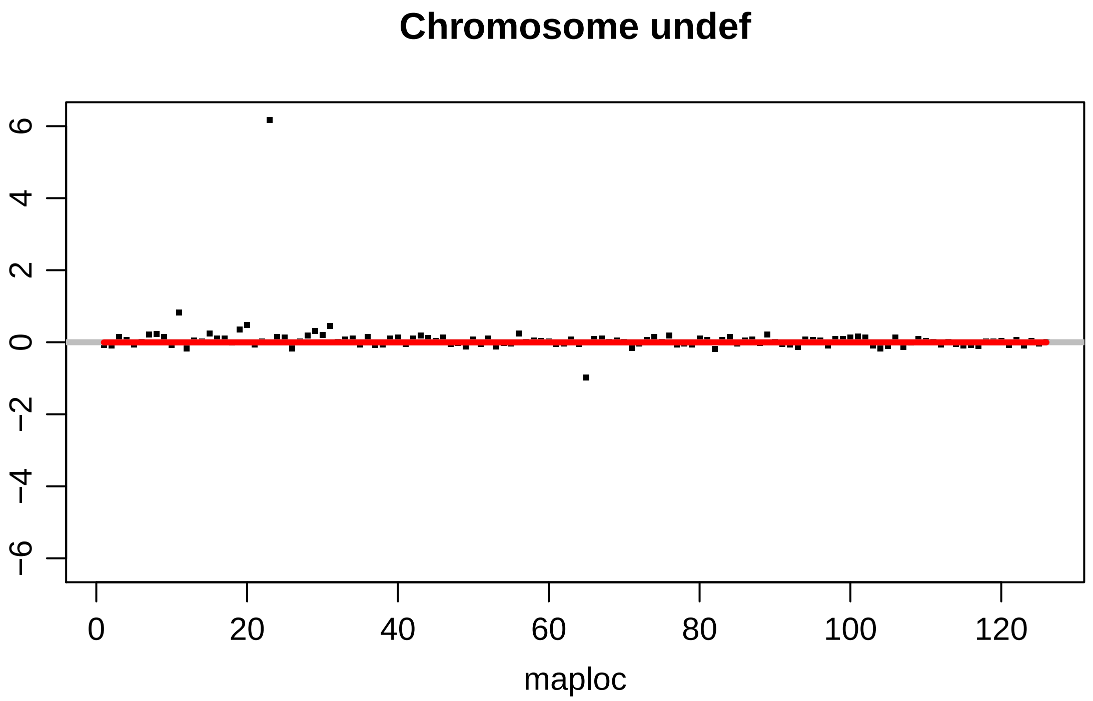

```
## Segplot might not work because of special characters in the sample names. Use only A-Z,a-z and 0-9!
##  There is a hidden function cn.mops:::.replaceNames that replaces the names in the "CNVDetectionResul
```

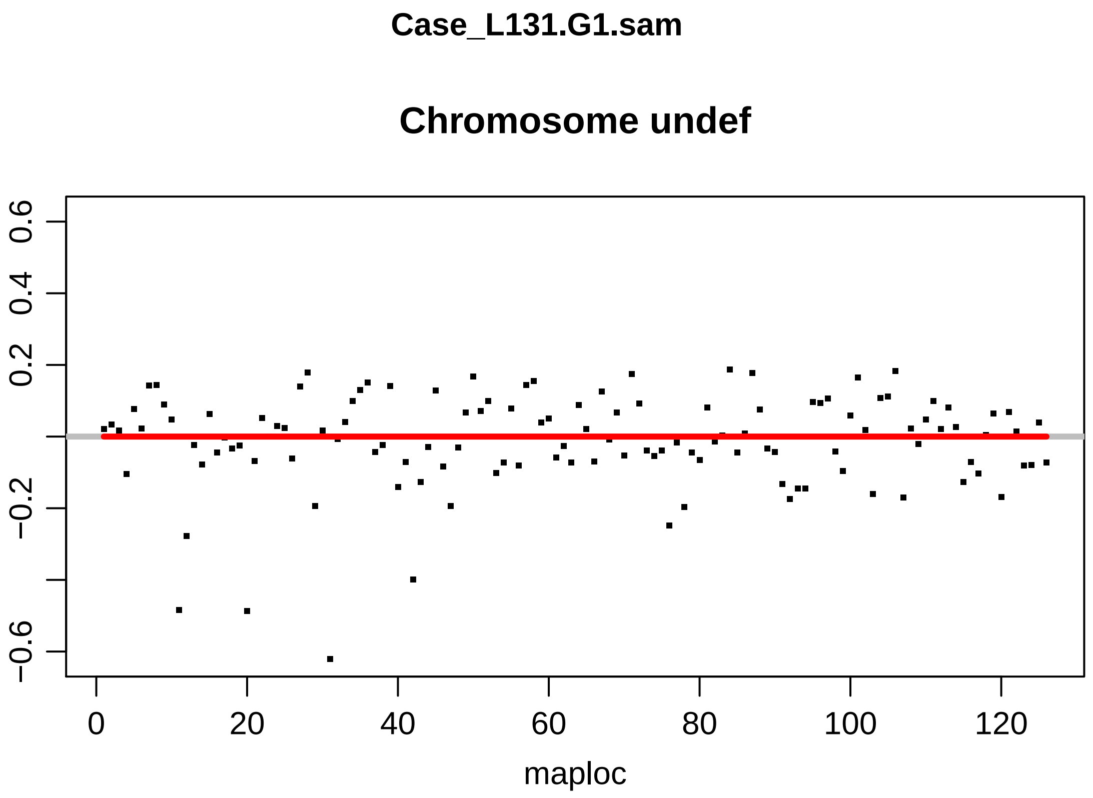


```
## Segplot might not work because of special characters in the sample names. Use only A-Z,a-z and 0-9!
##  There is a hidden function cn.mops:::.replaceNames that replaces the names in the "CNVDetectionResul
```

Case_L132.G1.sam

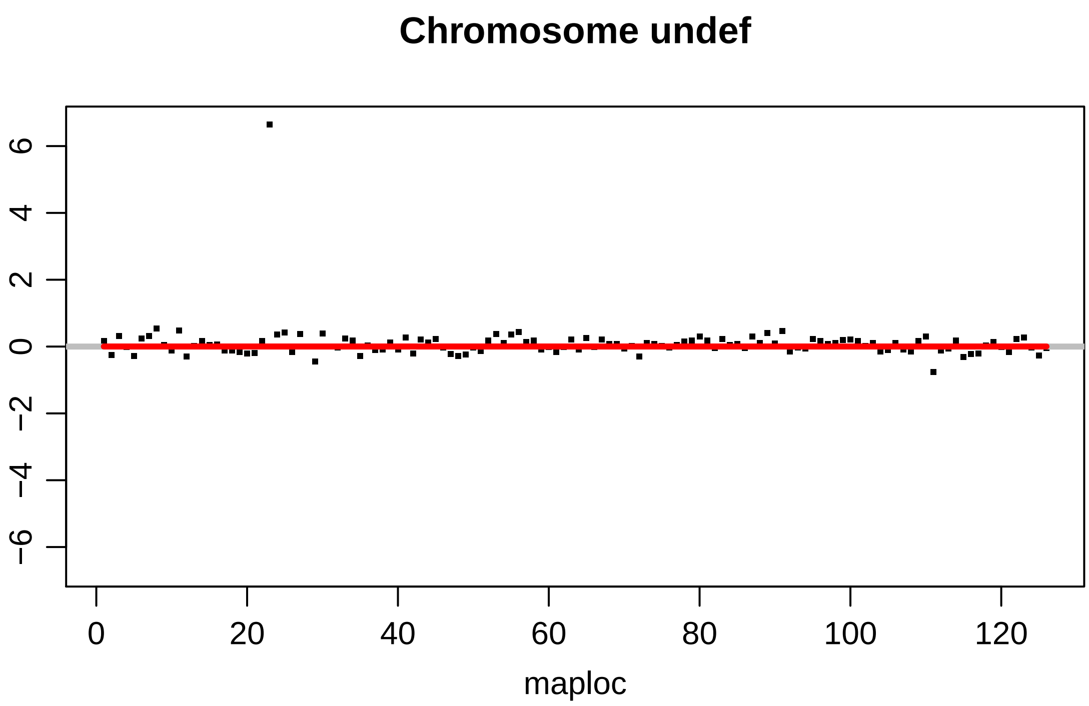


```
## Segplot might not work because of special characters in the sample names. Use only A-Z,a-z and 0-9!
##  There is a hidden function cn.mops:::.replaceNames that replaces the names in the "CNVDetectionResul
```

Case_L133.G1.sam

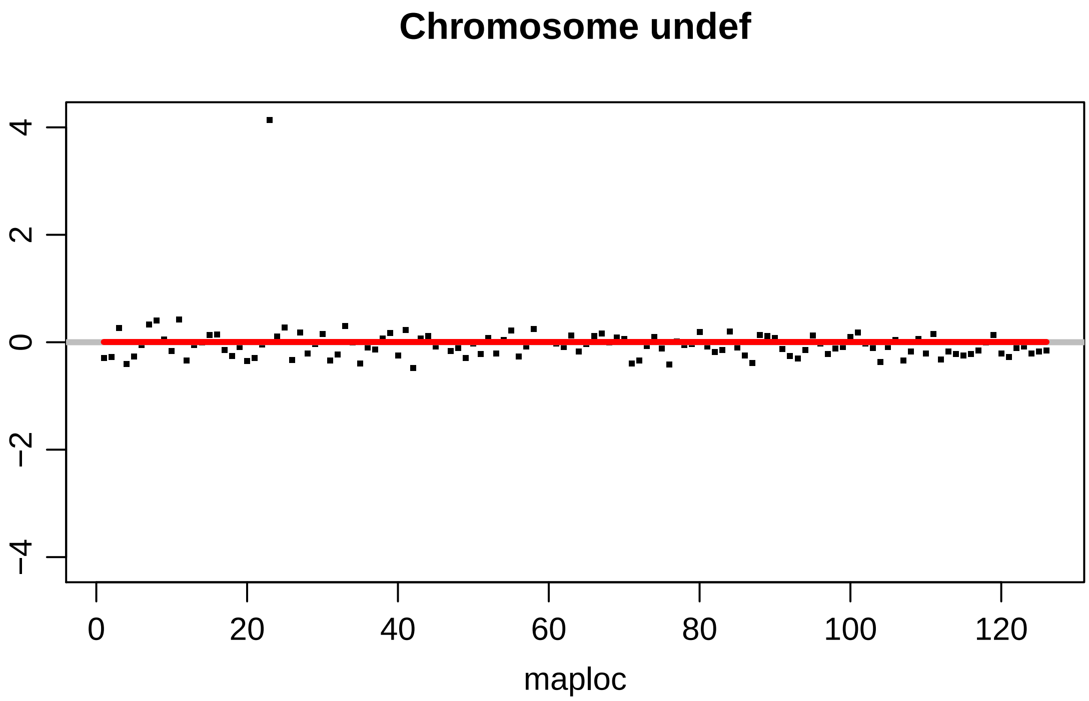

```
## Segplot might not work because of special characters in the sample names. Use only A-Z,a-z and 0-9!
##  There is a hidden function cn.mops:::.replaceNames that replaces the names in the "CNVDetectionResul
```

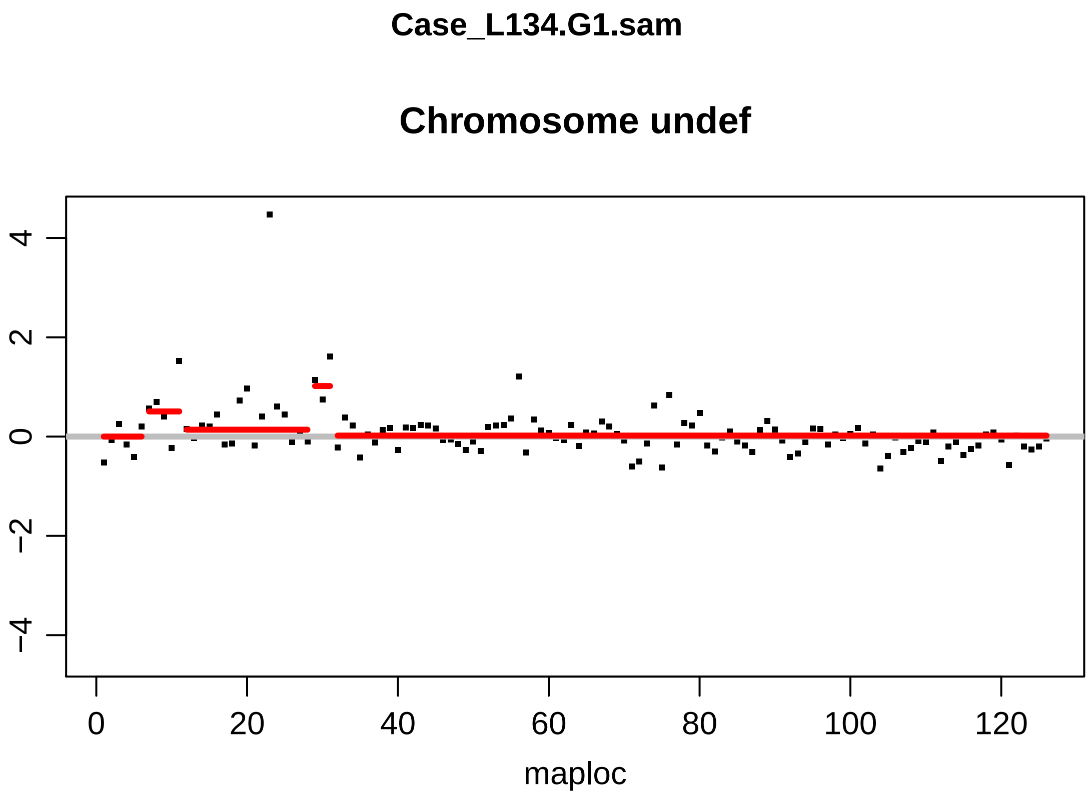


```
## Segplot might not work because of special characters in the sample names. Use only A-Z,a-z and 0-9!
##  There is a hidden function cn.mops:::.replaceNames that replaces the names in the "CNVDetectionResul
```

Case_L135.G1.sam

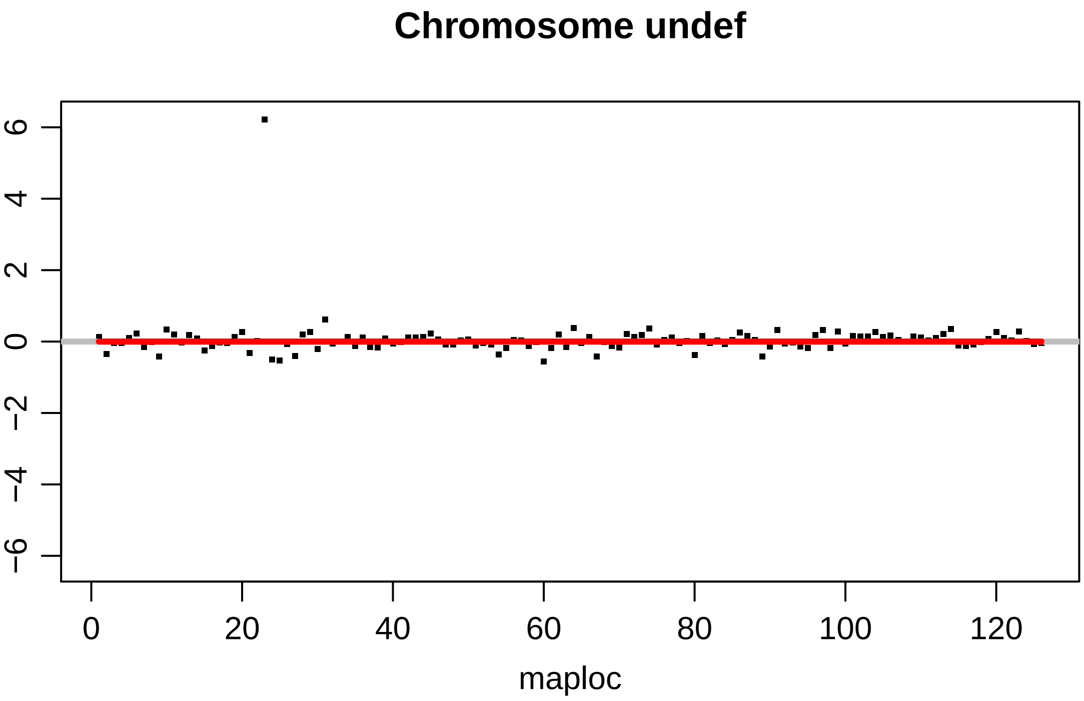


```
## Segplot might not work because of special characters in the sample names. Use only A-Z,a-z and 0-9!
##  There is a hidden function cn.mops:::.replaceNames that replaces the names in the "CNVDetectionResul
```

Case_L136.G1.sam

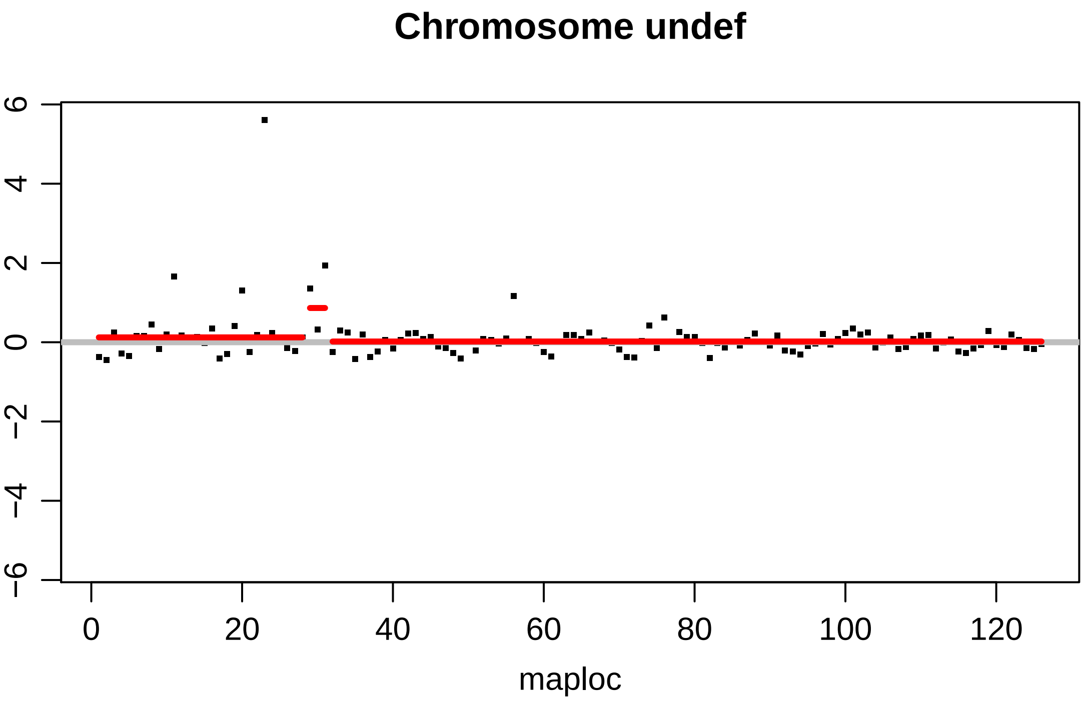

```
## Segplot might not work because of special characters in the sample names. Use only A-Z,a-z and 0-9!
##  There is a hidden function cn.mops:::.replaceNames that replaces the names in the "CNVDetectionResul
```

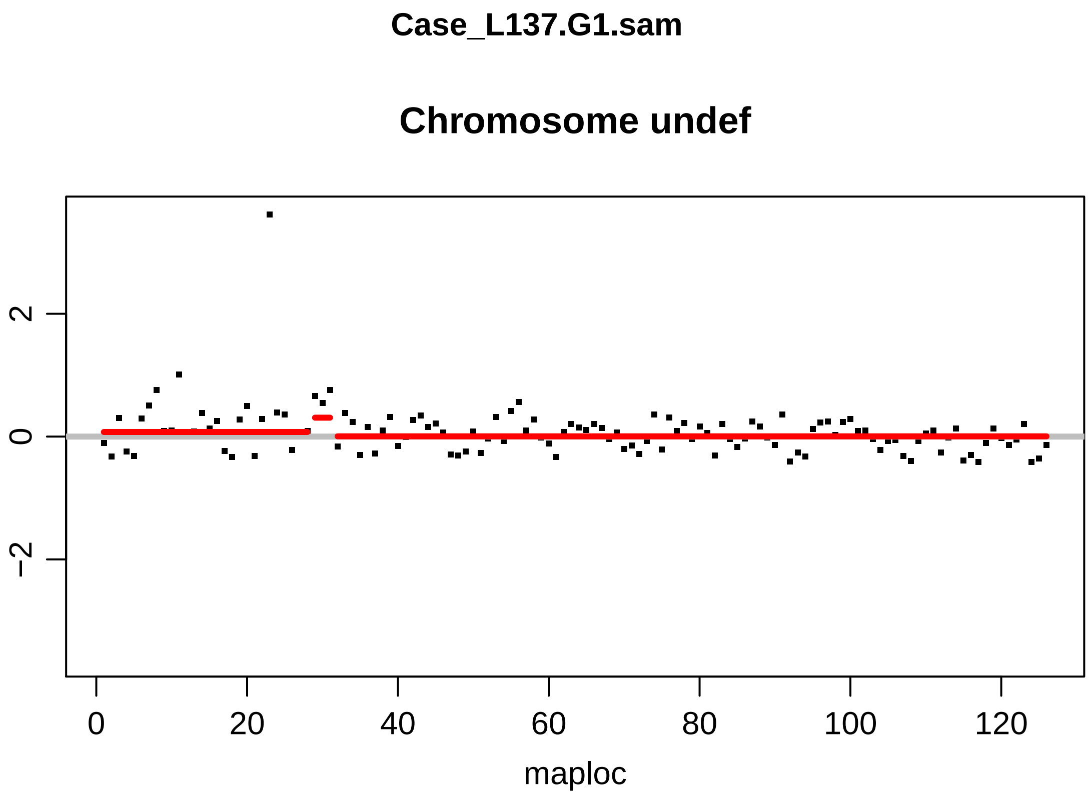


```
## Segplot might not work because of special characters in the sample names. Use only A-Z,a-z and 0-9!
##  There is a hidden function cn.mops:::.replaceNames that replaces the names in the "CNVDetectionResul
```

Case_L138.G1.sam

Chromosome undef

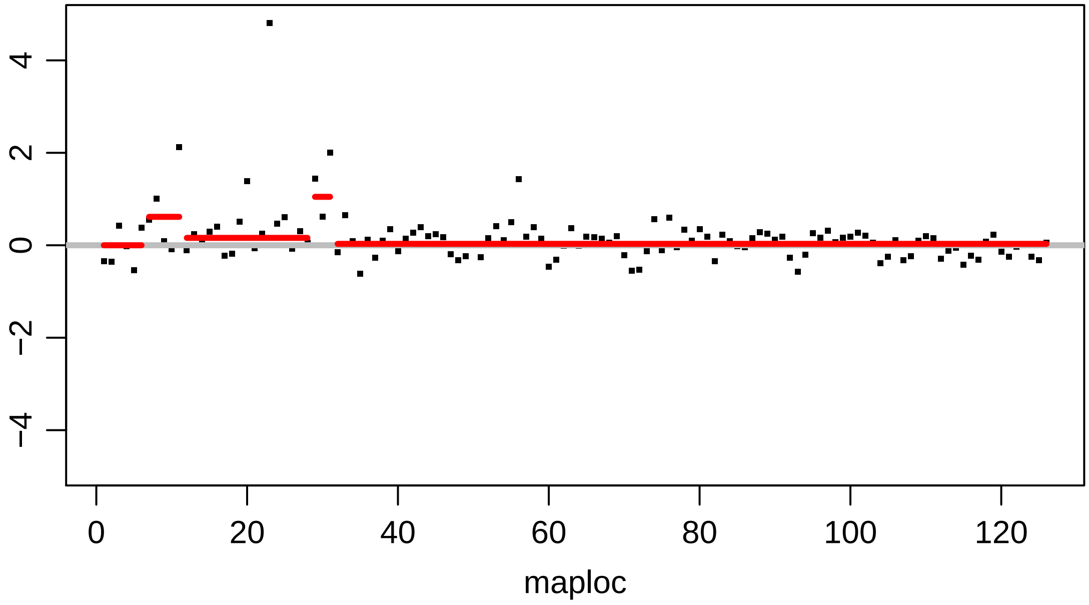


```
## Segplot might not work because of special characters in the sample names. Use only A-Z,a-z and 0-9!
##  There is a hidden function cn.mops:::.replaceNames that replaces the names in the "CNVDetectionResu
```

Case_L139.G1.sam

Chromosome undef

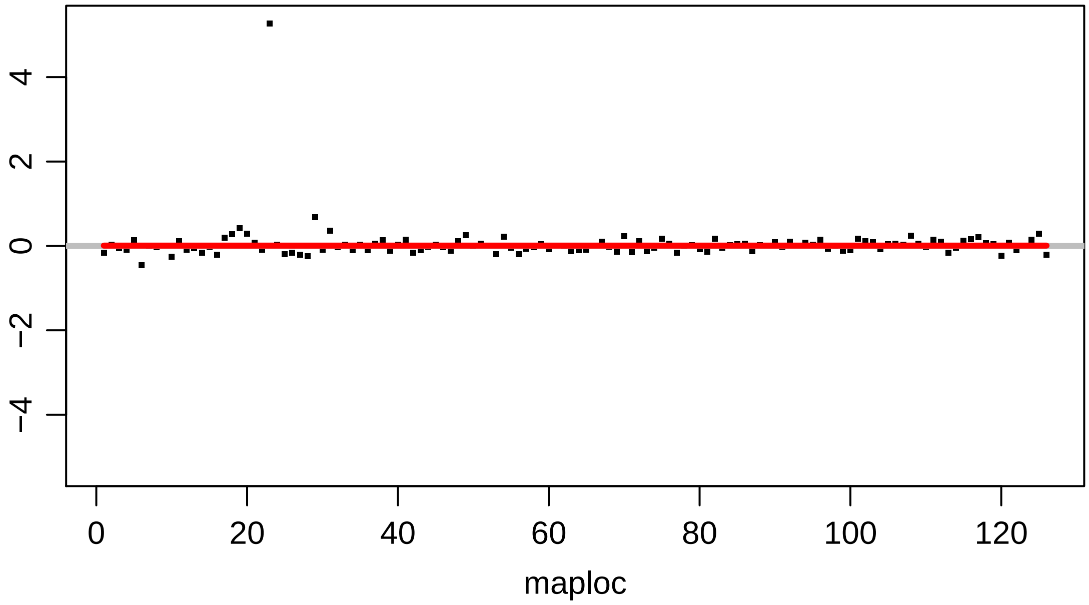

```
## Segplot might not work because of special characters in the sample names. Use only A-Z,a-z and 0-9!
##  There is a hidden function cn.mops:::.replaceNames that replaces the names in the "CNVDetectionResul
```

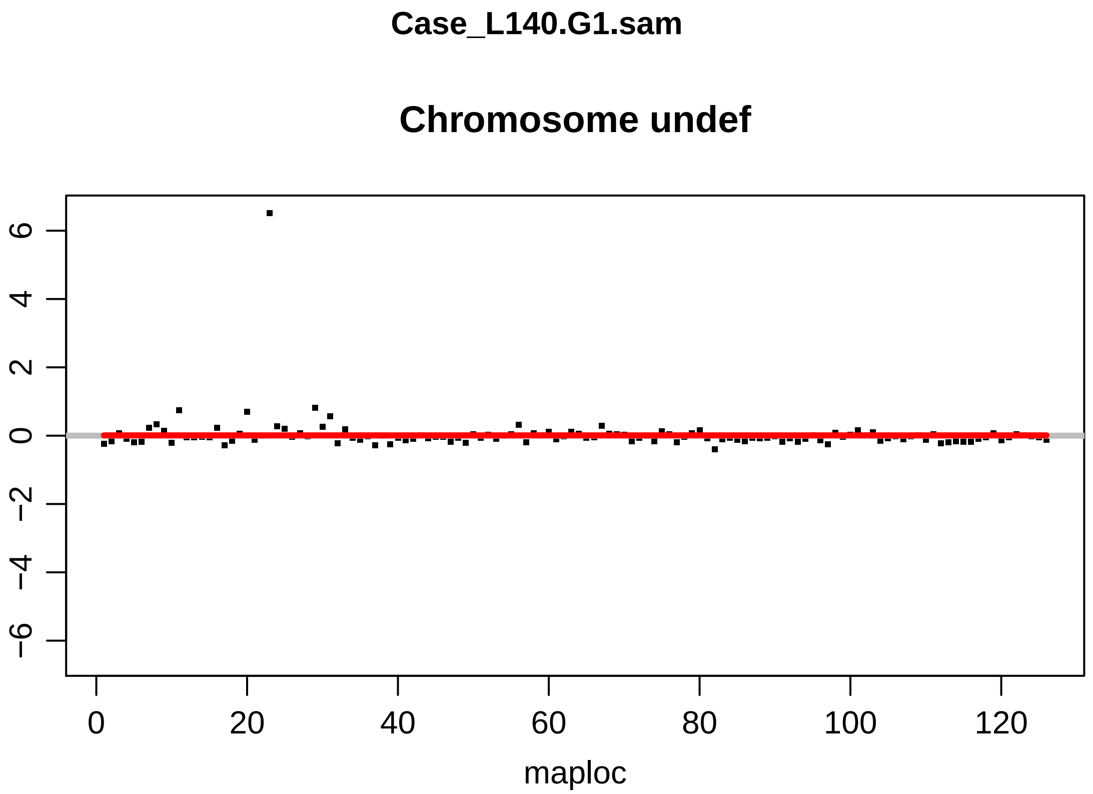


```
##
## CNV regions:
## GRanges object with 5 ranges and 46 metadata columns:
##       seqnames    ranges strand | Case_CONTROL.L1.sam Case_L096.G1.sam
##          <Rle> <IRanges>  <Rle> |            <factor>         <factor>
##   [1]    undef  [ 7, 11]      * |                 CN2              CN2
##   [2]    undef  [17, 20]      * |                 CN2              CN2
##   [3]    undef  [29, 31]      * |                 CN2              CN2
##   [4]    undef  [47, 49]      * |                 CN2              CN2
##   [5]    undef  [70, 72]      * |                 CN3              CN2
##       Case_L097.G1.sam Case_L098.G1.sam Case_L099.G1.sam Case_L100.G1.sam
##               <factor>         <factor>         <factor>         <factor>
##   [1]              CN2              CN2              CN2              CN2
##   [2]              CN2              CN2              CN2              CN2
##   [3]              CN2              CN2              CN2              CN2
##   [4]              CN2              CN2              CN3              CN2
##   [5]              CN2              CN2              CN3              CN2
##       Case_L101.G1.sam Case_L102.G1.sam Case_L103.G1.sam Case_L104.G1.sam
##               <factor>         <factor>         <factor>         <factor>
##   [1]              CN2              CN2              CN2              CN2
##   [2]              CN2              CN2              CN2              CN2
##   [3]              CN2              CN2              CN2              CN2
##   [4]              CN2              CN2              CN2              CN2
##   [5]              CN2              CN2              CN2              CN2
##       Case_L105.G1.sam Case_L106.G1.sam Case_L107.G1.sam Case_L108.G1.sam
```

```
##                <factor>          <factor>          <factor>          <factor>
##   [1]               CN2               CN2               CN2               CN2
##   [2]               CN2               CN2               CN2               CN2
##   [3]               CN2               CN2               CN2               CN2
##   [4]               CN2               CN2               CN2               CN2
##   [5]               CN2               CN2               CN2               CN2
##       Case_L109.G1.sam Case_L110.G1.sam Case_L111.G1.sam Case_L112.G1.sam
##                <factor>          <factor>          <factor>          <factor>
##   [1]               CN2               CN2               CN2               CN2
##   [2]               CN2               CN2               CN3               CN2
##   [3]               CN2               CN2               CN2               CN2
##   [4]               CN2               CN2               CN2               CN2
##   [5]               CN3               CN2               CN2               CN2
##       Case_L113.G1.sam Case_L114.G1.sam Case_L115.G1.sam Case_L116.G1.sam
##                <factor>          <factor>          <factor>          <factor>
##   [1]               CN2               CN2               CN2               CN2
##   [2]               CN2               CN3               CN3               CN2
##   [3]               CN3               CN2               CN2               CN2
##   [4]               CN2               CN2               CN2               CN3
##   [5]               CN2               CN2               CN4               CN4
##       Case_L117.G1.sam Case_L118.G1.sam Case_L119.G1.sam Case_L120.G1.sam
##                <factor>          <factor>          <factor>          <factor>
##   [1]               CN2               CN2               CN2               CN2
##   [2]               CN2               CN3               CN2               CN2
##   [3]               CN2               CN2               CN2               CN3
##   [4]               CN2               CN2               CN2               CN2
##   [5]               CN2               CN2               CN2               CN2
##       Case_L121.G1.sam Case_L122.G1.sam Case_L123.G1.sam Case_L124.G1.sam
##                <factor>          <factor>          <factor>          <factor>
##   [1]               CN2               CN2               CN2               CN2
##   [2]               CN2               CN2               CN2               CN2
##   [3]               CN2               CN2               CN2               CN2
##   [4]               CN2               CN2               CN2               CN2
##   [5]               CN2               CN2               CN2               CN2
##       Case_L125.G1.sam Case_L126.G1.sam Case_L128.G1.sam Case_L129.G1.sam
##                <factor>          <factor>          <factor>          <factor>
##   [1]               CN2               CN2               CN2               CN2
##   [2]               CN2               CN2               CN2               CN2
##   [3]               CN2               CN2               CN2               CN2
##   [4]               CN2               CN2               CN2               CN2
##   [5]               CN4               CN2               CN2               CN2
##       Case_L130.G1.sam Case_L131.G1.sam Case_L132.G1.sam Case_L133.G1.sam
##                <factor>          <factor>          <factor>          <factor>
##   [1]               CN2               CN2               CN2               CN2
##   [2]               CN2               CN2               CN2               CN2
##   [3]               CN2               CN2               CN2               CN2
##   [4]               CN2               CN2               CN2               CN2
##   [5]               CN2               CN2               CN2               CN2
##       Case_L134.G1.sam Case_L135.G1.sam Case_L136.G1.sam Case_L137.G1.sam
##                <factor>          <factor>          <factor>          <factor>
##   [1]               CN3               CN2               CN2               CN2
##   [2]               CN2               CN2               CN2               CN2
##   [3]               CN3               CN2               CN3               CN2
##   [4]               CN2               CN2               CN2               CN2
```

```
##   [5]              CN2              CN2              CN2              CN2
##       Case_L138.G1.sam Case_L139.G1.sam Case_L140.G1.sam Case_L141.G1.sam
##               <factor>         <factor>         <factor>         <factor>
##   [1]              CN3              CN2              CN2              CN2
##   [2]              CN2              CN2              CN2              CN2
##   [3]              CN3              CN2              CN2              CN3
##   [4]              CN2              CN2              CN2              CN2
##   [5]              CN2              CN2              CN2              CN2
##   -------
##   seqinfo: 1 sequence from an unspecified genome; no seqlengths
##
## Individual CNVs:
## GRanges object with 20 ranges and 4 metadata columns:
##        seqnames    ranges strand   |         sampleName    median
##           <Rle> <IRanges>  <Rle>   |           <factor> <numeric>
##    [1]    undef  [70, 72]      *   | Case_CONTROL.L1.sam 0.5060291
##    [2]    undef  [47, 49]      *   |    Case_L099.G1.sam 0.5849622
##    [3]    undef  [70, 72]      *   |    Case_L099.G1.sam 0.5844234
##    [4]    undef  [70, 72]      *   |    Case_L109.G1.sam 0.5720271
##    [5]    undef  [17, 20]      *   |    Case_L111.G1.sam 0.7593813
##    ...      ...       ...    ... ...                 ...       ...
##   [16]    undef  [29, 31]      *   |    Case_L134.G1.sam 0.9646638
##   [17]    undef  [29, 31]      *   |    Case_L136.G1.sam 1.0000569
##   [18]    undef  [ 7, 11]      *   |    Case_L138.G1.sam 0.5804489
##   [19]    undef  [29, 31]      *   |    Case_L138.G1.sam 1.0033021
##   [20]    undef  [29, 31]      *   |    Case_L141.G1.sam 0.9904262
##             mean          CN
##        <numeric> <character>
##    [1] 0.5198849         CN3
##    [2] 0.5771298         CN3
##    [3] 0.5662153         CN3
##    [4] 0.5406707         CN3
##    [5] 0.6292757         CN3
##    ...       ...         ...
##   [16] 1.0181033         CN3
##   [17] 0.8640099         CN3
##   [18] 0.6148906         CN3
##   [19] 1.0479801         CN3
##   [20] 0.7249402         CN3
##   -------
##   seqinfo: 1 sequence from an unspecified genome; no seqlengths
## [1] "/Users/gdemidov/Downloads/doc/Run_SN1_45_CRG_fin_05_qc.xls"

## Normalizing...

## Starting local modeling, please be patient...

## Reference sequence:  undef

## Starting segmentation algorithm...

## Using "fastseg" for segmentation.
```

```
## [1] ""
## [1] "/Users/gdemidov/Downloads/doc/Run_SN1_45_CRG_fin_05_qc.xls"
## [1] ""
```

```
## Segplot might not work because of special characters in the sample names. Use only A-Z,a-z and 0-9!
##  There is a hidden function cn.mops:::.replaceNames that replaces the names in the "CNVDetectionResul
```

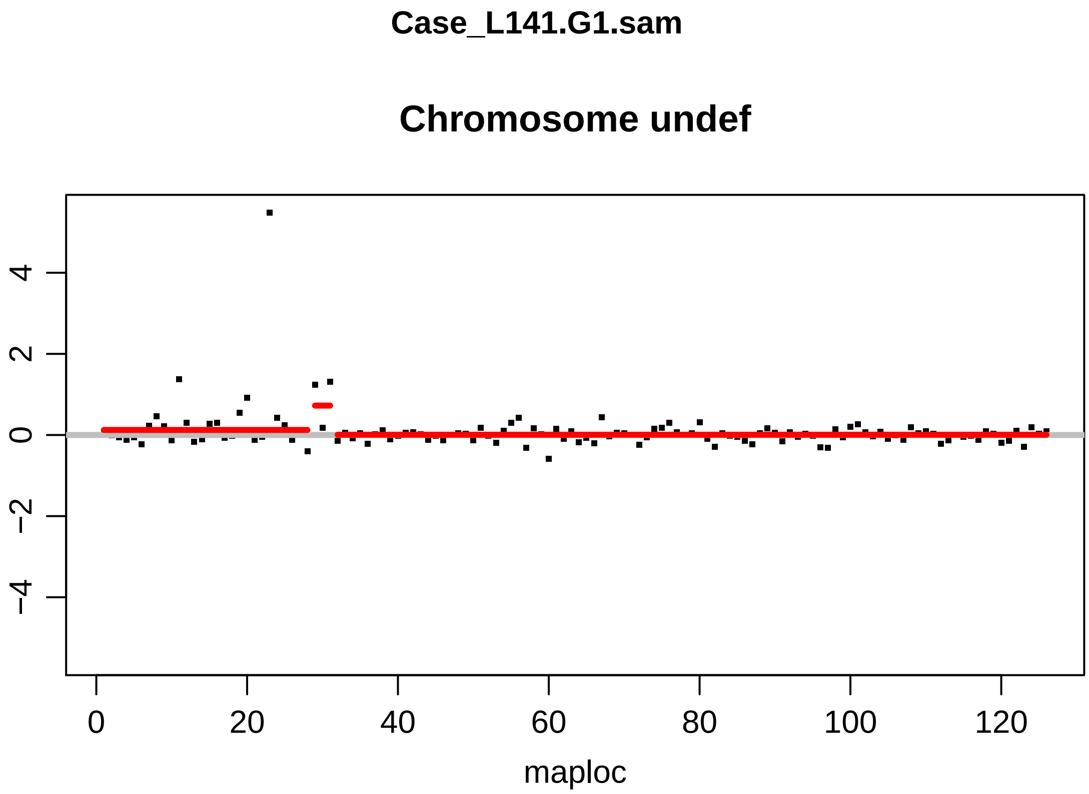


```
## Segplot might not work because of special characters in the sample names. Use only A-Z,a-z and 0-9!
##  There is a hidden function cn.mops:::.replaceNames that replaces the names in the "CNVDetectionResul
```

Case_L142.G1.sam

Chromosome undef

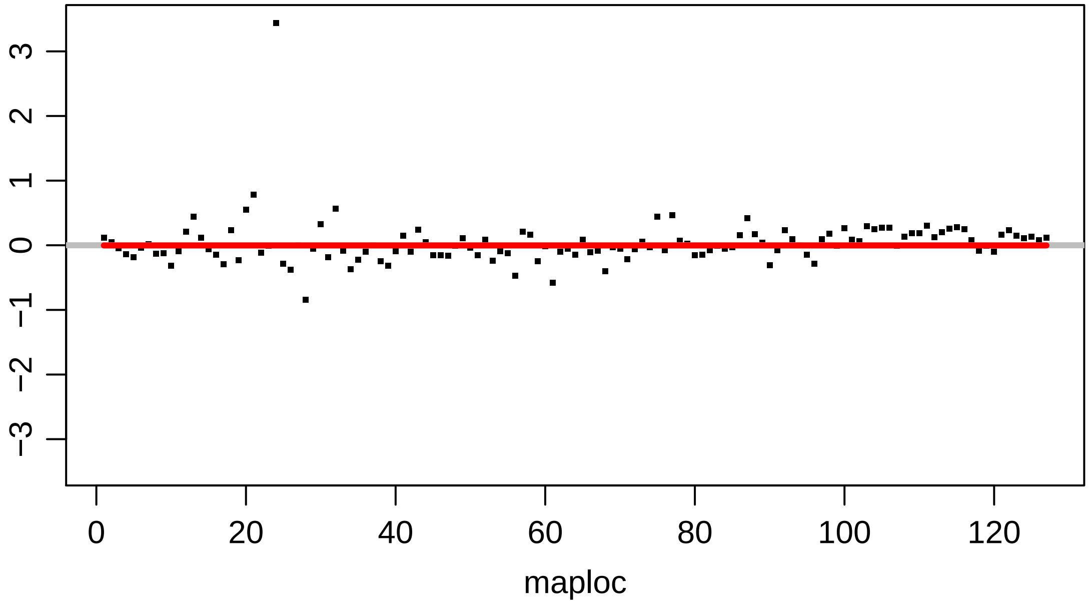


```
## Segplot might not work because of special characters in the sample names. Use only A-Z,a-z and 0-9!
##  There is a hidden function cn.mops:::.replaceNames that replaces the names in the "CNVDetectionResul
```

Case_L143.G1.sam

Chromosome undef

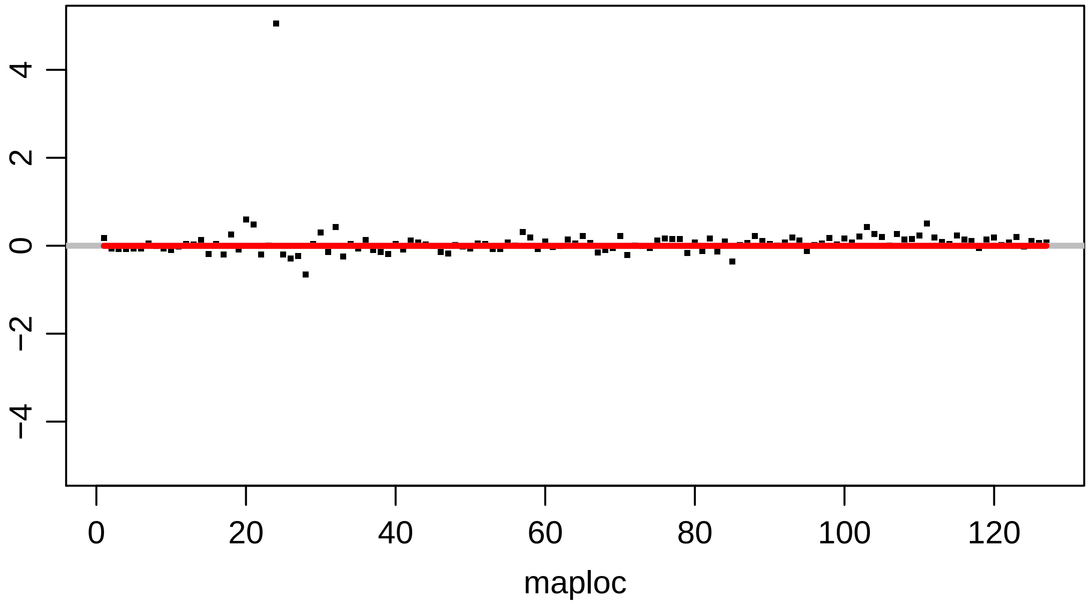

```
## Segplot might not work because of special characters in the sample names. Use only A-Z,a-z and 0-9!
##  There is a hidden function cn.mops:::.replaceNames that replaces the names in the "CNVDetectionResul
```

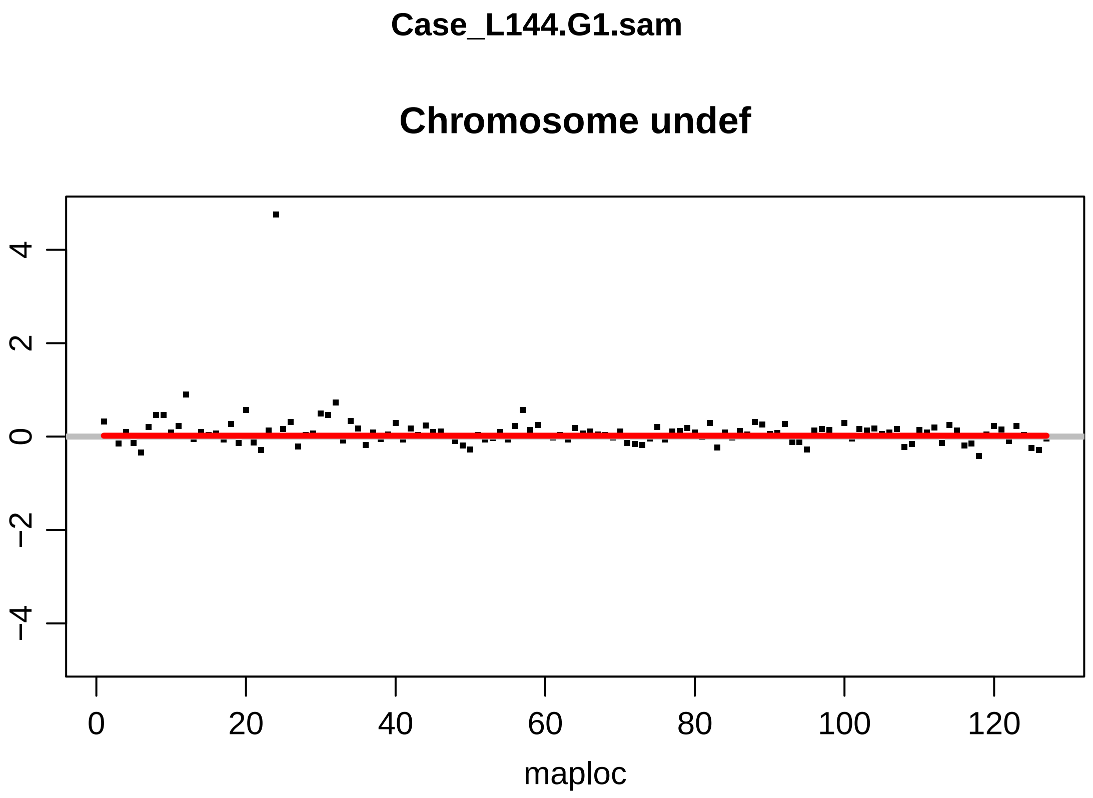


```
## Segplot might not work because of special characters in the sample names. Use only A-Z,a-z and 0-9!
##  There is a hidden function cn.mops:::.replaceNames that replaces the names in the "CNVDetectionResul
```

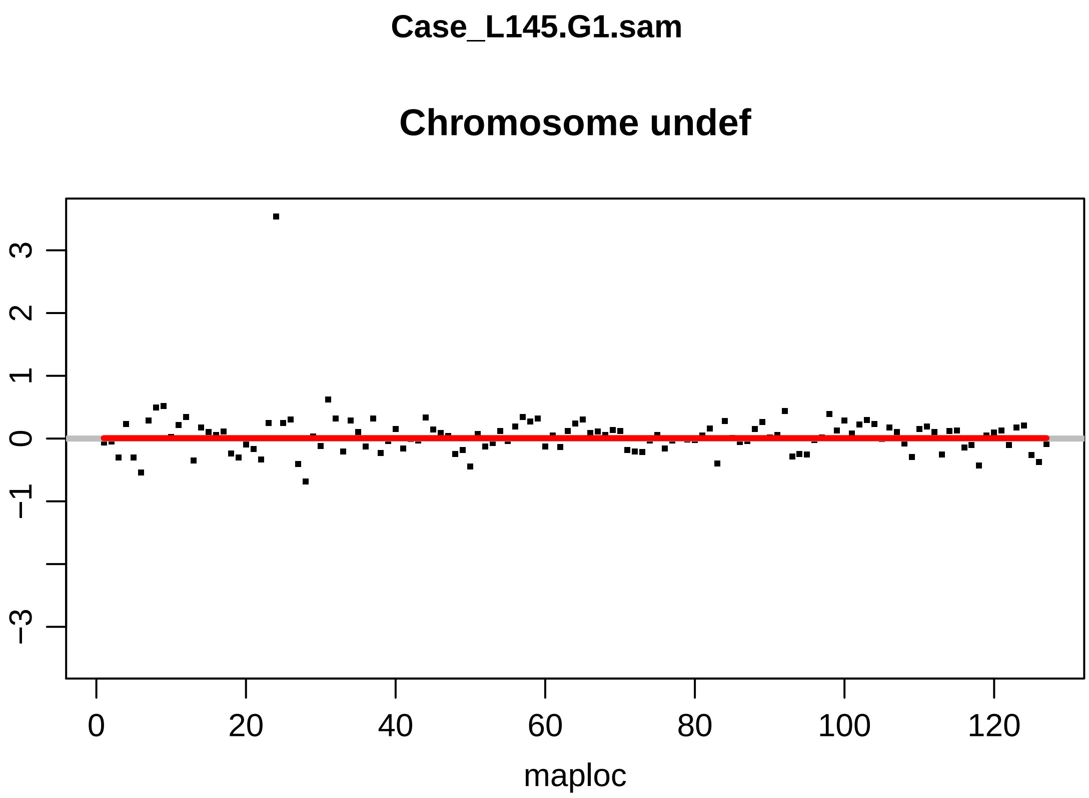


```
## Segplot might not work because of special characters in the sample names. Use only A-Z,a-z and 0-9!
##  There is a hidden function cn.mops:::.replaceNames that replaces the names in the "CNVDetectionResul
```

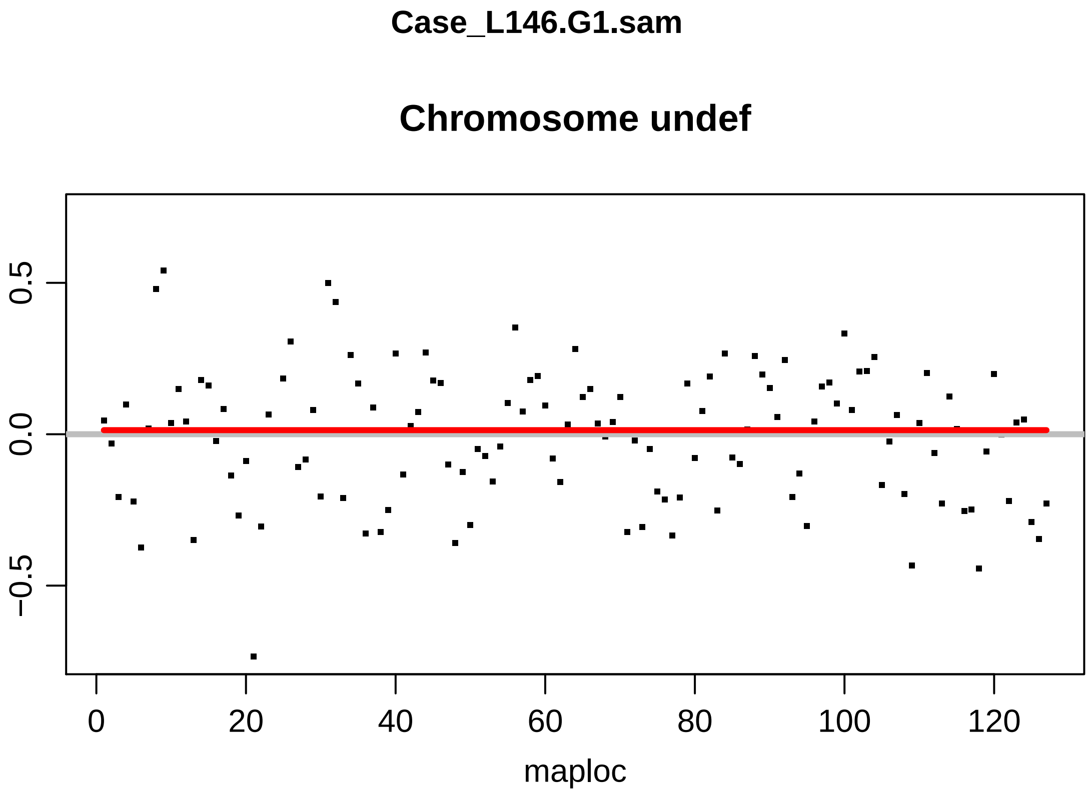

```
## Segplot might not work because of special characters in the sample names. Use only A-Z,a-z and 0-9!
##  There is a hidden function cn.mops:::.replaceNames that replaces the names in the "CNVDetectionResul
```

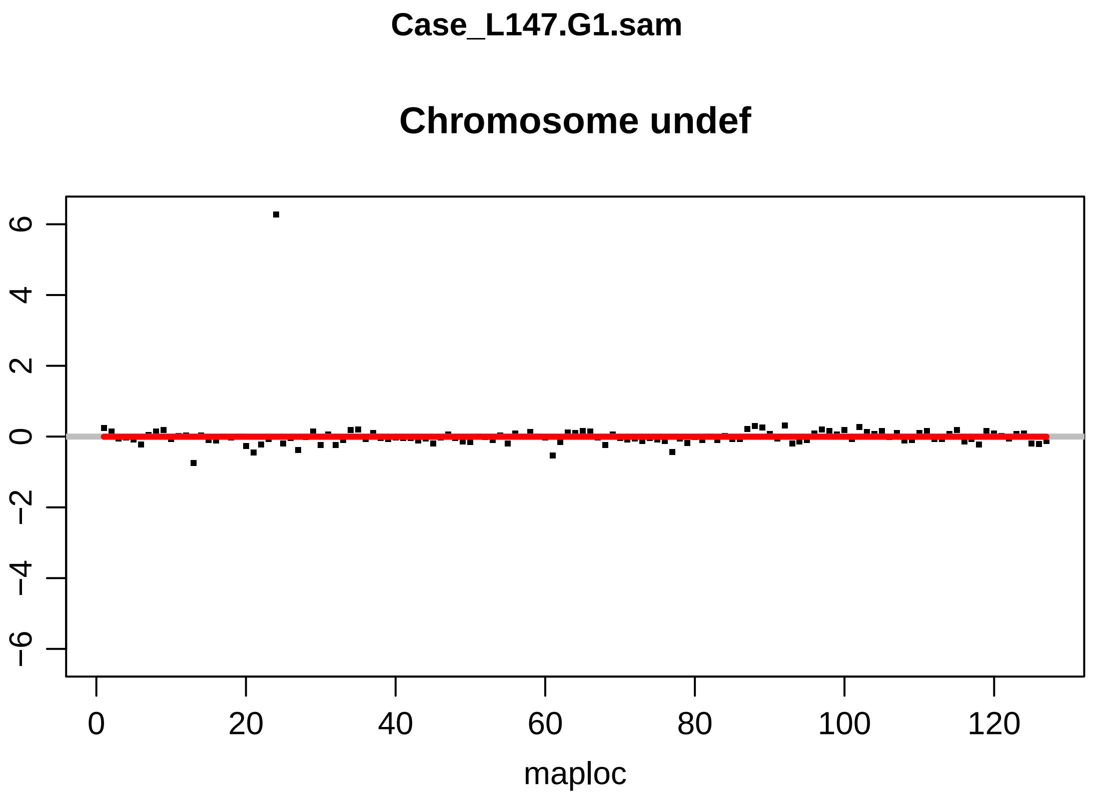


```
## Segplot might not work because of special characters in the sample names. Use only A-Z,a-z and 0-9!
##  There is a hidden function cn.mops:::.replaceNames that replaces the names in the "CNVDetectionResul
```

Case_L149.G1.sam

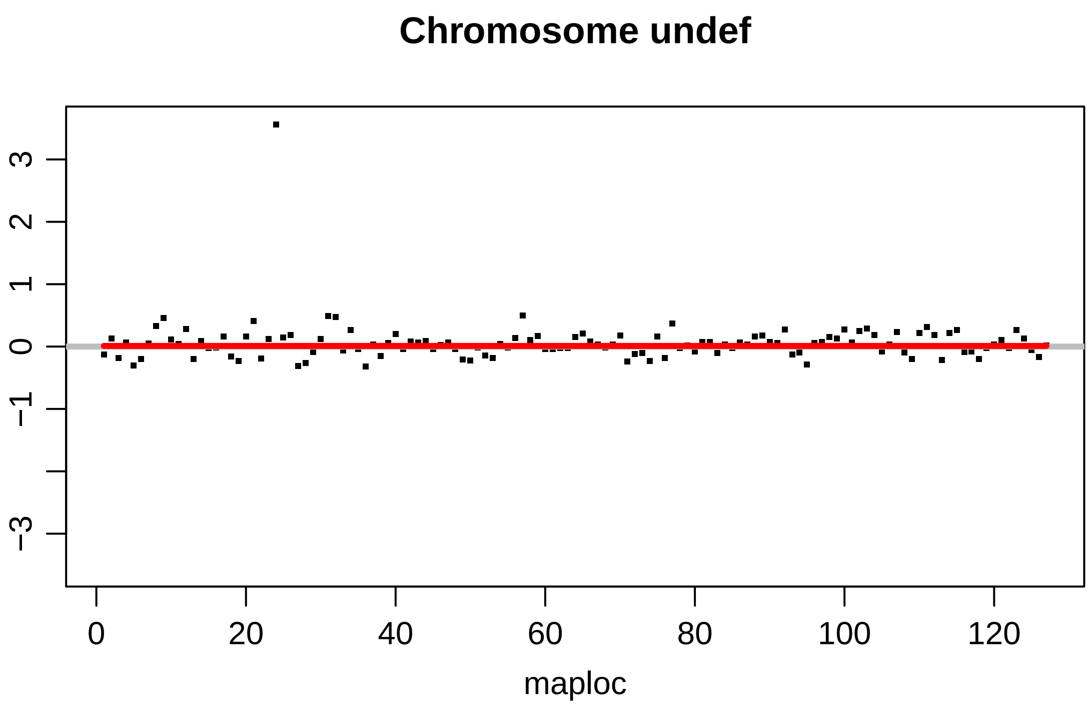


```
## Segplot might not work because of special characters in the sample names. Use only A-Z,a-z and 0-9!
##  There is a hidden function cn.mops:::.replaceNames that replaces the names in the "CNVDetectionResul
```

Case_L150.G1.sam

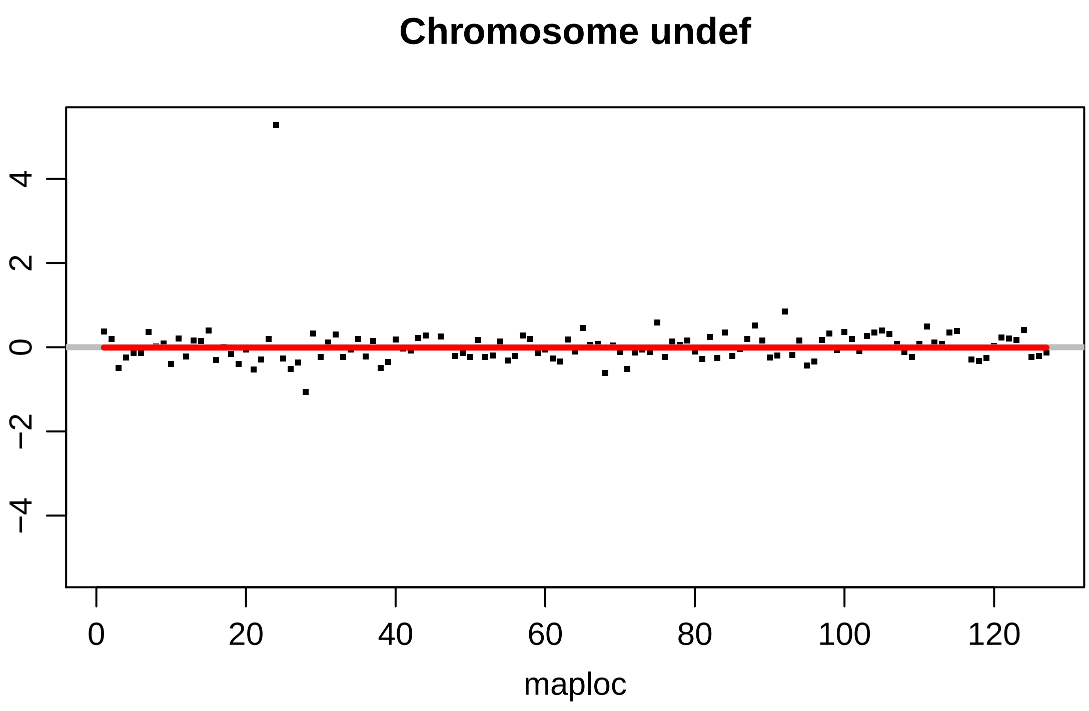

```
## Segplot might not work because of special characters in the sample names. Use only A-Z,a-z and 0-9!
##  There is a hidden function cn.mops:::.replaceNames that replaces the names in the "CNVDetectionResul
```

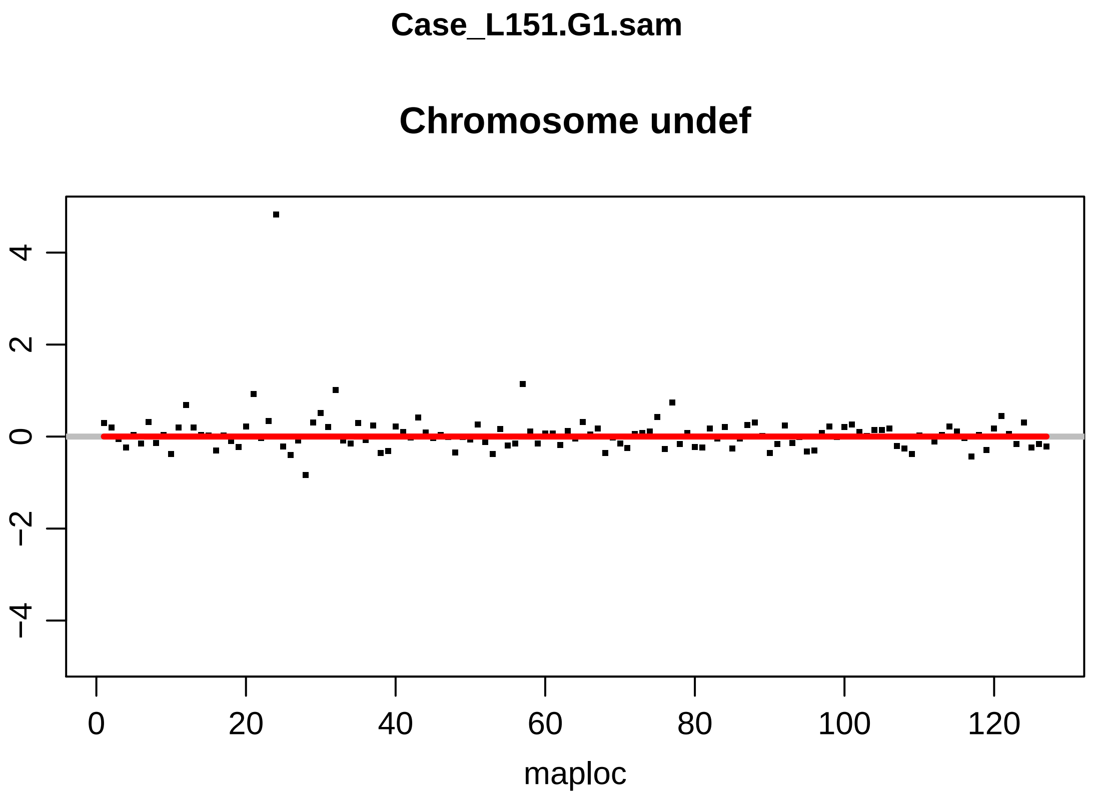


```
## Segplot might not work because of special characters in the sample names. Use only A-Z,a-z and 0-9!
##  There is a hidden function cn.mops:::.replaceNames that replaces the names in the "CNVDetectionResul
```

Case_L152.G1.sam

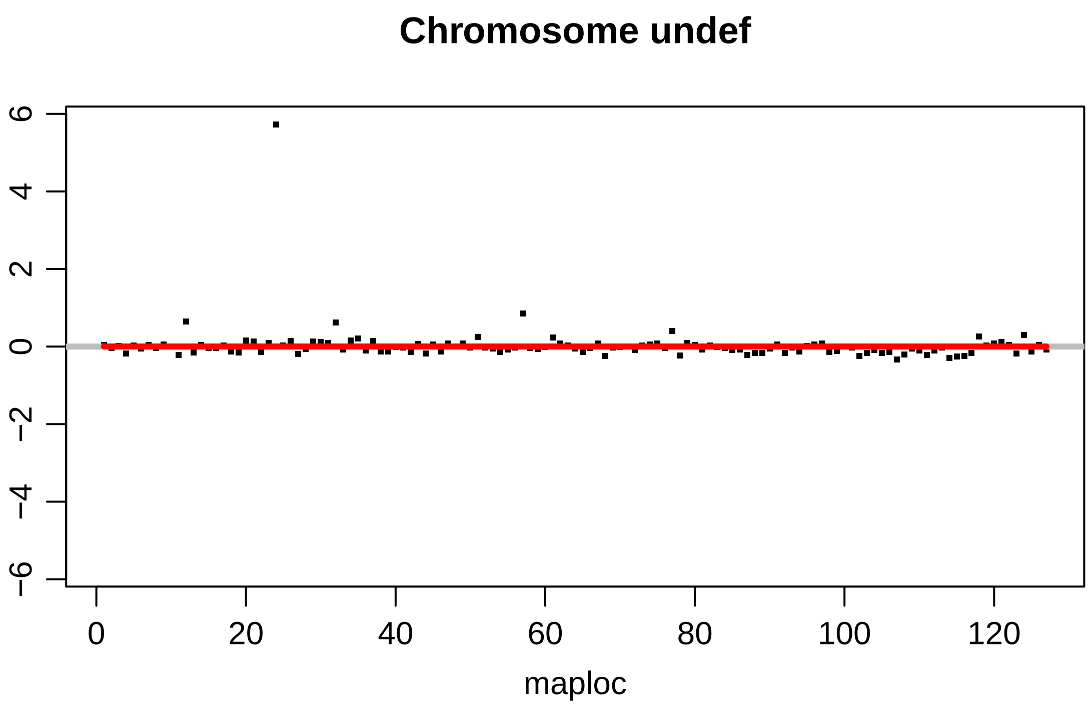


```
## Segplot might not work because of special characters in the sample names. Use only A-Z,a-z and 0-9!
##  There is a hidden function cn.mops:::.replaceNames that replaces the names in the "CNVDetectionResul
```

Case_L154.G1.sam

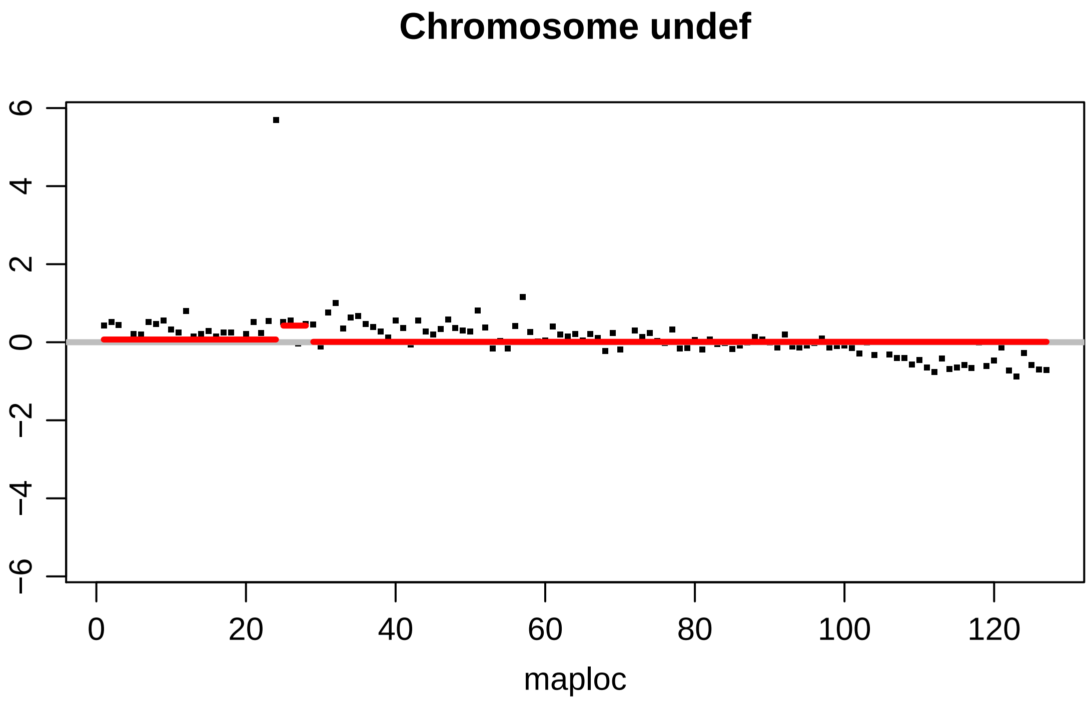

```
## Segplot might not work because of special characters in the sample names. Use only A-Z,a-z and 0-9!
##  There is a hidden function cn.mops:::.replaceNames that replaces the names in the "CNVDetectionResul
```

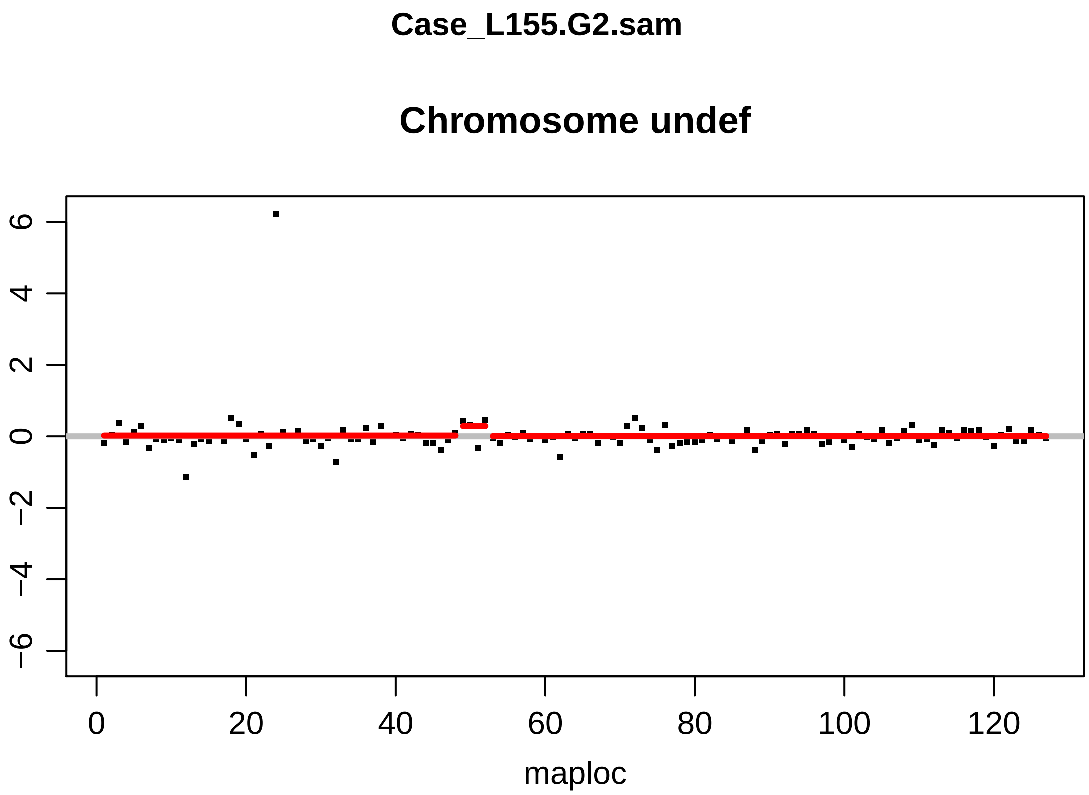


```
## Segplot might not work because of special characters in the sample names. Use only A-Z,a-z and 0-9!
##  There is a hidden function cn.mops:::.replaceNames that replaces the names in the "CNVDetectionResul
```

Case_L157.G2.sam

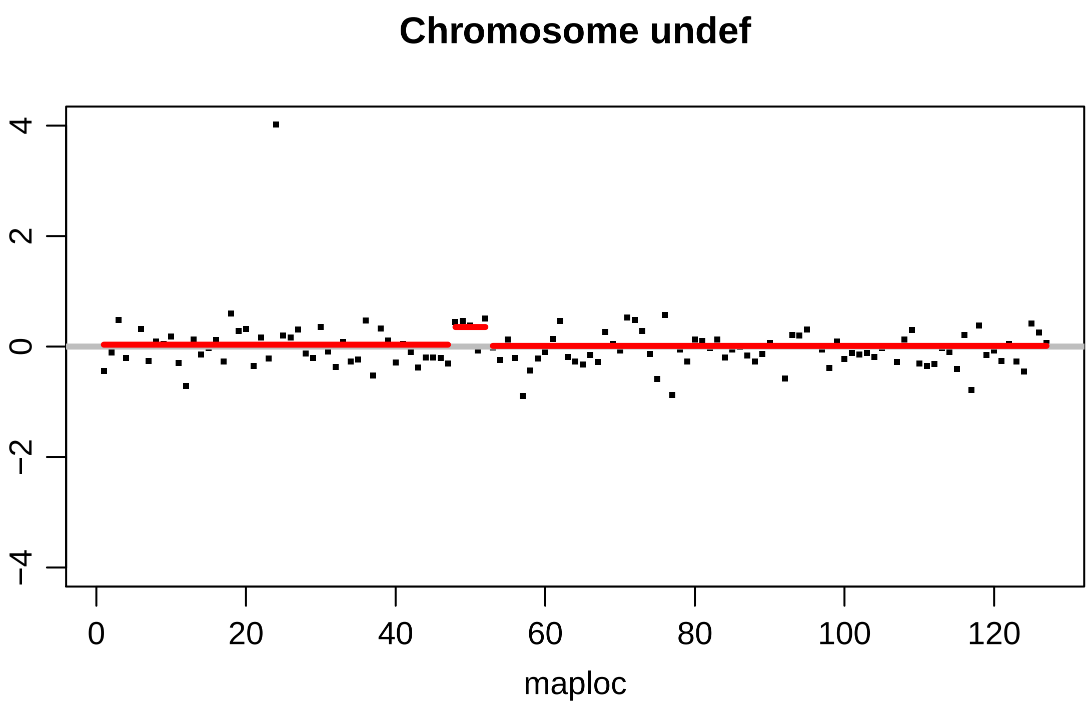


```
## Segplot might not work because of special characters in the sample names. Use only A-Z,a-z and 0-9!
##  There is a hidden function cn.mops:::.replaceNames that replaces the names in the "CNVDetectionResul
```

Case_L158.G2.sam

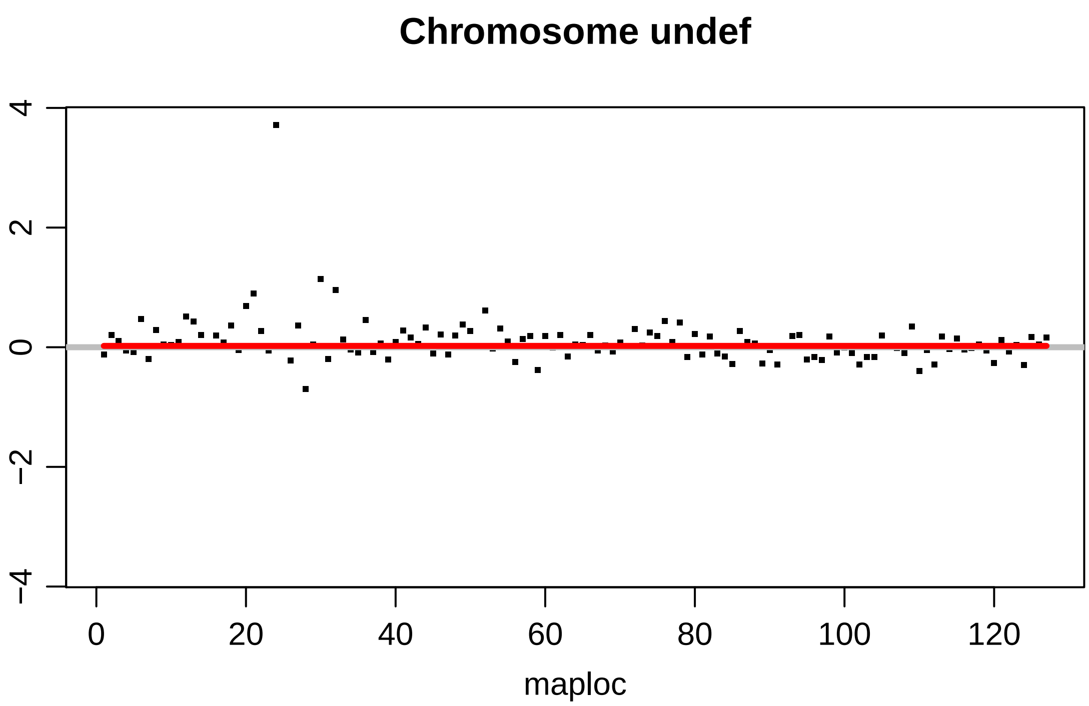

```
## Segplot might not work because of special characters in the sample names. Use only A-Z,a-z and 0-9!
##  There is a hidden function cn.mops:::.replaceNames that replaces the names in the "CNVDetectionResul
```

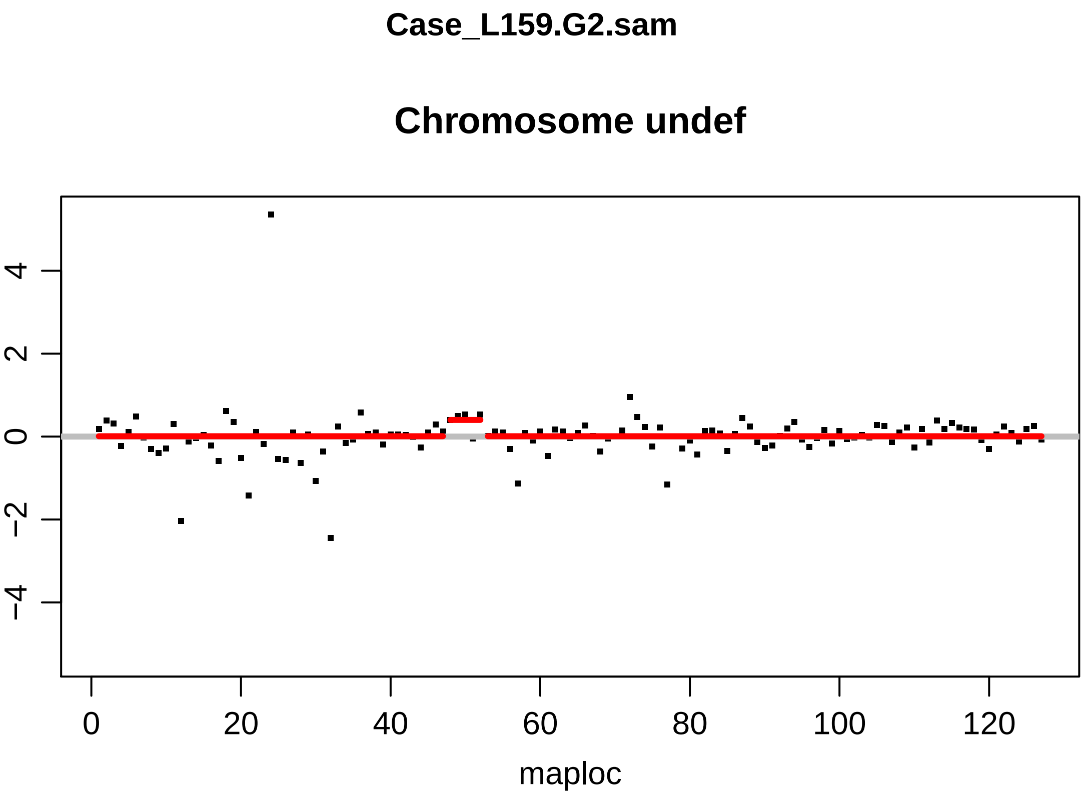


```
## Segplot might not work because of special characters in the sample names. Use only A-Z,a-z and 0-9!
##  There is a hidden function cn.mops:::.replaceNames that replaces the names in the "CNVDetectionResul
```

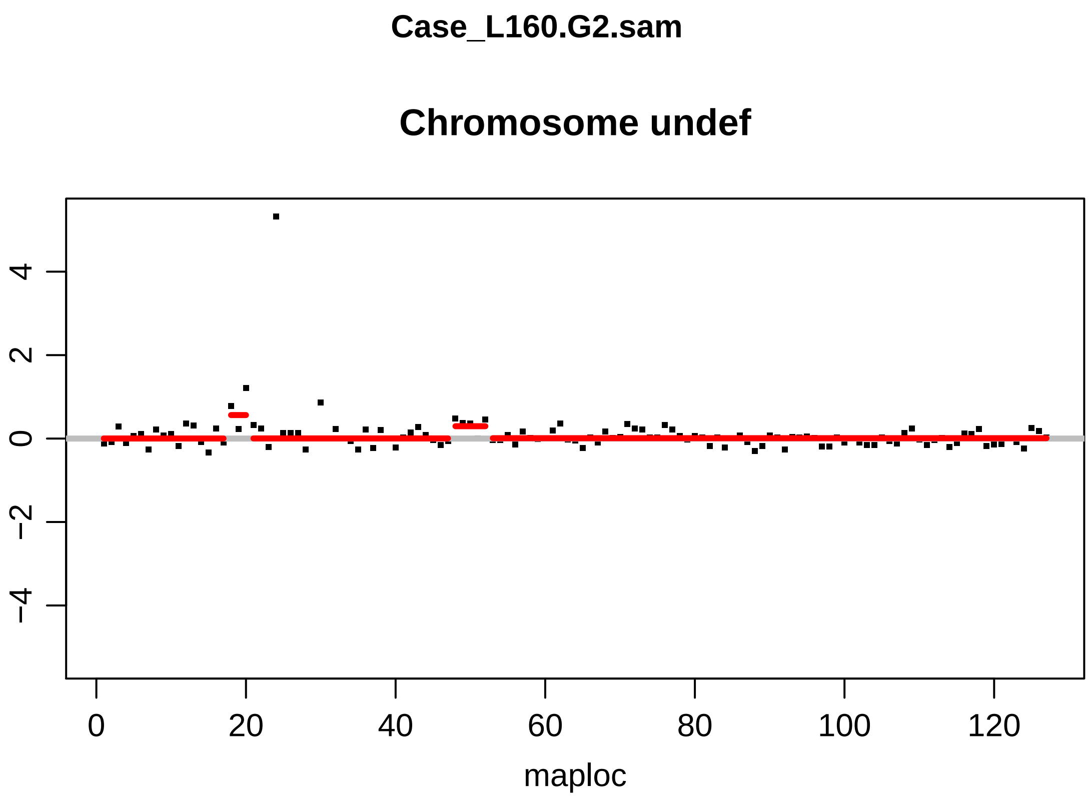


```
## Segplot might not work because of special characters in the sample names. Use only A-Z,a-z and 0-9!
##  There is a hidden function cn.mops:::.replaceNames that replaces the names in the "CNVDetectionResul
```

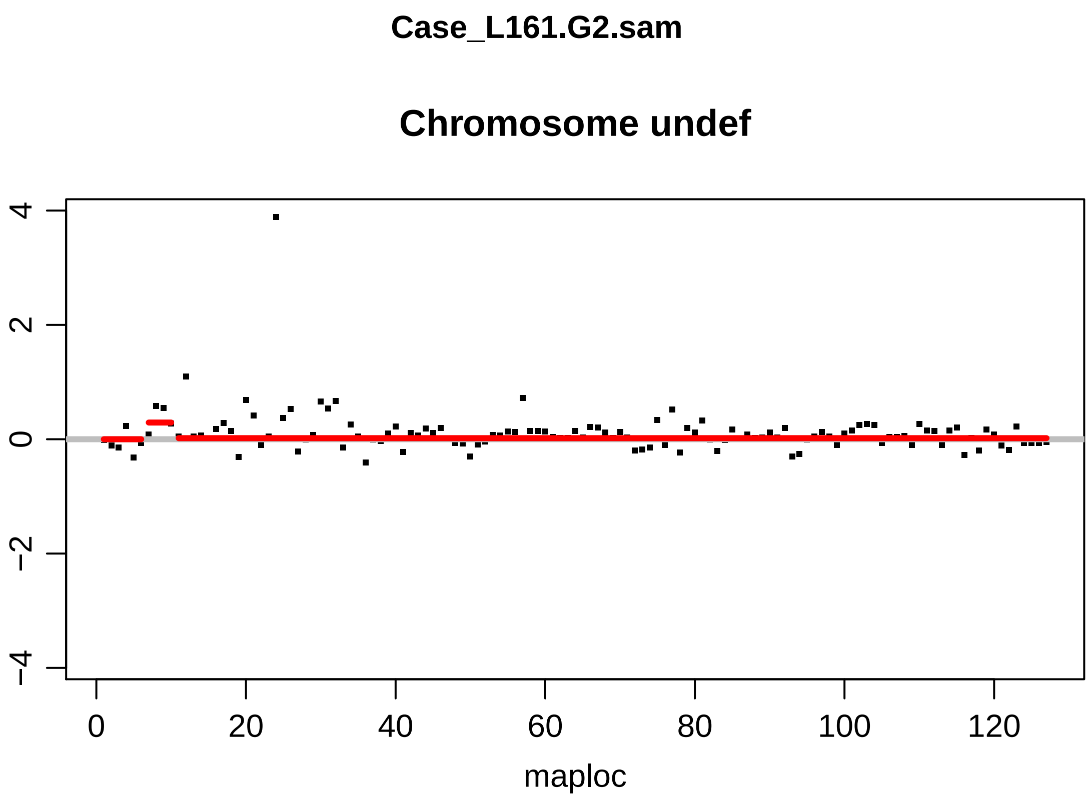

```
## Segplot might not work because of special characters in the sample names. Use only A-Z,a-z and 0-9!
##  There is a hidden function cn.mops:::.replaceNames that replaces the names in the "CNVDetectionResul
```

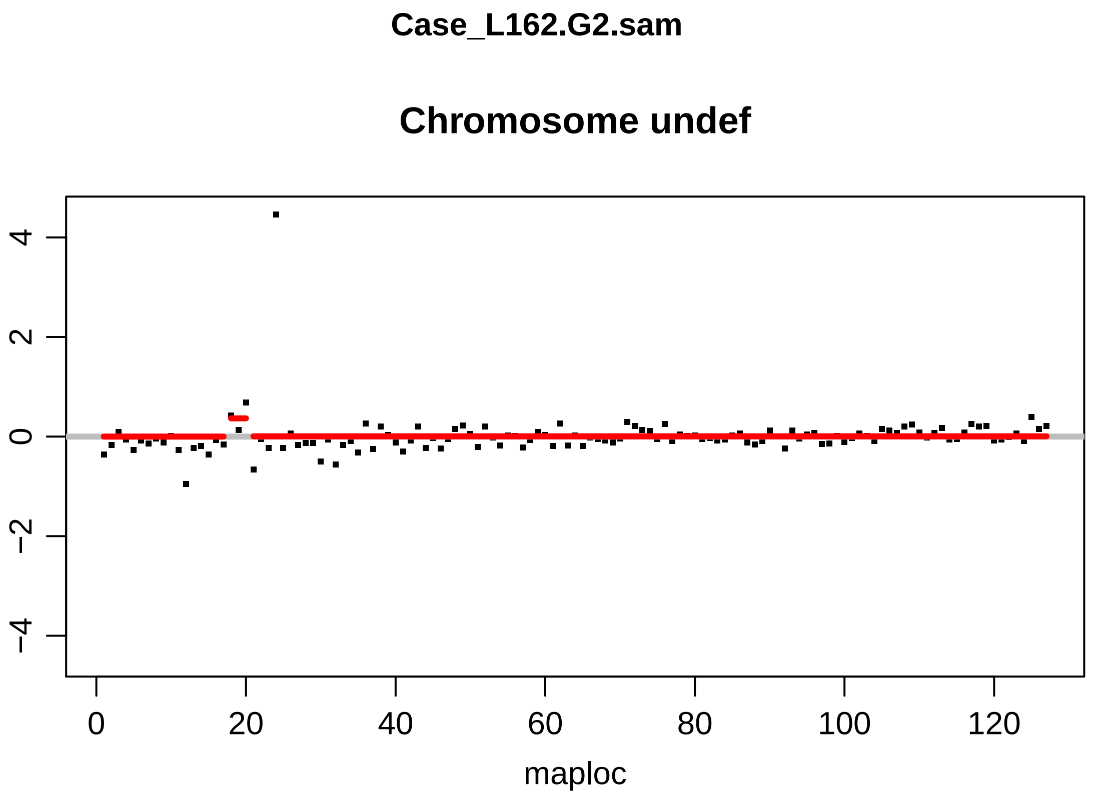


```
## Segplot might not work because of special characters in the sample names. Use only A-Z,a-z and 0-9!
##  There is a hidden function cn.mops:::.replaceNames that replaces the names in the "CNVDetectionResul
```

Case_L163.G2.sam

Chromosome undef

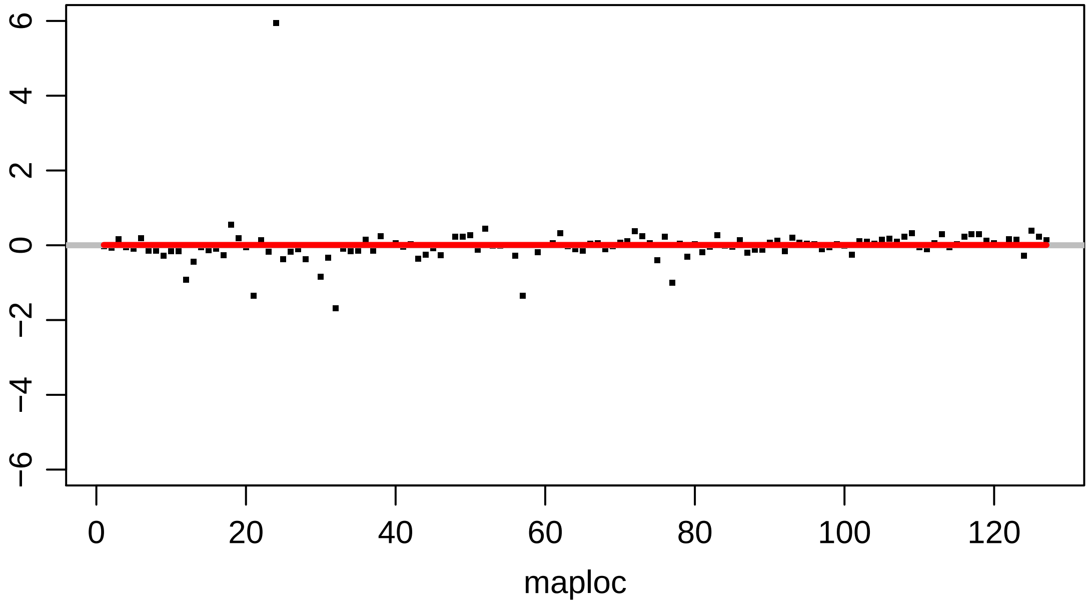


```
## Segplot might not work because of special characters in the sample names. Use only A-Z,a-z and 0-9!
##  There is a hidden function cn.mops:::.replaceNames that replaces the names in the "CNVDetectionResul
```

Case_L164.G2.sam

Chromosome undef

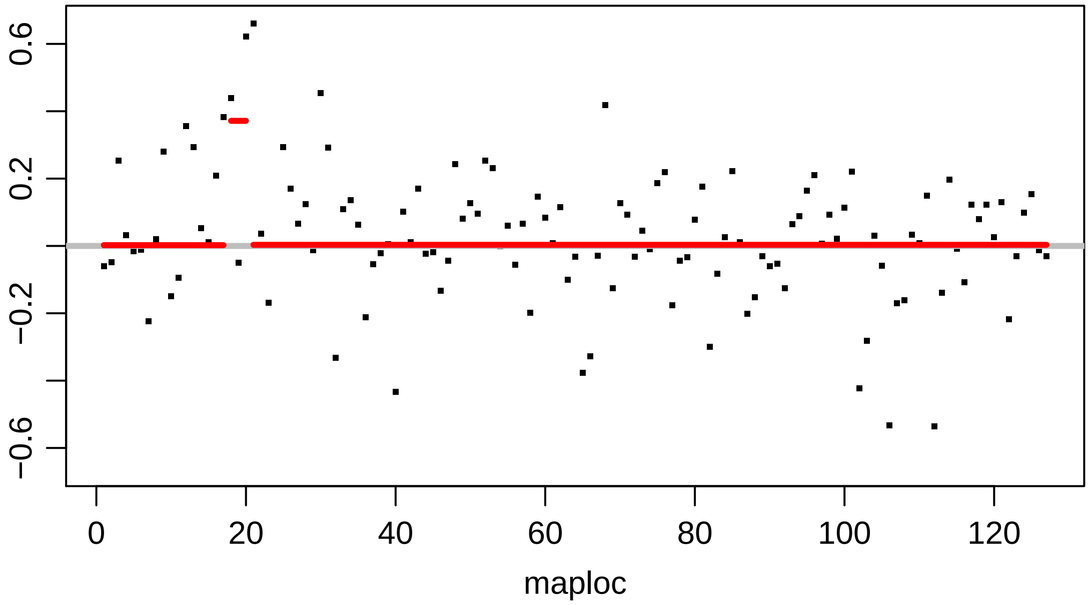

```
## Segplot might not work because of special characters in the sample names. Use only A-Z,a-z and 0-9!
##  There is a hidden function cn.mops:::.replaceNames that replaces the names in the "CNVDetectionResul
```

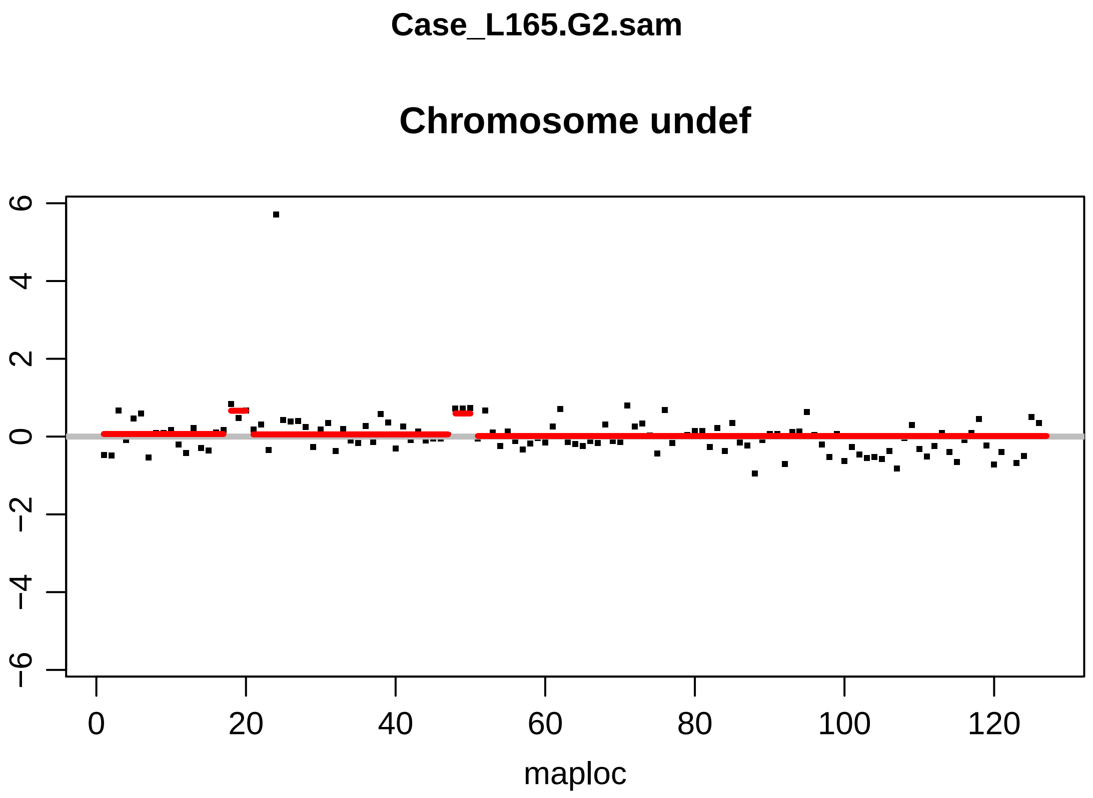


```
## Segplot might not work because of special characters in the sample names. Use only A-Z,a-z and 0-9!
##  There is a hidden function cn.mops:::.replaceNames that replaces the names in the "CNVDetectionResul
```

Case_L166.G2.sam

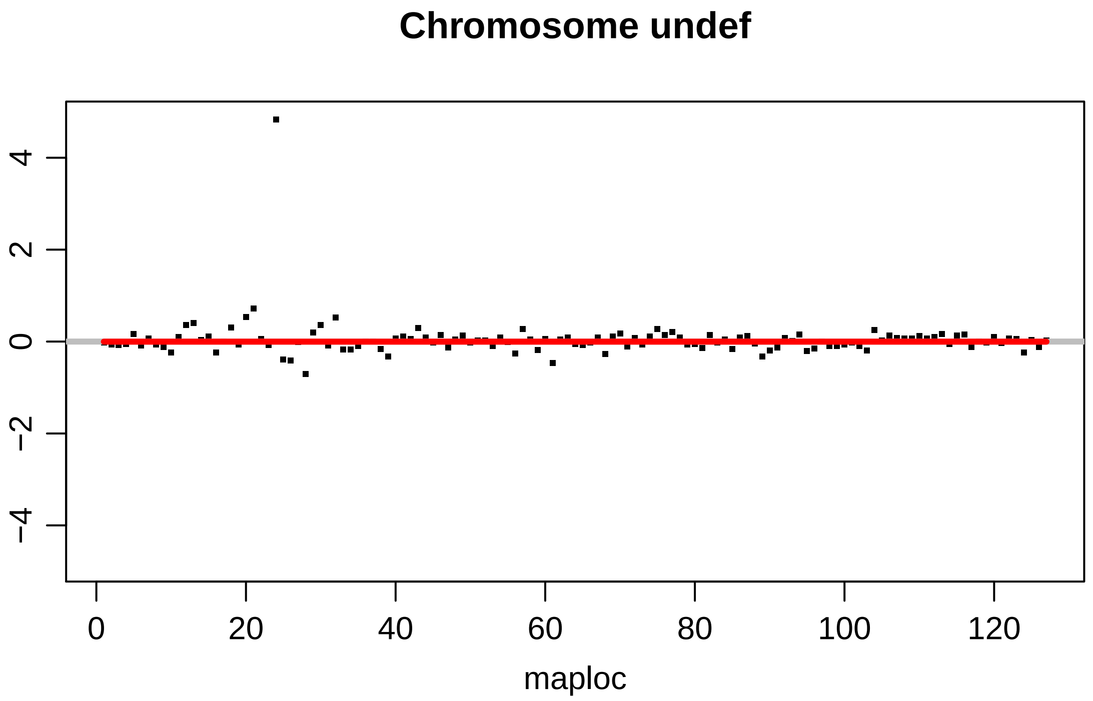


```
## Segplot might not work because of special characters in the sample names. Use only A-Z,a-z and 0-9!
##  There is a hidden function cn.mops:::.replaceNames that replaces the names in the "CNVDetectionResul
```

Case_L167.G2.sam

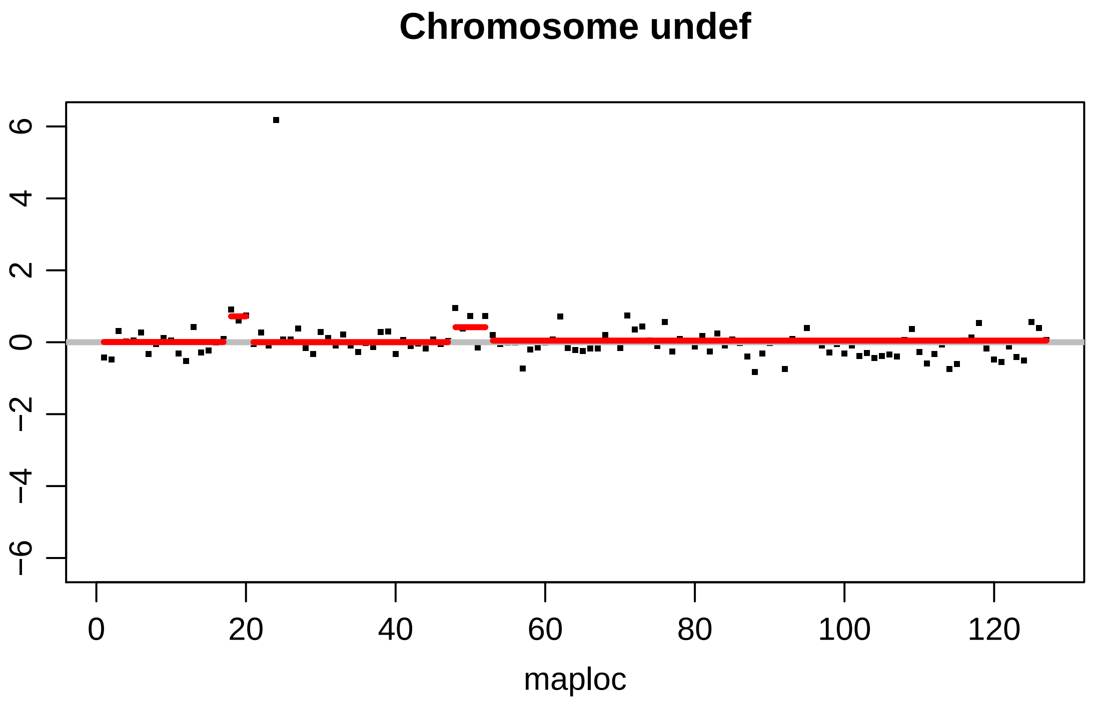

```
## Segplot might not work because of special characters in the sample names. Use only A-Z,a-z and 0-9!
##  There is a hidden function cn.mops:::.replaceNames that replaces the names in the "CNVDetectionResul
```

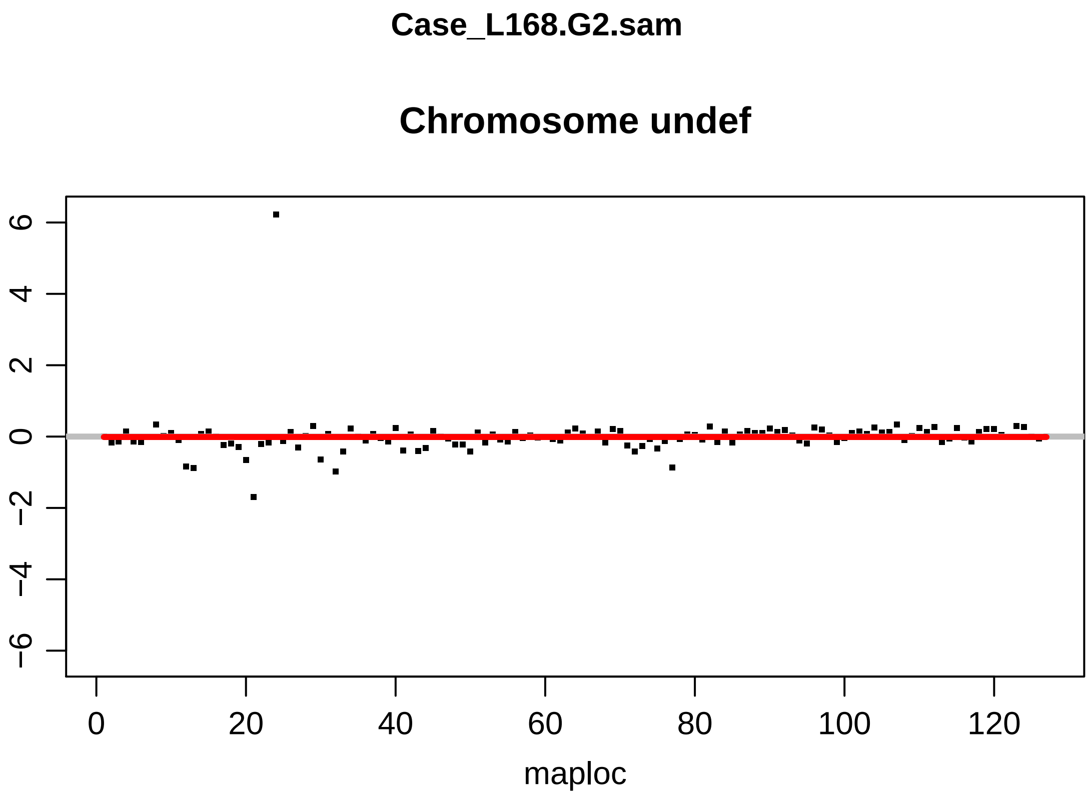


```
## Segplot might not work because of special characters in the sample names. Use only A-Z,a-z and 0-9!
##  There is a hidden function cn.mops:::.replaceNames that replaces the names in the "CNVDetectionResul
```

Case_L169.G2.sam

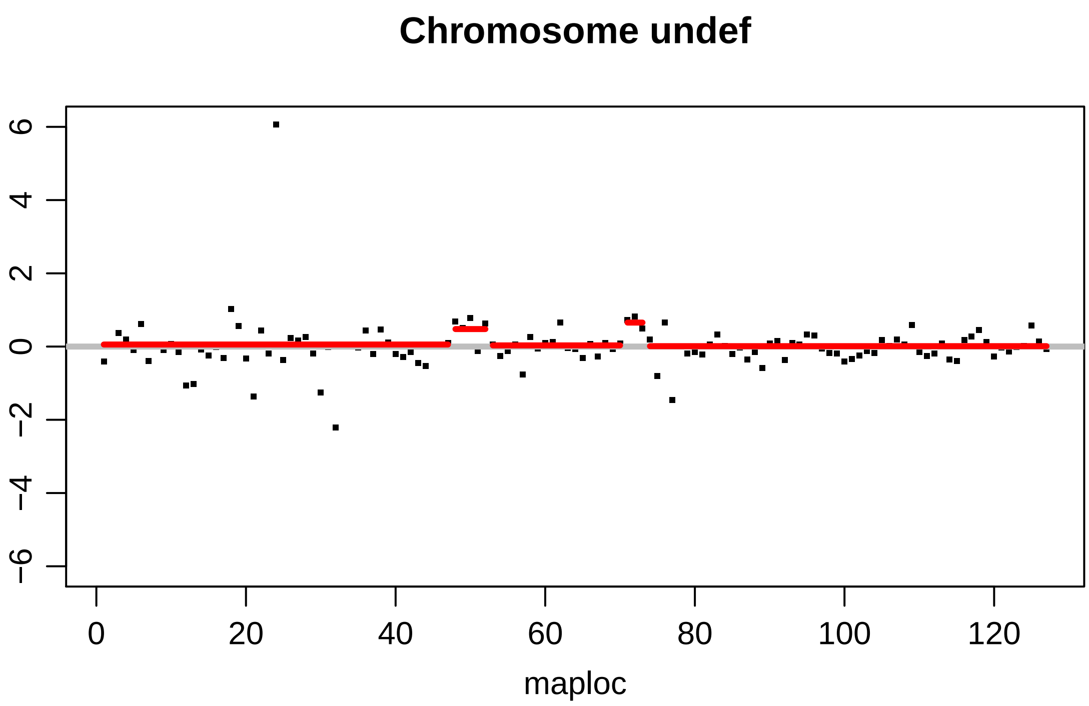


```
## Segplot might not work because of special characters in the sample names. Use only A-Z,a-z and 0-9!
##  There is a hidden function cn.mops:::.replaceNames that replaces the names in the "CNVDetectionResul
```

Case_L170.G2.sam

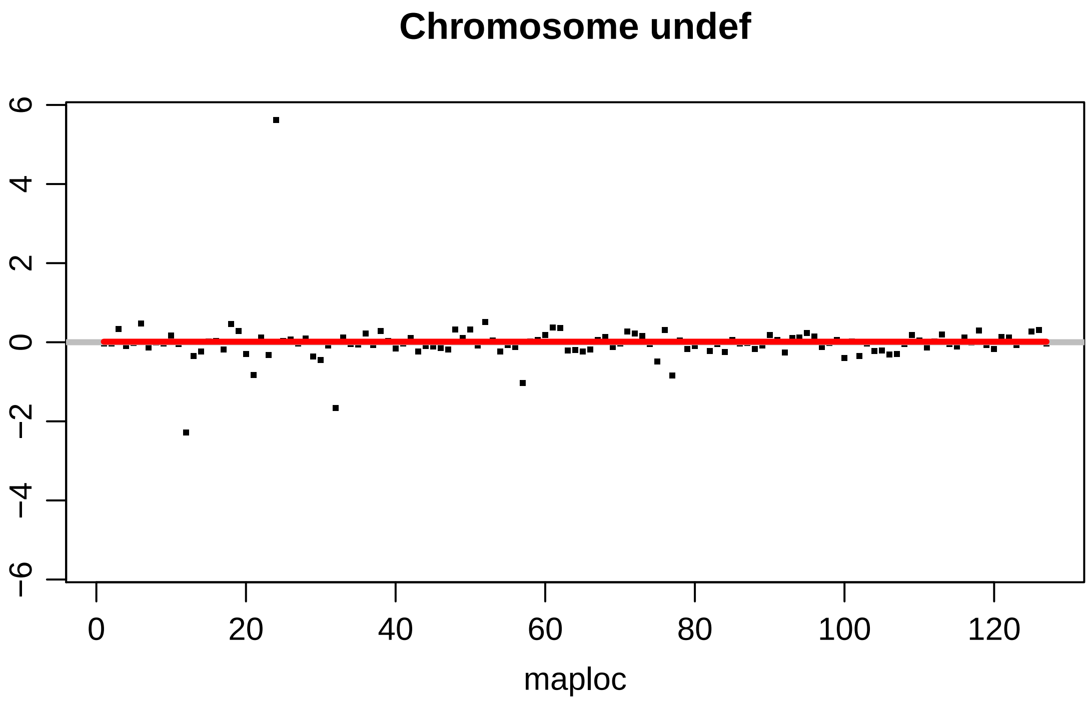

```
## Segplot might not work because of special characters in the sample names. Use only A-Z,a-z and 0-9!
##  There is a hidden function cn.mops:::.replaceNames that replaces the names in the "CNVDetectionResul
```

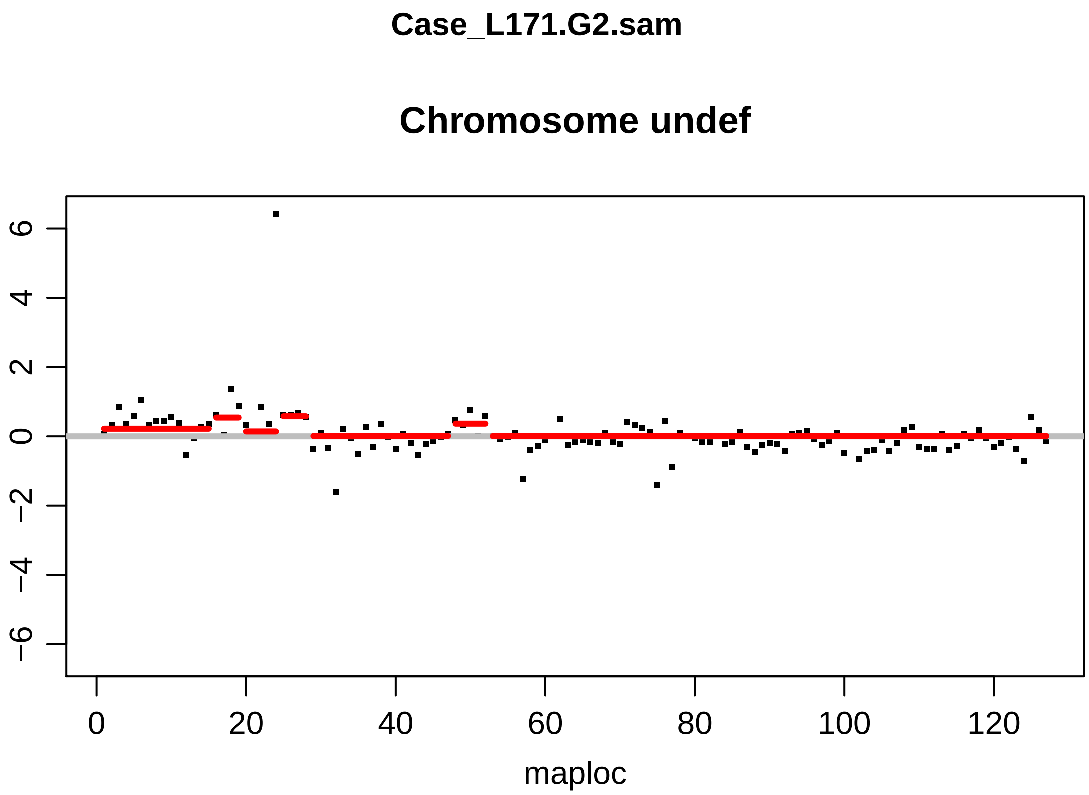


```
## Segplot might not work because of special characters in the sample names. Use only A-Z,a-z and 0-9!
##  There is a hidden function cn.mops:::.replaceNames that replaces the names in the "CNVDetectionResul
```

Case_L172.G2.sam

Chromosome undef

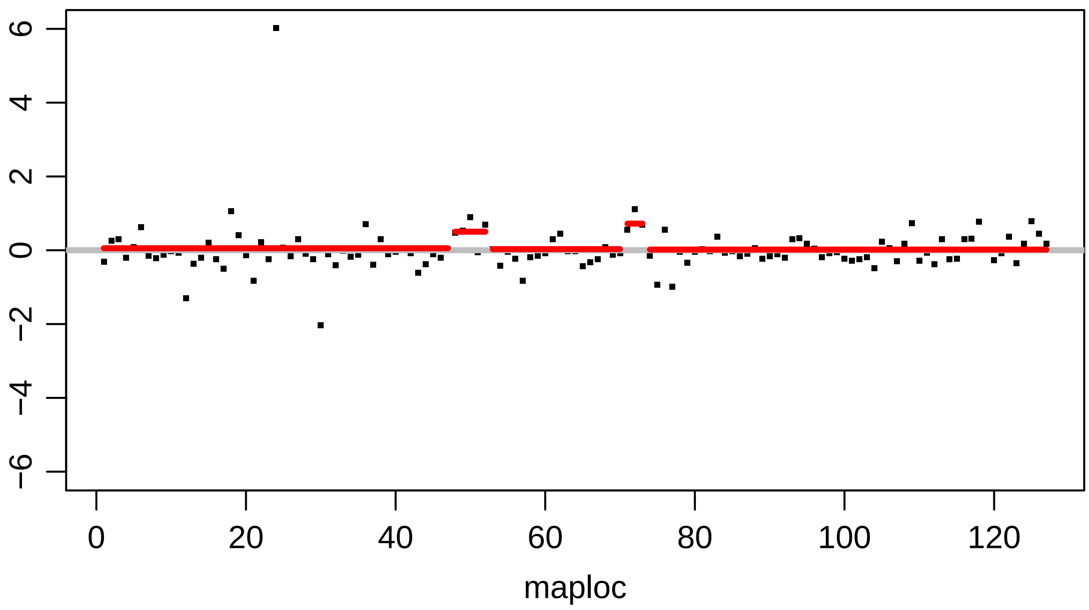


```
## Segplot might not work because of special characters in the sample names. Use only A-Z,a-z and 0-9!
##  There is a hidden function cn.mops:::.replaceNames that replaces the names in the "CNVDetectionResul
```

Case_L173.G2.sam

Chromosome undef

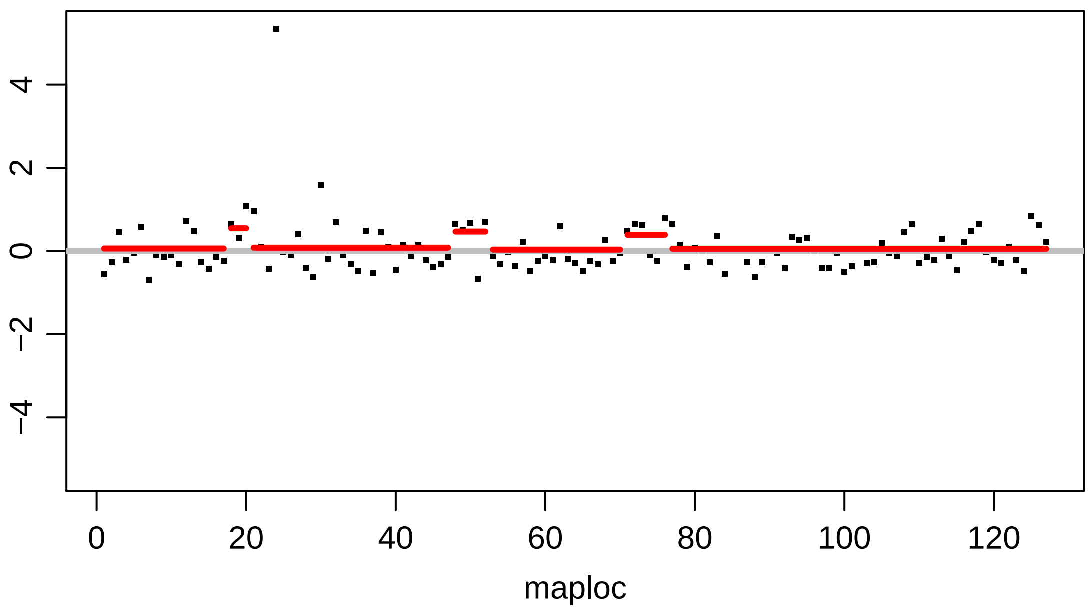

```
## Segplot might not work because of special characters in the sample names. Use only A-Z,a-z and 0-9!
##  There is a hidden function cn.mops:::.replaceNames that replaces the names in the "CNVDetectionResul
```

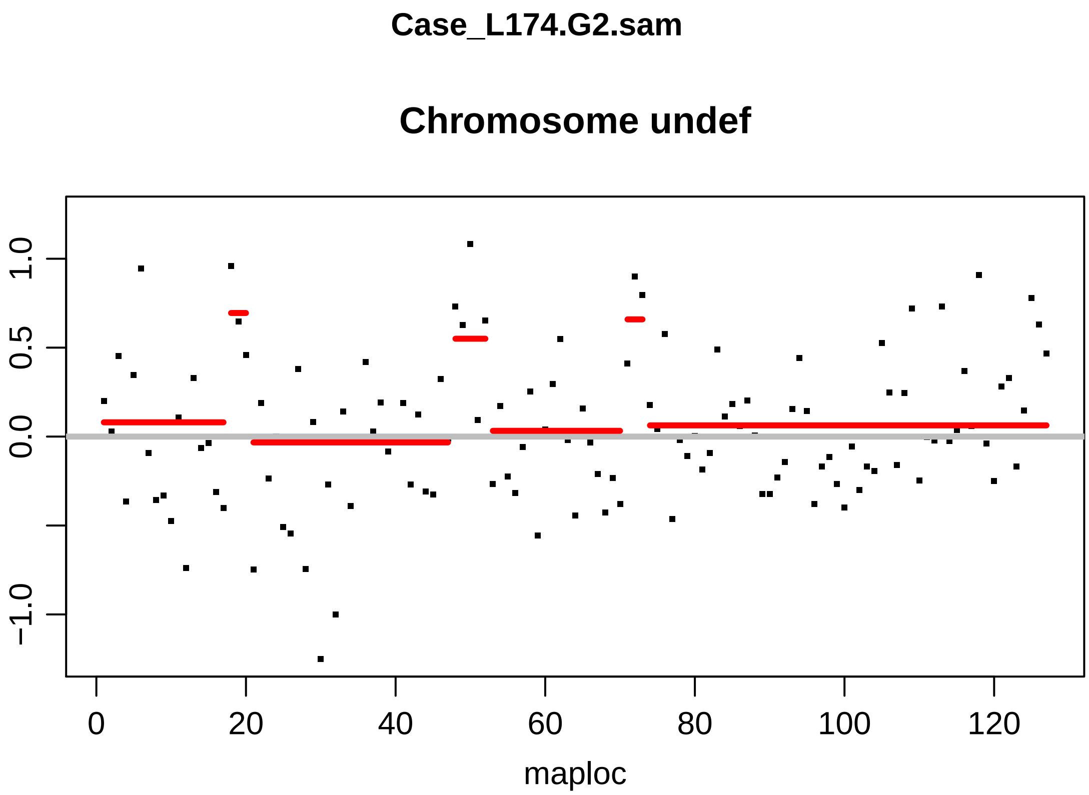


```
## Segplot might not work because of special characters in the sample names. Use only A-Z,a-z and 0-9!
##  There is a hidden function cn.mops:::.replaceNames that replaces the names in the "CNVDetectionResul
```

Case_L175.G2.sam

Chromosome undef

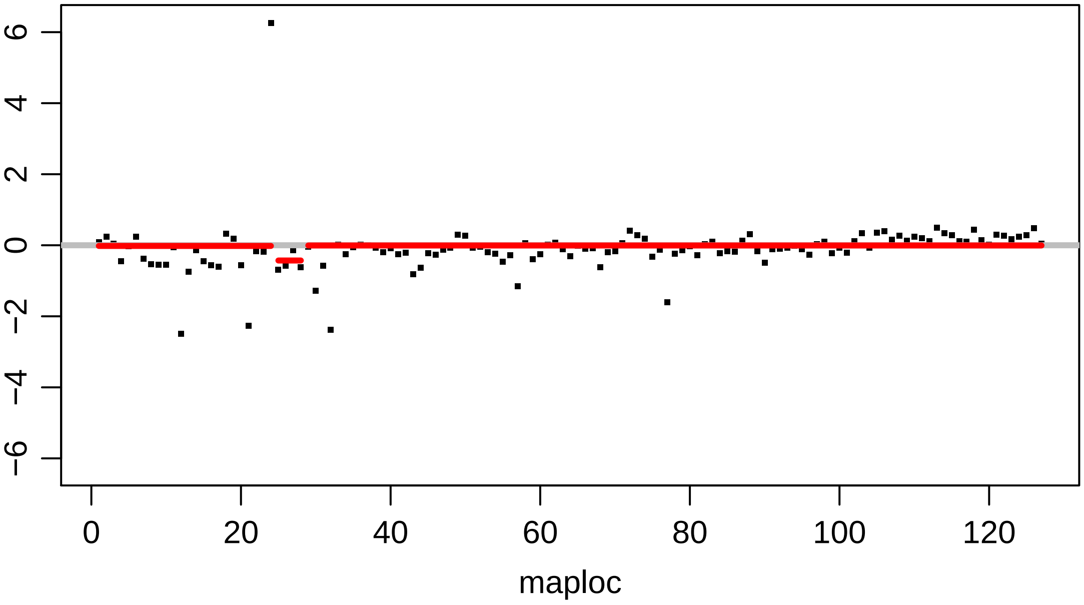


```
## Segplot might not work because of special characters in the sample names. Use only A-Z,a-z and 0-9!
##  There is a hidden function cn.mops:::.replaceNames that replaces the names in the "CNVDetectionResul
```

Case_L176.G2.sam

Chromosome undef

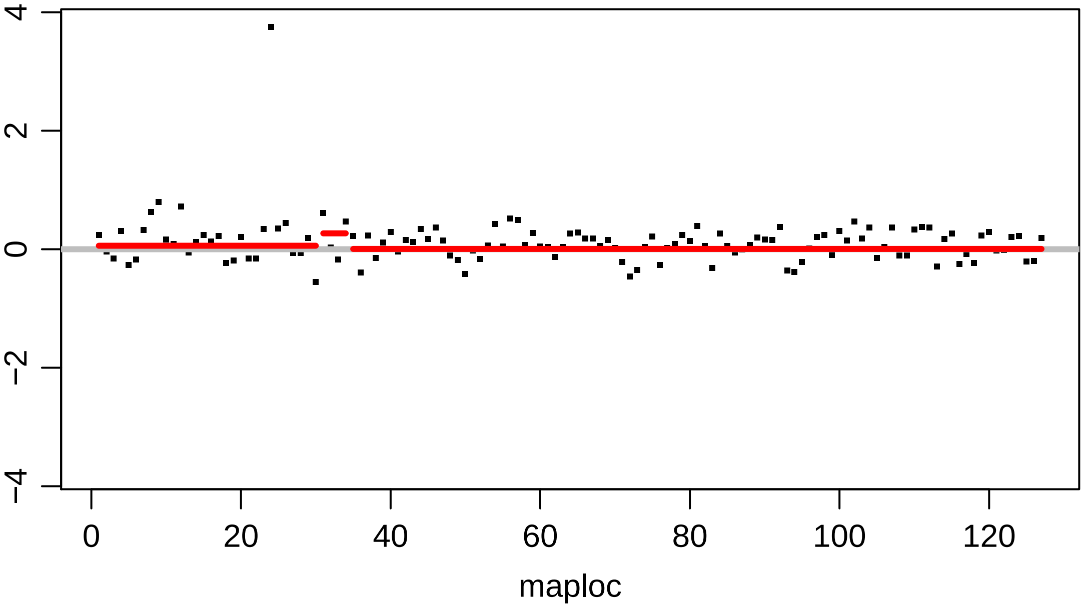

```
## Segplot might not work because of special characters in the sample names. Use only A-Z,a-z and 0-9!
##  There is a hidden function cn.mops:::.replaceNames that replaces the names in the "CNVDetectionResul
```

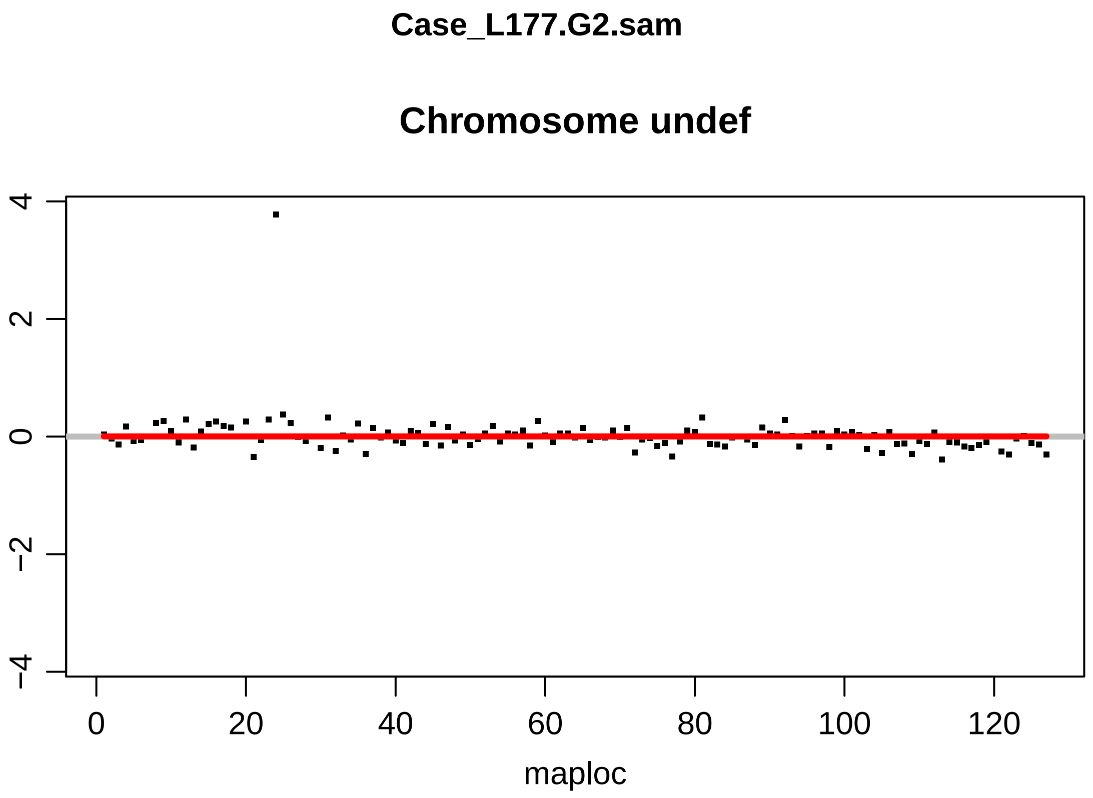


```
## Segplot might not work because of special characters in the sample names. Use only A-Z,a-z and 0-9!
##  There is a hidden function cn.mops:::.replaceNames that replaces the names in the "CNVDetectionResul
```

Case_L178.G2.sam

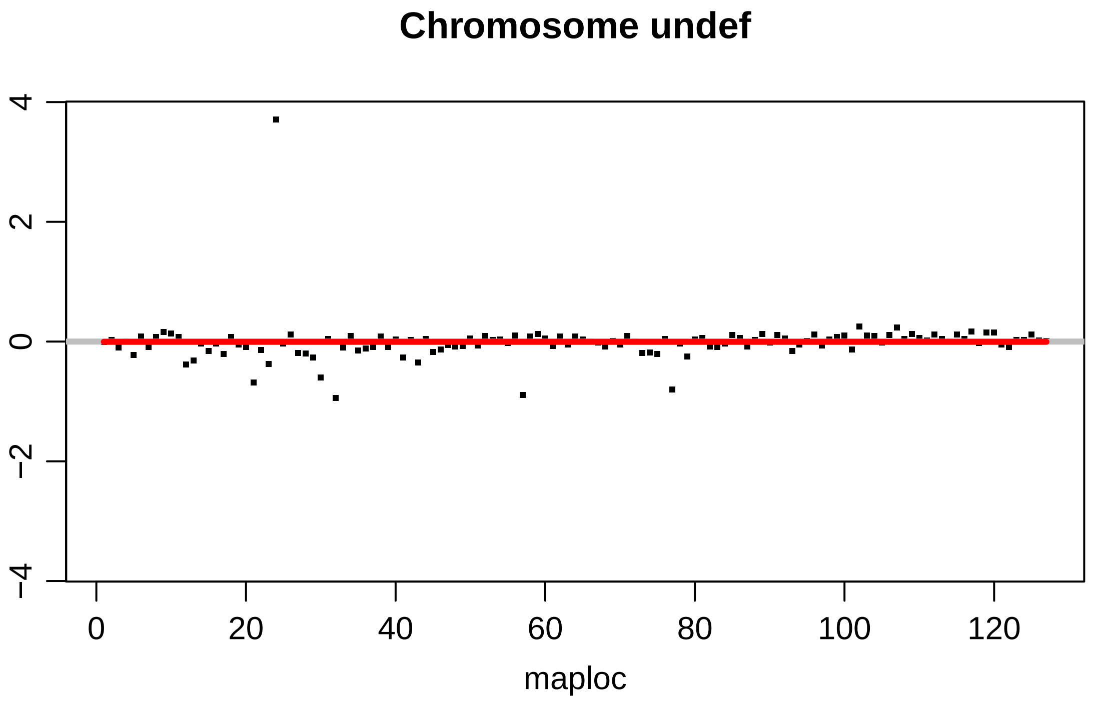


```
## Segplot might not work because of special characters in the sample names. Use only A-Z,a-z and 0-9!
##  There is a hidden function cn.mops:::.replaceNames that replaces the names in the "CNVDetectionResul
```

Case_L179.G2.sam

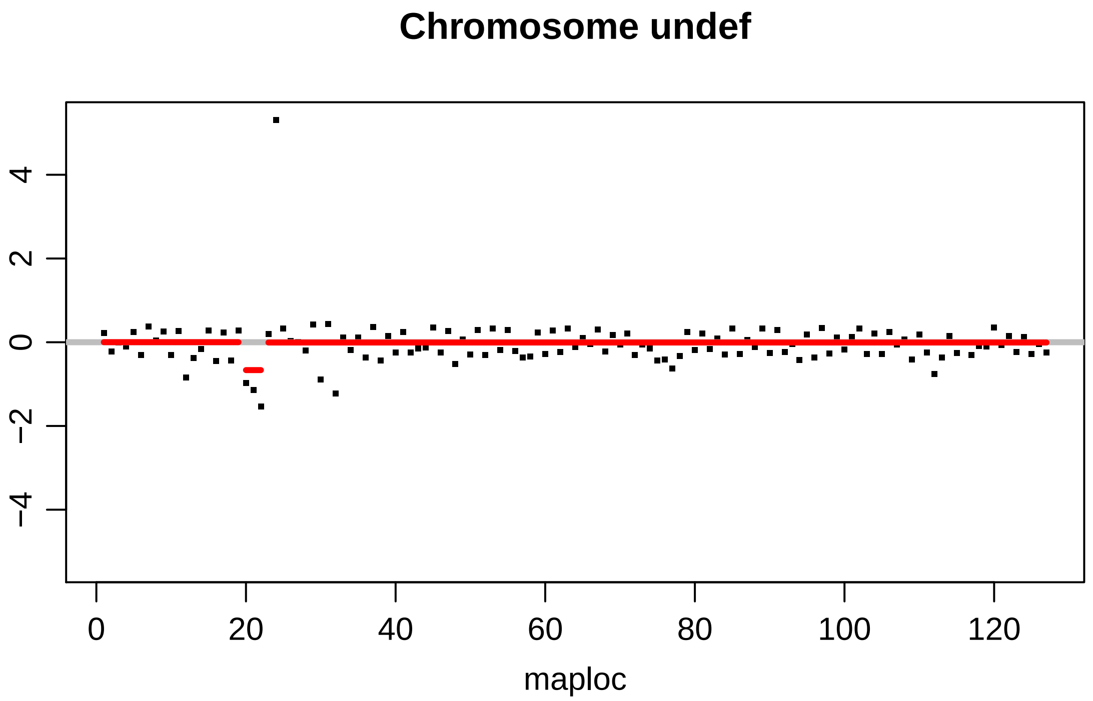

```
## Segplot might not work because of special characters in the sample names. Use only A-Z,a-z and 0-9!
##  There is a hidden function cn.mops:::.replaceNames that replaces the names in the "CNVDetectionResul
```

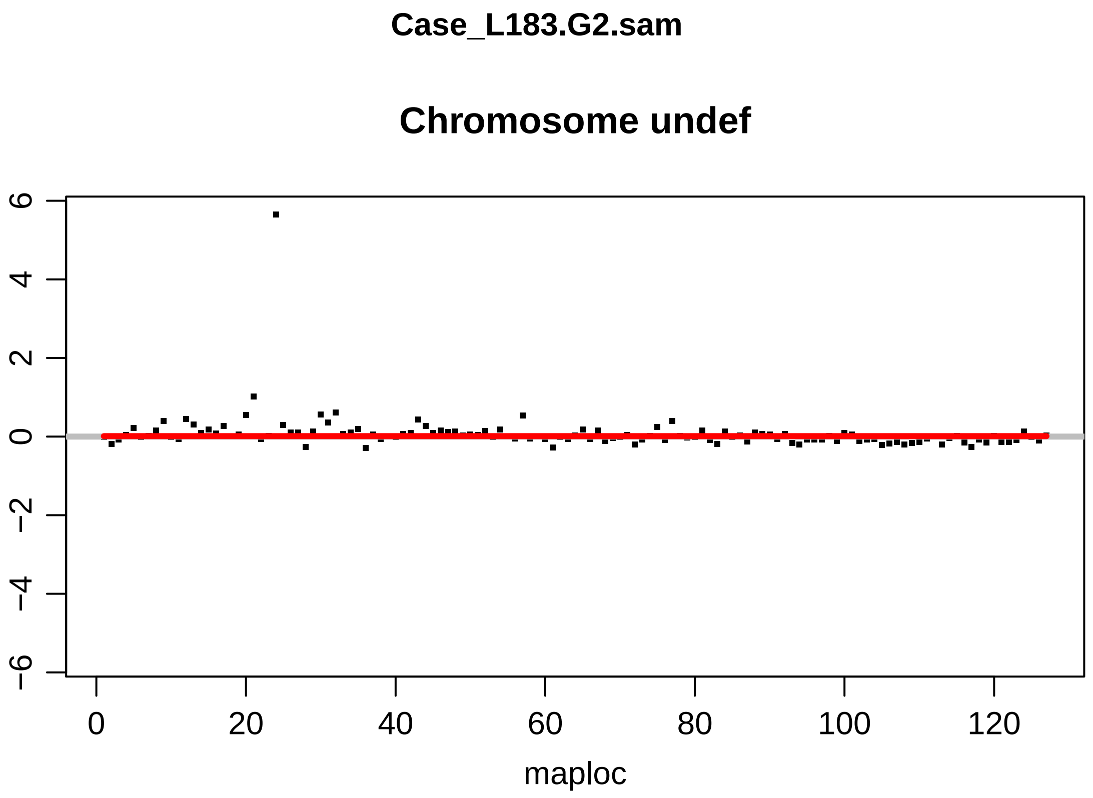


```
## Segplot might not work because of special characters in the sample names. Use only A-Z,a-z and 0-9!
##  There is a hidden function cn.mops:::.replaceNames that replaces the names in the "CNVDetectionResul
```

Case_L184.G2.sam

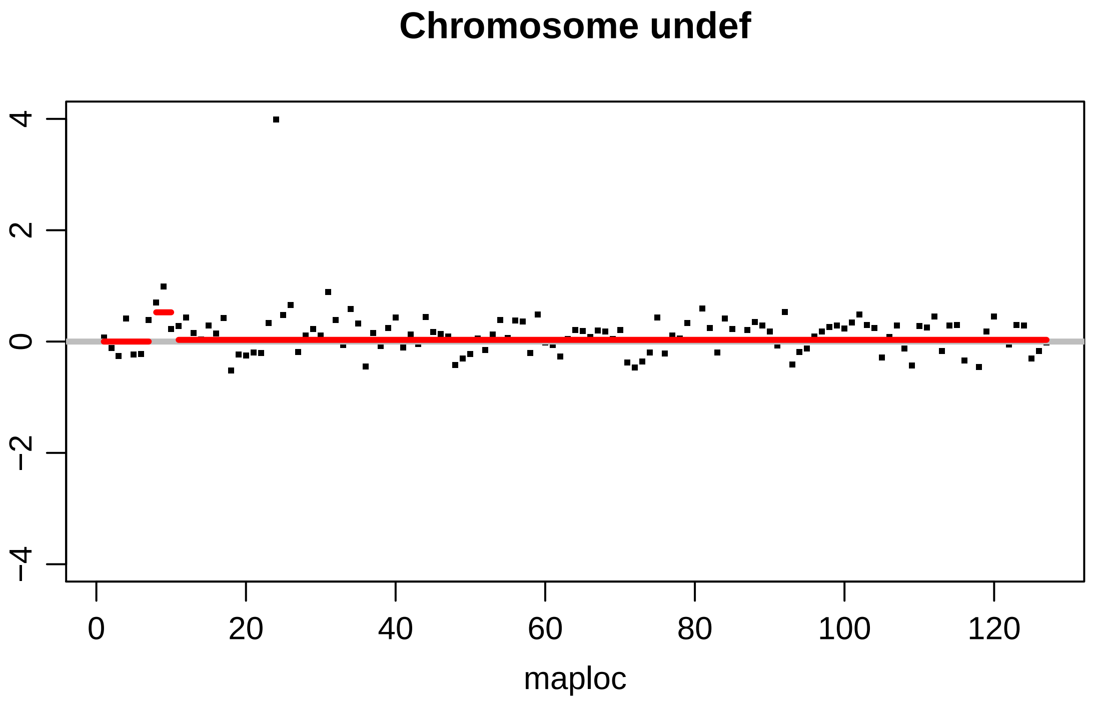


```
## Segplot might not work because of special characters in the sample names. Use only A-Z,a-z and 0-9!
##  There is a hidden function cn.mops:::.replaceNames that replaces the names in the "CNVDetectionResul
```

Case_L185.G2.sam

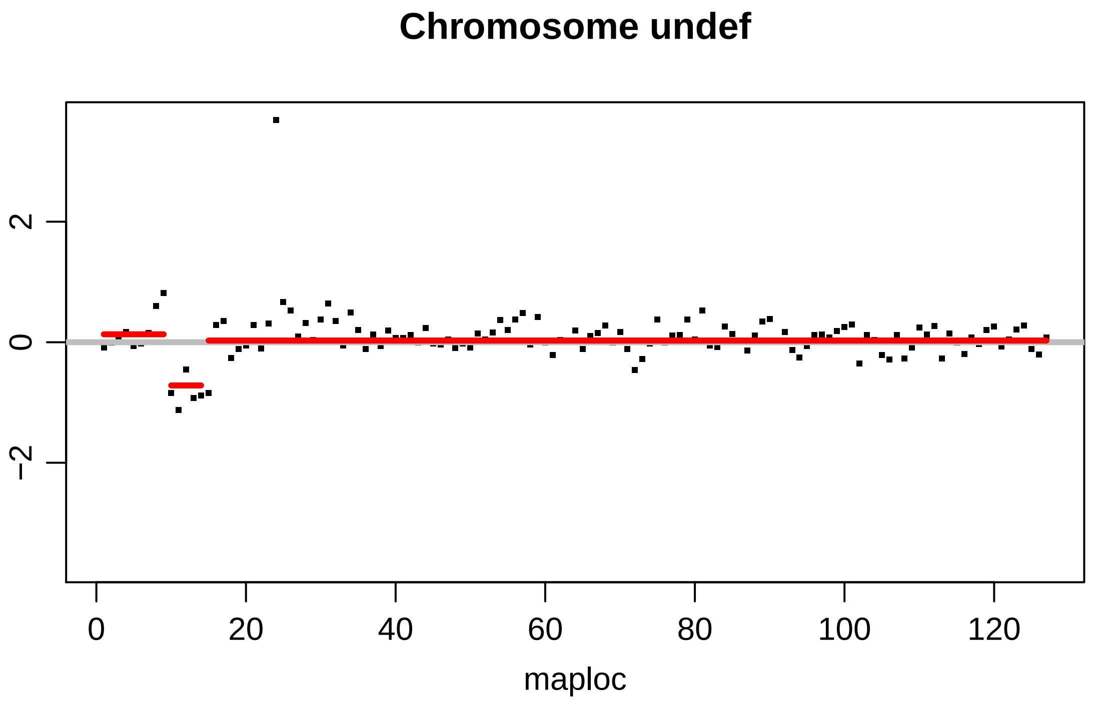

```
## Segplot might not work because of special characters in the sample names. Use only A-Z,a-z and 0-9!
##  There is a hidden function cn.mops:::.replaceNames that replaces the names in the "CNVDetectionResul
```

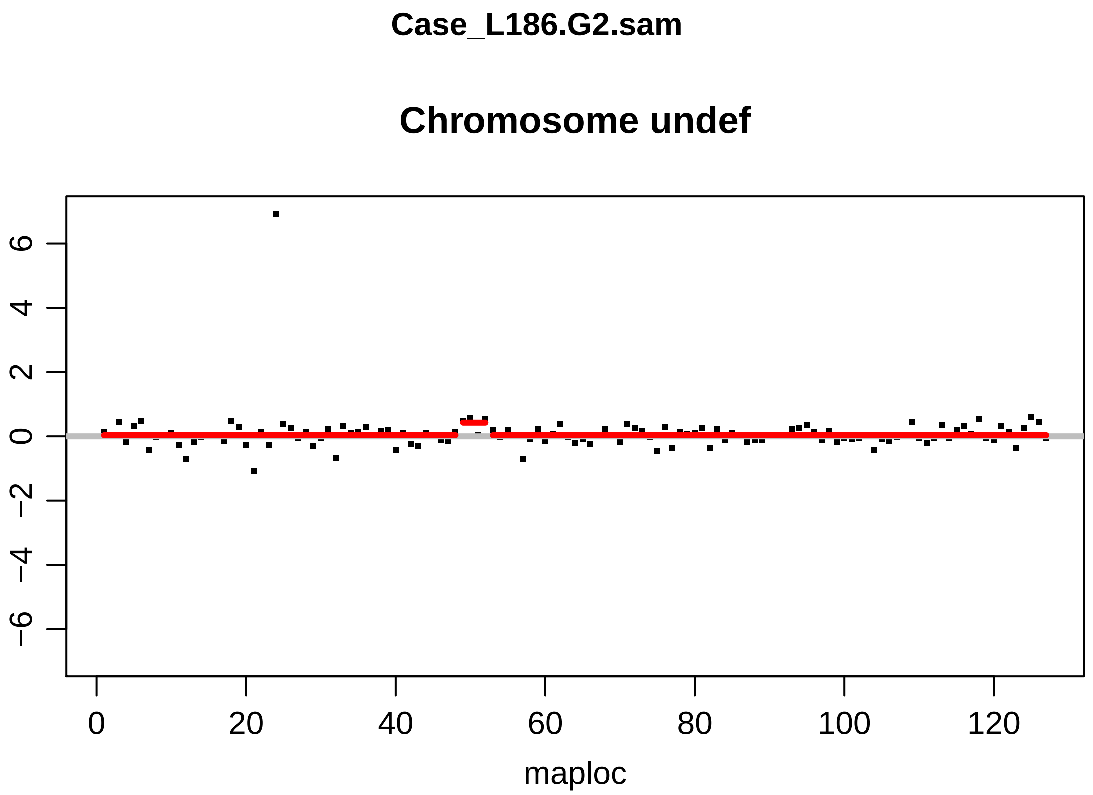


```
## Segplot might not work because of special characters in the sample names. Use only A-Z,a-z and 0-9!
##  There is a hidden function cn.mops:::.replaceNames that replaces the names in the "CNVDetectionResul
```

Case_L187.G2.sam

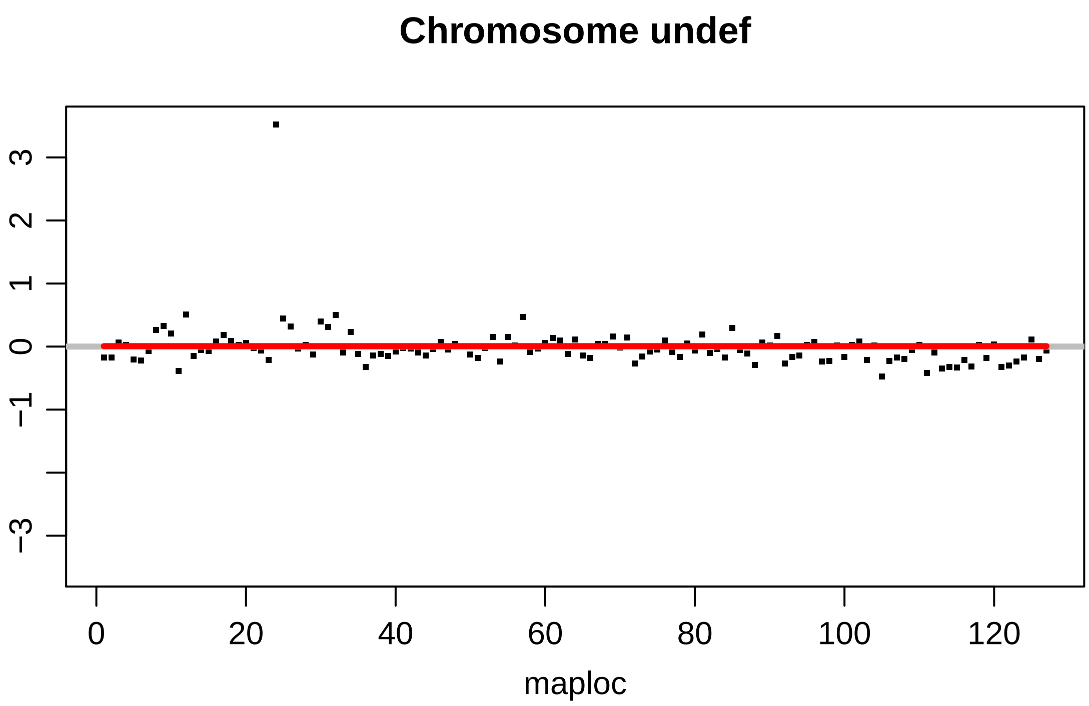


```
## Segplot might not work because of special characters in the sample names. Use only A-Z,a-z and 0-9!
##  There is a hidden function cn.mops:::.replaceNames that replaces the names in the "CNVDetectionResul
```

Case_L188.G2.sam

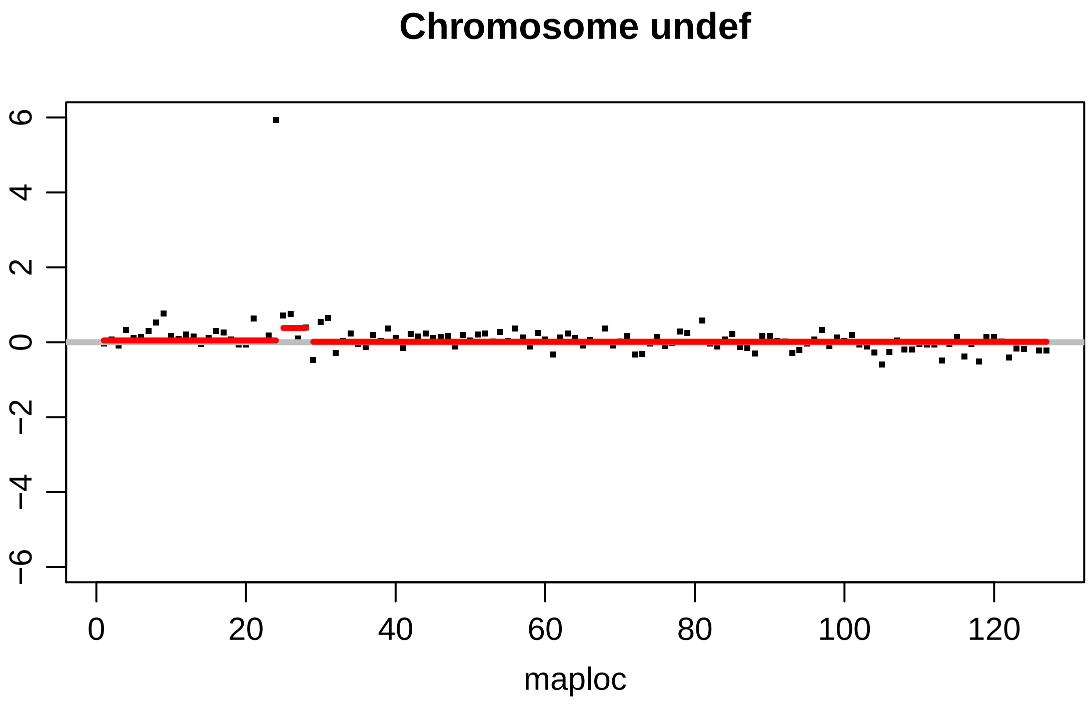

```
##
## CNV regions:
## GRanges object with 5 ranges and 42 metadata columns:
##       seqnames    ranges strand | Case_L142.G1.sam Case_L143.G1.sam
##          <Rle> <IRanges>  <Rle> |         <factor>         <factor>
##   [1]    undef  [ 8, 10]      * |              CN2              CN2
##   [2]    undef  [16, 20]      * |              CN2              CN2
##   [3]    undef  [25, 28]      * |              CN2              CN2
##   [4]    undef  [48, 52]      * |              CN2              CN2
##   [5]    undef  [71, 73]      * |              CN2              CN2
##       Case_L144.G1.sam Case_L145.G1.sam Case_L146.G1.sam Case_L147.G1.sam
##               <factor>         <factor>         <factor>         <factor>
##   [1]              CN2              CN2              CN2              CN2
##   [2]              CN2              CN2              CN2              CN2
##   [3]              CN2              CN2              CN2              CN2
##   [4]              CN2              CN2              CN2              CN2
##   [5]              CN2              CN2              CN2              CN2
##       Case_L149.G1.sam Case_L150.G1.sam Case_L151.G1.sam Case_L152.G1.sam
##               <factor>         <factor>         <factor>         <factor>
##   [1]              CN2              CN2              CN2              CN2
##   [2]              CN2              CN2              CN2              CN2
##   [3]              CN2              CN2              CN2              CN2
##   [4]              CN2              CN2              CN2              CN2
##   [5]              CN2              CN2              CN2              CN2
##       Case_L154.G1.sam Case_L155.G2.sam Case_L157.G2.sam Case_L158.G2.sam
##               <factor>         <factor>         <factor>         <factor>
##   [1]              CN2              CN2              CN2              CN2
##   [2]              CN2              CN2              CN2              CN2
##   [3]              CN2              CN2              CN2              CN2
##   [4]              CN2              CN2              CN2              CN2
##   [5]              CN2              CN2              CN2              CN2
##       Case_L159.G2.sam Case_L160.G2.sam Case_L161.G2.sam Case_L162.G2.sam
##               <factor>         <factor>         <factor>         <factor>
##   [1]              CN2              CN2              CN2              CN2
##   [2]              CN2              CN3              CN2              CN2
##   [3]              CN2              CN2              CN2              CN2
##   [4]              CN2              CN2              CN2              CN2
##   [5]              CN2              CN2              CN2              CN2
##       Case_L163.G2.sam Case_L164.G2.sam Case_L165.G2.sam Case_L166.G2.sam
##               <factor>         <factor>         <factor>         <factor>
##   [1]              CN2              CN2              CN2              CN2
##   [2]              CN2              CN2              CN3              CN2
##   [3]              CN2              CN2              CN2              CN2
##   [4]              CN2              CN2              CN3              CN2
##   [5]              CN2              CN2              CN2              CN2
##       Case_L167.G2.sam Case_L168.G2.sam Case_L169.G2.sam Case_L170.G2.sam
##               <factor>         <factor>         <factor>         <factor>
##   [1]              CN2              CN2              CN2              CN2
##   [2]              CN3              CN2              CN2              CN2
##   [3]              CN2              CN2              CN2              CN2
##   [4]              CN2              CN2              CN2              CN2
##   [5]              CN2              CN2              CN4              CN2
##       Case_L171.G2.sam Case_L172.G2.sam Case_L173.G2.sam Case_L174.G2.sam
##               <factor>         <factor>         <factor>         <factor>
```

```
##   [1]              CN2              CN2              CN2              CN2
##   [2]              CN3              CN2              CN3              CN3
##   [3]              CN3              CN2              CN2              CN2
##   [4]              CN2              CN3              CN2              CN3
##   [5]              CN2              CN4              CN2              CN4
##       Case_L175.G2.sam Case_L176.G2.sam Case_L177.G2.sam Case_L178.G2.sam
##               <factor>         <factor>         <factor>         <factor>
##   [1]              CN2              CN2              CN2              CN2
##   [2]              CN2              CN2              CN2              CN2
##   [3]              CN2              CN2              CN2              CN2
##   [4]              CN2              CN2              CN2              CN2
##   [5]              CN2              CN2              CN2              CN2
##       Case_L179.G2.sam Case_L183.G2.sam Case_L184.G2.sam Case_L185.G2.sam
##               <factor>         <factor>         <factor>         <factor>
##   [1]              CN2              CN2              CN4              CN2
##   [2]              CN2              CN2              CN2              CN2
##   [3]              CN2              CN2              CN2              CN2
##   [4]              CN2              CN2              CN2              CN2
##   [5]              CN2              CN2              CN2              CN2
##       Case_L186.G2.sam Case_L187.G2.sam Case_L188.G2.sam Case_control.sam
##               <factor>         <factor>         <factor>         <factor>
##   [1]              CN2              CN2              CN2              CN2
##   [2]              CN2              CN2              CN2              CN2
##   [3]              CN2              CN2              CN2              CN2
##   [4]              CN2              CN2              CN2              CN2
##   [5]              CN2              CN2              CN2              CN2
##   -------
##   seqinfo: 1 sequence from an unspecified genome; no seqlengths
##
## Individual CNVs:
## GRanges object with 14 ranges and 4 metadata columns:
##        seqnames    ranges strand   |       sampleName    median      mean
##           <Rle> <IRanges>  <Rle>   |         <factor> <numeric> <numeric>
##    [1]    undef  [18, 20]      *   | Case_L160.G2.sam 0.6823821 0.5629859
##    [2]    undef  [18, 20]      *   | Case_L165.G2.sam 0.5889807 0.6642116
##    [3]    undef  [48, 50]      *   | Case_L165.G2.sam 0.5849537 0.5940481
##    [4]    undef  [18, 20]      *   | Case_L167.G2.sam 0.6042094 0.7192269
##    [5]    undef  [71, 73]      *   | Case_L169.G2.sam 0.5849608 0.6544953
##    ...      ...       ...    ... ...              ...       ...       ...
##   [10]    undef  [18, 20]      *   | Case_L173.G2.sam 0.5883354 0.5454976
##   [11]    undef  [18, 20]      *   | Case_L174.G2.sam 0.5847664 0.6948650
##   [12]    undef  [48, 52]      *   | Case_L174.G2.sam 0.5847189 0.5502694
##   [13]    undef  [71, 73]      *   | Case_L174.G2.sam 0.5849506 0.6590507
##   [14]    undef  [ 8, 10]      *   | Case_L184.G2.sam 0.5848331 0.5255962
##                 CN
##        <character>
##    [1]         CN3
##    [2]         CN3
##    [3]         CN3
##    [4]         CN3
##    [5]         CN4
##    ...         ...
##   [10]         CN3
##   [11]         CN3
```

```
##   [12]         CN3
##   [13]         CN4
##   [14]         CN4
##   -------
##   seqinfo: 1 sequence from an unspecified genome; no seqlengths
## [1] "/Users/gdemidov/Downloads/doc/Run_SN2_2_fin_05_qc.xls"

## Normalizing...

## Starting local modeling, please be patient...

## Reference sequence:  undef

## Starting segmentation algorithm...

## Using "fastseg" for segmentation.

## [1] ""
## [1] "/Users/gdemidov/Downloads/doc/Run_SN2_2_fin_05_qc.xls"
## [1] ""

## Segplot might not work because of special characters in the sample names. Use only A-Z,a-z and 0-9!
##  There is a hidden function cn.mops:::.replaceNames that replaces the names in the "CNVDetectionResul
```

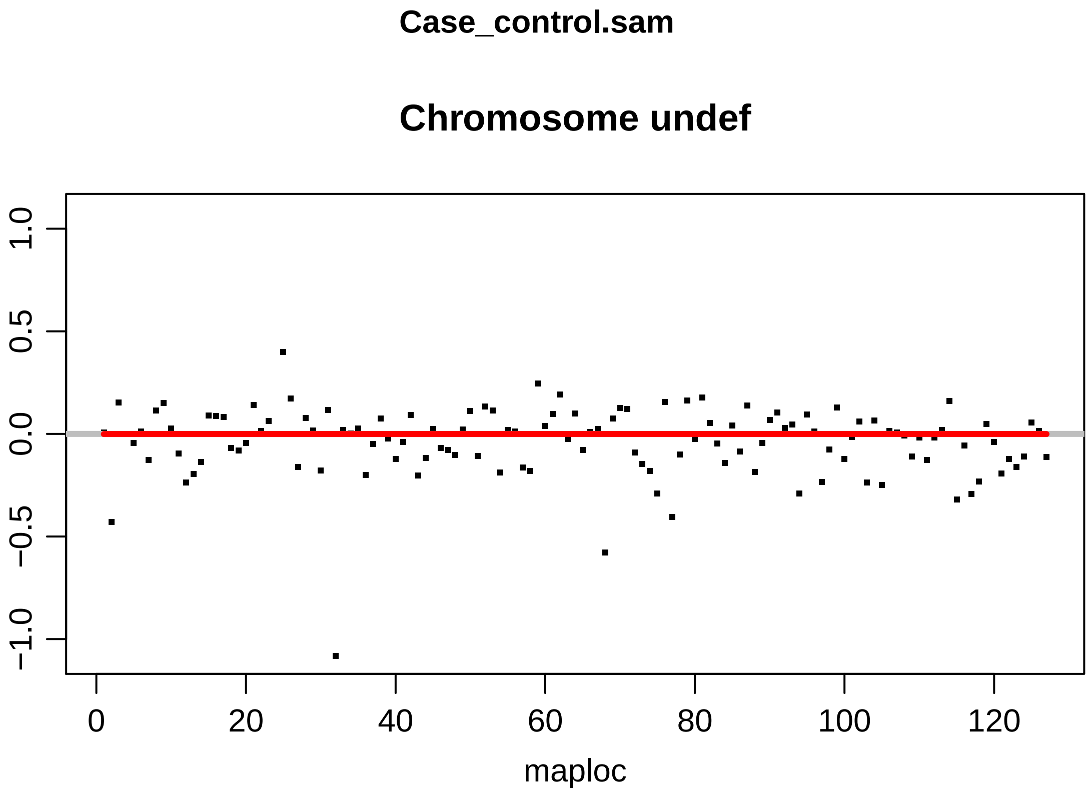


```
## Segplot might not work because of special characters in the sample names. Use only A-Z,a-z and 0-9!
##  There is a hidden function cn.mops:::.replaceNames that replaces the names in the "CNVDetectionResul
```

_10_21_46_user_SN2.2.Neonatal.Assay.LEx.2run.18.11.13_Auto_user_SN2.2.Neona

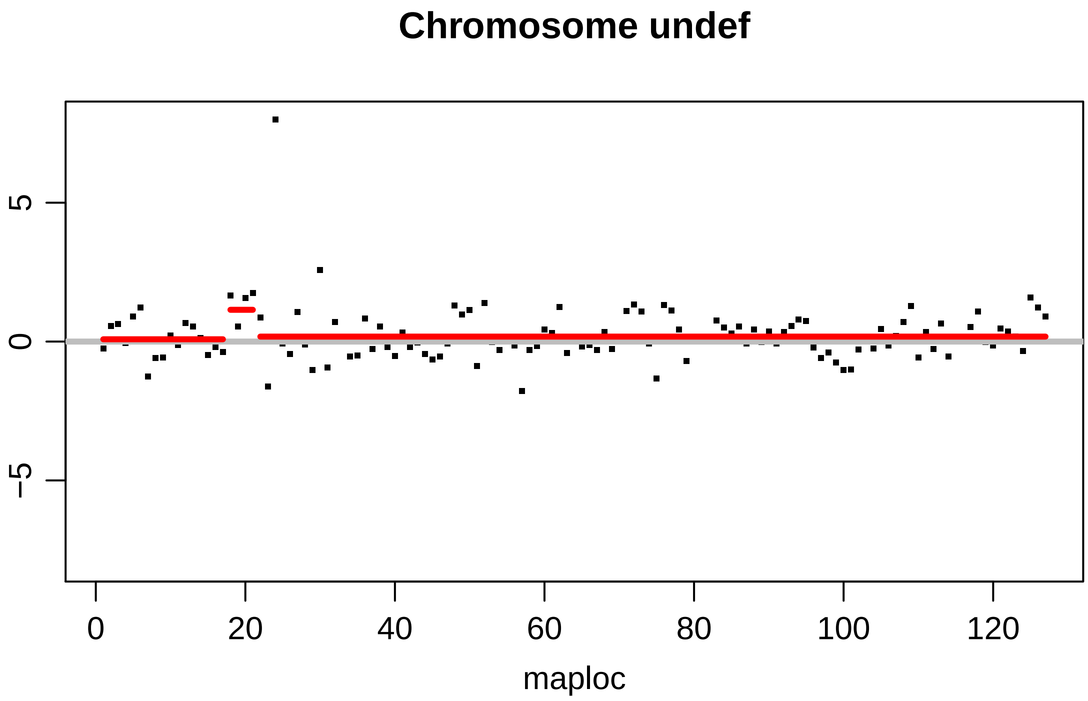


```
## Segplot might not work because of special characters in the sample names. Use only A-Z,a-z and 0-9!
##  There is a hidden function cn.mops:::.replaceNames that replaces the names in the "CNVDetectionResul
```

_10_21_46_user_SN2.2.Neonatal.Assay.LEx.2run.18.11.13_Auto_user_SN2.2.Neona

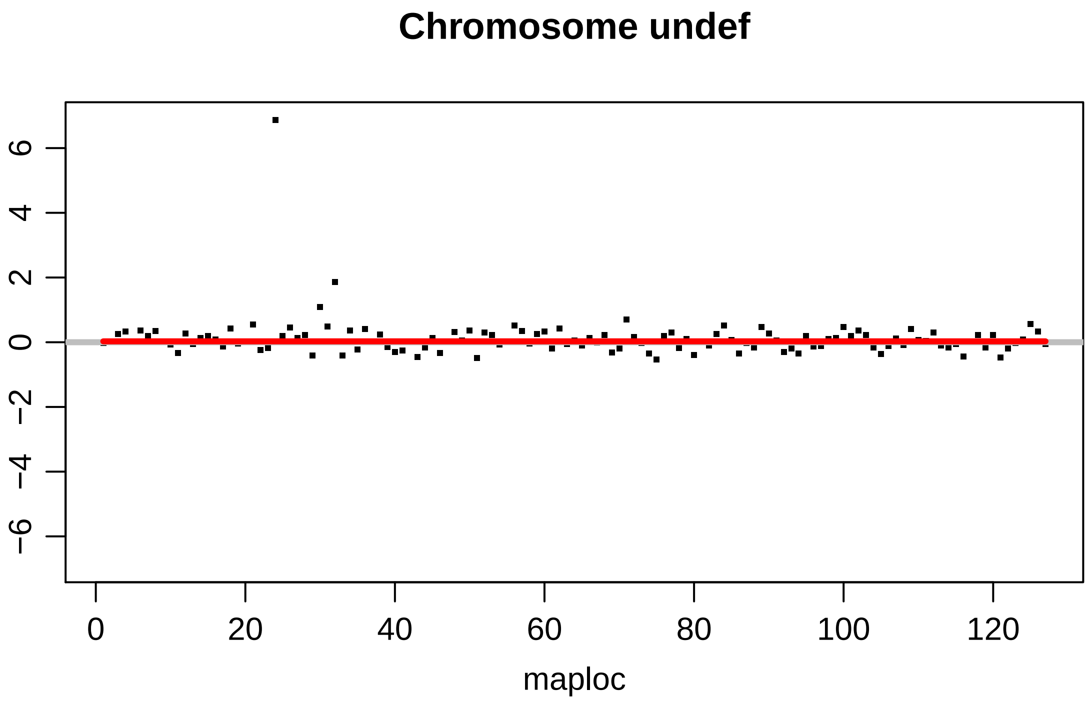

```
## Segplot might not work because of special characters in the sample names. Use only A-Z,a-z and 0-9!
##  There is a hidden function cn.mops:::.replaceNames that replaces the names in the "CNVDetectionResul
```

_10_21_46_user_SN2.2.Neonatal.Assay.LEx.2run.18.11.13_Auto_user_SN2.2.Neona

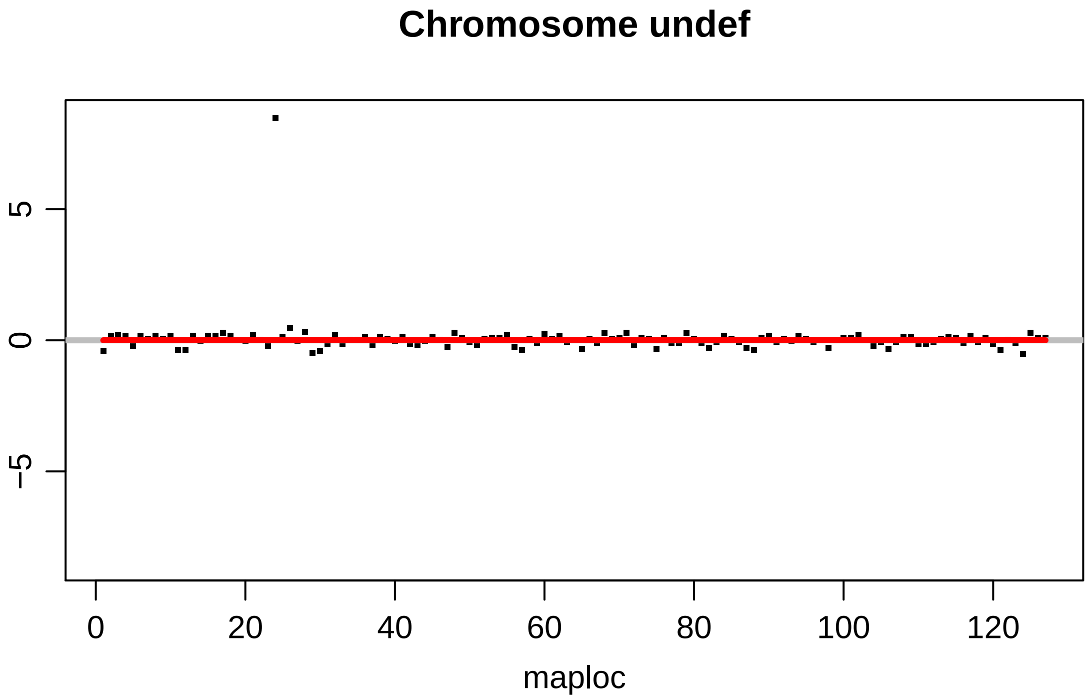


```
## Segplot might not work because of special characters in the sample names. Use only A-Z,a-z and 0-9!
##  There is a hidden function cn.mops:::.replaceNames that replaces the names in the "CNVDetectionResul
```

_10_21_46_user_SN2.2.Neonatal.Assay.LEx.2run.18.11.13_Auto_user_SN2.2.Neon

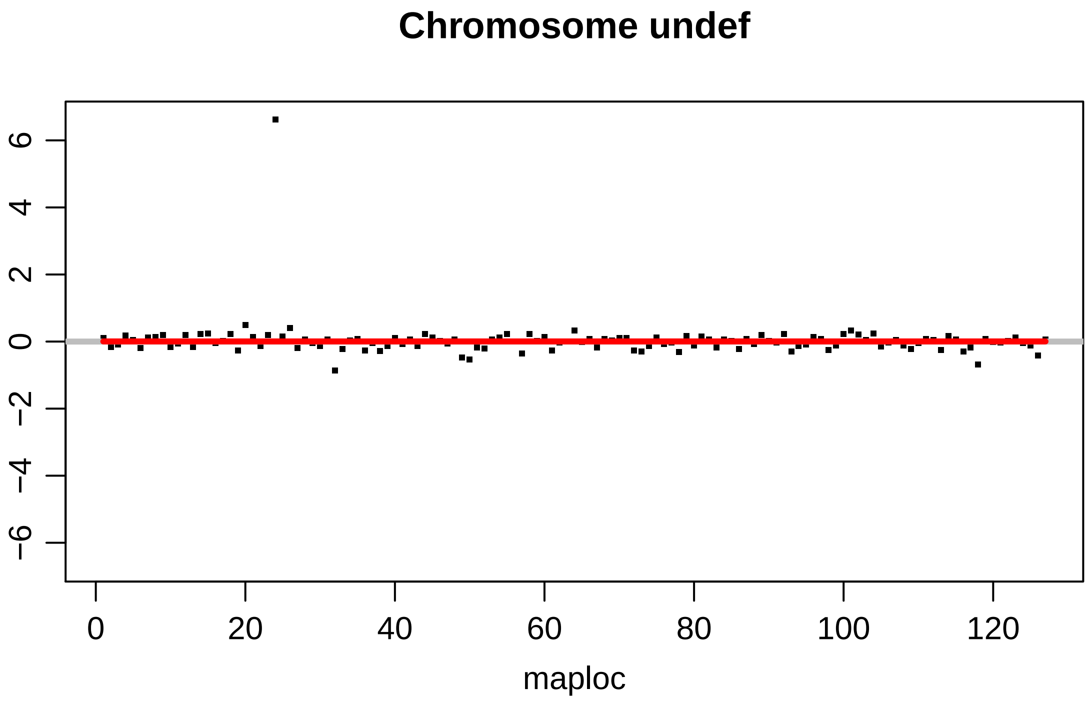


```
## Segplot might not work because of special characters in the sample names. Use only A-Z,a-z and 0-9!
##  There is a hidden function cn.mops:::.replaceNames that replaces the names in the "CNVDetectionResu
```

_10_21_46_user_SN2.2.Neonatal.Assay.LEx.2run.18.11.13_Auto_user_SN2.2.Neon

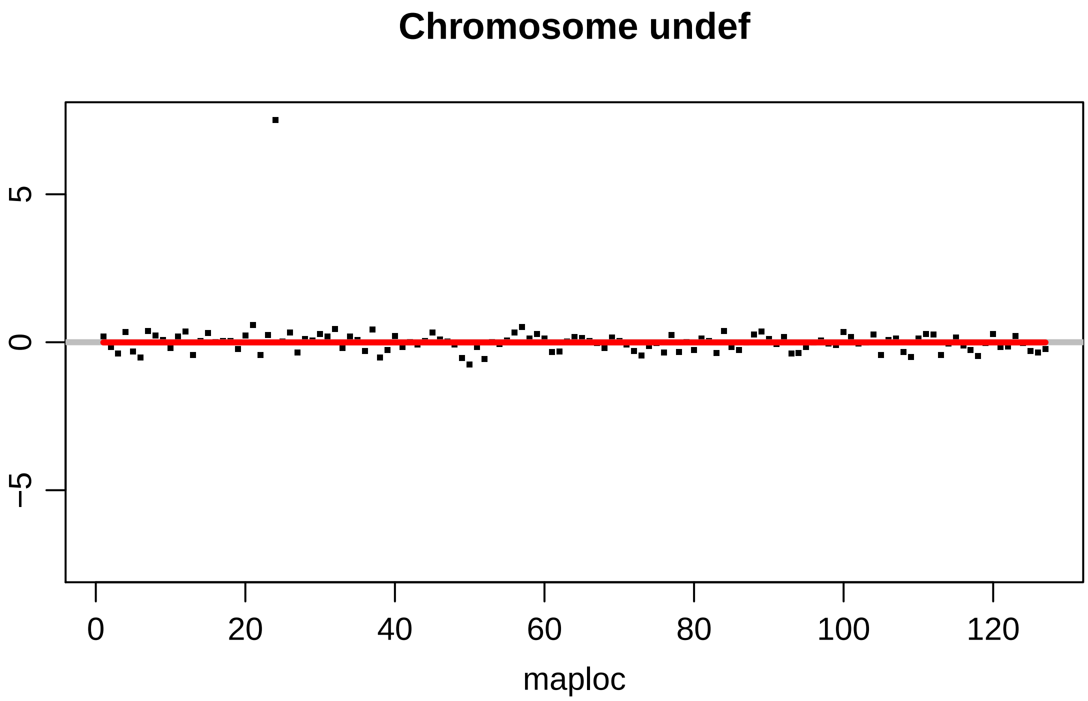

```
## Segplot might not work because of special characters in the sample names. Use only A-Z,a-z and 0-9!
##  There is a hidden function cn.mops:::.replaceNames that replaces the names in the "CNVDetectionResul
```

_10_21_46_user_SN2.2.Neonatal.Assay.LEx.2run.18.11.13_Auto_user_SN2.2.Neona

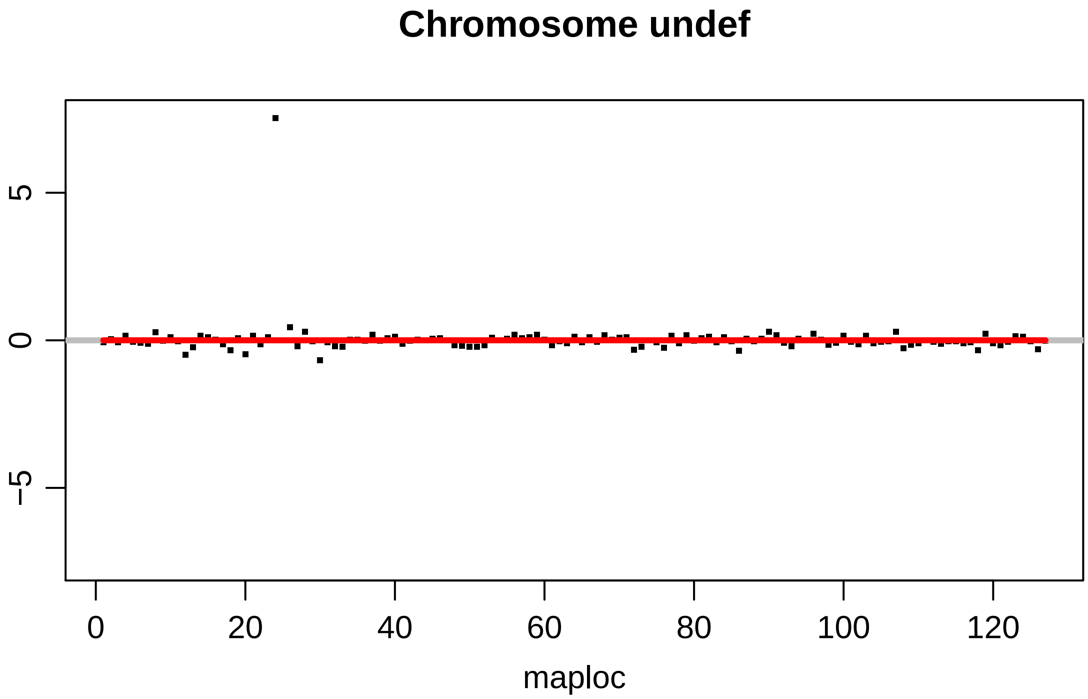


```
## Segplot might not work because of special characters in the sample names. Use only A-Z,a-z and 0-9!
##  There is a hidden function cn.mops:::.replaceNames that replaces the names in the "CNVDetectionResul
```

_10_21_46_user_SN2.2.Neonatal.Assay.LEx.2run.18.11.13_Auto_user_SN2.2.Neon

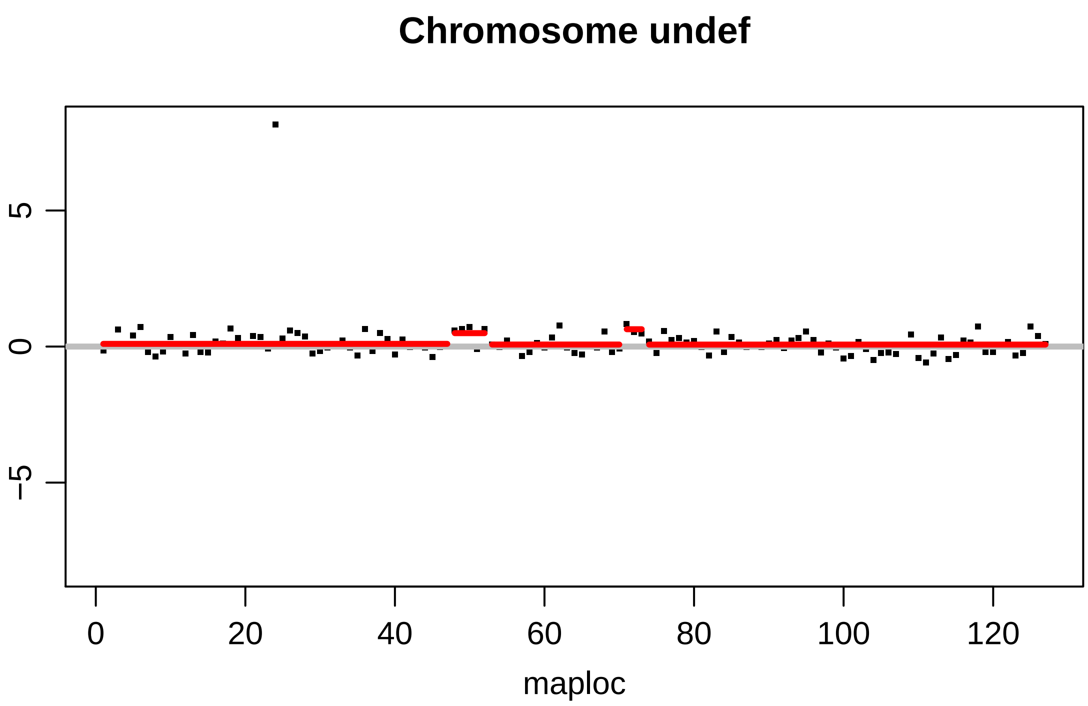


```
## Segplot might not work because of special characters in the sample names. Use only A-Z,a-z and 0-9!
##  There is a hidden function cn.mops:::.replaceNames that replaces the names in the "CNVDetectionResul
```

_10_21_46_user_SN2.2.Neonatal.Assay.LEx.2run.18.11.13_Auto_user_SN2.2.Neon

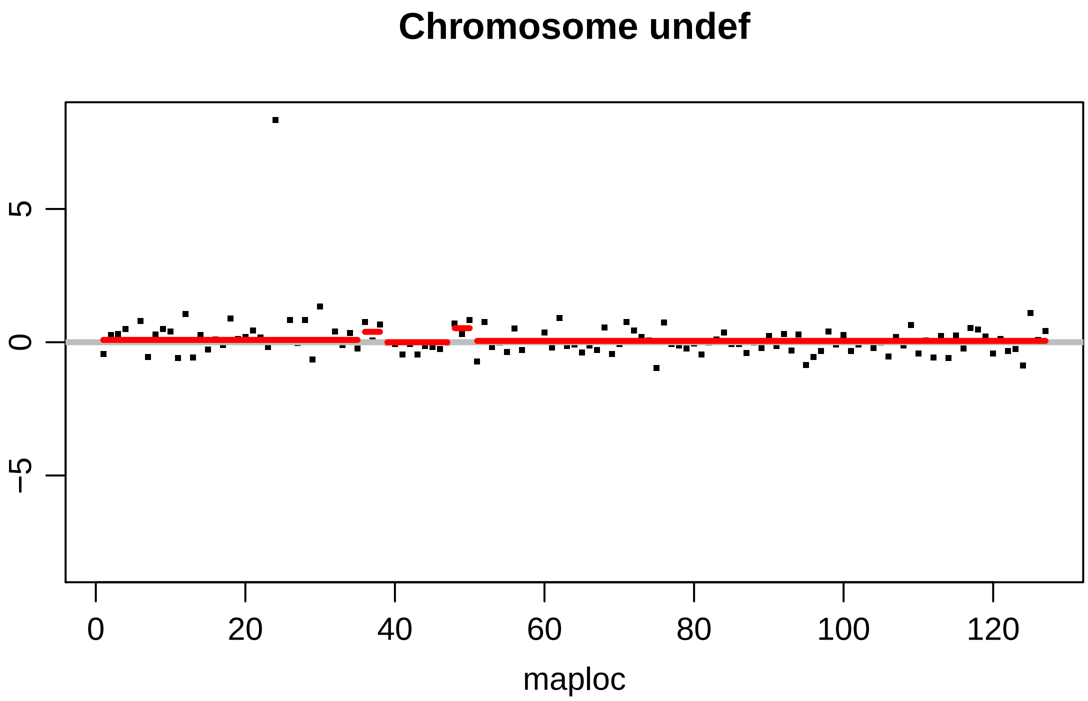

```
## Segplot might not work because of special characters in the sample names. Use only A-Z,a-z and 0-9!
##  There is a hidden function cn.mops:::.replaceNames that replaces the names in the "CNVDetectionResul
```

_10_21_46_user_SN2.2.Neonatal.Assay.LEx.2run.18.11.13_Auto_user_SN2.2.Neona

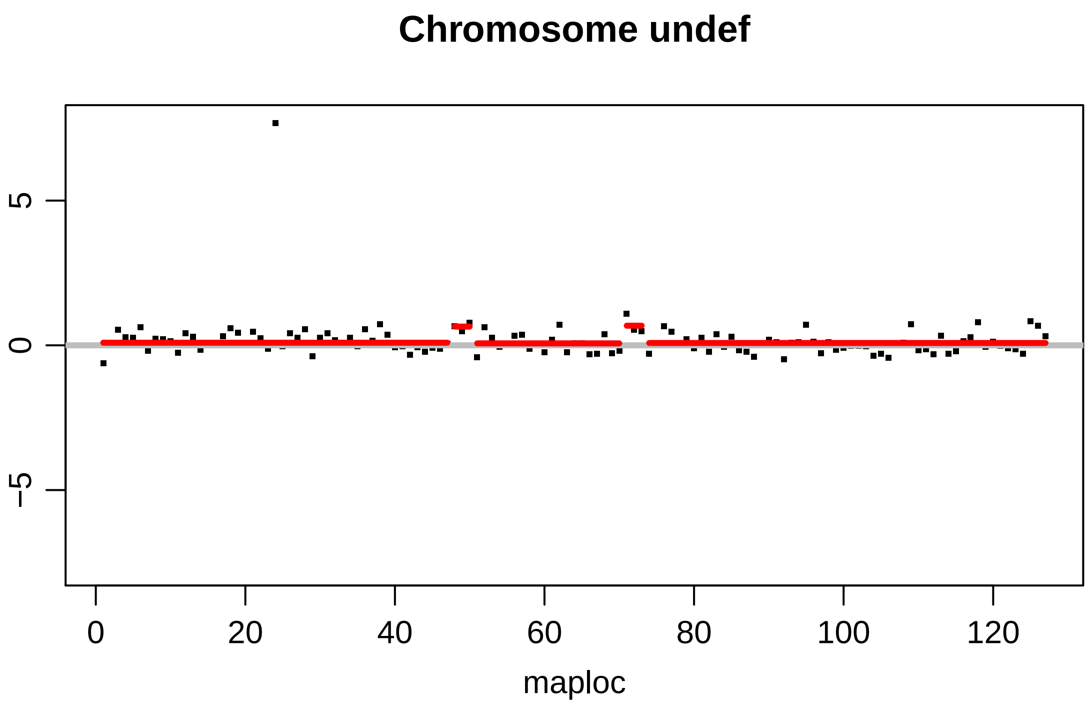


```
## Segplot might not work because of special characters in the sample names. Use only A-Z,a-z and 0-9!
##  There is a hidden function cn.mops:::.replaceNames that replaces the names in the "CNVDetectionResul
```

_10_21_46_user_SN2.2.Neonatal.Assay.LEx.2run.18.11.13_Auto_user_SN2.2.Neon

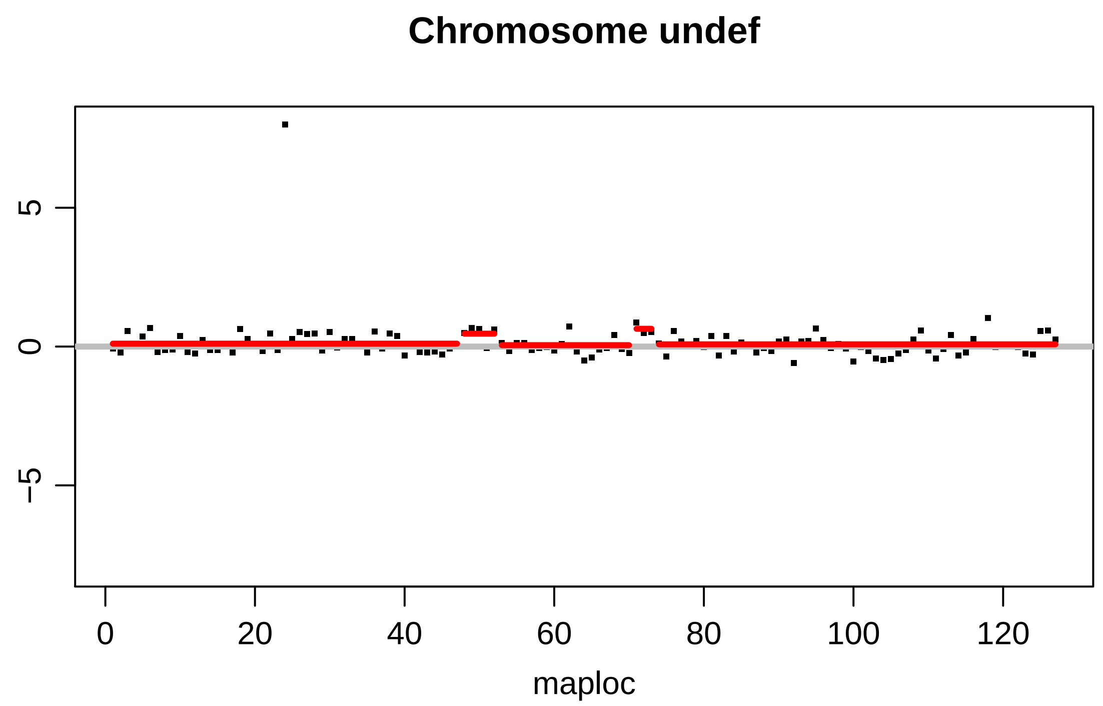


```
## Segplot might not work because of special characters in the sample names. Use only A-Z,a-z and 0-9!
##  There is a hidden function cn.mops:::.replaceNames that replaces the names in the "CNVDetectionResul
```

_10_21_46_user_SN2.2.Neonatal.Assay.LEx.2run.18.11.13_Auto_user_SN2.2.Neon

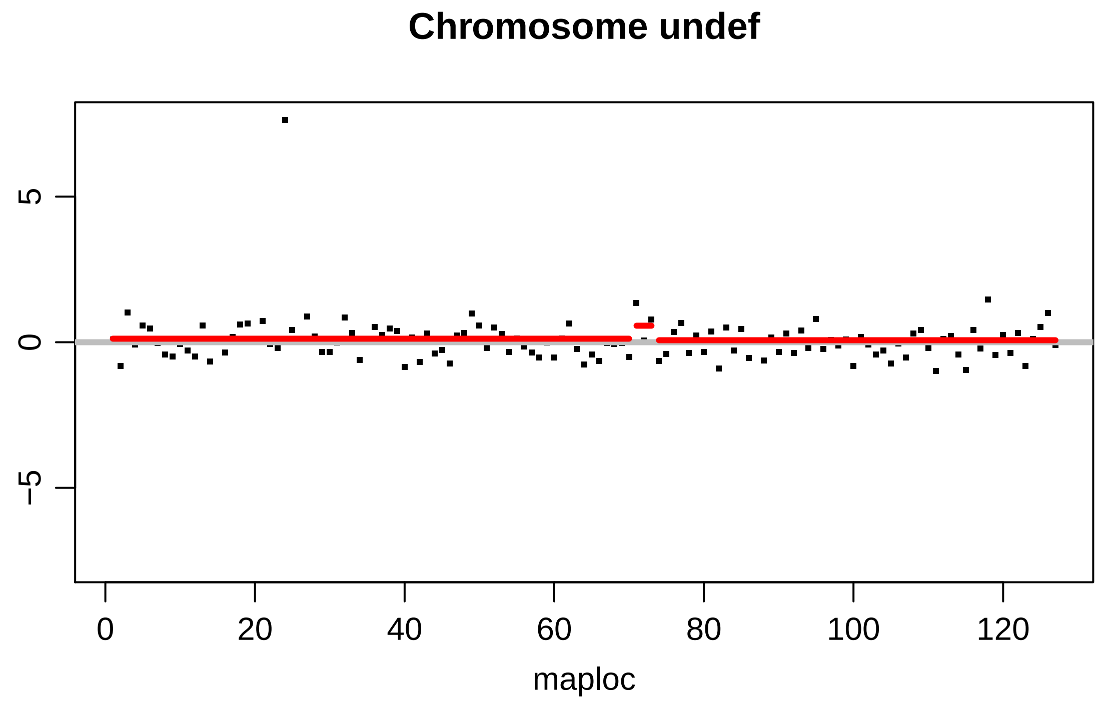

```
## Segplot might not work because of special characters in the sample names. Use only A-Z,a-z and 0-9!
##  There is a hidden function cn.mops:::.replaceNames that replaces the names in the "CNVDetectionResul
```

_10_21_46_user_SN2.2.Neonatal.Assay.LEx.2run.18.11.13_Auto_user_SN2.2.Neona

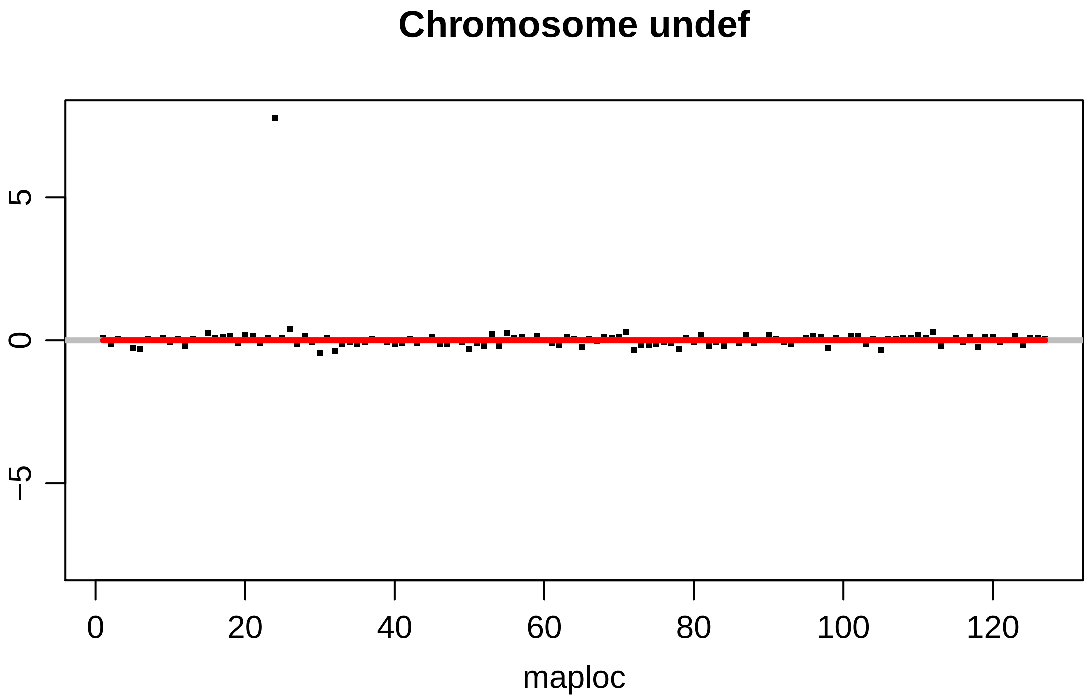


```
## Segplot might not work because of special characters in the sample names. Use only A-Z,a-z and 0-9!
##  There is a hidden function cn.mops:::.replaceNames that replaces the names in the "CNVDetectionResul
```

_10_21_46_user_SN2.2.Neonatal.Assay.LEx.2run.18.11.13_Auto_user_SN2.2.Neon

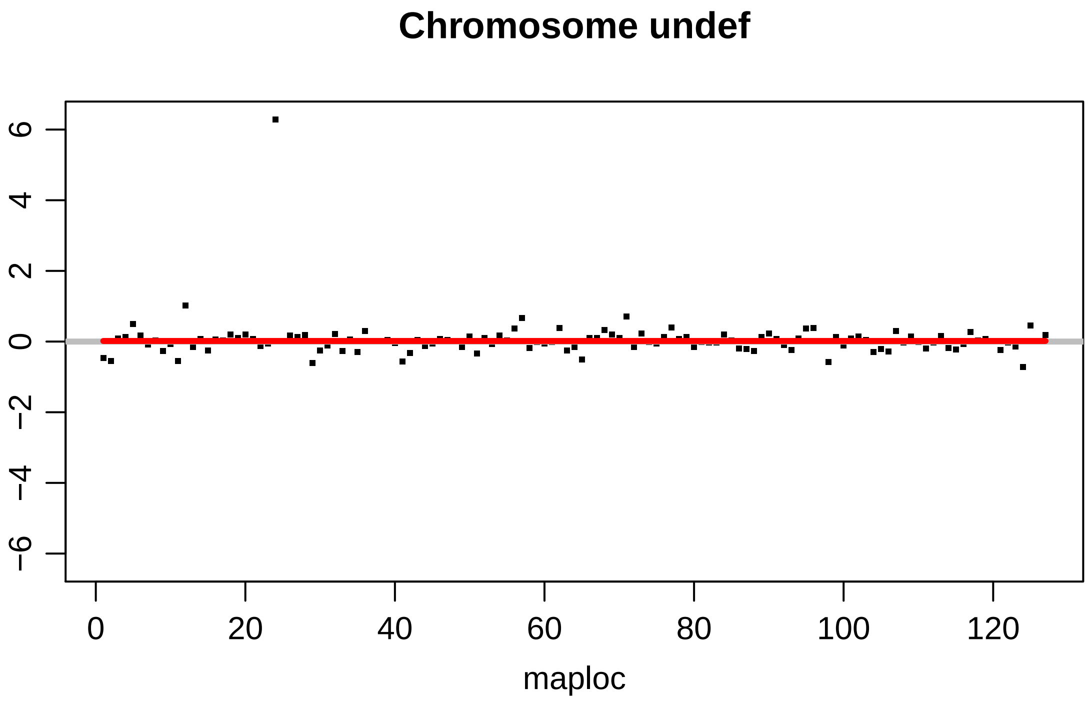


```
## Segplot might not work because of special characters in the sample names. Use only A-Z,a-z and 0-9!
##  There is a hidden function cn.mops:::.replaceNames that replaces the names in the "CNVDetectionResul
```

_10_21_46_user_SN2.2.Neonatal.Assay.LEx.2run.18.11.13_Auto_user_SN2.2.Neon

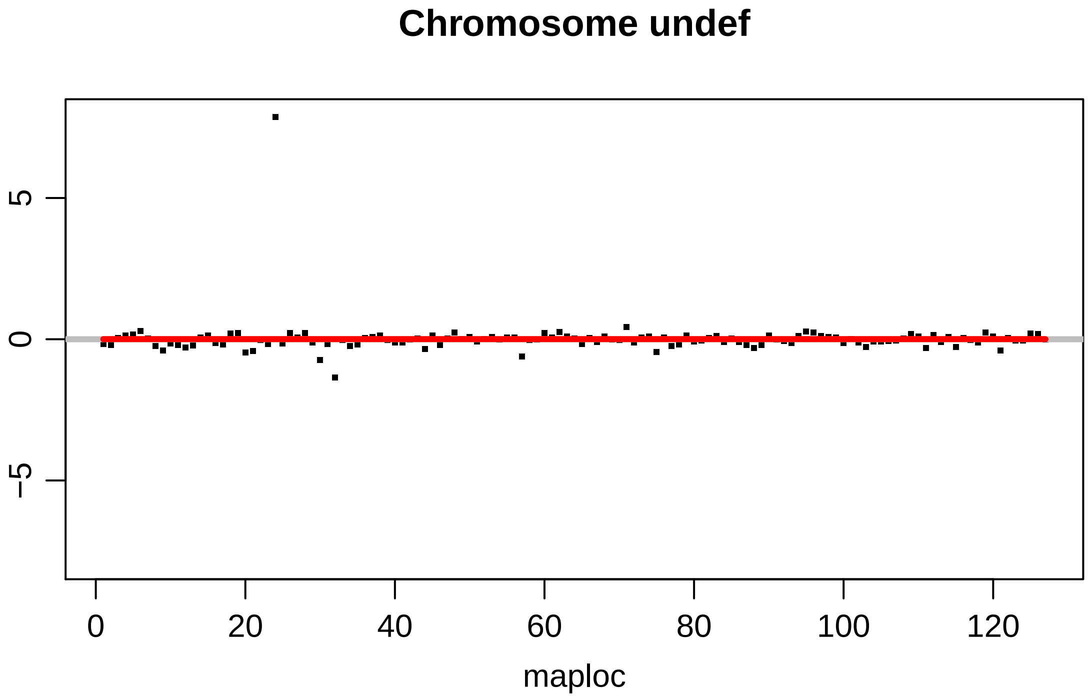

```
## Segplot might not work because of special characters in the sample names. Use only A-Z,a-z and 0-9!
##  There is a hidden function cn.mops:::.replaceNames that replaces the names in the "CNVDetectionResul
```

_10_21_46_user_SN2.2.Neonatal.Assay.LEx.2run.18.11.13_Auto_user_SN2.2.Neona

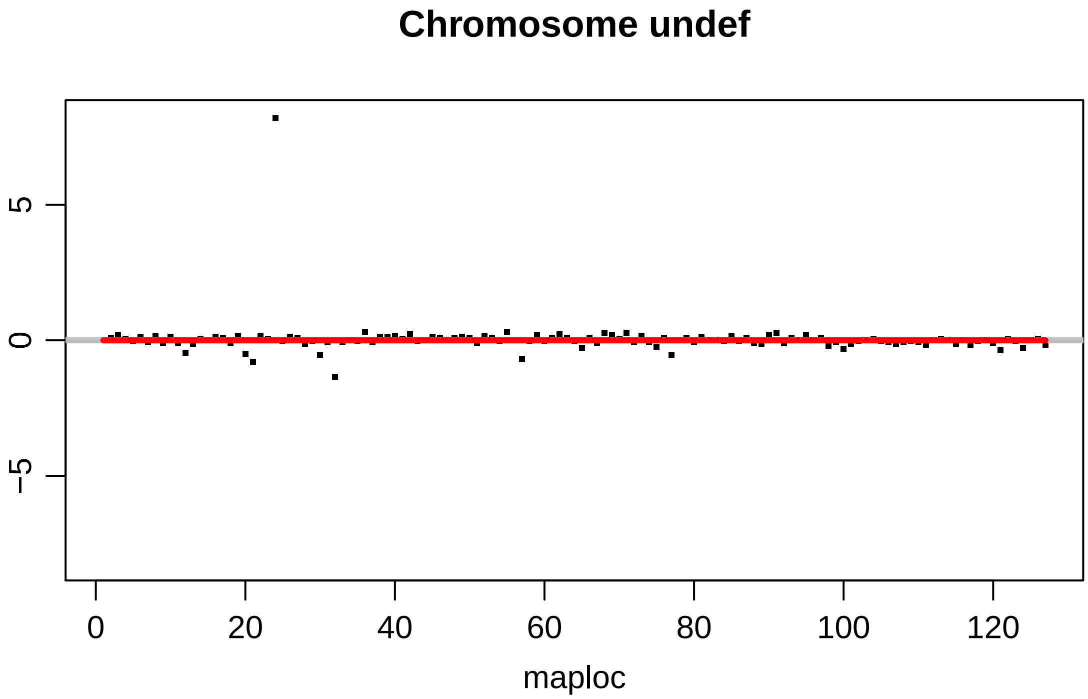


```
## Segplot might not work because of special characters in the sample names. Use only A-Z,a-z and 0-9!
##  There is a hidden function cn.mops:::.replaceNames that replaces the names in the "CNVDetectionResul
```

_10_21_46_user_SN2.2.Neonatal.Assay.LEx.2run.18.11.13_Auto_user_SN2.2.Neon

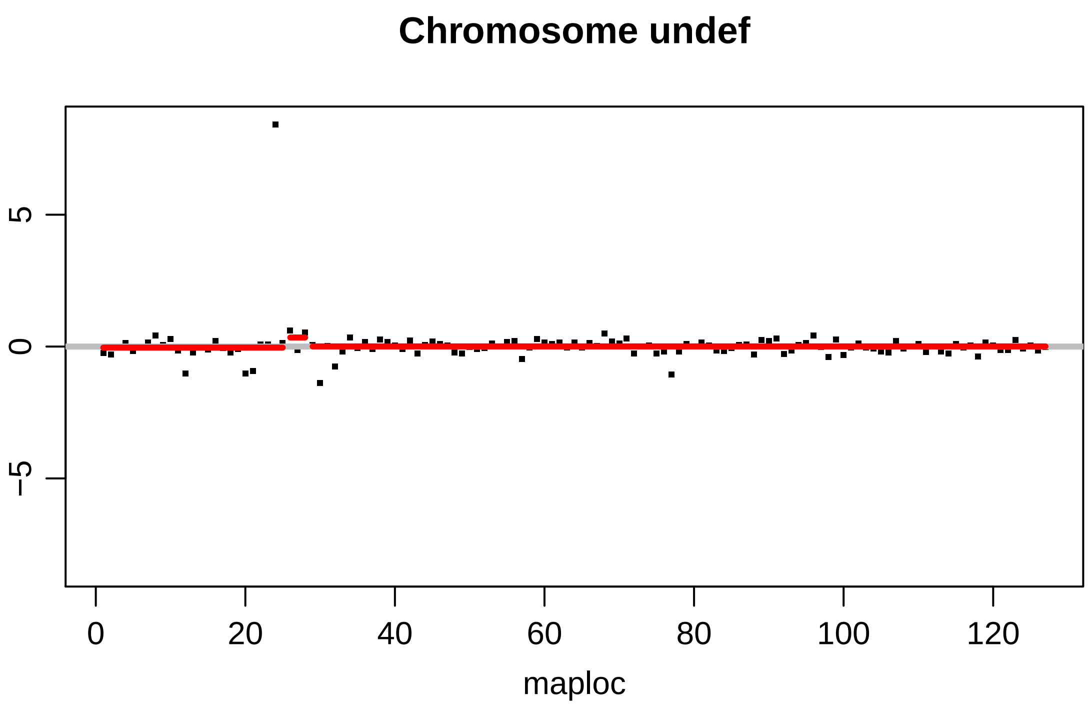


```
## Segplot might not work because of special characters in the sample names. Use only A-Z,a-z and 0-9!
##  There is a hidden function cn.mops:::.replaceNames that replaces the names in the "CNVDetectionResul
```

_10_21_46_user_SN2.2.Neonatal.Assay.LEx.2run.18.11.13_Auto_user_SN2.2.Neon

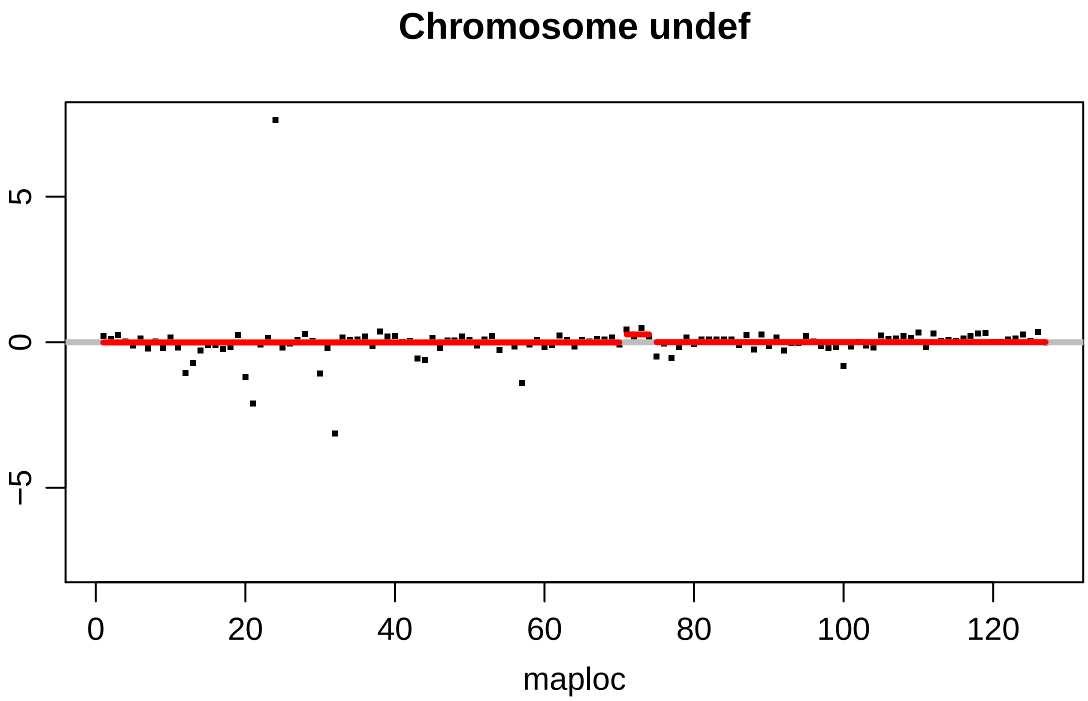

```
## Segplot might not work because of special characters in the sample names. Use only A-Z,a-z and 0-9!
##  There is a hidden function cn.mops:::.replaceNames that replaces the names in the "CNVDetectionResul
```

_10_21_46_user_SN2.2.Neonatal.Assay.LEx.2run.18.11.13_Auto_user_SN2.2.Neona

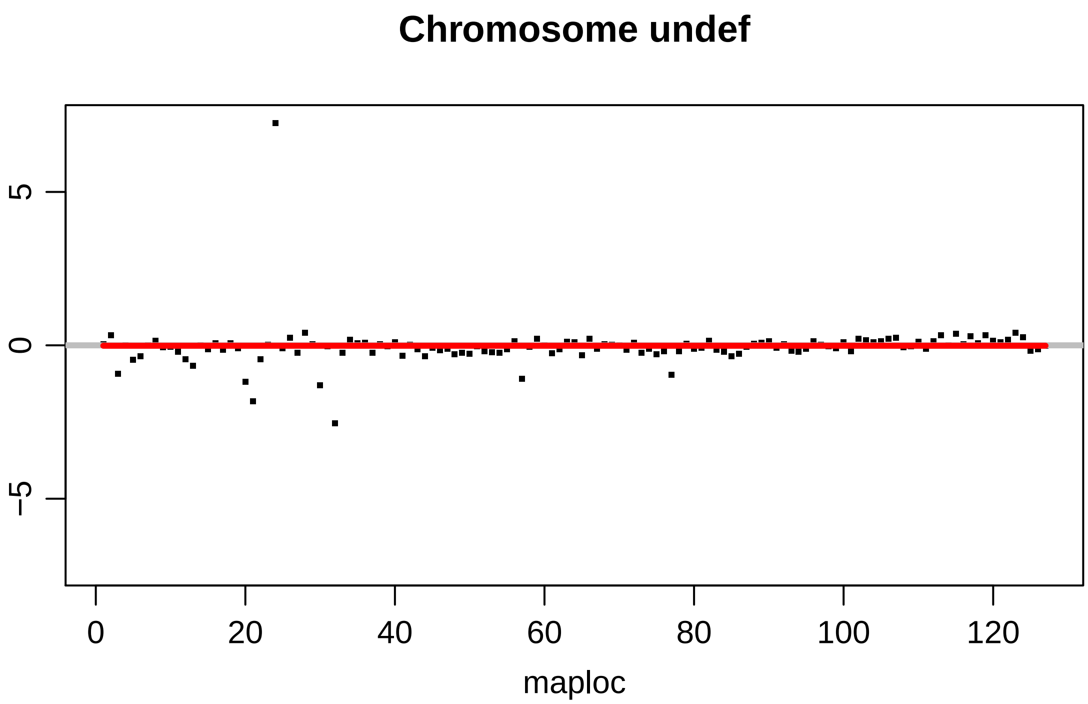


```
## Segplot might not work because of special characters in the sample names. Use only A-Z,a-z and 0-9!
##  There is a hidden function cn.mops:::.replaceNames that replaces the names in the "CNVDetectionResul
```

_10_21_46_user_SN2.2.Neonatal.Assay.LEx.2run.18.11.13_Auto_user_SN2.2.Neon

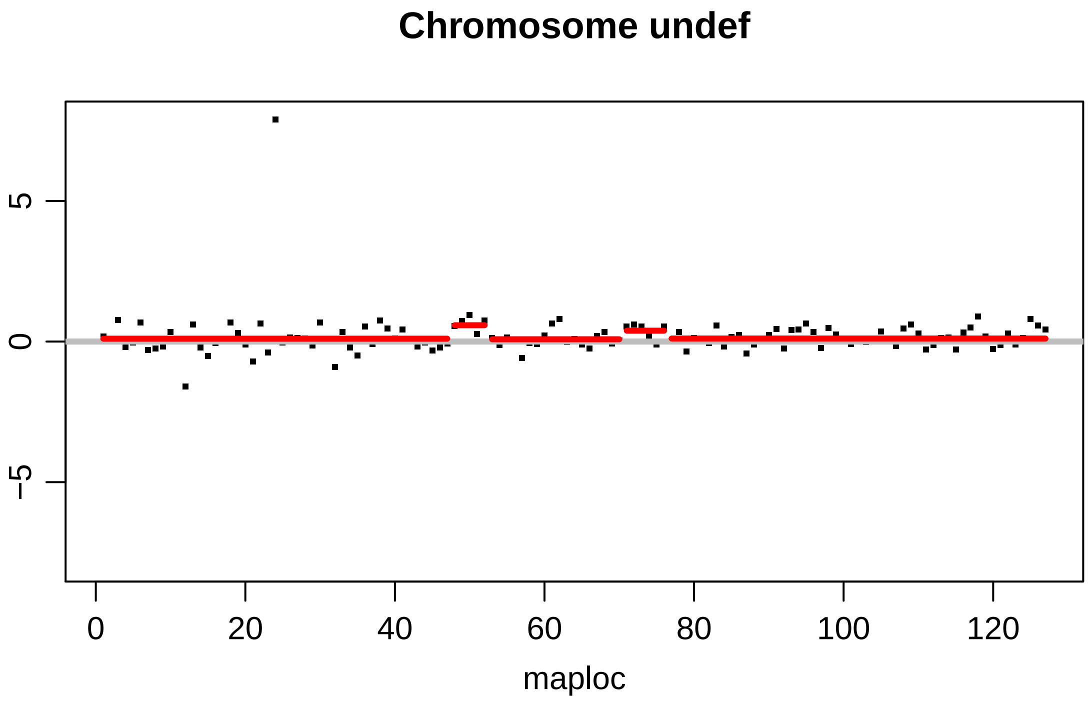


```
## Segplot might not work because of special characters in the sample names. Use only A-Z,a-z and 0-9!
##  There is a hidden function cn.mops:::.replaceNames that replaces the names in the "CNVDetectionResul
```

_10_21_46_user_SN2.2.Neonatal.Assay.LEx.2run.18.11.13_Auto_user_SN2.2.Neon

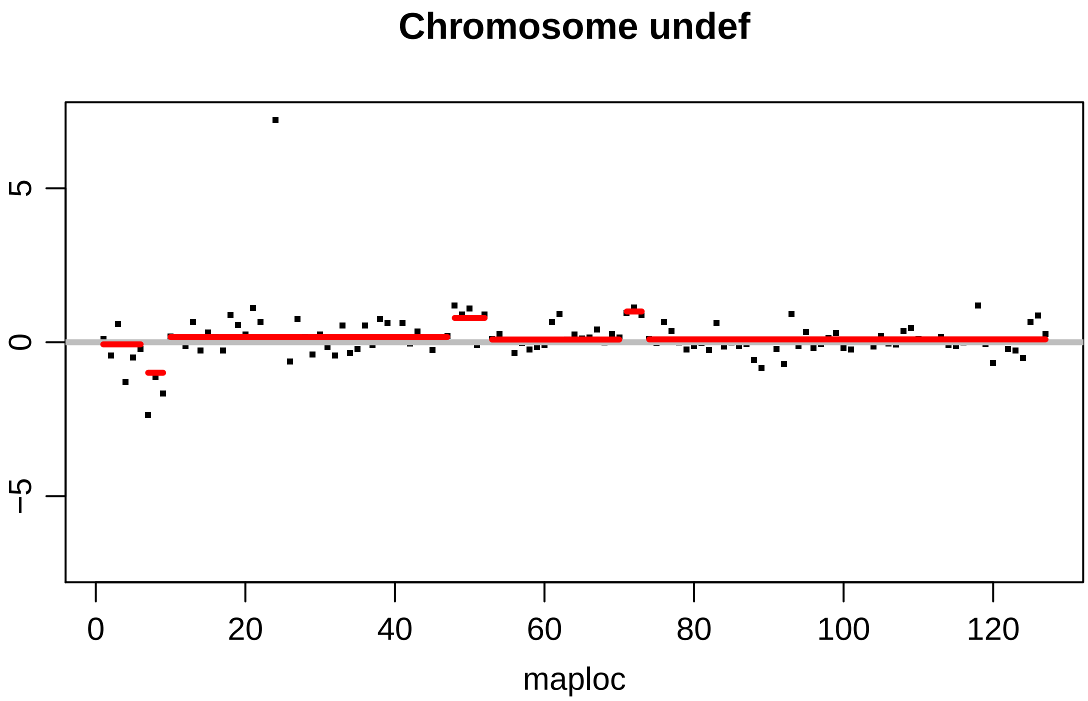

```
## Segplot might not work because of special characters in the sample names. Use only A-Z,a-z and 0-9!
##  There is a hidden function cn.mops:::.replaceNames that replaces the names in the "CNVDetectionResul
```

_10_21_46_user_SN2.2.Neonatal.Assay.LEx.2run.18.11.13_Auto_user_SN2.2.Neon

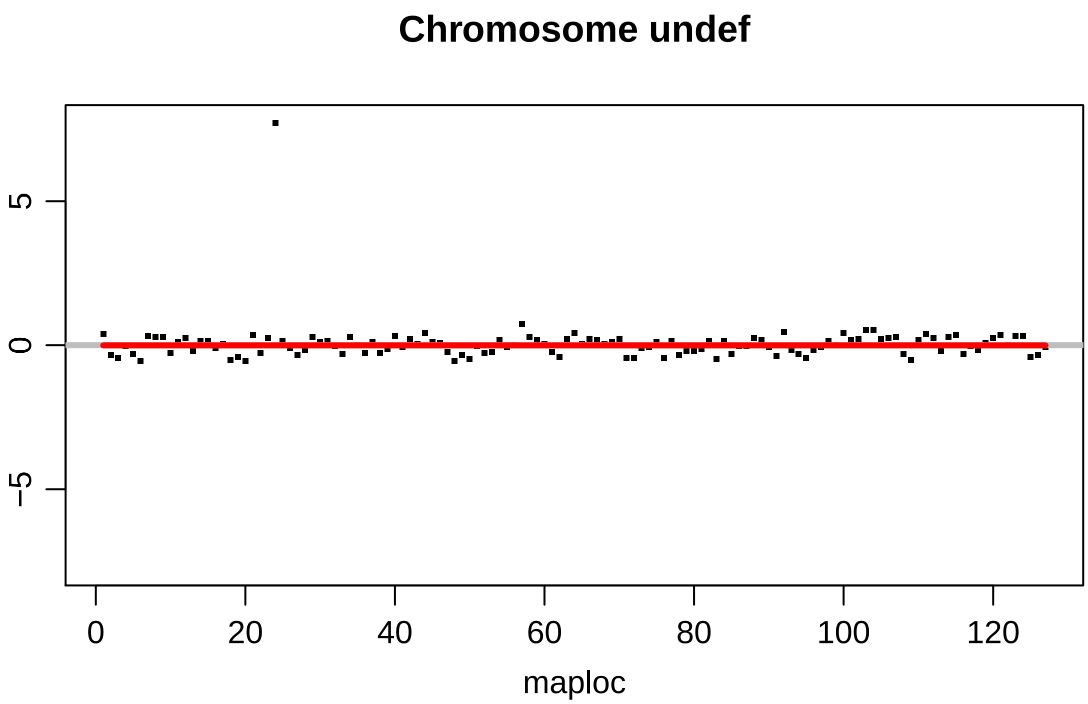


```
## Segplot might not work because of special characters in the sample names. Use only A-Z,a-z and 0-9!
##  There is a hidden function cn.mops:::.replaceNames that replaces the names in the "CNVDetectionResul
```

_10_21_46_user_SN2.2.Neonatal.Assay.LEx.2run.18.11.13_Auto_user_SN2.2.Neon

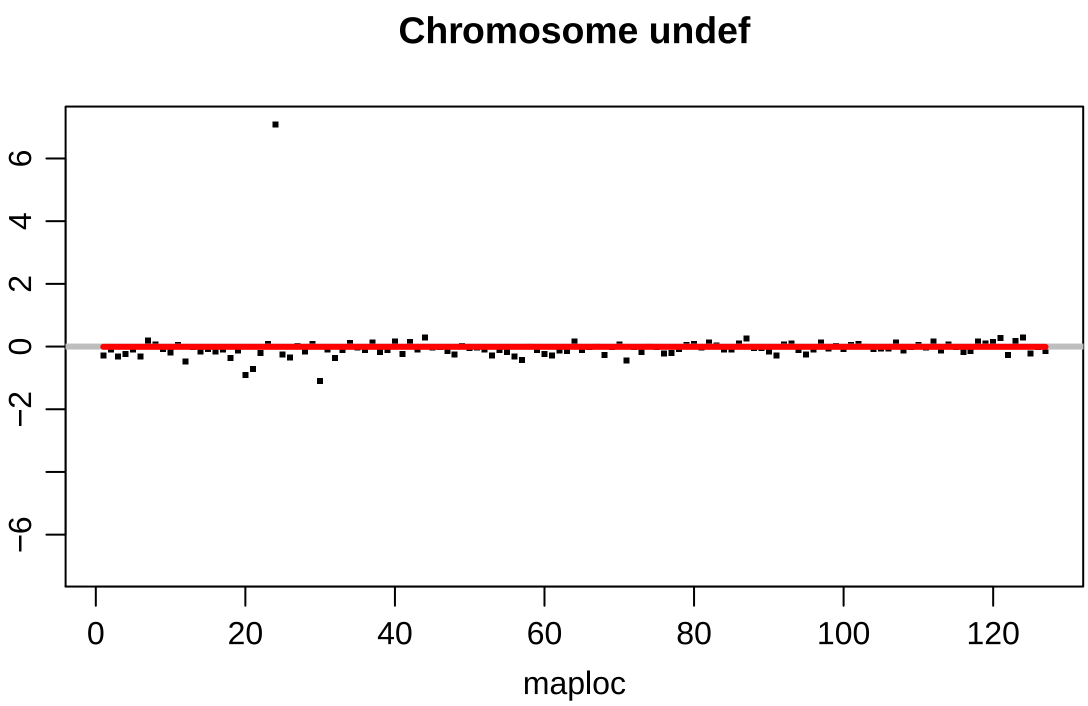


```
## Segplot might not work because of special characters in the sample names. Use only A-Z,a-z and 0-9!
##  There is a hidden function cn.mops:::.replaceNames that replaces the names in the "CNVDetectionResul
```

_10_21_46_user_SN2.2.Neonatal.Assay.LEx.2run.18.11.13_Auto_user_SN2.2.Neon

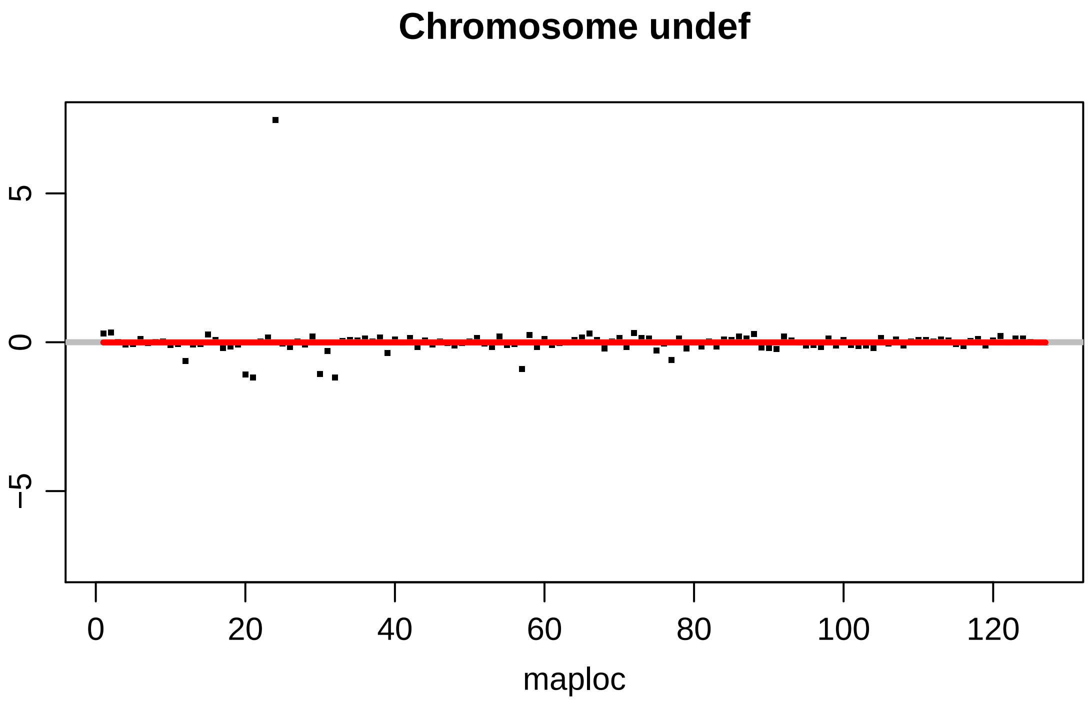

```
## Segplot might not work because of special characters in the sample names. Use only A-Z,a-z and 0-9!
##  There is a hidden function cn.mops:::.replaceNames that replaces the names in the "CNVDetectionResul
```

_10_21_46_user_SN2.2.Neonatal.Assay.LEx.2run.18.11.13_Auto_user_SN2.2.Neona

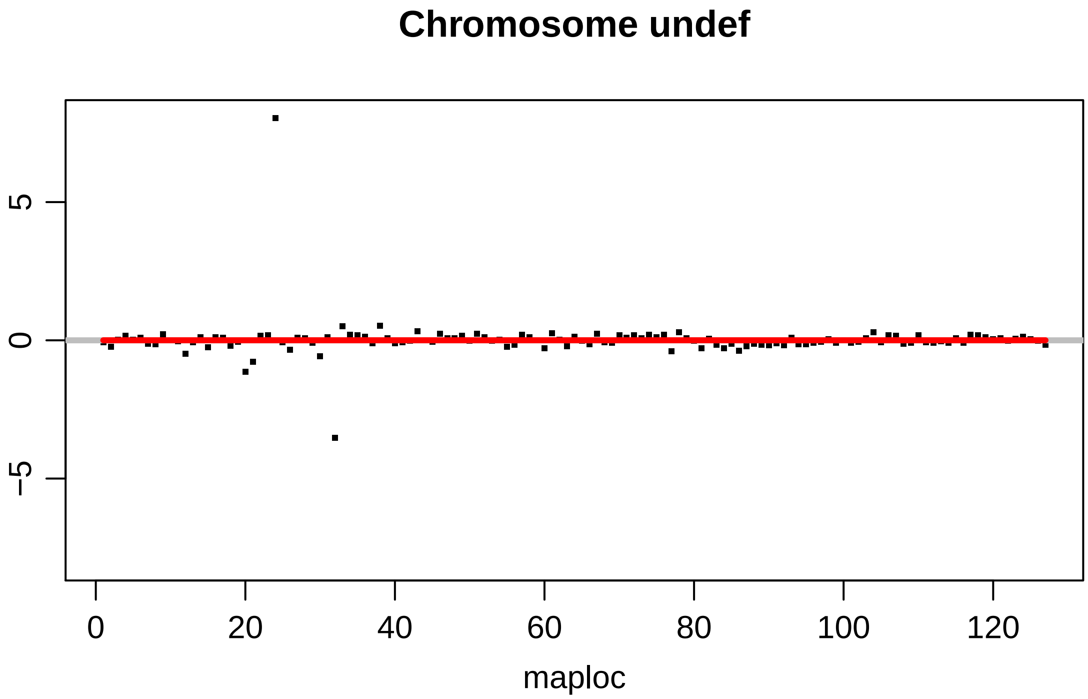


```
## Segplot might not work because of special characters in the sample names. Use only A-Z,a-z and 0-9!
##  There is a hidden function cn.mops:::.replaceNames that replaces the names in the "CNVDetectionResul
```

_10_21_46_user_SN2.2.Neonatal.Assay.LEx.2run.18.11.13_Auto_user_SN2.2.Neon

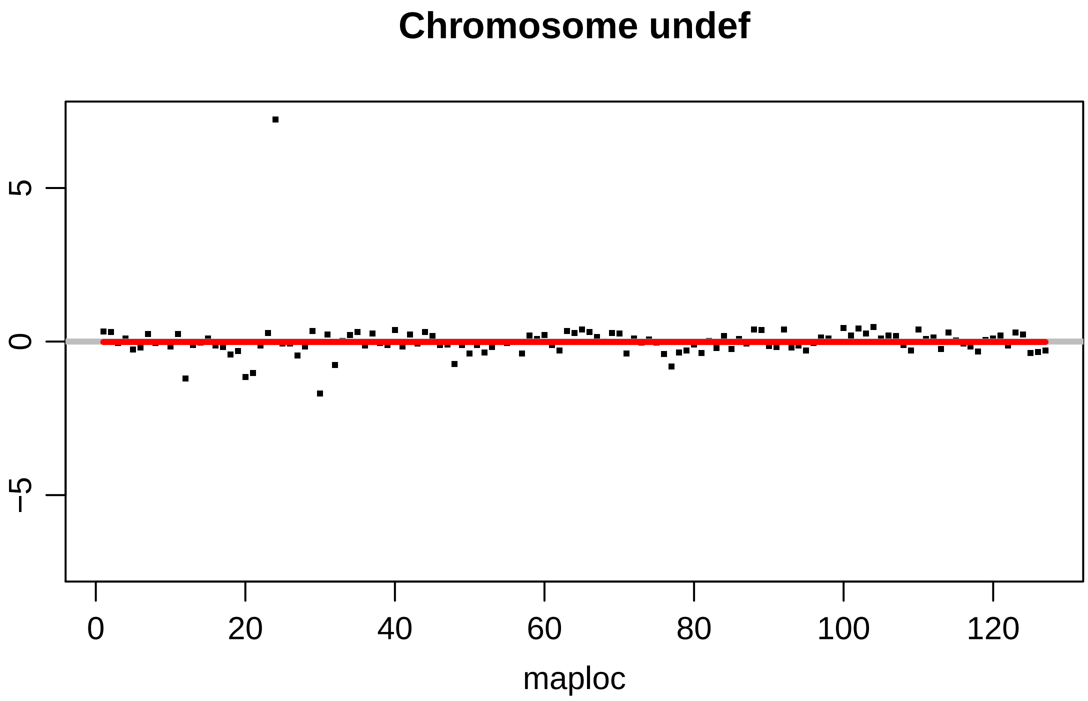


```
## Segplot might not work because of special characters in the sample names. Use only A-Z,a-z and 0-9!
##  There is a hidden function cn.mops:::.replaceNames that replaces the names in the "CNVDetectionResul
```

_10_21_46_user_SN2.2.Neonatal.Assay.LEx.2run.18.11.13_Auto_user_SN2.2.Neon

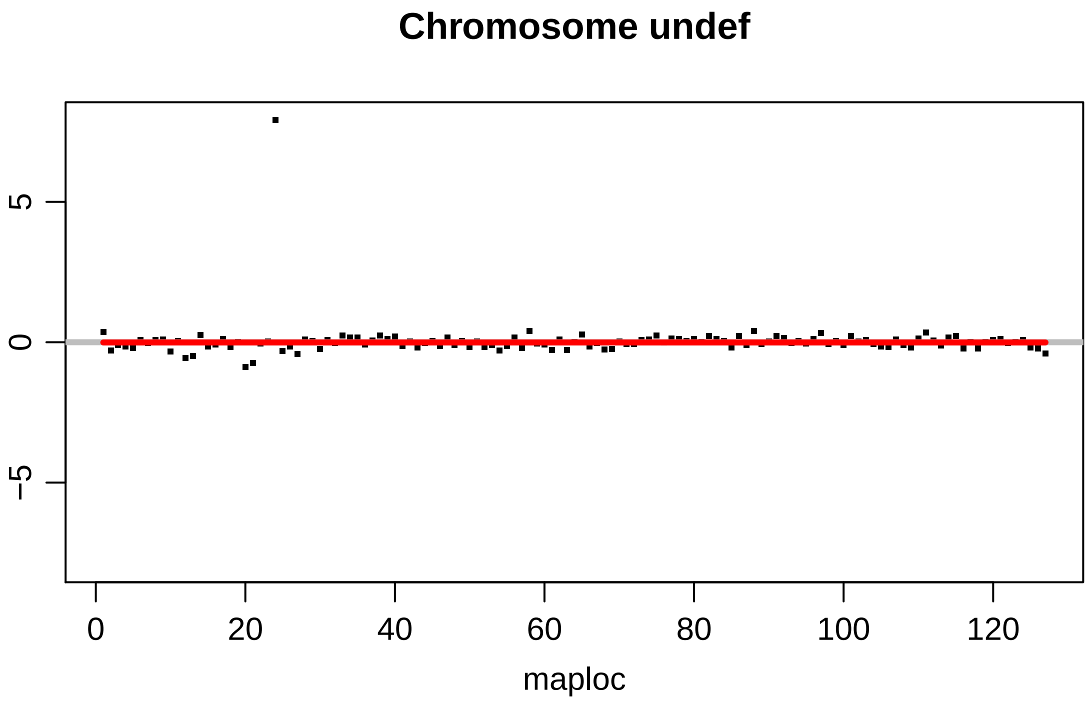

```
## Segplot might not work because of special characters in the sample names. Use only A-Z,a-z and 0-9!
##  There is a hidden function cn.mops:::.replaceNames that replaces the names in the "CNVDetectionResul
```

_10_21_46_user_SN2.2.Neonatal.Assay.LEx.2run.18.11.13_Auto_user_SN2.2.Neon

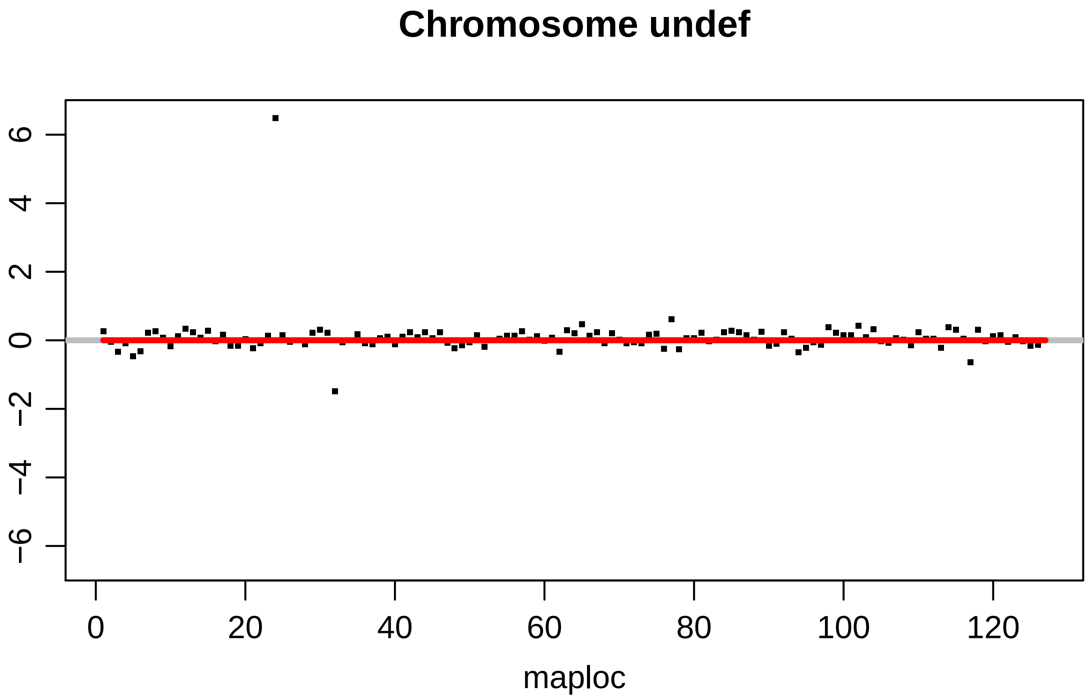


```
## Segplot might not work because of special characters in the sample names. Use only A-Z,a-z and 0-9!
##  There is a hidden function cn.mops:::.replaceNames that replaces the names in the "CNVDetectionResul
```

_10_21_46_user_SN2.2.Neonatal.Assay.LEx.2run.18.11.13_Auto_user_SN2.2.Neon[illegible]

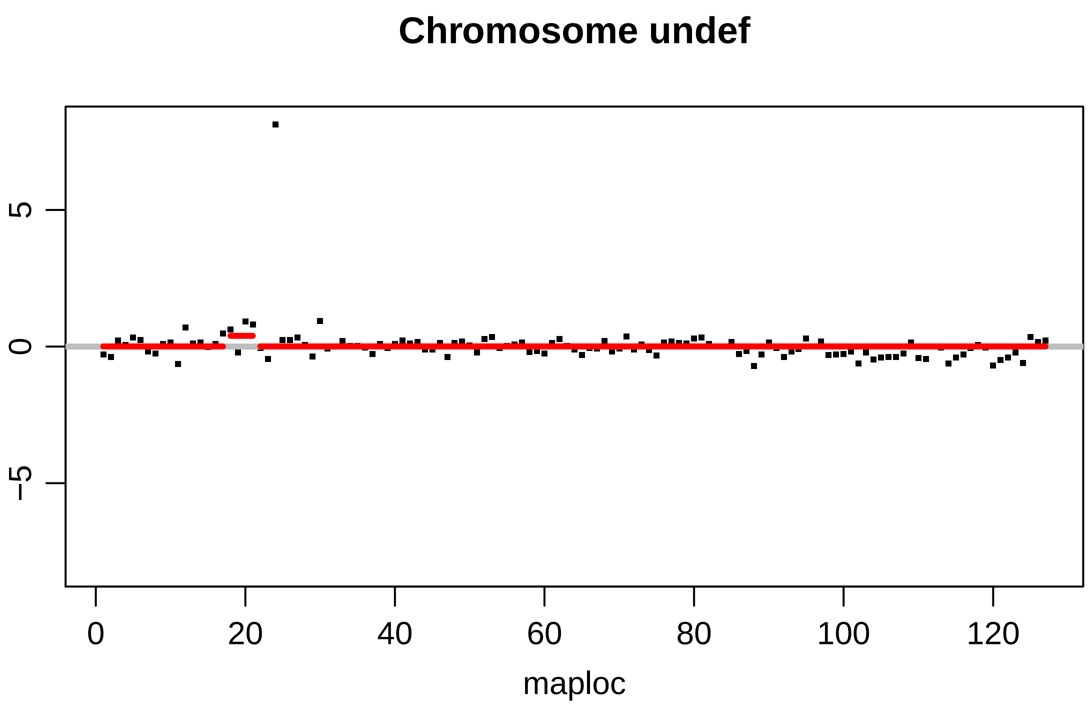


```
## Segplot might not work because of special characters in the sample names. Use only A-Z,a-z and 0-9!
##  There is a hidden function cn.mops:::.replaceNames that replaces the names in the "CNVDetectionResul
```

_10_21_46_user_SN2.2.Neonatal.Assay.LEx.2run.18.11.13_Auto_user_SN2.2.Neon[illegible]

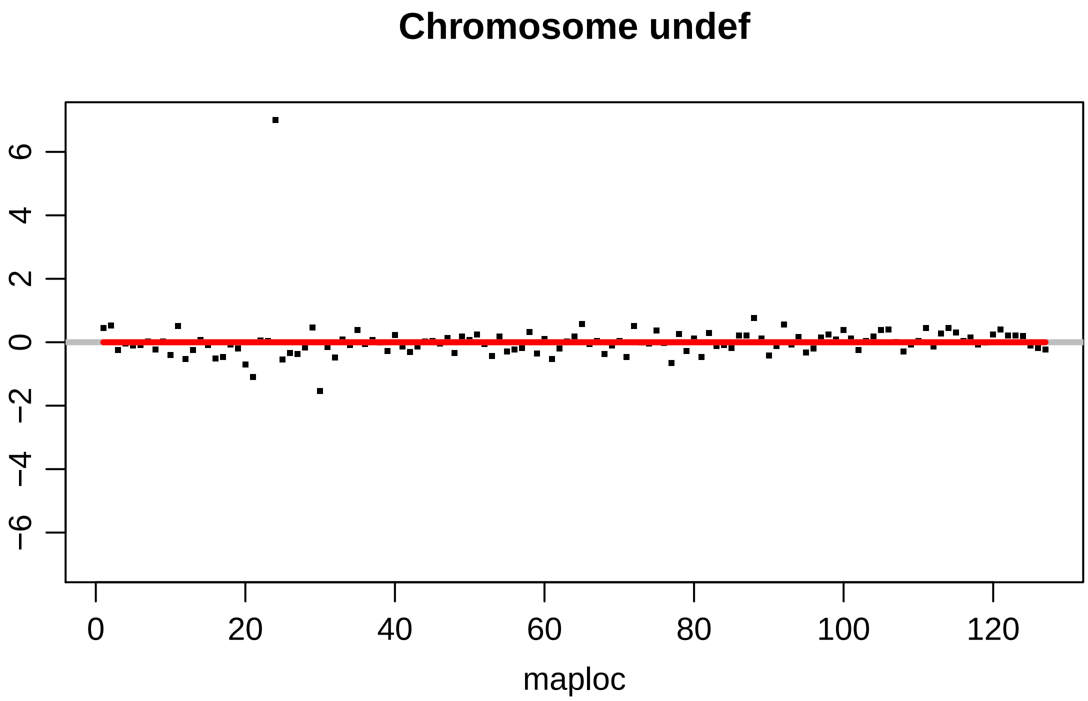

```
## Segplot might not work because of special characters in the sample names. Use only A-Z,a-z and 0-9!
##  There is a hidden function cn.mops:::.replaceNames that replaces the names in the "CNVDetectionResul
```

_10_21_46_user_SN2.2.Neonatal.Assay.LEx.2run.18.11.13_Auto_user_SN2.2.Neon

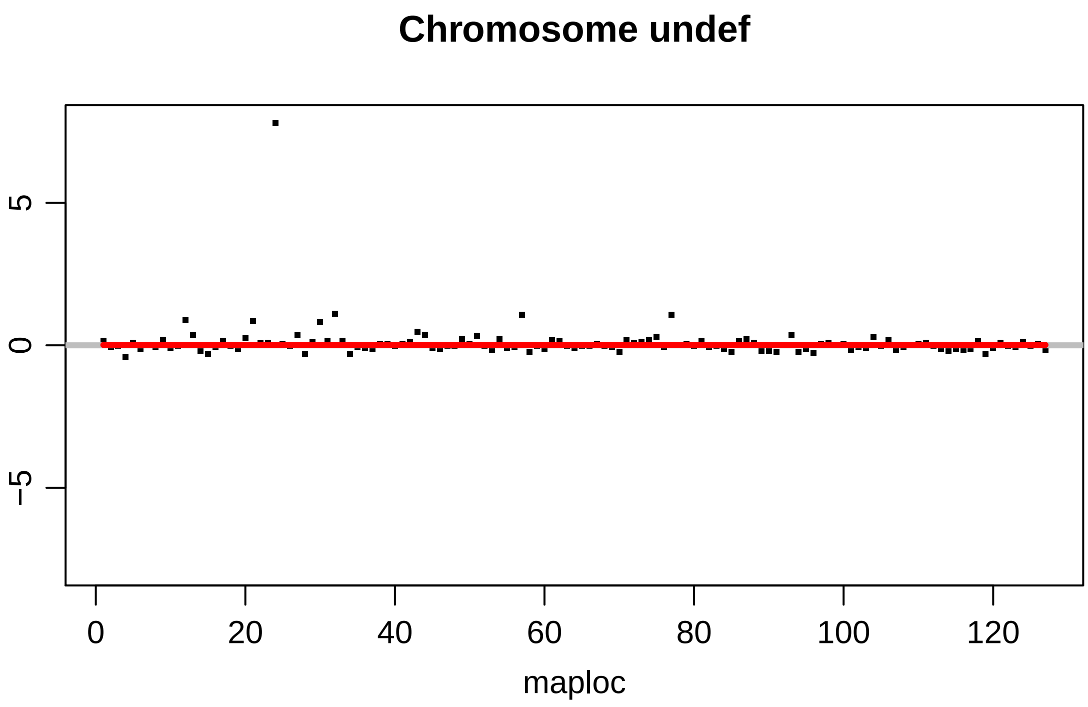


```
## Segplot might not work because of special characters in the sample names. Use only A-Z,a-z and 0-9!
##  There is a hidden function cn.mops:::.replaceNames that replaces the names in the "CNVDetectionResul
```

_10_21_46_user_SN2.2.Neonatal.Assay.LEx.2run.18.11.13_Auto_user_SN2.2.Neon

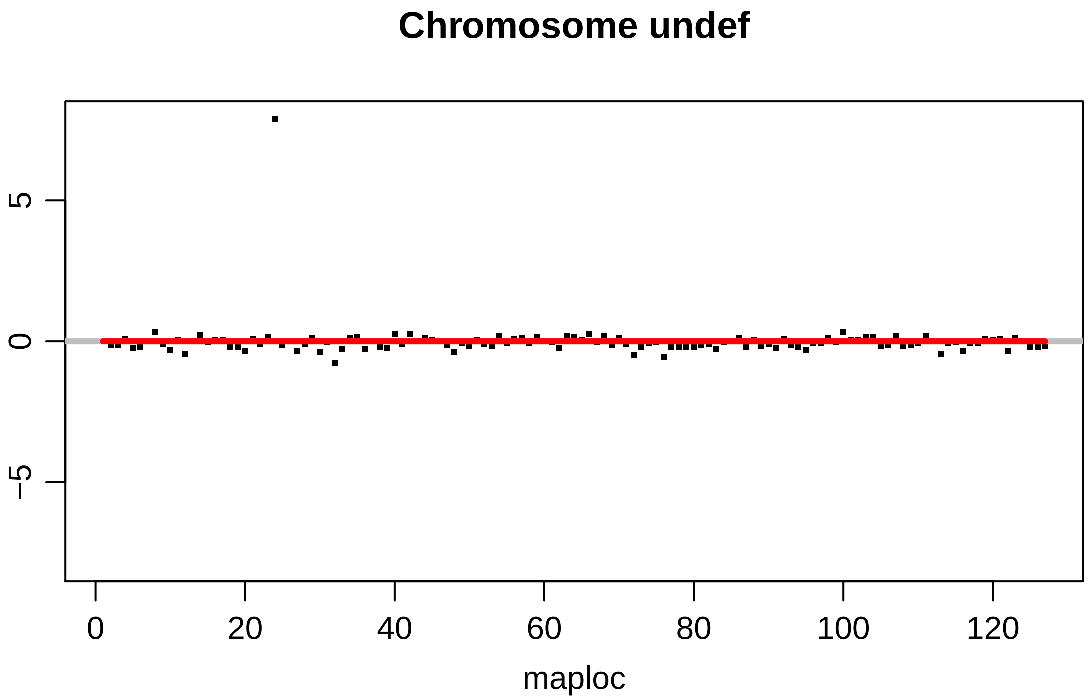


```
## Segplot might not work because of special characters in the sample names. Use only A-Z,a-z and 0-9!
##  There is a hidden function cn.mops:::.replaceNames that replaces the names in the "CNVDetectionResul
```

_10_21_46_user_SN2.2.Neonatal.Assay.LEx.2run.18.11.13_Auto_user_SN2.2.Neon

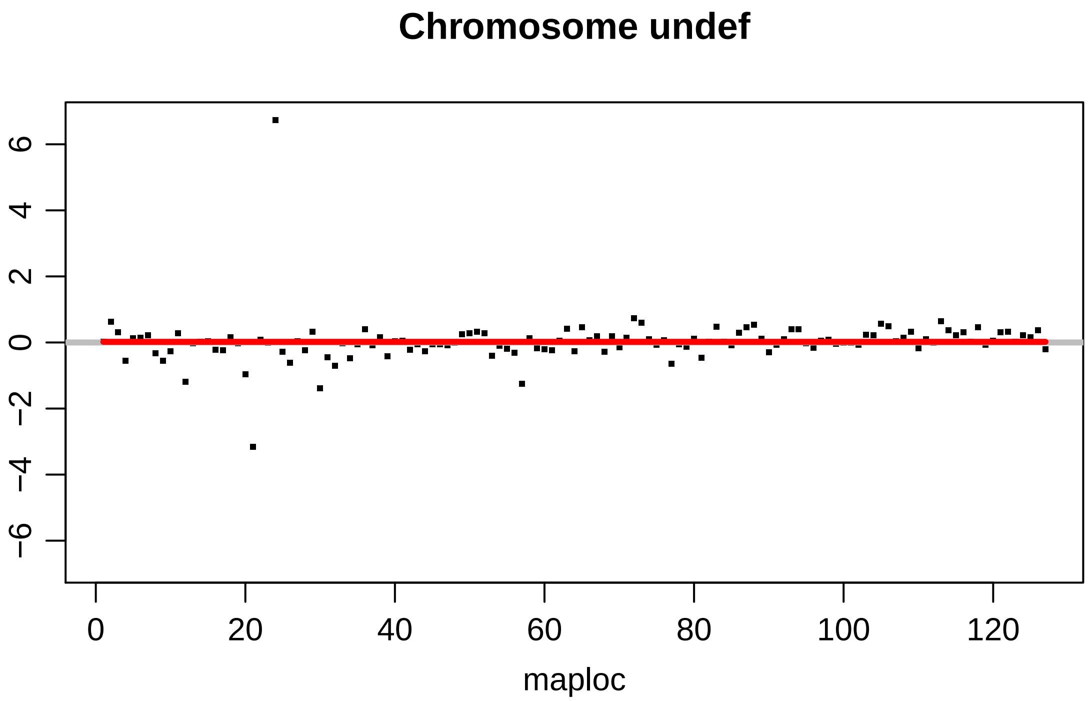

```
## Segplot might not work because of special characters in the sample names. Use only A-Z,a-z and 0-9!
##  There is a hidden function cn.mops:::.replaceNames that replaces the names in the "CNVDetectionResul
```

_10_21_46_user_SN2.2.Neonatal.Assay.LEx.2run.18.11.13_Auto_user_SN2.2.Neona

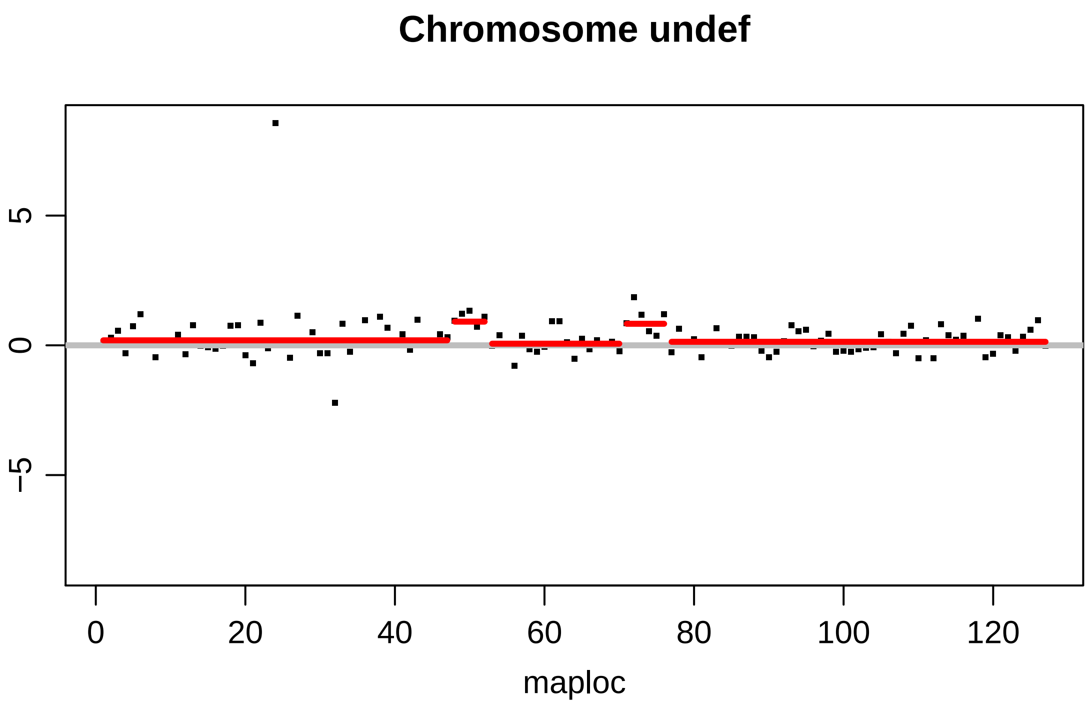


```
## Segplot might not work because of special characters in the sample names. Use only A-Z,a-z and 0-9!
##  There is a hidden function cn.mops:::.replaceNames that replaces the names in the "CNVDetectionResul
```

_10_21_46_user_SN2.2.Neonatal.Assay.LEx.2run.18.11.13_Auto_user_SN2.2.Neon

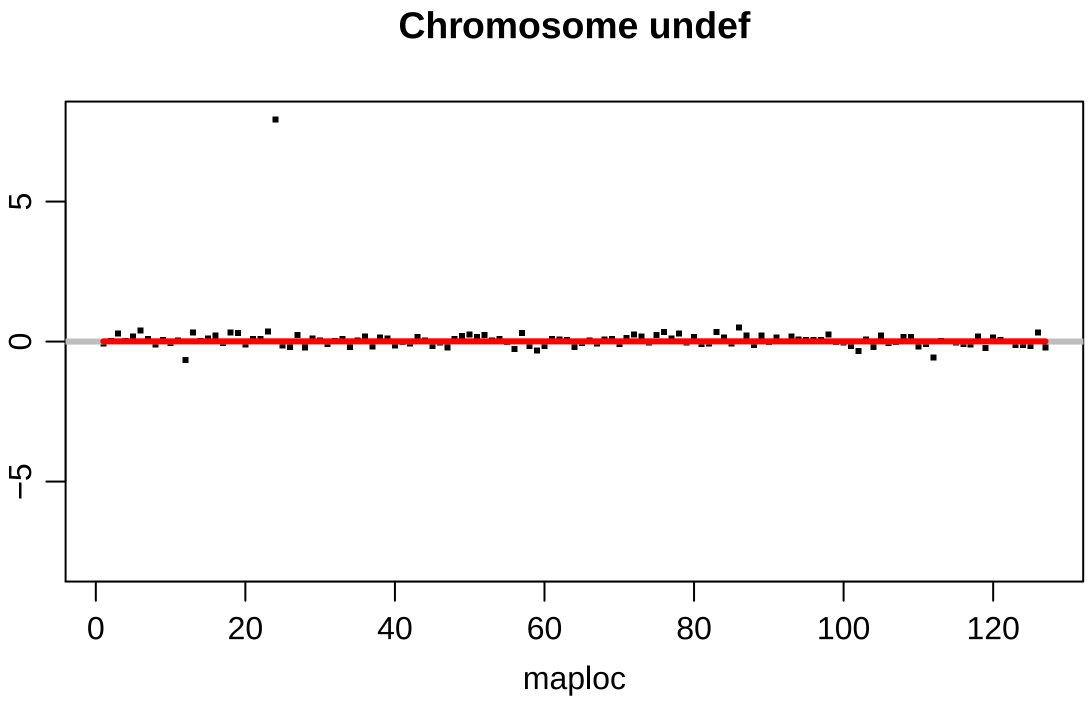


```
## Segplot might not work because of special characters in the sample names. Use only A-Z,a-z and 0-9!
##  There is a hidden function cn.mops:::.replaceNames that replaces the names in the "CNVDetectionResul
```

_10_21_46_user_SN2.2.Neonatal.Assay.LEx.2run.18.11.13_Auto_user_SN2.2.Neon

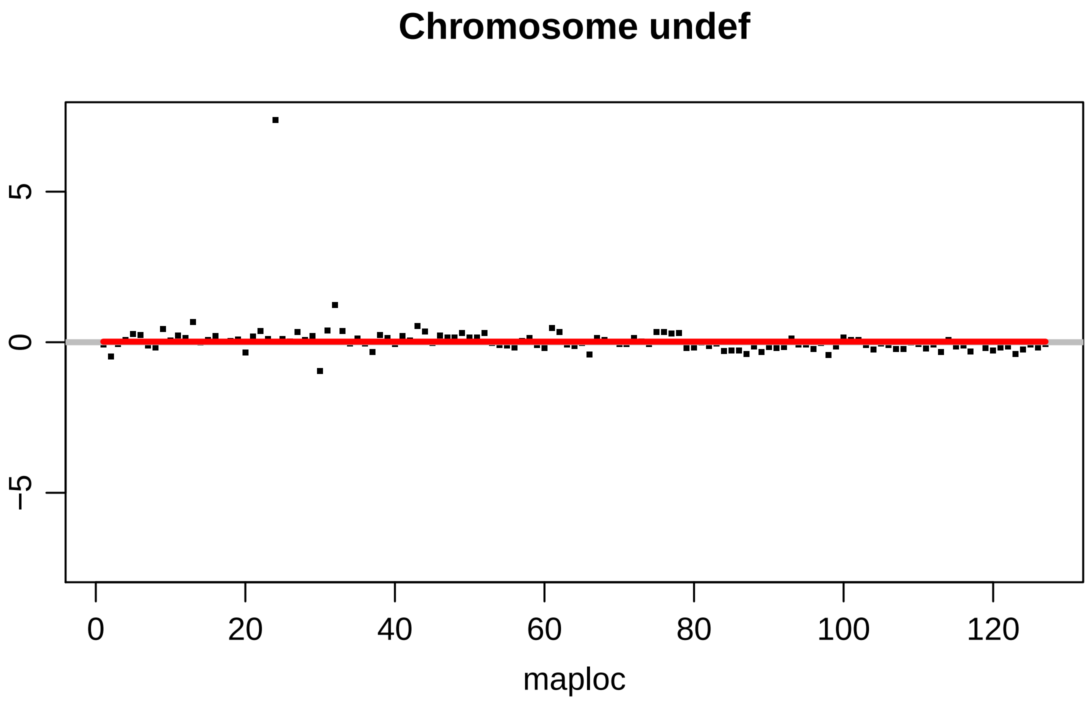

```
## Segplot might not work because of special characters in the sample names. Use only A-Z,a-z and 0-9!
##  There is a hidden function cn.mops:::.replaceNames that replaces the names in the "CNVDetectionResul
```

_10_21_46_user_SN2.2.Neonatal.Assay.LEx.2run.18.11.13_Auto_user_SN2.2.Neona

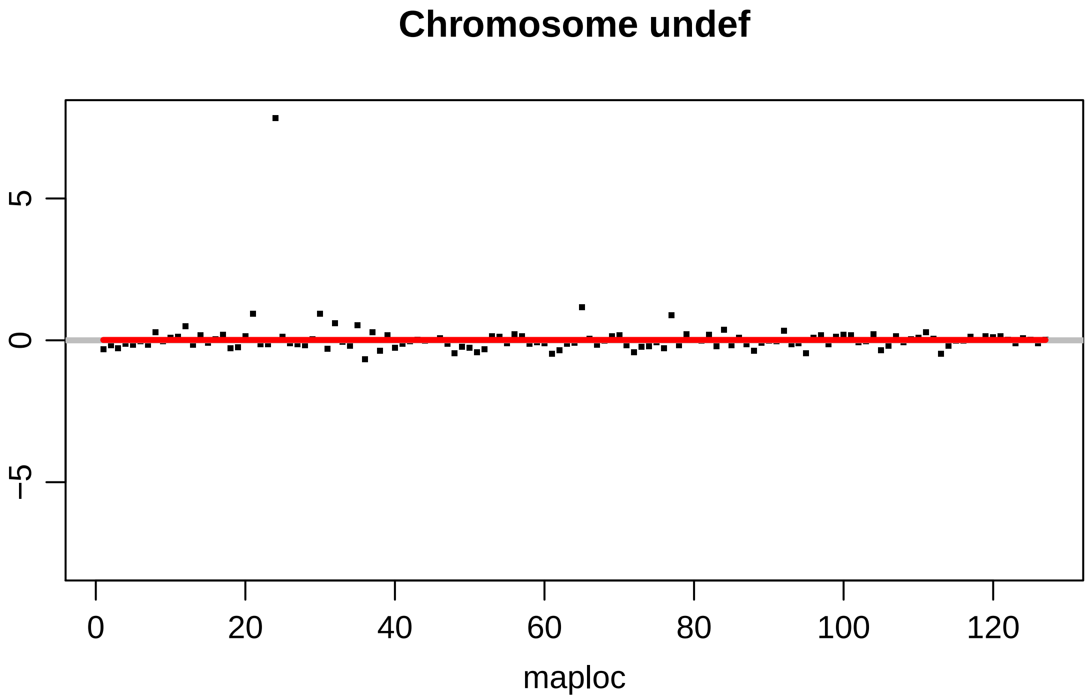


```
## Segplot might not work because of special characters in the sample names. Use only A-Z,a-z and 0-9!
##  There is a hidden function cn.mops:::.replaceNames that replaces the names in the "CNVDetectionResul
```

_10_21_46_user_SN2.2.Neonatal.Assay.LEx.2run.18.11.13_Auto_user_SN2.2.Neona

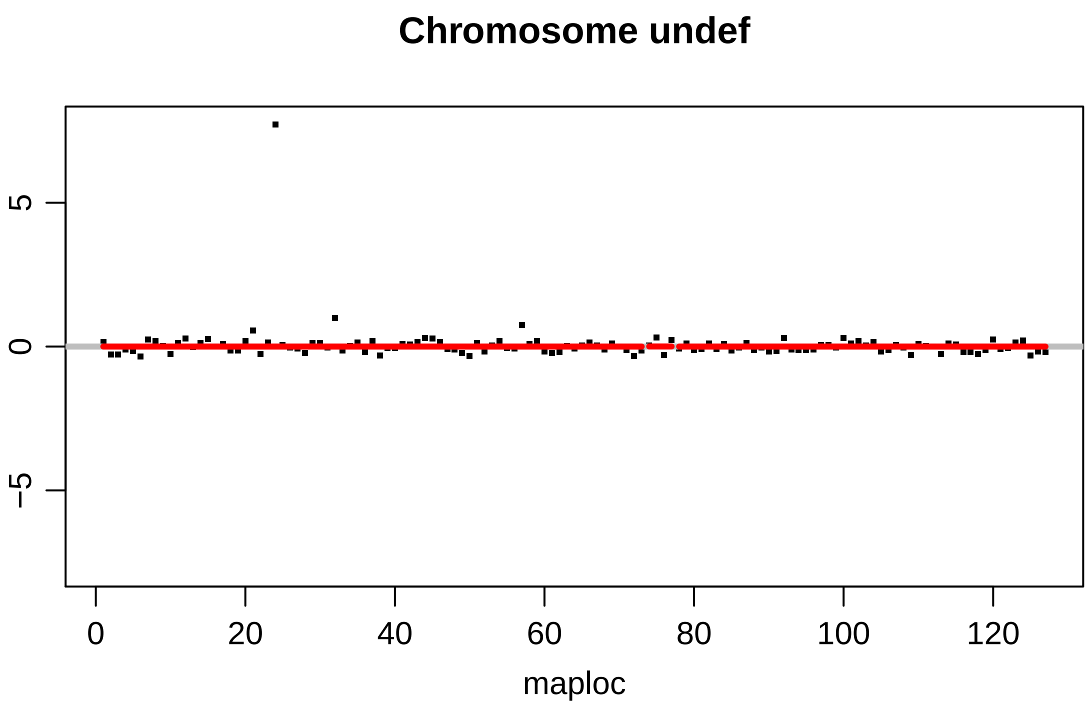


```
## Segplot might not work because of special characters in the sample names. Use only A-Z,a-z and 0-9!
##  There is a hidden function cn.mops:::.replaceNames that replaces the names in the "CNVDetectionResul
```

_10_21_46_user_SN2.2.Neonatal.Assay.LEx.2run.18.11.13_Auto_user_SN2.2.Neona

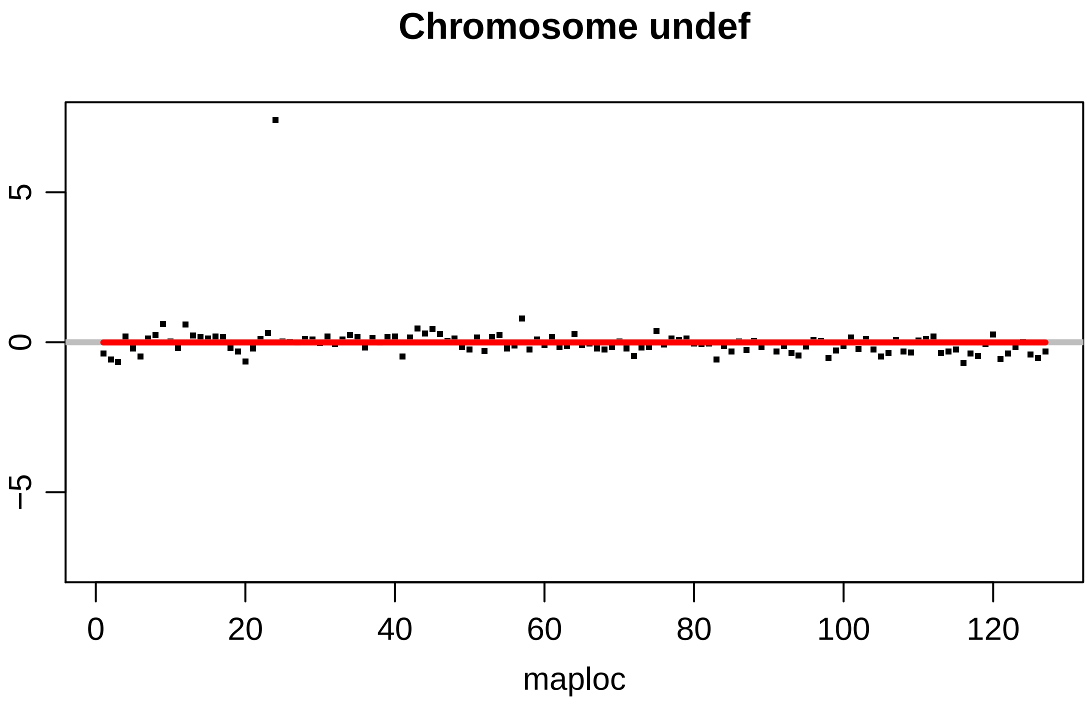

```
## Segplot might not work because of special characters in the sample names. Use only A-Z,a-z and 0-9!
##  There is a hidden function cn.mops:::.replaceNames that replaces the names in the "CNVDetectionResul
```

_10_21_46_user_SN2.2.Neonatal.Assay.LEx.2run.18.11.13_Auto_user_SN2.2.Neona

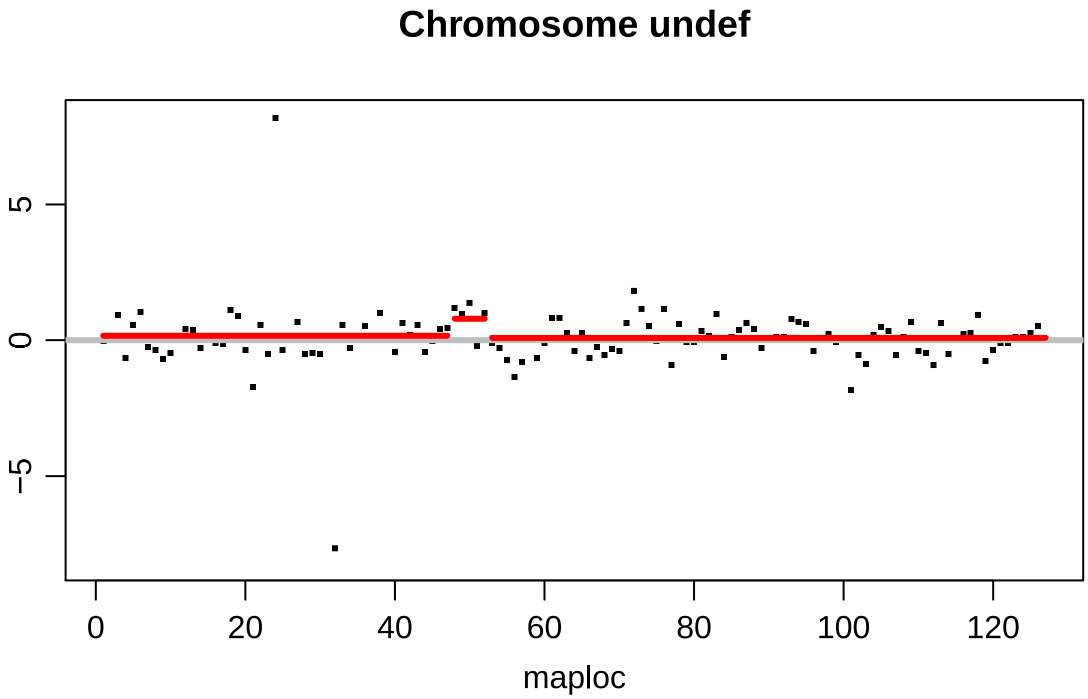


```
## Segplot might not work because of special characters in the sample names. Use only A-Z,a-z and 0-9!
##  There is a hidden function cn.mops:::.replaceNames that replaces the names in the "CNVDetectionResul
```

_10_21_46_user_SN2.2.Neonatal.Assay.LEx.2run.18.11.13_Auto_user_SN2.2.Neon

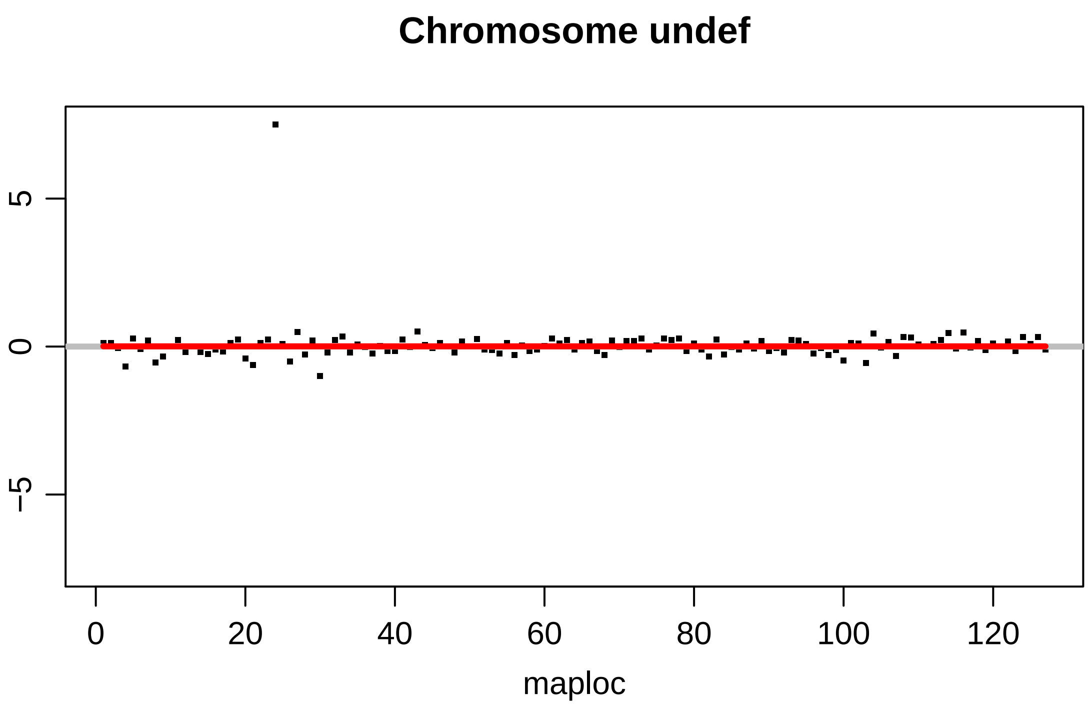


```
## Segplot might not work because of special characters in the sample names. Use only A-Z,a-z and 0-9!
##  There is a hidden function cn.mops:::.replaceNames that replaces the names in the "CNVDetectionResul
```

_10_21_46_user_SN2.2.Neonatal.Assay.LEx.2run.18.11.13_Auto_user_SN2.2.Neon

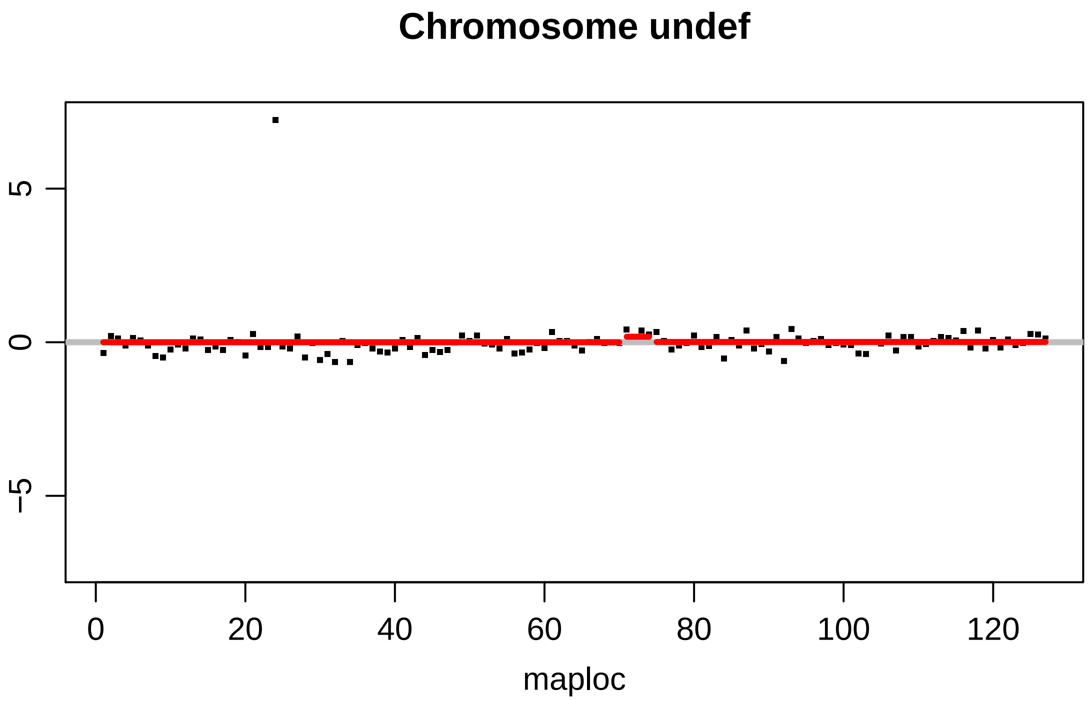

```
## Segplot might not work because of special characters in the sample names. Use only A-Z,a-z and 0-9!
##  There is a hidden function cn.mops:::.replaceNames that replaces the names in the "CNVDetectionResul
```

_10_21_46_user_SN2.2.Neonatal.Assay.LEx.2run.18.11.13_Auto_user_SN2.2.Neona

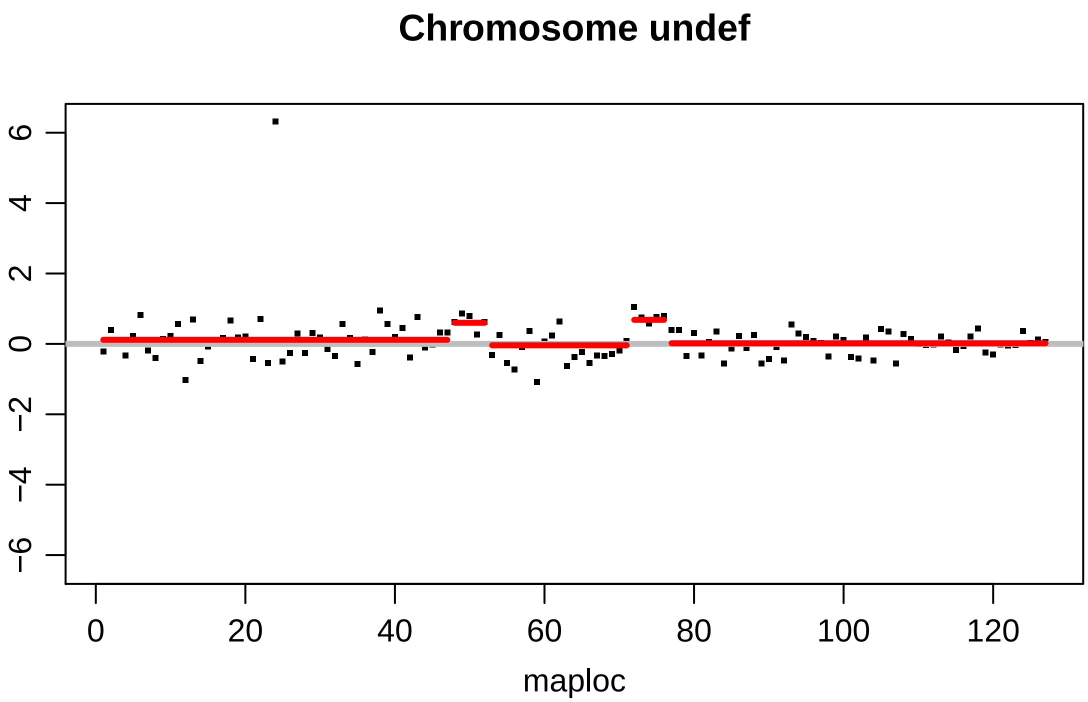


```
##
## CNV regions:
## GRanges object with 4 ranges and 43 metadata columns:
##       seqnames    ranges strand |
##          <Rle> <IRanges>  <Rle> |
##   [1]    undef  [ 7,  9]      * |
##   [2]    undef  [18, 21]      * |
##   [3]    undef  [48, 52]      * |
##   [4]    undef  [71, 76]      * |
##       Case_IonXpress_002_R_2013_10_17_10_21_46_user_SN2.2.Neonatal.Assay.LEx.2run.18.11.13_Auto_user_
##
##   [1]
##   [2]
##   [3]
##   [4]
##       Case_IonXpress_003_R_2013_10_17_10_21_46_user_SN2.2.Neonatal.Assay.LEx.2run.18.11.13_Auto_user_
##
##   [1]
##   [2]
##   [3]
##   [4]
##       Case_IonXpress_007_R_2013_10_17_10_21_46_user_SN2.2.Neonatal.Assay.LEx.2run.18.11.13_Auto_user_
##
##   [1]
##   [2]
```

```
##   [3]
##   [4]
##       Case_IonXpress_008_R_2013_10_17_10_21_46_user_SN2.2.Neonatal.Assay.LEx.2run.18.11.13_Auto_user_
##
##   [1]
##   [2]
##   [3]
##   [4]
##       Case_IonXpress_009_R_2013_10_17_10_21_46_user_SN2.2.Neonatal.Assay.LEx.2run.18.11.13_Auto_user_
##
##   [1]
##   [2]
##   [3]
##   [4]
##       Case_IonXpress_010_R_2013_10_17_10_21_46_user_SN2.2.Neonatal.Assay.LEx.2run.18.11.13_Auto_user_
##
##   [1]
##   [2]
##   [3]
##   [4]
##       Case_IonXpress_011_R_2013_10_17_10_21_46_user_SN2.2.Neonatal.Assay.LEx.2run.18.11.13_Auto_user_
##
##   [1]
##   [2]
##   [3]
##   [4]
##       Case_IonXpress_012_R_2013_10_17_10_21_46_user_SN2.2.Neonatal.Assay.LEx.2run.18.11.13_Auto_user_
##
##   [1]
##   [2]
##   [3]
##   [4]
##       Case_IonXpress_013_R_2013_10_17_10_21_46_user_SN2.2.Neonatal.Assay.LEx.2run.18.11.13_Auto_user_
##
##   [1]
##   [2]
##   [3]
##   [4]
##       Case_IonXpress_014_R_2013_10_17_10_21_46_user_SN2.2.Neonatal.Assay.LEx.2run.18.11.13_Auto_user_
##
##   [1]
##   [2]
##   [3]
##   [4]
##       Case_IonXpress_015_R_2013_10_17_10_21_46_user_SN2.2.Neonatal.Assay.LEx.2run.18.11.13_Auto_user_
##
##   [1]
##   [2]
##   [3]
##   [4]
##       Case_IonXpress_016_R_2013_10_17_10_21_46_user_SN2.2.Neonatal.Assay.LEx.2run.18.11.13_Auto_user_
##
##   [1]
##   [2]
```

```
##   [3]
##   [4]
##       Case_IonXpress_017_R_2013_10_17_10_21_46_user_SN2.2.Neonatal.Assay.LEx.2run.18.11.13_Auto_user_
##
##   [1]
##   [2]
##   [3]
##   [4]
##       Case_IonXpress_018_R_2013_10_17_10_21_46_user_SN2.2.Neonatal.Assay.LEx.2run.18.11.13_Auto_user_
##
##   [1]
##   [2]
##   [3]
##   [4]
##       Case_IonXpress_020_R_2013_10_17_10_21_46_user_SN2.2.Neonatal.Assay.LEx.2run.18.11.13_Auto_user_
##
##   [1]
##   [2]
##   [3]
##   [4]
##       Case_IonXpress_021_R_2013_10_17_10_21_46_user_SN2.2.Neonatal.Assay.LEx.2run.18.11.13_Auto_user_
##
##   [1]
##   [2]
##   [3]
##   [4]
##       Case_IonXpress_022_R_2013_10_17_10_21_46_user_SN2.2.Neonatal.Assay.LEx.2run.18.11.13_Auto_user_
##
##   [1]
##   [2]
##   [3]
##   [4]
##       Case_IonXpress_023_R_2013_10_17_10_21_46_user_SN2.2.Neonatal.Assay.LEx.2run.18.11.13_Auto_user_
##
##   [1]
##   [2]
##   [3]
##   [4]
##       Case_IonXpress_024_R_2013_10_17_10_21_46_user_SN2.2.Neonatal.Assay.LEx.2run.18.11.13_Auto_user_
##
##   [1]
##   [2]
##   [3]
##   [4]
##       Case_IonXpress_025_R_2013_10_17_10_21_46_user_SN2.2.Neonatal.Assay.LEx.2run.18.11.13_Auto_user_
##
##   [1]
##   [2]
##   [3]
##   [4]
##       Case_IonXpress_026_R_2013_10_17_10_21_46_user_SN2.2.Neonatal.Assay.LEx.2run.18.11.13_Auto_user_
##
##   [1]
##   [2]
```

```
## [3]
## [4]
##      Case_IonXpress_027_R_2013_10_17_10_21_46_user_SN2.2.Neonatal.Assay.LEx.2run.18.11.13_Auto_user_
##
## [1]
## [2]
## [3]
## [4]
##      Case_IonXpress_028_R_2013_10_17_10_21_46_user_SN2.2.Neonatal.Assay.LEx.2run.18.11.13_Auto_user_
##
## [1]
## [2]
## [3]
## [4]
##      Case_IonXpress_029_R_2013_10_17_10_21_46_user_SN2.2.Neonatal.Assay.LEx.2run.18.11.13_Auto_user_
##
## [1]
## [2]
## [3]
## [4]
##      Case_IonXpress_030_R_2013_10_17_10_21_46_user_SN2.2.Neonatal.Assay.LEx.2run.18.11.13_Auto_user_
##
## [1]
## [2]
## [3]
## [4]
##      Case_IonXpress_031_R_2013_10_17_10_21_46_user_SN2.2.Neonatal.Assay.LEx.2run.18.11.13_Auto_user_
##
## [1]
## [2]
## [3]
## [4]
##      Case_IonXpress_032_R_2013_10_17_10_21_46_user_SN2.2.Neonatal.Assay.LEx.2run.18.11.13_Auto_user_
##
## [1]
## [2]
## [3]
## [4]
##      Case_IonXpress_033_R_2013_10_17_10_21_46_user_SN2.2.Neonatal.Assay.LEx.2run.18.11.13_Auto_user_
##
## [1]
## [2]
## [3]
## [4]
##      Case_IonXpress_034_R_2013_10_17_10_21_46_user_SN2.2.Neonatal.Assay.LEx.2run.18.11.13_Auto_user_
##
## [1]
## [2]
## [3]
## [4]
##      Case_IonXpress_035_R_2013_10_17_10_21_46_user_SN2.2.Neonatal.Assay.LEx.2run.18.11.13_Auto_user_
##
## [1]
## [2]
```

```
##   [3]
##   [4]
##       Case_IonXpress_036_R_2013_10_17_10_21_46_user_SN2.2.Neonatal.Assay.LEx.2run.18.11.13_Auto_user_
##
##   [1]
##   [2]
##   [3]
##   [4]
##       Case_IonXpress_037_R_2013_10_17_10_21_46_user_SN2.2.Neonatal.Assay.LEx.2run.18.11.13_Auto_user_
##
##   [1]
##   [2]
##   [3]
##   [4]
##       Case_IonXpress_038_R_2013_10_17_10_21_46_user_SN2.2.Neonatal.Assay.LEx.2run.18.11.13_Auto_user_
##
##   [1]
##   [2]
##   [3]
##   [4]
##       Case_IonXpress_039_R_2013_10_17_10_21_46_user_SN2.2.Neonatal.Assay.LEx.2run.18.11.13_Auto_user_
##
##   [1]
##   [2]
##   [3]
##   [4]
##       Case_IonXpress_040_R_2013_10_17_10_21_46_user_SN2.2.Neonatal.Assay.LEx.2run.18.11.13_Auto_user_
##
##   [1]
##   [2]
##   [3]
##   [4]
##       Case_IonXpress_041_R_2013_10_17_10_21_46_user_SN2.2.Neonatal.Assay.LEx.2run.18.11.13_Auto_user_
##
##   [1]
##   [2]
##   [3]
##   [4]
##       Case_IonXpress_042_R_2013_10_17_10_21_46_user_SN2.2.Neonatal.Assay.LEx.2run.18.11.13_Auto_user_
##
##   [1]
##   [2]
##   [3]
##   [4]
##       Case_IonXpress_043_R_2013_10_17_10_21_46_user_SN2.2.Neonatal.Assay.LEx.2run.18.11.13_Auto_user_
##
##   [1]
##   [2]
##   [3]
##   [4]
##       Case_IonXpress_044_R_2013_10_17_10_21_46_user_SN2.2.Neonatal.Assay.LEx.2run.18.11.13_Auto_user_
##
##   [1]
##   [2]
```

```
##   [3]
##   [4]
##       Case_IonXpress_045_R_2013_10_17_10_21_46_user_SN2.2.Neonatal.Assay.LEx.2run.18.11.13_Auto_user_
##
##   [1]
##   [2]
##   [3]
##   [4]
##       Case_IonXpress_046_R_2013_10_17_10_21_46_user_SN2.2.Neonatal.Assay.LEx.2run.18.11.13_Auto_user_
##
##   [1]
##   [2]
##   [3]
##   [4]
##       Case_IonXpress_047_R_2013_10_17_10_21_46_user_SN2.2.Neonatal.Assay.LEx.2run.18.11.13_Auto_user_
##
##   [1]
##   [2]
##   [3]
##   [4]
##       Case_IonXpress_048_R_2013_10_17_10_21_46_user_SN2.2.Neonatal.Assay.LEx.2run.18.11.13_Auto_user_
##
##   [1]
##   [2]
##   [3]
##   [4]
##   -------
##   seqinfo: 1 sequence from an unspecified genome; no seqlengths
##
## Individual CNVs:
## GRanges object with 16 ranges and 4 metadata columns:
##        seqnames    ranges strand   |
##           <Rle> <IRanges>  <Rle>   |
##    [1]    undef  [18, 21]      *   |
##    [2]    undef  [71, 73]      *   |
##    [3]    undef  [48, 50]      *   |
##    [4]    undef  [48, 50]      *   |
##    [5]    undef  [71, 73]      *   |
##    ...      ...       ...    ... ...
##   [12]    undef  [48, 52]      *   |
##   [13]    undef  [71, 76]      *   |
##   [14]    undef  [48, 52]      *   |
##   [15]    undef  [48, 52]      *   |
##   [16]    undef  [72, 76]      *   |
##
##
##    [1] Case_IonXpress_002_R_2013_10_17_10_21_46_user_SN2.2.Neonatal.Assay.LEx.2run.18.11.13_Auto_user
##    [2] Case_IonXpress_011_R_2013_10_17_10_21_46_user_SN2.2.Neonatal.Assay.LEx.2run.18.11.13_Auto_user
##    [3] Case_IonXpress_012_R_2013_10_17_10_21_46_user_SN2.2.Neonatal.Assay.LEx.2run.18.11.13_Auto_user
##    [4] Case_IonXpress_013_R_2013_10_17_10_21_46_user_SN2.2.Neonatal.Assay.LEx.2run.18.11.13_Auto_user
##    [5] Case_IonXpress_013_R_2013_10_17_10_21_46_user_SN2.2.Neonatal.Assay.LEx.2run.18.11.13_Auto_user
##    ...
##   [12] Case_IonXpress_038_R_2013_10_17_10_21_46_user_SN2.2.Neonatal.Assay.LEx.2run.18.11.13_Auto_user
##   [13] Case_IonXpress_038_R_2013_10_17_10_21_46_user_SN2.2.Neonatal.Assay.LEx.2run.18.11.13_Auto_user
```

```
##   [14] Case_IonXpress_044_R_2013_10_17_10_21_46_user_SN2.2.Neonatal.Assay.LEx.2run.18.11.13_Auto_use
##   [15] Case_IonXpress_047_R_2013_10_17_10_21_46_user_SN2.2.Neonatal.Assay.LEx.2run.18.11.13_Auto_use
##   [16] Case_IonXpress_047_R_2013_10_17_10_21_46_user_SN2.2.Neonatal.Assay.LEx.2run.18.11.13_Auto_use
##           median      mean          CN
##        <numeric> <numeric> <character>
##    [1] 1.3219168 1.1435796         CN4
##    [2] 0.5196920 0.6366831         CN3
##    [3] 0.6111261 0.5274619         CN3
##    [4] 0.5947933 0.6473115         CN3
##    [5] 0.5264807 0.6787855         CN3
##    ...       ...       ...         ...
##   [12] 0.9998614 0.9133597         CN4
##   [13] 0.9660831 0.8297103         CN4
##   [14] 0.9968957 0.7985783         CN4
##   [15] 0.5887223 0.5999348         CN3
##   [16] 0.6412438 0.6831403         CN3
##   -------
##   seqinfo: 1 sequence from an unspecified genome; no seqlengths
## [1] "/Users/gdemidov/Downloads/doc/Run_SN2_3_fin_05_qc.xls"

## Normalizing...

## Starting local modeling, please be patient...

## Reference sequence:  undef

## Starting segmentation algorithm...

## Using "fastseg" for segmentation.

## [1] ""
## [1] "/Users/gdemidov/Downloads/doc/Run_SN2_3_fin_05_qc.xls"
## [1] ""

## Segplot might not work because of special characters in the sample names. Use only A-Z,a-z and 0-9!
##  There is a hidden function cn.mops:::.replaceNames that replaces the names in the "CNVDetectionResu
```

_10_21_46_user_SN2.2.Neonatal.Assay.LEx.2run.18.11.13_Auto_user_SN2.2.Neon

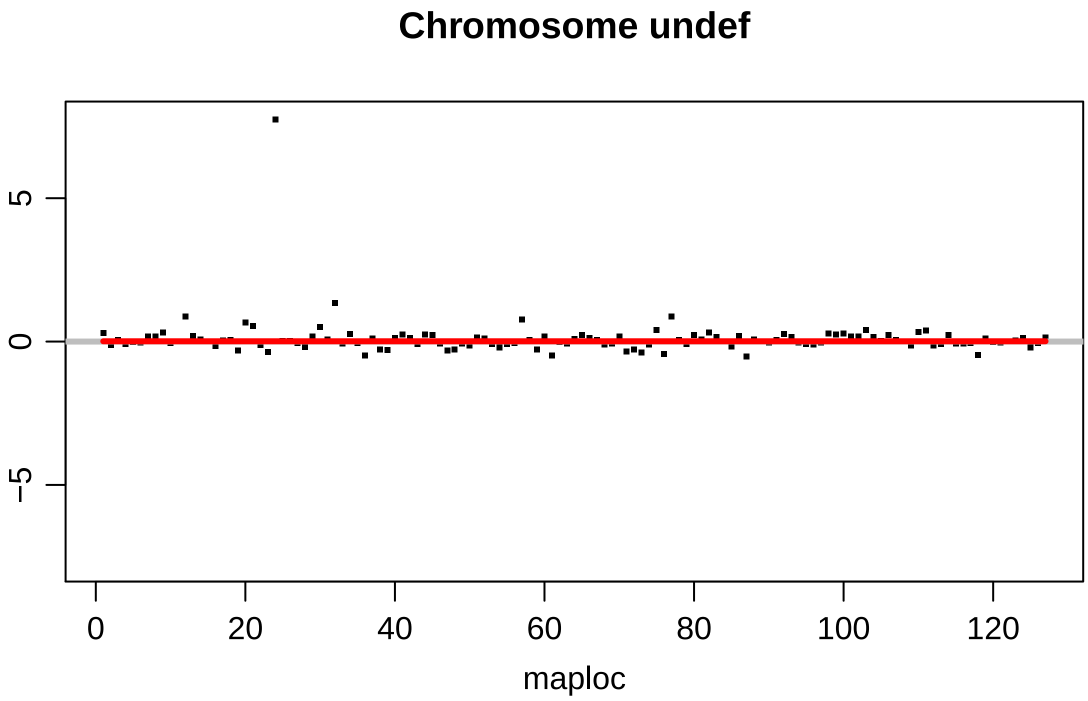


```
## Segplot might not work because of special characters in the sample names. Use only A-Z,a-z and 0-9!
##  There is a hidden function cn.mops:::.replaceNames that replaces the names in the "CNVDetectionResu
```

_55_39_user_SN2.3.Neonatal.Assay_Lex.1re.run.18.11.13_Auto_user_SN2.3.Neon

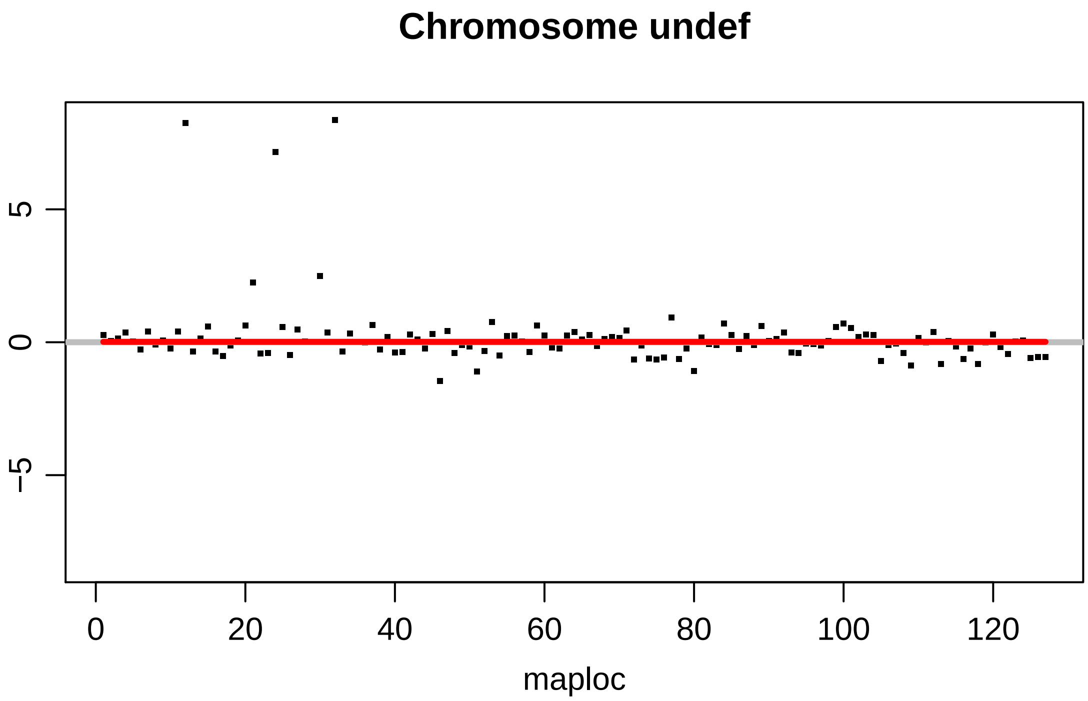

```
## Segplot might not work because of special characters in the sample names. Use only A-Z,a-z and 0-9!
##  There is a hidden function cn.mops:::.replaceNames that replaces the names in the "CNVDetectionResul
```

3_55_39_user_SN2.3.Neonatal.Assay_Lex.1re.run.18.11.13_Auto_user_SN2.3.Neona

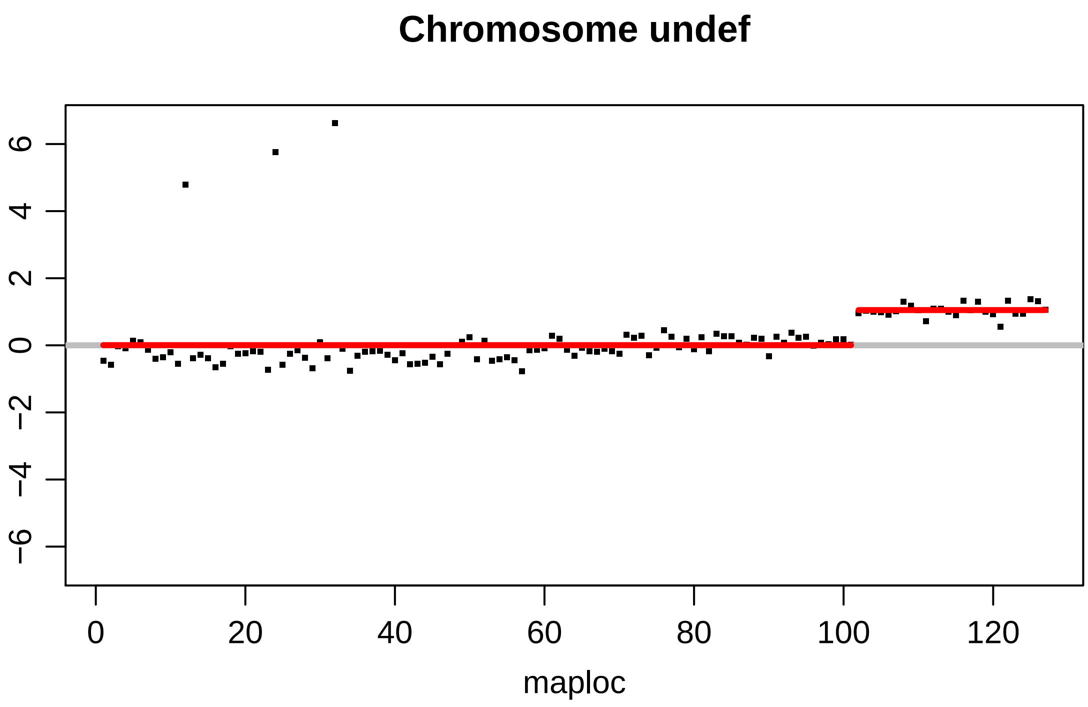


```
## Segplot might not work because of special characters in the sample names. Use only A-Z,a-z and 0-9!
##  There is a hidden function cn.mops:::.replaceNames that replaces the names in the "CNVDetectionResul
```

3_55_39_user_SN2.3.Neonatal.Assay_Lex.1re.run.18.11.13_Auto_user_SN2.3.Neona

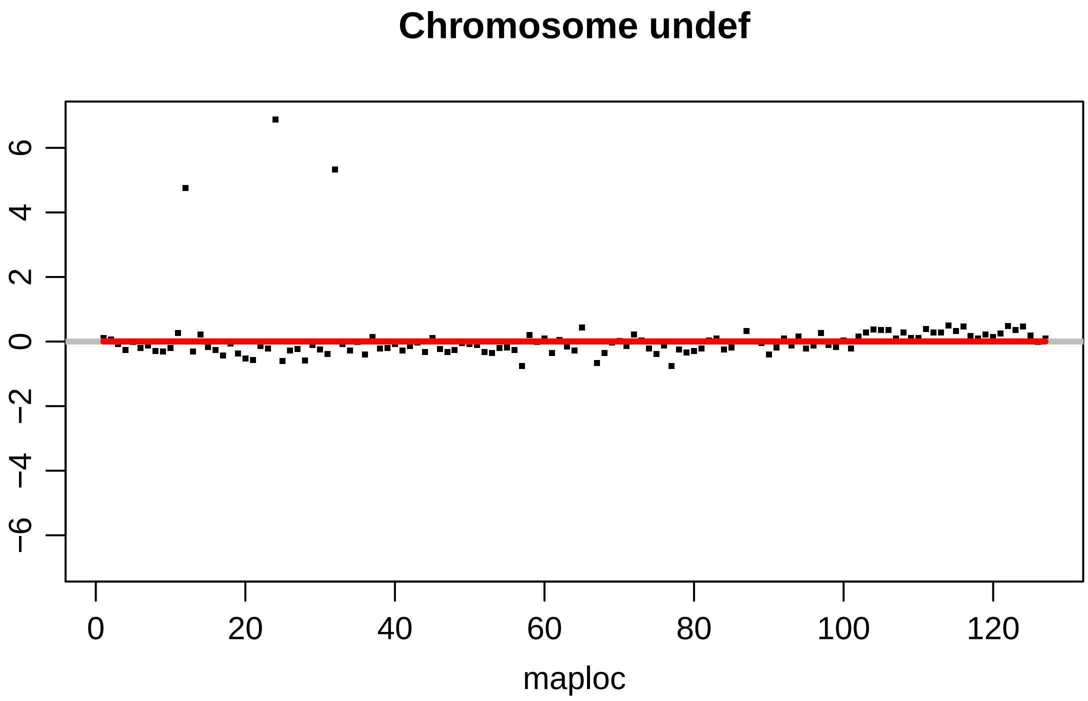


```
## Segplot might not work because of special characters in the sample names. Use only A-Z,a-z and 0-9!
##  There is a hidden function cn.mops:::.replaceNames that replaces the names in the "CNVDetectionResul
```

3_55_39_user_SN2.3.Neonatal.Assay_Lex.1re.run.18.11.13_Auto_user_SN2.3.Neona

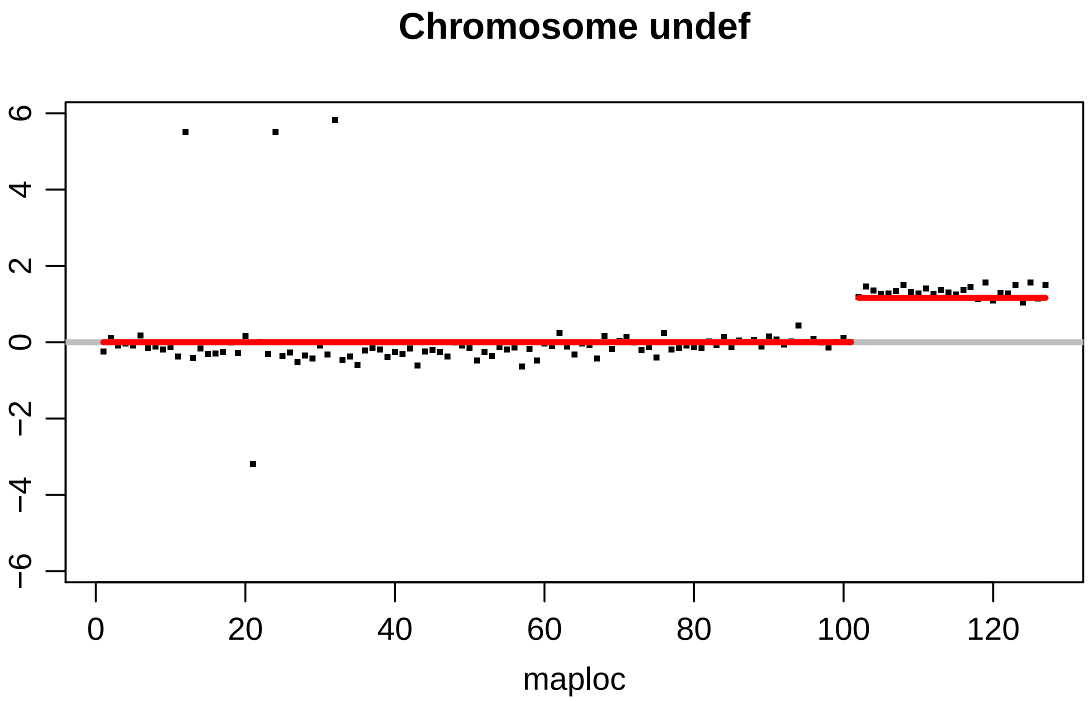

```
## Segplot might not work because of special characters in the sample names. Use only A-Z,a-z and 0-9!
##  There is a hidden function cn.mops:::.replaceNames that replaces the names in the "CNVDetectionResul
```

3_55_39_user_SN2.3.Neonatal.Assay_Lex.1re.run.18.11.13_Auto_user_SN2.3.Neona

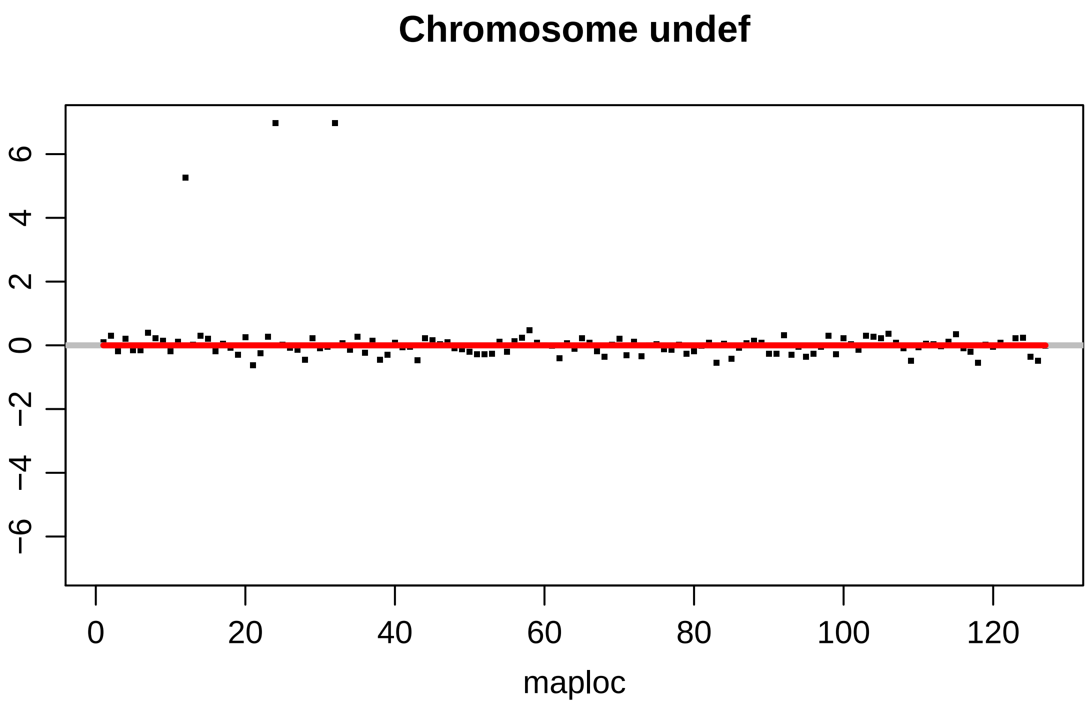


```
## Segplot might not work because of special characters in the sample names. Use only A-Z,a-z and 0-9!
##  There is a hidden function cn.mops:::.replaceNames that replaces the names in the "CNVDetectionResul
```

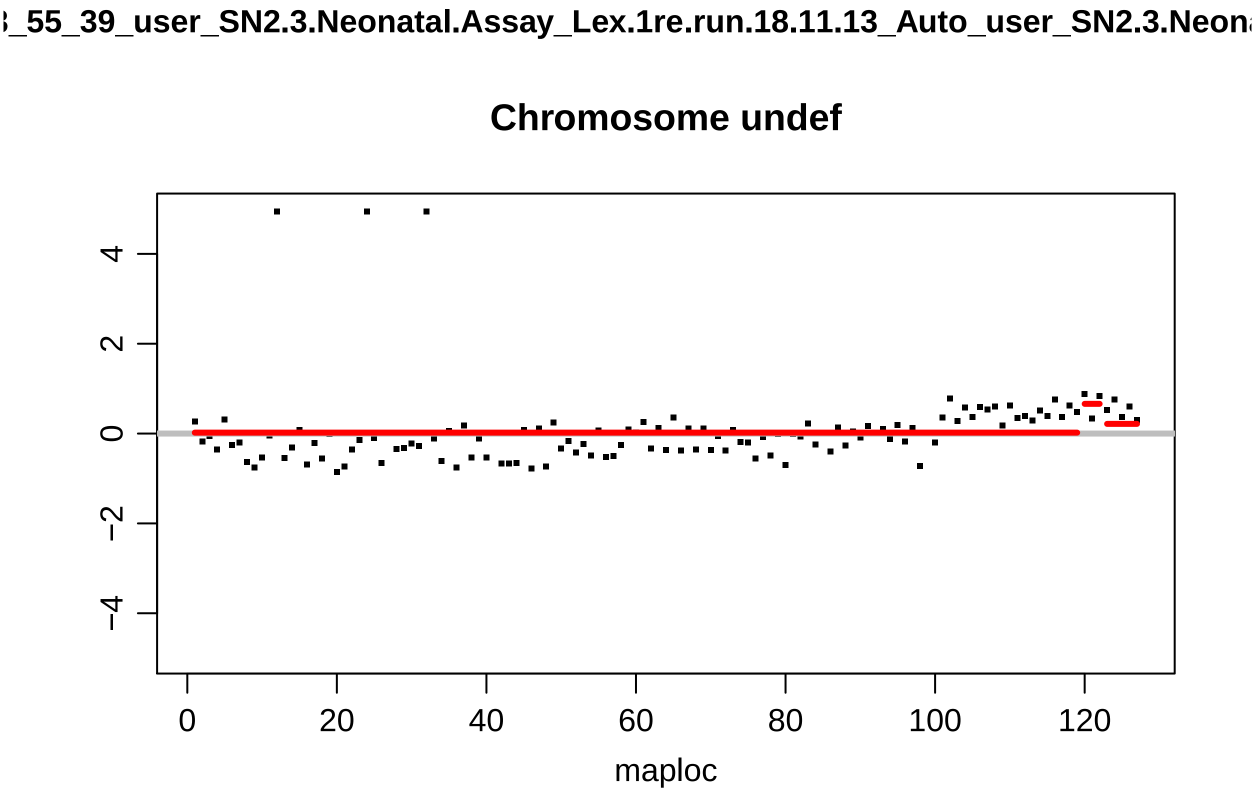

Segplot might not work because of special characters in the sample names. Use only A-Z,a-z and 0-9!
There is a hidden function cn.mops:::.replaceNames that replaces the names in the "CNVDetectionResul

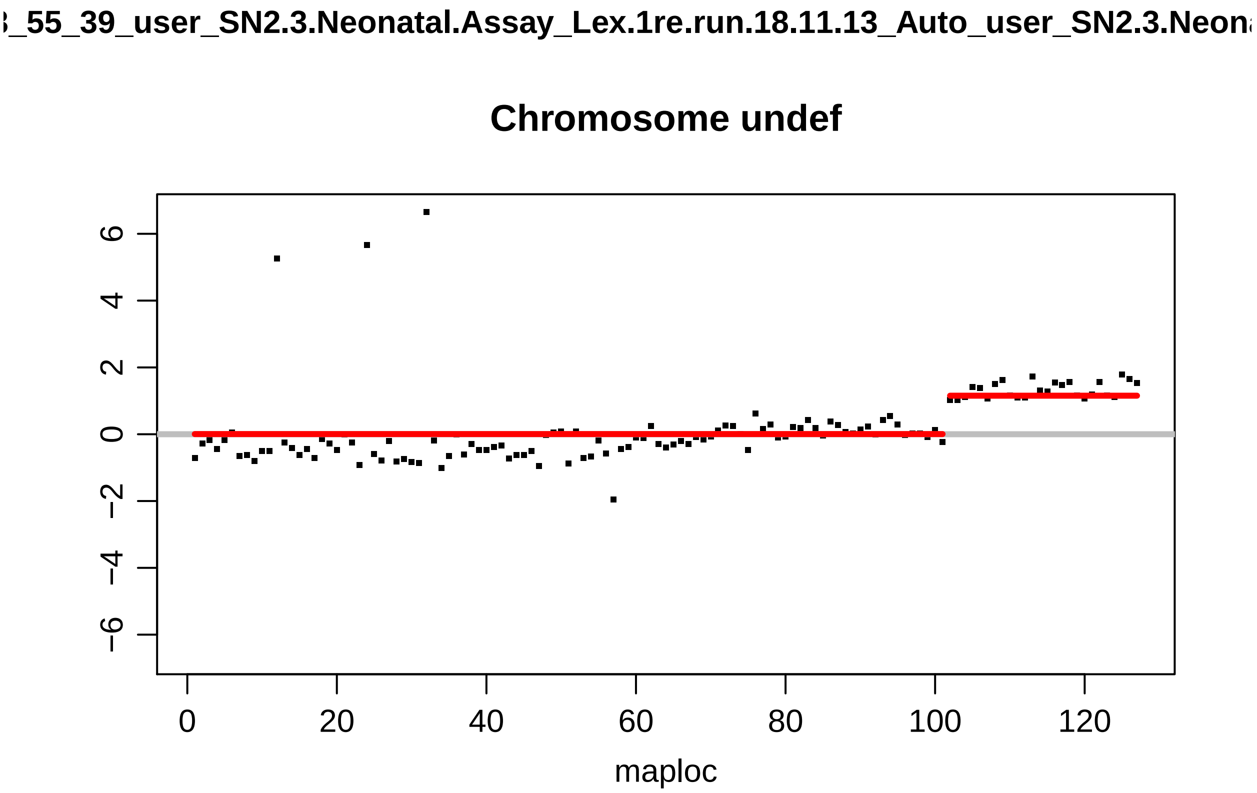

```
## Segplot might not work because of special characters in the sample names. Use only A-Z,a-z and 0-9!
##  There is a hidden function cn.mops:::.replaceNames that replaces the names in the "CNVDetectionResul
```

3_55_39_user_SN2.3.Neonatal.Assay_Lex.1re.run.18.11.13_Auto_user_SN2.3.Neona

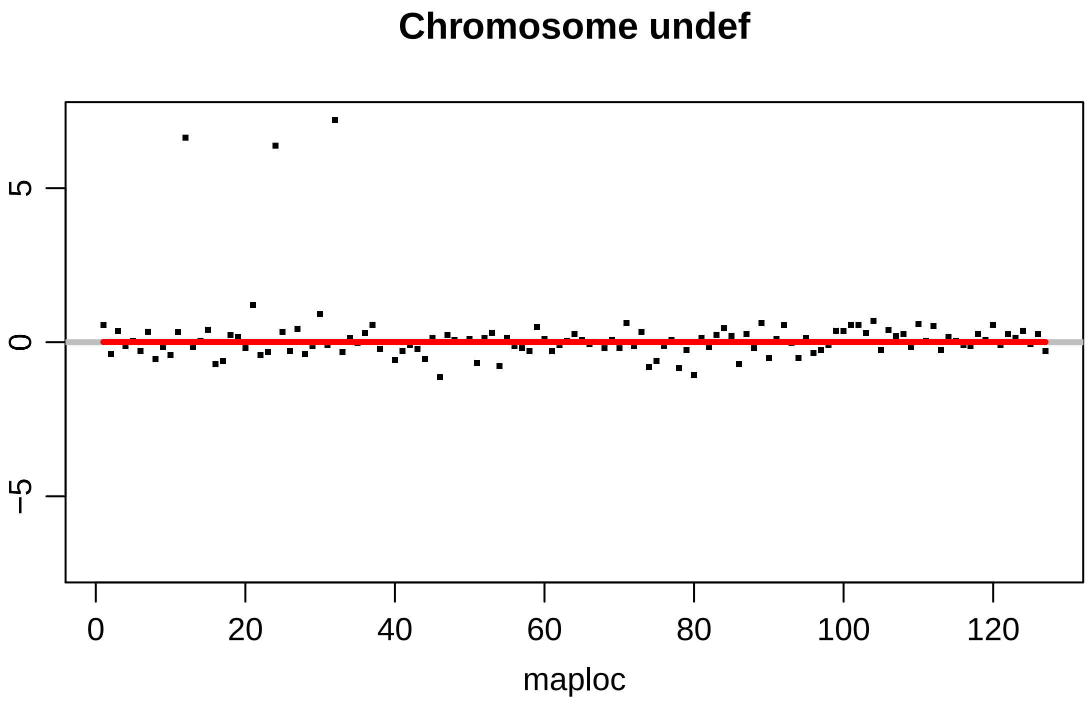


```
## Segplot might not work because of special characters in the sample names. Use only A-Z,a-z and 0-9!
##  There is a hidden function cn.mops:::.replaceNames that replaces the names in the "CNVDetectionResul
```

3_55_39_user_SN2.3.Neonatal.Assay_Lex.1re.run.18.11.13_Auto_user_SN2.3.Neona

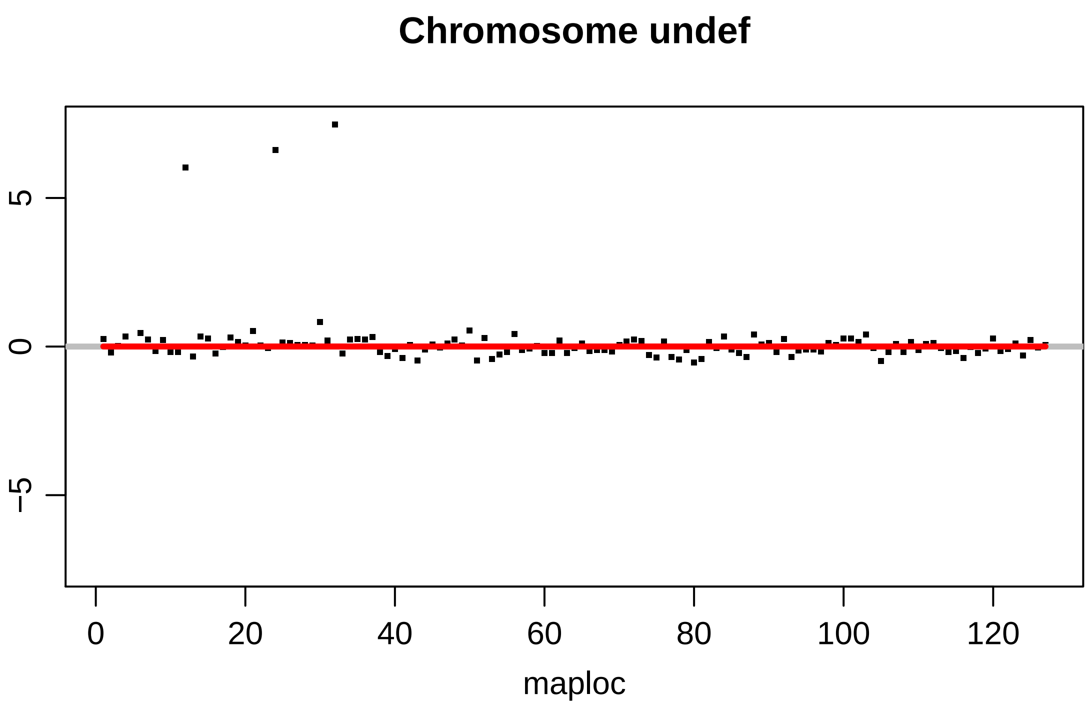


```
## Segplot might not work because of special characters in the sample names. Use only A-Z,a-z and 0-9!
##  There is a hidden function cn.mops:::.replaceNames that replaces the names in the "CNVDetectionResul
```

3_55_39_user_SN2.3.Neonatal.Assay_Lex.1re.run.18.11.13_Auto_user_SN2.3.Neona

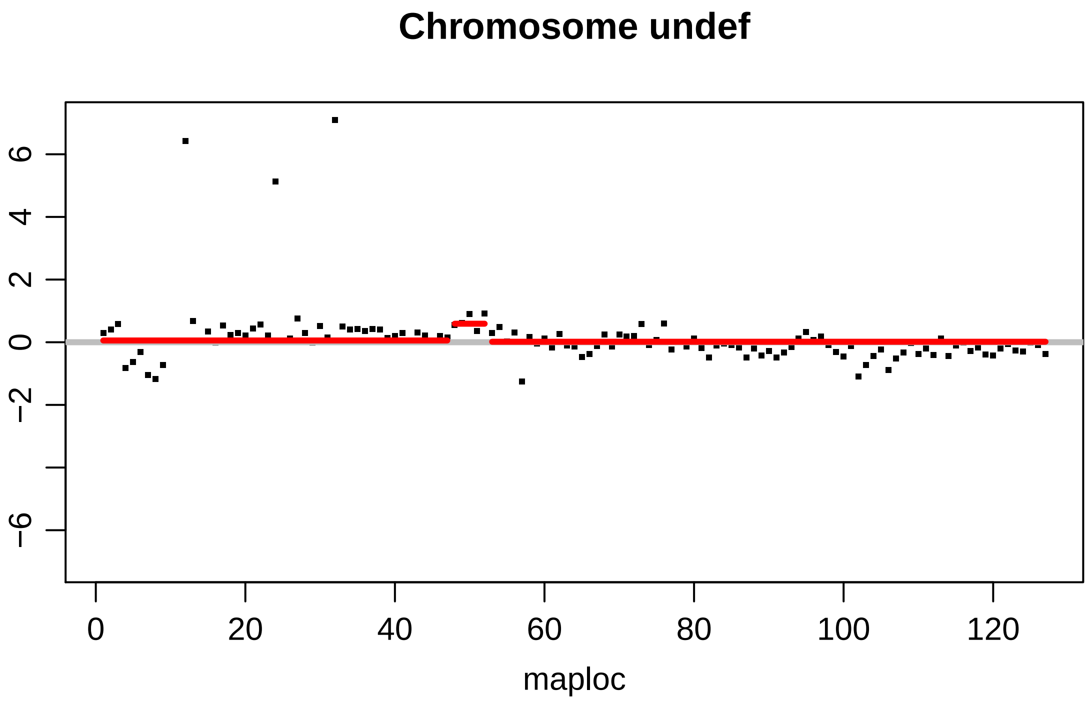

```
## Segplot might not work because of special characters in the sample names. Use only A-Z,a-z and 0-9!
##  There is a hidden function cn.mops:::.replaceNames that replaces the names in the "CNVDetectionResul
```

3_55_39_user_SN2.3.Neonatal.Assay_Lex.1re.run.18.11.13_Auto_user_SN2.3.Neona

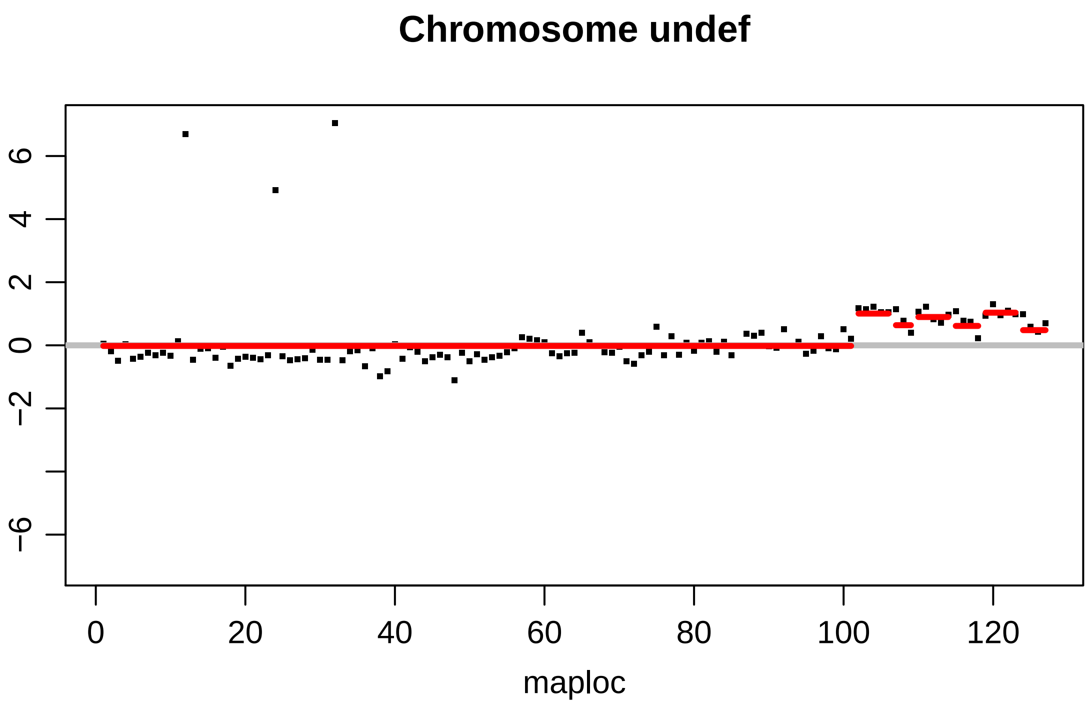


```
## Segplot might not work because of special characters in the sample names. Use only A-Z,a-z and 0-9!
##  There is a hidden function cn.mops:::.replaceNames that replaces the names in the "CNVDetectionResul
```

_55_39_user_SN2.3.Neonatal.Assay_Lex.1re.run.18.11.13_Auto_user_SN2.3.Neon

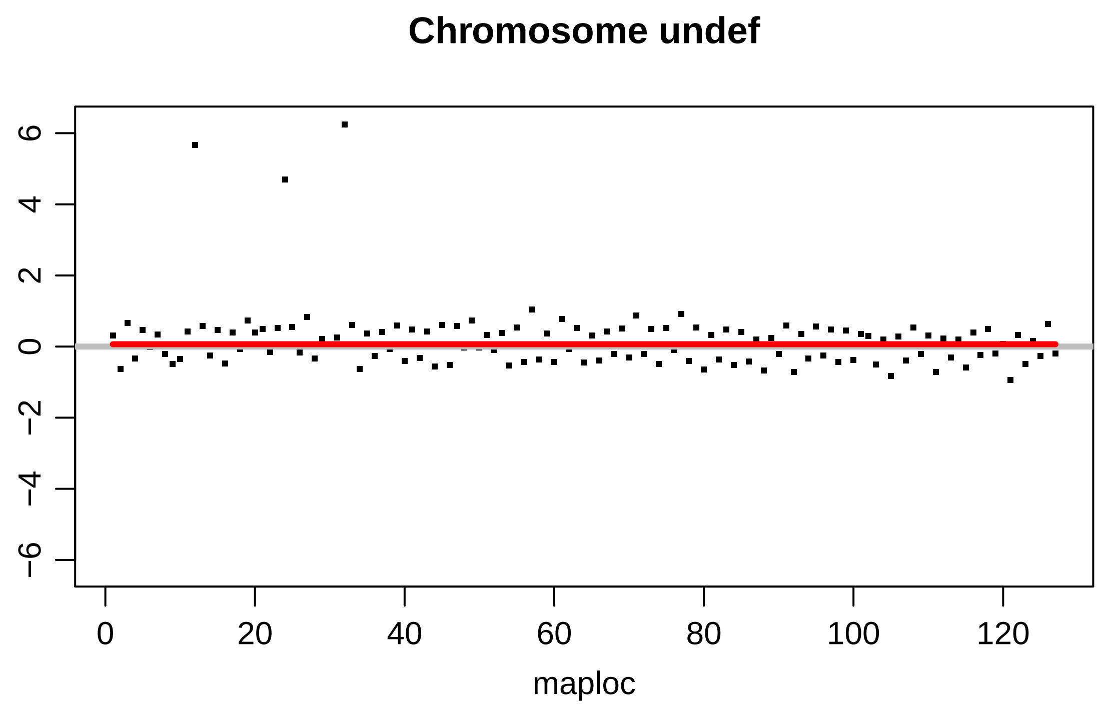


```
## Segplot might not work because of special characters in the sample names. Use only A-Z,a-z and 0-9!
##  There is a hidden function cn.mops:::.replaceNames that replaces the names in the "CNVDetectionResul
```

_55_39_user_SN2.3.Neonatal.Assay_Lex.1re.run.18.11.13_Auto_user_SN2.3.Neon

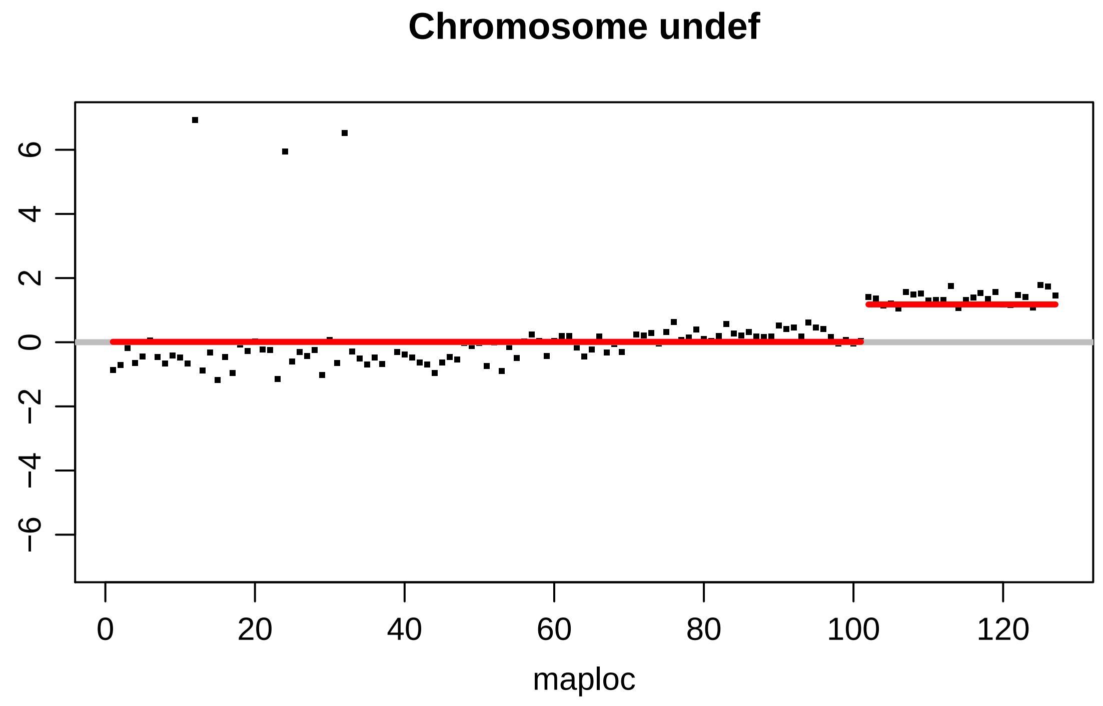

```
## Segplot might not work because of special characters in the sample names. Use only A-Z,a-z and 0-9!
##  There is a hidden function cn.mops:::.replaceNames that replaces the names in the "CNVDetectionResul
```

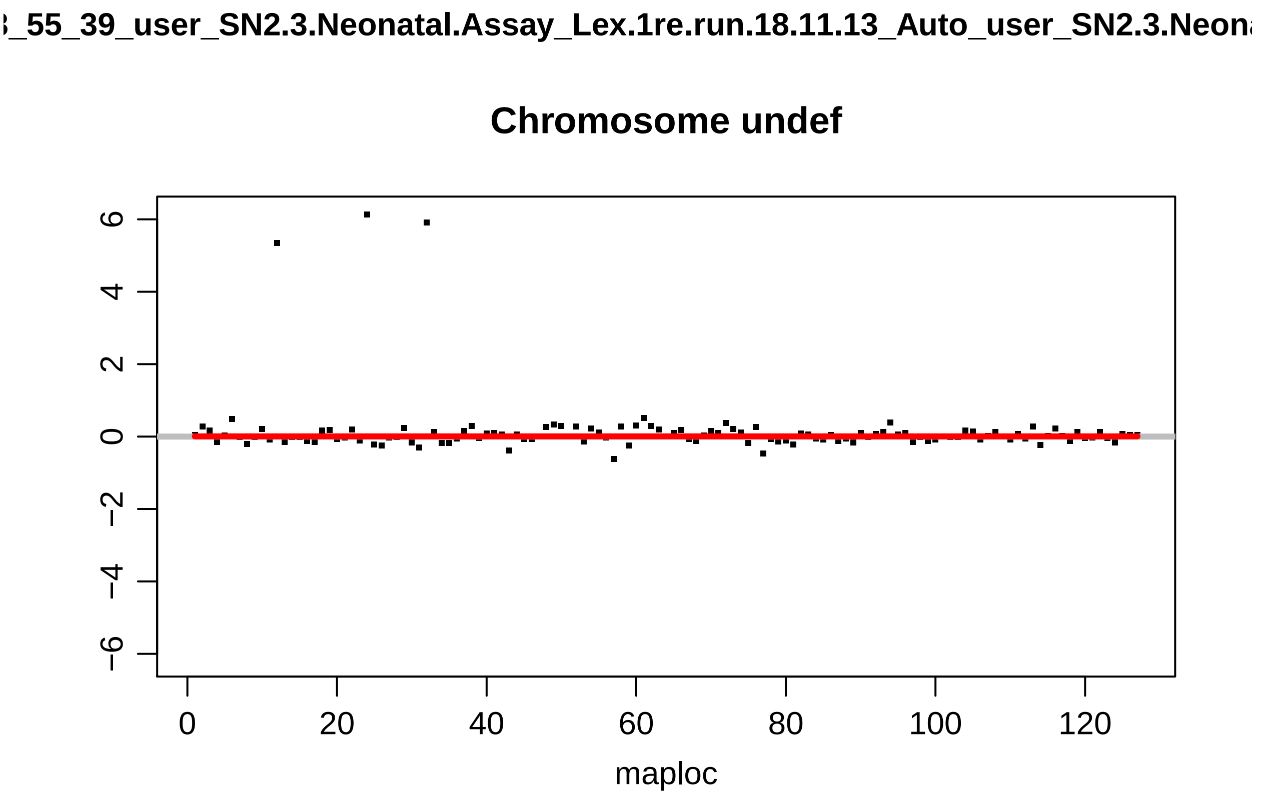


```
## Segplot might not work because of special characters in the sample names. Use only A-Z,a-z and 0-9!
##  There is a hidden function cn.mops:::.replaceNames that replaces the names in the "CNVDetectionResul
```

3_55_39_user_SN2.3.Neonatal.Assay_Lex.1re.run.18.11.13_Auto_user_SN2.3.Neona

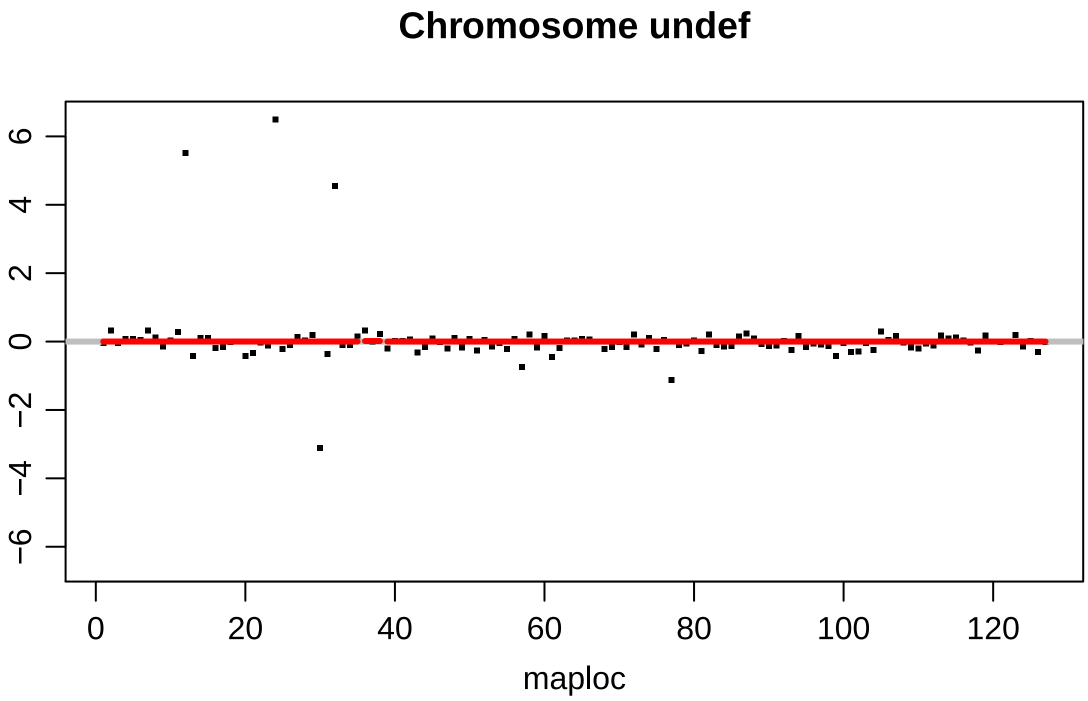


```
## Segplot might not work because of special characters in the sample names. Use only A-Z,a-z and 0-9!
##  There is a hidden function cn.mops:::.replaceNames that replaces the names in the "CNVDetectionResul
```

3_55_39_user_SN2.3.Neonatal.Assay_Lex.1re.run.18.11.13_Auto_user_SN2.3.Neona

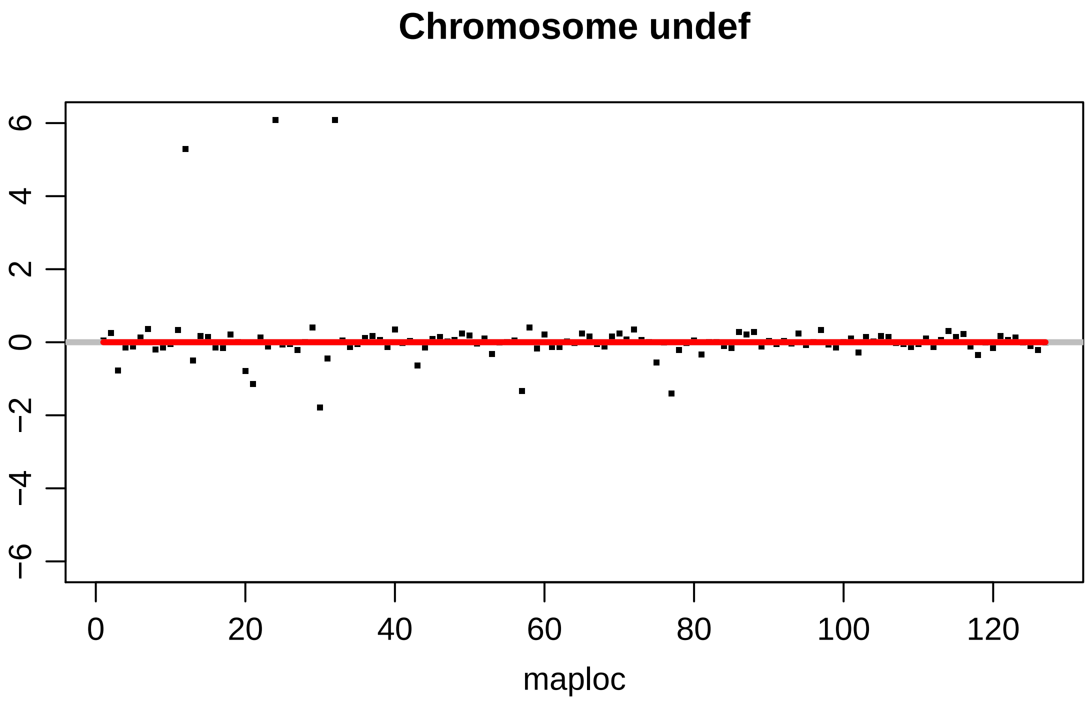

```
## Segplot might not work because of special characters in the sample names. Use only A-Z,a-z and 0-9!
##  There is a hidden function cn.mops:::.replaceNames that replaces the names in the "CNVDetectionResul
```

3_55_39_user_SN2.3.Neonatal.Assay_Lex.1re.run.18.11.13_Auto_user_SN2.3.Neona

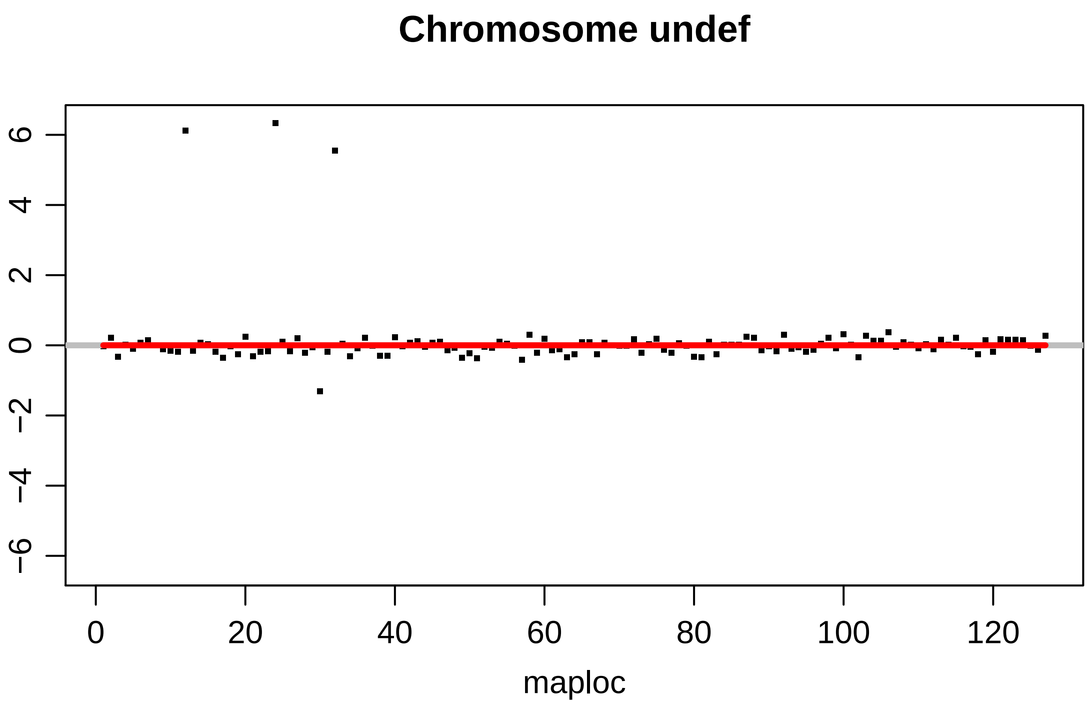


```
## Segplot might not work because of special characters in the sample names. Use only A-Z,a-z and 0-9!
##  There is a hidden function cn.mops:::.replaceNames that replaces the names in the "CNVDetectionResul
```

3_55_39_user_SN2.3.Neonatal.Assay_Lex.1re.run.18.11.13_Auto_user_SN2.3.Neona

Chromosome undef

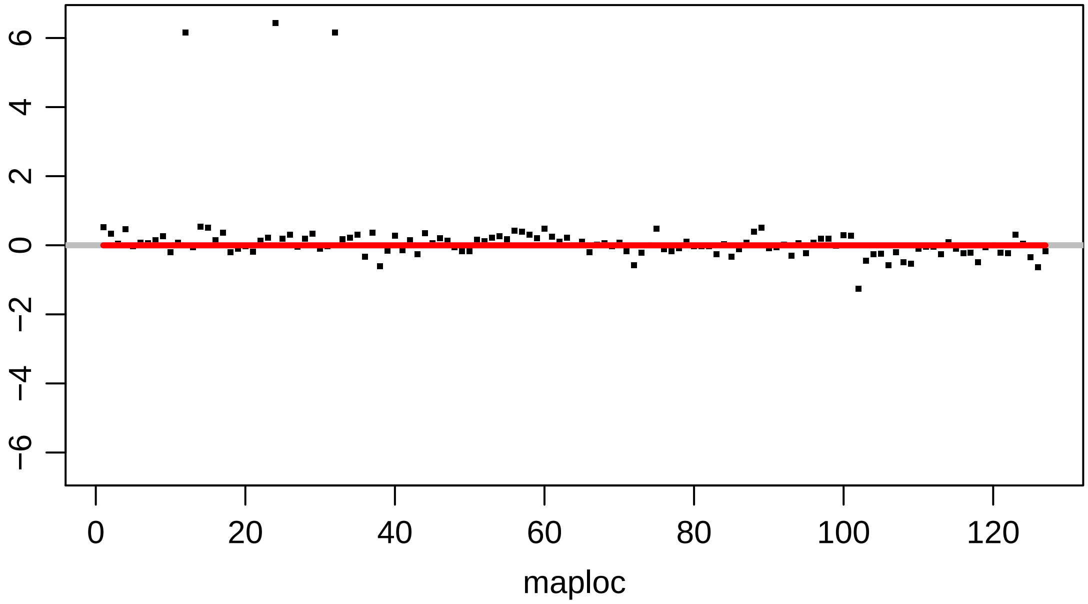

```
## Segplot might not work because of special characters in the sample names. Use only A-Z,a-z and 0-9!
##  There is a hidden function cn.mops:::.replaceNames that replaces the names in the "CNVDetectionResul
```

3_55_39_user_SN2.3.Neonatal.Assay_Lex.1re.run.18.11.13_Auto_user_SN2.3.Neona

Chromosome undef

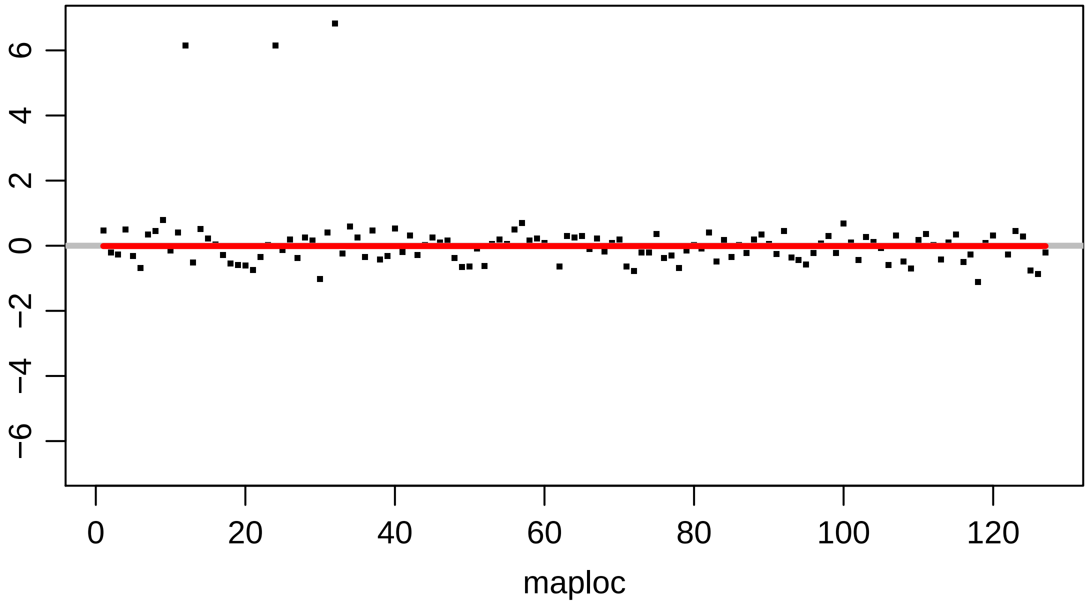

```
## Segplot might not work because of special characters in the sample names. Use only A-Z,a-z and 0-9!
##  There is a hidden function cn.mops:::.replaceNames that replaces the names in the "CNVDetectionResul
```

3_55_39_user_SN2.3.Neonatal.Assay_Lex.1re.run.18.11.13_Auto_user_SN2.3.Neona

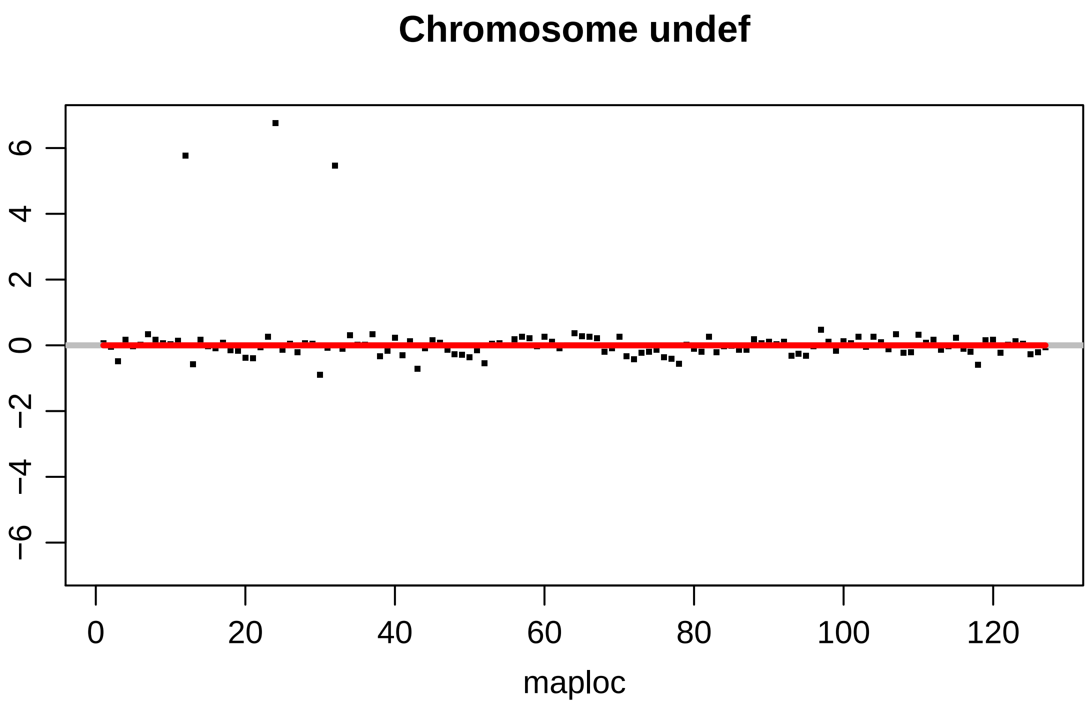


```
## Segplot might not work because of special characters in the sample names. Use only A-Z,a-z and 0-9!
##  There is a hidden function cn.mops:::.replaceNames that replaces the names in the "CNVDetectionResul
```

3_55_39_user_SN2.3.Neonatal.Assay_Lex.1re.run.18.11.13_Auto_user_SN2.3.Neona

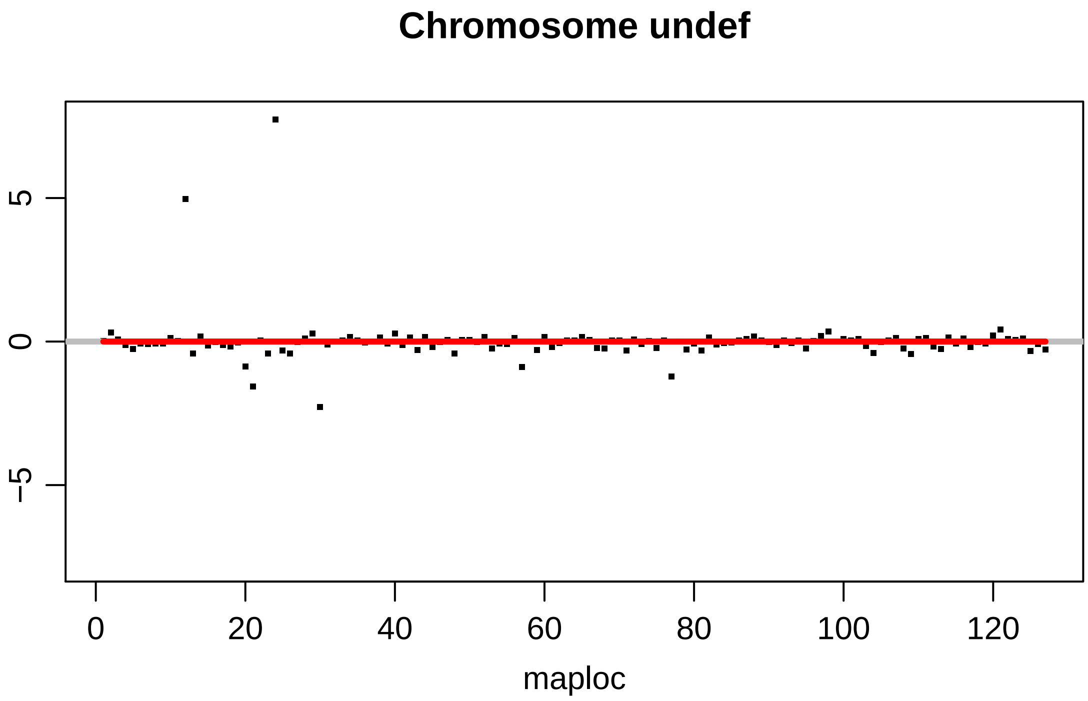


```
## Segplot might not work because of special characters in the sample names. Use only A-Z,a-z and 0-9!
##  There is a hidden function cn.mops:::.replaceNames that replaces the names in the "CNVDetectionResul
```

3_55_39_user_SN2.3.Neonatal.Assay_Lex.1re.run.18.11.13_Auto_user_SN2.3.Neona

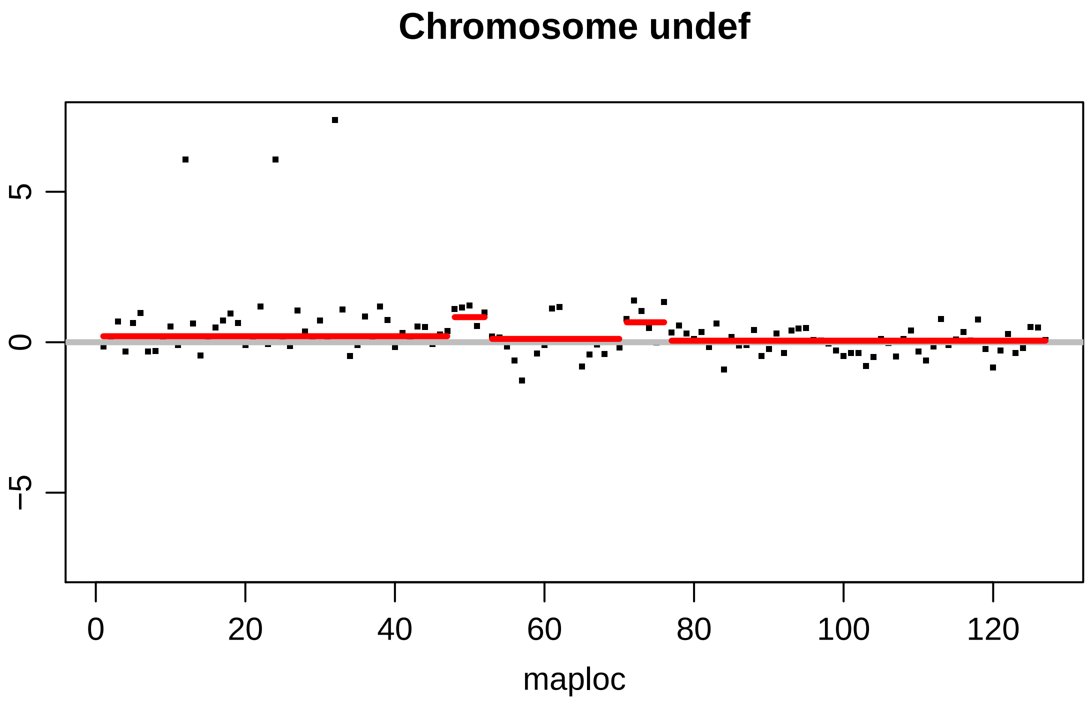

```
## Segplot might not work because of special characters in the sample names. Use only A-Z,a-z and 0-9!
##  There is a hidden function cn.mops:::.replaceNames that replaces the names in the "CNVDetectionResul
```

3_55_39_user_SN2.3.Neonatal.Assay_Lex.1re.run.18.11.13_Auto_user_SN2.3.Neona

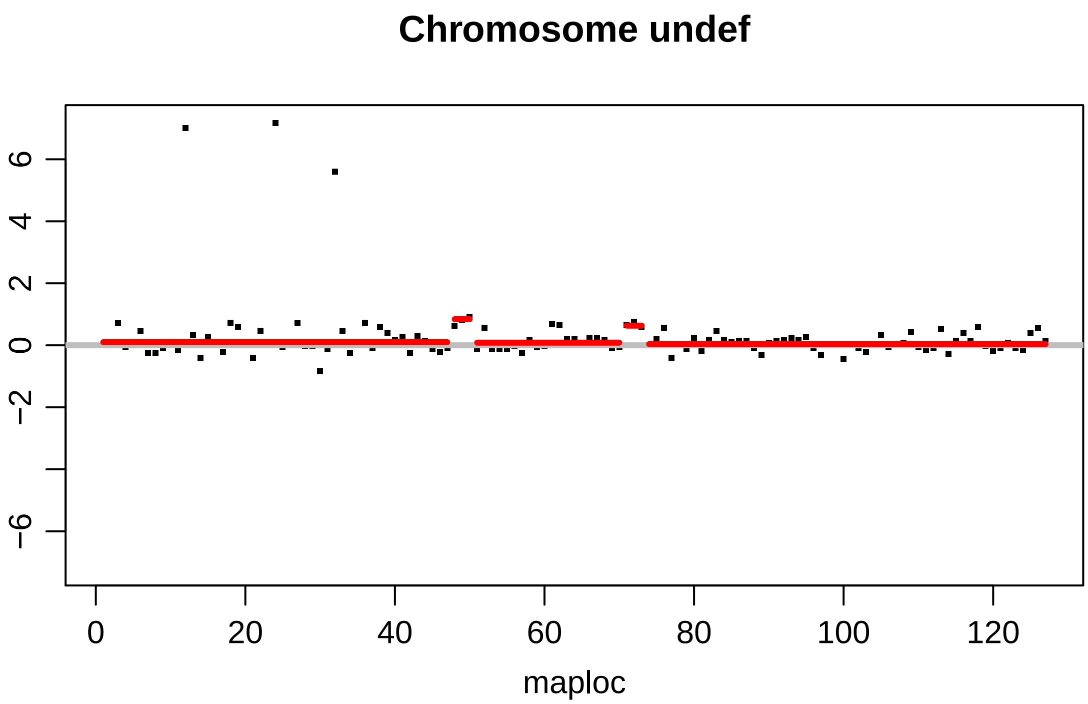


```
## Segplot might not work because of special characters in the sample names. Use only A-Z,a-z and 0-9!
##  There is a hidden function cn.mops:::.replaceNames that replaces the names in the "CNVDetectionResul
```

3_55_39_user_SN2.3.Neonatal.Assay_Lex.1re.run.18.11.13_Auto_user_SN2.3.Neona

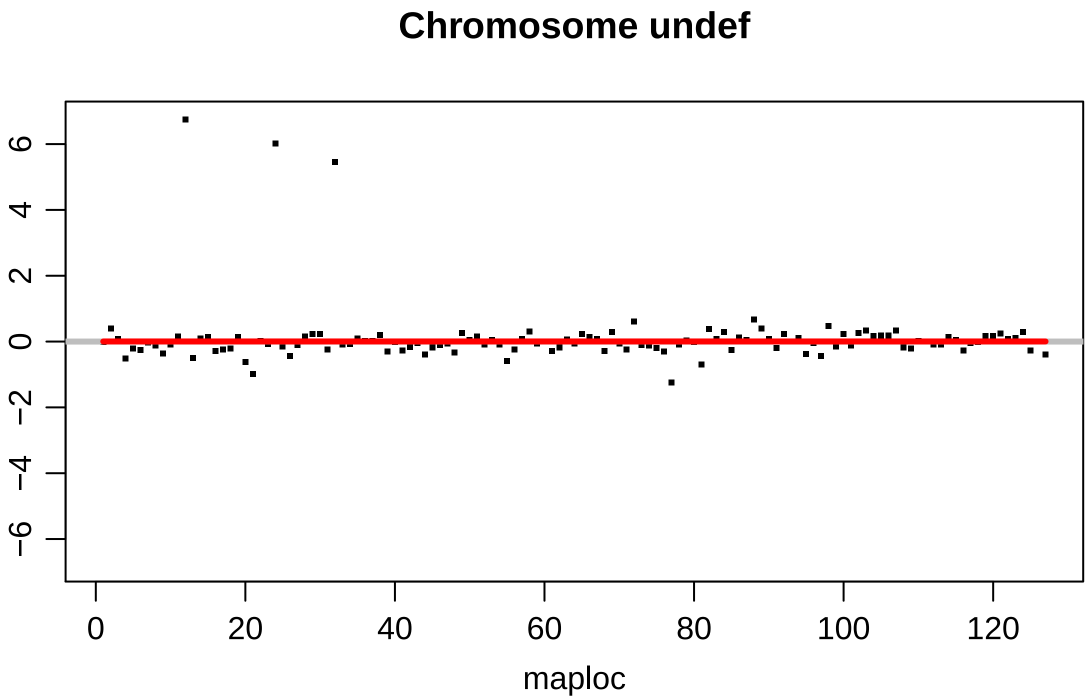


```
## Segplot might not work because of special characters in the sample names. Use only A-Z,a-z and 0-9!
##  There is a hidden function cn.mops:::.replaceNames that replaces the names in the "CNVDetectionResul
```

3_55_39_user_SN2.3.Neonatal.Assay_Lex.1re.run.18.11.13_Auto_user_SN2.3.Neona

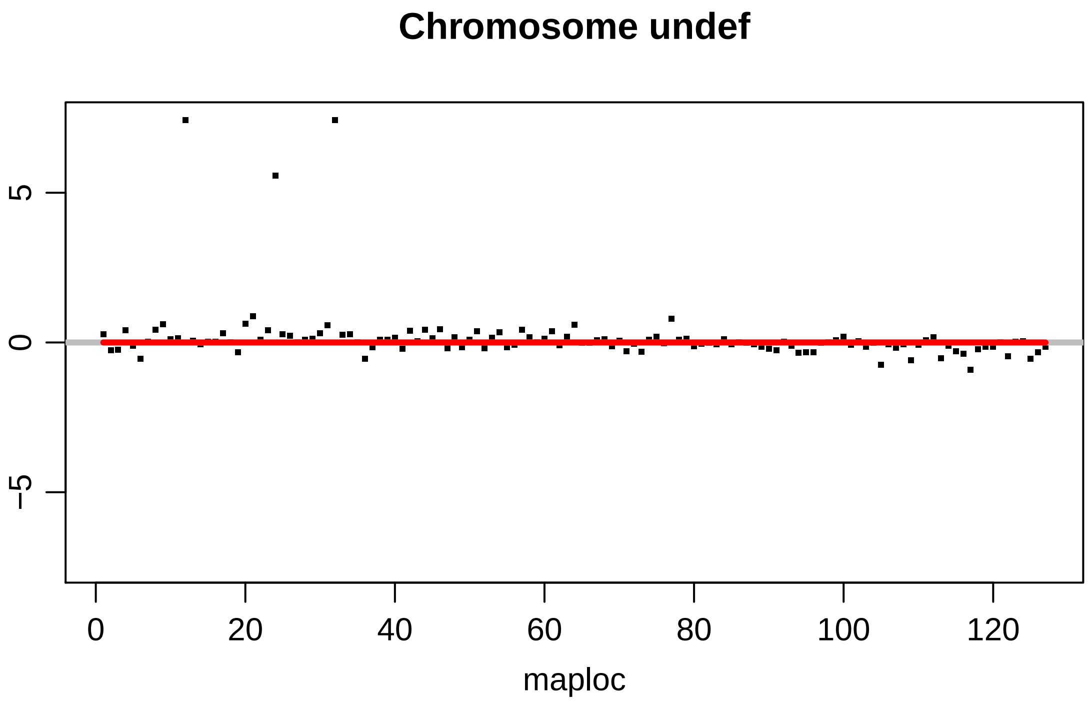

```
## Segplot might not work because of special characters in the sample names. Use only A-Z,a-z and 0-9!
##  There is a hidden function cn.mops:::.replaceNames that replaces the names in the "CNVDetectionResul
```

3_55_39_user_SN2.3.Neonatal.Assay_Lex.1re.run.18.11.13_Auto_user_SN2.3.Neona

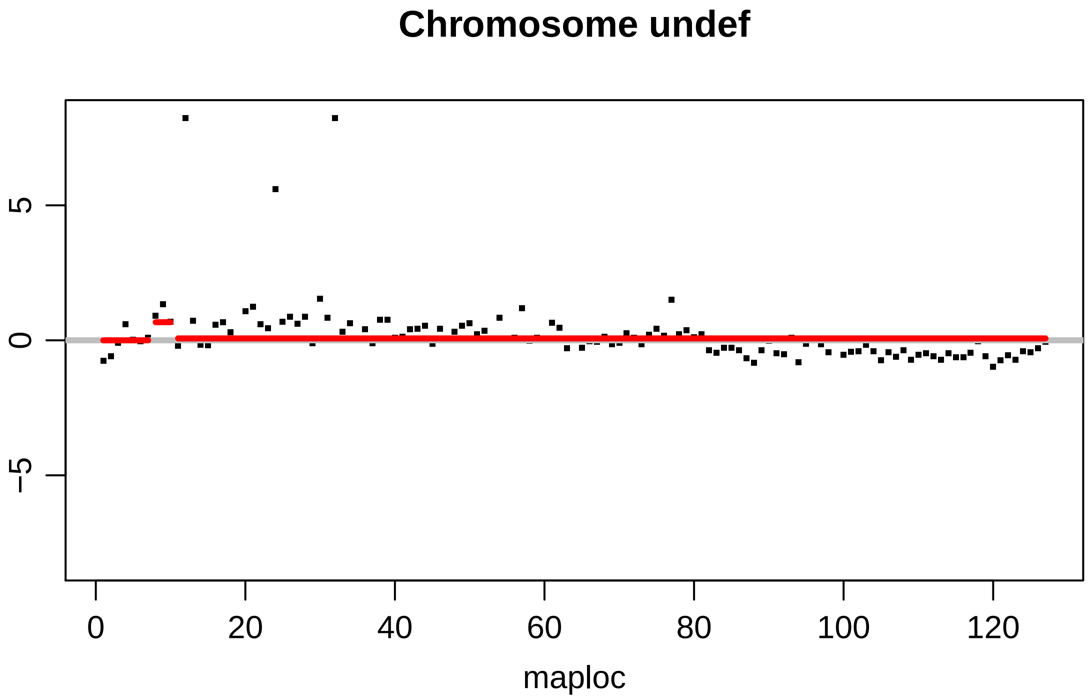


```
## Segplot might not work because of special characters in the sample names. Use only A-Z,a-z and 0-9!
##  There is a hidden function cn.mops:::.replaceNames that replaces the names in the "CNVDetectionResul
```

3_55_39_user_SN2.3.Neonatal.Assay_Lex.1re.run.18.11.13_Auto_user_SN2.3.Neona

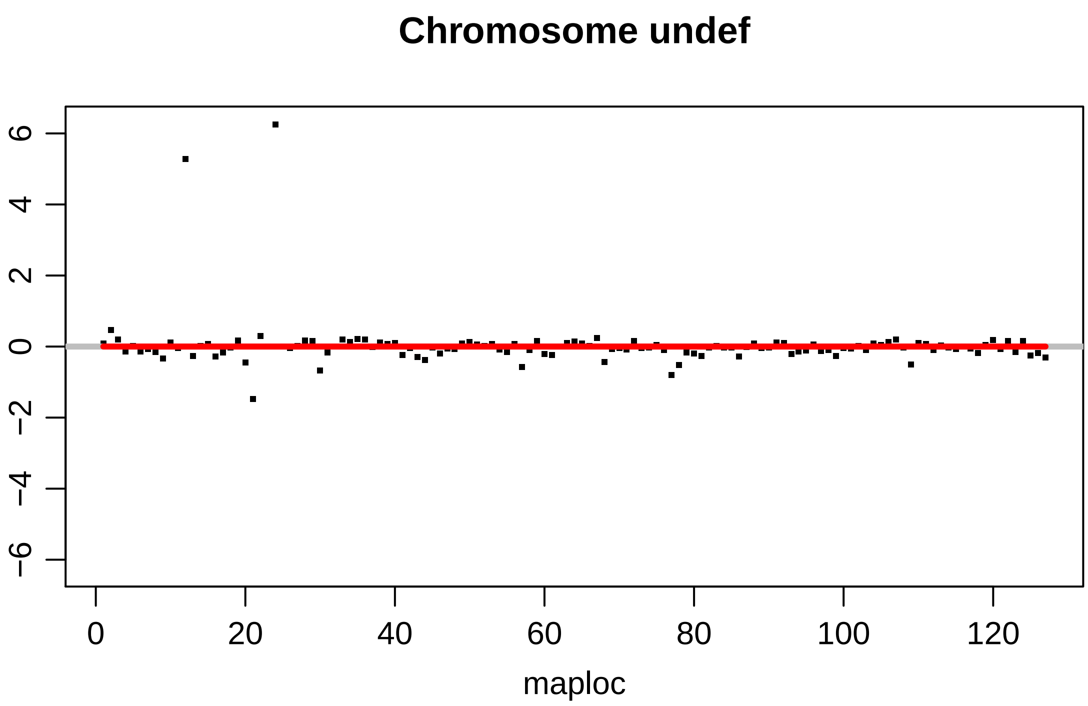


```
## Segplot might not work because of special characters in the sample names. Use only A-Z,a-z and 0-9!
##  There is a hidden function cn.mops:::.replaceNames that replaces the names in the "CNVDetectionResul
```

3_55_39_user_SN2.3.Neonatal.Assay_Lex.1re.run.18.11.13_Auto_user_SN2.3.Neona

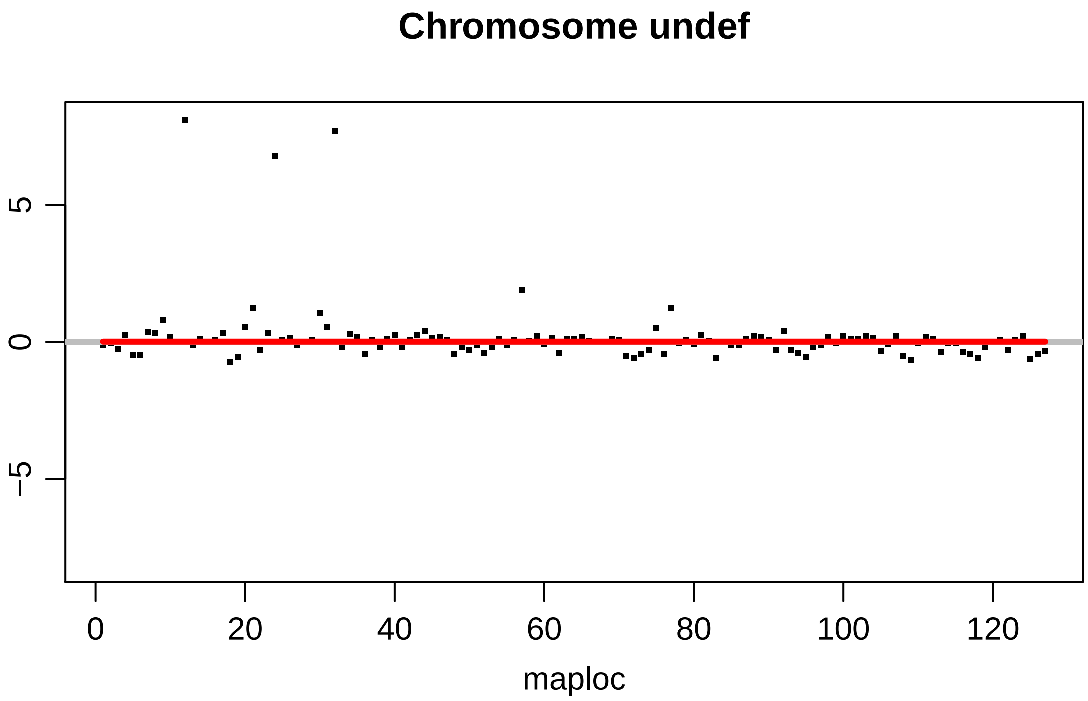

```
## Segplot might not work because of special characters in the sample names. Use only A-Z,a-z and 0-9!
##  There is a hidden function cn.mops:::.replaceNames that replaces the names in the "CNVDetectionResul
```

3_55_39_user_SN2.3.Neonatal.Assay_Lex.1re.run.18.11.13_Auto_user_SN2.3.Neona

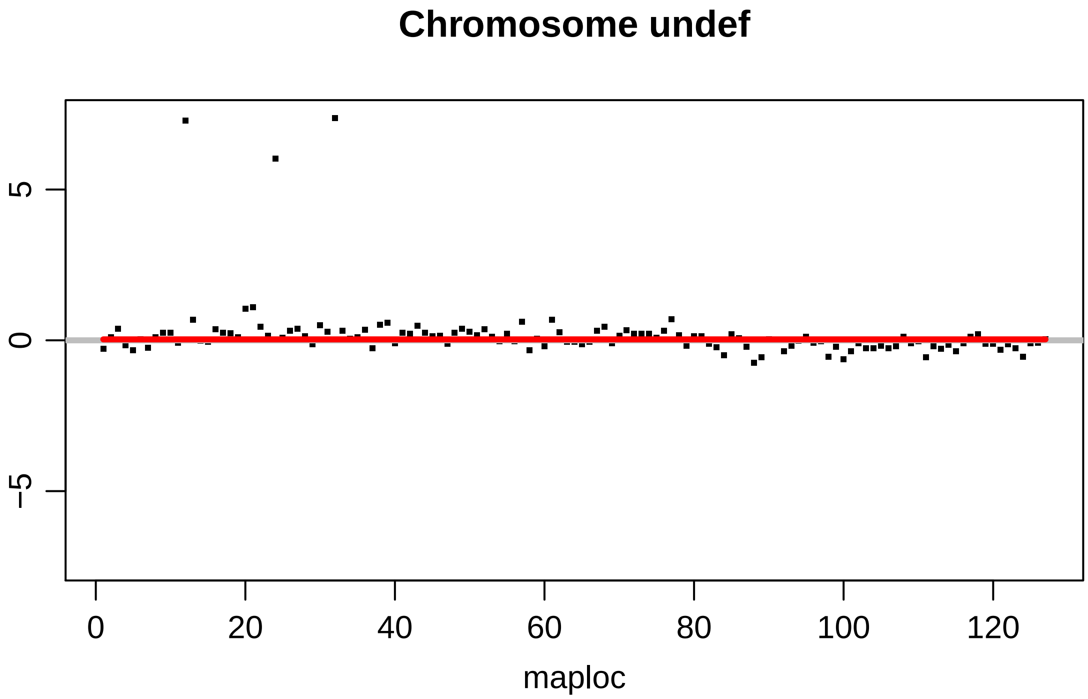


```
## Segplot might not work because of special characters in the sample names. Use only A-Z,a-z and 0-9!
##  There is a hidden function cn.mops:::.replaceNames that replaces the names in the "CNVDetectionResul
```

3_55_39_user_SN2.3.Neonatal.Assay_Lex.1re.run.18.11.13_Auto_user_SN2.3.Neona

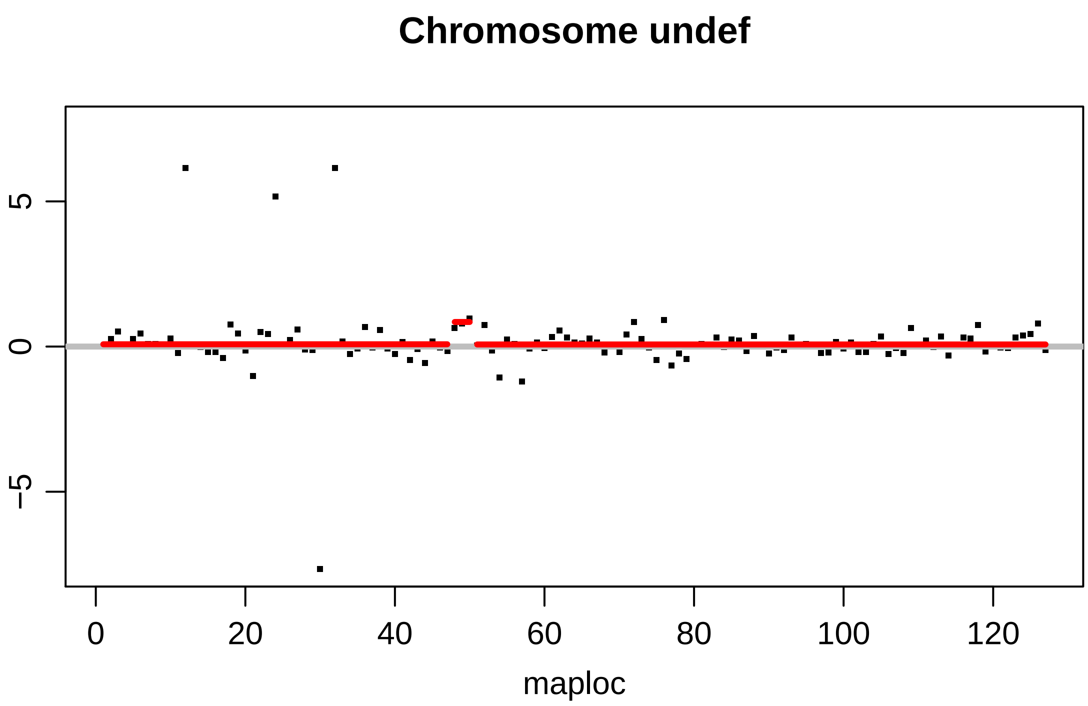


```
## Segplot might not work because of special characters in the sample names. Use only A-Z,a-z and 0-9!
##  There is a hidden function cn.mops:::.replaceNames that replaces the names in the "CNVDetectionResul
```

3_55_39_user_SN2.3.Neonatal.Assay_Lex.1re.run.18.11.13_Auto_user_SN2.3.Neona

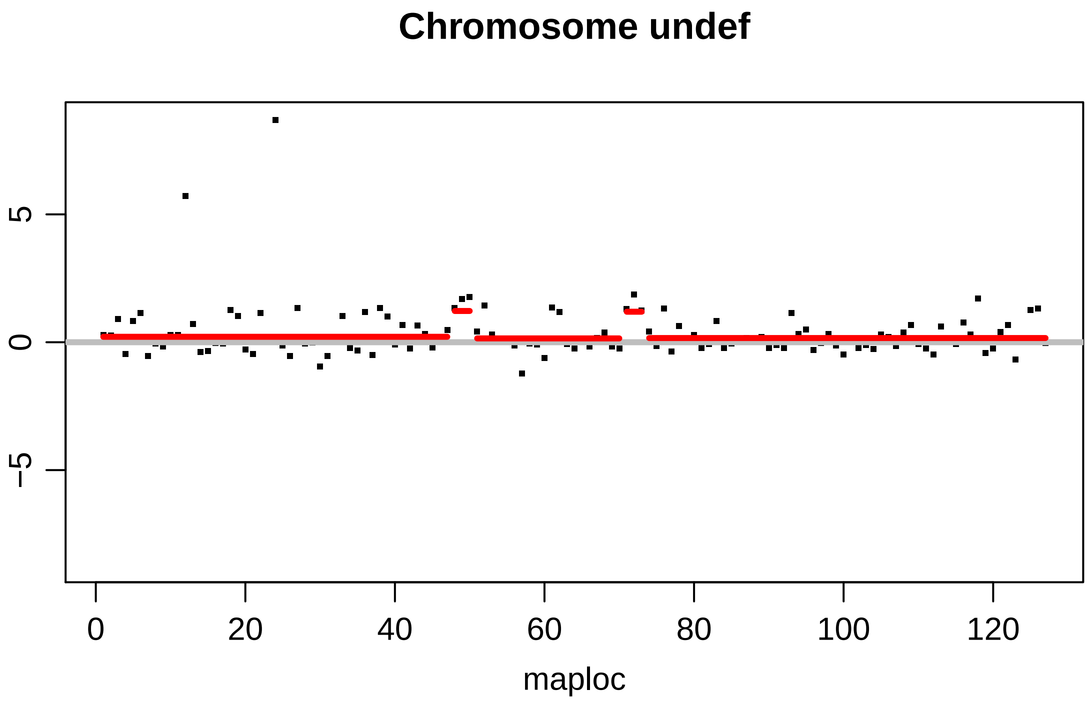

```
## Segplot might not work because of special characters in the sample names. Use only A-Z,a-z and 0-9!
##  There is a hidden function cn.mops:::.replaceNames that replaces the names in the "CNVDetectionResul
```

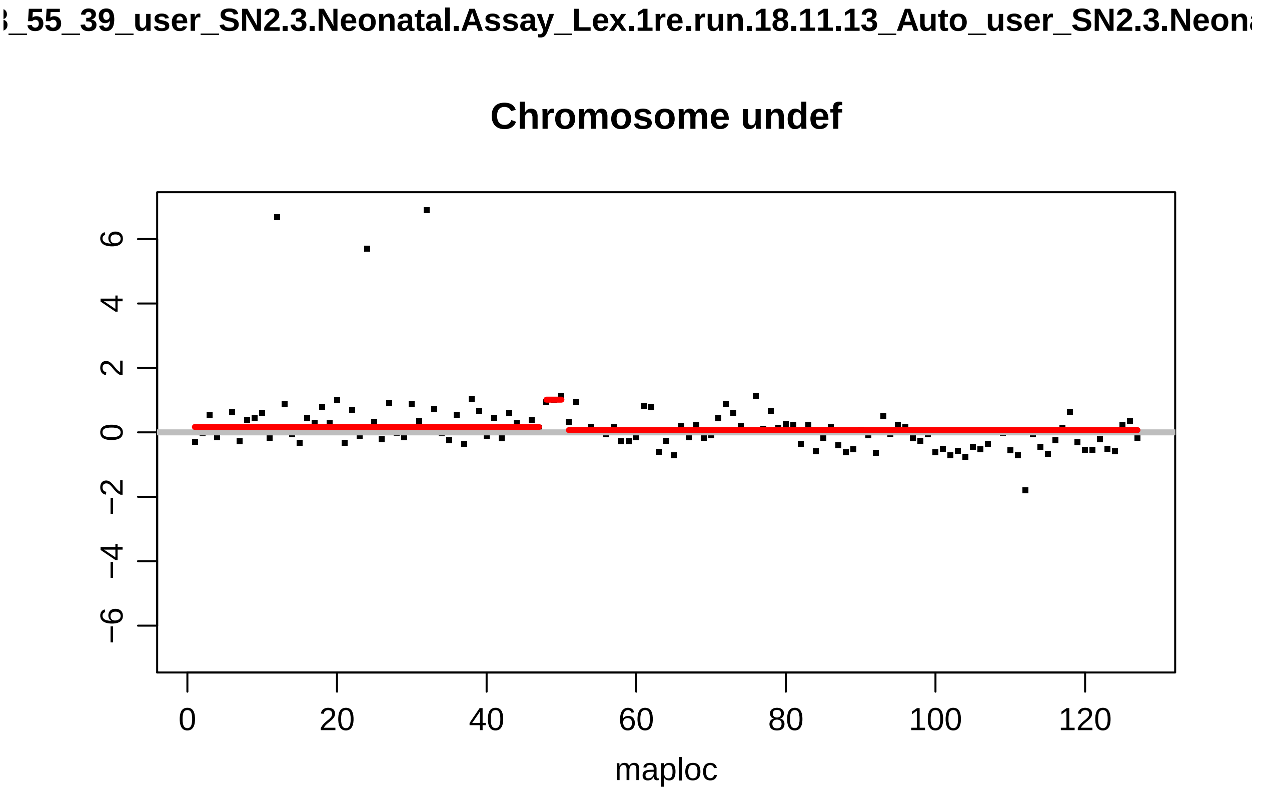


```
## Segplot might not work because of special characters in the sample names. Use only A-Z,a-z and 0-9!
##  There is a hidden function cn.mops:::.replaceNames that replaces the names in the "CNVDetectionResul
```

3_55_39_user_SN2.3.Neonatal.Assay_Lex.1re.run.18.11.13_Auto_user_SN2.3.Neon

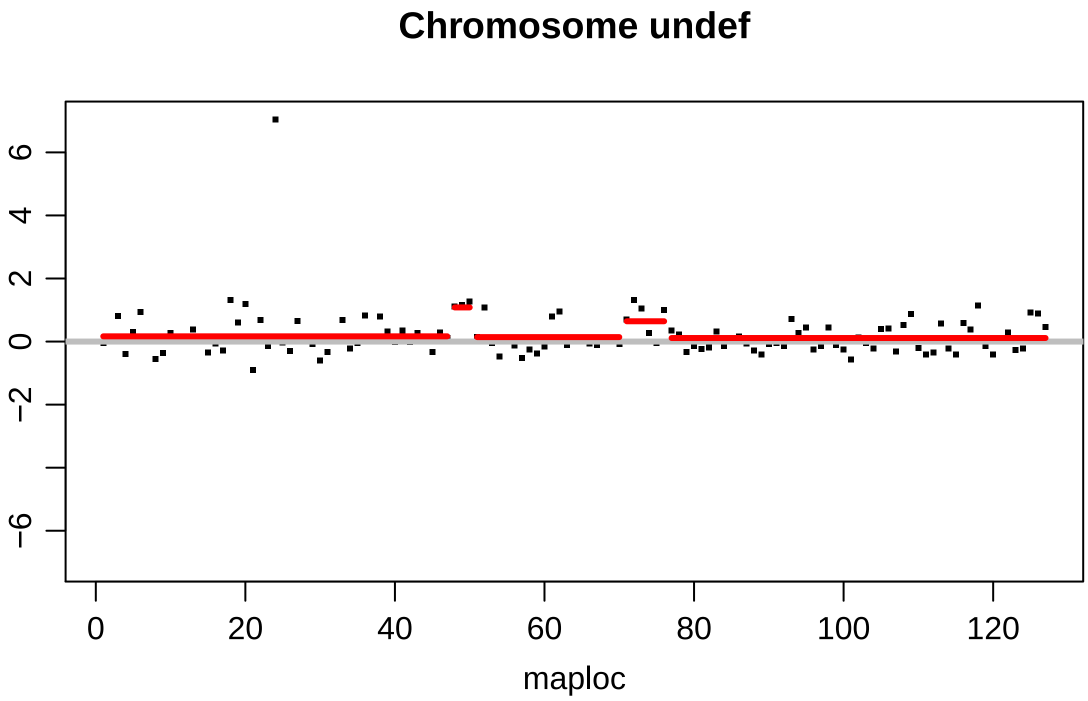


```
## Segplot might not work because of special characters in the sample names. Use only A-Z,a-z and 0-9!
##  There is a hidden function cn.mops:::.replaceNames that replaces the names in the "CNVDetectionResul
```

3_55_39_user_SN2.3.Neonatal.Assay_Lex.1re.run.18.11.13_Auto_user_SN2.3.Neon

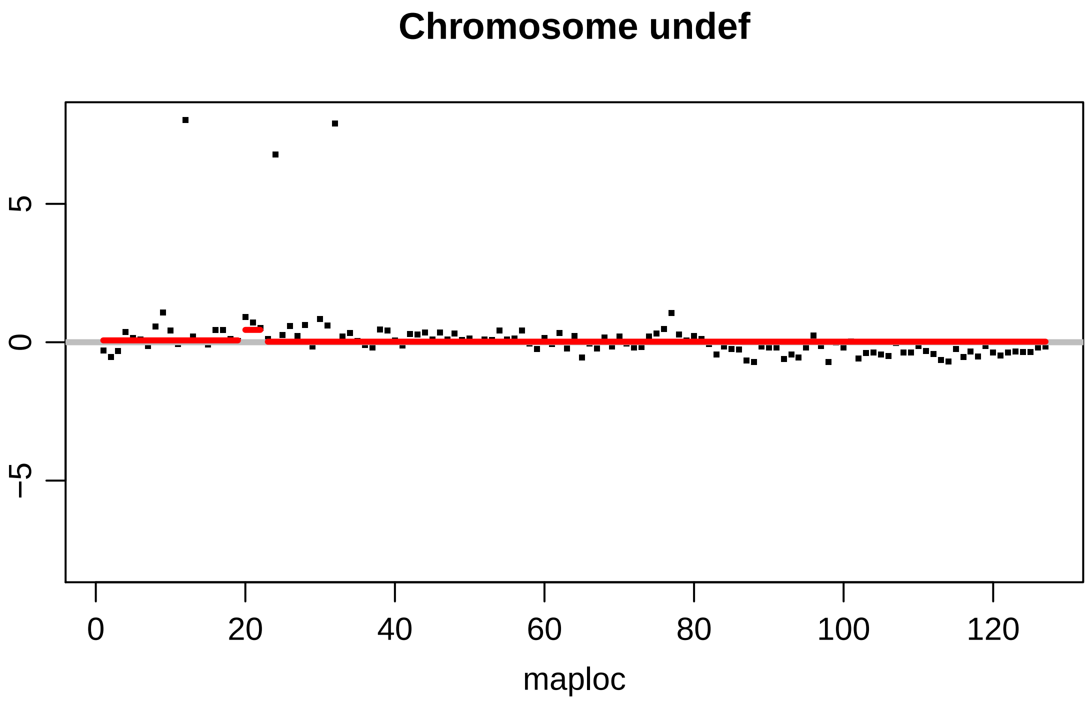

```
## Segplot might not work because of special characters in the sample names. Use only A-Z,a-z and 0-9!
##  There is a hidden function cn.mops:::.replaceNames that replaces the names in the "CNVDetectionResul
```

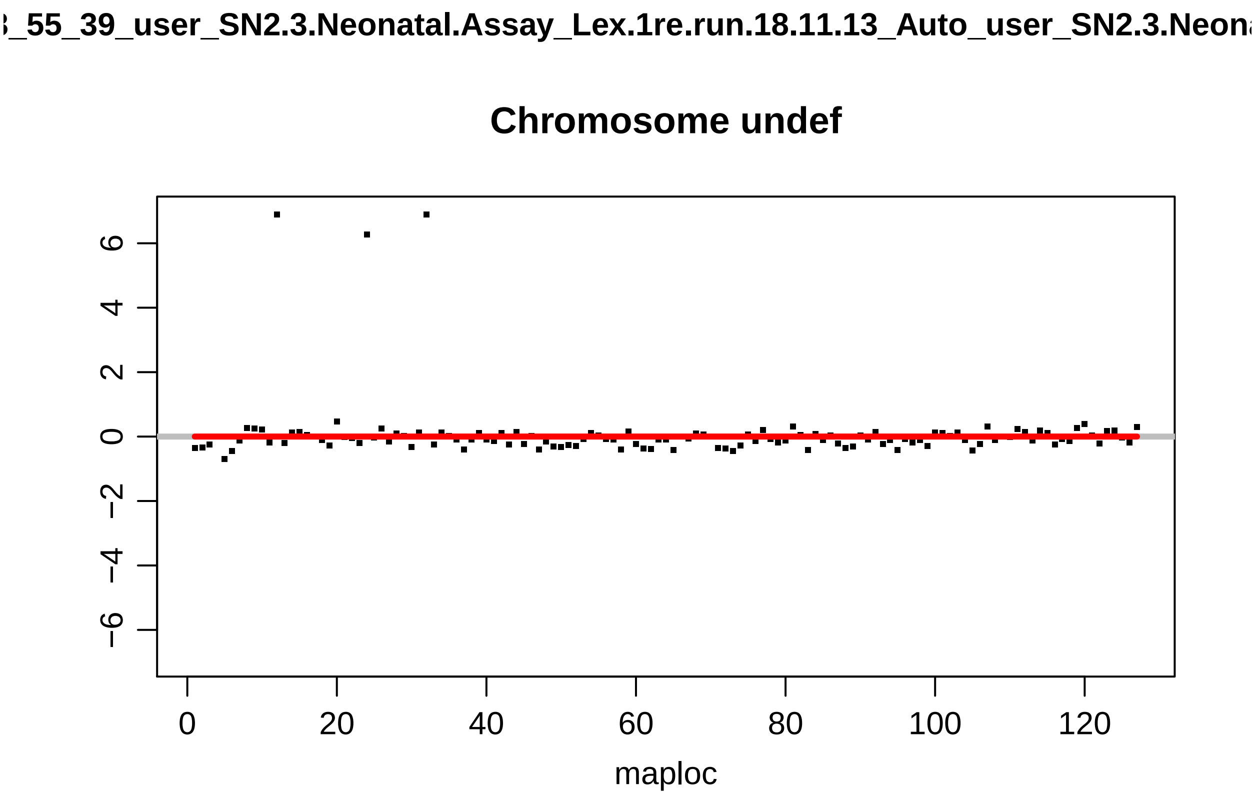


```
## Segplot might not work because of special characters in the sample names. Use only A-Z,a-z and 0-9!
##  There is a hidden function cn.mops:::.replaceNames that replaces the names in the "CNVDetectionResul
```

3_55_39_user_SN2.3.Neonatal.Assay_Lex.1re.run.18.11.13_Auto_user_SN2.3.Neona

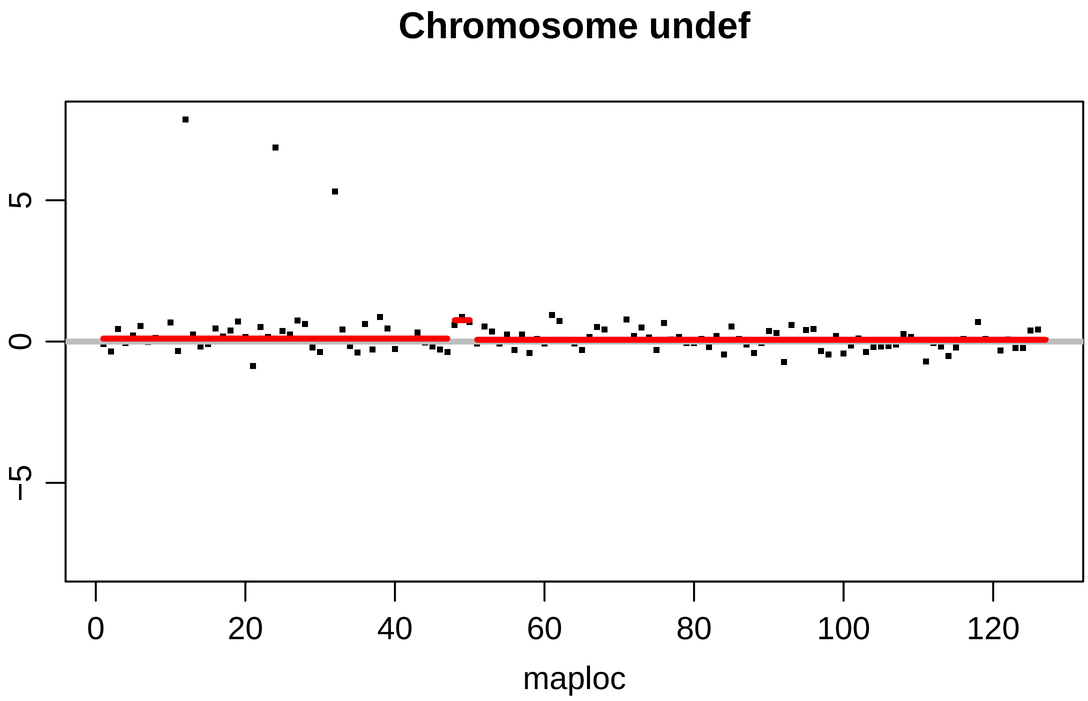


```
## Segplot might not work because of special characters in the sample names. Use only A-Z,a-z and 0-9!
##  There is a hidden function cn.mops:::.replaceNames that replaces the names in the "CNVDetectionResul
```

3_55_39_user_SN2.3.Neonatal.Assay_Lex.1re.run.18.11.13_Auto_user_SN2.3.Neona

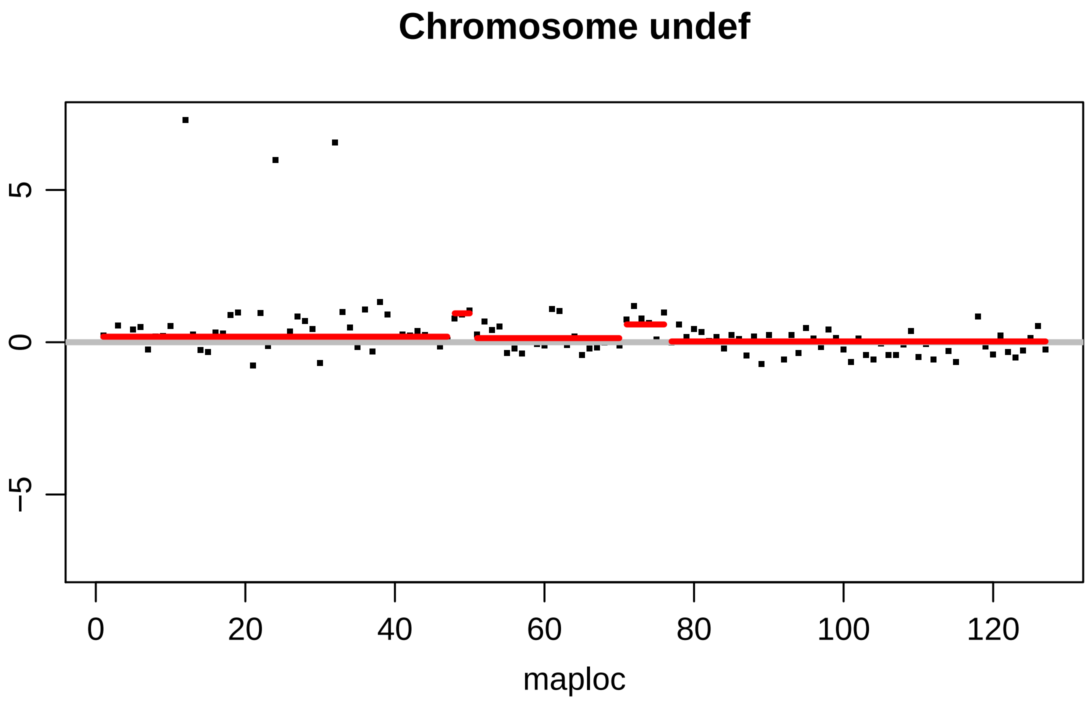

```
## Segplot might not work because of special characters in the sample names. Use only A-Z,a-z and 0-9!
##  There is a hidden function cn.mops:::.replaceNames that replaces the names in the "CNVDetectionResul
```

3_55_39_user_SN2.3.Neonatal.Assay_Lex.1re.run.18.11.13_Auto_user_SN2.3.Neona

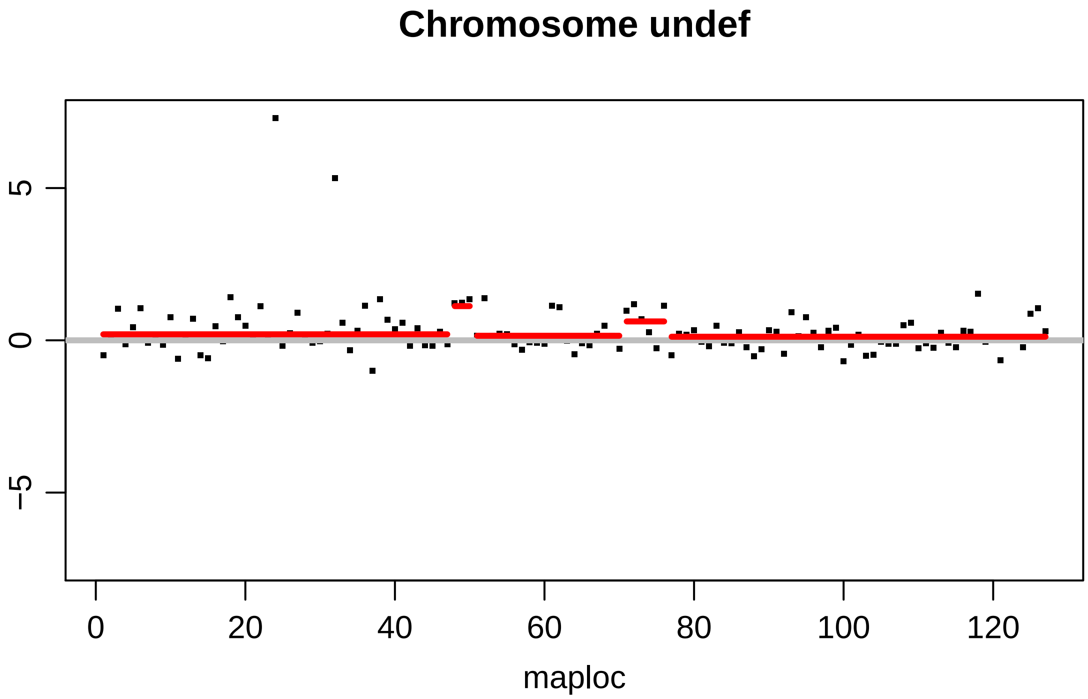


```
## Segplot might not work because of special characters in the sample names. Use only A-Z,a-z and 0-9!
##  There is a hidden function cn.mops:::.replaceNames that replaces the names in the "CNVDetectionResul
```

3_55_39_user_SN2.3.Neonatal.Assay_Lex.1re.run.18.11.13_Auto_user_SN2.3.Neon

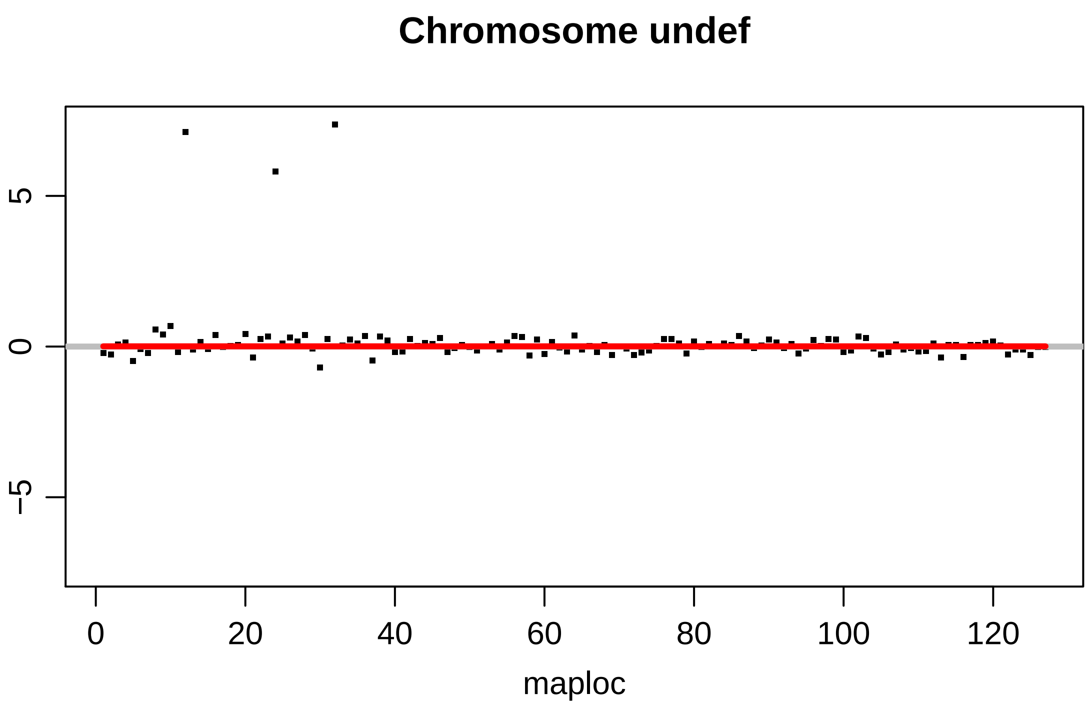

```
## Segplot might not work because of special characters in the sample names. Use only A-Z,a-z and 0-9!
##  There is a hidden function cn.mops:::.replaceNames that replaces the names in the "CNVDetectionResul
```

3_55_39_user_SN2.3.Neonatal.Assay_Lex.1re.run.18.11.13_Auto_user_SN2.3.Neon

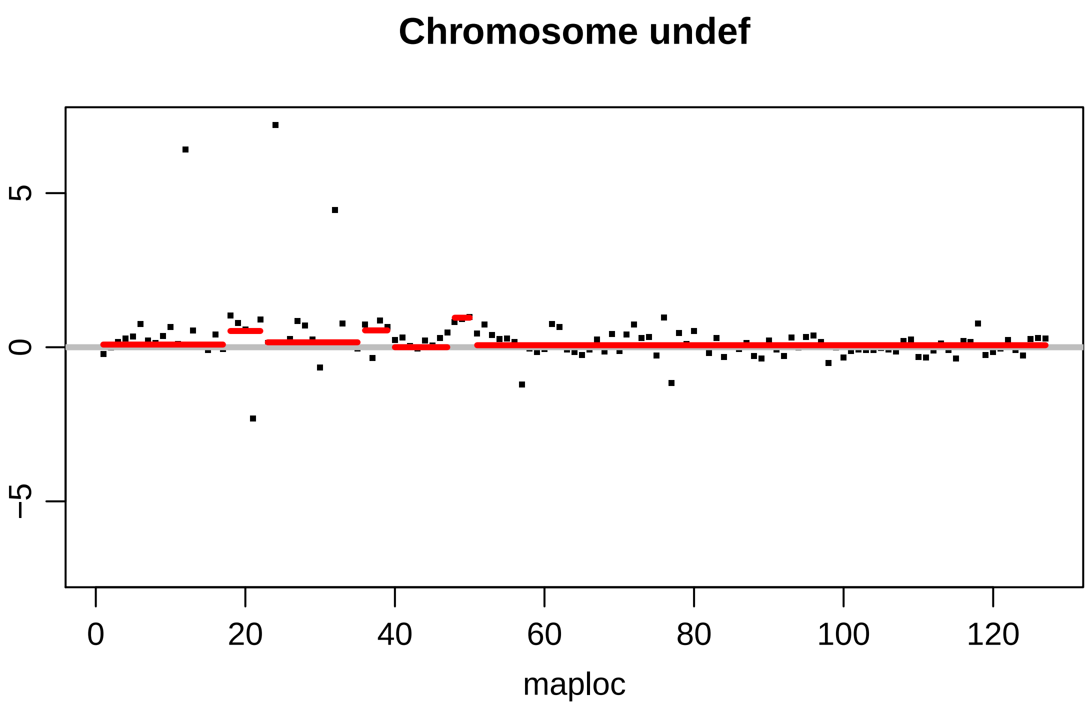

```
##
## CNV regions:
## GRanges object with 6 ranges and 41 metadata columns:
##       seqnames     ranges strand |
##          <Rle>  <IRanges>  <Rle> |
##   [1]    undef [  8,  10]      * |
##   [2]    undef [ 18,  22]      * |
##   [3]    undef [ 36,  39]      * |
##   [4]    undef [ 48,  52]      * |
##   [5]    undef [ 71,  76]      * |
##   [6]    undef [102, 127]      * |
##       Case_IonXpress_002_R_2013_10_17_13_55_39_user_SN2.3.Neonatal.Assay_Lex.1re.run.18.11.13_Auto_us
##
##   [1]
##   [2]
##   [3]
##   [4]
##   [5]
##   [6]
##       Case_IonXpress_004_R_2013_10_17_13_55_39_user_SN2.3.Neonatal.Assay_Lex.1re.run.18.11.13_Auto_us
##
##   [1]
##   [2]
##   [3]
##   [4]
##   [5]
##   [6]
##       Case_IonXpress_006_R_2013_10_17_13_55_39_user_SN2.3.Neonatal.Assay_Lex.1re.run.18.11.13_Auto_us
##
##   [1]
##   [2]
##   [3]
##   [4]
##   [5]
##   [6]
##       Case_IonXpress_007_R_2013_10_17_13_55_39_user_SN2.3.Neonatal.Assay_Lex.1re.run.18.11.13_Auto_us
##
##   [1]
##   [2]
##   [3]
##   [4]
##   [5]
##   [6]
##       Case_IonXpress_008_R_2013_10_17_13_55_39_user_SN2.3.Neonatal.Assay_Lex.1re.run.18.11.13_Auto_us
##
##   [1]
##   [2]
##   [3]
##   [4]
##   [5]
##   [6]
##       Case_IonXpress_010_R_2013_10_17_13_55_39_user_SN2.3.Neonatal.Assay_Lex.1re.run.18.11.13_Auto_us
##
##   [1]
```

```
##   [2]
##   [3]
##   [4]
##   [5]
##   [6]
##       Case_IonXpress_011_R_2013_10_17_13_55_39_user_SN2.3.Neonatal.Assay_Lex.1re.run.18.11.13_Auto_us
##
##   [1]
##   [2]
##   [3]
##   [4]
##   [5]
##   [6]
##       Case_IonXpress_012_R_2013_10_17_13_55_39_user_SN2.3.Neonatal.Assay_Lex.1re.run.18.11.13_Auto_us
##
##   [1]
##   [2]
##   [3]
##   [4]
##   [5]
##   [6]
##       Case_IonXpress_013_R_2013_10_17_13_55_39_user_SN2.3.Neonatal.Assay_Lex.1re.run.18.11.13_Auto_us
##
##   [1]
##   [2]
##   [3]
##   [4]
##   [5]
##   [6]
##       Case_IonXpress_015_R_2013_10_17_13_55_39_user_SN2.3.Neonatal.Assay_Lex.1re.run.18.11.13_Auto_us
##
##   [1]
##   [2]
##   [3]
##   [4]
##   [5]
##   [6]
##       Case_IonXpress_016_R_2013_10_17_13_55_39_user_SN2.3.Neonatal.Assay_Lex.1re.run.18.11.13_Auto_us
##
##   [1]
##   [2]
##   [3]
##   [4]
##   [5]
##   [6]
##       Case_IonXpress_018_R_2013_10_17_13_55_39_user_SN2.3.Neonatal.Assay_Lex.1re.run.18.11.13_Auto_us
##
##   [1]
##   [2]
##   [3]
##   [4]
##   [5]
##   [6]
##       Case_IonXpress_019_R_2013_10_17_13_55_39_user_SN2.3.Neonatal.Assay_Lex.1re.run.18.11.13_Auto_us
```

```
##
##   [1]
##   [2]
##   [3]
##   [4]
##   [5]
##   [6]
##       Case_IonXpress_021_R_2013_10_17_13_55_39_user_SN2.3.Neonatal.Assay_Lex.1re.run.18.11.13_Auto_us
##
##   [1]
##   [2]
##   [3]
##   [4]
##   [5]
##   [6]
##       Case_IonXpress_022_R_2013_10_17_13_55_39_user_SN2.3.Neonatal.Assay_Lex.1re.run.18.11.13_Auto_us
##
##   [1]
##   [2]
##   [3]
##   [4]
##   [5]
##   [6]
##       Case_IonXpress_023_R_2013_10_17_13_55_39_user_SN2.3.Neonatal.Assay_Lex.1re.run.18.11.13_Auto_us
##
##   [1]
##   [2]
##   [3]
##   [4]
##   [5]
##   [6]
##       Case_IonXpress_024_R_2013_10_17_13_55_39_user_SN2.3.Neonatal.Assay_Lex.1re.run.18.11.13_Auto_us
##
##   [1]
##   [2]
##   [3]
##   [4]
##   [5]
##   [6]
##       Case_IonXpress_025_R_2013_10_17_13_55_39_user_SN2.3.Neonatal.Assay_Lex.1re.run.18.11.13_Auto_us
##
##   [1]
##   [2]
##   [3]
##   [4]
##   [5]
##   [6]
##       Case_IonXpress_026_R_2013_10_17_13_55_39_user_SN2.3.Neonatal.Assay_Lex.1re.run.18.11.13_Auto_us
##
##   [1]
##   [2]
##   [3]
##   [4]
##   [5]
```

```
##   [6]
##       Case_IonXpress_027_R_2013_10_17_13_55_39_user_SN2.3.Neonatal.Assay_Lex.1re.run.18.11.13_Auto_us
##
##   [1]
##   [2]
##   [3]
##   [4]
##   [5]
##   [6]
##       Case_IonXpress_028_R_2013_10_17_13_55_39_user_SN2.3.Neonatal.Assay_Lex.1re.run.18.11.13_Auto_us
##
##   [1]
##   [2]
##   [3]
##   [4]
##   [5]
##   [6]
##       Case_IonXpress_029_R_2013_10_17_13_55_39_user_SN2.3.Neonatal.Assay_Lex.1re.run.18.11.13_Auto_us
##
##   [1]
##   [2]
##   [3]
##   [4]
##   [5]
##   [6]
##       Case_IonXpress_030_R_2013_10_17_13_55_39_user_SN2.3.Neonatal.Assay_Lex.1re.run.18.11.13_Auto_us
##
##   [1]
##   [2]
##   [3]
##   [4]
##   [5]
##   [6]
##       Case_IonXpress_031_R_2013_10_17_13_55_39_user_SN2.3.Neonatal.Assay_Lex.1re.run.18.11.13_Auto_us
##
##   [1]
##   [2]
##   [3]
##   [4]
##   [5]
##   [6]
##       Case_IonXpress_032_R_2013_10_17_13_55_39_user_SN2.3.Neonatal.Assay_Lex.1re.run.18.11.13_Auto_us
##
##   [1]
##   [2]
##   [3]
##   [4]
##   [5]
##   [6]
##       Case_IonXpress_033_R_2013_10_17_13_55_39_user_SN2.3.Neonatal.Assay_Lex.1re.run.18.11.13_Auto_us
##
##   [1]
##   [2]
##   [3]
```

```
## [4]
## [5]
## [6]
##     Case_IonXpress_034_R_2013_10_17_13_55_39_user_SN2.3.Neonatal.Assay_Lex.1re.run.18.11.13_Auto_us
##
## [1]
## [2]
## [3]
## [4]
## [5]
## [6]
##     Case_IonXpress_035_R_2013_10_17_13_55_39_user_SN2.3.Neonatal.Assay_Lex.1re.run.18.11.13_Auto_us
##
## [1]
## [2]
## [3]
## [4]
## [5]
## [6]
##     Case_IonXpress_036_R_2013_10_17_13_55_39_user_SN2.3.Neonatal.Assay_Lex.1re.run.18.11.13_Auto_us
##
## [1]
## [2]
## [3]
## [4]
## [5]
## [6]
##     Case_IonXpress_037_R_2013_10_17_13_55_39_user_SN2.3.Neonatal.Assay_Lex.1re.run.18.11.13_Auto_us
##
## [1]
## [2]
## [3]
## [4]
## [5]
## [6]
##     Case_IonXpress_038_R_2013_10_17_13_55_39_user_SN2.3.Neonatal.Assay_Lex.1re.run.18.11.13_Auto_us
##
## [1]
## [2]
## [3]
## [4]
## [5]
## [6]
##     Case_IonXpress_039_R_2013_10_17_13_55_39_user_SN2.3.Neonatal.Assay_Lex.1re.run.18.11.13_Auto_us
##
## [1]
## [2]
## [3]
## [4]
## [5]
## [6]
##     Case_IonXpress_040_R_2013_10_17_13_55_39_user_SN2.3.Neonatal.Assay_Lex.1re.run.18.11.13_Auto_us
##
## [1]
```

```
##   [2]
##   [3]
##   [4]
##   [5]
##   [6]
##       Case_IonXpress_041_R_2013_10_17_13_55_39_user_SN2.3.Neonatal.Assay_Lex.1re.run.18.11.13_Auto_us
##
##   [1]
##   [2]
##   [3]
##   [4]
##   [5]
##   [6]
##       Case_IonXpress_042_R_2013_10_17_13_55_39_user_SN2.3.Neonatal.Assay_Lex.1re.run.18.11.13_Auto_us
##
##   [1]
##   [2]
##   [3]
##   [4]
##   [5]
##   [6]
##       Case_IonXpress_043_R_2013_10_17_13_55_39_user_SN2.3.Neonatal.Assay_Lex.1re.run.18.11.13_Auto_us
##
##   [1]
##   [2]
##   [3]
##   [4]
##   [5]
##   [6]
##       Case_IonXpress_044_R_2013_10_17_13_55_39_user_SN2.3.Neonatal.Assay_Lex.1re.run.18.11.13_Auto_us
##
##   [1]
##   [2]
##   [3]
##   [4]
##   [5]
##   [6]
##       Case_IonXpress_045_R_2013_10_17_13_55_39_user_SN2.3.Neonatal.Assay_Lex.1re.run.18.11.13_Auto_us
##
##   [1]
##   [2]
##   [3]
##   [4]
##   [5]
##   [6]
##       Case_IonXpress_046_R_2013_10_17_13_55_39_user_SN2.3.Neonatal.Assay_Lex.1re.run.18.11.13_Auto_us
##
##   [1]
##   [2]
##   [3]
##   [4]
##   [5]
##   [6]
##       Case_IonXpress_047_R_2013_10_17_13_55_39_user_SN2.3.Neonatal.Assay_Lex.1re.run.18.11.13_Auto_us
```

```
##
##   [1]
##   [2]
##   [3]
##   [4]
##   [5]
##   [6]
##       Case_IonXpress_048_R_2013_10_17_13_55_39_user_SN2.3.Neonatal.Assay_Lex.1re.run.18.11.13_Auto_us
##
##   [1]
##   [2]
##   [3]
##   [4]
##   [5]
##   [6]
##   -------
##   seqinfo: 1 sequence from an unspecified genome; no seqlengths
##
## Individual CNVs:
## GRanges object with 30 ranges and 4 metadata columns:
##        seqnames     ranges strand   |
##           <Rle>  <IRanges>  <Rle>   |
##    [1]    undef [102, 127]      *   |
##    [2]    undef [102, 127]      *   |
##    [3]    undef [120, 122]      *   |
##    [4]    undef [102, 127]      *   |
##    [5]    undef [ 48,  52]      *   |
##    ...      ...        ...    ... ...
##   [26]    undef   [48, 50]      *   |
##   [27]    undef   [71, 76]      *   |
##   [28]    undef   [18, 22]      *   |
##   [29]    undef   [36, 39]      *   |
##   [30]    undef   [48, 50]      *   |
##
##
##    [1] Case_IonXpress_004_R_2013_10_17_13_55_39_user_SN2.3.Neonatal.Assay_Lex.1re.run.18.11.13_Auto_u
##    [2] Case_IonXpress_007_R_2013_10_17_13_55_39_user_SN2.3.Neonatal.Assay_Lex.1re.run.18.11.13_Auto_u
##    [3] Case_IonXpress_010_R_2013_10_17_13_55_39_user_SN2.3.Neonatal.Assay_Lex.1re.run.18.11.13_Auto_u
##    [4] Case_IonXpress_011_R_2013_10_17_13_55_39_user_SN2.3.Neonatal.Assay_Lex.1re.run.18.11.13_Auto_u
##    [5] Case_IonXpress_015_R_2013_10_17_13_55_39_user_SN2.3.Neonatal.Assay_Lex.1re.run.18.11.13_Auto_u
##    ...
##   [26] Case_IonXpress_045_R_2013_10_17_13_55_39_user_SN2.3.Neonatal.Assay_Lex.1re.run.18.11.13_Auto_u
##   [27] Case_IonXpress_045_R_2013_10_17_13_55_39_user_SN2.3.Neonatal.Assay_Lex.1re.run.18.11.13_Auto_u
##   [28] Case_IonXpress_047_R_2013_10_17_13_55_39_user_SN2.3.Neonatal.Assay_Lex.1re.run.18.11.13_Auto_u
##   [29] Case_IonXpress_047_R_2013_10_17_13_55_39_user_SN2.3.Neonatal.Assay_Lex.1re.run.18.11.13_Auto_u
##   [30] Case_IonXpress_047_R_2013_10_17_13_55_39_user_SN2.3.Neonatal.Assay_Lex.1re.run.18.11.13_Auto_u
##           median      mean          CN
##        <numeric> <numeric> <character>
##    [1] 1.0216644 1.0511149         CN4
##    [2] 1.1900763 1.1627005         CN4
##    [3] 0.9806880 0.6613861         CN3
##    [4] 1.0687611 1.1536716         CN4
##    [5] 0.5853173 0.5906254         CN3
##    ...       ...       ...         ...
```

```
##   [26] 1.1144146 1.1233220         CN4
##   [27] 0.7887270 0.6207454         CN3
##   [28] 0.5912174 0.5265980         CN3
##   [29] 0.6232883 0.5469160         CN3
##   [30] 0.9829114 0.9576635         CN4
##   -------
##   seqinfo: 1 sequence from an unspecified genome; no seqlengths
## [1] "/Users/gdemidov/Downloads/doc/Run_SN2_4_fin_05_qc.xls"

## Normalizing...

## Starting local modeling, please be patient...

## Reference sequence:  undef

## Starting segmentation algorithm...

## Using "fastseg" for segmentation.

## [1] ""
## [1] "/Users/gdemidov/Downloads/doc/Run_SN2_4_fin_05_qc.xls"
## [1] ""

## Segplot might not work because of special characters in the sample names. Use only A-Z,a-z and 0-9!
##  There is a hidden function cn.mops:::.replaceNames that replaces the names in the "CNVDetectionResul
```

3_55_39_user_SN2.3.Neonatal.Assay_Lex.1re.run.18.11.13_Auto_user_SN2.3.Neona

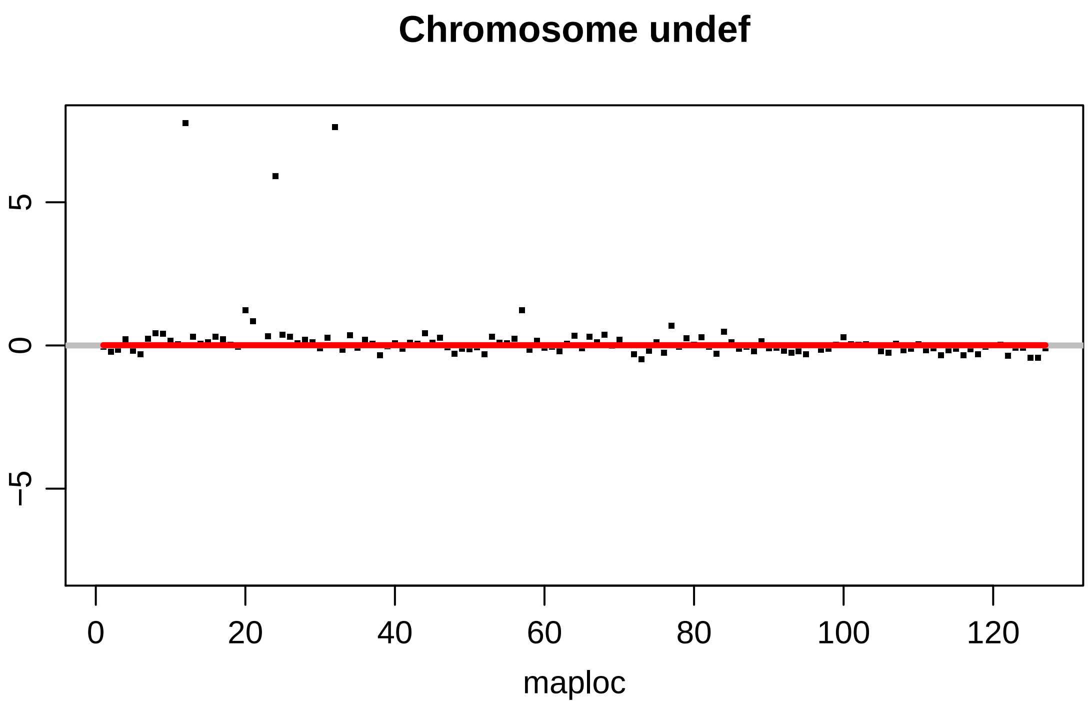

```
## Segplot might not work because of special characters in the sample names. Use only A-Z,a-z and 0-9!
##  There is a hidden function cn.mops:::.replaceNames that replaces the names in the "CNVDetectionResul
```

r_SN2.4.Neonatal.Assay2_new_bed_version_318_chip_v2_Auto_user_SN2.4.Neona

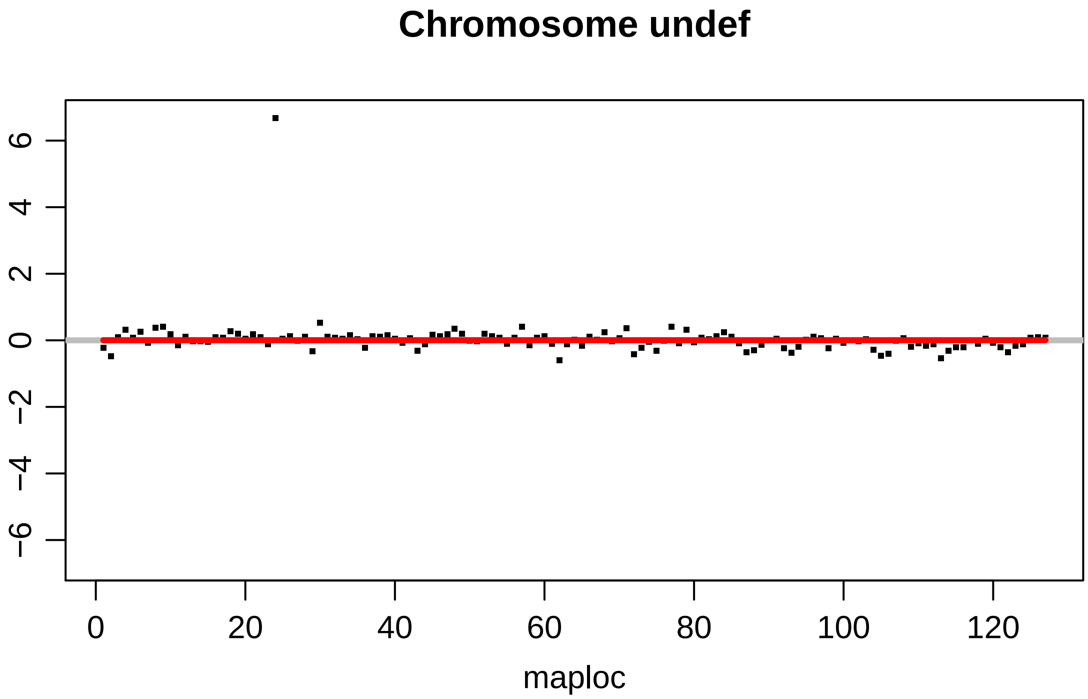


```
## Segplot might not work because of special characters in the sample names. Use only A-Z,a-z and 0-9!
##  There is a hidden function cn.mops:::.replaceNames that replaces the names in the "CNVDetectionResul
```

r_SN2.4.Neonatal.Assay2_new_bed_version_318_chip_v2_Auto_user_SN2.4.Neon

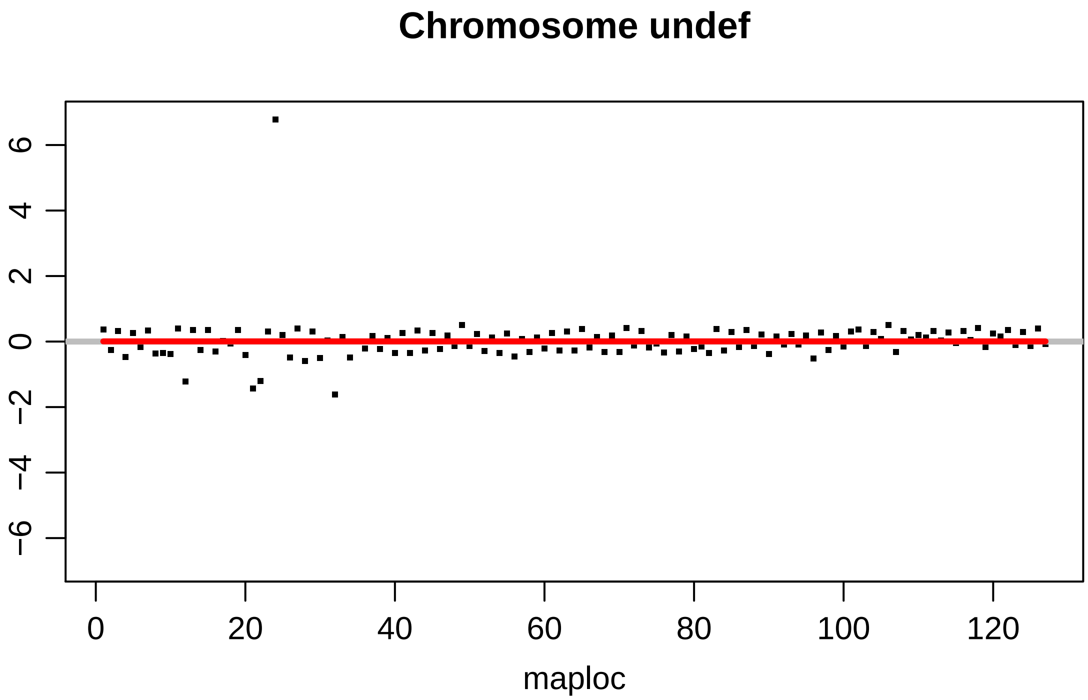


```
## Segplot might not work because of special characters in the sample names. Use only A-Z,a-z and 0-9!
##  There is a hidden function cn.mops:::.replaceNames that replaces the names in the "CNVDetectionResul
```

r_SN2.4.Neonatal.Assay2_new_bed_version_318_chip_v2_Auto_user_SN2.4.Neon

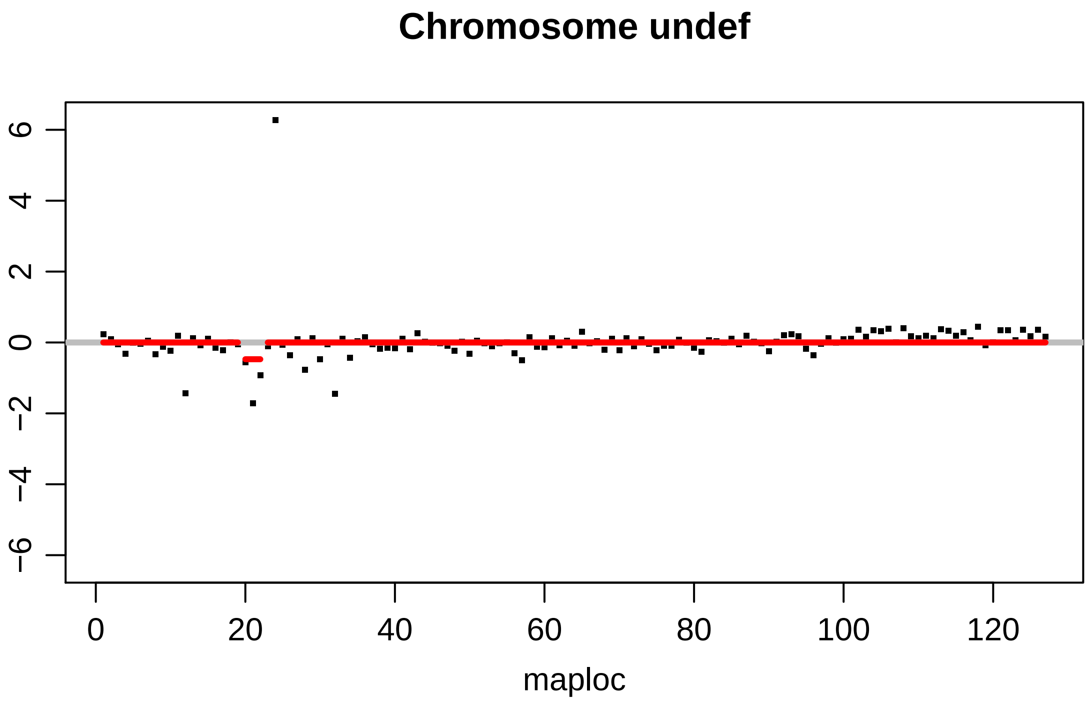

```
## Segplot might not work because of special characters in the sample names. Use only A-Z,a-z and 0-9!
##  There is a hidden function cn.mops:::.replaceNames that replaces the names in the "CNVDetectionResul
```

r_SN2.4.Neonatal.Assay2_new_bed_version_318_chip_v2_Auto_user_SN2.4.Neona

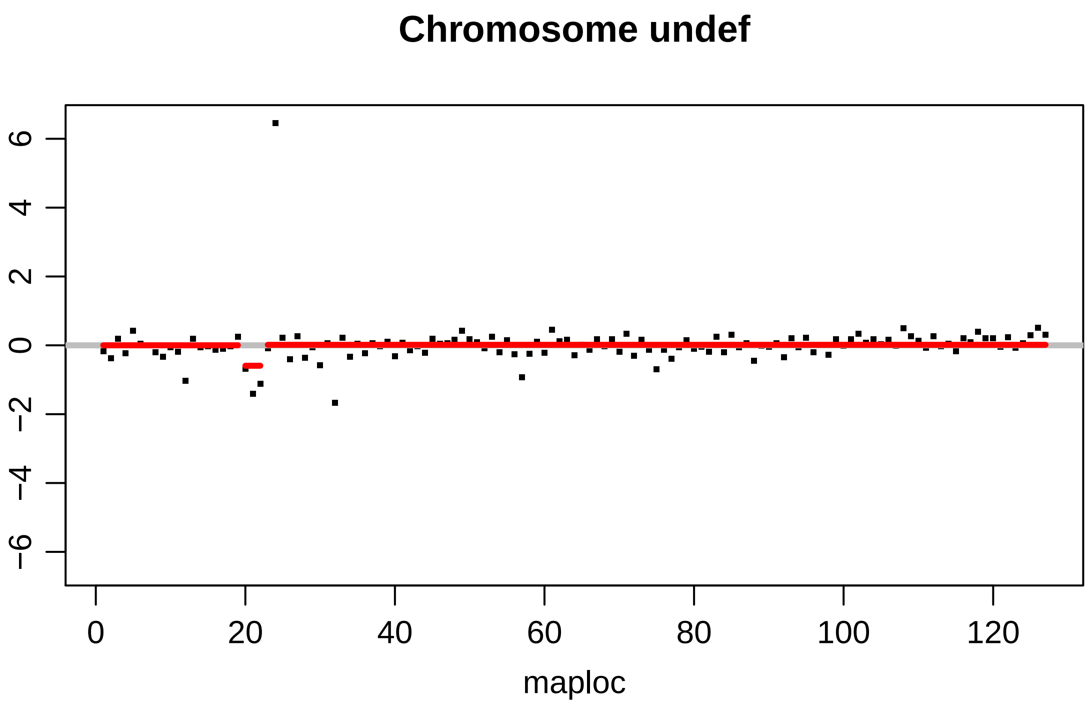


```
## Segplot might not work because of special characters in the sample names. Use only A-Z,a-z and 0-9!
##  There is a hidden function cn.mops:::.replaceNames that replaces the names in the "CNVDetectionResul
```

r_SN2.4.Neonatal.Assay2_new_bed_version_318_chip_v2_Auto_user_SN2.4.Neon

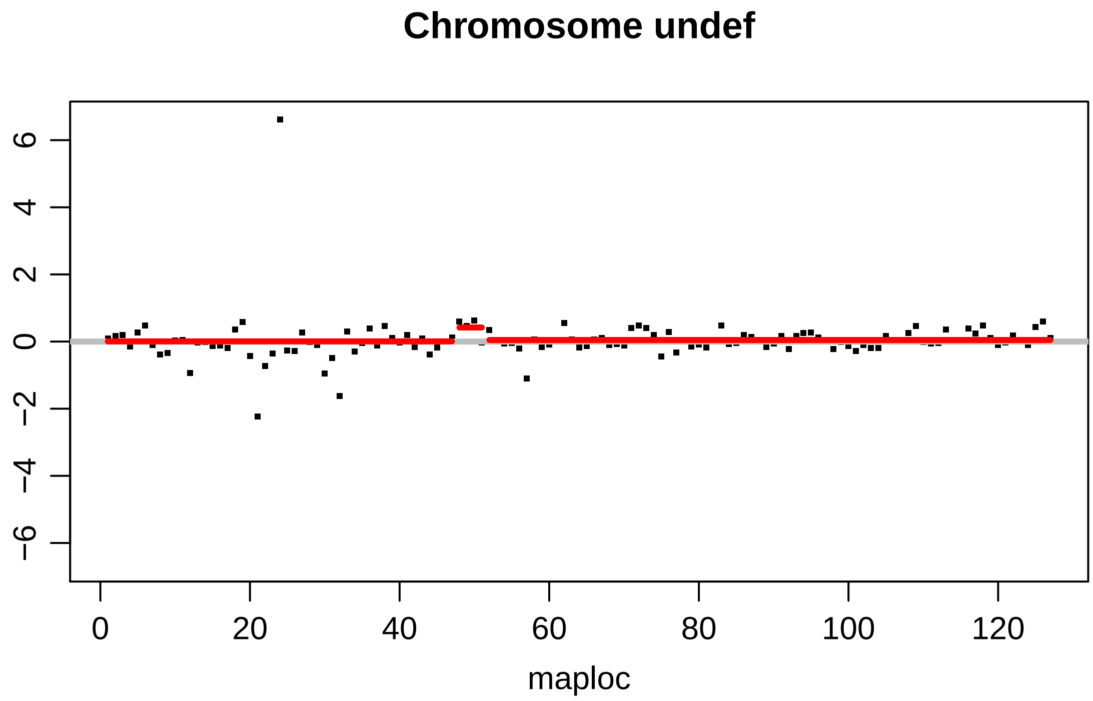


```
## Segplot might not work because of special characters in the sample names. Use only A-Z,a-z and 0-9!
##  There is a hidden function cn.mops:::.replaceNames that replaces the names in the "CNVDetectionResu
```

r_SN2.4.Neonatal.Assay2_new_bed_version_318_chip_v2_Auto_user_SN2.4.Neon

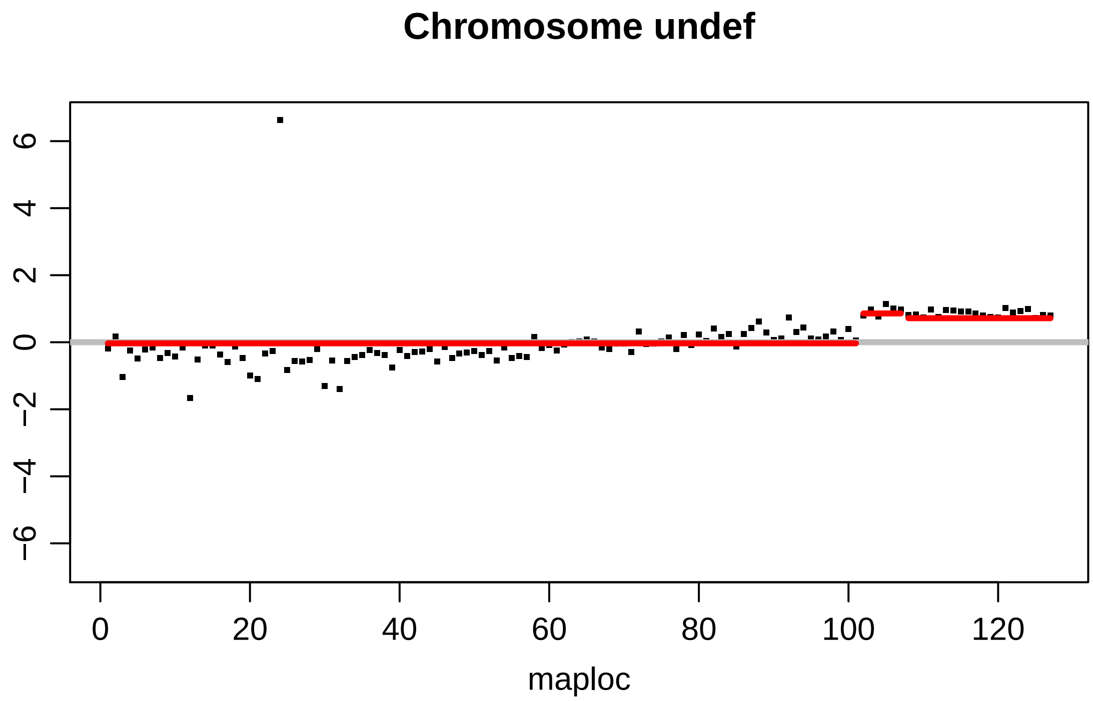

Segplot might not work because of special characters in the sample names. Use only A-Z,a-z and 0-9!
There is a hidden function cn.mops:::.replaceNames that replaces the names in the "CNVDetectionResul

r_SN2.4.Neonatal.Assay2_new_bed_version_318_chip_v2_Auto_user_SN2.4.Neona

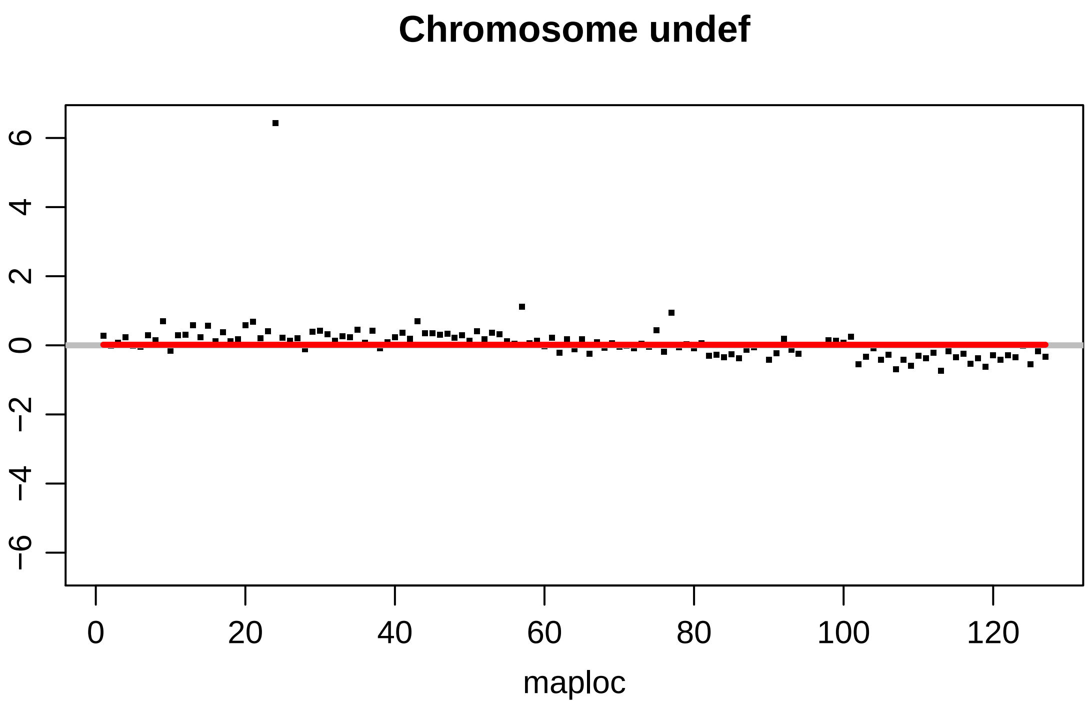

Segplot might not work because of special characters in the sample names. Use only A-Z,a-z and 0-9!
There is a hidden function cn.mops:::.replaceNames that replaces the names in the "CNVDetectionResul

r_SN2.4.Neonatal.Assay2_new_bed_version_318_chip_v2_Auto_user_SN2.4.Neona

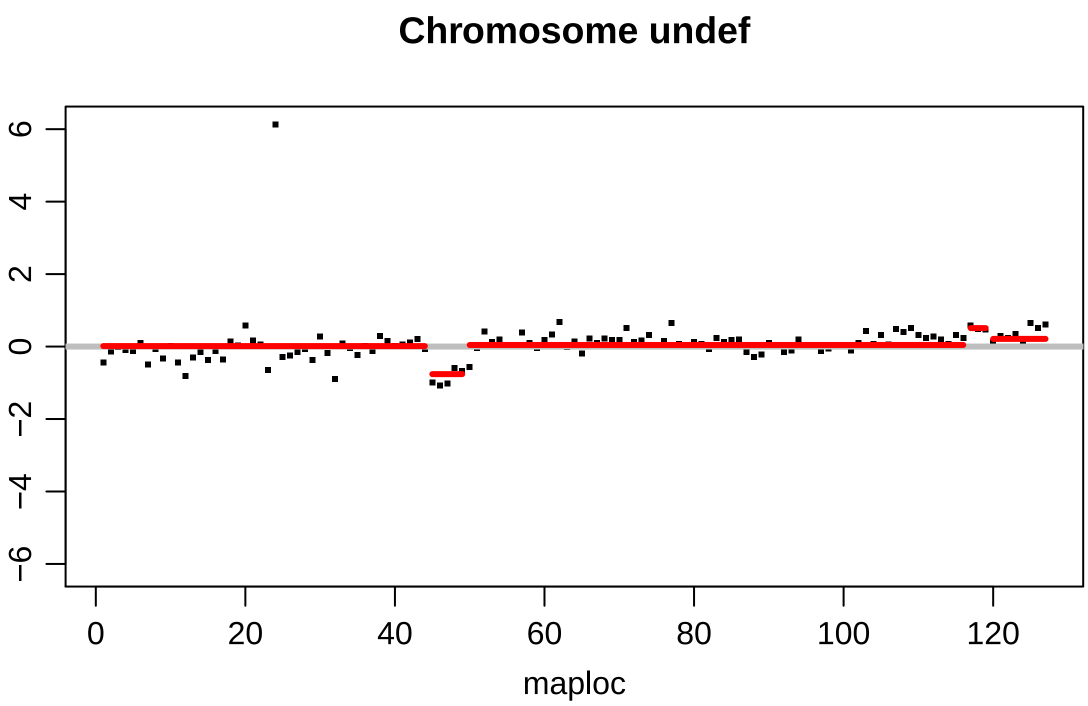


```
## Segplot might not work because of special characters in the sample names. Use only A-Z,a-z and 0-9!
##  There is a hidden function cn.mops:::.replaceNames that replaces the names in the "CNVDetectionResu
```

r_SN2.4.Neonatal.Assay2_new_bed_version_318_chip_v2_Auto_user_SN2.4.Neona

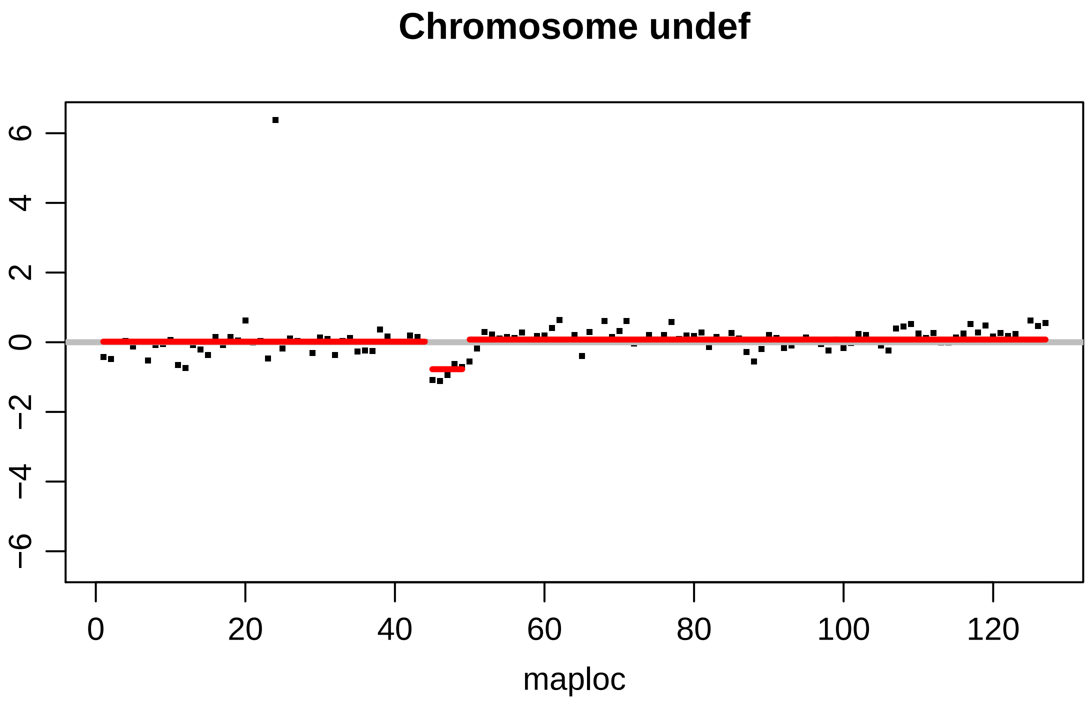

```
## Segplot might not work because of special characters in the sample names. Use only A-Z,a-z and 0-9!
##  There is a hidden function cn.mops:::.replaceNames that replaces the names in the "CNVDetectionResul
```

r_SN2.4.Neonatal.Assay2_new_bed_version_318_chip_v2_Auto_user_SN2.4.Neona

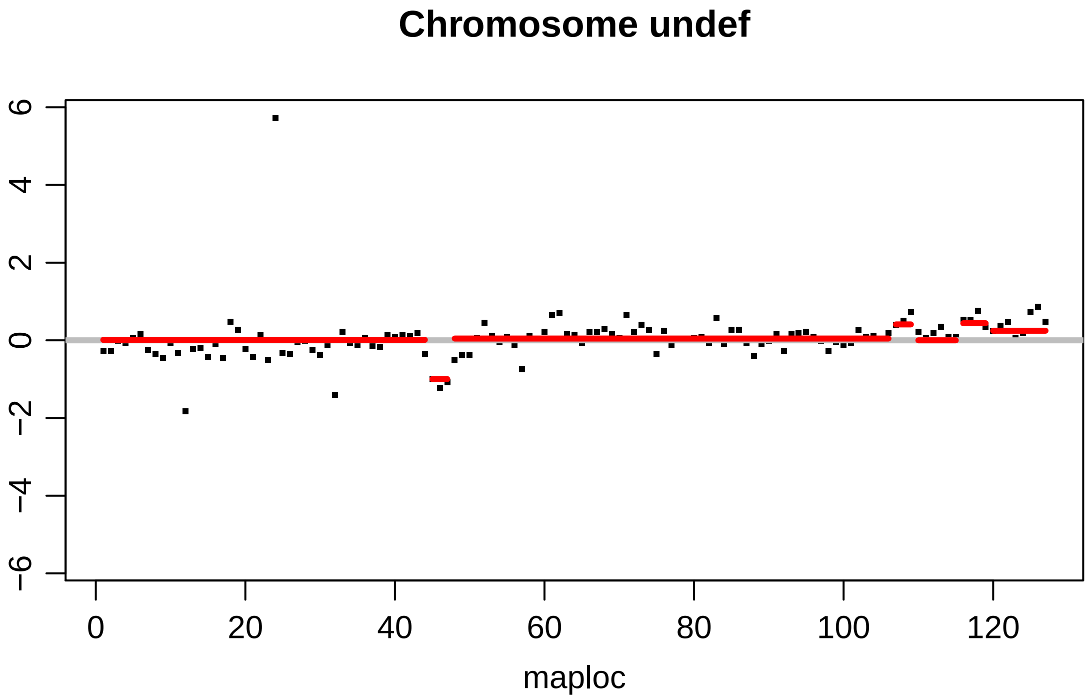


```
## Segplot might not work because of special characters in the sample names. Use only A-Z,a-z and 0-9!
##  There is a hidden function cn.mops:::.replaceNames that replaces the names in the "CNVDetectionResul
```

r_SN2.4.Neonatal.Assay2_new_bed_version_318_chip_v2_Auto_user_SN2.4.Neon

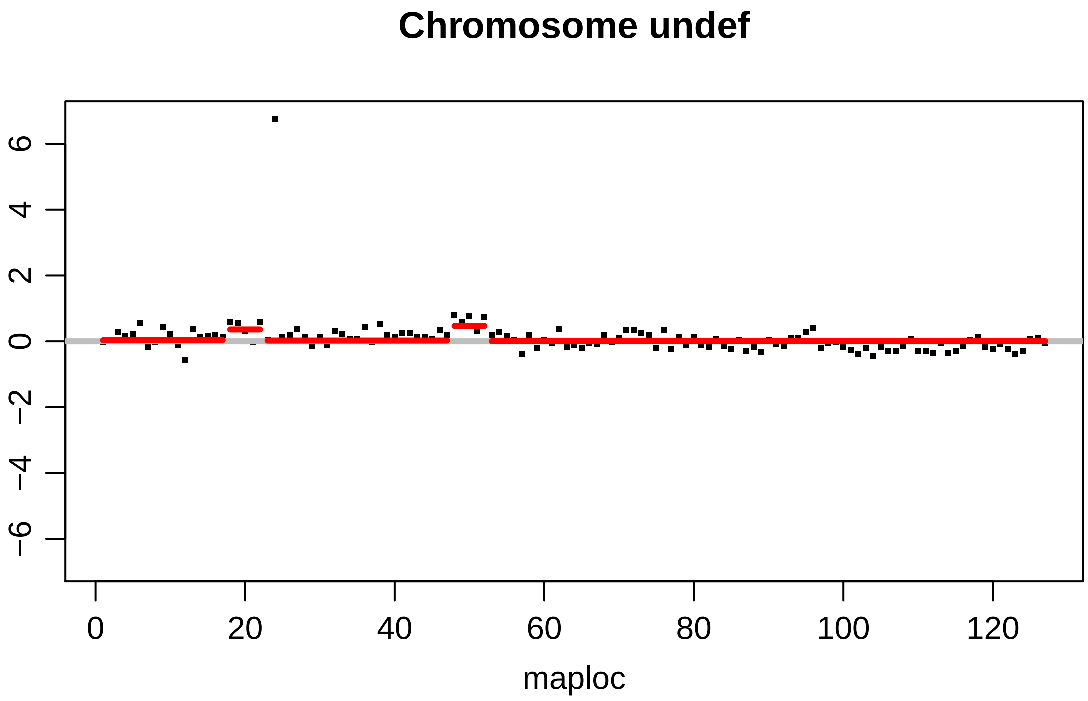


```
## Segplot might not work because of special characters in the sample names. Use only A-Z,a-z and 0-9!
##  There is a hidden function cn.mops:::.replaceNames that replaces the names in the "CNVDetectionResul
```

r_SN2.4.Neonatal.Assay2_new_bed_version_318_chip_v2_Auto_user_SN2.4.Neon

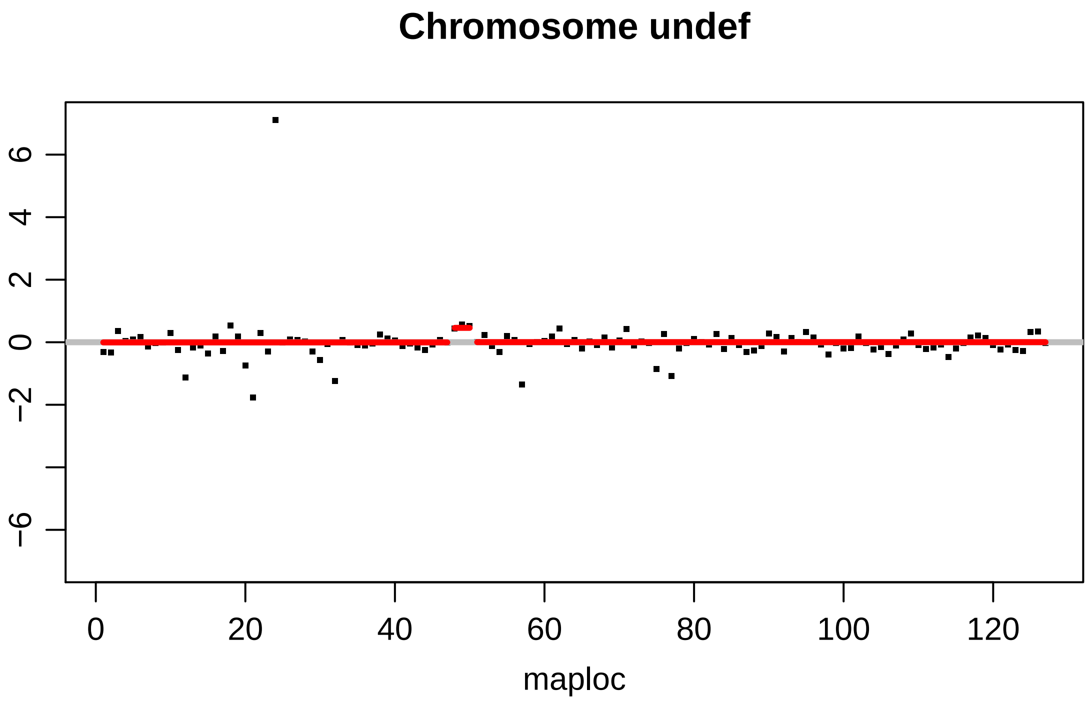

```
## Segplot might not work because of special characters in the sample names. Use only A-Z,a-z and 0-9!
##  There is a hidden function cn.mops:::.replaceNames that replaces the names in the "CNVDetectionResul
```

r_SN2.4.Neonatal.Assay2_new_bed_version_318_chip_v2_Auto_user_SN2.4.Neona

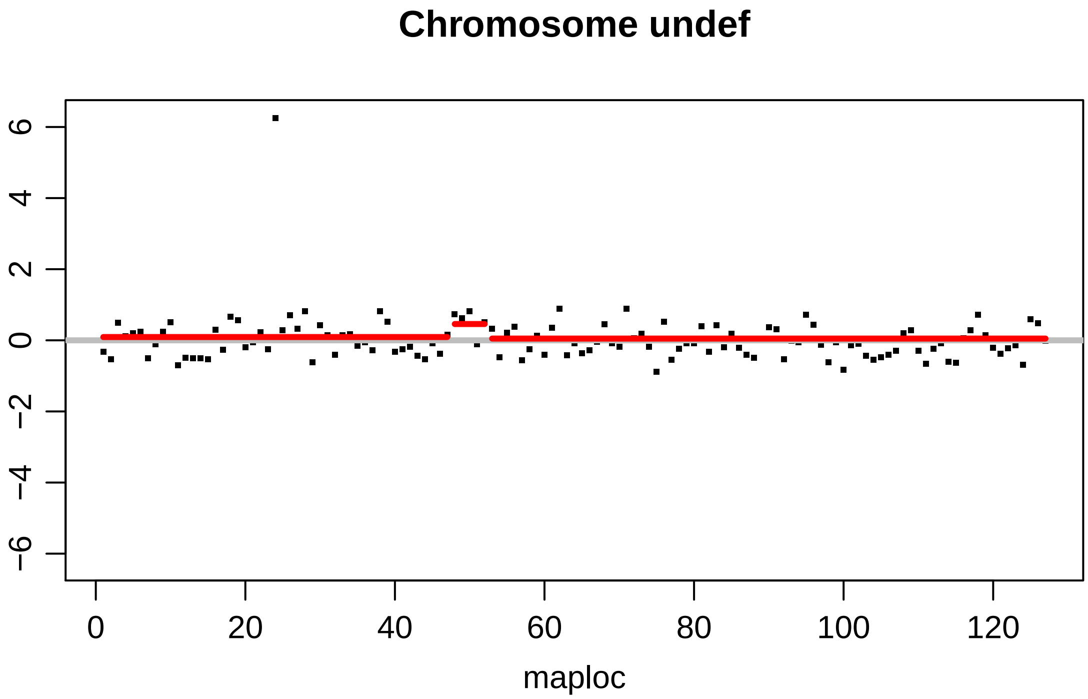


```
## Segplot might not work because of special characters in the sample names. Use only A-Z,a-z and 0-9!
##  There is a hidden function cn.mops:::.replaceNames that replaces the names in the "CNVDetectionResul
```

r_SN2.4.Neonatal.Assay2_new_bed_version_318_chip_v2_Auto_user_SN2.4.Neon

Chromosome undef

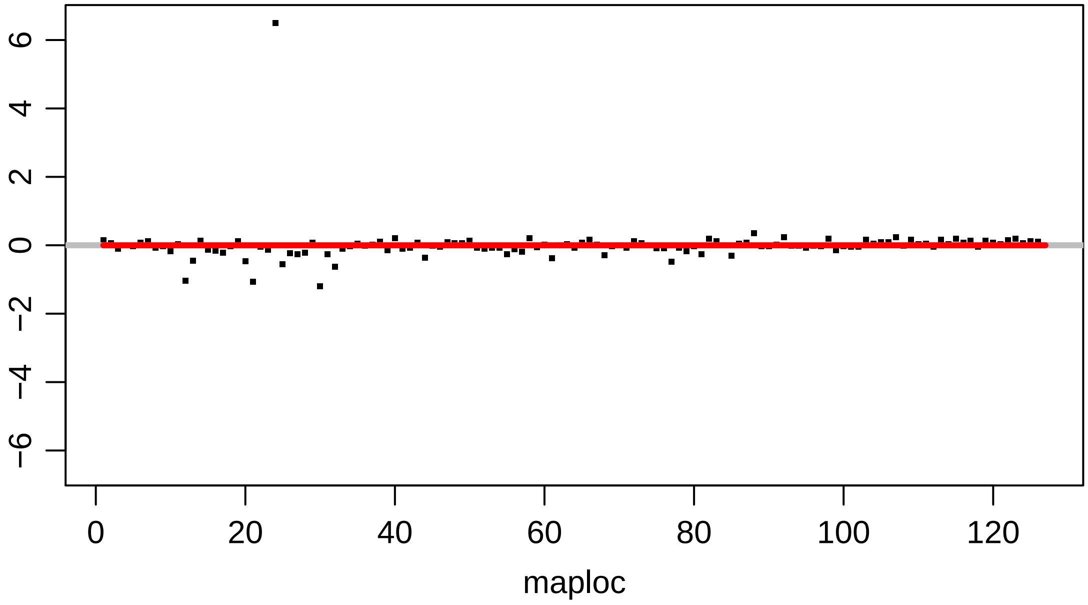


```
## Segplot might not work because of special characters in the sample names. Use only A-Z,a-z and 0-9!
##  There is a hidden function cn.mops:::.replaceNames that replaces the names in the "CNVDetectionResul
```

r_SN2.4.Neonatal.Assay2_new_bed_version_318_chip_v2_Auto_user_SN2.4.Neon

Chromosome undef

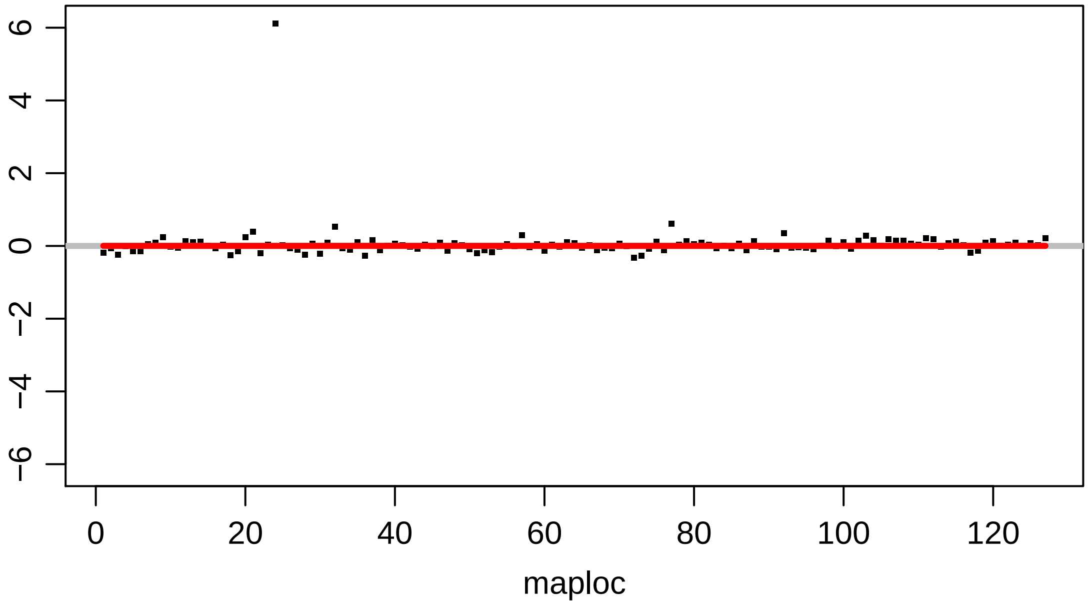

```
## Segplot might not work because of special characters in the sample names. Use only A-Z,a-z and 0-9!
##  There is a hidden function cn.mops:::.replaceNames that replaces the names in the "CNVDetectionResul
```

r_SN2.4.Neonatal.Assay2_new_bed_version_318_chip_v2_Auto_user_SN2.4.Neon

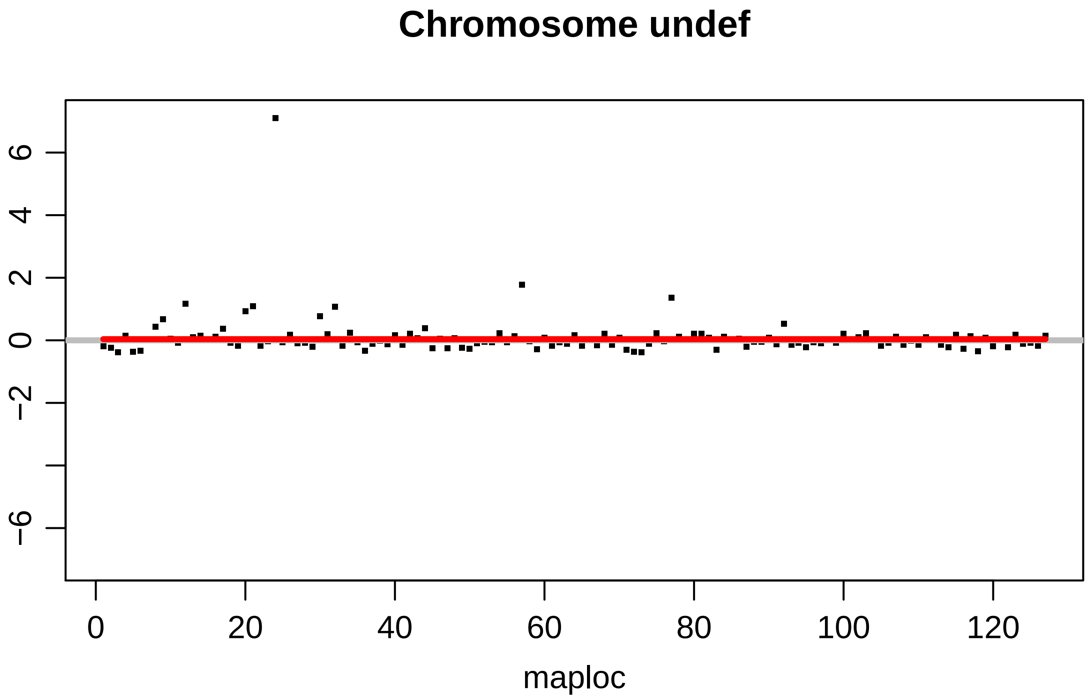


```
## Segplot might not work because of special characters in the sample names. Use only A-Z,a-z and 0-9!
##  There is a hidden function cn.mops:::.replaceNames that replaces the names in the "CNVDetectionResul
```

r_SN2.4.Neonatal.Assay2_new_bed_version_318_chip_v2_Auto_user_SN2.4.Neona

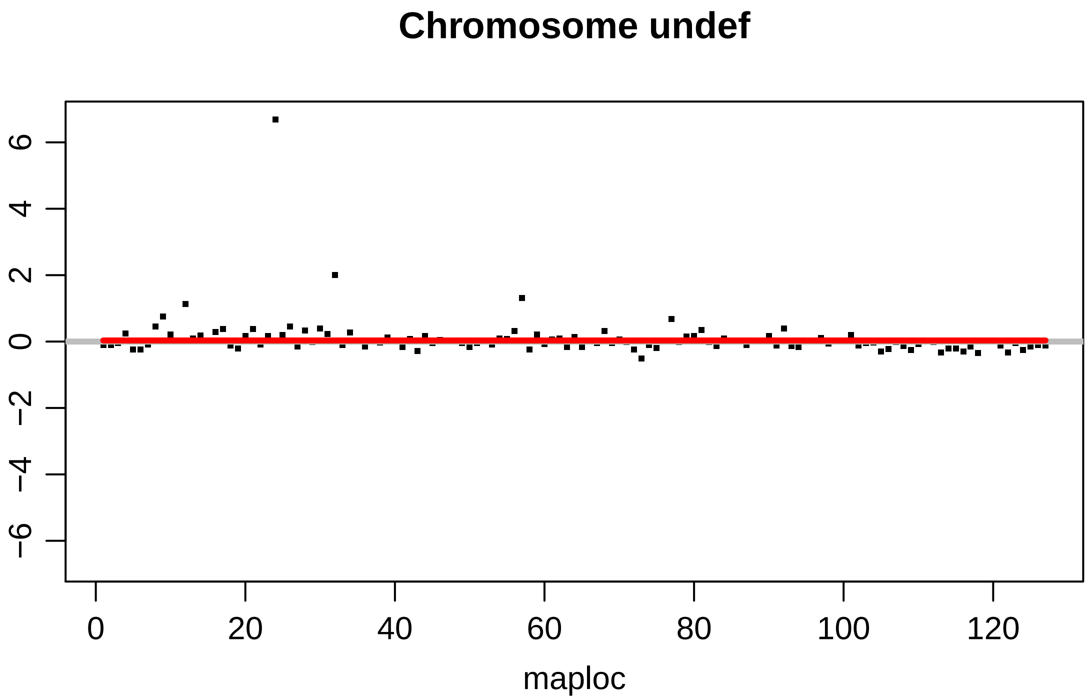


```
## Segplot might not work because of special characters in the sample names. Use only A-Z,a-z and 0-9!
##  There is a hidden function cn.mops:::.replaceNames that replaces the names in the "CNVDetectionResul
```

r_SN2.4.Neonatal.Assay2_new_bed_version_318_chip_v2_Auto_user_SN2.4.Neona

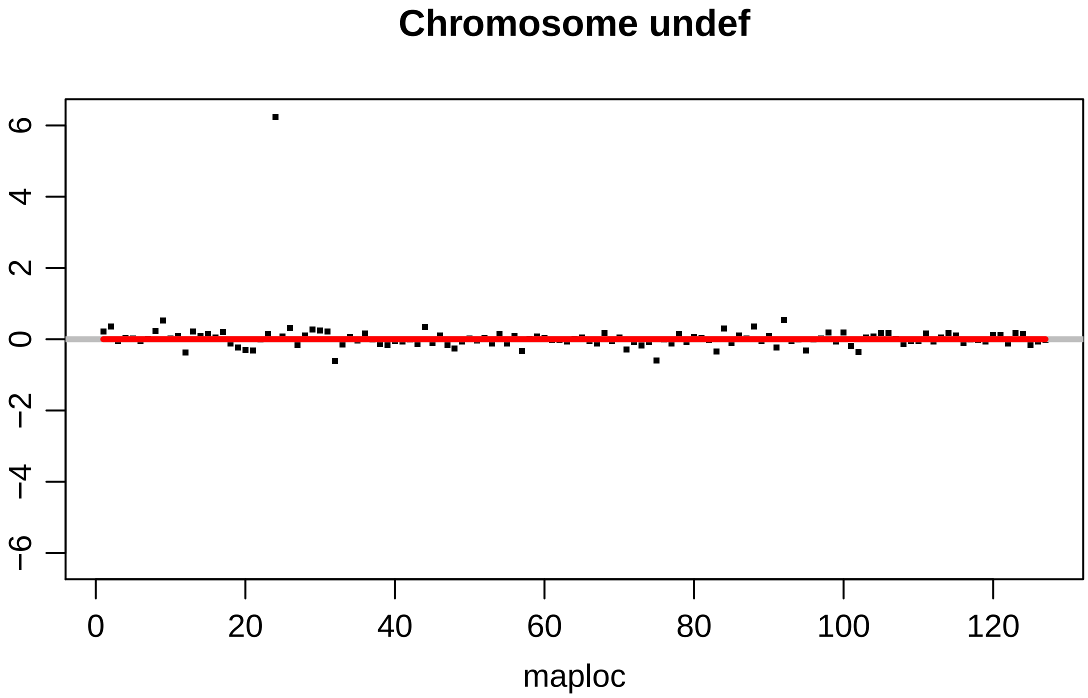

```
## Segplot might not work because of special characters in the sample names. Use only A-Z,a-z and 0-9!
##  There is a hidden function cn.mops:::.replaceNames that replaces the names in the "CNVDetectionResul
```

r_SN2.4.Neonatal.Assay2_new_bed_version_318_chip_v2_Auto_user_SN2.4.Neona

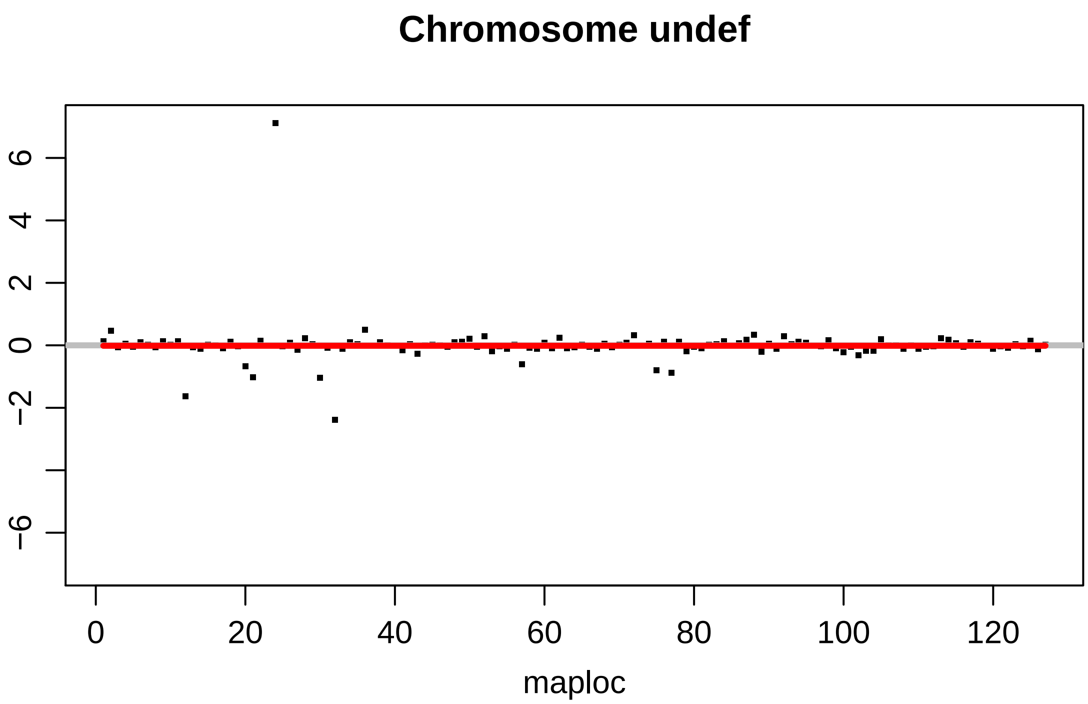


```
## Segplot might not work because of special characters in the sample names. Use only A-Z,a-z and 0-9!
##  There is a hidden function cn.mops:::.replaceNames that replaces the names in the "CNVDetectionResul
```

r_SN2.4.Neonatal.Assay2_new_bed_version_318_chip_v2_Auto_user_SN2.4.Neon

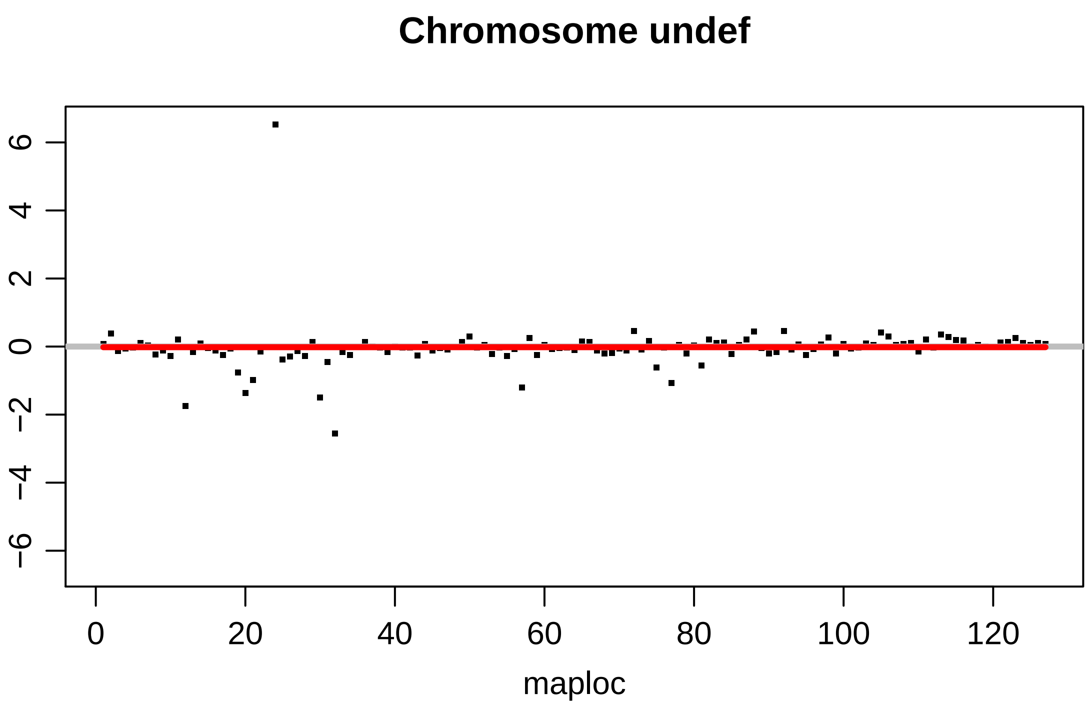


```
## Segplot might not work because of special characters in the sample names. Use only A-Z,a-z and 0-9!
##  There is a hidden function cn.mops:::.replaceNames that replaces the names in the "CNVDetectionResu
```

r_SN2.4.Neonatal.Assay2_new_bed_version_318_chip_v2_Auto_user_SN2.4.Neon

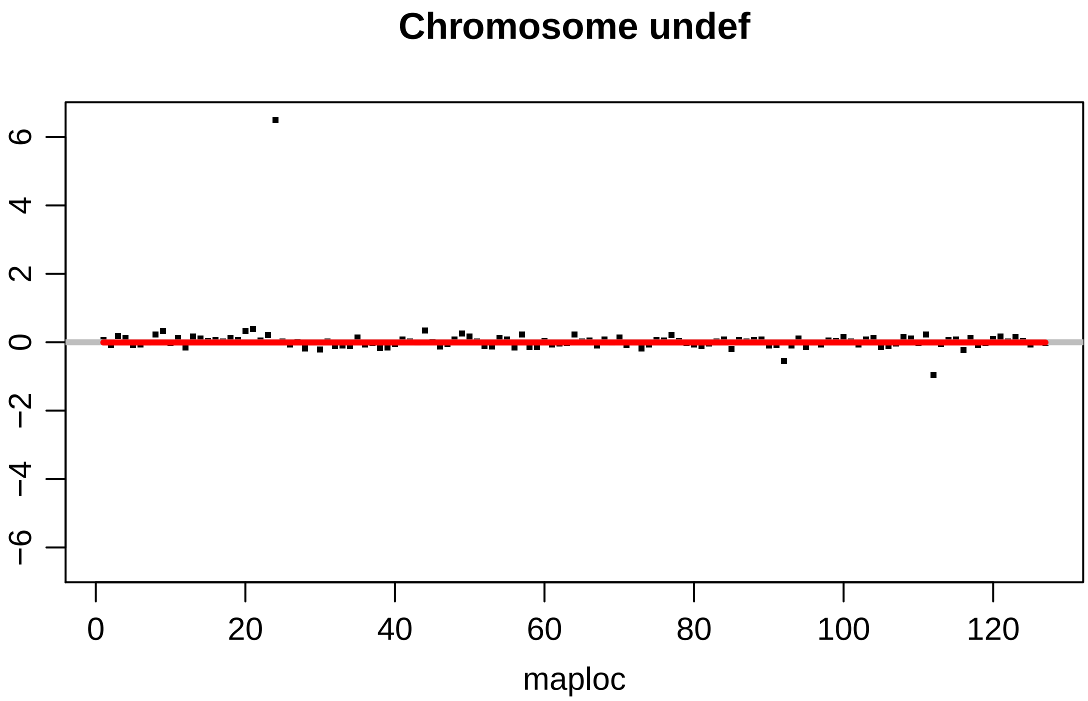

```
## Segplot might not work because of special characters in the sample names. Use only A-Z,a-z and 0-9!
##  There is a hidden function cn.mops:::.replaceNames that replaces the names in the "CNVDetectionResul
```

r_SN2.4.Neonatal.Assay2_new_bed_version_318_chip_v2_Auto_user_SN2.4.Neon

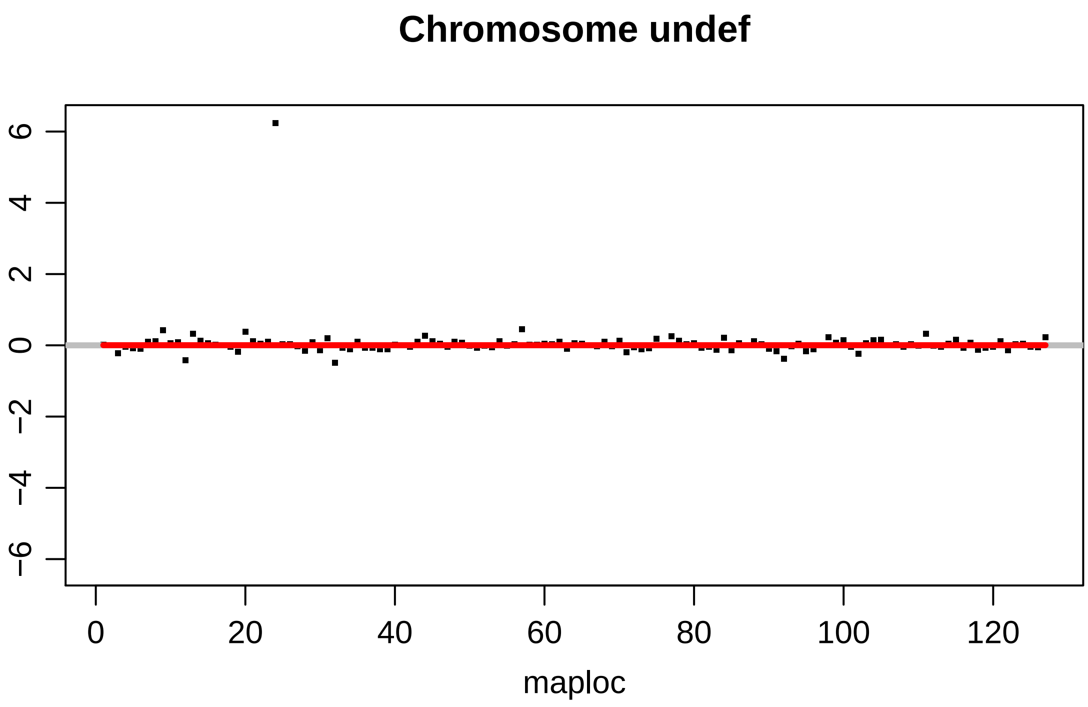


```
## Segplot might not work because of special characters in the sample names. Use only A-Z,a-z and 0-9!
##  There is a hidden function cn.mops:::.replaceNames that replaces the names in the "CNVDetectionResul
```

r_SN2.4.Neonatal.Assay2_new_bed_version_318_chip_v2_Auto_user_SN2.4.Neona

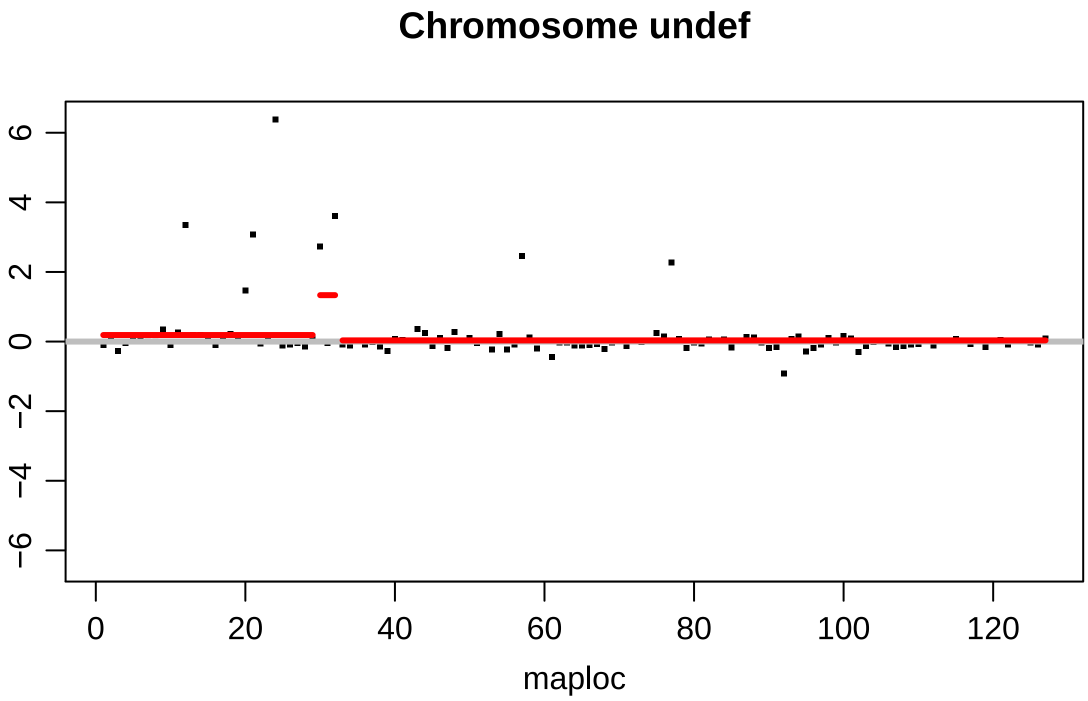


```
## Segplot might not work because of special characters in the sample names. Use only A-Z,a-z and 0-9!
##  There is a hidden function cn.mops:::.replaceNames that replaces the names in the "CNVDetectionResul
```

r_SN2.4.Neonatal.Assay2_new_bed_version_318_chip_v2_Auto_user_SN2.4.Neona

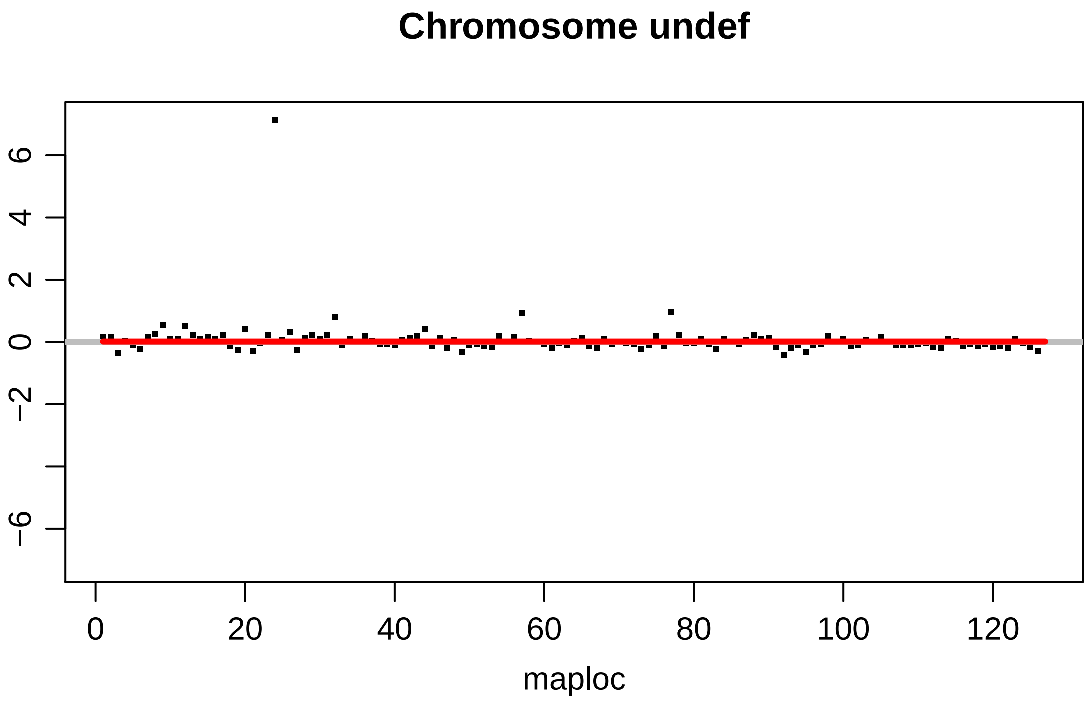

```
## Segplot might not work because of special characters in the sample names. Use only A-Z,a-z and 0-9!
##  There is a hidden function cn.mops:::.replaceNames that replaces the names in the "CNVDetectionResul
```

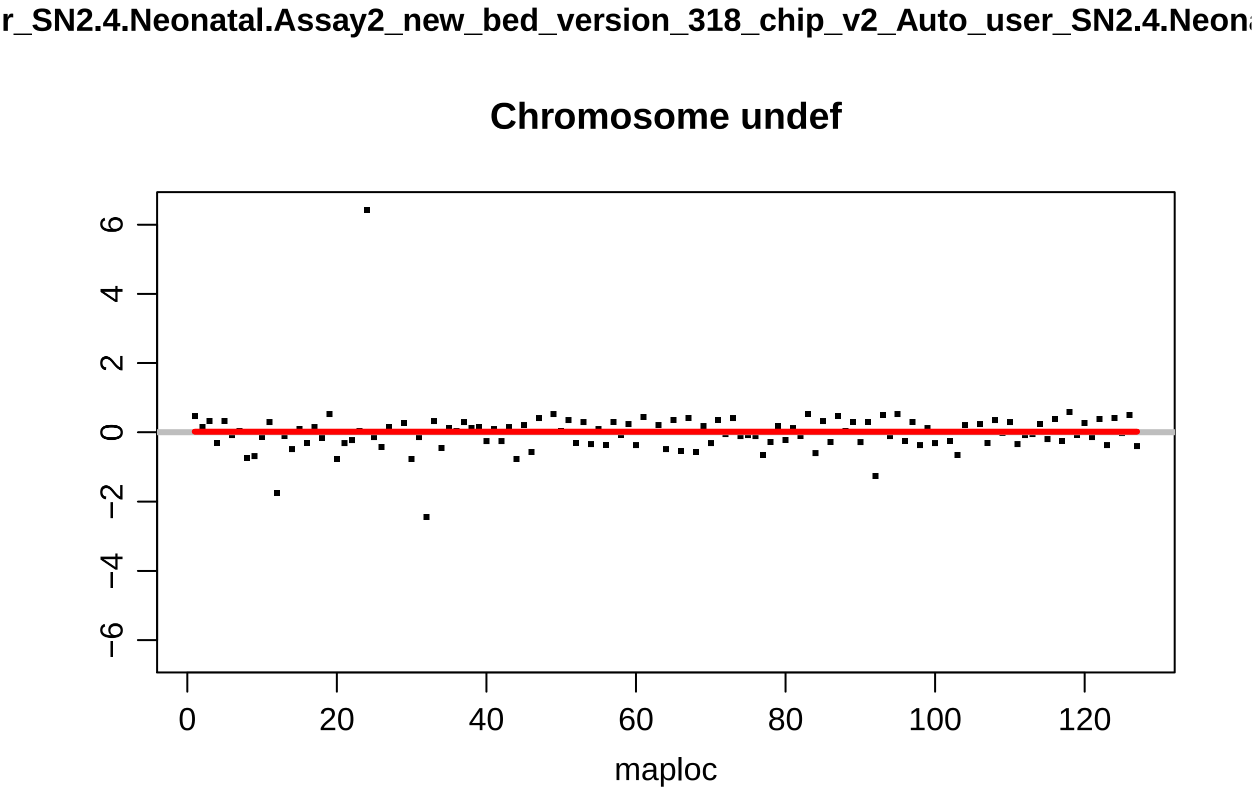


```
## Segplot might not work because of special characters in the sample names. Use only A-Z,a-z and 0-9!
##  There is a hidden function cn.mops:::.replaceNames that replaces the names in the "CNVDetectionResul
```

r_SN2.4.Neonatal.Assay2_new_bed_version_318_chip_v2_Auto_user_SN2.4.Neon

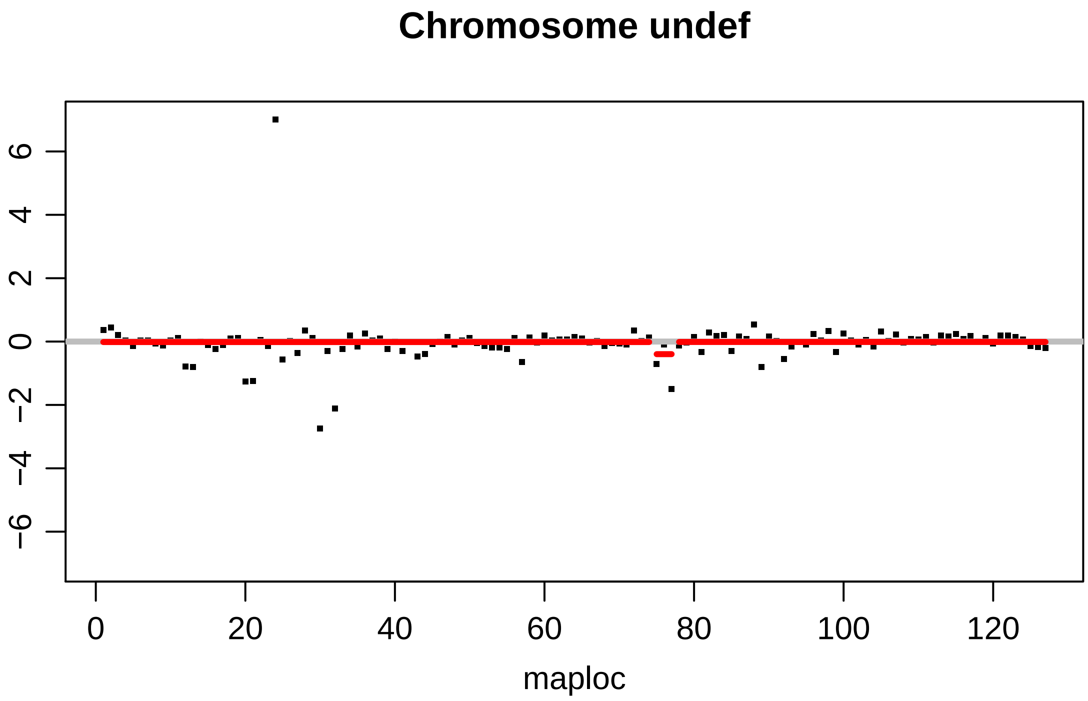


```
## Segplot might not work because of special characters in the sample names. Use only A-Z,a-z and 0-9!
##  There is a hidden function cn.mops:::.replaceNames that replaces the names in the "CNVDetectionResu
```

r_SN2.4.Neonatal.Assay2_new_bed_version_318_chip_v2_Auto_user_SN2.4.Neon

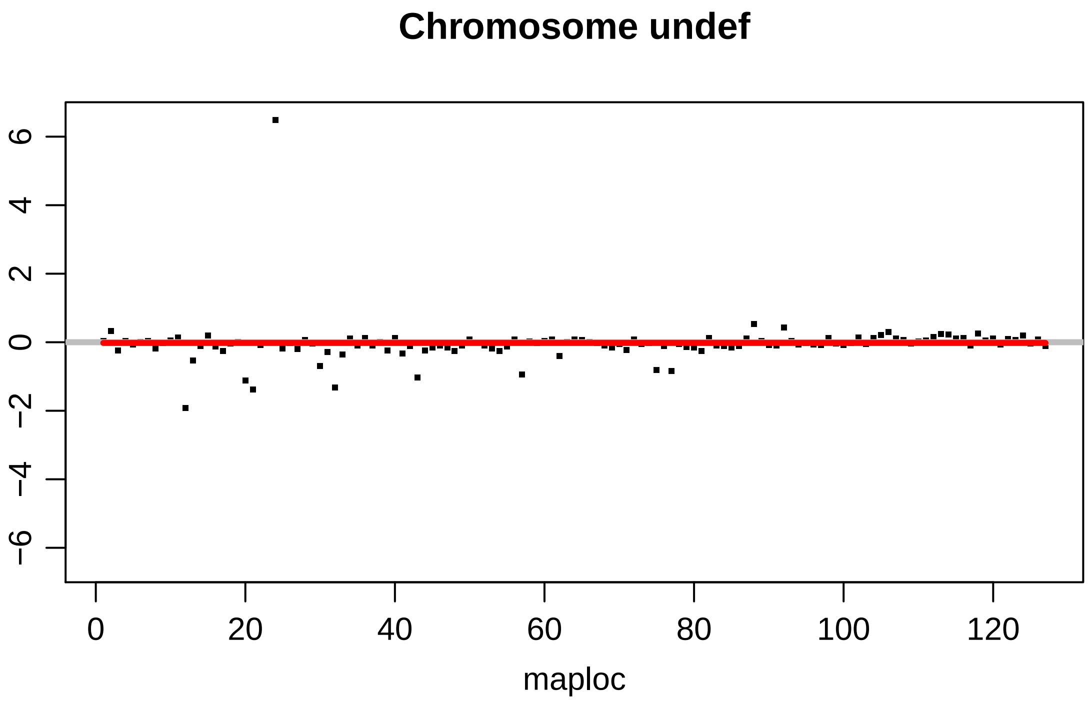

```
## Segplot might not work because of special characters in the sample names. Use only A-Z,a-z and 0-9!
##  There is a hidden function cn.mops:::.replaceNames that replaces the names in the "CNVDetectionResul
```

r_SN2.4.Neonatal.Assay2_new_bed_version_318_chip_v2_Auto_user_SN2.4.Neon

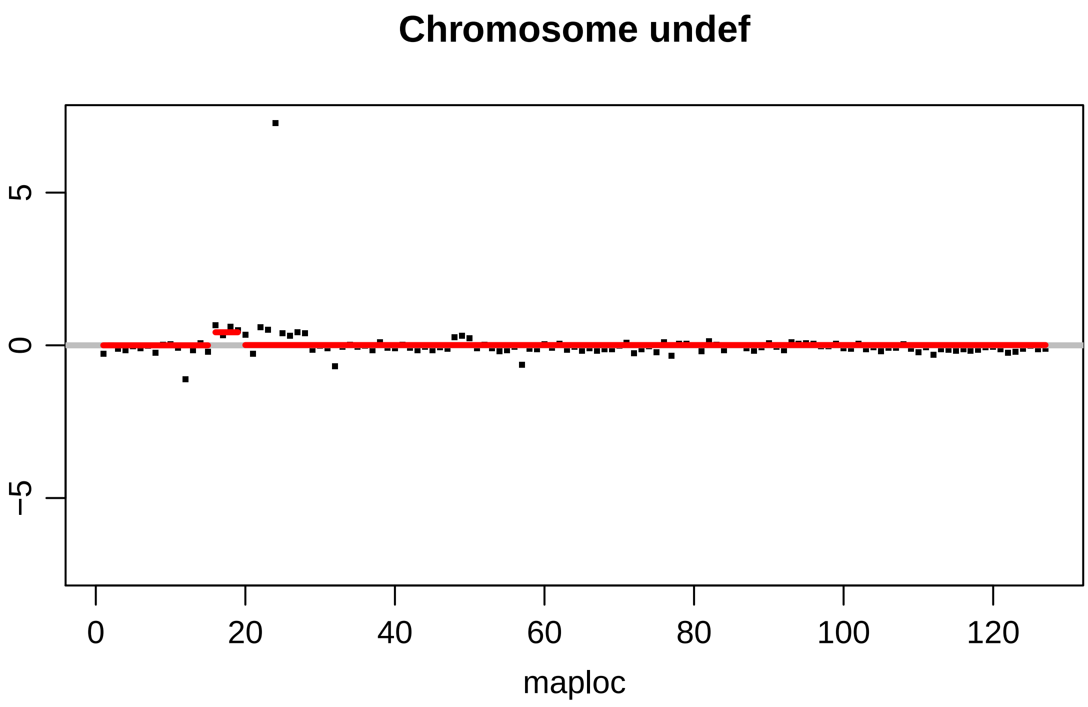


```
## Segplot might not work because of special characters in the sample names. Use only A-Z,a-z and 0-9!
##  There is a hidden function cn.mops:::.replaceNames that replaces the names in the "CNVDetectionResul
```

r_SN2.4.Neonatal.Assay2_new_bed_version_318_chip_v2_Auto_user_SN2.4.Neon

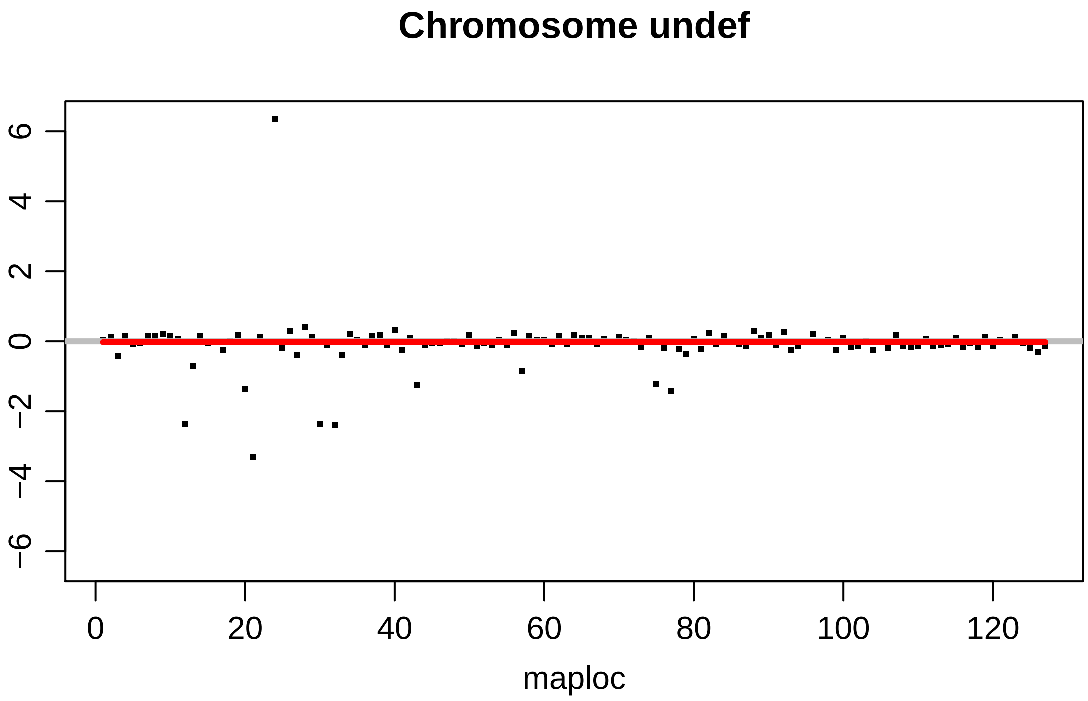


```
## Segplot might not work because of special characters in the sample names. Use only A-Z,a-z and 0-9!
##  There is a hidden function cn.mops:::.replaceNames that replaces the names in the "CNVDetectionResul
```

r_SN2.4.Neonatal.Assay2_new_bed_version_318_chip_v2_Auto_user_SN2.4.Neon

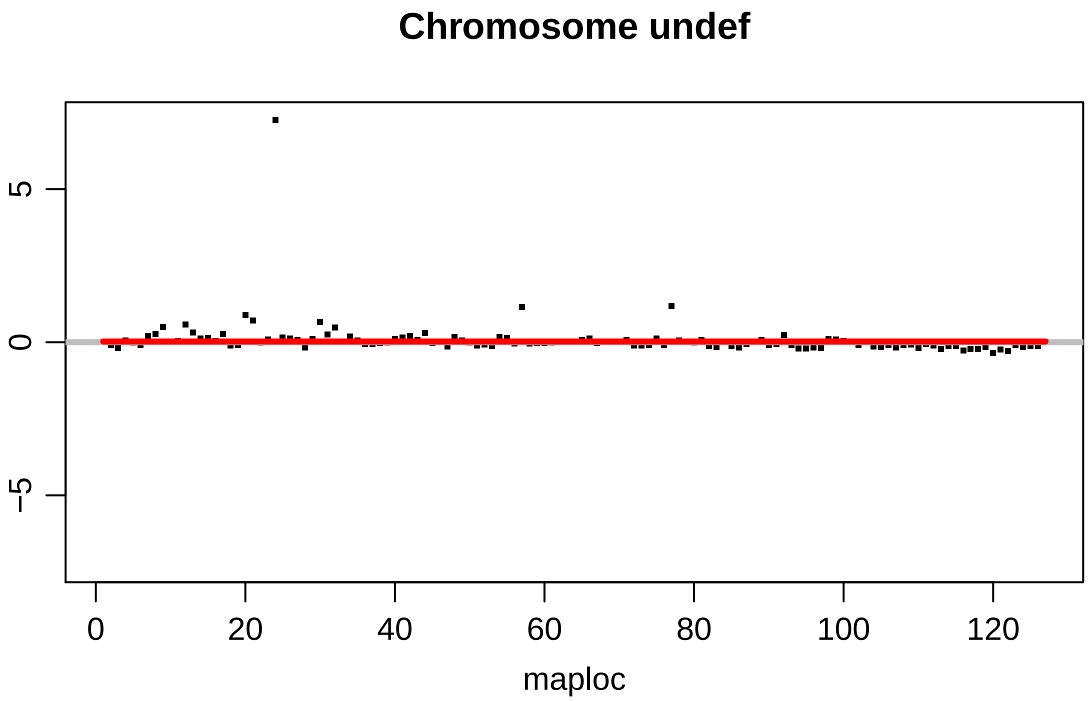

```
## Segplot might not work because of special characters in the sample names. Use only A-Z,a-z and 0-9!
##  There is a hidden function cn.mops:::.replaceNames that replaces the names in the "CNVDetectionResul
```

r_SN2.4.Neonatal.Assay2_new_bed_version_318_chip_v2_Auto_user_SN2.4.Neona

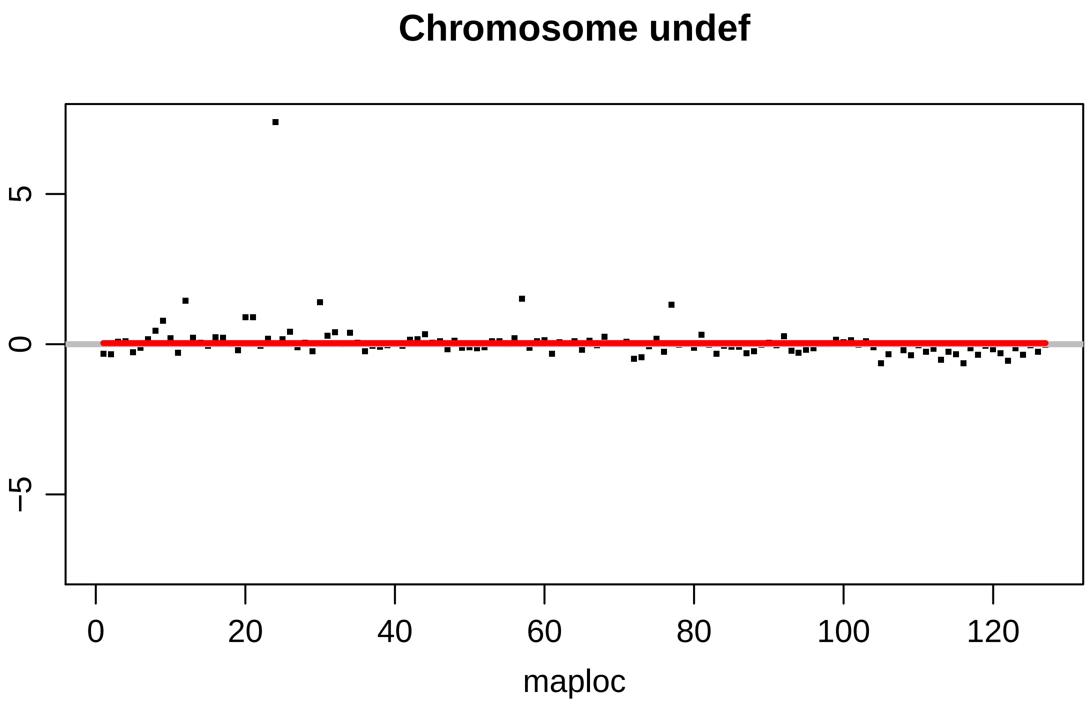

r_SN2.4.Neonatal.Assay2_new_bed_version_318_chip_v2_Auto_user_SN2.4.Neona

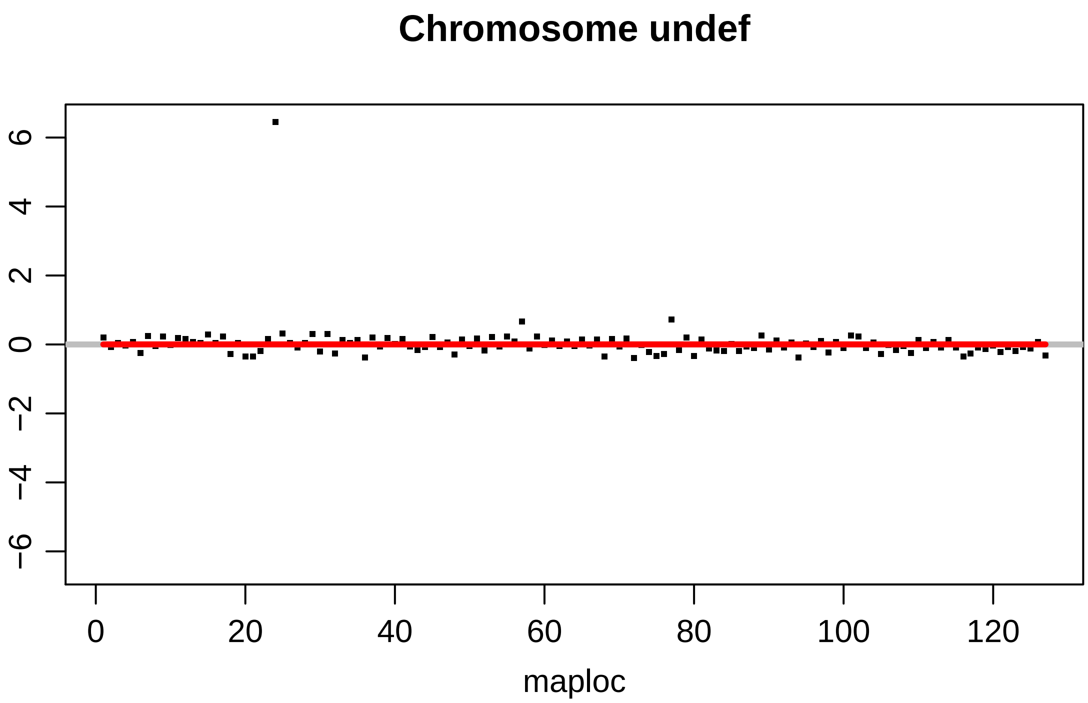


```
##
```

```
## CNV regions:
## GRanges object with 3 ranges and 32 metadata columns:
##       seqnames     ranges strand |
##          <Rle>  <IRanges>  <Rle> |
##   [1]    undef [ 30,  32]      * |
##   [2]    undef [ 45,  47]      * |
##   [3]    undef [102, 127]      * |
##       Case_IonXpress_017_R_2013_10_18_01_30_51_user_SN2.4.Neonatal.Assay2_new_bed_version_318_chip_v
##
##   [1]
##   [2]
##   [3]
##       Case_IonXpress_019_R_2013_10_18_01_30_51_user_SN2.4.Neonatal.Assay2_new_bed_version_318_chip_v
##
##   [1]
##   [2]
##   [3]
##       Case_IonXpress_020_R_2013_10_18_01_30_51_user_SN2.4.Neonatal.Assay2_new_bed_version_318_chip_v
##
##   [1]
##   [2]
##   [3]
##       Case_IonXpress_021_R_2013_10_18_01_30_51_user_SN2.4.Neonatal.Assay2_new_bed_version_318_chip_v
##
##   [1]
##   [2]
##   [3]
##       Case_IonXpress_022_R_2013_10_18_01_30_51_user_SN2.4.Neonatal.Assay2_new_bed_version_318_chip_v
##
##   [1]
##   [2]
##   [3]
##       Case_IonXpress_023_R_2013_10_18_01_30_51_user_SN2.4.Neonatal.Assay2_new_bed_version_318_chip_v
##
##   [1]
##   [2]
##   [3]
##       Case_IonXpress_024_R_2013_10_18_01_30_51_user_SN2.4.Neonatal.Assay2_new_bed_version_318_chip_v
##
##   [1]
##   [2]
##   [3]
##       Case_IonXpress_025_R_2013_10_18_01_30_51_user_SN2.4.Neonatal.Assay2_new_bed_version_318_chip_v
##
##   [1]
##   [2]
##   [3]
##       Case_IonXpress_026_R_2013_10_18_01_30_51_user_SN2.4.Neonatal.Assay2_new_bed_version_318_chip_v
##
##   [1]
##   [2]
##   [3]
##       Case_IonXpress_027_R_2013_10_18_01_30_51_user_SN2.4.Neonatal.Assay2_new_bed_version_318_chip_v
##
```

```
## [1]
## [2]
## [3]
##     Case_IonXpress_028_R_2013_10_18_01_30_51_user_SN2.4.Neonatal.Assay2_new_bed_version_318_chip_v[illegible]
##
## [1]
## [2]
## [3]
##     Case_IonXpress_029_R_2013_10_18_01_30_51_user_SN2.4.Neonatal.Assay2_new_bed_version_318_chip_v[illegible]
##
## [1]
## [2]
## [3]
##     Case_IonXpress_030_R_2013_10_18_01_30_51_user_SN2.4.Neonatal.Assay2_new_bed_version_318_chip_v[illegible]
##
## [1]
## [2]
## [3]
##     Case_IonXpress_031_R_2013_10_18_01_30_51_user_SN2.4.Neonatal.Assay2_new_bed_version_318_chip_v[illegible]
##
## [1]
## [2]
## [3]
##     Case_IonXpress_032_R_2013_10_18_01_30_51_user_SN2.4.Neonatal.Assay2_new_bed_version_318_chip_v[illegible]
##
## [1]
## [2]
## [3]
##     Case_IonXpress_033_R_2013_10_18_01_30_51_user_SN2.4.Neonatal.Assay2_new_bed_version_318_chip_v[illegible]
##
## [1]
## [2]
## [3]
##     Case_IonXpress_035_R_2013_10_18_01_30_51_user_SN2.4.Neonatal.Assay2_new_bed_version_318_chip_v[illegible]
##
## [1]
## [2]
## [3]
##     Case_IonXpress_036_R_2013_10_18_01_30_51_user_SN2.4.Neonatal.Assay2_new_bed_version_318_chip_v[illegible]
##
## [1]
## [2]
## [3]
##     Case_IonXpress_037_R_2013_10_18_01_30_51_user_SN2.4.Neonatal.Assay2_new_bed_version_318_chip_v[illegible]
##
## [1]
## [2]
## [3]
##     Case_IonXpress_038_R_2013_10_18_01_30_51_user_SN2.4.Neonatal.Assay2_new_bed_version_318_chip_v[illegible]
##
## [1]
## [2]
## [3]
##     Case_IonXpress_039_R_2013_10_18_01_30_51_user_SN2.4.Neonatal.Assay2_new_bed_version_318_chip_v[illegible]
```

```
##
##   [1]
##   [2]
##   [3]
##       Case_IonXpress_040_R_2013_10_18_01_30_51_user_SN2.4.Neonatal.Assay2_new_bed_version_318_chip_v
##
##   [1]
##   [2]
##   [3]
##       Case_IonXpress_041_R_2013_10_18_01_30_51_user_SN2.4.Neonatal.Assay2_new_bed_version_318_chip_v
##
##   [1]
##   [2]
##   [3]
##       Case_IonXpress_042_R_2013_10_18_01_30_51_user_SN2.4.Neonatal.Assay2_new_bed_version_318_chip_v
##
##   [1]
##   [2]
##   [3]
##       Case_IonXpress_043_R_2013_10_18_01_30_51_user_SN2.4.Neonatal.Assay2_new_bed_version_318_chip_v
##
##   [1]
##   [2]
##   [3]
##       Case_IonXpress_044_R_2013_10_18_01_30_51_user_SN2.4.Neonatal.Assay2_new_bed_version_318_chip_v
##
##   [1]
##   [2]
##   [3]
##       Case_IonXpress_045_R_2013_10_18_01_30_51_user_SN2.4.Neonatal.Assay2_new_bed_version_318_chip_v
##
##   [1]
##   [2]
##   [3]
##       Case_IonXpress_046_R_2013_10_18_01_30_51_user_SN2.4.Neonatal.Assay2_new_bed_version_318_chip_v
##
##   [1]
##   [2]
##   [3]
##       Case_IonXpress_047_R_2013_10_18_01_30_51_user_SN2.4.Neonatal.Assay2_new_bed_version_318_chip_v
##
##   [1]
##   [2]
##   [3]
##       Case_IonXpress_048_R_2013_10_18_01_30_51_user_SN2.4.Neonatal.Assay2_new_bed_version_318_chip_v
##
##   [1]
##   [2]
##   [3]
##       Case_IonXpress_063_R_2013_10_18_01_30_51_user_SN2.4.Neonatal.Assay2_new_bed_version_318_chip_v
##
##   [1]
##   [2]
##   [3]
```

```
##       Case_IonXpress_064_R_2013_10_18_01_30_51_user_SN2.4.Neonatal.Assay2_new_bed_version_318_chip_v1
##
##   [1]
##   [2]
##   [3]
##   -------
##   seqinfo: 1 sequence from an unspecified genome; no seqlengths
##
## Individual CNVs:
## GRanges object with 5 ranges and 4 metadata columns:
##       seqnames     ranges strand |
##          <Rle>  <IRanges>  <Rle> |
##   [1]    undef [102, 107]      * |
##   [2]    undef [108, 127]      * |
##   [3]    undef [117, 119]      * |
##   [4]    undef [ 45,  47]      * |
##   [5]    undef [ 30,  32]      * |
##
##
##   [1] Case_IonXpress_023_R_2013_10_18_01_30_51_user_SN2.4.Neonatal.Assay2_new_bed_version_318_chip_v1
##   [2] Case_IonXpress_023_R_2013_10_18_01_30_51_user_SN2.4.Neonatal.Assay2_new_bed_version_318_chip_v1
##   [3] Case_IonXpress_025_R_2013_10_18_01_30_51_user_SN2.4.Neonatal.Assay2_new_bed_version_318_chip_v1
##   [4] Case_IonXpress_027_R_2013_10_18_01_30_51_user_SN2.4.Neonatal.Assay2_new_bed_version_318_chip_v1
##   [5] Case_IonXpress_041_R_2013_10_18_01_30_51_user_SN2.4.Neonatal.Assay2_new_bed_version_318_chip_v1
##           median       mean          CN
##        <numeric>  <numeric> <character>
##   [1]  0.9979665  0.8589287         CN4
##   [2]  0.5849619  0.7170512         CN3
##   [3]  0.5282857  0.5091271         CN3
##   [4] -0.9977121 -0.9974170         CN1
##   [5]  1.9999992  1.3333331         CN3
##   -------
##   seqinfo: 1 sequence from an unspecified genome; no seqlengths
```
