# Supplementary material for: A statistical approach to detection of copy number variations in PCR-enriched targeted sequencing data
Source: BMC Bioinformatics. 2016 Oct 22;17:429. doi: 10.1186/s12859-016-1272-6 (PMC5075217; doi:10.1186/s12859-016-1272-6)
Supplement: Additional file 5 — Archive with cn.mops results. Zip archive with large pdf file (R markdown) with the results of cn.mops (plots) and short summary of calls. (ZIP 1771 kb) [file 12859_2016_1272_MOESM5_ESM.zip › summary.pdf]

cn.mops	TP	FP	FN
15	6	3,7,4,10,4,10,5,4	
16	6,6	0	
17	6	3, 3	6,6
18	6,6,6,6	0	
19	5,6	3,6,4,8,8,3,3,4	10
20	6,6	0	
SN1-27	6,6,6,6	6,7,10	6,6
SN2-28	6	3,7,4,4	
SN2-2	6	7,7,7,7,10,5,10	
SN2-3	0	10,10,10,10,10,7,7,7,7	6
SN2-4	2,2,6,6,6	7,10	2,2
SN1-41	10,	10,6	2,2,2
SN1-41 CGR	6,6,6,6,6,6	3,5,10	
SN1-42 CGR	6,6	4,4,8	6,6
SN1-43 CGR		8,6,4,10,4,4,10,4,7,7	
SN1-45 CGR	6	7,4,4,4,7,7,10,10,10,8	
IP1	6,6,6,6	2,2,3,3,6,3	0
IP2	6,6,6	3,4,3	7
IP3	0	3	0
IP4	2	4,4,6,6,3,3,3	6
SN1-56	0	0	0
SN1-57	0	10, 10	2
SN1-59	8,10	0	10,10,10,7,4,4,5,7
Merged_new_panel	5,8,5,5,5	0	10,10,10,10,5
	46	90	28
*CNVs of 1 amplicon length were not taken into account			
