# Supplementary material for: A statistical approach to detection of copy number variations in PCR-enriched targeted sequencing data
Source: BMC Bioinformatics. 2016 Oct 22;17:429. doi: 10.1186/s12859-016-1272-6 (PMC5075217; doi:10.1186/s12859-016-1272-6)
Supplement: Additional file 7 — Final reports on CNVs analysis of whole dataset by CONVector. Tab delimited xls files with final reports of CONVector and short summary of calls. (ZIP 42.1 kb) [file 12859_2016_1272_MOESM7_ESM.zip › summary.pdf]

Run	True Positive	False Negative	False Positive
15	1,1,4	10	10
16	1,1,1,6,6	0	1,1,1,2
17	6,6,6	0	1
18	6,6,6,6	2	1
19	5,6	0	10
20	1,1,6,6	1	1
SN1-27	6,6,6,6,6	6	1,1
SN2-28	6	0	6,4
SN2-2	6	0	10,2,2
SN2-3	0	6	20,2,1,20
SN2-4	1,1,1,1,5,5,5	10	1,10,2
SN1-41	2,1,1,10,10,10	0	2,1,10
SN1-41 CGR	6,6,6,6,6,6	0	2
SN1-42 CGR	6,6	0	0
SN1-43 CGR	0	0	1,1
SN1-45 CGR	5	0	10,1,1,1
IP1	6,6,6,6	0	1
IP2	6,6,6	0	2,1
IP3	0	0	1
IP4	6,2	0	1,1
SN1-56	0	0	1,1,1,1
SN1-57	2	0	1
SN1-59	5,10,4,4,7,10,10,8	10,7	10
Merged_new	7,10,2,4,1,5,8,4,5,1,5,4,5,5,5,10,10	5,10	1
	Unsupervised Algorithm		

Each number means a deteced CNV(s) of a specified length in one of the samples from the run

Run	True Positive	False Negative	False Positive
15	1,1,4,10	0	10
16	6,6	1,1,1	1,1,2
17	6,6,6	0	0
18	6,6,6,6,2	0	1,2
19	5,6,10	0	2,2
20	6,6	1,1,1	0
SN1-27	6,6,6,6,6,6	0	1,10,10,10,10,10,10
SN2-28	6	0	1,10
SN2-2	6	0	10,2,3,2
SN2-3	6	0	4,20,4,1,20
SN2-4	1,1,1,1,5,5,5,10	0	1,2,10,4,8
SN1-41	2,1,1,10,10,10	0	2,1,5,10,10
SN1-41 CGR	6,6,6,6,6,6	0	2,1,1,10
SN1-42 CGR	6,6	0	0
SN1-43 CGR	0	0	2
SN1-45 CGR	5	0	10,2
IP1	6,6,6,6	0	10,10
IP2	6,6,6	0	1,1
IP3	0	0	0
IP4	6,2	0	1,3,10
SN1-56	0	0	1,1,1,1,4
SN1-57	2	0	0
SN1-59	10,10,10,7,4,4,5,8,10	7	10,10
Merged_new	7,10,2,4,1,5,8,4,5,1,5,3,5,5,5,10,10,10	5	10,10,10
	Supervised Algorithm		

Each number means a deteced CNV(s) of a specified length in one of the samples from the run
